# Global, regional, and national progress towards Sustainable Development Goal 3.2 for neonatal and child health: all-cause and cause-specific mortality findings from the Global Burden of Disease Study 2019

**DOI:** 10.1016/S0140-6736(21)01207-1

**Published:** 2021-09-04

**Authors:** GBD 2019 Under-5 Mortality Collaborators, Katherine R Paulson, Aruna M Kamath, Tahiya Alam, Kelly Bienhoff, Gdiom Gebreheat Abady, Jaffar Abbas, Mohsen Abbasi-Kangevari, Hedayat Abbastabar, Foad Abd-Allah, Sherief M Abd-Elsalam, Amir Abdoli, Aidin Abedi, Hassan Abolhassani, Lucas Guimarães Abreu, Eman Abu-Gharbieh, Niveen ME Abu-Rmeileh, Abdelrahman I Abushouk, Aishatu L Adamu, Oladimeji M Adebayo, Adeyinka Emmanuel Adegbosin, Victor Adekanmbi, Olatunji O Adetokunboh, Daniel Adedayo Adeyinka, Jose C Adsuar, Khashayar Afshari, Mohammad Aghaali, Marcela Agudelo-Botero, Bright Opoku Ahinkorah, Tauseef Ahmad, Keivan Ahmadi, Muktar Beshir Ahmed, Budi Aji, Yonas Akalu, Oluwaseun Oladapo Akinyemi, Addis Aklilu, Ziyad Al-Aly, Khurshid Alam, Fahad Mashhour Alanezi, Turki M Alanzi, Jacqueline Elizabeth Alcalde-Rabanal, Ayman Al-Eyadhy, Tilahun Ali, Gianfranco Alicandro, Sheikh Mohammad Alif, Vahid Alipour, Hesam Alizade, Syed Mohamed Aljunid, Amir Almasi-Hashiani, Nihad A Almasri, Hesham M Al-Mekhlafi, Jordi Alonso, Rajaa M Al-Raddadi, Khalid A Altirkawi, Arwa Khalid Alumran, Nelson Alvis-Guzman, Nelson J Alvis-Zakzuk, Edward Kwabena Ameyaw, Saeed Amini, Mostafa Amini-Rarani, Arianna Maever L Amit, Dickson A Amugsi, Robert Ancuceanu, Deanna Anderlini, Catalina Liliana Andrei, Fereshteh Ansari, Alireza Ansari-Moghaddam, Carl Abelardo T Antonio, Ernoiz Antriyandarti, Davood Anvari, Razique Anwer, Muhammad Aqeel, Jalal Arabloo, Morteza Arab-Zozani, Timur Aripov, Johan Ärnlöv, Kurnia Dwi Artanti, Afsaneh Arzani, Malke Asaad, Mehran Asadi-Aliabadi, Ali A Asadi-Pooya, Mohammad Asghari Jafarabadi, Seyyed Shamsadin Athari, Seyyede Masoume Athari, Desta Debalkie Atnafu, Alok Atreya, Madhu Sudhan Atteraya, Marcel Ausloos, Asma Tahir Awan, Beatriz Paulina Ayala Quintanilla, Getinet Ayano, Martin Amogre Ayanore, Yared Asmare Aynalem, Samad Azari, Ghasem Azarian, Zelalem Nigussie Azene, Darshan B B, Ebrahim Babaee, Ashish D Badiye, Atif Amin Baig, Maciej Banach, Palash Chandra Banik, Suzanne Lyn Barker-Collo, Hiba Jawdat Barqawi, Quique Bassat, Sanjay Basu, Bernhard T Baune, Mohsen Bayati, Neeraj Bedi, Ettore Beghi, Massimiliano Beghi, Michelle L Bell, Salaheddine Bendak, Derrick A Bennett, Isabela M Bensenor, Kidanemaryam Berhe, Adam E Berman, Yihienew Mequanint Bezabih, Akshaya Srikanth Bhagavathula, Dinesh Bhandari, Nikha Bhardwaj, Pankaj Bhardwaj, Krittika Bhattacharyya, Suraj Bhattarai, Zulfiqar A Bhutta, Boris Bikbov, Antonio Biondi, Binyam Minuye Birihane, Raaj Kishore Biswas, Somayeh Bohlouli, Nicola Luigi Bragazzi, Alexey V Breusov, Andre R Brunoni, Katrin Burkart, Sharath Burugina Nagaraja, Reinhard Busse, Zahid A Butt, Florentino Luciano Caetano dos Santos, Lucero Cahuana-Hurtado, Paulo Camargos, Luis Alberto Cámera, Rosario Cárdenas, Giulia Carreras, Juan J Carrero, Felix Carvalho, Joao Mauricio Castaldelli-Maia, Carlos A Castañeda-Orjuela, Giulio Castelpietra, Ester Cerin, Jung-Chen Chang, Wagaye Fentahun Chanie, Jaykaran Charan, Souranshu Chatterjee, Soosanna Kumary Chattu, Vijay Kumar Chattu, Sarika Chaturvedi, Simiao Chen, Daniel Youngwhan Cho, Jee-Young Jasmine Choi, Dinh-Toi Chu, Liliana G Ciobanu, Massimo Cirillo, Joao Conde, Vera Marisa Costa, Rosa A S Couto, Berihun Assefa Dachew, Saad M A Dahlawi, Hancheng Dai, Xiaochen Dai, Lalit Dandona, Rakhi Dandona, Parnaz Daneshpajouhnejad, Gary L Darmstadt, Jai K Das, Claudio Alberto Dávila-Cervantes, Adrian C Davis, Kairat Davletov, Fernando Pio De la Hoz, Diego De Leo, Farah Deeba, Edgar Denova-Gutiérrez, Nikolaos Dervenis, Assefa Desalew, Keshab Deuba, Sagnik Dey, Samath Dhamminda Dharmaratne, Sameer Dhingra, Govinda Prasad Dhungana, Diana Dias da Silva, Daniel Diaz, Fariba Dorostkar, Leila Doshmangir, Eleonora Dubljanin, Andre Rodrigues Duraes, Arielle Wilder Eagan, Hisham Atan Edinur, Ferry Efendi, Sahar Eftekharzadeh, Iman El Sayed, Maha El Tantawi, Iffat Elbarazi, Islam Y Elgendy, Shaimaa I El-Jaafary, Amir Emami, Shymaa Enany, Oghenowede Eyawo, Sayeh Ezzikouri, Pawan Sirwan Faris, Farshad Farzadfar, Nazir Fattahi, Nelsensius Klau Fauk, Mehdi Fazlzadeh, Valery L Feigin, Tomas Y Ferede, Seyed-Mohammad Fereshtehnejad, Eduarda Fernandes, Pietro Ferrara, Irina Filip, Florian Fischer, James L Fisher, Nataliya A Foigt, Morenike Oluwatoyin Folayan, Masoud Foroutan, Richard Charles Franklin, Marisa Freitas, Sara D Friedman, Takeshi Fukumoto, Mohamed M Gad, Abhay Motiramji Gaidhane, Shilpa Gaidhane, Santosh Gaihre, Silvano Gallus, Alberto L Garcia-Basteiro, MA Garcia-Gordillo, William M Gardner, Mariana Gaspar Fonseca, Ketema Bizuwork Gebremedhin, Lemma Getacher, Ahmad Ghashghaee, Asadollah Gholamian, Syed Amir Gilani, Tiffany K Gill, Giorgia Giussani, Elena V Gnedovskaya, Myron Anthony Godinho, Amit Goel, Mahaveer Golechha, Philimon N Gona, Sameer Vali Gopalani, Houman Goudarzi, Michal Grivna, Harish Chander Gugnani, Davide Guido, Rafael Alves Guimarães, Rajat Das Gupta, Rajeev Gupta, Nima Hafezi-Nejad, Mohammad Rifat Haider, Arvin Haj-Mirzaian, Samer Hamidi, Asif Hanif, Graeme J Hankey, Arief Hargono, Ahmed I Hasaballah, Md Mehedi Hasan, Syed Shahzad Hasan, Amr Hassan, Soheil Hassanipour, Hadi Hassankhani, Rasmus J Havmoeller, Khezar Hayat, Reza Heidari-Soureshjani, Nathaniel J Henry, Claudiu Herteliu, Michael K Hole, Ramesh Holla, Naznin Hossain, Mostafa Hosseini, Mehdi Hosseinzadeh, Mihaela Hostiuc, Sorin Hostiuc, Mowafa Househ, Junjie Huang, Ayesha Humayun, Bing-Fang Hwang, Ivo Iavicoli, Segun Emmanuel Ibitoye, Kevin S Ikuta, Olayinka Stephen Ilesanmi, Irena M Ilic, Milena D Ilic, Sumant Inamdar, Leeberk Raja Inbaraj, Khalid Iqbal, Usman Iqbal, M Mofizul Islam, Sheikh Mohammed Shariful Islam, Hiroyasu Iso, Masao Iwagami, Chidozie C D Iwu, Jalil Jaafari, Kathryn H Jacobsen, Jagnoor Jagnoor, Vardhmaan Jain, Manthan Dilipkumar Janodia, Tahereh Javaheri, Fatemeh Javanmardi, Shubha Jayaram, Achala Upendra Jayatilleke, Ensiyeh Jenabi, Ravi Prakash Jha, John S Ji, Oommen John, Jost B Jonas, Tamas Joo, Nitin Joseph, Farahnaz Joukar, Jacek Jerzy Jozwiak, Mikk Jürisson, Ali Kabir, Zubair Kabir, Leila R Kalankesh, Naser Kamyari, Tanuj Kanchan, Neeti Kapoor, Behzad Karami Matin, André Karch, Salah Eddin Karimi, Getinet Kassahun, Gbenga A Kayode, Ali Kazemi Karyani, Laura Kemmer, Nauman Khalid, Rovshan Khalilov, Mohammad Khammarnia, Ejaz Ahmad Khan, Gulfaraz Khan, Maseer Khan, Md Nuruzzaman Khan, Young-Ho Khang, Khaled Khatab, Amir M Khater, Mona M Khater, Maryam Khayamzadeh, Ardeshir Khosravi, Daniel Kim, Young-Eun Kim, Yun Jin Kim, Ruth W Kimokoti, Adnan Kisa, Sezer Kisa, Niranjan Kissoon, Jacek A Kopec, Soewarta Kosen, Parvaiz A Koul, Sindhura Lakshmi Koulmane Laxminarayana, Ai Koyanagi, Kewal Krishan, Vijay Krishnamoorthy, Barthelemy Kuate Defo, Burcu Kucuk Bicer, Vaman Kulkarni, G Anil Kumar, Manasi Kumar, Nithin Kumar, Om P Kurmi, Dian Kusuma, Carlo La Vecchia, Ben Lacey, Ratilal Lalloo, Faris Hasan Lami, Iván Landires, Anders O Larsson, Savita Lasrado, Zohra S Lassi, Paolo Lauriola, Paul H Lee, Shaun Wen Huey Lee, Yo Han Lee, James Leigh, Matilde Leonardi, Sonia Lewycka, Bingyu Li, Shanshan Li, Juan Liang, Lee-Ling Lim, Miteku Andualem Limenih, Ro-Ting Lin, Xuefeng Liu, Rakesh Lodha, Alan D Lopez, Rafael Lozano, Alessandra Lugo, Raimundas Lunevicius, Mark T Mackay, Shilpashree Madhava Kunjathur, Francesca Giulia Magnani, D R Mahadeshwara Prasad, Mina Maheri, Morteza Mahmoudi, Azeem Majeed, Venkatesh Maled, Afshin Maleki, Shokofeh Maleki, Reza Malekzadeh, Ahmad Azam Malik, Deborah Carvalho Malta, Abdullah A Mamun, Borhan Mansouri, Mohammad Ali Mansournia, Gabriel Martinez, Santi Martini, Francisco Rogerlândio Martins-Melo, Seyedeh Zahra Masoumi, Pallab K Maulik, Colm McAlinden, John J McGrath, Carlo Eduardo Medina-Solís, Entezar Mehrabi Nasab, Fabiola Mejia-Rodriguez, Ziad A Memish, Walter Mendoza, Ritesh G Menezes, Endalkachew Worku Mengesha, George A Mensah, Atte Meretoja, Tuomo J Meretoja, Abera M Mersha, Tomislav Mestrovic, Bartosz Miazgowski, Tomasz Miazgowski, Irmina Maria Michalek, Ted R Miller, GK Mini, Mohammad Miri, Andreea Mirica, Erkin M Mirrakhimov, Hamed Mirzaei, Maryam Mirzaei, Babak Moazen, Masoud Moghadaszadeh, Bahram Mohajer, Osama Mohamad, Yousef Mohammad, Seyyede Momeneh Mohammadi, Abdollah Mohammadian-Hafshejani, Shafiu Mohammed, Ali H Mokdad, Mariam Molokhia, Lorenzo Monasta, Stefania Mondello, Mohammad Ali Moni, Catrin E Moore, Ghobad Moradi, Masoud Moradi, Rahmatollah Moradzadeh, Paula Moraga, Lidia Morawska, Shane Douglas Morrison, Jonathan F Mosser, Amin Mousavi Khaneghah, Ghulam Mustafa, Mehdi Naderi, Ahamarshan Jayaraman Nagarajan, Shankar Prasad Nagaraju, Mohsen Naghavi, Behshad Naghshtabrizi, Mukhammad David Naimzada, Vinay Nangia, Sreenivas Narasimha Swamy, Bruno Ramos Nascimento, Muhammad Naveed, Javad Nazari, Rawlance Ndejjo, Ionut Negoi, Ruxandra Irina Negoi, Evangelia Nena, Samata Nepal, Henok Biresaw Netsere, Georges Nguefack-Tsague, Josephine W Ngunjiri, Chi Thi Yen Nguyen, Cuong Tat Nguyen, Huong Lan Thi Nguyen, Yeshambel T Nigatu, Samuel Negash Nigussie, Molly R Nixon, Chukwudi A Nnaji, Shuhei Nomura, Nurulamin M Noor, Jean Jacques Noubiap, Virginia Nuñez-Samudio, Vincent Ebuka Nwatah, Bogdan Oancea, Oluwakemi Ololade Odukoya, Felix Akpojene Ogbo, Bolajoko Olubukunola Olusanya, Jacob Olusegun Olusanya, Ahmed Omar Bali, Obinna E Onwujekwe, Alberto Ortiz, Adrian Otoiu, Nikita Otstavnov, Stanislav S Otstavnov, Mayowa O Owolabi, Mahesh P A, Jagadish Rao Padubidri, Smita Pakhale, Keyvan Pakshir, Pramod Kumar Pal, Raffaele Palladino, Adrian Pana, Songhomitra Panda-Jonas, Anamika Pandey, Ashok Pandey, Seithikurippu R Pandi-Perumal, Helena Ullyartha Pangaribuan, Ana Melisa Pardo-Montaño, Eun-Kee Park, Sangram Kishor Patel, George C Patton, Shrikant Pawar, Hamidreza Pazoki Toroudi, Amy E Peden, Veincent Christian Filipino Pepito, Emmanuel K Peprah, Jeevan Pereira, Jorge Pérez-Gómez, Norberto Perico, Konrad Pesudovs, Thomas Pilgrim, Marina Pinheiro, Michael A Piradov, Meghdad Pirsaheb, James A Platts-Mills, Khem Narayan Pokhrel, Maarten J Postma, Hadi Pourjafar, Sergio I Prada, Sanjay Prakash, Elisabetta Pupillo, Zahiruddin Quazi Syed, Navid Rabiee, Amir Radfar, Ata Rafiee, Alireza Rafiei, Alberto Raggi, Shadi Rahimzadeh, Mohammad Hifz Ur Rahman, Amir Masoud Rahmani, Kiana Ramezanzadeh, Juwel Rana, Chhabi Lal Ranabhat, Sowmya J Rao, Davide Rasella, Prateek Rastogi, Priya Rathi, David Laith Rawaf, Salman Rawaf, Wasiq Faraz Rawasia, Reza Rawassizadeh, Robert C Reiner Jr, Giuseppe Remuzzi, Andre M N Renzaho, Bhageerathy Reshmi, Serge Resnikoff, Negar Rezaei, Nima Rezaei, Aziz Rezapour, Seyed Mohammad Riahi, Daniela Ribeiro, Jennifer Rickard, Leonardo Roever, Luca Ronfani, Dietrich Rothenbacher, Enrico Rubagotti, Susan Fred Rumisha, Paul MacDaragh Ryan, Basema Saddik, Ehsan Sadeghi, Sahar Saeedi Moghaddam, Rajesh Sagar, Amirhossein Sahebkar, Mohammad Reza Salahshoor, Sana Salehi, Marwa Rashad Salem, Hamideh Salimzadeh, Joshua A Salomon, Yoseph Leonardo Samodra, Abdallah M Samy, Juan Sanabria, Milena M Santric-Milicevic, Sivan Yegnanarayana Iyer Saraswathy, Abdur Razzaque Sarker, Nizal Sarrafzadegan, Arash Sarveazad, Brijesh Sathian, Thirunavukkarasu Sathish, Davide Sattin, Sonia Saxena, Ganesh Kumar Saya, Mete Saylan, Silvia Schiavolin, Markus P Schlaich, David C Schwebel, Falk Schwendicke, Subramanian Senthilkumaran, Sadaf G Sepanlou, Edson Serván-Mori, Feng Sha, Omid Shafaat, Saeed Shahabi, Mohammad Shahbaz, Amira A Shaheen, Izza Shahid, Masood Ali Shaikh, Saeed Shakiba, Ali S Shalash, Mehran Shams-Beyranvand, Mohammed Shannawaz, Kiomars Sharafi, Aziz Sheikh, Sara Sheikhbahaei, Wondimeneh Shibabaw Shiferaw, Mika Shigematsu, Jae Il Shin, Rahman Shiri, Ivy Shiue, Kerem Shuval, Tariq Jamal Siddiqi, Negussie Boti Sidemo, Inga Dora Sigfusdottir, Rannveig Sigurvinsdottir, João Pedro Silva, Jonathan I S Silverberg, Biagio Simonetti, Balbir Bagicha Singh, Jasvinder A Singh, Deepika Singhal, Dhirendra Narain Sinha, Eirini Skiadaresi, Valentin Yurievich Skryabin, Anna Aleksandrovna Skryabina, David A Sleet, Badr Hasan Sobaih, Mohammad Reza Sobhiyeh, Shahin Soltani, Joan B Soriano, Emma Elizabeth Spurlock, Chandrashekhar T Sreeramareddy, Paschalis Steiropoulos, Mark A Stokes, Stefan Stortecky, Mu'awiyyah Babale Sufiyan, Rizwan Suliankatchi Abdulkader, Gerhard Sulo, Carolyn B Swope, Bryan L Sykes, Mindy D Szeto, Miklós Szócska, Rafael Tabarés-Seisdedos, Eyayou Girma Tadesse, Amir Taherkhani, Animut Tagele Tamiru, Md Ismail Tareque, Arash Tehrani-Banihashemi, Mohamad-Hani Temsah, Fisaha Haile Tesfay, Gizachew Assefa Tessema, Zemenu Tadesse Tessema, Kavumpurathu Raman Thankappan, Rekha Thapar, Musliu Adetola Tolani, Marcos Roberto Tovani-Palone, Eugenio Traini, Bach Xuan Tran, Jaya Prasad Tripathy, Giorgos Tsapparellas, Aristidis Tsatsakis, Lorainne Tudor Car, Riaz Uddin, Anayat Ullah, Chukwuma David Umeokonkwo, Brigid Unim, Bhaskaran Unnikrishnan, Era Upadhyay, Muhammad Shariq Usman, Marco Vacante, Maryam Vaezi, Sahel Valadan Tahbaz, Pascual R Valdez, Tommi Juhani Vasankari, Narayanaswamy Venketasubramanian, Madhur Verma, Francesco S Violante, Vasily Vlassov, Bay Vo, Giang Thu Vu, Yohannes Dibaba Wado, Yasir Waheed, Richard G Wamai, Yanping Wang, Yanzhong Wang, Yuan-Pang Wang, Paul Ward, Andrea Werdecker, Ronny Westerman, Nuwan Darshana Wickramasinghe, Lauren B Wilner, Charles Shey Wiysonge, Ai-Min Wu, Chenkai Wu, Yang Xie, Seyed Hossein Yahyazadeh Jabbari, Kazumasa Yamagishi, Srikanth Yandrapalli, Sanni Yaya, Vahid Yazdi-Feyzabadi, Paul Yip, Naohiro Yonemoto, Seok-Jun Yoon, Mustafa Z Younis, Zabihollah Yousefi, Taraneh Yousefinezhadi, Chuanhua Yu, Sifat Shahana Yusuf, Syed Saoud Zaidi, Sojib Bin Zaman, Mohammad Zamani, Maryam Zamanian, Mikhail Sergeevich Zastrozhin, Anasthasia Zastrozhina, Yunquan Zhang, Zhi-Jiang Zhang, Xiu-Ju George Zhao, Arash Ziapour, Simon I Hay, Christopher J L Murray, Haidong Wang, Nicholas J Kassebaum

## Abstract

**Background:**

Sustainable Development Goal 3.2 has targeted elimination of preventable child mortality, reduction of neonatal death to less than 12 per 1000 livebirths, and reduction of death of children younger than 5 years to less than 25 per 1000 livebirths, for each country by 2030. To understand current rates, recent trends, and potential trajectories of child mortality for the next decade, we present the Global Burden of Diseases, Injuries, and Risk Factors Study (GBD) 2019 findings for all-cause mortality and cause-specific mortality in children younger than 5 years of age, with multiple scenarios for child mortality in 2030 that include the consideration of potential effects of COVID-19, and a novel framework for quantifying optimal child survival.

**Methods:**

We completed all-cause mortality and cause-specific mortality analyses from 204 countries and territories for detailed age groups separately, with aggregated mortality probabilities per 1000 livebirths computed for neonatal mortality rate (NMR) and under-5 mortality rate (U5MR). Scenarios for 2030 represent different potential trajectories, notably including potential effects of the COVID-19 pandemic and the potential impact of improvements preferentially targeting neonatal survival. Optimal child survival metrics were developed by age, sex, and cause of death across all GBD location-years. The first metric is a global optimum and is based on the lowest observed mortality, and the second is a survival potential frontier that is based on stochastic frontier analysis of observed mortality and Healthcare Access and Quality Index.

**Findings:**

Global U5MR decreased from 71·2 deaths per 1000 livebirths (95% uncertainty interval [UI] 68·3–74·0) in 2000 to 37·1 (33·2–41·7) in 2019 while global NMR correspondingly declined more slowly from 28·0 deaths per 1000 live births (26·8–29·5) in 2000 to 17·9 (16·3–19·8) in 2019. In 2019, 136 (67%) of 204 countries had a U5MR at or below the SDG 3.2 threshold and 133 (65%) had an NMR at or below the SDG 3.2 threshold, and the reference scenario suggests that by 2030, 154 (75%) of all countries could meet the U5MR targets, and 139 (68%) could meet the NMR targets. Deaths of children younger than 5 years totalled 9·65 million (95% UI 9·05–10·30) in 2000 and 5·05 million (4·27–6·02) in 2019, with the neonatal fraction of these deaths increasing from 39% (3·76 million [95% UI 3·53–4·02]) in 2000 to 48% (2·42 million; 2·06–2·86) in 2019. NMR and U5MR were generally higher in males than in females, although there was no statistically significant difference at the global level. Neonatal disorders remained the leading cause of death in children younger than 5 years in 2019, followed by lower respiratory infections, diarrhoeal diseases, congenital birth defects, and malaria. The global optimum analysis suggests NMR could be reduced to as low as 0·80 (95% UI 0·71–0·86) deaths per 1000 livebirths and U5MR to 1·44 (95% UI 1·27–1·58) deaths per 1000 livebirths, and in 2019, there were as many as 1·87 million (95% UI 1·35–2·58; 37% [95% UI 32–43]) of 5·05 million more deaths of children younger than 5 years than the survival potential frontier.

**Interpretation:**

Global child mortality declined by almost half between 2000 and 2019, but progress remains slower in neonates and 65 (32%) of 204 countries, mostly in sub-Saharan Africa and south Asia, are not on track to meet either SDG 3.2 target by 2030. Focused improvements in perinatal and newborn care, continued and expanded delivery of essential interventions such as vaccination and infection prevention, an enhanced focus on equity, continued focus on poverty reduction and education, and investment in strengthening health systems across the development spectrum have the potential to substantially improve U5MR. Given the widespread effects of COVID-19, considerable effort will be required to maintain and accelerate progress.

**Funding:**

Bill & Melinda Gates Foundation.

**Research in context**

**Evidence before this study**

During the Millennium Development Goal (MDG) era (2000–15), numerous organisations comprehensively described global progress in reducing child and neonatal mortality (MDG 4), but the early Sustainable Development Goal (SDG) period has seen few comparable efforts to track progress and none to date have attempted to quantify the preventable portion of child mortality (SDG 3.2). Past preventable mortality analyses have focused on health-care delivery, or were limited to high-income countries and adult populations. The most recent child mortality report from the UN Inter-agency Group for Child Mortality Estimation (UNIGME), published in 2017 for the year 2015, reports on all-cause mortality only. The Global Burden of Diseases, Injuries, and Risk Factors Study (GBD) is the only annual assessment of trends in all-cause mortality and cause-specific mortality by detailed age groups for all locations with a population greater than 50 000 people from 1990 to the present that is compliant with the Guidelines for Accurate and Transparent Health Estimates Reporting.

**Added value of this study**

This analysis presents levels and trends in all-cause and cause-specific neonatal and under-5 mortality from 2000 to 2019. Multiple future health scenarios for child mortality in 2030 were constructed to represent potential trajectories, including the potential impacts of the COVID-19 pandemic and scenarios with targeted improvements in neonatal survival. Additionally, this study presents for the first time all-cause mortality estimates for granular age groups of 0–6 days, 7–27 days, 1–5 months, 6–11 months, 12–23 months, and 2–4 years. SDG 3.2 explicitly prioritises ending preventable child deaths. Therefore, based on all-cause and cause-specific mortality estimates from GBD 2019, this study introduces a novel, reproducible, and holistic heuristic for quantifying optimal child survival. Within this framework are two complementary cause-specific benchmarks: a global optimum, based on the lowest observed neonatal and under-5 mortality, and a survival potential frontier, based on stochastic frontier analysis of observed mortality and the Healthcare Access and Quality Index. The latter allows for comparing performance between similar countries, and specifically helps those countries with high mortality to establish intermediate goals.

**Implications of all the available evidence**

The prevention of child deaths accelerated in the MDG era. In the emerging SDG period, progress to prevent child deaths remains slowest in neonates. The study findings highlight regions with potential imbalances in health priorities. The findings can also identify causes of death with the most potential for reduction, and those with the greatest need for resources, expertise, and service delivery, or for basic research into prevention and treatment. To reach the SDG targets by 2030, policy makers must focus on balancing priorities between early newborn care while continuing prenatal and older child health initiatives. Strengthening quality health systems and ensuring effective investment in high-burden countries are imperative in order to scale up interventions. Equally pressing are the needs to examine within-country disparities and pursue integrative action on other determinants of health.

## Introduction

Under-5 mortality rate (U5MR) and neonatal mortality rate (NMR) are important indicators reflecting multiple aspects of societal wellbeing such as access to nutrition and food; basic infrastructure such as housing, water, and sanitation; education; agency; financial security; access to preventive and treatment health services; and future human capital. The UN Millennium Development Goals (MDGs) are credited with mobilising global action on child health, and manifested as an unprecedented, accelerated reduction in child mortality and resulted in 58 countries achieving the MDG 4 target of reducing U5MR by two-thirds.^1^, ^2^ Sustainable Development Goal (SDG) 3.2 specifically calls to, “By 2030, end preventable deaths of newborn babies and children under 5 years of age, with all countries aiming to reduce neonatal mortality to at least as low as 12 per 1000 live births and under-5 mortality to at least as low as 25 per 1000 live births.”^3^ The SDG focus on equity was codified here in a shifting from relative global targets, that were mainstays in the MDG agenda, to absolute targets for each country.

The SDG framework aims to build on the successes of the MDG era, albeit with a notably broader lens in which health (SDG 3) is one of several goals related to healthier lives, wellbeing, and equity.^3^ Even within SDG 3, the SDG agenda is broader than the MDG agenda, reflecting a growing understanding of the intersectional nature of health outcomes with basic infrastructural considerations such as health system performance, sustainability, and environment. This intersectional perspective is illustrated in the language of initiatives such as the call from the UN Global Strategy for Women's, Children's and Adolescents' Health 2016–2030 to integrate survival, prevention, thriving, and enabling environments,^4^ the Every Newborn Action Plan, the World Bank's Global Financing Facility for Women, Children and Adolescents, *The Lancet Global Health* Commission on High Quality Health Systems, and the Countdown to 2030.^5^, ^6^, ^7^ Although this broader focus has not necessarily led to child and neonatal health receiving less investment in development assistance for health (DAH; which, for child and neonatal health, grew by 2·66% from 2015 to 2019 and remained the second largest DAH focus area in 2019), the growth in investment in this period was less than during the period between 2000 and 2015, when DAH for child and neonatal health increased by 314%.^8^

There has not yet been a comprehensive assessment of NMR and U5MR in the SDG era. Selected publications assessed interim progress towards part of SDG 3.2 or provided projections to 2030,^9^, ^10^, ^11^, ^12^, ^13^ but none have been comprehensive with respect to cause, age, trends, geography, and progress towards 2030 targets. The comprehensive nature of the Global Burden of Diseases, Injuries, and Risk Factors Study (GBD) 2019 lends itself to a detailed analysis of levels, trends, and drivers of change for specific age groups, causes, and locations. Additionally, there has not been any previous effort, to our knowledge, to empirically explore the concept of preventable mortality in children. Although preventable death has been theoretically defined since the early 2000s, the definitions has usually been through a health-care delivery lens^14^, ^15^ rather than a more holistic lens of preventability that might be interpreted as the intended wording of SDG 3.2. Furthermore, although the Organisation for Economic Co-operation and Development (OECD) and Eurostat convened to provide a more uniform approach to interpreting avoidable deaths in 2019, this was with a singular focus on high-income countries and the adult population.^16^

In this study, based on GBD 2019, we have three objectives. First, we aim to present a detailed, comprehensive numerical assessment of progress towards SDG 3.2 targets for all-cause NMR and U5MR at the global, regional, and national level, including a series of scenarios that reflect possible trends over the next decade including the potential effects of the COVID-19 pandemic on young children. Second, we aim to evaluate comparative progress in cause-specific mortality in neonates and children from 2000 to 2019 to highlight successes and potential focus areas for improvement. Third, we aim to better define a holistic focus of preventable mortality by exploring two different measures of optimal child survival that can both inform global progress and provide a benchmark for intermediate progress evaluation in high-mortality settings. In so doing, this study seeks to meet the needs of an expansive, integrative SDG agenda, and to highlight the locations, age groups, and causes of preventable deaths, to inform policy and public health priorities aiming to achieve SDG 3.2. This manuscript was produced as part of the GBD Collaborator Network and in accordance with the GBD Protocol.

## Methods

### Overview

This study is compliant with the Guidelines for Accurate and Transparent Health Estimates Reporting (GATHER;^17^ appendix p 9). A brief summary of each component of our study is described below. Extensive methodological details are provided in the appendix (pp 10–86).

### Dimensions of the GBD study

GBD 2019 includes all-cause and cause-specific mortality by age and sex for 204 countries and territories, 21 of which were estimated at the subnational level from 1990 to 2019, inclusive. Results in this study are presented only for countries and territories. All-cause mortality estimation covers six under-5 age groups: 0–6 days (early neonatal), 7–27 days (late neonatal), 1–5 months, 6–11 months, 12–23 months, and 2–4 years. Cause-specific mortality estimates cover four age groups: early neonatal, late neonatal, 28–364 days, and 1–4 years. Although we present all six age groups, we mainly focus on results for the aggregate neonatal age group (<28 days) and the under-5 age group (0–4 years), to best align with the SDG under-5 and neonatal targets. Similarly, we focus on the years 2000, which marks the establishment of the MDGs, 2015, which marks the establishment of the SDGs, and 2019, which is the most recent year of GBD estimates.

### Data sources

All-cause mortality data were compiled from 203 of 204 countries and territories ranging from the years 2000 to 2019, for a total of 3097 location-years. Vital registration covered a total of 14 889 022 global under-5 deaths in this period (appendix p 119). A total of 8000 unique sources were used in estimating cause-specific mortality in GBD 2019. All input data sources for each component of analysis are available for download from the GBD 2019 Data Input Sources Tool.

### All-cause mortality estimation and assessment of progress towards SDG 3.2

All-cause mortality estimation closely followed the estimation techniques as described for previous iterations of GBD,^2^, ^18^, ^19^ detailed in the appendix (p 9). Progress towards SDG 3.2 was assessed by examining U5MR and NMR in 2019. NMR is calculated as the probability of death between birth and 28 days and U5MR is calculated as the probability of death between birth and 5 years, and each metric is expressed as the number of deaths per 1000 livebirths. Aggregate mortality probabilities were benchmarked against the SDG thresholds of 25 under-5 deaths per 1000 livebirths and 12 neonatal deaths per 1000 livebirths.

To assess relative progress across age groups, we compared the proportion of under-5 deaths occurring in each age group with the ratio of change in age-specific deaths to change in total under-5 deaths, for the periods 2000–14 and 2015–19. If progress towards SDG 3.2 is equal across age groups, the percentage contribution to progress and the percentage of total deaths would be equal. If the percentage of deaths is greater than the percentage of progress for an age group, then that age group is making slower progress towards the target.

### Cause-specific mortality estimation

GBD 2019 includes 369 causes of disease and injury in a mutually exclusive and collectively exhaustive hierarchy of four levels (appendix p 87). Some conditions only result in fatal burden (eg, sudden infant death syndrome), whereas others cause only disability (eg, scabies); most causes have both fatal and non-fatal burden. Comprehensive methods for cause-specific mortality estimation for GBD have been previously described^20^ and are detailed in the appendix (p 35). We present most results at level 3 because this level is sufficiently detailed to reflect important cause groupings for the age groups presented in this analysis (eg, neonatal disorders and congenital birth defects), but not so detailed as to obscure important groupings of related conditions.

### Scenarios for 2030 and beyond

U5MR and NMR were projected for six scenarios, all computed at the national level, up to 2030 as previously described.^21^ The first three scenarios represent the reference, better-than-reference, and worse-than-reference scenarios, while a fourth represents the 2030 NMR and U5MR in the absence of COVID-19. The remaining two scenarios are intended to assess outcomes for interventions that focus only on specific age groups, to evaluate if opportunity is greater in a particular age group than in others, and to show the limits of achievement when efforts do not consider distinct needs of different age groups. For the first of these age-specific scenarios, neonatal mortality is at the better-than-reference level and remaining under-5 mortality stays at reference level (neonatal scenario), and for the second, mortality for children aged 28–364 days is at the better-than-reference level and neonatal mortality stays at the reference level (child scenario). Many strategies to address neonatal mortality are fundamentally different from strategies targeting older infants and children, so these two scenarios are a broad representation of those differences.

### Assessment of optimal survival potential

Our approach to inform an assessment of preventable mortality focused on the quantification of two different measures of optimal child survival based on historical trends. The first measure, what we term the global optimum, represents a universal level at which all additional mortality is theoretically avoidable given current medical knowledge and technology. This is analogous to the GBD method used for estimating global standard life expectancy. The second measure, what we term the survival potential frontier, aims to quantify the amount of mortality that is avoidable given the country's level of Healthcare Access and Quality (HAQ) Index, thereby accounting for the differential resources available for health investment in different locations.

First, we calculated the global optimum for NMR and U5MR based on the aggregate of the lowest observed age-specific and cause-specific mortality rates in locations with populations higher than 10 000 children younger than 5 years (to remove noise associated with small numbers) between 2000 and 2019, scaling them to match an all-cause mortality minimum that was calculated using the same approach. The scaling step was added to account for potential differences due to small numbers in low-mortality settings or geographical differences in cause assignment that can occur between, for example, subcauses of neonatal disorders. This method is analogous to that used by GBD to calculate a global standard life expectancy for the purposes of calculating years of life lost and therefore can be interpreted to represent the optimum potential for reductions in child mortality based on current technology and health delivery systems.

Second, to help with developing intermediate goals and to evaluate progress in higher-mortality settings, we calculated a survival potential frontier using stochastic frontier analysis^22^ to evaluate the historical relationship between cause-specific neonatal and under-5 mortality rates and HAQ Index,^23^ which is an aggregate metric of health system performance across all age groups combined. The specific formulation of the stochastic frontier analysis is described in detail in the appendix (p 70), but briefly, it uses a spline to estimate the expected lower bound of mortality for a given value of HAQ Index. Stochastic frontier analysis was chosen to quantify system inefficiency because of its flexibility in shape, its assumption of performance possibilities given static system inputs, and the fact that it allows for random effects in the model rather than assuming uniformity of inputs across locations.

All components of the analysis are based on 1000 draws of the posterior distribution of the quantity of interest by age, sex, location, and year. Point estimates are the mean of the draws, and 95% uncertainty intervals (UIs) represent the 2·5th and 97·5th percentiles.

### Presentation of results

Results are presented by country, GBD super-region, and Socio-demographic Index (SDI)^24^ quintile. SDI is a composite index of income per capita, educational attainment, and inverse fertility, and it is used to categorise countries into SDI quintiles: low SDI (ie, low income per capita, low educational attainment, high fertility), low-middle SDI, middle SDI, high-middle SDI, and high SDI. Full results for GBD 2019 are available in an online visualisation at GBD Compare and for download from the GBD Results Tool.

### Role of the funding source

The funders of the study had no role in study design, data collection, data analysis, data interpretation, or writing of the report.

## Results

### All-cause mortality and progress towards SDG 3.2

Over the past two decades, there has been a substantial decrease in global deaths of children younger than 5 years, from 9·65 million (95% UI 9·05–10·30) in 2000, to 6·10 million (5·35–6·91) in 2015, and to 5·05 million (4·27–6·02) in 2019 (table). Of these deaths, 3·76 million (95% UI 3·53–4·02; 39%) in 2000, 2·82 million (2·48–3·20; 46%) in 2015, and 2·42 million (2·06–2·86; 48%) in 2019 occurred in neonates (aged <28 days). In each year analysed, the largest share of the global deaths of children younger than 5 years occurred in the sub-Saharan Africa and south Asia super-regions. Although U5MR declined in each successive period in all super-regions, the proportion of global deaths in children younger than 5 years in these two super-regions increased from 73% (7·07 million deaths [95% UI 6·57–7·59]) in 2000 to 80% (4·04 million [3·36–4·86]) deaths in 2019. The share of under-5 deaths also shifted towards lower SDI groups in this period, with the proportion of under-5 deaths in the low SDI quintile increasing from 42% (4·01 million deaths [95% UI 3·78–4·26]) in 2000 to 53% (2·67 million deaths [2·22–3·24]) in 2019.

**Table:** Neonatal and under-5 deaths in 2000, 2015, and 2019, by country, GBD region, GBD super-region, and SDI, and at the global level for both sexes combined; and neonatal mortality rate in 2019 with reference scenario for 2030

		**Neonatal deaths**			**NMR**		**Under-5 deaths**			**U5MR**	
		2000	2015	2019	2019	2030*	2000	2015	2019	2019	2030*
**SDI regions**											
**Global**		**3 760 000 (3 530 000–4 020 000)**	**2 820 000 (2 480 000–3 200 000)**	**2 420 000 (2 060 000–2 860 000)**	**17·9 (16·3–19·8)**	**15·4**	**9 650 000 (9 050 000–10 300 000)**	**6 100 000 (5 350 000–6 910 000)**	**5 050 000 (4 270 000–6 020 000)**	**37·1 (33·2–41·7)**	**29·6**
Low SDI		1 260 000 (1 190 000–1 340 000)	1 190 000 (1 030 000–1 370 000)	1 110 000 (918 000–1 340 000)	27·0 (24·0–30·8)	21·4	4 010 000 (3 780 000–4 260 000)	3 040 000 (2 630 000–3 520 000)	2 670 000 (2 220 000–3 240 000)	71·8 (63·3–82·5)	47·0
Low-middle SDI		1 480 000 (1 370 000–1 600 000)	1 020 000 (883 000–1 170 000)	841 000 (716 000–985 000)	21·7 (19·7–24·0)	19·1	3 390 000 (3 140 000–3 630 000)	1 890 000 (1 640 000–2 150 000)	1 490 000 (1 260 000–1 750 000)	42·0 (37·8–46·7)	30·3
Middle SDI		777 000 (724 000–835 000)	479 000 (419 000–546 000)	368 000 (312 000–432 000)	10·1 (9·11–11·2)	16·3	1 730 000 (1 610 000–1 850 000)	912 000 (803 000–1 040 000)	686 000 (583 000–810 000)	18·9 (17·1–21·0)	27·3
High-middle SDI		199 000 (187 000–213 000)	104 000 (94 200–115 000)	78 100 (67 100–90 900)	5·10 (4·71–5·55)	3·30	427 000 (400 000–455 000)	197 000 (180 000–217 000)	150 000 (130 000–172 000)	9·36 (8·66–10·2)	6·12
High SDI		43 500 (41 600–45 300)	30 500 (29 300–31 700)	26 800 (24 300–29 600)	2·60 (2·51–2·70)	2·57	84 400 (81 000–88 300)	55 800 (54 200–57 600)	48 600 (44 500–53 200)	4·70 (4·56–4·86)	5·02
**GBD super-regions**											
**Central Europe, eastern Europe, and central Asia**		**57 800 (54 300–61 800)**	**39 400 (35 500–43 800)**	**30 800 (26 400–36 000)**	**5·88 (5·35–6·52)**	**4·95**	**127 000 (119 000–135 000)**	**77 900 (70 200–86 900)**	**61 100 (52 200–72 100)**	**11·5 (10·4–12·8)**	**9·34**
Central Asia		31 600 (28 300–35 100)	25 400 (21 900–29 400)	20 500 (17 200–24 600)	10·8 (9·62–12·2)	8·99	75 400 (68 200–82 900)	49 300 (42 200–57 700)	39 700 (33 200–48 400)	20·7 (18·3–23·7)	16·2
	Armenia	661 (595–733)	310 (256–378)	230 (181–292)	5·96 (5·04–7·21)	4·46	1290 (1140–1460)	605 (502–732)	452 (357–575)	11·4 (9·55–13·7)	8·90
	Azerbaijan	4450 (3710–5260)	3410 (2860–3990)	2590 (2130–3110)	16·8 (14·8–19·4)	14·0	9530 (8060–11 100)	5750 (4760–6910)	4310 (3430–5370)	27·6 (23·2–33·4)	21·5
	Georgia	1060 (892–1250)	376 (308–457)	266 (208–336)	5·79 (4·76–7·10)	4·04	1740 (1480–2040)	669 (553–811)	482 (382–603)	10·2 (8·57–12·5)	7·02
	Kazakhstan	3260 (2790–3780)	2520 (2050–3070)	1970 (1530–2600)	5·60 (4·61–6·88)	4·09	8300 (7250–9410)	5420 (4450–6550)	4330 (3410–5540)	12·1 (10·1–14·7)	8·86
	Kyrgyzstan	1960 (1760–2170)	2060 (1870–2230)	1560 (1360–1790)	10·8 (9·91–11·8)	8·57	4380 (3910–4890)	3290 (3090–3490)	2520 (2210–2870)	17·4 (15·9–19·0)	13·0
	Mongolia	1270 (1120–1430)	990 (836–1170)	773 (633–966)	9·29 (7·86–11·1)	6·89	3400 (3030–3780)	1810 (1550–2120)	1430 (1180–1770)	17·0 (14·5–20·4)	10·2
	Tajikistan	4310 (3650–5070)	4100 (3440–4800)	3730 (3180–4380)	14·7 (13·2–16·4)	11·8	12 500 (11 200–13 900)	9220 (7780–11 000)	8100 (6540–10 000)	32·1 (27·5–37·3)	21·8
	Turkmenistan	2260 (1900–2640)	1870 (1590–2170)	1510 (1290–1770)	13·4 (11·7–15·1)	10·3	5990 (5240–6870)	3620 (3000–4260)	2870 (2380–3480)	25·2 (21·4–29·6)	19·4
	Uzbekistan	12 300 (10 600–14 400)	9760 (7900–11 900)	7900 (6360–10 000)	11·1 (9·25–13·4)	10·0	28 300 (24 700–32 800)	18 900 (15 400–22 900)	15 200 (12 400–19 200)	21·2 (17·8–25·7)	18·2
Central Europe		8250 (7940–8590)	3720 (3560–3890)	2930 (2340–3670)	2·72 (2·44–3·03)	1·99	16 700 (16 200–17 200)	6990 (6690–7290)	5550 (4520–6800)	5·06 (4·52–5·63)	3·67
	Albania	845 (739–980)	266 (202–345)	217 (150–317)	5·77 (4·39–7·68)	4·81	1760 (1530–2030)	550 (450–676)	451 (344–595)	11·9 (9·94–14·4)	9·15
	Bosnia and Herzegovina	320 (294–344)	138 (125–153)	103 (85·3–126)	3·95 (3·35–4·67)	3·22	438 (406–471)	191 (173–211)	143 (120–173)	5·41 (4·59–6·39)	4·38
	Bulgaria	534 (487–582)	258 (236–280)	214 (168–271)	3·54 (3·03–4·16)	2·71	1220 (1160–1280)	532 (502–566)	447 (359–556)	7·29 (6·21–8·57)	5·61
	Croatia	236 (220–254)	115 (104–126)	90·5 (67·5–120)	2·56 (2·17–3·02)	1·93	361 (339–383)	181 (165–198)	141 (107–184)	3·95 (3·35–4·67)	2·96
	Czech Republic	236 (215–256)	174 (158–190)	159 (123–201)	1·45 (1·26–1·67)	1·08	483 (454–513)	342 (318–365)	293 (232–369)	2·64 (2·28–3·06)	1·87
	Hungary	572 (530–615)	252 (229–275)	173 (132–224)	2·09 (1·81–2·42)	1·37	1020 (970–1070)	489 (458–521)	336 (262–428)	4·00 (3·46–4·62)	2·80
	Montenegro	77·4 (68·7–86·7)	18·1 (15·4–21·4)	15·1 (12·5–18·3)	2·29 (1·96–2·67)	1·60	116 (104–130)	29·8 (25·5–34·6)	24·9 (20·6–30·0)	3·74 (3·19–4·38)	2·58
	North Macedonia	226 (203–248)	155 (140–170)	123 (99·9–149)	5·52 (4·70–6·49)	4·13	399 (366–434)	239 (215–263)	191 (155–230)	8·51 (7·23–10·0)	6·00
	Poland	1920 (1780–2060)	988 (916–1060)	787 (579–1060)	2·15 (1·84–2·52)	1·36	3530 (3380–3680)	1770 (1680–1850)	1420 (1070–1880)	3·85 (3·29–4·51)	2·60
	Romania	2090 (1920–2260)	884 (815–953)	690 (556–842)	3·98 (3·50–4·60)	3·17	5130 (5010–5260)	1790 (1710–1870)	1420 (1160–1730)	8·03 (7·02–9·33)	5·89
	Serbia	865 (736–1010)	269 (250–291)	196 (154–245)	2·45 (2·08–2·92)	1·47	1590 (1360–1860)	454 (426–486)	334 (263–421)	4·12 (3·51–4·93)	2·42
	Slovakia	266 (243–289)	174 (160–187)	141 (106–186)	2·52 (2·15–2·97)	1·85	533 (502–565)	364 (340–389)	301 (232–390)	5·33 (4·53–6·27)	4·45
	Slovenia	57·9 (53·4–63·0)	31·4 (28·1–35·3)	23·8 (17·9–31·5)	1·26 (1·09–1·46)	0·930	96·1 (88·8–104)	49·4 (44·1–55·4)	38·1 (29·3–49·5)	1·98 (1·70–2·31)	1·43
Eastern Europe		18 000 (17 400–18 600)	10 300 (9920–10 600)	7340 (6140–8710)	3·27 (3·02–3·55)	2·41	34 500 (33 900–35 200)	21 600 (21 000–22 200)	15 900 (13 300–18 600)	6·87 (6·26–7·54)	5·29
	Belarus	746 (628–875)	328 (271–394)	244 (188–310)	2·38 (1·99–2·90)	1·51	1510 (1280–1780)	730 (607–884)	562 (437–729)	5·31 (4·44–6·46)	3·64
	Estonia	67·4 (62·2–72·9)	19·7 (17·5–22·1)	15·1 (12·4–18·4)	1·14 (0·980–1·35)	0·710	142 (132–152)	46·0 (40·9–51·5)	35·6 (29·5–43·4)	2·65 (2·27–3·14)	1·64
	Latvia	139 (127–152)	55·4 (50·2–60·3)	41·0 (33·9–49·5)	2·14 (1·86–2·50)	1·54	272 (256–288)	108 (98·3–117)	82·8 (69·3–99·3)	4·21 (3·63–4·95)	3·01
	Lithuania	156 (145–167)	70·8 (63·4–78·3)	48·6 (41·9–56·2)	1·80 (1·65–1·98)	1·20	369 (350–389)	155 (143–168)	110 (93·8–130)	4·00 (3·52–4·62)	2·83
	Moldova	734 (639–847)	374 (304–460)	278 (214–361)	8·64 (7·14–10·7)	6·69	1240 (1090–1430)	536 (438–647)	399 (315–505)	12·2 (10·2–14·7)	8·96
	Russia	12 400 (11 900–12 900)	7040 (6790–7270)	4990 (4010–6050)	3·00 (2·65–3·38)	2·19	24 500 (24 200–24 900)	15 200 (15 000–15 500)	11 200 (9190–13 400)	6·53 (5·75–7·41)	5·04
	Ukraine	3760 (3460–4080)	2360 (2130–2600)	1720 (1390–2120)	4·45 (3·89–5·12)	3·45	6440 (6110–6770)	4770 (4360–5180)	3500 (2920–4260)	8·76 (7·85–9·73)	7·05
**High income**		**47 600 (46 200–49 000)**	**35 400 (34 300–36 500)**	**31 200 (27 400–35 500)**	**2·78 (2·70–2·88)**	**2·39**	**88 900 (88 200–89 700)**	**63 500 (62 700–64 400)**	**55 600 (49 700–62 600)**	**4·95 (4·78–5·12)**	**4·14**
Australasia		1060 (1020–1110)	886 (847–927)	794 (677–931)	2·14 (2·03–2·26)	1·77	1980 (1930–2030)	1530 (1480–1580)	1380 (1200–1590)	3·73 (3·53–3·95)	2·96
	Australia	863 (824–902)	710 (680–744)	647 (558–751)	2·08 (1·98–2·18)	1·68	1550 (1510–1590)	1220 (1180–1250)	1110 (973–1260)	3·57 (3·41–3·76)	2·80
	New Zealand	199 (187–212)	175 (165–187)	147 (120–180)	2·46 (2·26–2·68)	2·21	431 (411–452)	313 (296–331)	270 (225–325)	4·53 (4·16–4·95)	3·80
High-income Asia Pacific		3830 (3530–4140)	1730 (1590–1870)	1430 (1290–1590)	1·04 (0·990–1·08)	0·810	9500 (9190–9820)	4440 (4240–4650)	3670 (3350–4000)	2·62 (2·52–2·71)	2·03
	Brunei	36·4 (32·0–41·1)	34·7 (30·8–39·2)	31·9 (24·4–41·7)	4·85 (4·09–5·73)	4·43	70·8 (63·2–79·0)	66·8 (59·4–75·0)	60·2 (46·7–77·5)	9·19 (7·73–10·9)	8·27
	Japan	2100 (1860–2370)	964 (880–1050)	782 (697–880)	0·870 (0·850–0·890)	0·640	5290 (5190–5410)	2740 (2650–2830)	2240 (2060–2450)	2·43 (2·36–2·51)	1·86
	Singapore	95·7 (86·6–106)	58·8 (44·2–78·8)	50·5 (35·2–71·0)	0·880 (0·770–1·00)	0·650	198 (183–215)	123 (96·5–157)	105 (79·1–140)	1·82 (1·60–2·09)	1·35
	South Korea	1600 (1450–1770)	673 (589–774)	567 (481–658)	1·37 (1·23–1·51)	1·15	3930 (3660–4200)	1500 (1370–1650)	1260 (1100–1450)	3·03 (2·82–3·26)	2·43
High-income North America		19 900 (18 700–21 000)	16 800 (15 800–17 700)	15 200 (14 000–16 500)	3·61 (3·55–3·67)	3·29	35 400 (35 200–35 700)	29 500 (29 200–29 800)	26 600 (24 600–28 700)	6·32 (6·18–6·47)	5·57
	Canada	1200 (1120–1280)	1220 (1140–1310)	1110 (996–1250)	2·98 (2·86–3·10)	2·66	2040 (2000–2090)	2010 (1960–2060)	1820 (1640–2010)	4·86 (4·67–5·07)	4·23
	Greenland	9·98 (8·56–11·5)	4·92 (3·88–6·22)	4·10 (2·66–6·22)	5·14 (3·74–6·97)	3·78	18·8 (15·9–22·0)	9·09 (7·09–11·6)	7·59 (4·97–11·4)	9·47 (6·85–12·9)	6·69
	USA†	18 700 (17 500–19 800)	15 500 (14 600–16 400)	14 000 (13 000–15 300)	3·67 (3·62–3·73)	3·36	33 400 (33 100–33 600)	27 500 (27 200–27 800)	24 700 (23 000–26 700)	6·46 (6·33–6·60)	5·71
Southern Latin America		9300 (9060–9520)	6180 (5950–6420)	5240 (4140–6640)	5·38 (5·08–5·72)	4·30	17 100 (16 900–17 300)	11 100 (10 900–11 300)	9370 (7600–11 600)	9·61 (9·09–10·2)	7·59
	Argentina	7380 (7150–7610)	4810 (4590–5020)	4120 (3300–5160)	5·89 (5·70–6·10)	4·67	13 400 (13 200–13 500)	8710 (8580–8840)	7420 (6110–9050)	10·6 (10·3–11·0)	8·37
	Chile	1420 (1360–1500)	1140 (1090–1180)	907 (686–1200)	3·98 (3·41–4·65)	3·31	2840 (2750–2930)	1960 (1880–2030)	1560 (1200–2020)	6·84 (5·87–7·99)	5·44
	Uruguay	493 (442–545)	240 (207–276)	211 (154–286)	4·54 (3·97–5·19)	3·37	884 (818–955)	439 (395–486)	388 (289–516)	8·29 (7·26–9·48)	6·11
Western Europe		13 500 (13 000–14 000)	9810 (9300–10 300)	8550 (7370–9960)	2·00 (1·91–2·10)	1·61	24 900 (24 700–25 200)	17 000 (16 600–17 300)	14 700 (12 900–16 800)	3·42 (3·29–3·57)	2·69
	Andorra	1·11 (0·900–1·35)	0·585 (0·469–0·729)	0·516 (0·384–0·674)	0·820 (0·690–0·980)	0·540	2·59 (2·06–3·15)	1·30 (1·06–1·62)	1·11 (0·843–1·43)	1·77 (1·48–2·10)	1·16
	Austria	238 (217–257)	186 (170–201)	166 (141–192)	1·90 (1·69–2·10)	1·52	445 (427–463)	307 (294–322)	282 (252–316)	3·22 (3·03–3·42)	2·50
	Belgium	343 (303–387)	258 (222–291)	230 (189–279)	1·89 (1·78–2·01)	1·48	690 (666–715)	480 (457–502)	423 (354–505)	3·48 (3·27–3·71)	2·68
	Cyprus	43·4 (38·7–48·6)	28·7 (24·4–33·3)	27·3 (19·9–36·6)	1·80 (1·42–2·24)	1·31	77·3 (69·6–85·7)	49·3 (42·2–57·1)	47·9 (35·4–63·2)	3·17 (2·52–3·94)	2·32
	Denmark	216 (187–245)	157 (143–171)	145 (118–179)	2·31 (2·12–2·52)	1·90	371 (348–392)	237 (221–255)	218 (179–264)	3·48 (3·20–3·79)	2·73
	Finland	136 (124–149)	65·4 (59·4–71·9)	58·9 (49·3–70·5)	1·18 (1·08–1·29)	0·860	244 (230–258)	125 (117–134)	110 (94·0–130)	2·20 (2·01–2·41)	1·63
	France	2150 (1910–2370)	1740 (1590–1900)	1480 (1270–1720)	2·05 (1·95–2·16)	1·72	4160 (4080–4250)	3110 (3040–3190)	2600 (2280–2960)	3·60 (3·42–3·79)	2·87
	Germany	2110 (1920–2280)	1610 (1490–1730)	1440 (1320–1580)	1·95 (1·88–2·03)	1·64	4120 (4050–4190)	2730 (2660–2790)	2450 (2250–2670)	3·33 (3·21–3·47)	2·63
	Greece	390 (361–416)	242 (223–262)	188 (152–233)	2·17 (1·99–2·38)	1·54	643 (617–671)	484 (459–508)	339 (279–413)	3·85 (3·53–4·21)	2·87
	Iceland	9·77 (8·00–11·8)	6·69 (5·23–8·58)	6·28 (3·84–9·95)	1·45 (1·04–2·00)	1·20	15·9 (13·1–19·0)	10·9 (8·48–13·8)	9·95 (6·21–15·5)	2·31 (1·65–3·20)	1·84
	Ireland	221 (201–241)	157 (141–175)	124 (97·7–157)	2·04 (1·88–2·21)	1·60	385 (364–407)	253 (234–274)	200 (160–249)	3·25 (3·00–3·54)	2·48
	Israel	483 (425–544)	369 (325–413)	331 (265–408)	1·72 (1·65–1·79)	1·27	920 (896–944)	675 (656–696)	609 (508–724)	3·18 (3·06–3·30)	2·37
	Italy	1710 (1510–1890)	982 (906–1050)	770 (680–878)	1·75 (1·69–1·81)	1·20	2980 (2930–3030)	1710 (1670–1750)	1320 (1190–1480)	2·98 (2·88–3·08)	2·14
	Luxembourg	14·0 (11·7–16·5)	9·02 (7·20–11·1)	8·56 (5·35–13·3)	1·32 (0·930–1·83)	1·00	27·3 (23·4–31·9)	16·7 (13·5–20·6)	15·5 (9·89–23·7)	2·42 (1·71–3·38)	1·84
	Malta	19·7 (17·2–22·4)	18·7 (15·2–22·7)	16·3 (10·8–24·0)	3·80 (2·96–4·83)	3·08	31·1 (27·4–35·3)	27·7 (23·1–33·1)	24·7 (16·8–35·7)	5·74 (4·54–7·23)	4·68
	Monaco	0·477 (0·323–0·667)	0·320 (0·229–0·433)	0·287 (0·220–0·367)	1·02 (0·850–1·23)	0·830	1·26 (0·895–1·69)	0·808 (0·606–1·05)	0·727 (0·560–0·925)	2·58 (2·15–3·10)	2·03
	Netherlands	777 (723–828)	425 (383–469)	421 (345–513)	2·37 (2·26–2·49)	1·94	1280 (1240–1310)	674 (653–697)	659 (550–790)	3·72 (3·54–3·91)	2·96
	Norway	155 (145–164)	92·9 (87·3–99·0)	80·1 (68·0–94·5)	1·41 (1·32–1·51)	1·10	284 (272–297)	166 (157–174)	142 (123–164)	2·50 (2·34–2·67)	1·93
	Portugal	405 (363–445)	178 (154–202)	129 (100–166)	1·61 (1·49–1·75)	1·08	841 (802–884)	313 (289–340)	229 (181–286)	2·82 (2·60–3·06)	1·78
	San Marino	0·977 (0·724–1·27)	0·655 (0·493–0·861)	0·606 (0·466–0·773)	1·95 (1·62–2·33)	1·57	1·83 (1·35–2·39)	1·23 (0·928–1·61)	1·13 (0·873–1·44)	3·63 (3·03–4·36)	2·85
	Spain	1090 (983–1200)	760 (680–833)	603 (493–731)	1·63 (1·39–1·86)	1·20	2130 (2090–2170)	1400 (1360–1430)	1130 (996–1290)	2·98 (2·88–3·10)	2·19
	Sweden	193 (182–203)	180 (153–208)	165 (141–191)	1·41 (1·27–1·54)	1·12	357 (341–375)	335 (317–352)	302 (265–345)	2·58 (2·38–2·81)	2·07
	Switzerland	269 (244–294)	263 (242–284)	227 (200–259)	2·57 (2·43–2·72)	2·26	457 (440–475)	375 (358–392)	323 (286–365)	3·66 (3·46–3·88)	3·04
	UK†	2510 (2420–2610)	2070 (1800–2290)	1920 (1590–2310)	2·45 (2·14–2·76)	2·09	4440 (4350–4530)	3470 (3400–3550)	3210 (2810–3660)	4·10 (3·97–4·25)	3·41
**Latin America and Caribbean**		**181 000 (164 000–198 000)**	**112 000 (94 800–131 000)**	**93 900 (74 900–116 000)**	**9·56 (8·28–11·1)**	**7·77**	**397 000 (369 000–427 000)**	**226 000 (192 000–263 000)**	**187 000 (149 000–231 000)**	**19·0 (16·2–22·3)**	**14·3**
Andean Latin America		22 700 (20 100–25 400)	14 900 (12 400–17 800)	12 600 (9620–16 300)	9·42 (8·38–10·6)	7·58	56 000 (51 000–61 500)	29 600 (25 000–34 800)	24 900 (19 200–31 900)	18·6 (16·5–21·1)	14·3
	Bolivia	6710 (5980–7470)	5560 (4680–6610)	4840 (3880–5990)	14·8 (12·6–17·7)	12·2	18 300 (16 500–20 100)	11 400 (9660–13 400)	9630 (7800–11 800)	29·5 (25·2–35·1)	22·7
	Ecuador	5400 (4430–6510)	3050 (2290–3990)	2720 (1870–3830)	7·74 (6·19–9·65)	6·22	11 300 (9570–13 100)	5980 (4730–7430)	5300 (3790–7240)	15·1 (12·7–18·1)	11·5
	Peru	10 600 (9120–12 200)	6290 (4750–8220)	5010 (3550–6960)	7·61 (6·37–9·11)	5·92	26 400 (23 100–29 700)	12 200 (9430–15 400)	9980 (7070–13 800)	15·1 (12·6–18·1)	11·4
Caribbean		18 100 (16 500–19 800)	17 200 (14 200–20 300)	15 800 (12 300–20 000)	19·3 (15·8–23·3)	16·5	44 900 (40 800–49 200)	36 300 (31 000–42 500)	32 000 (26 000–39 000)	38·8 (33·2–45·3)	28·9
	Antigua and Barbuda	13·4 (10·8–16·4)	6·15 (4·56–8·09)	5·34 (3·80–7·37)	5·35 (4·61–6·19)	4·47	20·9 (16·9–25·4)	12·2 (9·04–16·2)	10·4 (7·22–14·6)	10·3 (8·61–12·3)	8·97
	The Bahamas	38·0 (31·0–45·1)	28·1 (20·4–39·2)	24·7 (18·5–32·7)	6·08 (4·94–7·62)	5·32	80·5 (67·4–94·1)	53·8 (41·1–71·1)	48·2 (37·0–62·2)	11·7 (9·94–14·2)	10·3
	Barbados	40·6 (33·5–48·9)	27·0 (20·5–34·8)	24·7 (17·0–35·3)	8·64 (7·24–10·3)	7·85	57·4 (47·5–68·7)	38·0 (29·2–48·6)	34·9 (24·1–49·7)	12·2 (10·2–14·5)	10·9
	Belize	87·6 (76·5–101)	72·9 (60·3–86·7)	69·1 (56·4–83·9)	9·13 (7·97–10·6)	8·21	174 (150–202)	124 (101–147)	116 (92·3–144)	15·4 (13·0–18·6)	12·4
	Bermuda	2·43 (1·91–3·08)	1·66 (1·32–2·05)	1·40 (0·971–1·95)	2·71 (2·24–3·28)	2·31	4·76 (3·94–5·80)	2·96 (2·39–3·63)	2·44 (1·76–3·34)	4·66 (3·92–5·54)	3·72
	Cuba	608 (553–664)	330 (294–368)	236 (188–291)	2·26 (1·98–2·59)	1·67	1260 (1210–1310)	703 (666–741)	503 (409–608)	4·74 (4·12–5·43)	3·40
	Dominica	15·8 (12·6–19·4)	14·6 (11·2–18·6)	13·5 (9·43–18·9)	16·2 (13·6–19·3)	19·0	25·8 (20·6–31·5)	23·6 (18·3–30·1)	21·7 (15·3–30·2)	26·0 (21·8–31·0)	29·7
	Dominican Republic	5570 (4700–6490)	4450 (3460–5560)	3910 (2800–5370)	16·9 (14·1–20·2)	14·8	10 100 (8860–11 400)	6740 (5430–8290)	5850 (4230–7950)	25·2 (21·1–30·2)	20·4
	Grenada	19·8 (15·0–25·6)	14·1 (10·4–18·9)	12·1 (8·43–17·1)	8·62 (7·26–10·2)	7·58	35·4 (27·5–44·3)	23·1 (17·1–30·9)	19·5 (13·6–27·5)	13·8 (11·6–16·5)	11·7
	Guyana	418 (354–490)	241 (179–319)	217 (153–303)	15·0 (12·6–17·9)	12·7	692 (606–777)	377 (278–495)	333 (236–462)	23·1 (19·4–27·4)	19·1
	Haiti	8840 (7850–9890)	10 300 (8020–12 900)	9810 (7690–12 200)	29·5 (23·0–36·8)	24·4	28 200 (24 900–31 800)	25 400 (21 600–29 900)	22 600 (18 800–27 000)	68·3 (58·7–79·5)	47·8
	Jamaica	732 (578–913)	525 (407–670)	454 (320–627)	12·6 (10·6–15·0)	11·7	1010 (807–1240)	656 (513–839)	568 (403–785)	15·7 (13·1–18·7)	13·4
	Puerto Rico	432 (399–467)	150 (133–167)	128 (94·5–171)	5·03 (4·27–5·92)	4·17	650 (617–686)	239 (223–258)	197 (148–259)	7·67 (6·52–9·04)	6·49
	Saint Kitts and Nevis	12·2 (10·3–14·5)	8·43 (6·57–10·8)	7·10 (5·55–9·00)	10·2 (8·82–11·9)	8·77	18·9 (16·1–22·2)	12·7 (9·88–16·1)	10·7 (8·32–13·6)	15·3 (13·1–17·9)	11·4
	Saint Lucia	38·1 (30·9–46·0)	22·6 (16·5–30·8)	18·9 (12·9–27·1)	10·6 (8·89–12·6)	9·87	55·9 (45·5–67·7)	31·4 (22·9–42·9)	26·3 (18·0–37·4)	14·6 (12·2–17·4)	13·0
	Saint Vincent and the Grenadines	35·3 (29·0–41·9)	18·1 (13·8–23·7)	14·8 (10·5–20·5)	9·74 (8·26–11·5)	8·49	55·2 (44·3–68·0)	28·4 (21·6–37·3)	23·4 (16·4–32·8)	15·2 (12·8–18·2)	12·5
	Suriname	246 (210–287)	182 (146–224)	155 (109–215)	16·8 (14·1–20·0)	13·8	414 (353–481)	281 (228–344)	238 (169–326)	25·7 (21·6–30·6)	20·4
	Trinidad and Tobago	327 (274–393)	185 (140–238)	156 (110–218)	10·1 (8·48–12·1)	8·84	478 (397–575)	281 (219–354)	238 (169–329)	15·2 (12·7–18·2)	13·1
	Virgin Islands	14·9 (12·2–17·8)	7·08 (5·50–8·98)	5·90 (4·21–8·11)	4·62 (3·91–5·46)	3·57	22·7 (18·8–27·2)	10·6 (8·16–13·3)	8·76 (6·28–12·0)	6·79 (5·74–8·05)	5·26
Central Latin America		70 000 (61 400–79 900)	40 600 (34 000–48 200)	33 200 (26 400–41 200)	7·50 (6·65–8·47)	6·02	143 000 (130 000–157 000)	80 100 (67 100–94 100)	65 400 (50 700–83 700)	14·8 (12·4–17·5)	11·2
	Colombia	13 400 (11 300–15 700)	6610 5020–8340)	5410 (3660–7660)	6·68 (5·27–8·35)	5·21	24 900 (22 000–28 200)	12 500 (9900–15 400)	10 300 (7410–13 900)	12·6 (10·6–15·0)	9·85
	Costa Rica	553 (503–606)	412 (375–452)	338 (242–466)	5·07 (4·33–5·94)	4·32	959 (889–1040)	641 (589–695)	532 (388–717)	7·93 (6·76–9·30)	6·59
	El Salvador	1770 (1400–2150)	792 (606–1020)	593 (412–835)	5·26 (4·11–6·59)	3·86	4290 (3730–4950)	1680 (1340–2100)	1240 (896–1690)	10·9 (9·18–13·1)	7·90
	Guatemala	7130 (6170–8200)	3830 (3090–4680)	3440 (2460–4720)	8·35 (7·07–9·86)	6·22	18 100 (16 000–20 300)	10 400 (8520–12 500)	8870 (6410–12 100)	21·7 (18·3–25·8)	14·8
	Honduras	3300 (2790–3850)	2500 (1990–3110)	2180 (1710–2720)	9·33 (7·81–11·1)	7·53	6880 (5900–7990)	4570 (3650–5630)	3970 (3140–4940)	17·2 (14·4–20·5)	12·9
	Mexico	35 300 (28 000–43 400)	19 200 (15 400–24 200)	15 700 (12 100–20 200)	7·44 (6·52–8·53)	6·12	69 800 (61 700–78 900)	37 400 (30 500–45 200)	30 300 (24 200–37 500)	14·4 (12·2–17·1)	11·2
	Nicaragua	1930 (1580–2280)	1070 (823–1360)	860 (653–1100)	6·58 (5·40–8·01)	4·84	4570 (3930–5230)	2350 (1930–2830)	1880 (1530–2300)	14·4 (12·1–17·1)	10·0
	Panama	617 (509–741)	539 (447–646)	490 (355–665)	6·42 (5·39–7·64)	4·98	1430 (1200–1700)	1210 (1000–1440)	1100 (818–1460)	14·5 (12·1–17·3)	11·5
	Venezuela	5990 (5650–6320)	5640 (4850–6590)	4240 (3000–5870)	8·83 (7·42–10·5)	7·64	11 700 (11 100–12 400)	9370 (8140–10 800)	7220 (5200–9880)	14·8 (12·4–17·6)	11·8
Tropical Latin America		70 000 (61 000–79 000)	39 600 (32 300–47 300)	32 300 (25 500–39 700)	9·96 (8·41–11·8)	8·10	154 000 (138 000–169 000)	79 800 (66 800–95 400)	64 600 (51 300–79 200)	19·8 (16·7–23·4)	15·1
	Brazil†	68 100 (59 200–77 200)	38 700 (31 500–46 500)	31 600 (24 900–38 700)	10·1 (8·55–12·0)	8·26	150 000 (135 000–166 000)	77 700 (65 100–92 700)	62 800 (49 900–77 000)	20·0 (16·9–23·7)	15·3
	Paraguay	1900 (1590–2220)	853 (648–1100)	733 (525–1010)	5·80 (4·88–6·90)	4·53	3490 (2950–4040)	2060 (1580–2650)	1770 (1280–2410)	14·0 (11·8–16·6)	12·0
**North Africa and Middle East**		**298 000 (268 000–328 000)**	**182 000 (159 000–210 000)**	**150 000 (129 000–173 000)**	**12·2 (11·1–13·3)**	**9·82**	**682 000 (623 000–742 000)**	**382 000 (333 000–442 000)**	**300 000 (255 000–353 000)**	**24·4 (22·3–26·7)**	**18·2**
	Afghanistan	38 900 (32 600–45 400)	34 900 (29 100–41 100)	37 400 (31 300–44 200)	25·0 (21·6–28·4)	19·5	120 000 (108 000–133 000)	83 800 (71 000–99 000)	81 400 (67 900–97 200)	55·3 (47·9–63·5)	37·2
	Algeria	15 600 (12 800–18 600)	13 600 (11 200–16 100)	10 700 (8220–13 400)	12·0 (9·83–14·4)	10·4	29 400 (25 400–33 700)	22 200 (19 000–25 900)	17 300 (14 500–20 500)	19·5 (17·0–22·4)	16·0
	Bahrain	64·5 (57·6–72·6)	42·9 (35·9–51·4)	30·7 (24·6–38·7)	2·36 (2·13–2·59)	1·57	158 (142–175)	126 (107–147)	87·3 (69·8–109)	6·53 (5·79–7·36)	5·02
	Egypt	40 500 (33 400–47 600)	15 400 (12 000–20 100)	11 800 (8250–16 200)	5·55 (4·20–7·20)	3·11	84 400 (72 600–96 300)	47 400 (38 200–57 600)	32 600 (24 600–42 600)	15·3 (12·8–18·3)	8·27
	Iran	33 700 (27 500–40 300)	18 000 (15 500–20 800)	9140 (7440–11 100)	6·77 (6·09–7·44)	5·19	60 800 (50 200–72 500)	29 100 (25 500–33 200)	15 200 (12 700–18 400)	11·1 (10·2–12·0)	7·83
	Iraq	22 100 (19 500–25 000)	12 300 (9300–16 300)	9130 (6610–12 800)	9·49 (7·92–11·5)	7·51	40 700 (35 800–46 000)	23 000 (18 100–29 100)	15 000 (11 000–20 700)	15·7 (13·2–18·9)	11·9
	Jordan	2160 (1830–2520)	2070 (1610–2660)	2130 (1570–2930)	8·80 (7·46–10·6)	6·72	3610 (3040–4220)	3540 (2750–4520)	3640 (2680–5010)	15·3 (13·0–18·3)	11·3
	Kuwait	262 (234–292)	369 (324–422)	310 (242–402)	5·09 (4·30–5·99)	4·46	500 (463–545)	659 (599–730)	555 (440–708)	9·18 (7·75–10·8)	8·03
	Lebanon	1050 (813–1330)	684 (497–938)	521 (373–734)	4·82 (4·02–5·89)	3·65	1920 (1510–2420)	1290 (958–1730)	983 (708–1380)	8·99 (7·51–11·0)	6·99
	Libya	1650 (1350–1990)	560 (436–712)	458 (351–583)	5·62 (4·73–6·69)	4·43	3240 (2640–3890)	1470 (1180–1800)	1110 (869–1390)	13·3 (11·4–15·7)	10·4
	Morocco	22 200 (17 600–27 300)	9820 (7510–12 500)	6760 (5120–8700)	11·1 (9·74–12·5)	7·92	40 800 (33 500–48 200)	16 200 (11 700–21 900)	11 100 (7860–15 400)	17·9 (15·0–21·4)	11·9
	Oman	478 (408–553)	504 (445–564)	418 (357–482)	5·38 (4·86–5·90)	4·25	926 (792–1070)	958 (842–1070)	809 (690–937)	10·4 (9·40–11·4)	8·17
	Palestine	1720 (1450–2000)	1050 (803–1320)	800 (606–1060)	6·36 (5·38–7·63)	5·67	3750 (3350–4160)	2060 (1620–2580)	1560 (1180–2060)	12·4 (10·5–14·8)	10·1
	Qatar	107 (85·9–133)	127 (103–155)	113 (90·6–144)	4·22 (3·52–5·13)	3·45	199 (165–239)	239 (195–291)	214 (172–274)	8·02 (6·71–9·74)	6·62
	Saudi Arabia	5910 (4690–7240)	1670 (1320–2100)	1200 (950–1520)	2·64 (2·20–3·24)	1·48	13 000 (9880–16 500)	3830 (3060–4750)	2610 (2070–3300)	5·73 (4·77–7·01)	3·39
	Sudan	43 600 (36 800–50 700)	30 700 (24 800–37 300)	25 700 (20 600–32 500)	21·3 (18·9–24·1)	17·0	124 000 (111 000–137 000)	65 100 (51 100–81 800)	50 700 (37 300–68 600)	41·9 (35·7–50·0)	30·0
	Syria	5300 (4670–5950)	3320 (2850–3830)	1590 (1320–1900)	6·88 (5·72–8·32)	5·92	10 800 (10 000–11 700)	8940 (7980–10 100)	3210 (2560–3990)	13·6 (11·7–15·8)	12·0
	Tunisia	3590 (2900–4370)	1610 (1260–2010)	1140 (903–1420)	6·82 (5·73–8·12)	5·33	6140 (5160–7180)	2700 (2170–3290)	1920 (1530–2380)	11·3 (9·49–13·5)	8·45
	Turkey	28 200 (23 400–33 500)	10 900 (8610–13 800)	8380 (6710–10 400)	8·54 (7·15–10·2)	6·62	61 200 (51 700–71 300)	19 500 (15 500–24 300)	15 100 (12 100–18 700)	15·4 (12·9–18·4)	11·4
	United Arab Emirates	305 (269–341)	227 (173–298)	145 (109–197)	2·58 (2·18–3·10)	2·40	587 (532–641)	455 (352–599)	295 (220–399)	4·96 (4·19–5·97)	4·37
	Yemen	30 100 (25 300–35 200)	23 900 (19 300–29 200)	21 600 (17 700–26 000)	22·8 (18·8–27·4)	18·7	75 800 (67 600–84 400)	49 200 (40 800–58 800)	44 200 (36 700–53 100)	46·7 (40·2–54·4)	35·1
**South Asia**		**1 560 000 (1 410 000–1 730 000)**	**1 110 000 (958 000–1 280 000)**	**899 000 (761 000–1 060 000)**	**26·9 (24·2–30·0)**	**21·8**	**3 040 000 (2 780 000–3 330 000)**	**1 760 000 (1 510 000–2 020 000)**	**1 360 000 (1 140 000–1 610 000)**	**40·5 (36·0–46·0)**	**29·0**
	Bangladesh	146 000 (130 000–163 000)	70 100 (54 500–87 600)	52 600 (40 400–66 300)	19·8 (16·4–23·7)	14·2	295 000 (267 000–325 000)	108 000 (90 000–128 000)	77 900 (63 000–94 200)	29·2 (24·9–34·2)	18·4
	Bhutan	796 (661–929)	379 (279–499)	287 (207–393)	20·6 (17·5–24·2)	15·0	1580 (1320–1830)	602 (443–788)	445 (322–602)	31·5 (26·7–36·9)	22·2
	India	1 070 000 (938 000–1 220 000)	720 000 (606 000–844 000)	558 000 (456 000–689 000)	23·8 (20·1–28·8)	18·8	2 130 000 (1 900 000–2 380 000)	1 150 000 (970 000–1 350 000)	841 000 (692 000–1 040 000)	35·8 (30·2–43·0)	24·6
	Nepal	32 500 (28 500–36 700)	14 700 (12 000–18 000)	11 700 (9610–14 100)	19·0 (15·6–23·0)	13·5	65 900 (59 300–73 100)	24 200 (20 500–28 600)	18 000 (14 700–21 800)	29·1 (24·8–34·1)	18·9
	Pakistan	309 000 (264 000–356 000)	305 000 (246 000–374 000)	277 000 (226 000–333 000)	41·6 (34·2–50·0)	34·5	552 000 (495 000–614 000)	478 000 (395 000–569 000)	420 000 (346 000–507 000)	63·3 (53·9–74·3)	48·0
**Southeast Asia, east Asia, and Oceania**		**507 000 (471 000–543 000)**	**250 000 (215 000–288 000)**	**194 000 (162 000–230 000)**	**7·23 (6·29–8·27)**	**6·17**	**1 280 000 (1 200 000–1 380 000)**	**519 000 (456 000–590 000)**	**405 000 (349 000–467 000)**	**14·8 (13·2–16·4)**	**11·7**
East Asia		243 000 (222 000–263 000)	86 700 (77 800–96 600)	59 300 (51 500–67 700)	3·83 (3·37–4·37)	2·89	652 000 (594 000–717 000)	185 000 (166 000–206 000)	136 000 (119 000–155 000)	8·56 (7·52–9·76)	5·89
	China	229 000 (208 000–249 000)	82 700 (74 000–92 300)	56 400 (49 100–64 400)	3·78 (3·31–4·31)	2·77	610 000 (556 000–666 000)	177 000 (158 000–198 000)	131 000 (114 000–149 000)	8·52 (7·45–9·71)	5·70
	North Korea	13 100 (9580–18 000)	3510 (2720–4470)	2420 (1920–3110)	6·91 (5·74–8·45)	5·85	39 200 (25 000–69 700)	6430 (5000–8140)	4460 (3530–5680)	12·6 (10·5–15·4)	9·55
	Taiwan (province of China)	967 (889–1050)	532 (498–570)	405 (330–497)	2·31 (1·97–2·75)	2·30	2300 (2230–2380)	1030 (988–1080)	815 (672–998)	4·51 (3·85–5·38)	3·98
Oceania		5900 (5080–6800)	7360 (5910–9040)	7540 (5970–9450)	18·1 (15·2–21·4)	15·4	17 400 (15 000–20 000)	20 200 (16 300–24 700)	19 900 (15 800–25 000)	48·4 (40·7–57·5)	39·0
	American Samoa	12·2 (10·5–14·0)	5·67 (4·58–6·85)	5·14 (3·94–6·47)	4·86 (4·23–5·52)	3·88	24·6 (20·7–28·9)	12·2 (9·61–15·3)	10·8 (7·95–14·4)	10·2 (8·72–12·0)	7·72
	Cook Islands	2·21 (1·71–2·81)	0·412 (0·326–0·519)	0·303 (0·243–0·375)	1·10 (0·930–1·31)	0·940	5·00 (3·87–6·33)	0·942 (0·742–1·20)	0·685 (0·547–0·848)	2·46 (2·06–2·94)	1·98
	Federated States of Micronesia	42·2 (34·0–51·4)	16·4 (12·7–20·5)	13·4 (10·4–16·9)	6·85 (5·72–8·22)	5·05	105 (87·3–125)	35·6 (27·8–44·4)	28·4 (22·1–35·9)	14·5 (12·1–17·4)	10·5
	Fiji	217 (172–272)	198 (152–255)	177 (128–241)	10·0 (8·41–12·0)	8·70	471 (376–590)	432 (338–554)	387 (281–521)	21·7 (18·2–25·9)	18·8
	Guam	20·2 (18·1–22·5)	24·6 (21·6–28·1)	23·6 (18·8–29·6)	7·18 (6·16–8·40)	7·31	41·3 (36·8–46·1)	46·0 (41·5–51·3)	43·9 (35·6–53·7)	13·3 (11·6–15·3)	12·6
	Kiribati	54·1 (44·8–65·3)	48·5 (37·0–63·4)	44·5 (32·3–60·2)	14·6 (12·3–17·5)	12·0	157 (130–190)	116 (88·7–151)	101 (74·0–137)	33·6 (28·1–40·0)	25·4
	Marshall Islands	24·5 (19·7–29·6)	13·9 (11·1–17·6)	11·7 (9·32–15·1)	9·38 (7·84–11·4)	7·74	57·9 (49·9–66·6)	29·2 (23·5–36·5)	24·3 (19·4–31·0)	19·3 (16·2–23·4)	16·9
	Nauru	5·85 (4·75–7·19)	3·71 (2·96–4·61)	3·04 (2·45–3·89)	10·1 (8·44–12·2)	8·58	17·7 (15·2–20·3)	8·94 (7·27–11·0)	7·03 (5·65–8·95)	23·2 (19·5–28·1)	18·7
	Niue	0·415 (0·313–0·540)	0·224 (0·173–0·284)	0·205 (0·160–0·259)	8·12 (6·81–9·68)	6·92	1·01 (0·775–1·28)	0·520 (0·399–0·656)	0·472 (0·367–0·595)	18·6 (15·5–22·2)	15·0
	Northern Mariana Islands	9·67 (7·70–11·8)	2·73 (2·25–3·28)	2·22 (1·82–2·69)	4·40 (3·60–5·29)	3·89	17·5 (14·0–21·3)	6·00 (4·77–7·39)	4·69 (3·68–5·83)	9·11 (7·73–10·7)	8·07
	Palau	3·22 (2·44–4·16)	1·52 (1·15–1·97)	1·15 (0·876–1·53)	6·04 (5·05–7·38)	5·15	7·78 (5·78–10·3)	3·72 (2·82–4·76)	2·77 (2·11–3·66)	14·0 (11·7–17·0)	11·3
	Papua New Guinea	4690 (4010–5470)	6280 (5020–7740)	6530 (5180–8160)	19·6 (16·5–23·3)	16·8	14 400 (12 300–16 700)	17 600 (14 100–21 600)	17 600 (14 000–22 000)	53·8 (45·3–63·9)	43·4
	Samoa	30·9 (21·8–42·2)	24·0 (17·5–32·9)	22·3 (15·8–31·0)	6·28 (5·24–7·52)	5·35	65·1 (47·2–86·9)	49·9 (36·6–67·4)	46·4 (32·8–64·1)	13·2 (11·1–15·9)	11·4
	Solomon Islands	325 (261–398)	272 (216–333)	245 (197–301)	11·5 (9·65–13·8)	9·36	767 (620–935)	593 (474–728)	519 (421–638)	24·6 (20·6–29·4)	19·2
	Tokelau	0·406 (0·322–0·506)	0·139 (0·110–0·176)	0·119 (0·0935–0·149)	3·19 (2·54–3·97)	2·71	0·961 (0·767–1·19)	0·338 (0·265–0·420)	0·286 (0·229–0·351)	7·73 (6·45–9·28)	6·24
	Tonga	26·9 (22·0–32·6)	17·5 (13·5–22·8)	14·5 (10·5–19·7)	6·36 (5·31–7·61)	5·37	56·4 (46·7–67·1)	38·0 (29·4–48·7)	31·4 (22·9–42·3)	13·6 (11·4–16·3)	13·7
	Tuvalu	4·62 (4·00–5·32)	1·70 (1·26–2·22)	1·42 (1·06–1·93)	6·63 (5·51–8·10)	5·65	10·6 (9·00–12·5)	3·50 (2·66–4·52)	2·92 (2·19–3·90)	13·7 (11·4–16·7)	11·0
	Vanuatu	99·6 (78·1–125)	93·4 (75·2–115)	88·3 (70·4–110)	11·6 (9·68–13·8)	9·73	223 (175–277)	201 (163–246)	185 (148–228)	24·4 (20·5–29·2)	20·0
Southeast Asia		259 000 (233 000–283 000)	156 000 (129 000–187 000)	127 000 (102 000–156 000)	11·6 (9·92–13·6)	9·17	615 000 (563 000–665 000)	314 000 (269 000–367 000)	250 000 (209 000–297 000)	22·6 (20·1–25·6)	16·5
	Cambodia	12 200 (11 100–13 300)	7330 (5800–9240)	6280 (4810–8280)	16·9 (14·4–20·3)	13·1	35 200 (31 600–38 900)	14 200 (11 200–17 800)	11 600 (8890–15 300)	31·3 (26·5–37·5)	21·6
	Indonesia	115 000 (102 000–129 000)	66 500 (50 800–83 100)	52 400 (40 900–65 500)	13·7 (10·9–17·0)	10·5	260 000 (236 000–283 000)	129 000 (106 000–153 000)	98 900 (80 600–121 000)	25·5 (21·6–30·2)	17·8
	Laos	7790 (6960–8770)	3960 (3270–4670)	3470 (2760–4280)	19·9 (16·9–23·2)	12·8	21 600 (19 600–23 900)	8680 (7220–10 300)	7100 (5650–8900)	40·9 (35·0–47·5)	18·8
	Malaysia	2460 (2240–2680)	2340 (2060–2590)	1910 (1540–2330)	3·55 (3·01–4·20)	2·82	4840 (4720–4980)	4270 (4160–4380)	3410 (2790–4150)	6·42 (5·43–7·59)	4·91
	Maldives	134 (112–159)	99·9 (76·4–131)	81·9 (60·0–113)	9·58 (8·05–11·5)	8·08	241 (205–283)	169 (131–217)	140 (103–192)	16·2 (13·7–19·4)	13·6
	Mauritius	228 (208–249)	120 (109–132)	104 (77·5–136)	8·07 (6·90–9·39)	6·79	349 (326–372)	192 (177–206)	163 (124–211)	12·6 (10·8–14·7)	10·7
	Myanmar	48 300 (40 000–57 000)	26 600 (21 200–32 400)	22 200 (16 300–28 700)	21·0 (17·3–24·8)	16·2	135 000 (117 000–156 000)	55 000 (43 800–67 900)	42 800 (32 100–56 300)	40·3 (34·3–47·1)	28·6
	Philippines	37 800 (32 500–44 100)	31 200 (21 800–41 700)	27 400 (19 700–36 400)	10·2 (7·91–13·0)	8·59	87 100 (77 600–97 500)	69 000 (55 700–84 400)	60 000 (47 800–73 200)	22·6 (19·0–26·7)	17·5
	Seychelles	13·6 (11·7–15·7)	12·7 (10·5–15·0)	10·7 (8·09–14·0)	7·15 (6·18–8·26)	6·12	20·6 (17·7–23·7)	20·3 (16·8–24·2)	17·3 (13·1–22·6)	11·5 (9·89–13·4)	9·79
	Sri Lanka	3300 (3050–3560)	1750 (1430–2130)	1300 (882–1860)	4·37 (3·46–5·45)	3·16	5880 (5510–6240)	3060 (2560–3630)	2290 (1640–3150)	7·61 (6·38–9·08)	5·38
	Thailand	7780 (6400–9410)	2790 (2380–3230)	2120 (1660–2670)	3·63 (2·99–4·26)	2·39	16 100 (13 800–19 000)	6030 (5510–6560)	4570 (3760–5480)	7·63 (6·84–8·48)	4·97
	Timor-Leste	966 (869–1080)	622 (512–748)	602 (486–741)	15·5 (13·1–18·6)	12·4	3150 (2790–3550)	1340 (1110–1610)	1230 (1000–1520)	32·1 (27·2–38·5)	21·0
	Vietnam	22 200 (19 400–25 200)	12 300 (9830–15 400)	9200 (7010–12 300)	6·83 (5·76–8·27)	5·21	44 300 (39 400–49 800)	22 600 (18 100–28 400)	17 100 (13 000–22 700)	12·4 (10·5–15·0)	9·42
**Sub-Saharan Africa**		**1 120 000 (1 050 000–1 190 000)**	**1 090 000 (938 000–1 270 000)**	**1 020 000 (847 000–1 250 000)**	**27·9 (24·7–31·6)**	**23·6**	**4 020 000 (3 790 000–4 270 000)**	**3 070 000 (2 640 000–3 550 000)**	**2 680 000 (2 220 000–3 250 000)**	**74·1 (65·3–85·2)**	**54·4**
Central sub-Saharan Africa		124 000 (111 000–137 000)	114 000 (98 400–132 000)	100 000 (85 800–120 000)	22·5 (19·8–25·8)	17·7	509 000 (468 000–553 000)	333 000 (289 000–387 000)	260 000 (222 000–310 000)	58·8 (51·7–67·5)	36·5
	Angola	28 700 (25 200–32 100)	26 200 (21 900–31 100)	24 000 (20 100–28 400)	21·7 (19·1–24·5)	16·9	116 000 (105 000–127 000)	73 000 (60 900–85 700)	58 800 (48 100–70 800)	54·2 (46·4–62·9)	33·7
	Central African Republic	7730 (6570–8970)	8060 (6320–10 200)	7770 (6150–9930)	39·3 (33·2–47·4)	35·4	28 300 (24 500–32 100)	26 900 (21 900–32 600)	24 000 (19 200–30 000)	123 (105–146)	95·2
	Congo (Brazzaville)	3400 (2910–3940)	3240 (2770–3770)	2680 (2250–3170)	18·4 (16·0–21·0)	15·3	12 100 (10 900–13 400)	7540 (6410–8770)	5760 (4810–6890)	39·5 (33·7–46·1)	27·8
	DR Congo	81 900 (71 700–93 100)	74 700 (62 800–88 800)	64 700 (53 500–79 500)	22·0 (18·7–26·3)	17·6	346 000 (311 000–385 000)	222 000 (187 000–263 000)	168 000 (141 000–205 000)	57·9 (49·2–69·1)	36·0
	Equatorial Guinea	1090 (886–1300)	795 (589–1060)	683 (492–952)	17·7 (14·8–21·6)	14·4	3710 (3060–4430)	1890 (1420–2500)	1450 (1050–2010)	38·1 (31·9–46·3)	24·2
	Gabon	1140 (921–1380)	797 (594–1070)	680 (487–952)	15·8 (13·2–19·0)	13·5	2940 (2370–3530)	1600 (1200–2110)	1300 (928–1810)	30·1 (25·3–36·4)	21·4
Eastern sub-Saharan Africa		424 000 (392 000–457 000)	378 000 (317 000–448 000)	353 000 (286 000–439 000)	24·9 (21·6–29·0)	20·6	1 450 000 (1 360 000–1 550 000)	941 000 (794 000–1 110 000)	814 000 (658 000–1 010 000)	58·3 (50·5–68·1)	41·1
	Burundi	10 700 (8930–12 600)	11 100 (9470–12 900)	11 200 (9420–13 400)	24·0 (21·3–27·2)	19·3	43 300 (39 100–47 900)	32 100 (26 600–38 400)	29 700 (23 800–37 400)	65·4 (55·7–77·8)	42·7
	Comoros	934 (763–1130)	553 (439–680)	469 (369–589)	27·9 (23·5–33·8)	22·0	2140 (1770–2560)	1050 (846–1290)	836 (663–1040)	49·8 (42·1–59·9)	35·2
	Djibouti	864 (729–1020)	863 (693–1060)	750 (593–943)	21·2 (17·6–25·8)	17·2	2670 (2260–3130)	2080 (1680–2530)	1670 (1330–2080)	47·0 (39·4–56·9)	34·4
	Eritrea	4510 (3690–5480)	4220 (3180–5620)	3870 (2800–5360)	19·3 (16·1–23·5)	15·7	16 000 (13 300–19 100)	11 300 (8600–14 800)	9400 (6860–12 900)	47·5 (39·8–57·5)	30·7
	Ethiopia	144 000 (130 000–161 000)	110 000 (88 300–135 000)	97 900 (77 200–126 000)	26·6 (22·6–31·6)	21·5	426 000 (387 000–469 000)	229 000 (187 000–277 000)	190 000 (150 000–243 000)	52·4 (44·7–62·4)	34·6
	Kenya	32 000 (27 700–36 400)	29 200 (23 900–35 100)	26 400 (21 200–32 300)	19·7 (16·8–23·1)	16·3	99 300 (89 300–111 000)	64 500 (52 900–76 800)	54 100 (43 700–65 700)	40·6 (34·6–47·7)	28·1
	Madagascar	21 400 (18 500–24 600)	21 100 (17 000–25 600)	19 800 (15 900–24 500)	23·0 (19·4–27·7)	18·7	70 600 (61 900–79 800)	55 100 (45 200–65 800)	48 200 (39 100–59 000)	56·6 (48·1–67·7)	37·3
	Malawi	19 500 (17 900–21 400)	14 700 (12 000–17 900)	13 700 (10 600–17 600)	25·0 (21·4–29·6)	19·6	80 000 (74 100–86 300)	38 600 (32 000–46 800)	31 800 (24 900–40 800)	59·1 (50·6–70·0)	39·6
	Mozambique	34 600 (31 200–38 300)	30 400 (25 400–36 200)	29 000 (23 700–35 800)	25·8 (21·9–30·7)	20·2	130 000 (117 000–143 000)	88 900 (74 700–105 000)	76 500 (62 800–93 800)	69·4 (59·0–82·8)	47·0
	Rwanda	11 100 (9090–13 100)	7490 (6050–8990)	7030 (5750–8640)	19·9 (17·5–22·8)	15·1	47 000 (42 900–50 900)	18 800 (15 300–23 200)	16 300 (12 600–21 200)	46·6 (39·6–55·8)	28·3
	Somalia	20 800 (17 300–24 700)	25 800 (20 500–32 300)	27 000 (20 900–35 200)	30·9 (25·9–37·5)	25·2	77 700 (66 700–89 600)	81 300 (65 100–100 000)	80 600 (62 900–104 000)	95·4 (80·8–114)	67·6
	South Sudan	13 200 (11 300–15 100)	13 300 (10 600–16 300)	12 000 (9670–14 900)	33·0 (28·0–38·8)	29·7	47 100 (41 100–53 200)	41 400 (33 500–50 200)	33 100 (26 800–40 600)	92·6 (78·9–108)	66·3
	Tanzania	52 100 (46 700–57 900)	52 300 (42 700–63 600)	50 000 (39 300–63 700)	23·9 (20·4–28·3)	20·0	186 000 (171 000–202 000)	133 000 (110 000–161 000)	118 000 (93 200–150 000)	57·1 (48·9–67·7)	41·8
	Uganda	42 600 (39 500–46 100)	43 000 (36 700–50 200)	40 800 (33 300–50 400)	25·6 (22·0–30·0)	21·9	163 000 (153 000–174 000)	107 000 (91 600–124 000)	91 700 (75 000–112 000)	58·4 (50·4–68·7)	43·3
	Zambia	14 600 (12 900–16 400)	13 800 (10 900–17 500)	13 100 (9930–17 300)	21·1 (17·9–25·4)	17·4	62 300 (56 300–68 700)	36 000 (28 800–44 500)	31 500 (24 200–41 300)	51·8 (44·0–61·8)	35·8
Southern sub-Saharan Africa		45 700 (40 100–51 300)	41 000 (33 800–49 500)	35 900 (28 600–45 700)	21·4 (18·5–25·1)	19·9	128 000 (113 000–145 000)	83 800 (69 400–101 000)	70 700 (56 300–89 800)	42·0 (36·3–49·3)	36·0
	Botswana	1200 (920–1510)	1070 (796–1430)	1000 (735–1380)	20·7 (17·5–25·0)	18·8	3360 (2710–4060)	2190 (1630–2880)	2000 (1460–2750)	41·3 (34·7–49·9)	36·0
	Eswatini	746 (645–857)	557 (458–678)	506 (413–628)	16·9 (14·4–19·8)	14·8	3160 (2780–3570)	1680 (1390–2030)	1430 (1170–1760)	47·3 (40·4–55·3)	37·7
	Lesotho	2330 (2010–2670)	1540 (1070–2080)	1350 (919–1840)	28·6 (22·1–36·2)	24·7	5890 (5230–6620)	3750 (3050–4560)	3030 (2370–3740)	64·4 (54·9–75·6)	52·8
	Namibia	1260 (1100–1460)	1100 (845–1400)	1020 (767–1360)	16·2 (13·5–19·8)	14·3	3610 (3110–4200)	2480 (1930–3180)	2200 (1680–2900)	35·0 (29·6–42·2)	28·4
	South Africa	30 300 (25 700–35 300)	25 300 (20 200–31 800)	21 400 (16 900–27 600)	20·7 (17·7–24·5)	18·5	80 600 (67 900–94 900)	47 100 (37 600–58 900)	38 500 (30 200–49 400)	36·9 (31·6–43·6)	29·7
	Zimbabwe	9850 (8870–10 900)	11 400 (9280–14 000)	10 500 (8310–13 500)	23·4 (20·1–27·7)	22·1	31 600 (28 900–34 600)	26 600 (22 000–31 800)	23 600 (18 600–29 900)	52·4 (45·0–62·0)	45·8
Western sub-Saharan Africa		522 000 (483 000–563 000)	557 000 (487 000–643 000)	535 000 (448 000–644 000)	32·5 (29·3–36·4)	27·8	1 930 000 (1 820 000–2 040 000)	1 710 000 (1 490 000–1 960 000)	1 530 000 (1 280 000–1 860 000)	95·3 (84·7–109)	71·1
	Benin	12 300 (10 200–14 900)	15 600 (12 700–18 700)	15 400 (12 600–19 100)	30·5 (26·4–35·6)	26·8	42 000 (38 800–45 700)	45 700 (39 000–53 300)	42 000 (34 300–51 500)	85·0 (73·8–99·3)	65·9
	Burkina Faso	21 500 (18 400–24 700)	26 000 (20 800–32 200)	26 900 (21 100–34 500)	28·6 (24·2–34·4)	24·0	99 300 (89 400–109 000)	101 000 (83 800–122 000)	98 800 (78 300–125 000)	109 (93·0–129)	82·5
	Cameroon	20 900 (18 200–24 100)	23 400 (18 200–29 300)	21 700 (17 100–27 000)	24·2 (18·5–30·7)	18·6	80 000 (72 300–88 000)	75 400 (62 100–89 300)	64 000 (52 500–77 200)	71·7 (61·2–84·0)	48·6
	Cape Verde	226 (189–271)	135 (106–171)	103 (74·3–141)	9·46 (8·16–11·0)	7·65	647 (544–773)	246 (194–309)	187 (135–253)	17·0 (14·7–19·8)	11·6
	Chad	18 200 (16 700–19 700)	23 600 (19 300–28 600)	25 400 (20 800–31 100)	32·2 (27·6–38·0)	26·9	76 600 (70 000–83 900)	84 600 (72 200–99 100)	85 600 (70 400–105 000)	113 (97·0–133)	83·2
	Côte d'Ivoire	35 300 (28 800–42 100)	34 500 (27 600–42 300)	30 400 (24 500–36 900)	34·2 (27·9–41·4)	28·3	101 000 (90 700–112 000)	78 900 (66 800–92 500)	64 800 (54 300–77 100)	73·3 (62·8–85·6)	48·2
	The Gambia	2020 (1730–2360)	1610 (1210–2110)	1410 (1030–1920)	19·4 (16·4–23·4)	14·7	5460 (4690–6260)	3330 (2550–4300)	2710 (1990–3680)	37·8 (31·9–45·5)	23·2
	Ghana	24 100 (21 100–27 100)	21 600 (16 800–27 000)	19 800 (14 000–27 000)	23·1 (18·7–28·1)	19·5	66 800 (60 700–72 900)	52 900 (43 200–63 400)	44 300 (33 300–58 400)	52·2 (44·9–60·7)	40·5
	Guinea	17 600 (16 100–19 200)	15 100 (12 000–18 700)	14 600 (11 600–18 200)	30·6 (26·4–35·4)	23·4	61 800 (56 100–67 700)	51 300 (43 000–60 800)	45 400 (36 400–55 800)	97·1 (83·8–112)	67·1
	Guinea-Bissau	2700 (2300–3130)	2210 (1770–2720)	1980 (1630–2330)	31·5 (27·9–35·4)	24·3	8630 (7560–9700)	5280 (4280–6370)	4430 (3540–5420)	71·2 (60·7–83·0)	45·6
	Liberia	5380 (4810–6020)	3650 (2820–4680)	3220 (2370–4420)	23·6 (20·0–28·3)	18·3	21 000 (19 300–22 800)	10 800 (8520–13 300)	8280 (6120–11 200)	60·9 (51·9–72·6)	37·8
	Mali	29 400 (25 300–33 700)	36 400 (29 600–44 400)	38 000 (30 800–47 200)	39·5 (34·2–46·2)	32·0	104 000 (95 300–113 000)	111 000 (94 400–129 000)	110 000 (89 500–135 000)	118 (103–138)	86·2
	Mauritania	3800 (3290–4370)	3100 (2610–3680)	2610 (2130–3270)	23·9 (21·3–26·9)	18·9	9230 (8260–10 300)	5860 (4790–7160)	4660 (3580–6100)	42·8 (36·6–50·8)	29·2
	Niger	26 200 (23 100–29 500)	28 300 (23 600–33 800)	30 600 (25 900–36 200)	26·8 (23·6–30·6)	21·1	138 000 (125 000–153 000)	121 000 (101 000–143 000)	120 000 (99 500–147 000)	111 (94·5–131)	71·1
	Nigeria	271 000 (239 000–305 000)	294 000 (247 000–350 000)	277 000 (224 000–346 000)	36·4 (31·7–42·3)	32·0	1 010 000 (936 000–1 080 000)	886 000 (760 000–1 030 000)	773 000 (628 000–957 000)	104 (90·6–120)	78·0
	São Tomé and Príncipe	108 (92·1–125)	71·9 (55·3–91·2)	56·8 (41·0–77·4)	12·1 (10·2–14·3)	9·77	386 (333–441)	160 (124–203)	119 (87·0–161)	25·4 (21·4–30·0)	17·4
	Senegal	13 900 (12 400–15 600)	12 900 (11 200–14 900)	11 700 (9750–14 100)	25·0 (22·5–27·9)	20·1	43 800 (40 600–47 300)	27 100 (23 400–31 400)	22 800 (18 400–28 600)	49·3 (42·8–57·6)	32·6
	Sierra Leone	10 000 (8720–11 400)	8370 (6630–10 400)	8030 (6410–10 100)	28·6 (24·2–33·7)	20·8	39 100 (35 000–43 400)	32 500 (27 600–38 200)	28 100 (22 900–34 300)	102 (88·6–117)	67·7
	Togo	7270 (6170–8540)	6710 (5610–7970)	5930 (4770–7340)	24·9 (21·4–28·8)	19·0	23 700 (20 900–26 600)	18 500 (15 400–22 300)	15 000 (12 000–19 000)	63·2 (53·9–75·3)	41·8

Count data are given to three significant figures. Data in parentheses are 95% uncertainty intervals. NMR=neonatal mortality rate. U5MR=under-5 mortality rate. SDI=Socio-demographic Index. GBD=Global Burden of Diseases, Injuries, and Risk Factors Study.

* Reference scenario.

† Subnational analyses are done in these countries and data is available in the appendix (p 109).

Global U5MR and NMR both are falling short of SDG targets. Global U5MR declined from 71·2 (95% UI 68·3–74·0) in 1990 to 37·1 (95% UI 33·2–41·7) deaths per 1000 livebirths in 2019, with corresponding changes in NMR from 28·0 (95% UI 26·8–29·5) in 1990 to 17·9 (16·3–19·8) deaths per 1000 livebirths (table). The countries with the highest U5MR in 2019 were Central African Republic, Mali, and Chad, whereas Andorra, Singapore, and Slovenia were found to have the lowest U5MR. As for 2019 neonatal mortality, the highest rate was observed in Pakistan, followed by Mali and Central African Republic. The countries with the lowest 2019 NMR were Andorra, Japan, and Singapore. U5MR and NMR declined in every country between 2000 and 2019, apart from Dominica, Guam, and Northern Mariana Islands (appendix p 311).

**Figure 1:**
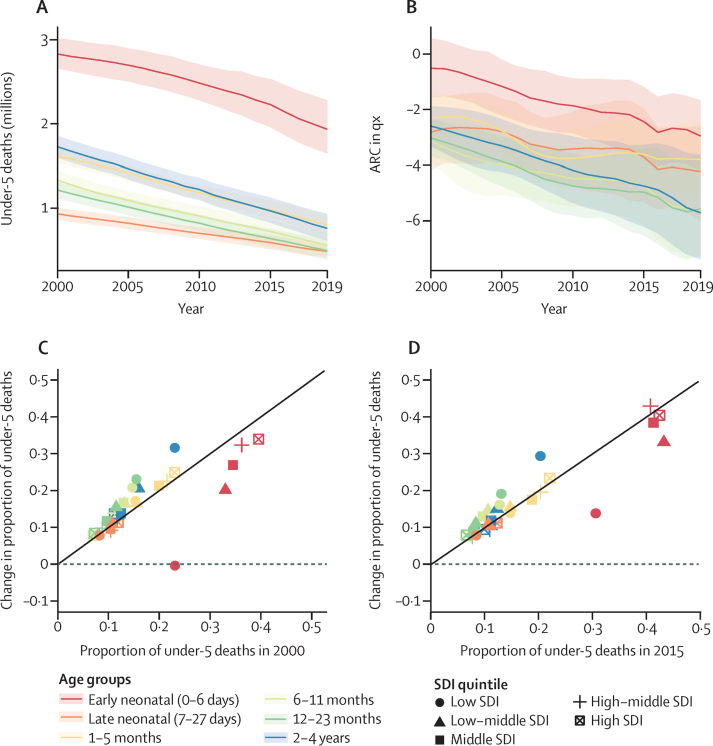
Global all-cause under-5 mortality by age, year, and SDI (A) Under-5 deaths (in millions) for 2000–19. (B) ARC in qx for each year between 2000 and 2019. (C) Proportion of under-5 deaths in 2000 compared with the ratio of age-specific to total under-5 absolute change in deaths between 2000 and 2015 by SDI quintile. (D) Proportion of under-5 deaths in 2015 compared to ratio of age-specific to total under-5 absolute change in deaths between 2015 and 2019 by SDI quintile. The shaded areas in panels A and B represent 95% uncertainty intervals. The black line in panels C and D represents line of equivalence, such that points above the line indicate age and SDI groups in which change outpaces overall under-5 mortality change and points below the line indicate age and SDI groups in which change underperforms relative to overall under-5 mortality change. ARC=annualised rate of change. qx=probability of death. SDI=Socio-demographic Index.

We found evidence of accelerated reduction in global U5MR, but the largest number of deaths, as well as the slowest progress, occurred in the early neonatal age group (figure 1A, B). In all SDI quintiles, decline in NMR lagged behind mortality declines in other age groups (figure 1C, D). There is evidence of relative progress in neonatal mortality in the time period between 2015 and 2019, compared with between 2000 and 2015, but early neonatal progress in this more recent time period is still slower than overall under-5 progress in low SDI settings (figure 1D). The proportion of neonatal death broadly increases as SDI increases: in 2019, in the low SDI quintile, 1·11 million (41%) of 2·67 million deaths in children younger than 5 years were neonatal deaths, and in the high SDI quintile 26 800 (55%) of 48 600 deaths in children younger than 5 years were neonatal deaths (appendix p 120).

**Figure 2:**
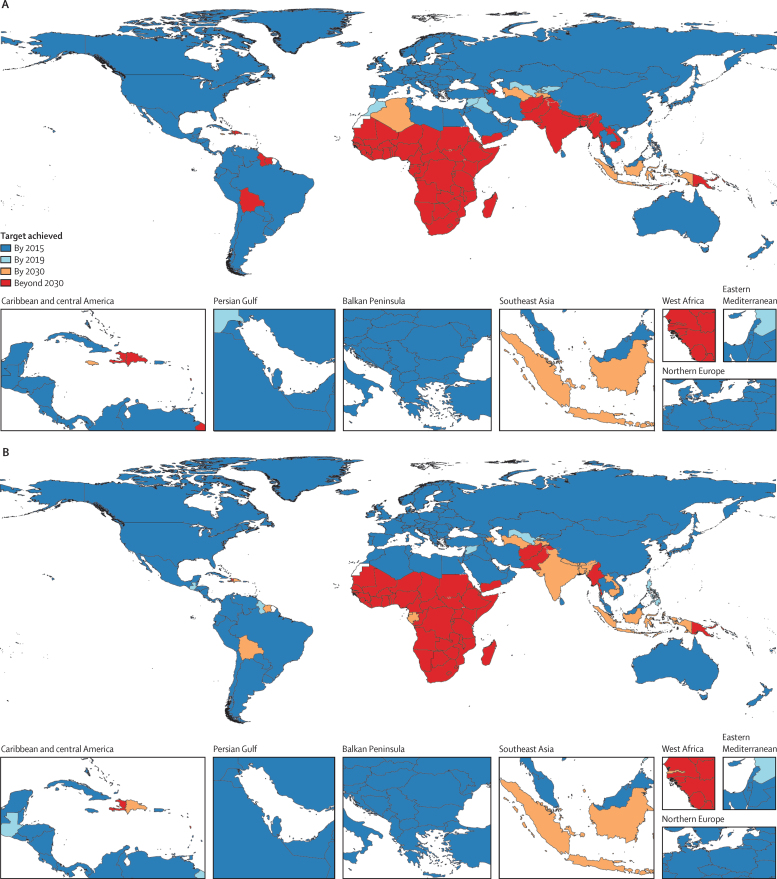
Map of individual countries' progress toward achieving the Sustainable Development Goals 3.2 target of (A) reducing neonatal mortality rate to the threshold of 12 neonatal deaths per 1000 livebirths, and reducing under-5 mortality rate to the threshold of 25 under-5 deaths per 1000 livebirths (B), under the reference scenario

In 2015, 128 (63%) of 204 countries already had an U5MR below the SDG 3.2 threshold of 25 deaths of children younger than 5 years per 1000 livebirths (figure 2A). By 2019, eight additional countries—Syria, Uzbekistan, Guatemala, Philippines, Guyana, Nauru, Vanuatu, and Solomon Islands—had a U5MR below this threshold, making a total of 136 (67%; table). In 2015, 126 (62%) of 204 countries had an NMR below the SDG 3.2 threshold of 12 neonatal deaths per 1000 livebirths (figure 2B). By 2019, an additional seven countries—Syria, Iraq, Kyrgyzstan, Uzbekistan, Morocco, Solomon Islands, and Vanuatu—had achieved an NMR below this threshold, making a total of 133 (65%).

Under-5 mortality in each analysed year was somewhat higher in males than in females, although this difference was not statistically significant at the global level (appendix p 99). U5MR declined in both males and females in the periods between 2000 and 2015, and between 2015 and 2019 (appendix p 99). The 2019 male-to-female ratio of U5MR does not change meaningfully with SDI; this ratio ranges from 1·08 in low-middle SDI to 1·18 in high SDI in 2019 (appendix p 99).

### Levels and trends in cause-specific mortality

**Figure 3:**
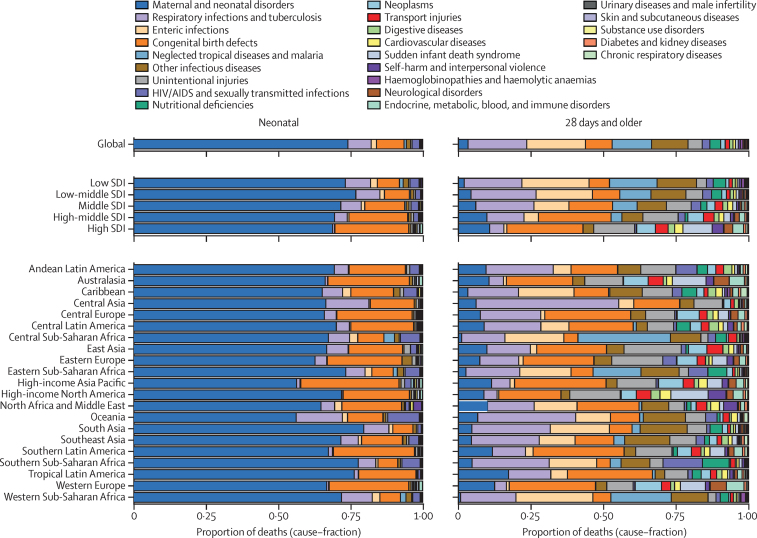
Neonatal and remaining under-5 cause-specific mortality, by region and SDI Values presented are cause fractions: the proportion of total age-specific deaths with a particular underlying cause of death. Causes are presented at Level 2 in the hierarchy, with other non-communicable diseases disaggregated to include congenital birth defects, sudden infant death syndrome, haemoglobinopathies and haemolytic anaemias, endocrine, metabolic, blood, and immune disorders, and urinary diseases and male infertility separately. Total under-5 mortality is split at 28 days to include neonatal (<28 days) separately from children between 28 days and 5 years of age. SDI=Socio-demographic Index.

The leading level 3 causes of global under-5 mortality in 2019 were neonatal disorders, which accounted for 37·3% (95% UI 35·6–38·8) of deaths in children younger than 5 years, followed by lower respiratory infections (13·3% [12·1–14·4]), diarrhoeal diseases (9·9% [8·3–11·6]), congenital birth defects (9·4% [8·0–11·8]), and malaria (7·1% [3·5–12·0]; figure 3; appendix p 100). Leading subcauses of neonatal disorders and congenital birth defects and leading global aetiologies of lower respiratory infections and diarrhoeal disease can be found in the appendix (pp 106, 121).

Of the 15 level 3 causes that accounted for more than 30 000 global under-5 deaths in 2019, the greatest reduction in deaths between 2000 and 2015 was observed in measles, which saw a −9·2% (95% UI −10·4 to −8·0) mean annual percentage change (appendix p 129). Measles was followed by protein-energy malnutrition (−6·5% [–8·2 to −4·7]) and HIV/AIDS (−6·0% [–6·9 to −5·0]). Among these same 15 high-mortality causes, and for the period 2015–19, the three with the greatest reduction in deaths were measles (−11·3% [95% UI −13·7 to −9·0]), HIV/AIDS (−10·2% [–12·3 to −7·8]), and tuberculosis (−7·8 [–9·9 to −5·6]).

In 2019, causes of death varied by age, sex, and SDI (figure 3; appendix p 100). The most common level 3 causes of death in children younger than 5 years were neonatal disorders, lower respiratory infections, and diarrhoeal diseases in the low SDI quintile, and neonatal disorders, congenital birth defects, and sudden infant death syndrome in the high SDI quintile (appendix p 100). The level 3 causes with the largest male-to-female ratio of mortality in the under-5 age group at the global level in 2019 were vascular intestinal disorders (5·99) and inguinal, femoral, and abdominal hernia (2·90), and those with the lowest ratio were gallbladder and biliary diseases (0·29) and pancreatitis (0·29; appendix p 100).

### Scenarios for 2030 and beyond

In our reference scenario, by 2030, 154 (75%) of 204 countries are projected to have a U5MR lower than the SDG threshold of 25 under-5 deaths per 1000 livebirths, and 139 (68%) are expected to have an NMR lower than the SDG threshold of 12 neonatal deaths per 1000 livebirths (figure 2, appendix p 93). In the better-than-reference scenario, 164 (80%) countries would reach the SDG U5MR target, and 145 (71%) countries would reach the SDG NMR target (appendix p 93). In the neonatal scenario, 155 (76%) countries would meet the U5MR target, and in the child scenario, 158 (77%) countries would meet the U5MR target. In the counterfactual scenario without the COVID-19 pandemic, our results suggest 154 (75%) countries would have a U5MR below the SDG threshold and 140 (69%) countries would have an NMR below the SDG threshold by 2030.

### Global optimum and survival potential frontier

There were an estimated 9·45 million (95% UI 8·86–10·05) under-5 deaths more than the global optimum in 2000 and 4·85 million (4·09–5·80) more than the global optimum in 2019 (appendix p 130). These deaths represent 98% of all 9·65 million under-5 deaths in 2000 and 96% of all 5·05 million under-5 deaths in 2019. In 2019, only 198 000 (95% UI 169 000–224 000) under-5 deaths worldwide were below the global optimum, and of these 108 000 (93 000–122 000; 55%) were neonatal deaths. Based on this analysis, and on current technology and health delivery systems, the global optimum NMR is 0·80 (95% UI 0·71–0·86) and the global optimum U5MR is 1·44 (95% UI 1·27–1·58). Sex differences in mortality are similar below the global optimum as compared to overall mortality, with an NMR male-to-female ratio of 1·05 (95% UI 1·00–1·09) and a U5MR male-to-female ratio of 1·12 (95% UI 1·05–1·18).

16 causes of death have a global optimum of zero deaths and are therefore classified as 100% preventable by this framework. With the exceptions of exposure to forces of nature and conflict and terrorism, all of these preventable deaths are infectious conditions. If all countries reduced mortality to the global optimum, the leading level 3 global under-5 causes of death would be neonatal disorders; congenital birth defects; lower respiratory infections; sudden infant death syndrome; and endocrine, metabolic, blood, and immune disorders.

**Figure 4:**
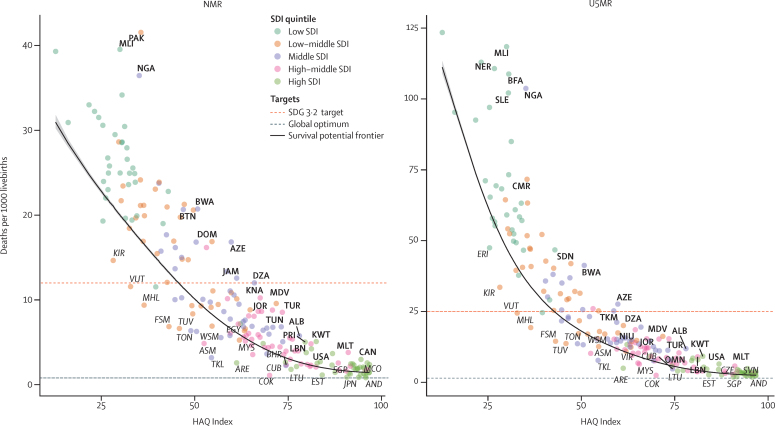
2019 NMR and U5MR by HAQ Index at the national level 204 countries were analysed, and the colour of each point indicates the SDI quintile that the country belongs to. HAQ Index ranges from 0 (worst) to 100 (best). The survival potential frontier, global optimum, and SDG targets are indicated as lines on the graph. Grey shaded bands represent 95% UIs. Countries are labelled with their ISO3 country code in bold when their ratio to the survival potential frontier is in the highest 10% of all countries and in italics when their ratio to the survival potential frontier is in the lowest 10% of all countries. ISO3 codes and corresponding location names are listed in the appendix (p 11). NMR=neonatal mortality rate. HAQ=Healthcare Access and Quality. SDG=Sustainable Development Goal. SDI=Socio-demographic Index. U5MR=under-5 mortality rate. UI=uncertainty interval.

When looking at mortality along the spectrum of HAQ Index, our analysis suggests that in 2000, as many as 1·50 million (95% UI 1·31–1·72) neonatal deaths were above the survival potential frontier, accounting for 40% (95% UI 37–43) of 3·76 million neonatal deaths. In the same year, analysis suggests that 3·94 million (95% UI 3·49–4·40) under-5 deaths were above the survival potential frontier: 41% (95% UI 39–43) of 9·65 million under-5 deaths). In 2019, the number of deaths occurring above the survival potential frontier was smaller, but the fraction of the overall mortality above the survival potential frontier remained similar: 0·88 million (95% UI 0·62–1·20; 36% [95% UI 30–42]) of 2·42 million neonatal deaths and 1·87 million (1·35–2·58; 37% [32–43]) of 5·05 million under-5 deaths (figure 4). If all 204 countries were to improve performance to meet the survival potential frontier without changing their HAQ Index level from 2019, 143 (70%) would have mortality below the NMR SDG threshold and 149 (73%) would have mortality below the U5MR SDG threshold, and 43 (70%) out of 61 countries not achieving both SDG targets would be from the sub-Saharan Africa super-region. The countries where U5MR lags the most relative to HAQ Index in 2019 are Nigeria, Turkey, Mali, and Maldives. The countries where NMR lags the most relative to HAQ Index in 2019 are Maldives, Turkey, and Azerbaijan. The countries with the most success at preventing under-5 mortality and neonatal mortality relative to their HAQ Index are Cook Islands, United Arab Emirates, and Tokelau (figure 4).

Global under-5 mortality above the survival potential frontier in 2019 consisted of 1·56 million (95% UI 1·11–2·17; 83%) deaths due to communicable, maternal, neonatal, and nutritional (CMNN) diseases, 0·23 million (0·15–0·33; 12%) deaths due to non-communicable diseases, and 0·08 million (0·06–0·11; 5%) deaths due to injuries (appendix p 130). If all regions had mortality rates at their survival potential frontier levels in 2019, the distribution of under-5 deaths would skew slightly towards non-communicable diseases but would not fundamentally change; 2·58 million (95% UI 2·35–2·81; 81%) deaths would be due to CMNN diseases, 0·46 million (0·40–0·52; 15%) deaths would be due to non-communicable diseases, and 0·13 million (0·11–0·15; 4%) deaths would be due to injuries (appendix p 130). Of the 48 level 3 causes that were accountable for more than 5000 global under-5 deaths in 2019, those with the lowest proportion of cause-specific deaths above the survival potential frontier were sudden infant death syndrome (27% [95% UI 15–43] of SIDS deaths above the survival potential frontier), other malignant neoplasms (28% [21–36]), varicella and herpes zoster (29% [23–36]), and congenital birth defects (30% [23–37]; appendix p 94). Of the same 48 causes, those with the highest proportion of cause-specific deaths above the survival potential frontier were invasive non-typhoidal salmonella, other neglected tropical diseases, haemoglobinopathies and haemolytic anaemias, and malaria, all with over 50% above the survival potential frontier. The leading causes of death overall were also those with the highest above-survival potential frontier mortality rates, and the rank order would remain similar even if all regions had cause-specific mortality rates at their survival potential frontier levels in 2019: 33% of each of neonatal disorders and lower respiratory infections deaths were above the survival frontier (neonatal disorders ranked first and lower respiratory infections ranked second in both observed and expected), while 40% of diarrhoea deaths were above the frontier (ranked third in observed and fourth in expected; appendix p 94).

## Discussion

### Main findings

Declines of U5MR and NMR have continued to accelerate worldwide. Of 204 countries, our reference scenario suggests that, by 2030, 154 (75%) are likely to meet the U5MR SDG target and 139 (68%) the NMR SDG target. However, the concomitant findings of growing relative inequity and a large remaining proportion of preventable deaths shows there is much more work to be done. If every country were at the global optimum in 2019, global U5MR would have been 1·44 (95% UI 1·27–1·58) deaths per 1000 livebirths and NMR would have been 0·80 (95% UI 0·71–0·86) deaths per 1000 livebirths.

Thankfully, although children have been found to be at risk of developing multisystem inflammatory syndrome^25^ as result of COVID-19, they appear to be less at risk of severe illness and death. It is important to reiterate, however, how the complex, multisector determinants of health that substantially affect child survival could be negatively affected by COVID-19, an understanding that is likely to continue to evolve in the coming months and years. Risks include,^26^ but are not limited to, the potential disruption of routine perinatal and clinical care for children, worsened in-facility outcomes due to overburdened medical systems, loss of caretakers from the pandemic impacting child health and wellbeing, suspended vaccination campaigns, financial and economic pressures leading to food insecurity and malnutrition, disruption of supply chains leading to decreased availability of highly active antiretroviral therapy medications for HIV/AIDS, interrupted prevention of mother-to-child transmission programmes, decreased malaria prevention and treatment, and disruption of domestic economies and education systems. Mitigating these risks will require even more focus and attention on an equilibrium strategy for neonatal and child health.

Our analysis suggests the need for a five-pronged strategy to optimise child survival in the SDG era that augments community-based strategies and efforts to address social determinants of health (eg, education, family planning, financial security) that proved effective during the MDG era. The central theme is that, to achieve SDG targets by 2030, investments should strive for equilibrium and overall system strengthening, with a particular focus on inequality, rather than simply shifting attention to individual priorities.

### Comprehensive neonatal care

Neonatal deaths comprise an increasing share of global under-5 deaths, indicating a generalised need to improve neonatal programmes along the entire SDI spectrum. Although not explicitly stated in SDG targets or in our analysis, reductions in stillbirths should also be targeted through comprehensive maternal and neonatal care. Reducing early neonatal mortality, and stillbirth mortality, should start with expansion of community and facility-based strategies targeted towards pregnancy, labour, delivery, and the postnatal period.^27^ Nepal is an example of a country that explicitly prioritised the neonatal period and integrated community and facility-based approaches, leading to accelerated improvements in neonatal and under-5 mortality.^28^ The first step is encouraging and supporting facility-based delivery by skilled providers with the training and resources available to perform resuscitative efforts for women and neonates when needed.^5^ Basic activities include skin-to-skin contact, timely breathing assistance for intrapartum asphyxia, chlorhexidine umbilical cord cleansing for sepsis prevention, and early screening for congenital birth defects.^5^ Improvements also need to be made to neonatal care after delivery. Advancements are needed for in-hospital activities such as intensive care for prematurity, advanced resuscitation for intrapartum asphyxia, full support for sepsis beyond antibiotics, breastfeeding education and support, and surgical care for neonatal emergencies and birth defects that have been shown to be associated with improved neonatal survival.^27^, ^29^, ^30^ Postnatal check-ups are also required for prompt diagnosis and treatment of new illnesses that can be life-threatening in young neonates. Crosscutting, longitudinal neonatal care is not possible without augmenting hospital infrastructure, supply chains, and qualified health-care workers, and must be accounted for in national health plans.^5^

### Optimising health systems to scale up interventions

Providing technology and supplies alone, without coordinated investment in the strengthening of health systems, will be insufficient for achieving the SDG targets. Moving beyond survival is the cornerstone of the SDGs, which requires enabling environments, as outlined in the UN Global Strategy for Women's, Children's and Adolescents' Health 2016–30 agenda.^4^ Per our analysis, more than 90% of countries have the potential to achieve the SDG targets by optimising their current health systems. Efforts to counter shortages and retain skilled health-care workers, reinforce facility infrastructure and supplies including oxygen,^31^ develop and strengthen referral networks, and expand integrated services^7^ are needed to achieve access and quality of care for improving survival rates for children younger than 5 years, particularly around the time of birth.^27^, ^29^ Liberia is an example of a country that has made important progress in health system strengthening. Despite the odds of civil war and the Ebola virus epidemic, Liberia heavily invested in paying and supervising community health workers, providing medical supply chains to remote areas, and creating a health information system, leading to better survival.^32^

### Continued investment and scale-up of community-based initiatives

Community-based strategies such as primary health-care promotion and integrated management of childhood illness^33^ are an important pillar of prevention. Successful community activities include vaccination campaigns, insecticide-treated bednets for malaria, and mother-to-child HIV/AIDS transmission prevention.^34^ Further efforts are required, however, to increase uptake and coverage of additional community-based activities such as ensuring optimal maternal nutrition and iron and folic acid supplementation^35^ (to target low birthweight and neural tube defects), reducing household air pollution and second-hand smoke, *Haemophilus influenzae* type B and pneumococcal vaccination, and access to antibiotics^36^ for lower respiratory infections. Similarly, treatment campaigns for diarrhoea such as oral rehydration solution, zinc, and rotavirus vaccines have been successful, but must be accompanied by reductions in malnutrition and improvements in clean water and sanitation to achieve more than 90% reduction in rates of diarrhoea from the 2015 levels.^37^

### Targeting inequity across and within countries

Relative inequity has grown over the 29 years since the first GBD study, with the 51 countries in the Countdown to 2030 initiative in sub-Saharan Africa and south Asia now accounting for 80% of all child mortality and facing stark within-country disparities.^6^, ^38^ Within-country disparities exist throughout the SDI spectrum and are related to race and ethnicity, urban-rural geography, mother's education, and income.^34^ Global and national achievement of SDG 3.2 will hinge on our collective ability to target inequality both across and within countries.

Progress for the countries in the Countdown to 2030 programme is monitored by key intervention coverage milestones,^6^ but must be met with national ownership and effective international investment. On an international level, the World Bank's Global Financing Facility is an example of a performance-based, country-led mechanism to strengthen health systems and multisectoral approaches,^6^ but the promise of this programme has not reached countries like Central African Republic and Chad, which are not only the furthest from achieving the SDG targets with lowest key intervention coverage, but are also cited as receiving the least development assistance funding.^39^ These countries contrasts with countries like Rwanda and Bangladesh. In Rwanda, a revised national health policy successfully aligned international donors to nationally driven goals of comprehensive child health care and health system strengthening, and were associated with a dramatic reduction in under-5 mortality.^32^ In Bangladesh, the government partnered with domestic and international non-governmental organisations to target areas of the country most in need with delivering known interventions, performing local effectiveness research, and prioritising women's empowerment.^28^

Peru and Brazil are examples of middle and high-middle SDI countries that have targeted inequity internally. Peru substantially reduced under-5 mortality by adopting the 2002 Acuerdo Nacional,^28^ a national health policy targeting extreme poverty that deployed health workers to impoverished communities, completed community-based intervention research to increase perinatal care coverage, and codified collective responsibility for improving health outcomes. Brazil sanctioned governmental conditional cash transfers targeting prenatal care, immunisation, child health check-ups, and nutritional education.^28^ Although the specific solutions for targeting inequity and marginalised populations vary, the essential component is that the efforts to increase equity must be explicit, sustained, and universal because it is present throughout the world.

### Prioritising research into specific causes of child mortality

Many of the leading causes of death are also the source of the most mortality above both the global optimum and the survival potential frontier, include neonatal disorders, congenital birth defects, sudden infant death syndrome, many childhood cancers, and important infections like lower respiratory infections, diarrhoea, and meningitis. These causes are prime targets for additional dedicated primary research on disease mechanisms for effective prevention, detection, and treatment. Sudden infant death syndrome is particularly notable as only 27% of the mortality burden is above the survival potential frontier, it is the top cause of death in older infants and children in the high SDI quintile, and comparatively little is known about its pathophysiology.

This entire analysis draws on the overall strength and rigour of GBD 2019, the only comprehensive analysis of fertility, population, mortality, and outcomes for specific diseases and injuries that currently exists. The UN Inter-agency Group for Child Mortality Estimation last published estimates for 2017^11^ but has not reported on causes of mortality since 2015,^12^, ^40^ at which time there was broad agreement in the top causes of death globally, but some important differences existed in cause categories that limited our ability to make direct comparisons.

Measuring preventable death with the intersection of HAQ Index and SDG targets has not been explored in previous literature and necessarily extends beyond the scope of the OECD and Eurostat taskforce that only focuses on adult health outcomes.^16^ This method is more holistic than previous avertable mortality frameworks such as the Countdown to 2030 report that analysed only a composite coverage index of specific interventions, but did not measure the health system performance as a whole.^6^ Uses of our preventable mortality analyses include being able to identify the causes with the most potential for improvement (largest proportion above the global optimum or stochastic frontier analysis), the regions with potential imbalances in health priorities (largest ratio above frontier or discrepancies in ratio between neonates and children aged 1–59 months), causes where there are needs for better distributional allocation of resources, expertise, or delivery (those where the frontier is largely flat until decreasing sharply in high HAQ Index settings), and the causes where there is the greatest need for basic research into prevention and treatment (largest proportion below the global optimum). This preventable death framework thus introduces a novel, useful, and potentially powerful tool for developing comprehensive, evidence-based strategies for advancing child survival on multiple fronts.

### Limitations

This analysis has several limitations. First, it shares the limitations of the overall GBD analysis,^20^, ^24^ including it being a descriptive study; limitations on data availability because of reporting lags or because of disruptions in settings with conflict, natural disasters, or domestic governance crises; variable data granularity with respect to age, sex, and cause detail; varying quality and completeness of mortality reporting systems; and the core GBD assumption of each death having only a single underlying cause, where, clinically, there is close inter-relatedness of many causes, especially in the very young. Second, our future health scenario analyses are benchmarked against past trends and are ecological in nature. This limits the ability of the analysis to be used for causal inference, and also means it is limited in its ability to capture disruptions that could arise as a consequence of future crises, such as the COVID-19 pandemic. Third, although our framework for preventable mortality is conceptually simple, reproducible, and a powerful tool for tracking context-specific progress, it is also limited by its inherently retrospective nature, its inability to parse competing risks or factors that might influence geographical variability, and that it does not make special consideration for causes like vaccine-preventable diseases that some experts contend are entirely preventable. Finally, the definition of livebirth has varied in countries and over time. Although our study has utilised a large amount of empirical data on death in the under-5 age groups, directly or indirectly measured, such information is based on potentially different definitions of livebirths, thus affecting the accuracy of our results. Although we do account for source specific biases, difference in definitions of livebirths as one of them, in our U5MR estimation process, future model development should be done to explicitly account for the effect of definition of livebirths on the accurate estimation of mortality in the under-5 age groups.

### Future directions

Future work is required to measure and understand the direct (severe illness and death) and indirect (determinants of health) effects of COVID-19 on child mortality. First, this work will include collecting data on disruptions in basic childhood health services (eg, vaccines, integrated management of childhood illness, well-child visits), nutritional status (eg, food supply and distribution), perinatal health (eg, maternal and neonatal care), and socioeconomic indicators such as fertility, education, and household income. A second direction is to work towards an integrated framework for women's, maternal, and child health because of the inherent links between the health of mothers and their children. Third, integrating information from prevention and intervention trials into developing future health scenarios is a priority in order to provide information to motivated policy makers as to what their most effective options might be. Fourth, following the momentum of the Institute for Health Metrics and Evaluation's Local Burden of Disease project, developing increasing local estimates of cause-specific and age-specific disease burden is crucial to guide local efforts at improving survival, and assess within-country disparities further.

Achieving SDG 3.2 will require focus on equilibrium, which will involve balancing early newborn care with continuing prenatal and older child health initiatives, strengthening quality health systems, scaling up interventions, addressing within-country disparities, and pursuing integrative action on social determinants of health. All these steps forward promote the SDG agenda of moving beyond mere survival, for the wellbeing of young children worldwide.

## Data sharing

To download the data used in these analyses, please visit the Global Health Data Exchange at http://ghdx.healthdata.org/gbd-2019.

## Declaration of interests

Robert Ancuceanu reports consulting fees from AbbVie and AstraZeneca; payment or honoraria for lectures, presentations, speakers' bureaus, manuscript writing, or educational events from Sandoz and AbbVie; support for attending meetings and/or travel from AbbVie and AstraZeneca, all outside the submitted work. Marcel Ausloos reports grants or contracts from Romanian National Authority for Scientific Research and Innovation, CNDS-UEFISCDI, project number PN-III-P4-ID-PCCF-2016-0084, outside the submitted work. Ettore Beghi reports grants or contracts paid to their institutions from ALSA, the Italian Ministry of Health and SOBI; payment or honoraria for lectures, presentations, speakers' bureaus, manuscript writing, or educational events from Arvell Therapeutics; support for attending meetings and/or travel from ILAE and EAN, all outside the submitted work. Reinhard Busse reports leadership or fiduciary role in other board, society, committee, or advocacy group, paid or unpaid with the Robert Koch Institute as member of the scientific advisory committee, German Burden 2020 project, all outside the submitted work. Joao Conde reports grants or contracts from the European Research Council grant agreement No 848325 (ERC starting grant); patents planned issued or pending for TRPV2 antagonists WO2019054891 - Instituto de Medicina Molecular (PT), Hydrogel Particles, Compositions, and Methods- WO US US20170333304A1 - Massachusetts Institute of Technology (USA), and Theranostic nanoprobes for overcoming cancer multidrug resistance and methods- WO US WO2016134232A1 - Massachusetts Institute Of Technology (USA), all outside the submitted work. Irina Filip reports payment or honoraria for lectures, presentations, speakers' bureaus, manuscript writing, or educational events from Avicenna Medical and Clinical Research Institute. Claudiu Herteliu reports grants or contracts from Romanian National Authority for scientific research and innovation, CNDS-UEFISCDI, project number PN-III-P4-ID-PCCF-2016-0084, research grant (October, 2018, to September, 2022), understanding and modelling time–space patterns of psychology-related inequalities and polarisation, and project number PN-III-P2-2.1-SOL-2020-2-0351, research grant (June, 2021, to October, 2021), approaches within public health management in the context of COVID-19 pandemic, and from the Ministry of Labour and Social Justice Romania, project number 30/PSCD/2018, research grant (September, 2018 to June, 2019), agenda for skills Romania 2020–25, all outside the submitted work. Sheikh Mohammed Shariful Islam reports grants or contracts from National Health and Medical Research Council (NHMRC) and National Heart Foundation of Australia Fellowships, outside the submitted work. Jacek Jerzy Jozwiak reports payment or honoraria for lectures, presentations, speakers' bureaus, manuscript writing, or educational events from Teva, Amgen, Synexus, Boehringer Ingelheim, ALAB Laboratories, and Zentiva, all outside the submitted work. Nicholas J Kassebaum reports support for the present manuscript from the Bill & Melinda Gates Foundation as grant funding for the GBD. Kewal Krishan reports non-financial support from UGC Centre of Advanced Study, CAS II, Department of Anthropology, Panjab University, Chandigarh, India, outside the submitted work. Morteza Mahmoudi reports payment or honoraria for lectures, presentations, speakers' bureaus, manuscript writing, or educational events for his published books, plenary lectures, and licensed patent to Seer; leadership or fiduciary role in other board, society, committee or advocacy group, paid or unpaid with the Academic Parity Movement, a non-profit organisation dedicated to addressing academic discrimination, violence, and incivility, as a cofounder, all outside the submitted work. Shuhei Nomura reports support for the present manuscript from Ministry of Education, Culture, Sports, Science and Technology of Japan (MEXT) as grant funding. Adrian Pana reports grants or contracts from Romanian National Authority for Scientific Research and Innovation, CNDS-UEFISCDI, project number PN-III-P4-ID-PCCF-2016-0084, research grant (October, 2018, to September, 2022), understanding and modelling time-space patterns of psychology-related inequalities and polarisation, and project number PN-III-P2-2.1-SOL-2020-2-0351, research grant (June, 2021, to October, 2021), approaches within public health management in the context of COVID-19 pandemic, all outside the submitted work. Seithikurippu R Pandi-Perumal reports payment or honoraria for lectures, presentations, speakers' bureaus, manuscript writing, or educational events for the volumes he edited; leadership or fiduciary role in other board, society, committee, or advocacy group, paid or unpaid, with Somnogen Canada, Toronto, Canada, as the President and Chief Executive Officer, all outside the submitted work. Thomas Pilgrim reports grants or contracts from Biotronik, Boston Scientific, and Edwards Lifesciences; payment or honoraria for lectures, presentations, speakers' bureaus, manuscript writing, or educational events from Biotronik, Boston Scientific, and HighLifeSAS; and being proctor for Medtronic and Boston Scientific, all outside the submitted work. Maarten J Postma reports grants or contacts from Merck, Sharp & Dohme, GlaxoSmithKline, Pfizer, Boehringer Ingelheim, Novavax, Bayer, Bristol Myers Squibb, AstraZeneca, Sanofi, IQVIA, BioMerieux, WHO, EU, Seqirus, FIND, Antilope, DIKTI, LPDP, and Budi; consulting fees from Merck, Sharp & Dohme, GlaxoSmithKline, Pfizer, Boehringer Ingelheim, Novavax, Quintiles, Bristol Myers Squibb, AstraZeneca, Sanofi, Novartis, Pharmerit, IQVIA, and Seqirus; participation on a data safety monitoring board or advisory board to Asc Academics as adviser; stock or stock options in Health-Ecore and PAG, all outside the submitted work. Amir Radfar reports payment or honoraria for lectures, presentations, speakers' bureaus, manuscript writing, or educational events from Avicenna Medical and Clinical Research Institute. Jasvinder A Singh reports consulting fees from Crealta/Horizon, Medisys, Fidia, Two labs, Adept Field Solutions, Clinical Care options, Clearview health-care partners, Putnam associates, Focus forward, Navigant consulting, Spherix, MedIQ, UBM, Trio Health, Medscape, WebMD, and Practice Point communications, and the National Institutes of Health and the American College of Rheumatology; payment or honoraria for lectures, presentations, speakers' bureaus, manuscript writing, or educational events from Simply Speaking; support for attending meetings and travel from OMERACT, an international organisation that develops measures for clinical trials and receives arm's length funding from 12 pharmaceutical companies, when travelling biannually to OMERACT meetings; leadership or fiduciary role in other board, society, committee, or advocacy group, paid or unpaid, with OMERACT as a member of the steering committee, with the US Food and Drug Administration (FDA) Arthritis Advisory Committee, with the Veterans Affairs Rheumatology Field Advisory Committee as a member, and with the UAB Cochrane Musculoskeletal Group Satellite Center on Network Meta-analysis as a director and editor; stock or stock options in TPT Global Tech, Vaxart pharmaceuticals, Charlotte's Web Holdings, and previously owned stock options in Amarin, Viking, and Moderna pharmaceuticals, all outside the submitted work. Mark A Stokes reports payment or honoraria for lectures, presentations, speakers' bureaus, manuscript writing, or educational events at the Autism Teaching Institute (Victoria, Australia); unpaid participation on a data safety monitoring board or advisory board with the Deakin University Human Research Ethics Committee; leadership or fiduciary role in other board, society, committee, or advocacy group, paid or unpaid, with the Australasian Society for Autism Research as a past president, with the Australasian Society for Autism Research as a board member, with Kidsafe (Victoria, Australia) as vice president, with Mindful as a member of the research advisory board, and with Autism Teaching Institute as chair of the research advisory board; stock or stock options in Cochlear and Medical Developments, all outside the submitted work. Stefan Stortecky reports grants or contracts to their institute from Edwards Lifesciences, Medtronic, Boston Scientific, and Abbott; consulting fees from BTG and Teleflex; payment or honoraria for lectures, presentations, speakers' bureaus, manuscript writing, or educational events from BTG and Boston Scientific; support for attending meetings and/or travel from BTG, all outside the submitted work. Carolyn B Swope reports support for the present manuscript from Delos Living as a former employee; and consulting fees from Delos Living, outside the submitted work. Riaz Uddin reports grants or contracts from Alfred Deakin Postdoctoral Research Fellowship, Deakin University, Australia; support for attending meetings and/or travel from Deakin University Institute for Physical Activity and Nutrition, all outside the submitted work.
