# Supplementary material for: Global, regional, and national progress towards Sustainable Development Goal 3.2 for neonatal and child health: all-cause and cause-specific mortality findings from the Global Burden of Disease Study 2019
Source: Lancet. 2021 Sep 4;398(10303):870–905. doi: 10.1016/S0140-6736(21)01207-1 (PMC8429803; doi:10.1016/S0140-6736(21)01207-1)
Supplement: Supplementary appendix [file mmc1.pdf]

THE LANCET

Supplementary appendix

This appendix formed part of the original submission and has been peer reviewed. We post it as supplied by the authors.

Supplement to: GBD 2019 Under-5 Mortality Collaborators. Global, regional, and national progress towards Sustainable Development Goal 3.2 for neonatal and child health: all-cause and cause-specific mortality findings from the Global Burden of Disease Study 2019. *Lancet* 2021; published online Aug 17. http://dx.doi.org/10.1016/S0140-6736(21)01207-1.

Appendix to "Global, regional, and national progress toward Sustainable Development Goal 3.2 for neonatal and child health: all-cause and cause-specific mortality findings from the Global Burden of Disease Study 2019"

Contents

Section 1: Supplemental Tables and Figures Table of Contents 6
Section 1.1: Supplemental tables 6
Section 1.1.1: Methods 6
Section 1.1.2: Results 6
Section 1.2: Supplemental figures 7
Section 2: GATHER checklist 9
Section 3: GBD overview 10
Section 3.1: Global Burden of Diseases, Injuries, and Risk Factors Study 2019 10
Section 3.2: Geographical locations of the analysis 11
Section 3.3: Age groups included in the analysis 11
Section 3.4: Causes of death in the analysis 12
Section 3.5: Statement of GATHER compliance 12
Section 3.6: GBD results resources 13
Section 3.7: Data input sources overview 13
Section 3.8: Funding sources 13
Section 4: All-cause mortality, HIV mortality, and fatal discontinuities 13
Section 4.1: Overview 13
Section 4.2: Child mortality 14
Section 4.2.1: Data sources 14
Overview 14
VRs, sample registration systems, and DSPs from other sources 14
Under-5 populations and livebirths 14
CBH microdata 14
CBH tabulated data 15
SBH microdata 15
Under-5 age-sex patterns for VR/SRS/DSP 15
Under-5 age-sex patterns from CBH 15

VR prioritisation 15
Section 4.2.2: Computation of 5q0 from CBH 16
Processing of CBH microdata 16
Processing of tabular CBH 16
Section 4.2.3: Computation of 5q0 from SBH 16
Section 4.2.4: Computation of 5q0 from other sources 16
Section 4.2.5: 5q0 data synthesis 16
Overview 16
First-stage non-linear mixed effects model 17
Bias correction 18
Spatiotemporal smoothing 19
Gaussian process regression (GPR) 20
Data variance 21
Hyperparameter selection 22
Section 4.2.6: Identify and remove outliers 23
Section 4.2.7: Rake subnational estimates to national level (excluding South Africa) 23
Section 4.2.8: Review estimates for quality 23
Section 4.2.9: Under-5 age and sex pattern model estimation 23
Section 4.2.10: Identify and remove outliers from age and sex models 26
Section 4.2.11: Under-5 death number estimation 27
Section 4.3: HIV/AIDS estimation 27
Section 4.3.1: HIV-free survival rates (for Spectrum) 27
Section 4.3.2: EPP and Spectrum 27
Section 4.3.3: HIV mortality reckoning 28
Section 4.4: Age-specific mortality estimation for all GBD age groups: with and without HIV 29
Section 4.4.1: HIV-deleted age-specific mortality 29
Section 4.4.2: Age-specific mortality without discontinuities (with HIV/AIDS) 29
Section 4.4.3: Add fatal discontinuities 29
Section 4.5: Fatal discontinuities 30
Section 4.5.1: Input data 30
Overview 30
Discontinuities only (non-CODEm) 30
Conflict and terrorism 30

Exposure to forces of nature ... 32
Partial discontinuity (CODEm) ... 32
Executions and policy conflict ... 32
Homicide ... 32
Protein-energy malnutrition (PEM) ... 32
Other injury causes ... 33
Meningococcal meningitis and other diseases ... 33
Section 4.5.2: Location mapping ... 33
Section 4.5.3: Side splitting ... 34
Section 4.5.4: Prioritisation ... 34
Section 4.5.5: Age-sex splitting ... 34
Section 4.5.6: Assigning uncertainty and generating draws ... 34
Section 5: Cause-specific mortality ... 35
Section 5.1: GBD 2019 Causes of Death database ... 35
Section 5.1.1: Background ... 35
Section 5.1.2: CoD data identification ... 35
Overview of data types ... 35
ICD-detail ... 35
ICD-tabulations list ... 36
China Disease Surveillance Points /China Center for Disease Control and Prevention ... 36
Sample registration system ... 36
India Medical Certification of Cause of Death ... 37
Verbal autopsy ... 37
Verbal autopsy coded to ICD-10 and other lists ... 37
InterVA-modelled verbal autopsy ... 37
Other data types ... 38
Maternal mortality data ... 38
Surveys and censuses reporting fraction of deaths due to selected injuries ... 38
Police records ... 38
Population-based cancer registries ... 38
Cancer registries with incidence ... 38
Cancer registries with incidence and high-quality mortality data ... 39
Section 5.1.3: Standardise input data (step 1) ... 39

Disaggregation (step 1.1) 39
State splitting (step 1.2) 40
Calculate non-maternal deaths (step 1.3) 40
Section 5.1.4: Map to GBD cause list (step 2) 41
Section 5.1.5: Age-sex splitting (step 3) 42
Standardizing all inputs to have age and sex detail matching GBD demographics 42
Correct age-sex violations 43
Section 5.1.6: Correction for miscoding of Alzheimer's and other dementias, Parkinson's disease, and atrial fibrillation and flutter (step 4) 43
Objective 43
Correction process 44
Section 5.1.7: Redistribute (Step 5) 45
Redistribute HIV-related garbage codes (step 5.1) 45
Regress garbage codes versus non-garbage codes (step 5.2) 46
Development of an algorithm for redistribution of garbage codes based on multiple CoD data .. 47
Verbal autopsy anaemia adjustment (step 5.3) 49
Calculate redistribution uncertainty (step 5.4) 49
Section 5.1.8: HIV/AIDS misclassification correction (step 6) 51
Section 5.1.9: Scale strata to province (step 7) 52
Section 5.1.10: Restrictions post-redistribution (step 8) 52
Section 5.1.11: Drop VR country years or mark as non-representative (step 9) 53
Section 5.1.12: Cause aggregation (step 10) 53
Section 5.1.13: Remove shocks and HIV/AIDS maternal adjustments (step 11) 53
Remove HIV/AIDS and shocks from denominator where cause list includes HIV/AIDS (step 11.1) 54
Remove HIV/AIDS deaths from maternal mortality sources (step 11.2) 54
HIV/AIDS correction of sibling history, census, and survey data (step 11.3) 55
HIV/AIDS correction of other maternal mortality data (step 11.4) 57
Section 5.1.14: Noise Reduction (step 12) 58
Section 5.1.15: Cause of death database and outlier identification (step 13) 58
Section 5.1.16: Causes of death data star rating calculation 59
Section 5.2: Causes of death modeling methods 61
Section 5.2.1: CODEm 61

Overview of methods 61
Model pool development 62
Data variance estimation 62
Testing model pool on 15% sample 62
Ensemble development and testing 63
Final estimation 63
Selection of causes for which CODEm is used 63
Model-specific covariates 63
Section 5.2.2: Causes modeled outside of CODEm 63
Overview 63
Negative binomial models 64
DisMod-MR 2.1 64
Overview of DisMod 64
DisMod-MR 2.1 likelihood estimation 65
Natural history models 67
Prevalence-based models 67
Sub-cause proportion models 67
Section 5.2.3: Central computation 68
Imported cases 68
CoDCorrect 68
Objective 68
Algorithm and levels 68
Years of life lost calculation 69
GBD world population age standard 69
Section 6: Secondary analyses 69
Section 6.1: Demographic methods for converting between mortality rates and probabilities 69
Section 6.2: Scenarios for 2030 70
Section 6.3: Mortality benchmarks 70
Section 6.3.1: Global Optimum 70
Section 6.3.2: Stochastic frontier analysis on HAQ Index 70
Appendix Contributions 71
References 79

Section 1: **Supplemental Tables and Figures Table of Contents**

The following is a list of supplementary tables and figures provided in this appendix.

Note that additional tables and figures for core GBD 2019 results, by age, sex, location, and year, can be generated using interactive visualizations in GBD Compare tool (http://vizhub.healthdata.org/gbd-compare), and the all-cause and cause-specific visualization tools (https://vizhub.healthdata.org/mortality/ ; https://vizhub.healthdata.org/cod/). Alternatively, results may be downloaded in tabular form from the GBD results tool (http://ghdx.healthdata.org/gbd-results-tool).

Section 1.1: Supplemental tables

Section 1.1.1: **Methods**

Table S1. GATHER checklist

Table S2. Hyperparameter selection according to data density

Table S3. Conflict and terrorism data sources

Table S4. ICD 10 codes for substances or drugs used to assign deaths coded to an underlying cause of unintentional poisoning by using multiple CoD data

Table S5. Algorithm for the selection and assignment of a substance or drug use CoD for deaths coded to an underlying cause of unintentional poisoning by using multiple CoD data

Table S6. GBD complete cause hierarchy with associated metadata.

Section 1.1.2: **Results**

Table S7. Number and proportion of countries and territories (n=204) reducing under-5 mortality rate (U5MR) and neonatal mortality rate (NMR) to below the SDG 3.2 targets of 25 under-5 and 12 neonatal deaths per 1000 live births, by 2030, under six future health scenarios. The scenarios include *reference*, *better*, *worse*, accelerated change only in neonates (*neonatal* scenario*)*, accelerated change only in children between 1 months and 4 years of age (*child* scenario), and a counterfactual *no Covid* scenario representing predicted U5MR and NMR in the absence of the COVID-19 pandemic. Mean and 95% uncertainty intervals are presented. The table also includes a list of countries and territories remaining above the U5MR and NMR SDG targets for each age group (neonatal, under-5) and scenario in the table.

Table S8. By level-3 cause in the GBD cause hierarchy, global rank (a) in 2019, (b) under the condition where all countries have mortality at least as low as the Global Optimum, and (c) under the condition where all countries have mortality at least as low as the Survival Potential Frontier. Additionally, the table includes the proportion of global deaths in 2019 that were above each of our two benchmarks.

S8a. The results as described, for the complete under-5 age group.

S8b. The results as described, for the neonatal (<28 days) age group.

Table S9. Neonatal and under-5 mortality rate (deaths per 1000 live births) by sex (male, female, both-sex), year (2000, 2015, 2019), and location (global and socio-demographic index (SDI) quintiles).

Table S10. Cause-fraction (cause-specific proportion of total deaths) and cause rank for level-3 causes of death for children under 5 years of age, globally and for socio-demographic index (SDI) quintiles, in 2019.

S10a. Both-sex

S10b. Males

S10c. Females

Table S11. Population attributable fraction (PAF) for etiologies of global both-sex under-5 mortality, in 2019, for all levels of the cause of death hierarchy.

Table S12. Neonatal and under-5 deaths [for 2000, 2015, and 2019], and neonatal and under-5 mortality rate (NMR; U5MR) [for 2019] for subnational units in the United States of America, the United Kingdom, and Brazil.

Section 1.2: Supplemental figures

Figure S1. Supplemental maps for child mortality analyses and input data.

S1a. Under-5 mortality rate in 2000.

S1b. Under-5 mortality rate in 2015.

S1c. Under-5 mortality rate in 2019.

S1d. Neonatal mortality rate in 2000.

S1e. Neonatal mortality rate in 2015.

S1f. Neonatal mortality rate in 2019.

S1g. Last year of available all-cause data at the aggregate under-5 level. In some cases, summary under-5 data is available when data for more detailed under-5 age groups is not available. Data are included here whether or not they passed final quality checks and were included in the modelling process.

S1h. Last year of available cause-specific data for any cause. In some cases, data is available for some causes but not others. Data are included here whether or not they passed final quality checks and were included in the modelling process.

S1i. Proportion of estimated under-5 deaths between 2000 and 2019 which were captured by extracted vital registration data.

Figure S2. Ratio of under−5 deaths occurring before to after 28 days, by Socio-demographic index (SDI) quintile and year.

Figure S3. Supplemental cause-fraction plots globally, and by Socio-demographic index (SDI) quintile and GBD region. Causes are included at level-2 in the hierarchy, with "other non-communicable diseases" disaggregated to report on congenital birth defects, sudden infant death syndrome, and hemoglobinopathies and hemolytic anemias separately.

S3a. Neonatal and under-5 deaths by level-2 cause, 2000.

S3b. Neonatal and under-5 deaths by level-2 cause, 2015.

S3c. Neonatal and under-5 deaths by neonatal disorder, 2000.

S3d. Neonatal and under-5 deaths by neonatal disorder, 2015.

S3e. Neonatal and under-5 deaths by neonatal disorder, 2019.

S3f. Neonatal and under-5 deaths by congenital birth defect, 2000.

S3g. Neonatal and under-5 deaths by congenital birth defect, 2015.

S3h. Neonatal and under-5 deaths by congenital birth defect, 2019.

Figure S4. Annualized rate of change among level-3 causes with greater than 30,000 global deaths in 2019, comparing change during the Millennium Development Goals (MDG) period (2000-2015) to change during the Sustainable Development Goals (SDG) period (2015-2019).

Figure S5. Global deaths by mortality benchmarks, 2000−2019**.** Under-5 and neonatal deaths (in millions) are displayed by level-1 cause, and split by mortality above and mortality below each of two benchmarks: the Global Optimum and the Survival Potential Frontier (SPF) on Healthcare Access and Quality (HAQ) Index. Shaded bands represent 95% uncertainty intervals.

Figure S6. Deaths by mortality benchmarks, for GBD super regions and level-2 cause, 2000−2019**.** Under-5 and neonatal deaths (in millions) are split by mortality above and mortality below each of two benchmarks: the Global Optimum and the Survival Potential Frontier on Healthcare Access and Quality (HAQ) Index. Shaded bands represent 95% uncertainty intervals.

Figure S7. Global outcomes for the two mortality benchmarks, at the most detailed cause level, for neonatal and under-5 age groups.

7a. Global Optimum mortality rate, neonatal, in deaths per 1000 live births.

7b. Global Optimum mortality rate, under-5, in deaths per 1000 live births.

7c. The Survival Potential Frontier on Healthcare Access and Quality (HAQ) Index for 2019, in the neonatal age group, in deaths per 1000 live births.

7d. The Survival Potential Frontier on Healthcare Access and Quality (HAQ) Index for 2019, in the complete under-5 age group, in deaths per 1000 live births.

7e. Mortality above both the Global Optimum benchmark and the Survival Potential Frontier on Healthcare Access and Quality (HAQ) index, in 2000 and 2019, for neonates, in deaths per 1000 live births.

7f. Mortality above both the Global Optimum benchmark and the Survival Potential Frontier on Healthcare Access and Quality (HAQ) index, in 2000 and 2019, for all children under 5 years, in deaths per 1000 live births.

7g. Proportion of deaths that is above the benchmark, among neonates, by both Global Optimum and Survival Potential Frontier benchmarks, in 2000 and 2019.

7h. Proportion of deaths that is above the benchmark, among all children under 5 years, by both Global Optimum and Survival Potential Frontier benchmarks, in 2000 and 2019.

Figure S8. Cause- and age- specific mortality by country and Healthcare Access and Quality (HAQ) Index, with the Survival Potential Frontier (SPF) (black line) and uncertainty interval around the frontier (grey band). Countries are labelled in bold when their ratio to the frontier is in the top 10 percent (performing poorly relative to HAQ Index) and in italics when their ratio to the frontier is in the bottom 10 percent (performing well relative to HAQ Index). These results show the prediction of the cause-specific SPF prior to scaling to the all-cause frontier.

Figure S9. Results by location

Section 2: **GATHER checklist**

Table S1. Checklist for compliance with the Guidelines for Accurate and Transparent Health Estimates Reporting (GATHER).

Item #	Checklist item	Reported on page #
Objectives and funding		
1	Define the indicator(s), populations (including age, sex, and geographic entities), and time period(s) for which estimates were made.	Main manuscript methods and appendix Section 2
2	List the funding sources for the work.	Main manuscript funding statement
Data Inputs		
For all data inputs from multiple sources that are synthesized as part of the study:		
3	Describe how the data were identified and how the data were accessed.	Main manuscript methods and appendix Sections 3-4
4	Specify the inclusion and exclusion criteria. Identify all ad-hoc exclusions.	Main manuscript methods and appendix Sections 3-4
5	Provide information on all included data sources and their main characteristics. For each data source used, report reference information or contact name/institution, population represented, data collection method, year(s) of data collection, sex and age range, diagnostic criteria or measurement method, and sample size, as relevant.	Main manuscript methods and appendix Sections 3-4. Detailed data sources for each component available online at GBD Input Data Sources Tool (http://ghdx.healthdata.org/gbd-2019/data-input-sources)

6	Identify and describe any categories of input data that have potentially important biases (e.g., based on characteristics listed in item 5).	Main manuscript methods and appendix Sections 3-4
For data inputs that contribute to the analysis but were not synthesized as part of the study:		
7	Describe and give sources for any other data inputs.	Main manuscript methods and appendix Sections 3-4
For all data inputs:		
8	Provide all data inputs in a file format from which data can be efficiently extracted (e.g., a spreadsheet rather than a PDF), including all relevant meta-data listed in item 5. For any data inputs that cannot be shared because of ethical or legal reasons, such as third-party ownership, provide a contact name or the name of the institution that retains the right to the data.	Detailed data sources for each component available online (http://ghdx.healthdata.org/gbd-2019)
Data analysis		
9	Provide a conceptual overview of the data analysis method. A diagram may be helpful.	Main manuscript methods and appendix sections 3-5
10	Provide a detailed description of all steps of the analysis, including mathematical formulae. This description should cover, as relevant, data cleaning, data pre-processing, data adjustments and weighting of data sources, and mathematical or statistical model(s).	Main manuscript methods and appendix sections 3-5
11	Describe how candidate models were evaluated and how the final model(s) were selected.	
12	Provide the results of an evaluation of model performance, if done, as well as the results of any relevant sensitivity analysis.	
13	Describe methods for calculating uncertainty of the estimates. State which sources of uncertainty were, and were not, accounted for in the uncertainty analysis.	Main manuscript methods and appendix sections 3-5
14	State how analytic or statistical source code used to generate estimates can be accessed.	All statistical code is published on GitHub (Core GBD 2019 code: link; secondary analyses: link; stochastic frontier analysis tool: https://github.com/UW-AMO/StochasticFrontier).
Results and Discussion		
15	Provide published estimates in a file format from which data can be efficiently extracted.	GBD 2019 results are publicly available from the GHDx online Results Tool (http://ghdx.healthdata.org/gbd-results-tool).
16	Report a quantitative measure of the uncertainty of the estimates (e.g. uncertainty intervals).	Main manuscript results text includes 95% uncertainty intervals. Uncertainty intervals are also present in the online Results Tool.
17	Interpret results in light of existing evidence. If updating a previous set of estimates, describe the reasons for changes in estimates.	Main manuscript discussion
18	Discuss limitations of the estimates. Include a discussion of any modelling assumptions or data limitations that affect interpretation of the estimates.	Main manuscript discussion

This checklist should be used in conjunction with the GATHER statement and Explanation and Elaboration document, found on gather-statement.org

Section 3: **GBD overview**

Section 3.1: Global Burden of Diseases, Injuries, and Risk Factors Study 2019

The Global Burden of Diseases, Injuries, and Risk Factors Study (GBD) is a collaborative research effort aimed at estimating worldwide population, fertility, morbidity, and mortality. GBD 2019

includes estimated all-cause and cause-specific mortality under the age of 5 years, by age, sex, and location. All-cause estimation was completed for the years 1950–2019 and cause-specific estimation was completed for the years 1990–2019.

Section 3.2: Geographical locations of the analysis

We produced estimates for 204 countries and territories that were grouped into 21 regions and seven super regions. For GBD 2019, nine countries and territories (Cook Islands, Monaco, San Marino, Nauru, Niue, Palau, Saint Kitts and Nevis, Tokelau, and Tuvalu) were added, such that the GBD location hierarchy now includes all WHO member states. This year, GBD includes subnational analyses for Italy, Nigeria, Pakistan, the Philippines, Poland, and 16 countries previously estimated at subnational levels (Brazil, China, Ethiopia, India, Indonesia, Iran, Japan, Kenya, Mexico, New Zealand, Norway, Russia, South Africa, Sweden, the UK, and the USA). All analyses are at the first level of administrative organization within each country except for New Zealand (by Māori ethnicity), Sweden (by Stockholm and non-Stockholm), the UK (by local government authorities), and the Philippines (by provinces).

In this analysis we present subnational estimates for only Brazil, the UK, and the USA.

A complete list of all locations in the GBD 2019 analysis, including which Socio-demographic index (SDI) quintile each location belongs to, can be found in Wang et al 2020[1].

For reference, ISO3 country codes referenced in this paper are:

ALB = Albania; AND = Andorra; ARE = United Arab Emirates; ASM = American Samoa; AZE = Azerbaijan; BEN = Benin; BFA = Burkina Faso; BHR = Bahrain; BTN = Bhutan; BWA = Botswana; CAN = Canada; CMR = Cameroon; COK = Cook Islands; CUB = Cuba; CZE = Czechia; DMA = Dominica; DOM = Dominican Republic; DZA = Algeria; EGY = Egypt; ERI = Eritrea; EST = Estonia; FSM = Micronesia (Federated States of); GHA = Ghana; JAM = Jamaica; JOR = Jordan; JPN = Japan; KIR = Kiribati; KNA = Saint Kitts and Nevis; KWT = Kuwait; LBN = Lebanon; LTU = Lithuania; LVA = Latvia; MCO = Monaco; MDV = Maldives; MHL = Marshall Islands; MLI = Mali; MLT = Malta; MNE = Montenegro; MYS = Malaysia; NER = Niger; NGA = Nigeria; NIU = Niue; OMN = Oman; PAK = Pakistan; PRI = Puerto Rico; SDN = Sudan; SGP = Singapore; SLE = Sierra Leone; SRB = Serbia; SVN = Slovenia; TKL = Tokelau; TKM = Turkmenistan; TON = Tonga; TUN = Tunisia; TUR = Turkey; TUV = Tuvalu; USA = United States of America; VIR = United States Virgin Islands; VUT = Vanuatu; WSM = Samoa; YEM = Yemen; ZAF = South Africa.

Section 3.3: Age groups included in the analysis

While the GBD includes additional adult age groups, the focus of this paper is ages under 5 years. This includes both detailed and aggregate ages in the following hierarchy:

- Under-5
    - Infant (<1 year)
        - Neonatal (<28 days month)
            - Early neonatal (0–6 days)

            - Late neonatal (7–27 days)
        - Post-Neonatal (28–364 days)
            - 1–5 months*
            - 6–11 months*
    - 1-4 years
        - 1 year*
        - 2–4 years*

Where * indicates age groups only included for all-cause mortality estimation.

These age groups are written with demographic notation, such that, for example, 1-4 years indicates the age group starting at the 1st birthday and ending with the 5th birthday, and 28-364 days and 1-5 months/ 6-11 months, indicates the age group starting at the beginning of the first month of life and ending at the 1st birthday. Where data sources present age detail in days, the 1-5 month age group starts at 28 days,

In some cases, we also combine post-neonatal with 1-4 years, to get the following split of under-5:

- Neonatal (0-27 days)
- Under-5 greater than 28 days (28 days to 5th birthday)

Section 3.4: Causes of death in the analysis

A complete cause hierarchy with information about which causes are on each level of the hierarchy, which causes only have non-fatal outcomes (not a cause of death), and which causes are relevant for children under the age of 5, can be found in Table S6.

Of note, one important cause to children under-5 is "neonatal disorders". This cause includes neonatal preterm birth, neonatal encephalopathy due to birth asphyxia and trauma, neonatal sepsis and other neonatal infections, hemolytic disease and other neonatal jaundice, and a residual other neonatal disorders category. It does not include all causes of death for neonates, rather, causes that are specific concerns within the neonatal period.

Section 3.5: Statement of GATHER compliance

This study complies with the Guidelines for Accurate and Transparent Health Estimates Reporting (GATHER) recommendations. We have documented the steps involved in our analytical procedures and detailed the data sources used. See Appendix Table S1 for the GATHER checklist. The GATHER recommendations can be found here: http://gather-statement.org/

Section 3.6: GBD results resources

Results are available for download on the Global Health Data exchange (GHDx): http://ghdx.healthdata.org/gbd-results-tool. GBD 2019 results are also viewable at the GBD Compare visualization: http://vizhub.healthdata.org/gbd-compare.

Section 3.7: Data input sources overview

Citations for specific GBD components and locations can be found through the Data Input Sources Tool in GHDx: http://ghdx.healthdata.org/gbd-2019/data-input-sources. This tool includes metadata for input sources as required by GATHER.

Section 3.8: Funding sources

Research reported in this publication was supported by the Bill & Melinda Gates Foundation, the University of Melbourne, Public Health England, the Norwegian Institute of Public Health, the National Institute on Aging of the National Institutes of Health (award P30AG047845), and the National Institute of Mental Health of the National Institutes of Health (award R01MH110163). The content is solely the responsibility of the authors and does not necessarily represent the official views of the Bill & Melinda Gates Foundation or the National Institutes of Health. The funders of the study had no role in study design, data collection, data analysis, data interpretation, or writing of the report. All authors had full access to all data in the study and had final responsibility for the decision to submit for publication.

Section 4: **All-cause mortality, HIV mortality, and fatal discontinuities**

All-cause mortality for both sexes, for all age-groups, locations, and years, were estimated as published in the core GBD 2019 papers. The following text is reproduced from the appendix of the ***"Global, regional, and national age-specific fertility, mortality and population estimates, 1950–2019: a comprehensive analysis for the Global Burden of Disease Study 2019"*** *manuscript. Excepting adult mortality estimation, this text is reproduced here in its entirety so not all sections may be specifically applicable to the current analysis of neonatal and under-5 mortality.*

Section 4.1: Overview

We aimed to generate the most accurate estimates for all-cause mortality estimates for all GBD age groups, by sex, for all 1062 locations in the GBD 2019 location hierarchy, for a 70-year time series from 1950 to 2019.

One challenge to estimating under-5 mortality is data availability and quality. Not all countries and territories have complete vital registration (VR) systems recording the event of death and periodic censuses. Several methods were used to estimate completeness and adjust VR accordingly, and to incorporate other source types such as household surveys.

Here, we provide a detailed description of the methodology we used to estimate all-cause mortality under the age of 5 years, within the GBD analytical framework. Due to the interdependence between HIV mortality estimation and all-cause mortality estimation, this appendix includes a partial overview of the epidemiological modelling of HIV. In what follows, we describe the major methodological tasks: estimating the both-sex probability of death

between birth and age 5 years ($_5q_0$); estimating the age- and sex- specific probabilities of death for detailed age groups under 5 years; calculating under-5 death numbers using live births and these probabilities of death; estimating under-5 HIV mortality; and producing final estimates of age-specific mortality, including HIV mortality and fatal discontinuities.

Section 4.2: Child mortality

Section 4.2.1: **Data sources**

Overview

For the estimation of child mortality, we used data from vital registration (VR) systems, sample registration systems (SRS), and disease surveillance point systems, household surveys (complete birth histories [CBH], summary birth histories [SBH]), censuses (SBHs, CBH in rare occasions), and Demographic Surveillance Sites (DSS). It was important that data were fully representative of the given age-sex group and location-year.

VRs, sample registration systems, and DSPs from other sources

We tried to incorporate all available data from VR systems in our all-cause mortality estimation process. These included multi-country VR sources such as the WHO Mortality Database, United Nations Demographic Yearbooks, Human Mortality Database, country statistics offices, and the Organization for Economic Co-operation and Development (OECD) databases. We updated the data from these sources in our systems as new data were updated. Wherever possible, we also catalogued all data sources from each system for ongoing national VR systems (eg, the USA National Vital Statistics System).

When vital registration data were not available, we also extracted data from the Sample Registration System from India and the Disease Surveillance Points system from China. Data from the Health and Demographic Surveillance System were also used.

Under-5 populations and livebirths

Livebirths and population in the under-5 age groups were produced as part of the GBD fertility and population estimation.[1]

CBH microdata

Where VR data were unavailable or unreliable, complete birth histories (CBHs) were the preferred source for child mortality data. CBHs are surveys conducted with mothers about all livebirths they have ever had by the time of the survey regardless of their survival status, including birthdate, survival status, and date of death if deceased. Since CBH data include the age at which mothers gave birth to each child, we could calculate period and age-specific mortality rates in the time period before the survey, assuming no survivor, migration-related, or recall bias. Many surveys, including the Demographic and Health Surveys (DHS), World Fertility Surveys (WFS), Multiple Indicator Cluster Surveys (MICS), and many national surveys include CBH modules. When available, we calculated mortality rates using micro-level data from surveys instead of the reported values for three five-year periods that are typically reported by surveys.

CBH tabulated data

Some reports release tabulated results before they release microdata, so we also included these datapoints in our probability of death from birth to age 5 years ($_5q_0$) database. Once we were able to obtain microdata from the same surveys, however, we replaced tabulated report estimates in our database with the microdata.

SBH microdata

Summary birth history (SBH) questionnaires are shorter and less detailed than CBHs and lack information on dates of birth and death of children. They simply ask mothers the number of livebirths they have ever had and how many of those children have died, as well as the age of the mother at the time of the interview. For this study, we collected all available SBH data that had microdata, or those that reported data on the proportion of a mother's children who died, by maternal age group. GBD has developed its own summary birth history method to generate U5MR using the aforementioned SBH data.

Under-5 age-sex patterns for VR/SRS/DSP

In high-income countries, under-5 mortality estimates were derived primarily from VR system data. Often, these data were divided into age groups: early neonatal (0-6 days), late neonatal (7-27 days), post-neonatal (28-364 days), and 1 to 4 years. Some country-years of data had less specific age groups, with early and late neonatal or all under-1 groups combined. Age patterns of mortality in the under-5 age groups were also available from sample registration system and disease surveillance point systems.

Under-5 age-sex patterns from CBH

Aside from VR/SRS/DSP, complete birth histories (CBHs) from surveys provide another important source on the distribution of mortality among under-5 age groups. These data reflect age patterns primarily from low-income countries and countries with higher levels of under-5 mortality. We only used datapoints from the 15 years leading up to the survey, so children who were born to mothers more than 15 years before they were surveyed were not included in our analysis. We divided observations into 5-year time periods in order to produce more robust input point estimates of the probability of death for each under-5 age-sex group.

VR prioritisation

As we worked with a variety of sources of vital registration data, we developed a rank ordered list of preferred VR data sources. For a given location-year, our first preference was to use WHO data from GBD cause-specific mortality estimation, followed by unadjusted WHO data, then Human Mortality Database (HMD) data, and then UN Demographic Yearbook data. We made exceptions in some cases, however. We assessed single-country VR data sources according to whether there were inconsistencies with other data sources or VR system documentation. We used HMD VR data for Germany, Taiwan (Province of China), and Spain, for instance, because WHO data produced mortality rates inconsistent with established trends.

Section 4.2.2: **Computation of 5q0 from CBH**

Processing of CBH microdata

We used CBH microdata from unpooled surveys to calculate biennial under-5 mortality rate. We analyzed each survey separately (eg, Kenya DHS 2014; Bangladesh MICS 2012–2013) instead of pooling all DHS or MICS CBH surveys from a single country (eg, Nigeria MICS surveys from 2007, 2011, and 2016–2017) so that we could better understand and address data quality issues in specific surveys. By grouping observation points into two-year intervals to generate biennial estimates, we overcame the issues of smaller sample sizes and lower stability that came from having unpooled data. We only included biennial estimates that contained more than 10 000 person-months of data.

Processing of tabular CBH

Microdata were not available for all CBH surveys. We still added results from these surveys to our database if survey reports provided ${}_5q_0$ datapoint estimates based on CBH.

Section 4.2.3: **Computation of 5q0 from SBH**

We used an SBH method developed and updated by Rajaratnam and colleagues to estimate ${}_5q_0$ from SBH data from censuses and surveys.[2] We found this method to be more accurate and provide more timely estimates than previous methods.

In cases where the only data available were tabular data of number of livebirths ever born and number of children who have died, aggregated by the mother's age, we applied Rajaratnam and colleagues' Maternal Age Cohort model.[2]

Section 4.2.4: **Computation of 5q0 from other sources**

Death count data from VR, SRS, and DSP are divided by population estimates for under-1 and 1-4 year ages to get mortality rate, then converted to ${}_5q_0$ using traditional demographic techniques. Household surveys with complete birth histories (CBH) provide detailed information on all live births, including date of birth and date of death when applicable, by mother. Period-specific deaths under age 5 per live births can be directly calculated from this survey data.

Section 4.2.5: **5q0 data synthesis**

Overview

To synthesize 5q0 data into both-sex 5q0 estimates for every GBD location-year, we used a multi-stage process. First, we ran a non-linear mixed effects model to predict 5q0, including fixed effects on each source-type and random-effects on individual sources. Next, we utilize the source-based fixed and random effects to define a bias correction on sources deemed to be non-reference by expert opinion. The second-stage predictions are made by smoothing the residuals from the first stage model and the bias-adjusted data, over the space (distance along the GBD location hierarchy) and time (in years). Finally, the second-stage predictions are used as a mean prior to Gaussian process regression, which incorporates data variance to produce final estimates of under-5 mortality with 95% uncertainty intervals (UIs).

First-stage non-linear mixed effects model

To estimate data bias and provide first stage estimates for $_5m_0$, we fit a non-linear mixed effects regression model:

$$ {}_5m_{0_{ctsi}} = \exp[\alpha_k + \gamma_{0c} + \gamma_{0cs} + (\beta_1 + \gamma_{1c}) * \log(LDI_{ct}) + (\beta_2 + \gamma_{2c}) * education_{ct}] + \beta_3 * HIV_{ct} + \varepsilon_{cysi} $$

$$ \gamma_{0c} \sim N\left(0, \sigma^2_{\gamma_0}\right) $$
$$ \gamma_{1c} \sim N\left(0, \sigma^2_{\gamma_1}\right) $$
$$ \gamma_{2c} \sim N\left(0, \sigma^2_{\gamma_2}\right) $$
$$ \gamma_{0cs} \sim N\left(0, \sigma^2_{\gamma_{0s}}\right) $$
$$ \varepsilon_{cts} \sim N(0, \sigma^2_{\varepsilon}) $$

Where

Index c is location, t is time, s is source, k is source type of datapoint i

$_5m_0$ is under-5 mortality rate

LDI is lag-distributed income per capita

education is mean years of education for women of reproductive age (15-49 years)

HIV is crude death rate due to HIV in age groups 0-4

γ_{0c} is a country random intercept

γ_{0cs} is a country-source random intercept

γ_{1c} is a country random slope for *LDI*

γ_{2c} is a country random slope for *education*

α is a fixed intercept for source

β_i is a fixed covariate coefficient

ε is the residual

$\gamma_{0c}, \gamma_{0cs}, \gamma_{1c}, \gamma_{2c}$ are all pairwise independent

Sources were categorized into one of the 17 source types (k) listed below. LDI and education estimates come from the GBD covariates database. This regression used HIV mortality rates estimated as part of GBD 2019.

First-stage prediction of 5q0 is generated using the following equation:

$$ predicted_5m_{0,ct} = \exp[\alpha_t + \beta_1 * \log(LDI_{ct}) + \beta_2 * education_{ct}] + \beta_3 * HIV_{ct} $$

Here, the prediction only uses the fixed effects from the non-linear mixed effects model, and the fixed effects for source type are set to zero, the value for complete vital registration systems.

Bias correction

Removing systematic differences in sources is necessary such that we avoid estimating false trends. In particular, it allows us to utilize death count data from registration systems known to have under-enumeration and helps us avoid trends caused by overlapping sources with different levels of non-sampling variance.

The basis of our bias correction method is a comparison to a subset of sources deemed to be unbiased, which we call reference sources. We turned to country experts to identify data sources in each location that were likely to be least biased, of higher data quality to be used as reference source or sources. When it was believed that a VR system was complete, we used it as the reference source. When data from complete VR systems were not available, we used DHS estimates from CBHs as the reference source if available. Specific source or sources were selected for a location by location basis when neither complete VR nor CBH from DHS or other surveys were available.

The combination of source-type fixed effect and source-specific random effect in the first stage model are used to formulate the bias adjustment. The following source types were included separately in the first stage model:

CBH: AIDS Indicator Survey and Malaria Indicator Survey

CBH: Census

CBH: Demographic and Health Survey

CBH: Multiple Indicator Cluster Survey

CBH: World Fertility Survey

CBH: Other survey series

Household Death Recall: Census

Household Death Recall: Other survey series

Household Death Recall: Incomplete VR/Sample Registration/Surveillance

SBH: AIDS Indicator Survey and Malaria Indicator Survey

SBH: Census

SBH: Demographic and Health Survey

SBH: Multiple Indicator Cluster Survey

SBH: World Fertility Survey

SBH: Other survey series

VR/Sample Registration/Surveillance: complete

VR/Sample Registration/Surveillance: incomplete

To produce a dataset of bias-adjusted input data, we performed the following calculations. All non-reference sources were adjusted based on the difference between the bias value from the

reference source and the source of interest. We did not adjust reference sources when more than one source was used as the reference source. In this case, all other sources were adjusted to the average bias values of all selected reference sources. For incomplete VR data, we calculated an upward adjustment using a five-year rolling mean of the difference between incomplete VR data and a Loess regression of the already-adjusted survey data.

Spatiotemporal smoothing

To smooth the residuals between the adjusted data and the $_5q_0$ time series predicted using the non-linear mixed effect model, we applied a combination of spatiotemporal smoothing functions. This stage smoothed the residuals over time and across locations within each GBD region, in order to bring predictions closer to the data while maintaining smooth trends over space and time.

First, we calculated the residuals between the first stage prediction and the adjusted data. Next, we applied the smoothing functions to the residuals. We weighted residuals in each region for each location-year based on how close the data were to this location-year in space and time. 99% of the weight went to in-location residuals, with the remaining 1% going to out-of-location but within GBD region residuals. The weight for time is defined using the equation below:

$$w_t = \left(1 - \left(\frac{|r_t - r_{est}|}{1 + argmax_t|r_t - r_{est}|}\right)^{\lambda}\right)^3$$

Where

r_t is the year of interest

r_{est} is the year of the residual being weighted

$argmax_t|r_t - r_{est}|$ is the maximum distance between the year of interest and a residual within the region

λ is the weighting function that dictates how quickly the weights fall off as the distance in time increases

When λ is larger, the assigned weights diminish more slowly over time compared to a smaller λ. Next, we used a linear fit to the weighted data (akin to a Loess fit) to generate a single estimate of the smoothed residuals. We also generated a second estimate of the smoothed residuals using the weighted average of the data.

We then pooled the two estimates of the smoothed residuals into one final estimate. We assigned more weight to the local linear fit in countries and territories with high quality and completeness of data and more weight to the weighted average in countries and territories with sparse data using the following equation:

$$final\ smoothed\ residual = k * linear\ estimate + (1 - k) * weighted\ average$$

Where

$$k = \frac{number\ of\ in\ country\ datapoints}{number\ of\ in\ country\ datapoints\ +\ number\ of\ country\ years\ with\ no\ data}$$

The pooling of two estimates of the smoothed residuals is developed to account for artificial trend in smoothed residuals from either method especially when the year of interest is further away from the last year where there is empirical data. Such a method also helps improve comparability among estimates for different locations that otherwise might need to employ different method for smoothing the residuals.

Last, we incorporated the smoothed residuals back into the above predictions to produce a smoothed approximation of the adjusted data. We used this estimate as the prior for the GPR explained in the next section.

To void producing space-time smoothed U5MR that is over 1 or under zero, both space-time smoothing and the Gaussian process regression described below are done using U5MR in logit space.

Gaussian process regression (GPR)

We used Gaussian process regression (GPR) to generate a final time series of point estimates as well as UIs.

The GPR model was as follows:

$$\mu_{c,t} = f_c(t) + S_{c,t}$$

$$f_c(t) \sim GP(M, C)$$

Where

t is time

c is location

μ_t is the true $\log_{10}({}_5q_0)$

$f(t)$ is the baseline mortality risk

S_t is excess mortality due to fatal discontinuities estimated independently of $f(t)$

M is the mean function for the Gaussian process

C is the covariance function for the Gaussian process

The mean function (M) is an initial guess at our unknown function f_c. We choose this to be our second-stage predictions. The covariance function (C) defines the covariance between any two points t_i and t_j. It controls the smoothness of realizations from the Gaussian process, and the amount of deviation from the mean that is tolerated. There are some conventions regarding good choices for the covariance function in Gaussian process regression, but no single function that must be used. We select the Matérn covariance function:

$$C_{matern}(x_i, x_j \mid a, s, v) = a^2 \frac{1}{\Gamma(v)2^{(v-1)}} \left[\sqrt{2v}\frac{|x_i - x_j|}{s}\right]^v K_v\left(\sqrt{2v}\ \frac{|x_i - x_j|}{s}\right)$$

Where a is amplitude, s is scale, ν is degree of differentiability, Γ is the gamma function, and K_ν is the modified Bessel function of the second kind and order ν. To select the *amplitude* parameter, we first calculated the variance of logit-difference between the first-stage and second-stage predictions in high data-density years separately by location. Then, this location-specific variance was used as amplitude for countries with complete VR and the mean of these variances was used as amplitude for countries with incomplete VR. *Scale* is fixed by location based on data density (Appendix Table S2), and degree of differentiability is fixed at 1.

In addition to our Gaussian process prior for f, we observe the value of f for some set of years o_i – this is our data. We assume the observation error is normally distributed and calculate the data variance as described in the next section. Then, the posterior distribution for f given the observations and the prior is another Gaussian process with a closed form solution for posterior $\widetilde{M}$ and $\tilde{C}$. Let V be a matrix with observation variances $[v_0, \ldots \; , v_{n-1}]$ on its diagonal and o be the vector of years for which we've observed $f(o)$. Then the posterior distribution, $\tilde{f}$, is:

$$\tilde{f}_c \mid data \sim GP(\widetilde{M}, \tilde{C})$$

$$\widetilde{M}(t) = M(t) + C(t,o)[C(o,o) + V]^{-1}\big(f(o) - M(o)\big)$$

$$\tilde{C}\big(t_i, t_j\big) = C\big(t_i, t_j\big) - \; C(t_i, o)[C(o,o) + V]^{-1} \, C\big(o, t_j\big)$$

From this Gaussian process posterior distribution, we take realizations, where one realization is a complete function from our domain of years to the outcome of interest. 1000 realizations are draws that can be collapsed to get a mean and 95% uncertainty interval for every location and year separately.

GPR was implemented using the PyMC package in Python.

Data variance

We calculated data variance for each $_5q_0$ input datapoint, which reflects sampling errors. Additional non-sampling errors were added when the datapoint was from a non-reference data source. Specifically, we calculated data variance using the following methods, depending on the type of data:

Complete VR data: we assumed there was only sampling error, not non-sampling error, as complete VR is assumed to be unbiased. We calculated this error using the binomial distribution $p(1-p)/N$, where N is the population aged 0 to 5 years for the location and p is the under-5 mortality rate, $_5m_0$. We then used the delta method[3] to transform the variance of $_5m_0$ to the variance of $\log_{10}(_5q_0)$.

Incomplete VR data: we included both sampling error and non-sampling error from uncertainty in our estimate of data completeness. We calculated the total data variance as the sum of the variances from sampling error (see calculation method for complete VR data) and non-sampling error based on the completeness estimate.

CBH data: f draws of $_5q_0$, transformed these draws into $\log_{10}$ space, and calculated the sampling variance from the transformed draws.

SBH data: we used the standard error from the mean residuals.

Other: we used the maximum standard error from non-VR points in a country or territory if we could not produce data variance for a source. When no other source was available, we set the data variance to be the maximum standard error from non-VR data sources within the GBD region the location belongs to.

Finally, for all data types other than complete VR data, we calculated the within-source-type variance of the source-specific random effect for each source type. We then converted it to $\log_{10}$ space and added it to the variance already calculated using one of the methods above.

Hyperparameter selection

We selected hyperparameters based on a data density score for each location, first used in GBD 2017.[4] We calculated data density scores based on the number of deaths from VR sources plus the number of unique CBH and SBH sources available for the given location, using the following computational methods for each type of data source:

Complete VR data density score: calculated based on the number of deaths reported from unbiased VR sources in a single location. We capped the number of deaths for each source-year at 500 and then divided that number by 500 to generate a score between 0 and 1 where 1 represents a complete VR system with at least 500 registered deaths and a 0 represents a complete VR system with 0 registered deaths. Final complete VR scores ranged from 0 to 68.

Incomplete VR data density score: calculated the same way as with complete VR data, using biased VR sources rather than unbiased VR sources.

Total CBH sources: the number of unique complete birth histories for a single location.

Total SBH sources: the number of unique summary birth histories for a single location.

After making these calculations, we used the following formula to compute the final data density for each location:

$$data\_density = complete\_vr\_deaths + (0.5 \times incomplete\_vr\_deaths) + (2 \times cbh\_sources) + (0.25 * sbh\_sources)$$

We then assigned hyperparameters based on final data densities, as designated in Table S2. Zeta determines the magnitude of space weights in the spatiotemporal smoothing. Lambda determines the magnitude of time weights. Scale is the scale parameter of the Matérn covariance function, which controls correlation over time.

Table S2: Hyperparameter selection based on data density

Data density	Zeta	Lambda	Scale
0 to 9	0.7	0.7	15
10 to 19	0.7	0.5	15
20 to 29	0.8	0.4	15
30 to 49	0.9	0.3	10
50 plus	0.99	0.2	5

Section 4.2.6: **Identify and remove outliers**

We carefully reviewed child mortality data and estimates to ensure quality. First, we outliered datapoints from years in which fatal discontinuities occurred, unless cause of death information was available for the VR datapoint such that deaths from specific causes of death identified as fatal discontinuity could be removed. We excluded fatal discontinuity data because our objective was to identify the underlying mortality risk, which would be warped by mortality data from large stochastic events. We added back fatal discontinuity data later in the estimation process. Second, we outliered data sources whose data quality we were concerned about, such as the Afghanistan DHS from 2010. Collaborators from our extensive network reviewed sources in which they had expert knowledge and identified those with known quality issues.

Section 4.2.7: **Rake subnational estimates to national level (excluding South Africa)**

One strength of GBD is that we ensure consistency throughout our location hierarchy, meaning that subnational mortality estimates always add up to national mortality estimates. The estimation process for ${}_5q_0$ described above does not enforce this consistency, so the following adjustments in locations with subnational estimates after the aforementioned estimation process are made. We first population-weighted the subnational estimates to form an estimate at the national level. A scalar was then calculated by year by dividing the separately estimated national value by this weighted average of subnational estimates. We then multiplied the scalars from this calculation by all the subnational estimates to get scaled estimates for each subnational location. We chose this strategy for subnational scaling because national-level estimates are generally more reliable as they are more likely to be based on more empirical data and the data themselves are less likely to be affected by the small number issue. The effect of scaling was minimal in locations where VR data were high-quality.

South Africa was the one country where we did not use this method. We instead aggregated provincial-level estimates from the GPR to the national level. This was to ensure consistency between our estimates at the subnational level and the observed deaths from the vital registration system in South Africa at the national level.

Section 4.2.8: **Review estimates for quality**

We compared our ${}_5q_0$ estimates with GBD 2017 and UNICEF estimates from 2017 and 2019.[3, 4] We identified all differences in results and determined whether they were caused by updates to the available data, changes to the hyperparameters we set, or modifications to input covariates. We made revisions to our estimates as part of this review process and through consultation with collaborators in the GBD mortality network.

Section 4.2.9: **Under-5 age and sex pattern model estimation**

To disaggregate under-5 mortality into age- and sex-specific groups, we used a multi-stage modelling process similar to the method first published in GBD 2016.[5] The result of this process is sex-specific mortality for the early neonatal (0-6 days), late neonatal (7-27 days), post-neonatal (28-364 days), and childhood (1-4 years) age groups.

First, we estimated the ratio of male to female $_5q_0$, and second, we estimated the sex-specific age distributions of under-5 mortality. Both the sex and age components have the following stages: (1) generalized linear model, (2) spatiotemporal smoothing, and (3) Gaussian process regression. Finally, the components are scaled and synthesized into age- and sex-specific mortality.

Data from VR, sample registration system, and CBH sources were converted to mortality risks for each age group. Some sources included age-specific data for all four age groups, while others only had infant (early, late, and post-neonatal) and child distinctions.

The sex model first estimated the ratio of male to female $_5q_0$ for each location i in region j in year y, in rescaled logit space. We excluded observed ratios below 0.8 or above 1.5, then rescaled the ratio data from 0.8–1.5 to 0–1 to place our data within the domain of the logit function. Data from non-standard locations were also excluded in the first-stage model fitting.

With these data, we fit the following sex-ratio model:

$$logit\ \left(\frac{Male\ {}_5q_0\ scaled}{Female\ {}_5q_0\ \ scaled}\right)_{ijt} = \beta_0 + \delta_j + \gamma_i + S_{{}_5q_0} + \varepsilon_{ijt}$$
$$\delta_j \sim N\left(0, \sigma_{\delta_j}^2\right)$$
$$\gamma_i \sim N\left(0, \sigma_{\gamma_i}^2\right)$$
$$\varepsilon_{ijt} \sim N(0, \sigma_\varepsilon^2)$$

Where

i is location

j is region

t is time

β_0 is an intercept term

$S_{{}_5q_0}$ is a natural spline on both-sex $_5q_0$

δ_j is the region random intercept

γ_i is the location random intercept (nested in region)

ε is the residual

We then used the parameter estimates from this model, and our estimated $_5q_0$, to predict first-stage sex-ratio of under-5 mortality for all GBD location-years. Spatiotemporal smoothing and GPR were used to produce final sex-ratio estimates. We chose ST-GPR hyperparameters using the same method as in Section 3.2.5.

Next, we estimated $_5q_0$ for males and females using the equation:

$${}_5q_0 = \left(\frac{1}{1 + r_{birth}}\right) * (female\,{}_5q_0) + \left(\frac{r_{birth}}{1 + r_{birth}}\right) * (male\,{}_5q_0)$$

where r_{birth} is the sex-ratio at birth, ${}_5q_0$ is the direct output from the estimation process described in Section 3.2.5, and male ${}_5q_0$ can be replaced with female ${}_5q_0$ times the predicted ${}_5q_0$ sex-ratio to create a system with one unknown.

We then fit a separate age-specific model for each sex-specific age group, resulting in five models for each sex: early neonatal, late neonatal, post-neonatal, infant (neonatal, late neonatal, and post-neonatal combined), and child. The outcome modelled was the log-proportion of sex-specific under-5 deaths occurring in age group x, in other words, the probability of death in age group x conditional on death before age 5. This outcome facilitates scaling to our estimates for ${}_5q_0$.

The first-stage model for estimating this age distribution is:

$$log(Pr(death\ at\ age\ x|u5\ death)_{jits})$$
$$= \beta_0 + \gamma_{js} + \gamma_{is} + a_{1x}\beta_{1s} * HIV_{its} + a_{2x}\beta_{2s} * Mat.Ed._{it} +$$
$$a_{3x}\beta_{3s} * Completeness_{it} + S_{{}_5q_0} + \varepsilon_{jits}$$

$$\gamma_{js} \sim N(0, \sigma^2)$$
$$\gamma_{is} \sim N(0, \tau^2)$$

Where

x is age, s is sex, i is location, j is region, t is time

γ_i is a country random intercept (nested in region)

$\gamma_{j[i]}$ is a region random intercept

$S_{{}_5q_0}$ is a natural spline on ${}_5q_0$

β_i is a fixed covariate coefficient

a_{1x} is an indicator for HIV covariate inclusion: 0 for enn, 1 otherwise

a_{2x} is an indicator for maternal education covariate inclusion: 1 for child, 0 otherwise

a_{3x} is an indicator for source completeness covariate inclusion: 1 for child, 0 otherwise

ε is the residual

The literature on HIV mortality in different under-5 age groups is limited, and there is no clear guidance on how to age-split under-1 deaths due to HIV.[6,7] Prior research has, however, suggested that HIV mortality risk differs between different under-5 age groups,[7,8] with essentially all HIV deaths that occur in the first year of life occurring in the post-neonatal stage. We therefore included under-5 crude death rates from HIV from the GBD 2019 model in the model for post-neonatal, infant, and child, but not the neonatal age groups. Including this covariate improved the fit and predictive validity of the model in countries with high HIV prevalence. In locations where HIV/AIDS contributes a large burden to under-5 mortality, using ${}_5q_0$ as the only predictor in this model typically leads to an overestimation of neonatal mortality.[9]

We also included a maternal education covariate and a source-specific child completeness covariate for the 1 to 4 age group only. We calculated child completeness by dividing the source-specific $_5q_0$ point estimate by the final GPR $_5q_0$ estimate. In the prediction of this model, we used a completeness value of 1, to predict from a hypothetically complete source.

After prediction of the first-stage model for all GBD location-years, ST-GPR was used to generate stage-three results by age and sex. We used the same ST-GPR process as described previously for the overall under-5 mortality rate.

Finally, we ensured consistency in probability of death between the separate and collective under-5 age groups by scaling. We first scaled the infant and child conditional probabilities to add to 1 and then scaled the early neonatal, late neonatal, and post-neonatal conditional probabilities to add to the infant conditional probability. We also scaled 1-5 month and 6-11 month conditional probabilities to overall post-neonatal probability and 12-23 month and 2-4 year conditional probabilities to overall infant (1-4 year) probability.

Next, we calculated the probability of early neonatal death by multiplying the rescaled conditional probability of early neonatal death given under-5 death by $_5q_0$ using the equation below:

$$q_{enn} = \Pr(death\ in\ enn\ |\ u5\ death) * 5q0$$

where enn is early neonatal.

To calculate the probability of late neonatal death, we multiplied the rescaled conditional probability of late neonatal death given under-5 death by $_5q_0$ and then divided that by the probability of survival to the start of the age group (7 days), using the following equation:

$$q_{lnn} = \Pr(death\ in\ lnn\ |\ u5\ death) * 5q0/(1 - q_{enn})$$

where lnn refers to late neonatal.

We calculated probability of death for the remaining age groups in the same way.

Section 4.2.10: **Identify and remove outliers from age and sex models**

We removed outliers from the under-5 age-sex pattern model in several instances.

For the sex model, we removed non-VR datapoints from locations with high-quality VR data (based on the GBD VR quality rating system) to make sure we were only using the highest-quality data.

For the age model, we outliered the following:

1. VR data that were incomplete. We compared the nine-year rolling average of the $_5q_0$ value from the VR data to the nine-year rolling average of the $_5q_0$ estimates and then the raw data value of $_5q_0$ to the final $_5q_0$ estimate for each data-year. We outliered data for which the second comparison yielded a ratio of 0.85 or less, unless the ratio of the nine-year rolling average was above 0.85.
2. Data that were identified as outliers earlier in the $_5q_0$ analysis.
3. CBH datapoints from more than 15 years before the survey was administered.
4. CBH data in locations with both VR and CBH data, but only if the data conflicted.

5. Other datapoints that were manually outliered for various reasons. For instance, the definition of a livebirth included a minimum weight requirement in some eastern European countries before the 1990s, which led to inconsistencies in livebirth data over time. We therefore outliered age group data in ages that would include childbirth deaths when and where the definition of livebirth included a minimum weight.

Section 4.2.11: **Under-5 death number estimation**

We estimated the number of under-5 deaths by ageing birth cohorts through our estimated age-sex-specific probabilities of death. To do this, we divided the number of births for each location-year into weekly birth cohorts and moved these cohorts through our mortality estimates in weekly increments. This process produced estimates of the number of person-years and deaths in each under-5 age group.

Section 4.3: HIV/AIDS estimation

Section 4.3.1: **HIV-free survival rates (for Spectrum)**

Our inputs for Spectrum were single-year HIV-free mortality based on five-year abridged life tables, generated from the age-specific HIV-free survival probabilities produced through the process described above.

Section 4.3.2: **EPP and Spectrum**

For our HIV analysis, we modified two tools developed by UNAIDS:

1. EPP and EPP-Age Sex Model (EPP-ASM):
    a. These tools generate HIV burden estimates in line with observed prevalence data. They incorporate many of the same assumptions as Spectrum. EPP fits a simpler model to HIV prevalence data from representative surveys and surveillance sites to estimate prevalence and incidence for the 15–49 year age groups. EPP-ASM incorporates the full population project of Spectrum and produces age-sex-specific prevalence, incidence, and mortality.
    b. We modified EPP-ASM by building a paediatric module that mirrored that of Spectrum. Perinatal and breastfeeding transmission was calculated as a function of prevalence among pregnant women and maternal-to-child transmission programme data. We additionally improved the fit to prevalence data by allowing flexibility in the age distribution of incidence over time. We parameterised the ratio of incidence among ages 15–24:25+ as a constant before year 2000 and a linear regression thereafter. This allowed for the shifts in the age distribution of incidence observed over the course of the HIV epidemic to be reflected in our results.
2. Spectrum natural history model:
    a. This model generates HIV incidence, prevalence, and death by age, sex, and year using time series estimates of HIV incidence, demographic inputs (such as HIV-free mortality and population), assumptions about CD4 progression rates, and assumptions about on-ART and off-ART HIV mortality rates by age, sex, and CD4 rate. We used treatment coverage time series from UNAIDS.

b. We made several modifications after creating a replica of Spectrum in Python (starting with GBD 2013). First, we sex-split incidence based on a model that was fit to the sex ratio of observed prevalence in countries and territories with nationally representative surveys. Second, our child model included CD4 progression and CD4-specific mortality rates that came from a model fit to survival data from the International epidemiology Databases to Evaluate AIDS (IeDEA), as well as ART distribution data from IeDEA. Additionally, we scaled all input values by a uniformly sampled factor between 0.9 and 1.1 to generate estimates with realistic ranges of uncertainty.

Section 4.3.3: **HIV mortality reckoning**

We used the reckoning process to resolve and merge separate estimates of HIV mortality produced by two different estimation processes in the GBD framework. The separate estimates were those from the model life table system and from the natural history model of EPP-Spectrum. We also used space-time GPR-smoothed VR data on HIV-specific mortality in countries and territories with high-quality VR systems instead of mortality estimates from Spectrum.

We assigned all GBD 2019 locations to a modelling strategy group based on the level of HIV in the country or territory and the completeness of HIV and VR data. The group a location was assigned to determine which sources we used for HIV-specific mortality data and how we calculated final HIV and all-cause mortality estimates. The groups were as follows:

- Group 1A: locations with greater than 0.25% adult HIV prevalence and available HIV prevalence survey data and/or antenatal care clinic data. For all Group 1A locations, we ran the EPP model using these available data from each location. The demographic assessments for Group 1 locations relied heavily on sibling history data analyses, which had large UIs and for which there were often varied subnational response biases.
- Group 1B: India, Sudan, and Somalia. These locations constituted their own group because they had available surveillance data but fairly low adult HIV prevalence – less than 0.5%. We used the model life table system to get all-cause mortality estimates in these locations. We used EPP-Spectrum to get HIV-specific deaths. For India, we used SRS data to adjust age-specific incidence in Spectrum and generate the mortality age pattern. The adjusted result better matched observed deaths.
- Group 2A: locations with high-quality (4 or 5 star) VR systems, based on the GBD VR quality rating system.[10] All-cause mortality estimates in these locations used national data such as the VR system.
- Group 2B: locations with lower-quality VR systems.
- Group 2C: locations without any VR data.

For HIV-specific mortality estimates, Group 1A and 1B locations used Spectrum output, Group 2A locations used mortality output straight from the ST-GPR process, and Group 2B and 2C locations used output cohort incidence bias adjusted (CIBA) deaths caused by HIV/AIDS from the Spectrum model.[11]

The outputs from the model included the under-1 age group but not early-, late-, and post-neonatal age groups. We assumed that all HIV deaths that occurred in the first year of life occurred in the post-neonatal stage. Previous research is limited but suggests higher mortality in the post-neonatal stage.[6,7] There was no definitive guidance on how better to age-split under-1 HIV deaths.

We conducted a separate calculation for the under-5 age group using results from the under-5 age-sex process. For Group 1A and 1B locations, we produced a scalar from HIV-specific and non-HIV deaths Spectrum results, which we then used to produce an HIV-deleted envelope from the all-cause results in the envelope. For locations in other groups, we capped HIV mortality, which was taken directly from ST-GPR or Spectrum, at 90% of the all-cause envelope, and then subtracted it from the all-cause envelope to produce an HIV-deleted envelope. We assumed that all under-1 HIV-specific deaths happened in the post-neonatal stage.

Section 4.4: Age-specific mortality estimation for all GBD age groups: with and without HIV

Section 4.4.1: **HIV-deleted age-specific mortality**

We used HIV-deleted age-specific mortality in our CoD analyses to prevent the spillover effect of HIV mortality into other causes, which was a potential problem especially in locations with a high HIV burden. We calculated this as the difference between the HIV-specific mortality from the HIV reckoning process and the with-HIV age-specific mortality without fatal discontinuities.

Section 4.4.2: **Age-specific mortality without discontinuities (with HIV/AIDS)**

For all locations, we used our age-sex model, which split U5MR into detailed sex and age groups, to calculate under-5 age-specific with-HIV mortality.

Section 4.4.3: **Add fatal discontinuities**

We excluded data from years with fatal discontinuities from our mortality estimation process because we did not want sudden and temporary jumps in mortality to warp long-term mortality trends in that country or territory. The following section explains how we added deaths caused by fatal discontinuities to the all-cause mortality envelope and life tables.

To add fatal discontinuities mortality into the mortality envelope, we added 1000 draws of deaths due to fatal discontinuities pairwise to 1000 draws of the with-HIV mortality envelope for each location, sex, and age group. We calculated 95% UIs in the same way as for other estimates, using the 2.5% and 97.5% quantiles of the summed draws.

We then had to add fatal discontinuity deaths into the life table. To do this, we first produced full single-year with-HIV life tables, to which the fatal discontinuity deaths would later be added. We then calculated fatal discontinuity mortality rates for each single-year age by assuming the same mortality rate for each year in an age group. This kept the five-year mortality rates consistent between the abridged and full life tables. We then added the fatal discontinuity mortality rates to the with-HIV mortality rates in the full life tables, creating full and abridged life tables that had both fatal discontinuities and HIV included. To calculate the probability of death in the under-1 age group (${}_1q_0$), we used results from the under-5 age-sex model, calculated the with-fatal-discontinuities mortality rate for each of the under-1 age

groups, back-calculated $_nq_x$ for the same age groups, and aggregated to $_1q_0$. We also used the probability of death between ages 1 and 4 ($_4q_1$) that were produced by the age-sex model.

While using the same mortality rate for fatal discontinuities did, for the most part, lead to a corresponding increase in the mortality rate in the abridged life tables, there were instances where the difference in abridged mortality rate between the with- and without-fatal-discontinuities life tables did not quite align with the fatal discontinuity mortality rate that was input. There were very few instances where this occurred, and they were primarily in age groups such as 90 to 94, where without-fatal-discontinuities mortality was very high and varied within the age group, and where fatal discontinuities mortality was fairly low. As a result, the difference between the mortality rate from the final abridged life tables and those calculated using the mortality envelopes and population results was very minor.

Section 4.5: Fatal discontinuities

Fatal discontinuities are defined as events that are stochastic in nature and cannot be modelled because they do not have a predictable time trend. Some causes have both fatal discontinuities as well as a continuous background mortality that has a smooth time trend and can be modelled. These include police violence and executions; interpersonal violence; other transport injuries; fire, heat, and hot substances; poisoning by other means; other exposure to mechanical forces; non-venomous animal contact; environmental heat and cold exposure; protein-energy malnutrition; diarrhoeal disease; malaria; meningitis; measles; dengue; and other unspecified infectious diseases. Causes without a continuous background mortality and that are exclusively estimated using the fatal discontinuity method are conflict and terrorism and exposure to forces of nature. Any other causes are not captured in fatal discontinuities.

Section 4.5.1: **Input data**

Overview

We collected data on fatal discontinuities from a range of sources, namely from country-level VR systems and international databases that reported several cause-specific fatal discontinuities. We also collected supplemental data that had known issues related to quality, representativeness, or time lags in reporting. We used a Twitter scrape in place of a systematic literature review as a way to identify supplemental input data for missing fatal discontinuities. We describe the different fatal discontinuity data sources, presented by fatal discontinuity sub-cause, below.

Discontinuities only (non-CODEm)

For causes not modelled in CODEm, all deaths captured in VR were considered to be fatal discontinuities. Deaths that were extracted from cause-specific VR were then subtracted from the all-cause VR data used in the all-cause mortality estimation process.

Conflict and terrorism

In GBD 2019, war is defined as “a state of armed conflict between states, governments, societies, and paramilitary groups.” It is “generally characterised by extreme violence, aggression, destruction, and mortality, and the use of regular or irregular military forces.” Terrorism is defined as “the unlawful use or threatened use of force or violence against

individuals or property in an attempt to coerce or intimidate governments or societies to achieve political, religious, or ideological objectives." We used conflict and terrorism data from the Uppsala Conflict Data Program (UCDP), International Institute for Strategic Studies (IISS), Armed Conflict Location & Event Data Project (ACLED), Global Terrorism Database (GTD), and vital registration systems and other supplemental data sources. Case were assigned for each event using the source's cause coding and any description from available notes.

Table S3. Conflict and terrorism data sources

Data source name	Date accessed	Years of data downloaded	Type of data included
**Uppsala Conflict Data Program**[12]			
Georeferenced Event Dataset, Version 19.1	6/10/2019	1989-2018	UCDP battles, non-state, and one-sided conflict deaths with the most disaggregated location information available
PRIO Battles Deaths Dataset, Version 3.1	1/16/2018	1946-2008	Armed conflict (civil wars, etc.)
**International Institute for Strategic Studies**			
Armed Conflict Dataset	11/17/2016	1997-2016	Insurgency, Inter-state, Intra-state conflict deaths
**Robert S. Strauss Center for International Security And Law**			
Armed Conflict Location and Event Dataset (ACLED)	2/5/2019	1997-2019	Actions of opposition groups, governments, and militias in selected locations in Africa, Asia, and the Middle East specifying the exact location and date of battle events, transfers of military control, headquarter establishment, civilian violence, and rioting
**University of Maryland, Global Terrorism Database**			
Global Terrorism Database (GTD)	6/10/2019	1970-2017	Attacks aimed at attaining political, economic, religious, or social goal, includes evidence of intention to coerce, action was outside precepts of International Humanitarian Law.
**University of Chicago, Chicago Project on Security and Threats**			
Suicide Attack Database (CPOST SAD)	11/26/2018	1982-2018	Attacks in which an attacker kills him/herself in a deliberate attempt to kill others, includes only attacks perpetrated by non-state actors
**Amnesty International**			
Amnesty	6/20/2019	1991-2018	Police conflict and executions

Four major conflicts were identified that were not represented in these databases: genocide in Bangladesh in 1971,[13] genocide in Burundi in 1972 and 1993,[14] and civil conflict in Albania in 1997.[15] In these cases, literature sources were used to account for these fatal discontinuities.

Exposure to forces of nature

In GBD 2019, exposure to forces of nature is defined as "a force that is beyond human control." The Centre for Research on the Epidemiology of Disasters' International Disaster Database (EM-DAT)[16] served as the primary non-VR source of fatal discontinuities due to exposure to forces of nature (ie, natural disasters, lightning, earthquakes, volcanic eruptions, avalanches, storms, and floods). Data from EM-DAT were last accessed June 20, 2019. Supplemental online research was conducted for events where EM-DAT and VR were not up-to-date.

Partial discontinuity (CODEm)

For causes modelled in CODEm with fatal discontinuities hiding in the time trend, a process was established to avoid duplication of fatal discontinuity deaths in CODEm and the fatal discontinuity estimates. First, location-cause-years were identified through outside non-VR sources. If these location-cause-years also had VR death estimates that were greater than the average of the immediate surrounding years, the difference between the identified year and the average of the surrounding years was included in the relevant cause for the fatal discontinuities database. The extracted deaths for all fatal discontinuity causes from VR are then subtracted from the all-cause VR data used in the all-cause mortality estimation process.

Executions and policy conflict

In GBD 2019, executions and police conflict is defined as "the lawful use or threatened use of force or violence against individuals or groups of people or property in an attempt to achieve political or socioeconomic objectives for a state." Data for executions and police conflict came primarily from Amnesty International, but other sources such as UCDP, ACLED, and VR that reported deaths due to legal intervention were also cause-mapped to executions and police conflict.

Homicide

In GBD 2019, homicide is defined as "the use of violence against an individual or group of people in an attempt to achieve non-political, religious, or ideological objectives." Data for homicide came from VR, IISS, GED, ACLED, and other supplements. Events were mapped to homicide where the notes found in the raw data indicated gang violence. Deaths from IISS, GED, and ACLED were then split among three homicide sub-types; physical violence by firearms, physical violence by sharp object, and physical violence by other means, based on the rates calculated from VR by country and territory if available, and by region if country VR was unavailable.

Protein-energy malnutrition (PEM)

In GBD 2019, protein-energy malnutrition is defined as "a lack of dietary protein and/or energy" and covers famines as well as severe droughts. The primary source for PEM data, other than VR, was EM-DAT. The Tombstone report was used to estimate deaths attributed to the famine during the Great Leap Forward in China in the 1960s.[17]

Other injury causes

Other injury causes include other transport injuries (ie, plane, train, and boat accidents); poisonings; fire, heat, and hot substances; and other exposure to mechanical forces (eg, building collapse). The primary data source for these events other than VR was EM-DAT. Supplemental online research was conducted for events where EM-DAT and VR were not up to date.

Meningococcal meningitis and other diseases

In GBD 2019, fatal discontinuities due to a subset of infectious diseases were estimated, including meningococcal meningitis (or meningococcal infection), diarrhoeal disease caused by cholera, dengue, and malaria. These infectious diseases were first included on the fatal discontinuity cause list for GBD 2016 because (1) their current modelling strategies in the Cause of Death Ensemble model (CODEm) does not optimally capture the potentially highly variable—or epidemic—mortality levels and trends characteristic of these causes; and (2) they can contribute to significant total fatalities in a given location-year. Other infectious diseases for which the latter is true—high death rates in the presence of an outbreak or epidemic—are currently modelled with alternative cause of death methods (eg, natural history models for measles and yellow fever), which allow for greater variation year-over-year if or when outbreaks occur.

The Global Infectious Diseases and Epidemiology Network (GIDEON) and EM-DAT served as the primary data sources for collating cholera and meningococcal meningitis or meningococcal infection death reports.[18,19] For any year that cholera or meningococcal meningitis deaths were recorded in a country or territory covered by GBD, reported deaths were directly extracted from 1950 to 2019. If GIDEON or EMDAT had reporting gaps in cholera or meningococcal meningitis deaths, and WHO reports had coverage for those years, the WHO reports were used. For the Yemen Cholera outbreak in 2016 and 2017, estimates from local collaborators were used in the absence of other data sources.

Section 4.5.2: **Location mapping**

Every event in the fatal discontinuities database was mapped to a GBD location using a four-step process that includes the following steps in succession: (1) manual mapping, (2) string matching, (3) GPS overlay, and (4) geocoding. If an event was manually mapped, the location was assigned without the use of any other map types. In manual mapping, events are manually assigned to locations by matching the location provided in the raw data to a GBD location. During string matching, an event's location strings were directly compared to the GBD ASCII location names. During GPS overlay, events that have GPS coordinates provided were overlaid onto a map of GBD locations. If the event was placed over a GBD most-detailed location, the event was assigned to that location. During geocoding, the event's location string was entered into Open Street Maps, which returns GPS coordinates. These coordinates were processed using GPS overlay to return GBD locations. This hierarchy provides results where the results of manual mappings are considered the most reliable, followed successively by string matching, GPS coordinates, and then geocoding.

Section 4.5.3: **Side splitting**

Many fatal discontinuities, such as war, have deaths that are reported across multiple locations. In these instances, deaths were split between the population from both locations, unless estimates by side were provided. If the resulting locations were at the most detailed GBD location level, no further splitting was needed. If a location was not most detailed, the deaths were distributed among the child locations by population.

Section 4.5.4: **Prioritisation**

Where multiple sources reported shock deaths for the same location-year-cause, a cause-specific prioritisation scheme was followed that reflected the available detail in the cause-specific datasets. For example, the Georeferenced Event Dataset from UCDP was prioritised above all other non-VR sources because it included detail on how deaths were distributed between multiple actors and locations in each conflict event. In most cases, VR from 4-star or 5-star locations was used where available. In some cases, VR from 4-star or 5-star locations was not chosen if there were well-known data quality issues or discrepancies in the cause of death data reporting related to a particular event (eg, supplemental death data for Louisiana were used for Hurricane Katrina because of established data reporting issues).

Section 4.5.5: **Age-sex splitting**

We ran all compiled data through the causes of death age-sex splitting process, except in cases where we had substantial and reliable information about the age distribution of specific events with high mortality, such as United States mortality during the Vietnam war and Iran mortality during the Iran-Iraq conflict in the early 1980s.[10]

Section 4.5.6: **Assigning uncertainty and generating draws**

We generated UIs for deaths caused by conflict and terrorism using UCDP high and low mortality estimates, except for deaths in Iraq from 2003 to 2016. During this time period, deaths due to conflict and terrorism were estimated using a combination of supplemental sources. We used death counts from the Iraq Body Count (IBC),[20] which had the lowest number of deaths from among the sources we found, as the lower bound of the UI from 2003 to 2016. We used estimates from the Iraq Mortality Study (IMS) by Hagopian and colleagues[21] from 2003 to 2006, the deadliest years of the war, to scale deaths to produce the upper UI limits using the formula below:

$$deaths_{GBD\ 2017,\ high} = deaths_{IBC} \cdot \overline{\left[\frac{deaths_{IMS}}{deaths_{IBC}}\right]}_{2003-2006}$$

GBD 2019 used the average ratio between IMS and IBC reported deaths between 2003 and 2006, multiplied by the number of deaths reported by the IBC. This high estimate was carried forward through 2017 under the assumption that IBC similarly undercounts the number of deaths due to the ongoing civil war in Iraq. The final, best estimate for conflict and terrorism deaths in Iraq from 2003 to 2016 is the midpoint of the high and low estimates given above.

When high and low estimates were not included in the existing data, we applied the regional average UI to the available mortality estimate, for all fatal discontinuity causes.

We assumed a log-normal distribution using mean mortality rates and standard error based on high and low estimates. When the standard error was less than 10e-8, we set the draws equal to the mean rate. We then sampled 1000 draws from the log-normal distribution and converted the draws back to counts space. We used the count space draws to calculate final means and UIs.

Section 5: **Cause-specific mortality**

*Cause-specific mortality for both sexes, for all age-groups, locations, and years, were estimated as published in the core GBD 2019 papers. The following text is reproduced from the appendix of the **"Global burden of 369 diseases and injuries, 1990– 2019: a systematic analysis for the Global Burden of Disease Study 2019"** manuscript. This text is reproduced here in its entirety so not all sections may be specifically applicable to the current analysis of neonatal and under-5 mortality.*

Section 5.1: GBD 2019 Causes of Death database

Section 5.1.1: **Background**

All available data on causes of death (CoD) data are standardized and pooled into a single database used to generate cause-specific mortality estimates by age, sex, year, and geography.

Section 5.1.2: **CoD data identification**

Overview of data types

The CoD database contains seven types of data sources: vital registration (VR), verbal autopsy (VA), cancer registry, police records, sibling history, surveillance, survey/census, and minimally invasive tissue sample (MITS) diagnoses. In countries with complete VR systems, there is no need to use any other data source. Less than half the world's population has deaths captured in a VR system, therefore, for countries with incomplete VR systems, vital statistics for causes of death may be supplemented with other data types.

ICD-detail

A majority of the CoD data is VR data obtained from the World Health Organization (WHO) Mortality Database, a compilation of data submitted to the WHO by individual countries. VR is also obtained from country-specific mortality databases operated by official offices. Each cause is coded directly to the most detailed CoD when possible, whereas cause codes in data tabulated by International Classification of Disease (ICD-) are coded to aggregated cause groups. The CoD database contains 2,525 country-years of detailed data from 1980 to 2018, which includes underlying CoD coded with 3-5 digit codes, by country, year, sex, and age groups. Detailed causes are coded to one of the following ICD-detail coding systems: ICD-8, ICD-9, or ICD-10. Each coding system has a similar cause hierarchy and cause list that has continually developed over time. ICD-10 is the current standard and the most exhaustive cause list. Within the cause lists, 5-digit codes are truncated to 4-digit codes to condense the lists. Updates to ICD-detail occur biannually as WHO releases new versions or as country collaborators provide additional data. Updates to data from WHO increasingly include ICD-10 CoD data as it is the most current classification of CoD, while updates to ICD-8 and ICD-9 detailed lists are less common. In the case

of overlapping data, preference is given to data from pre-determined country collaborations, which are updated annually.

ICD-tabulations list

The ICD-tabulation lists include the ICD-8 List A (ICD-8A), ICD-9 Basic Tabulation List (BTL), ICD-10 Mortality Tabulation, Russia Tabulation, and India Medical Certification of Cause of Death (MCCD). These data sources make up 1,096 country-years from 1980 to 2016 in the CoD database. All are condensed versions of the ICD-8, ICD-9 and ICD-10 detail lists with some differences in the format of cause lists depending on the data source. ICD-8A, ICD-9 BTL, and ICD-10 Mortality Tabulation CoD are assigned to subtotal groups (referred to as chapters) and cause groups respective to ICD-detail groups. Additionally, ICD-9 BTL includes ICD-9 detail codes for some cancers and a custom tabulation scheme for the former Union of Soviet Socialist Republics (USSR) countries. The Russia Tabulation lists and India MCCD cause lists each have custom nomenclatures based on ICD-detail cause codes.

Two of the drawbacks in using tabulation lists are discrepancies in the accuracy of death counts and lack of detail due to aggregated cause groups. There are instances where the sum of deaths in chapter subtotals are not equal to the sum of cause groups within the chapter. To account for any missing or duplicate deaths reported within the cause groupings, death counts are systematically adjusted by calculating the differences between subtotals and sub-causes within the cause groups. Any differences are assigned to a remainder cause group. To account for the lack of cause code detail, select cause groups are disaggregated (Step 1.1) to create a complete cause list. Updates to ICD-Tabulation lists obtained from WHO occur less frequently compared to ICD-detailed lists as more countries are reporting deaths in ICD-detail. In instances of overlapping data, preference is given first to detailed collaborator data, followed by detailed WHO data, then tabulated collaborator data, and finally tabulated WHO data.

China Disease Surveillance Points /China Center for Disease Control and Prevention

The two primary sources of data for China are surveillance data from the China Disease Surveillance Points (DSP) system and VR data collected by the Chinese Center for Disease Control and Prevention (CDC). In the China DSP data, deaths were reported across 145 disease surveillance points used from 1991 to 2003, 161 disease surveillance points from 2004 to 2012, and 605 disease surveillance points from 2013 to 2017. While China DSP with ICD-10 coding is considered sample VR data, it provides national coverage and cause detail. Thus, it receives similar processing and treatment to the China CDC VR from 2008 to 2016. From 2008 to 2017, all of the deaths and CoD information from the DSP system and other system points throughout China were collected and reported via the Mortality Registration and Reporting System, an online reporting system of the Chinese CDC. The deaths in these data are reported at the strata level, a metric that is specific to China. Counties are stratified by urban and rural classification, but definitions of urbanity vary across counties. In Step 7, we use a method developed to scale up deaths from strata level to the province level.

Sample registration system

Sample registration systems are expanding in several countries, and are key sources of data in Indonesia and India. The Sample Registration System (SRS) is a dual-record system wherein a

resident part-time enumerator continuously records births and deaths in each household within the sample unit every month. A full-time SRS supervisor thereafter independently collects the vital events along with other related details for each of the preceding six month periods during the calendar year.

India Medical Certification of Cause of Death

The India MCCD has data for the urban parts of the majority of the states and union territories beginning in 1980. Deaths reported in this data source have been medically certified and are considered VR data. The CoD are reported in a tabulation list with a unique numbering scheme that conforms to ICD-9 and ICD-10 detail codes, which must be disaggregated. MCCD is state-split to fill in data gaps (Step 1.2 State Splitting) prior to age-sex splitting. Because SRS is widely considered a more credible assessment of CoD in India, we chose to use MCCD data only in certain cases for modelling with CODEm. We preserved MCCD data in the database for two primary reasons. First, where the three midpoint years of SRS data resulted in the loss of a clear time trend, as was the case for maternal mortality, we chose to preserve MCCD in addition to SRS. Second, MCCD has an advantage over SRS in cases where VA is not a valid instrument for ascertaining CoD, like encephalitis, dengue fever, and peptic ulcer disease. In these cases, we kept MCCD over SRS.

Verbal autopsy

Verbal autopsy coded to ICD-10 and other lists

In countries without VR systems, VA studies are a viable data source to inform CoD. Data are obtained by trained interviewers who use a standardised questionnaire to ask relatives about the signs, symptoms, and demographic characteristics of recently deceased family members. CoD is assigned based on the answers to the questionnaires.

VA data are highly heterogeneous: studies use different instruments, different cause lists (from single causes to full ICD-cause lists), different methods for assigning CoD, different recall periods, and different age groups. Cultural differences may also affect the interpretation of specific questions. CoD validity must be considered when mapping to a GBD cause. VAs are likely accurate in assigning CoD to road injury or homicide but less accurate for causes requiring medical certification, such as cardiovascular causes. Studies may also occur as stand-alone assessments or as part of an extended network, such as The International Network for the Demographic Evaluation of Populations and their Health (INDEPTH) Network[22]— a continuous surveillance source with several Demographic Surveillance Systems sites that collect data coded to ICD-detail causes.

InterVA-modelled verbal autopsy

InterVA (**Inter**preting **V**erbal **A**utopsy),[23] a set of computer models intended to facilitate interpreting VAs, was found to be non-credible by the Population Health Metrics Research Consortium (PHMRC). As a result, INTERVA-modelled VAs are typically excluded from our analysis because of low validations, except for injuries and maternal causes, used to fill gaps and stabilise patterns.

Other data types

Maternal mortality data

In locations with low-quality, or no VR, maternal mortality metrics can be found in surveillance, surveys, census, and sibling history data sources. The best data have death counts due to maternal causes and the total number of deaths for women within the reproductive ages of 10 to 54 by year. If a data source is missing these components, creating a complete cause list is necessary by using live births and all-cause mortality deaths.[1] Though death counts are the preferred metric, maternal mortality is often measured by using the maternal mortality ratio (MMR), which is easily converted to deaths by using live births. The China Maternal and Child Surveillance data is adjusted by scaling data from the strata to the province level (Step 7).

Surveys and censuses reporting fraction of deaths due to selected injuries

Surveys and censuses are often used in countries with less developed VR systems; in countries with adequate VR, surveys and censuses are supplementary. Much like VAs, the CoD validity is a concern because of lack of medical certification at the time of death. For these data sources, we keep only causes related to maternal mortality and injuries. The remaining causes are accounted for as a remainder of total deaths in the sample size.

Police records

In most countries, police and crime reports are an important source of information for some types of injury deaths, notably road injuries and interpersonal violence. Our police data come from reports on road traffic and crime trends. The police reports used in this analysis were obtained from published studies, national agencies, and institutional surveys such as the United Nations (UN) Crime Trends survey and the UN Office on Drugs and Crime Global Study on Homicides. We assessed whether police reports were likely to be complete and to cover the entire country by comparing police trends with those seen in VR. Data are excluded in instances where police data for road traffic injuries are significantly lower than the VR. Police data that meet our inclusion criteria and provide complete coverage are uploaded to the database for use in road injuries and interpersonal violence deaths estimation.

Population-based cancer registries

Cancer registries with incidence

Data on cancer incidence were sought from individual population-based cancer registries as well as from databases that include multiple registries, including Cancer Incidence in Five Continents, NORDCAN, and EUREG. Cancer registries were identified through the membership list of the International Association of Cancer Registries, through the GBD collaborator network, through publications, or through the GHDx. Registries were excluded if they were not representative of the coverage population, if the data were limited to years prior to 1980, if the source did not provide details on the population covered, or if the list of cancer types included was not comprehensive for the age group covered. Beginning in GBD 2019 childhood cancer-specific population-based cancer registry data was sought and included.

Cancer registries with incidence and high-quality mortality data

In addition to incidence, some high-quality cancer registries also report cancer mortality data. These data were also extracted and used as inputs to mortality-to-incidence modeling.

Section 5.1.3: **Standardise input data (step 1)**

The input data to the CoD database are received in various formats and must be standardised to run through central CoD machinery to then upload to the database. Raw data inputs come from data sources such as mortality databases, literature reviews, or reports. Usable data sources must have a clear sample size of the number of deaths in the population and exhaustive cause lists. The complexity of the data cleaning process varies drastically across data sources. For VR microdata with the location, age, sex, year, and ICD--coded cause of every death, very little effort is necessary to standardise it into a consistent structure. Other sources may require weeks of careful review to accurately extract scans of hardcover CoD reports into spreadsheets that can be transformed and standardised.

At this point, data are assigned source identifiers so that they can be linked to the GHDx and cited appropriately. Any aggregate age and sex categories are flagged for age-sex splitting. The methods of cause-of-death assignment and data collection are reviewed to determine which source type to assign; for example, we distinguish sibling history data from surveys with a VA module. Only data at the most detailed level of the GBD location hierarchy are used. Documentation from the source is reviewed to determine if the population is representative of the location or only a subset of the population in that location. Data sources representing a subset of the population are flagged as non-representative; this flag is used by Cause of Death Ensemble modelling (CODEm) to increase the variance associated with such data points.

Finally, diagnostics are reviewed at this stage to avoid sending cleaning errors downstream. We review cause-specific deaths for each demographic group to ensure the data are reasonable. For example, it is unlikely that male breast cancer deaths are higher than female breast cancer deaths or deaths from neonatal causes occur in age groups over one year. All death totals are compared with the sum of cause-specific deaths to ensure the observed deaths are accounted for and sample size is complete.

Disaggregation (step 1.1)

CoD in tabulated VR data are condensed into aggregated groups, some of which can be mapped directly to GBD causes, while other aggregated cause groups are not informative and cannot be mapped to them. To correct for this, aggregated causes were mapped and split onto multiple ICD-8, ICD-9, and ICD-10 detail causes, or targets, based on the ICD-groupings within the aggregated causes. ICD-8, ICD-9, and ICD-10 detail codes serve as targets because they are the highest-quality VR data and enable the calculation of proportions used to split the aggregated cause data into detailed causes. The proportions of deaths from nearby countries within the super-region were used to fill in data gaps as they were likely to have similar CoD trends.

We determined the targets based on detail causes missing from the tabulated cause list. For example, in ICD-9 BTL, the tabulated cause list includes a viral diseases group. In the hierarchy of causes, this group is comprised of “measles”, “yellow fever”, “encephalitis”, “hepatitis”, “rabies”,

“other infectious diseases”, “garbage code”, and “remainder of viral diseases”. We did not consider this list to be an exhaustive list of viral diseases based on the range of ICD-detail codes given in the ICD-9 BTL documentation. To make the cause list exhaustive and inclusive of other viral diseases, we split the remainder of the viral diseases group into “other meningitis”, “other infectious diseases”, “herpes”, “dengue”, “other neglected tropical diseases”, and “garbage code”. After a list of targets was determined, the aggregated deaths were disaggregated to the target causes by using ICD-8, ICD-9, and ICD-10 detail proportions generated at the super-region level for the corresponding sex and age groups across all years in the time series. For example, in ICD-9 detail data, 54.8% of deaths in males in Latin America and the Caribbean within the target group for the BTL “remainder of viral diseases” group were designated to “other meningitis.” Thus, 54.8% of deaths in the tabulated group “remainder of viral diseases” were assigned to “other meningitis” for any country within that particular super-region. For any cause and demographic group for which we lacked ICD-detail, global proportions were used.

State splitting (step 1.2)

Two sources for CoD estimation in India are the MCCD report, which reports medically certified deaths from health facilities in mostly urban areas,[24] and the SRS, which collects information via VA about one-half of 1% of the total population in India, including both urban and rural areas, from 8853 sampling units as of 2014.[25] For MCCD, missing data impedes estimation of trends at the state level. We used a first-order, log-linear model of the four-way contingency table of deaths by sex, age, state, and year to estimate the missing state-years. We fit the model to all available data for MCCD separately for each cause, including state-specific all-age measurements and age-specific national measurements. From this, we produced estimates for each combination of sex, age, state, and year. We then used these estimates wherever the raw data did not include sex-, age-, and state-specific death counts.

For MCCD, the model was fit separately for ICD-10- and ICD-9-based reports by using the tabulated cause list present in the data.

Calculate non-maternal deaths (step 1.3)

In cases when maternal mortality metrics do not include both deaths due to maternal causes and deaths due to non-maternal causes for women of reproductive age, live births and all-cause mortality estimates can be used to calculate deaths. Many studies report maternal deaths as the MMR. MMR is the number of maternal deaths per 100,000 live births and can be used to calculate deaths when it has been derived from primary data and not estimated. Maternal deaths were calculated by using MMR and live births; if live births were missing we substituted live birth estimates and used the following equation:

$$Maternal\ deaths\ = \frac{MMR}{100{,}000} \times Live\ births$$

If a study was non-representative, we extracted sample size and live births from that study. After maternal deaths were calculated, we used the difference from all-cause mortality estimates to determine non-maternal deaths.

A more accurate and data-inclusive method of calculating maternal and non-maternal deaths incorporates coverage and splits deaths for a range of years into individual years. If there were live births in the study, we adjusted the coverage.

$$Coverage = \frac{Live\ births}{GBD\ estimated\ live\ births}$$

After coverage was calculated, totals deaths were scaled to be more representative. This gives a more accurate death count since the envelope assumes representative coverage. We then calculated non-maternal deaths by using all-cause mortality as an all-cause total.

$$Maternal\ envelope\ with\ coverage\ = Maternal\ envelope \times Coverage$$

An additional adjustment can be applied to maternal data spanning over a range of consecutive years, which allows for more data inclusion. The years within specified year ranges are separated into individual years, and total deaths within the year range were split between each individual year by using the fixed proportions of maternal deaths from VR in that particular country. We used only VR data to inform the proportions because it was both high-quality and representative.

Section 5.1.4: **Map to GBD cause list (step 2)**

In GBD 2019, we used 439 maps to translate causes found in the input data to the GBD 2019 cause list. This included 31 maps for VR data, 314 for VA data sources, and 98 for other data types. The largest, and most universal, maps used were those for ICD-9 and ICD-10 VR data. The input data causes varied from 3-4 digit ICD-codes to custom cause lists with cause names such as "cholera" or "hepatitis". Our mapping process enabled us to compare these various data sources across demographic groups.

In GBD 2019, we developed additional maps to translate ICD-codes found in the input data that are non-underlying causes to appropriate target codes based on the levels of the GBD cause list. These garbage codes were mapped to Levels 1-4 of the GBD cause list according to the following criteria:

1. **Level 1** includes all garbage codes for which a Level 1 GBD cause cannot be directly assigned. For example, the underlying causes of "sepsis" or "peritonitis", if not specified in the data, could be an injury, a non-communicable disease, or a type of communicable disease. In these cases, deaths will be redistributed across all three of these Level 1 causes. In addition, deaths coded to impossible or ill-defined causes of death (including "senility" and "unspecified causes") fall into this category, as they will be redistributed onto all causes.

2. **Level 2** includes all garbage codes that can be assigned to Level 1 causes in the GBD cause list. This would include deaths coded to "unspecified injuries" (X59), which are redistributed onto all injuries.

3. **Level 3** includes all garbage codes for which we know the Level 2 CoD and can redistribute onto Level 3 causes. This includes deaths coded to causes such as "unspecified cardiovascular disease", which falls within the Level 2 cause "cardiovascular diseases", as well as those coded to "unspecified cancer site", which falls within the Level 2 cause "neoplasms".

4. **Level 4** includes all garbage codes for underlying causes of death that can be redistributed within a Level 3 cause. This includes garbage codes such as "unspecified stroke" or "unspecified road injuries."

Section 5.1.5: **Age-sex splitting (step 3)**

Standardizing all inputs to have age and sex detail matching GBD demographics

Different sources, particularly VA studies, report deaths for a wide range of age groups with varying intervals. For the analysis of CoD, we mapped these different age intervals to the GBD standard set of age groups. The approach to undertake this mapping was the same as in the prior GBD studies (GBD 2017, GBD 2016, GBD 2015, GBD 2013, and GBD 2010).

In the process of assembling a consolidated demographic database, we found that the aggregation of age groups is perhaps the strongest source of inconsistency. By convention, such data are reported in broad age groupings such as "0-4", "5-14", and "15-49," or with both sexes together. The issue of comparability between age-sex groups arose when assembling the GBD CoD database. We developed a tool called age-sex splitting that takes aggregated age groupings and the "both sexes combined" grouping and divides them into what their constituent age groups would likely have been if respective cause-specific and country-specific age distributions had been used. The analytical framework for GBD includes three infant age categories: early neonatal (0-6 days), late neonatal (7-27 days), and post-neonatal (28-364 days), and 20 non-infant age categories: 1-4 years, 5-9 years, and so forth proceeding in five-year age groups until the terminal age group of 95+. We treat unknown ages and sexes in the same manner we treated the "all ages combined" age category and "both sexes combined" sex group. Through this process, we were able to directly compare all data sources on even terms.

The approach to age splitting is based on the following formula. The key assumption underlying this formula is that the relative risk of death by age group compared to a reference age group is invariant across populations. Although this assumption is likely violated in specific cases, a strong biologically based pattern of the relative risk of death for a cause by age is observed for most causes. The basic formula is as follows:

$$D_a = R_a N_a \left(\frac{D_a^{a+x}}{\sum_a^{a+x}(R_a N_a)}\right)$$

Where:

D_a= the number of deaths from a cause in age group *a*

R_a= global cause-specific mortality rate of age group *a*

N_a= the country-year-sex-specific population in age group *a*

D_a^{a+x}= the number of deaths in the age group *a* to *a+x*

With the assumption of invariant relative risks of death by age with respect to a reference age group, this equation can be used, along with population distribution by age, to split an aggregate number of deaths for the age groups *a* to *a+x* into specific deaths for each age group within the aggregate interval.

$$D_{as} = R_{as}N_{as}\left(\frac{D_{as}^{a+x,s}}{\sum_{a}^{a+x}(R_{as}N_{as})}\right)$$

Where:

D_{as}= the number of deaths from a cause in age group *a,* sex *s*

R_{as}= global cause-specific mortality rate of age group *a,* sex *s*

N_{as}= the country-year-sex-specific population in age group *a* for sex *s*

$D_{a,s}^{a+x,s}$= the number of deaths in the age group *a* to *a+x* for sex *s*

In some cases, deaths are reported for an aggregate age group for both sexes combined. The task in this case is more complicated, but the same principle can be applied. In this case we assumed that the relative risks of death by age and sex are constant.

This equation can be used to split data aggregated by age and sex. The assumption, however, of invariant relative risks across age and sex is a stronger assumption. Fortunately, data pooled across sexes are less common in the published or unpublished CoD data.

The relative risk of death in a particular age group for a given sex is derived from the global distribution of cause-specific mortality rates found in available VR data. Location-years from the following code systems are used, provided they report the requisite age- and sex-detail: ICD-7, ICD-8, ICD-9 BTL, ICD-10 tabulated, ICD-9, and ICD-10. Upon compiling these data, we mapped them to GBD causes and aggregated up to cause Level 3. This is the level at which a particular cause is split – that is, any child cause of a Level 3 parent is split by using the age distribution of that parent (so, chronic kidney disease due to diabetes would be split by using the age pattern of chronic kidney disease).

We next adjusted separately for estimated adult and child VR completeness. Location-year-age-sex-cause specific deaths and population were then aggregated across all location-years, to produce cause-specific mortality rates by age and sex. These were used to determine the risk of death at any age relative to any reference age group, as shown in the above equations.

Correct age-sex violations

Occasionally, data sources include deaths by a cause for which medical consensus exists that death is impossible for the sex and age. For example, some number of deaths may be attributed to cervical cancer in males, or to maternal causes in children younger than 10 years. We have constructed a conservative list of age-sex restrictions. When deaths violate these restrictions, we redistribute them proportionally onto all causes.

Section 5.1.6: **Correction for miscoding of Alzheimer's and other dementias, Parkinson's disease, and atrial fibrillation and flutter (step 4)**

Objective

For certain CoD, mortality rates reported in VR systems are impossible to reconcile with observed trends in disease prevalence and excess mortality. For dementia, Parkinson's disease, and atrial fibrillation and flutter, these disparities can largely be attributed to death certification practices. We sought to address the known bias in CoD data by first identifying the proportion

of all deaths that should be assigned to these causes and next determining the GBD causes and garbage groups to which these deaths are being incorrectly assigned.

In past GBD iterations, we estimated Alzheimer's disease and other dementias, Parkinson's disease, and atrial fibrillation and flutter on the basis of longitudinal prevalence and excess-mortality data to help account for changing patterns in death certification and corresponding implausible time trends in many VR sources. This method was first implemented for Alzheimer's disease and other dementias in GBD 2013. We added atrial fibrillation and flutter to the causes modelled in GBD 2015 and Parkinson's disease to the causes modelled in GBD 2016 by using this strategy. All of these causes were processed in CoDCorrect in a manner that was agnostic to the likely targets of misclassification, which inappropriately led to changes in mortality estimates for causes unrelated to these three in GBD 2015. For GBD 2016, we improved this process by completing a literature review to identify the causes of death most closely associated with Parkinson's and Alzheimer's diseases[26-29] and limiting the CoDCorrect adjustments to include only those causes. For GBD 2017, we refined this approach further by using multiple CoD data to determine the GBD causes and garbage codes from which we move deaths as well as the pattern of misclassification.

Correction process

For Parkinson's disease and atrial fibrillation and flutter, we first estimated excess mortality from prevalence and CoD data in countries with the highest ratio of cause-specific mortality to prevalence, which represents the greatest willingness to code to an under-coded cause. Then, using DisMod-MR 2.1 (named from disease model Bayesian meta-regression), we derived estimates of cause-specific mortality rates from available prevalence surveys as well as the estimates of excess mortality rate, applied across all countries and over time. We divide this value by the all-cause mortality rate to determine the fraction of overall mortality to attribute to each under-coded cause. For dementia, the modeling process was redesigned in 2019 to no longer depend on vital registration data from the highest dementia mortality locations. Instead, we used relative risk data from cohort studies to calculate total number of excess deaths due to dementia, and end-stage disease proportions from linked hospital to death records to subset these deaths to the proportion of excess deaths with end-stage conditions, which we attributed to dementia. Finally, we used log-linear interpolation to interpolate final estimates of death due to dementia for the entire time series and saved as a custom CoD model.

To ascertain the causes from which we would move deaths to under-coded causes, we leveraged multiple CoD data from the United States – by looking to the combinations of intermediate and immediate causes (i.e., chain causes) present on death certificates with an under-coded cause listed as underlying, and identifying other causes with similar or identical chain causes, we can determine the expected pattern of miscoded deaths.

The first stage in this process is to parse out years we believe coding practices in the United States to be relatively stable. For dementia, this "gold standard" dataset features 2010-2015, for Parkinson's 2005-2015, and for atrial fibrillation and flutter 2014-2015. We then collect all deaths in those years with the under-coded cause listed as underlying and remove any mention of the under-coded cause from the death certificate. Next, for each unique chain, we search the

entire time series of data (1980-2015) to identify the distribution of underlying causes that share that chain. The premise here is that if the diagnosis of dementia, Parkinson's, or atrial fibrillation and flutter were missed, the other causes listed on the death certificate would have been the basis for certification. We then reallocate the under-coded deaths by chain based on that alternative underlying cause distribution.

Upon iterating through all unique chains, we are left with a dataset excluding under-coded causes of death, each remaining cause able to be subdivided into correctly coded deaths and deaths that have been recoded from an under-coded cause by the process described (although not all causes are necessarily targeted by the recoding algorithm). The quantity of interest is the ratio of miscoded deaths to total deaths by cause, age, and sex in our counterfactual dataset.

We apply the ratios derived from the multiple cause data to all VR data to determine the local pattern of miscoding. In this way, the method is sensitive to the observed epidemiology of a given place and time. Then, we calculate the deficit in under-coded cause mortality for each location, year, age, and sex by taking the difference in the expected cause fraction based on prevalence and excess mortality compared to the proportion of deaths actually certified by the VR system. Finally, we scale the cause-specific miscoded deaths to match the deficit and then move them accordingly. We assumed that misclassification of actual dementia and Parkinson's deaths in past years occurred only for reported causes of death that might have plausibly been the direct result of dementia or resulted from misdiagnosis of other organic brain diseases based on clinical expert judgement. A similar assumption is used for atrial fibrillation and flutter, for which only cardiovascular causes and ill-defined garbage codes are considered.

Because the deaths being reallocated vary by location-year, we need a mechanism to ensure plausible limits to how many deaths are extracted from each GBD cause and garbage code. To achieve this, we first run the above-mentioned algorithm on all 5-star VR data. Then, we determine the 95th percentile of the proportion of deaths moved for each GBD cause and garbage code group by age and sex across location-years among these data. Those values are subsequently stored and applied as the limits for deaths moved by this process.

Section 5.1.7: **Redistribute (Step 5)**

A crucial aspect of enhancing the comparability of data for CoD is to deal with uninformative, so-called garbage codes. Garbage codes to which deaths were assigned should not be considered as the underlying CoD--for example: "heart failure", "ill-defined cancer site", "senility", "ill-defined external causes of injuries", and "septicaemia". The methods for redistributing these garbage-coded deaths were outlined in detail in Naghavi et al,[30] and the underlying algorithm for redistributing deaths assigned to these codes has not changed since GBD 2013.

Redistribute HIV-related garbage codes (step 5.1)

Because of the disparate nature of HIV/AIDS mortality across space and time, dynamic redistribution of HIV/AIDS-related garbage codes was needed. To inform this redistribution, we generated target proportions for each garbage group by age band (under 1 month, 1-59 months, 5-19 years, 20-49 years, 50-59 years, 60-69 years, 70-79 years, and 80+ years), five-year time interval, and sex. The garbage groups either target HIV or a remainder target. The allotment of deaths to either of these is based on the regional increase in the mortality rate of all codes in the

group relative to the rates seen in 1980–1984 – an increase greater than 5% is assumed to be HIV/AIDS-related, and the proportion of those deaths exceeding 5% are redistributed to HIV/AIDS. Any increase less than or equal to 5% is then assigned to the remainder target.

Regress garbage codes versus non-garbage codes (step 5.2)

For each redistribution package, we defined the "universe" of data as all deaths coded to either the package's garbage codes or the package's redistribution targets for each country, year, age, and sex. We then ran a regression based on the following equation separately for each target group and sex:

$$TG_{crt} = \alpha + \beta_1 Gar_{crt} + \beta_2 Age_{crt} Gar_{crt} + \theta_r Gar_{crt} + \gamma_r + \varepsilon_{ct}$$

Where:

TG_{crt} = percentage of deaths within the given garbage code's universe that were coded to a given target group, by country

Gar_{crt} = percentage of deaths within the given garbage code's universe that were coded to a given set of garbage codes

Define Age(CRT)

α = constant

β_1 = slope coefficient describing the association between Gar_{crt} and TG_{crt}

β_2 = slope coefficient describing the association between the interaction $Age_{crt}Gar_{crt}$ and G_{crt}

γ_r = region-specific random intercept (or super-region if the random effect on region is not significant)

θ_r = region-specific random slope (or super-region if the random effect on region is not significant)

ε_{ct} = standard error, normally distributed and calculated by bootstrapping

This regression was adjusted from GBD 2013 to include fixed effects on the interaction of garbage and age to ensure smooth age patterns. We made this decision after investigating diagnostic visualisations that showed unlikely gaps between proportions assigned to different age groups.

Once proportions were produced for each country, sex, age, and target group, certain adjustments were made to conform our packages to the best medical evidence available. In some cases, we implemented restrictions on the proportions that the regressions could yield. For example, we did not allow any redistribution onto "Chagas disease" outside of Latin America and the Caribbean or "suicide" under the age of 15 years. In other cases, we capped the proportion for some targets to the level that would be produced from proportional redistribution; for example, "haemoglobinopathy" and "haemolytic anaemia" were restricted to the level of proportional redistribution in the redistribution of "left heart failure". Occasionally, further adjustments were made on a case-by-case basis per country, age, sex, and target group

to suppress the impact of outliers based on existing epidemiological evidence and expert judgment.

In GBD 2019, we updated the regressions for stroke and diabetes. We dropped the proportion of garbage from the regression formula and ran regression on high-quality, low proportion garbage data (4/5 stars, < 50% GC). We also included all covariates included in the CODEm models for both stroke and diabetes.

Development of an algorithm for redistribution of garbage codes based on multiple CoD data

Multiple CoD data are a form of individual record causes of death data that include an underlying CoD along with other causes in the death chain, including intermediate and immediate causes. By analysing this type of data, we can sometimes find the true underlying CoD in other CoD data where the underlying cause is a garbage code or a misassigned CoD.

For GBD 2019, this method was expanded and used in redistribution of the following intermediate causes: sepsis, embolism (pulmonary and arterial), heart failure (left, right, and unspecified), acute kidney injury, hepatic failure, acute respiratory failure, pneumonitis, and unspecified central nervous system disorders. Using multiple CoD records for the United States, Mexico, Brazil, Taiwan, Italy, and Colombia we identified the fraction of deaths where the underlying cause of death and the intermediate cause was in the causal chain. Using a mixed effect linear regression, we estimated the fraction of intermediate-cause related deaths by underlying GBD cause. These fractions were multiplied by the GBD 2017 CoDCorrect result to calculate the number of deaths intermediate cause-related deaths for each GBD cause. Lastly, we calculated the "intermediate cause fraction", with total intermediate-cause related deaths as the denominator, by age, sex, location, year GBD cause. These fractions were used to redistribute the intermediate-cause-related deaths to a GBD cause.

To redistribute X59 and Y34 (unspecified injuries) deaths, we used a multi-step approach that utilized the pattern of N-codes in the causal chain in the multiple CoD data. First, we looked at deaths where X59, Y34, and GBD injuries causes were the underlying cause of death and got the pattern of N-codes in the chain. We then derived a cause-specific redistribution proportion based on the probability of a given pattern being coded to X59/Y34 or a GBD injuries cause and summing up these proportions for all patterns. An example below is given for X59:

$$P_{(pattern_j|UCoD\ X59)} = \frac{\#\ of\ pattern_j\ deaths\ |\ UCoD\ X59}{\sum_{j=0}^{m}(\#\ of\ pattern_j\ deaths\ |UCoD\ X59)}$$

$$P_{(GBD\ injuries\ cause_i|pattern_j)} = \frac{\#\ of\ UCoD\ GBD\ injuries\ cause_i\ deaths\ |\ pattern_j}{\sum_{i=0}^{n}(\#\ of\ UCoD\ GBD\ injuries\ cause_i\ deaths\ |pattern_j)}$$

$$redistribution\ proportion_{GBD\ injuries\ cause_i} = \sum_{j=0}^{m}\left(P\left(pattern_j\middle|UCoDX59\right) * P(GBD\ injuries\ cause_i|pattern_j)\right)$$

Where:

$pattern_j$ = a given N-code pattern in the chain of the multiple CoD data

UCoD X59 = a death with X59 coded as the underlying cause of death (UCoD)

UCoD GBD injuries $cause_i$ = a death with a GBD injuries causes coded as the UCoD

We applied these cause-specific redistribution proportions on the data where X59/Y34 were the underlying cause of death to get the number of X59/Y34 deaths "attributable" to each GBD injuries cause. Then, for each GBD injuries cause in the multiple CoD data, we calculated the fraction of redistributed X59/Y34 deaths over the fraction of total injuries death for that cause and modeled this intermediate cause fraction using a mixed effects linear regression similar to the one mentioned above. Like mentioned above, these fractions were then multiplied by GBD 2017 CoDCorrect results, and the cause fractions for X59 and Y34 were calculated by age, sex, location, year, and GBD injuries cause, and then used to redistribute X59 and Y34 deaths to GBD injuries causes.

Additionally, multiple cause of death data was used in the correction of the mis-assignment of deaths due to drug overdoses to unintentional other poisoning. More than 90% of these types of poisonings are due to exposure to narcotics, psychodysleptics, and other drugs, specified or unspecified. More than 97% of these poisonings by substance or drug occurred in ages 15–65 years. These are clearly not cases of accidental ingestion of substances but rather deliberate ingestion and unintentional poisoning. Using multiple CoD records for the United States, Mexico, Brazil, Taiwan, Italy, Colombia, Australia, and various European countries from 1980 to 2017, we selected all deaths with underlying causes coded to X40–X44. Table S4 shows the combination of other potential causes that can be found in the multiple CoD data for these underlying causes, and Table S5 shows the ICD 10 codes corresponding to these causes. On the basis of this table, we proportionally redistributed mis-assigned unintentional poisoning deaths to one of these causes. The main assumption behind this algorithm is the predominance of the fatality of some substances when a combination of drugs is considered. Given the combination of different drugs and substances in these codes, opium is the main cause of fatality.[31,32] Other substances, like cocaine, methamphetamine, and alcohol in combination with cannabis are less likely to be dominant in fatality.[33]

For example, if the multiple CoD data show that 40% of deaths include opioid use disorders as an intermediate cause where the underlying cause is X40–X44, the redistribution proportion for opioid use disorders will be exactly 40% due to the dominance of the fatality of opioid use disorders compared to other drugs in the above table. Additionally, in our final results, cannabis and psychoactive and psychedelic drug use disorder deaths were mapped to other drug use disorders.

Table S4. ICD 10 codes for substances or drugs used to assign deaths coded to an underlying cause of unintentional poisoning by using multiple CoD data

Accidental poisoning codes	All X40, X41, X42, X43, X44 codes
Opioid Codes	T40.0, T40.1, T40.2, T40.3, T40.4, T40.6, F11.0, F11.1, F11.2, F11.3, F11.4, F11.5, F11.6, F11.7, F11.8, F11.9
Amphetamine Codes	T43.6, F15.0, F15.1, F15.2, F15.3, F15.4, F15.5, F15.6, F15.7, F15.8, F15.9
Cocaine Codes	T40.5, F14.0, F14.1, F14.2, F14.3, F14.4, F14.5, F14.6, F14.7, F14.8, F14.9
Psychoactive and psychedelic drug	T40.8, T40.9, T43.6, F16.0, F16.1, F16.2, F16.3, F16.4, F16.5, F16.6, F16.7, F16.8, F16.9
Alcohol Codes	T51.0, F10.0, F10.1, F10.2, F10.3, F10.4, F10.5, F10.6, F10.7, F10.8, F10.9
Cannabis Codes	T40.7, F12.0, F12.1, F12.2, F12.3, F12.4, F12.5, F12.6, F12.7, F12.8, F12.9

Table S5. Algorithm for the selection and assignment of a substance or drug use CoD for deaths coded to an underlying cause of unintentional poisoning by using multiple CoD data

Selection Algorithm						
	Opioids	Cannabis	Cocaine	Amphetamines	Alcohol	Psychoactive and psychedelic drugs
Opioids	Opioids	Opioids	Opioids	Opioids	Opioids	Opioids
Cannabis	Opioids	Cannabis	Cocaine	Amphetamines	Alcohol	Psychoactive and psychedelic drugs
Cocaine	Opioids	Cocaine	Cocaine	Amphetamines + cocaine	Cocaine + alcohol	Cocaine
Amphetamines	Opioids	Amphetamines	Amphetamines + cocaine	Amphetamines	Amphetamines + alcohol	Amphetamines
Alcohol	Opioids	Alcohol	Cocaine + alcohol	Amphetamines + alcohol	Alcohol	Psychoactive and psychedelic drugs
Psychoactive and psychedelic drugs	Opioids	Psychoactive and psychedelic drugs	Cocaine	Amphetamines	Psychoactive and psychedelic drugs	Psychoactive and psychedelic drugs

Multiple CoD data were only available to us for the United States, Mexico, Brazil, Taiwan, Italy, Colombia, Australia, and various European countries. Because of this limited sample, we applied the result from the multiple CoD analysis from each country to its respective super region and used global proportions for Sub-Saharan Africa. We hope for increased availability of multiple CoD data in future analyses to achieve a more precise distribution for more locations.

Verbal autopsy anaemia adjustment (step 5.3)

To compensate for the over-representative cause fractions from anaemia found in VA studies, we redistributed these deaths based on the causal attribution of severe anaemia from the GBD 2015 study. The proportions were country-year-age-sex specific.

Calculate redistribution uncertainty (step 5.4)

We categorized garbage codes into four levels in order of increasing specificity. Some garbage codes are redistributed on all causes (e.g., unspecified causes of death) and others are only redistributed onto specific causes (e.g., unspecified cancer). Major garbage refers to garbage codes in Levels 1 or 2. Because of the variation in redistribution, estimating uncertainty from garbage redistribution for CODEm modeling was an important goal for GBD 2019.

We assigned redistribution variance to each data point in the CoD database by calculating residual variance from a regression predicting the percentage of garbage coded deaths

redistributed to a cause, given the proportion of garbage codes we observed for that location, year, age, sex, cause, and the age standardized relative rate of major garbage codes across all causes. If there is a cause that has greater residual variance, we assume greater redistribution uncertainty.

The two model inputs are the observed percentage of Levels 1, 2, and 3 garbage codes (by cause, age, sex, location, and year) in redistributed CoD data and the percentage of garbage codes in the raw data (calculated as the age standardized mortality rate ratio of major garbage coded deaths to all deaths in the raw data by location, year, and sex). Level 4 garbage codes were excluded from the model to avoid over estimating uncertainty in countries with high percentages of major garbage codes. Additionally, the classification of Level 4 garbage codes is not stable between successive GBD rounds – for example, "unspecified diabetes" was not a garbage code in GBD 2016, and in GBD 2017 was re-classified as a level 4 garbage code to permit estimation of diabetes by type. These deaths are still taken into account later in the uncertainty estimation process. The model predicts the percentage of garbage coded deaths redistributed to a cause, given the proportion of garbage codes we observed for that location, year, age, sex, cause, and the age standardized relative rate of major garbage codes across all causes. From this model we calculate residual variance. It is important to note that the variance here is a measurement of uncertainty of redistribution, not of the level of miscoding in the raw CoD data for a given demographic.

To calculate variance, a dataset was generated containing percent garbage by location, year, age, sex, and cause, where percent garbage is determined by the equation

$$pct_{garbage} = \frac{deaths_{redistributed} - deaths_{raw}}{deaths_{redistributed}}$$

A mixed-effect linear regression model was then fit to predict the logit percent of deaths from redistribution by age-standardized relative rate of major garbage codes.

$$logit\left(pct_{garbage_{ij}}\right) = \beta_0 + \beta_1 * \log\left(ASR_{majorgarbage_{ij}}\right) + \beta_2 * 15yearage_{ij} + \gamma_{1j} * \log\left(ASR_{majorgarbage_{ij}}\right) + u_j + e_{ij},\ \ \theta_{\{i\}} \sim N(0, \sigma^2)$$

Where:

i indexes dataset-location-year-age-sex-cause data points nested within *j* groups by GBD region

ASR major garbage: age standardized relative rate of major garbage

Residual variance, as estimated by the MAD, was calculated for each cause, sex, and age.

The next step was to use the residual variance to calculate uncertainty around each data point in the CoD database. First, we calculated the percent garbage of each data point by treating all deaths that could not be directly mapped to a GBD cause as garbage, including Level 4 garbage codes. Percent garbage was calculated as:

$$pct_{garbage} = \frac{deaths_{redistributed} - deaths_{corrected}}{deaths_{corrected}}$$

Where:

death corrected: deaths post misdiagnosis correction

deaths redistributed: deaths post redistribution

Residual variance was matched to each data point and 100 draws were sampled from a normal distribution by using the cause, age, sex, specific residual variance, and mean of 0. The logit transformed percent garbage was added to each value in the distribution. Each draw was then transformed out of logit space, and the post-redistribution deaths were calculated as

$$deaths = \frac{deaths_{corrected}}{1 - pct\_garbage}$$

Draws of deaths were processed through noise reduction before calculating the final redistribution variance passed to CODEm, which was added to the total data variance. The mean of the draws was not used as the final estimate because it was found that the logit transformation biases the distribution of cause fractions higher than if only point estimates are used.

Section 5.1.8: **HIV/AIDS misclassification correction (step 6)**

In many location-years, certain causes of death known to be comorbid with HIV/AIDS (e.g., tuberculosis, other infectious diseases) are seen to have age patterns that diverge from those observed in location-years without widespread HIV epidemics and are in fact more reflective of HIV mortality trends. To identify these instances, a global relative age pattern is generated by using all VR deaths in countries with observed HIV prevalence less than 1% by using the following equation

$$RR_{asc} = \frac{R_{asc}}{\bar{x}(R_{65sc}, R_{70sc}, R_{75sc})}$$

Where:

RR_{asc} is the relative death rate for age group *a*, sex *s*, cause *c*;

R_{asc} is the rate for that age group

$\bar{x}(R_{65sc}, R_{70sc}, R_{75sc})$ is the mean of the rates in ages 65–69, 60–74, and 75–79 for that sex and cause.

This is preferable to comparing mortality rates because we are able to isolate divergence in age pattern while accounting for varying levels of overall mortality by fixing death rates to age groups that are unlikely to be confounded by the presence of HIV. Expected deaths for an identified cause were then determined by the equation

$$ED_{lyasc} = \bar{x}\left(R_{ly65sc}, R_{ly70sc}, R_{ly75sc}\right) \times p_{lasc} \times RR_{asc}$$

Where

ED_{lasc} are deaths for location *l*, year *y*, age group *a*, sex *s*, and cause *c*;

$\bar{x}(R_{l65sc}, R_{l70sc}, R_{l75sc})$ is the mean of the rates for ages 65–69, 60–74, and 75–79 for that location-year-sex-cause;

p_{lasc} is the population for that location-year-age-sex-cause

RR_{asc} is the global standard relative rate determined in the previous step for that age-sex-cause.

The expected deaths remain attributed to that particular cause, while the difference between observed and expected are reallocated to HIV/AIDS.

Section 5.1.9: **Scale strata to province (step 7)**

Over time, a higher proportion of deaths have been registered in China through the expansion of the DSP system and provincial/county efforts to increase CoD registration. With the expansion of coverage, it is possible that province aggregates do not accurately represent the population distribution between urban and rural areas in each year. For this reason, we stratified the data preparation by urban and rural status for each county within each province. Stratification was based on the median level of urbanisation across counties within each province as recorded in the 2010 China census. In the provinces of Tibet and Hainan, all counties were placed into one strata based on largely homogeneous urbanisation levels within each province. This yielded a total of 62 analytical province-strata. Macao and Hong Kong were not included in this stratification system as the VR systems there are independent from that on the mainland; no weighting scheme needs to be carried out in these complete VR systems with quality data on CoDs.

Within each province-strata, a larger proportion of deaths in-hospital might be reported than that of deaths outside of hospital because of the internet hospital reporting system. To avoid bias, we reweighted in-hospital and out-of-hospital deaths based on the age-sex-province-specific fraction of deaths in and out of hospital in the DSP system. DSP data have been used to establish these percentages because, in these communities, there is a concerted effort to identify all out-of-hospital deaths. Province-strata death rates are combined to produce overall province death rates by weighting each strata by population in each age-sex-year group. Province death rates are rescaled so that all-cause mortality equals the estimated death rate in each age-sex-year estimated in the life-table analysis. The Bayesian noise reduction algorithm was used to deal with zero counts and small number issues for rare causes.[30]

Section 5.1.10: **Restrictions post-redistribution (step 8)**

Some causes of death can only be reliably assigned through an autopsy by a trained physician. For example, a VA would be unlikely to reliably distinguish between ischaemic and haemorrhagic stroke.

This step ensures that the detail of the cause list at this point in the data prep process is reasonable given the detail of the original data source and the methods by which the CoD was assigned. Two primary corrections are applied. First, any cause that is purely an artifact of the redistribution machinery targeting too detailed a cause is aggregated up to the parent cause. Second, a “bridge map” is applied over a certain set of sources to ensure that these sources do

not contain causes that could not reliably be determined by the methods used. These two corrections are applied to ICD-9 detail, ICD-9 BTL, ICD-10 tabulated, ICD-8 detail, ICD-8 A, China DSP (tabulated ICD-9), India MCCD, India SRS, USSR tabulated ICD-9, the Philippine Vital Statistics Reports, Iran ICD-10 VR from the Ministry of Health and Medical Education, and all VA.

Section 5.1.11: **Drop VR country years or mark as non-representative (step 9)**

Lozano and colleagues[34] describe the negative impact that low-completeness VR data could have on CoD modelling for GBD 2010. In particular, in settings where a data source does not capture all deaths in a population, the cause composition of deaths captured might be different from those that are not. However, a completeness sensitivity test found that low- completeness VR data had little impact on the cause-specific mortality trends at the global level.

For GBD 2019, we investigated the impact of these data at the country and subnational levels by using the more thorough diagnostic visualisations available to us. We determined that these data produced unlikely trends in the models affected. Despite the minimal impact on global trends, better models were produced by eliminating or marking as non-representative data with extremely low completeness. VR completeness was estimated as the number of deaths registered divided by the number of deaths estimated in the GBD mortality envelope.

For this round, VR location-years with completeness less than 50% were dropped, while location-years with completeness between 50% and 69% were marked as non-representative.

In addition, any country-year with a number of deaths registered to major garbage codes greater than 50% of the deaths registered was dropped.

Section 5.1.12: **Cause aggregation (step 10)**

The cause list is organised in a top-down hierarchical format containing four levels. The first group, or Level 1, sums all causes. Following all cause-mortality are Level 2 causes, which include three broad groupings of causes of deaths: “communicable, maternal, neonatal, and nutritional diseases”; “non-communicable diseases”; and “injuries”. Within those Level 2 groupings are finer levels used for modelling. Level 3, or parent causes, are aggregated; the mortality estimate for a parent cause in the hierarchy represents the sum of the causes under that rubric. Sub-causes within Level 3 causes – Level 4 – are more detailed. For example, the parent cause “intestinal infectious diseases” contains the three sub-causes: “typhoid fever”, “paratyphoid fever”, and “other intestinal infectious diseases”. Included in the parent cause estimate are deaths mapped directly to the parent and any Level 4 sub-causes. In data where there was not enough information to assign a Level 4 cause, we aggregated to the Level 3 parent cause. Exceptions to aggregating the Level 4 sub-causes to the parent are instances when certain sub-causes are not present. The United Nations Crime Trends police data only identify homicides, and aggregating homicides to injuries would not accurately represent all injuries.

Section 5.1.13: **Remove shocks and HIV/AIDS maternal adjustments (step 11)**

For GBD 2019, CODEm models use an HIV/AIDS- and shock-free envelope. To be comparable, cause fractions must also be HIV/AIDS- and shock-free. Cause fractions were uploaded to the CoD database as the number of deaths due to the cause over an adjusted sample in which the

number of deaths due to "HIV/AIDS", "conflict and terrorism", "executions and police conflict", and "exposure to forces of nature" were removed.

Remove HIV/AIDS and shocks from denominator where cause list includes HIV/AIDS (step 11.1)

The first step to generate HIV- and shock-free cause fractions was to remove any deaths from the sample that were directly coded to "HIV/AIDS", "collective violence and legal intervention", or "exposure to forces of nature". The cause fraction uploaded to the database can be calculated by a simple equation.

$$CF_{l,t,a,x,c} = \frac{D_{l,t,a,x,c}}{D_{l,t,a,x} - D_{l,t,a,x,hiv} - D_{l,t,a,x,war} - D_{l,t,a,x,disaster}}$$

Where:

$CF_{l,t,a,x,c}$ is the cause fraction for a location *l*, year *t*, age *a*, sex *x*, and cause *c*

$D_{l,t,a,x,c}$ is the number of deaths observed for cause *c* in location *l*, year *t*, age *a*, and sex *x*

$D_{l,t,a,x}$ is the total number of deaths due to all causes observed in location *l*, year *t*, age *a*, and sex *x*

$D_{l,t,a,x,hiv}$, $D_{l,t,a,x,war}$, and $D_{l,t,a,x,disaster}$ are the numbers of deaths observed in location *l*, year *t*, age *a*, and sex *x* for causes "HIV/AIDS," "collective violence and legal intervention," and "exposure to forces of nature," respectively

Cause fractions for HIV/AIDS and shock causes were also uploaded to the database for use in separate estimation processes described by Wang et al.[5] In this case, cause fractions followed the standard equation, with variables following the same explanation.

$$CF_{l,t,a,x,c} = \frac{D_{l,t,a,x,c}}{D_{l,t,a,x}}$$

Remove HIV/AIDS deaths from maternal mortality sources (step 11.2)

HIV-free cause fractions were also uploaded for sources on mortality due to maternal causes. In these cases, the sample of all deaths observed in the study is likely to contain some amount of deaths due to HIV/AIDS and shocks, but the sample only includes cause information on maternal deaths. To account for the presence of HIV/AIDS and shocks in the entire sample, we assumed the same proportion of total deaths due to HIV/AIDS by location, age, sex, and year as provided from the estimation of HIV/AIDS and all-cause mortality described by Wang et al.[5]

Maternal mortality studies were only corrected for HIV/AIDS if the sample of total deaths was provided in the data source. Where sources provided only the MMR, we applied the rate to the HIV- and shock-free envelope produced by the analysis described in Wang et al.[5] and thus did not need to adjust cause fractions at this point in the process.

Where a correction was applied, we used the following equation:

$$CF_{l,t,a,x,mat} = \frac{D_{l,t,a,x,maternal}}{D_{l,t,a,x,maternal} + \frac{E[D_{l,t,a,x,hiv\_shock\_free}]}{E[D_{l,t,a,x}]} D_{l,t,a,x,non-maternal}}$$

Where:

$CF_{l,t,a,x,mat}$ is the resulting cause fraction due to maternal causes for the location (l), year (t), age (a), sex (x);

$D_{l,t,a,x,mat}$ is the number of observed deaths in the sample due to maternal causes

$D_{l,t,a,x,non-maternal}$ is the number of observed deaths in the sample due to non-maternal causes

$E[D_{l,t,a,x}]$ is the GBD estimate of all-cause mortality in the location, year, age, and sex

$E[D_{l,t,a,x,hiv\_shock\_free}]$ is the GBD estimate of HIV- and shock-free mortality in the location, year, age, and sex

HIV/AIDS correction of sibling history, census, and survey data (step 11.3)

As described in our analysis from GBD 2013, many studies have failed to find increased mortality in HIV+ pregnant mothers, but those who have advanced HIV are known to have increased baseline mortality. Prior to GBD 2013, we did not distinguish between deaths in HIV+ women that were caused by pregnancy and those for whom the pregnancy was incidental to their death. To more explicitly quantify the contribution of pregnancy to death in HIV+ women, and therefore more accurately estimate the maternal death count, we completed two additional analyses for GBD 2013 and all subsequent GBD analyses. First, we determined the population attributable fraction (PAF) of HIV/AIDS to pregnancy-related death. Second, we determined the proportion of pregnancy-related deaths in HIV+ pregnant mothers that are aggravated by pregnancy and are therefore by definition maternal deaths.

$$PAF = \frac{P(RR-1)}{1+P(RR-1)}$$

Where

PAF is the population attributable fraction

P denotes the prevalence of HIV in pregnancy

RR is relative risk of mortality in HIV+ vs HIV- pregnant mothers.

To recap our analysis for GBD 2013, we used the paper published by Calvert and Ronsmans to identify sources[35] that could inform Step 1 of our HIV-correction analysis. We independently reviewed each of the component studies in Calvert and Ronsmans' review and extracted data directly, not from the systematic review paper. We identified only one additional study that was not used in Calvert and Ronsmans' analysis. We have, however, not used all the studies included in that review. Specific details are as follows:

1) Figueroa-Damian, et al. was excluded for not including any postpartum deaths at all.[36]

2) In the case of Ryder, et al. and Zvandasara, et al. we excluded those deaths that occurred more than 12 months after delivery.[37,38]

3) We excluded the results from Chilongozi, et al. from the site that did not include any HIV- patients.[39]

4) Leroy, et al. was not in the bibliography. We could not locate it for review so it was excluded.[40]

5) Kourtis, et al. was extracted with adjustment of the denominator based on the average number of hospitalisations per delivery in each group.[41]

6) Ticconi, et al. was excluded for being both non- representative and including subgroup data from mothers with malaria infection.[42]

A total of 21 sources were included in our analysis of the increased mortality risk of HIV+ versus HIV- women in pregnancy.[43] We performed DerSimonian-Laird random effects meta-analysis to derive a pooled estimate of RR of death during pregnancy given HIV positivity.[44] The pooled effect size was 6·40 (95% uncertainty interval [UI] 3·98−10·29), which was then used to calculate an HIV PAF for each country, age group, and year. To determine the proportion of those HIV-related deaths that were attributable to maternal causes, we performed a second systematic literature review. This time we sought evidence for the excess mortality risk of pregnancy in those women who are already HIV+. Most studies have failed to find such an effect, but most also did not stratify their study population by stage of HIV or ART (antiretroviral therapy) status. Only two studies did this stratification, with a pooled effect size of 1·13 (95% UI 0·73−1·77).

An updated literature review to inform the relative risk of mortality in pregnancy in HIV+ versus HIV- women had 14 non-usable sources. We completed this search on May 10, 2019, using the following search strings:

((HIV[Title/Abstract] OR "Acquired Immunodeficiency Syndrome"[Title/Abstract] OR AIDS[Title/Abstract]) AND (“pregnant”[Title/Abstract] OR “pregnancy”[Title/Abstract] OR “postpartum”[Title/Abstract] OR "post partum"[Title/Abstract]) AND (“mortality”[Title/Abstract] OR “death”[Title/Abstract]) NOT "case report" NOT (animals[MeSH] NOT humans[MeSH]) AND (2016/08/15[PDat] : 3000/12/31[PDat]))

Prevalence of HIV in pregnant women was calculated by using the Joint United Nations Programme on HIV and AIDS (UNAIDS) Spectrum model, a compartmental HIV progression model used to generate age-specific incidence, prevalence, and death rates from pre-calculated incidence curves and assumptions about intervention scale-up and local variation in epidemiology. For each location, we used UNAIDS’ age-specific ratios of fertility in women living with HIV to fertility in women not living with HIV. In most locations, this ratio is assumed to be greater than one in women aged 15–24 years and less than one and decreasing as age increases beyond 24 years. Since Spectrum assumes fertile ages of 15–49 years, we used the ratio of HIV prevalence in pregnant women to HIV prevalence in the general population at either end of that range to extend estimates to age bands 10–14 years and 50–54 years.

Unlike GBD 2013, when we applied the population attributable fraction (PAF) correction to the envelope of maternal deaths predicted by CODEm, we instead applied country-year-age-group-

specific PAF to maternal mortality input data prior to modelling in CODEm. This ensured that both the numerator and denominator of all CF data were internally consistent in their exclusion of background HIV/AIDS mortality. The cause fractions for maternal deaths in sibling history, survey, and census data were therefore adjusted as follows:

$$CF_{l,t,a,x,mat_{adj}} = CF_{l,t,a,x,mat} \times \left(1 - ProP_{hiv_{l,t,a,x}}\right)$$

$$ProP_{hiv_{l,t,a,x}} = PAF_{l,t,a,x,hivpos} \times (1 - rr_{mat})$$

$$CF_{l,t,a,x,mat_{hiv}} = CF_{l,t,a,x,mat} \times ProP_{maternalhiv_{l,t,a,x}}$$

$$ProP_{maternalhiv_{l,t,a,x}} = PAF_{l,t,a,x,hivpos} \times rr_{mat}$$

Where:

$CF_{l,t,a,x,mat}$ = The proportion of deaths due to all maternal causes before HIV/AIDS correction for the location, year, age, and sex.

$CF_{l,t,a,x,mat_{adj}}$= The proportion of deaths due to maternal causes after the adjustment for the location, year, age, and sex.

$CF_{l,t,a,x,mat_{hiv}}$ = The proportion of deaths due to maternal deaths aggravated by HIV/AIDS after the adjustment for the location, year, age, and sex.

$PAF_{l,t,a,x,hivpos}$ = The PAF that describes the percentage of all maternal deaths that were HIV-related for the location, year, age, and sex

$ProP_{hiv_{l,t,a,x}}$ = The proportion of deaths in pregnancy for the location, year, age, and sex that are estimated to be incidental deaths due to HIV/AIDS and therefore not a maternal CoD.

$ProP_{maternalhiv_{l,t,a,x}}$ =The proportion of deaths in pregnancy for the location, year, age, and sex that are estimated to be HIV+ and maternal deaths that are aggravated by HIV/AIDS.

rr_{mat} = .13/1.13 = The proportion of HIV/AIDS deaths during pregnancy that were exacerbated by the pregnancy.

HIV/AIDS correction of other maternal mortality data (step 11.4)

Although a specific subset of codes in ICD-10 corresponds to HIV/AIDS deaths aggravated by pregnancy, these codes are sparsely used and unreliable. We therefore adapted the method described to also correct VR and VA sources for the systematic exclusion of HIV-related maternal deaths. This correction was calculated in the same manner, by using the same input data as above, with the only difference being that HIV correction of VR and VA sources resulted in a net increase in the maternal correction factor Maternal deaths aggravated by HIV/AIDS are calculated in the following way:

$$CF_{l,t,a,x,mat_{hiv}} = CF_{l,t,a,x,mat} \times ProP_{maternalhiv_{l,t,a,x}}$$

$$ProP_{maternalhiv_{l,t,a,x}} = \frac{PAF_{l,t,a,x,hivpos} \times rr_{mat}}{1 - PAF_{l,t,a,x,hivpos} \times rr_{mat}}$$

Section 5.1.14: **Noise Reduction (step 12)**

To deal with problems of zero counts in VR, VA, cancer registries, or sibling histories for a given age group in a given year, we use a Bayesian noise-reduction algorithm. For this algorithm, we assume a normal prior and a normal data likelihood. We estimate the normal prior for a given country-series of data by running a Poisson regression to estimate the number of deaths due to each respective cause and sex with dummy variables for age and year. With two notable exceptions (detailed below), these regressions are sex-, cause-, and country-specific, so borrowing strength over age and year is only within a given data type, country, cause, and sex. The variance of the prior, τ^2, is estimated from the Poisson regression, taking into account the variance-covariance matrix of the regression coefficients. For the data variance, we use the Wilson approximation which provides an estimate of σ^2 even in cases with a zero count of cause-specific deaths. The posterior estimate for each data point is

$$Mean = \left(\frac{\tau^2}{\tau^2+\sigma^2}X + \frac{\sigma^2}{\tau^2+\sigma^2}\mu\right)$$

$$Variance = \left(\frac{\tau^2\sigma^2}{\tau^2+\sigma^2}\right)$$

Where

X is the mean of the data

μ is the mean of the prior.

This approach to noise reduction avoids the problem that zero counts in an *ln* rates model or a logit cause fraction model will be dropped from the regression and lead to upward bias in the estimates. This is particularly important in two settings: high-income countries with small numbers of cause-specific deaths, and the analysis of sibling history data where for any given age group in any given year the number of deaths reported in the survey that are pregnancy-related or the number of deaths from all causes in that age group may be small.

Regarding the exceptions to the regression, the first is that country-years with populations under 1 million are pooled with the region data to prevent overdispersion and provide a stronger signal. Additionally, VA data diverge from the above description in two ways. First, all data for a given super-region are pooled together and a study dummy variable is added, allowing for different studies and surveillance sites to borrow strength from one another within a super-region. Second, unless the data are part of a time series (e.g., the Matlab Health and Demographic Surveillance System), the regression has no year component.

Section 5.1.15: **Cause of death database and outlier identification (step 13)**

Death rates for different CoD generally have a stable age pattern. In large populations, these patterns will not change very rapidly over time. We can assume a relatively stable pattern in death rates for all causes except for some epidemic diseases and specific types of injuries. Rare causes in large populations and prevalent causes in small populations usually have stochastic patterns. To correct for these stochastic patterns, we implemented a noise-reduction process, explained in Step 12.

In VR data, we infrequently find one or more data points for specific geography/age/sex/year combinations that lie very far from the stable pattern of death rates. In these situations, the model usually ignores the data point(s). If the model fails to ignore these data, dramatic jumps or drops can occur in the death rates. When no logical explanation exists for variation in the death rates to this degree, we regard the data point(s) as outlier(s). The selection of data points to regard as outliers occurs after data have been prepped for modelling, as well as during preliminary reviews of the models.

In non-VR sources, data-collection methods and data quality can vary widely from source to source. Where data points in each age-sex-geography-year are very sparse, extreme data points can have a bad effect on regional estimation. In these situations, we investigate the study's methods and consider lower-quality data points as outliers.

Identifying outliers in the CoD data occurs prior to finalisation of models for each cause. We do not automate the selection of outliers but investigate the source of the offending data as well as reviewing other data sources for the same cause, geography, and year. Ultimately, outliers are identified based on the judgement of the modeller and senior faculty. Outlier decisions are reversible and may be revisited.

Section 5.1.16: **Causes of death data star rating calculation**

GBD estimates are most accurate when computed with a full time series of complete VR with a low percentage of garbage codes. For GBD 2016, we developed a simple star-rating system from 0 to 5 to give a picture of the quality of data available in a given country over the full time series used in GBD estimates. Countries improve in the star rating as they increase availability, completeness, and detail of their mortality data and reduce the percentage of deaths coded to ill-defined garbage codes or highly aggregated causes.

We assign "star" ratings to rate the quality of data for any given location year. The inputs that determine this star rating are the percentage of total deaths determined to be major garbage (such as All, Ill-defined), and the level of completeness in the dataset. Causes such as "injuries" or "cancer" will also be included in major garbage percentage because this percentage includes use of highly aggregated causes. These three values were used to create a "percent well-certified" value between 0 and 1, determined as:

$$pct_{wellcertified} = \text{Completeness} \times (1 - pct_{majgarbage})$$

The mapping of percent well certified to star rating is as followed:

0 star: 0% = $pct_{wellcertified}$

1 star: 0% < $pct_{wellcertified}$ < 10%

2 star: 10% <= $pct_{wellcertified}$ < 35%

3 star: 35% <= $pct_{wellcertified}$ < 65%

4 star: 65% <= $pct_{wellcertified}$ < 85%

5 star: $pct_{wellcertified}$ >= 85%

While stars are calculated for each five-year time interval as well as the full time series from 1980 to 2019, stars in the main text are presented for the full time series only.

In the case of VA, all garbage codes are considered ill-defined because redistribution for VA is highly imprecise.

For each VA data source, percent well-certified is

$$pct_{wellcertified} = VerbalAutopsyAdjustment \times (1 - pct_{majgarbage})$$

Where:

$$VerbalAutopsyAdjustment = SubAdj \times RegAdj \times AgeSexCoverage$$

SubAdj is 10% for subnationally representative studies, 100% for nationally representative studies. This adjustment, while arbitrary in its specific value, reflects the bias that can be associated with studies that only cover a potentially non-representative sample of a country's population.

RegAdj is 64% for all VA data sources. This accounts for the inaccuracy of VA in assigning CoD compared to medically verified VR. The specific multiplier 0·64 is based on the chance-corrected concordance of Physician Certified Verbal Autopsy (PCVA) versus medical certification by the Population Health Metrics Research Consortium.[45]

Age-Sex Coverage is the number of deaths estimated in the GBD mortality envelope for the ages and sexes in the study for the country and year divided by the number of deaths estimated in the GBD mortality envelope for the country and year. Studies that only cover children younger than 5 or maternal mortality, for example, will be highly discounted by this multiplier.

Once percent well-certified is calculated for each location-year of VR and each VA study-year, we then combine these into one measurement for each five-year time interval and the full time series 1980–2019. For each five-year time interval, we take the maximum percent well-certified. Then for 1980–2019, we take the average of the maximum percentages well-certified for the seven five-year time intervals, including any five-year time interval in which no data were available as a zero.

Prior to GBD2019, the causes of death team used an all ages, both sex cause fraction to estimate the percentage of garbage coded deaths in a given location year. Thus, the percentage of garbage for a given location year was determined as:

$$CF_G = \frac{D_G}{D}$$

Where CF_G represent the cause fraction of percent garbage. D_G represents total garbage coded deaths, and D represents the total deaths in a given location/year.

In GBD2019 we moved to calculating the percentage of garbage coded deaths using an age standardized cause fraction. The steps for creating these age standardized cause fractions, in the case of garbage, are as followed:

1. Create both sex, **age specific** cause fractions of garbage for each age group

2. Scale these cause fractions by a set of both sex age weights, determined by global mortality estimates from 2010 to present. That is, weights for each GBD age group were determined as:

$$W_a = \frac{D_a}{D}$$

Where W_a is the weight for given age group "a", D_a is the total both sex, global deaths from 2010 to present in age group "a", and D is the total both sex, global deaths from 2010 to present across all ages.

3. Sum these weighted cause fractions across all age groups to produce the age standardized cause fraction

In the case of percent garbage for a given location year, the formula to calculate percent garbage would be given as the sum of the weighted age specific cause fractions across all age groups "a":

$$CF_G = \Sigma_a\left(\frac{G_a}{D_a} \times W_a\right)$$

Where G_a represents the total both sex garbage deaths in age group "a," D_a represents the total both sex deaths in age group "a," and W_a represents the weight generated from mortality estimates for age group "a."

Section 5.2: Causes of death modeling methods

Section 5.2.1: **CODEm**

Overview of methods

CODEm is the framework used to model most cause-specific death rates in the GBD.[46] It relies on four key components:

First, all available data are identified and gathered to be used in the modelling process. Although the data may vary in quality, they all contain some signal of the true epidemiological process.

Second, a diverse set of plausible models are developed to capture well-documented associations in the estimates. Using a wide variety of individual models to create an ensemble predictive model has been shown to outperform techniques using only a single model both in CoD estimation[46]and in more general prediction applications.[33,47]

Third, the out-of-sample predictive validity is assessed for all individual models, which are then ranked for use in the ensemble modelling stage.

Finally, differently weighted combinations of individual models are evaluated to select the ensemble model with the highest out-of-sample predictive validity.

For some causes (e.g., "lower respiratory infections"), evidence exists that the relationship between covariates and death rates might differ between children and adults. Separate models are therefore run for different age ranges, when applicable. Additionally, separate models are developed for countries with extensive, complete, and representative VR for every cause to ensure that uncertainty can better reflect the more complete data in these locations.

In order to ensure the addition of subnational locations are not driving changes in estimates, in GBD 2019, we run a global model that excludes data from non-standard locations; the resulting covariate betas are then used as priors for the true global model.

Model pool development

Because many factors may covary with any given CoD, a range of plausible statistical models are developed for each cause. In the CODEm framework, four families of statistical models are used: linear mixed effects regression (LMER) models of the natural log of the cause-specific death rate, LMER models of the logit of the cause fraction, spatiotemporal Gaussian process regression (ST-GPR) models of the natural logarithm of the cause-specific death rate, and ST-GPR models of the logit of the cause fraction (see the 2x2 table in Foreman et al).[46] For each family of models, all plausible relationships between covariates and the response variable are identified. Because all possible combinations of selected covariates are considered for each family of models, multi-collinearity between covariates may produce implausible signs on coefficients or unstable coefficients. Each combination is therefore tested for statistical significance (covariate coefficients must have a coefficient with p-value < 0·05) and plausibility (the coefficients must have the directions expected on the basis of the literature). Only covariate combinations meeting these criteria are retained. This selection process is run for both cause fractions and death rates, then ST-GPR and LMER-only models are created for each set of covariates. For a detailed explanation of the covariate selection algorithm, see Foreman et al 2012.[46]

Data variance estimation

The families of models that go through ST-GPR incorporate information about data variance. The main inputs for a Gaussian process regression (GPR) are a mean function, a covariance function, and data variance for each data point. These inputs are described in detail in Foreman, et al. 2012.[46] For GBD 2019, we have updated this calculation to incorporate garbage code redistribution uncertainty.

Three components of data variance are now used in CODEm: sampling variance, non-sampling variance, and garbage code redistribution variance. The computation of sampling variance and non-sampling variance has not changed since previous iterations of the GBD and is also described in Foreman, et al. 2012.[46] Garbage code redistribution variance is computed in the CoD database process. Since variance is additive, we calculate total data variance as the sum of sampling variance, non-sampling variance, and redistribution variance. Increased data variance in GPR results in the GPR draws not following the data point as closely.

Testing model pool on 15% sample

The performance of all models (individual and ensemble) is evaluated by means of out-of-sample predictive validity tests. Thirty percent of the data are excluded from the initial model fits. These individual model fits are evaluated and ranked by using half of the excluded data (15% of the total), then used to construct the ensembles on the basis of their performance. Data are held out from the analysis on the basis of the cause-specific missingness patterns for ages and years across locations. Out-of-sample predictive validity testing is repeated 20 times for each model, which has been shown to produce stable results.[46] These performance tests

include the root mean square error (RMSE) for the log of the cause-specific death rate, the direction of the predicted versus actual trend in the data, and the coverage of the predicted 95% UI.

Ensemble development and testing

The component models are weighted on the basis of their predictive validity rank to determine their contribution to the ensemble estimate. The relative weights are determined both by the model ranks and by a parameter ψ, whose value determines how quickly the weights taper off as rank decreases. The distribution of ψ is described in more detail in Foreman, et al. 2012.[46] A set of ensemble models is then created by using the weights constructed from the combinations of ranks and ψ values. These ensembles are tested by using the predictive validity metrics described on the remaining 15% of the data, and the ensemble with the best performance in out-of-sample trend and RMSE is chosen as the final model.

Final estimation

Once a weighting scheme has been chosen, 1,000 draws are created for the final ensemble, and the number of draws contributed by each model is proportional to its weight. The mean of the draws is used as the final estimate for the CODEm process, and a 95% UI is created from the 0·025 and 0·975 quantiles of the draws. The validity of the UI can be checked via its coverage of the out-of-sample data; ideally, the 95% UI would capture 95% of these data. Higher coverage suggests that the UIs are too large, and lower coverage suggests overfitting.

Selection of causes for which CODEm is used

CODEm is used to model 193 causes. However, it is unsuitable for use in modelling certain causes, including those with very low death counts, those where cause-specific death record availability is inadequate, or those for which there are marked biases or variability for CoD certification over time that cannot be fully accounted for with the current garbage code redistribution algorithms. Criteria for causes where CODEm is not used are discussed in further detail in Section 4.2.2.

Model-specific covariates

Modelers select covariates to be used in CODEm, but those covariates may not be significant or in the direction specified during the covariate selection step of CODEm and will therefore not be used in the model. These covariates are listed with a '—' for number of draws. Additionally, covariates may be selected by CODEm but only exist in submodels that perform poorly and may end up with zero draws included in the final ensemble. Finally, all other covariates are listed with the number of draws in the final ensemble from submodels that had the covariate.

Section 5.2.2: **Causes modeled outside of CODEm**

Overview

A number of causes required alternative modelling strategies to those used for CODEm because they were not compatible with CODEm estimation infrastructure and processes. Such unsuitability included having very low death counts; inadequate availability of cause-specific death records; and marked biases or variability for CoD certification over time that could not be fully accounted for with current garbage code redistribution algorithms. The inclusion of these

causes in CODEm often renders its out-of-sample predictive validity testing unstable, but the validity of this type of testing is a key advantage of using CODEm for CoD estimation. Alternately, CODEm simply fails to generate plausible mortality rates in the absence of enough VR or VA data when these causes are included. Because of increased data availability and redistribution algorithm refinements, we were able to incorporate several new causes, which were modelled separately for GBD 2013, into CODEm for this iteration of the GBD study; with each annual update of GBD, we aim to add more causes within the CODEm estimation space. For GBD 2019, we used alternative modelling approaches for these causes, including negative binomial models, natural history models, sub-cause proportion models, and prevalence-based models.

Negative binomial models

For eight rare CoDs, too few observed deaths were included in the CoD database to produce stable estimates. For these causes, we ran negative binomial regression models, with either a constant or a constant multiplied by the mean assumption for the dispersion parameter, by using reverse step-wise model building. We selected one of the two model dispersion assumptions based on best fit to the data by using the same method as GBD 2013. For GBD 2015, we also tested zero-inflated Poisson models for these rare CoDs but rejected them after finding that they did not substantially affect the mean predictions but instead produced unrealistically large UIs. Descriptions of the modeling process for each of these causes follows in the next sections.

DisMod-MR 2.1

Overview of DisMod

Until GBD 2010, non-fatal estimates were based on a single data source on prevalence, incidence, remission, or a mortality risk selected by the researcher as most relevant to a particular location and time. For GBD 2010, we set a more ambitious goal: to evaluate all available information on a disease that passes a minimum quality standard. That required a different analytical tool that would be able to pool disparate information presented in varying age groupings and from data sources by using different methods. The DisMod-MR 1.0 tool used in GBD 2010 evaluated and pooled all available data, adjusted data for systematic bias associated with methods that varied from the reference and produced estimates with UIs by world regions. For GBD 2013, the improved DisMod-MR 2.0 had increased computational speed, allowing computations that were consistent between all disease parameters at the country rather than the region level. The hundred-fold increase in speed of DisMod-MR 2.0 was partly due to a more efficient rewrite of the code in C++ but also to changing to a model specification using log rates rather than a negative binomial model used in DisMod-MR 1.0. In cross-validation tests, the log rates specification worked as well as or better than the negative binomial specification.[48] For GBD 2015, the computational engine (DisMod-MR 2.1) remained substantively unchanged, but we re-wrote the “wrapper” code that organised the flow of data and settings at each level of the analytical cascade. The sequence of estimation occurred at five levels: global, super-region, region, country, and, where applicable, subnational locations (see flow diagram of DisMod-MR 2.1 cascade that follows). The super-region priors were generated at the global level with mixed-effects, non-linear regression by using all available data; the

super-region fit, in turn, informed the region fit and so on down the cascade. The wrapper gave analysts the choice to branch the cascade in terms of time and sex at different levels depending on data density. The default used in most models was to branch by sex after the global fit but to retain all years of data until the lowest level in the cascade. For GBD 2015, we generated fits for the years 1990, 1995, 2000, 2005, 2010, and 2015.

In updating the wrapper, we consolidated the code base into a single language, Python, to make the code more transparent and efficient and to better deal with subnational estimation. The computational engine is limited to three levels of random effects; we differentiated estimates at the super-region, region, and country levels. In GBD 2013, the subnational units of China, Mexico, and the UK were treated as "countries" such that a random effect was estimated for every location with contributing data. However, the lack of a hierarchy between country and subnational units meant that the fit to country data contributed as much to the estimation of a subnational unit as the fits for all other countries in the region. We found inconsistency between the country fit and the aggregation of subnational estimates when the country's epidemiology varied from the average of the region. Adding an additional level of random effects required a prohibitively comprehensive rewrite of the underlying DisMod-MR engine. Instead, we added a fifth layer to the cascade, with subnational estimation informed by the country fit and country covariates, plus an adjustment based on the average of the residuals between the subnational unit's available data and its prior. This procedure mimicked the impact of a random effect on estimates between subnationals.

For GBD 2015, we improved how country covariates differentiate non-fatal estimates for diseases with sparse data. The coefficients for country covariates were re-estimated at each level of the cascade. For a given location, country coefficients were calculated by using both data and prior information available for that location. In the absence of data, the coefficient of its parent location was chosen to utilise the predictive power of our covariates in data sparse situations.

For GBD 2017, the DisMod-MR 2.1 tool was used. Updates included estimation of new age groups through the GBD 2017 terminal age group of 95+ in addition to the new locations added for the GBD 2017 cycle.

DisMod-MR 2.1 likelihood estimation

Analysts have the choice of using a Gaussian, log-Gaussian, Laplace, or log-Laplace likelihood function in DisMod-MR 2.1. The default log-Gaussian equation for the data likelihood is as follows:

$$-log\left[p\left(y_j|\Phi\right)\right] = \log\left(\sqrt{2\pi}\right) + \log\left(\delta_j + s_j\right) + \frac{1}{2}\left(\frac{\log\left(a_j + \eta_j\right) - \log\left(m_j + \eta_j\right)}{\delta_j + s_j}\right)^2$$

Where:

y_j is a "measurement value" (i.e., data point)

Φ denotes all model random variables

η_j is the offset value, *eta*, for a particular "integrand" (prevalence, incidence, remission, excess mortality rate, with-condition mortality rate, cause-specific mortality rate, relative risk, or standardised mortality ratio)

a_j is the adjusted measurement for data point j, defined by

$$a_j = e^{(-u_j - c_j)} y_j$$

Where:

u_j is the total "area effect" (i.e., the sum of the random effects at three levels of the cascade: super-region, region, and country)

c_j is the total covariate effect (i.e., the mean combined fixed effects for sex, study-level, and country-level covariates), defined by

$$c_j = \sum_{k=0}^{K[I(j)]-1} \beta_{I(j),k} \hat{X}_{k,j}$$

with standard deviation

$$s_j = \sum_{l=0}^{L[I(j)]-1} \zeta_{I(j),l} \hat{Z}_{k,j}$$

Where:

k denotes the mean value of each data point in relation to a covariate (also called x-covariate)

$I(j)$ denotes a data point for a particular integrand, j

$\beta_{I(j),k}$ is the multiplier of the kth x-covariate for the ith integrand

$\hat{X}_{k,j}$ is the covariate value corresponding to the data point j for covariate k

l denotes the standard deviation of each data point in relation to a covariate (also called z-covariate)

$\zeta_{I(j),k}$ is the multiplier of the lth z-covariate for the ith integrand

δ_j is the standard deviation for adjusted measurement j, defined by

$$\delta_j = log\left[y_j + e^{(-u_j - c_j)}\eta_j + c_j\right] - log\left[y_j + e^{(-u_j - c_j)}\eta_j\right]$$

Where mj denotes the model for the jth measurement, not counting effects or measurement noise and defined by

$$m_j = \frac{1}{B(j) - A(j)} \int_{A(j)}^{B(j)} I_j(\mathrm{a})\ \mathrm{da}$$

Where:

$A(j)$ is the lower bound of the age range for a data point j

$B(j)$ is the upper bound of the age range for a data point j

$I(j)$ denotes the function of age corresponding to the integrand for data point j

The source code for DisMod-MR 2.1 as well as the wrapper code is available at https://github.com/ihmeuw/ihme-modeling/tree/master/gbd_2017/shared_code/central_comp/nonfatal/dismod .

Natural history models

For some causes for which CoD data may be systematically biased either owing to misclassification or because the disease exists in focal communities without VR or VA studies, we have developed natural history models. In natural history models, incidence and case-fatality rates are modelled separately and then combined to produce estimates of cause-specific mortality.

Prevalence-based models

The modelling strategies for atrial fibrillation and flutter are distinct from those used for other causes modelled as natural history models. These models use prevalence estimates and excess mortality rates (EMR) generated through DisMod-MR 2.1 rather than incidence and case-fatality rates.

Sub-cause proportion models

For certain sub-causes for which accurate diagnoses are known to be very difficult, we first modelled the parent cause in the GBD hierarchy with CODEm and then allocated deaths to specific causes by using proportions of the parent cause for each age-sex-location-year for each sub-cause. For these causes, we identified no significant predictors in negative binomial regressions. This approach was taken because the available data on these specific causes may come from sources other than VR, such as end-stage renal disease registries, or may come from too few places to model the death rates directly. Details for each cluster of causes analysed in this way follow.

Section 5.2.3: **Central computation**

Imported cases

Imported cases are fatalities that occur in a geographic area where a particular CoD is known to be eradicated in a specific time period or where infection cannot occur. We apply space-time restrictions to these causes in the modeling strategy for that location and time period. However, in some rare cases, deaths from these causes occur outside of restricted locations and time periods. These deaths are referred to as imported cases.

Illustrating this concept, Chagas disease is transmitted by insect vectors that only exist in the Americas. For this reason, Chagas disease is restricted in the models for countries such as Russia. However, someone traveling in Latin America could contract Chagas disease and then die after returning home to Russia. Imported cases accounts for these kinds of deaths.

To calculate these imported cases, we find all cases from the VRs of data-rich countries for any CoD that is otherwise geographically or temporally restricted. We then create a beta distribution from that data point by using the sample size of the VR for that data point and upload these draws as a custom CoD model. This model is then used as an input to CoDCorrect.

CoDCorrect

Objective

As mentioned in the main text, the CoD models are cause-specific. As such, there is no guarantee that the sum of these models will equal the results of the all-cause mortality estimates or that model results of child causes add up to the parent model results. The CoDCorrect process is used to make the CoD and all-cause mortality estimates internally consistent by using a very simple algorithm.

Algorithm and levels

The core algorithm remains the same as it did in GBD 2013. The equation can be written as follows:

$$CD_{lyasjd} = D_{lyasjd}\left(\frac{PD_{lyasjd}}{\sum_{j=1}^{j=k} D_{lyasjd}}\right)$$

Where:

CD_{lyasjd} is the corrected number of deaths for a location l, year y, age a, sex s, cause j, and draw d

PD_{lyasjd} is the parent CoD for a location l, year y, age a, sex s, cause j, and draw d

D_{lyasjd} is the uncorrected number of deaths estimated from a cause-specific model for a l, year y, age a, sex s, cause j, and draw d

The CoDCorrect process starts by rescaling the Level 1 causes to match the all-cause mortality estimates (used for PD_{lyasjd} in the previous equation). Level 2 causes are then rescaled to their corrected parent causes. This process continues until all levels of the hierarchy have been rescaled.

Beginning in GBD 2017, HIV has not been included in the CoDCorrect process. To account for this change, Level 1 CoDCorrect causes are rescaled to HIV-deleted mortality estimates that are produced as part of the mortality and HIV estimation process. Results from the GBD version of Spectrum are added to the post-CoDCorrect death estimates with fatal discontinuities and imported cases to generate the full set of death estimates.

Years of life lost calculation

Years of life lost (YLL) owing to premature mortality were computed for 1082 locations and 39 years. First, we used the lowest observed age-specific mortality rates by location and sex across all estimation years from locations with total populations greater than 5 million in 2016 to establish a theoretical minimum risk reference life table.

The YLL is a metric that is computed by multiplying the number of estimated deaths by the standard life expectancy at age of death. The metric therefore highlights premature deaths by applying a larger weight to deaths that occur in younger age groups. We propagated uncertainty from CoDCorrected deaths for all demographics. The core equation can be written as follows:

$$YLL = \sum_{c=1,a=0,s=1}^{\infty} d_{cas}e_a$$

GBD world population age standard

Age-standardized populations in the GBD were calculated by using the GBD world population age standard. For GBD 2013, GBD 2015, and GBD 2016, the age-specific proportional distributions of all national locations from the UNPOP World Population Prospects 2012 revision for all years from 2010 to 2035 were used to generate a standard population age structure by using the non-weighted mean across all the aforementioned country-years. For GBD 2017, we used the non-weighted mean of 2017 age-specific proportional distributions from the GBD 2017 population estimates for all national locations with a population greater than 5 million people in 2017 to generate an updated standard population age structure.[49] For GBD 2019, we have continued to use this method using GBD 2019 population estimates.[1]

Section 6: **Secondary analyses**

Section 6.1: Demographic methods for converting between mortality rates and probabilities

All-cause mortality was modelled in conditional probability space (${}_nq_x$; probability of death between age x and x+n conditional on survival to age x). For age groups that start at birth, this measure is equivalent to deaths per 1 live birth. To convert to deaths per 1000 live births, the common units for under-5 mortality rate (U5MR) and neonatal mortality rate (NMR), we multiplied qx by 1000.

Cause-specific mortality, conversely, was modelled in rate (deaths per person-year) and cause-fraction (cause-specific deaths over total deaths) space. To make cause-specific results comparable to all-cause results, and also to the SDG targets which are presented as deaths per 1000 live births, we converted these results into conditional probabilities. For each age group,

we calculated the probability of dying from cause *c* as the probability of dying from any cause during the age-interval (all-cause qx) times the proportion of deaths in that age interval where the underlying cause of death is *c*.

Section 6.2: Scenarios for 2030

We considered six different scenarios for future U5MR and NMR based on methods previously described. See main text for more details.

Section 6.3: Mortality benchmarks

Our analysis included two mortality benchmarks. First, in order to benchmark overall child mortality, we calculated a "Global Optimum" NMR and U5MR.

Second, to help with developing intermediate goals and evaluate progress in higher mortality settings, we completed a stochastic frontier analysis (SFA), evaluating the historical relationship between cause-specific neonatal and under-5 mortality and Healthcare Access and Quality (HAQ) Index[23]. The prediction of the frontier is our second benchmark, termed the "Survival Potential Frontier" for NMR and U5MR.

We propose that this two-part approach is necessary to have a framework that both recognizes that equitable classification deaths should not be dependent on income or health system performance, and cedes that a universal benchmark may not have much utility to a country with relatively high child mortality as compared to a benchmark that measures a country against its peers.

Section 6.3.1: **Global Optimum**

We calculated a Global Optimum mortality rate for every age-sex-cause combination. We first subset our mortality rate results to countries with under-5 populations greater than 10,000 (181 out of 204 countries), in order to remove cases with low cause-specific probability of death as a feature of small population size and random noise.

Next, we identified the lowest estimated probability of death between 2000 and 2019 among these countries, for every age-sex-cause grouping. We also identified the lowest estimated all-cause probability of death for these groupings. Then, cause-specific values were scaled to sum to the estimated all-cause Global Optimum.

Section 6.3.2: **Stochastic frontier analysis on HAQ Index**

To benchmark country performance at reducing preventable under-5 deaths against the Healthcare Access and Quality (HAQ) Index[50] , we used a stochastic frontier analysis (SFA). Our SFA model evaluated cause specific probability of death (q) as a function of an intercept (β_{ca}), HAQ Index (HAQ), inefficiency (μ), noise (ν), and error (ϵ):

$$\text{logit}(q_{cal}) = \beta_{ca} + spline(HAQ_l) + \mu_{cal} - \nu_{cal} + \epsilon_{cal}$$

for cause *c*, age *a*, and location *l*, where the model was fit separately by cause and age group. The prediction of the model with zero inefficiency or noise is called the *Survival Potential*

Frontier, and it generally represents a low but observed level of mortality for any value of HAQ Index.

Again, countries with under-5 populations of less than 10,000 were excluded from this analysis to remove random noise due to small population size. For each cause, we also removed countries where cause-specific mortality is equal to zero. The rationale for this is that the stochastic frontier is then interpreted as the level of achievable mortality, by HAQ index, among countries where the deaths due to the cause are observed. For example, for malaria, the stochastic frontier would be specific to malaria endemic countries and would not be reduced to zero or near-zero by countries outside of malaria endemic regions. We also limited input values to the model fit to results for the years 2000-2019. As we did for the Global Optimum benchmark, we scaled cause-specific Survival Potential Frontier estimates to sum to the corresponding all-cause Survival Potential Frontier estimates.

To implement the stochastic frontier analysis, we used a Python-based tool publicly available on GitHub: https://github.com/UW-AMO/StochasticFrontier. B-splines were placed at the following quantiles of HAQ Index: [0, 0.33, 0.66, 1]. Ten percent of countries were trimmed in model fitting.

See attachment to this appendix for more details about the tool used for fitting the SFA.

Appendix Contributions

Providing data or critical feedback on data sources

Gdiom Gebreheat Abady, Jaffar Abbas, Hedayat Abbastabar, Foad Abd-Allah, Amir Abdoli, Hassan Abolhassani, Lucas Guimarães Abreu, Niveen ME Abu-Rmeileh, Victor Adekanmbi, Olatunji O Adetokunboh, Daniel Adedayo Adeyinka, Khashayar Afshari, Mohammad Aghaali, Marcela Agudelo-Botero, Bright Opoku Ahinkorah, Tauseef Ahmad, Muktar Beshir Ahmed, Budi Aji, Oluwaseun Oladapo Akinyemi, Addis Aklilu, Fahad Mashhour Alanezi, Turki M Alanzi, Jacqueline Elizabeth Alcalde-Rabanal, Sheikh Mohammad Alif, Hesam Alizade, Syed Mohamed Aljunid, Amir Almasi-Hashiani, Nelson Alvis-Guzman, Nelson J Alvis-Zakzuk, Saeed Amini, Dickson A Amugsi, Deanna Anderlini, Fereshteh Ansari, Ernoiz Antriyandarti, Davood Anvari, Razique Anwer, Muhammad Aqeel, Jalal Arabloo, Morteza Arab-Zozani, Timur Aripov, Johan Ärnlöv, Ali A Asadi-Pooya, Seyyed Shamsadin Athari, Seyyede Masoume Athari, Desta Debalkie Atnafu, Alok Atreya, Marcel Ausloos, Beatriz Paulina Ayala Quintanilla, Getinet Ayano, Martin Amogre Ayanore, Yared Asmare Aynalem, Samad Azari, Atif Amin Baig, Maciej Banach, Palash Chandra Banik, Sanjay Basu, Mohsen Bayati, Massimiliano Beghi, Derrick A Bennett, Adam E Berman, Akshaya Srikanth Bhagavathula, Dinesh Bhandari, Zulfiqar A Bhutta, Boris Bikbov, Binyam Minuye Birihane, Somayeh Bohlouli, Nicola Luigi Bragazzi, Alexey V Breusov, Andre R Brunoni, Sharath Burugina Nagaraja, Florentino Luciano Caetano dos Santos, Lucero Cahuana-Hurtado, Luis Alberto Cámera, Joao Mauricio Castaldelli-Maia, Carlos A Castañeda-Orjuela, Souranshu Chatterjee, Soosanna Kumary Chattu, Vijay Kumar Chattu, Daniel Youngwhan Cho, Dinh-Toi Chu, Joao Conde, Rosa A S Couto, Lalit Dandona, Rakhi Dandona, Parnaz Daneshpajouhnejad, Farah Deeba, Nikolaos Dervenis, Sagnik Dey, Samath Dhamminda Dharmaratne, Sameer Dhingra, Govinda Prasad Dhungana, Diana Dias da Silva, Leila Doshmangir, Andre Rodrigues Duraes, Iman El Sayed, Shymaa Enany, Sayeh Ezzikouri, Farshad

Farzadfar, Seyed-Mohammad Fereshtehnejad, Irina Filip, Richard Charles Franklin, Takeshi Fukumoto, Mohamed M Gad, Shilpa Gaidhane, Santosh Gaihre, MA Garcia-Gordillo, Ketema Bizuwork Gebremedhin, Lemma Getacher, Ahmad Ghashghaee, Syed Amir Gilani, Myron Anthony Godinho, Mahaveer Golechha, Harish Chander Gugnani, Rajat Das Gupta, Nima Hafezi-Nejad, Arvin Haj-Mirzaian, Asif Hanif, Syed Shahzad Hasan, Soheil Hassanipour, Hadi Hassankhani, Reza Heidari-Soureshjani, Claudiu Herteliu, Mehdi Hosseinzadeh, Mowafa Househ, Segun Emmanuel Ibitoye, Olayinka Stephen Ilesanmi, Sumant Inamdar, Khalid Iqbal, Usman Iqbal, Sheikh Mohammed Shariful Islam, Chidozie C D Iwu, Kathryn H Jacobsen, Vardhmaan Jain, Manthan Dilipkumar Janodia, Tahereh Javaheri, Shubha Jayaram, Achala Upendra Jayatilleke, Ensiyeh Jenabi, John S Ji, Oommen John, Jost B Jonas, Tamas Joo, Farahnaz Joukar, Jacek Jerzy Jozwiak, Mikk Jürisson, Zubair Kabir, Leila R Kalankesh, Naser Kamyari, André Karch, Salah Eddin Karimi, Gbenga A Kayode, Laura Kemmer, Nauman Khalid, Rovshan Khalilov, Maseer Khan, Md Nuruzzaman Khan, Young-Ho Khang, Khaled Khatab, Amir M Khater, Mona M Khater, Ardeshir Khosravi, Daniel Kim, Young-Eun Kim, Yun Jin Kim, Adnan Kisa, Sezer Kisa, Soewarta Kosen, Parvaiz A Koul, Sindhura Lakshmi Koulmane Laxminarayana, Kewal Krishan, Vijay Krishnamoorthy, Barthelemy Kuate Defo, Burcu Kucuk Bicer, G Anil Kumar, Dian Kusuma, Savita Lasrado, Shaun Wen Huey Lee, Yo Han Lee, James Leigh, Juan Liang, Lee-Ling Lim, Ro-Ting Lin, Xuefeng Liu, Alan D Lopez, Shilpashree Madhava Kunjathur, D R Mahadeshwara Prasad, Reza Malekzadeh, Deborah Carvalho Malta, Abdullah A Mamun, Borhan Mansouri, Mohammad Ali Mansournia, Francisco Rogerlândio Martins-Melo, Colm McAlinden, Carlo Eduardo Medina-Solís, Entezar Mehrabi Nasab, Walter Mendoza, Ritesh G Menezes, Endalkachew Worku Mengesha, Atte Meretoja, Bartosz Miazgowski, Irmina Maria Michalek, Ted R Miller, GK Mini, Erkin M Mirrakhimov, Babak Moazen, Masoud Moghadaszadeh, Abdollah Mohammadian-Hafshejani, Shafiu Mohammed, Ali H Mokdad, Mariam Molokhia, Lorenzo Monasta, Mohammad Ali Moni, Ghobad Moradi, Masoud Moradi, Ghulam Mustafa, Ahamarshan Jayaraman Nagarajan, Shankar Prasad Nagaraju, Mohsen Naghavi, Behshad Naghshtabrizi, Sreenivas Narasimha Swamy, Bruno Ramos Nascimento, Javad Nazari, Ionut Negoi, Ruxandra Irina Negoi, Samata Nepal, Henok Biresaw Netsere, Georges Nguefack-Tsague, Josephine W Ngunjiri, Cuong Tat Nguyen, Huong Lan Thi Nguyen, Samuel Negash Nigussie, Chukwudi A Nnaji, Shuhei Nomura, Jean Jacques Noubiap, Vincent Ebuka Nwatah, Bogdan Oancea, Felix Akpojene Ogbo, Bolajoko Olubukunola Olusanya, Jacob Olusegun Olusanya, Ahmed Omar Bali, Obinna E Onwujekwe, Alberto Ortiz, Adrian Otoiu, Mayowa O Owolabi, Mahesh P A, Jagadish Rao Padubidri, Keyvan Pakshir, Raffaele Palladino, Adrian Pana, Songhomitra Panda-Jonas, Anamika Pandey, Ashok Pandey, Sangram Kishor Patel, Shrikant Pawar, Hamidreza Pazoki Toroudi, Amy E Peden, Veincent Christian Filipino Pepito, Jeevan Pereira, Marina Pinheiro, Maarten J Postma, Hadi Pourjafar, Sergio I Prada, Zahiruddin Quazi Syed, Navid Rabiee, Amir Radfar, Mohammad Hifz Ur Rahman, Amir Masoud Rahmani, Kiana Ramezanzadeh, Chhabi Lal Ranabhat, Sowmya J Rao, Prateek Rastogi, Priya Rathi, David Laith Rawaf, Salman Rawaf, Reza Rawassizadeh, Andre M N Renzaho, Bhageerathy Reshmi, Nima Rezaei, Seyed Mohammad Riahi, Leonardo Roever, Luca Ronfani, Enrico Rubagotti, Basema Saddik, Ehsan Sadeghi, Sahar Saeedi Moghaddam, Rajesh Sagar, Mohammad Reza Salahshoor, Abdallah M Samy, Milena M Santric-Milicevic, Sivan Yegnanarayana Iyer Saraswathy, Abdur Razzaque Sarker, Nizal Sarrafzadegan, Arash Sarveazad, Brijesh Sathian, Sonia Saxena, Ganesh Kumar Saya, Mete Saylan, Markus P Schlaich, David C Schwebel, Subramanian Senthilkumaran, Sadaf G Sepanlou, Edson Serván-Mori, Feng Sha,

Amira A Shaheen, Izza Shahid, Masood Ali Shaikh, Ali S Shalash, Mehran Shams-Beyranvand, Mohammed Shannawaz, Sara Sheikhbahaei, Jae Il Shin, Ivy Shiue, Tariq Jamal Siddiqi, Negussie Boti Sidemo, Jonathan I S Silverberg, Biagio Simonetti, Jasvinder A Singh, Deepika Singhal, Eirini Skiadaresi, Valentin Yurievich Skryabin, Anna Aleksandrovna Skryabina, David A Sleet, Badr Hasan Sobaih, Mohammad Reza Sobhiyeh, Chandrashekhar T Sreeramareddy, Mark A Stokes, Rizwan Suliankatchi Abdulkader, Bryan L Sykes, Miklós Szócska, Rafael Tabarés-Seisdedos, Amir Taherkhani, Animut Tagele Tamiru, Gizachew Assefa Tessema, Musliu Adetola Tolani, Marcos Roberto Tovani-Palone, Bach Xuan Tran, Jaya Prasad Tripathy, Giorgos Tsapparellas, Riaz Uddin, Anayat Ullah, Bhaskaran Unnikrishnan, Era Upadhyay, Muhammad Shariq Usman, Maryam Vaezi, Pascual R Valdez, Tommi Juhani Vasankari, Narayanaswamy Venketasubramanian, Vasily Vlassov, Bay Vo, Giang Thu Vu, Yohannes Dibaba Wado, Yasir Waheed, Richard G Wamai, Yanping Wang, Ronny Westerman, Charles Shey Wiysonge, Ai-Min Wu, Naohiro Yonemoto, Seok-Jun Yoon, Mustafa Z Younis, Taraneh Yousefinezhadi, Chuanhua Yu, Zhi-Jiang Zhang, Xiu-Ju George Zhao, Arash Ziapour, Christopher J L Murray, Haidong Wang, and Nicholas J Kassebaum.

Developing methods or computational machinery

Katherine R Paulson, Muktar Beshir Ahmed, Sheikh Mohammad Alif, Hesam Alizade, Amir Almasi-Hashiani, Davood Anvari, Yared Asmare Aynalem, Xiaochen Dai, Nikolaos Dervenis, Sameer Dhingra, Shilpa Gaidhane, William M Gardner, Ketema Bizuwork Gebremedhin, Reza Heidari-Soureshjani, Mehdi Hosseinzadeh, Mowafa Househ, Jalil Jaafari, Tahereh Javaheri, Ensiyeh Jenabi, Laura Kemmer, Rovshan Khalilov, Mohammad Khammarnia, Young-Eun Kim, Adnan Kisa, Sezer Kisa, James Leigh, Alan D Lopez, Borhan Mansouri, Endalkachew Worku Mengesha, Masoud Moghadaszadeh, Ali H Mokdad, Mohsen Naghavi, Behshad Naghshtabrizi, Josephine W Ngunjiri, Ahmed Omar Bali, Hamidreza Pazoki Toroudi, Zahiruddin Quazi Syed, Amir Masoud Rahmani, Chhabi Lal Ranabhat, Reza Rawassizadeh, Robert C Reiner Jr, Seyed Mohammad Riahi, Mohammad Reza Salahshoor, Abdallah M Samy, Abdur Razzaque Sarker, Ganesh Kumar Saya, Negussie Boti Sidemo, Deepika Singhal, Dhirendra Narain Sinha, Animut Tagele Tamiru, Maryam Vaezi, Bay Vo, Ronny Westerman, Lauren B Wilner, Seok-Jun Yoon, Arash Ziapour, Christopher J L Murray, Haidong Wang, and Nicholas J Kassebaum.

Providing critical feedback on methods or results

Katherine R Paulson, Hedayat Abbastabar, Foad Abd-Allah, Sherief M Abd-Elsalam, Aidin Abedi, Hassan Abolhassani, Lucas Guimarães Abreu, Eman Abu-Gharbieh, Abdelrahman I Abushouk, Adeyinka Emmanuel Adegbosin, Victor Adekanmbi, Olatunji O Adetokunboh, Daniel Adedayo Adeyinka, Jose C Adsuar, Khashayar Afshari, Mohammad Aghaali, Bright Opoku Ahinkorah, Tauseef Ahmad, Keivan Ahmadi, Muktar Beshir Ahmed, Budi Aji, Yonas Akalu, Oluwaseun Oladapo Akinyemi, Addis Aklilu, Ziyad Al-Aly, Khurshid Alam, Fahad Mashhour Alanezi, Turki M Alanzi, Jacqueline Elizabeth Alcalde-Rabanal, Tilahun Ali, Gianfranco Alicandro, Sheikh Mohammad Alif, Vahid Alipour, Hesam Alizade, Syed Mohamed Aljunid, Amir Almasi-Hashiani, Hesham M Al-Mekhlafi, Jordi Alonso, Rajaa M Al-Raddadi, Khalid A Altirkawi, Arwa Khalid Alumran, Nelson Alvis-Guzman, Nelson J Alvis-Zakzuk, Edward Kwabena Ameyaw, Arianna Maever L Amit, Dickson A Amugsi, Robert Ancuceanu, Deanna Anderlini, Fereshteh Ansari, Alireza Ansari-Moghaddam, Carl Abelardo T Antonio, Davood Anvari, Razique Anwer, Muhammad Aqeel, Jalal Arabloo, Morteza Arab-Zozani, Timur Aripov, Kurnia Dwi Artanti,

Afsaneh Arzani, Ali A Asadi-Pooya, Mohammad Asghari Jafarabadi, Alok Atreya, Madhu Sudhan Atteraya, Marcel Ausloos, Asma Tahir Awan, Beatriz Paulina Ayala Quintanilla, Getinet Ayano, Martin Amogre Ayanore, Samad Azari, Ghasem Azarian, Zelalem Nigussie Azene, Darshan B B, Ebrahim Babaee, Ashish D Badiye, Atif Amin Baig, Maciej Banach, Palash Chandra Banik, Hiba Jawdat Barqawi, Sanjay Basu, Bernhard T Baune, Mohsen Bayati, Neeraj Bedi, Ettore Beghi, Massimiliano Beghi, Derrick A Bennett, Adam E Berman, Dinesh Bhandari, Nikha Bhardwaj, Pankaj Bhardwaj, Krittika Bhattacharyya, Suraj Bhattarai, Zulfiqar A Bhutta, Boris Bikbov, Antonio Biondi, Raaj Kishore Biswas, Somayeh Bohlouli, Nicola Luigi Bragazzi, Alexey V Breusov, Andre R Brunoni, Katrin Burkart, Sharath Burugina Nagaraja, Reinhard Busse, Zahid A Butt, Florentino Luciano Caetano dos Santos, Lucero Cahuana-Hurtado, Luis Alberto Cámera, Rosario Cárdenas, Juan J Carrero, Joao Mauricio Castaldelli-Maia, Carlos A Castañeda-Orjuela, Giulio Castelpietra, Ester Cerin, Jung-Chen Chang, Wagaye Fentahun Chanie, Jaykaran Charan, Souranshu Chatterjee, Soosanna Kumary Chattu, Vijay Kumar Chattu, Sarika Chaturvedi, Daniel Youngwhan Cho, Jee-Young Jasmine Choi, Dinh-Toi Chu, Liliana G Ciobanu, Massimo Cirillo, Joao Conde, Vera Marisa Costa, Berihun Assefa Dachew, Saad M A Dahlawi, Hancheng Dai, Xiaochen Dai, Lalit Dandona, Rakhi Dandona, Jai K Das, Claudio Alberto Dávila-Cervantes, Kairat Davletov, Fernando Pio De la Hoz, Nikolaos Dervenis, Assefa Desalew, Keshab Deuba, Sagnik Dey, Samath Dhamminda Dharmaratne, Sameer Dhingra, Govinda Prasad Dhungana, Diana Dias da Silva, Daniel Diaz, Fariba Dorostkar, Leila Doshmangir, Arielle Wilder Eagan, Hisham Atan Edinur, Ferry Efendi, Iman El Sayed, Maha El Tantawi, Islam Y Elgendy, Amir Emami, Oghenowede Eyawo, Pawan Sirwan Faris, Farshad Farzadfar, Nazir Fattahi, Mehdi Fazlzadeh, Valery L Feigin, Pietro Ferrara, Irina Filip, Florian Fischer, James L Fisher, Nataliya A Foigt, Morenike Oluwatoyin Folayan, Masoud Foroutan, Richard Charles Franklin, Sara D Friedman, Takeshi Fukumoto, Mohamed M Gad, Abhay Motiramji Gaidhane, Shilpa Gaidhane, Santosh Gaihre, William M Gardner, Mariana Gaspar Fonseca, Lemma Getacher, Ahmad Ghashghaee, Asadollah Gholamian, Tiffany K Gill, Giorgia Giussani, Elena V Gnedovskaya, Myron Anthony Godinho, Amit Goel, Mahaveer Golechha, Philimon N Gona, Sameer Vali Gopalani, Houman Goudarzi, Michal Grivna, Harish Chander Gugnani, Davide Guido, Rafael Alves Guimarães, Rajat Das Gupta, Rajeev Gupta, Nima Hafezi-Nejad, Mohammad Rifat Haider, Samer Hamidi, Arief Hargono, Ahmed I Hasaballah, Md Mehedi Hasan, Syed Shahzad Hasan, Soheil Hassanipour, Hadi Hassankhani, Rasmus J Havmoeller, Khezar Hayat, Nathaniel J Henry, Claudiu Herteliu, Ramesh Holla, Naznin Hossain, Mostafa Hosseini, Mehdi Hosseinzadeh, Mihaela Hostiuc, Mowafa Househ, Ayesha Humayun, Bing-Fang Hwang, Segun Emmanuel Ibitoye, Kevin S Ikuta, Olayinka Stephen Ilesanmi, Irena M Ilic, Milena D Ilic, Sumant Inamdar, Leeberk Raja Inbaraj, Khalid Iqbal, Usman Iqbal, M Mofizul Islam, Sheikh Mohammed Shariful Islam, Hiroyasu Iso, Masao Iwagami, Chidozie C D Iwu, Jalil Jaafari, Kathryn H Jacobsen, Jagnoor Jagnoor, Vardhmaan Jain, Manthan Dilipkumar Janodia, Tahereh Javaheri, Fatemeh Javanmardi, Shubha Jayaram, Achala Upendra Jayatilleke, Ensiyeh Jenabi, Ravi Prakash Jha, John S Ji, Oommen John, Jost B Jonas, Farahnaz Joukar, Jacek Jerzy Jozwiak, Mikk Jürisson, Ali Kabir, Zubair Kabir, Leila R Kalankesh, Naser Kamyari, Tanuj Kanchan, Neeti Kapoor, Behzad Karami Matin, André Karch, Salah Eddin Karimi, Getinet Kassahun, Gbenga A Kayode, Ali Kazemi Karyani, Rovshan Khalilov, Mohammad Khammarnia, Ejaz Ahmad Khan, Gulfaraz Khan, Maseer Khan, Md Nuruzzaman Khan, Young-Ho Khang, Khaled Khatab, Amir M Khater, Mona M Khater, Maryam Khayamzadeh, Daniel Kim, Young-Eun Kim, Yun Jin Kim, Ruth W Kimokoti, Adnan Kisa, Sezer Kisa, Niranjan

Kissoon, Jacek A Kopec, Sindhura Lakshmi Koulmane Laxminarayana, Ai Koyanagi, Kewal Krishan, Vijay Krishnamoorthy, Barthelemy Kuate Defo, Burcu Kucuk Bicer, Vaman Kulkarni, G Anil Kumar, Manasi Kumar, Nithin Kumar, Om P Kurmi, Dian Kusuma, Carlo La Vecchia, Faris Hasan Lami, Anders O Larsson, Savita Lasrado, Zohra S Lassi, Paolo Lauriola, Shaun Wen Huey Lee, Yo Han Lee, James Leigh, Matilde Leonardi, Sonia Lewycka, Bingyu Li, Shanshan Li, Lee-Ling Lim, Miteku Andualem Limenih, Xuefeng Liu, Rakesh Lodha, Alan D Lopez, Rafael Lozano, Raimundas Lunevicius, Mark T Mackay, Shilpashree Madhava Kunjathur, D R Mahadeshwara Prasad, Mina Maheri, Morteza Mahmoudi, Azeem Majeed, Venkatesh Maled, Shokofeh Maleki, Reza Malekzadeh, Ahmad Azam Malik, Deborah Carvalho Malta, Abdullah A Mamun, Borhan Mansouri, Mohammad Ali Mansournia, Gabriel Martinez, Santi Martini, Francisco Rogerlândio Martins-Melo, Seyedeh Zahra Masoumi, Colm McAlinden, John J McGrath, Carlo Eduardo Medina-Solís, Fabiola Mejia-Rodriguez, Ziad A Memish, Walter Mendoza, Ritesh G Menezes, Endalkachew Worku Mengesha, Atte Meretoja, Tuomo J Meretoja, Abera M Mersha, Tomislav Mestrovic, Tomasz Miazgowski, Irmina Maria Michalek, Ted R Miller, GK Mini, Mohammad Miri, Andreea Mirica, Erkin M Mirrakhimov, Hamed Mirzaei, Maryam Mirzaei, Babak Moazen, Masoud Moghadaszadeh, Osama Mohamad, Yousef Mohammad, Seyyede Momeneh Mohammadi, Abdollah Mohammadian-Hafshejani, Shafiu Mohammed, Ali H Mokdad, Mariam Molokhia, Stefania Mondello, Mohammad Ali Moni, Ghobad Moradi, Rahmatollah Moradzadeh, Lidia Morawska, Jonathan F Mosser, Amin Mousavi Khaneghah, Mehdi Naderi, Ahamarshan Jayaraman Nagarajan, Shankar Prasad Nagaraju, Mohsen Naghavi, Behshad Naghshtabrizi, Mukhammad David Naimzada, Vinay Nangia, Sreenivas Narasimha Swamy, Bruno Ramos Nascimento, Muhammad Naveed, Rawlance Ndejjo, Ionut Negoi, Ruxandra Irina Negoi, Evangelia Nena, Samata Nepal, Henok Biresaw Netsere, Georges Nguefack-Tsague, Josephine W Ngunjiri, Chi Thi Yen Nguyen, Cuong Tat Nguyen, Huong Lan Thi Nguyen, Yeshambel T Nigatu, Samuel Negash Nigussie, Molly R Nixon, Chukwudi A Nnaji, Shuhei Nomura, Jean Jacques Noubiap, Vincent Ebuka Nwatah, Bogdan Oancea, Oluwakemi Ololade Odukoya, Felix Akpojene Ogbo, Bolajoko Olubukunola Olusanya, Jacob Olusegun Olusanya, Ahmed Omar Bali, Obinna E Onwujekwe, Alberto Ortiz, Adrian Otoiu, Nikita Otstavnov, Stanislav S Otstavnov, Mayowa O Owolabi, Mahesh P A, Jagadish Rao Padubidri, Smita Pakhale, Pramod Kumar Pal, Raffaele Palladino, Adrian Pana, Songhomitra Panda-Jonas, Anamika Pandey, Ashok Pandey, Seithikurippu R Pandi-Perumal, Helena Ullyartha Pangaribuan, Ana Melisa Pardo-Montaño, Eun-Kee Park, Sangram Kishor Patel, Shrikant Pawar, Hamidreza Pazoki Toroudi, Amy E Peden, Veincent Christian Filipino Pepito, Emmanuel K Peprah, Jeevan Pereira, Jorge Pérez-Gómez, Michael A Piradov, Meghdad Pirsaheb, James A Platts-Mills, Khem Narayan Pokhrel, Maarten J Postma, Hadi Pourjafar, Sergio I Prada, Sanjay Prakash, Elisabetta Pupillo, Zahiruddin Quazi Syed, Navid Rabiee, Amir Radfar, Ata Rafiee, Alireza Rafiei, Alberto Raggi, Shadi Rahimzadeh, Mohammad Hifz Ur Rahman, Amir Masoud Rahmani, Juwel Rana, Chhabi Lal Ranabhat, Sowmya J Rao, Prateek Rastogi, Priya Rathi, David Laith Rawaf, Salman Rawaf, Wasiq Faraz Rawasia, Reza Rawassizadeh, Robert C Reiner Jr, Andre M N Renzaho, Bhageerathy Reshmi, Serge Resnikoff, Negar Rezaei, Nima Rezaei, Aziz Rezapour, Seyed Mohammad Riahi, Daniela Ribeiro, Jennifer Rickard, Leonardo Roever, Dietrich Rothenbacher, Enrico Rubagotti, Susan Fred Rumisha, Paul MacDaragh Ryan, Basema Saddik, Ehsan Sadeghi, Sahar Saeedi Moghaddam, Rajesh Sagar, Mohammad Reza Salahshoor, Sana Salehi, Marwa Rashad Salem, Joshua A Salomon, Yoseph Leonardo Samodra, Abdallah M Samy, Juan Sanabria, Milena M

Santric-Milicevic, Sivan Yegnanarayana Iyer Saraswathy, Abdur Razzaque Sarker, Arash Sarveazad, Brijesh Sathian, Thirunavukkarasu Sathish, Sonia Saxena, Ganesh Kumar Saya, Mete Saylan, Markus P Schlaich, David C Schwebel, Falk Schwendicke, Sadaf G Sepanlou, Edson Serván-Mori, Feng Sha, Omid Shafaat, Saeed Shahabi, Mohammad Shahbaz, Amira A Shaheen, Izza Shahid, Masood Ali Shaikh, Ali S Shalash, Mehran Shams-Beyranvand, Mohammed Shannawaz, Kiomars Sharafi, Aziz Sheikh, Sara Sheikhbahaei, Wondimeneh Shibabaw Shiferaw, Mika Shigematsu, Jae Il Shin, Rahman Shiri, Ivy Shiue, Kerem Shuval, Tariq Jamal Siddiqi, Negussie Boti Sidemo, João Pedro Silva, Jonathan I S Silverberg, Biagio Simonetti, Balbir Bagicha Singh, Jasvinder A Singh, Deepika Singhal, Eirini Skiadaresi, Valentin Yurievich Skryabin, Anna Aleksandrovna Skryabina, David A Sleet, Badr Hasan Sobaih, Shahin Soltani, Chandrashekhar T Sreeramareddy, Paschalis Steiropoulos, Mark A Stokes, Stefan Stortecky, Mu'awiyyah Babale Sufiyan, Rizwan Suliankatchi Abdulkader, Gerhard Sulo, Bryan L Sykes, Mindy D Szeto, Rafael Tabarés-Seisdedos, Eyayou Girma Tadesse, Animut Tagele Tamiru, Md Ismail Tareque, Arash Tehrani-Banihashemi, Mohamad-Hani Temsah, Fisaha Haile Tesfay, Gizachew Assefa Tessema, Rekha Thapar, Musliu Adetola Tolani, Marcos Roberto Tovani-Palone, Eugenio Traini, Bach Xuan Tran, Giorgos Tsapparellas, Aristidis Tsatsakis, Lorainne Tudor Car, Riaz Uddin, Anayat Ullah, Chukwuma David Umeokonkwo, Bhaskaran Unnikrishnan, Era Upadhyay, Muhammad Shariq Usman, Marco Vacante, Maryam Vaezi, Pascual R Valdez, Narayanaswamy Venketasubramanian, Madhur Verma, Francesco S Violante, Bay Vo, Giang Thu Vu, Yohannes Dibaba Wado, Yasir Waheed, Richard G Wamai, Yanzhong Wang, Paul Ward, Andrea Werdecker, Ronny Westerman, Nuwan Darshana Wickramasinghe, Charles Shey Wiysonge, Ai-Min Wu, Chenkai Wu, Yang Xie, Srikanth Yandrapalli, Sanni Yaya, Vahid Yazdi-Feyzabadi, Paul Yip, Naohiro Yonemoto, Seok-Jun Yoon, Mustafa Z Younis, Zabihollah Yousefi, Taraneh Yousefinezhadi, Chuanhua Yu, Syed Saoud Zaidi, Sojib Bin Zaman, Mohammad Zamani, Maryam Zamanian, Mikhail Sergeevich Zastrozhin, Yunquan Zhang, Zhi-Jiang Zhang, Arash Ziapour, Simon I Hay, Christopher J L Murray, Haidong Wang, and Nicholas J Kassebaum.

Drafting the work or revising is critically for important intellectual content

Katherine R Paulson, Aruna M Kamath, Kelly Bienhoff, Mohsen Abbasi-Kangevari, Foad Abd-Allah, Sherief M Abd-Elsalam, Hassan Abolhassani, Lucas Guimarães Abreu, Eman Abu-Gharbieh, Niveen ME Abu-Rmeileh, Abdelrahman I Abushouk, Aishatu L Adamu, Victor Adekanmbi, Olatunji O Adetokunboh, Daniel Adedayo Adeyinka, Jose C Adsuar, Khashayar Afshari, Mohammad Aghaali, Bright Opoku Ahinkorah, Muktar Beshir Ahmed, Budi Aji, Yonas Akalu, Khurshid Alam, Jacqueline Elizabeth Alcalde-Rabanal, Ayman Al-Eyadhy, Hesam Alizade, Amir Almasi-Hashiani, Nihad A Almasri, Jordi Alonso, Arwa Khalid Alumran, Nelson Alvis-Guzman, Nelson J Alvis-Zakzuk, Saeed Amini, Mostafa Amini-Rarani, Dickson A Amugsi, Robert Ancuceanu, Deanna Anderlini, Carl Abelardo T Antonio, Ernoiz Antriyandarti, Jalal Arabloo, Morteza Arab-Zozani, Timur Aripov, Johan Ärnlöv, Kurnia Dwi Artanti, Malke Asaad, Mehran Asadi-Aliabadi, Ali A Asadi-Pooya, Alok Atreya, Marcel Ausloos, Beatriz Paulina Ayala Quintanilla, Martin Amogre Ayanore, Zelalem Nigussie Azene, Darshan B B, Ebrahim Babaee, Atif Amin Baig, Maciej Banach, Suzanne Lyn Barker-Collo, Hiba Jawdat Barqawi, Quique Bassat, Sanjay Basu, Bernhard T Baune, Neeraj Bedi, Ettore Beghi, Michelle L Bell, Salaheddine Bendak, Isabela M Bensenor, Kidanemaryam Berhe, Adam E Berman, Yihienew Mequanint Bezabih, Dinesh Bhandari, Krittika Bhattacharyya, Suraj Bhattarai, Boris Bikbov, Antonio Biondi, Nicola

Luigi Bragazzi, Alexey V Breusov, Sharath Burugina Nagaraja, Florentino Luciano Caetano dos Santos, Lucero Cahuana-Hurtado, Paulo Camargos, Giulia Carreras, Juan J Carrero, Felix Carvalho, Joao Mauricio Castaldelli-Maia, Carlos A Castañeda-Orjuela, Ester Cerin, Souranshu Chatterjee, Soosanna Kumary Chattu, Vijay Kumar Chattu, Sarika Chaturvedi, Simiao Chen, Daniel Youngwhan Cho, Dinh-Toi Chu, Liliana G Ciobanu, Joao Conde, Vera Marisa Costa, Berihun Assefa Dachew, Saad M A Dahlawi, Gary L Darmstadt, Claudio Alberto Dávila-Cervantes, Adrian C Davis, Fernando Pio De la Hoz, Diego De Leo, Farah Deeba, Edgar Denova-Gutiérrez, Nikolaos Dervenis, Assefa Desalew, Samath Dhamminda Dharmaratne, Sameer Dhingra, Govinda Prasad Dhungana, Diana Dias da Silva, Daniel Diaz, Eleonora Dubljanin, Arielle Wilder Eagan, Sahar Eftekharzadeh, Iman El Sayed, Maha El Tantawi, Iffat Elbarazi, Islam Y Elgendy, Shaimaa I El-Jaafary, Oghenowede Eyawo, Sayeh Ezzikouri, Nelsensius Klau Fauk, Valery L Feigin, Tomas Y Ferede, Seyed-Mohammad Fereshtehnejad, Eduarda Fernandes, Pietro Ferrara, Irina Filip, Florian Fischer, Nataliya A Foigt, Morenike Oluwatoyin Folayan, Masoud Foroutan, Marisa Freitas, Takeshi Fukumoto, Mohamed M Gad, Shilpa Gaidhane, Santosh Gaihre, Silvano Gallus, Alberto L Garcia-Basteiro, Mariana Gaspar Fonseca, Lemma Getacher, Ahmad Ghashghaee, Giorgia Giussani, Elena V Gnedovskaya, Myron Anthony Godinho, Philimon N Gona, Sameer Vali Gopalani, Michal Grivna, Rafael Alves Guimarães, Rajat Das Gupta, Rajeev Gupta, Nima Hafezi-Nejad, Asif Hanif, Graeme J Hankey, Ahmed I Hasaballah, Amr Hassan, Rasmus J Havmoeller, Reza Heidari-Soureshjani, Claudiu Herteliu, Michael K Hole, Ramesh Holla, Naznin Hossain, Mostafa Hosseini, Sorin Hostiuc, Mowafa Househ, Junjie Huang, Ayesha Humayun, Ivo Iavicoli, Segun Emmanuel Ibitoye, Olayinka Stephen Ilesanmi, Irena M Ilic, Milena D Ilic, Sumant Inamdar, Sheikh Mohammed Shariful Islam, Hiroyasu Iso, Chidozie C D Iwu, Jalil Jaafari, Kathryn H Jacobsen, Vardhmaan Jain, Shubha Jayaram, Ensiyeh Jenabi, Ravi Prakash Jha, Oommen John, Jost B Jonas, Tamas Joo, Nitin Joseph, Jacek Jerzy Jozwiak, Mikk Jürisson, Ali Kabir, André Karch, Getinet Kassahun, Gbenga A Kayode, Rovshan Khalilov, Mohammad Khammarnia, Ejaz Ahmad Khan, Maseer Khan, Md Nuruzzaman Khan, Young-Ho Khang, Khaled Khatab, Mona M Khater, Young-Eun Kim, Yun Jin Kim, Adnan Kisa, Sezer Kisa, Niranjan Kissoon, Parvaiz A Koul, Sindhura Lakshmi Koulmane Laxminarayana, Ai Koyanagi, Kewal Krishan, Barthelemy Kuate Defo, Vaman Kulkarni, Manasi Kumar, Om P Kurmi, Dian Kusuma, Carlo La Vecchia, Ben Lacey, Ratilal Lalloo, Iván Landires, Anders O Larsson, Savita Lasrado, Paul H Lee, Matilde Leonardi, Shanshan Li, Lee-Ling Lim, Alan D Lopez, Rafael Lozano, Alessandra Lugo, Raimundas Lunevicius, Mark T Mackay, Shilpashree Madhava Kunjathur, Francesca Giulia Magnani, Azeem Majeed, Afshin Maleki, Reza Malekzadeh, Ahmad Azam Malik, Deborah Carvalho Malta, Abdullah A Mamun, Borhan Mansouri, Francisco Rogerlândio Martins-Melo, Seyedeh Zahra Masoumi, Pallab K Maulik, Colm McAlinden, John J McGrath, Carlo Eduardo Medina-Solís, Ziad A Memish, Walter Mendoza, Ritesh G Menezes, Endalkachew Worku Mengesha, Atte Meretoja, Tuomo J Meretoja, Tomislav Mestrovic, Tomasz Miazgowski, Irmina Maria Michalek, Ted R Miller, Babak Moazen, Masoud Moghadaszadeh, Bahram Mohajer, Osama Mohamad, Yousef Mohammad, Shafiu Mohammed, Ali H Mokdad, Mariam Molokhia, Lorenzo Monasta, Stefania Mondello, Mohammad Ali Moni, Catrin E Moore, Paula Moraga, Shane Douglas Morrison, Jonathan F Mosser, Amin Mousavi Khaneghah, Ghulam Mustafa, Ahamarshan Jayaraman Nagarajan, Shankar Prasad Nagaraju, Mohsen Naghavi, Behshad Naghshtabrizi, Mukhammad David Naimzada, Sreenivas Narasimha Swamy, Bruno Ramos Nascimento, Javad Nazari, Ionut Negoi, Ruxandra Irina Negoi, Samata Nepal, Georges Nguefack-

Tsague, Josephine W Ngunjiri, Cuong Tat Nguyen, Huong Lan Thi Nguyen, Molly R Nixon, Nurulamin M Noor, Jean Jacques Noubiap, Virginia Nuñez-Samudio, Vincent Ebuka Nwatah, Felix Akpojene Ogbo, Bolajoko Olubukunola Olusanya, Jacob Olusegun Olusanya, Obinna E Onwujekwe, Alberto Ortiz, Adrian Otoiu, Nikita Otstavnov, Stanislav S Otstavnov, Mayowa O Owolabi, Mahesh P A, Jagadish Rao Padubidri, Smita Pakhale, Raffaele Palladino, Adrian Pana, Songhomitra Panda-Jonas, Seithikurippu R Pandi-Perumal, Ana Melisa Pardo-Montaño, George C Patton, Shrikant Pawar, Hamidreza Pazoki Toroudi, Amy E Peden, Veincent Christian Filipino Pepito, Emmanuel K Peprah, Jeevan Pereira, Jorge Pérez-Gómez, Norberto Perico, Thomas Pilgrim, Michael A Piradov, James A Platts-Mills, Khem Narayan Pokhrel, Maarten J Postma, Sergio I Prada, Elisabetta Pupillo, Zahiruddin Quazi Syed, Amir Radfar, Ata Rafiee, Alireza Rafiei, Alberto Raggi, Mohammad Hifz Ur Rahman, Chhabi Lal Ranabhat, Sowmya J Rao, Davide Rasella, David Laith Rawaf, Salman Rawaf, Wasiq Faraz Rawasia, Giuseppe Remuzzi, Andre M N Renzaho, Bhageerathy Reshmi, Nima Rezaei, Seyed Mohammad Riahi, Daniela Ribeiro, Jennifer Rickard, Leonardo Roever, Luca Ronfani, Susan Fred Rumisha, Paul MacDaragh Ryan, Basema Saddik, Rajesh Sagar, Amirhossein Sahebkar, Mohammad Reza Salahshoor, Marwa Rashad Salem, Hamideh Salimzadeh, Joshua A Salomon, Yoseph Leonardo Samodra, Abdallah M Samy, Juan Sanabria, Milena M Santric-Milicevic, Abdur Razzaque Sarker, Nizal Sarrafzadegan, Arash Sarveazad, Thirunavukkarasu Sathish, Davide Sattin, Ganesh Kumar Saya, Mete Saylan, Silvia Schiavolin, David C Schwebel, Falk Schwendicke, Sadaf G Sepanlou, Saeed Shahabi, Izza Shahid, Ali S Shalash, Mehran Shams-Beyranvand, Sara Sheikhbahaei, Mika Shigematsu, Kerem Shuval, Tariq Jamal Siddiqi, Negussie Boti Sidemo, Inga Dora Sigfusdottir, Rannveig Sigurvinsdottir, João Pedro Silva, Jasvinder A Singh, Deepika Singhal, Dhirendra Narain Sinha, Eirini Skiadaresi, Valentin Yurievich Skryabin, Anna Aleksandrovna Skryabina, Joan B Soriano, Chandrashekhar T Sreeramareddy, Paschalis Steiropoulos, Mark A Stokes, Stefan Stortecky, Mu'awiyyah Babale Sufiyan, Gerhard Sulo, Carolyn B Swope, Bryan L Sykes, Miklós Szócska, Rafael Tabarés-Seisdedos, Eyayou Girma Tadesse, Animut Tagele Tamiru, Arash Tehrani-Banihashemi, Mohamad-Hani Temsah, Zemenu Tadesse Tessema, Kavumpurathu Raman Thankappan, Musliu Adetola Tolani, Marcos Roberto Tovani-Palone, Bach Xuan Tran, Jaya Prasad Tripathy, Aristidis Tsatsakis, Riaz Uddin, Anayat Ullah, Brigid Unim, Bhaskaran Unnikrishnan, Era Upadhyay, Muhammad Shariq Usman, Marco Vacante, Maryam Vaezi, Sahel Valadan Tahbaz, Tommi Juhani Vasankari, Narayanaswamy Venketasubramanian, Madhur Verma, Francesco S Violante, Vasily Vlassov, Giang Thu Vu, Yohannes Dibaba Wado, Yasir Waheed, Richard G Wamai, Yanzhong Wang, Yuan-Pang Wang, Paul Ward, Andrea Werdecker, Ronny Westerman, Nuwan Darshana Wickramasinghe, Charles Shey Wiysonge, Ai-Min Wu, Seyed Hossein Yahyazadeh Jabbari, Kazumasa Yamagishi, Srikanth Yandrapalli, Sanni Yaya, Vahid Yazdi-Feyzabadi, Naohiro Yonemoto, Seok-Jun Yoon, Sifat Shahana Yusuf, Sojib Bin Zaman, Maryam Zamanian, Mikhail Sergeevich Zastrozhin, Anasthasia Zastrozhina, Xiu-Ju George Zhao, Arash Ziapour, Simon I Hay, Haidong Wang, and Nicholas J Kassebaum.

Extracting, cleaning, or cataloging data; designing or coding figures and tables

Katherine R Paulson, Hesam Alizade, Saeed Amini, Takeshi Fukumoto, Shilpa Gaidhane, Kevin S Ikuta, Jalil Jaafari, Ensiyeh Jenabi, Laura Kemmer, Rovshan Khalilov, Young-Eun Kim, Borhan Mansouri, Masoud Moghadaszadeh, Ali H Mokdad, Mohsen Naghavi, Behshad Naghshtabrizi, Javad Nazari, Josephine W Ngunjiri, Hamidreza Pazoki Toroudi, Zahiruddin Quazi Syed, Nima

Rezaei, Seyed Mohammad Riahi, Enrico Rubagotti, Mohammad Reza Salahshoor, Abdallah M Samy, Ganesh Kumar Saya, Negussie Boti Sidemo, Deepika Singhal, Emma Elizabeth Spurlock, Maryam Vaezi, Seok-Jun Yoon, Mikhail Sergeevich Zastrozhin, Anasthasia Zastrozhina, Arash Ziapour, Haidong Wang, and Nicholas J Kassebaum.

Managing the overall research enterprise

Katherine R Paulson, Tahiya Alam, Kelly Bienhoff, Lalit Dandona, Adrian C Davis, Ali H Mokdad, Mohsen Naghavi, Molly R Nixon, George C Patton, Joshua A Salomon, Simon I Hay, Christopher J L Murray, Haidong Wang, and Nicholas J Kassebaum.

References

1. Wang H, Abbas KM, Abbasifard M, et al. Global, regional, and national age-sex-specific fertility, mortality, healthy life expectancy (HALE), and population estimates in 204 countries and territories, 1950–2019: a comprehensive demographic analysis for the Global Burden of Disease Study 2019. The Lancet 2020; 396: 1160-203
2. Rajaratnam JK, Tran LN, Lopez AD, Murray CJL. Measuring under-five mortality: validation of new low-cost methods. PLOS Med 2010; 7: e1000253.
3. Dicker D, Nguyen G, Abate D, et al. Global, regional, and national age-sex-specific mortality and life expectancy, 1950–2017: a systematic analysis for the Global Burden of Disease Study 2017. The Lancet 2018; 392: 1684-735.
4. The UN inter-agency group for child mortality estimation. Child Mortality Estimates. http://www.childmortality.org/.(accessed May 28, 2020).
5. Wang H, Abajobir AA, Abate KH, et al. Global, regional, and national under-5 mortality, adult mortality, age-specific mortality, and life expectancy, 1970–2016: a systematic analysis for the Global Burden of Disease Study 2016. The Lancet 2017; 390: 1084–150.
6. Brocklehurst P, French R. The association between maternal HIV infection and perinatal outcome: a systematic review of the literature and meta-analysis. Br J Obstet Gynaecol 1998; 105: 836–48.
7. Kim H-Y, Kasonde P, Mwiya M, et al. Pregnancy loss and role of infant HIV status on perinatal mortality among HIV-infected women. BMC Pediatr 2012; 12: 138.
8. Kerber KJ, Lawn JE, Johnson LF, et al. South African child deaths 1990-2011: have HIV services reversed the trend enough to meet Millennium Development Goal 4? AIDS Lond Engl 2013; 27: 2637–48.
9. Bradshaw D, Dorrington R. Child mortality in South Africa - we have lost touch. S Afr Med J 2007; 97: 582.
10. Roth GA, Abate D, Abate KH, et al. Global, regional, and national age-sex-specific mortality for 282 causes of death in 195 countries and territories, 1980–2017: a systematic analysis for the Global Burden of Disease Study 2017. The Lancet 2018; 392: 1736-788.
11. Wang H, Wolock TM, Carter A, et al. Estimates of global, regional, and national incidence, prevalence, and mortality of HIV, 1980–2015: the Global Burden of Disease Study 2015. Lancet HIV 2016; 3: e361–87.
12. Department of Peace and Conflict Research, Uppsala University. UCDP georeferenced event dataset, version 17.1, 2016. Uppsala, Sweden: Department of Peace and Conflict Research, Uppsala University, 2017.

13. Obermeyer Z, Murray CJL, Gakidou E. Fifty years of violent war deaths from Vietnam to Bosnia: analysis of data from the world health survey programme. BMJ 2008; 336: 1482–6.
14. Milton Leitenberg, 'Rwanda, 1994: International Incompetence produces Genocide,' Peacekeeping and International Relations, November/December 1994.
15. Jarvis C. The rise and fall of Albania's pyramid schemes. Finance Dev 2000; 37. http://www.imf.org/external/pubs/ft/fandd/2000/03/jarvis.htm. (accessed May 28, 2020).
16. Centre for Research on the Epidemiology of Disasters (CRED). EM-DAT: The OFDA/CRED International Disaster Database. Brussels, Belgium: Catholic University of Leuven.
17. Jisheng Y, Friedman E, Guo J, Mosher S. Tombstone: the great Chinese famine, 1958-1962. New York: Farrar, Straus and Giroux (Macmillan), 2012.
18. Inc GI, Berger DS. Bacterial meningitis: global status: 2017 edition. GIDEON Informatics Inc, 2017.
19. Inc GI, Berger DS. Cholera: global status: 2017 edition. GIDEON Informatics Inc, 2017.
20. Iraq Body Count. https://www.iraqbodycount.org/database/ (accessed May 28, 2020).
21. Hagopian A, Flaxman AD, Takaro TK, et al. Mortality in Iraq associated with the 2003–2011 war and occupation: findings from a national cluster sample survey by the University Collaborative Iraq Mortality Study. PLOS Med 2013; 10: e1001533.
22. Sankoh O, Byass P. The INDEPTH Network: filling vital gaps in global epidemiology. Int J Epidemiol 2012; 41: 579–88.
23. James SL, Abate D, Abate KH, et al. Global, regional, and national incidence, prevalence, and years lived with disability for 354 diseases and injuries for 195 countries and territories, 1990–2017: a systematic analysis for the Global Burden of Disease Study 2017. The Lancet 2018; 392: 1789–858.
24. Office of the Registrar General and Census Commissioner. India Medical Certification of Cause of Death Reports 1990-2010. India, 2014.
25. Office of the Registrar General and Census Commissione. India Sample Registration System Statistical Report 2017. India, 2018.
26. Todd S, Barr S, Passmore AP. Cause of death in Alzheimer's disease: a cohort study. QJM Mon J Assoc Physicians 2013; 106: 747–53.
27. Brunnström HR, Englund EM. Cause of death in patients with dementia disorders. Eur J Neurol 2009; 16: 488–92.
28. Keene J, Hope T, Fairburn CG, Jacoby R. Death and dementia. Int J Geriatr Psychiatry 2001; 16: 969–74.
29. Thomas BM, Starr JM, Whalley LJ. Death certification in treated cases of presenile Alzheimer's disease and vascular dementia in Scotland. Age Ageing 1997; 26: 401–6.
30. Naghavi M, Makela S, Foreman K, O'Brien J, Pourmalek F, Lozano R. Algorithms for enhancing public health utility of national causes-of-death data. Popul Health Metr 2010; 8: 9.
31. Barker B, Degenhardt L, National Drug and Alcohol Research Centre (Australia). Accidental drug-induced deaths in Australia 1997-2001. Sydney, Australia: National Drug and Alcohol Research Centre, University of New South Wales, 2003.
32. Roxburgh A, Burns L. Accidental drug-induced deaths due to opioids in Australia, 2011. Sydney, Australia: National Drug and Alcohol Research Centre, University of New South Wales, 2015.
33. Bell RM, Koren Y, Volinsky C. All Together Now: A Perspective on the Netflix Prize. CHANCE 2010; 23: 24–9.
34. Lozano R, Naghavi M, Foreman K, et al. Global and regional mortality from 235 causes of death for 20 age groups in 1990 and 2010: a systematic analysis for the Global Burden of Disease Study 2010. The Lancet 2012; 380: 2095–128.

35. Calvert C, Ronsmans C. The contribution of HIV to pregnancy-related mortality: a systematic review and meta-analysis. Aids 2013; 27: 1631–9.
36. Figueroa-Damián R. Pregnancy outcome in women infected with the human immunodeficiency virus. Salud Publica Mex 1999; 41: 362–7.
37. Ryder RW, Nsuami M, Nsa W, et al. Mortality in HIV-1-seropositive women, their spouses and their newly born children during 36 months of follow-up in Kinshasa, Zaïre. AIDS Lond Engl 1994; 8: 667–72.
38. Zvandasara P, Saungweme G, Mlambo JT, Moyo J. Post Caesarean section infective morbidity in HIV- positive women at a tertiary training hospital in Zimbabwe. Cent Afr J Med 2007; 53: 43–7.
39. Chilongozi D, Wang L, Brown L, et al. Morbidity and mortality among a cohort of human immunodeficiency virus type 1-infected and uninfected pregnant women and their infants from Malawi, Zambia, and Tanzania. Pediatr Infect Dis J 2008; 27: 808–14.
40. Leroy V, Ladner J, Nyiraziraje M, et al. Effect of HIV-1 infection on pregnancy outcome in women in Kigali, Rwanda, 1992-1994. Pregnancy and HIV Study Group. AIDS 1998; 12: 643–50.
41. Kourtis A, Bansil P, McPheeters M, Meikle S, Posner S, Jamieson D. Hospitalizations of pregnant HIV- infected women in the USA prior to and during the era of HAART, 1994–2003. AIDS 2006; 20: 1823–31.
42. Ticconi C, Mapfumo M, Dorrucci M, et al. Effect of Maternal HIV and Malaria Infection on Pregnancy and Perinatal Outcome in Zimbabwe. Jaids J Acquir Immune Defic Syndr 2003; 34: 289–94.
43. Brown T, Peerapatanapokin W. The Asian Epidemic Model: a process model for exploring HIV policy and programme alternatives in Asia. Sex Transm Infect 2004; 80: i19–24.
44. DerSimonian R, Laird N. Meta-analysis in clinical trials. Control Clin Trials 1986; 7: 177–88.
45. Lozano R, Lopez AD, Atkinson C, Naghavi M, Flaxman AD, Murray CJ. Performance of physician-certified verbal autopsies: multisite validation study using clinical diagnostic gold standards. Popul Health Metr 2011; 9: 32.
46. Foreman KJ, Lozano R, Lopez AD, Murray CJ. Modelling causes of death: an integrated approach using CODEm. Popul Health Metr 2012; 10: 1.
47. Bell RM, Koren Y. Lessons from the Netflix Prize Challenge. SIGKDD Explor Newsl 2007; 9: 75–79.
48. Flaxman AD, Vos T, Murray CJL, Kiyono P, editors. An integrative metaregression framework for descriptive epidemiology, 1 edition. Seattle: University of Washington Press, 2015.
49. Murray CJL, Callender CSKH, Kulikoff XR, et al. Population and fertility by age and sex for 195 countries and territories, 1950–2017: a systematic analysis for the Global Burden of Disease Study 2017. Lancet 2018; 392: 1995–2051.
50. Fullman N, Yearwood J, Abay SM, Abbafati C, Abd-Allah F, Abdela J, et al. Measuring performance on the Healthcare Access and Quality Index for 195 countries and territories and selected subnational locations: a systematic analysis from the Global Burden of Disease Study 2016. The Lancet. 2018 Jun;391(10136):2236–71.

Appendix: Stochastic Frontier Meta-Analysis (SFM)

1. Stochastic Frontier Meta-Analysis (SFM)

Stochastic frontier analysis (SFA) [1] is a stochastic analysis of the frontier production function, which expresses the maximum amount of output obtainable from a linear combination of variables of interest. The SFA model we start with is given by

$$y_i = \langle \boldsymbol{x}_i, \boldsymbol{\beta} \rangle - v_i, \tag{1}$$

where y_i are observations, $\langle \boldsymbol{x}_i, \boldsymbol{\beta} \rangle$ is the linear model (linear combination of variables in $\boldsymbol{x}_i$ with weights $\boldsymbol{\beta}$), while v_i is the deviation either from the *maximum output*, modeled as a *non-negative* random effect, or *minimum output*, modeled as a *non-positve* random effect. We focus on deviations from the maximum below, with the analysis for deviation from minimum completely analogous.

This appendix gives technical details for the Stochastic Frontier Meta-analysis (SFM) extension. Every observation y_i is subject to random error (computed from aggregated data). We consider the model

$$\boldsymbol{y} = \boldsymbol{X}\boldsymbol{\beta}^* - \boldsymbol{v} + \boldsymbol{\epsilon}, \tag{2}$$

with each entry v_i of $\boldsymbol{v}$ a half-normal non-negative random effect with unknown variance η, while each entry ϵ_i of $\boldsymbol{\epsilon}$ is Gaussian $\mathcal{N}(0, \sigma_i^2)$, and represents the reported study-specific error sources with known variances σ_i^2.

Roadmap. The remainder of the Appendix proceeds as follows. In Section 2 we formulate the likelihood problem for the SFM model, assuming a half-normal model for the non-negative random effects v_i. In Section 3, we describe spline models, as well as how to impose priors and constraints on the estimation parameters. Finally in Section 4 we discuss the robust trimming extension that is used to guard against outliers. The fitting procedure is detailed in Section 5, and closed form solutions for the inefficiencies are given in Seciton 6.

2. SFM: modeling non-negative random effects.

In this section we derive all likelihood formulations for the Stochastic Frontier Meta-analysis (SFM) approach. We use the half-normal model for the random effects v_i:

$$f(v_i|\eta) = \begin{cases} \frac{\sqrt{2}}{\sqrt{\pi\eta}} \exp\left(-\frac{v_i^2}{2\eta}\right) & v_i \geq 0 \\ 0 & v_i < 0. \end{cases}$$

The goal is to estimate $\boldsymbol{\beta}^*$ and η^* from observations. The mixed effects framework provides a natural statistical model which can be used for this inference. The joint distribution of fixed and random effects is then given by

$$\begin{aligned} p(\boldsymbol{\beta}, \eta, \boldsymbol{v}|\boldsymbol{y}) &= p(\boldsymbol{\beta}, \eta, |\boldsymbol{v}, \boldsymbol{y}) p(\boldsymbol{v}|\boldsymbol{y}) \\ &\propto \prod_{i=1}^{m} \frac{1}{\sqrt{\sigma_i^2 \eta}} \exp\left(-\frac{(y_i - \langle \boldsymbol{x}_i, \boldsymbol{\beta} \rangle + v_i)^2}{2\sigma_i^2}\right) \exp\left(-\frac{v_i^2}{2\eta}\right) \mathbb{1}_{\mathbb{R}_+}(v_i) \end{aligned} \tag{3}$$

Integrating out the random effects, and taking the negative log of the resulting distribution, we arrive at equivalent maximum likelihood formulation that does not depend on the random effects $\boldsymbol{v}$, but only depends on $\boldsymbol{\beta}$ and η. Define $\widetilde{\Phi}$ to be the complementary error function

$$\widetilde{\Phi}(z) = 1 - \frac{2}{\sqrt{\pi}} \int_0^z \exp(-t^2) dt.$$

Then we have the following closed form likelihood.

$$\begin{aligned}\mathcal{M}(\boldsymbol{\beta}, \eta|\boldsymbol{y}) &= -\ln\left(\int_{\mathbb{R}_+^m} p(\boldsymbol{\beta}, \eta, \boldsymbol{v}|\boldsymbol{y})\, d\boldsymbol{v}\right) \\ &= \sum_{i=1}^{m} \frac{(y_i - \langle \boldsymbol{x}_i, \boldsymbol{\beta}\rangle)^2}{2(\eta + \sigma_i^2)} + \frac{1}{2}\ln\left(\eta + \sigma_i^2\right) - \ln\left(\widetilde{\Phi}\left(\frac{\sqrt{\eta}(y_i - \langle \boldsymbol{x}_i, \boldsymbol{\beta}\rangle)}{\sqrt{2(\eta + \sigma_i^2)(\sigma_i^2)}}\right)\right)\end{aligned} \tag{4}$$

The SFM approach optimizes these likelihoods to estimate $(\boldsymbol{\beta}, \eta)$.

3. Priors, Constraints, and Splines.

In this section we describe how to set up Bayesian priors, constraints for parameters of interest, and spline models for nonlinear relationships in the SFM setup.

3.1. Priors

The likelihood $\mathcal{M}$ can be updated using prior information. Imposing priors is equivalent to adding penalties to the likelihood function. For the SFM analysis, the only priors we use are those related to the final section of the frontier.

Given a Gaussian prior on $\boldsymbol{\beta} \sim N(\overline{\boldsymbol{\beta}})$, we find the *a posteriori* estimate by solving the problem

$$\min_{\boldsymbol{\beta}, \eta} \mathcal{M}(\boldsymbol{\beta}, \eta) + \frac{1}{2}(\boldsymbol{\beta} - \overline{\boldsymbol{\beta}})^T \boldsymbol{\Sigma}_{\boldsymbol{\beta}}^{-1}(\boldsymbol{\beta} - \overline{\boldsymbol{\beta}}). \tag{5}$$

3.2. Constraints

We allow box constraints and general linear inequality constraints on $(\boldsymbol{\beta}, \eta)$. Taking (5) as a running example, we can impose constraints of the form

$$\begin{aligned}\min_{\boldsymbol{\beta}, \eta} \quad & \mathcal{M}(\boldsymbol{\beta}, \eta) + \frac{1}{2}(\boldsymbol{\beta} - \overline{\boldsymbol{\beta}})^T \boldsymbol{\Sigma}_{\boldsymbol{\beta}}^{-1}(\boldsymbol{\beta} - \overline{\boldsymbol{\beta}}) + \frac{1}{2}(\eta - \overline{\eta})^T \boldsymbol{\Sigma}_{\eta}^{-1}(\eta - \overline{\eta}) \\ \text{such that} \quad & \boldsymbol{l}_f \leq \begin{bmatrix}\boldsymbol{\beta} \\ \eta\end{bmatrix} \leq \boldsymbol{u}_f, \quad \boldsymbol{C}\begin{bmatrix}\boldsymbol{\beta} \\ \eta\end{bmatrix} \leq \boldsymbol{c},\end{aligned} \tag{6}$$

where $(\boldsymbol{l}_f, \boldsymbol{u}_f)$ are lower and upper bounds on the variables, while $\boldsymbol{C}$ is any matrix. This functionality can be used to impose shape constraints on spline models, including increasing/decreasing, convex/concave, and combinations of these designs.

3.3. Splines

In this section we discuss spline models for dose-response relationships. For general background on splines and spline regression see [5] and [6].

B-splines and bases. A spline basis is a set of piecewise polynomial functions with designated degree and domain. If we denote polynomial order by p, and the number of knots by k, we need $p + k$ basis elements s_j^p, which can be generated recursively as illustrated in Figure 1.

Given such a basis, we can represent any dose-response relationship as the linear combination of the spline basis elements, with coefficients $\boldsymbol{\beta} \in \mathbb{R}^{p+k}$:

$$f(t) = \sum_{j=1}^{p+k} \beta_j^p s_j^p(t). \tag{7}$$

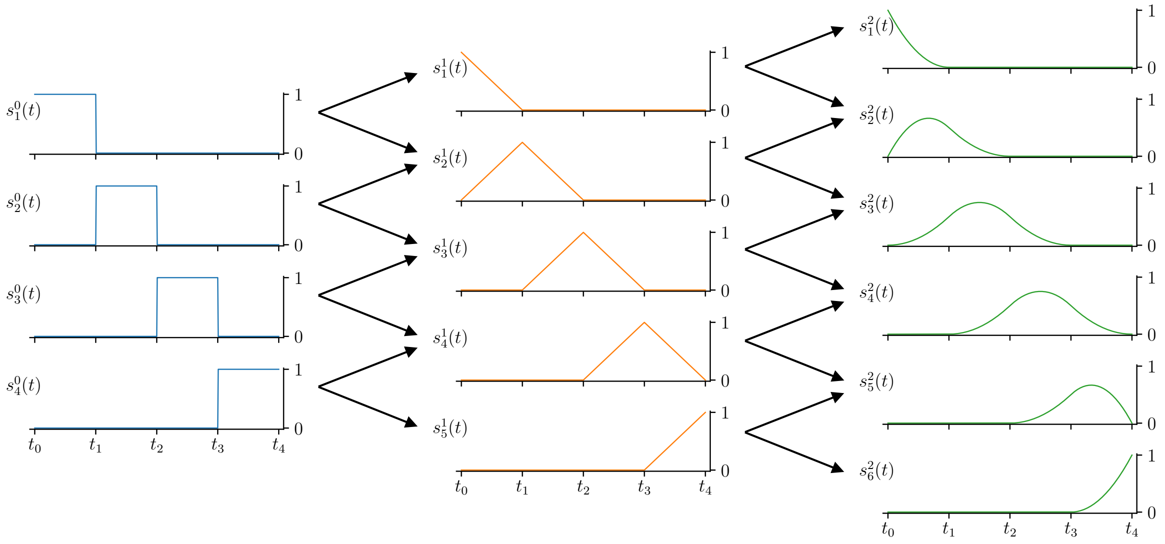

Figure 1. Recursive generation of bspline basis elements (orders 0, 1, 2).

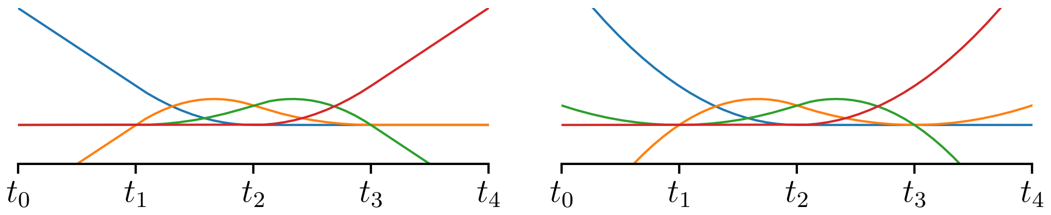

Figure 2. Spline extrapolation. Left: linear extrapolation. Right: nonlinear extrapolation.

An explicit representation of (7) is obtained by building a design matrix $\mathbf{X}$. Given a set of t values at which we have data, the jth column of $\mathbf{X}$ is given by the expression

$$\mathbf{X}_{\cdot,j} = \begin{bmatrix} s_j^p(t_0) \\ \vdots \\ s_j^p(t_k) \end{bmatrix}.$$

The model for direct observations data coming from the spline (7) can now be written compactly as

$$\boldsymbol{y} = \mathbf{X}\boldsymbol{\beta} + \boldsymbol{v} + \boldsymbol{\epsilon}_i,$$

and has the same form as (1).

Enforcing linear tails. For the frontier analysis, we need to ensure that the last segment of the spline does not go above a theoretical limit, typically set at 1. To do this, we allow an option to make the last segment linear. The prior capabilities can then be used to set a prior for the slope of this segment to be 0 (i.e. flat). The estimated spline is then a best fit to the data, subject to this specification.

In general, using linear head and/or tail pieces to extrapolate outside the original domain or interpolate in the data sparse region is far more stable that using higher order polynomials, see Figure 2. The figure shows symmetric linear tail modifications, but for the analyses in the paper we only impose a right linear tail shape constraint.

Shape constraints. We can use constraints to enforce monotonicity, convexity, and concavity. Monotonicity across the domain of interest follows from monotonicity of the spline coefficients. This relationship is derived for particular basis constructions by [5], and has been used in the literature to enforce shape constraints [8]. Current approaches work around the natural inequality constraints by using additional 'exponentiated' variables. Instead we impose these constraints directly as described below.

Focusing just on α, the relationship $\alpha_1 \leq \alpha_2$ can be written as $\alpha_1 - \alpha_2 \leq 0$. Stacking these inequality constraints for each pair (α_i, α_{i+1}) we can write all constraints simultaneously as

$$\underbrace{\begin{bmatrix} 1 & -1 & 0 & \dots & 0 \\ 0 & 1 & -1 & \dots & 0 \\ \ddots & \ddots & \ddots & \ddots & \vdots \\ 0 & \dots & \dots & 1 & -1 \end{bmatrix}}_{\boldsymbol{C}} \begin{bmatrix} \alpha_1 \\ \alpha_2 \\ \alpha_3 \\ \vdots \\ \alpha_n \end{bmatrix} \leq \begin{bmatrix} 0 \\ 0 \\ \vdots \\ 0 \end{bmatrix}.$$

These constraints are directly imposed through the IPOPT interface, along with any lower- and upper-limit constraints on α.

Convexity and Concavity. For any $\mathcal{C}^2$ (twice continuously differentiable) function $f : \mathbb{R} \to \mathbb{R}$, convexity and concavity are captured by the signs of the second derivative. Specifically, f is convex if $f''(t) \geq 0$ is everywhere, an concave if $f''(t) \leq 0$ everywhere. We impose linear inequality constraints on the expressions for $f''(t)$ over each interval. We can therefore easily pick any of the eight shape combinations given in [8, Table 1], as well as imposing any other constraints on α (including bounds).

4. Robust Extension via Trimming

Trimming estimators is a general methodology for robust estimation [10, 2]. For convenience, define

$$\boldsymbol{\theta} = \begin{bmatrix} \beta \\ \eta \end{bmatrix}.$$

Given any likelihood problem of form

$$\min_{\boldsymbol{\theta}} \sum_{i=1}^{m} f_i(\boldsymbol{\theta}) + R(\boldsymbol{\theta}),$$

with f_i is the contribution from the ith datapoint, while $R(\boldsymbol{\theta})$ collects all terms that do not depend on the data, including priors in Section 3.1 and constraints in Section 3.2 [1].

Then the trimmed estimator is formulated as

$$\min_{\boldsymbol{\theta},\boldsymbol{w}} \sum_{i=1}^{m} w_i f_i(\boldsymbol{\theta}) + R(\boldsymbol{\theta}), \quad 0 \leq w_i \leq 1, \quad \mathbf{1}^T \boldsymbol{w} = h \tag{8}$$

where $h \leq m$ is the estimate of inlier datapoints. The set

$$\Delta_h := \left\{\boldsymbol{w} : 0 \leq w_i \leq 1, \mathbf{1}^T \boldsymbol{w} = h\right\}$$

is known as the *capped simplex*, since it is the intersection of the simplex with the unit box [2]. The estimator (8) is compactly written as

$$\min_{\boldsymbol{\theta},\boldsymbol{w}\in\Delta_h} \sum_{i=1}^{m} w_i f_i(\boldsymbol{\theta}) + R(\boldsymbol{\theta}). \tag{9}$$

[1] In particular, since R includes constraints, it is infinite-valued off of the feasible region.

5. Optimization

The SFM model is fit using an algorithm based on variable projection [4, 7, 3], which allows us to leverage a third-party solver, IPOPT [9] to optimize over $\boldsymbol{\theta}$, significantly reducing complexity. In particular, defining the value function $v(\boldsymbol{w})$ by

$$v(\boldsymbol{w}) = \min_{\boldsymbol{\theta}} \sum_{i=1}^{m} w_i f_i(\boldsymbol{\theta}) + R(\boldsymbol{\theta}), \tag{10}$$

the top level algorithm is simply a projected gradient method to solve

$$\min_{\boldsymbol{w} \in \Delta_h} v(\boldsymbol{w}).$$

The projection onto the capped simplex is detailed in [2].

6. Estimating Random Effects (Inefficiencies).

Once fixed effects $\boldsymbol{\theta}$ have been estimated, we want to obtain estimates of inefficiency from the joint likelihood (3). We optimize

$$\min_{v_i \geq 0} \frac{(y_i - \langle \boldsymbol{x}_i, \boldsymbol{\beta} \rangle + v_i)^2}{2\sigma_i^2} + \frac{v_i^2}{2\eta} \tag{11}$$

We get the closed form solution

$$\hat{v}_i = \max\left(0, \frac{\frac{1}{\sigma_i^2}(\boldsymbol{x}_i^T \boldsymbol{\beta} - y_i)}{\frac{1}{\sigma_i^2} + \frac{1}{\eta}}\right) = \max\left(0, \frac{\eta(\boldsymbol{x}_i^T \boldsymbol{\beta} - y_i)}{\sigma_i^2 + \eta}\right). \tag{12}$$

References

[1] D. Aigner, C. K. Lovell, and P. Schmidt. Formulation and estimation of stochastic frontier production function models. *Journal of econometrics*, 6(1):21–37, 1977.

[2] A. Aravkin and D. Davis. Trimmed statistical estimation via variance reduction. *Mathematics of Optimization Research*, 2019.

[3] A. Y. Aravkin and T. Van Leeuwen. Estimating nuisance parameters in inverse problems. *Inverse Problems*, 28(11):115016, 2012.

[4] B. M. Bell, J. V. Burke, and A. Schumitzky. A relative weighting method for estimating parameters and variances in multiple data sets. *Computational statistics & data analysis*, 22(2):119–135, 1996.

[5] C. De Boor, C. De Boor, E.-U. Mathématicien, C. De Boor, and C. De Boor. *A practical guide to splines*, volume 27. springer-verlag New York, 1978.

[6] J. H. Friedman. Multivariate adaptive regression splines. *The annals of statistics*, pages 1–67, 1991.

[7] G. Golub and V. Pereyra. Separable nonlinear least squares: the variable projection method and its applications. *Inverse problems*, 19(2):R1, 2003.

[8] N. Pya and S. N. Wood. Shape constrained additive models. *Statistics and Computing*, 25(3):543–559, 2015.

[9] A. Wächter and L. T. Biegler. On the implementation of an interior-point filter line-search algorithm for large-scale nonlinear programming. *Mathematical programming*, 106(1):25–57, 2006.

[10] E. Yang, A. C. Lozano, and A. Aravkin. A general family of trimmed estimators for robust high-dimensional data analysis. *Electronic Journal of Statistics*, 12(2):3519–3553, 2018.

Table S6. GBD complete cause hierarchy with associated metadata

Cause	Hierarchy	Level	Most detailed	Male	Female	Nonfatal only	Age start	Age end
**All causes**	**Total**	**0**	**0**	**1**	**1**	**0**	**0**	**95**
**Communicable, maternal, neonatal, and nutritional disorders**	**A**	**1**	**0**	**1**	**1**	**0**	**0**	**95**
HIV/AIDS and sexually transmitted infections	A.1	2	0	1	1	0	0	95
HIV/AIDS	A.1.1	3	0	1	1	0	0.1	95
HIV/AIDS - Drug-susceptible Tuberculosis	A.1.1.1	4	1	1	1	0	0.1	95
HIV/AIDS - Multidrug-resistant Tuberculosis without extensive drug resistance	A.1.1.2	4	1	1	1	0	0.1	95
HIV/AIDS - Extensively drug-resistant Tuberculosis	A.1.1.3	4	1	1	1	0	0.1	95
HIV/AIDS resulting in other diseases	A.1.1.4	4	1	1	1	0	0.1	95
Sexually transmitted infections excluding HIV	A.1.2	3	0	1	1	0	0	95
Syphilis	A.1.2.1	4	1	1	1	0	0	95
Chlamydial infection	A.1.2.2	4	1	1	1	0	10	95
Gonococcal infection	A.1.2.3	4	1	1	1	0	10	95
Trichomoniasis	A.1.2.4	4	1	1	1	1	10	95
Genital herpes	A.1.2.5	4	1	1	1	1	0	95
Other sexually transmitted infections	A.1.2.6	4	1	1	1	0	10	95
Respiratory infections and tuberculosis	A.2	2	0	1	1	0	0.01	95
Tuberculosis	A.2.1	3	0	1	1	0	0.1	95
Latent tuberculosis infection	A.2.1.1	4	1	1	1	1	0	95
Drug-susceptible tuberculosis	A.2.1.2	4	1	1	1	0	0.1	95
Multidrug-resistant tuberculosis without extensive drug resistance	A.2.1.3	4	1	1	1	0	0.1	95
Extensively drug-resistant tuberculosis	A.2.1.4	4	1	1	1	0	0.1	95
Lower respiratory infections	A.2.2	3	1	1	1	0	0	95
Upper respiratory infections	A.2.3	3	1	1	1	0	0	95
Otitis media	A.2.4	3	1	1	1	0	0	95
Enteric infections	A.3	2	0	1	1	0	0	95
Diarrhoeal diseases	A.3.1	3	1	1	1	0	0	95
Typhoid and paratyphoid	A.3.2	3	0	1	1	0	0.1	95
Typhoid fever	A.3.2.1	4	1	1	1	0	0.1	95
Paratyphoid fever	A.3.2.2	4	1	1	1	0	0.1	95
iNTS	A.3.3	3	1	1	1	0	0.01	95
Other intestinal infectious diseases	A.3.4	3	1	1	1	0	0.1	95
Neglected tropical diseases and malaria	A.4	2	0	1	1	0	0	95
Malaria	A.4.1	3	1	1	1	0	0	95
Chagas disease	A.4.2	3	1	1	1	0	0.1	95
Leishmaniasis	A.4.3	3	0	1	1	0	0.1	95
Visceral leishmaniasis	A.4.3.1	4	1	1	1	0	0.1	95
Cutaneous and mucocutaneous leishmaniasis	A.4.3.2	4	1	1	1	1	0.1	95
African trypanosomiasis	A.4.4	3	1	1	1	0	1	95
Schistosomiasis	A.4.5	3	1	1	1	0	0.1	95
Cysticercosis	A.4.6	3	1	1	1	0	1	95
Cystic echinococcosis	A.4.7	3	1	1	1	0	1	95
Lymphatic filariasis	A.4.8	3	1	1	1	1	1	95
Onchocerciasis	A.4.9	3	1	1	1	1	1	95
Trachoma	A.4.10	3	1	1	1	1	15	95
Dengue	A.4.11	3	1	1	1	0	0.01	95
Yellow fever	A.4.12	3	1	1	1	0	0.01	95
Rabies	A.4.13	3	1	1	1	0	0.1	95
Intestinal nematode infections	A.4.14	3	0	1	1	0	0.1	95
Ascariasis	A.4.14.1	4	1	1	1	0	0.1	95
Trichuriasis	A.4.14.2	4	1	1	1	1	0.1	95
Hookworm disease	A.4.14.3	4	1	1	1	1	0.1	95
Food-borne trematodiases	A.4.15	3	1	1	1	1	1	95
Leprosy	A.4.16	3	1	1	1	1	1	95
Ebola virus disease	A.4.17	3	1	1	1	0	0	95
Zika virus disease	A.4.18	3	1	1	1	0	0	95
Guinea worm disease	A.4.19	3	1	1	1	1	1	95
Other neglected tropical diseases	A.4.20	3	1	1	1	0	0	95
Other infectious diseases	A.5	2	0	1	1	0	0	95
Meningitis	A.5.1	3	1	1	1	0	0	95
Encephalitis	A.5.2	3	1	1	1	0	0	95
Diphtheria	A.5.3	3	1	1	1	0	0.1	55
Whooping cough	A.5.4	3	1	1	1	0	0.1	55

Tetanus	A.5.5	3	1	1	1	0	0	95
Measles	A.5.6	3	1	1	1	0	0.1	55
Varicella and herpes zoster	A.5.7	3	1	1	1	0	0	95
Acute hepatitis	A.5.8	3	0	1	1	0	0.1	95
Acute hepatitis A	A.5.8.1	4	1	1	1	0	0.1	95
Acute hepatitis B	A.5.8.2	4	1	1	1	0	0.1	95
Acute hepatitis C	A.5.8.3	4	1	1	1	0	0.1	95
Acute hepatitis E	A.5.8.4	4	1	1	1	0	0.1	95
Other unspecified infectious diseases	A.5.9	3	1	1	1	0	0	95
Maternal and neonatal disorders	A.6	2	0	1	1	0	0	50
Maternal disorders	A.6.1	3	0	0	1	0	10	50
Maternal haemorrhage	A.6.1.1	4	1	0	1	0	10	50
Maternal sepsis and other pregnancy related infections	A.6.1.2	4	1	0	1	0	10	50
Maternal hypertensive disorders	A.6.1.3	4	1	0	1	0	10	50
Maternal obstructed labour and uterine rupture	A.6.1.4	4	1	0	1	0	10	50
Maternal abortive outcome	A.6.1.5	4	1	0	1	0	10	50
Ectopic pregnancy	A.6.1.6	4	1	0	1	0	10	50
Indirect maternal deaths	A.6.1.7	4	1	0	1	0	10	50
Late maternal deaths	A.6.1.8	4	1	0	1	0	10	50
Maternal deaths aggravated by HIV/AIDS	A.6.1.9	4	1	0	1	0	10	50
Other maternal disorders	A.6.1.10	4	1	0	1	0	10	50
Neonatal disorders	A.6.2	3	0	1	1	0	0	1
Neonatal preterm birth	A.6.2.1	4	1	1	1	0	0	1
Neonatal encephalopathy due to birth asphyxia and trauma	A.6.2.2	4	1	1	1	0	0	1
Neonatal sepsis and other neonatal infections	A.6.2.3	4	1	1	1	0	0	1
Hemolytic disease and other neonatal jaundice	A.6.2.4	4	1	1	1	0	0	1
Other neonatal disorders	A.6.2.5	4	1	1	1	0	0	1
Nutritional deficiencies	A.7	2	0	1	1	0	0.1	95
Protein-energy malnutrition	A.7.1	3	1	1	1	0	0.1	95
Iodine deficiency	A.7.2	3	1	1	1	1	0	95
Vitamin A deficiency	A.7.3	3	1	1	1	1	0	95
Dietary iron deficiency	A.7.4	3	1	1	1	1	0	95
Other nutritional deficiencies	A.7.5	3	1	1	1	0	0.1	95
**Non-communicable diseases**	**B**	**1**	**0**	**1**	**1**	**0**	**0**	**95**
Neoplasms	B.1	2	0	1	1	0	0	95
Lip and oral cavity cancer	B.1.1	3	1	1	1	0	5	95
Nasopharynx cancer	B.1.2	3	1	1	1	0	5	95
Other pharynx cancer	B.1.3	3	1	1	1	0	20	95
Oesophageal cancer	B.1.4	3	1	1	1	0	20	95
Stomach cancer	B.1.5	3	1	1	1	0	15	95
Colon and rectum cancer	B.1.6	3	1	1	1	0	5	95
Liver cancer	B.1.7	3	0	1	1	0	0	95
Liver cancer due to hepatitis B	B.1.7.1	4	1	1	1	0	10	95
Liver cancer due to hepatitis C	B.1.7.2	4	1	1	1	0	10	95
Liver cancer due to alcohol use	B.1.7.3	4	1	1	1	0	15	95
Liver cancer due to NASH	B.1.7.4	4	1	1	1	0	15	95
Liver cancer due to other causes	B.1.7.5	4	1	1	1	0	0	95
Gallbladder and biliary tract cancer	B.1.8	3	1	1	1	0	20	95
Pancreatic cancer	B.1.9	3	1	1	1	0	15	95
Larynx cancer	B.1.10	3	1	1	1	0	20	95
Tracheal, bronchus, and lung cancer	B.1.11	3	1	1	1	0	10	95
Malignant skin melanoma	B.1.12	3	1	1	1	0	0	95
Non-melanoma skin cancer	B.1.13	3	0	1	1	0	20	95
Non-melanoma skin cancer (squamous-cell carcinoma)	B.1.13.1	4	1	1	1	0	20	95
Non-melanoma skin cancer (basal-cell carcinoma)	B.1.13.2	4	1	1	1	1	20	95
Breast cancer	B.1.14	3	1	1	1	0	15	95
Cervical cancer	B.1.15	3	1	0	1	0	15	95
Uterine cancer	B.1.16	3	1	0	1	0	20	95
Ovarian cancer	B.1.17	3	1	0	1	0	5	95
Prostate cancer	B.1.18	3	1	1	0	0	20	95
Testicular cancer	B.1.19	3	1	1	0	0	0	95
Kidney cancer	B.1.20	3	1	1	1	0	0	95
Bladder cancer	B.1.21	3	1	1	1	0	15	95

Brain and nervous system cancer	B.1.22	3	1	1	1	0	0	95
Thyroid cancer	B.1.23	3	1	1	1	0	5	95
Mesothelioma	B.1.24	3	1	1	1	0	20	95
Hodgkin lymphoma	B.1.25	3	1	1	1	0	1	95
Non-Hodgkin's lymphoma	B.1.26	3	1	1	1	0	1	95
Multiple myeloma	B.1.27	3	1	1	1	0	20	95
Leukaemia	B.1.28	3	0	1	1	0	0	95
Acute lymphoid leukaemia	B.1.28.1	4	1	1	1	0	0	95
Chronic lymphoid leukaemia	B.1.28.2	4	1	1	1	0	20	95
Acute myeloid leukaemia	B.1.28.3	4	1	1	1	0	0	95
Chronic myeloid leukaemia	B.1.28.4	4	1	1	1	0	0	95
Other leukaemia	B.1.28.5	4	1	1	1	0	0	95
Other malignant neoplasms	B.1.29	3	1	1	1	0	0	95
Other neoplasms	B.1.30	3	0	1	1	0	0	95
Myelodysplastic, myeloproliferative, and other hematopoietic neoplasms	B.1.30.1	4	1	1	1	0	0	95
Benign and in situ intestinal neoplasms	B.1.30.2	4	1	1	1	1	0	95
Benign and in situ cervical and uterine neoplasms	B.1.30.3	4	1	0	1	1	0	95
Other benign and in situ neoplasms	B.1.30.4	4	1	1	1	1	0	95
Cardiovascular diseases	B.2	2	0	1	1	0	0	95
Rheumatic heart disease	B.2.1	3	1	1	1	0	1	95
Ischaemic heart disease	B.2.2	3	1	1	1	0	15	95
Stroke	B.2.3	3	0	1	1	0	0	95
Ischaemic stroke	B.2.3.1	4	1	1	1	0	0	95
Intracerebral hemorrhage	B.2.3.2	4	1	1	1	0	0	95
Subarachnoid hemorrhage	B.2.3.3	4	1	1	1	0	0	95
Hypertensive heart disease	B.2.4	3	1	1	1	0	15	95
Non-rheumatic valvular heart disease	B.2.5	3	0	1	1	0	15	95
Non-rheumatic calcific aortic valve disease	B.2.5.1	4	1	1	1	0	15	95
Non-rheumatic degenerative mitral valve disease	B.2.5.2	4	1	1	1	0	15	95
Other non-rheumatic valve diseases	B.2.5.3	4	1	1	1	0	15	95
Cardiomyopathy and myocarditis	B.2.6	3	0	1	1	0	0	95
Myocarditis	B.2.6.1	4	1	1	1	0	0	95
Alcoholic cardiomyopathy	B.2.6.2	4	1	1	1	0	15	95
Other cardiomyopathy	B.2.6.3	4	1	1	1	0	0	95
Atrial fibrillation and flutter	B.2.8	3	1	1	1	0	30	95
Aortic aneurysm	B.2.9	3	1	1	1	0	15	95
Peripheral vascular disease	B.2.10	3	1	1	1	0	40	95
Endocarditis	B.2.11	3	1	1	1	0	0	95
Other cardiovascular and circulatory diseases	B.2.12	3	1	1	1	0	0	95
Chronic respiratory diseases	B.3	2	0	1	1	0	0	95
Chronic obstructive pulmonary disease	B.3.1	3	1	1	1	0	0.1	95
Pneumoconiosis	B.3.2	3	0	1	1	0	15	95
Silicosis	B.3.2.1	4	1	1	1	0	15	95
Asbestosis	B.3.2.2	4	1	1	1	0	15	95
Coal workers pneumoconiosis	B.3.2.3	4	1	1	1	0	15	95
Other pneumoconiosis	B.3.2.4	4	1	1	1	0	15	95
Asthma	B.3.3	3	1	1	1	0	1	95
Interstitial lung disease and pulmonary sarcoidosis	B.3.4	3	1	1	1	0	1	95
Other chronic respiratory diseases	B.3.5	3	1	1	1	0	0	95
Digestive diseases	B.4	2	0	1	1	0	0	95
Cirrhosis and other chronic liver diseases	B.4.1	3	0	1	1	0	1	95
Cirrhosis and other chronic liver diseases due to hepatitis B	B.4.1.1	4	1	1	1	0	1	95
Cirrhosis and other chronic liver diseases due to hepatitis C	B.4.1.2	4	1	1	1	0	1	95
Cirrhosis and other chronic liver diseases due to alcohol use	B.4.1.3	4	1	1	1	0	15	95
Cirrhosis and other chronic liver diseases due to NAFLD	B.4.1.4	4	1	1	1	0	15	95
Cirrhosis and other chronic liver diseases due to other causes	B.4.1.5	4	1	1	1	0	1	95
Upper digestive system diseases	B.4.2	3	0	1	1	0	1	95
Peptic ulcer disease	B.4.2.1	4	1	1	1	0	1	95
Gastritis and duodenitis	B.4.2.2	4	1	1	1	0	1	95
GERD	B.4.2.3	4	1	1	1	1	0.1	95
Appendicitis	B.4.3	3	1	1	1	0	1	95
Paralytic ileus and intestinal obstruction	B.4.4	3	1	1	1	0	0	95
Inguinal, femoral, and abdominal hernia	B.4.5	3	1	1	1	0	0	95

Inflammatory bowel disease	B.4.6	3	1	1	1	0	1	95
Vascular intestinal disorders	B.4.7	3	1	1	1	0	1	95
Gallbladder and biliary diseases	B.4.8	3	1	1	1	0	1	95
Pancreatitis	B.4.9	3	1	1	1	0	1	95
Other digestive diseases	B.4.10	3	1	1	1	0	1	95
Neurological disorders	B.5	2	0	1	1	0	0.1	95
Alzheimer's disease and other dementias	B.5.1	3	1	1	1	0	40	95
Parkinson's disease	B.5.2	3	1	1	1	0	20	95
Idiopathic epilepsy	B.5.3	3	1	1	1	0	0.1	95
Multiple sclerosis	B.5.4	3	1	1	1	0	5	95
Motor neuron disease	B.5.5	3	1	1	1	0	0	95
Headache disorders	B.5.6	3	0	1	1	1	5	95
Migraine	B.5.6.1	4	1	1	1	1	5	95
Tension-type headache	B.5.6.2	4	1	1	1	1	5	95
Other neurological disorders	B.5.7	3	1	1	1	0	0.1	95
Mental disorders	B.6	2	0	1	1	0	0	95
Schizophrenia	B.6.1	3	1	1	1	1	10	95
Depressive disorders	B.6.2	3	0	1	1	1	1	95
Major depressive disorder	B.6.2.1	4	1	1	1	1	1	95
Dysthymia	B.6.2.2	4	1	1	1	1	1	95
Bipolar disorder	B.6.3	3	1	1	1	1	10	95
Anxiety disorders	B.6.4	3	1	1	1	1	1	95
Eating disorders	B.6.5	3	0	1	1	0	5	45
Anorexia nervosa	B.6.5.1	4	1	1	1	0	5	45
Bulimia nervosa	B.6.5.2	4	1	1	1	0	5	45
Autism spectrum disorders	B.6.6	3	1	1	1	1	0	95
Attention-deficit/hyperactivity disorder	B.6.7	3	1	1	1	1	1	95
Conduct disorder	B.6.8	3	1	1	1	1	5	15
Idiopathic developmental intellectual disability	B.6.9	3	1	1	1	1	0	95
Other mental disorders	B.6.10	3	1	1	1	1	1	95
Substance use disorders	B.7	2	0	1	1	0	0	95
Alcohol use disorders	B.7.1	3	1	1	1	0	10	95
Drug use disorders	B.7.2	3	0	1	1	0	0	95
Opioid use disorders	B.7.2.1	4	1	1	1	0	0	95
Cocaine use disorders	B.7.2.2	4	1	1	1	0	10	95
Amphetamine use disorders	B.7.2.3	4	1	1	1	0	10	95
Cannabis use disorders	B.7.2.4	4	1	1	1	1	10	95
Other drug use disorders	B.7.2.5	4	1	1	1	0	10	95
Diabetes and kidney diseases	B.8	2	0	1	1	0	0	95
Diabetes mellitus	B.8.1	3	0	1	1	0	0	95
Diabetes mellitus type 1	B.8.1.1	4	1	1	1	0	0	95
Diabetes mellitus type 2	B.8.1.2	4	1	1	1	0	15	95
Chronic kidney disease	B.8.2	3	0	1	1	0	0.1	95
Chronic kidney disease due to diabetes mellitus type 1	B.8.2.1	4	1	1	1	0	0.1	95
Chronic kidney disease due to diabetes mellitus type 2	B.8.2.2	4	1	1	1	0	15	95
Chronic kidney disease due to hypertension	B.8.2.3	4	1	1	1	0	15	95
Chronic kidney disease due to glomerulonephritis	B.8.2.4	4	1	1	1	0	0.1	95
Chronic kidney disease due to other and unspecified causes	B.8.2.5	4	1	1	1	0	0.1	95
Acute glomerulonephritis	B.8.3	3	1	1	1	0	0.1	95
Skin and subcutaneous diseases	B.9	2	0	1	1	0	0.1	95
Dermatitis	B.9.1	3	0	1	1	1	1	95
Atopic dermatitis	B.9.1.1	4	1	1	1	1	1	95
Contact dermatitis	B.9.1.2	4	1	1	1	1	1	95
Seborrhoeic dermatitis	B.9.1.3	4	1	1	1	1	1	95
Psoriasis	B.9.2	3	1	1	1	1	1	95
Bacterial skin diseases	B.9.3	3	0	1	1	0	0	95
Cellulitis	B.9.3.1	4	1	1	1	0	0	95
Pyoderma	B.9.3.2	4	1	1	1	0	0	95
Scabies	B.9.4	3	1	1	1	1	0	95
Fungal skin diseases	B.9.5	3	1	1	1	1	0	95
Viral skin diseases	B.9.6	3	1	1	1	1	1	95
Acne vulgaris	B.9.7	3	1	1	1	1	5	95
Alopecia areata	B.9.8	3	1	1	1	1	1	95

Pruritus	B.9.9	3	1	1	1	1	0	95
Urticaria	B.9.10	3	1	1	1	1	0	95
Decubitus ulcer	B.9.11	3	1	1	1	0	1	95
Other skin and subcutaneous diseases	B.9.12	3	1	1	1	0	0	95
Sense organ diseases	B.10	2	0	1	1	1	0	95
Blindness and vision loss	B.10.1	3	0	1	1	1	0	95
Glaucoma	B.10.1.1	4	1	1	1	1	20	95
Cataract	B.10.1.2	4	1	1	1	1	20	95
Age-related macular degeneration	B.10.1.3	4	1	1	1	1	45	95
Refraction disorders	B.10.1.4	4	1	1	1	1	0	95
Near vision loss	B.10.1.5	4	1	1	1	1	0	95
Other vision loss	B.10.1.6	4	1	1	1	1	0	95
Age-related and other hearing loss	B.10.2	3	1	1	1	1	0	95
Other sense organ diseases	B.10.3	3	1	1	1	1	0	95
Musculoskeletal disorders	B.11	2	0	1	1	0	5	95
Rheumatoid arthritis	B.11.1	3	1	1	1	0	5	95
Osteoarthritis	B.11.2	3	0	1	1	1	30	95
Osteoarthritis hip	B.11.2.1	4	1	1	1	1	30	95
Osteoarthritis knee	B.11.2.2	4	1	1	1	1	30	95
Osteoarth Hand	B.11.2.3	4	1	1	1	1	30	95
	B.11.2.4	4	1	1	1	1	30	95
Low back pain	B.11.3	3	1	1	1	1	5	95
Neck pain	B.11.4	3	1	1	1	1	5	95
Gout	B.11.5	3	1	1	1	1	15	95
Other musculoskeletal disorders	B.11.6	3	1	1	1	0	5	95
Other non-communicable diseases	B.12	2	0	1	1	0	0	95
Congenital anomalies	B.12.1	3	0	1	1	0	0	65
Neural tube defects	B.12.1.1	4	1	1	1	0	0	65
Congenital heart anomalies	B.12.1.2	4	1	1	1	0	0	65
Orofacial clefts	B.12.1.3	4	1	1	1	0	0	1
Down's syndrome	B.12.1.4	4	1	1	1	0	0	65
Turner syndrome	B.12.1.5	4	1	0	1	1	0	95
Klinefelter syndrome	B.12.1.6	4	1	1	0	1	0	95
Other chromosomal abnormalities	B.12.1.7	4	1	1	1	0	0	65
Congenital musculoskeletal and limb anomalies	B.12.1.8	4	1	1	1	0	0	65
Urogenital congenital anomalies	B.12.1.9	4	1	1	1	0	0	65
Digestive congenital anomalies	B.12.1.10	4	1	1	1	0	0	65
Other congenital anomalies	B.12.1.11	4	1	1	1	0	0	65
Urinary diseases and male infertility	B.12.2	3	0	1	1	0	0	95
Urinary tract infections and interstitial nephritis	B.12.2.1	4	1	1	1	0	0	95
Urolithiasis	B.12.2.2	4	1	1	1	0	1	95
Benign prostatic hyperplasia	B.12.2.3	4	1	1	0	1	40	95
Male infertility	B.12.2.4	4	1	1	0	1	20	60
Other urinary diseases	B.12.2.5	4	1	1	1	0	0	95
Gynecological diseases	B.12.3	3	0	0	1	0	10	95
Uterine fibroids	B.12.3.1	4	1	0	1	0	10	95
Polycystic ovarian syndrome	B.12.3.2	4	1	0	1	1	10	50
Female infertility	B.12.3.3	4	1	0	1	1	20	50
Endometriosis	B.12.3.4	4	1	0	1	0	10	50
Genital prolapse	B.12.3.5	4	1	0	1	0	10	95
Premenstrual syndrome	B.12.3.6	4	1	0	1	1	10	50
Other gynecological diseases	B.12.3.7	4	1	0	1	0	10	95
Hemoglobinopathies and hemolytic anaemias	B.12.4	3	0	1	1	0	0	95
Thalassemias	B.12.4.1	4	1	1	1	0	0	95
Thalassaemias trait	B.12.4.2	4	1	1	1	1	0	95
Sickle cell disorders	B.12.4.3	4	1	1	1	0	0	95
Sickle cell trait	B.12.4.4	4	1	1	1	1	0	95
G6PD deficiency	B.12.4.5	4	1	1	1	0	0	95
G6PD trait	B.12.4.6	4	1	1	1	1	0	95
Other hemoglobinopathies and hemolytic anaemias	B.12.4.7	4	1	1	1	0	0	95
Endocrine, metabolic, blood, and immune disorders	B.12.5	3	1	1	1	0	0	95
Oral disorders	B.12.6	3	0	1	1	1	1	95
Caries of deciduous teeth	B.12.6.1	4	1	1	1	1	1	10

Caries of permanent teeth	B.12.6.2	4	1	1	1	1	5	95
Periodontal disease	B.12.6.3	4	1	1	1	1	15	95
Edentulism	B.12.6.4	4	1	1	1	1	20	95
Other oral disorders	B.12.6.5	4	1	1	1	1	1	95
Sudden infant death syndrome	B.12.7	3	1	1	1	0	0.01	0.1
**Injuries**	**C**	**1**	**0**	**1**	**1**	**0**	**0**	**95**
Transport injuries	C.1	2	0	1	1	0	0	95
Road injuries	C.1.1	3	0	1	1	0	0	95
Pedestrian road injuries	C.1.1.1	4	1	1	1	0	0	95
Cyclist road injuries	C.1.1.2	4	1	1	1	0	1	95
Motorcyclist road injuries	C.1.1.3	4	1	1	1	0	0	95
Motor vehicle road injuries	C.1.1.4	4	1	1	1	0	0	95
Other road injuries	C.1.1.5	4	1	1	1	0	0	95
Other transport injuries	C.1.2	3	1	1	1	0	0	95
Unintentional injuries	C.2	2	0	1	1	0	0	95
Falls	C.2.1	3	1	1	1	0	0	95
Drowning	C.2.2	3	1	1	1	0	0	95
Fire, heat, and hot substances	C.2.3	3	1	1	1	0	0	95
Poisonings	C.2.4	3	0	1	1	0	0	95
Poisoning by carbon monoxide	C.2.4.1	4	1	1	1	0	0	95
Poisoning by other means	C.2.4.2	4	1	1	1	0	0	95
Exposure to mechanical forces	C.2.5	3	0	1	1	0	0	95
Unintentional firearm injuries	C.2.5.1	4	1	1	1	0	0	95
Other exposure to mechanical forces	C.2.5.2	4	1	1	1	0	0	95
Adverse effects of medical treatment	C.2.6	3	1	1	1	0	0	95
Animal contact	C.2.7	3	0	1	1	0	0	95
Venomous animal contact	C.2.7.1	4	1	1	1	0	0	95
Non-venomous animal contact	C.2.7.2	4	1	1	1	0	0	95
Foreign body	C.2.8	3	0	1	1	0	0	95
Pulmonary aspiration and foreign body in airway	C.2.8.1	4	1	1	1	0	0	95
Foreign body in eyes	C.2.8.2	4	1	1	1	1	0	95
Foreign body in other body part	C.2.8.3	4	1	1	1	0	0	95
Environmental heat and cold exposure	C.2.9	3	1	1	1	0	0	95
Exposure to forces of nature	C.2.10	3	1	1	1	0	0	95
Other unintentional injuries	C.2.11	3	1	1	1	0	0	95
Self-harm and interpersonal violence	C.3	2	0	1	1	0	0	95
Self-harm	C.3.1	3	0	1	1	0	10	95
Self-harm by firearm	C.3.1.1	4	1	1	1	0	10	95
Self-harm by other specified means	C.3.1.2	4	1	1	1	0	10	95
Interpersonal violence	C.3.2	3	0	1	1	0	0	95
Assault by firearm	C.3.2.1	4	1	1	1	0	0	95
Assault by sharp object	C.3.2.2	4	1	1	1	0	0	95
Sexual violence	C.3.2.3	4	1	1	1	1	0	95
Assault by other means	C.3.2.4	4	1	1	1	0	0	95
Conflict and terrorism	C.3.3	3	1	1	1	0	0	95
Executions and police conflict	C.3.4	3	1	1	1	0	0	95

Table S7. Number and proportion of countries and territories (n=204) reducing under-5 mortality rate (U5MR) and neonatal mortality rate (NMR) to below the SDG 3.2 targets of 25 under-5 and 12 neonatal deaths per 1000 live births, by 2030, under six future health scenarios.

Age	Scenario	N below SDG target	Proportion below SDG target	Countries above SDG target
Neonatal	Reference	139	0.68	Azerbaijan, Bolivia (Plurinational State of), Dominica, Dominican Republic, Guyana, Haiti, Suriname, Afghanistan, Sudan, Yemen, Bangladesh, Bhutan, India, Nepal, Pakistan, Kiribati, Papua New Guinea, Cambodia, Lao People's Democratic Republic, Myanmar, Timor-Leste, Angola, Central African Republic, Congo, Democratic Republic of the Congo, Equatorial Guinea, Gabon, Burundi, Comoros, Djibouti, Eritrea, Ethiopia, Kenya, Madagascar, Malawi, Mozambique, Rwanda, Somalia, South Sudan, Uganda, United Republic of Tanzania, Zambia, Botswana, Eswatini, Lesotho, Namibia, South Africa, Zimbabwe, Benin, Burkina Faso, Cameroon, Chad, Côte d'Ivoire, Gambia, Ghana, Guinea, Guinea-Bissau, Liberia, Mali, Mauritania, Niger, Nigeria, Senegal, Sierra Leone, Togo
	Better	145	0.71	Azerbaijan, Dominica, Dominican Republic, Haiti, Suriname, Afghanistan, Sudan, Yemen, Bangladesh, Bhutan, India, Nepal, Pakistan, Papua New Guinea, Cambodia, Myanmar, Angola, Central African Republic, Congo, Democratic Republic of the Congo, Equatorial Guinea, Burundi, Comoros, Djibouti, Eritrea, Ethiopia, Kenya, Madagascar, Malawi, Mozambique, Rwanda, Somalia, South Sudan, Uganda, United Republic of Tanzania, Zambia, Botswana, Eswatini, Lesotho, Namibia, South Africa, Zimbabwe, Benin, Burkina Faso, Cameroon, Chad, Côte d'Ivoire, Gambia, Ghana, Guinea, Guinea-Bissau, Liberia, Mali, Mauritania, Niger, Nigeria, Senegal, Sierra Leone, Togo
	Worse	137	0.67	Azerbaijan, Tajikistan, Bolivia (Plurinational State of), Dominica, Dominican Republic, Guyana, Haiti, Jamaica, Suriname, Afghanistan, Sudan, Yemen, Bangladesh, Bhutan, India, Nepal, Pakistan, Kiribati, Papua New Guinea, Cambodia, Lao People's Democratic Republic, Myanmar, Timor-Leste, Angola, Central African Republic, Congo, Democratic Republic of the Congo, Equatorial Guinea, Gabon, Burundi, Comoros, Djibouti, Eritrea, Ethiopia, Kenya, Madagascar, Malawi, Mozambique, Rwanda, Somalia, South Sudan, Uganda, United Republic of Tanzania, Zambia, Botswana, Eswatini, Lesotho, Namibia, South Africa, Zimbabwe, Benin, Burkina Faso, Cameroon, Chad, Côte d'Ivoire, Gambia, Ghana, Guinea, Guinea-Bissau, Liberia, Mali, Mauritania, Niger, Nigeria, Senegal, Sierra Leone, Togo
	No Covid	140	0.69	Azerbaijan, Bolivia (Plurinational State of), Dominica, Dominican Republic, Guyana, Haiti, Suriname, Afghanistan, Sudan, Yemen, Bangladesh, Bhutan, India, Nepal, Pakistan, Papua New Guinea, Cambodia, Lao People's Democratic Republic, Myanmar, Timor-Leste, Angola, Central African Republic, Congo, Democratic Republic of the Congo, Equatorial Guinea, Gabon, Burundi, Comoros, Djibouti, Eritrea, Ethiopia, Kenya, Madagascar, Malawi, Mozambique, Rwanda, Somalia, South Sudan, Uganda, United Republic of Tanzania, Zambia, Botswana, Eswatini, Lesotho, Namibia, South Africa, Zimbabwe, Benin, Burkina Faso, Cameroon, Chad, Côte d'Ivoire, Gambia, Ghana, Guinea, Guinea-Bissau, Liberia, Mali, Mauritania, Niger, Nigeria, Senegal, Sierra Leone, Togo
	Neonatal	145	0.71	Azerbaijan, Dominica, Dominican Republic, Haiti, Suriname, Afghanistan, Sudan, Yemen, Bangladesh, Bhutan, India, Nepal, Pakistan, Papua New Guinea, Cambodia, Myanmar, Angola, Central African Republic, Congo, Democratic Republic of the Congo, Equatorial Guinea, Burundi, Comoros, Djibouti, Eritrea, Ethiopia, Kenya, Madagascar, Malawi, Mozambique, Rwanda, Somalia, South Sudan, Uganda, United Republic of Tanzania, Zambia, Botswana, Eswatini, Lesotho, Namibia, South Africa, Zimbabwe, Benin, Burkina Faso, Cameroon, Chad, Côte d'Ivoire, Gambia, Ghana, Guinea, Guinea-Bissau, Liberia, Mali, Mauritania, Niger, Nigeria, Senegal, Sierra Leone, Togo
	Child	139	0.68	Azerbaijan, Bolivia (Plurinational State of), Dominica, Dominican Republic, Guyana, Haiti, Suriname, Afghanistan, Sudan, Yemen, Bangladesh, Bhutan, India, Nepal, Pakistan, Kiribati, Papua New Guinea, Cambodia, Lao People's Democratic Republic, Myanmar, Timor-Leste, Angola, Central African Republic, Congo, Democratic Republic of the Congo, Equatorial Guinea, Gabon, Burundi, Comoros, Djibouti, Eritrea, Ethiopia, Kenya, Madagascar, Malawi, Mozambique, Rwanda, Somalia, South Sudan, Uganda, United Republic of Tanzania, Zambia, Botswana, Eswatini, Lesotho, Namibia, South Africa, Zimbabwe, Benin, Burkina Faso, Cameroon, Chad, Côte d'Ivoire, Gambia, Ghana, Guinea, Guinea-Bissau, Liberia, Mali, Mauritania, Niger, Nigeria, Senegal, Sierra Leone, Togo
Under 5	Reference	154	0.75	Dominica, Haiti, Afghanistan, Sudan, Yemen, Pakistan, Kiribati, Papua New Guinea, Myanmar, Angola, Central African Republic, Congo, Democratic Republic of the Congo, Burundi, Comoros, Djibouti, Eritrea, Ethiopia, Kenya, Madagascar, Malawi, Mozambique, Rwanda, Somalia, South Sudan, Uganda, United Republic of Tanzania, Zambia, Botswana, Eswatini, Lesotho, Namibia, South Africa, Zimbabwe, Benin, Burkina Faso, Cameroon, Chad, Côte d'Ivoire, Ghana, Guinea, Guinea-Bissau, Liberia, Mali, Mauritania, Niger, Nigeria, Senegal, Sierra Leone, Togo
	Better	164	0.8	Dominica, Haiti, Afghanistan, Yemen, Pakistan, Papua New Guinea, Angola, Central African Republic, Democratic Republic of the Congo, Burundi, Comoros, Djibouti, Ethiopia, Madagascar, Malawi, Mozambique, Somalia, South Sudan, Uganda, United Republic of Tanzania, Zambia, Botswana, Eswatini, Lesotho, Zimbabwe, Benin, Burkina Faso, Cameroon, Chad, Côte d'Ivoire, Ghana, Guinea, Guinea-Bissau, Liberia, Mali, Niger, Nigeria, Senegal, Sierra Leone, Togo
	Worse	142	0.7	Azerbaijan, Tajikistan, Bolivia (Plurinational State of), Dominica, Haiti, Afghanistan, Sudan, Yemen, Bhutan, India, Pakistan, Kiribati, Papua New Guinea, Vanuatu, Cambodia, Lao People's Democratic Republic, Myanmar, Timor-Leste, Angola, Central African Republic, Congo, Democratic Republic of the Congo, Equatorial Guinea, Gabon, Burundi, Comoros, Djibouti, Eritrea, Ethiopia, Kenya, Madagascar, Malawi, Mozambique, Rwanda, Somalia, South Sudan, Uganda, United Republic of Tanzania, Zambia, Botswana, Eswatini, Lesotho, Namibia, South Africa, Zimbabwe, Benin, Burkina Faso, Cameroon, Chad, Côte d'Ivoire, Gambia, Ghana, Guinea, Guinea-Bissau, Liberia, Mali, Mauritania, Niger, Nigeria, Senegal,
	No Covid	154	0.75	Dominica, Haiti, Afghanistan, Sudan, Yemen, Pakistan, Kiribati, Papua New Guinea, Myanmar, Angola, Central African Republic, Congo, Democratic Republic of the Congo, Burundi, Comoros, Djibouti, Eritrea, Ethiopia, Kenya, Madagascar, Malawi, Mozambique, Rwanda, Somalia, South Sudan, Uganda, United Republic of Tanzania, Zambia, Botswana, Eswatini, Lesotho, Namibia, South Africa, Zimbabwe, Benin, Burkina Faso, Cameroon, Chad, Côte d'Ivoire, Ghana, Guinea, Guinea-Bissau, Liberia, Mali, Mauritania, Niger, Nigeria, Senegal, Sierra Leone, Togo
	Neonatal	155	0.76	Dominica, Haiti, Afghanistan, Sudan, Yemen, Pakistan, Papua New Guinea, Myanmar, Angola, Central African Republic, Congo, Democratic Republic of the Congo, Burundi, Comoros, Djibouti, Eritrea, Ethiopia, Kenya, Madagascar, Malawi, Mozambique, Rwanda, Somalia, South Sudan, Uganda, United Republic of Tanzania, Zambia, Botswana, Eswatini, Lesotho, Namibia, South Africa, Zimbabwe, Benin, Burkina Faso, Cameroon, Chad, Côte d'Ivoire, Ghana, Guinea, Guinea-Bissau, Liberia, Mali, Mauritania, Niger, Nigeria, Senegal, Sierra Leone, Togo
	Child	158	0.77	Dominica, Haiti, Afghanistan, Sudan, Yemen, Pakistan, Papua New Guinea, Myanmar, Angola, Central African Republic, Democratic Republic of the Congo, Burundi, Comoros, Djibouti, Eritrea, Ethiopia, Madagascar, Malawi, Mozambique, Rwanda, Somalia, South Sudan, Uganda, United Republic of Tanzania, Zambia, Botswana, Eswatini, Lesotho, South Africa, Zimbabwe, Benin, Burkina Faso, Cameroon, Chad, Côte d'Ivoire, Ghana, Guinea, Guinea-Bissau, Liberia, Mali, Mauritania, Niger, Nigeria, Senegal, Sierra Leone, Togo

Table S8a. By level-3 cause in the GBD cause hierarchy, global rank for the complete under-5 age group (a) in 2019, (b) under the condition where all countries have mortality at least as low as the Global Optimum, and (c) under the condition where all countries have mortality at least as low as the Survival Potential Frontier.

	Rank			Proportion of global deaths above benchmark	
**Cause of Death**	**2019**	**Global Optimum**	**Survival Potential Frontier**	**Global Optimum**	**Survival Potential Frontier**
Neonatal disorders	1	1	1	0·952 (0·948–0·958)	0·328 (0·278–0·384)
Lower respiratory infections	2	3	2	0·975 (0·971–0·979)	0·332 (0·277–0·395)
Diarrhoeal diseases	3	11	4	0·983 (0·980–0·986)	0·400 (0·335–0·473)
Congenital anomalies	4	2	3	0·918 (0·899–0·936)	0·301 (0·234–0·371)
Malaria	5	82	37	0·988 (0·986–0·990)	0·535 (0·457–0·618)
Meningitis	6	10	5	0·978 (0·974–0·982)	0·444 (0·387–0·507)
Whooping cough	7	61	7	0·981 (0·977–0·986)	0·391 (0·269–0·515)
Protein-energy malnutrition	8	38	6	0·983 (0·980–0·986)	0·366 (0·298–0·446)
Sexually transmitted infections excluding HIV	9	69	18	0·979 (0·976–0·983)	0·417 (0·332–0·502)
Measles	10	81	13	0·984 (0·981–0·988)	0·423 (0·326–0·521)
Tuberculosis	11	47	9	0·983 (0·981–0·986)	0·359 (0·298–0·436)
HIV/AIDS	12	59	20	0·978 (0·974–0·983)	0·407 (0·314–0·501)
iNTS	13	73	46	0·990 (0·988–0·992)	0·670 (0·616–0·724)
Road injuries	14	7	11	0·939 (0·927–0·952)	0·385 (0·336–0·439)
Drowning	15	6	8	0·932 (0·920–0·946)	0·319 (0·272–0·381)
Sudden infant death syndrome	16	4	10	0·875 (0·777–0·947)	0·269 (0·154–0·430)
Foreign body	17	8	16	0·903 (0·891–0·916)	0·454 (0·407–0·501)
Hemoglobinopathies and hemolytic anaemias	18	60	66	0·985 (0·980–0·990)	0·627 (0·522–0·703)
Typhoid and paratyphoid	19	74	74	0·977 (0·974–0·981)	0·493 (0·428–0·562)
Paralytic ileus and intestinal obstruction	20	19	14	0·951 (0·942–0·960)	0·358 (0·293–0·434)
Tetanus	21	72	45	0·982 (0·979–0·986)	0·449 (0·346–0·541)
Encephalitis	22	20	28	0·947 (0·939–0·956)	0·397 (0·329–0·473)
Other unspecified infectious diseases	23	17	22	0·952 (0·924–0·963)	0·383 (0·309–0·462)
Leukaemia	24	13	21	0·935 (0·921–0·948)	0·357 (0·299–0·422)
Falls	25	18	25	0·940 (0·928–0·950)	0·393 (0·331–0·463)
Fire, heat, and hot substances	26	14	15	0·949 (0·928–0·963)	0·325 (0·258–0·401)
Other malignant neoplasms	27	9	17	0·900 (0·872–0·921)	0·281 (0·213–0·356)
Acute hepatitis	28	68	58	0·976 (0·971–0·981)	0·461 (0·376–0·543)
Endocrine, metabolic, blood, and immune disorders	29	5	19	0·860 (0·830–0·887)	0·325 (0·263–0·383)
Interpersonal violence	30	23	31	0·943 (0·931–0·953)	0·459 (0·406–0·519)

Conflict and terrorism	31	96	83	0·982 (0·979–0·984)	0·323 (0·254–0·395)
Exposure to mechanical forces	32	24	27	0·952 (0·929–0·964)	0·409 (0·332–0·485)
Poisonings	33	36	33	0·968 (0·962–0·974)	0·466 (0·411–0·528)
Adverse effects of medical treatment	34	22	23	0·954 (0·939–0·966)	0·373 (0·292–0·452)
Animal contact	35	40	41	0·977 (0·972–0·981)	0·448 (0·377–0·524)
Stroke	36	37	39	0·966 (0·960–0·972)	0·484 (0·424–0·545)
Chronic kidney disease	37	21	24	0·945 (0·936–0·955)	0·342 (0·282–0·403)
Brain and nervous system cancer	38	15	26	0·911 (0·890–0·928)	0·320 (0·248–0·384)
Idiopathic epilepsy	39	12	29	0·927 (0·905–0·948)	0·384 (0·323–0·456)
Cardiomyopathy and myocarditis	40	29	35	0·938 (0·927–0·949)	0·391 (0·333–0·457)
Cirrhosis and other chronic liver diseases	41	34	34	0·970 (0·963–0·976)	0·397 (0·331–0·474)
Other cardiovascular and circulatory diseases	42	27	40	0·949 (0·940–0·957)	0·465 (0·409–0·519)
Dengue	43	89	95	0·966 (0·962–0·970)	0·312 (0·203–0·431)
Other unintentional injuries	44	26	32	0·959 (0·950–0·967)	0·376 (0·320–0·434)
Other neglected tropical diseases	45	64	69	0·986 (0·982–0·990)	0·670 (0·614–0·731)
Asthma	46	49	36	0·974 (0·970–0·978)	0·307 (0·231–0·383)
Varicella and herpes zoster	47	53	30	0·976 (0·971–0·980)	0·290 (0·228–0·358)
Urinary diseases and male infertility	48	33	48	0·966 (0·960–0·972)	0·459 (0·389–0·528)
Upper respiratory infections	49	57	67	0·963 (0·915–0·984)	0·408 (0·291–0·542)
Other neurological disorders	50	16	38	0·862 (0·831–0·893)	0·302 (0·241–0·364)
Bacterial skin diseases	51	46	50	0·971 (0·964–0·976)	0·405 (0·326–0·489)
Other nutritional deficiencies	52	50	56	0·972 (0·966–0·977)	0·510 (0·451–0·569)
Diphtheria	53	77	70	0·991 (0·987–0·995)	0·695 (0·625–0·753)
Inguinal, femoral, and abdominal hernia	54	54	51	0·967 (0·957–0·974)	0·358 (0·280–0·433)
Diabetes mellitus	55	30	42	0·946 (0·934–0·958)	0·303 (0·230–0·392)
Other transport injuries	56	31	44	0·944 (0·923–0·960)	0·348 (0·234–0·432)
Other digestive diseases	57	45	52	0·957 (0·938–0·974)	0·458 (0·359–0·557)
Non-Hodgkin's lymphoma	58	28	43	0·918 (0·884–0·941)	0·307 (0·227–0·381)
Environmental heat and cold exposure	59	39	60	0·955 (0·928–0·969)	0·442 (0·371–0·518)
Rabies	60	79	88	0·985 (0·980–0·989)	0·581 (0·489–0·663)
Kidney cancer	61	25	49	0·898 (0·866–0·923)	0·381 (0·305–0·453)
Liver cancer	62	32	54	0·925 (0·912–0·938)	0·371 (0·313–0·437)
Other chronic respiratory diseases	63	43	53	0·950 (0·929–0·966)	0·396 (0·323–0·469)

Rheumatic heart disease	64	52	57	0·973 (0·966–0·979)	0·460 (0·393–0·522)
Endocarditis	65	44	61	0·959 (0·944–0·969)	0·516 (0·462–0·572)
Leishmaniasis	66	84	80	0·983 (0·970–0·991)	0·349 (0·206–0·517)
Upper digestive system diseases	67	63	64	0·983 (0·977–0·988)	0·570 (0·497–0·655)
Intestinal nematode infections	68	91	62	0·988 (0·985–0·990)	0·537 (0·474–0·604)
Appendicitis	69	58	65	0·975 (0·961–0·983)	0·412 (0·320–0·499)
Chronic obstructive pulmonary disease	70	51	59	0·963 (0·952–0·974)	0·345 (0·267–0·428)
Other intestinal infectious diseases	71	80	55	0·983 (0·978–0·988)	0·381 (0·289–0·469)
Inflammatory bowel disease	72	55	68	0·943 (0·911–0·963)	0·436 (0·370–0·510)
Motor neuron disease	73	65	79	0·869 (0·856–0·884)	0·569 (0·528–0·610)
Gallbladder and biliary diseases	74	67	72	0·979 (0·963–0·986)	0·516 (0·420–0·601)
Exposure to forces of nature	75	93	73	0·972 (0·969–0·976)	0·325 (0·237–0·418)
Yellow fever	76	90	71	0·987 (0·983–0·990)	0·485 (0·372–0·593)
Testicular cancer	77	35	63	1·00 (1·00–1·00)	0·569 (0·368–0·928)
Ebola virus disease	78	94	12	0·986 (0·983–0·989)	0·0760 (0·00305–0·227)
Vascular intestinal disorders	79	66	82	0·966 (0·952–0·978)	0·560 (0·486–0·637)
Interstitial lung disease and pulmonary sarcoidosis	80	62	76	0·925 (0·902–0·942)	0·362 (0·289–0·436)
Acute glomerulonephritis	81	71	91	0·956 (0·941–0·966)	0·422 (0·351–0·496)
Other neoplasms	82	41	75	0·843 (0·809–0·882)	0·442 (0·387–0·496)
Otitis media	83	78	92	0·977 (0·958–0·987)	0·292 (0·153–0·447)
Executions and police conflict	84	48	78	0·883 (0·849–0·914)	0·402 (0·355–0·462)
Pancreatitis	85	70	85	0·976 (0·947–0·988)	0·582 (0·424–0·675)
Schistosomiasis	86	86	84	0·990 (0·987–0·993)	0·579 (0·504–0·662)
Hodgkin lymphoma	87	56	81	0·923 (0·879–0·953)	0·469 (0·345–0·566)
Malignant skin melanoma	88	42	77	0·778 (0·696–0·874)	0·305 (0·140–0·442)
Cystic echinococcosis	89	88	89	0·984 (0·980–0·988)	0·475 (0·396–0·561)
Other skin and subcutaneous diseases	90	76	93	0·942 (0·930–0·956)	0·533 (0·469–0·590)
African trypanosomiasis	91	85	87	0·990 (0·986–0·994)	0·430 (0·217–0·590)
Cysticercosis	92	87	94	0·986 (0·969–0·992)	0·447 (0·0474–0·607)
Decubitus ulcer	93	75	96	0·944 (0·924–0·961)	0·372 (0·277–0·474)
Chagas disease	94	83	86	0·937 (0·929–0·945)	0·513 (0·420–0·598)
Zika virus disease	95	95	90	0·933 (0·916–0·947)	0·431 (0·305–0·553)

Table S8b. By level-3 cause in the GBD cause hierarchy, global rank for the neonatal (<28 days) age group (a) in 2019, (b) under the condition where all countries have mortality at least as low as the Global Optimum, and (c) under the condition where all countries have mortality at least as low as the Survival Potential Frontier.

	Rank			Proportion of global deaths above benchmark	
**Cause of Death**	**2019**	**Global Optimum**	**Survival Potential Frontier**	**Global Optimum**	**Survival Potential Frontier**
Neonatal disorders	1	1	1	0·957 (0·953–0·962)	0·360 (0·306–0·421)
Congenital anomalies	2	2	2	0·908 (0·890–0·924)	0·282 (0·228–0·344)
Lower respiratory infections	3	3	3	0·977 (0·973–0·981)	0·385 (0·319–0·454)
Sexually transmitted infections excluding HIV	4	38	6	0·979 (0·976–0·983)	0·431 (0·334–0·521)
Diarrhoeal diseases	5	6	4	0·979 (0·975–0·983)	0·377 (0·299–0·458)
Malaria	6	49	31	0·985 (0·981–0·988)	0·458 (0·362–0·559)
Tetanus	7	44	12	0·984 (0·981–0·987)	0·499 (0·377–0·594)
Meningitis	8	7	5	0·975 (0·971–0·980)	0·399 (0·333–0·470)
Other unspecified infectious diseases	9	9	8	0·950 (0·930–0·962)	0·335 (0·258–0·419)
Foreign body	10	10	11	0·933 (0·922–0·943)	0·512 (0·454–0·569)
Conflict and terrorism	11	59	47	0·981 (0·978–0·984)	0·265 (0·111–0·405)
Paralytic ileus and intestinal obstruction	12	11	10	0·952 (0·944–0·959)	0·373 (0·303–0·446)
Exposure to mechanical forces	13	28	13	0·973 (0·958–0·981)	0·410 (0·252–0·529)
Sudden infant death syndrome	14	5	7	0·852 (0·758–0·929)	0·255 (0·137–0·420)
Endocrine, metabolic, blood, and immune disorders	15	4	9	0·815 (0·765–0·851)	0·323 (0·269–0·377)
Stroke	16	27	28	0·959 (0·951–0·967)	0·473 (0·409–0·533)
Falls	17	17	22	0·965 (0·956–0·973)	0·472 (0·381–0·562)
Interpersonal violence	18	13	17	0·944 (0·932–0·955)	0·418 (0·348–0·487)
Cardiomyopathy and myocarditis	19	18	18	0·950 (0·938–0·960)	0·366 (0·289–0·454)
Encephalitis	20	15	21	0·958 (0·950–0·965)	0·458 (0·381–0·538)
Other cardiovascular and circulatory diseases	21	22	25	0·945 (0·936–0·954)	0·463 (0·407–0·513)
Adverse effects of medical treatment	22	12	14	0·946 (0·926–0·958)	0·361 (0·292–0·433)
Other malignant neoplasms	23	8	15	0·896 (0·870–0·915)	0·364 (0·292–0·428)
Road injuries	24	16	16	0·957 (0·950–0·963)	0·350 (0·285–0·414)
Hemoglobinopathies and hemolytic anaemias	25	42	42	0·975 (0·970–0·979)	0·554 (0·448–0·630)
Leukaemia	26	21	24	0·957 (0·933–0·970)	0·388 (0·297–0·489)
Inguinal, femoral, and abdominal hernia	27	25	19	0·968 (0·958–0·974)	0·351 (0·276–0·425)
Urinary diseases and male infertility	28	31	33	0·977 (0·972–0·981)	0·478 (0·384–0·568)
Other neglected tropical diseases	29	32	44	0·981 (0·975–0·987)	0·595 (0·524–0·664)

iNTS	30	46	43	0·986 (0·983–0·989)	0·573 (0·495–0·647)
Brain and nervous system cancer	31	14	30	0·935 (0·887–0·955)	0·451 (0·364–0·531)
Drowning	32	20	20	0·950 (0·942–0·958)	0·321 (0·254–0·393)
Fire, heat, and hot substances	33	23	27	0·965 (0·956–0·972)	0·398 (0·310–0·482)
Animal contact	34	39	35	0·978 (0·974–0·983)	0·523 (0·429–0·609)
Poisonings	35	33	29	0·970 (0·964–0·975)	0·420 (0·346–0·495)
Other unintentional injuries	36	24	26	0·962 (0·954–0·969)	0·356 (0·280–0·438)
Varicella and herpes zoster	37	40	23	0·975 (0·970–0·979)	0·275 (0·208–0·346)
Liver cancer	38	29	36	0·963 (0·955–0·969)	0·442 (0·363–0·519)
Dengue	39	50	54	0·972 (0·967–0·976)	0·321 (0·166–0·481)
Environmental heat and cold exposure	40	37	40	0·964 (0·941–0·975)	0·508 (0·406–0·606)
Endocarditis	41	34	38	0·962 (0·952–0·969)	0·526 (0·467–0·586)
Otitis media	42	47	49	0·982 (0·971–0·987)	0·325 (0·154–0·506)
Diabetes mellitus	43	19	32	0·882 (0·828–0·931)	0·266 (0·190–0·354)
Upper respiratory infections	44	43	45	0·955 (0·923–0·974)	0·503 (0·390–0·603)
Other transport injuries	45	30	34	0·954 (0·946–0·961)	0·338 (0·268–0·408)
Kidney cancer	46	26	37	0·905 (0·882–0·925)	0·334 (0·266–0·407)
Exposure to forces of nature	47	55	46	0·979 (0·975–0·982)	0·294 (0·181–0·400)
Testicular cancer	48	35	39	1·00 (1·00–1·00)	0·589 (0·418–1·00)
Other neoplasms	49	36	48	0·848 (0·826–0·875)	0·521 (0·474–0·569)
Motor neuron disease	50	45	52	0·852 (0·833–0·871)	0·605 (0·567–0·643)
Malignant skin melanoma	51	41	50	0·770 (0·718–0·851)	0·362 (0·281–0·464)
Yellow fever	52	51	51	0·984 (0·980–0·988)	0·431 (0·300–0·545)
Executions and police conflict	53	57	53	0·958 (0·947–0·967)	0·545 (0·427–0·639)
Zika virus disease	54	58	41	0·942 (0·925–0·955)	0·403 (0·263–0·546)

Table S9. Neonatal and under-5 mortality rate (deaths per 1000 live births) by sex (male, female, both-sex), year (2000, 2015, 2019), and location (global and socio-demographic index (SDI) quintiles)

		Neonatal Mortality Rate (NMR)			Under-5 Mortality Rate (U5MR)		
**Location**	**Year**	**Both**	**Male**	**Female**	**Both**	**Male**	**Female**
Global	2000	28·0 (26·8–29·5)	30·7 (29·1–32·5)	25·2 (24·1–26·3)	71·2 (68·3–74·0)	73·3 (70·0–76·8)	69·0 (66·5–71·6)
	2015	20·3 (18·7–22·1)	22·2 (20·2–24·2)	18·3 (17·0–19·7)	43·9 (40·2–47·8)	45·7 (41·6–50·1)	42·0 (38·9–45·4)
	2019	17·9 (16·3–19·8)	19·6 (17·6–21·8)	16·1 (14·9–17·6)	37·1 (33·2–41·7)	38·8 (34·5–44·0)	35·2 (31·9–39·2)
High SDI	2000	4·00 (3·88–4·14)	4·36 (4·21–4·53)	3·62 (3·50–3·75)	7·75 (7·53–7·99)	8·40 (8·16–8·68)	7·05 (6·86–7·27)
	2015	2·87 (2·79–2·95)	3·09 (2·99–3·20)	2·64 (2·56–2·71)	5·22 (5·11–5·34)	5·64 (5·50–5·79)	4·77 (4·70–4·85)
	2019	2·60 (2·51–2·70)	2·80 (2·69–2·91)	2·39 (2·31–2·48)	4·70 (4·56–4·86)	5·08 (4·88–5·29)	4·30 (4·20–4·41)
High-middle SDI	2000	12·6 (12·0–13·2)	13·8 (13·0–14·6)	11·3 (10·8–11·8)	25·4 (24·1–26·6)	27·2 (25·7–28·7)	23·4 (22·4–24·5)
	2015	6·22 (5·86–6·61)	6·67 (6·23–7·14)	5·72 (5·41–6·05)	11·6 (10·9–12·3)	12·3 (11·5–13·2)	10·8 (10·2–11·4)
	2019	5·10 (4·71–5·55)	5·46 (5·01–5·97)	4·71 (4·38–5·07)	9·36 (8·66–10·1)	9·97 (9·14–10·9)	8·70 (8·12–9·39)
Low SDI	2000	40·2 (38·5–41·9)	44·6 (42·4–46·8)	35·5 (34·0–37·0)	138 (133–143)	143 (137–149)	133 (128–138)
	2015	30·2 (27·5–33·2)	33·8 (30·4–37·6)	26·4 (24·4–28·7)	84·7 (76·8–93·6)	88·8 (79·2–99·2)	80·4 (73·7–87·4)
	2019	27·1 (24·0–30·8)	30·3 (26·6–35·0)	23·6 (21·2–26·5)	71·8 (63·3–82·5)	75·7 (65·7–88·3)	67·7 (60·7–76·4)
Low-middle SDI	2000	37·6 (35·6–39·7)	40·7 (38·3–43·2)	34·2 (32·4–36·0)	88·2 (83·6–93·3)	90·0 (84·8–95·7)	86·2 (81·9–91·0)
	2015	25·6 (23·3–28·2)	27·8 (25·1–30·7)	23·3 (21·3–25·4)	51·7 (47·1–56·7)	53·3 (48·3–58·8)	50·0 (45·8–54·5)
	2019	21·7 (19·7–24·0)	23·5 (21·2–26·2)	19·7 (18·0–21·6)	42·0 (37·8–46·7)	43·5 (38·9–48·9)	40·3 (36·6–44·4)
Middle SDI	2000	20·4 (19·5–21·3)	22·6 (21·5–23·8)	18·0 (17·3–18·7)	44·1 (42·2–46·0)	46·7 (44·4–49·1)	41·3 (39·7–42·9)
	2015	12·3 (11·3–13·3)	13·3 (12·3–14·5)	11·1 (10·4–12·0)	23·9 (22·0–25·9)	25·2 (23·1–27·6)	22·4 (20·8–24·0)
	2019	10·1 (9·11–11·1)	10·9 (9·81–12·2)	9·15 (8·35–10·0)	18·9 (17·1–21·0)	20·1 (18·0–22·5)	17·6 (16·1–19·4)

Table S10a. Cause-fraction (cause-specific proportion of total deaths) and cause rank for level-3 causes of death for children under 5 years of age, globally and for socio-demographic index (SDI) quintiles, in 2019, both sexes

Cause of death	Cause Fraction (2019, both sexes) Global	High SDI	High-middle SDI	Middle SDI	Low-middle SDI	Low SDI	Rank (2019, both sexes) Global	High SDI	High-middle SDI	Middle SDI	Low-middle SDI	Low SDI
Neonatal disorders	0·373 (0·356–0·388)	0·428 (0·399–0·443)	0·409 (0·390–0·428)	0·412 (0·390–0·428)	0·454 (0·431–0·472)	0·316 (0·300–0·330)	1	1	1	1	1	1
Lower respiratory infections	0·133 (0·121–0·144)	0·0245 (0·0226–0·0261)	0·0809 (0·0735–0·0879)	0·125 (0·113–0·134)	0·135 (0·123–0·146)	0·139 (0·122–0·154)	2	5	3	3	2	2
Diarrhoeal diseases	0·0992 (0·0828–0·116)	0·00491 (0·00421–0·00577)	0·0232 (0·0196–0·0278)	0·0566 (0·0486–0·0649)	0·0817 (0·0688–0·0966)	0·126 (0·102–0·150)	3	20	4	4	4	3
Congenital anomalies	0·0939 (0·0798–0·118)	0·258 (0·240–0·310)	0·226 (0·201–0·248)	0·144 (0·129–0·162)	0·0896 (0·0816–0·103)	0·0731 (0·0451–0·112)	4	2	2	2	3	5
Malaria	0·0706 (0·0346–0·120)	0·000000228 (0·0000000487–0·000000933)	0·0159 (0·00895–0·0233)	0·0357 (0·0221–0·0520)	0·0464 (0·0230–0·0765)	0·0975 (0·0448–0·169)	5	88	7	5	5	4
Meningitis	0·0222 (0·0187–0·0263)	0·00608 (0·00545–0·00654)	0·0104 (0·00945–0·0116)	0·0121 (0·0107–0·0141)	0·0164 (0·0139–0·0201)	0·0290 (0·0240–0·0350)	6	17	11	10	7	6
Whooping cough	0·0214 (0·00999–0·0380)	0·00204 (0·00101–0·00396)	0·00909 (0·00396–0·0190)	0·0174 (0·00847–0·0310)	0·0183 (0·00758–0·0364)	0·0251 (0·0107–0·0473)	7	30	14	7	6	7
Protein-energy malnutrition	0·0186 (0·0154–0·0220)	0·000514 (0·000462–0·000586)	0·00395 (0·00341–0·00457)	0·00930 (0·00819–0·0106)	0·0144 (0·0118–0·0174)	0·0244 (0·0196–0·0295)	8	52	25	13	8	8
Sexually transmitted infections excluding HIV	0·0162 (0·00550–0·0333)	0·00298 (0·000943–0·00638)	0·0114 (0·00369–0·0243)	0·0176 (0·00562–0·0374)	0·0135 (0·00436–0·0287)	0·0178 (0·00615–0·0363)	9	26	10	6	9	10
Measles	0·0139 (0·00492–0·0300)	0·000368 (0·000127–0·000888)	0·00312 (0·00101–0·00708)	0·00663 (0·00256–0·0138)	0·00725 (0·00247–0·0168)	0·0202 (0·00704–0·0440)	10	57	30	16	13	9
Tuberculosis	0·00994 (0·00847–0·0115)	0·000247 (0·000220–0·000278)	0·00255 (0·00226–0·00288)	0·00575 (0·00518–0·00648)	0·00886 (0·00762–0·0102)	0·0122 (0·0100–0·0146)	11	61	32	19	11	12
iNTS	0·00988 (0·00570–0·0156)	0·000318 (0·000188–0·000518)	0·000703 (0·000361–0·00120)	0·00207 (0·00115–0·00349)	0·00448 (0·00246–0·00724)	0·0156 (0·00909–0·0247)	12	58	53	37	17	11
HIV/AIDS	0·00971 (0·00809–0·0115)	0·00178 (0·00118–0·00534)	0·00855 (0·00582–0·0108)	0·0116 (0·00960–0·0141)	0·00999 (0·00820–0·0123)	0·00928 (0·00725–0·0115)	13	31	15	12	10	13
Road injuries	0·00763 (0·00650–0·00997)	0·0192 (0·0174–0·0207)	0·0172 (0·0152–0·0188)	0·0126 (0·0110–0·0141)	0·00551 (0·00466–0·00747)	0·00680 (0·00531–0·00979)	14	7	6	9	15	14
Drowning	0·00725 (0·00636–0·00866)	0·0138 (0·0127–0·0146)	0·0146 (0·0131–0·0162)	0·0135 (0·0109–0·0150)	0·00761 (0·00655–0·00922)	0·00490 (0·00397–0·00645)	15	9	8	8	12	16
Sudden infant death syndrome	0·00548 (0·00253–0·0114)	0·0491 (0·0440–0·0530)	0·0123 (0·0106–0·0150)	0·00704 (0·00446–0·0115)	0·00478 (0·00185–0·0103)	0·00430 (0·000968–0·0117)	16	3	9	15	16	18
Foreign body	0·00503 (0·00458–0·00551)	0·0333 (0·0309–0·0351)	0·0228 (0·0201–0·0251)	0·0119 (0·0104–0·0133)	0·00437 (0·00396–0·00483)	0·00211 (0·00181–0·00249)	17	4	5	11	18	30
Hemoglobinopathies and hemolytic anaemias	0·00459 (0·00327–0·00612)	0·00134 (0·00113–0·00152)	0·00248 (0·00215–0·00303)	0·00240 (0·00188–0·00332)	0·00301 (0·00219–0·00404)	0·00621 (0·00391–0·00899)	18	33	34	33	25	15
Typhoid and paratyphoid	0·00430 (0·00164–0·00845)	0·000163 (0·0000441–0·000431)	0·00284 (0·000971–0·00588)	0·00363 (0·00124–0·00749)	0·00679 (0·00258–0·0131)	0·00324 (0·00122–0·00637)	19	66	31	23	14	21
Tetanus	0·00363 (0·00287–0·00525)	0·0000632 (0·0000399–0·000110)	0·00109 (0·000738–0·00156)	0·00226 (0·00153–0·00316)	0·00402 (0·00286–0·00582)	0·00397 (0·00303–0·00637)	20	76	45	35	22	19
Paralytic ileus and intestinal obstruction	0·00359 (0·00278–0·00464)	0·00750 (0·00682–0·00807)	0·00530 (0·00457–0·00617)	0·00557 (0·00425–0·00711)	0·00322 (0·00241–0·00432)	0·00312 (0·00229–0·00435)	21	15	20	20	23	22
Other unspecified infectious diseases	0·00335 (0·00226–0·00430)	0·00784 (0·00595–0·0106)	0·00527 (0·00291–0·00672)	0·00325 (0·00190–0·00399)	0·00315 (0·00186–0·00479)	0·00330 (0·00227–0·00427)	22	13	21	27	24	20
Encephalitis	0·00334 (0·00288–0·00424)	0·00300 (0·00249–0·00363)	0·00759 (0·00626–0·0113)	0·00714 (0·00567–0·00831)	0·00422 (0·00328–0·00586)	0·00165 (0·00132–0·00222)	23	25	17	14	19	35
Leukaemia	0·00324 (0·00276–0·00369)	0·00828 (0·00743–0·00901)	0·00997 (0·00833–0·0120)	0·00529 (0·00453–0·00625)	0·00242 (0·00195–0·00299)	0·00270 (0·00199–0·00347)	24	12	13	21	27	24
Falls	0·00314 (0·00217–0·00398)	0·00506 (0·00431–0·00661)	0·00852 (0·00651–0·0101)	0·00592 (0·00372–0·00740)	0·00404 (0·00268–0·00547)	0·00159 (0·00115–0·00210)	25	19	16	17	21	37
Fire, heat, and hot substances	0·00278 (0·00197–0·00380)	0·00548 (0·00494–0·00630)	0·00464 (0·00373–0·00574)	0·00273 (0·00177–0·00384)	0·00224 (0·00150–0·00310)	0·00294 (0·00207–0·00412)	26	18	22	32	29	23
Other malignant neoplasms	0·00264 (0·00216–0·00316)	0·00902 (0·00812–0·00997)	0·00592 (0·00540–0·00639)	0·00325 (0·00293–0·00358)	0·00222 (0·00179–0·00268)	0·00241 (0·00179–0·00314)	27	10	19	26	30	26
Conflict and terrorism	0·00254 (0·00207–0·00307)	0·0000360 (0·0000311–0·0000410)	0·000932 (0·000779–0·00111)	0·00102 (0·000832–0·00122)	0·000231 (0·000189–0·000278)	0·00435 (0·00349–0·00536)	28	79	47	45	65	17
Endocrine, metabolic, blood, and immune disorders	0·00248 (0·00198–0·00288)	0·0214 (0·0189–0·0315)	0·0104 (0·00877–0·0125)	0·00488 (0·00390–0·00568)	0·00233 (0·00176–0·00281)	0·00114 (0·000857–0·00141)	29	6	12	22	28	44
Acute hepatitis	0·00246 (0·00185–0·00329)	0·000194 (0·000158–0·000224)	0·00171 (0·00124–0·00230)	0·00225 (0·00160–0·00308)	0·00416 (0·00290–0·00601)	0·00166 (0·00123–0·00226)	30	64	39	36	20	33
Interpersonal violence	0·00245 (0·00193–0·00286)	0·0182 (0·0165–0·0197)	0·00450 (0·00404–0·00493)	0·00308 (0·00270–0·00340)	0·00181 (0·00135–0·00217)	0·00224 (0·00160–0·00281)	31	8	24	29	32	27
Exposure to mechanical forces	0·00228 (0·00113–0·00332)	0·00264 (0·00246–0·00289)	0·00375 (0·00258–0·00479)	0·00328 (0·00153–0·00439)	0·00194 (0·000907–0·00324)	0·00213 (0·00102–0·00339)	32	29	26	25	31	29
Poisonings	0·00210 (0·00171–0·00275)	0·00176 (0·00160–0·00189)	0·00359 (0·00320–0·00405)	0·00188 (0·00143–0·00227)	0·00180 (0·00140–0·00228)	0·00224 (0·00173–0·00319)	33	32	27	38	33	28
Adverse effects of medical treatment	0·00209 (0·00159–0·00256)	0·00315 (0·00262–0·00350)	0·00205 (0·00170–0·00238)	0·00144 (0·00124–0·00168)	0·00169 (0·00132–0·00196)	0·00246 (0·00171–0·00320)	34	24	37	42	36	25
Animal contact	0·00191 (0·00127–0·00275)	0·000468 (0·000421–0·000515)	0·000583 (0·000476–0·000808)	0·00114 (0·000814–0·00161)	0·00255 (0·00136–0·00383)	0·00185 (0·00131–0·00270)	35	54	59	44	26	31
Stroke	0·00190 (0·00160–0·00256)	0·00284 (0·00246–0·00333)	0·00168 (0·00142–0·00216)	0·00344 (0·00245–0·00509)	0·00163 (0·00136–0·00199)	0·00165 (0·00132–0·00233)	36	27	40	24	38	34
Chronic kidney disease	0·00175 (0·00154–0·00200)	0·00271 (0·00243–0·00297)	0·00237 (0·00216–0·00259)	0·00230 (0·00201–0·00259)	0·00167 (0·00144–0·00195)	0·00160 (0·00133–0·00192)	37	28	36	34	37	36
Brain and nervous system cancer	0·00169 (0·00130–0·00197)	0·00836 (0·00594–0·00956)	0·00690 (0·00448–0·00806)	0·00290 (0·00208–0·00352)	0·00170 (0·00132–0·00204)	0·000963 (0·000684–0·00135)	38	11	18	30	35	48
Idiopathic epilepsy	0·00163 (0·00140–0·00201)	0·00338 (0·00286–0·00385)	0·00320 (0·00252–0·00390)	0·00179 (0·00152–0·00245)	0·00171 (0·00137–0·00241)	0·00143 (0·00119–0·00170)	39	23	29	39	34	39
Cardiomyopathy and myocarditis	0·00148 (0·00124–0·00191)	0·00759 (0·00621–0·00830)	0·00456 (0·00375–0·00522)	0·00289 (0·00235–0·00368)	0·000906 (0·000732–0·00117)	0·00114 (0·000841–0·00166)	40	14	23	31	47	45
Cirrhosis and other chronic liver diseases	0·00146 (0·00113–0·00189)	0·000757 (0·000591–0·000905)	0·00137 (0·00108–0·00178)	0·00157 (0·00118–0·00209)	0·00143 (0·00101–0·00203)	0·00146 (0·00108–0·00193)	41	45	42	41	40	38
Other cardiovascular and circulatory diseases	0·00145 (0·00121–0·00174)	0·00454 (0·00372–0·00541)	0·00245 (0·00214–0·00287)	0·00318 (0·00229–0·00456)	0·00121 (0·00100–0·00148)	0·00103 (0·000783–0·00125)	42	21	35	28	43	47
Dengue	0·00132 (0·000318–0·00176)	0·00000671 (0·00000431–0·0000106)	0·00251 (0·000661–0·00376)	0·00578 (0·00148–0·00796)	0·00124 (0·000262–0·00186)	0·000174 (0·0000373–0·000256)	43	84	33	18	42	71
Other unintentional injuries	0·00131 (0·00101–0·00161)	0·000969 (0·000771–0·00128)	0·00185 (0·00169–0·00206)	0·00128 (0·00111–0·00145)	0·00145 (0·000808–0·00192)	0·00121 (0·000953–0·00161)	44	40	38	43	39	42
Other neglected tropical diseases	0·00129 (0·000709–0·00399)	0·000296 (0·000121–0·000364)	0·000481 (0·000352–0·000799)	0·000540 (0·000342–0·00123)	0·000831 (0·000488–0·00212)	0·00180 (0·000890–0·00611)	45	60	61	57	49	32
Asthma	0·00121 (0·000970–0·00151)	0·000791 (0·000639–0·000942)	0·000591 (0·000488–0·000804)	0·00177 (0·00142–0·00210)	0·000898 (0·000713–0·00112)	0·00128 (0·000909–0·00175)	46	44	58	40	48	40
Varicella and herpes zoster	0·00103 (0·000891–0·00120)	0·000625 (0·000456–0·00116)	0·000928 (0·000725–0·00115)	0·000809 (0·000691–0·000968)	0·000951 (0·000767–0·00119)	0·00115 (0·000941–0·00139)	47	48	48	48	44	43
Urinary diseases and male infertility	0·00101 (0·000820–0·00120)	0·00109 (0·000942–0·00126)	0·00104 (0·000916–0·00120)	0·000870 (0·000755–0·00107)	0·00131 (0·000910–0·00164)	0·000880 (0·000737–0·00102)	48	38	46	47	41	49
Upper respiratory infections	0·000796 (0·000199–0·00183)	0·000651 (0·000573–0·000809)	0·000822 (0·000633–0·00164)	0·000608 (0·000464–0·000864)	0·000446 (0·000124–0·000823)	0·00104 (0·0000540–0·00283)	49	47	51	52	52	46
Other neurological disorders	0·000744 (0·000610–0·000872)	0·00622 (0·00544–0·00676)	0·00330 (0·00298–0·00365)	0·000937 (0·000817–0·00110)	0·000657 (0·000473–0·000876)	0·000499 (0·000353–0·000628)	50	16	28	46	50	54
Diphtheria	0·000722 (0·000472–0·00104)	0·00000857 (0·00000700–0·0000105)	0·0000200 (0·0000142–0·0000269)	0·0000608 (0·0000480–0·0000760)	0·000163 (0·000102–0·000255)	0·00126 (0·000811–0·00182)	51	83	86	79	67	41
Bacterial skin diseases	0·000719 (0·000452–0·000863)	0·000373 (0·000244–0·000540)	0·000646 (0·000416–0·000874)	0·000728 (0·000374–0·000903)	0·000949 (0·000446–0·00129)	0·000600 (0·000466–0·000723)	52	56	54	49	46	51
Other nutritional deficiencies	0·000706 (0·000515–0·000939)	0·000161 (0·000146–0·000177)	0·000602 (0·000512–0·000701)	0·000597 (0·000514–0·000713)	0·000951 (0·000635–0·00139)	0·000615 (0·000429–0·000858)	53	67	57	53	45	50
Inguinal, femoral, and abdominal hernia	0·000559 (0·000438–0·000672)	0·00119 (0·000876–0·00143)	0·000776 (0·000584–0·000987)	0·000724 (0·000532–0·00100)	0·000436 (0·000327–0·000546)	0·000561 (0·000360–0·000718)	54	36	52	50	53	53
Diabetes mellitus	0·000536 (0·000443–0·000627)	0·000848 (0·000709–0·00109)	0·000610 (0·000533–0·000704)	0·000516 (0·000448–0·000612)	0·000459 (0·000358–0·000546)	0·000574 (0·000444–0·000713)	55	42	55	58	51	52
Other transport injuries	0·000471 (0·000307–0·000592)	0·00101 (0·000901–0·00112)	0·000867 (0·000739–0·00103)	0·000565 (0·000467–0·000683)	0·000375 (0·000274–0·000470)	0·000467 (0·000207–0·000657)	56	39	49	56	55	56
Other digestive diseases	0·000400 (0·000265–0·000596)	0·000515 (0·000360–0·000683)	0·000570 (0·000353–0·000755)	0·000489 (0·000364–0·000656)	0·000290 (0·000181–0·000394)	0·000427 (0·000178–0·000797)	57	51	60	59	59	59
Non-Hodgkin's lymphoma	0·000381 (0·000316–0·000441)	0·000955 (0·000816–0·00112)	0·00113 (0·00100–0·00129)	0·000581 (0·000497–0·000692)	0·000364 (0·000299–0·000435)	0·000286 (0·000181–0·000376)	58	41	44	55	56	66
Environmental heat and cold exposure	0·000358 (0·000151–0·000534)	0·000675 (0·000634–0·000714)	0·000443 (0·000347–0·000512)	0·000313 (0·000109–0·000480)	0·000393 (0·0000862–0·000718)	0·000339 (0·000175–0·000500)	59	46	63	62	54	62
Rabies	0·000355 (0·000120–0·000574)	0·0000161 (0·0000136–0·0000192)	0·000105 (0·0000352–0·000186)	0·000134 (0·0000377–0·000256)	0·000289 (0·000119–0·000517)	0·000468 (0·000150–0·000863)	60	81	74	70	60	55
Kidney cancer	0·000348 (0·000281–0·000411)	0·00130 (0·00112–0·00145)	0·00138 (0·00125–0·00152)	0·000632 (0·000555–0·000709)	0·000236 (0·000188–0·000285)	0·000263 (0·000163–0·000362)	61	34	41	51	64	67
Liver cancer	0·000344 (0·000264–0·000420)	0·00114 (0·000888–0·00131)	0·000861 (0·000736–0·00102)	0·000590 (0·000456–0·000745)	0·000303 (0·000227–0·000393)	0·000259 (0·000180–0·000347)	62	37	50	54	58	68
Endocarditis	0·000335 (0·000228–0·000444)	0·000412 (0·000255–0·000487)	0·000480 (0·000383–0·000622)	0·000470 (0·000347–0·000632)	0·000284 (0·000190–0·000376)	0·000320 (0·000186–0·000458)	63	55	62	61	61	63
Other chronic respiratory diseases	0·000335 (0·000188–0·000501)	0·00127 (0·000929–0·00143)	0·000605 (0·000509–0·000747)	0·000488 (0·000410–0·000646)	0·000260 (0·000161–0·000364)	0·000303 (0·0000952–0·000565)	64	35	56	60	62	64

Rheumatic heart disease	0·000328<br>(0·000267–0·000422)	0·000150<br>(0·000123–0·000178)	0·000186<br>(0·000148–0·000237)	0·000259<br>(0·000205–0·000402)	0·000340<br>(0·000255–0·000443)	0·000350<br>(0·000268–0·000476)	65	69	68	64	57	61
Leishmaniasis	0·000308<br>(0·000105–0·000895)	0·0000139<br>(0·000000000413–0·000133)	0·000124<br>(0·0000000455–0·000687)	0·0000886<br>(0·000000141–0·000498)	0·000258<br>(0·0000432–0·00125)	0·000409<br>(0·000176–0·000872)	66	82	72	75	63	60
Upper digestive system diseases	0·000300<br>(0·000196–0·000499)	0·0000697<br>(0·0000570–0·0000777)	0·000119<br>(0·0000970–0·000145)	0·000146<br>(0·000121–0·000174)	0·000139<br>(0·0000918–0·000228)	0·000444<br>(0·000270–0·000781)	67	74	73	69	69	58
Intestinal nematode infections	0·000282<br>(0·000216–0·000353)	0·00000349<br>(0·000000829–0·00000901)	0·0000259<br>(0·0000169–0·0000365)	0·0000588<br>(0·0000469–0·0000710)	0·000110<br>(0·0000859–0·000136)	0·000455<br>(0·000343–0·000573)	68	86	85	80	71	57
Appendicitis	0·000230<br>(0·000141–0·000320)	0·000116<br>(0·0000825–0·000145)	0·000149<br>(0·000114–0·000193)	0·000164<br>(0·000126–0·000220)	0·000158<br>(0·000108–0·000221)	0·000293<br>(0·000122–0·000447)	69	70	70	67	68	65
Chronic obstructive pulmonary disease	0·000223<br>(0·000150–0·000371)	0·000315<br>(0·000254–0·000372)	0·000231<br>(0·000192–0·000309)	0·000272<br>(0·000217–0·000362)	0·000170<br>(0·000123–0·000237)	0·000236<br>(0·000126–0·000478)	70	59	67	63	66	70
Other intestinal infectious diseases	0·000169<br>(0·000105–0·000244)	0·0000682<br>(0·000000788–0·000152)	0·0000647<br>(0·0000109–0·000129)	0·0000707<br>(0·00000967–0·000146)	0·000103<br>(0·0000485–0·000163)	0·000239<br>(0·000157–0·000344)	71	75	80	78	73	69
Inflammatory bowel disease	0·000142<br>(0·0000819–0·000202)	0·000817<br>(0·000644–0·000931)	0·000362<br>(0·000270–0·000460)	0·000174<br>(0·000134–0·000211)	0·000107<br>(0·0000580–0·000159)	0·000129<br>(0·0000415–0·000224)	72	43	64	66	72	74
Motor neuron disease	0·000122<br>(0·000106–0·000138)	0·00386<br>(0·00352–0·00416)	0·00125<br>(0·00105–0·00152)	0·000175<br>(0·000150–0·000208)	0·0000503<br>(0·0000407–0·0000649)	0·0000153<br>(0·0000119–0·0000203)	73	22	43	65	77	89
Gallbladder and biliary diseases	0·000113<br>(0·0000564–0·000168)	0·0000630<br>(0·0000426–0·0000783)	0·0000891<br>(0·0000607–0·000132)	0·0000869<br>(0·0000639–0·000118)	0·0000642<br>(0·0000366–0·000102)	0·000149<br>(0·0000534–0·000233)	74	77	76	76	74	72
Exposure to forces of nature	0·000112<br>(0·0000912–0·000135)	0·000153<br>(0·000132–0·000175)	0·0000631<br>(0·0000527–0·0000751)	0·000108<br>(0·0000881–0·000129)	0·000118<br>(0·0000965–0·000142)	0·000111<br>(0·0000891–0·000137)	75	68	82	74	70	76
Yellow fever	0·0000974<br>(0·0000349–0·000214)	0<br>(0–0)	0·0000196<br>(0·00000222–0·0000813)	0·0000144<br>(0·00000476–0·0000328)	0·0000600<br>(0·0000167–0·000152)	0·000146<br>(0·0000520–0·000329)	76	94	87	86	75	73
Ebola virus disease	0·0000646<br>(0·0000492–0·0000814)	0<br>(0–0)	0<br>(0–0)	0<br>(0–0)	0<br>(0–0)	0·000122<br>(0·0000913–0·000156)	77	94	95	95.5	95.5	75
Testicular cancer	0·0000639<br>(0·0000444–0·000137)	0·000197<br>(0·000121–0·000668)	0·000238<br>(0·000168–0·000568)	0·000133<br>(0·0000902–0·000321)	0·0000578<br>(0·0000418–0·0000979)	0·0000375<br>(0·0000223–0·0000839)	78	62	66	71	76	82
Vascular intestinal disorders	0·0000592<br>(0·0000339–0·000102)	0·000195<br>(0·000151–0·000226)	0·0000640<br>(0·0000483–0·0000823)	0·0000333<br>(0·0000235–0·0000488)	0·0000366<br>(0·0000238–0·0000567)	0·0000756<br>(0·0000327–0·000143)	79	63	81	84	81	78
Otitis media	0·0000555<br>(0·0000140–0·000181)	0·000107<br>(0·0000769–0·000155)	0·0000290<br>(0·0000170–0·0000562)	0·00000828<br>(0·00000607–0·0000117)	0·0000127<br>(0·00000558–0·0000304)	0·0000920<br>(0·0000169–0·000321)	80	71	84	87	88	77
Interstitial lung disease and pulmonary sarcoidosis	0·0000553<br>(0·0000376–0·0000821)	0·000612<br>(0·000514–0·000830)	0·000144<br>(0·000106–0·000181)	0·0000828<br>(0·0000644–0·000122)	0·0000437<br>(0·0000278–0·0000680)	0·0000387<br>(0·0000177–0·0000712)	81	49	71	77	78	81
Acute glomerulonephritis	0·0000522<br>(0·0000208–0·0000741)	0·0000246<br>(0·0000214–0·0000275)	0·0000755<br>(0·0000531–0·000100)	0·000153<br>(0·0000671–0·000198)	0·0000386<br>(0·0000139–0·0000551)	0·0000332<br>(0·00000945–0·0000607)	82	80	77	68	79	84
Other neoplasms	0·0000502<br>(0·0000415–0·0000624)	0·000591<br>(0·000424–0·000694)	0·000292<br>(0·000226–0·000341)	0·000123<br>(0·0000997–0·000149)	0·0000381<br>(0·0000305–0·0000521)	0·0000146<br>(0·00000972–0·0000272)	83	50	65	73	80	90
Pancreatitis	0·0000439<br>(0·0000137–0·0000755)	0·0000553<br>(0·0000406–0·0000644)	0·0000395<br>(0·0000282–0·0000493)	0·0000264<br>(0·0000183–0·0000384)	0·0000282<br>(0·0000130–0·0000512)	0·0000571<br>(0·00000999–0·000107)	84	78	83	85	83	79
Executions and police conflict	0·0000433<br>(0·0000367–0·0000536)	0·000180<br>(0·000165–0·000192)	0·0000962<br>(0·0000844–0·000108)	0·000130<br>(0·0000970–0·000203)	0·0000283<br>(0·0000250–0·0000320)	0·0000239<br>(0·0000204–0·0000279)	85	65	75	72	82	86
Schistosomiasis	0·0000306<br>(0·0000216–0·0000413)	0·000000158<br>(0·0000000000891–0·000000694)	0·00000125<br>(0·000000369–0·00000282)	0·00000212<br>(0·00000114–0·00000349)	0·00000870<br>(0·00000430–0·0000146)	0·0000524<br>(0·0000362–0·0000704)	86	89	91	90	89	80
Hodgkin lymphoma	0·0000298<br>(0·0000165–0·0000432)	0·0000793<br>(0·0000725–0·0000853)	0·0000651<br>(0·0000536–0·0000762)	0·0000396<br>(0·0000304–0·0000475)	0·0000216<br>(0·0000136–0·0000342)	0·0000289<br>(0·0000104–0·0000486)	87	72	78	81	84	85
Malignant skin melanoma	0·0000281<br>(0·0000149–0·0000558)	0·000481<br>(0·000115–0·00160)	0·000158<br>(0·0000612–0·000386)	0·0000358<br>(0·0000275–0·0000584)	0·0000165<br>(0·0000122–0·0000285)	0·0000170<br>(0·00000742–0·0000511)	88	53	69	83	85	88
Cystic echinococcosis	0·0000239<br>(0·00000732–0·0000443)	0·00000152<br>(0–0·00000488)	0·00000919<br>(0·000000369–0·0000244)	0·00000679<br>(0·000000271–0·0000187)	0·0000145<br>(0·00000353–0·0000300)	0·0000348<br>(0·0000113–0·0000611)	89	87	88	88	86	83
Other skin and subcutaneous diseases	0·0000167<br>(0·0000132–0·0000222)	0·0000698<br>(0·0000516–0·000102)	0·0000648<br>(0·0000453–0·0000917)	0·0000361<br>(0·0000273–0·0000457)	0·0000140<br>(0·00000943–0·0000218)	0·00000959<br>(0·00000606–0·0000167)	90	73	79	82	87	91
African trypanosomiasis	0·0000109<br>(0·00000173–0·0000481)	0<br>(0–0)	0<br>(0–0)	0·000000934<br>(0·0000000865–0·00000422)	0·000000588<br>(0·000000117–0·00000177)	0·0000201<br>(0·00000279–0·0000882)	91	94	95	91	92	87
Cysticercosis	0·00000312<br>(0·0000000327–0·00000866)	0·00000000494<br>(0–0·0000000139)	0·000000183<br>(0·00000000113–0·000000512)	0·000000629<br>(0·000000000599–0·00000186)	0·00000180<br>(0·00000000100–0·00000510)	0·00000472<br>(0·0000000544–0·0000130)	92	91	93	92	90	92
Decubitus ulcer	0·00000156<br>(0·000000635–0·00000245)	0·00000373<br>(0·00000294–0·00000557)	0·00000417<br>(0·00000164–0·00000650)	0·00000490<br>(0·00000111–0·00000718)	0·000000705<br>(0·000000343–0·00000109)	0·000000980<br>(0·000000355–0·00000239)	93	85	89	89	91	93
Chagas disease	0·000000339<br>(0·000000174–0·000000782)	0<br>(0–0)	0·00000392<br>(0·00000146–0·00000827)	0·000000562<br>(0·000000154–0·000000978)	0·000000473<br>(0·000000186–0·00000156)	0·0000000104<br>(0·00000000270–0·0000000373)	94	94	90	93	93	94
Zika virus disease	0·0000000250<br>(0·0000000141–0·0000000385)	0·000000129<br>(0–0·000000375)	0·000000183<br>(0·0000000653–0·000000354)	0·0000000658<br>(0·0000000272–0·000000115)	0·0000000276<br>(0·0000000117–0·0000000484)	0·00000000162<br>(0–0·00000000558)	95	90	92	94	94	95
Drug use disorders	0<br>(0–0)	0<br>(0–0)	0<br>(0–0)	0<br>(0–0)	0<br>(0–0)	0<br>(0–0)	96	94	95	95.5	95.5	96

Table S10b. Cause-fraction (cause-specific proportion of total deaths) and cause rank for level-3 causes of death for children under 5 years of age, globally and for socio-demographic index (SDI) quintiles, in 2019, males

	Cause Fraction (2019, males)						Rank (2019, males)					
Cause of death	Global	High SDI	High-middle SDI	Middle SDI	Low-middle SDI	Low SDI	Global	High SDI	High-middle SDI	Middle SDI	Low-middle SDI	Low SDI
Neonatal disorders	0·394 (0·376–0·409)	0·431 (0·395–0·446)	0·414 (0·393–0·435)	0·424 (0·401–0·440)	0·477 (0·452–0·496)	0·338 (0·321–0·354)	1	1	1	1	1	1
Lower respiratory infections	0·125 (0·113–0·136)	0·0246 (0·0224–0·0264)	0·0798 (0·0718–0·0877)	0·120 (0·109–0·130)	0·124 (0·112–0·138)	0·131 (0·114–0·147)	2	5	3	3	2	2
Diarrhoeal diseases	0·0974 (0·0814–0·113)	0·00483 (0·00404–0·00591)	0·0214 (0·0174–0·0262)	0·0529 (0·0451–0·0612)	0·0781 (0·0646–0·0936)	0·126 (0·102–0·147)	3	20	5	4	4	3
Congenital anomalies	0·0959 (0·0790–0·126)	0·246 (0·225–0·302)	0·223 (0·191–0·250)	0·144 (0·127–0·165)	0·0896 (0·0802–0·108)	0·0766 (0·0447–0·118)	4	2	2	2	3	5
Malaria	0·0638 (0·0306–0·110)	0·000000118 (0·0000000429–0·000000388)	0·0144 (0·00760–0·0218)	0·0320 (0·0194–0·0476)	0·0425 (0·0205–0·0708)	0·0879 (0·0393–0·155)	5	90	8	5	5	4
Meningitis	0·0228 (0·0193–0·0278)	0·00603 (0·00530–0·00658)	0·0108 (0·00950–0·0121)	0·0126 (0·0111–0·0150)	0·0166 (0·0139–0·0215)	0·0299 (0·0248–0·0362)	6	16	12	10	6	6
Whooping cough	0·0181 (0·00833–0·0321)	0·00176 (0·000860–0·00341)	0·00781 (0·00344–0·0163)	0·0152 (0·00737–0·0275)	0·0158 (0·00667–0·0313)	0·0210 (0·00899–0·0395)	7	31	16	8	7	8
Sexually transmitted infections excluding HIV	0·0164 (0·00556–0·0342)	0·00297 (0·000928–0·00638)	0·0115 (0·00369–0·0246)	0·0178 (0·00566–0·0378)	0·0138 (0·00447–0·0298)	0·0180 (0·00624–0·0372)	8	25	10	6	8	10
Protein-energy malnutrition	0·0163 (0·0134–0·0193)	0·000514 (0·000451–0·000592)	0·00343 (0·00294–0·00403)	0·00834 (0·00745–0·00934)	0·0128 (0·0105–0·0154)	0·0214 (0·0168–0·0262)	9	51	27	13	9	7
Measles	0·0132 (0·00469–0·0288)	0·000350 (0·000124–0·000842)	0·00298 (0·000955–0·00668)	0·00647 (0·00248–0·0136)	0·00705 (0·00238–0·0163)	0·0191 (0·00674–0·0422)	10	58	30	16	14	9
iNTS	0·00998 (0·00571–0·0157)	0·000339 (0·000200–0·000575)	0·000718 (0·000365–0·00122)	0·00215 (0·00118–0·00363)	0·00468 (0·00259–0·00751)	0·0157 (0·00901–0·0247)	11	59	52	35	17	11
Tuberculosis	0·00922 (0·00778–0·0108)	0·000215 (0·000185–0·000252)	0·00234 (0·00204–0·00272)	0·00570 (0·00512–0·00640)	0·00782 (0·00668–0·00894)	0·0115 (0·00930–0·0139)	12	62	34	18	12	12
HIV/AIDS	0·00918 (0·00758–0·0110)	0·00164 (0·00108–0·00485)	0·00828 (0·00572–0·0105)	0·0109 (0·00889–0·0133)	0·00957 (0·00781–0·0118)	0·00871 (0·00668–0·0109)	13	32	15	12	10	13
Drowning	0·00787 (0·00656–0·0100)	0·0159 (0·0145–0·0169)	0·0172 (0·0152–0·0196)	0·0156 (0·0109–0·0178)	0·00821 (0·00678–0·0107)	0·00495 (0·00381–0·00724)	14	9	6	7	11	16
Road injuries	0·00787 (0·00618–0·0107)	0·0195 (0·0175–0·0213)	0·0169 (0·0131–0·0189)	0·0129 (0·00974–0·0148)	0·00568 (0·00461–0·00823)	0·00699 (0·00512–0·0106)	15	7	7	9	15	14
Sudden infant death syndrome	0·00559 (0·00250–0·0112)	0·0524 (0·0466–0·0568)	0·0130 (0·0104–0·0162)	0·00735 (0·00442–0·0113)	0·00475 (0·00183–0·0101)	0·00427 (0·000838–0·0117)	16	3	9	14	16	17
Foreign body	0·00516 (0·00458–0·00589)	0·0352 (0·0324–0·0372)	0·0238 (0·0208–0·0266)	0·0119 (0·0101–0·0137)	0·00434 (0·00382–0·00502)	0·00218 (0·00181–0·00277)	17	4	4	11	18	28
Typhoid and paratyphoid	0·00444 (0·00172–0·00868)	0·000164 (0·0000442–0·000427)	0·00290 (0·00103–0·00592)	0·00386 (0·00134–0·00784)	0·00707 (0·00278–0·0138)	0·00332 (0·00124–0·00649)	18	66	31	24	13	20
Hemoglobinopathies and hemolytic anaemias	0·00436 (0·00308–0·00621)	0·00123 (0·00105–0·00136)	0·00224 (0·00189–0·00278)	0·00213 (0·00160–0·00307)	0·00252 (0·00164–0·00351)	0·00614 (0·00360–0·00982)	19	35	36	36	26	15
Paralytic ileus and intestinal obstruction	0·00377 (0·00280–0·00506)	0·00774 (0·00691–0·00849)	0·00583 (0·00491–0·00699)	0·00604 (0·00437–0·00805)	0·00342 (0·00244–0·00481)	0·00317 (0·00216–0·00464)	20	14	19	17	23	21
Tetanus	0·00357 (0·00269–0·00579)	0·0000606 (0·0000351–0·000114)	0·00104 (0·000640–0·00164)	0·00195 (0·00124–0·00304)	0·00388 (0·00259–0·00613)	0·00404 (0·00282–0·00767)	21	76	47	37	21	18
Leukaemia	0·00349 (0·00277–0·00409)	0·00812 (0·00686–0·00924)	0·00974 (0·00719–0·0133)	0·00552 (0·00459–0·00701)	0·00255 (0·00200–0·00329)	0·00301 (0·00202–0·00397)	22	12	13	21	25	23
Other unspecified infectious diseases	0·00332 (0·00200–0·00449)	0·00775 (0·00475–0·0118)	0·00533 (0·00260–0·00717)	0·00339 (0·00168–0·00439)	0·00328 (0·00183–0·00542)	0·00313 (0·00197–0·00428)	23	13	21	25	24	22
Encephalitis	0·00329 (0·00273–0·00436)	0·00288 (0·00227–0·00345)	0·00737 (0·00583–0·0118)	0·00723 (0·00542–0·00888)	0·00427 (0·00320–0·00613)	0·00148 (0·00112–0·00214)	24	27	17	15	19	37
Falls	0·00302 (0·00211–0·00401)	0·00571 (0·00465–0·00879)	0·00898 (0·00614–0·0121)	0·00552 (0·00333–0·00728)	0·00372 (0·00264–0·00525)	0·00158 (0·00114–0·00222)	25	18	14	20	22	35
Other malignant neoplasms	0·00292 (0·00228–0·00350)	0·00846 (0·00740–0·00964)	0·00564 (0·00495–0·00630)	0·00334 (0·00287–0·00379)	0·00232 (0·00173–0·00293)	0·00287 (0·00200–0·00366)	26	11	20	26	28	24
Endocrine, metabolic, blood, and immune disorders	0·00261 (0·00184–0·00327)	0·0230 (0·0195–0·0373)	0·0110 (0·00887–0·0148)	0·00498 (0·00356–0·00607)	0·00252 (0·00156–0·00335)	0·00115 (0·000672–0·00151)	27	6	11	22	27	43
Fire, heat, and hot substances	0·00257 (0·00173–0·00370)	0·00544 (0·00495–0·00605)	0·00450 (0·00380–0·00530)	0·00254 (0·00171–0·00358)	0·00191 (0·00129–0·00271)	0·00276 (0·00178–0·00415)	28	19	23	32	33	25
Acute hepatitis	0·00248 (0·00174–0·00345)	0·000198 (0·000158–0·000230)	0·00183 (0·00123–0·00280)	0·00243 (0·00166–0·00344)	0·00415 (0·00268–0·00602)	0·00166 (0·00115–0·00233)	29	63	39	33	20	33
Interpersonal violence	0·00242 (0·00191–0·00284)	0·0188 (0·0171–0·0201)	0·00443 (0·00400–0·00489)	0·00306 (0·00268–0·00341)	0·00183 (0·00134–0·00228)	0·00216 (0·00150–0·00275)	30	8	24	28	34	29
Conflict and terrorism	0·00232 (0·00187–0·00282)	0·0000265 (0·0000227–0·0000304)	0·000688 (0·000572–0·000824)	0·000867 (0·000703–0·00105)	0·000175 (0·000143–0·000214)	0·00402 (0·00317–0·00504)	31	79	53	48	65	19
Exposure to mechanical forces	0·00219 (0·00111–0·00344)	0·00283 (0·00257–0·00328)	0·00360 (0·00235–0·00486)	0·00288 (0·00140–0·00428)	0·00200 (0·000922–0·00394)	0·00201 (0·00101–0·00358)	32	28	26	31	32	30
Poisonings	0·00209 (0·00155–0·00294)	0·00184 (0·00163–0·00204)	0·00361 (0·00315–0·00418)	0·00195 (0·00124–0·00243)	0·00176 (0·00120–0·00241)	0·00222 (0·00167–0·00351)	33	30	25	38	37	27
Stroke	0·00208 (0·00172–0·00285)	0·00290 (0·00246–0·00364)	0·00174 (0·00140–0·00242)	0·00400 (0·00264–0·00610)	0·00182 (0·00146–0·00230)	0·00172 (0·00132–0·00242)	34	26	40	23	35	32
Adverse effects of medical treatment	0·00199 (0·00136–0·00258)	0·00332 (0·00265–0·00381)	0·00201 (0·00156–0·00250)	0·00137 (0·00113–0·00169)	0·00149 (0·00104–0·00185)	0·00240 (0·00144–0·00328)	35	23	38	43	40	26
Brain and nervous system cancer	0·00192 (0·00132–0·00232)	0·00851 (0·00569–0·0110)	0·00669 (0·00407–0·00861)	0·00289 (0·00179–0·00386)	0·00206 (0·00137–0·00259)	0·00119 (0·000688–0·00182)	36	10	18	30	30	41
Idiopathic epilepsy	0·00171 (0·00144–0·00218)	0·00322 (0·00272–0·00366)	0·00322 (0·00256–0·00400)	0·00180 (0·00154–0·00257)	0·00162 (0·00130–0·00239)	0·00162 (0·00131–0·00197)	37	24	28	39	38	34
Chronic kidney disease	0·00166 (0·00142–0·00203)	0·00268 (0·00236–0·00297)	0·00237 (0·00213–0·00266)	0·00225 (0·00192–0·00265)	0·00178 (0·00146–0·00225)	0·00138 (0·00111–0·00182)	38	29	33	34	36	40
Animal contact	0·00164 (0·00109–0·00242)	0·000471 (0·000422–0·000523)	0·000500 (0·000412–0·000707)	0·00101 (0·000787–0·00143)	0·00229 (0·00127–0·00351)	0·00155 (0·00111–0·00243)	39	53	61	45	29	36
Other unintentional injuries	0·00161 (0·00106–0·00210)	0·00110 (0·000836–0·00165)	0·00211 (0·00188–0·00241)	0·00151 (0·00115–0·00177)	0·00201 (0·000784–0·00286)	0·00139 (0·00101–0·00201)	40	39	37	42	31	39
Cirrhosis and other chronic liver diseases	0·00149 (0·00111–0·00200)	0·000728 (0·000542–0·000915)	0·00137 (0·00103–0·00188)	0·00161 (0·00119–0·00227)	0·00151 (0·00102–0·00227)	0·00146 (0·00101–0·00206)	41	46	41	40	39	38
Cardiomyopathy and myocarditis	0·00148 (0·00119–0·00203)	0·00738 (0·00585–0·00837)	0·00468 (0·00356–0·00554)	0·00297 (0·00232–0·00402)	0·000945 (0·000730–0·00131)	0·00107 (0·000766–0·00169)	42	15	22	29	44	47
Other neglected tropical diseases	0·00140 (0·000686–0·00520)	0·000268 (0·0000755–0·000352)	0·000500 (0·000329–0·000959)	0·000566 (0·000313–0·00159)	0·000899 (0·000451–0·00287)	0·00196 (0·000826–0·00795)	43	61	60	56	47	31
Dengue	0·00129 (0·000237–0·00178)	0·00000582 (0·00000288–0·0000115)	0·00260 (0·000519–0·00426)	0·00560 (0·00110–0·00813)	0·00119 (0·000191–0·00194)	0·000157 (0·0000232–0·000279)	44	84	32	19	42	71
Other cardiovascular and circulatory diseases	0·00127 (0·00104–0·00162)	0·00426 (0·00326–0·00503)	0·00230 (0·00192–0·00277)	0·00313 (0·00198–0·00481)	0·00113 (0·000863–0·00145)	0·000729 (0·000531–0·000946)	45	21	35	27	43	50
Asthma	0·00112 (0·000896–0·00136)	0·000820 (0·000685–0·00100)	0·000502 (0·000406–0·000755)	0·00160 (0·00133–0·00198)	0·000917 (0·000715–0·00116)	0·00114 (0·000812–0·00149)	46	44	59	41	46	44
Urinary diseases and male infertility	0·00106 (0·000857–0·00128)	0·00102 (0·000837–0·00124)	0·00106 (0·000877–0·00130)	0·000889 (0·000757–0·00111)	0·00133 (0·000898–0·00174)	0·000966 (0·000801–0·00114)	47	41	46	46	41	48
Varicella and herpes zoster	0·000984 (0·000830–0·00116)	0·000625 (0·000359–0·00126)	0·000890 (0·000591–0·00120)	0·000783 (0·000653–0·000954)	0·000922 (0·000699–0·00119)	0·00108 (0·000857–0·00135)	48	48	50	49	45	46
Upper respiratory infections	0·000848 (0·000216–0·00194)	0·000741 (0·000645–0·000911)	0·000893 (0·000687–0·00172)	0·000632 (0·000480–0·000891)	0·000474 (0·000139–0·000877)	0·00111 (0·0000670–0·00299)	49	45	49	52	52	45
Other neurological disorders	0·000817 (0·000679–0·000966)	0·00581 (0·00496–0·00643)	0·00302 (0·00270–0·00344)	0·000875 (0·000758–0·00106)	0·000708 (0·000530–0·000881)	0·000640 (0·000463–0·000867)	50	17	29	47	48	51
Inguinal, femoral, and abdominal hernia	0·000782 (0·000588–0·000993)	0·00140 (0·000980–0·00173)	0·00106 (0·000805–0·00137)	0·00106 (0·000717–0·00154)	0·000575 (0·000413–0·000743)	0·000794 (0·000462–0·00107)	51	34	45	44	51	49
Diphtheria	0·000679 (0·000415–0·00107)	0·00000797 (0·00000626–0·0000105)	0·0000198 (0·0000125–0·0000293)	0·0000618 (0·0000473–0·0000804)	0·000161 (0·0000867–0·000291)	0·00118 (0·000704–0·00187)	52	83	87	77	66	42
Diabetes mellitus	0·000568 (0·000454–0·000700)	0·000872 (0·000703–0·00113)	0·000569 (0·000481–0·000690)	0·000506 (0·000426–0·000632)	0·000464 (0·000354–0·000574)	0·000635 (0·000476–0·000831)	53	42	56	58	53	52
Other nutritional deficiencies	0·000526 (0·000392–0·000705)	0·000154 (0·000135–0·000174)	0·000578 (0·000468–0·000715)	0·000596 (0·000495–0·000729)	0·000667 (0·000422–0·000995)	0·000435 (0·000287–0·000638)	54	67	55	55	49	57
Other transport injuries	0·000516 (0·000332–0·000680)	0·00119 (0·00101–0·00140)	0·000973 (0·000746–0·00122)	0·000655 (0·000480–0·000849)	0·000375 (0·000262–0·000502)	0·000517 (0·000215–0·000759)	55	36	48	50	55	54
Bacterial skin diseases	0·000476 (0·000248–0·000601)	0·000358 (0·000189–0·000587)	0·000554 (0·000305–0·000839)	0·000514 (0·000243–0·000672)	0·000628 (0·000253–0·000945)	0·000379 (0·000225–0·000474)	56	56	57	57	50	60
Non-Hodgkin's lymphoma	0·000454 (0·000366–0·000535)	0·00104 (0·000865–0·00130)	0·00123 (0·00104–0·00146)	0·000641 (0·000529–0·000782)	0·000454 (0·000363–0·000557)	0·000349 (0·000205–0·000470)	57	40	44	51	54	64
Rabies	0·000416 (0·000118–0·000808)	0·0000109 (0·00000822–0·0000149)	0·000121 (0·0000304–0·000230)	0·000137 (0·0000354–0·000269)	0·000309 (0·000104–0·000615)	0·000574 (0·000146–0·00133)	58	82	71	71	60	53
Other digestive diseases	0·000412 (0·000268–0·000599)	0·000510 (0·000329–0·000740)	0·000552 (0·000336–0·000790)	0·000495 (0·000360–0·000671)	0·000313 (0·000199–0·000444)	0·000434 (0·000168–0·000774)	59	52	58	59	59	58
Kidney cancer	0·000410 (0·000307–0·000505)	0·00116 (0·00100–0·00136)	0·00125 (0·00108–0·00147)	0·000612 (0·000503–0·000724)	0·000284 (0·000213–0·000354)	0·000363 (0·000197–0·000520)	60	37	43	54	62	62
Environmental heat and cold exposure	0·000370 (0·000150–0·000547)	0·000717 (0·000670–0·000769)	0·000458 (0·000371–0·000552)	0·000323 (0·000104–0·000475)	0·000372 (0·0000813–0·000678)	0·000370 (0·000173–0·000567)	61	47	63	62	56	61
Leishmaniasis	0·000369 (0·000127–0·00108)	0·0000162 (0·000000000476–0·000155)	0·000141 (0·0000000539–0·000797)	0·000103 (0·000000166–0·000585)	0·000313 (0·0000537–0·00151)	0·000491 (0·000215–0·00105)	62	81	69	74	58	55
Endocarditis	0·000363 (0·000239–0·000494)	0·000411 (0·000238–0·000496)	0·000487 (0·000364–0·000651)	0·000480 (0·000331–0·000688)	0·000315 (0·000206–0·000438)	0·000351 (0·000202–0·000513)	63	54	62	60	57	63
Liver cancer	0·000326 (0·000244–0·000402)	0·00115 (0·000845–0·00134)	0·000862 (0·000718–0·00106)	0·000630 (0·000467–0·000837)	0·000279 (0·000208–0·000356)	0·000225 (0·000147–0·000308)	64	38	51	53	63	67
Rheumatic heart disease	0·000312 (0·000241–0·000422)	0·000134 (0·000103–0·000167)	0·000167 (0·000120–0·000231)	0·000249 (0·000181–0·000370)	0·000305 (0·000225–0·000419)	0·000343 (0·000244–0·000492)	65	69	68	63	61	65
Upper digestive system diseases	0·000283 (0·000172–0·000484)	0·0000680 (0·0000532–0·0000771)	0·000108 (0·0000828–0·000140)	0·000156 (0·000129–0·000188)	0·000128 (0·0000825–0·000215)	0·000415 (0·000227–0·000768)	66	74	74	69	69	59
Intestinal nematode infections	0·000276 (0·000213–0·000351)	0·00000340 (0·000000389–0·0000112)	0·0000265 (0·0000139–0·0000425)	0·0000591 (0·0000451–0·0000748)	0·000114 (0·0000850–0·000147)	0·000442 (0·000332–0·000568)	67	86	85	79	70	56
Other chronic respiratory diseases	0·000274 (0·000164–0·000410)	0·00141 (0·00114–0·00158)	0·000584 (0·000481–0·000754)	0·000480 (0·000394–0·000630)	0·000248 (0·000150–0·000371)	0·000193 (0·0000584–0·000381)	68	33	54	61	64	68
Appendicitis	0·000204 (0·000109–0·000305)	0·000113 (0·0000644–0·000170)	0·000141 (0·0000930–0·000215)	0·000175 (0·000128–0·000247)	0·000143 (0·0000898–0·000212)	0·000249 (0·0000724–0·000414)	69	71	70	68	68	66
Chronic obstructive pulmonary disease	0·000146 (0·000107–0·000220)	0·000315 (0·000259–0·000376)	0·000203 (0·000163–0·000264)	0·000237 (0·000178–0·000320)	0·000150 (0·000102–0·000232)	0·000114 (0·0000597–0·000224)	70	60	67	65	67	74
Other intestinal infectious diseases	0·000131 (0·0000641–0·000219)	0·0000562 (0–0·000172)	0·0000528 (0·000000998–0·000134)	0·0000582 (0·00000122–0·000158)	0·0000792 (0·0000215–0·000154)	0·000185 (0·0000994–0·000294)	71	77	82	80	75	70
Yellow fever	0·000129 (0·0000451–0·000290)	0 (0–0)	0·0000247 (0·00000237–0·000117)	0·0000190 (0·00000603–0·0000442)	0·0000827 (0·0000212–0·000227)	0·000191 (0·0000648–0·000463)	72	94	86	86	74	69

Inflammatory bowel disease	0·000123 (0·0000699–0·000193)	0·000849 (0·000600–0·00101)	0·000368 (0·000250–0·000504)	0·000179 (0·000126–0·000231)	0·0000921 (0·0000475–0·000140)	0·0000980 (0·0000279–0·000212)	73	43	65	67	73	76
Motor neuron disease	0·000122 (0·000103–0·000144)	0·00374 (0·00331–0·00408)	0·00130 (0·000997–0·00169)	0·000183 (0·000145–0·000240)	0·0000490 (0·0000374–0·0000675)	0·0000103 (0·00000643–0·0000167)	74	22	42	66	78	90
Testicular cancer	0·000118 (0·0000822–0·000253)	0·000355 (0·000218–0·00120)	0·000426 (0·000302–0·00102)	0·000238 (0·000163–0·000575)	0·000108 (0·0000783–0·000183)	0·0000692 (0·0000413–0·000155)	75	57	64	64	72	78
Exposure to forces of nature	0·000105 (0·0000850–0·000128)	0·000140 (0·000120–0·000161)	0·0000581 (0·0000483–0·0000696)	0·0000991 (0·0000804–0·000120)	0·000111 (0·0000905–0·000136)	0·000106 (0·0000832–0·000132)	76	68	81	75	71	75
Vascular intestinal disorders	0·0000946 (0·0000503–0·000175)	0·000193 (0·000150–0·000237)	0·0000681 (0·0000493–0·000101)	0·0000385 (0·0000255–0·0000658)	0·0000575 (0·0000364–0·0000972)	0·000129 (0·0000530–0·000258)	77	64	77	81	76	72
Otitis media	0·0000689 (0·0000128–0·000259)	0·000128 (0·0000893–0·000193)	0·0000306 (0·0000159–0·0000670)	0·00000893 (0·00000626–0·0000131)	0·0000151 (0·00000513–0·0000449)	0·000115 (0·0000132–0·000453)	78	70	84	87	86	73
Interstitial lung disease and pulmonary sarcoidosis	0·0000540 (0·0000317–0·0000891)	0·000540 (0·000400–0·000749)	0·000119 (0·0000807–0·000156)	0·0000731 (0·0000539–0·000101)	0·0000493 (0·0000255–0·0000917)	0·0000379 (0·0000132–0·0000825)	79	50	72	76	77	81
Other neoplasms	0·0000511 (0·0000366–0·0000688)	0·000606 (0·000357–0·000821)	0·000297 (0·000194–0·000375)	0·000123 (0·0000909–0·000159)	0·0000376 (0·0000249–0·0000566)	0·0000147 (0·00000875–0·0000316)	80	49	66	72	79	89
Ebola virus disease	0·0000501 (0·0000379–0·0000635)	0 (0–0)	0 (0–0)	0 (0–0)	0 (0–0)	0·0000949 (0·0000703–0·000122)	81	94	95	95.5	95.5	77
Gallbladder and biliary diseases	0·0000495 (0·0000247–0·0000791)	0·0000609 (0·0000358–0·0000781)	0·0000726 (0·0000444–0·000111)	0·0000617 (0·0000349–0·0000854)	0·0000352 (0·0000198–0·0000566)	0·0000526 (0·0000182–0·0000994)	82	75	76	78	81	79
Acute glomerulonephritis	0·0000464 (0·0000136–0·0000654)	0·0000210 (0·0000184–0·0000240)	0·0000638 (0·0000408–0·0000873)	0·000138 (0·0000492–0·000177)	0·0000364 (0·00000979–0·0000557)	0·0000269 (0·00000473–0·0000541)	83	80	79	70	80	84
Executions and police conflict	0·0000399 (0·0000331–0·0000505)	0·000166 (0·000151–0·000177)	0·0000931 (0·0000800–0·000107)	0·000117 (0·0000849–0·000189)	0·0000263 (0·0000231–0·0000300)	0·0000216 (0·0000184–0·0000254)	84	65	75	73	82	85
Hodgkin lymphoma	0·0000330 (0·0000153–0·0000532)	0·0000694 (0·0000630–0·0000742)	0·0000581 (0·0000426–0·0000769)	0·0000370 (0·0000280–0·0000476)	0·0000244 (0·0000128–0·0000436)	0·0000346 (0·00000882–0·0000657)	85	73	80	83	83	82
Schistosomiasis	0·0000277 (0·0000176–0·0000399)	0·000000142 (0–0·000000916)	0·000000998 (0·000000174–0·00000259)	0·00000203 (0·000000948–0·00000370)	0·00000842 (0·00000323–0·0000162)	0·0000470 (0·0000296–0·0000701)	86	89	91	90	89	80
Malignant skin melanoma	0·0000268 (0·0000136–0·0000684)	0·000368 (0·000105–0·00231)	0·000114 (0·0000541–0·000510)	0·0000333 (0·0000247–0·0000683)	0·0000172 (0·0000113–0·0000341)	0·0000189 (0·00000705–0·0000652)	87	55	73	84	85	87
Cystic echinococcosis	0·0000236 (0·00000595–0·0000498)	0·00000152 (0–0·00000600)	0·00000931 (0·000000168–0·0000304)	0·00000706 (0·000000139–0·0000236)	0·0000147 (0·00000292–0·0000350)	0·0000341 (0·00000953–0·0000673)	88	87	88	88	87	83
Pancreatitis	0·0000195 (0·0000102–0·0000312)	0·0000522 (0·0000377–0·0000619)	0·0000382 (0·0000275–0·0000494)	0·0000233 (0·0000168–0·0000292)	0·0000190 (0·0000108–0·0000323)	0·0000170 (0·00000504–0·0000335)	89	78	83	85	84	88
Other skin and subcutaneous diseases	0·0000148 (0·0000110–0·0000229)	0·0000758 (0·0000488–0·000118)	0·0000680 (0·0000396–0·000114)	0·0000377 (0·0000230–0·0000505)	0·00000991 (0·00000647–0·0000146)	0·00000711 (0·00000327–0·0000184)	90	72	78	82	88	91
African trypanosomiasis	0·0000111 (0·00000104–0·0000593)	0 (0–0)	0 (0–0)	0·00000110 (0·0000000563–0·00000632)	0·000000583 (0·0000000733–0·00000199)	0·0000203 (0·00000161–0·000110)	91	94	95	91	92	86
Cysticercosis	0·00000256 (0·000000000202–0·00000806)	0·00000000428 (0–0·0000000140)	0·000000126 (0–0·000000392)	0·000000512 (0–0·00000175)	0·00000155 (0–0·00000510)	0·00000385 (0–0·0000119)	92	91	93	92	90	92
Decubitus ulcer	0·00000144 (0·000000390–0·00000313)	0·00000388 (0·00000256–0·00000655)	0·00000357 (0·00000115–0·00000684)	0·00000423 (0·000000645–0·00000676)	0·000000731 (0·000000242–0·00000124)	0·000000899 (0·000000186–0·00000334)	93	85	90	89	91	93
Chagas disease	0·000000311 (0·000000100–0·000000644)	0 (0–0)	0·00000393 (0·000000816–0·00000843)	0·000000429 (0·000000100–0·000000775)	0·000000432 (0·000000180–0·00000127)	0·00000000945 (0·00000000224–0·0000000387)	94	94	89	93	93	94
Zika virus disease	0·0000000313 (0·0000000180–0·0000000490)	0·000000153 (0–0·000000454)	0·000000223 (0·0000000805–0·000000448)	0·0000000804 (0·0000000340–0·000000147)	0·0000000354 (0·0000000145–0·0000000611)	0·00000000204 (0–0·00000000729)	95	88	92	94	94	95
Drug use disorders	0 (0–0)	0 (0–0)	0 (0–0)	0 (0–0)	0 (0–0)	0 (0–0)	96	94	95	95.5	95.5	96

Table S10c. Cause-fraction (cause-specific proportion of total deaths) and cause rank for level-3 causes of death for children under 5 years of age, globally and for socio-demographic index (SDI) quintiles, in 2019, females

	Cause Fraction (2019, females)						Rank (2019, females)					
Cause of death	Global	High SDI	High-middle SDI	Middle SDI	Low-middle SDI	Low SDI	Global	High SDI	High-middle SDI	Middle SDI	Low-middle SDI	Low SDI
Neonatal disorders	0·349 (0·332–0·364)	0·424 (0·387–0·442)	0·403 (0·382–0·423)	0·396 (0·374–0·415)	0·428 (0·403–0·447)	0·289 (0·274–0·304)	1	1	1	1	1	1
Lower respiratory infections	0·143 (0·127–0·156)	0·0243 (0·0220–0·0263)	0·0822 (0·0742–0·0910)	0·130 (0·117–0·142)	0·148 (0·130–0·162)	0·149 (0·128–0·167)	2	5	3	3	2	2
Diarrhoeal diseases	0·101 (0·0826–0·123)	0·00501 (0·00414–0·00627)	0·0254 (0·0207–0·0320)	0·0613 (0·0510–0·0747)	0·0857 (0·0689–0·104)	0·126 (0·101–0·158)	3	19	4	4	4	3
Congenital anomalies	0·0916 (0·0771–0·116)	0·274 (0·250–0·334)	0·229 (0·200–0·256)	0·144 (0·127–0·164)	0·0895 (0·0790–0·104)	0·0691 (0·0420–0·113)	4	2	2	2	3	5
Malaria	0·0788 (0·0389–0·132)	0·000000366 (0·0000000528–0·00000161)	0·0178 (0·0103–0·0259)	0·0404 (0·0256–0·0579)	0·0509 (0·0255–0·0820)	0·109 (0·0510–0·187)	5	87	6	5	5	4
Whooping cough	0·0252 (0·0119–0·0450)	0·00240 (0·00115–0·00468)	0·0107 (0·00469–0·0222)	0·0200 (0·00982–0·0355)	0·0212 (0·00878–0·0425)	0·0299 (0·0128–0·0567)	6	30	11	6	6	6
Meningitis	0·0216 (0·0174–0·0271)	0·00615 (0·00547–0·00671)	0·0100 (0·00888–0·0114)	0·0116 (0·00956–0·0146)	0·0162 (0·0131–0·0221)	0·0280 (0·0221–0·0351)	7	17	13	11	8	7
Protein-energy malnutrition	0·0212 (0·0169–0·0271)	0·000514 (0·000441–0·000618)	0·00460 (0·00376–0·00561)	0·0105 (0·00882–0·0128)	0·0162 (0·0122–0·0217)	0·0280 (0·0216–0·0363)	8	53	23	13	7	8
Sexually transmitted infections excluding HIV	0·0158 (0·00527–0·0323)	0·00299 (0·000941–0·00640)	0·0112 (0·00361–0·0240)	0·0173 (0·00547–0·0368)	0·0132 (0·00423–0·0279)	0·0174 (0·00607–0·0351)	9	25	10	7	9	10
Measles	0·0147 (0·00520–0·0315)	0·000389 (0·000132–0·000934)	0·00331 (0·00107–0·00760)	0·00684 (0·00267–0·0141)	0·00749 (0·00253–0·0171)	0·0215 (0·00758–0·0466)	10	57	29	15	12	9
Tuberculosis	0·0108 (0·00903–0·0131)	0·000287 (0·000252–0·000327)	0·00281 (0·00245–0·00327)	0·00582 (0·00514–0·00673)	0·0100 (0·00816–0·0121)	0·0131 (0·0104–0·0167)	11	61	31	19	11	12
HIV/AIDS	0·0103 (0·00873–0·0121)	0·00195 (0·00131–0·00586)	0·00889 (0·00597–0·0112)	0·0125 (0·0104–0·0151)	0·0105 (0·00865–0·0128)	0·00995 (0·00788–0·0123)	12	31	15	8	10	13
iNTS	0·00976 (0·00558–0·0155)	0·000292 (0·000171–0·000493)	0·000683 (0·000351–0·00118)	0·00198 (0·00107–0·00337)	0·00425 (0·00227–0·00699)	0·0155 (0·00888–0·0245)	13	60	55	38	19	11
Road injuries	0·00736 (0·00632–0·00902)	0·0187 (0·0170–0·0207)	0·0176 (0·0157–0·0197)	0·0121 (0·0104–0·0140)	0·00532 (0·00445–0·00675)	0·00657 (0·00509–0·00874)	14	7	7	9	15	14
Drowning	0·00651 (0·00572–0·00741)	0·0110 (0·0100–0·0118)	0·0113 (0·00996–0·0127)	0·0109 (0·00968–0·0122)	0·00692 (0·00580–0·00819)	0·00485 (0·00390–0·00597)	15	9	9	12	13	16
Sudden infant death syndrome	0·00536 (0·00225–0·0122)	0·0450 (0·0390–0·0502)	0·0114 (0·00943–0·0157)	0·00666 (0·00378–0·0127)	0·00481 (0·00169–0·0117)	0·00432 (0·000930–0·0125)	16	3	8	16	16	18
Foreign body	0·00488 (0·00442–0·00537)	0·0309 (0·0282–0·0330)	0·0214 (0·0189–0·0241)	0·0120 (0·0105–0·0136)	0·00439 (0·00388–0·00502)	0·00204 (0·00175–0·00235)	17	4	5	10	18	30
Hemoglobinopathies and hemolytic anaemias	0·00487 (0·00316–0·00684)	0·00149 (0·00119–0·00181)	0·00278 (0·00234–0·00402)	0·00274 (0·00198–0·00514)	0·00358 (0·00237–0·00549)	0·00630 (0·00373–0·00936)	18	33	32	32	23	15
Typhoid and paratyphoid	0·00413 (0·00152–0·00816)	0·000162 (0·0000447–0·000425)	0·00275 (0·000913–0·00581)	0·00334 (0·00113–0·00703)	0·00647 (0·00238–0·0128)	0·00314 (0·00117–0·00623)	19	68	33	24	14	22
Tetanus	0·00369 (0·00274–0·00525)	0·0000664 (0·0000412–0·000121)	0·00114 (0·000765–0·00191)	0·00263 (0·00173–0·00414)	0·00418 (0·00295–0·00647)	0·00388 (0·00269–0·00651)	20	74	45	34	20	19
Encephalitis	0·00340 (0·00280–0·00465)	0·00315 (0·00262–0·00436)	0·00786 (0·00634–0·0121)	0·00703 (0·00547–0·00839)	0·00415 (0·00299–0·00663)	0·00184 (0·00139–0·00253)	21	24	17	14	22	33
Paralytic ileus and intestinal obstruction	0·00339 (0·00265–0·00430)	0·00720 (0·00638–0·00787)	0·00464 (0·00395–0·00548)	0·00498 (0·00394–0·00639)	0·00299 (0·00222–0·00411)	0·00308 (0·00229–0·00421)	22	15	22	21	25	23
Other unspecified infectious diseases	0·00338 (0·00208–0·00437)	0·00796 (0·00579–0·0109)	0·00521 (0·00266–0·00665)	0·00308 (0·00190–0·00381)	0·00300 (0·00155–0·00480)	0·00349 (0·00216–0·00462)	23	13	20	28	24	20
Falls	0·00328 (0·00195–0·00421)	0·00425 (0·00331–0·00623)	0·00793 (0·00573–0·00986)	0·00641 (0·00346–0·00848)	0·00441 (0·00241–0·00619)	0·00161 (0·00102–0·00213)	24	21	16	17	17	35
Fire, heat, and hot substances	0·00304 (0·00196–0·00427)	0·00553 (0·00478–0·00696)	0·00481 (0·00345–0·00688)	0·00298 (0·00166–0·00466)	0·00261 (0·00150–0·00383)	0·00315 (0·00209–0·00446)	25	18	21	29	27	21
Leukaemia	0·00296 (0·00251–0·00338)	0·00847 (0·00744–0·00957)	0·0103 (0·00840–0·0120)	0·00501 (0·00416–0·00605)	0·00227 (0·00175–0·00284)	0·00234 (0·00172–0·00304)	26	11	12	20	28	25
Conflict and terrorism	0·00280 (0·00231–0·00335)	0·0000478 (0·0000417–0·0000541)	0·00124 (0·00104–0·00146)	0·00121 (0·000999–0·00145)	0·000294 (0·000244–0·000350)	0·00474 (0·00387–0·00577)	27	78	43	44	57	17
Interpersonal violence	0·00248 (0·00193–0·00299)	0·0174 (0·0154–0·0193)	0·00459 (0·00401–0·00511)	0·00311 (0·00268–0·00351)	0·00178 (0·00132–0·00222)	0·00234 (0·00165–0·00306)	28	8	24	27	35	26
Acute hepatitis	0·00244 (0·00179–0·00350)	0·000189 (0·000149–0·000230)	0·00155 (0·00107–0·00231)	0·00201 (0·00142–0·00300)	0·00418 (0·00277–0·00661)	0·00166 (0·00119–0·00239)	29	64	39	36	21	34
Exposure to mechanical forces	0·00240 (0·00106–0·00355)	0·00241 (0·00206–0·00285)	0·00395 (0·00243–0·00540)	0·00378 (0·00142–0·00550)	0·00188 (0·000814–0·00321)	0·00226 (0·000997–0·00386)	30	29	26	23	32	27
Endocrine, metabolic, blood, and immune disorders	0·00232 (0·00179–0·00279)	0·0195 (0·0161–0·0264)	0·00966 (0·00719–0·0116)	0·00475 (0·00358–0·00597)	0·00212 (0·00165–0·00269)	0·00113 (0·000806–0·00144)	31	6	14	22	29	45
Other malignant neoplasms	0·00231 (0·00191–0·00291)	0·00973 (0·00836–0·0108)	0·00628 (0·00553–0·00694)	0·00314 (0·00277–0·00362)	0·00210 (0·00169–0·00270)	0·00187 (0·00139–0·00271)	32	10	19	26	30	31
Animal contact	0·00223 (0·00121–0·00341)	0·000464 (0·000407–0·000524)	0·000689 (0·000500–0·00106)	0·00130 (0·000731–0·00211)	0·00286 (0·00118–0·00475)	0·00222 (0·00139–0·00334)	33	54	54	43	26	29
Adverse effects of medical treatment	0·00220 (0·00146–0·00282)	0·00295 (0·00246–0·00371)	0·00210 (0·00170–0·00262)	0·00152 (0·00122–0·00184)	0·00190 (0·00134–0·00229)	0·00253 (0·00140–0·00354)	34	26	37	41	31	24
Poisonings	0·00211 (0·00174–0·00261)	0·00166 (0·00150–0·00181)	0·00357 (0·00310–0·00411)	0·00180 (0·00151–0·00213)	0·00185 (0·00143–0·00238)	0·00225 (0·00173–0·00295)	35	32	28	39	33	28
Chronic kidney disease	0·00185 (0·00161–0·00209)	0·00274 (0·00237–0·00312)	0·00236 (0·00211–0·00269)	0·00235 (0·00198–0·00276)	0·00154 (0·00129–0·00181)	0·00186 (0·00149–0·00224)	36	28	36	35	36	32
Stroke	0·00169 (0·00137–0·00225)	0·00277 (0·00225–0·00322)	0·00159 (0·00134–0·00193)	0·00273 (0·00198–0·00404)	0·00141 (0·00111–0·00182)	0·00157 (0·00122–0·00225)	37	27	38	33	37	37
Other cardiovascular and circulatory diseases	0·00167 (0·00133–0·00205)	0·00490 (0·00380–0·00650)	0·00265 (0·00221–0·00330)	0·00324 (0·00231–0·00483)	0·00130 (0·00102–0·00168)	0·00138 (0·00101–0·00171)	38	20	34	25	41	40
Idiopathic epilepsy	0·00154 (0·00128–0·00186)	0·00358 (0·00292–0·00440)	0·00317 (0·00236–0·00406)	0·00179 (0·00135–0·00243)	0·00181 (0·00137–0·00251)	0·00121 (0·000963–0·00150)	39	23	30	40	34	44
Cardiomyopathy and myocarditis	0·00148 (0·00118–0·00187)	0·00785 (0·00623–0·00881)	0·00441 (0·00366–0·00499)	0·00279 (0·00221–0·00379)	0·000862 (0·000672–0·00113)	0·00123 (0·000855–0·00168)	40	14	25	31	47	43
Cirrhosis and other chronic liver diseases	0·00143 (0·00106–0·00187)	0·000793 (0·000620–0·000957)	0·00136 (0·00107–0·00180)	0·00152 (0·00112–0·00209)	0·00133 (0·000901–0·00199)	0·00147 (0·00104–0·00200)	41	42	42	42	38	38
Brain and nervous system cancer	0·00142 (0·00110–0·00170)	0·00817 (0·00535–0·0104)	0·00716 (0·00457–0·00860)	0·00291 (0·00203–0·00371)	0·00130 (0·00103–0·00165)	0·000696 (0·000487–0·00112)	42	12	18	30	42	51
Dengue	0·00135 (0·000258–0·00188)	0·00000782 (0·00000457–0·0000133)	0·00239 (0·000380–0·00378)	0·00602 (0·00120–0·00862)	0·00130 (0·000233–0·00214)	0·000194 (0·0000375–0·000331)	43	83	35	18	40	71
Asthma	0·00132 (0·000975–0·00180)	0·000755 (0·000529–0·000984)	0·000704 (0·000546–0·000933)	0·00199 (0·00141–0·00250)	0·000875 (0·000644–0·00118)	0·00144 (0·000919–0·00222)	44	45	53	37	46	39
Other neglected tropical diseases	0·00115 (0·000646–0·00345)	0·000330 (0·0000985–0·000421)	0·000456 (0·000281–0·000772)	0·000509 (0·000335–0·00111)	0·000753 (0·000442–0·00190)	0·00160 (0·000809–0·00508)	45	58	61	55	49	36
Varicella and herpes zoster	0·00109 (0·000915–0·00131)	0·000625 (0·000452–0·00128)	0·000977 (0·000688–0·00120)	0·000841 (0·000698–0·00105)	0·000985 (0·000755–0·00129)	0·00123 (0·000967–0·00155)	46	47	48	49	45	42
Bacterial skin diseases	0·00101 (0·000635–0·00124)	0·000390 (0·000259–0·000548)	0·000763 (0·000497–0·000995)	0·000997 (0·000457–0·00132)	0·00132 (0·000615–0·00190)	0·000861 (0·000651–0·00108)	47	56	50	46	39	48
Other unintentional injuries	0·000958 (0·000829–0·00112)	0·000803 (0·000646–0·00104)	0·00153 (0·00134–0·00178)	0·000985 (0·000839–0·00118)	0·000808 (0·000646–0·00103)	0·00101 (0·000825–0·00125)	48	41	41	47	48	46
Urinary diseases and male infertility	0·000950 (0·000749–0·00115)	0·00119 (0·00101–0·00134)	0·00101 (0·000884–0·00120)	0·000846 (0·000717–0·00107)	0·00128 (0·000864–0·00168)	0·000780 (0·000627–0·000942)	49	35	46	48	43	50
Other nutritional deficiencies	0·000920 (0·000573–0·00131)	0·000170 (0·000150–0·000188)	0·000631 (0·000513–0·000773)	0·000598 (0·000471–0·000795)	0·00128 (0·000683–0·00205)	0·000828 (0·000474–0·00125)	50	65	57	51	44	49
Diphtheria	0·000773 (0·000464–0·00121)	0·00000932 (0·00000732–0·0000122)	0·0000202 (0·0000133–0·0000294)	0·0000595 (0·0000448–0·0000789)	0·000165 (0·0000936–0·000292)	0·00135 (0·000782–0·00220)	51	82	85	78	68	41
Upper respiratory infections	0·000734 (0·000176–0·00177)	0·000539 (0·000460–0·000694)	0·000733 (0·000549–0·00155)	0·000577 (0·000430–0·000833)	0·000413 (0·000108–0·000812)	0·000957 (0·0000373–0·00277)	52	51	52	52	53	47
Other neurological disorders	0·000658 (0·000501–0·000871)	0·00673 (0·00581–0·00744)	0·00365 (0·00321–0·00408)	0·00101 (0·000864–0·00119)	0·000600 (0·000377–0·000926)	0·000333 (0·000194–0·000529)	53	16	27	45	50	62
Diabetes mellitus	0·000499 (0·000395–0·000597)	0·000819 (0·000638–0·00112)	0·000661 (0·000550–0·000807)	0·000529 (0·000440–0·000642)	0·000453 (0·000323–0·000580)	0·000502 (0·000375–0·000646)	54	40	56	54	51	52
Other transport injuries	0·000417 (0·000274–0·000539)	0·000783 (0·000689–0·000864)	0·000734 (0·000627–0·000868)	0·000452 (0·000385–0·000534)	0·000374 (0·000268–0·000480)	0·000409 (0·000176–0·000610)	55	43	51	60	55	57
Other chronic respiratory diseases	0·000406 (0·000187–0·000682)	0·00110 (0·000605–0·00138)	0·000630 (0·000485–0·000862)	0·000498 (0·000389–0·000726)	0·000274 (0·000147–0·000420)	0·000433 (0·000118–0·000864)	56	37	58	57	59	55
Other digestive diseases	0·000387 (0·000214–0·000644)	0·000522 (0·000341–0·000719)	0·000593 (0·000351–0·000809)	0·000481 (0·000339–0·000700)	0·000264 (0·000150–0·000390)	0·000420 (0·000144–0·000911)	57	52	59	58	61	56
Liver cancer	0·000365 (0·000272–0·000460)	0·00114 (0·000878–0·00132)	0·000860 (0·000726–0·00105)	0·000540 (0·000402–0·000685)	0·000331 (0·000232–0·000446)	0·000300 (0·000206–0·000409)	58	36	49	53	56	66
Rheumatic heart disease	0·000348 (0·000261–0·000449)	0·000170 (0·000132–0·000206)	0·000209 (0·000164–0·000271)	0·000272 (0·000206–0·000442)	0·000380 (0·000254–0·000540)	0·000357 (0·000254–0·000480)	59	66	68	64	54	59
Environmental heat and cold exposure	0·000343 (0·000110–0·000546)	0·000623 (0·000573–0·000671)	0·000423 (0·000281–0·000530)	0·000301 (0·0000812–0·000532)	0·000417 (0·0000656–0·000858)	0·000303 (0·000124–0·000461)	60	48	62	63	52	65
Upper digestive system diseases	0·000321 (0·000209–0·000556)	0·0000718 (0·0000571–0·0000826)	0·000132 (0·000104–0·000174)	0·000134 (0·000105–0·000182)	0·000152 (0·0000940–0·000269)	0·000477 (0·000293–0·000871)	61	73	71	70	69	53
Chronic obstructive pulmonary disease	0·000313 (0·000160–0·000586)	0·000316 (0·000225–0·000423)	0·000266 (0·000208–0·000413)	0·000316 (0·000222–0·000495)	0·000192 (0·000107–0·000295)	0·000381 (0·000153–0·000836)	62	59	66	61	65	58
Endocarditis	0·000302 (0·000183–0·000433)	0·000412 (0·000242–0·000505)	0·000471 (0·000345–0·000691)	0·000456 (0·000313–0·000632)	0·000248 (0·000146–0·000359)	0·000283 (0·000134–0·000452)	63	55	60	59	63	68
Inguinal, femoral, and abdominal hernia	0·000297 (0·000137–0·000444)	0·000922 (0·000671–0·00110)	0·000413 (0·000260–0·000579)	0·000306 (0·000211–0·000421)	0·000278 (0·000151–0·000385)	0·000287 (0·0000795–0·000526)	64	38	63	62	58	67

Non-Hodgkin's lymphoma	0·000294 (0·000249–0·000340)	0·000845 (0·000732–0·000961)	0·00101 (0·000901–0·00115)	0·000506 (0·000434–0·000601)	0·000262 (0·000213–0·000322)	0·000211 (0·000144–0·000281)	65	39	47	56	62	70
Intestinal nematode infections	0·000289 (0·000216–0·000371)	0·00000360 (0·000000350–0·0000123)	0·0000252 (0·0000138–0·0000407)	0·0000583 (0·0000434–0·0000751)	0·000104 (0·0000763–0·000135)	0·000470 (0·000345–0·000613)	66	84	84	79	73	54
Rabies	0·000281 (0·0000687–0·000498)	0·0000227 (0·0000193–0·0000270)	0·0000856 (0·0000205–0·000169)	0·000131 (0·0000210–0·000262)	0·000267 (0·0000738–0·000527)	0·000343 (0·0000749–0·000663)	67	80	76	71	60	61
Kidney cancer	0·000275 (0·000242–0·000315)	0·00148 (0·00122–0·00167)	0·00154 (0·00134–0·00175)	0·000657 (0·000561–0·000756)	0·000181 (0·000151–0·000219)	0·000144 (0·000108–0·000199)	68	34	40	50	66	74
Appendicitis	0·000261 (0·000135–0·000425)	0·000120 (0·0000916–0·000146)	0·000159 (0·000125–0·000201)	0·000152 (0·000113–0·000213)	0·000175 (0·000102–0·000267)	0·000345 (0·000108–0·000629)	69	69	70	68	67	60
Leishmaniasis	0·000236 (0·0000794–0·000678)	0·0000111 (0·000000000332–0·000102)	0·000103 (0·0000000345–0·000557)	0·0000707 (0·000000110–0·000389)	0·000194 (0·0000320–0·000889)	0·000311 (0·000132–0·000680)	70	81	73	77	64	63
Other intestinal infectious diseases	0·000214 (0·000118–0·000345)	0·0000831 (0–0·000251)	0·0000796 (0·00000130–0·000194)	0·0000864 (0·00000340–0·000220)	0·000130 (0·0000483–0·000235)	0·000303 (0·000179–0·000484)	71	71	77	76	70	64
Gallbladder and biliary diseases	0·000188 (0·0000887–0·000284)	0·0000656 (0·0000462–0·0000848)	0·000110 (0·0000736–0·000166)	0·000119 (0·0000814–0·000172)	0·0000974 (0·0000523–0·000163)	0·000262 (0·0000886–0·000423)	72	75	72	74	74	69
Inflammatory bowel disease	0·000165 (0·0000745–0·000296)	0·000778 (0·000599–0·000887)	0·000354 (0·000264–0·000471)	0·000169 (0·000130–0·000208)	0·000125 (0·0000509–0·000236)	0·000165 (0·0000354–0·000355)	73	44	64	66	72	72
Motor neuron disease	0·000121 (0·000105–0·000138)	0·00401 (0·00354–0·00442)	0·00119 (0·000968–0·00154)	0·000165 (0·000140–0·000191)	0·0000519 (0·0000408–0·0000673)	0·0000212 (0·0000172–0·0000266)	74	22	44	67	75	85
Exposure to forces of nature	0·000119 (0·0000984–0·000143)	0·000170 (0·000148–0·000192)	0·0000694 (0·0000584–0·0000818)	0·000119 (0·0000982–0·000142)	0·000125 (0·000104–0·000149)	0·000118 (0·0000962–0·000143)	75	67	79	73	71	75
Ebola virus disease	0·0000816 (0·0000628–0·000102)	0 (0–0)	0 (0–0)	0 (0–0)	0 (0–0)	0·000154 (0·000116–0·000194)	76	93	94	94.5	94.5	73
Pancreatitis	0·0000726 (0·0000169–0·000133)	0·0000593 (0·0000386–0·0000720)	0·0000411 (0·0000250–0·0000554)	0·0000303 (0·0000174–0·0000554)	0·0000387 (0·0000133–0·0000791)	0·000104 (0·0000156–0·000203)	77	77	82	83	77	76
Yellow fever	0·0000605 (0·0000213–0·000135)	0 (0–0)	0·0000131 (0·00000109–0·0000748)	0·00000862 (0·00000276–0·0000204)	0·0000342 (0·00000951–0·0000837)	0·0000920 (0·0000314–0·000217)	78	93	86	85	80	77
Acute glomerulonephritis	0·0000592 (0·0000227–0·0000951)	0·0000291 (0·0000247–0·0000335)	0·0000903 (0·0000602–0·000128)	0·000171 (0·0000660–0·000242)	0·0000411 (0·0000147–0·0000647)	0·0000405 (0·00000875–0·0000879)	79	79	75	65	76	80
Interstitial lung disease and pulmonary sarcoidosis	0·0000568 (0·0000372–0·0000934)	0·000701 (0·000530–0·00118)	0·000174 (0·000109–0·000251)	0·0000950 (0·0000671–0·000178)	0·0000374 (0·0000237–0·0000633)	0·0000397 (0·0000168–0·0000901)	80	46	69	75	79	81
Other neoplasms	0·0000492 (0·0000399–0·0000631)	0·000572 (0·000449–0·000648)	0·000286 (0·000239–0·000344)	0·000124 (0·0000999–0·000151)	0·0000386 (0·0000298–0·0000535)	0·0000144 (0·00000921–0·0000313)	81	50	65	72	78	88
Executions and police conflict	0·0000474 (0·0000392–0·0000647)	0·000199 (0·000179–0·000213)	0·000100 (0·0000885–0·000114)	0·000148 (0·000105–0·000279)	0·0000306 (0·0000271–0·0000346)	0·0000265 (0·0000228–0·0000308)	82	62	74	69	81	83
Otitis media	0·0000397 (0·00000678–0·000167)	0·0000808 (0·0000614–0·000110)	0·0000271 (0·0000176–0·0000460)	0·00000746 (0·00000545–0·0000105)	0·00000999 (0·00000387–0·0000241)	0·0000644 (0·00000370–0·000300)	83	72	83	86	87	78
Schistosomiasis	0·0000341 (0·0000224–0·0000488)	0·000000177 (0–0·00000115)	0·00000156 (0·000000201–0·00000423)	0·00000224 (0·000000984–0·00000440)	0·00000903 (0·00000367–0·0000176)	0·0000587 (0·0000371–0·0000843)	84	88	90	89	88	79
Malignant skin melanoma	0·0000296 (0·0000147–0·0000623)	0·000622 (0·000115–0·00174)	0·000214 (0·0000618–0·000447)	0·0000388 (0·0000273–0·0000647)	0·0000156 (0·0000121–0·0000270)	0·0000148 (0·00000753–0·0000415)	85	49	67	81	84	87
Hodgkin lymphoma	0·0000259 (0·0000151–0·0000359)	0·0000917 (0·0000822–0·000101)	0·0000738 (0·0000571–0·0000910)	0·0000429 (0·0000291–0·0000551)	0·0000183 (0·0000118–0·0000268)	0·0000222 (0·00000994–0·0000368)	86	70	78	80	83	84
Cystic echinococcosis	0·0000243 (0·00000517–0·0000513)	0·00000152 (0–0·00000567)	0·00000903 (0·000000139–0·0000289)	0·00000645 (0·000000150–0·0000209)	0·0000142 (0·00000207–0·0000346)	0·0000356 (0·00000834–0·0000717)	87	86	87	87	85	82
Other skin and subcutaneous diseases	0·0000191 (0·0000136–0·0000262)	0·0000625 (0·0000448–0·0000925)	0·0000607 (0·0000403–0·0000796)	0·0000340 (0·0000247–0·0000476)	0·0000187 (0·0000105–0·0000338)	0·0000125 (0·00000701–0·0000215)	88	76	80	82	82	89
Vascular intestinal disorders	0·0000174 (0·0000115–0·0000270)	0·000197 (0·000143–0·000230)	0·0000588 (0·0000442–0·0000726)	0·0000269 (0·0000192–0·0000358)	0·0000126 (0·00000688–0·0000231)	0·0000122 (0·00000398–0·0000276)	89	63	81	84	86	90
African trypanosomiasis	0·0000107 (0·00000116–0·0000445)	0 (0–0)	0 (0–0)	0·000000729 (0·0000000448–0·00000356)	0·000000593 (0·0000000804–0·00000238)	0·0000198 (0·00000178–0·0000831)	90	93	94	92	91	86
Cysticercosis	0·00000378 (0·000000000904–0·0000129)	0·00000000578 (0–0·0000000206)	0·000000253 (0–0·000000905)	0·000000777 (0–0·00000295)	0·00000208 (0–0·00000743)	0·00000574 (0–0·0000196)	91	90	91	90	89	91
Decubitus ulcer	0·00000171 (0·000000653–0·00000267)	0·00000355 (0·00000274–0·00000523)	0·00000491 (0·00000183–0·00000809)	0·00000574 (0·00000120–0·00000934)	0·000000676 (0·000000333–0·00000128)	0·00000107 (0·000000374–0·00000233)	92	85	88	88	90	92
Chagas disease	0·000000371 (0·000000172–0·00000114)	0 (0–0)	0·00000392 (0·00000126–0·0000111)	0·000000730 (0·000000146–0·00000128)	0·000000519 (0·000000167–0·00000225)	0·0000000116 (0·00000000313–0·0000000490)	93	93	89	91	92	93
Zika virus disease	0·0000000174 (0·00000000708–0·0000000310)	0·0000000987 (0–0·000000316)	0·000000133 (0·0000000358–0·000000284)	0·0000000476 (0·0000000152–0·0000000948)	0·0000000187 (0·00000000626–0·0000000394)	0·00000000111 (0–0·00000000426)	94	89	92	93	93	94
Drug use disorders	0 (0–0)	0 (0–0)	0 (0–0)	0 (0–0)	0 (0–0)	0 (0–0)	95	93	94	94.5	94.5	95

Table S11. Population attributable fraction (PAF) for etiologies of global both-sex under-5 mortality, in 2019, for all levels of the cause of death hierarchy

Cause of death	Etiology	Etiological PAF (Global, under-5, both-sex, 2019)
All causes	Pneumococcus	0·0746 (0·0511–0·101)
	Rotavirus	0·0300 (0·0139–0·0506)
	Respiratory syncytial virus	0·0245 (0·0113–0·0398)
	Shigella	0·0186 (0·00718–0·0348)
	Adenovirus	0·0166 (0·00892–0·0283)
	Cryptosporidium	0·0154 (0·00339–0·0362)
	H influenzae type B	0·0130 (0·00539–0·0222)
	Campylobacter	0·0116 (0·00483–0·0220)
	Cholera	0·0111 (0·00547–0·0191)
	Norovirus	0·00860 (0·00233–0·0188)
	Non-typhoidal Salmonella	0·00783 (0·000845–0·0212)
	Influenza	0·00734 (0·00238–0·0156)
	Meningococcal meningitis	0·00473 (0·00148–0·00956)
	Aeromonas	0·00389 (0·00166–0·00751)
	Entamoeba	0·00376 (0·00102–0·00968)
	Enteropathogenic E coli	0·00314 (0·00150–0·00577)
	Enterotoxigenic E coli	0·00246 (0·00100–0·00516)
	Clostridium difficile	0·000420 (0·000248–0·000657)
Communicable, maternal, neonatal, and nutritional disorders	Pneumococcus	0·0908 (0·0619–0·124)
	Rotavirus	0·0365 (0·0170–0·0618)
	Respiratory syncytial virus	0·0298 (0·0137–0·0480)
	Shigella	0·0226 (0·00870–0·0425)
	Adenovirus	0·0201 (0·0108–0·0342)
	Cryptosporidium	0·0187 (0·00412–0·0437)
	H influenzae type B	0·0159 (0·00655–0·0271)
	Campylobacter	0·0142 (0·00586–0·0268)
	Cholera	0·0135 (0·00662–0·0232)
	Norovirus	0·0105 (0·00280–0·0229)
	Non-typhoidal Salmonella	0·00952 (0·00103–0·0258)
	Influenza	0·00892 (0·00290–0·0192)
	Meningococcal meningitis	0·00576 (0·00180–0·0116)
	Aeromonas	0·00473 (0·00200–0·00918)
	Entamoeba	0·00457 (0·00123–0·0118)

Cause of death	Etiology	Etiological PAF (Global, under-5, both-sex, 2019)
	**Enteropathogenic E coli**	**0·00382 (0·00183–0·00697)**
	**Enterotoxigenic E coli**	**0·00299 (0·00122–0·00628)**
	**Clostridium difficile**	**0·000510 (0·000299–0·000797)**
Respiratory infections and tuberculosis	Pneumococcus	0·477 (0·320–0·644)
	Respiratory syncytial virus	0·170 (0·0791–0·268)
	H influenzae type B	0·0626 (0·0174–0·116)
	Influenza	0·0509 (0·0165–0·108)
Lower respiratory infections	Pneumococcus	0·516 (0·345–0·696)
	Respiratory syncytial virus	0·184 (0·0849–0·290)
	H influenzae type B	0·0676 (0·0188–0·125)
	Influenza	0·0551 (0·0179–0·117)
Enteric infections	Rotavirus	0·264 (0·119–0·433)
	Shigella	0·164 (0·0669–0·305)
	Adenovirus	0·146 (0·0816–0·242)
	Cryptosporidium	0·135 (0·0307–0·324)
	Campylobacter	0·103 (0·0425–0·190)
	Cholera	0·0986 (0·0475–0·172)
	Norovirus	0·0757 (0·0202–0·164)
	Non-typhoidal Salmonella	0·0690 (0·00743–0·192)
	Aeromonas	0·0342 (0·0148–0·0651)
	Entamoeba	0·0331 (0·00921–0·0838)
	Enteropathogenic E coli	0·0277 (0·0133–0·0505)
	Enterotoxigenic E coli	0·0216 (0·00918–0·0454)
	Clostridium difficile	0·00373 (0·00211–0·00608)
Diarrhoeal diseases	Rotavirus	0·303 (0·139–0·498)
	Shigella	0·187 (0·0786–0·346)
	Adenovirus	0·167 (0·0935–0·278)
	Cryptosporidium	0·154 (0·0349–0·367)
	Campylobacter	0·118 (0·0486–0·220)
	Cholera	0·113 (0·0552–0·197)
	Norovirus	0·0866 (0·0229–0·189)
	Non-typhoidal Salmonella	0·0791 (0·00864–0·217)
	Aeromonas	0·0392 (0·0168–0·0766)

Cause of death	Etiology	Etiological PAF (Global, under-5, both-sex, 2019)
	Entamoeba	0·0379 (0·0105–0·0971)
	Enteropathogenic E coli	0·0316 (0·0152–0·0568)
	Enterotoxigenic E coli	0·0248 (0·0105–0·0521)
	Clostridium difficile	0·00427 (0·00244–0·00706)
Other infectious diseases	Pneumococcus	0·0834 (0·0262–0·168)
	Meningococcal meningitis	0·0672 (0·0200–0·141)
	H influenzae type B	0·0570 (0·0143–0·125)
Meningitis	Pneumococcus	0·264 (0·0877–0·498)
	Meningococcal meningitis	0·213 (0·0663–0·419)
	H influenzae type B	0·181 (0·0494–0·381)

Table S12. Neonatal and under-5 deaths [for 2000, 2015, and 2019], and neonatal and under-5 mortality rate (NMR; U5MR) [for 2019] for subnational units in the United States of America, the United Kingdom, and Brazil

	Neonatal deaths			NMR	Under-5 deaths			U5MR
Location	2000	2015	2019	2019	2000	2015	2019	2019
United States of America	18700 (17500–19800)	15500 (14600–16400)	14000 (13000–15300)	3·67 (3·62–3·73)	33400 (33100–33600)	27500 (27200–27800)	24700 (23000–26700)	6·46 (6·33–6·60)
Alabama	371 (339–403)	311 (285–337)	315 (288–343)	5·08 (4·59–5·50)	695 (660–733)	583 (552–617)	585 (540–633)	9·53 (9·15–9·88)
Alaska	31·7 (28·0–35·5)	33·8 (29·8–38·0)	31·1 (23·5–41·0)	2·89 (2·29–3·63)	88·7 (81·6–95·9)	84·6 (77·1–92·8)	74·6 (63·7–89·3)	6·96 (5·87–8·21)
Arizona	371 (341–400)	303 (277–327)	276 (249–304)	3·27 (2·88–3·59)	716 (678–753)	590 (555–627)	528 (483–576)	6·29 (6·04–6·53)
Arkansas	186 (166–208)	166 (146–188)	160 (146–175)	4·17 (3·72–4·55)	382 (357–408)	339 (318–361)	324 (299–352)	8·52 (8·16–8·85)
California	1940 (1800–2060)	1500 (1410–1600)	1240 (1130–1370)	2·73 (2·45–2·96)	3660 (3530–3790)	2580 (2500–2660)	2140 (1970–2330)	4·69 (4·52–4·86)
Colorado	278 (250–307)	217 (198–238)	191 (174–211)	3·31 (2·96–3·60)	482 (454–511)	362 (340–384)	315 (288–343)	5·44 (5·21–5·65)
Connecticut	207 (183–231)	130 (116–146)	126 (99·7–159)	3·31 (2·77–3·91)	308 (288–328)	203 (188–218)	192 (158–237)	5·09 (4·28–6·02)
Delaware	68·5 (59·8–77·3)	60·8 (53·0–69·8)	57·8 (44·4–75·2)	5·11 (4·12–6·29)	113 (104–121)	97·9 (90·3–107)	92·1 (76·0–113)	8·19 (6·92–9·65)
District of Columbia	68·3 (59·3–78·2)	51·7 (41·1–63·9)	46·8 (32·6–66·5)	4·84 (3·86–6·04)	132 (119–147)	97·0 (79·1–117)	87·0 (64·3–116)	9·04 (7·62–10·7)
Florida	946 (879–1010)	945 (875–1010)	869 (790–953)	3·80 (3·40–4·14)	1820 (1760–1880)	1710 (1640–1780)	1580 (1450–1710)	6·96 (6·70–7·21)
Georgia	752 (693–812)	660 (614–708)	593 (540–648)	4·42 (3·98–4·79)	1320 (1260–1370)	1180 (1130–1230)	1050 (974–1140)	7·93 (7·63–8·22)
Hawaii	84·7 (76·0–93·2)	70·8 (62·0–80·3)	65·8 (50·5–86·1)	3·68 (2·95–4·55)	147 (138–156)	122 (113–131)	113 (93·9–137)	6·32 (5·35–7·43)
Idaho	91·0 (82·0–101)	76·7 (67·7–85·9)	74·8 (59·8–93·9)	3·27 (2·70–3·94)	162 (153–173)	136 (127–147)	131 (112–155)	5·77 (4·89–6·77)
Illinois	1050 (976–1130)	679 (632–729)	615 (562–671)	4·05 (3·68–4·36)	1780 (1710–1850)	1100 (1060–1140)	998 (921–1080)	6·57 (6·32–6·81)
Indiana	456 (414–494)	393 (362–422)	371 (339–405)	4·44 (4·01–4·80)	802 (763–841)	689 (656–723)	646 (596–698)	7·78 (7·47–8·07)
Iowa	148 (131–166)	115 (103–127)	111 (88·6–140)	2·93 (2·47–3·46)	269 (254–286)	228 (214–242)	219 (185–263)	5·78 (4·87–6·84)
Kansas	184 (166–203)	155 (139–172)	140 (111–175)	3·78 (3·18–4·45)	334 (317–353)	272 (256–289)	243 (204–294)	6·57 (5·54–7·77)
Kentucky	243 (225–261)	229 (212–248)	215 (196–235)	3·86 (3·46–4·21)	462 (435–489)	442 (416–469)	416 (383–452)	7·54 (7·23–7·83)
Louisiana	399 (358–438)	283 (261–305)	253 (230–278)	4·37 (3·88–4·78)	754 (709–804)	612 (578–651)	537 (494–584)	9·27 (8·90–9·61)
Maine	54·0 (48·3–59·9)	54·9 (48·6–61·5)	51·0 (39·8–65·5)	4·08 (3·39–4·90)	86·6 (80·5–93·1)	90·1 (82·6–98·6)	82·9 (68·7–102)	6·66 (5·63–7·86)
Maryland	414 (383–446)	359 (328–389)	342 (311–374)	4·60 (4·17–4·97)	655 (623–689)	570 (535–604)	536 (494–583)	7·27 (6·97–7·54)
Massachusetts	304 (271–337)	226 (208–246)	203 (165–249)	2·90 (2·49–3·32)	460 (426–494)	353 (331–376)	311 (251–385)	4·47 (3·68–5·41)
Michigan	760 (699–820)	489 (452–524)	452 (412–493)	4·21 (3·81–4·55)	1250 (1200–1310)	851 (810–894)	771 (713–834)	7·20 (6·92–7·47)
Minnesota	246 (222–269)	249 (230–272)	219 (199–240)	3·26 (2·93–3·53)	432 (407–460)	412 (388–439)	366 (336–398)	5·45 (5·23–5·66)
Mississippi	288 (264–314)	212 (193–231)	197 (163–237)	5·18 (4·52–5·85)	563 (530–592)	411 (385–434)	381 (312–465)	10·1 (8·33–12·2)
Missouri	368 (341–398)	308 (283–333)	286 (261–313)	3·88 (3·47–4·22)	650 (614–689)	571 (536–603)	523 (480–567)	7·11 (6·83–7·37)
Montana	43·3 (38·9–47·7)	41·9 (36·0–48·4)	36·7 (27·7–48·5)	3·25 (2·59–4·04)	88·2 (82·1–95·0)	85·5 (78·5–92·7)	74·5 (62·7–90·0)	6·59 (5·56–7·77)
Nebraska	118 (104–132)	99·3 (88·0–111)	90·7 (72·1–114)	3·53 (2·96–4·16)	200 (188–215)	176 (163–190)	160 (134–194)	6·24 (5·24–7·39)
Nevada	113 (102–126)	127 (114–142)	125 (96·5–166)	3·14 (2·51–3·88)	236 (224–249)	236 (222–251)	231 (196–276)	5·84 (4·97–6·85)
New Hampshire	53·3 (47·6–58·8)	37·9 (33·0–43·4)	35·3 (26·6–46·7)	2·83 (2·29–3·48)	83·8 (78·0–89·8)	60·8 (55·2–67·3)	55·9 (46·3–68·8)	4·51 (3·80–5·34)
New Jersey	512 (473–554)	334 (307–361)	293 (265–322)	2·86 (2·58–3·10)	846 (808–887)	557 (526–589)	485 (444–528)	4·75 (4·56–4·93)
New Mexico	107 (97·9–118)	94·8 (84·4–105)	88·1 (70·2–111)	3·55 (2·98–4·20)	217 (203–232)	173 (160–186)	160 (136–193)	6·48 (5·45–7·68)
New York	1150 (1060–1230)	744 (694–796)	651 (591–716)	2·85 (2·57–3·08)	1960 (1880–2040)	1300 (1250–1350)	1130 (1040–1220)	4·94 (4·75–5·12)
North Carolina	737 (680–799)	599 (553–643)	567 (517–619)	4·48 (4·06–4·84)	1190 (1130–1250)	1020 (974–1070)	959 (886–1040)	7·65 (7·36–7·93)
North Dakota	37·3 (32·8–42·5)	39·4 (34·3–45·1)	34·2 (25·7–45·4)	3·55 (2·81–4·46)	65·0 (58·4–72·6)	77·5 (70·8–85·0)	64·8 (53·4–79·9)	6·71 (5·66–7·92)
Ohio	812 (744–873)	673 (622–723)	642 (587–699)	4·76 (4·32–5·14)	1360 (1300–1420)	1130 (1080–1180)	1070 (989–1150)	7·96 (7·66–8·25)
Oklahoma	244 (219–272)	245 (224–266)	214 (194–235)	4·37 (3·88–4·79)	487 (461–515)	475 (446–503)	419 (385–455)	8·57 (8·23–8·90)
Oregon	169 (151–187)	148 (133–164)	123 (98·2–155)	3·06 (2·57–3·60)	305 (291–320)	254 (240–270)	211 (176–256)	5·20 (4·38–6·15)
Pennsylvania	733 (673–793)	606 (563–651)	586 (535–639)	4·15 (3·76–4·48)	1190 (1140–1240)	980 (938–1030)	942 (870–1020)	6·70 (6·45–6·95)
Rhode Island	63·1 (56·7–70·5)	45·8 (39·7–52·6)	42·2 (32·2–55·2)	3·90 (3·15–4·80)	94·2 (86·7–102)	70·3 (64·1–77·2)	64·0 (52·2–79·4)	5·94 (5·00–7·03)
South Carolina	349 (317–381)	267 (244–289)	254 (232–278)	4·33 (3·88–4·71)	576 (544–611)	474 (447–504)	448 (411–486)	7·71 (7·40–8·00)
South Dakota	41·9 (37·5–46·5)	45·6 (39·6–52·0)	40·9 (31·2–53·8)	3·44 (2·73–4·31)	93·2 (86·8–100)	95·9 (87·9–105)	84·8 (72·0–102)	7·15 (6·04–8·42)
Tennessee	445 (408–481)	321 (295–351)	316 (288–345)	3·88 (3·50–4·20)	815 (769–859)	655 (616–693)	629 (581–680)	7·79 (7·49–8·08)
Texas	1300 (1220–1390)	1510 (1410–1610)	1260 (1140–1380)	3·49 (3·12–3·80)	2630 (2540–2740)	2780 (2690–2870)	2340 (2150–2530)	6·47 (6·24–6·70)
Utah	161 (144–179)	183 (164–203)	159 (128–200)	3·40 (2·88–4·00)	300 (280–321)	308 (291–326)	267 (225–322)	5·69 (4·80–6·72)
Vermont	26·7 (23·6–29·9)	17·1 (14·8–19·9)	16·0 (12·2–20·7)	2·69 (2·17–3·32)	43·2 (39·4–47·3)	29·7 (26·4–33·7)	27·9 (23·2–33·8)	4·75 (3·96–5·67)
Virginia	482 (448–513)	408 (375–440)	395 (359–432)	3·80 (3·44–4·11)	806 (772–843)	711 (675–748)	679 (626–736)	6·56 (6·30–6·80)
Washington	265 (246–286)	279 (256–303)	221 (200–243)	2·74 (2·46–2·98)	501 (473–531)	492 (463–522)	391 (359–426)	4·85 (4·65–5·03)
West Virginia	104 (92·3–115)	84·8 (74·1–96·2)	79·9 (63·3–101)	4·13 (3·46–4·94)	191 (177–204)	167 (155–181)	154 (129–187)	8·00 (6·72–9·48)
Wisconsin	307 (275–341)	271 (249–294)	257 (234–280)	3·88 (3·51–4·20)	528 (495–561)	444 (418–469)	423 (390–458)	6·41 (6·15–6·65)

Location	Neonatal deaths 2000	Neonatal deaths 2015	Neonatal deaths 2019	NMR 2019	Under-5 deaths 2000	Under-5 deaths 2015	Under-5 deaths 2019	U5MR 2019
Wyoming	27·1 (24·2–30·5)	27·2 (23·8–31·1)	25·8 (19·2–34·6)	3·55 (2·87–4·39)	51·1 (46·8–55·8)	48·1 (43·8–52·8)	45·3 (37·8–54·7)	6·29 (5·28–7·46)
United Kingdom	2510 (2420–2610)	2070 (1800–2290)	1920 (1590–2310)	2·45 (2·14–2·76)	4440 (4350–4530)	3470 (3400–3550)	3210 (2810–3660)	4·10 (3·97–4·25)
England	2120 (2040–2210)	1770 (1540–1950)	1650 (1380–1950)	2·51 (2·22–2·78)	3750 (3660–3840)	2990 (2930–3050)	2780 (2480–3120)	4·16 (4·05–4·28)
East Midlands	181 (170–193)	162 (139–183)	148 (124–174)	2·68 (2·48–2·89)	300 (285–316)	255 (242–268)	232 (207–260)	4·30 (4·17–4·43)
East of England	187 (176–199)	164 (139–187)	149 (126–175)	2·09 (1·93–2·26)	331 (315–350)	276 (265–287)	250 (223–280)	3·49 (3·39–3·60)
Greater London	406 (385–427)	303 (268–333)	295 (244–353)	2·23 (1·97–2·47)	737 (709–763)	529 (504–556)	511 (450–579)	3·78 (3·68–3·89)
North East England	89·5 (82·2–97·0)	59·4 (50·2–68·3)	53·7 (44·6–64·2)	2·11 (1·95–2·28)	172 (162–183)	114 (107–122)	102 (92·0–114)	3·69 (3·60–3·81)
North West England	299 (283–316)	242 (213–268)	236 (198–277)	2·82 (2·47–3·15)	545 (520–569)	433 (408–457)	418 (372–467)	4·88 (4·75–5·03)
South East England	267 (252–283)	233 (205–259)	234 (195–278)	2·34 (2·09–2·56)	469 (445–494)	384 (363–406)	382 (338–431)	3·72 (3·62–3·83)
South West England	162 (150–173)	135 (115–153)	119 (99·7–140)	2·11 (1·96–2·26)	284 (268–300)	225 (215–234)	197 (176–221)	3·37 (3·27–3·47)
West Midlands	295 (279–311)	290 (254–320)	260 (217–308)	3·73 (3·32–4·10)	480 (458–502)	461 (440–482)	413 (369–464)	5·83 (5·68–6·01)
Yorkshire and the Humber	238 (224–252)	177 (152–199)	158 (132–186)	2·50 (2·18–2·80)	435 (417–455)	314 (299–328)	279 (250–312)	4·36 (4·25–4·49)
Northern Ireland	83·2 (73·0–92·7)	91·1 (77·3–105)	80·3 (51·2–123)	3·21 (2·68–3·85)	142 (130–153)	128 (116–142)	113 (74·0–166)	4·57 (3·81–5·50)
Scotland	195 (171–221)	128 (107–149)	113 (83·3–151)	2·05 (1·82–2·31)	347 (326–371)	216 (199–234)	187 (141–247)	3·45 (3·07–3·90)
Wales	112 (104–121)	83·4 (68·6–98·1)	74·6 (56·6–96·0)	2·20 (1·91–2·51)	196 (185–209)	137 (127–147)	122 (103–144)	3·66 (3·20–4·17)
Brazil	68100 (59200–77200)	38700 (31500–46500)	31600 (24900–38700)	10·1 (8·55–12·0)	150000 (135000–166000)	77700 (65100–92700)	62800 (49900–77000)	20·0 (16·9–23·7)
Acre	369 (310–432)	290 (226–369)	261 (203–326)	16·2 (12·8–20·3)	829 (722–951)	585 (460–730)	509 (393–638)	31·2 (24·6–39·0)
Alagoas	2200 (1710–2670)	1050 (739–1410)	816 (542–1150)	12·0 (8·18–16·7)	6110 (5300–6970)	2230 (1780–2770)	1690 (1290–2160)	23·4 (18·5–29·3)
Amapá	232 (203–261)	251 (213–291)	238 (200–283)	19·0 (16·8–21·4)	369 (333–407)	398 (344–455)	373 (313–443)	29·4 (26·1–33·2)
Amazonas	1210 (1050–1370)	925 (778–1090)	812 (698–945)	12·2 (10·9–13·5)	2470 (2250–2700)	1820 (1600–2070)	1580 (1360–1830)	23·3 (20·8–25·9)
Bahia	7660 (6400–8990)	4390 (3470–5420)	3570 (2700–4570)	12·6 (9·92–15·7)	20200 (17500–23000)	9550 (7520–11900)	7600 (5690–9910)	25·7 (20·3–32·2)
Ceará	5150 (4310–6020)	2720 (2130–3380)	2200 (1650–2830)	12·2 (9·40–15·5)	12600 (11000–14300)	5410 (4270–6710)	4320 (3280–5510)	22·1 (17·4–27·7)
Distrito Federal	651 (566–739)	403 (355–456)	352 (296–410)	9·22 (8·17–10·4)	1110 (1010–1230)	762 (670–862)	659 (551–770)	16·9 (15·0–19·0)
Espírito Santo	1030 (890–1180)	653 (494–843)	537 (404–694)	10·1 (7·90–12·7)	1800 (1610–2000)	1240 (952–1610)	1010 (758–1320)	18·5 (14·5–23·3)
Goiás	1420 (1260–1590)	1020 (882–1170)	841 (709–979)	10·0 (8·96–11·1)	2410 (2200–2640)	1940 (1720–2180)	1580 (1330–1840)	18·3 (16·4–20·4)
Maranhão	5010 (4180–5820)	3160 (2510–3890)	2520 (1940–3160)	12·2 (9·50–15·6)	12900 (11300–14600)	6520 (5270–7960)	5090 (3950–6380)	23·9 (18·9–29·9)
Mato Grosso	652 (571–739)	460 (384–545)	373 (315–435)	8·72 (7·76–9·77)	1440 (1310–1580)	1010 (869–1150)	814 (689–953)	18·2 (16·2–20·5)
Mato Grosso do Sul	883 (778–990)	511 (439–595)	411 (347–484)	11·4 (9·99–13·0)	1450 (1320–1580)	820 (718–935)	661 (557–776)	18·0 (15·9–20·2)
Minas Gerais	7370 (6180–8620)	3590 (2730–4620)	2860 (2170–3670)	10·5 (8·28–13·1)	12900 (11400–14500)	6100 (4740–7670)	4880 (3700–6330)	17·8 (14·1–22·3)
Pará	3050 (2500–3650)	2170 (1630–2840)	1820 (1330–2410)	12·0 (8·95–15·9)	6760 (5870–7670)	4300 (3430–5270)	3550 (2730–4460)	23·2 (18·3–29·0)
Paraíba	1860 (1530–2200)	897 (684–1150)	706 (508–950)	9·44 (7·07–12·4)	3970 (3420–4600)	1720 (1360–2140)	1350 (1020–1760)	17·0 (13·4–21·3)
Paraná	3000 (2610–3400)	1600 (1210–2050)	1310 (984–1690)	9·52 (7·45–12·0)	5290 (4800–5850)	3030 (2330–3910)	2460 (1850–3220)	17·5 (13·7–22·0)
Pernambuco	5430 (4540–6320)	2680 (2130–3300)	2100 (1590–2690)	11·6 (9·14–14·5)	13100 (11500–14700)	5240 (4150–6510)	4040 (3030–5270)	21·7 (17·1–27·1)
Piauí	2550 (2130–3000)	1360 (1070–1680)	1100 (836–1400)	15·0 (11·8–18·9)	4590 (3950–5270)	2150 (1710–2650)	1730 (1310–2210)	23·0 (18·2–28·8)
Rio de Janeiro	2970 (2600–3350)	1820 (1600–2040)	1490 (1270–1740)	9·05 (8·32–9·86)	6790 (6230–7380)	4080 (3640–4550)	3330 (2860–3840)	18·8 (17·2–20·5)
Rio Grande do Norte	1220 (1010–1440)	594 (440–772)	464 (340–613)	7·58 (5·67–10·0)	3190 (2730–3670)	1420 (1090–1800)	1110 (833–1420)	16·4 (12·9–20·5)
Rio Grande do Sul	2160 (1930–2390)	1210 (1040–1390)	1000 (849–1160)	8·86 (8·07–9·72)	3910 (3600–4220)	2260 (2020–2530)	1880 (1590–2160)	16·2 (14·7–17·7)
Rondônia	546 (480–616)	313 (269–362)	244 (206–289)	10·3 (9·16–11·7)	1010 (929–1100)	575 (501–655)	447 (376–530)	18·4 (16·4–20·8)
Roraima	255 (224–287)	246 (210–288)	217 (187–254)	16·9 (15·1–19·0)	346 (312–380)	340 (293–391)	298 (256–348)	23·2 (20·7–26·0)
Santa Catarina	1640 (1420–1880)	1010 (776–1280)	843 (639–1080)	10·4 (8·17–13·0)	2990 (2630–3380)	1880 (1460–2400)	1570 (1180–2040)	18·9 (14·9–23·7)
São Paulo	7760 (6830–8800)	4380 (3780–5070)	3650 (3110–4230)	7·34 (6·73–8·00)	18000 (16700–19400)	10500 (9450–11800)	8810 (7520–10100)	16·8 (15·4–18·3)
Sergipe	1060 (839–1300)	605 (480–746)	495 (397–608)	13·6 (11·4–16·0)	2150 (1850–2450)	1080 (858–1340)	876 (665–1120)	23·7 (18·7–29·6)
Tocantins	762 (641–892)	406 (322–510)	327 (255–427)	11·5 (9·63–14·0)	1590 (1370–1820)	758 (606–929)	604 (482–781)	20·9 (17·5–25·4)

Figure S1a. Under−5 deaths per 1000 live births, 2000

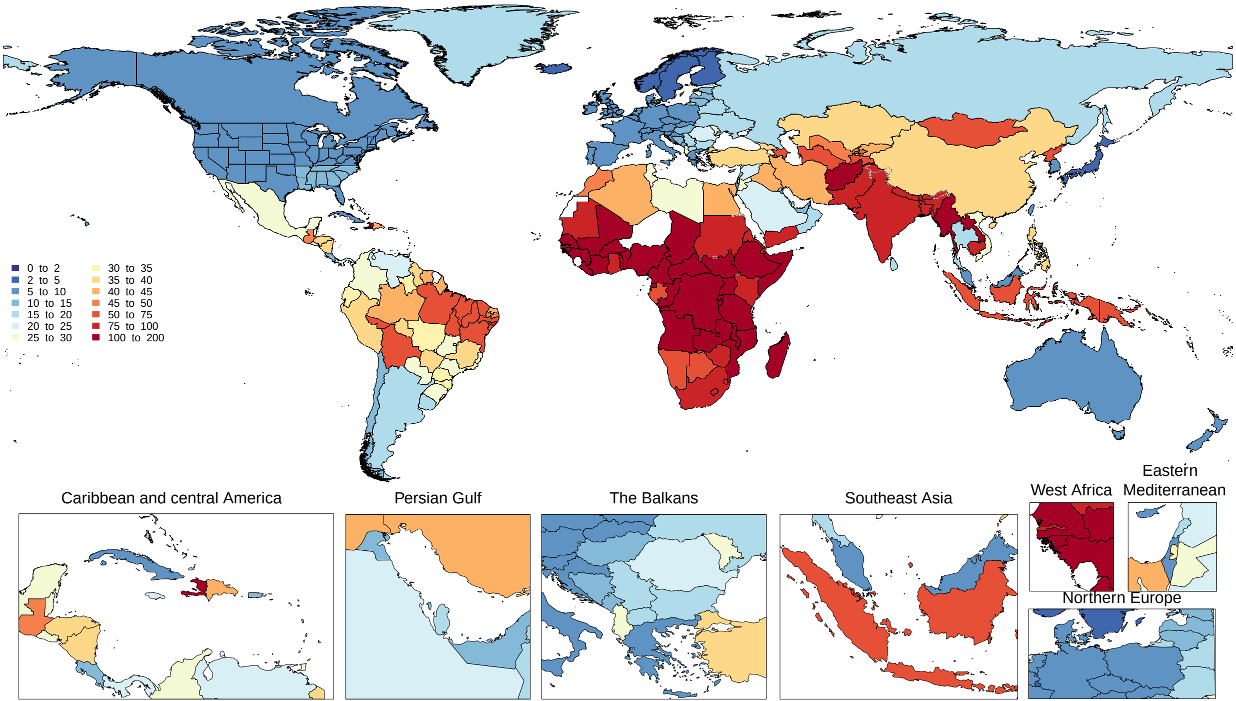

Figure S1b. Under−5 deaths per 1000 live births, 2015

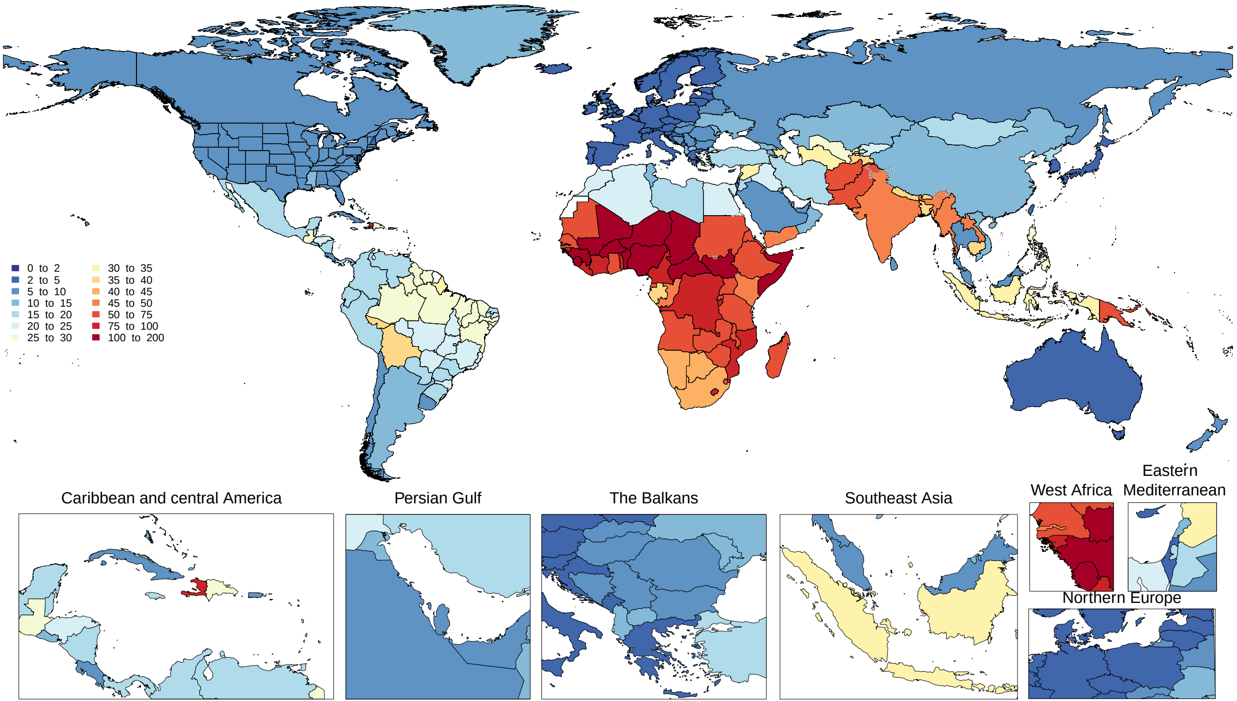

Figure S1c. Under−5 deaths per 1000 live births, 2019

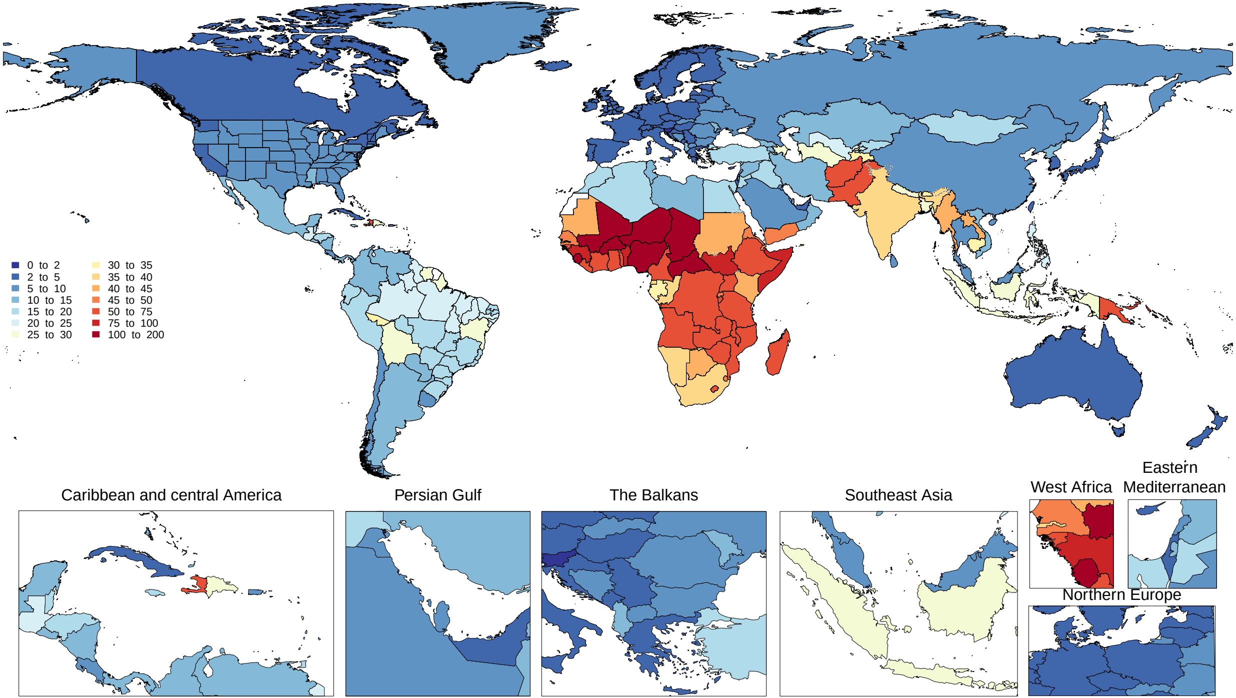

Figure S1d. Neonatal deaths per 1000 live births, 2000

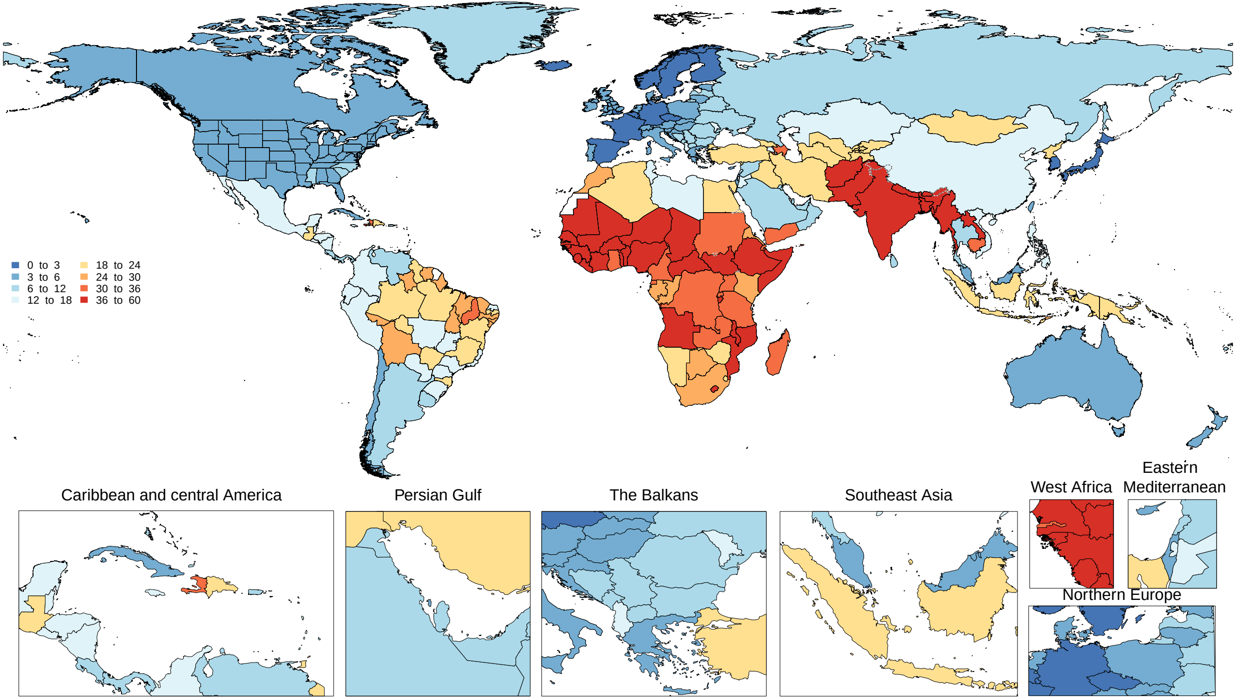

Figure S1e. Neonatal deaths per 1000 live births, 2015

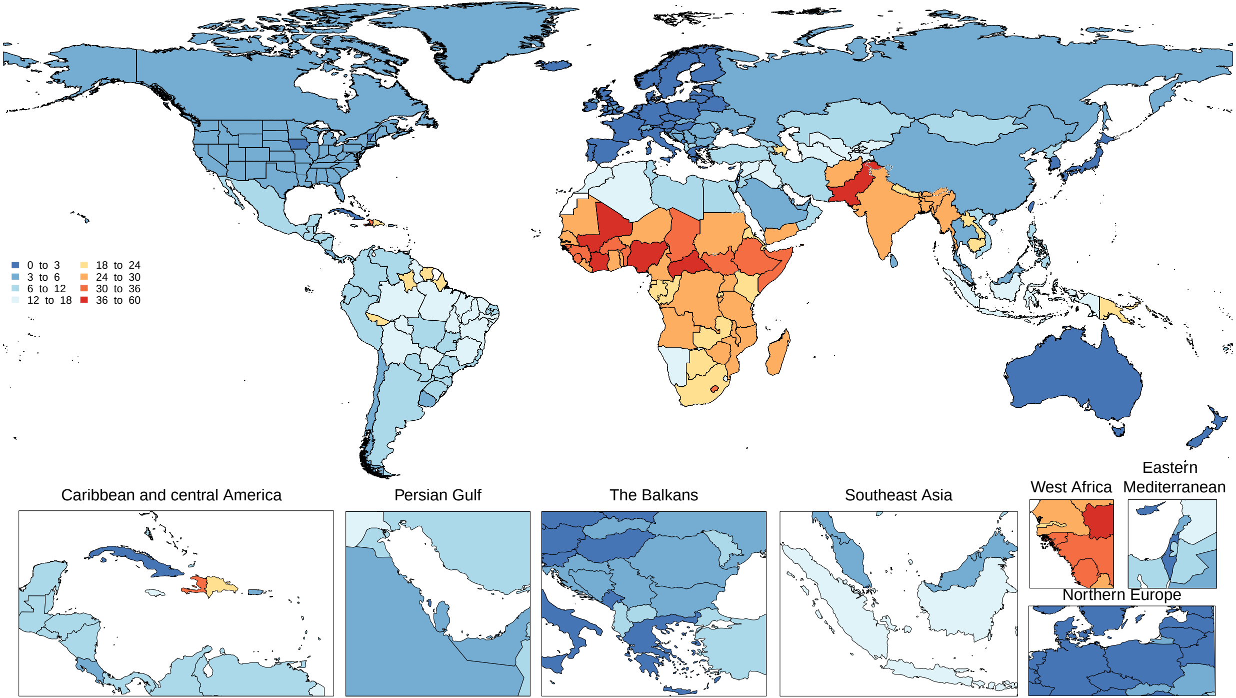

Figure S1f. Neonatal deaths per 1000 live births, 2019

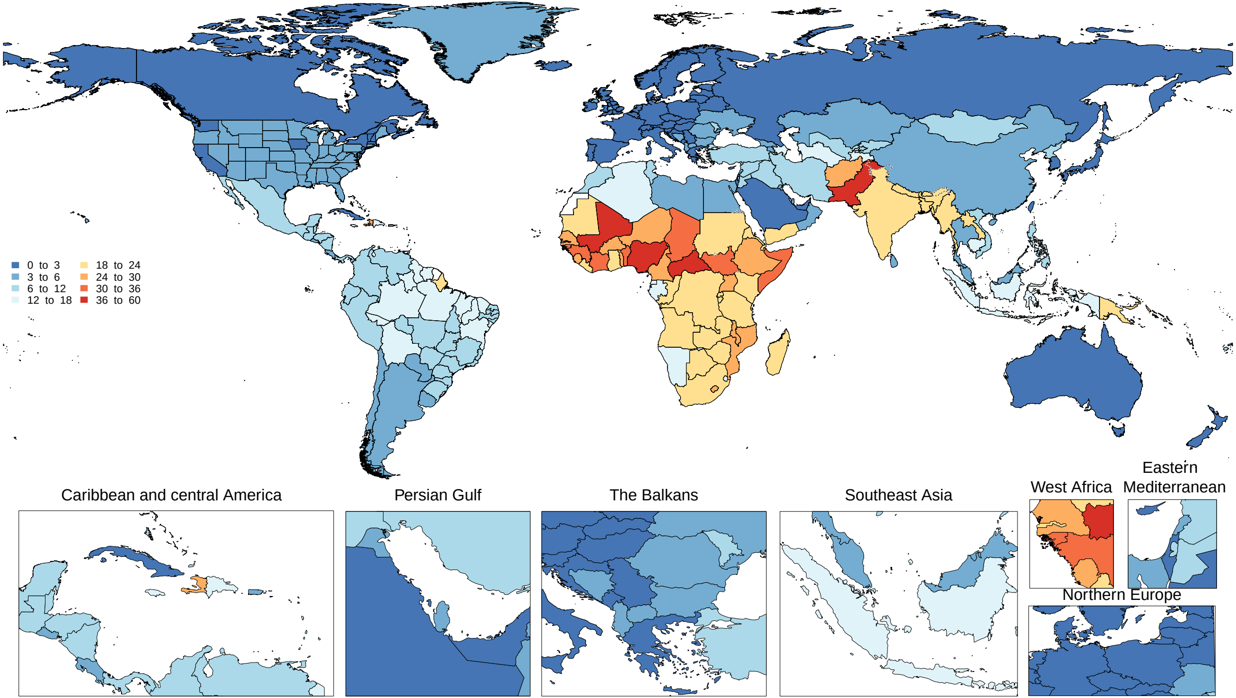

Figure S1g. Last year with available all−cause data at the aggregate under-5 level

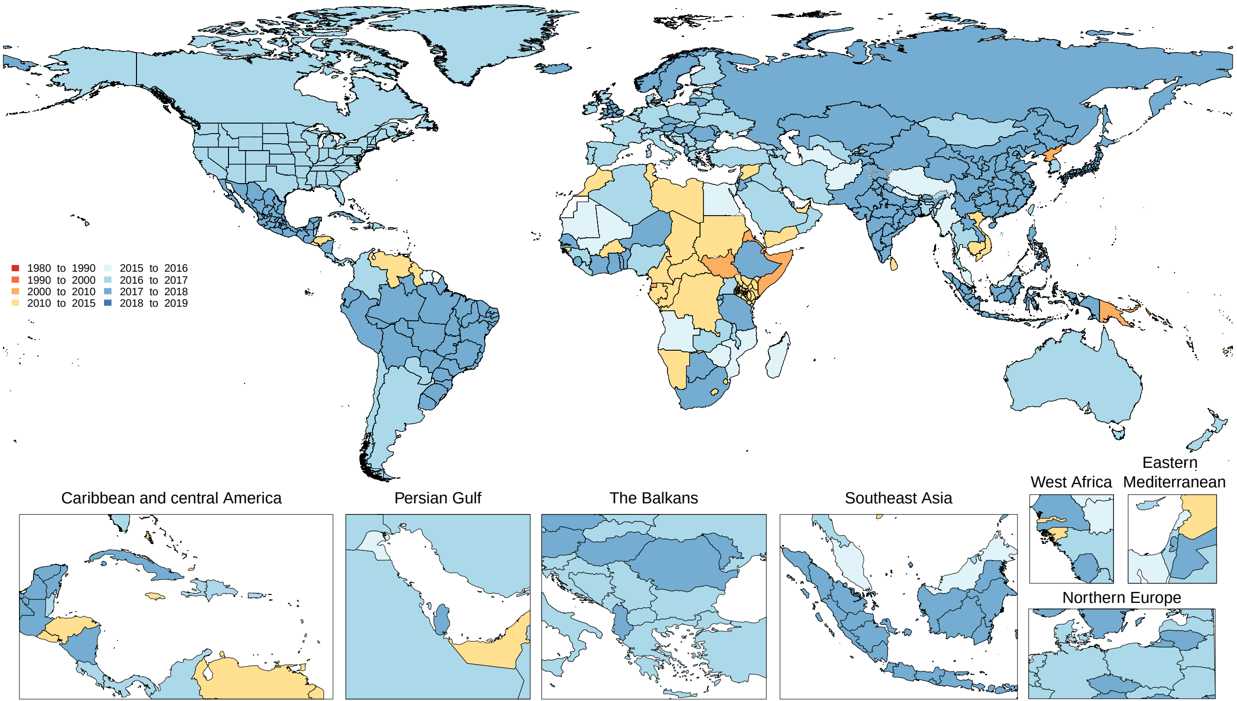

Figure S1h. Last year with available cause−specific data for any cause

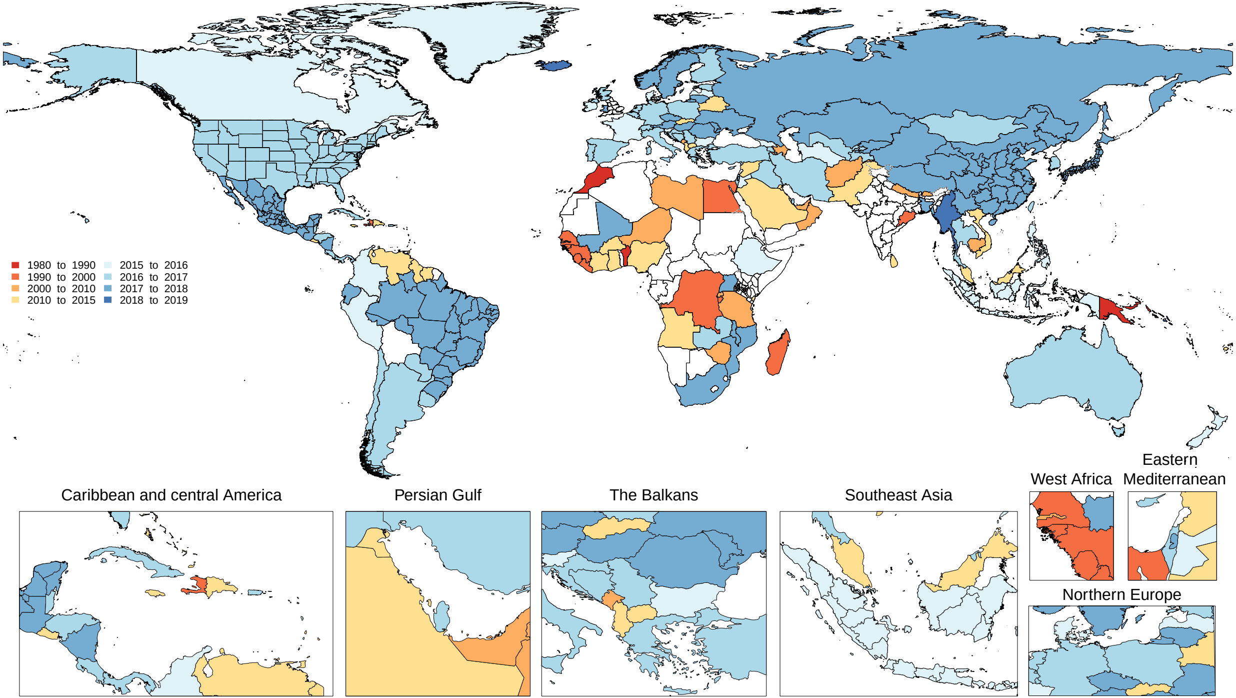

Figure S1i. Proportion of estimated under-5 deaths between 2000 and 2019 which were captured by extracted vital registration data

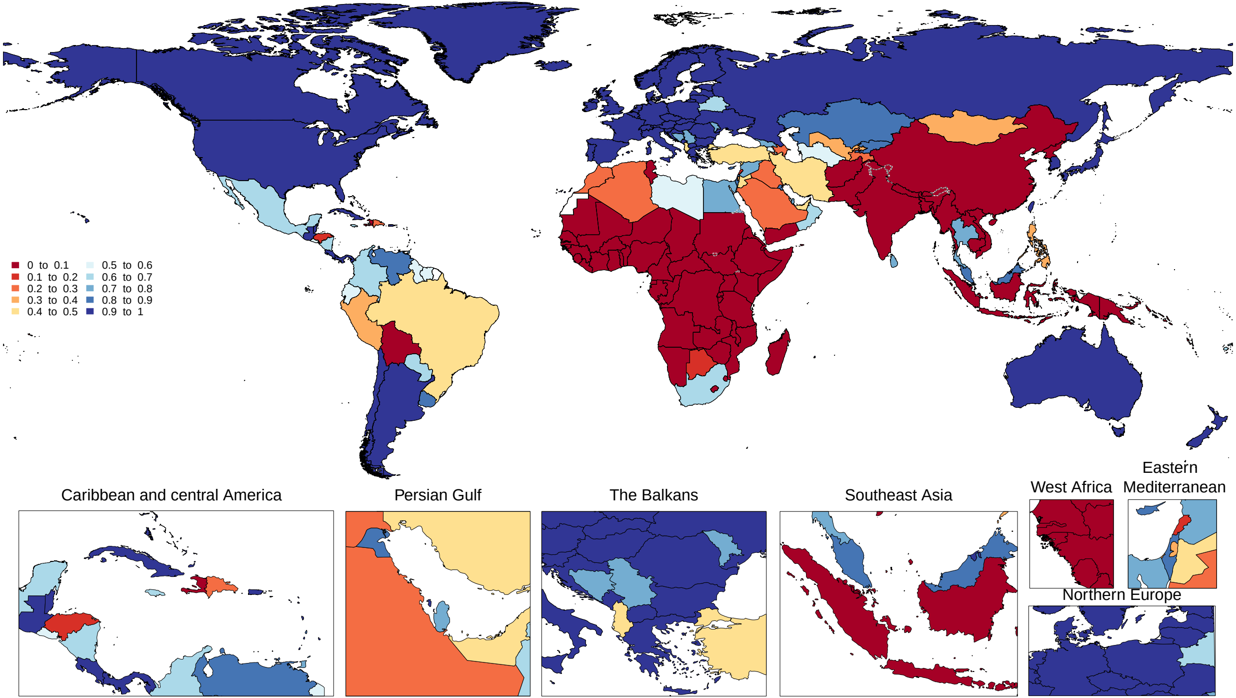

Figure S2. Ratio of under−5 deaths occurring before to after 28 days, by SDI quintile and year

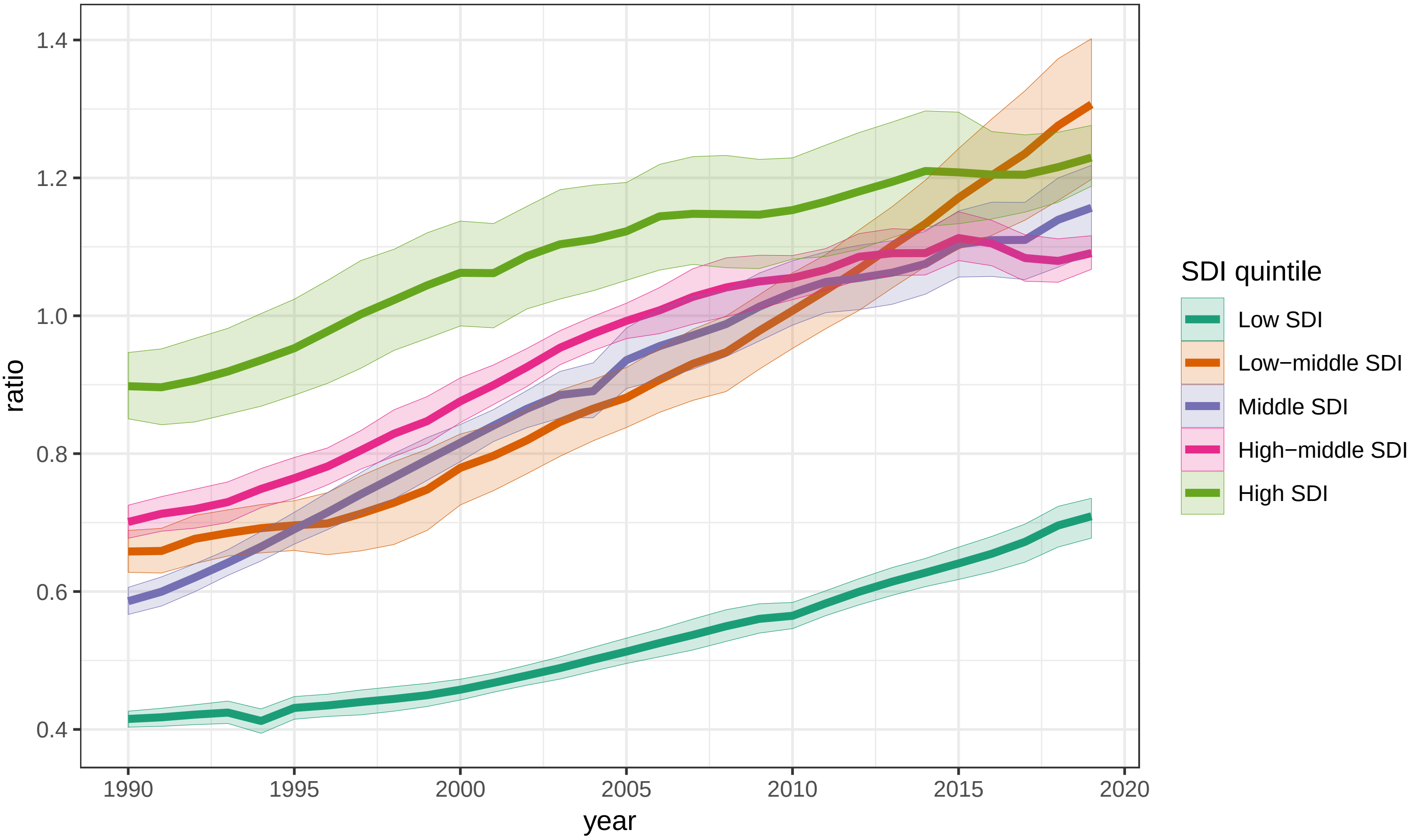

Figure S3a. Proportion of neonatal and under-5 deaths (cause-fraction) by level-2 cause, and by Socio-demographic index (SDI) quintile and GBD region, 2000

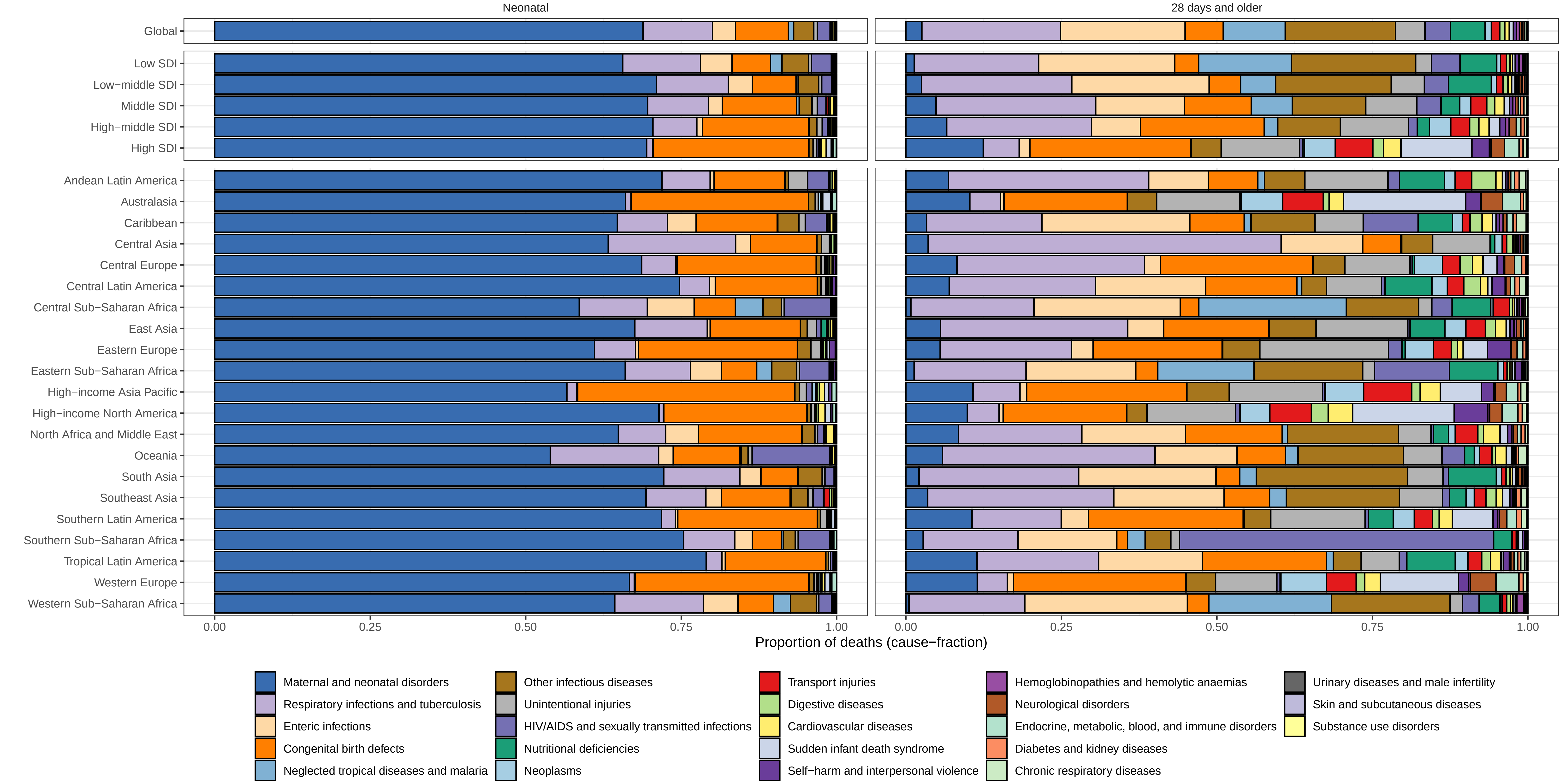

Figure S3b. Proportion of neonatal and under-5 deaths (cause-fraction) by level-2 cause, and by Socio-demographic index (SDI) quintile and GBD region, 2015

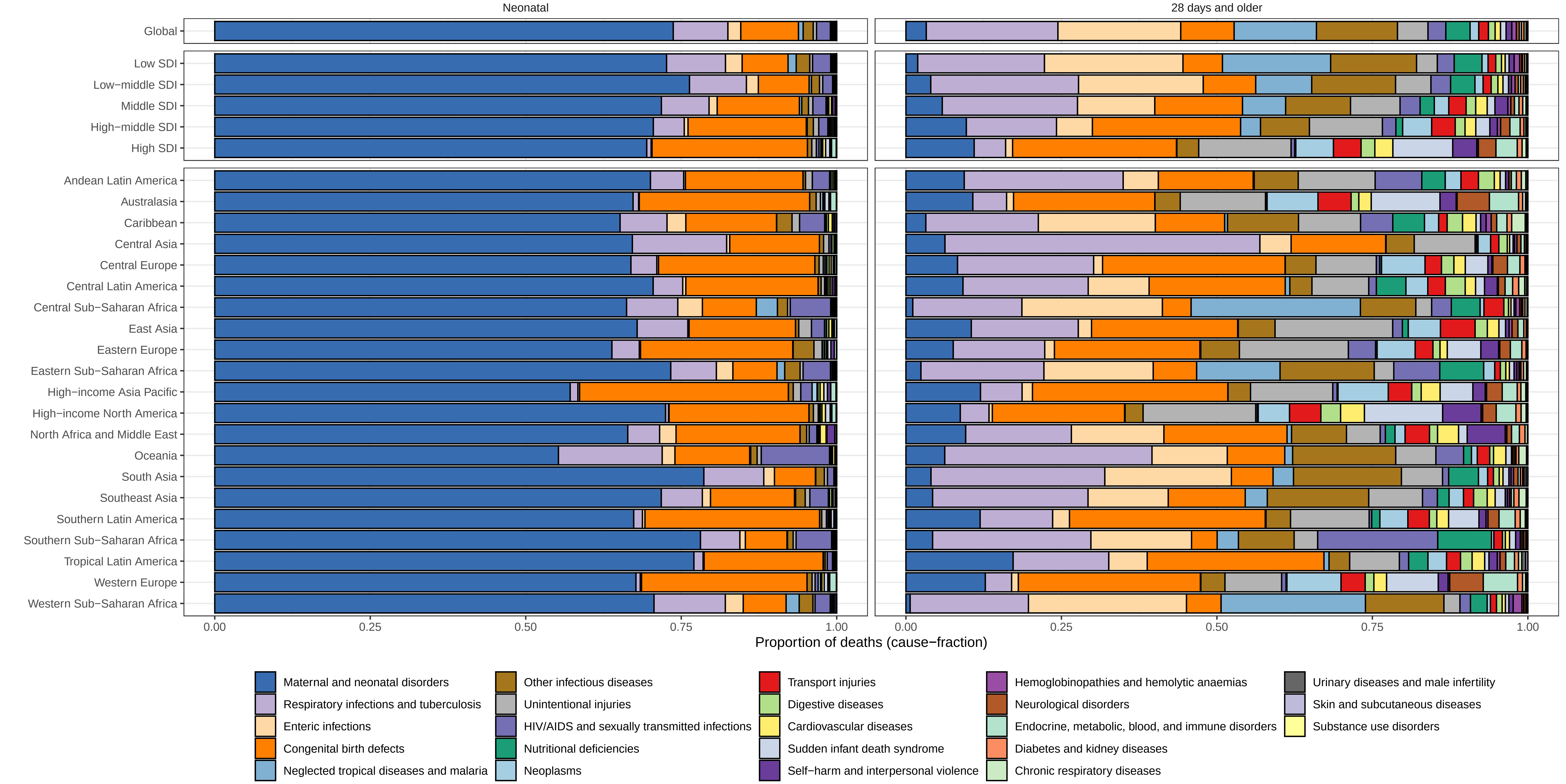

Figure S3c. Proportion of neonatal and under-5 deaths (cause-fraction) by neonatal disorder, and by Socio-demographic index (SDI) quintile and GBD region, 2000

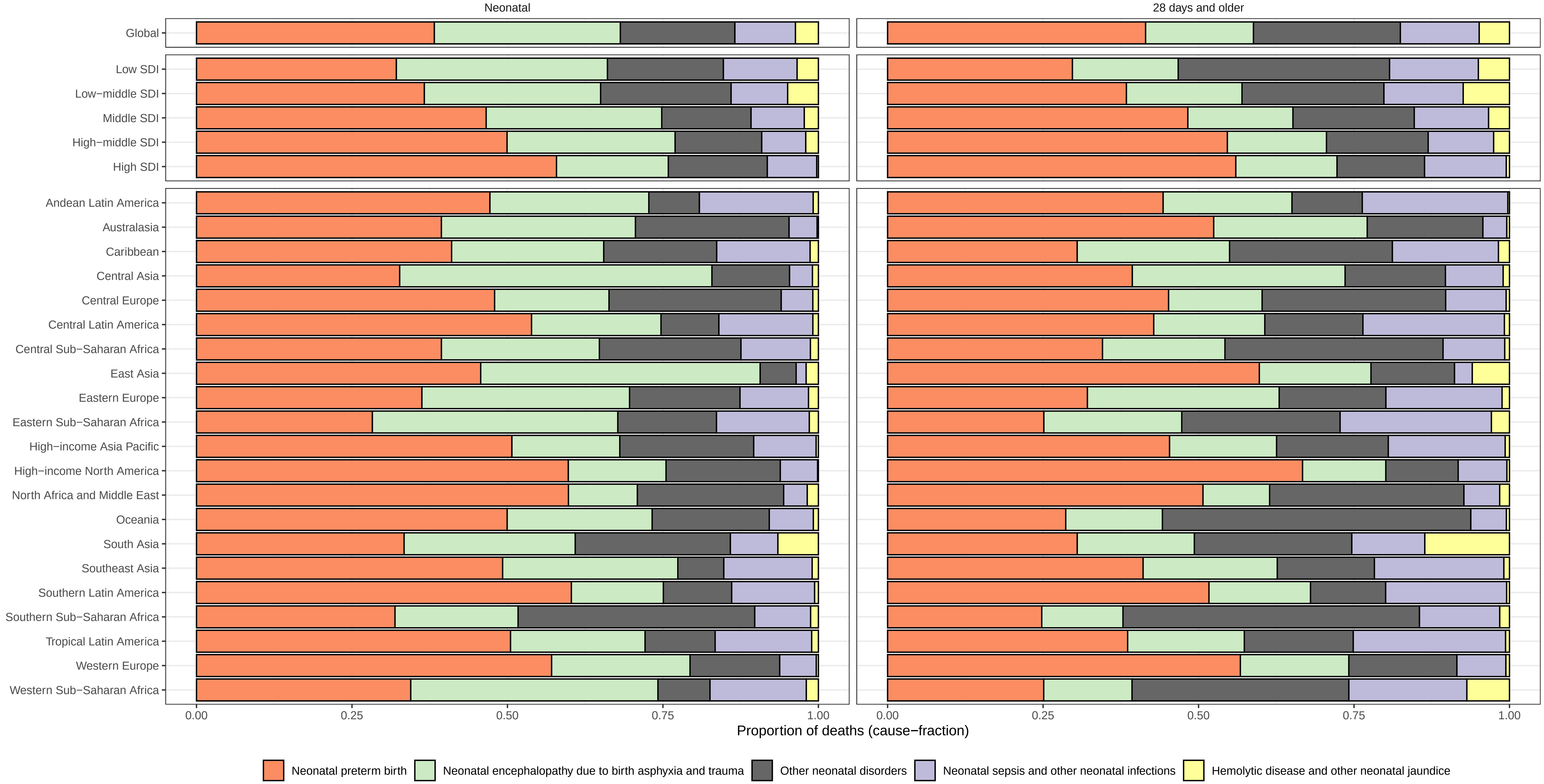

Figure S3d. Proportion of neonatal and under-5 deaths (cause-fraction) by neonatal disorder, and by Socio-demographic index (SDI) quintile and GBD region, 2015

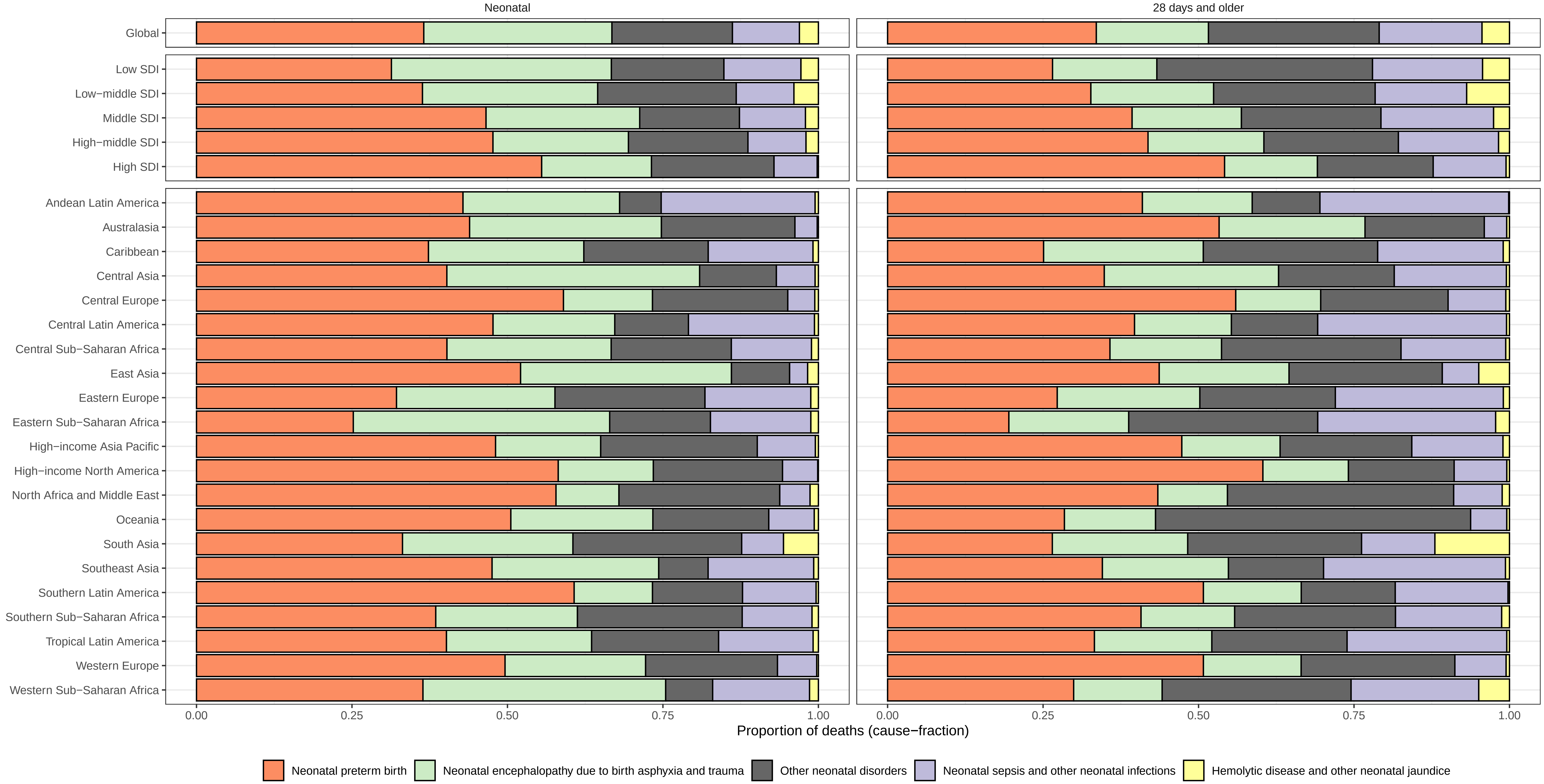

Figure S3e. Proportion of neonatal and under-5 deaths (cause-fraction) by neonatal disorder, and by Socio-demographic index (SDI) quintile and GBD region, 2019

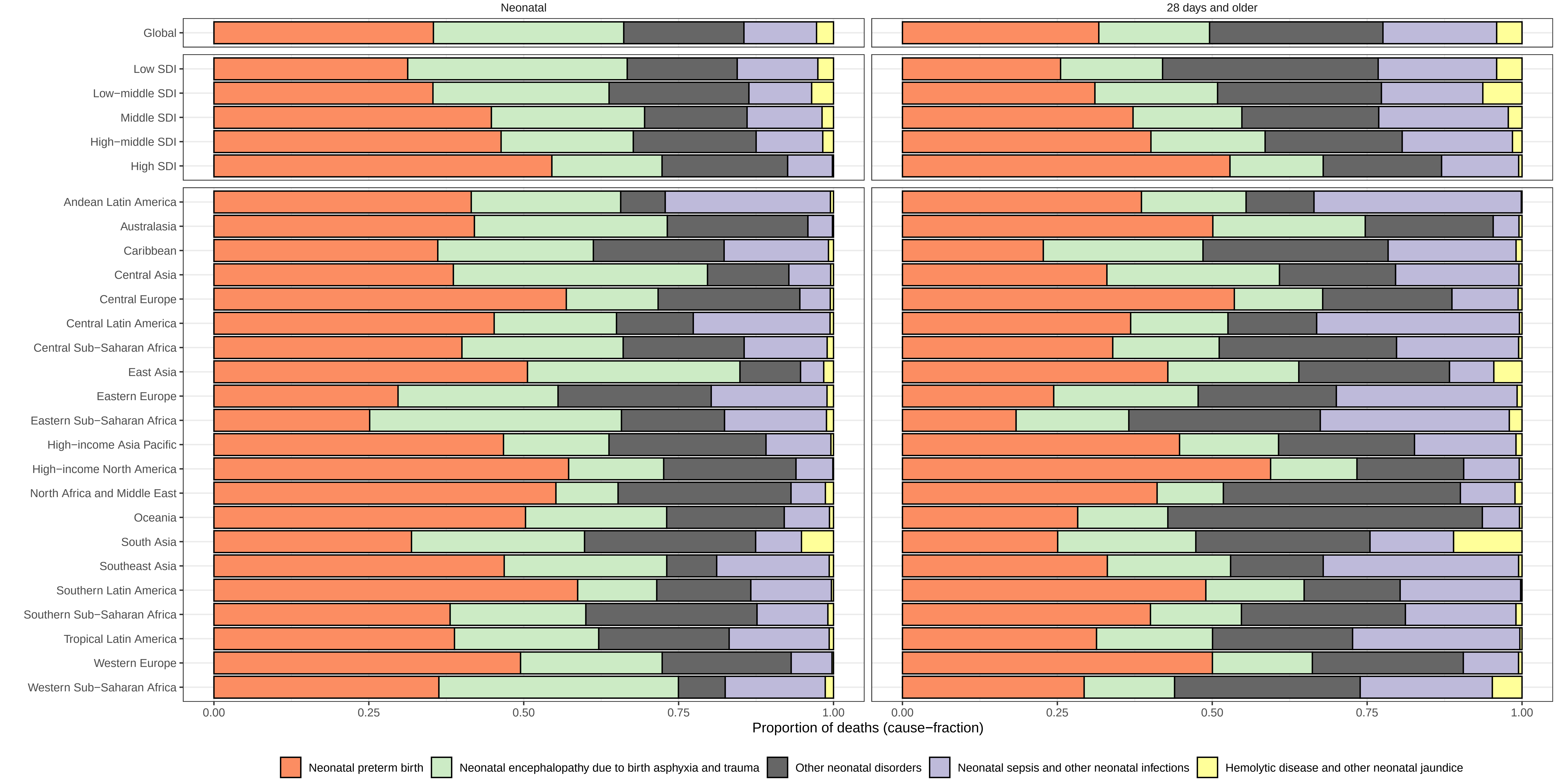

Figure S3f. Proportion of neonatal and under-5 deaths (cause-fraction) by congenital birth defect, and by Socio-demographic index (SDI) quintile and GBD region, 2000

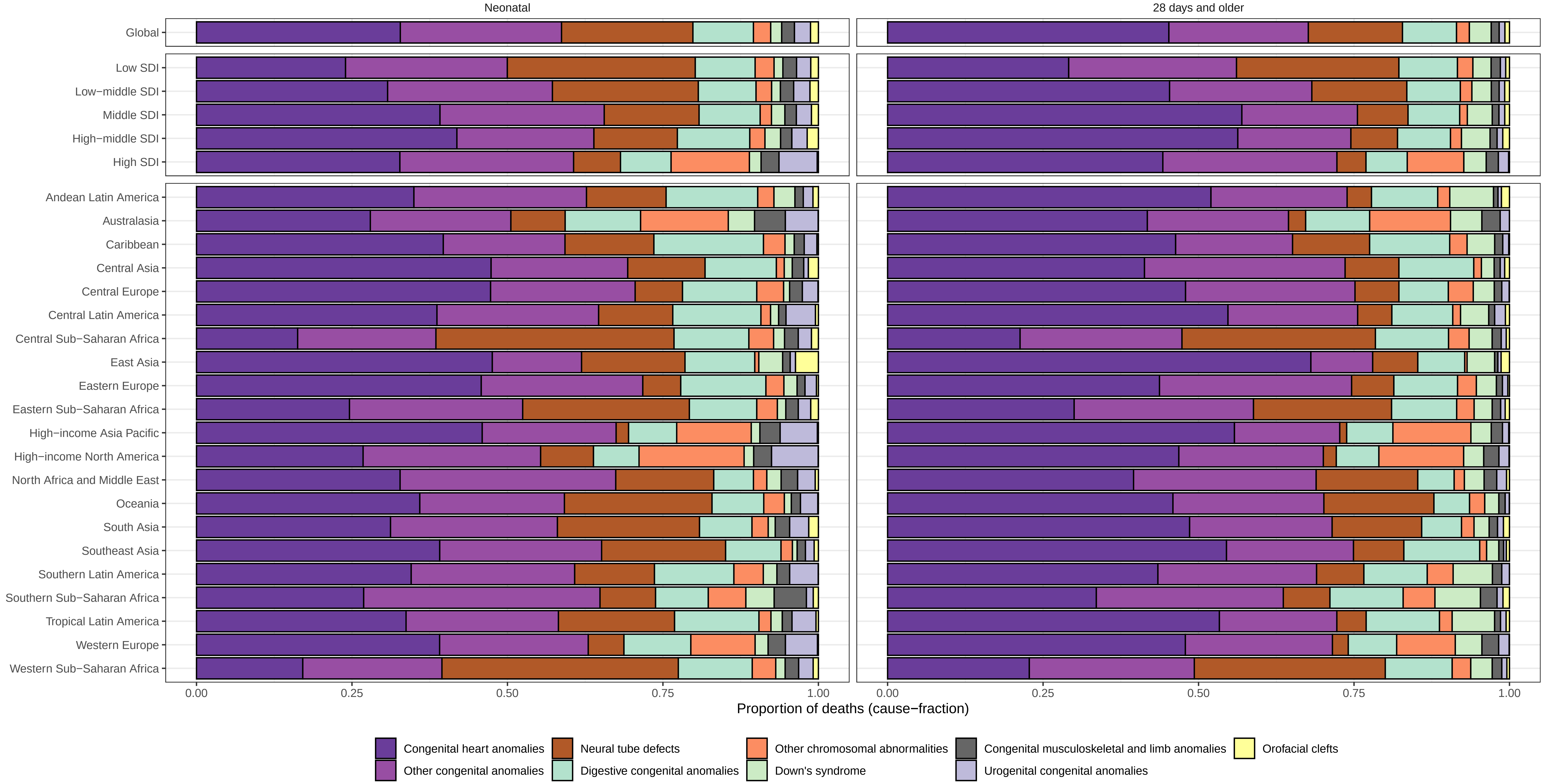

Figure S3g. Proportion of neonatal and under-5 deaths (cause-fraction) by congenital birth defect, and by Socio-demographic index (SDI) quintile and GBD region, 2015

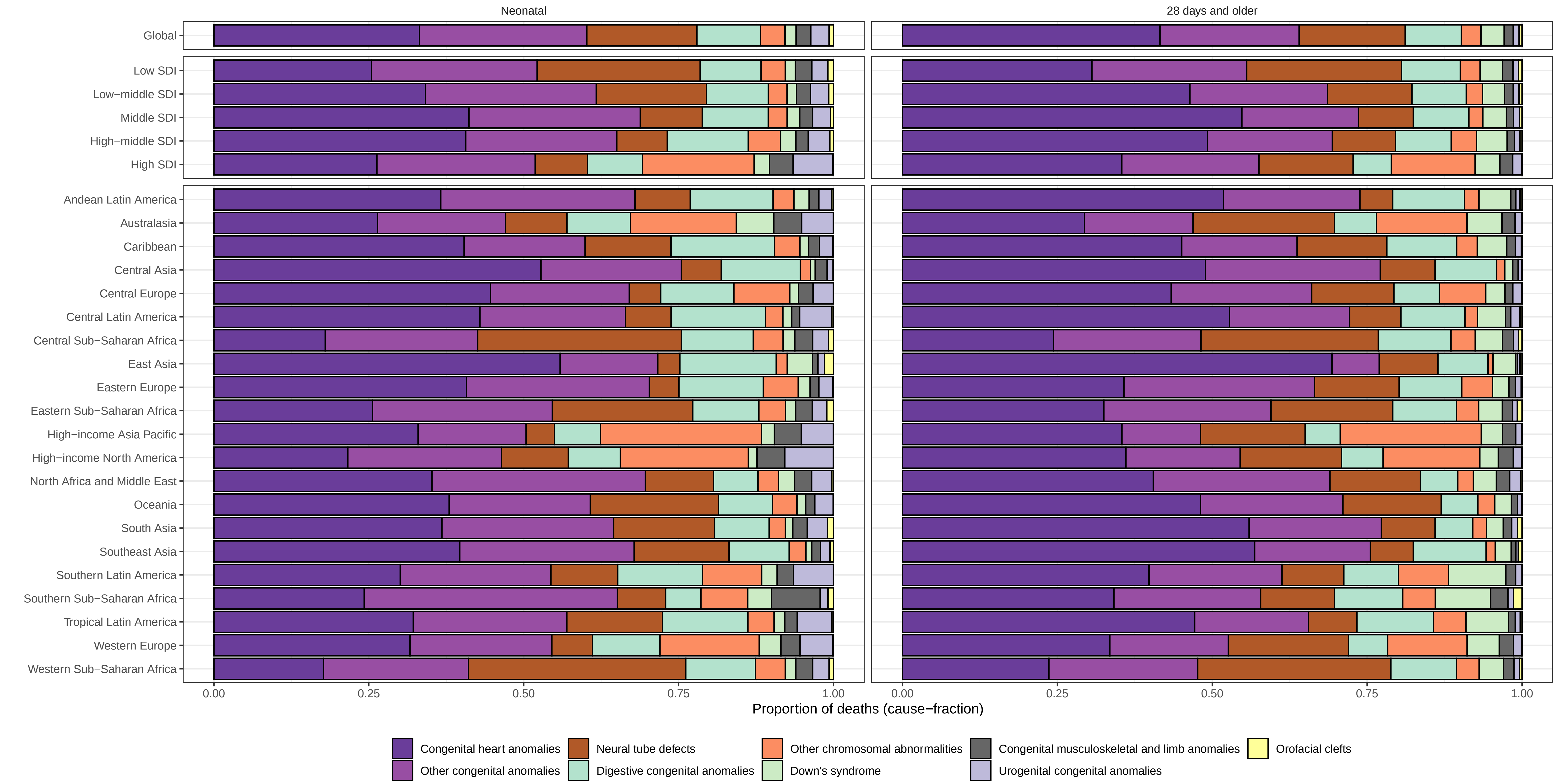

Figure S3h. Proportion of neonatal and under-5 deaths (cause-fraction) by congenital birth defect, and by Socio-demographic index (SDI) quintile and GBD region, 2019

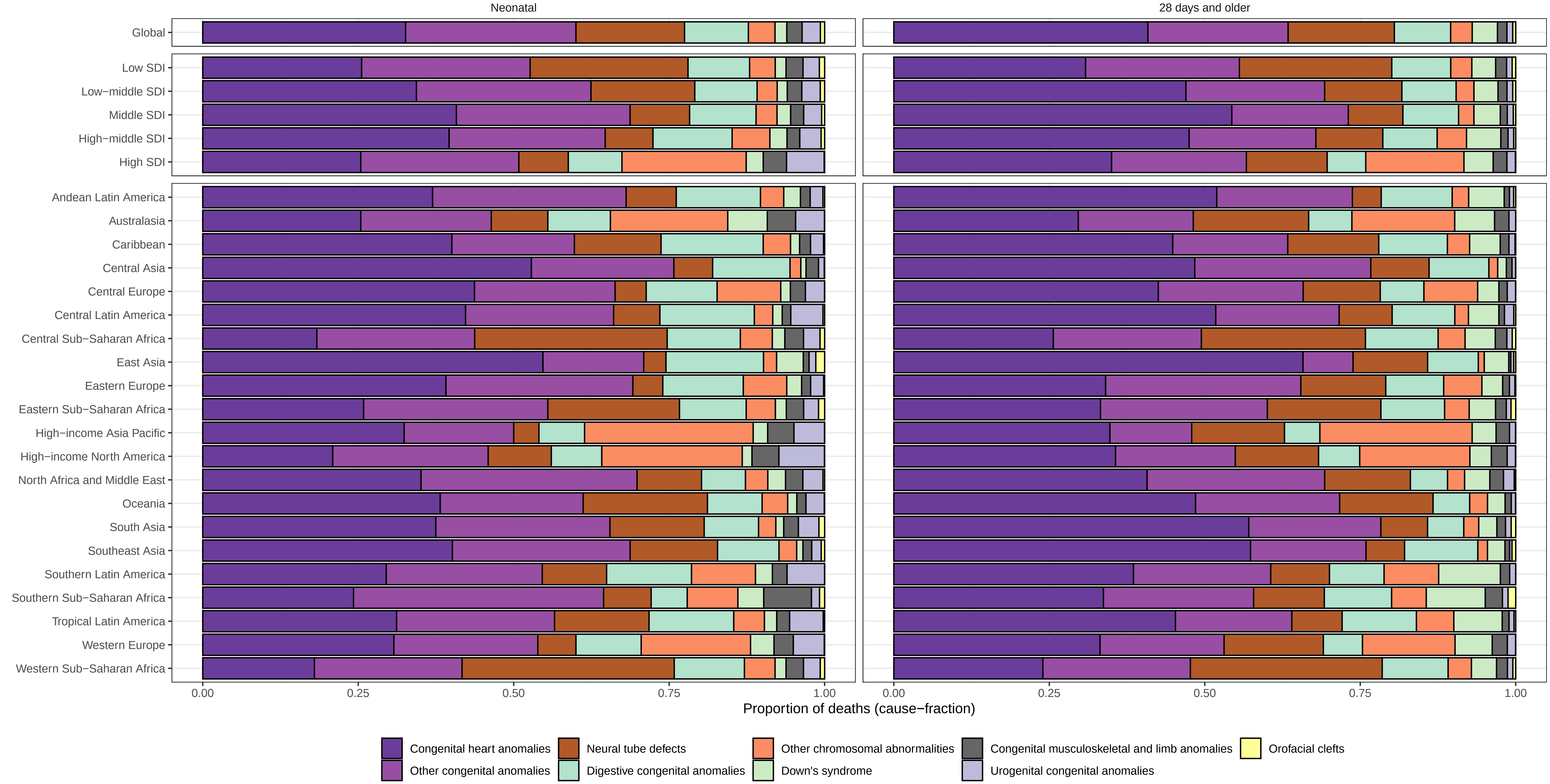

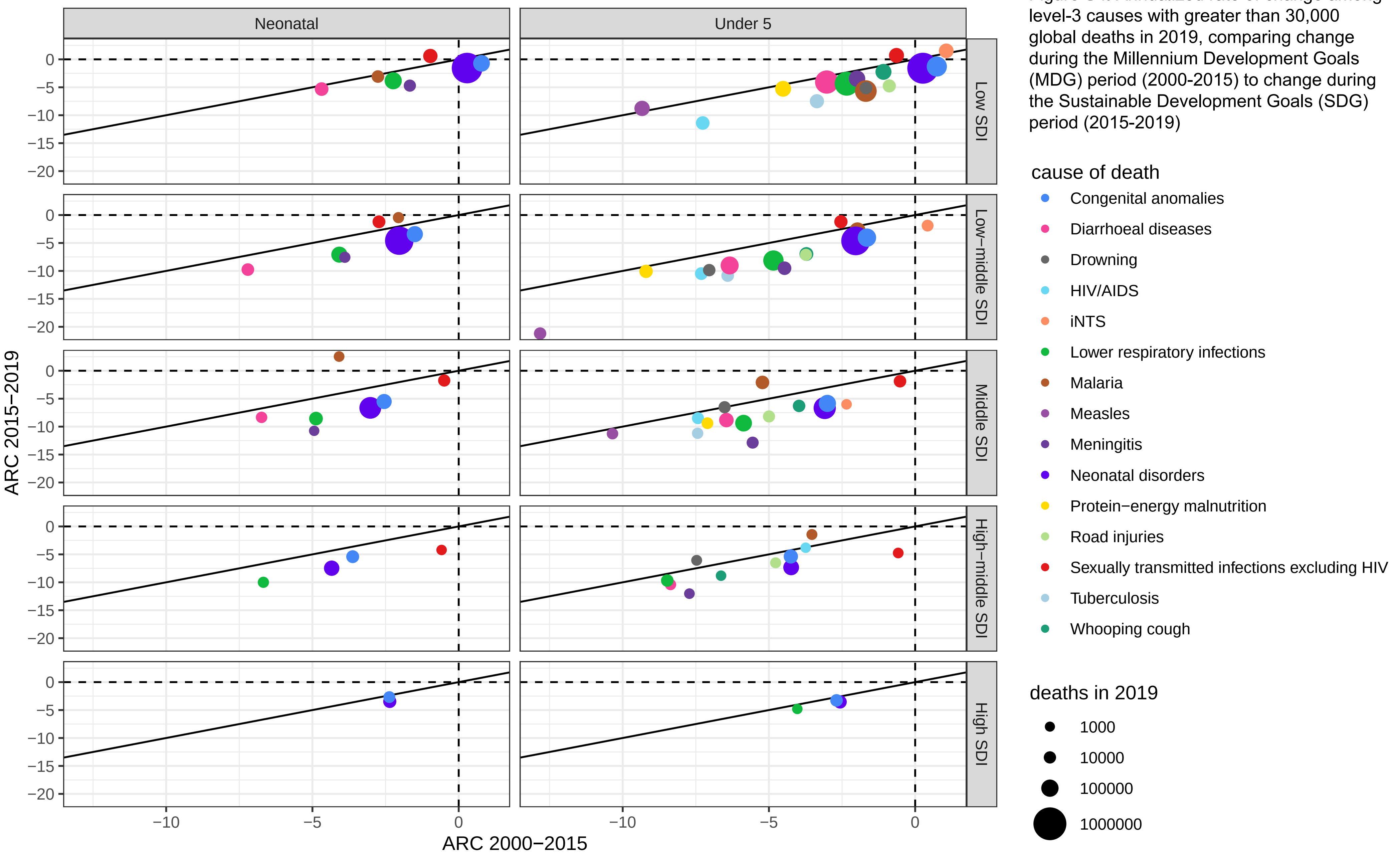

Figure S4. Annualized rate of change among level-3 causes with greater than 30,000 global deaths in 2019, comparing change during the Millennium Development Goals (MDG) period (2000-2015) to change during the Sustainable Development Goals (SDG) period (2015-2019)

Figure S5. Global deaths by mortality benchmark, 2000−2019

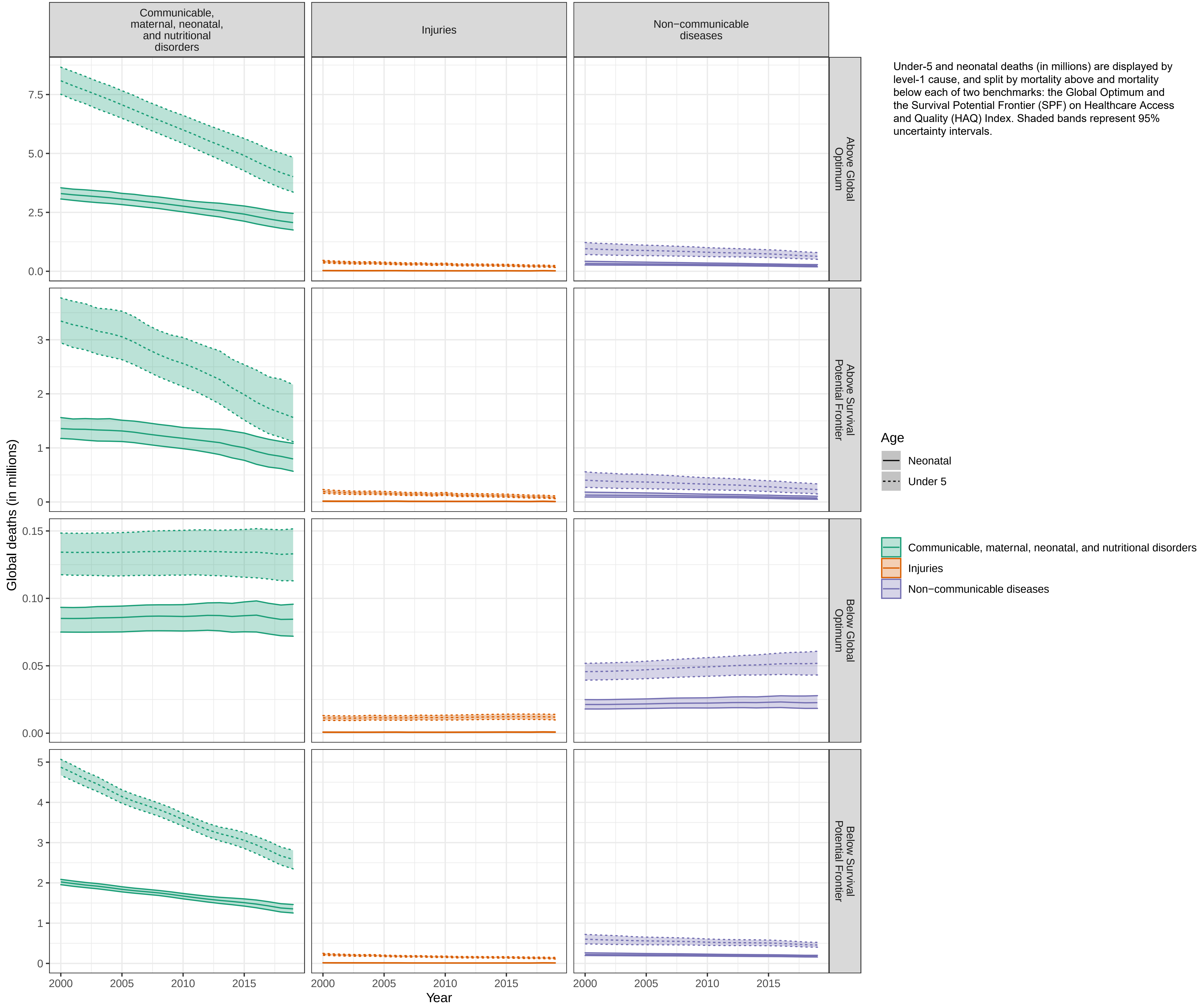

Under-5 and neonatal deaths (in millions) are displayed by level-1 cause, and split by mortality above and mortality below each of two benchmarks: the Global Optimum and the Survival Potential Frontier (SPF) on Healthcare Access and Quality (HAQ) Index. Shaded bands represent 95% uncertainty intervals.

Figure S6aa. Above Global Optimum, Southeast Asia, East Asia, and Oceania

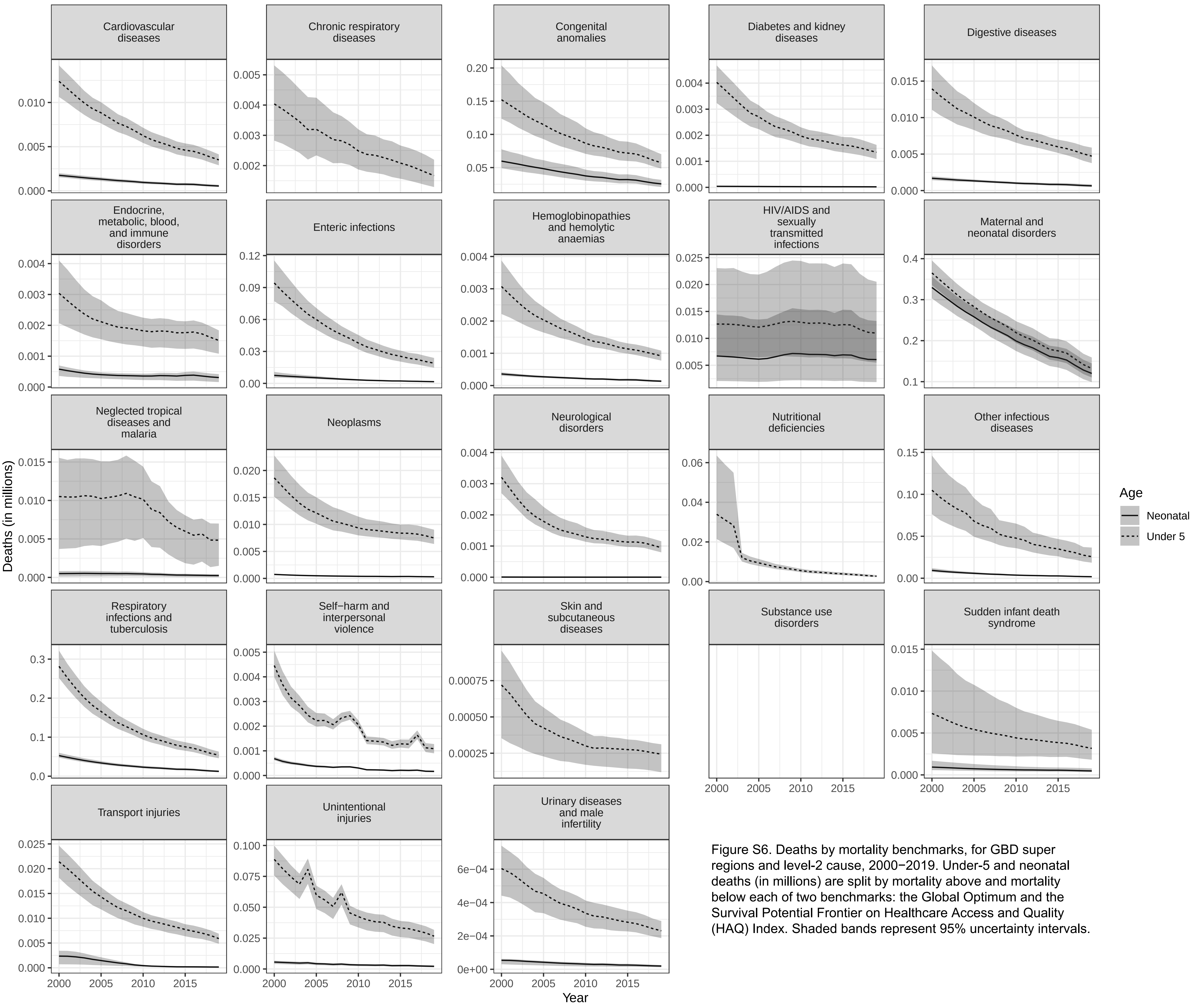

Figure S6. Deaths by mortality benchmarks, for GBD super regions and level-2 cause, 2000−2019. Under-5 and neonatal deaths (in millions) are split by mortality above and mortality below each of two benchmarks: the Global Optimum and the Survival Potential Frontier on Healthcare Access and Quality (HAQ) Index. Shaded bands represent 95% uncertainty intervals.

Figure S6ab. Above Survival Potential Frontier, Southeast Asia, East Asia, and Oceania

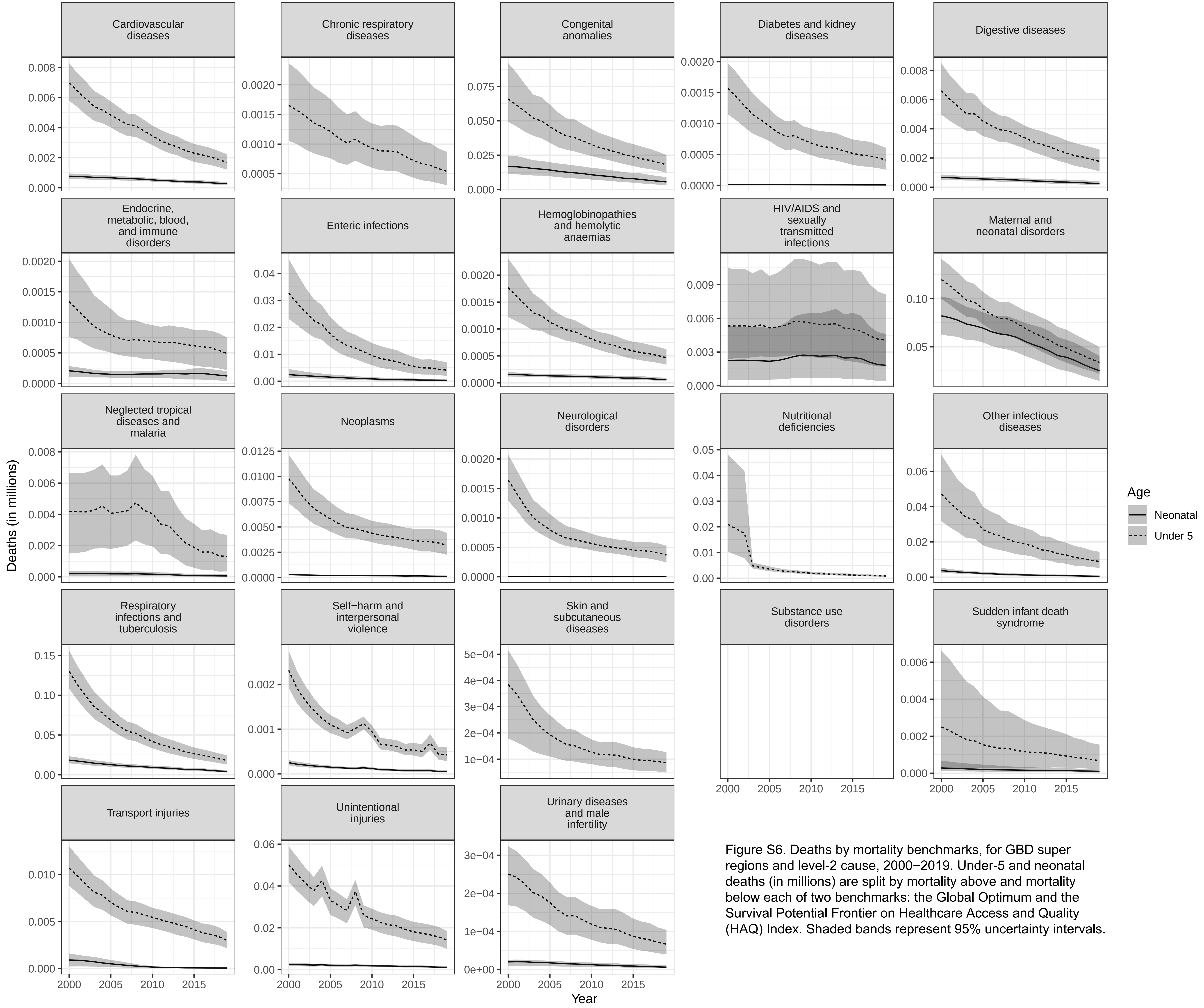

Figure S6. Deaths by mortality benchmarks, for GBD super regions and level-2 cause, 2000−2019. Under-5 and neonatal deaths (in millions) are split by mortality above and mortality below each of two benchmarks: the Global Optimum and the Survival Potential Frontier on Healthcare Access and Quality (HAQ) Index. Shaded bands represent 95% uncertainty intervals.

Figure S6ac. Below Global Optimum, Southeast Asia, East Asia, and Oceania

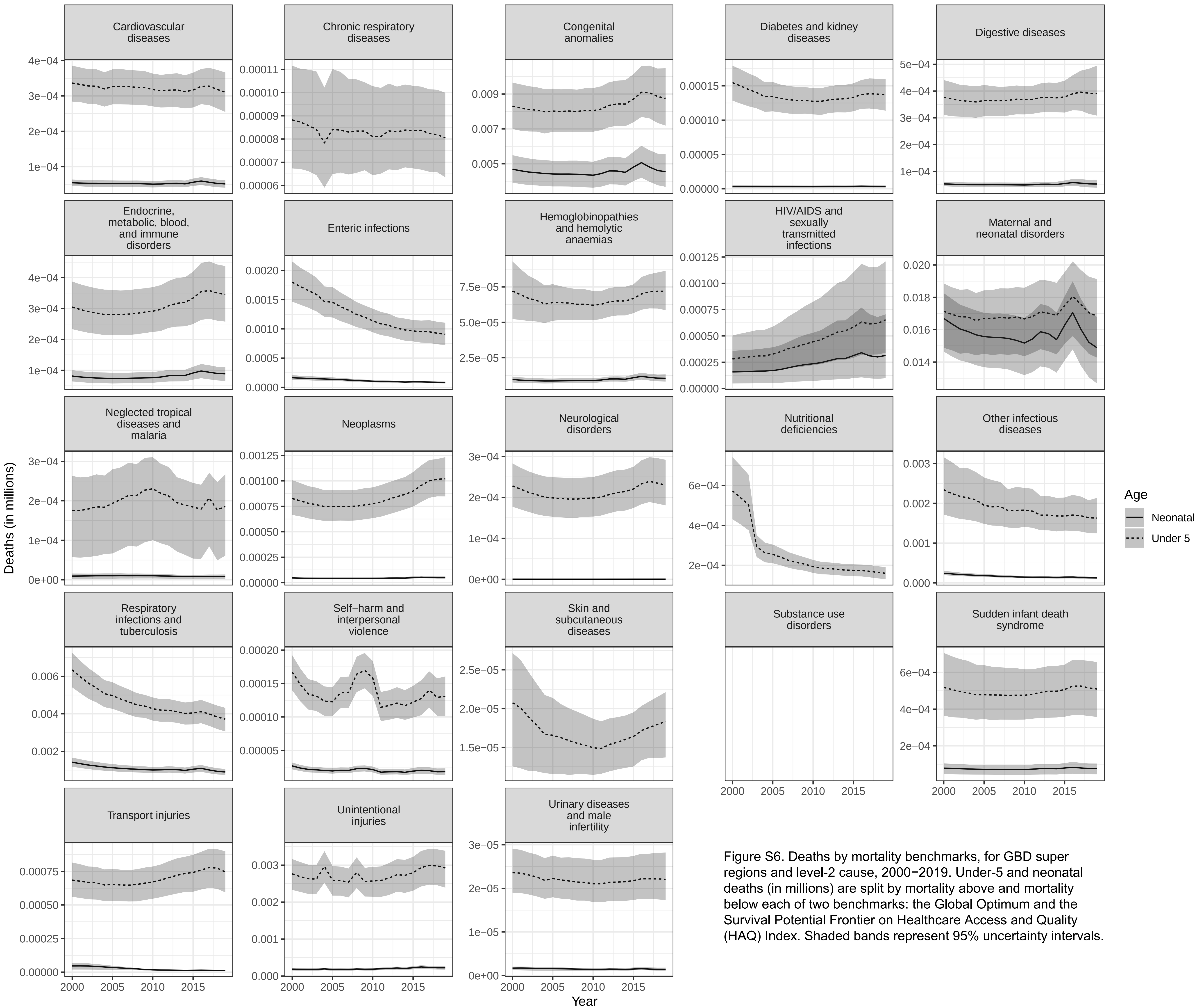

Figure S6. Deaths by mortality benchmarks, for GBD super regions and level-2 cause, 2000−2019. Under-5 and neonatal deaths (in millions) are split by mortality above and mortality below each of two benchmarks: the Global Optimum and the Survival Potential Frontier on Healthcare Access and Quality (HAQ) Index. Shaded bands represent 95% uncertainty intervals.

Figure S6ad. Below Survival Potential Frontier, Southeast Asia, East Asia, and Oceania

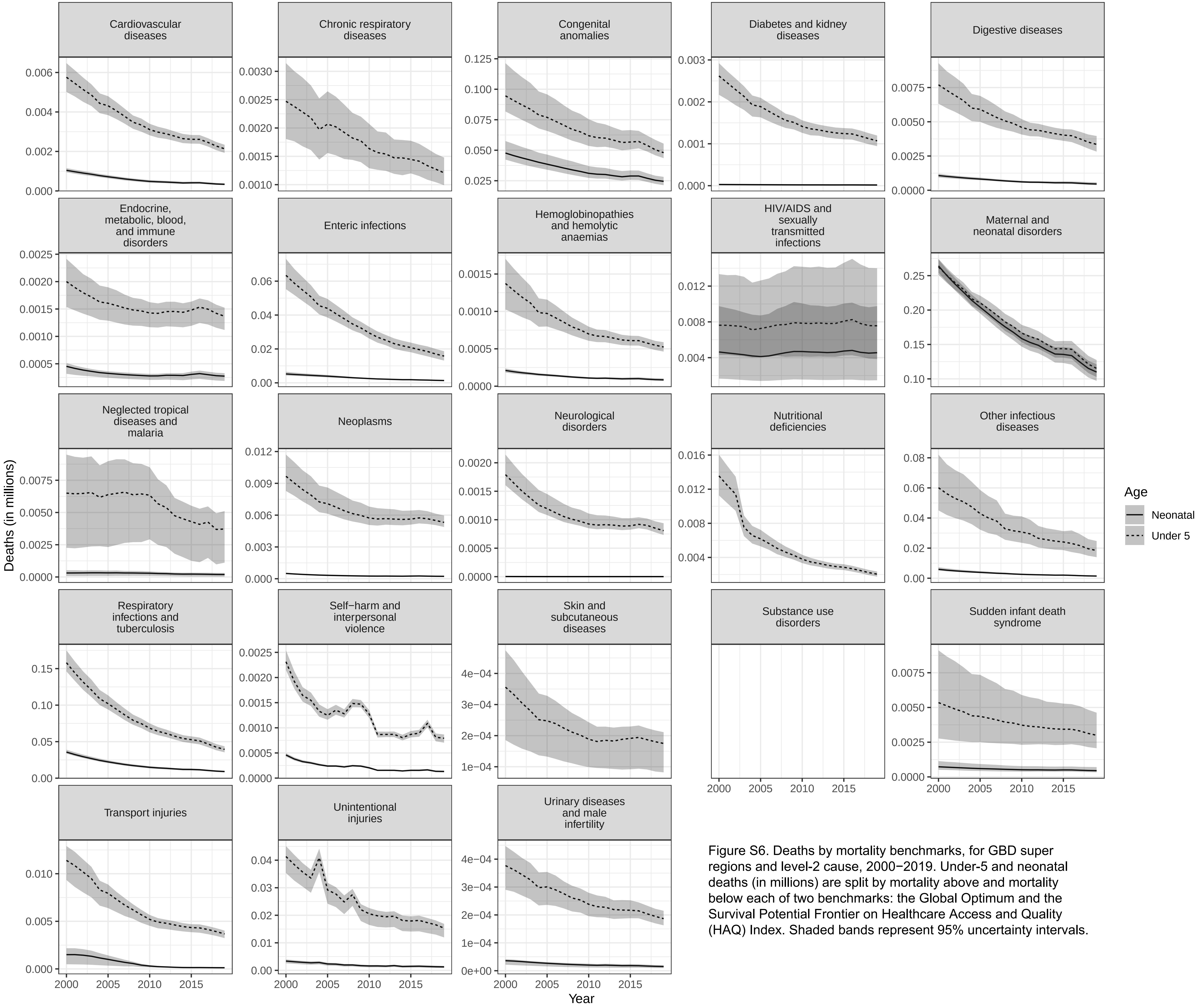

Figure S6. Deaths by mortality benchmarks, for GBD super regions and level-2 cause, 2000−2019. Under-5 and neonatal deaths (in millions) are split by mortality above and mortality below each of two benchmarks: the Global Optimum and the Survival Potential Frontier on Healthcare Access and Quality (HAQ) Index. Shaded bands represent 95% uncertainty intervals.

Figure S6ae. Above Global Optimum, Central Europe, Eastern Europe, and Central Asia

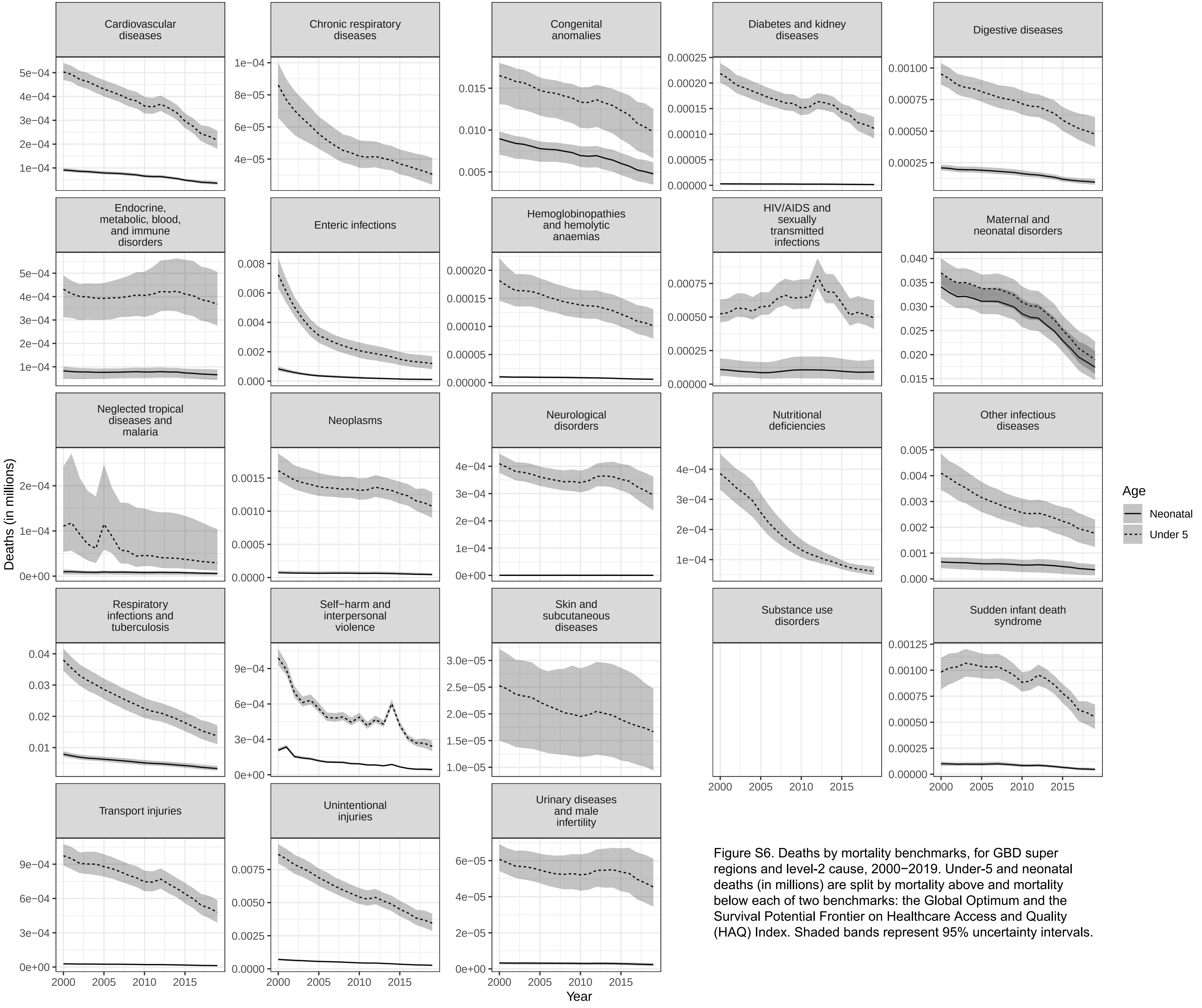

Figure S6. Deaths by mortality benchmarks, for GBD super regions and level-2 cause, 2000−2019. Under-5 and neonatal deaths (in millions) are split by mortality above and mortality below each of two benchmarks: the Global Optimum and the Survival Potential Frontier on Healthcare Access and Quality (HAQ) Index. Shaded bands represent 95% uncertainty intervals.

Figure S6af. Above Survival Potential Frontier, Central Europe, Eastern Europe, and Central Asia

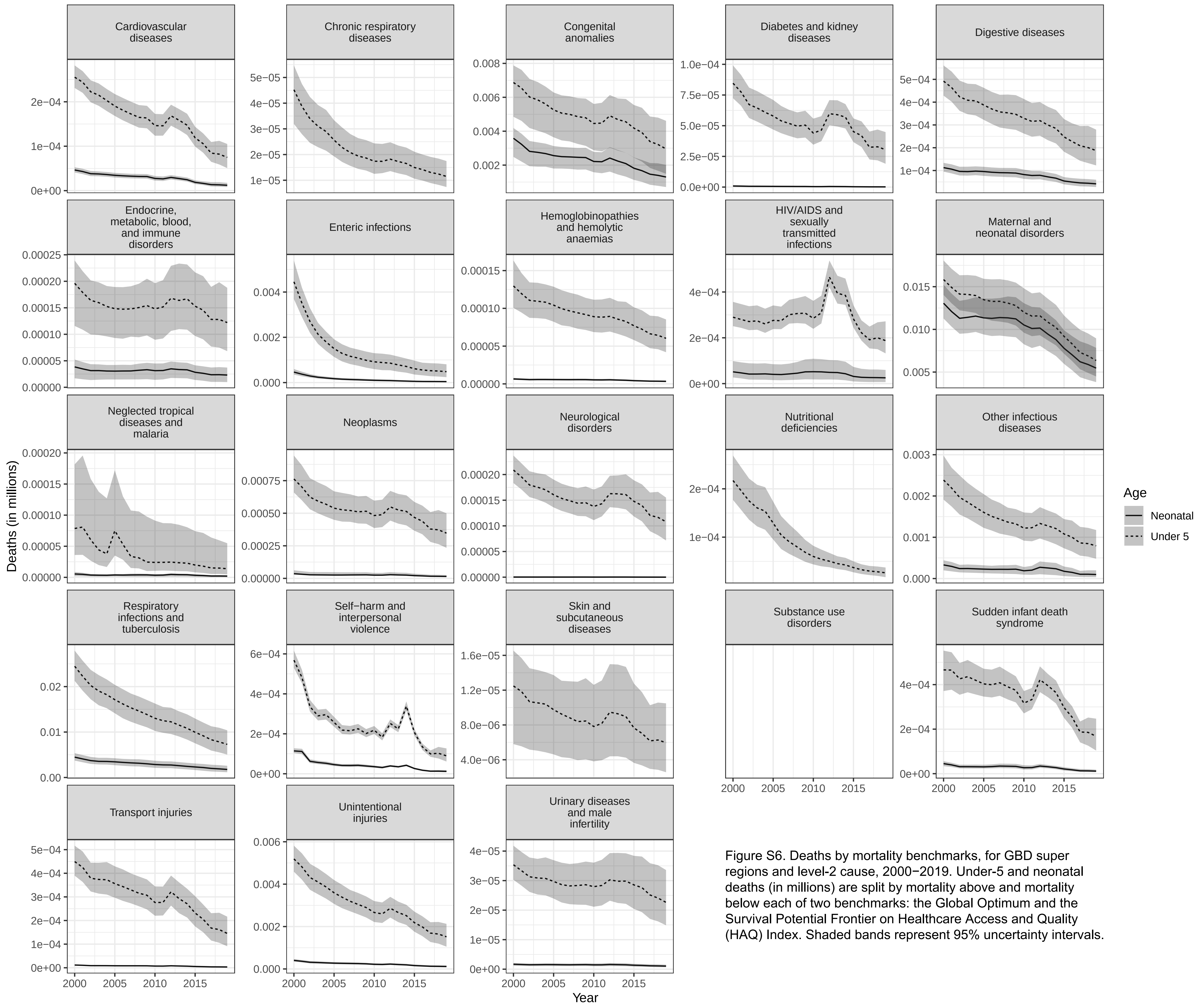

Figure S6. Deaths by mortality benchmarks, for GBD super regions and level-2 cause, 2000−2019. Under-5 and neonatal deaths (in millions) are split by mortality above and mortality below each of two benchmarks: the Global Optimum and the Survival Potential Frontier on Healthcare Access and Quality (HAQ) Index. Shaded bands represent 95% uncertainty intervals.

Figure S6ag. Below Global Optimum, Central Europe, Eastern Europe, and Central Asia

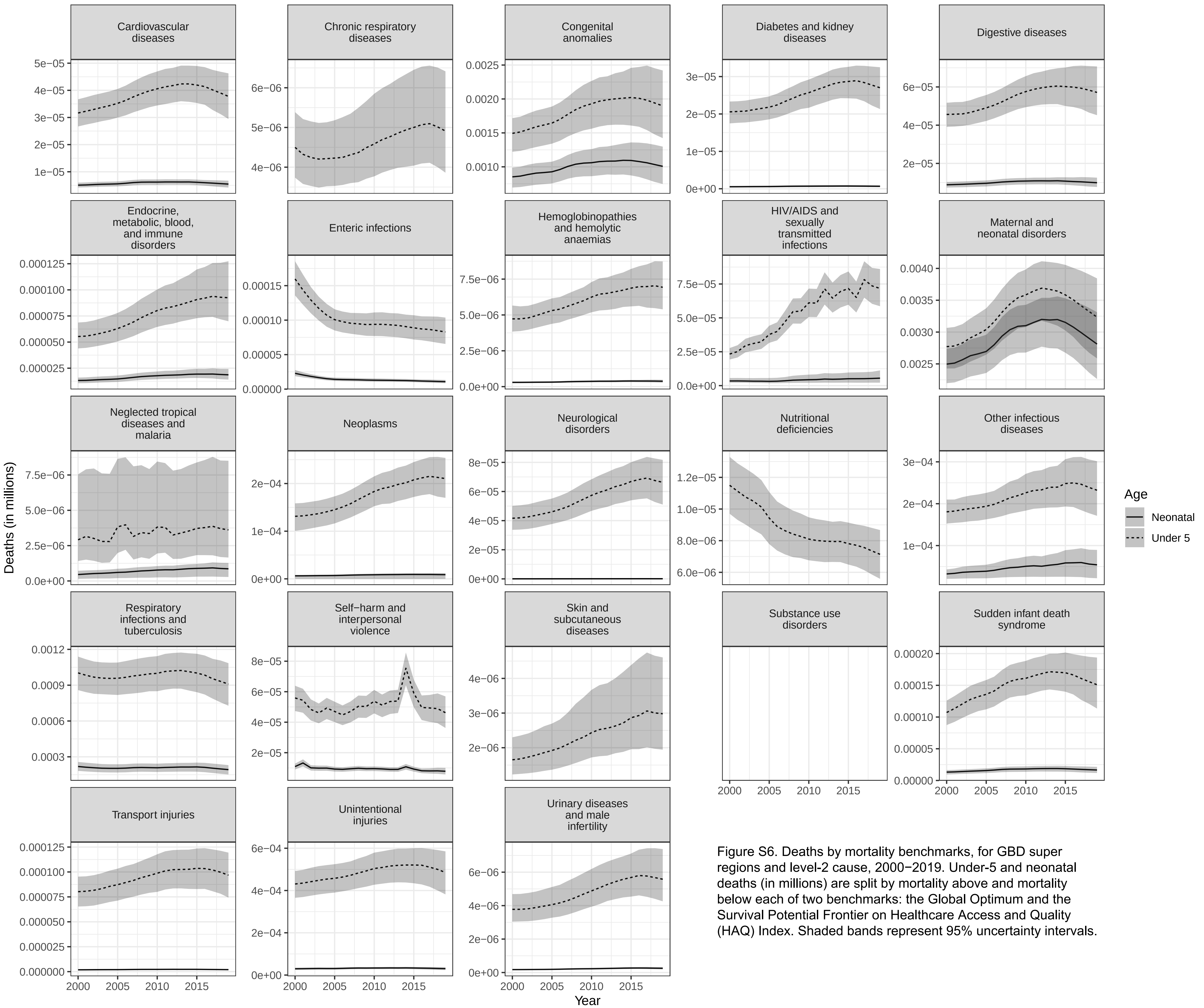

Figure S6. Deaths by mortality benchmarks, for GBD super regions and level-2 cause, 2000−2019. Under-5 and neonatal deaths (in millions) are split by mortality above and mortality below each of two benchmarks: the Global Optimum and the Survival Potential Frontier on Healthcare Access and Quality (HAQ) Index. Shaded bands represent 95% uncertainty intervals.

Figure S6ah. Below Survival Potential Frontier, Central Europe, Eastern Europe, and Central Asia

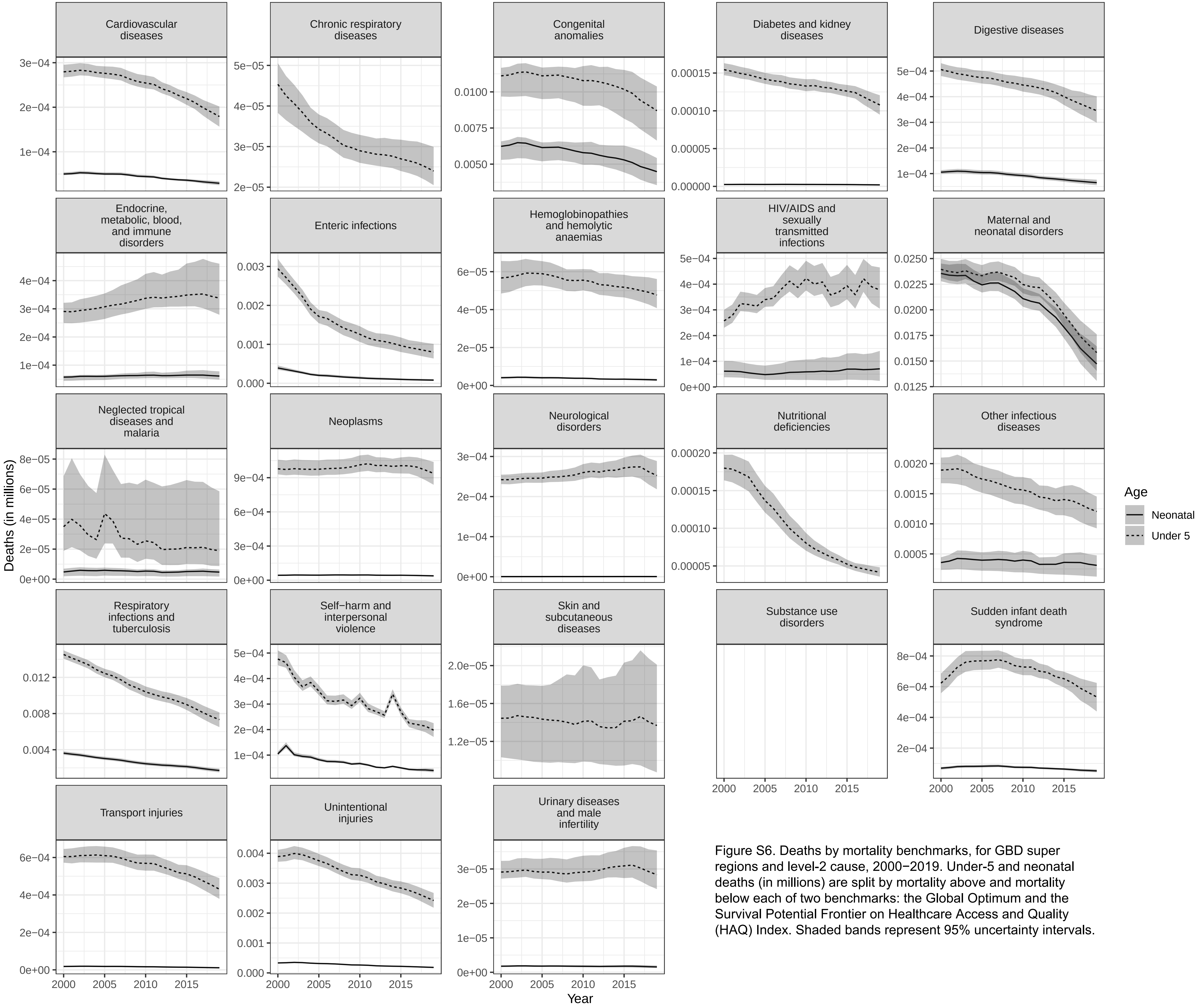

Figure S6. Deaths by mortality benchmarks, for GBD super regions and level-2 cause, 2000−2019. Under-5 and neonatal deaths (in millions) are split by mortality above and mortality below each of two benchmarks: the Global Optimum and the Survival Potential Frontier on Healthcare Access and Quality (HAQ) Index. Shaded bands represent 95% uncertainty intervals.

Figure S6ai. Above Global Optimum, High−income

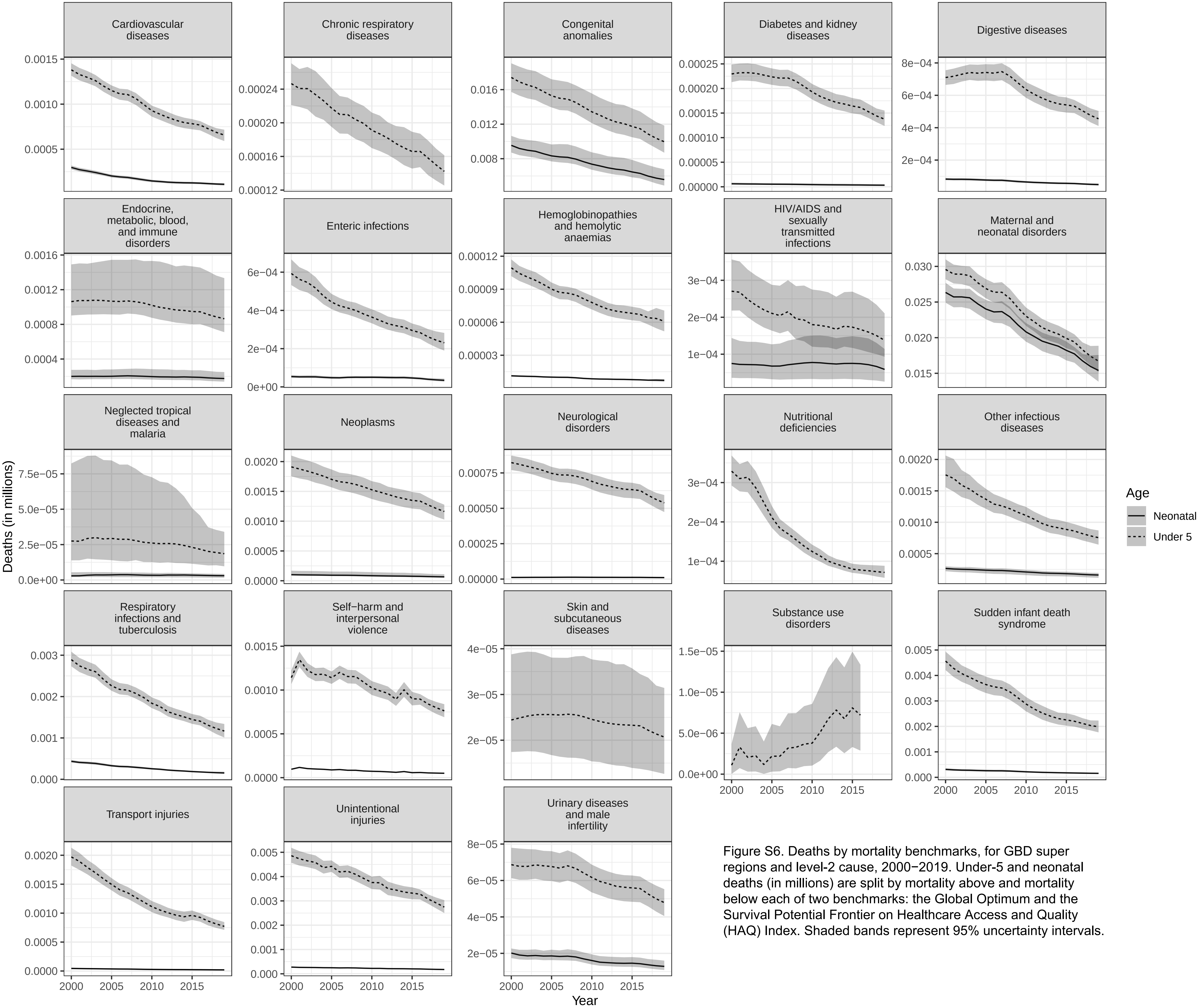

Figure S6. Deaths by mortality benchmarks, for GBD super regions and level-2 cause, 2000−2019. Under-5 and neonatal deaths (in millions) are split by mortality above and mortality below each of two benchmarks: the Global Optimum and the Survival Potential Frontier on Healthcare Access and Quality (HAQ) Index. Shaded bands represent 95% uncertainty intervals.

Figure S6aj. Above Survival Potential Frontier, High−income

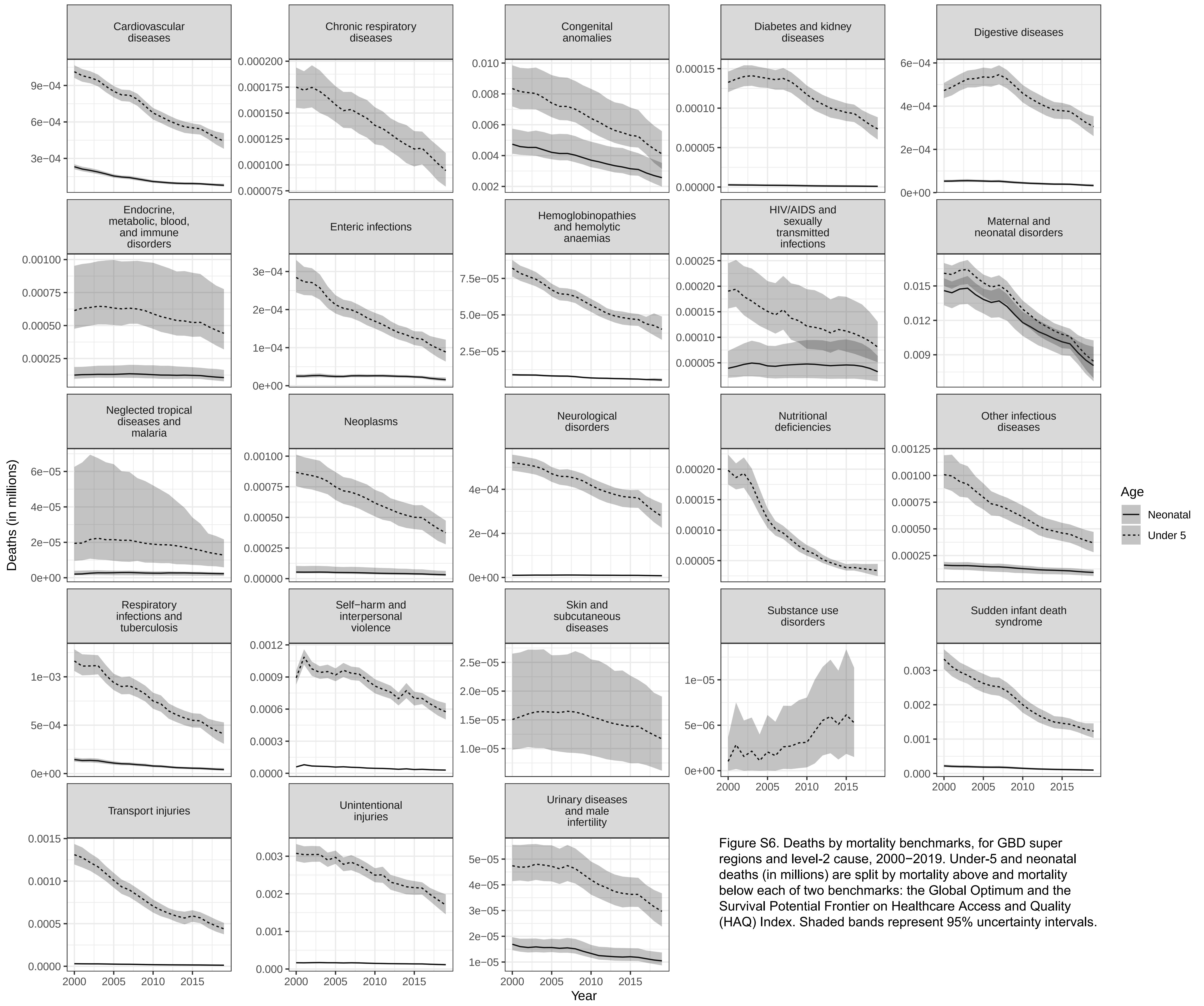

Figure S6. Deaths by mortality benchmarks, for GBD super regions and level-2 cause, 2000−2019. Under-5 and neonatal deaths (in millions) are split by mortality above and mortality below each of two benchmarks: the Global Optimum and the Survival Potential Frontier on Healthcare Access and Quality (HAQ) Index. Shaded bands represent 95% uncertainty intervals.

Figure S6ak. Below Global Optimum, High−income

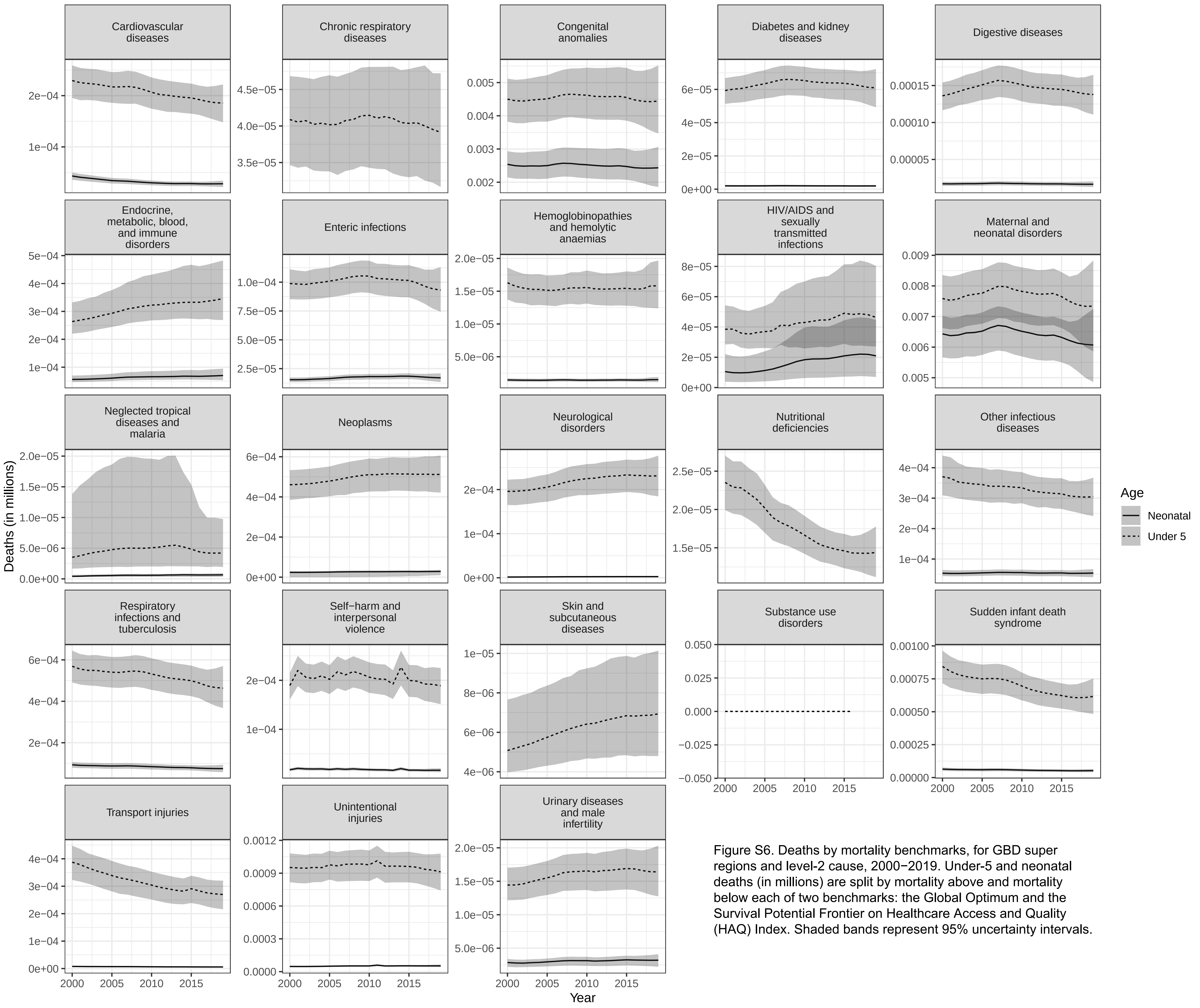

Figure S6. Deaths by mortality benchmarks, for GBD super regions and level-2 cause, 2000−2019. Under-5 and neonatal deaths (in millions) are split by mortality above and mortality below each of two benchmarks: the Global Optimum and the Survival Potential Frontier on Healthcare Access and Quality (HAQ) Index. Shaded bands represent 95% uncertainty intervals.

Figure S6al. Below Survival Potential Frontier, High−income

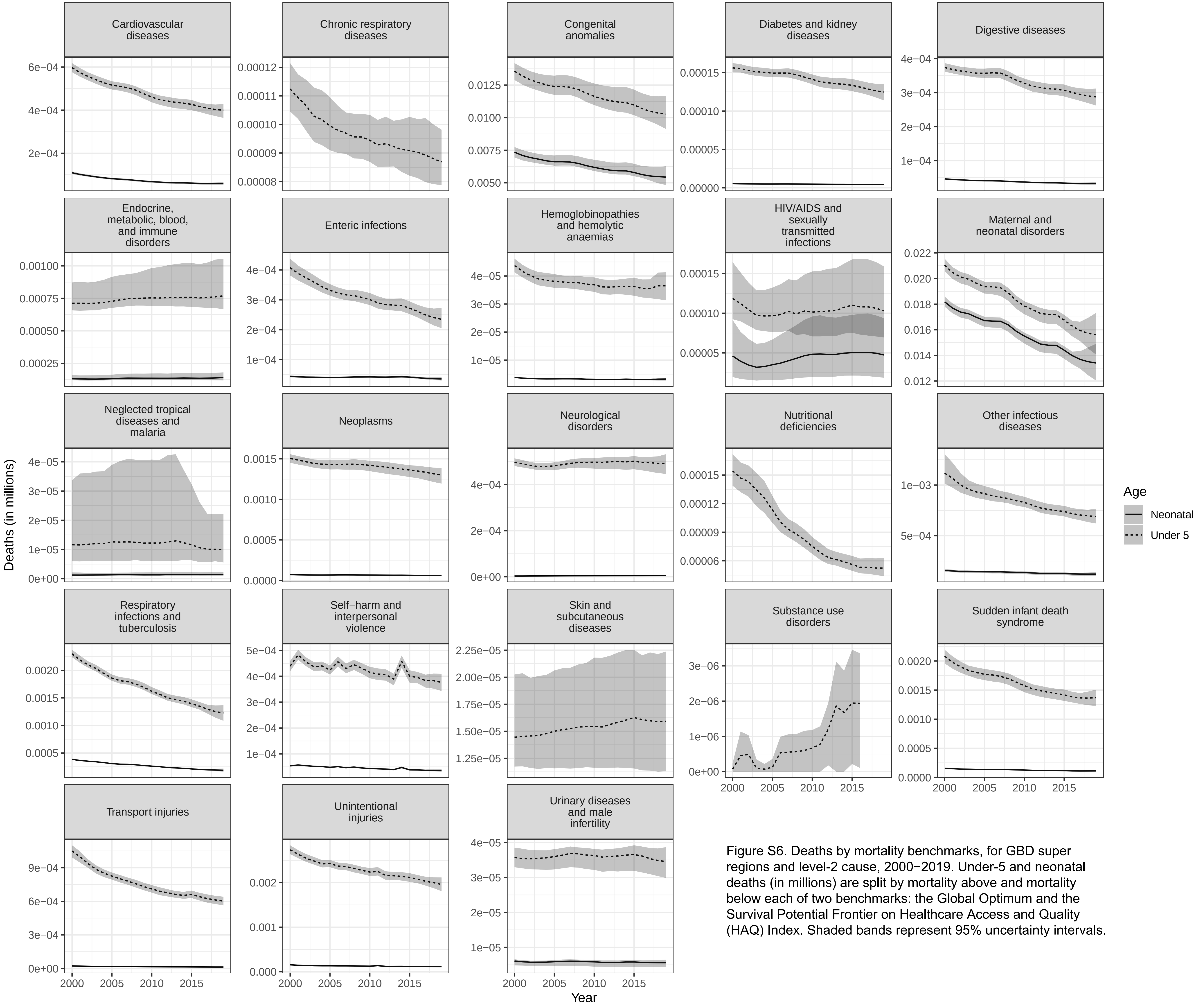

Figure S6. Deaths by mortality benchmarks, for GBD super regions and level-2 cause, 2000−2019. Under-5 and neonatal deaths (in millions) are split by mortality above and mortality below each of two benchmarks: the Global Optimum and the Survival Potential Frontier on Healthcare Access and Quality (HAQ) Index. Shaded bands represent 95% uncertainty intervals.

Figure S6am. Above Global Optimum, Latin America and Caribbean

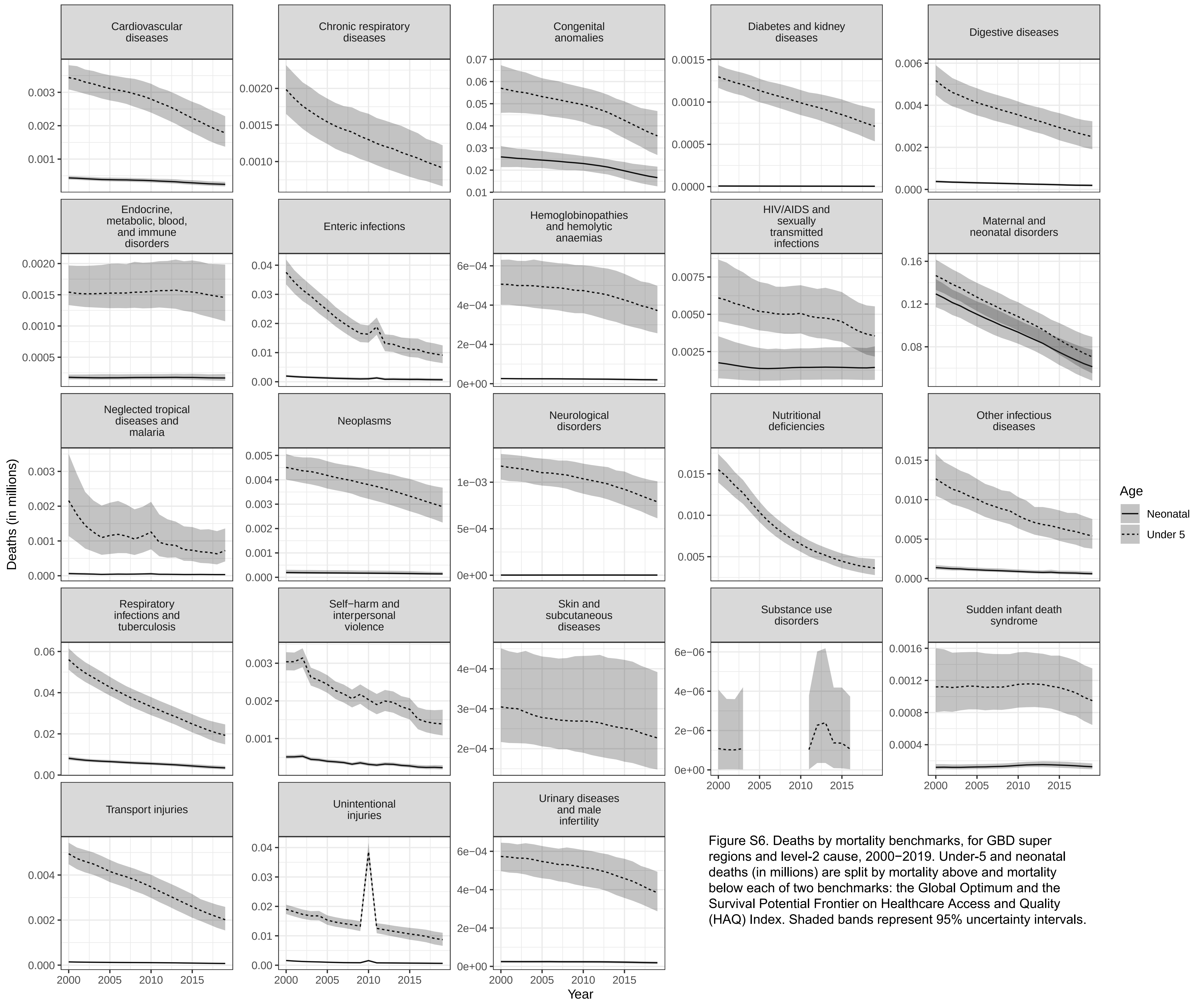

Figure S6. Deaths by mortality benchmarks, for GBD super regions and level-2 cause, 2000−2019. Under-5 and neonatal deaths (in millions) are split by mortality above and mortality below each of two benchmarks: the Global Optimum and the Survival Potential Frontier on Healthcare Access and Quality (HAQ) Index. Shaded bands represent 95% uncertainty intervals.

Figure S6an. Above Survival Potential Frontier, Latin America and Caribbean

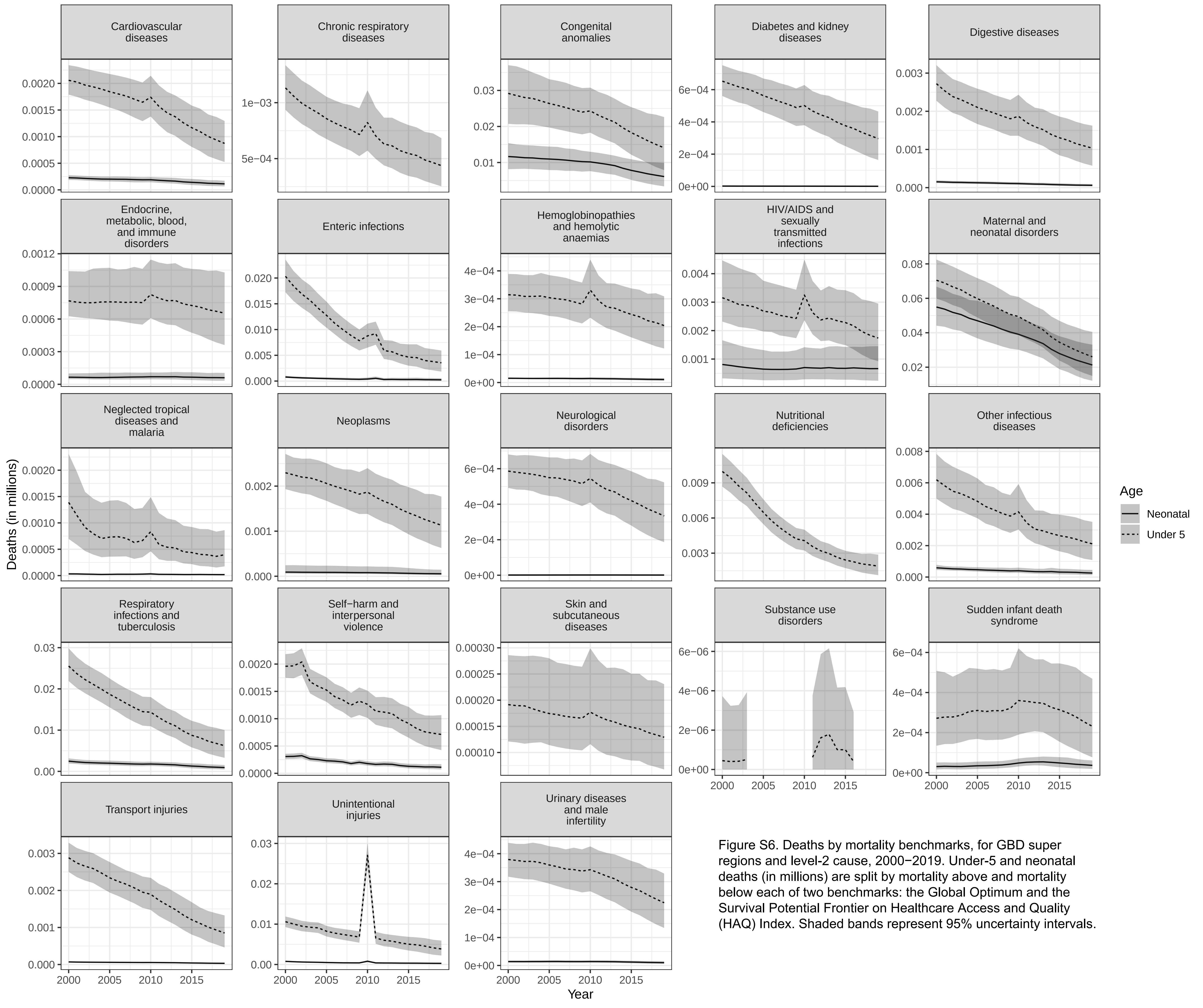

Figure S6. Deaths by mortality benchmarks, for GBD super regions and level-2 cause, 2000−2019. Under-5 and neonatal deaths (in millions) are split by mortality above and mortality below each of two benchmarks: the Global Optimum and the Survival Potential Frontier on Healthcare Access and Quality (HAQ) Index. Shaded bands represent 95% uncertainty intervals.

Figure S6ao. Below Global Optimum, Latin America and Caribbean

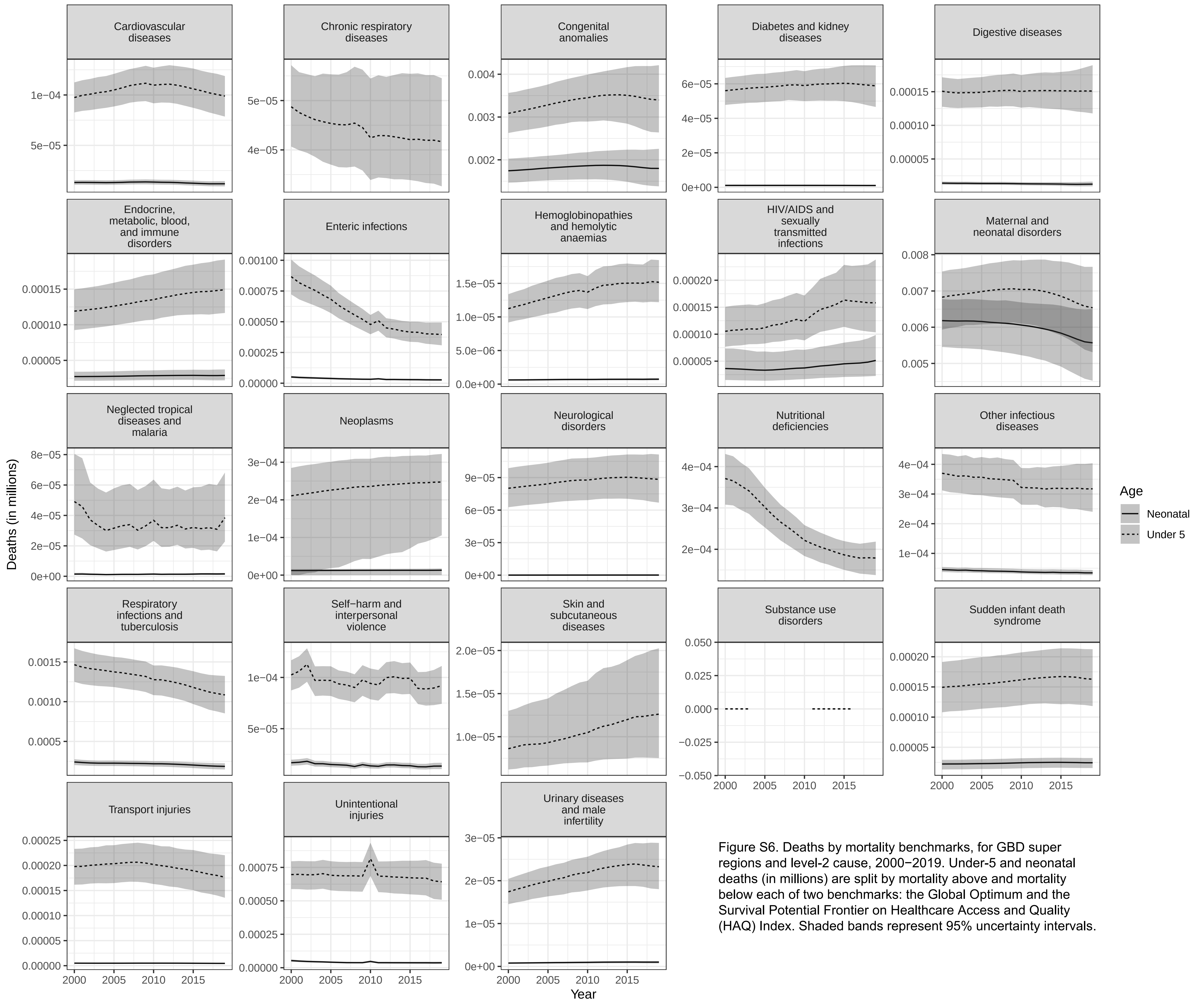

Figure S6. Deaths by mortality benchmarks, for GBD super regions and level-2 cause, 2000−2019. Under-5 and neonatal deaths (in millions) are split by mortality above and mortality below each of two benchmarks: the Global Optimum and the Survival Potential Frontier on Healthcare Access and Quality (HAQ) Index. Shaded bands represent 95% uncertainty intervals.

Figure S6ap. Below Survival Potential Frontier, Latin America and Caribbean

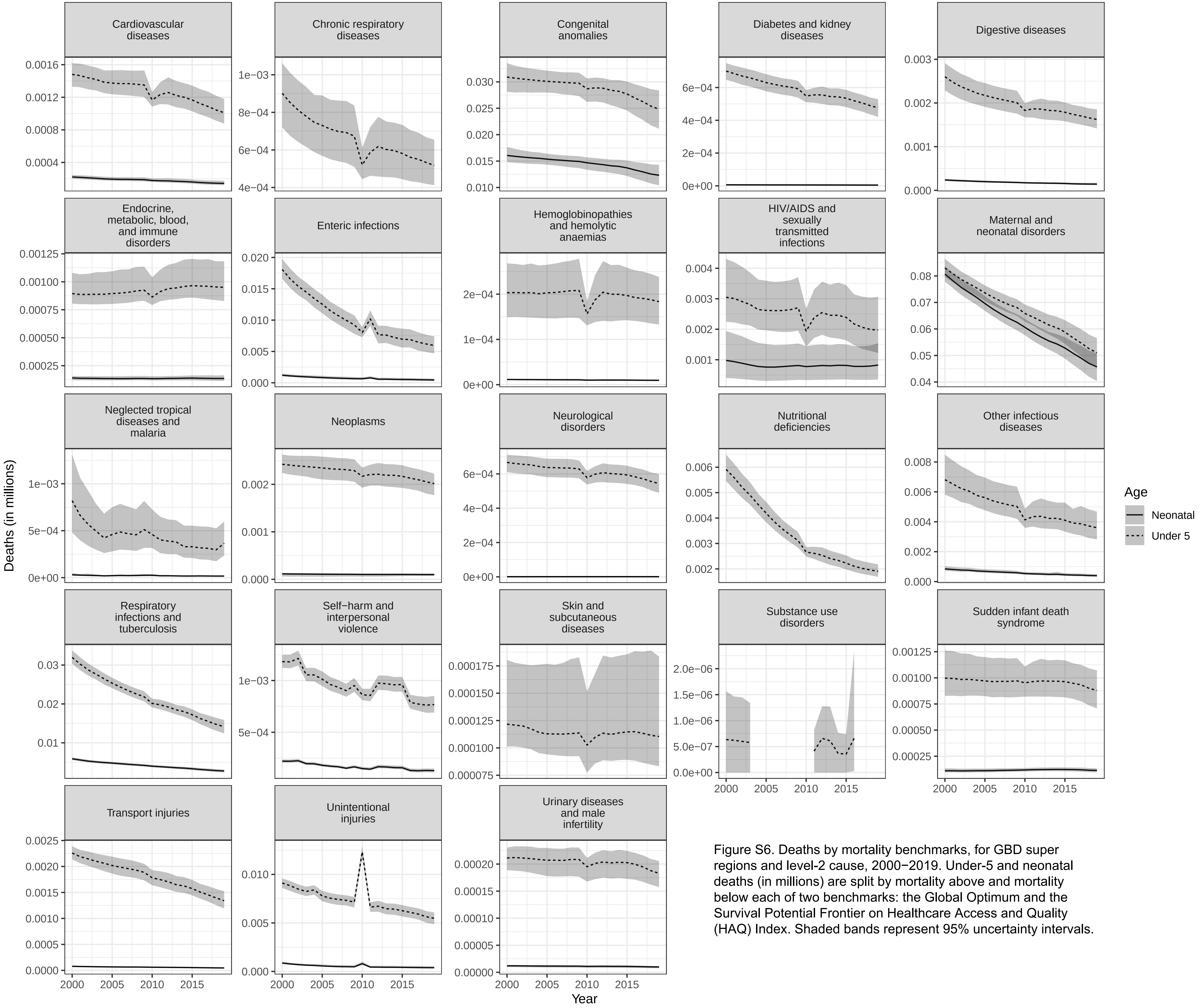

Figure S6. Deaths by mortality benchmarks, for GBD super regions and level-2 cause, 2000−2019. Under-5 and neonatal deaths (in millions) are split by mortality above and mortality below each of two benchmarks: the Global Optimum and the Survival Potential Frontier on Healthcare Access and Quality (HAQ) Index. Shaded bands represent 95% uncertainty intervals.

Figure S6aq. Above Global Optimum, North Africa and Middle East

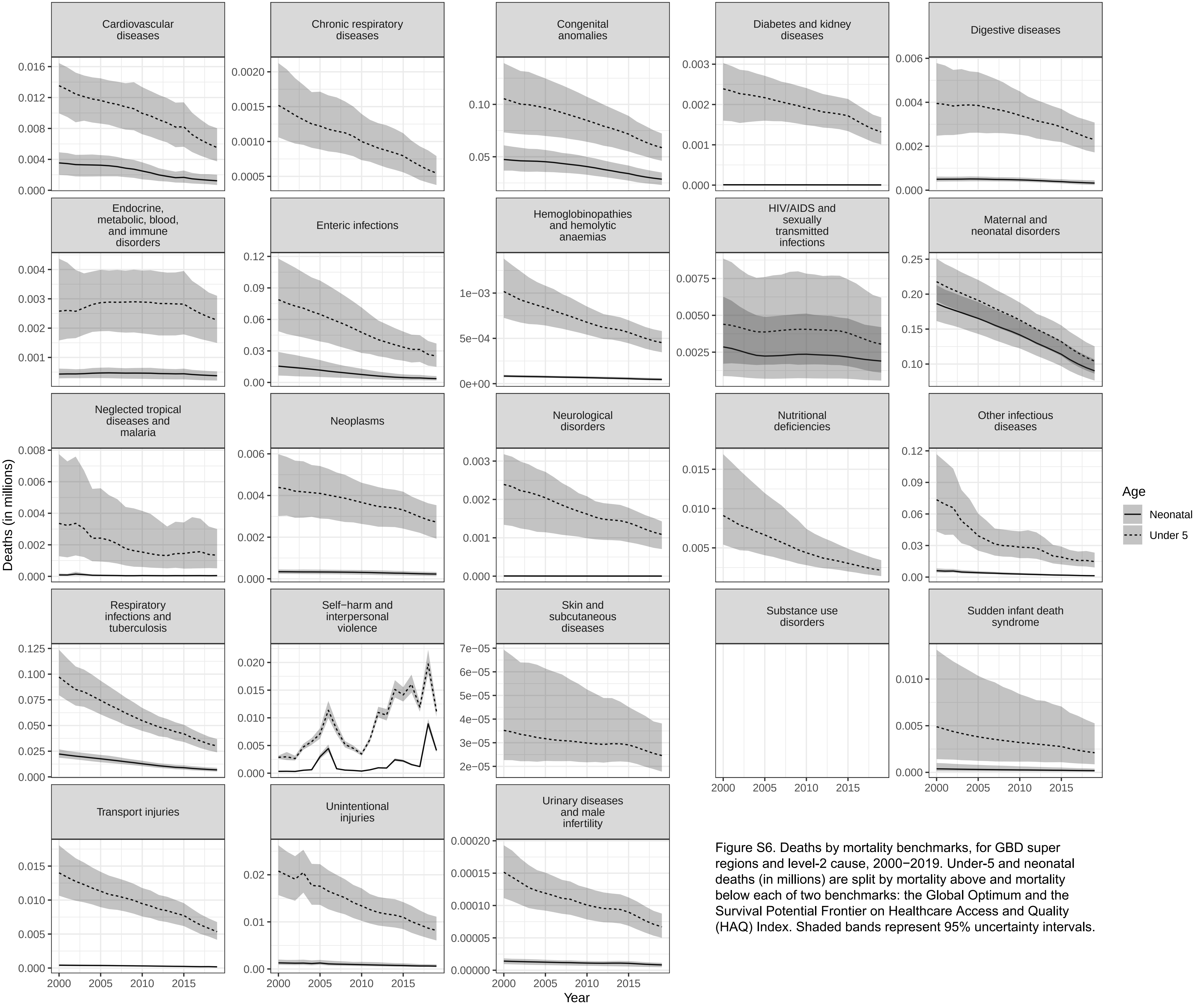

Figure S6. Deaths by mortality benchmarks, for GBD super regions and level-2 cause, 2000−2019. Under-5 and neonatal deaths (in millions) are split by mortality above and mortality below each of two benchmarks: the Global Optimum and the Survival Potential Frontier on Healthcare Access and Quality (HAQ) Index. Shaded bands represent 95% uncertainty intervals.

Figure S6ar. Above Survival Potential Frontier, North Africa and Middle East

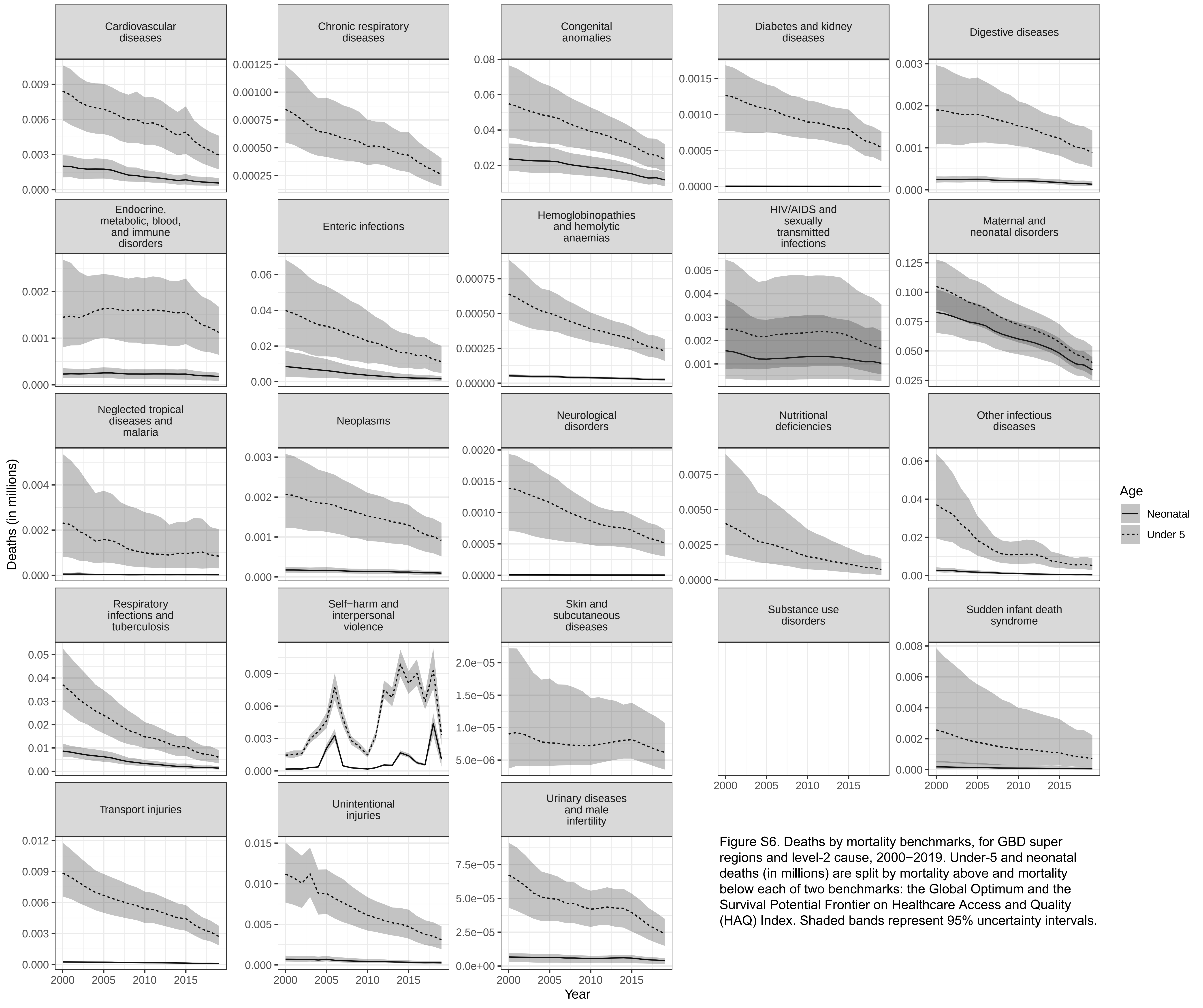

Figure S6. Deaths by mortality benchmarks, for GBD super regions and level-2 cause, 2000−2019. Under-5 and neonatal deaths (in millions) are split by mortality above and mortality below each of two benchmarks: the Global Optimum and the Survival Potential Frontier on Healthcare Access and Quality (HAQ) Index. Shaded bands represent 95% uncertainty intervals.

Figure S6as. Below Global Optimum, North Africa and Middle East

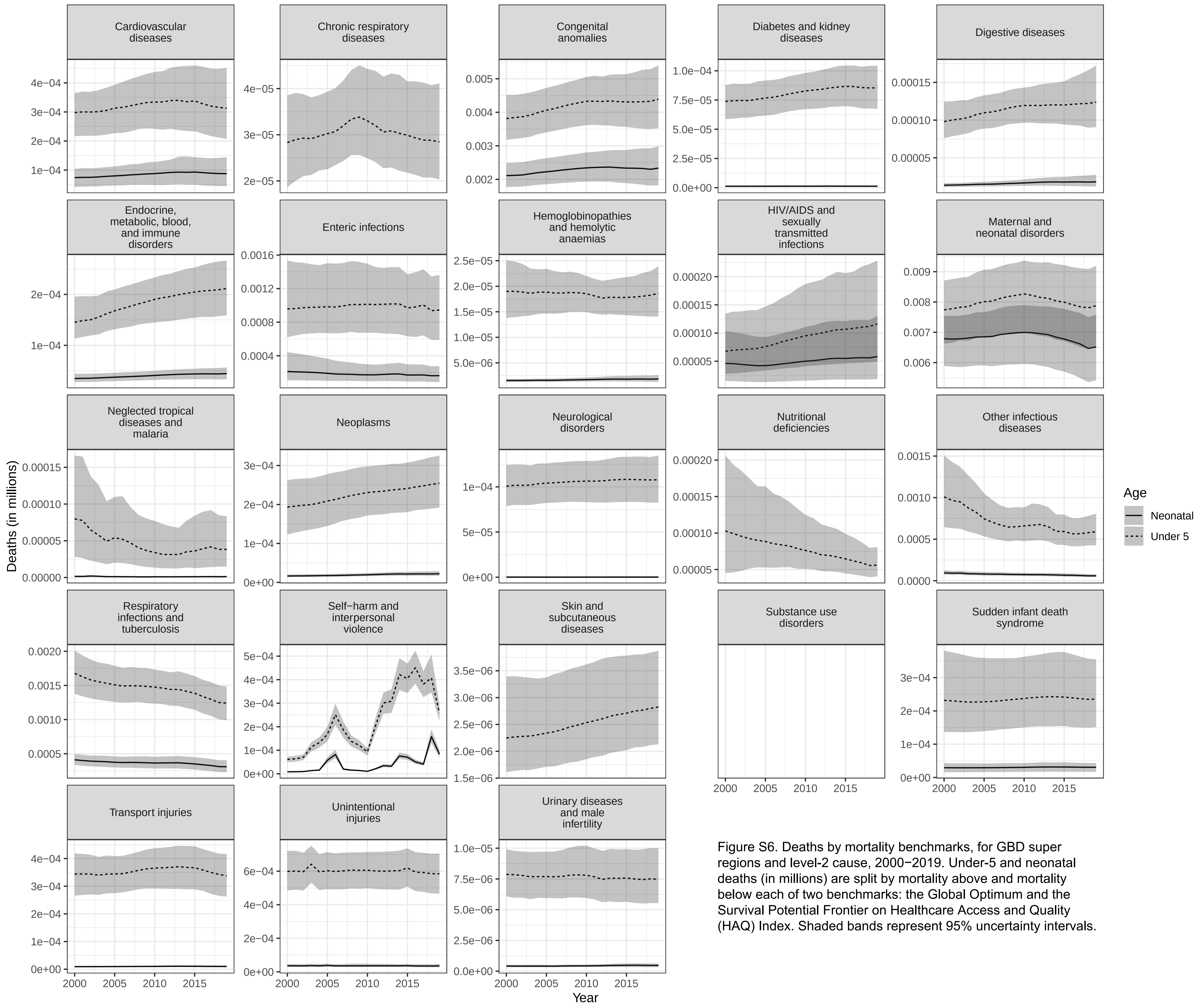

Figure S6. Deaths by mortality benchmarks, for GBD super regions and level-2 cause, 2000−2019. Under-5 and neonatal deaths (in millions) are split by mortality above and mortality below each of two benchmarks: the Global Optimum and the Survival Potential Frontier on Healthcare Access and Quality (HAQ) Index. Shaded bands represent 95% uncertainty intervals.

Figure S6at. Below Survival Potential Frontier, North Africa and Middle East

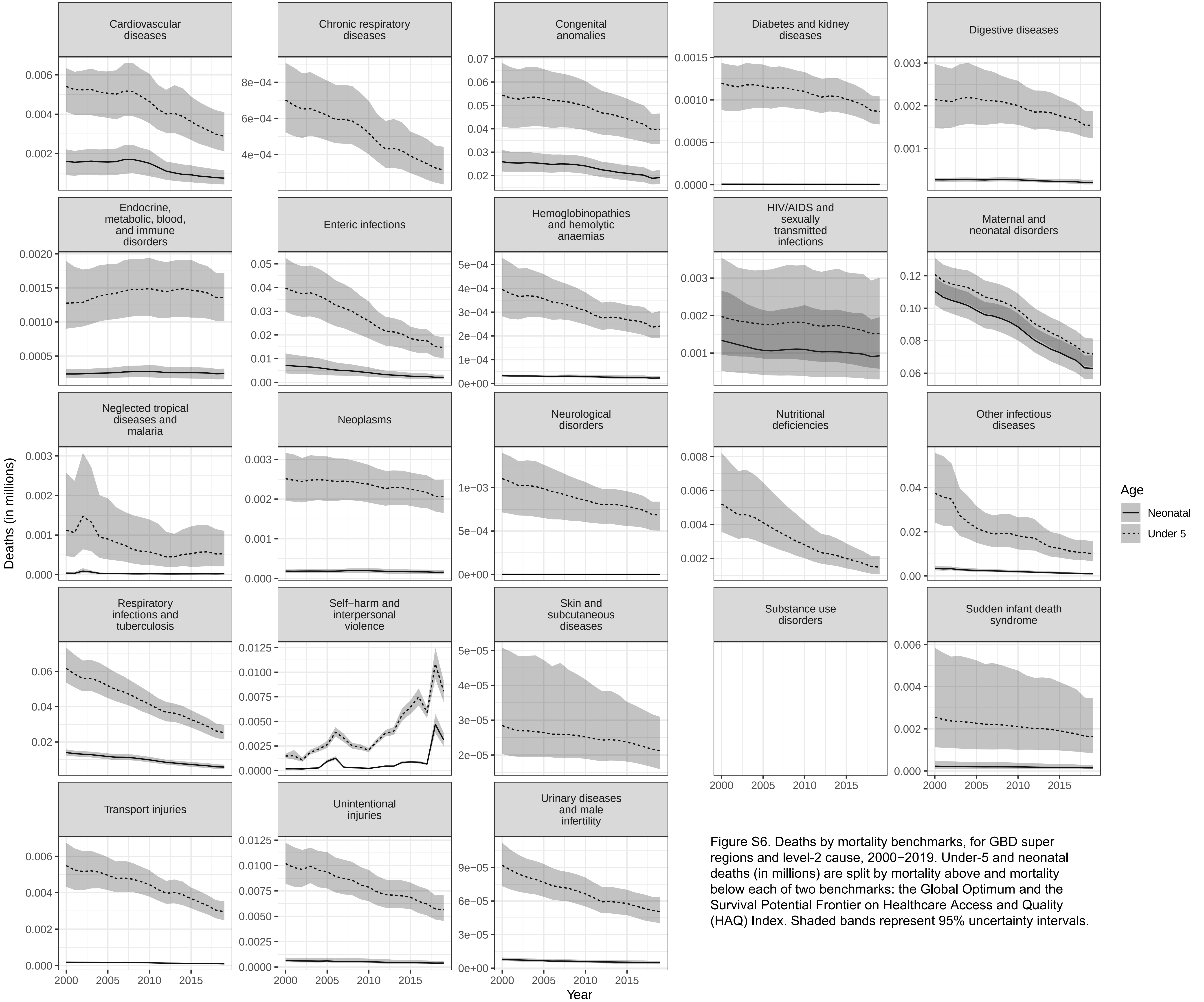

Figure S6. Deaths by mortality benchmarks, for GBD super regions and level-2 cause, 2000−2019. Under-5 and neonatal deaths (in millions) are split by mortality above and mortality below each of two benchmarks: the Global Optimum and the Survival Potential Frontier on Healthcare Access and Quality (HAQ) Index. Shaded bands represent 95% uncertainty intervals.

Figure S6au. Above Global Optimum, South Asia

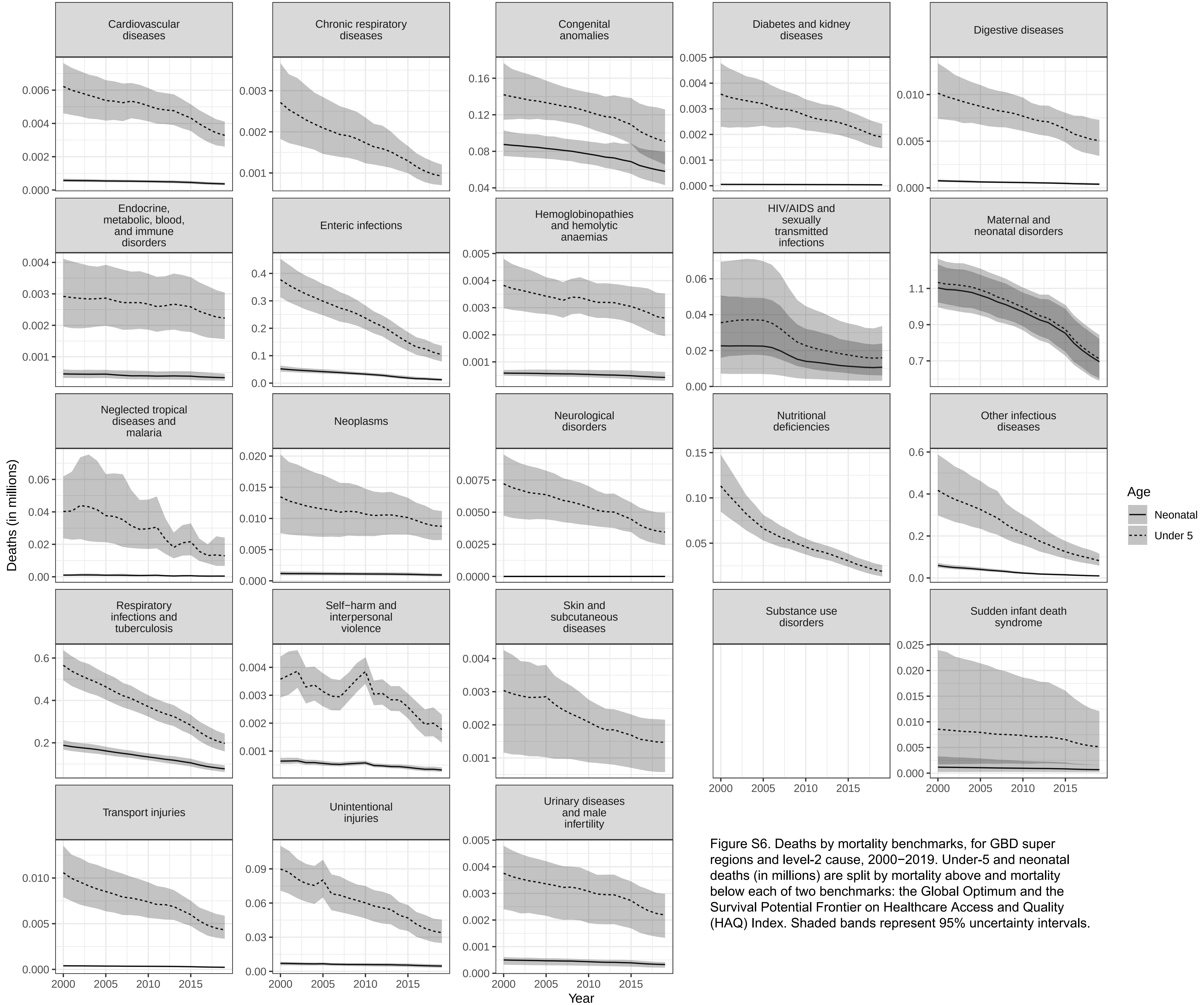

Figure S6. Deaths by mortality benchmarks, for GBD super regions and level-2 cause, 2000−2019. Under-5 and neonatal deaths (in millions) are split by mortality above and mortality below each of two benchmarks: the Global Optimum and the Survival Potential Frontier on Healthcare Access and Quality (HAQ) Index. Shaded bands represent 95% uncertainty intervals.

Figure S6av. Above Survival Potential Frontier, South Asia

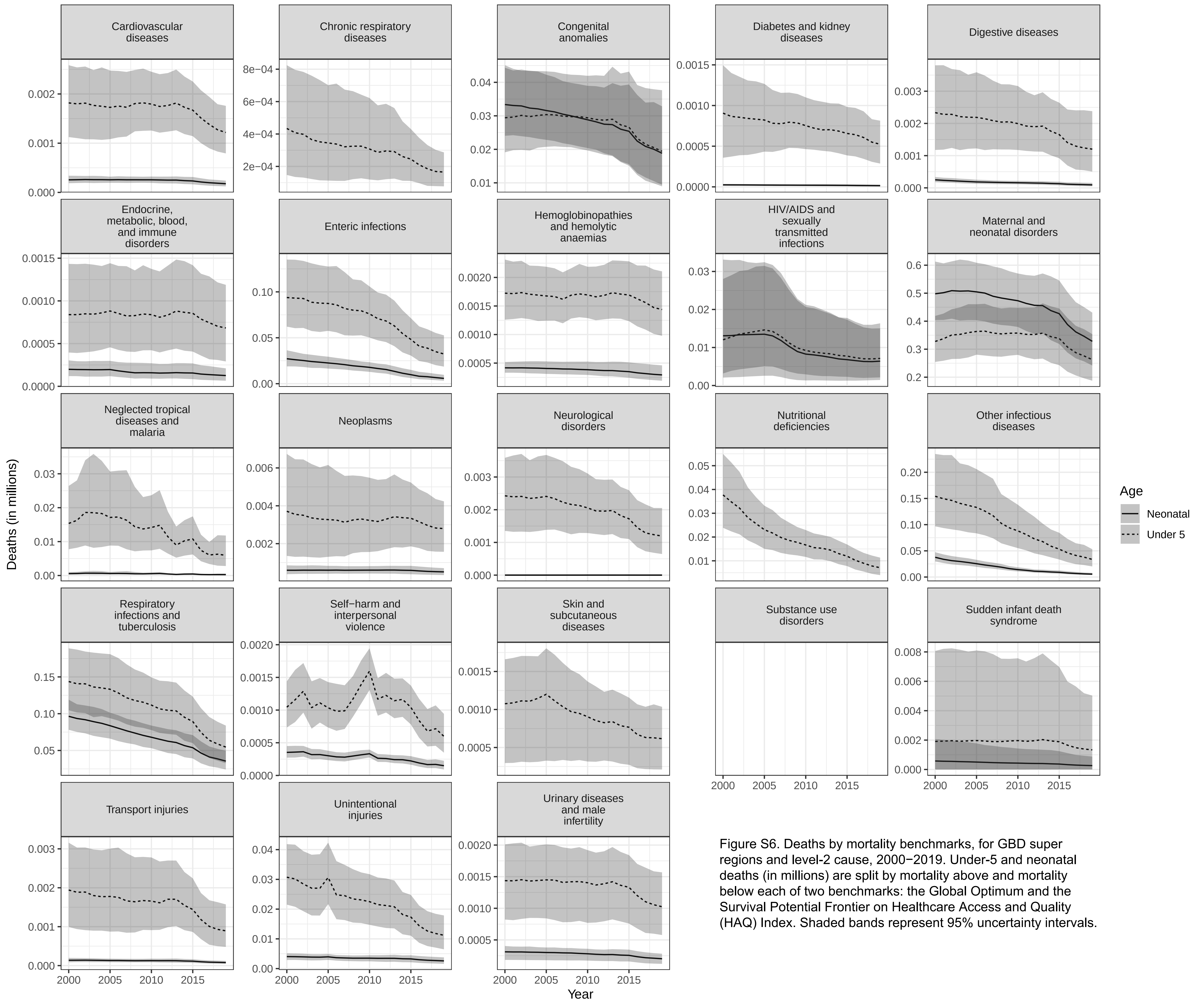

Figure S6. Deaths by mortality benchmarks, for GBD super regions and level-2 cause, 2000−2019. Under-5 and neonatal deaths (in millions) are split by mortality above and mortality below each of two benchmarks: the Global Optimum and the Survival Potential Frontier on Healthcare Access and Quality (HAQ) Index. Shaded bands represent 95% uncertainty intervals.

Figure S6aw. Below Global Optimum, South Asia

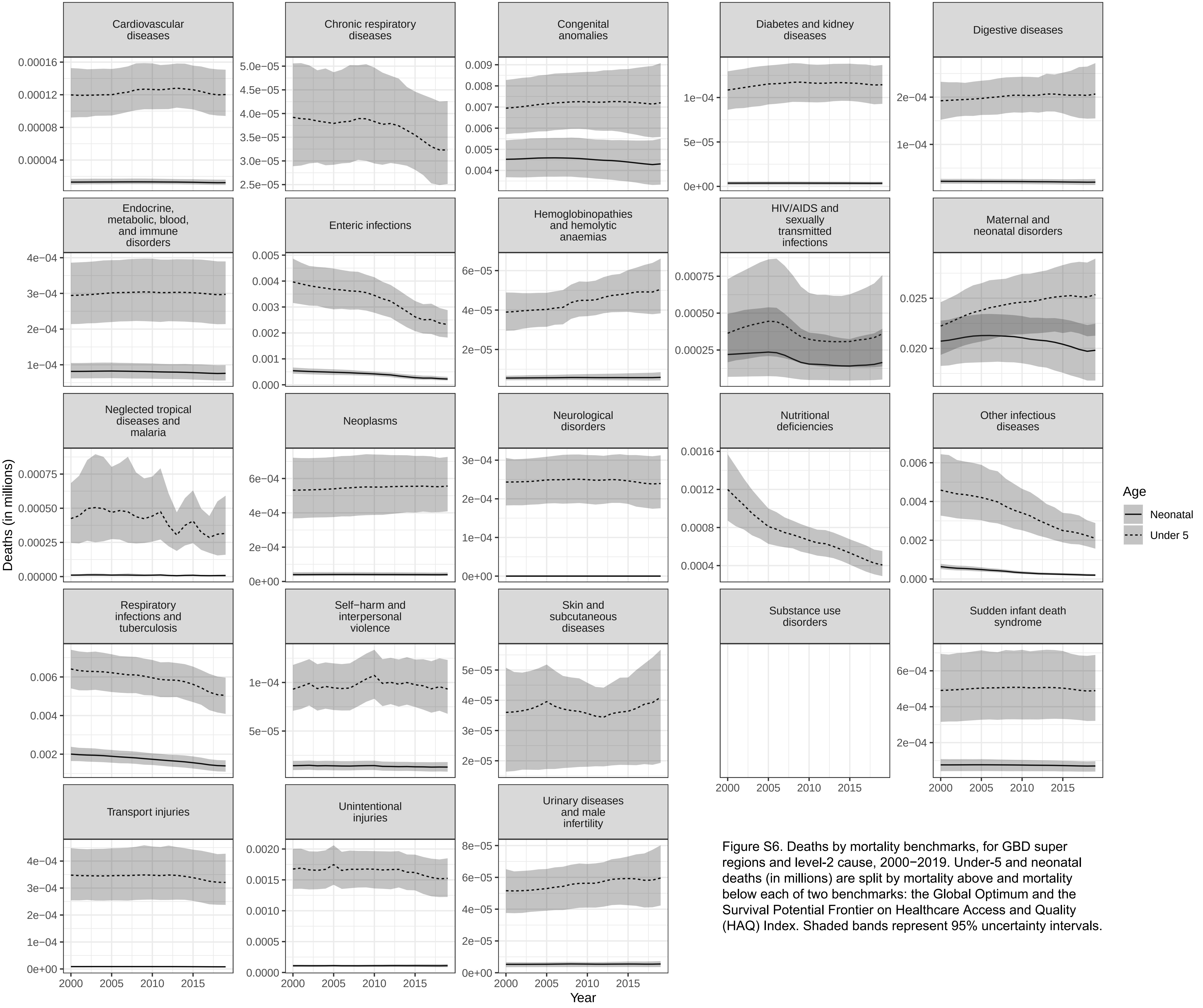

Figure S6. Deaths by mortality benchmarks, for GBD super regions and level-2 cause, 2000−2019. Under-5 and neonatal deaths (in millions) are split by mortality above and mortality below each of two benchmarks: the Global Optimum and the Survival Potential Frontier on Healthcare Access and Quality (HAQ) Index. Shaded bands represent 95% uncertainty intervals.

Figure S6ax. Below Survival Potential Frontier, South Asia

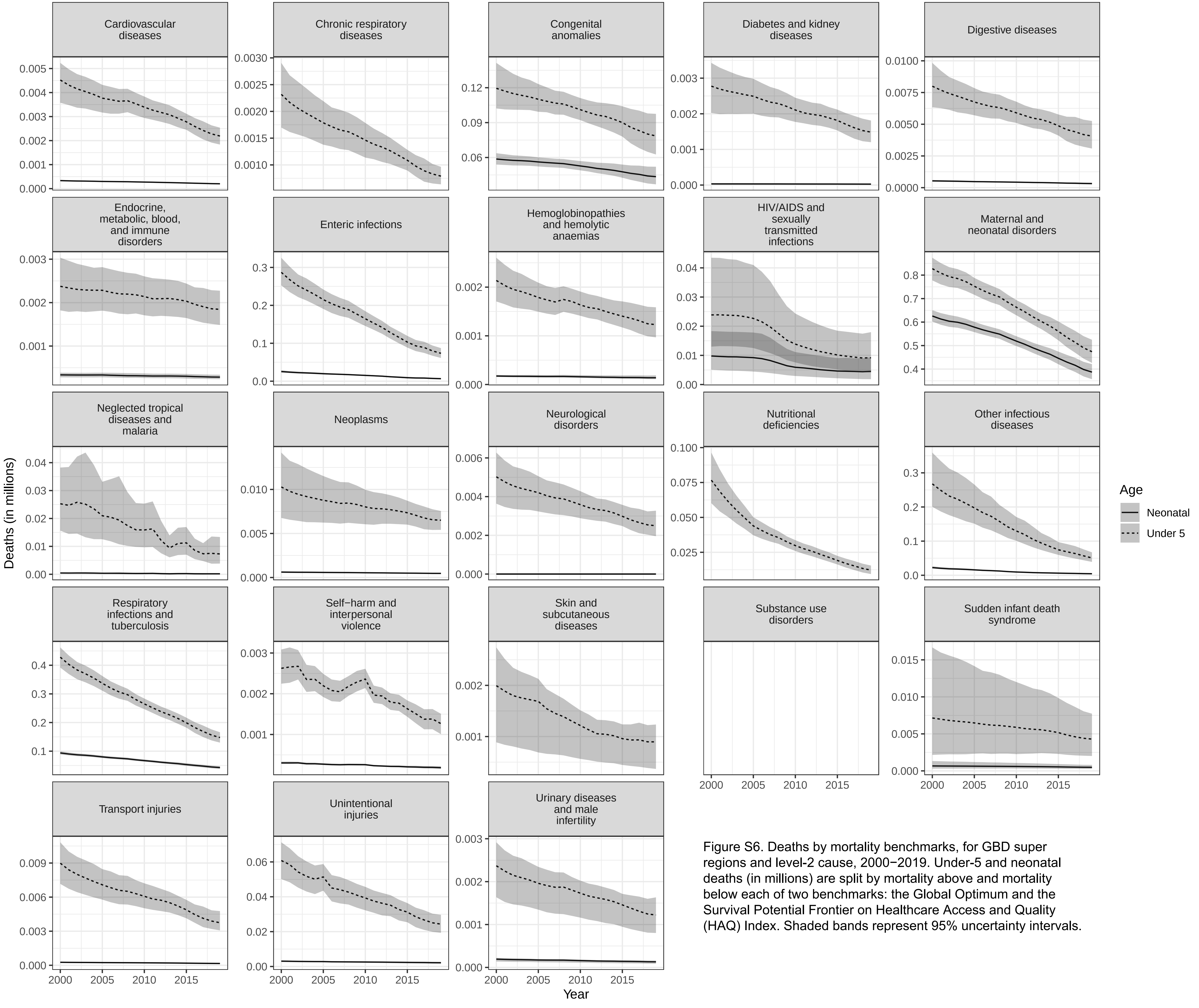

Figure S6. Deaths by mortality benchmarks, for GBD super regions and level-2 cause, 2000−2019. Under-5 and neonatal deaths (in millions) are split by mortality above and mortality below each of two benchmarks: the Global Optimum and the Survival Potential Frontier on Healthcare Access and Quality (HAQ) Index. Shaded bands represent 95% uncertainty intervals.

Figure S6ay. Above Global Optimum, Sub−Saharan Africa

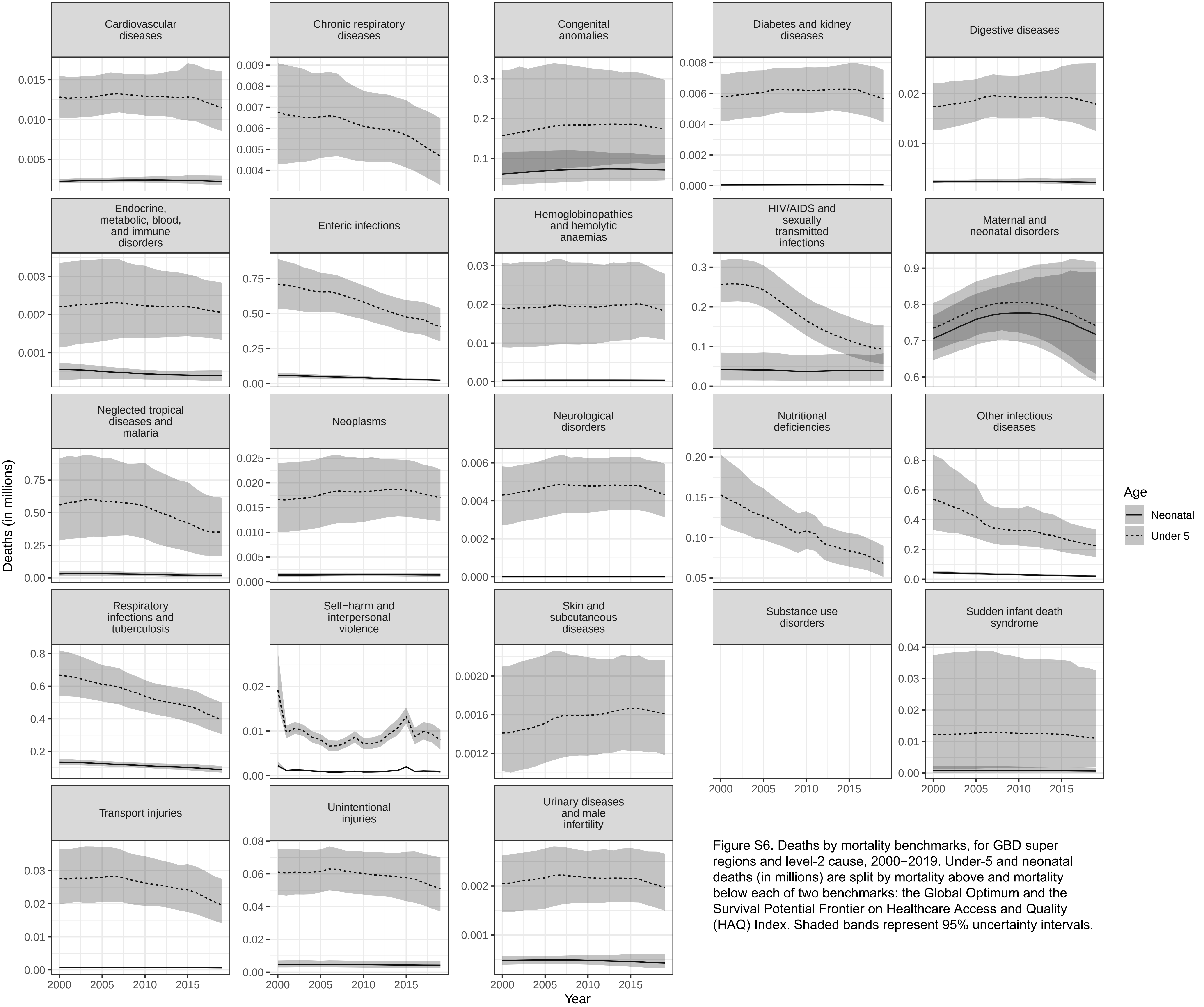

Figure S6. Deaths by mortality benchmarks, for GBD super regions and level-2 cause, 2000−2019. Under-5 and neonatal deaths (in millions) are split by mortality above and mortality below each of two benchmarks: the Global Optimum and the Survival Potential Frontier on Healthcare Access and Quality (HAQ) Index. Shaded bands represent 95% uncertainty intervals.

Figure S6az. Above Survival Potential Frontier, Sub−Saharan Africa

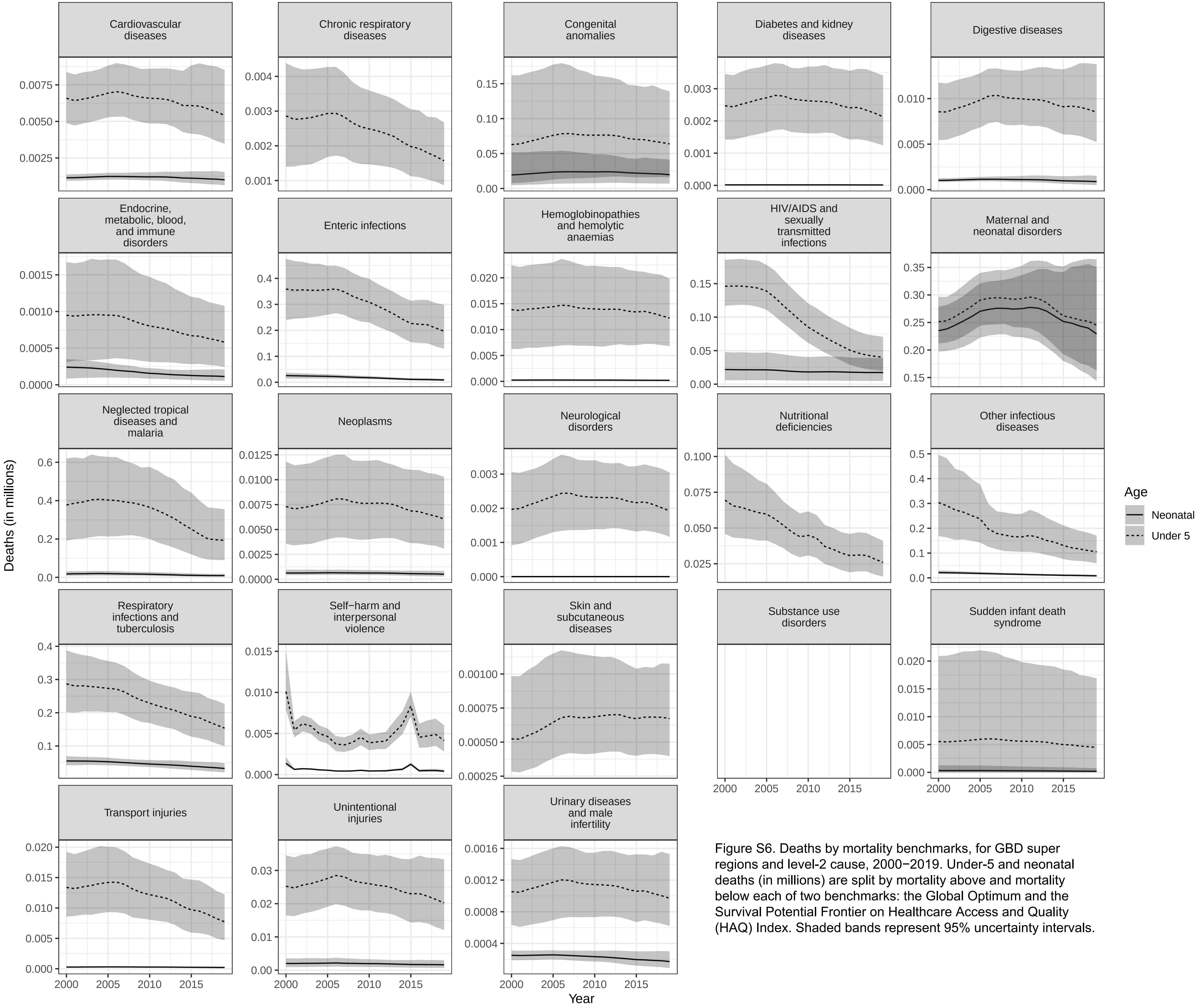

Figure S6. Deaths by mortality benchmarks, for GBD super regions and level-2 cause, 2000−2019. Under-5 and neonatal deaths (in millions) are split by mortality above and mortality below each of two benchmarks: the Global Optimum and the Survival Potential Frontier on Healthcare Access and Quality (HAQ) Index. Shaded bands represent 95% uncertainty intervals.

Figure S6ba. Below Global Optimum, Sub−Saharan Africa

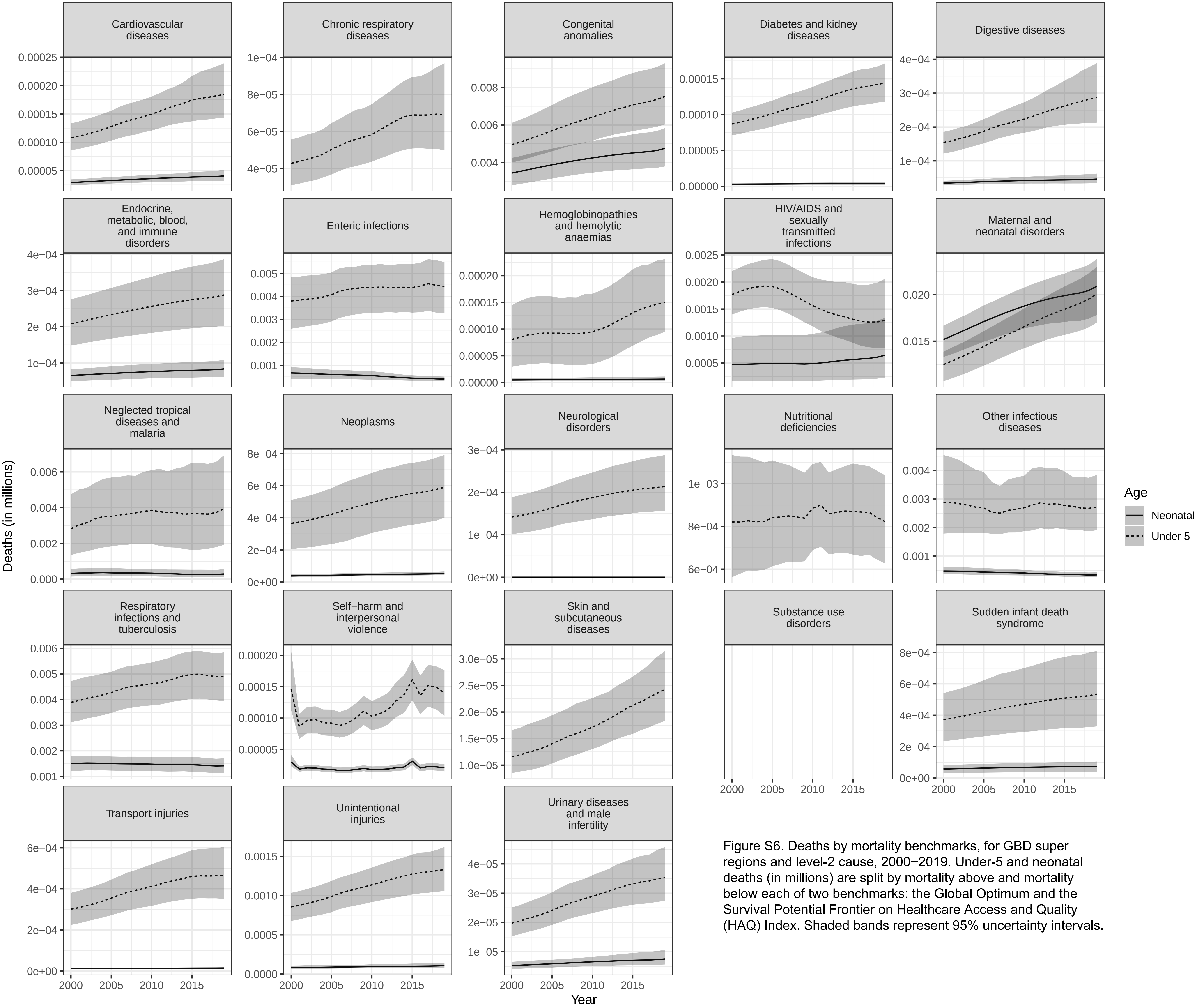

Figure S6. Deaths by mortality benchmarks, for GBD super regions and level-2 cause, 2000−2019. Under-5 and neonatal deaths (in millions) are split by mortality above and mortality below each of two benchmarks: the Global Optimum and the Survival Potential Frontier on Healthcare Access and Quality (HAQ) Index. Shaded bands represent 95% uncertainty intervals.

Figure S6bb. Below Survival Potential Frontier, Sub−Saharan Africa

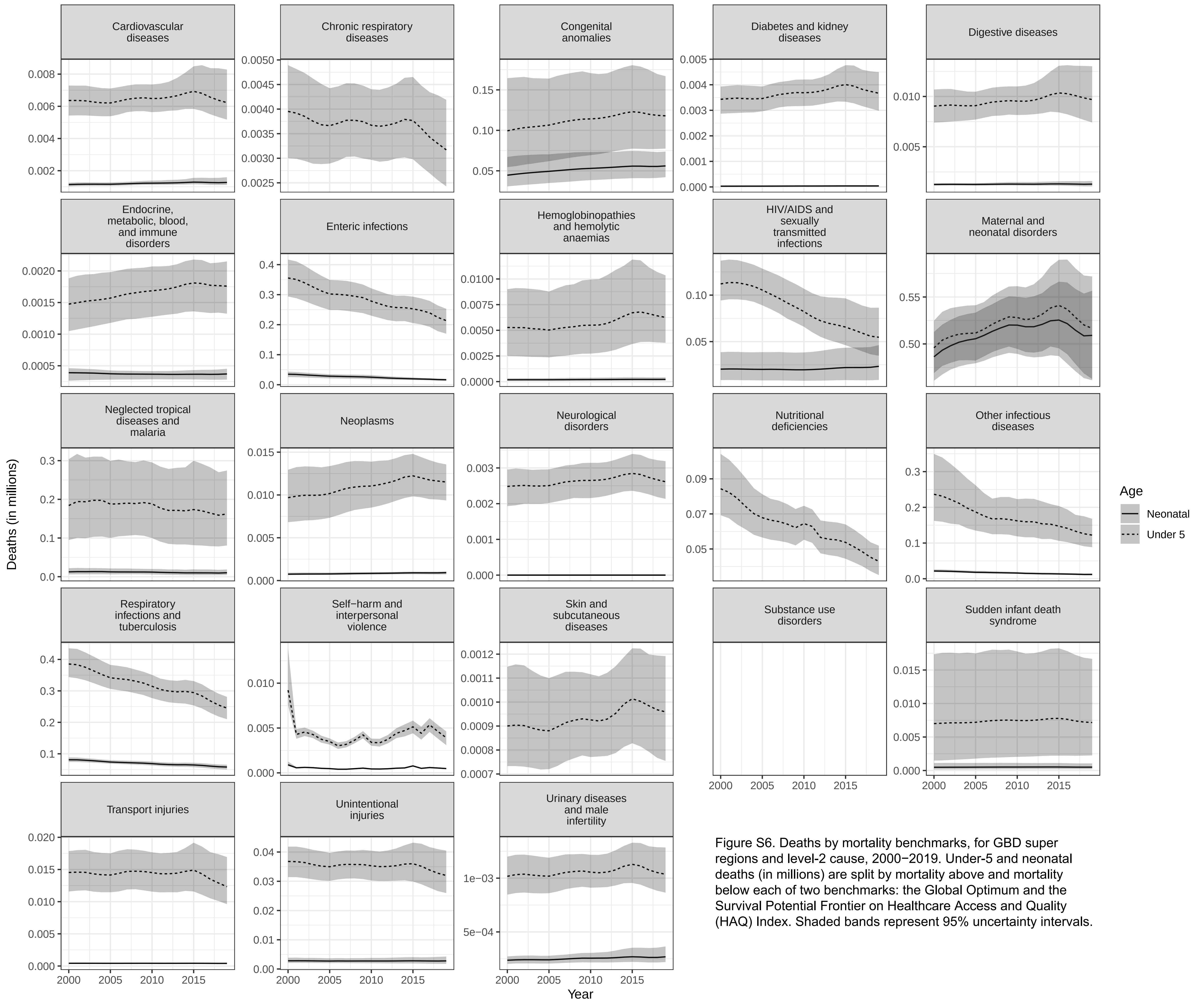

Figure S6. Deaths by mortality benchmarks, for GBD super regions and level-2 cause, 2000−2019. Under-5 and neonatal deaths (in millions) are split by mortality above and mortality below each of two benchmarks: the Global Optimum and the Survival Potential Frontier on Healthcare Access and Quality (HAQ) Index. Shaded bands represent 95% uncertainty intervals.

Figure S7a. Global Optimum mortality rate, among neonates, in deaths per 1000 live births

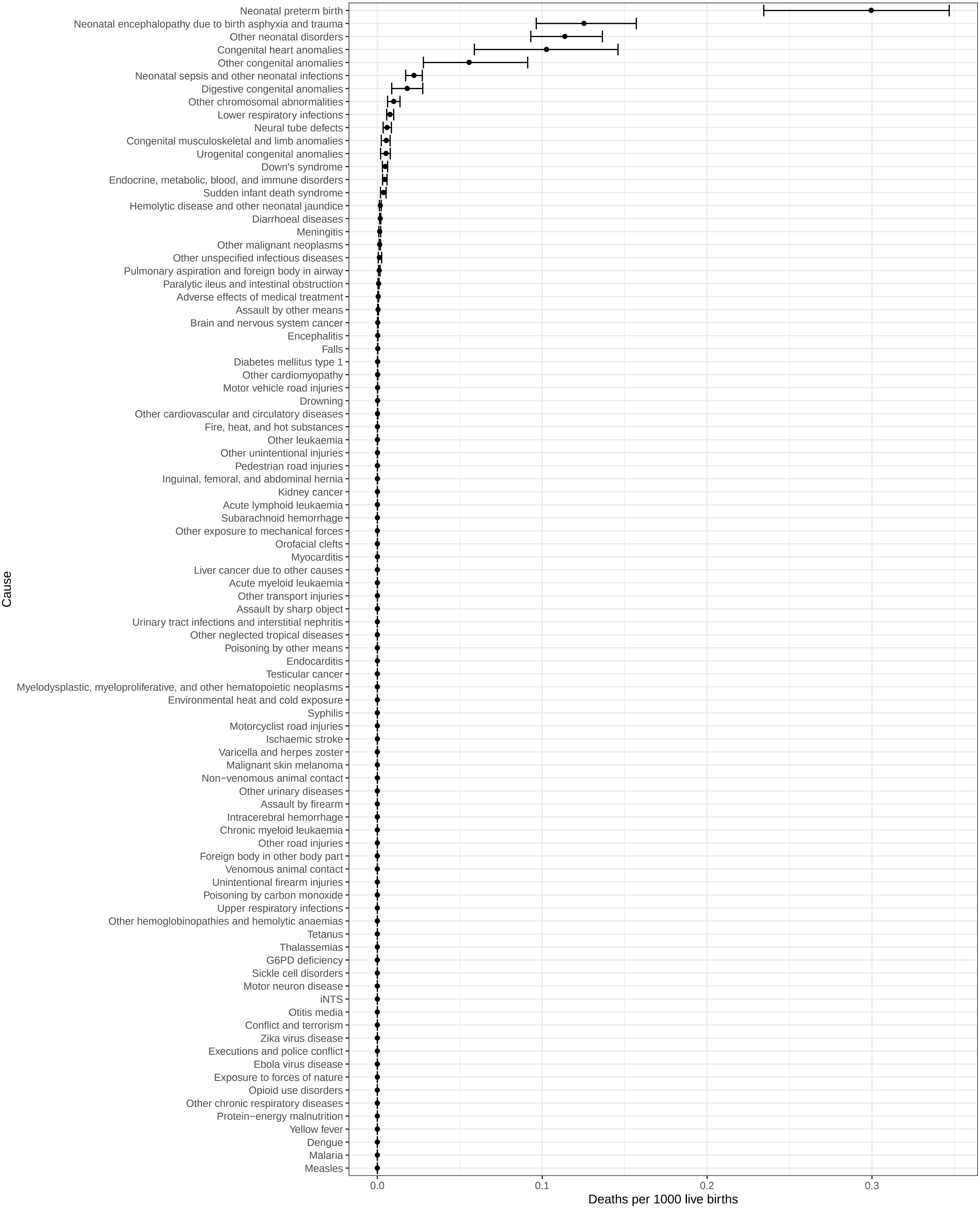

Figure S7b. Global Optimum mortality rate, among all children under 5, in deaths per 1000 live births

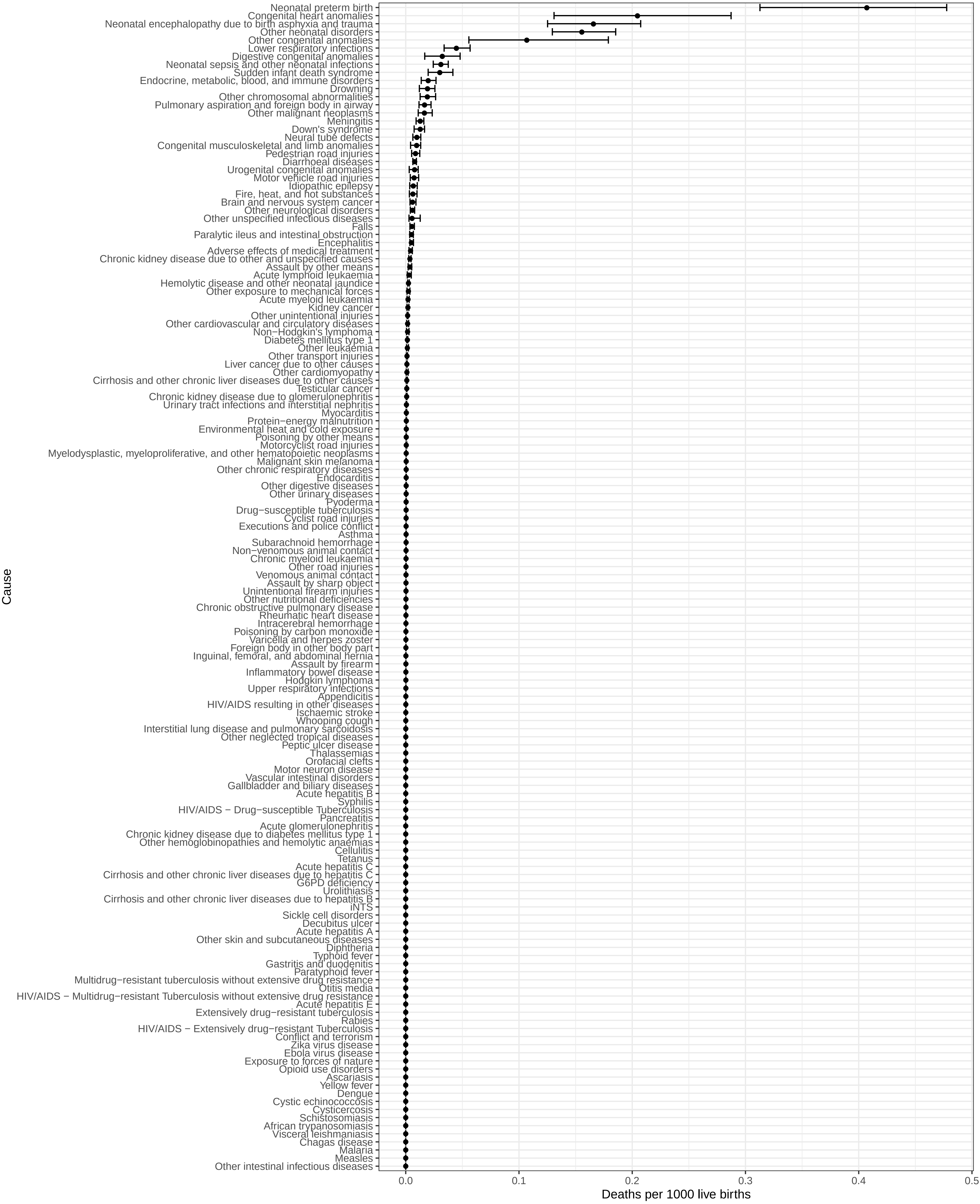

Figure S7c. The Survival Potential Frontier on Healthcare Access and Quality (HAQ) Index for 2019, in the neonatal age group, in deaths per 1000 live births.

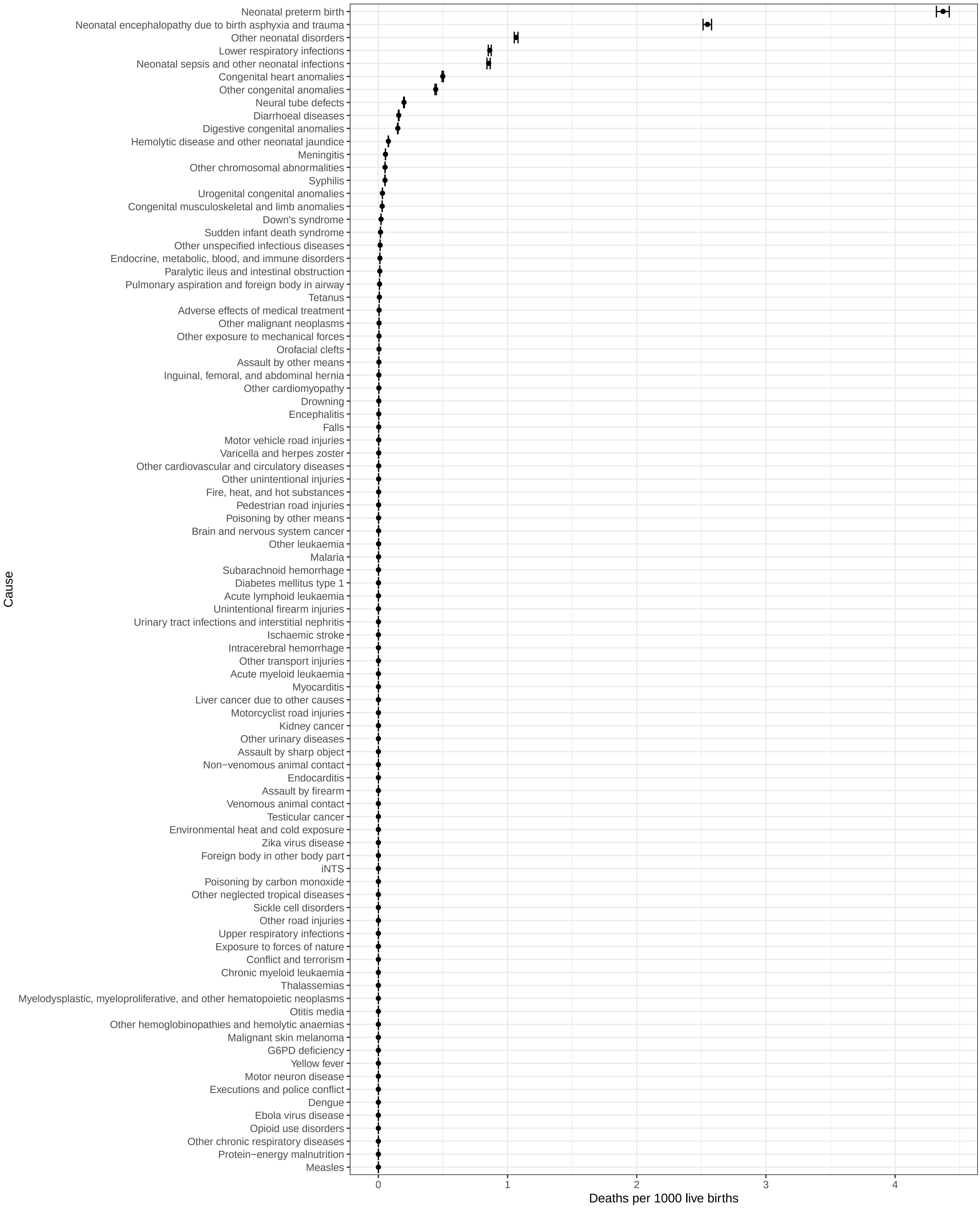

Figure S7d. The Survival Potential Frontier on Healthcare Access and Quality (HAQ) Index for 2019, in the complete under-5 age group, in deaths per 1000 live births.

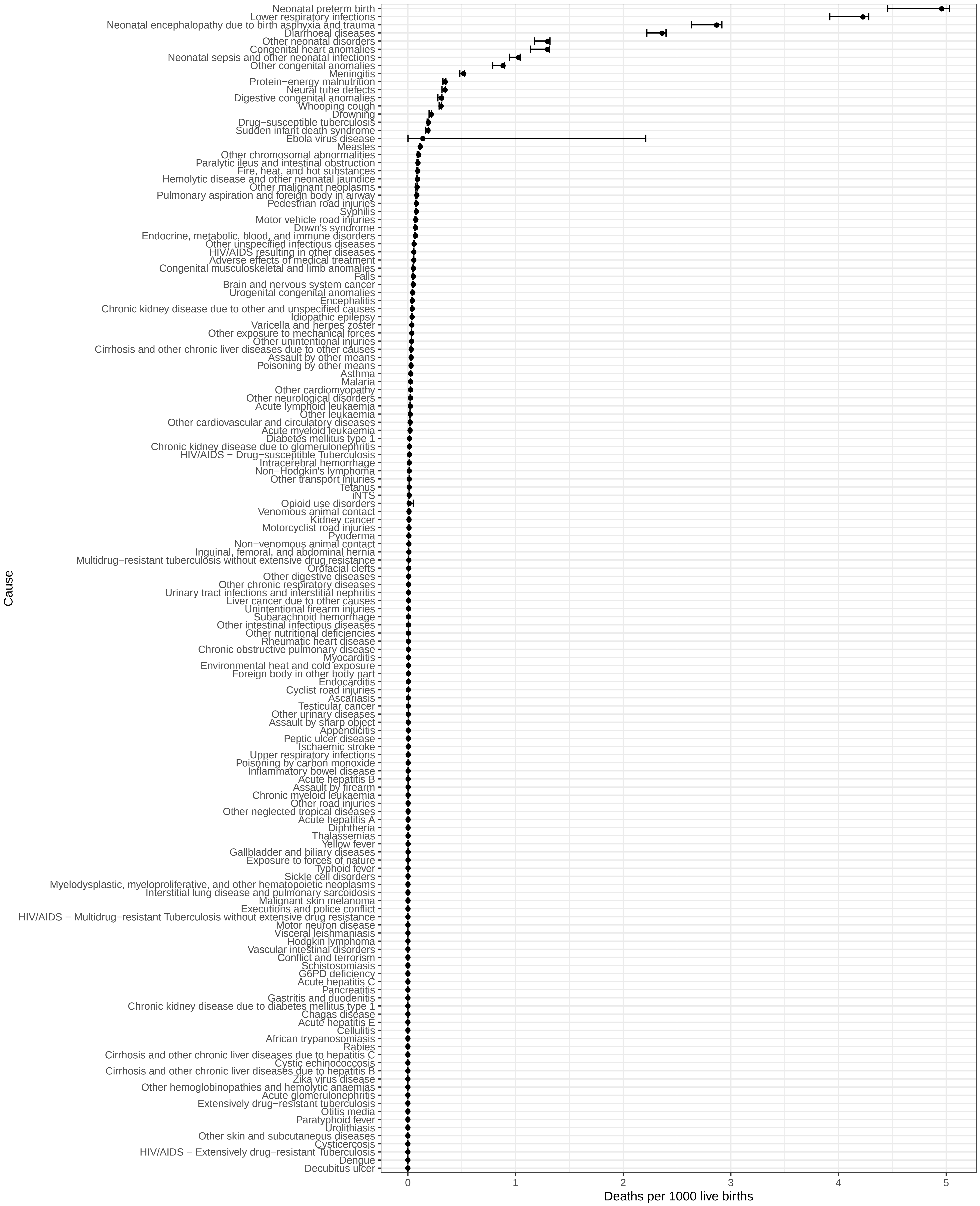

Figure S7e. Mortality above both the Global Optimum benchmark and the Survival Potential Frontier on Healthcare Access and Quality (HAQ) index, in 2000 and 2019, for neonates, in deaths per 1000 live births.

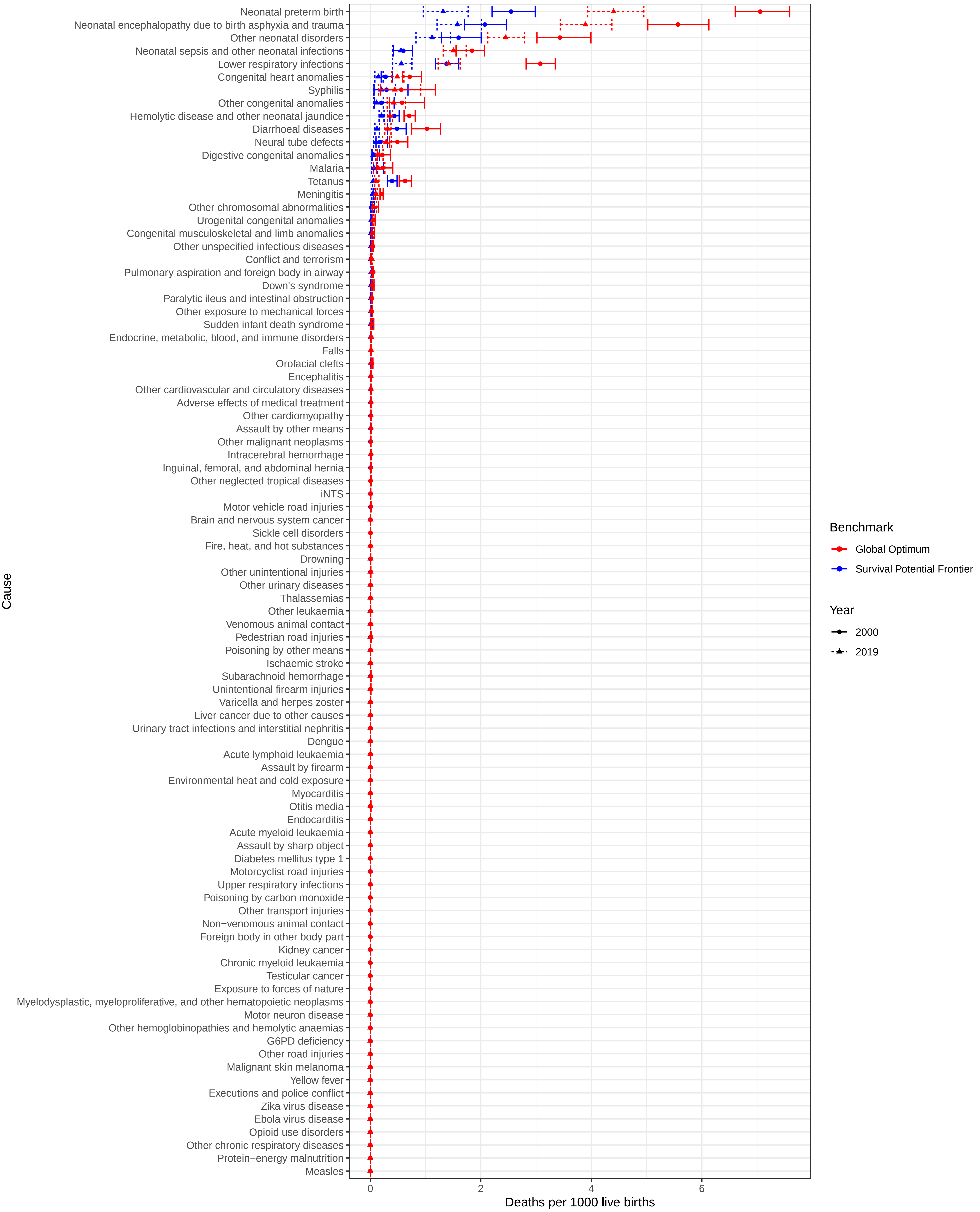

Figure S7f. Mortality above both the Global Optimum benchmark and the Survival Potential Frontier on Healthcare Access and Quality (HAQ) index, in 2000 and 2019, for all children under 5 years, in deaths per 1000 live births.

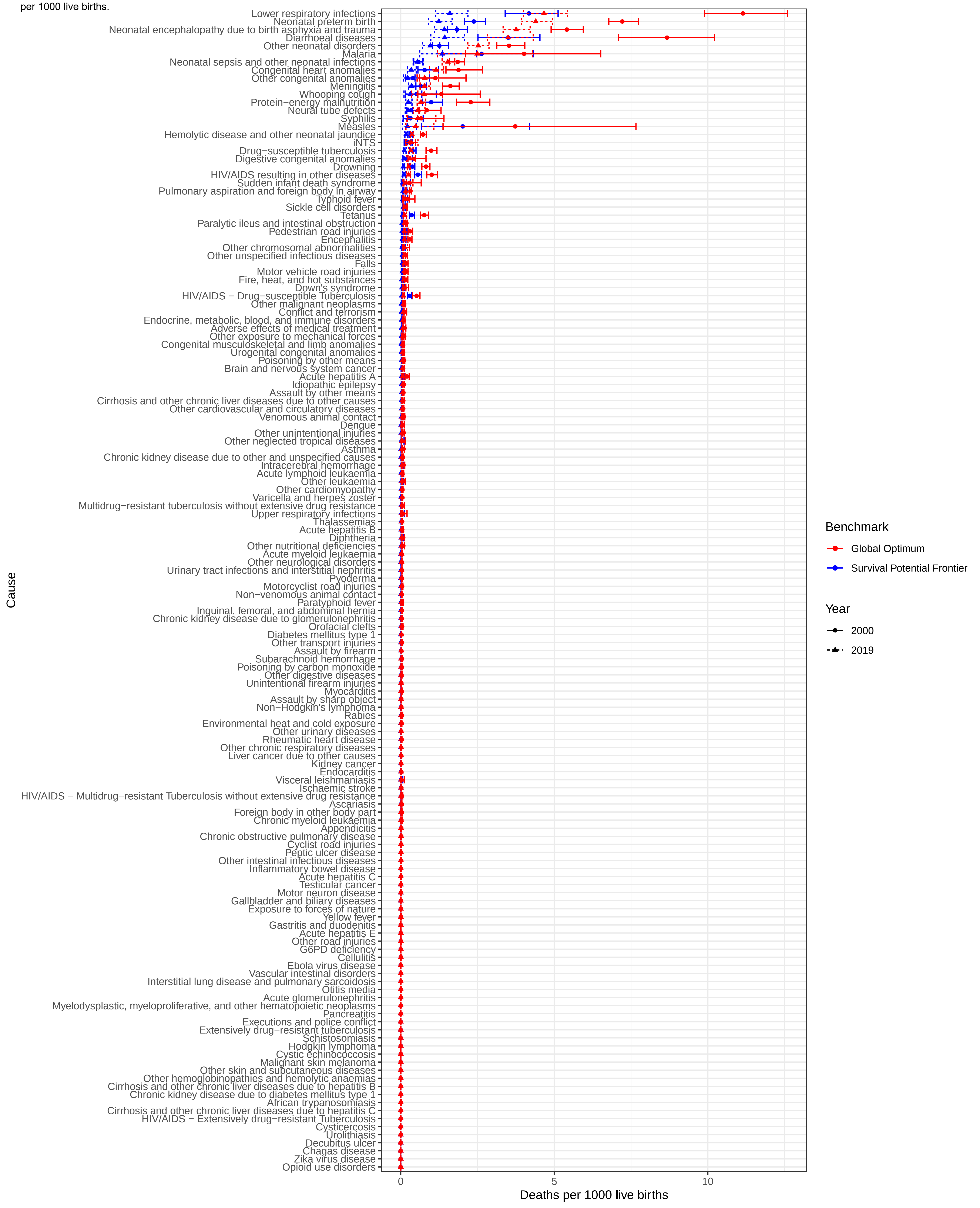

Figure S7g. Proportion of deaths that is above the benchmark, among neonates, by both Global Optimum and Survival Potential Frontier benchmarks, in 2000 and 2019.

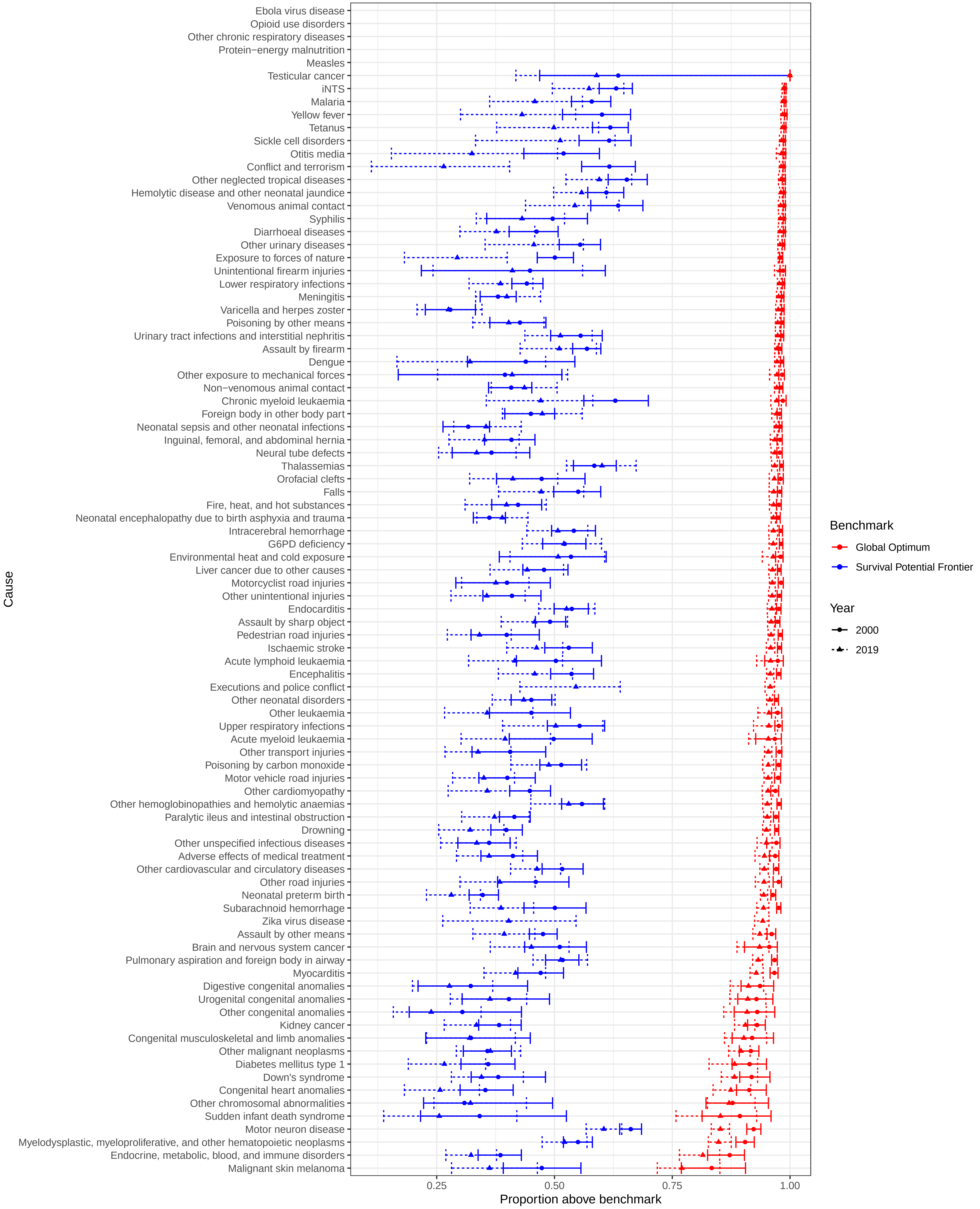

Figure S7h. Proportion of deaths that is above the benchmark, among all children under 5 years, by both Global Optimum and Survival Potential Frontier benchmarks, in 2000 and 2019.

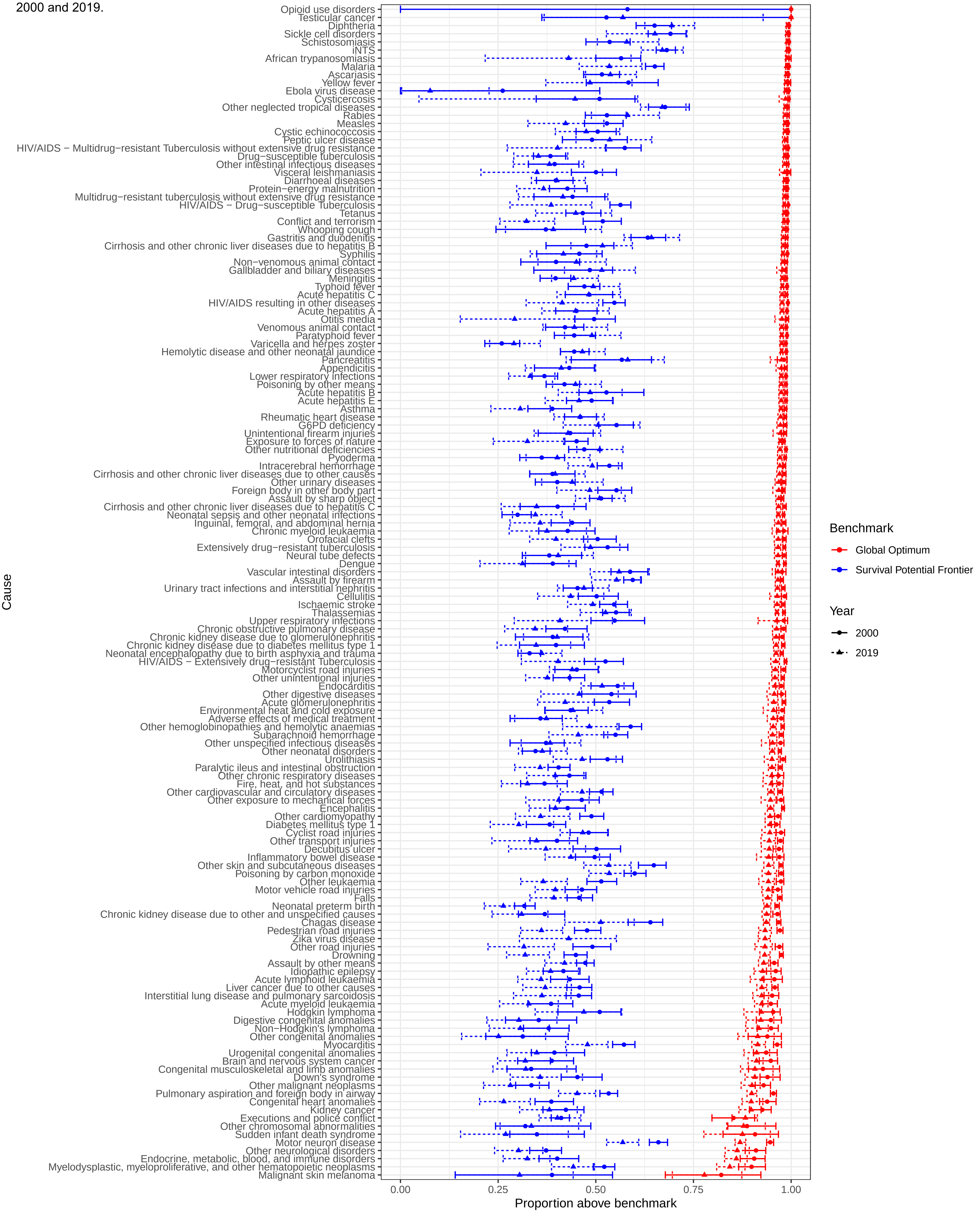

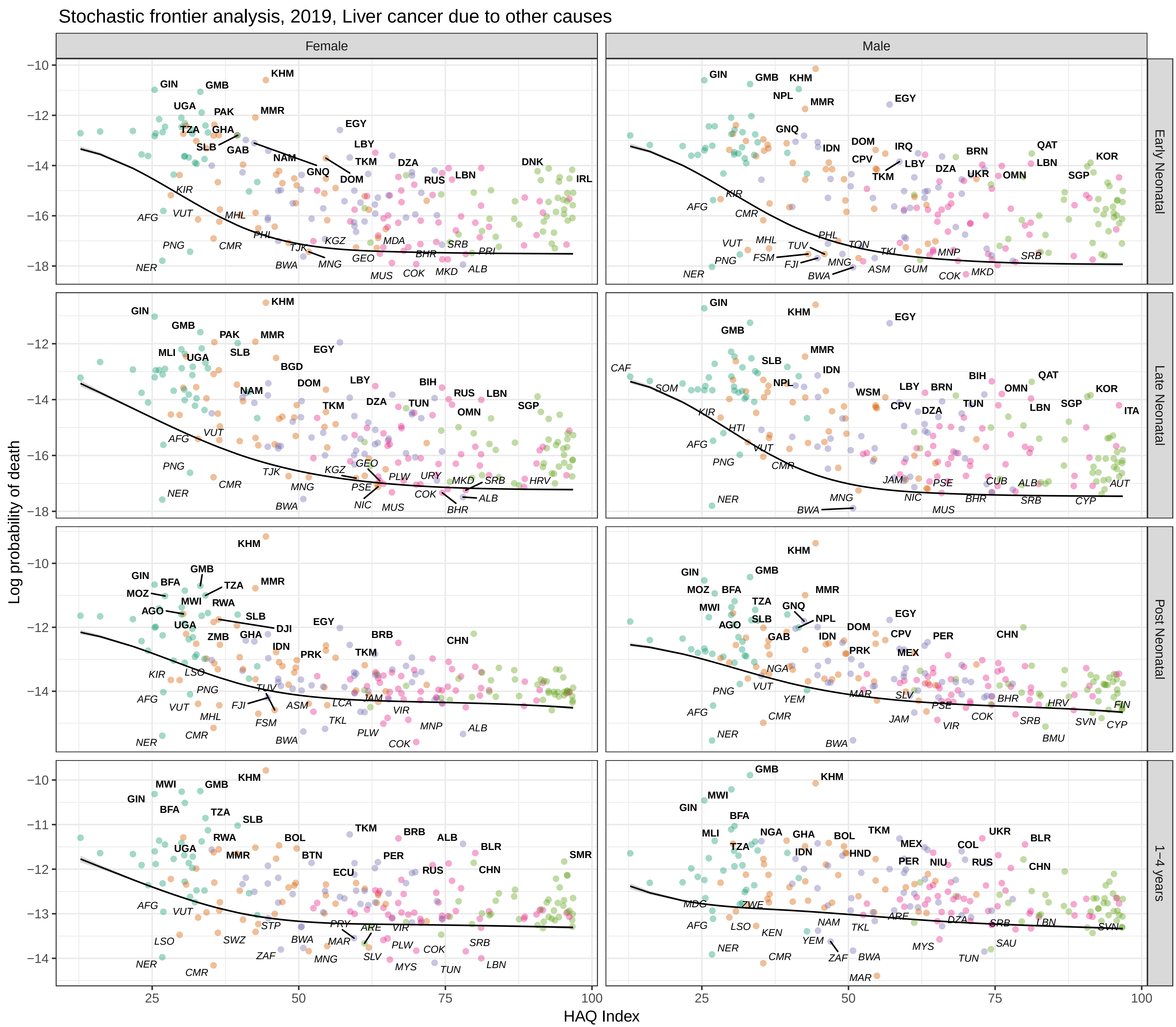

Figure S8
Cause- and age-specific mortality by country and Healthcare Access and Quality (HAQ) Index, with the Survival Potential Frontier (SPF) (black line) and uncertainty interval around the frontier (grey band). Countries are labelled in bold when their ratio to the frontier is in the top 10 percent (performing poorly relative to HAQ Index) and in italics when their ratio to the frontier is in the bottom 10 percent (performing well relative to HAQ Index). These results show the prediction of the cause-specific SPF prior to scaling to the all-cause frontier.

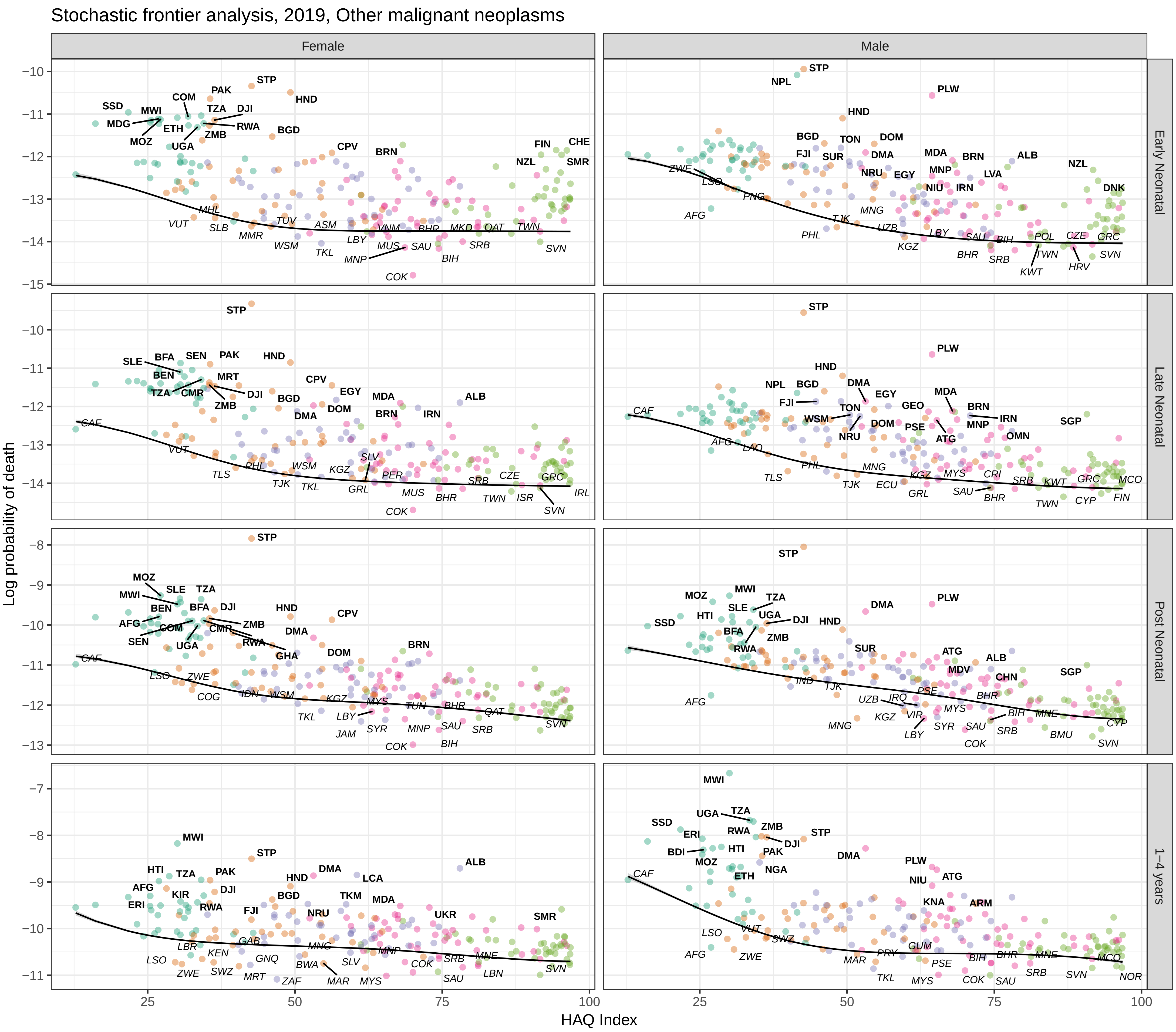

Figure S8
Cause- and age-specific mortality by country and Healthcare Access and Quality (HAQ) Index, with the Survival Potential Frontier (SPF) (black line) and uncertainty interval around the frontier (grey band). Countries are labelled in bold when their ratio to the frontier is in the top 10 percent (performing poorly relative to HAQ Index) and in italics when their ratio to the frontier is in the bottom 10 percent (performing well relative to HAQ Index). These results show the prediction of the cause-specific SPF prior to scaling to the all-cause frontier.

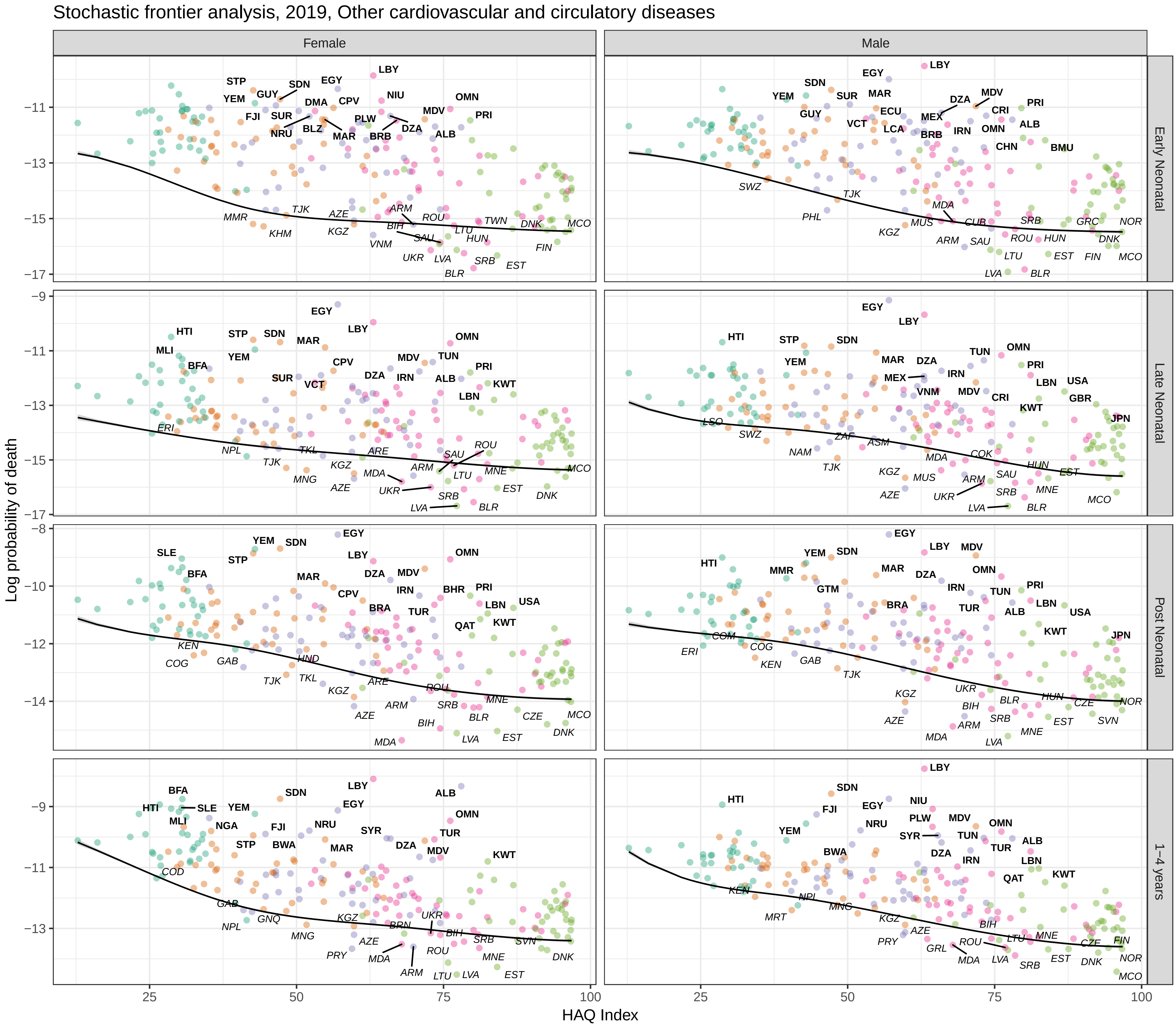

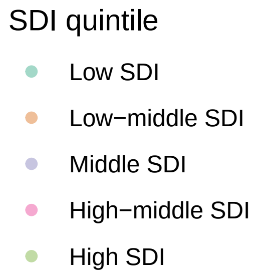

Figure S8
Cause- and age-specific mortality by country and Healthcare Access and Quality (HAQ) Index, with the Survival Potential Frontier (SPF) (black line) and uncertainty interval around the frontier (grey band). Countries are labelled in bold when their ratio to the frontier is in the top 10 percent (performing poorly relative to HAQ Index) and in italics when their ratio to the frontier is in the bottom 10 percent (performing well relative to HAQ Index). These results show the prediction of the cause-specific SPF prior to scaling to the all-cause frontier.

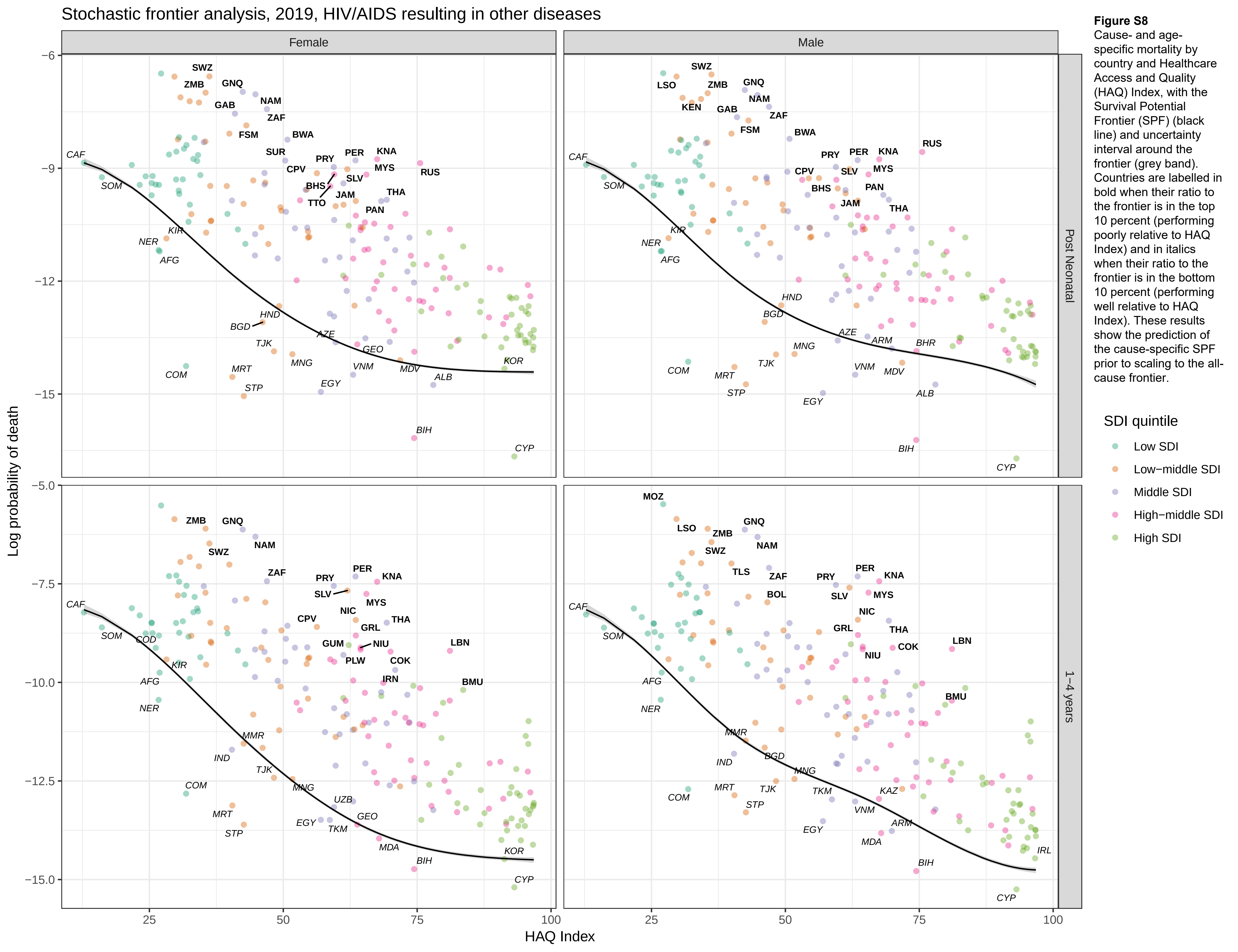

Figure S8
Cause- and age-specific mortality by country and Healthcare Access and Quality (HAQ) Index, with the Survival Potential Frontier (SPF) (black line) and uncertainty interval around the frontier (grey band). Countries are labelled in bold when their ratio to the frontier is in the top 10 percent (performing poorly relative to HAQ Index) and in italics when their ratio to the frontier is in the bottom 10 percent (performing well relative to HAQ Index). These results show the prediction of the cause-specific SPF prior to scaling to the all-cause frontier.

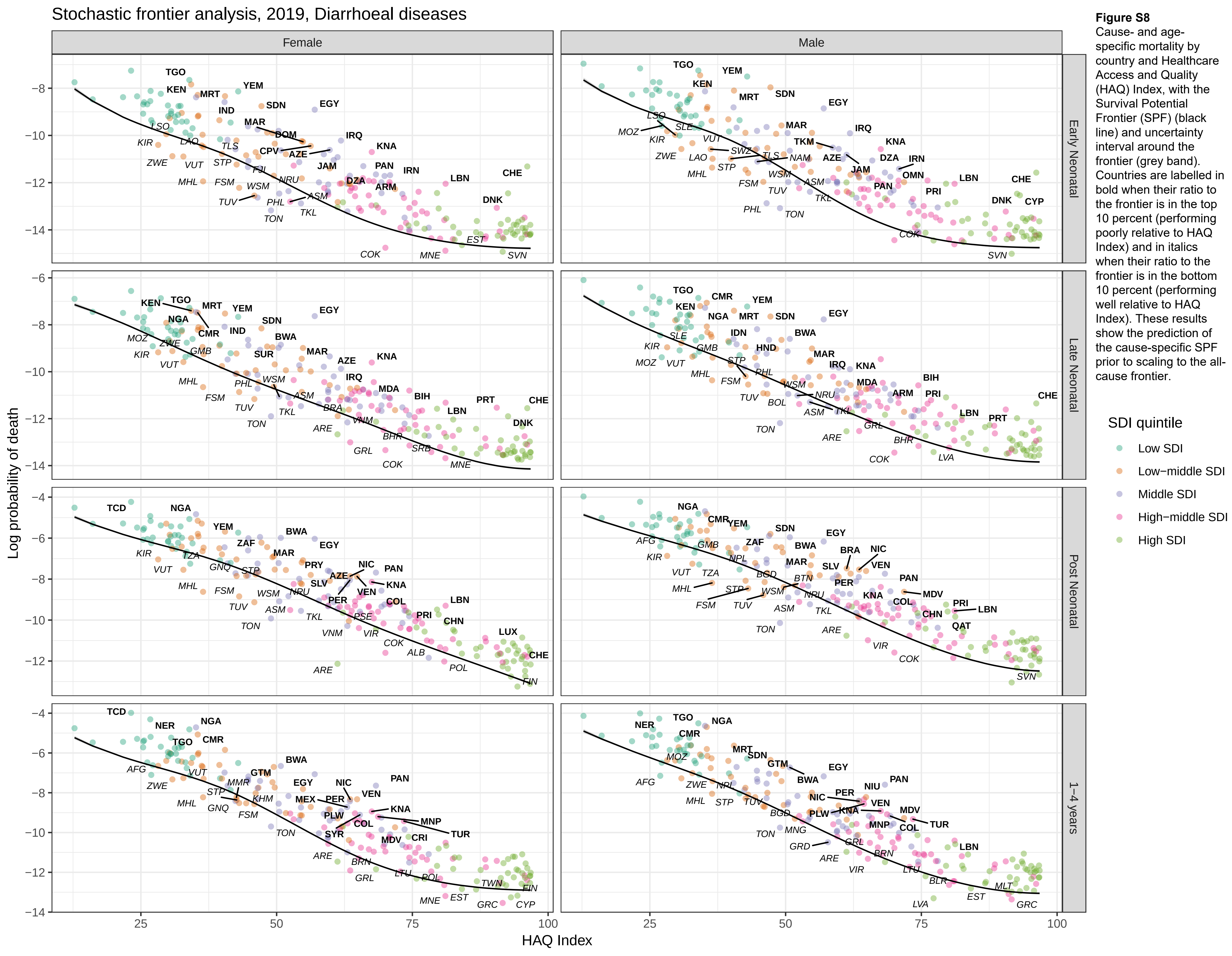

Figure S8
Cause- and age-specific mortality by country and Healthcare Access and Quality (HAQ) Index, with the Survival Potential Frontier (SPF) (black line) and uncertainty interval around the frontier (grey band). Countries are labelled in bold when their ratio to the frontier is in the top 10 percent (performing poorly relative to HAQ Index) and in italics when their ratio to the frontier is in the bottom 10 percent (performing well relative to HAQ Index). These results show the prediction of the cause-specific SPF prior to scaling to the all-cause frontier.

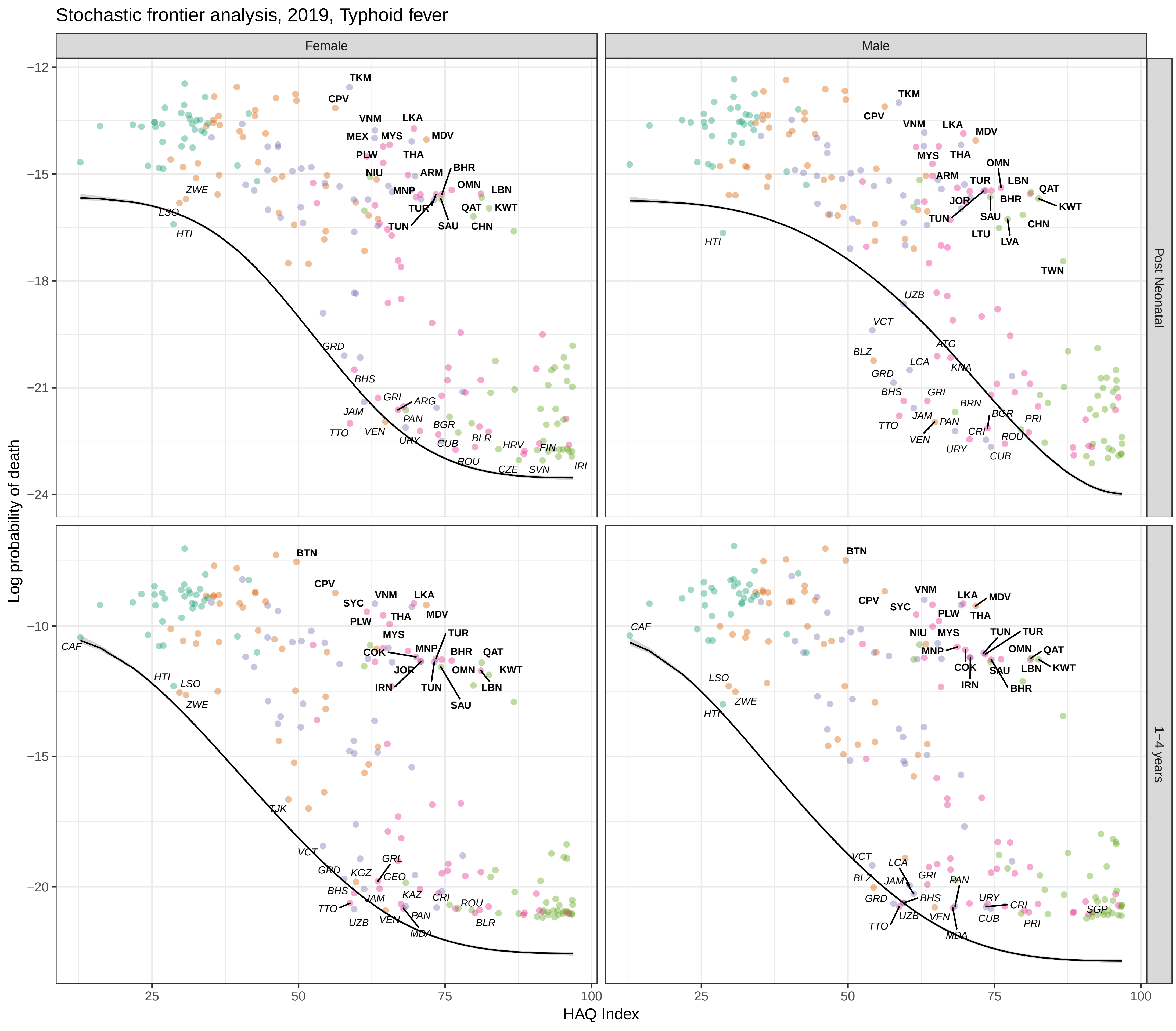

Figure S8
Cause- and age-specific mortality by country and Healthcar[illegible] Access and Quality (HAQ) Index, with the Survival Potential Frontier (SPF) (black line) and uncertainty interval around the frontier (grey band). Countries are labelled bold when their ratio to[illegible] the frontier is in the top 10 percent (performing poorly relative to HAQ Index) and in italics when their ratio to the frontier is in the bottom 10 percent (performing well relative to HAQ Index). These results show the prediction of the cause-specific SPF prior to scaling to the a[illegible] cause frontier.

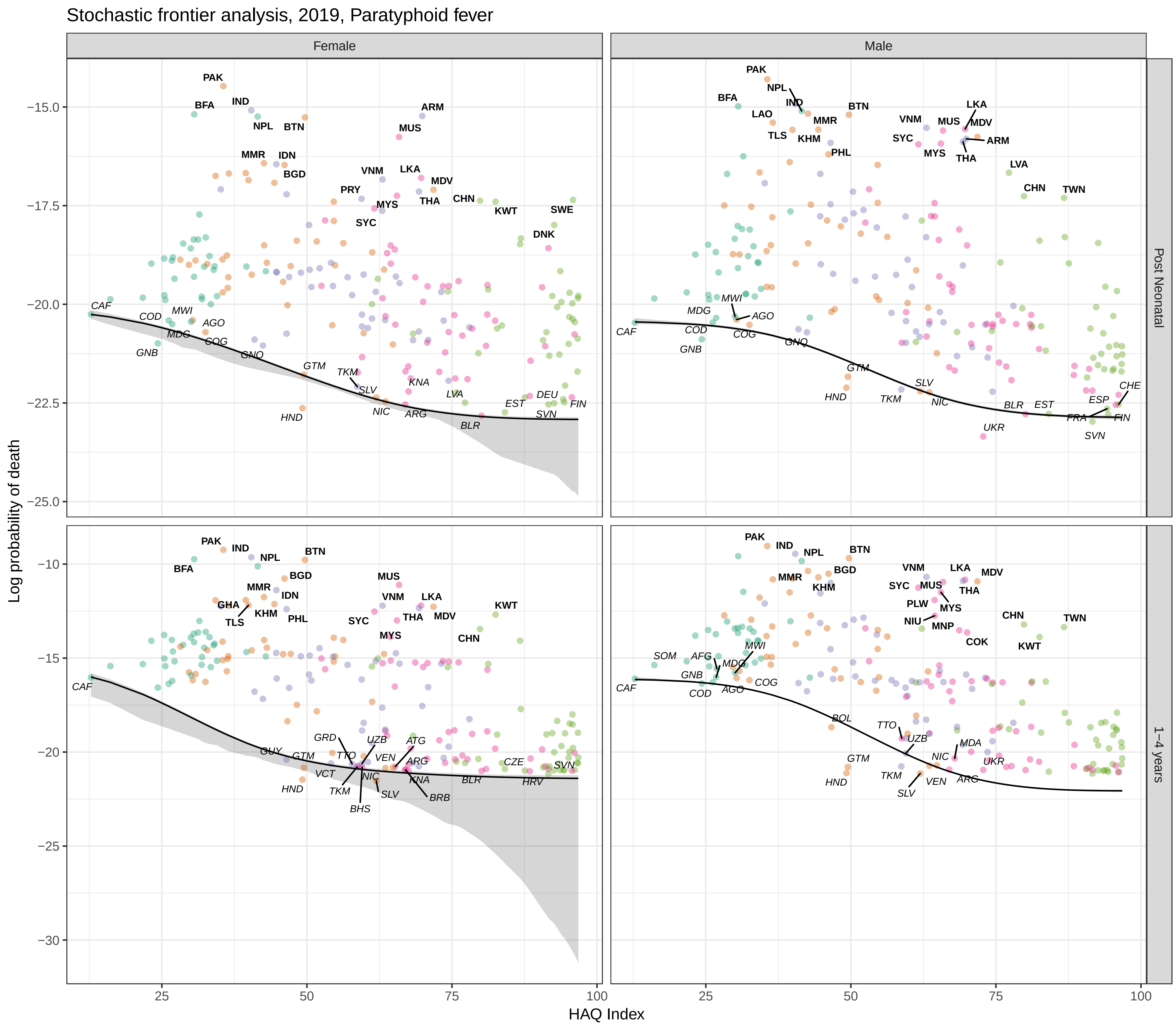

Figure S8
Cause- and age-specific mortality by country and Healthcare Access and Quality (HAQ) Index, with the Survival Potential Frontier (SPF) (black line) and uncertainty interval around the frontier (grey band). Countries are labelled in bold when their ratio to the frontier is in the top 10 percent (performing poorly relative to HAQ Index) and in italics when their ratio to the frontier is in the bottom 10 percent (performing well relative to HAQ Index). These results show the prediction of the cause-specific SPF prior to scaling to the all-cause frontier.

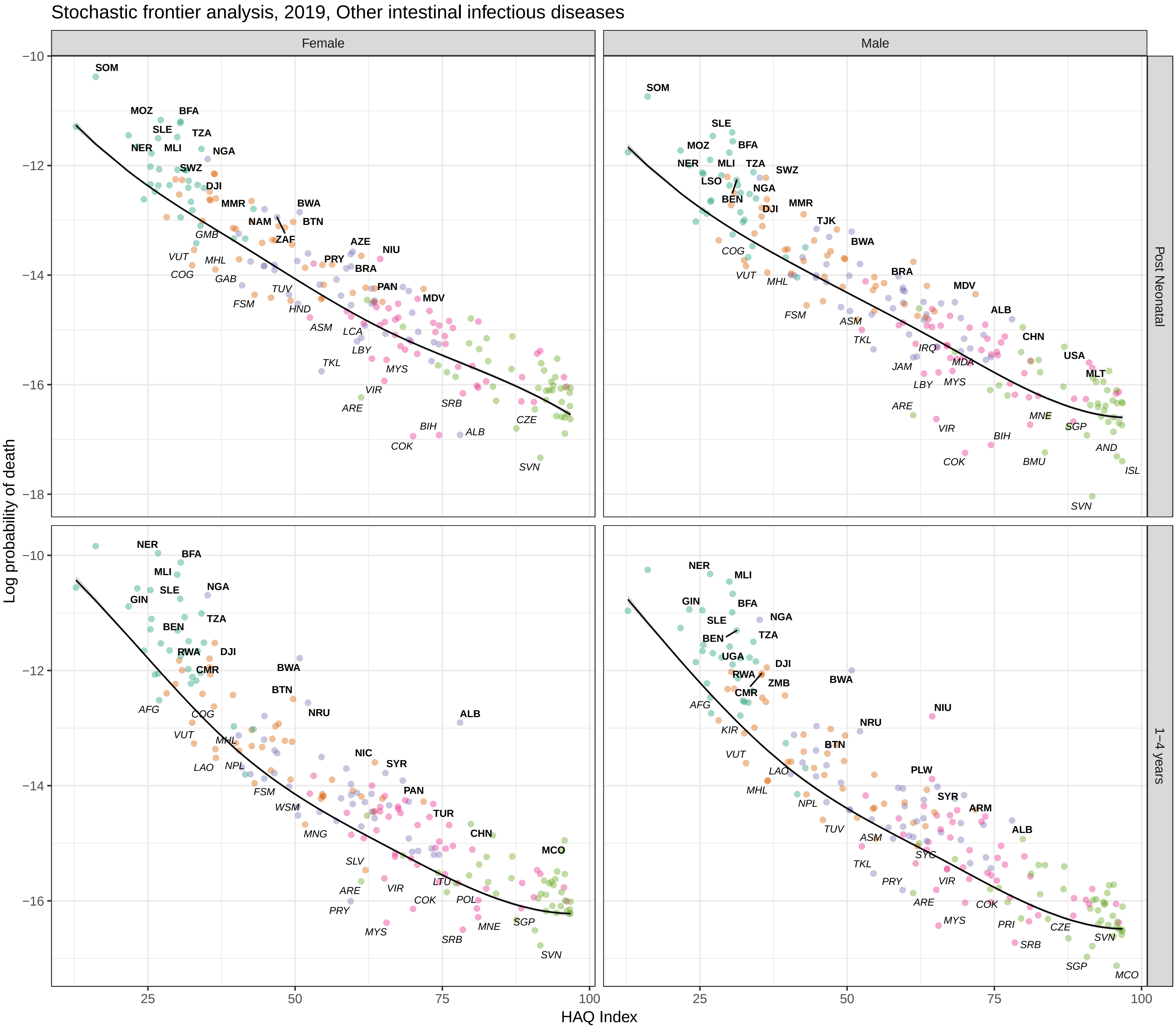

Figure S8
Cause- and age-specific mortality by country and Healthcare Access and Quality (HAQ) Index, with the Survival Potential Frontier (SPF) (black line) and uncertainty interval around the frontier (grey band). Countries are labelled in bold when their ratio to the frontier is in the top 10 percent (performing poorly relative to HAQ Index) and in italics when their ratio to the frontier is in the bottom 10 percent (performing well relative to HAQ Index). These results show the prediction of the cause-specific SPF prior to scaling to the all-cause frontier.

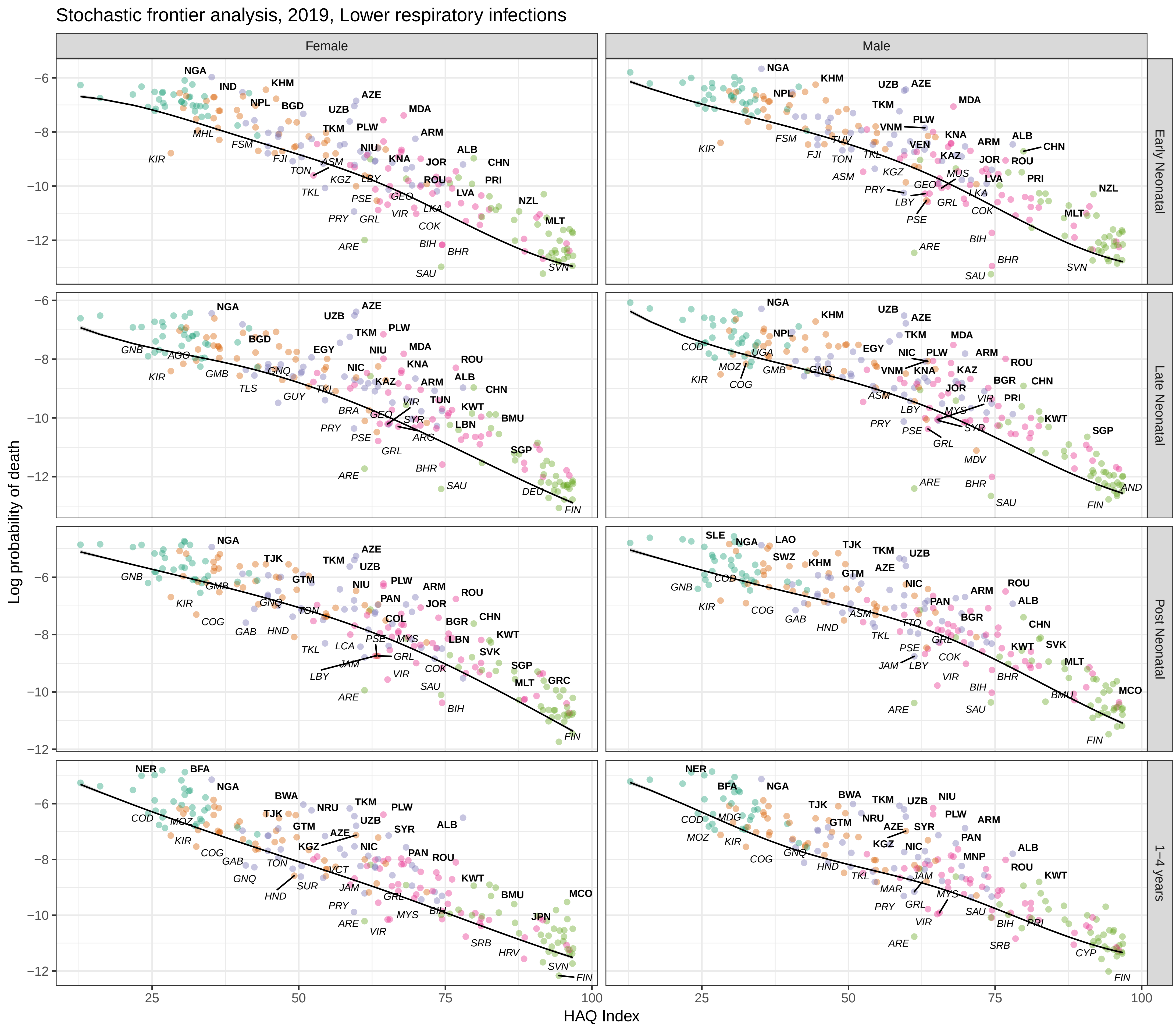

Figure S8
Cause- and age-specific mortality by country and Healthcare Access and Quality (HAQ) Index, with the Survival Potential Frontier (SPF) (black line) and uncertainty interval around the frontier (grey band). Countries are labelled in bold when their ratio to the frontier is in the top 10 percent (performing poorly relative to HAQ Index) and in italics when their ratio to the frontier is in the bottom 10 percent (performing well relative to HAQ Index). These results show the prediction of the cause-specific SPF prior to scaling to the all-cause frontier.

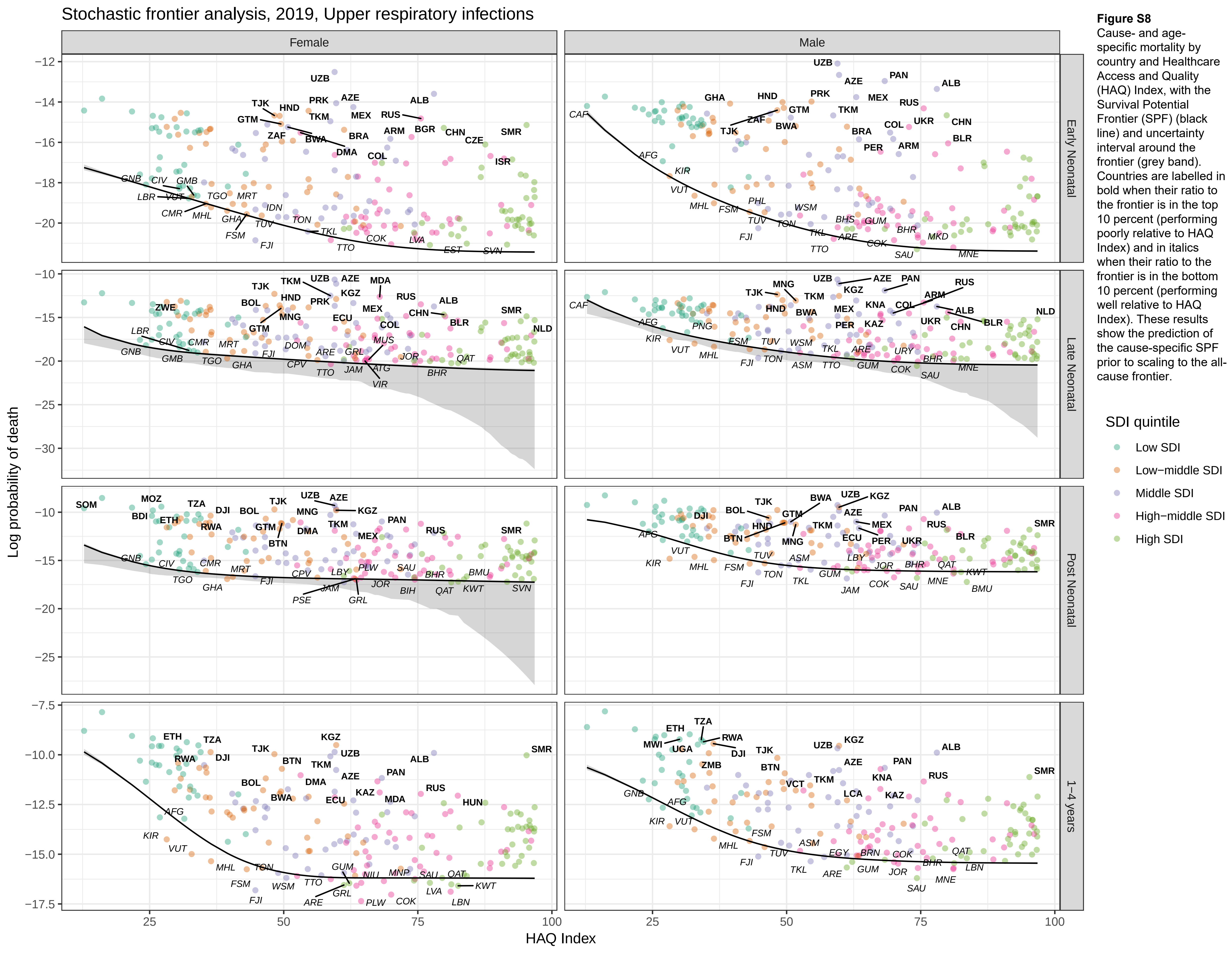

Figure S8
Cause- and age-specific mortality by country and Healthcare Access and Quality (HAQ) Index, with the Survival Potential Frontier (SPF) (black line) and uncertainty interval around the frontier (grey band). Countries are labelled in bold when their ratio to the frontier is in the top 10 percent (performing poorly relative to HAQ Index) and in italics when their ratio to the frontier is in the bottom 10 percent (performing well relative to HAQ Index). These results show the prediction of the cause-specific SPF prior to scaling to the all-cause frontier.

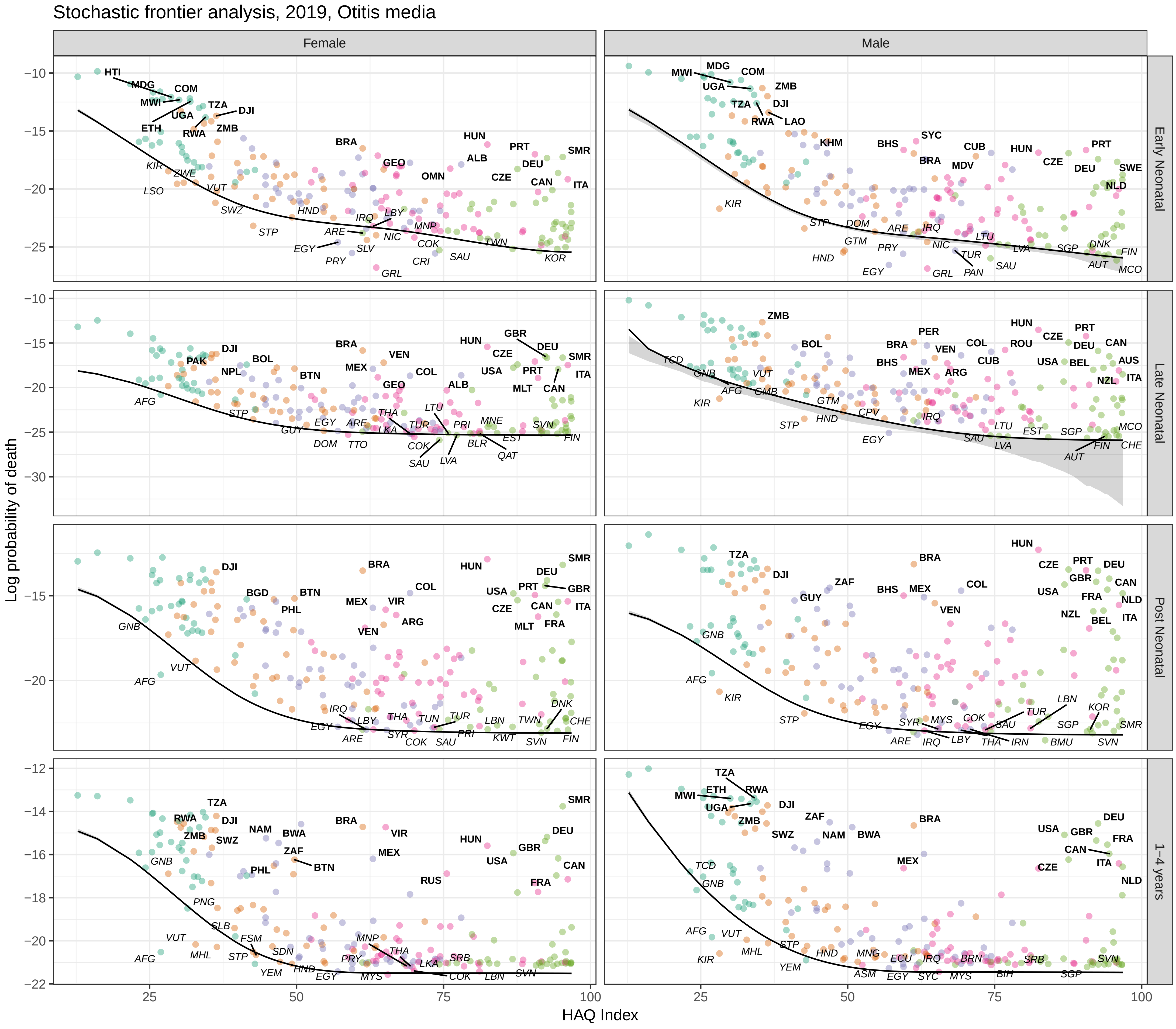

Figure S8
Cause- and age-specific mortality by country and Healthcare Access and Quality (HAQ) Index, with the Survival Potential Frontier (SPF) (black line) and uncertainty interval around the frontier (grey band). Countries are labelled in bold when their ratio to the frontier is in the top 10 percent (performing poorly relative to HAQ Index) and in italics when their ratio to the frontier is in the bottom 10 percent (performing well relative to HAQ Index). These results show the prediction of the cause-specific SPF prior to scaling to the all-cause frontier.

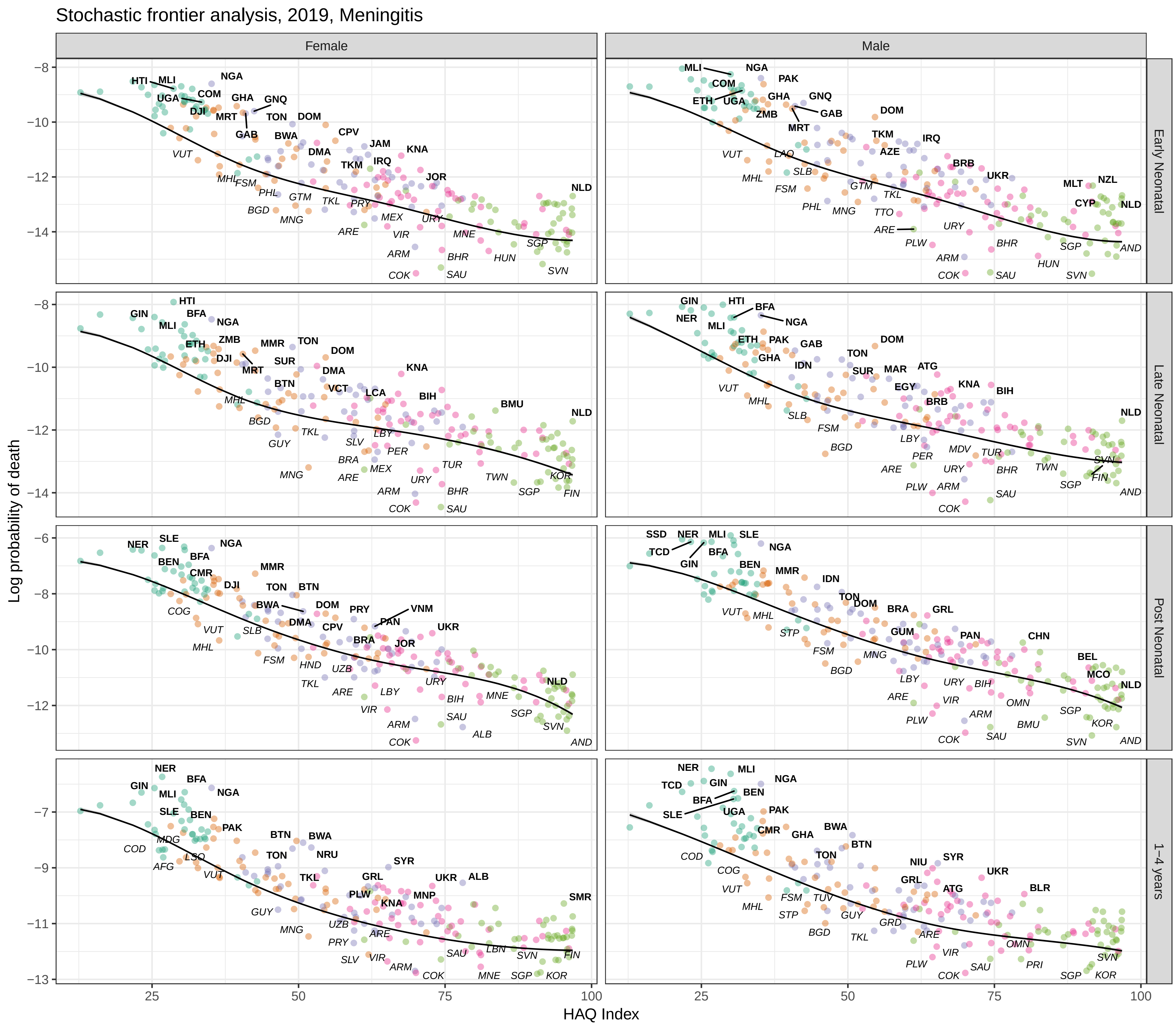

Figure S8
Cause- and age-specific mortality by country and Healthcare Access and Quality (HAQ) Index, with the Survival Potential Frontier (SPF) (black line) and uncertainty interval around the frontier (grey band). Countries are labelled in bold when their ratio to the frontier is in the top 10 percent (performing poorly relative to HAQ Index) and in italics when their ratio to the frontier is in the bottom 10 percent (performing well relative to HAQ Index). These results show the prediction of the cause-specific SPF prior to scaling to the all-cause frontier.

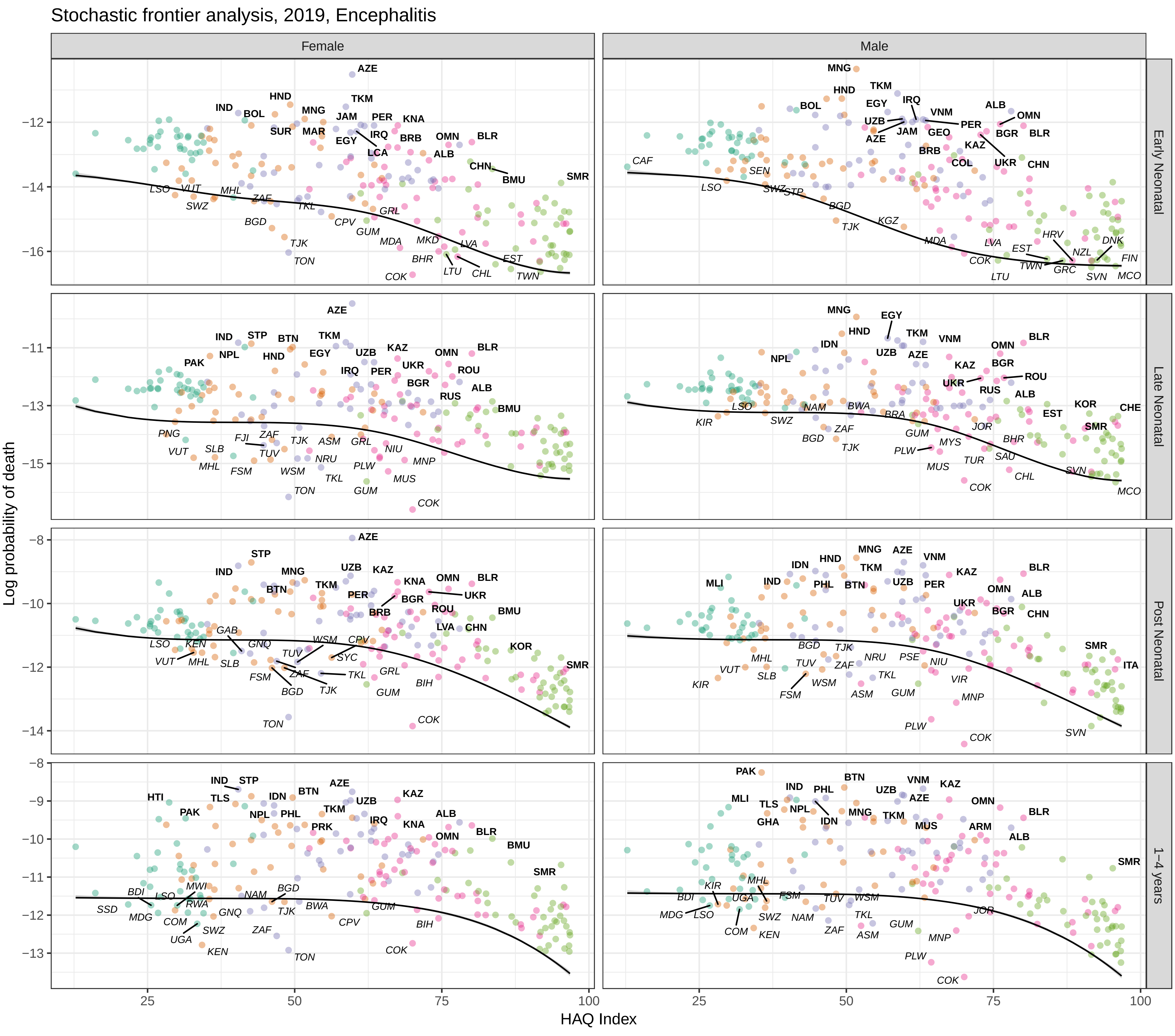

Figure S8
Cause- and age-specific mortality by country and Healthcare Access and Quality (HAQ) Index, with the Survival Potential Frontier (SPF) (black line) and uncertainty interval around the frontier (grey band). Countries are labelled in bold when their ratio to the frontier is in the top 10 percent (performing poorly relative to HAQ Index) and in italics when their ratio to the frontier is in the bottom 10 percent (performing well relative to HAQ Index). These results show the prediction of the cause-specific SPF prior to scaling to the all-cause frontier.

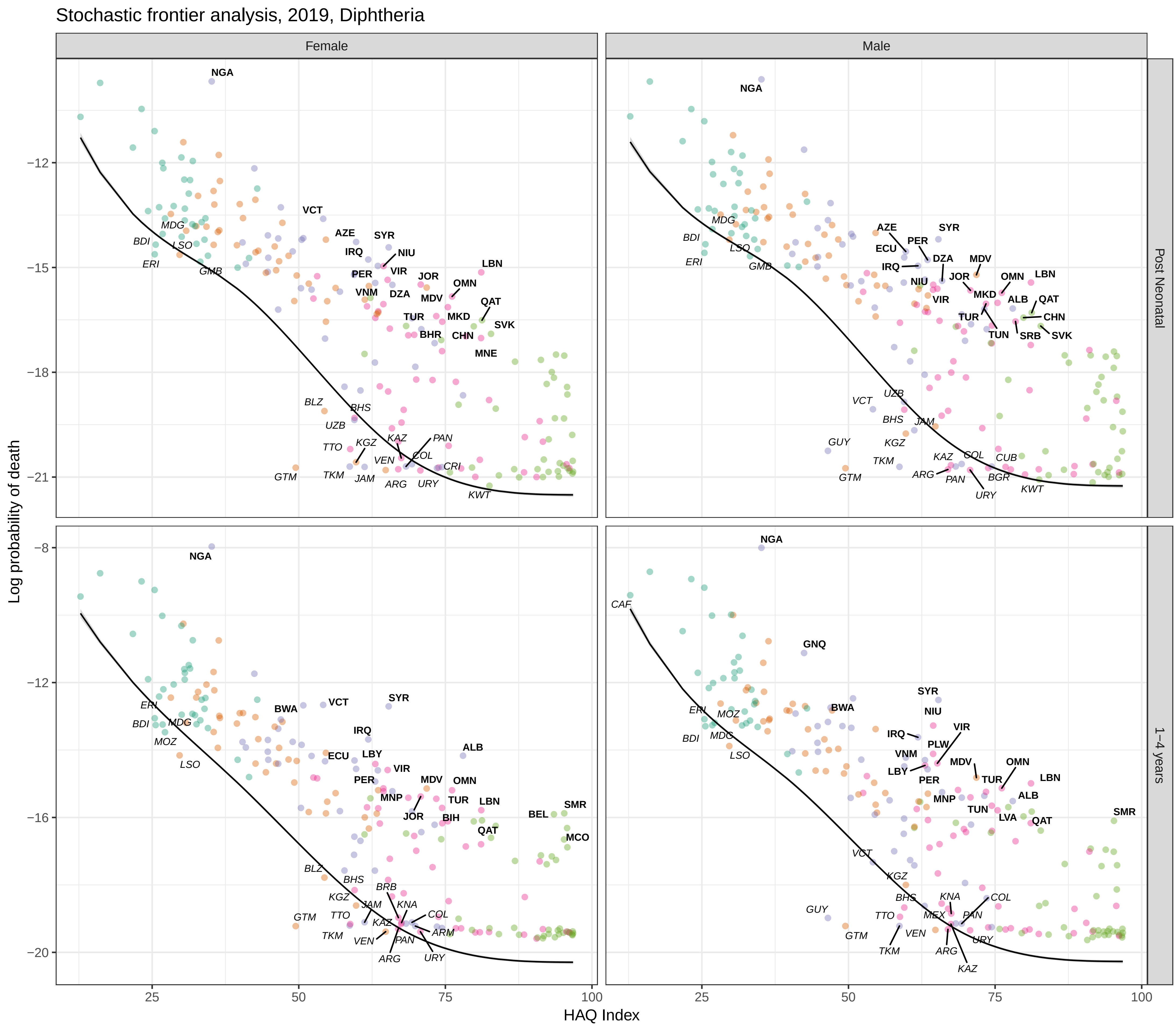

Figure S8
Cause- and age-specific mortality by country and Healthcare Access and Quality (HAQ) Index, with the Survival Potential Frontier (SPF) (black line) and uncertainty interval around the frontier (grey band). Countries are labelled in bold when their ratio to the frontier is in the top 10 percent (performing poorly relative to HAQ Index) and in italics when their ratio to the frontier is in the bottom 10 percent (performing well relative to HAQ Index). These results show the prediction of the cause-specific SPF prior to scaling to the all-cause frontier.

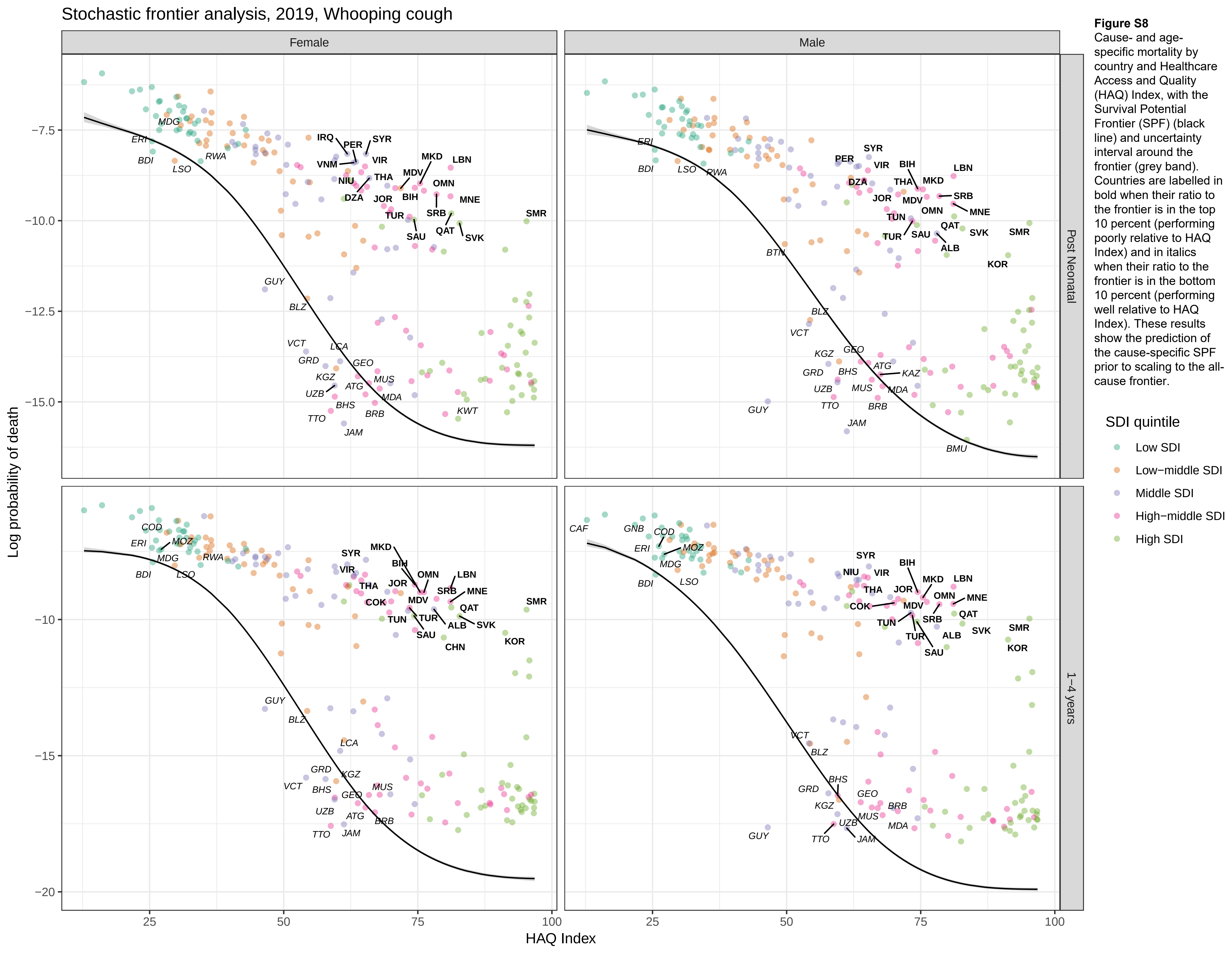

Figure S8
Cause- and age-specific mortality by country and Healthcare Access and Quality (HAQ) Index, with the Survival Potential Frontier (SPF) (black line) and uncertainty interval around the frontier (grey band). Countries are labelled in bold when their ratio to the frontier is in the top 10 percent (performing poorly relative to HAQ Index) and in italics when their ratio to the frontier is in the bottom 10 percent (performing well relative to HAQ Index). These results show the prediction of the cause-specific SPF prior to scaling to the all-cause frontier.

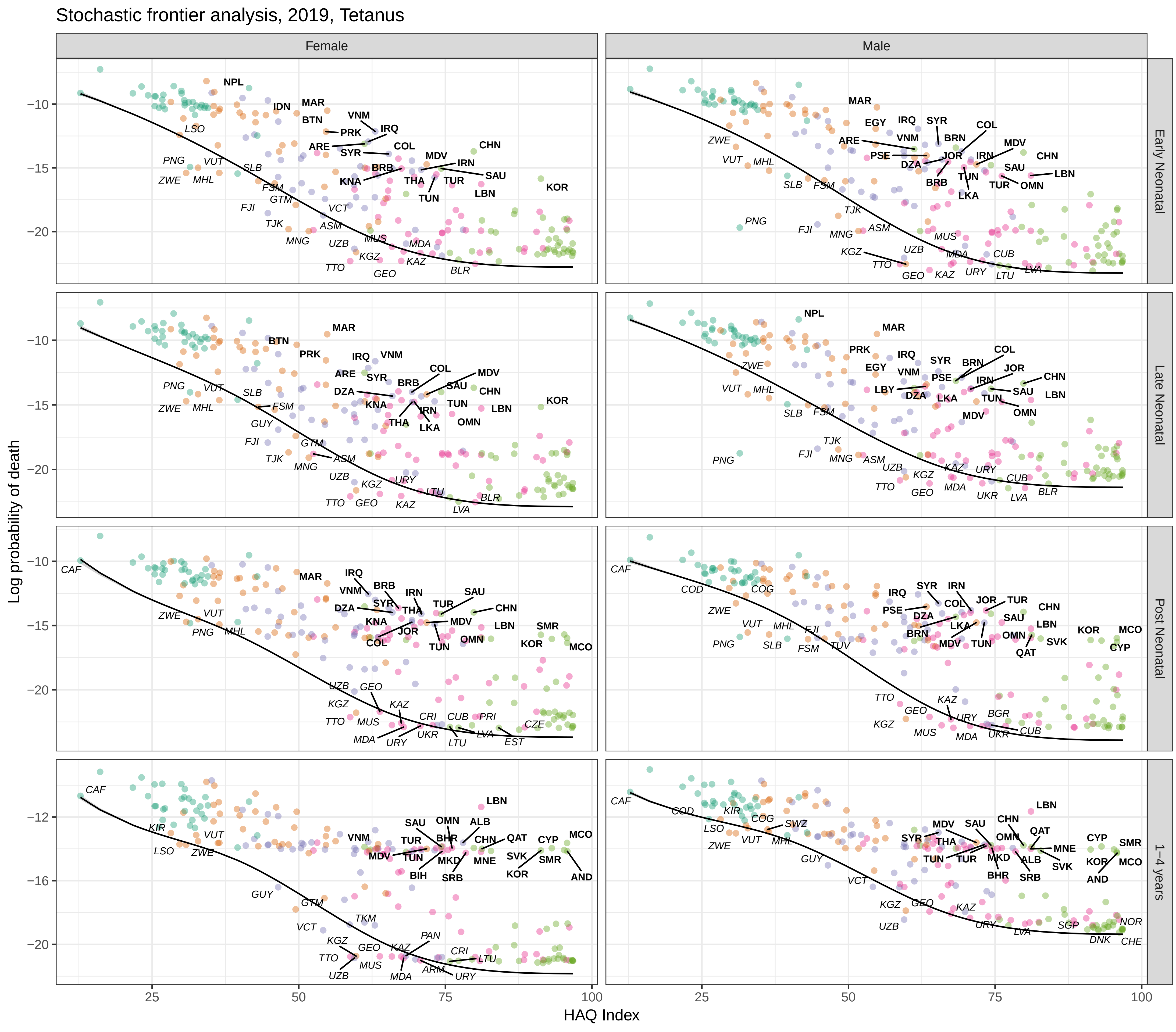

Figure S8
Cause- and age-specific mortality by country and Healthcare Access and Quality (HAQ) Index, with the Survival Potential Frontier (SPF) (black line) and uncertainty interval around the frontier (grey band). Countries are labelled in bold when their ratio to the frontier is in the top 10 percent (performing poorly relative to HAQ Index) and in italics when their ratio to the frontier is in the bottom 10 percent (performing well relative to HAQ Index). These results show the prediction of the cause-specific SPF prior to scaling to the all-cause frontier.

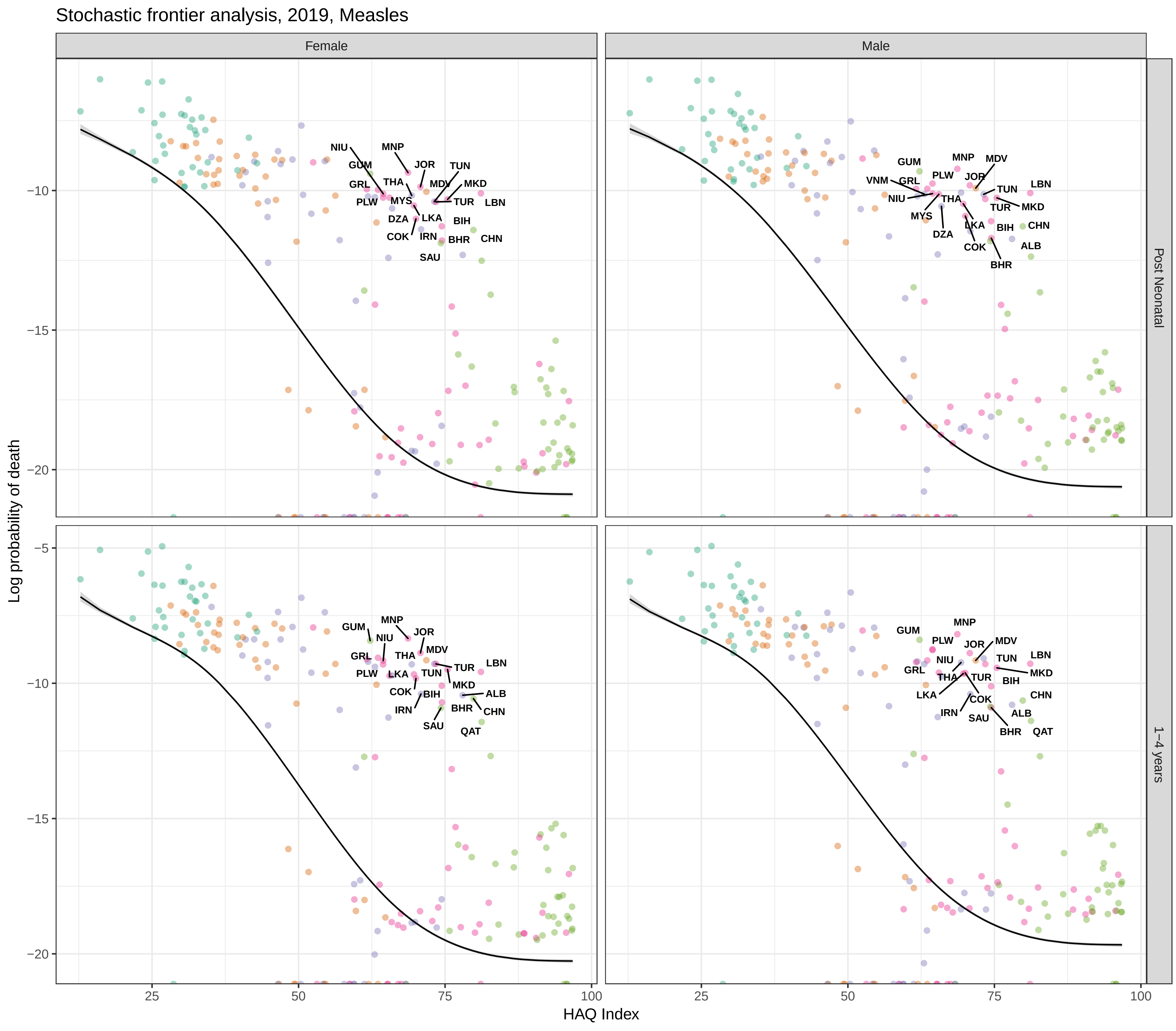

Figure S8
Cause- and age-specific mortality by country and Healthcare Access and Quality (HAQ) Index, with the Survival Potential Frontier (SPF) (black line) and uncertainty interval around the frontier (grey band). Countries are labelled in bold when their ratio to the frontier is in the top 10 percent (performing poorly relative to HAQ Index) and in italics when their ratio to the frontier is in the bottom 10 percent (performing well relative to HAQ Index). These results show the prediction of the cause-specific SPF prior to scaling to the all-cause frontier.

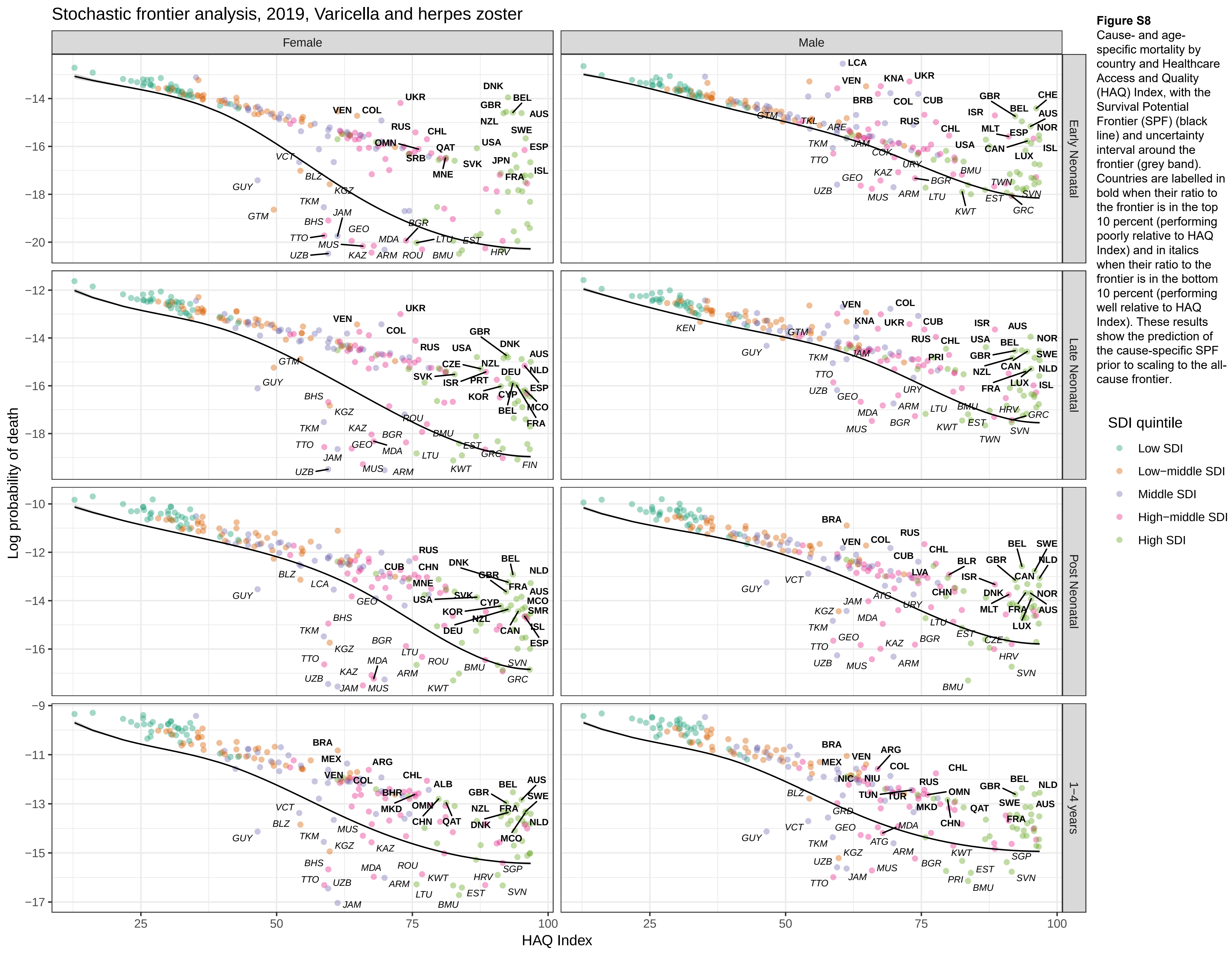

Figure S8
Cause- and age-specific mortality by country and Healthcare Access and Quality (HAQ) Index, with the Survival Potential Frontier (SPF) (black line) and uncertainty interval around the frontier (grey band). Countries are labelled in bold when their ratio to the frontier is in the top 10 percent (performing poorly relative to HAQ Index) and in italics when their ratio to the frontier is in the bottom 10 percent (performing well relative to HAQ Index). These results show the prediction of the cause-specific SPF prior to scaling to the all-cause frontier.

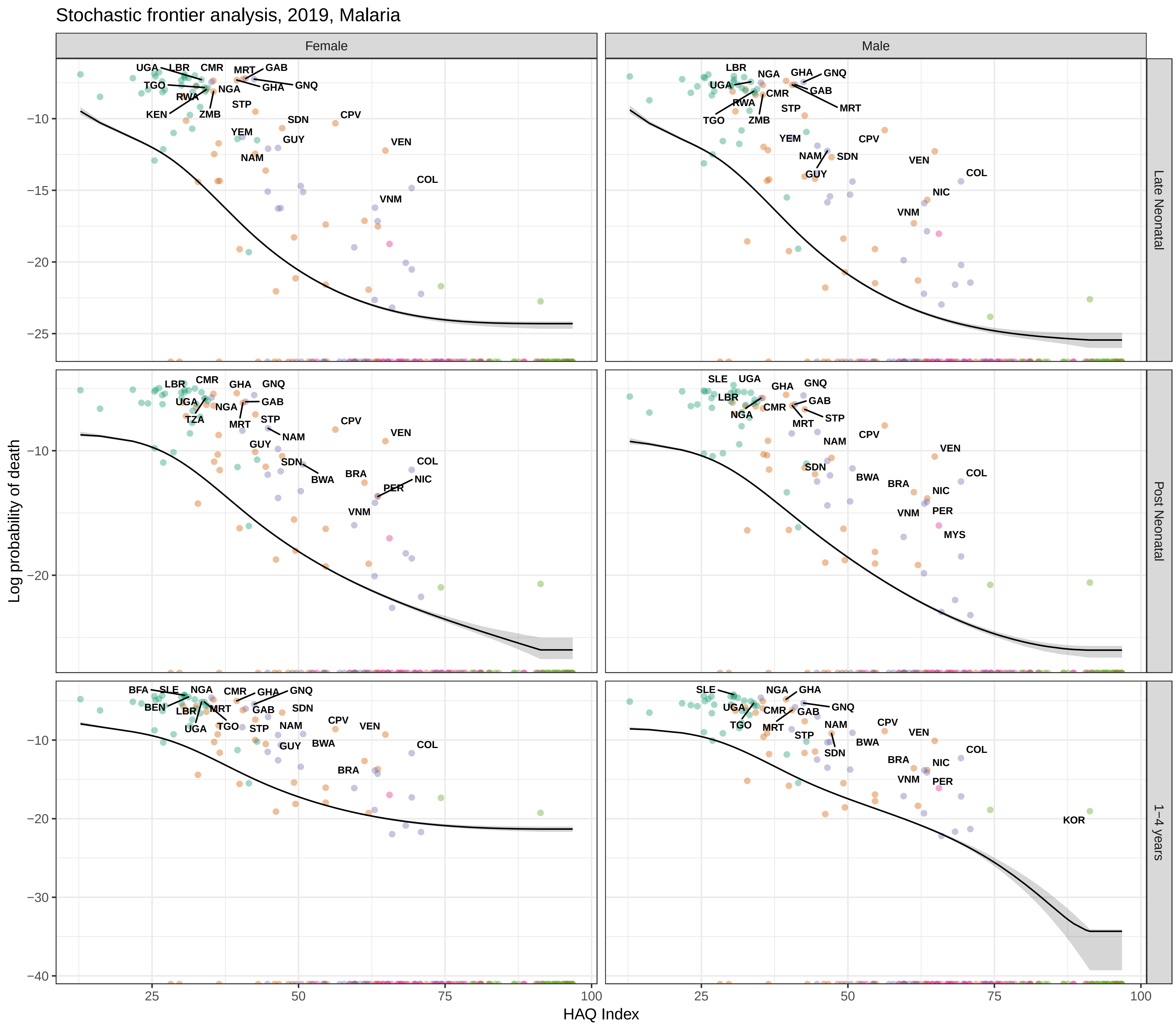

Figure S8
Cause- and age-specific mortality by country and Healthcare Access and Quality (HAQ) Index, with the Survival Potential Frontier (SPF) (black line) and uncertainty interval around the frontier (grey band). Countries are labelled in bold when their ratio to the frontier is in the top 10 percent (performing poorly relative to HAQ Index) and in italics when their ratio to the frontier is in the bottom 10 percent (performing well relative to HAQ Index). These results show the prediction of the cause-specific SPF prior to scaling to the all-cause frontier.

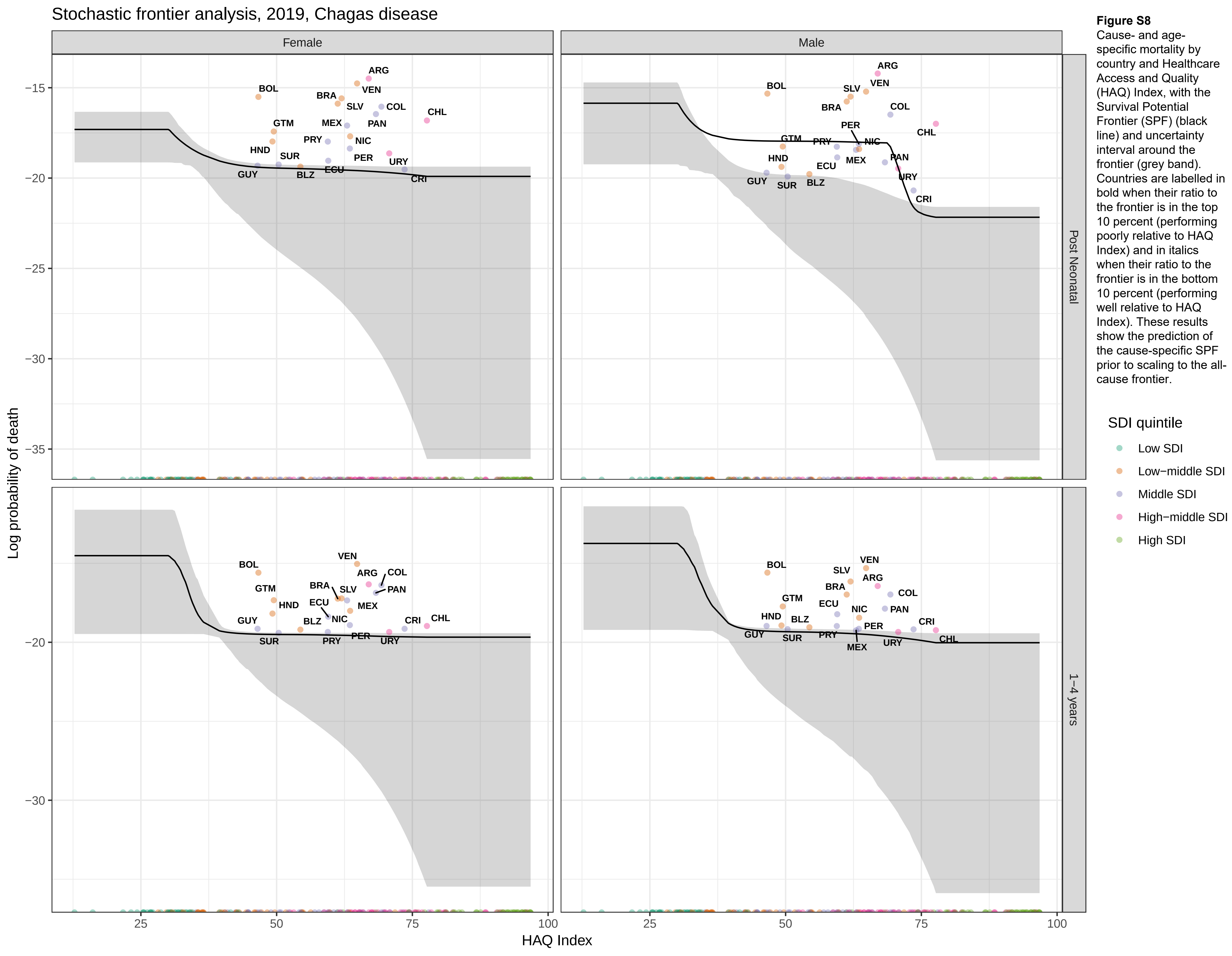

Figure S8
Cause- and age-specific mortality by country and Healthcare Access and Quality (HAQ) Index, with the Survival Potential Frontier (SPF) (black line) and uncertainty interval around the frontier (grey band). Countries are labelled in bold when their ratio to the frontier is in the top 10 percent (performing poorly relative to HAQ Index) and in italics when their ratio to the frontier is in the bottom 10 percent (performing well relative to HAQ Index). These results show the prediction of the cause-specific SPF prior to scaling to the all-cause frontier.

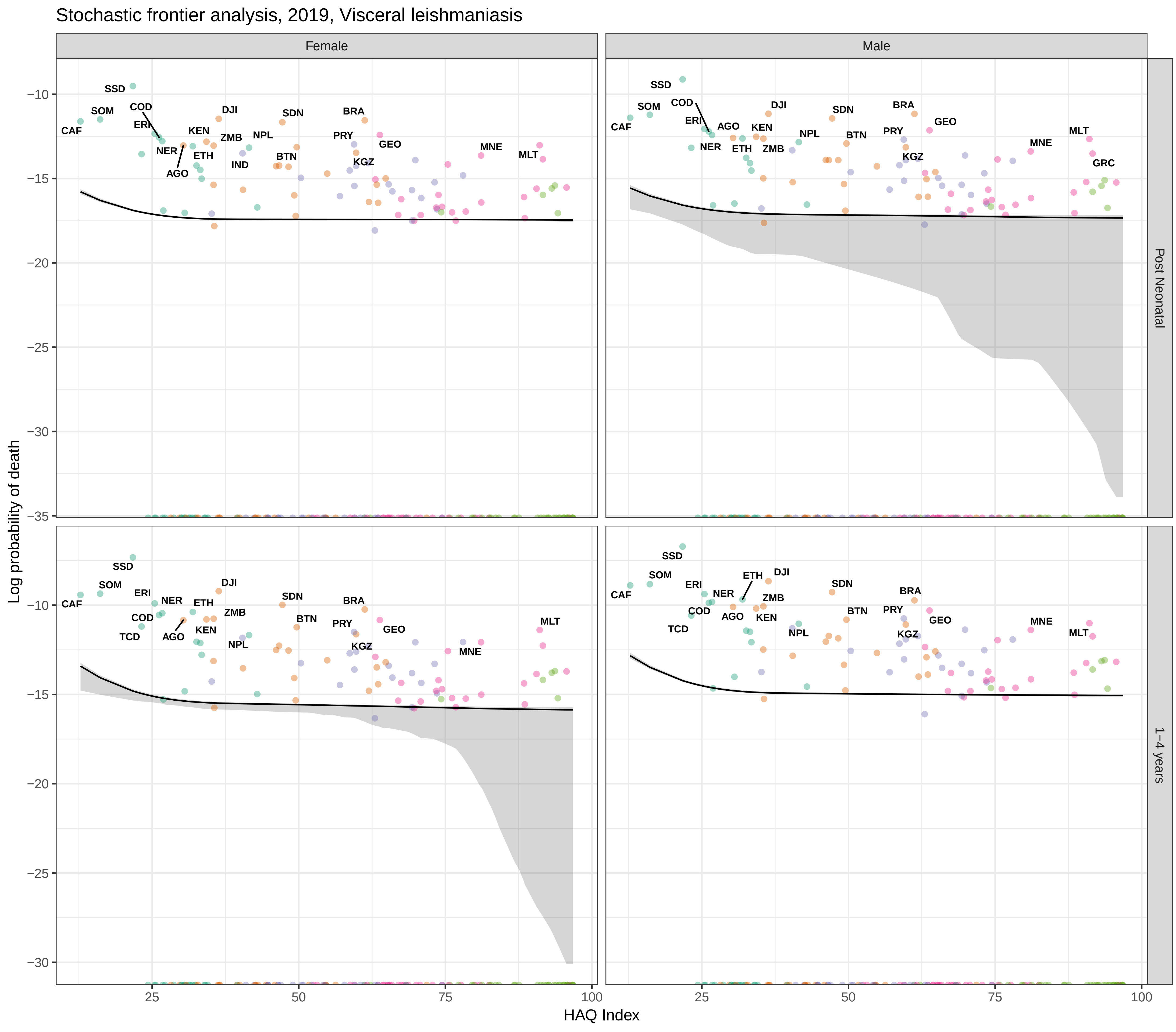

Figure S8
Cause- and age-specific mortality by country and Healthcare Access and Quality (HAQ) Index, with the Survival Potential Frontier (SPF) (black line) and uncertainty interval around the frontier (grey band). Countries are labelled in bold when their ratio to the frontier is in the top 10 percent (performing poorly relative to HAQ Index) and in italics when their ratio to the frontier is in the bottom 10 percent (performing well relative to HAQ Index). These results show the prediction of the cause-specific SPF prior to scaling to the all-cause frontier.

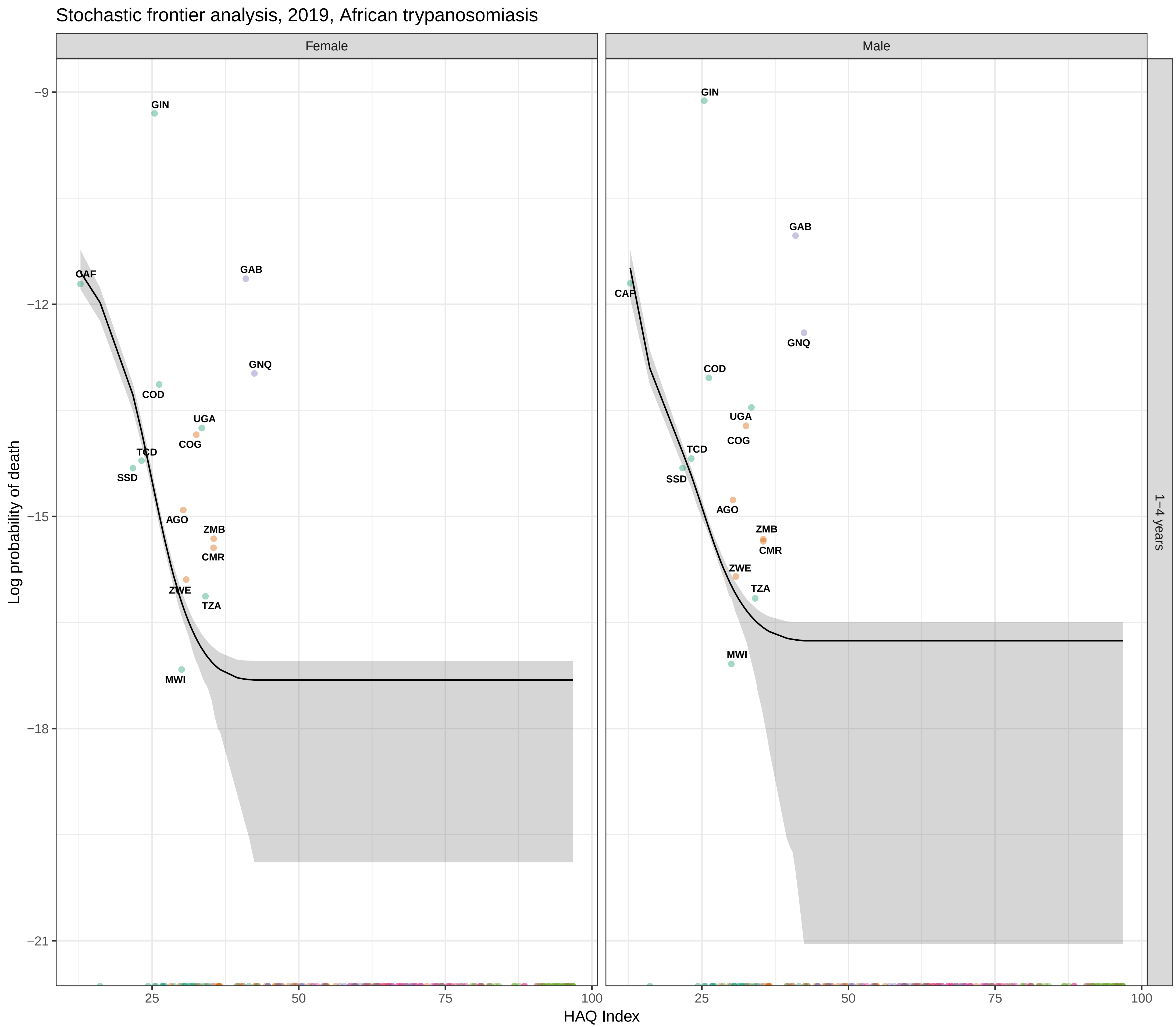

Figure S8
Cause- and age-specific mortality by country and Healthcare Access and Quality (HAQ) Index, with the Survival Potential Frontier (SPF) (black line) and uncertainty interval around the frontier (grey band). Countries are labelled in bold when their ratio to the frontier is in the top 10 percent (performing poorly relative to HAQ Index) and in italics when their ratio to the frontier is in the bottom 10 percent (performing well relative to HAQ Index). These results show the prediction of the cause-specific SPF prior to scaling to the all-cause frontier.

SDI quintile
- Low SDI
- Low−middle SDI
- Middle SDI
- High−middle SDI
- High SDI

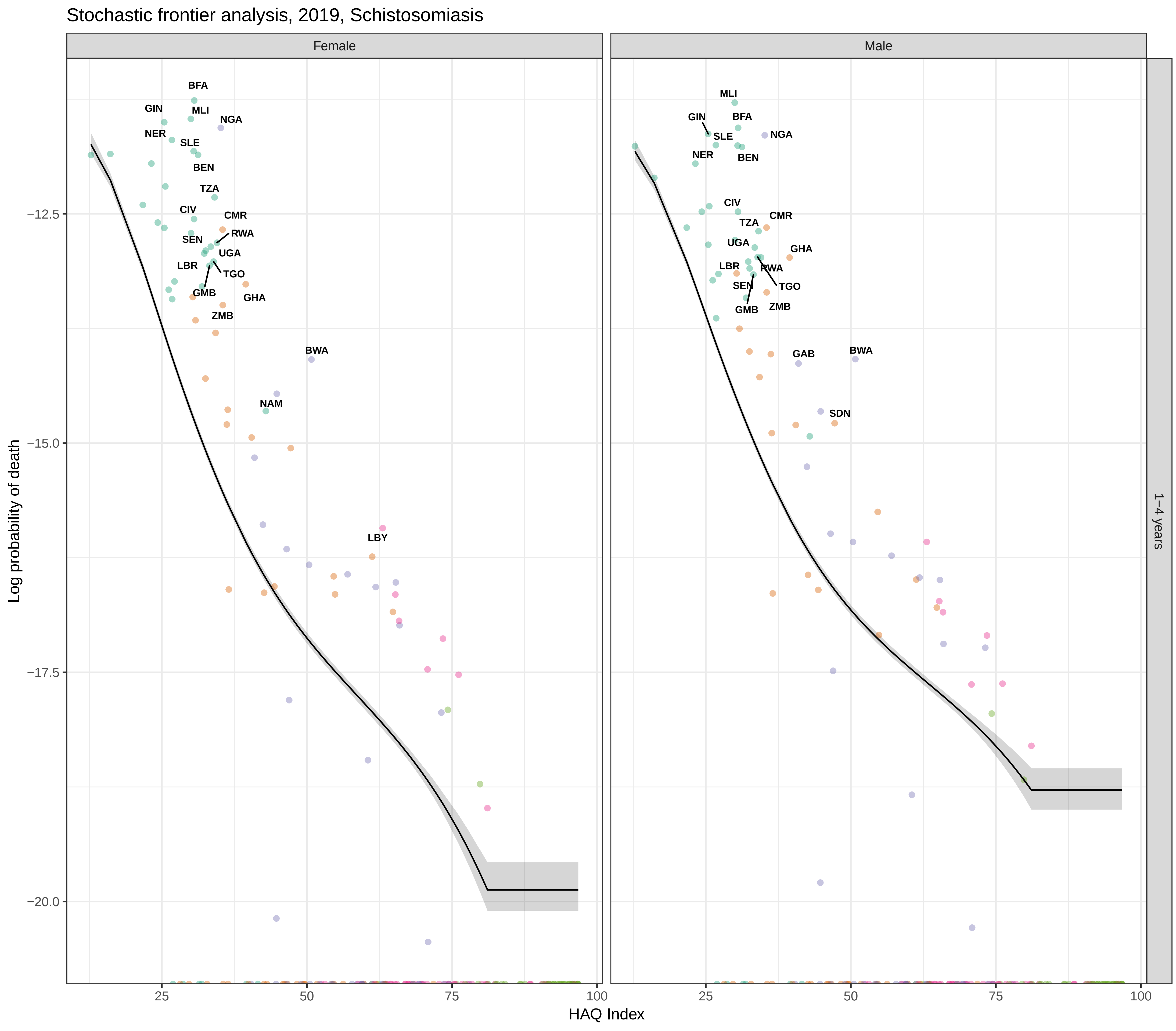

Figure S8
Cause- and age-specific mortality by country and Healthcare Access and Quality (HAQ) Index, with the Survival Potential Frontier (SPF) (black line) and uncertainty interval around the frontier (grey band). Countries are labelled in bold when their ratio to the frontier is in the top 10 percent (performing poorly relative to HAQ Index) and in italics when their ratio to the frontier is in the bottom 10 percent (performing well relative to HAQ Index). These results show the prediction of the cause-specific SPF prior to scaling to the all-cause frontier.

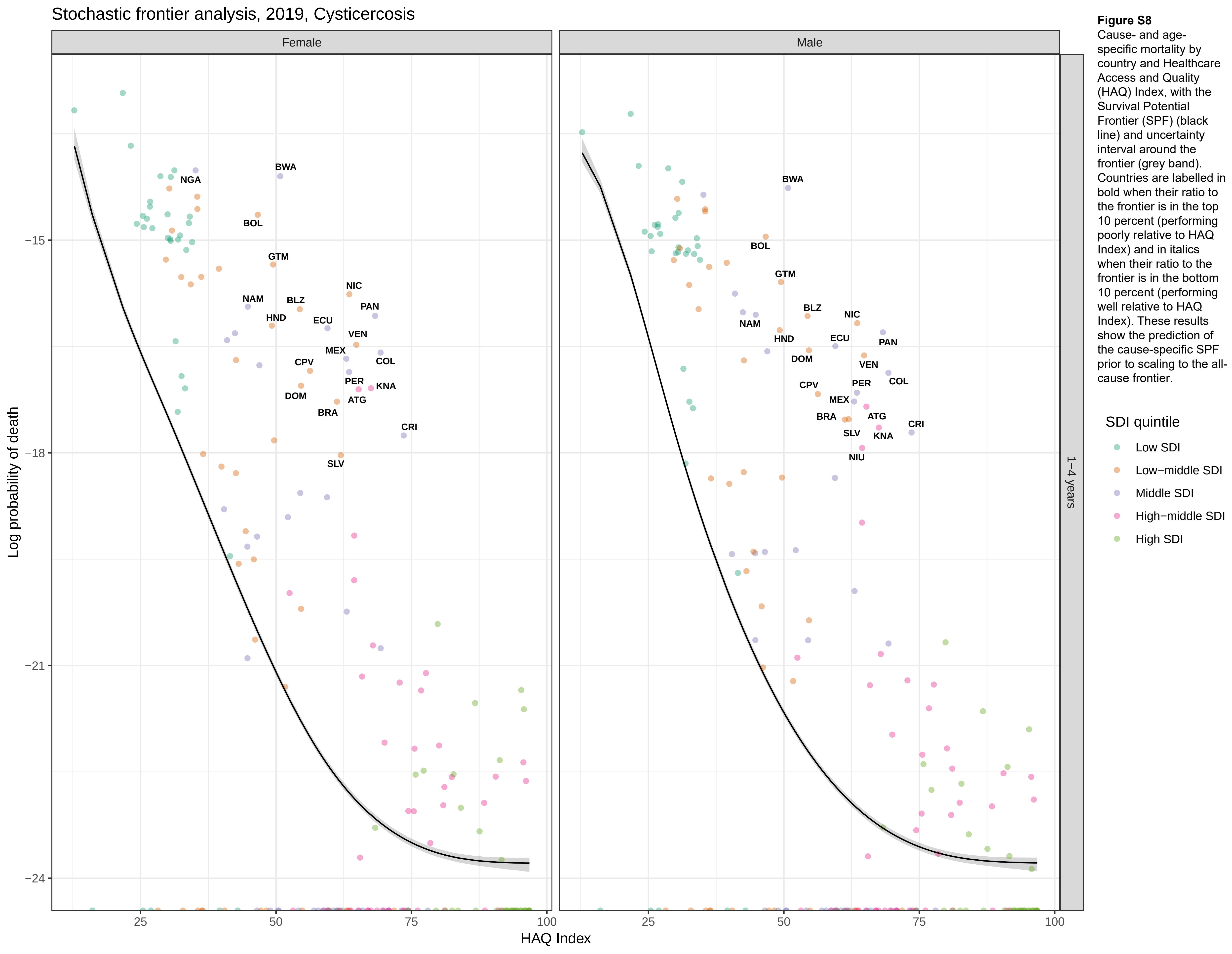

Figure S8
Cause- and age-specific mortality by country and Healthcare Access and Quality (HAQ) Index, with the Survival Potential Frontier (SPF) (black line) and uncertainty interval around the frontier (grey band). Countries are labelled in bold when their ratio to the frontier is in the top 10 percent (performing poorly relative to HAQ Index) and in italics when their ratio to the frontier is in the bottom 10 percent (performing well relative to HAQ Index). These results show the prediction of the cause-specific SPF prior to scaling to the all-cause frontier.

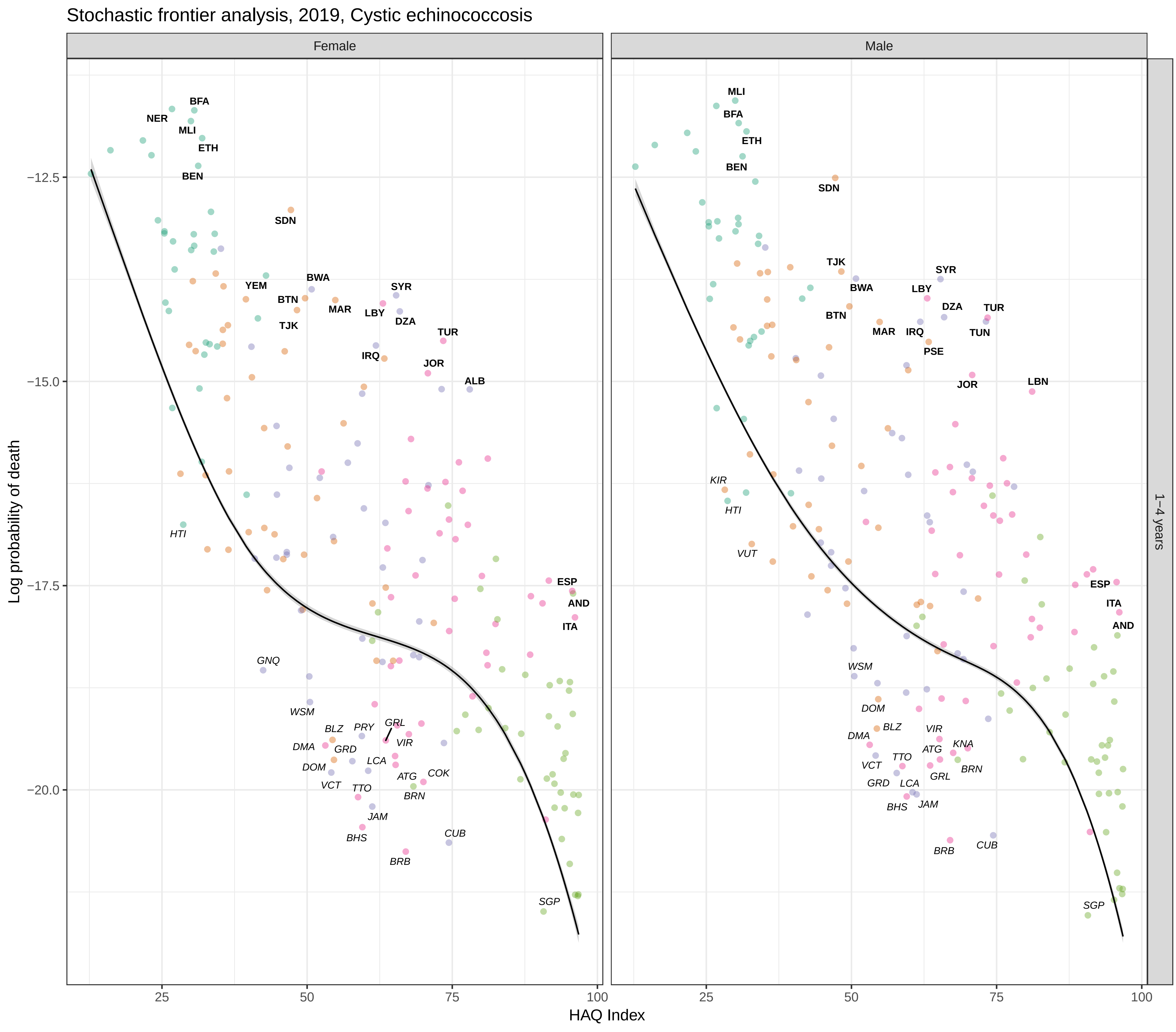

Figure S8
Cause- and age-specific mortality by country and Healthcare Access and Quality (HAQ) Index, with the Survival Potential Frontier (SPF) (black line) and uncertainty interval around the frontier (grey band). Countries are labelled in bold when their ratio to the frontier is in the top 10 percent (performing poorly relative to HAQ Index) and in italics when their ratio to the frontier is in the bottom 10 percent (performing well relative to HAQ Index). These results show the prediction of the cause-specific SPF prior to scaling to the all-cause frontier.

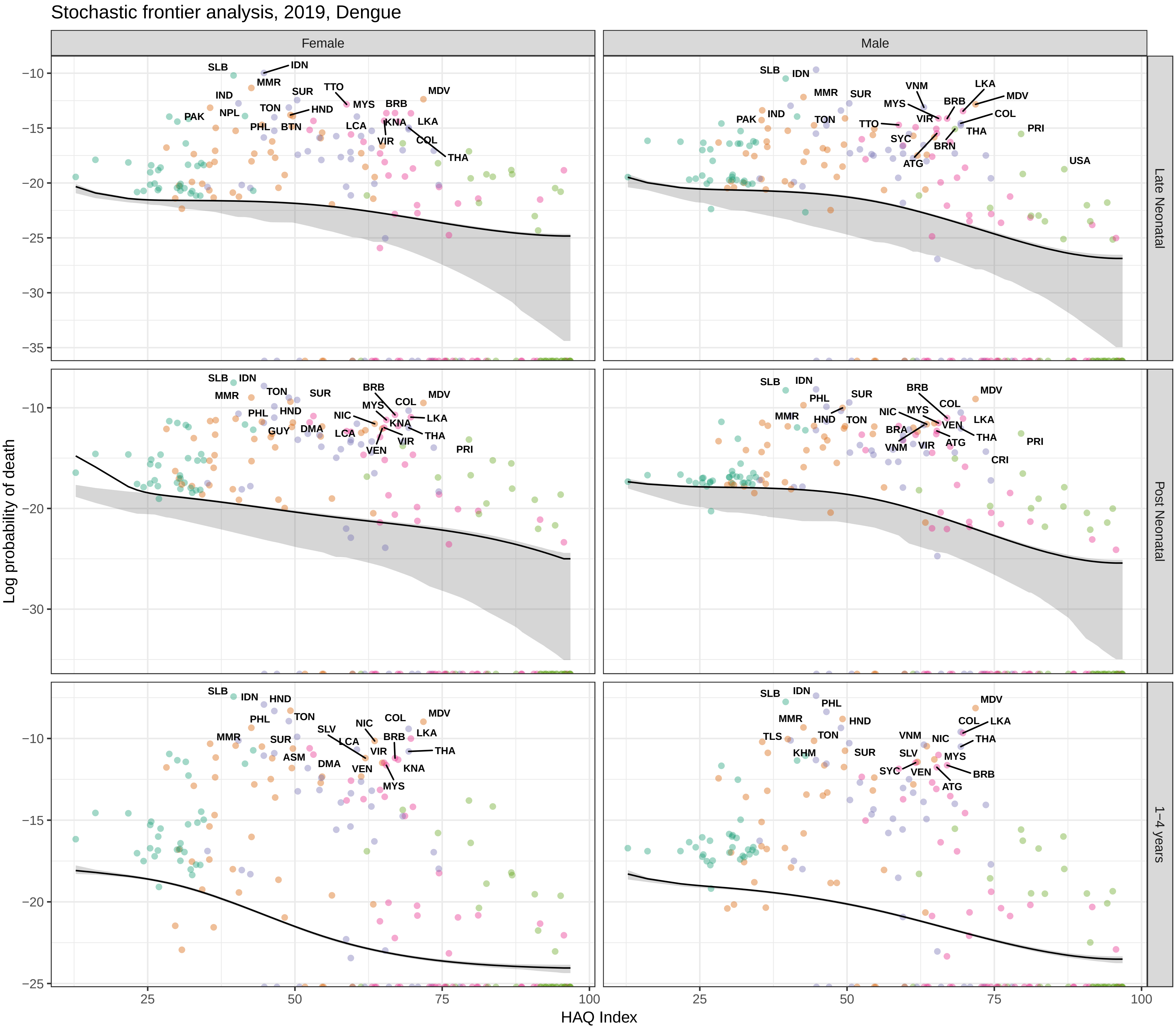

Figure S8
Cause- and age-specific mortality by country and Healthcare Access and Quality (HAQ) Index, with the Survival Potential Frontier (SPF) (black line) and uncertainty interval around the frontier (grey band). Countries are labelled in bold when their ratio to the frontier is in the top 10 percent (performing poorly relative to HAQ Index) and in italics when their ratio to the frontier is in the bottom 10 percent (performing well relative to HAQ Index). These results show the prediction of the cause-specific SPF prior to scaling to the all-cause frontier.

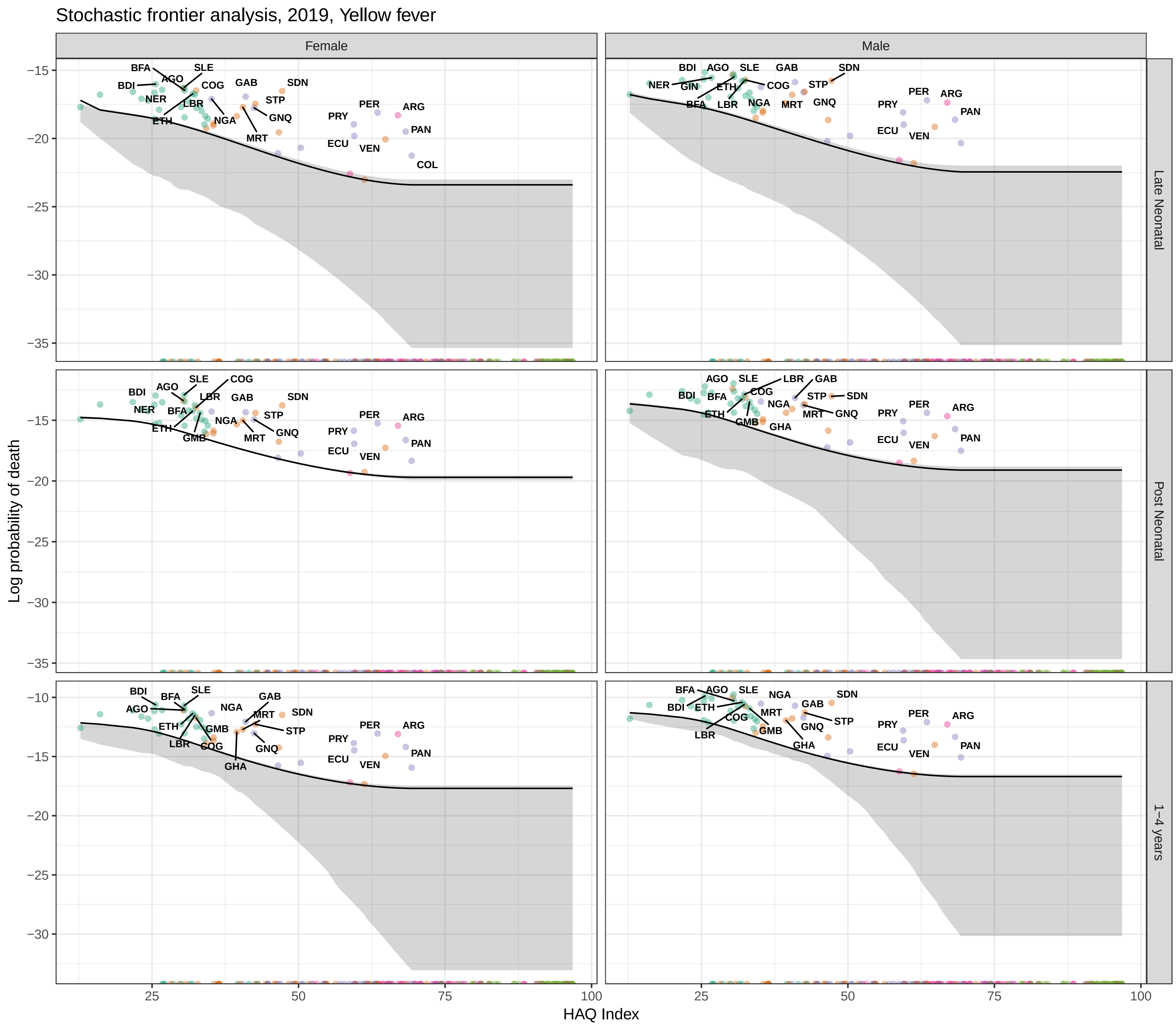

Figure S8
Cause- and age-specific mortality by country and Healthcare Access and Quality (HAQ) Index, with the Survival Potential Frontier (SPF) (black line) and uncertainty interval around the frontier (grey band). Countries are labelled in bold when their ratio to the frontier is in the top 10 percent (performing poorly relative to HAQ Index) and in italics when their ratio to the frontier is in the bottom 10 percent (performing well relative to HAQ Index). These results show the prediction of the cause-specific SPF prior to scaling to the all-cause frontier.

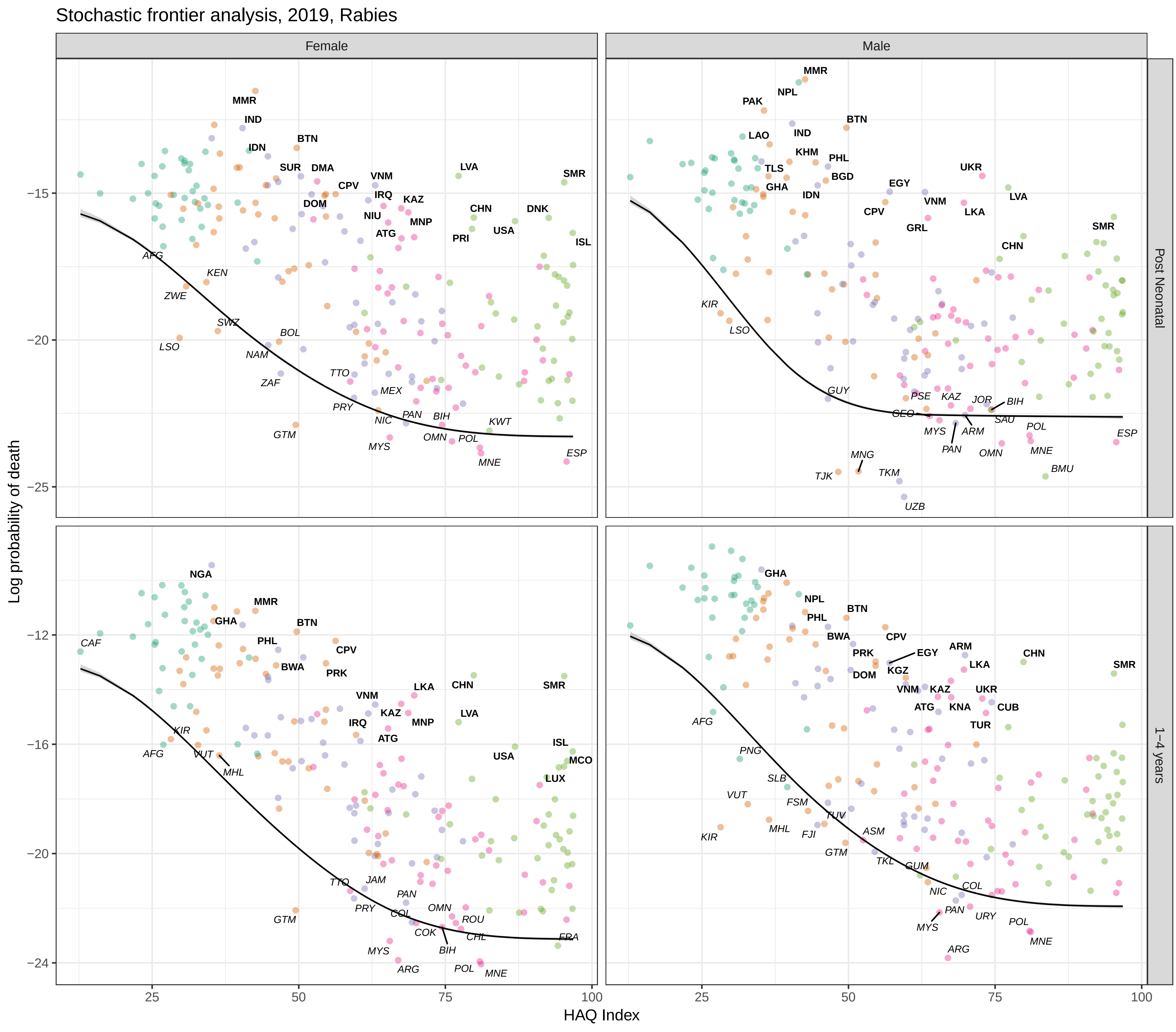

Figure S8
Cause- and age-specific mortality by country and Healthcare Access and Quality (HAQ) Index, with the Survival Potential Frontier (SPF) (black line) and uncertainty interval around the frontier (grey band). Countries are labelled in bold when their ratio to the frontier is in the top 10 percent (performing poorly relative to HAQ Index) and in italics when their ratio to the frontier is in the bottom 10 percent (performing well relative to HAQ Index). These results show the prediction of the cause-specific SPF prior to scaling to the all-cause frontier.

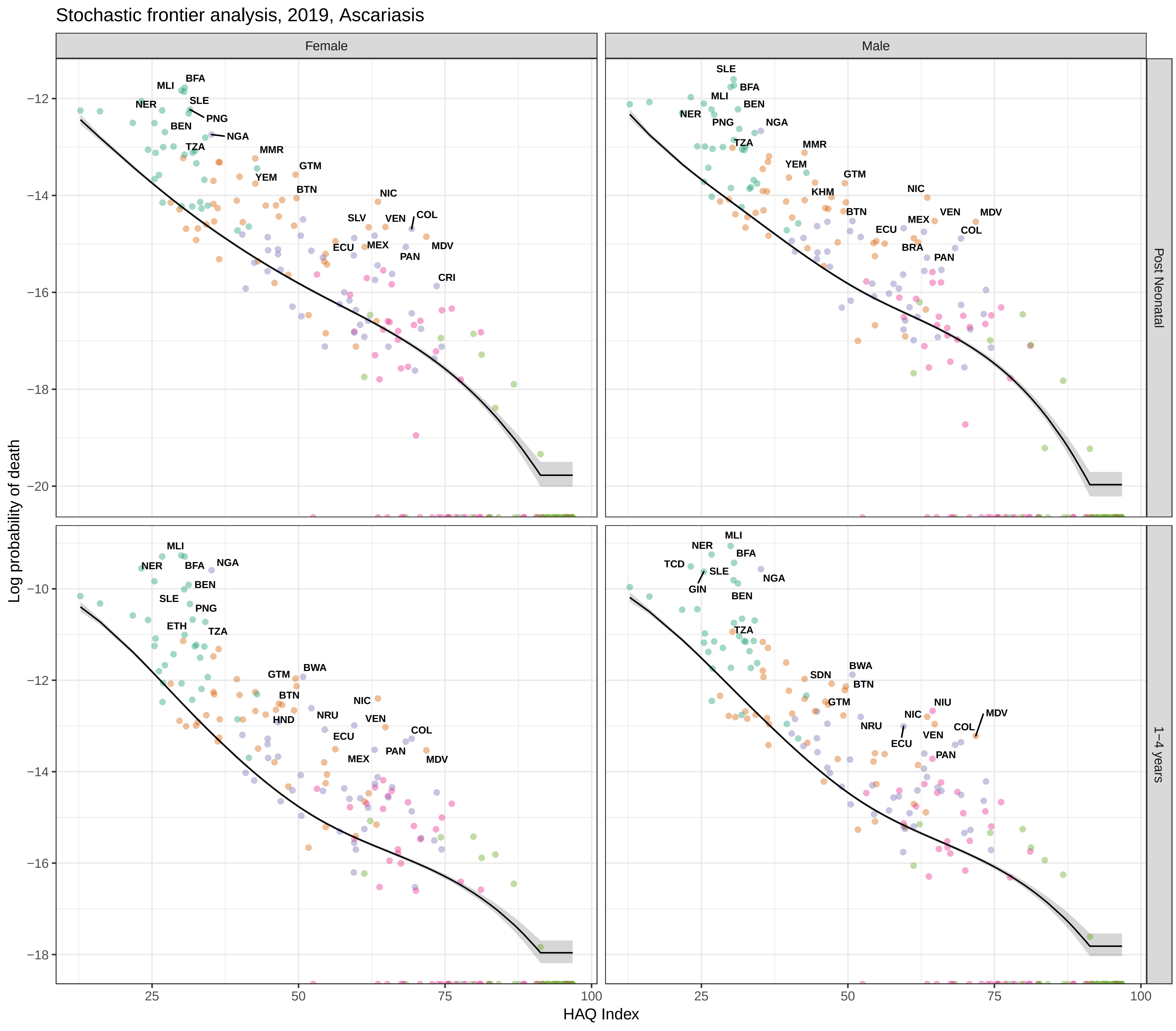

Figure S8
Cause- and age-specific mortality by country and Healthcare Access and Quality (HAQ) Index, with the Survival Potential Frontier (SPF) (black line) and uncertainty interval around the frontier (grey band). Countries are labelled in bold when their ratio to the frontier is in the top 10 percent (performing poorly relative to HAQ Index) and in italics when their ratio to the frontier is in the bottom 10 percent (performing well relative to HAQ Index). These results show the prediction of the cause-specific SPF prior to scaling to the all-cause frontier.

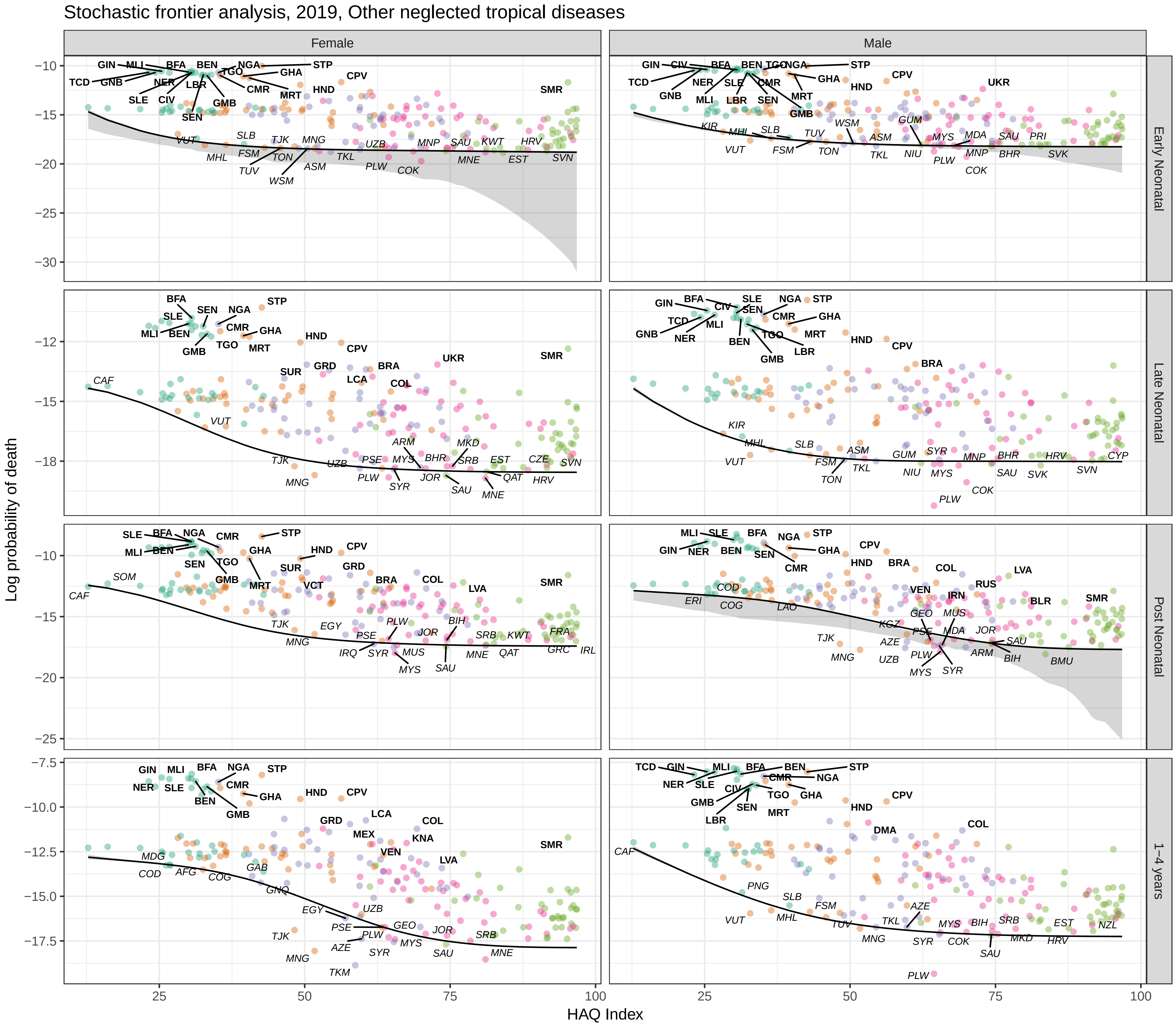

Figure S8
Cause- and age-specific mortality by country and Healthcare Access and Quality (HAQ) Index, with the Survival Potential Frontier (SPF) (black line) and uncertainty interval around the frontier (grey band). Countries are labelled in bold when their ratio to the frontier is in the top 10 percent (performing poorly relative to HAQ Index) and in italics when their ratio to the frontier is in the bottom 10 percent (performing well relative to HAQ Index). These results show the prediction of the cause-specific SPF prior to scaling to the all-cause frontier.

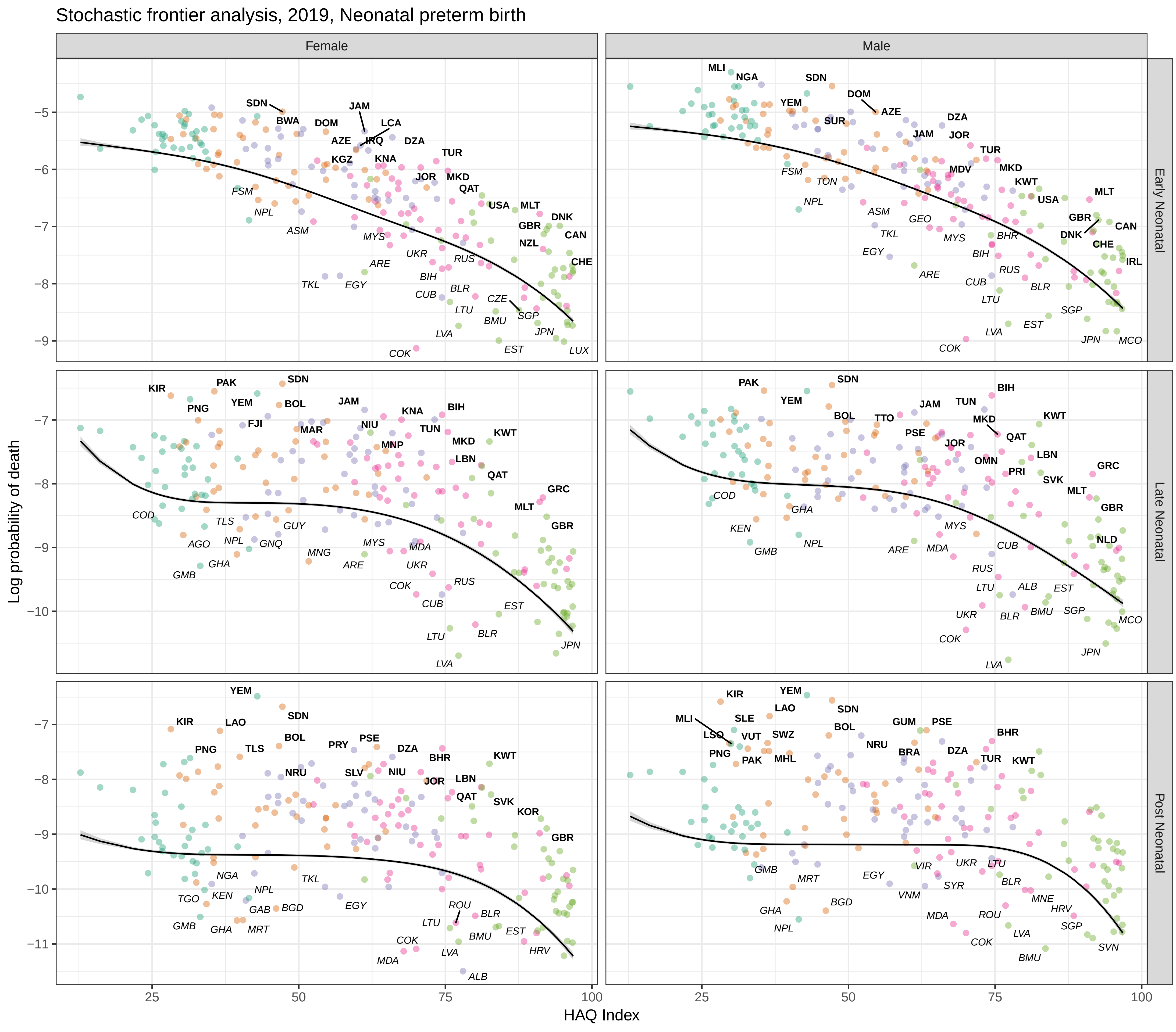

Figure S8
Cause- and age-specific mortality by country and Healthcare Access and Quality (HAQ) Index, with the Survival Potential Frontier (SPF) (black line) and uncertainty interval around the frontier (grey band). Countries are labelled in bold when their ratio to the frontier is in the top 10 percent (performing poorly relative to HAQ Index) and in italics when their ratio to the frontier is in the bottom 10 percent (performing well relative to HAQ Index). These results show the prediction of the cause-specific SPF prior to scaling to the all-cause frontier.

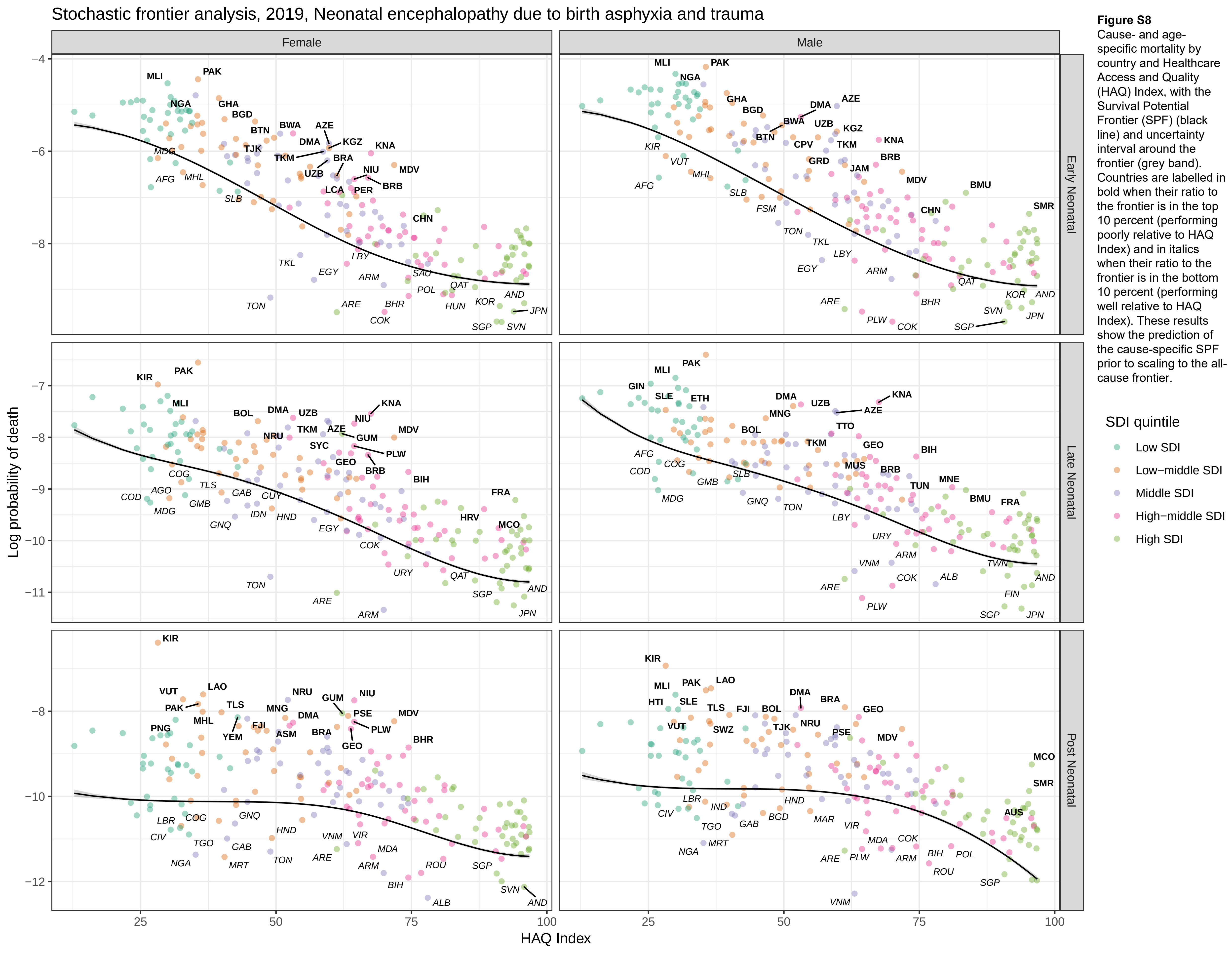

Figure S8
Cause- and age-specific mortality by country and Healthcare Access and Quality (HAQ) Index, with the Survival Potential Frontier (SPF) (black line) and uncertainty interval around the frontier (grey band). Countries are labelled in bold when their ratio to the frontier is in the top 10 percent (performing poorly relative to HAQ Index) and in italics when their ratio to the frontier is in the bottom 10 percent (performing well relative to HAQ Index). These results show the prediction of the cause-specific SPF prior to scaling to the all-cause frontier.

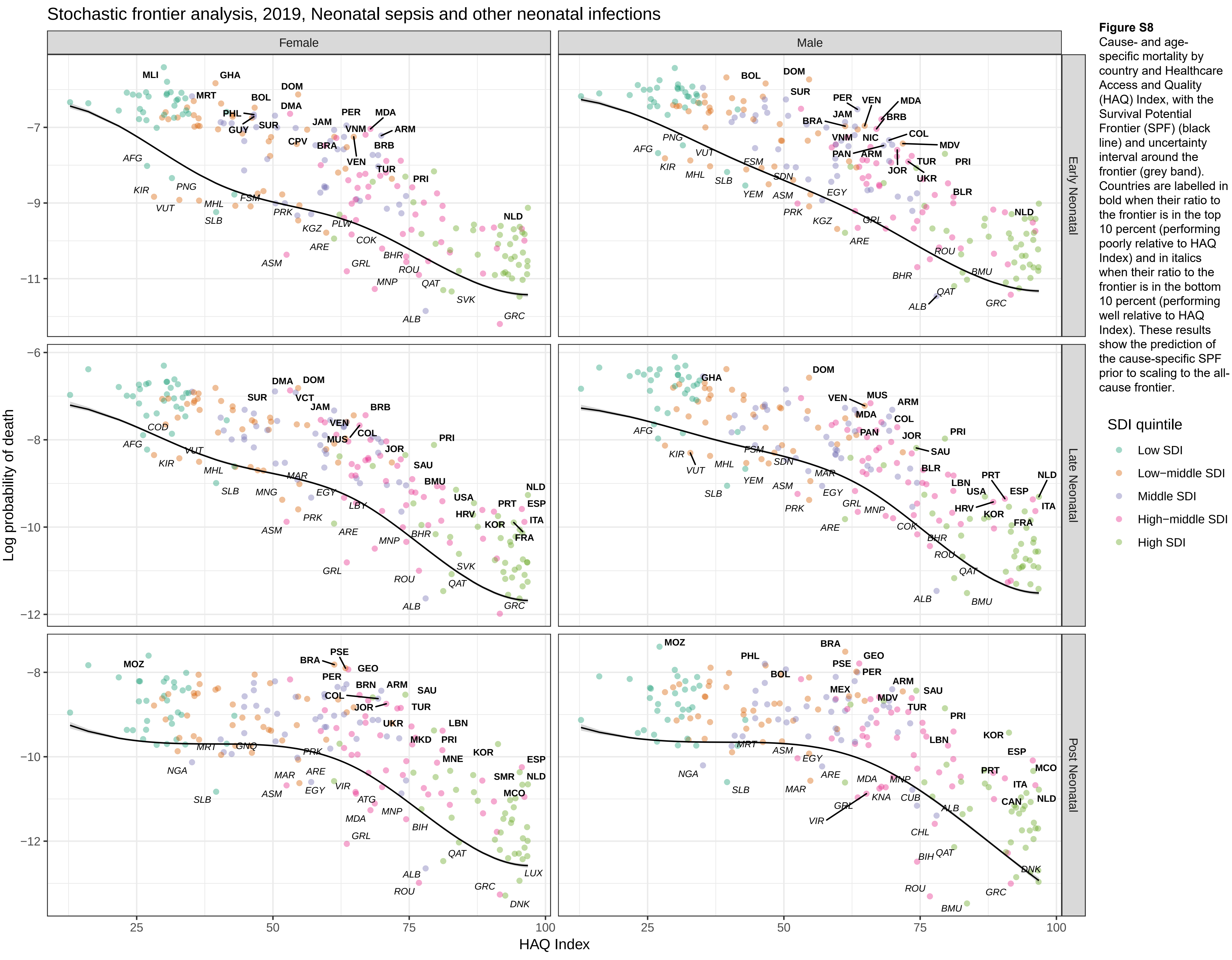

Figure S8
Cause- and age-specific mortality by country and Healthcare Access and Quality (HAQ) Index, with the Survival Potential Frontier (SPF) (black line) and uncertainty interval around the frontier (grey band). Countries are labelled in bold when their ratio to the frontier is in the top 10 percent (performing poorly relative to HAQ Index) and in italics when their ratio to the frontier is in the bottom 10 percent (performing well relative to HAQ Index). These results show the prediction of the cause-specific SPF prior to scaling to the all-cause frontier.

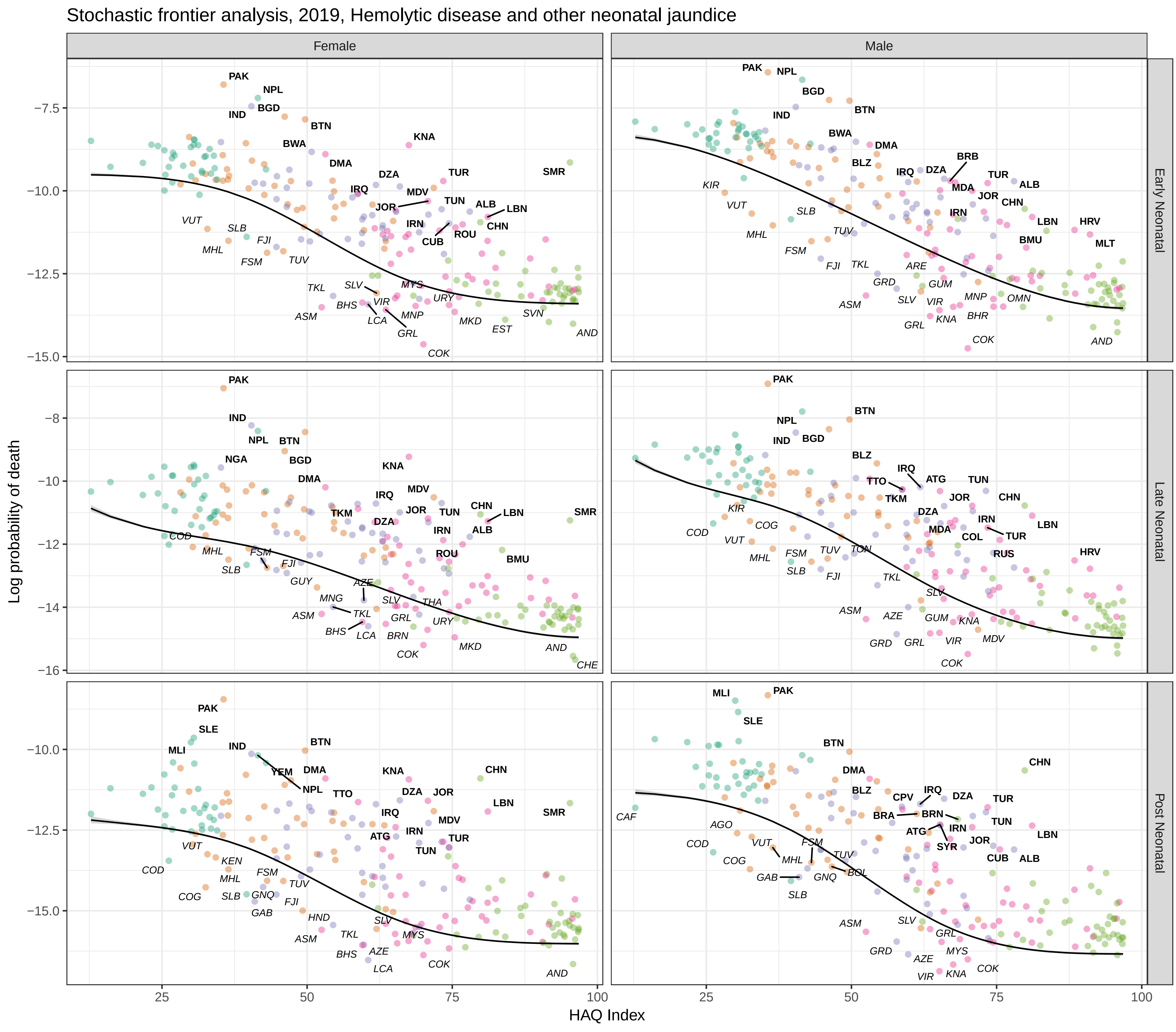

Figure S8
Cause- and age-specific mortality by country and Healthcare Access and Quality (HAQ) Index, with the Survival Potential Frontier (SPF) (black line) and uncertainty interval around the frontier (grey band). Countries are labelled in bold when their ratio to the frontier is in the top 10 percent (performing poorly relative to HAQ Index) and in italics when their ratio to the frontier is in the bottom 10 percent (performing well relative to HAQ Index). These results show the prediction of the cause-specific SPF prior to scaling to the all-cause frontier.

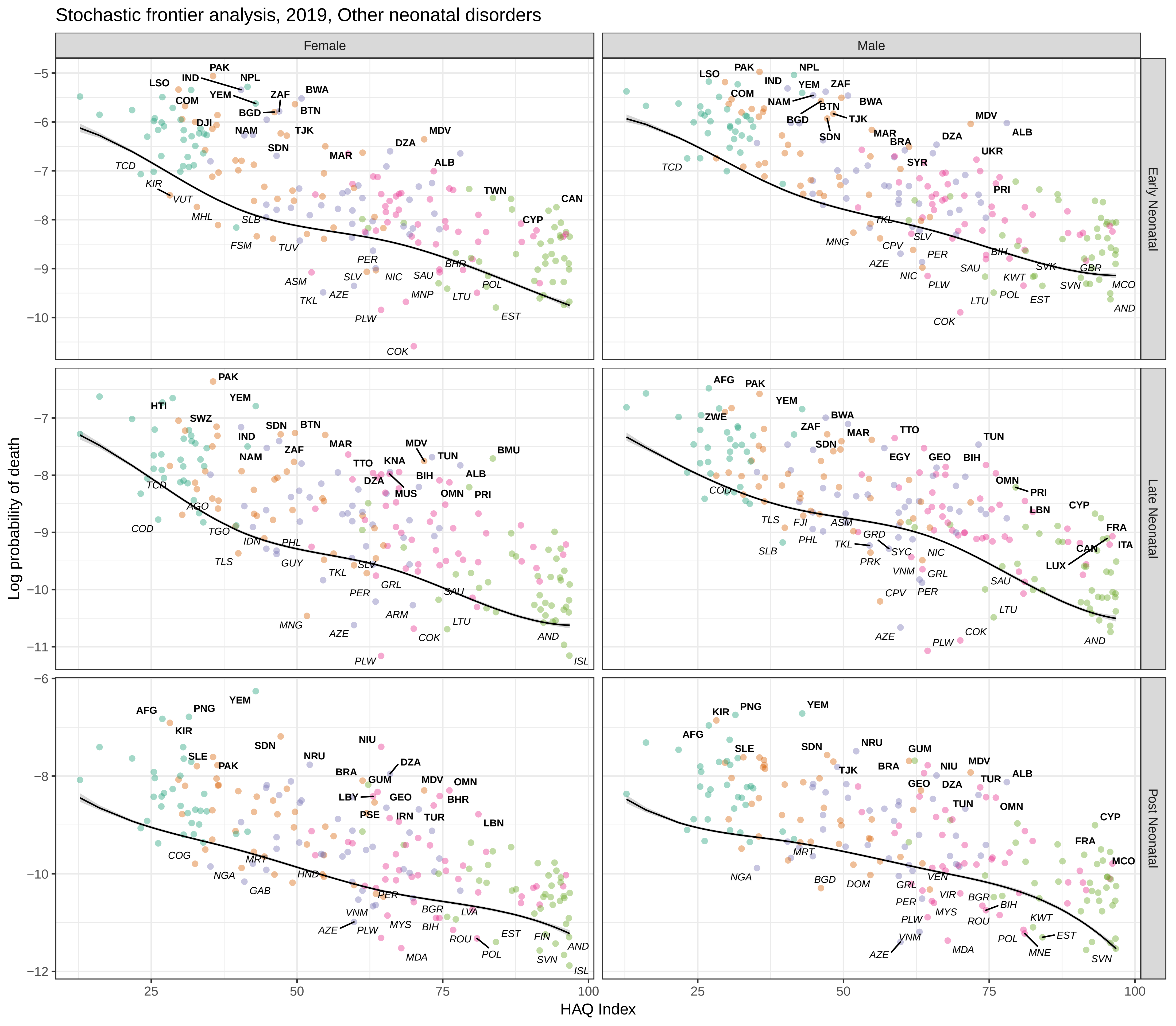

Figure S8
Cause- and age-specific mortality by country and Healthcare Access and Quality (HAQ) Index, with the Survival Potential Frontier (SPF) (black line) and uncertainty interval around the frontier (grey band). Countries are labelled in bold when their ratio to the frontier is in the top 10 percent (performing poorly relative to HAQ Index) and in italics when their ratio to the frontier is in the bottom 10 percent (performing well relative to HAQ Index). These results show the prediction of the cause-specific SPF prior to scaling to the all-cause frontier.

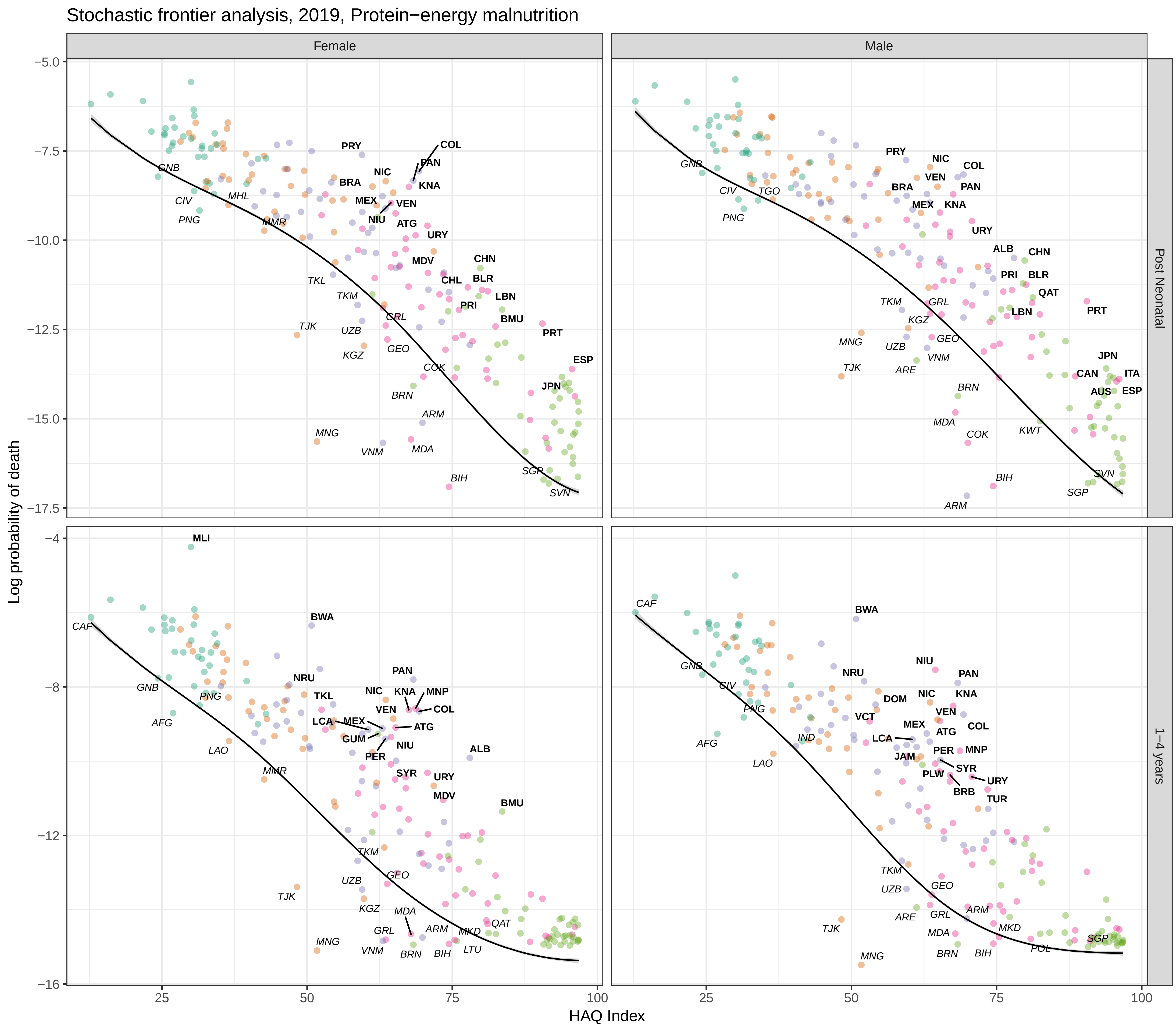

Figure S8
Cause- and age-specific mortality by country and Healthcare Access and Quality (HAQ) Index, with the Survival Potential Frontier (SPF) (black line) and uncertainty interval around the frontier (grey band). Countries are labelled in bold when their ratio to the frontier is in the top 10 percent (performing poorly relative to HAQ Index) and in italics when their ratio to the frontier is in the bottom 10 percent (performing well relative to HAQ Index). These results show the prediction of the cause-specific SPF prior to scaling to the all-cause frontier.

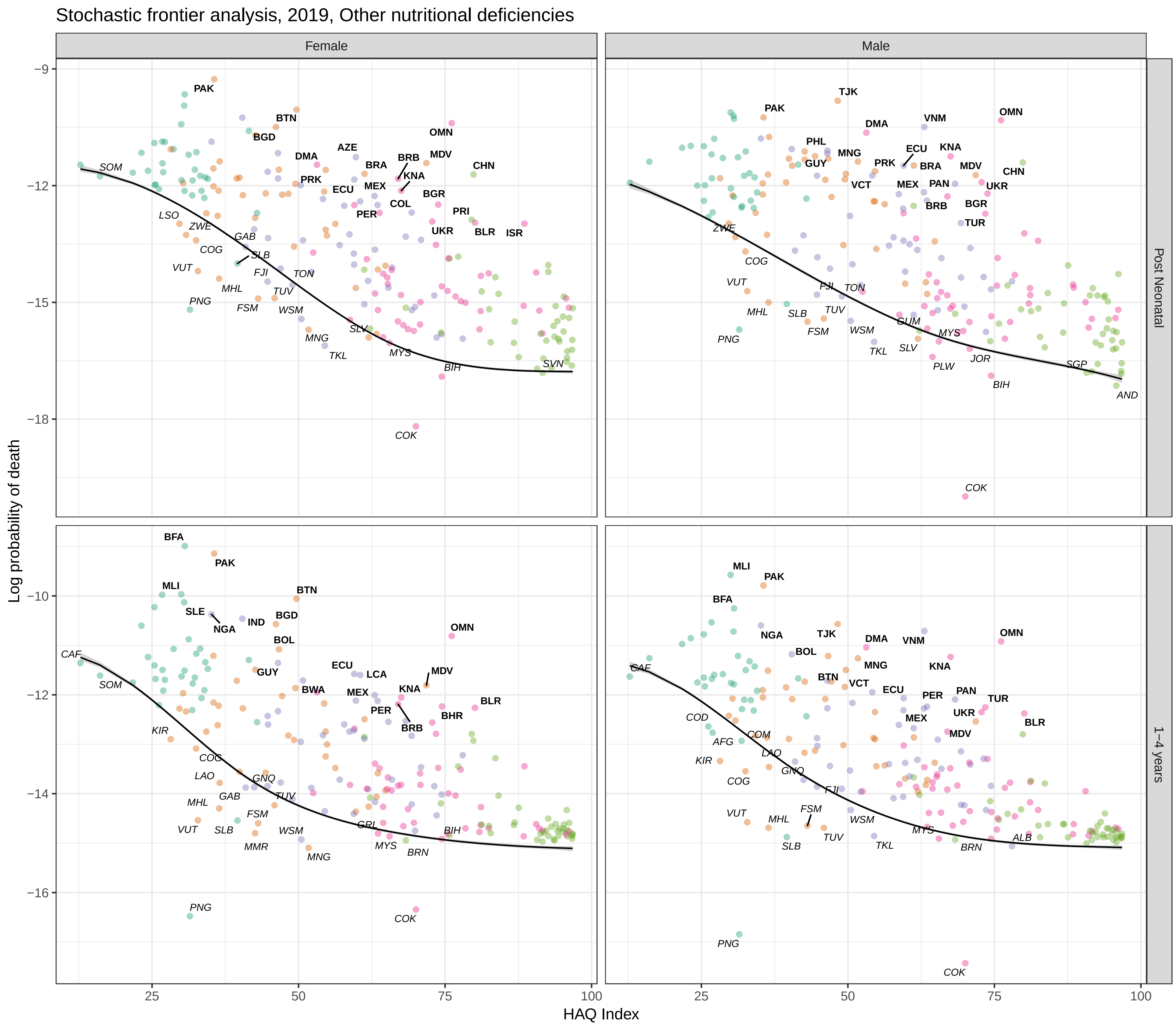

Figure S8
Cause- and age-specific mortality by country and Healthcare Access and Quality (HAQ) Index, with the Survival Potential Frontier (SPF) (black line) and uncertainty interval around the frontier (grey band). Countries are labelled in bold when their ratio to the frontier is in the top 10 percent (performing poorly relative to HAQ Index) and in italics when their ratio to the frontier is in the bottom 10 percent (performing well relative to HAQ Index). These results show the prediction of the cause-specific SPF prior to scaling to the all-cause frontier.

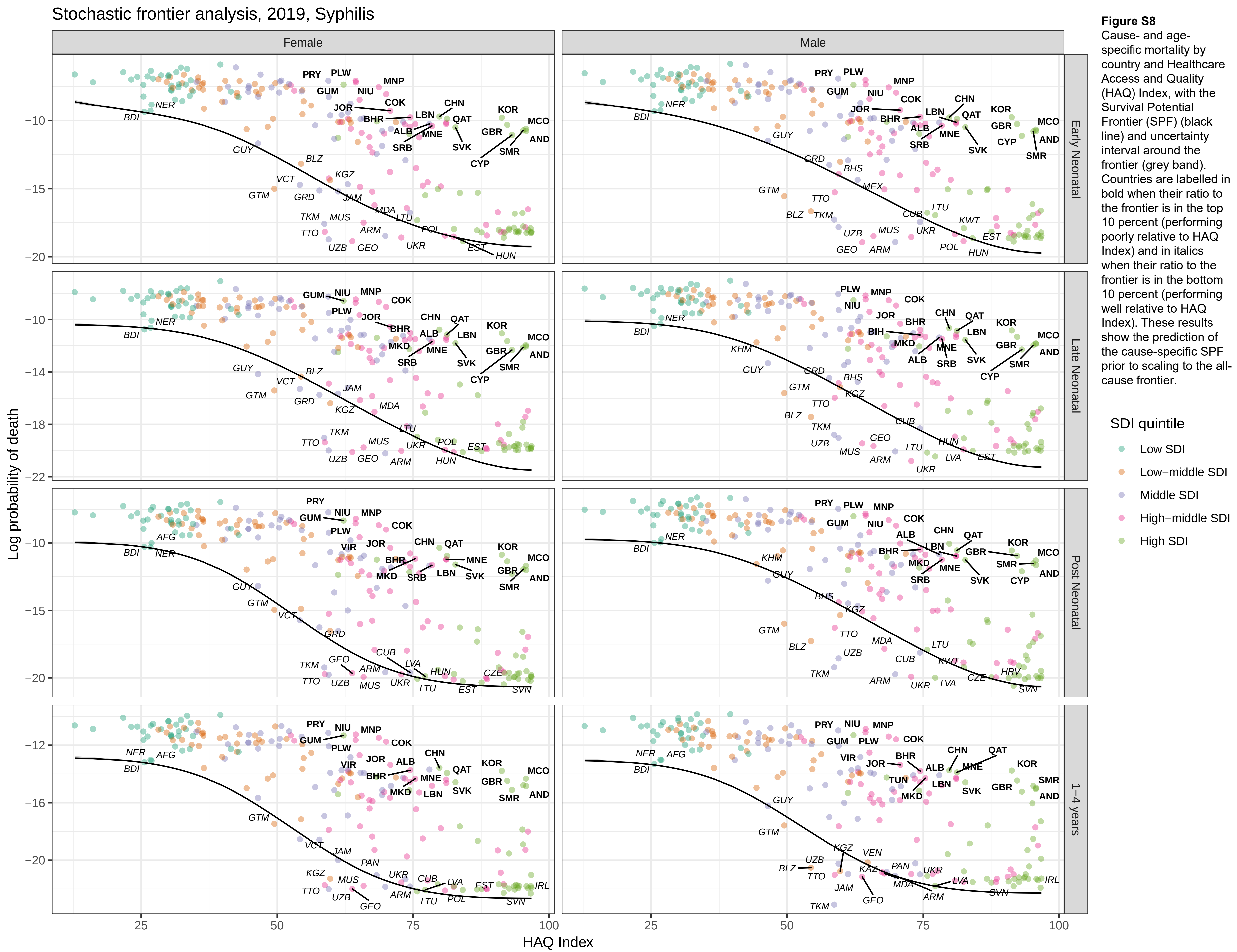

Figure S8
Cause- and age-specific mortality by country and Healthcare Access and Quality (HAQ) Index, with the Survival Potential Frontier (SPF) (black line) and uncertainty interval around the frontier (grey band). Countries are labelled in bold when their ratio to the frontier is in the top 10 percent (performing poorly relative to HAQ Index) and in italics when their ratio to the frontier is in the bottom 10 percent (performing well relative to HAQ Index). These results show the prediction of the cause-specific SPF prior to scaling to the all-cause frontier.

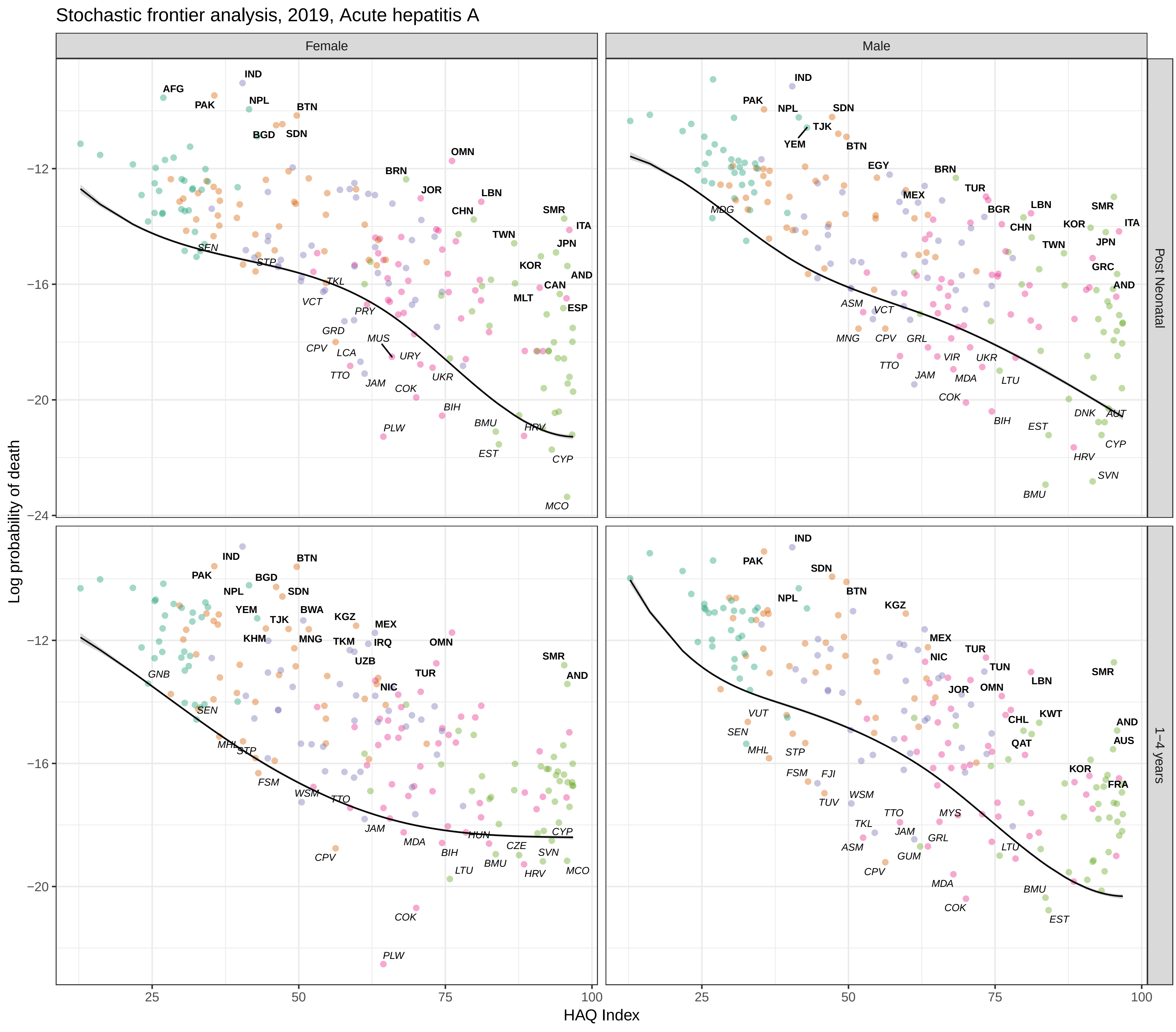

Figure S8
Cause- and age-specific mortality by country and Healthcare Access and Quality (HAQ) Index, with the Survival Potential Frontier (SPF) (black line) and uncertainty interval around the frontier (grey band). Countries are labelled in bold when their ratio to the frontier is in the top 10 percent (performing poorly relative to HAQ Index) and in italics when their ratio to the frontier is in the bottom 10 percent (performing well relative to HAQ Index). These results show the prediction of the cause-specific SPF prior to scaling to the all-cause frontier.

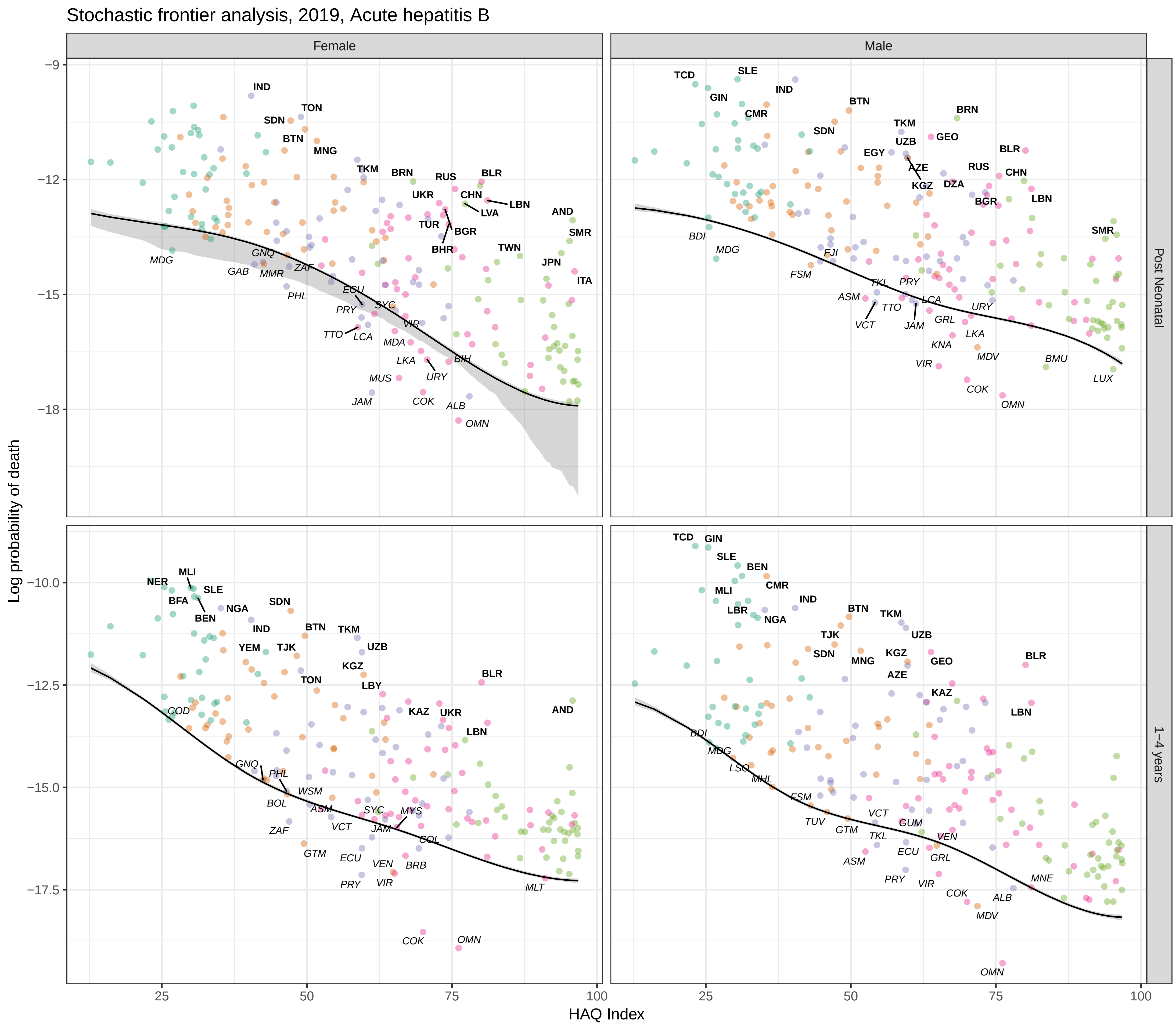

Figure S8
Cause- and age-specific mortality by country and Healthcare Access and Quality (HAQ) Index, with the Survival Potential Frontier (SPF) (black line) and uncertainty interval around the frontier (grey band). Countries are labelled in bold when their ratio to the frontier is in the top 10 percent (performing poorly relative to HAQ Index) and in italics when their ratio to the frontier is in the bottom 10 percent (performing well relative to HAQ Index). These results show the prediction of the cause-specific SPF prior to scaling to the all-cause frontier.

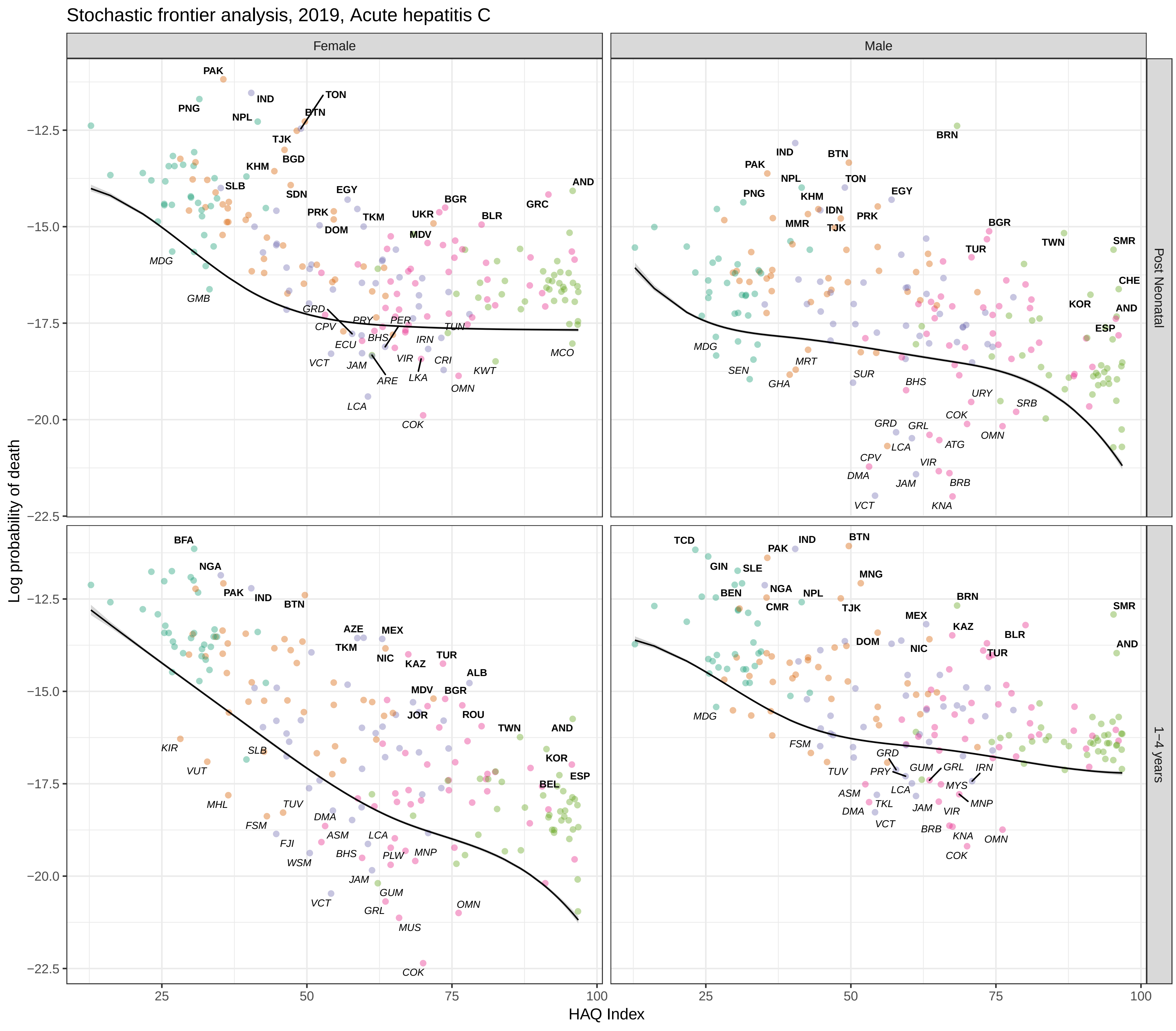

Figure S8
Cause- and age-specific mortality by country and Healthcare Access and Quality (HAQ) Index, with the Survival Potential Frontier (SPF) (black line) and uncertainty interval around the frontier (grey band). Countries are labelled in bold when their ratio to the frontier is in the top 10 percent (performing poorly relative to HAQ Index) and in italics when their ratio to the frontier is in the bottom 10 percent (performing well relative to HAQ Index). These results show the prediction of the cause-specific SPF prior to scaling to the all-cause frontier.

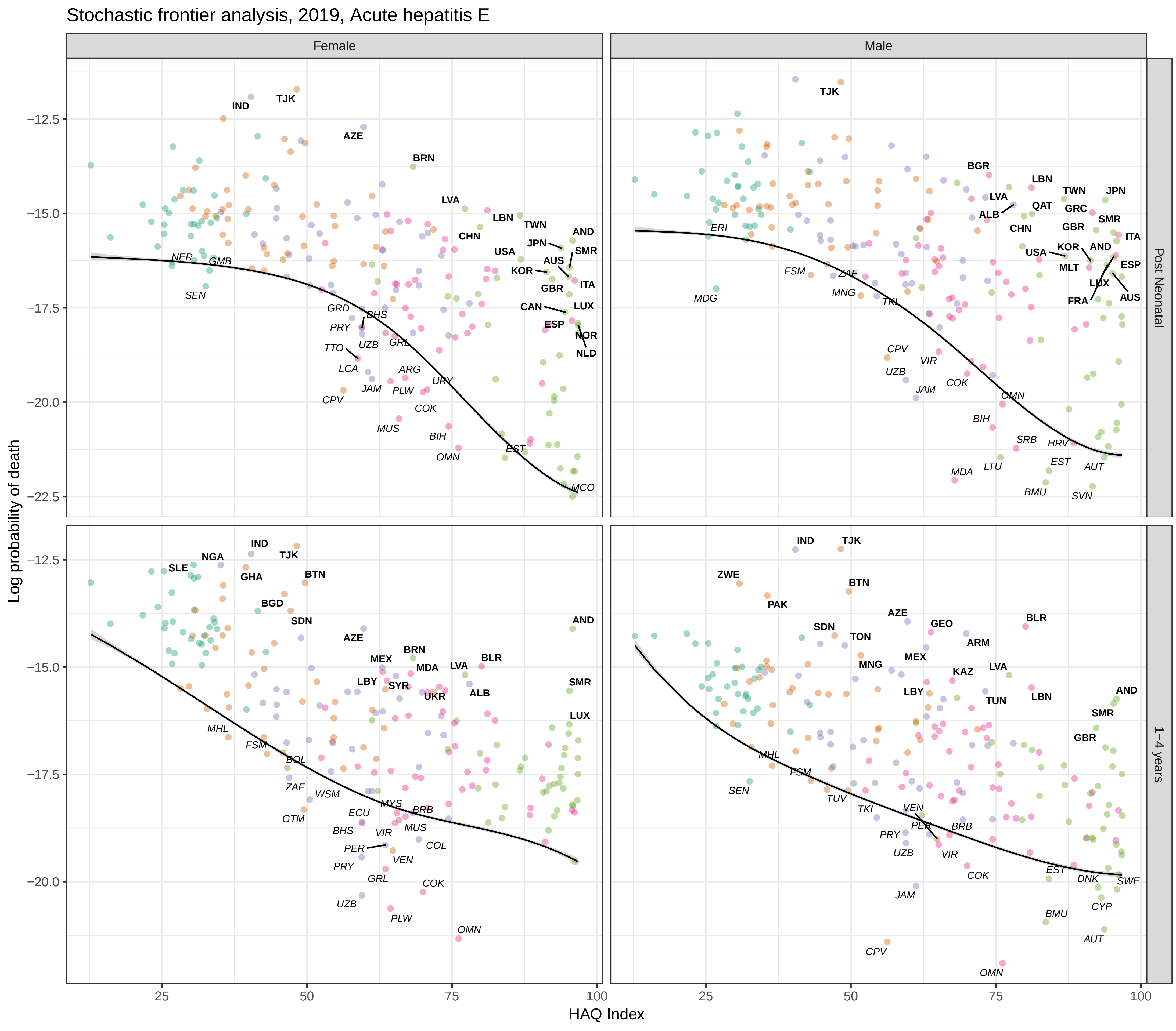

Figure S8
Cause- and age-specific mortality by country and Healthcare Access and Quality (HAQ) Index, with the Survival Potential Frontier (SPF) (black line) and uncertainty interval around the frontier (grey band). Countries are labelled in bold when their ratio to the frontier is in the top 10 percent (performing poorly relative to HAQ Index) and in italics when their ratio to the frontier is in the bottom 10 percent (performing well relative to HAQ Index). These results show the prediction of the cause-specific SPF prior to scaling to the all-cause frontier.

SDI quintile

- Low SDI
- Low−middle SDI
- Middle SDI
- High−middle SDI
- High SDI

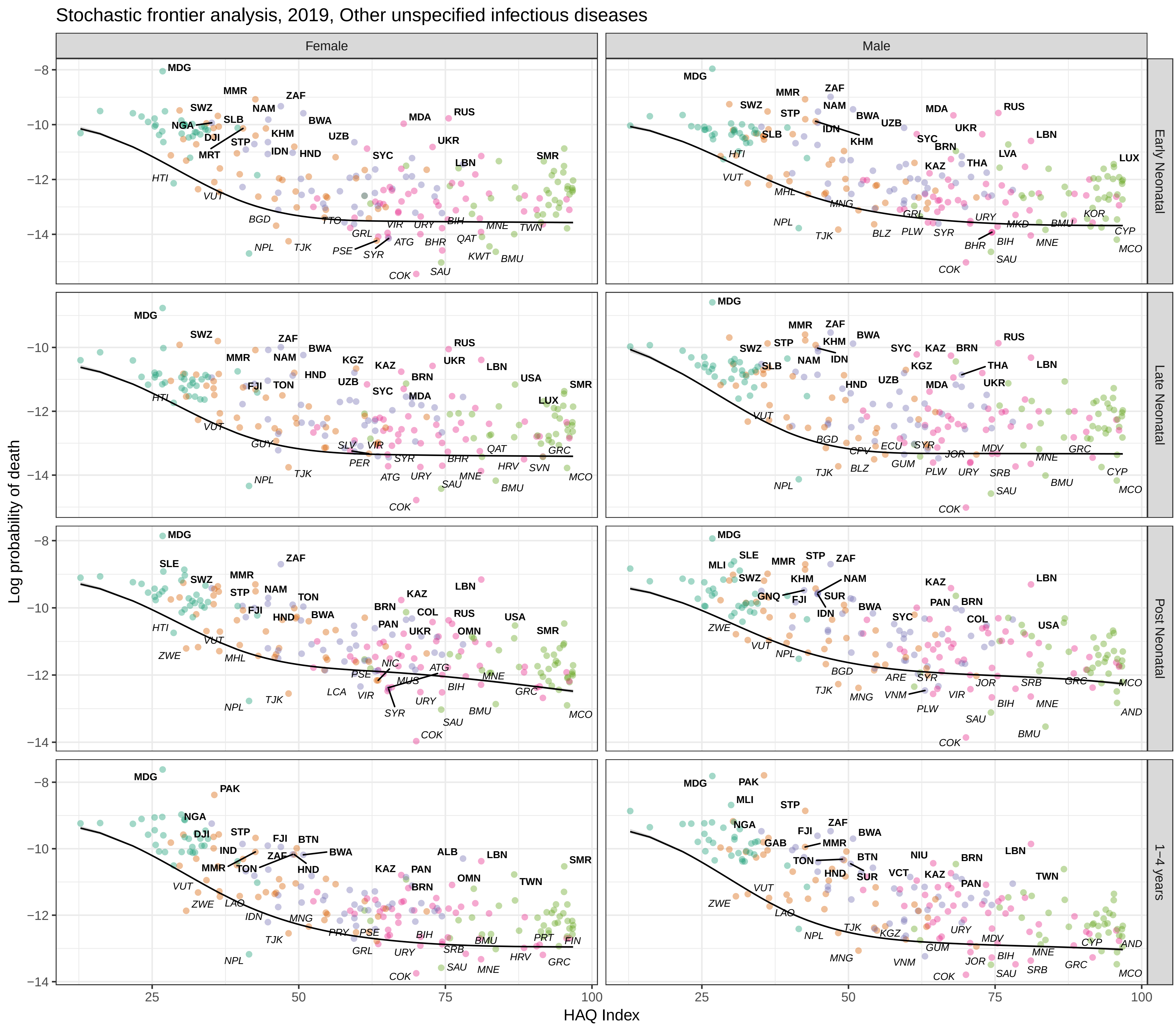

Figure S8
Cause- and age-specific mortality by country and Healthcare Access and Quality (HAQ) Index, with the Survival Potential Frontier (SPF) (black line) and uncertainty interval around the frontier (grey band). Countries are labelled in bold when their ratio to the frontier is in the top 10 percent (performing poorly relative to HAQ Index) and in italics when their ratio to the frontier is in the bottom 10 percent (performing well relative to HAQ Index). These results show the prediction of the cause-specific SPF prior to scaling to the all-cause frontier.

SDI quintile
- Low SDI
- Low−middle SDI
- Middle SDI
- High−middle SDI
- High SDI

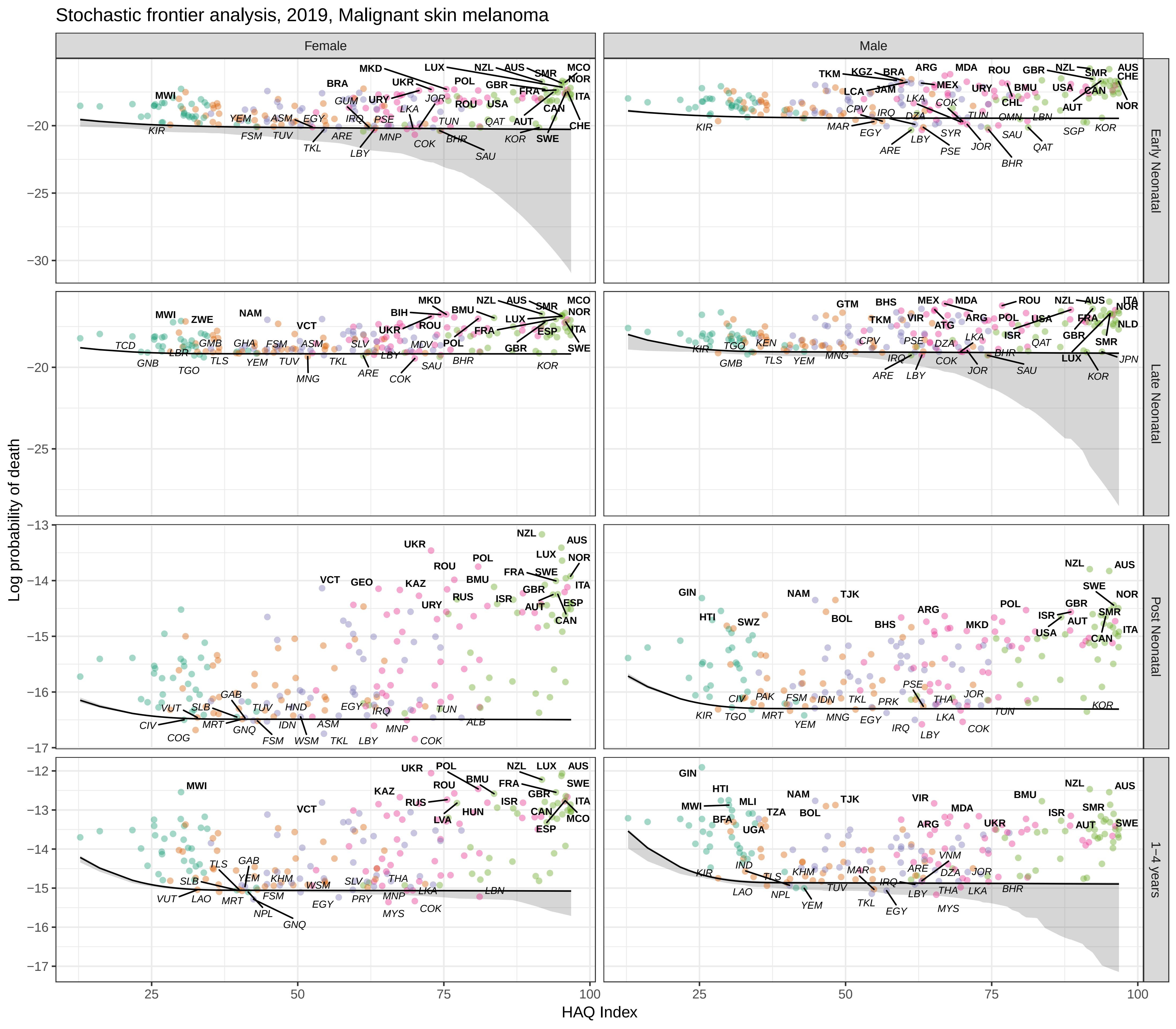

Figure S8
Cause- and age-specific mortality by country and Healthcare Access and Quality (HAQ) Index, with the Survival Potential Frontier (SPF) (black line) and uncertainty interval around the frontier (grey band). Countries are labelled in bold when their ratio to the frontier is in the top 10 percent (performing poorly relative to HAQ Index) and in italics when their ratio to the frontier is in the bottom 10 percent (performing well relative to HAQ Index). These results show the prediction of the cause-specific SPF prior to scaling to the all-cause frontier.

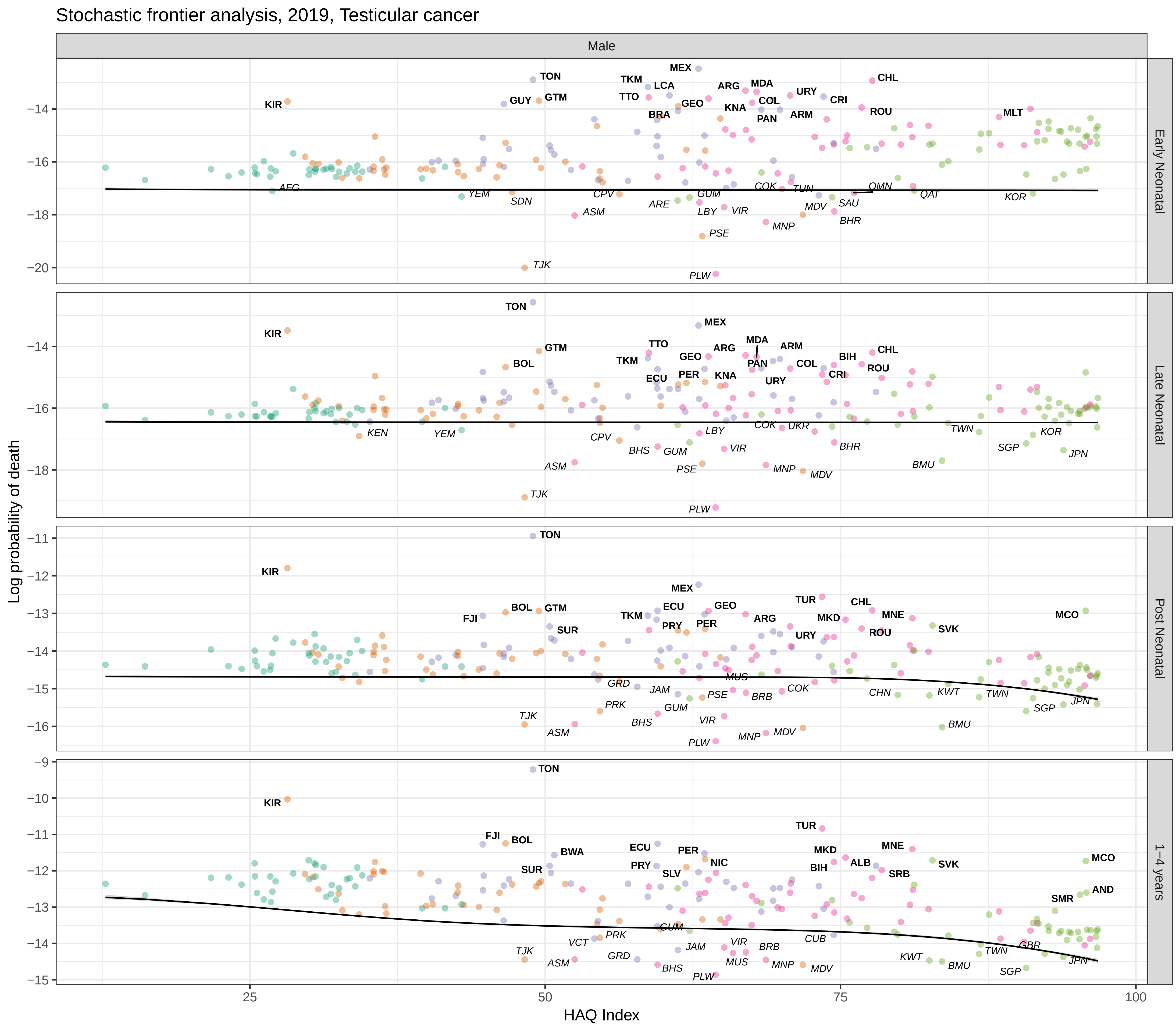

Figure S8
Cause- and age-specific mortality by country and Healthcare Access and Quality (HAQ) Index, with the Survival Potential Frontier (SPF) (black line) and uncertainty interval around the frontier (grey band). Countries are labelled in bold when their ratio to the frontier is in the top 10 percent (performing poorly relative to HAQ Index) and in italics when their ratio to the frontier is in the bottom 10 percent (performing well relative to HAQ Index). These results show the prediction of the cause-specific SPF prior to scaling to the all-cause frontier.

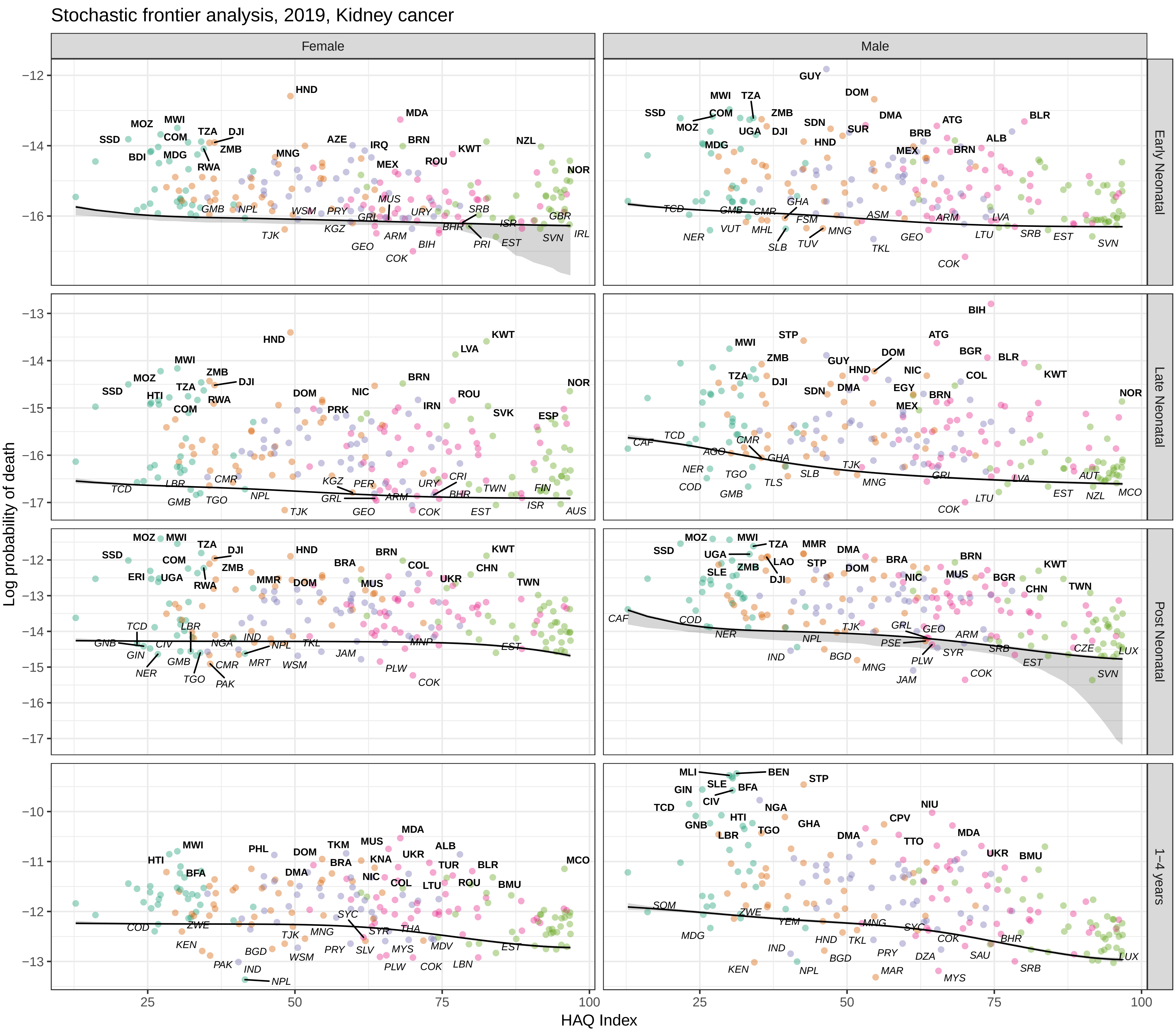

Figure S8
Cause- and age-specific mortality by country and Healthcare Access and Quality (HAQ) Index, with the Survival Potential Frontier (SPF) (black line) and uncertainty interval around the frontier (grey band). Countries are labelled in bold when their ratio to the frontier is in the top 10 percent (performing poorly relative to HAQ Index) and in italics when their ratio to the frontier is in the bottom 10 percent (performing well relative to HAQ Index). These results show the prediction of the cause-specific SPF prior to scaling to the all-cause frontier.

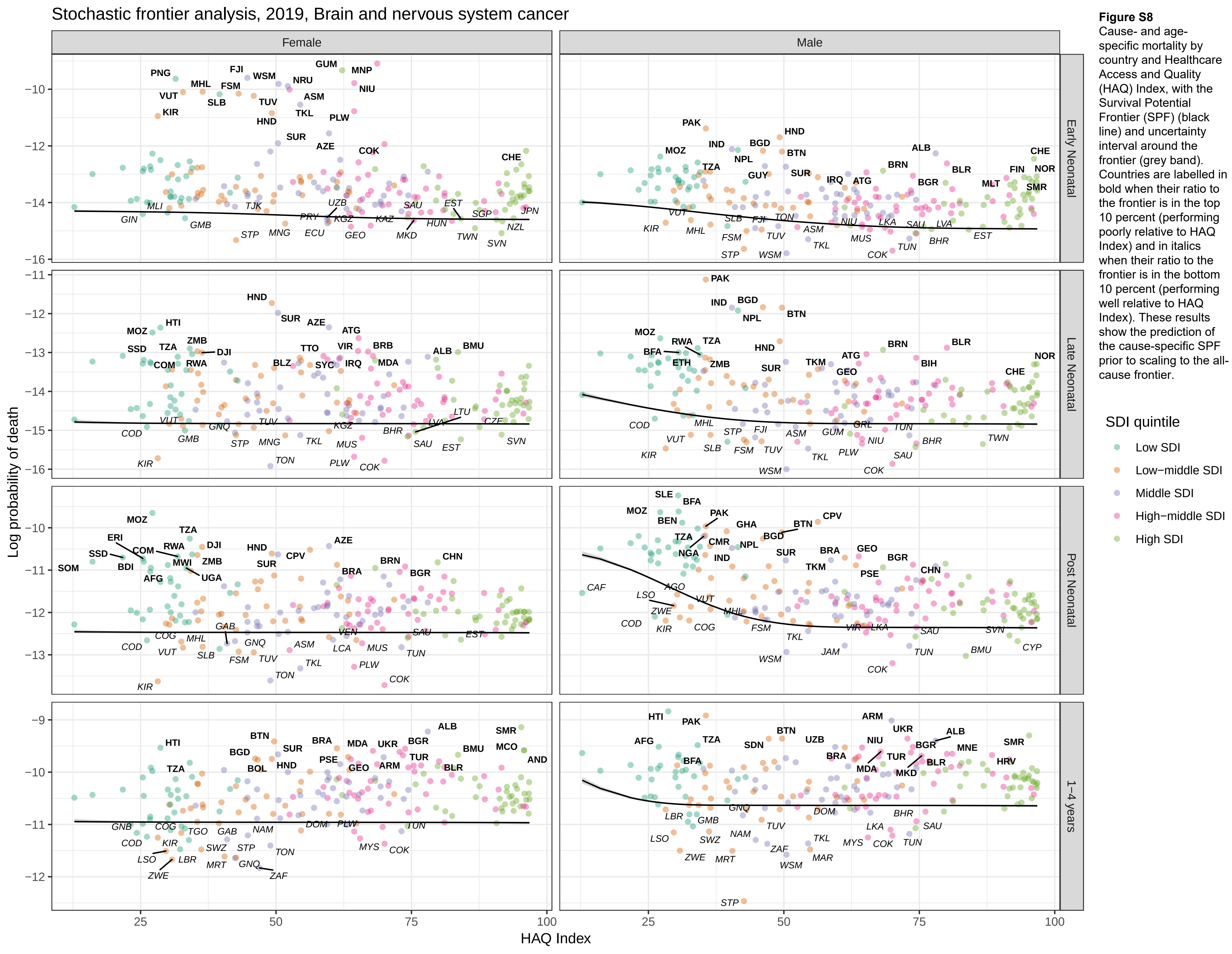

Figure S8
Cause- and age-specific mortality by country and Healthcare Access and Quality (HAQ) Index, with the Survival Potential Frontier (SPF) (black line) and uncertainty interval around the frontier (grey band). Countries are labelled in bold when their ratio to the frontier is in the top 10 percent (performing poorly relative to HAQ Index) and in italics when their ratio to the frontier is in the bottom 10 percent (performing well relative to HAQ Index). These results show the prediction of the cause-specific SPF prior to scaling to the all-cause frontier.

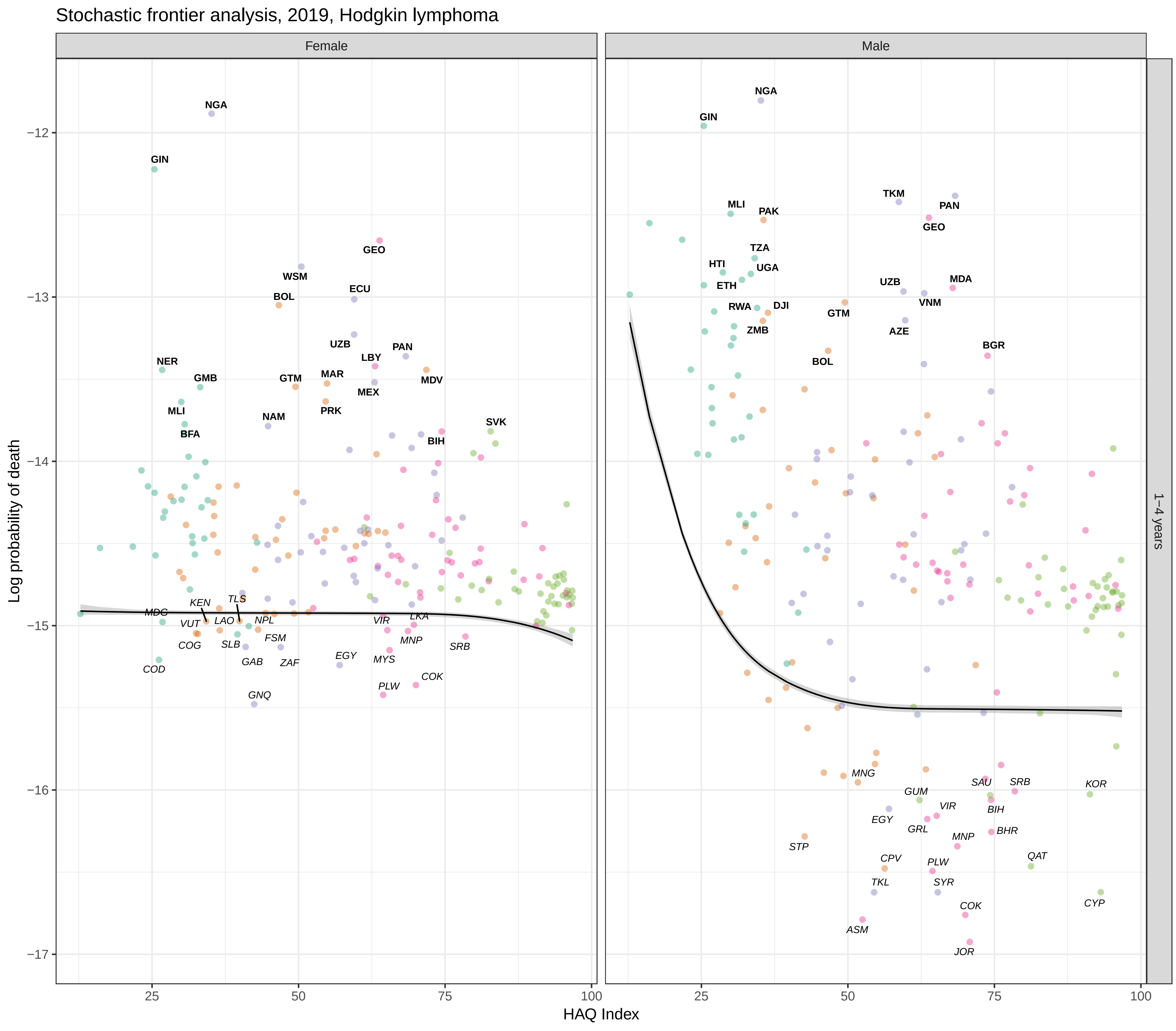

Figure S8
Cause- and age-specific mortality by country and Healthcare Access and Quality (HAQ) Index, with the Survival Potential Frontier (SPF) (black line) and uncertainty interval around the frontier (grey band). Countries are labelled in bold when their ratio to the frontier is in the top 10 percent (performing poorly relative to HAQ Index) and in italics when their ratio to the frontier is in the bottom 10 percent (performing well relative to HAQ Index). These results show the prediction of the cause-specific SPF prior to scaling to the all-cause frontier.

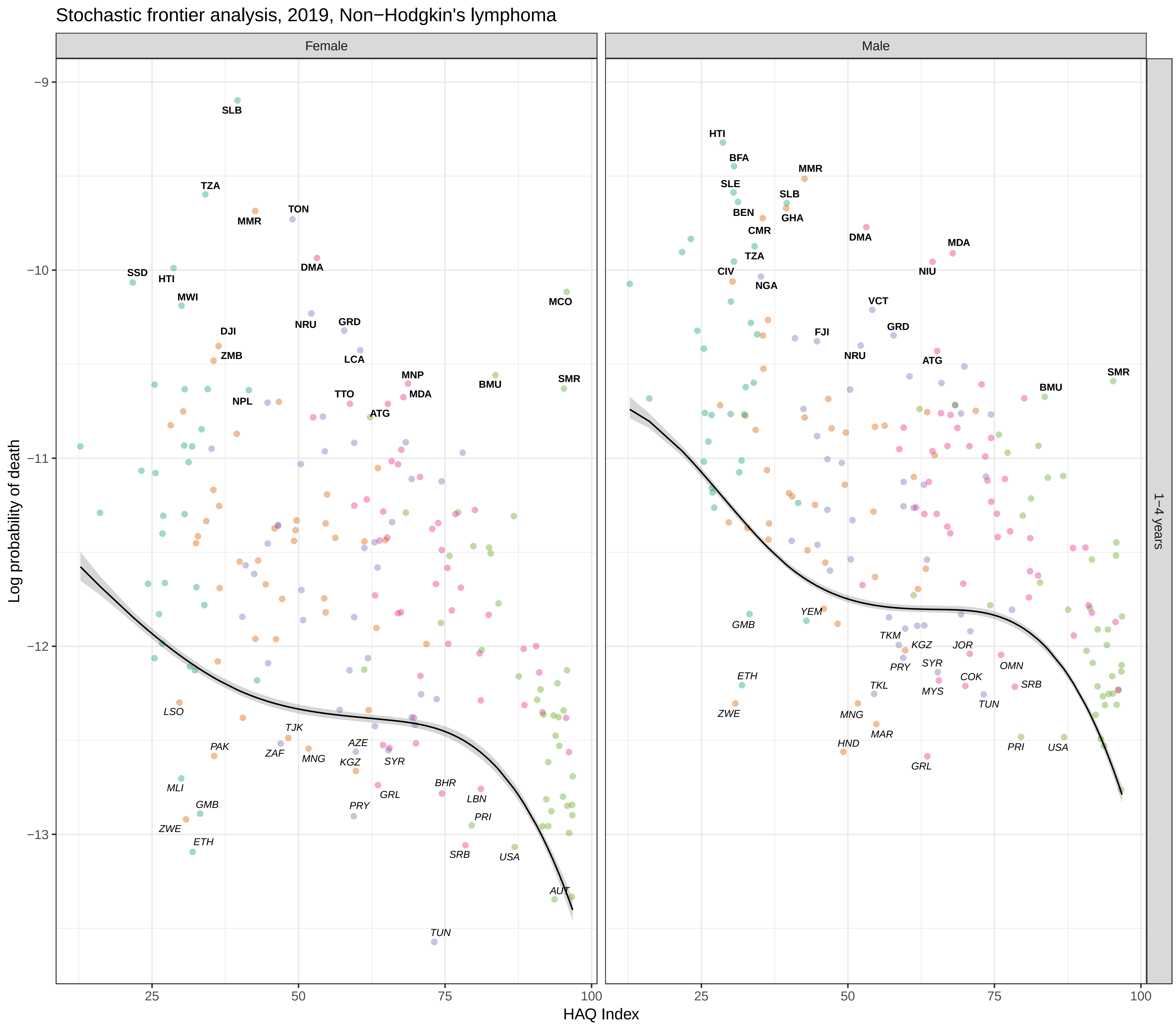

Figure S8
Cause- and age-specific mortality by country and Healthcare Access and Quality (HAQ) Index, with the Survival Potential Frontier (SPF) (black line) and uncertainty interval around the frontier (grey band). Countries are labelled in bold when their ratio to the frontier is in the top 10 percent (performing poorly relative to HAQ Index) and in italics when their ratio to the frontier is in the bottom 10 percent (performing well relative to HAQ Index). These results show the prediction of the cause-specific SPF prior to scaling to the all-cause frontier.

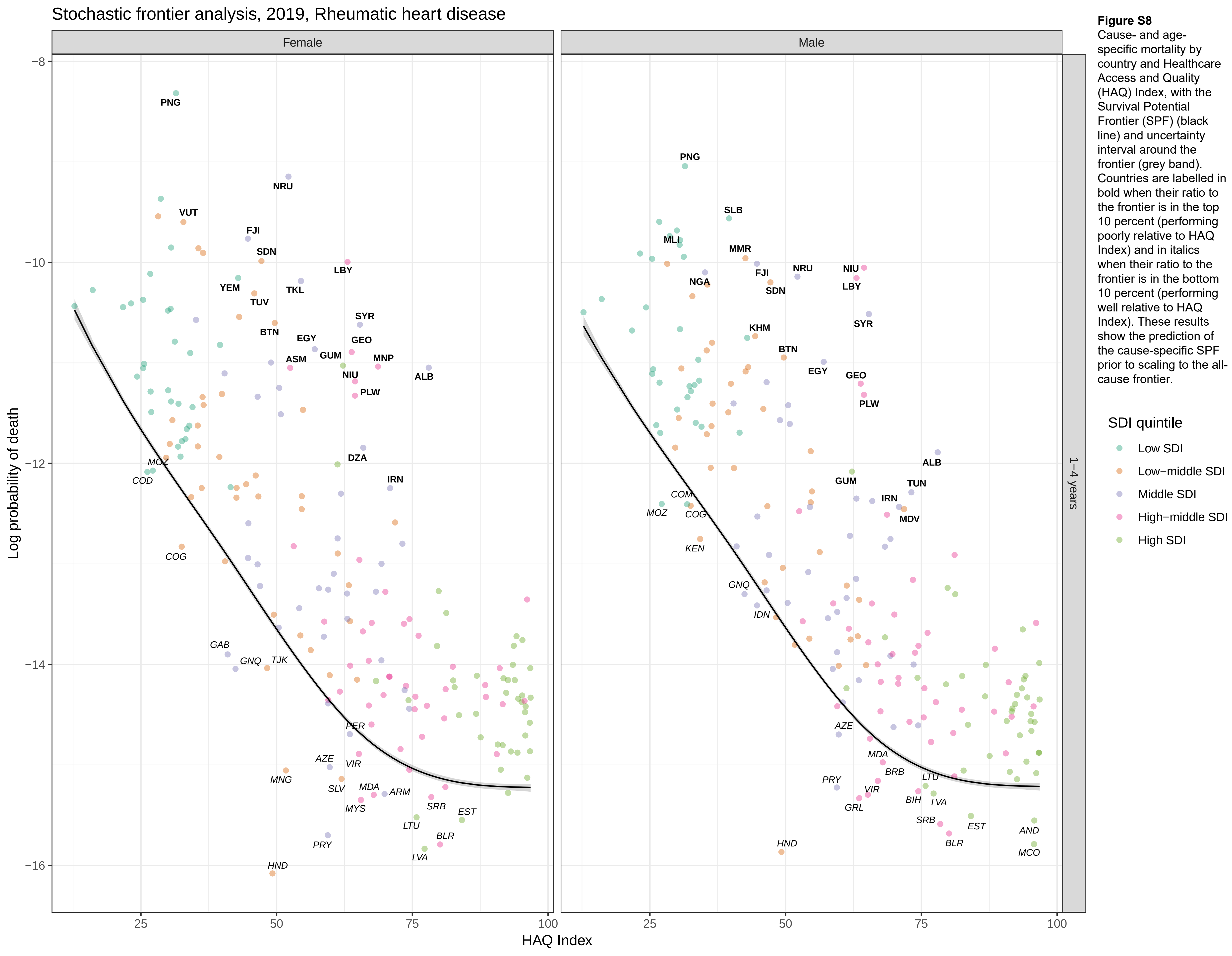

Figure S8
Cause- and age-specific mortality by country and Healthcare Access and Quality (HAQ) Index, with the Survival Potential Frontier (SPF) (black line) and uncertainty interval around the frontier (grey band). Countries are labelled in bold when their ratio to the frontier is in the top 10 percent (performing poorly relative to HAQ Index) and in italics when their ratio to the frontier is in the bottom 10 percent (performing well relative to HAQ Index). These results show the prediction of the cause-specific SPF prior to scaling to the all-cause frontier.

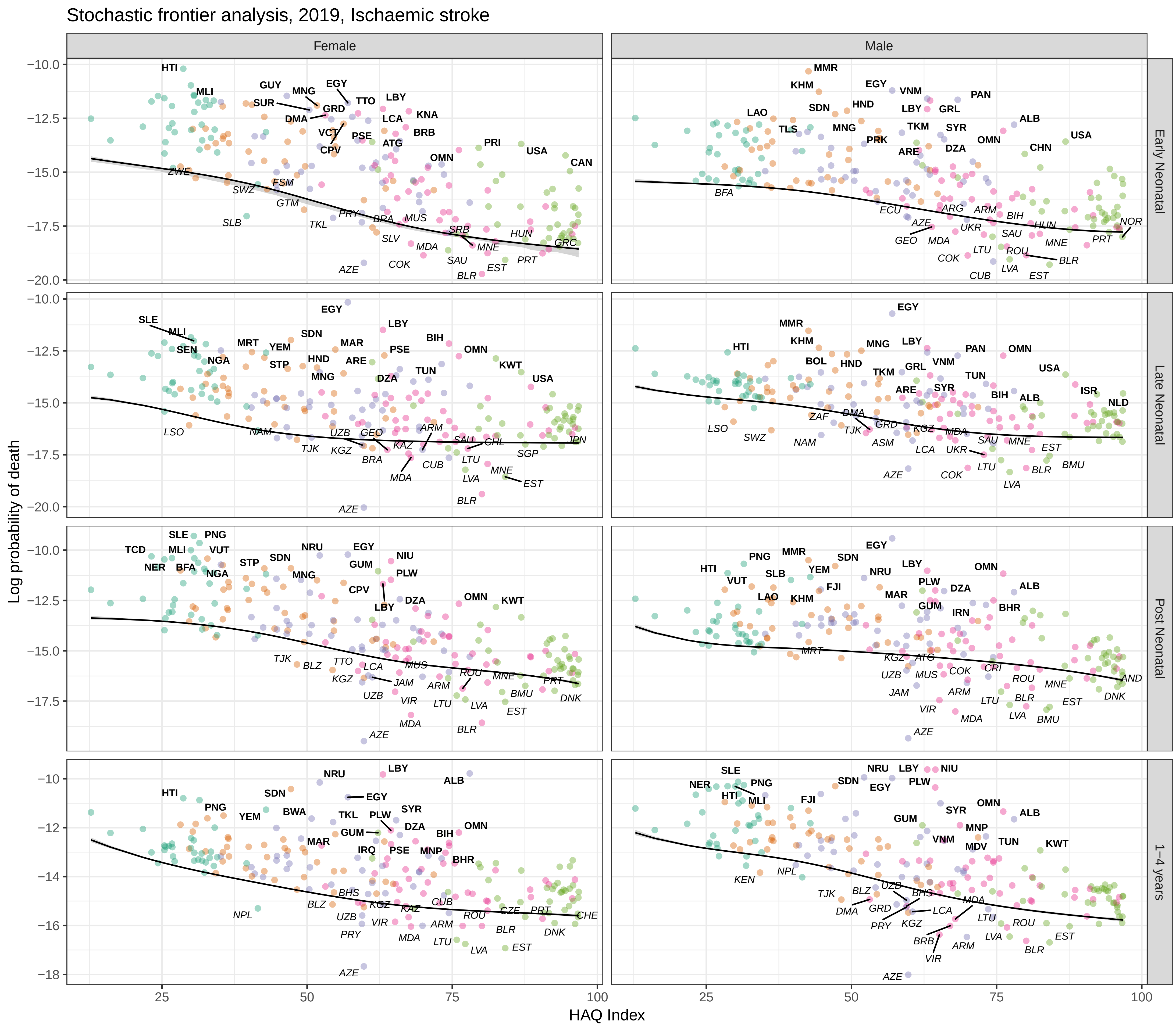

Figure S8
Cause- and age-specific mortality by country and Healthcare Access and Quality (HAQ) Index, with the Survival Potential Frontier (SPF) (black line) and uncertainty interval around the frontier (grey band). Countries are labelled in bold when their ratio to the frontier is in the top 10 percent (performing poorly relative to HAQ Index) and in italics when their ratio to the frontier is in the bottom 10 percent (performing well relative to HAQ Index). These results show the prediction of the cause-specific SPF prior to scaling to the all-cause frontier.

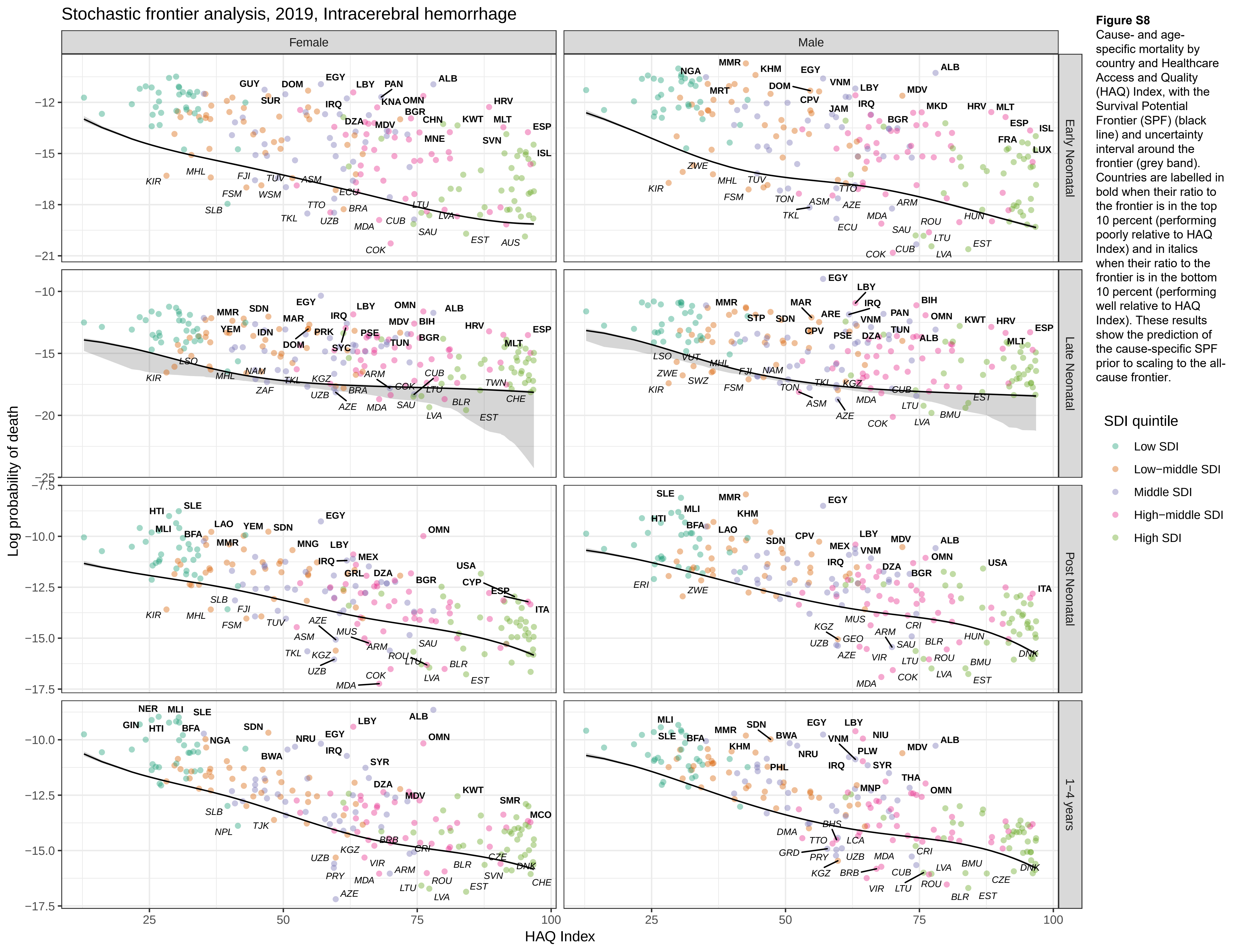

Figure S8
Cause- and age-specific mortality by country and Healthcare Access and Quality (HAQ) Index, with the Survival Potential Frontier (SPF) (black line) and uncertainty interval around the frontier (grey band). Countries are labelled in bold when their ratio to the frontier is in the top 10 percent (performing poorly relative to HAQ Index) and in italics when their ratio to the frontier is in the bottom 10 percent (performing well relative to HAQ Index). These results show the prediction of the cause-specific SPF prior to scaling to the all-cause frontier.

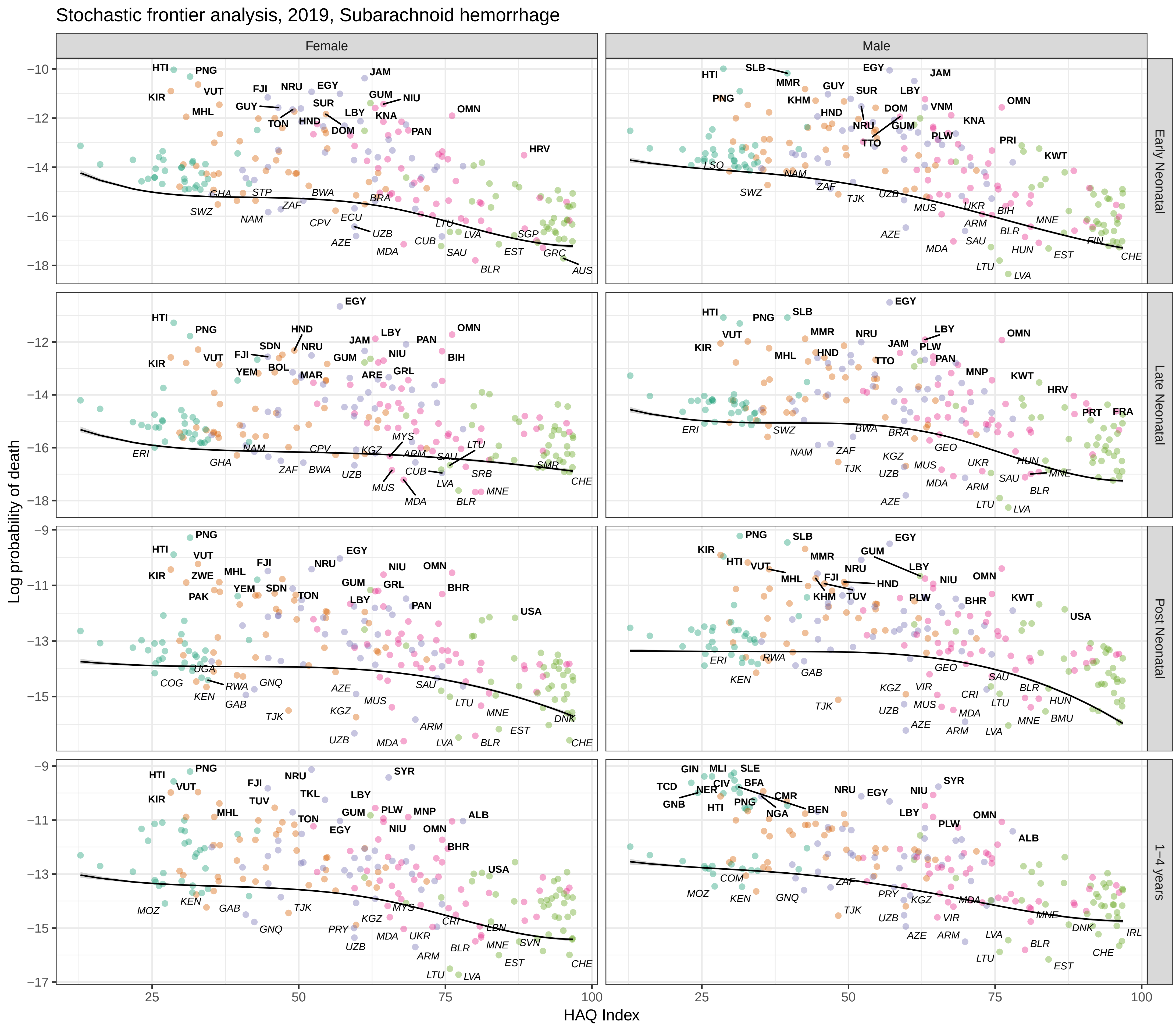

Figure S8
Cause- and age-specific mortality by country and Healthcare Access and Quality (HAQ) Index, with the Survival Potential Frontier (SPF) (black line) and uncertainty interval around the frontier (grey band). Countries are labelled in bold when their ratio to the frontier is in the top 10 percent (performing poorly relative to HAQ Index) and in italics when their ratio to the frontier is in the bottom 10 percent (performing well relative to HAQ Index). These results show the prediction of the cause-specific SPF prior to scaling to the all-cause frontier.

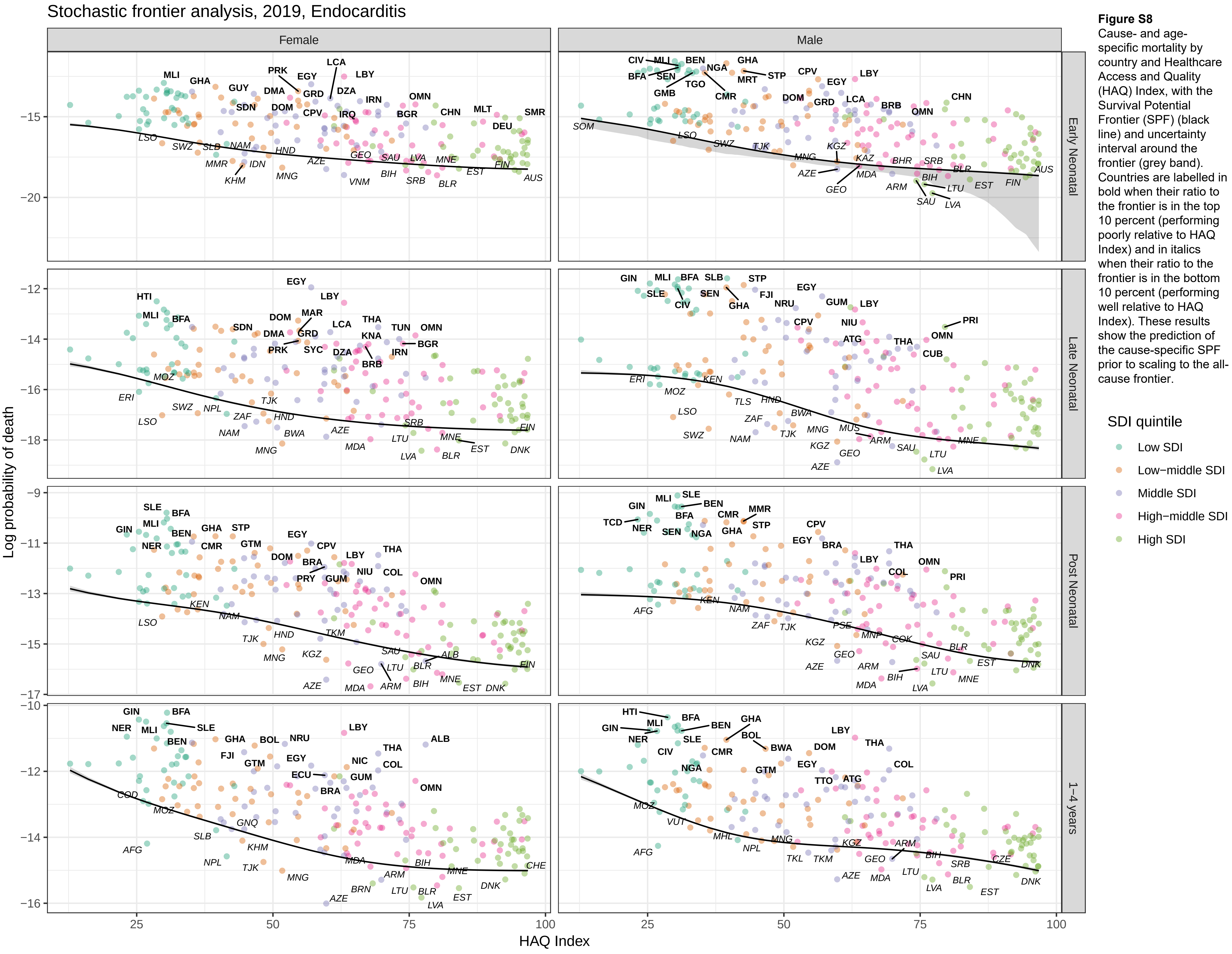

Figure S8
Cause- and age-specific mortality by country and Healthcare Access and Quality (HAQ) Index, with the Survival Potential Frontier (SPF) (black line) and uncertainty interval around the frontier (grey band). Countries are labelled in bold when their ratio to the frontier is in the top 10 percent (performing poorly relative to HAQ Index) and in italics when their ratio to the frontier is in the bottom 10 percent (performing well relative to HAQ Index). These results show the prediction of the cause-specific SPF prior to scaling to the all-cause frontier.

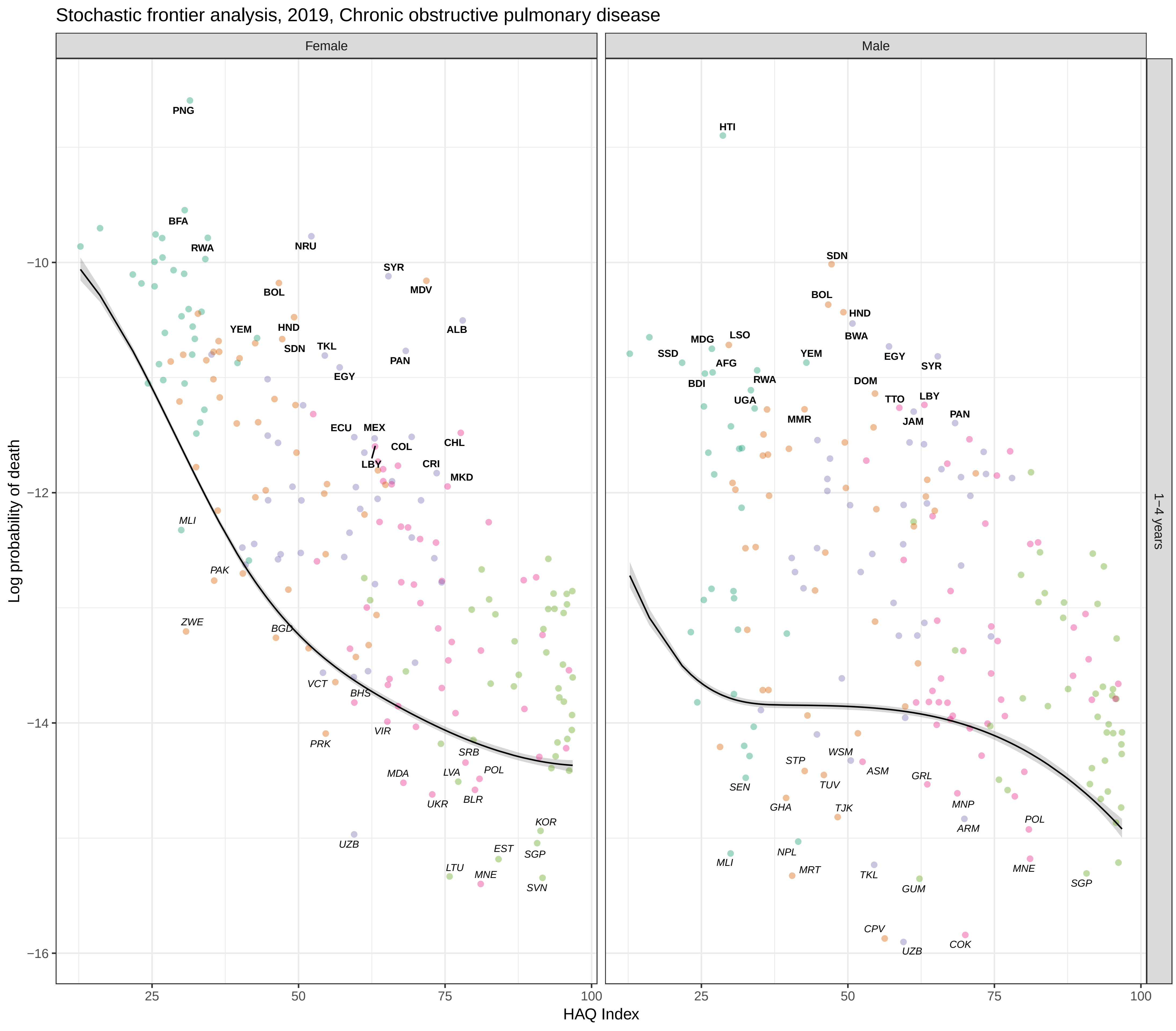

Figure S8
Cause- and age-specific mortality by country and Healthcare Access and Quality (HAQ) Index, with the Survival Potential Frontier (SPF) (black line) and uncertainty interval around the frontier (grey band). Countries are labelled in bold when their ratio to the frontier is in the top 10 percent (performing poorly relative to HAQ Index) and in italics when their ratio to the frontier is in the bottom 10 percent (performing well relative to HAQ Index). These results show the prediction of the cause-specific SPF prior to scaling to the all-cause frontier.

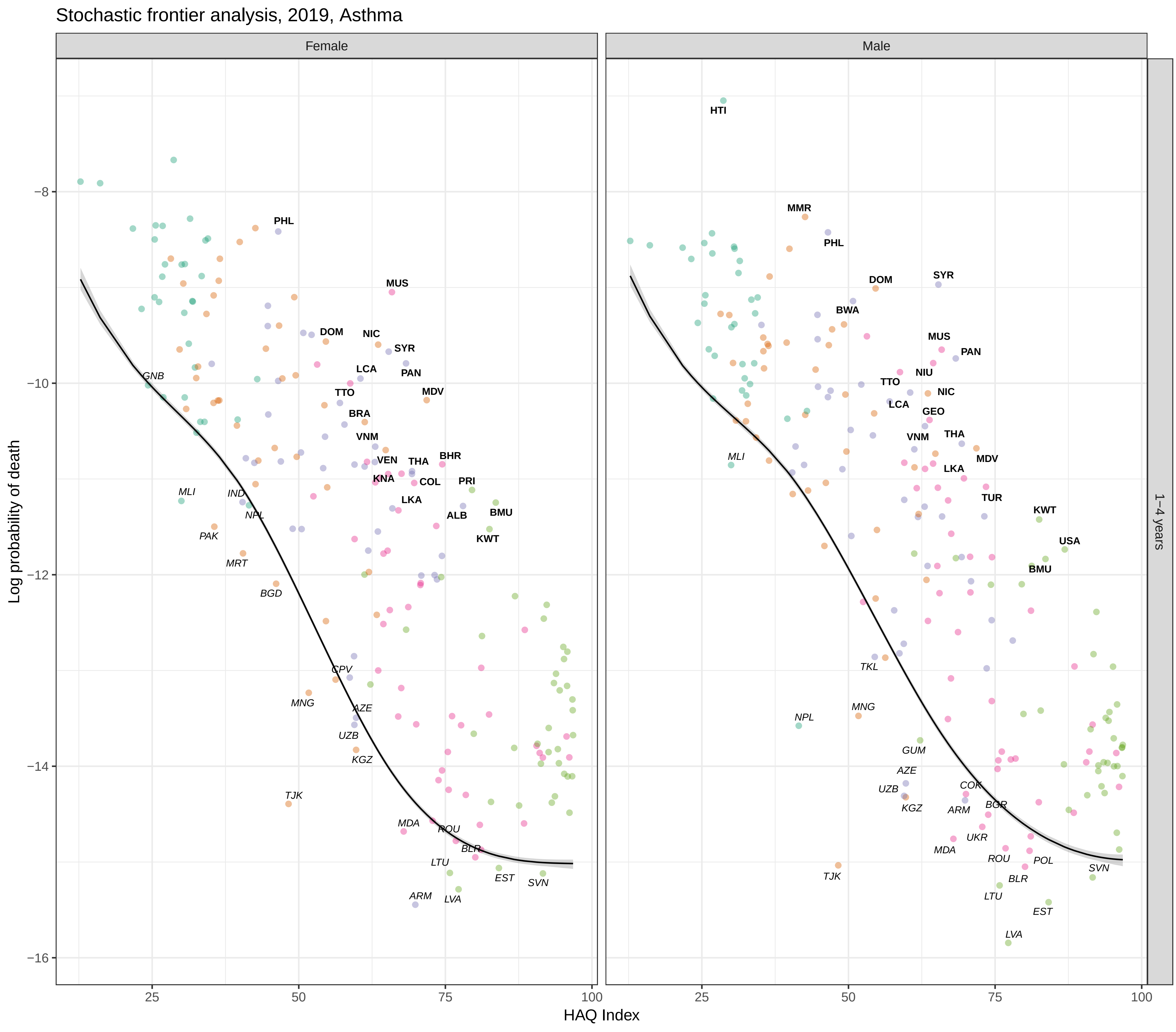

Figure S8
Cause- and age-specific mortality by country and Healthcare Access and Quality (HAQ) Index, with the Survival Potential Frontier (SPF) (black line) and uncertainty interval around the frontier (grey band). Countries are labelled in bold when their ratio to the frontier is in the top 10 percent (performing poorly relative to HAQ Index) and in italics when their ratio to the frontier is in the bottom 10 percent (performing well relative to HAQ Index). These results show the prediction of the cause-specific SPF prior to scaling to the all-cause frontier.

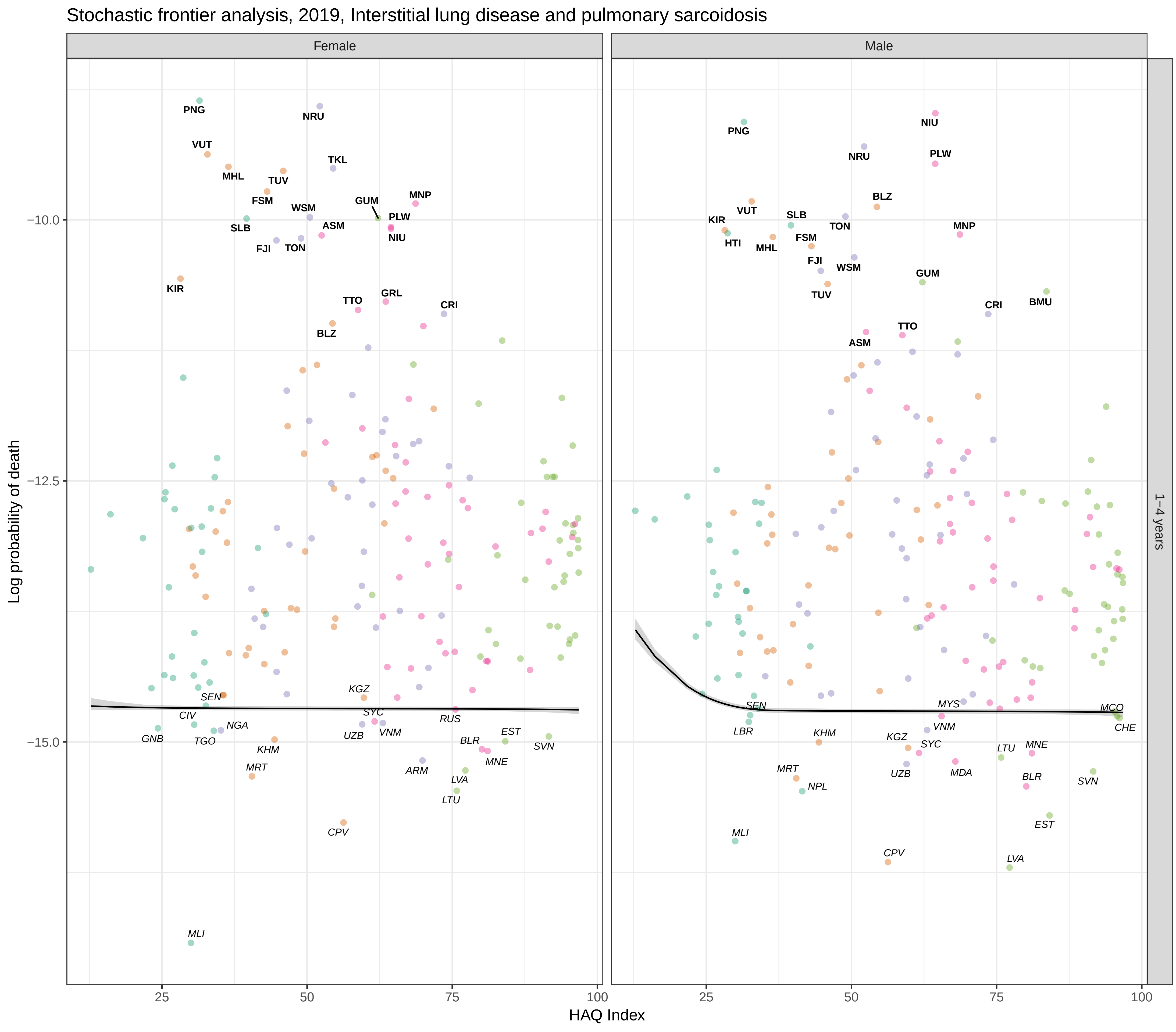

Figure S8
Cause- and age-specific mortality by country and Healthcare Access and Quality (HAQ) Index, with the Survival Potential Frontier (SPF) (black line) and uncertainty interval around the frontier (grey band). Countries are labelled in bold when their ratio to the frontier is in the top 10 percent (performing poorly relative to HAQ Index) and in italics when their ratio to the frontier is in the bottom 10 percent (performing well relative to HAQ Index). These results show the prediction of the cause-specific SPF prior to scaling to the all-cause frontier.

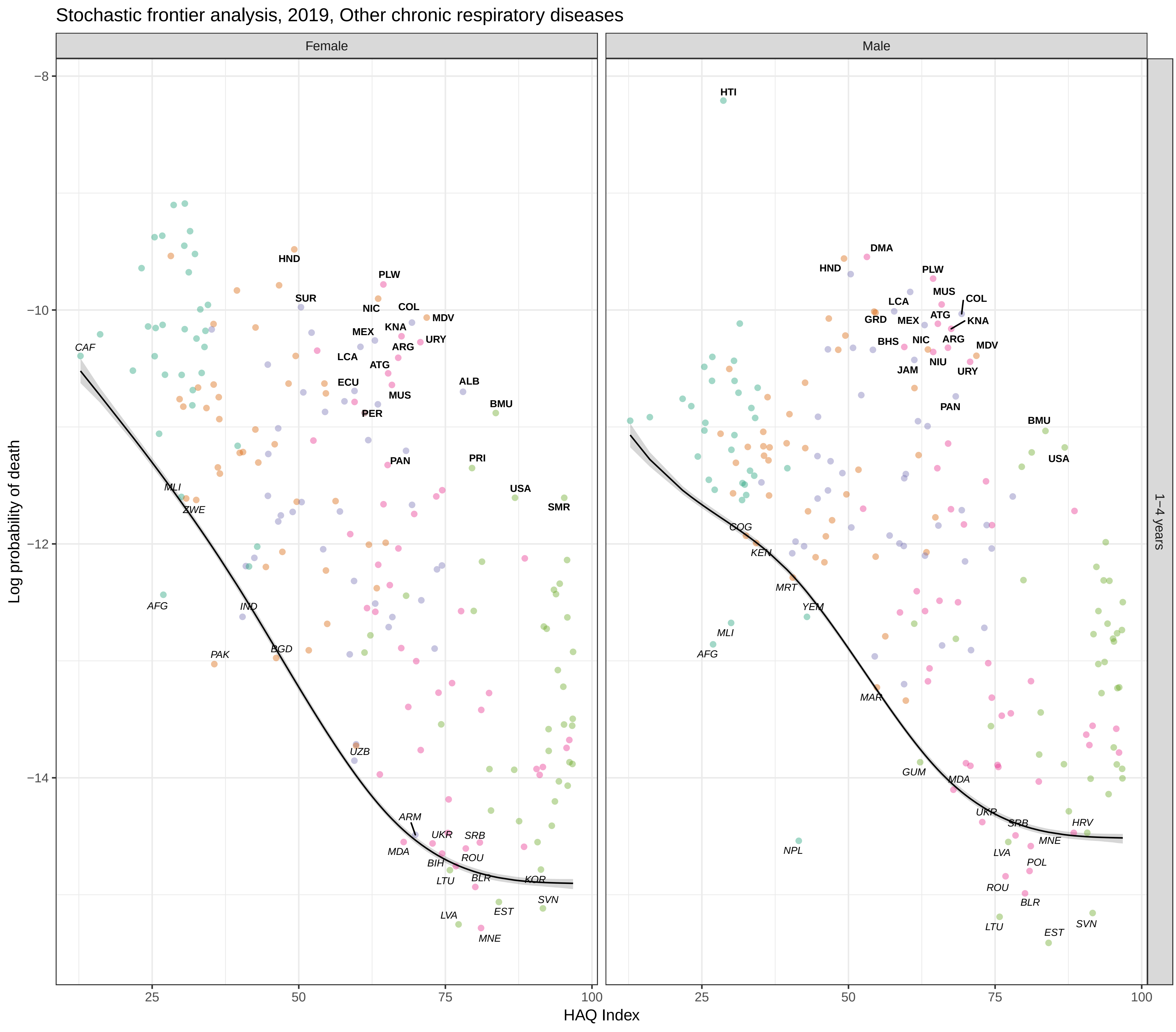

Figure S8
Cause- and age-specific mortality by country and Healthcare Access and Quality (HAQ) Index, with the Survival Potential Frontier (SPF) (black line) and uncertainty interval around the frontier (grey band). Countries are labelled in bold when their ratio to the frontier is in the top 10 percent (performing poorly relative to HAQ Index) and in italics when their ratio to the frontier is in the bottom 10 percent (performing well relative to HAQ Index). These results show the prediction of the cause-specific SPF prior to scaling to the all-cause frontier.

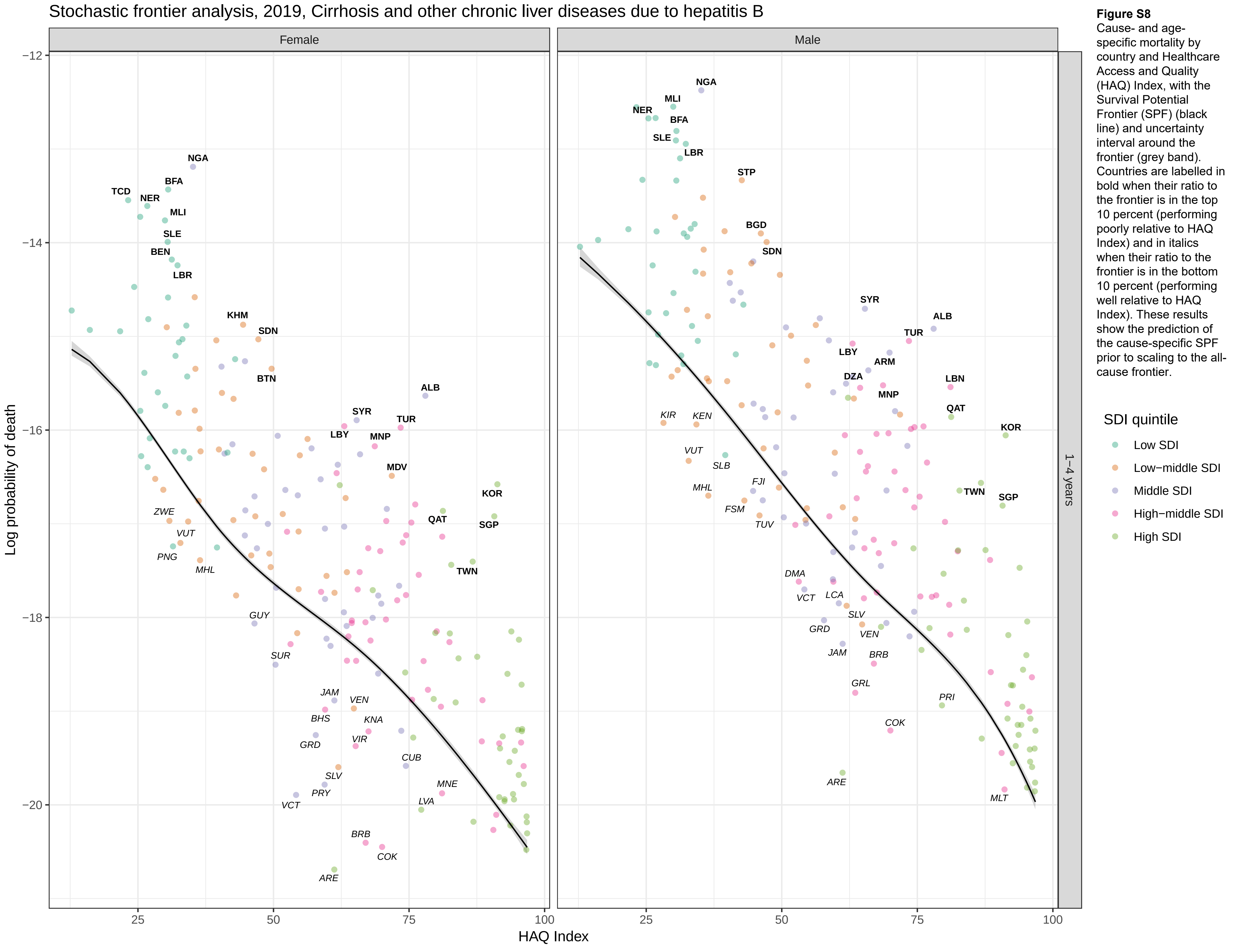

Figure S8
Cause- and age-specific mortality by country and Healthcare Access and Quality (HAQ) Index, with the Survival Potential Frontier (SPF) (black line) and uncertainty interval around the frontier (grey band). Countries are labelled in bold when their ratio to the frontier is in the top 10 percent (performing poorly relative to HAQ Index) and in italics when their ratio to the frontier is in the bottom 10 percent (performing well relative to HAQ Index). These results show the prediction of the cause-specific SPF prior to scaling to the all-cause frontier.

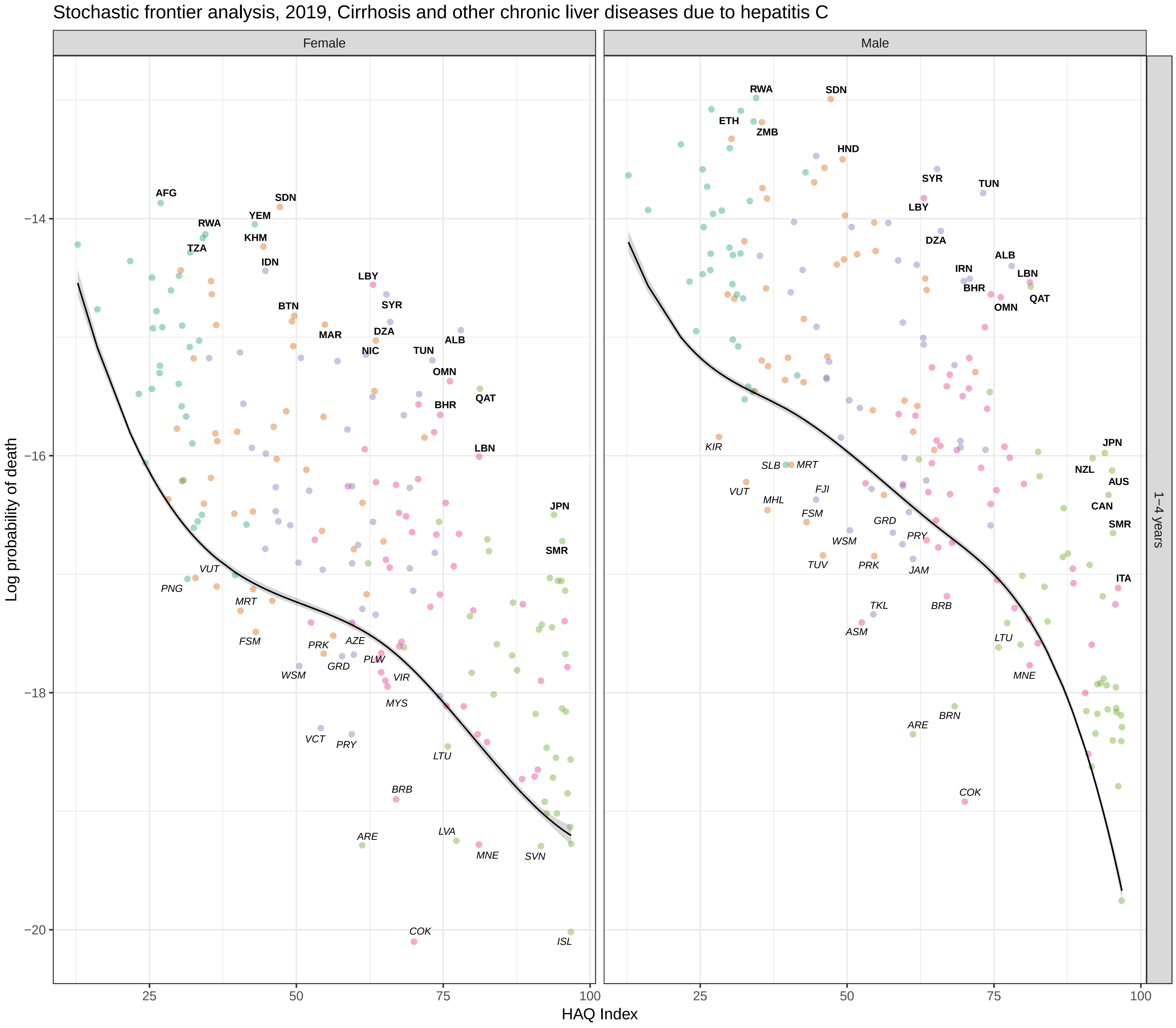

Figure S8
Cause- and age-specific mortality by country and Healthcare Access and Quality (HAQ) Index, with the Survival Potential Frontier (SPF) (black line) and uncertainty interval around the frontier (grey band). Countries are labelled in bold when their ratio to the frontier is in the top 10 percent (performing poorly relative to HAQ Index) and in italics when their ratio to the frontier is in the bottom 10 percent (performing well relative to HAQ Index). These results show the prediction of the cause-specific SPF prior to scaling to the all-cause frontier.

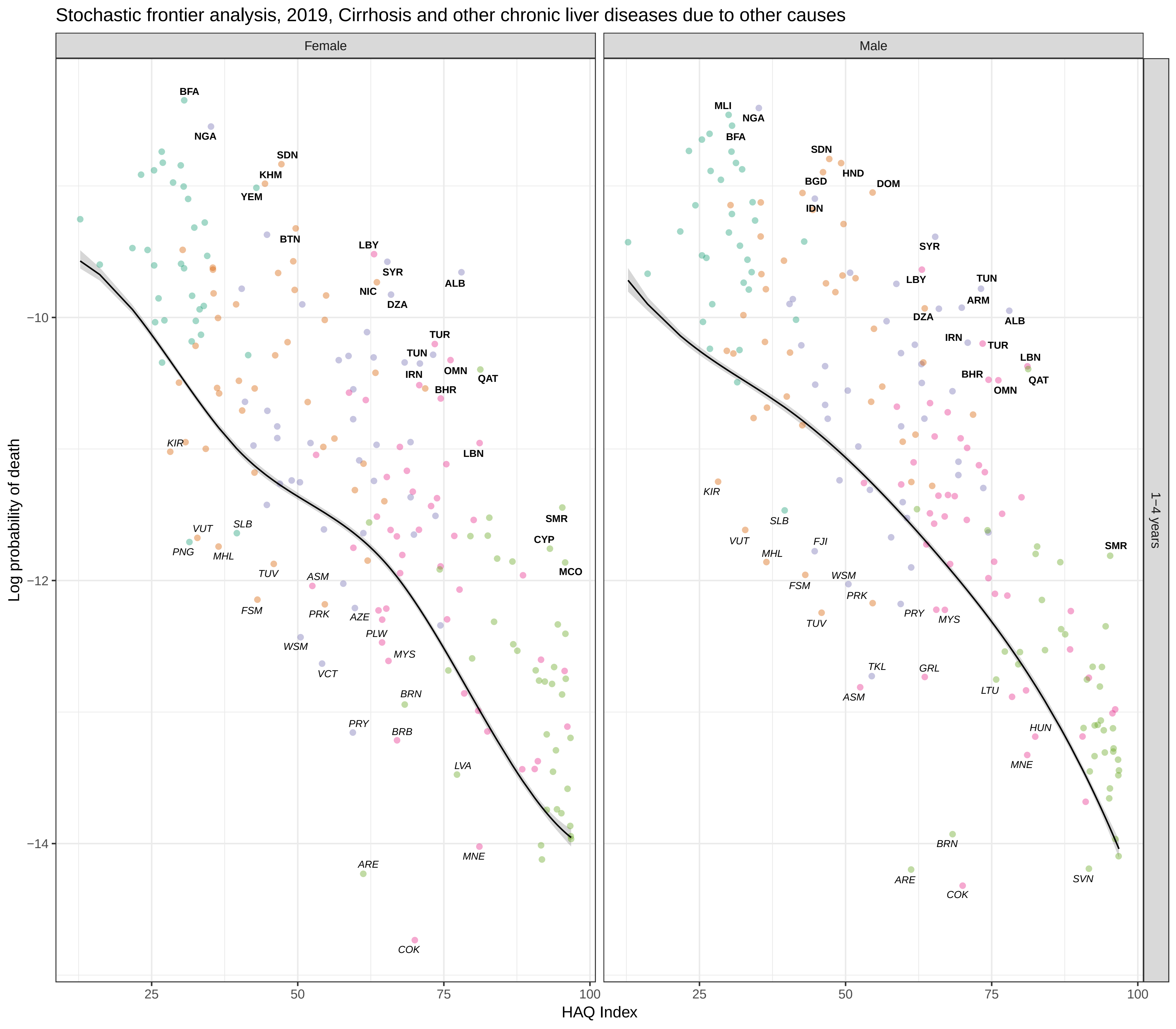

Figure S8
Cause- and age-specific mortality by country and Healthcare Access and Quality (HAQ) Index, with the Survival Potential Frontier (SPF) (black line) and uncertainty interval around the frontier (grey band). Countries are labelled in bold when their ratio to the frontier is in the top 10 percent (performing poorly relative to HAQ Index) and in italics when their ratio to the frontier is in the bottom 10 percent (performing well relative to HAQ Index). These results show the prediction of the cause-specific SPF prior to scaling to the all-cause frontier.

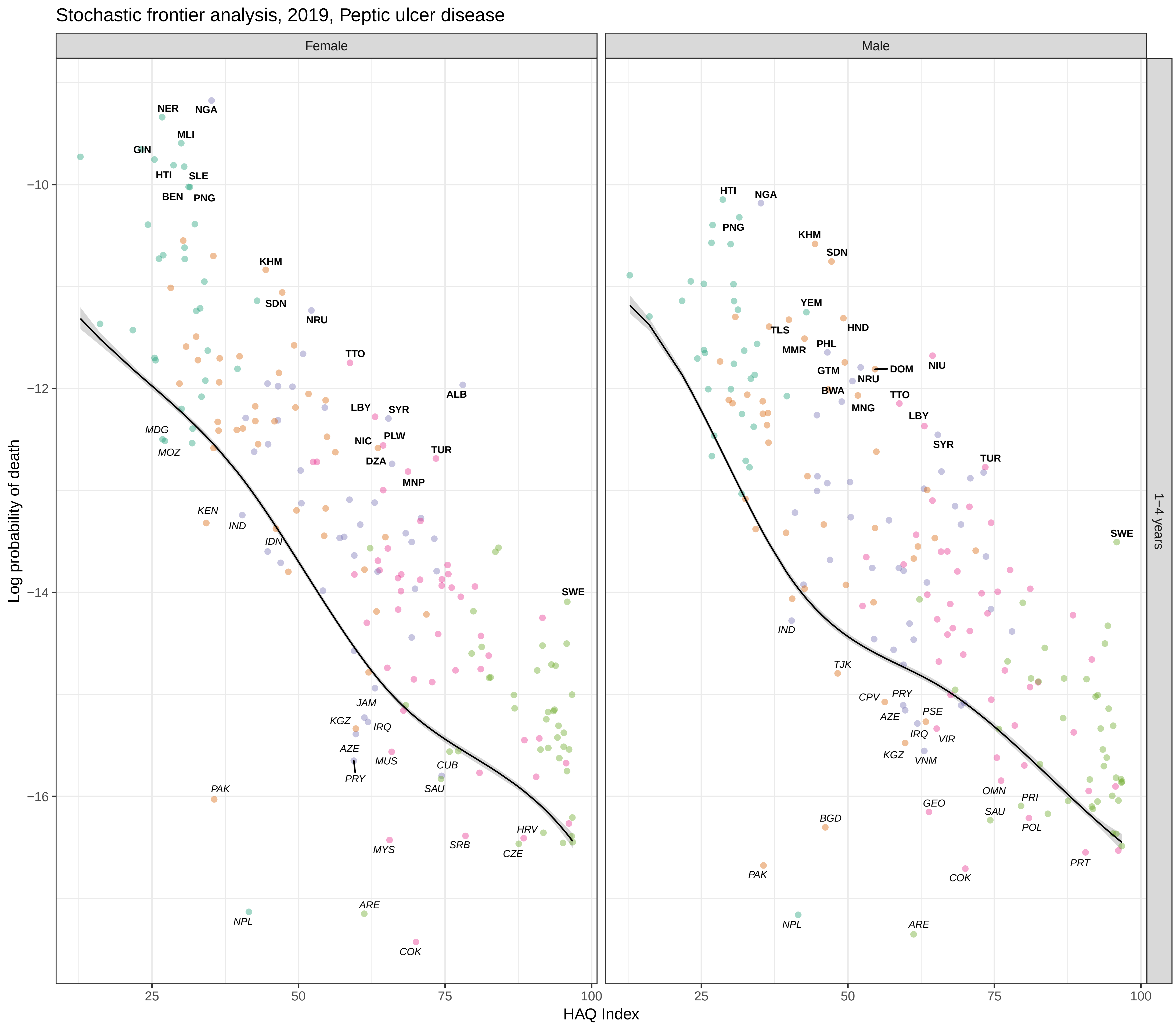

Figure S8
Cause- and age-specific mortality by country and Healthcare Access and Quality (HAQ) Index, with the Survival Potential Frontier (SPF) (black line) and uncertainty interval around the frontier (grey band). Countries are labelled in bold when their ratio to the frontier is in the top 10 percent (performing poorly relative to HAQ Index) and in italics when their ratio to the frontier is in the bottom 10 percent (performing well relative to HAQ Index). These results show the prediction of the cause-specific SPF prior to scaling to the all-cause frontier.

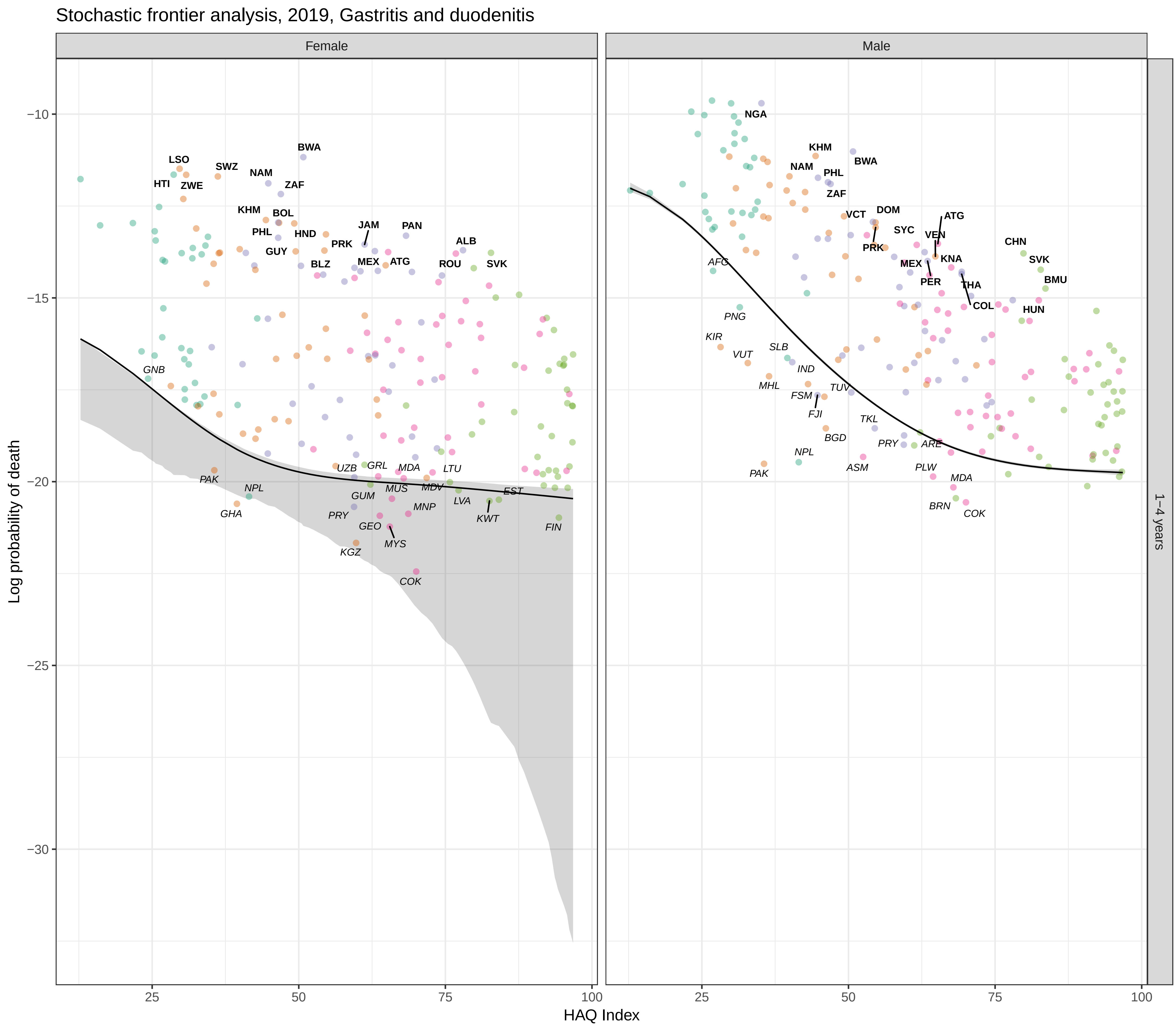

Figure S8
Cause- and age-specific mortality by country and Healthcare Access and Quality (HAQ) Index, with the Survival Potential Frontier (SPF) (black line) and uncertainty interval around the frontier (grey band). Countries are labelled in bold when their ratio to the frontier is in the top 10 percent (performing poorly relative to HAQ Index) and in italics when their ratio to the frontier is in the bottom 10 percent (performing well relative to HAQ Index). These results show the prediction of the cause-specific SPF prior to scaling to the all-cause frontier.

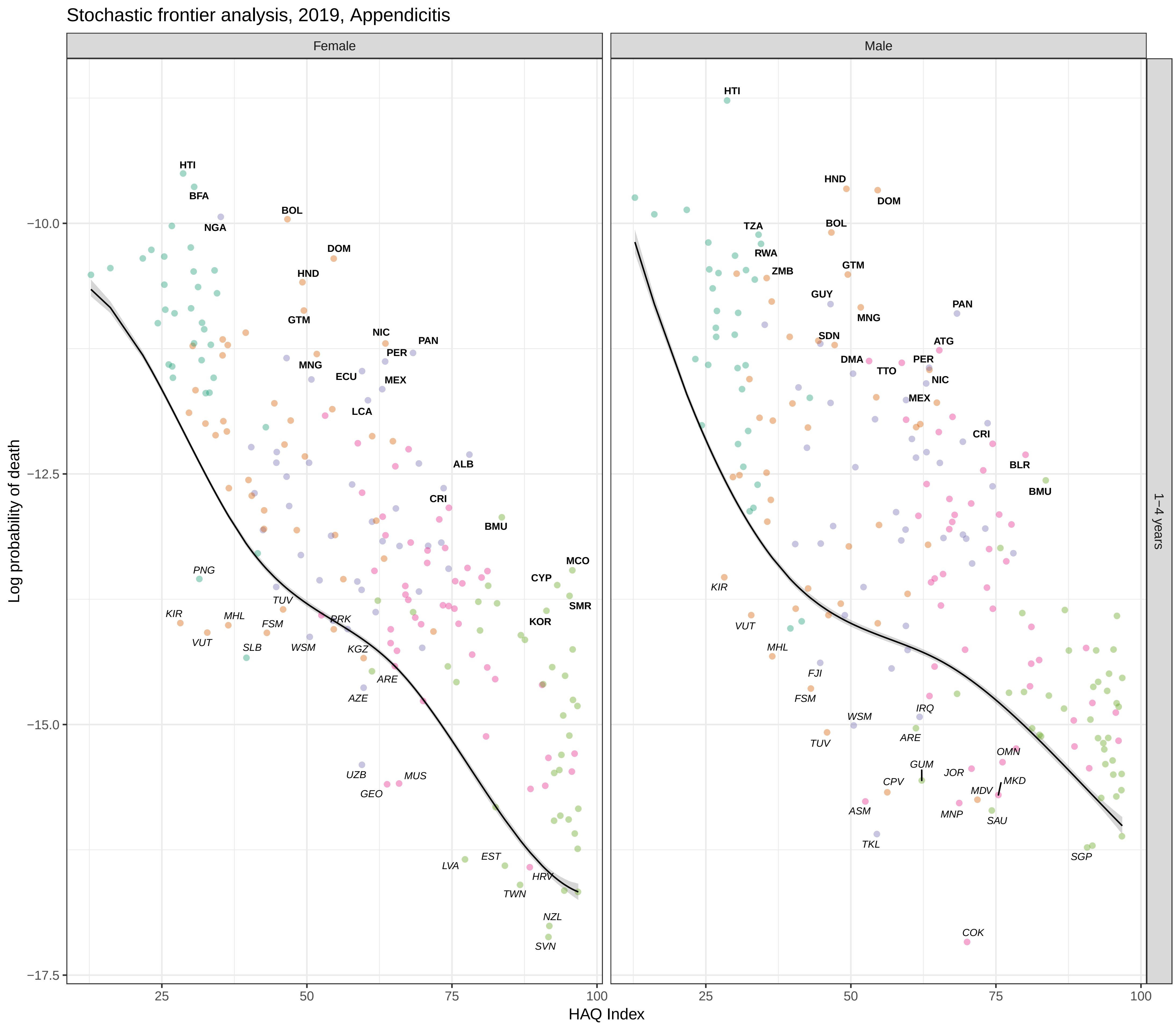

Figure S8
Cause- and age-specific mortality by country and Healthcare Access and Quality (HAQ) Index, with the Survival Potential Frontier (SPF) (black line) and uncertainty interval around the frontier (grey band). Countries are labelled in bold when their ratio to the frontier is in the top 10 percent (performing poorly relative to HAQ Index) and in italics when their ratio to the frontier is in the bottom 10 percent (performing well relative to HAQ Index). These results show the prediction of the cause-specific SPF prior to scaling to the all-cause frontier.

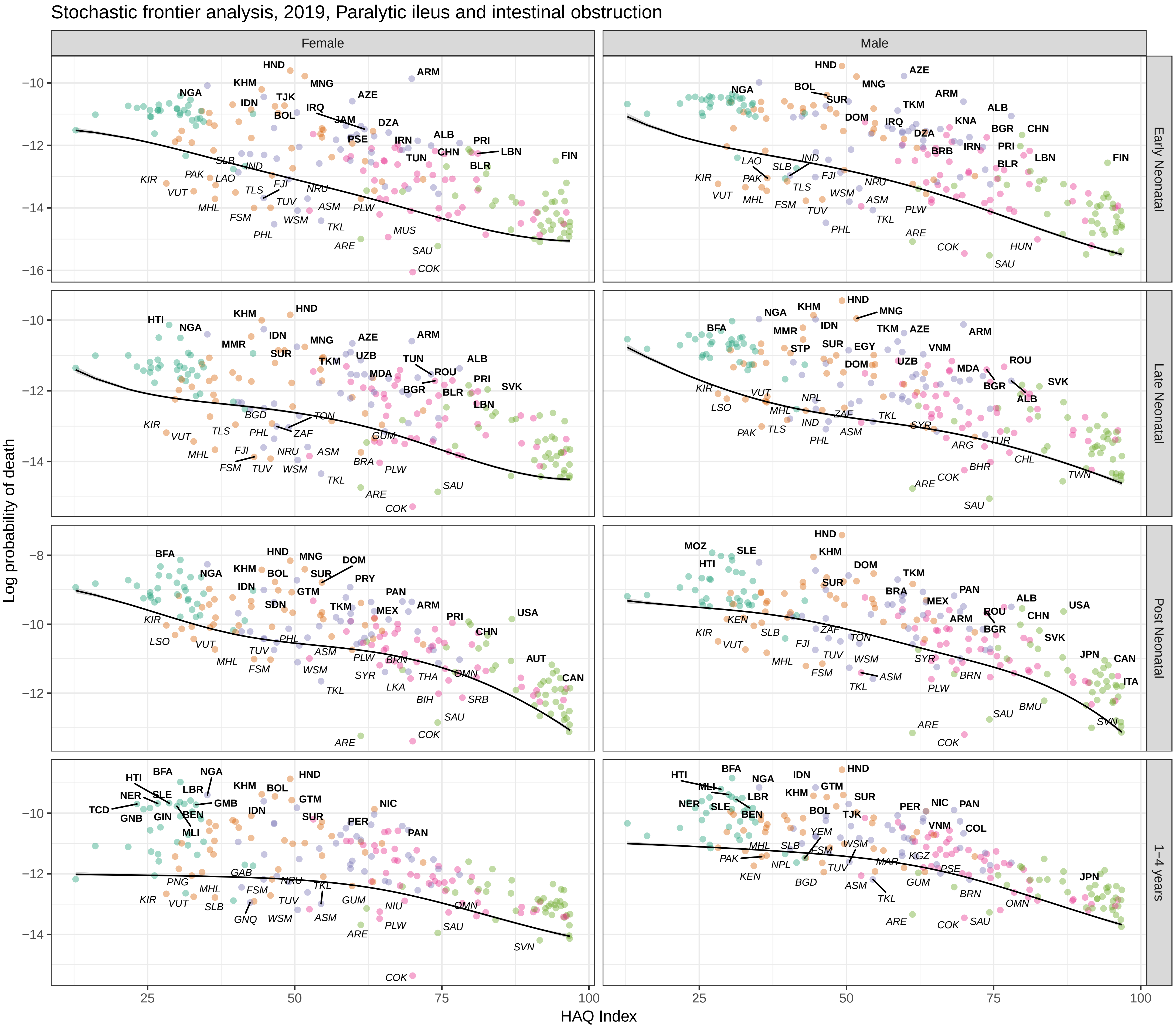

Figure S8
Cause- and age-specific mortality by country and Healthcare Access and Quality (HAQ) Index, with the Stochastic Survival Potential Frontier (SPF) (black line) and uncertainty interval around the frontier (grey band). Countries are labelled in bold when their ratio to the frontier is in the top 10 percent (performing poorly relative to HAQ Index) and in italics when their ratio to the frontier is in the bottom 10 percent (performing well relative to HAQ Index). These results show the prediction of the cause-specific SPF prior to scaling to the all-cause frontier.

SDI quintile

- Low SDI
- Low−middle SDI
- Middle SDI
- High−middle SDI
- High SDI

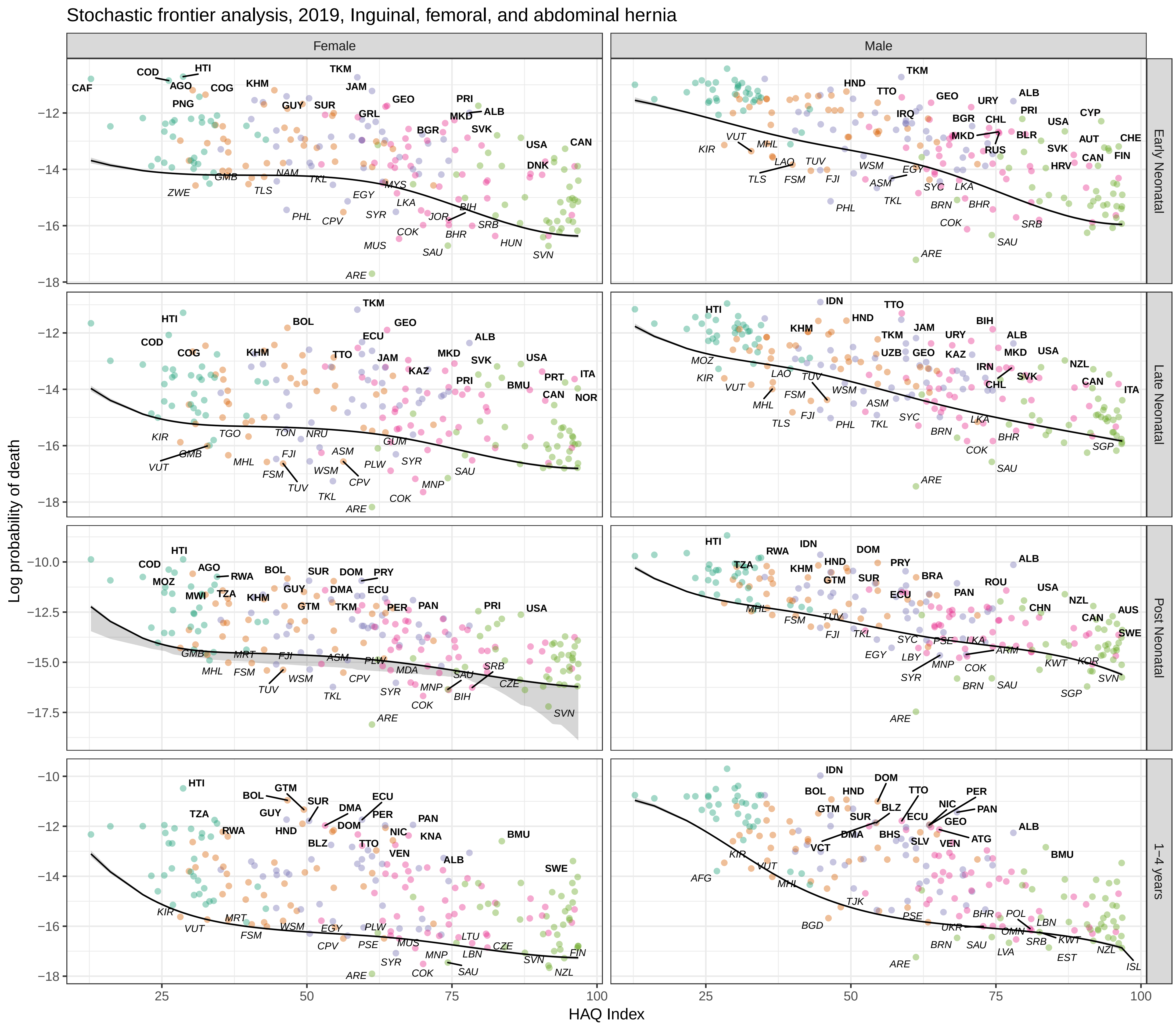

Figure S8
Cause- and age-specific mortality by country and Healthcare Access and Quality (HAQ) Index, with the Survival Potential Frontier (SPF) (black line) and uncertainty interval around the frontier (grey band). Countries are labelled in bold when their ratio to the frontier is in the top 10 percent (performing poorly relative to HAQ Index) and in italics when their ratio to the frontier is in the bottom 10 percent (performing well relative to HAQ Index). These results show the prediction of the cause-specific SPF prior to scaling to the all-cause frontier.

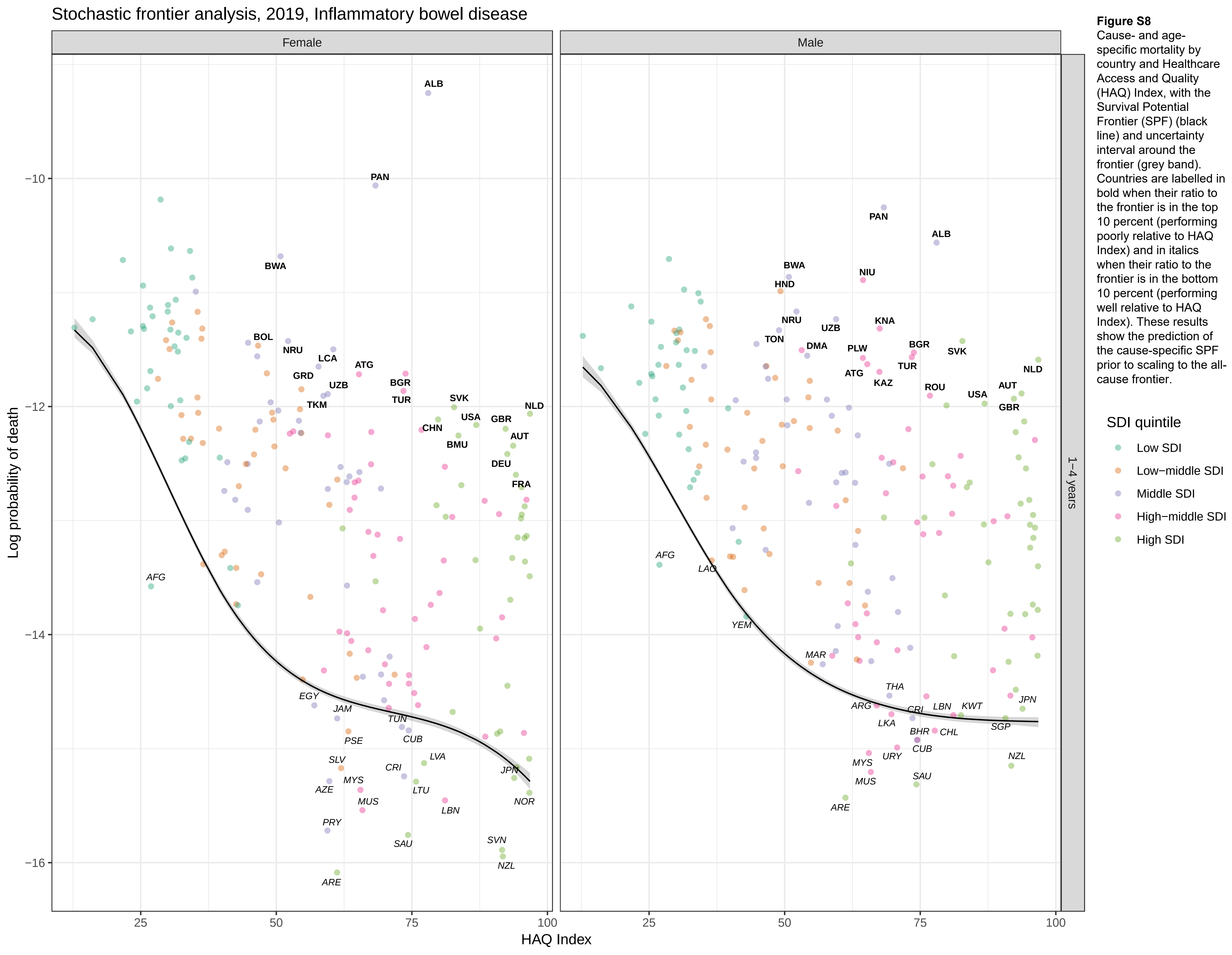

Figure S8
Cause- and age-specific mortality by country and Healthcare Access and Quality (HAQ) Index, with the Survival Potential Frontier (SPF) (black line) and uncertainty interval around the frontier (grey band). Countries are labelled in bold when their ratio to the frontier is in the top 10 percent (performing poorly relative to HAQ Index) and in italics when their ratio to the frontier is in the bottom 10 percent (performing well relative to HAQ Index). These results show the prediction of the cause-specific SPF prior to scaling to the all-cause frontier.

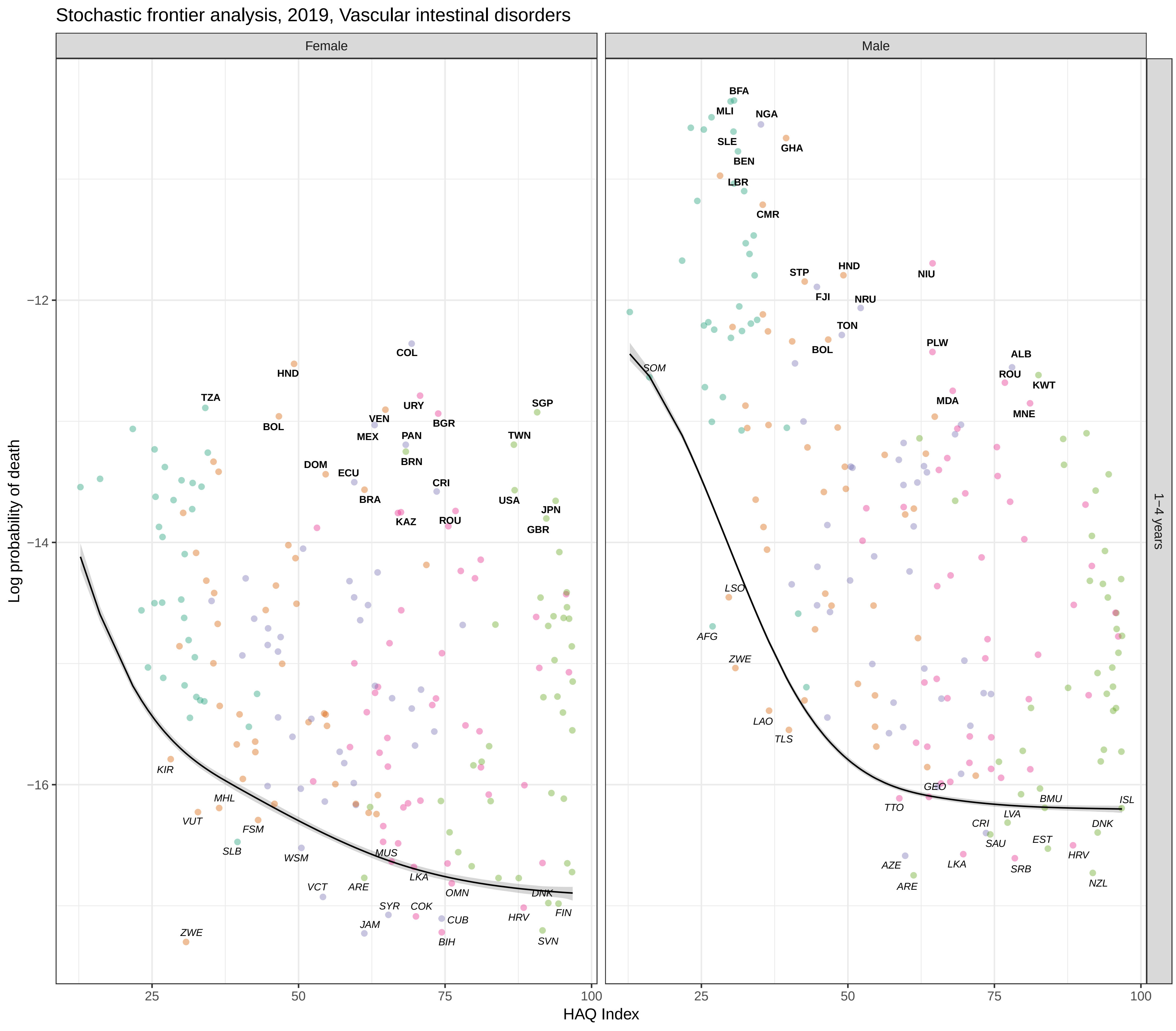

Figure S8
Cause- and age-specific mortality by country and Healthcare Access and Quality (HAQ) Index, with the Survival Potential Frontier (SPF) (black line) and uncertainty interval around the frontier (grey band). Countries are labelled in bold when their ratio to the frontier is in the top 10 percent (performing poorly relative to HAQ Index) and in italics when their ratio to the frontier is in the bottom 10 percent (performing well relative to HAQ Index). These results show the prediction of the cause-specific SPF prior to scaling to the all-cause frontier.

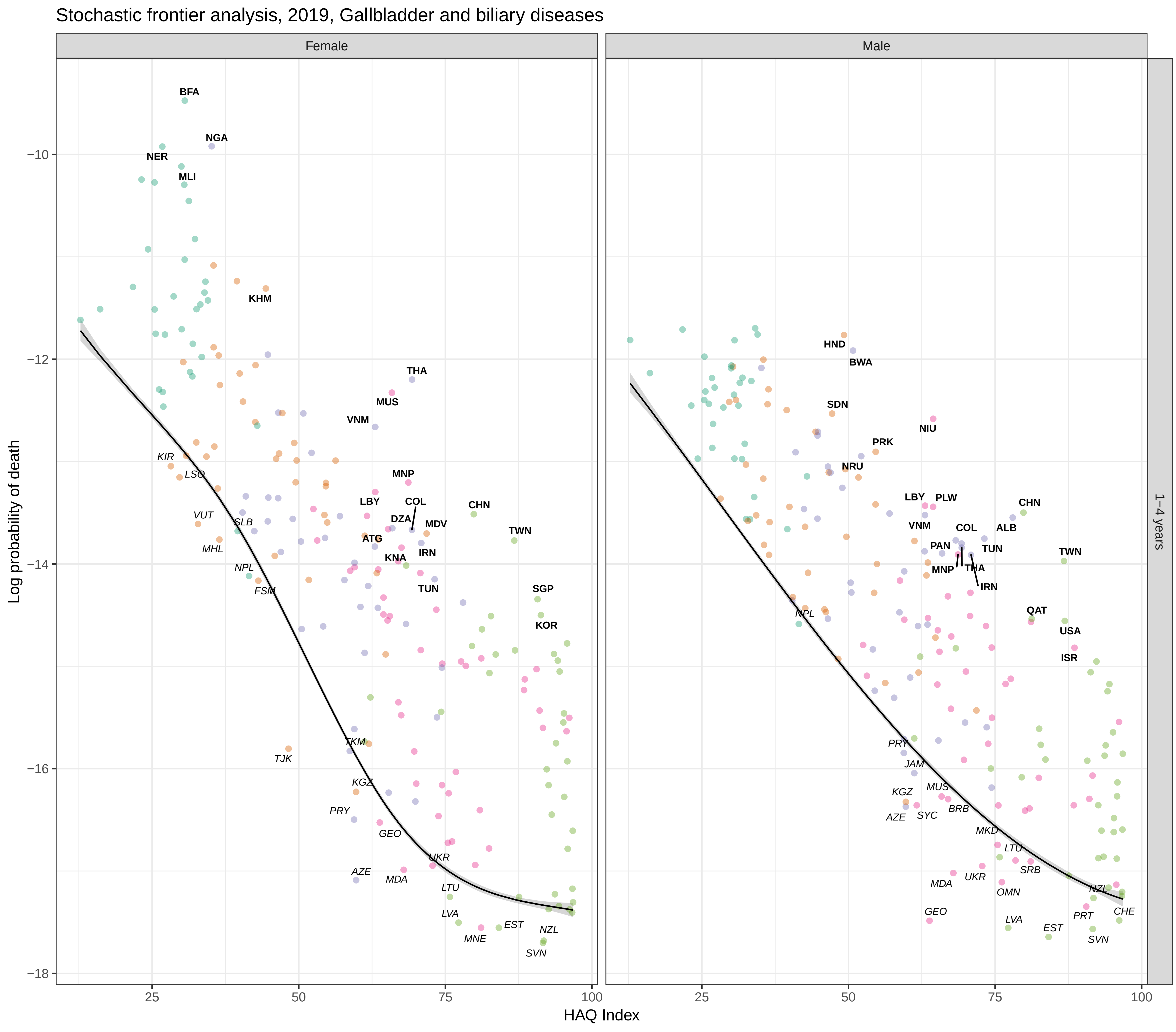

Figure S8
Cause- and age-specific mortality by country and Healthcare Access and Quality (HAQ) Index, with the Survival Potential Frontier (SPF) (black line) and uncertainty interval around the frontier (grey band). Countries are labelled in bold when their ratio to the frontier is in the top 10 percent (performing poorly relative to HAQ Index) and in italics when their ratio to the frontier is in the bottom 10 percent (performing well relative to HAQ Index). These results show the prediction of the cause-specific SPF prior to scaling to the all-cause frontier.

SDI quintile
- Low SDI
- Low−middle SDI
- Middle SDI
- High−middle SDI
- High SDI

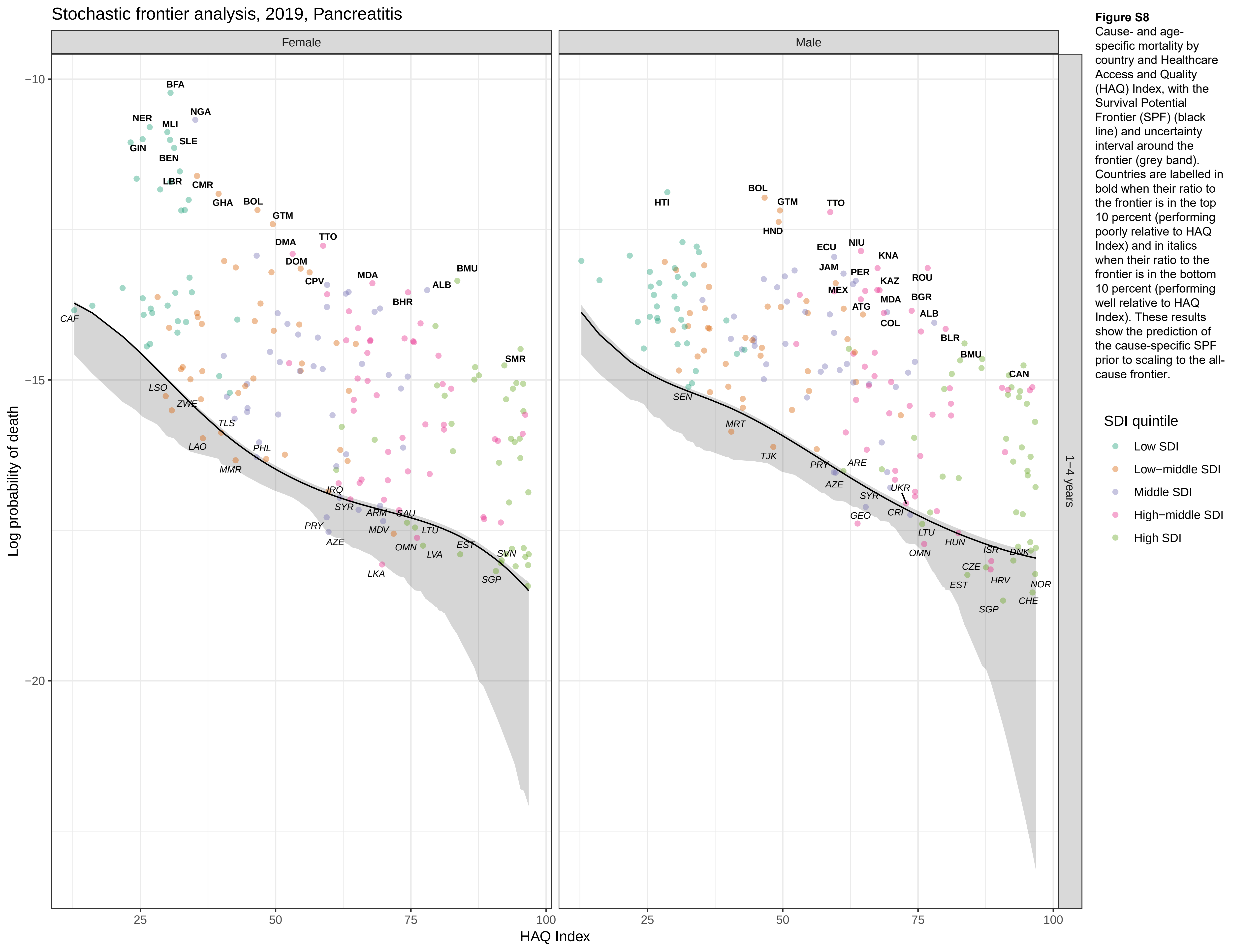

Figure S8
Cause- and age-specific mortality by country and Healthcare Access and Quality (HAQ) Index, with the Survival Potential Frontier (SPF) (black line) and uncertainty interval around the frontier (grey band). Countries are labelled in bold when their ratio to the frontier is in the top 10 percent (performing poorly relative to HAQ Index) and in italics when their ratio to the frontier is in the bottom 10 percent (performing well relative to HAQ Index). These results show the prediction of the cause-specific SPF prior to scaling to the all-cause frontier.

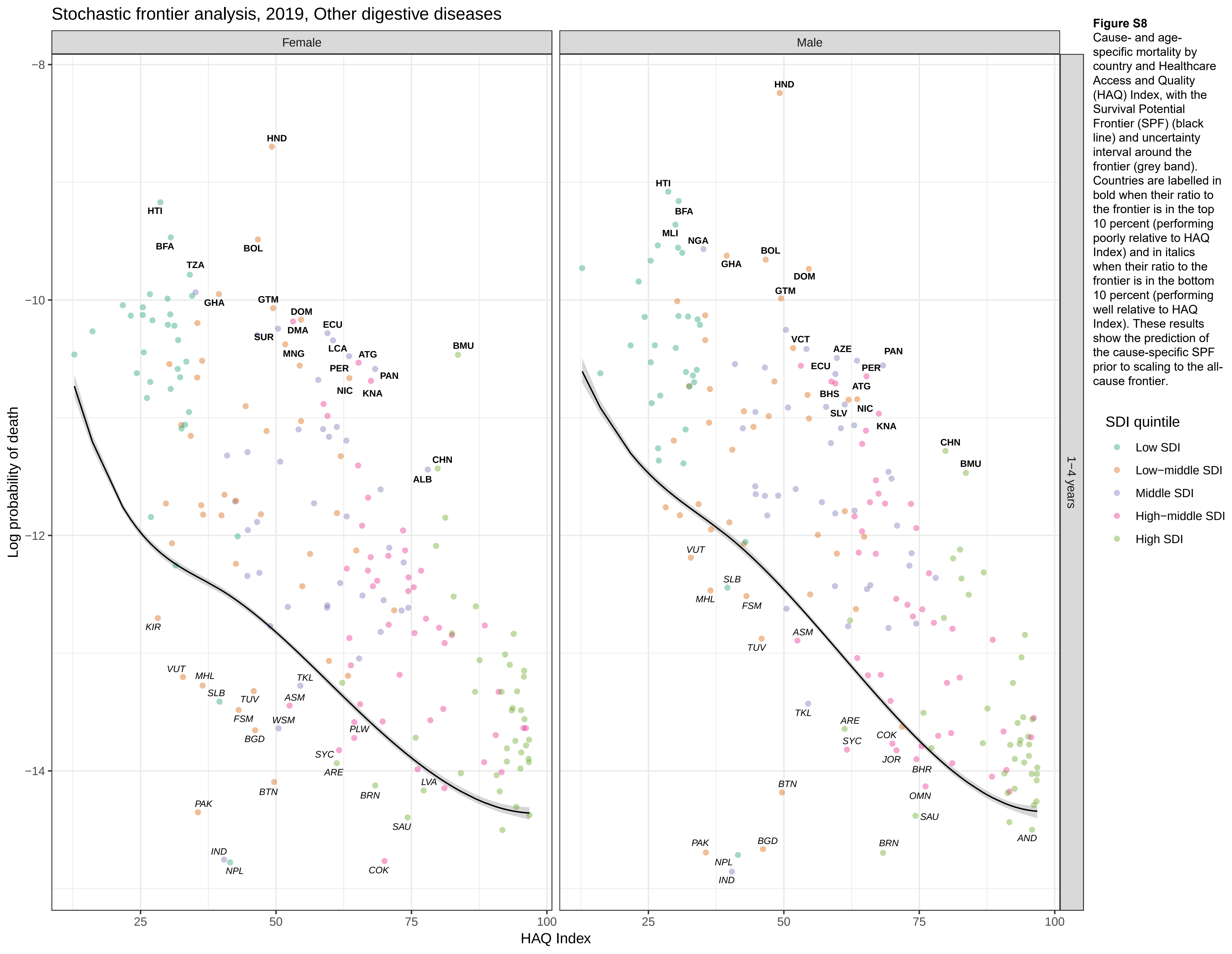

Figure S8
Cause- and age-specific mortality by country and Healthcare Access and Quality (HAQ) Index, with the Survival Potential Frontier (SPF) (black line) and uncertainty interval around the frontier (grey band). Countries are labelled in bold when their ratio to the frontier is in the top 10 percent (performing poorly relative to HAQ Index) and in italics when their ratio to the frontier is in the bottom 10 percent (performing well relative to HAQ Index). These results show the prediction of the cause-specific SPF prior to scaling to the all-cause frontier.

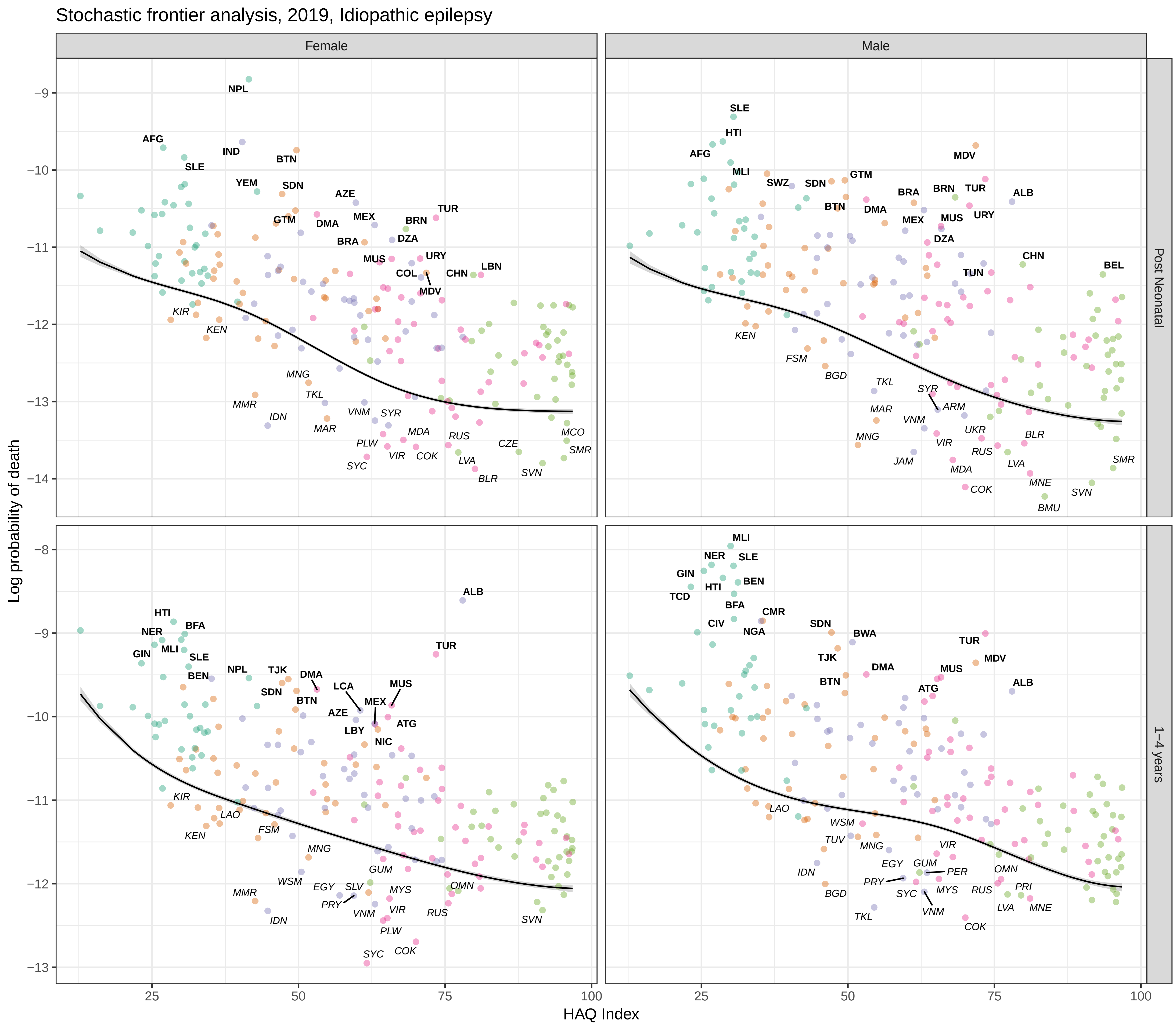

Figure S8
Cause- and age-specific mortality by country and Healthcare Access and Quality (HAQ) Index, with the Survival Potential Frontier (SPF) (black line) and uncertainty interval around the frontier (grey band). Countries are labelled in bold when their ratio to the frontier is in the top 10 percent (performing poorly relative to HAQ Index) and in italics when their ratio to the frontier is in the bottom 10 percent (performing well relative to HAQ Index). These results show the prediction of the cause-specific SPF prior to scaling to the all-cause frontier.

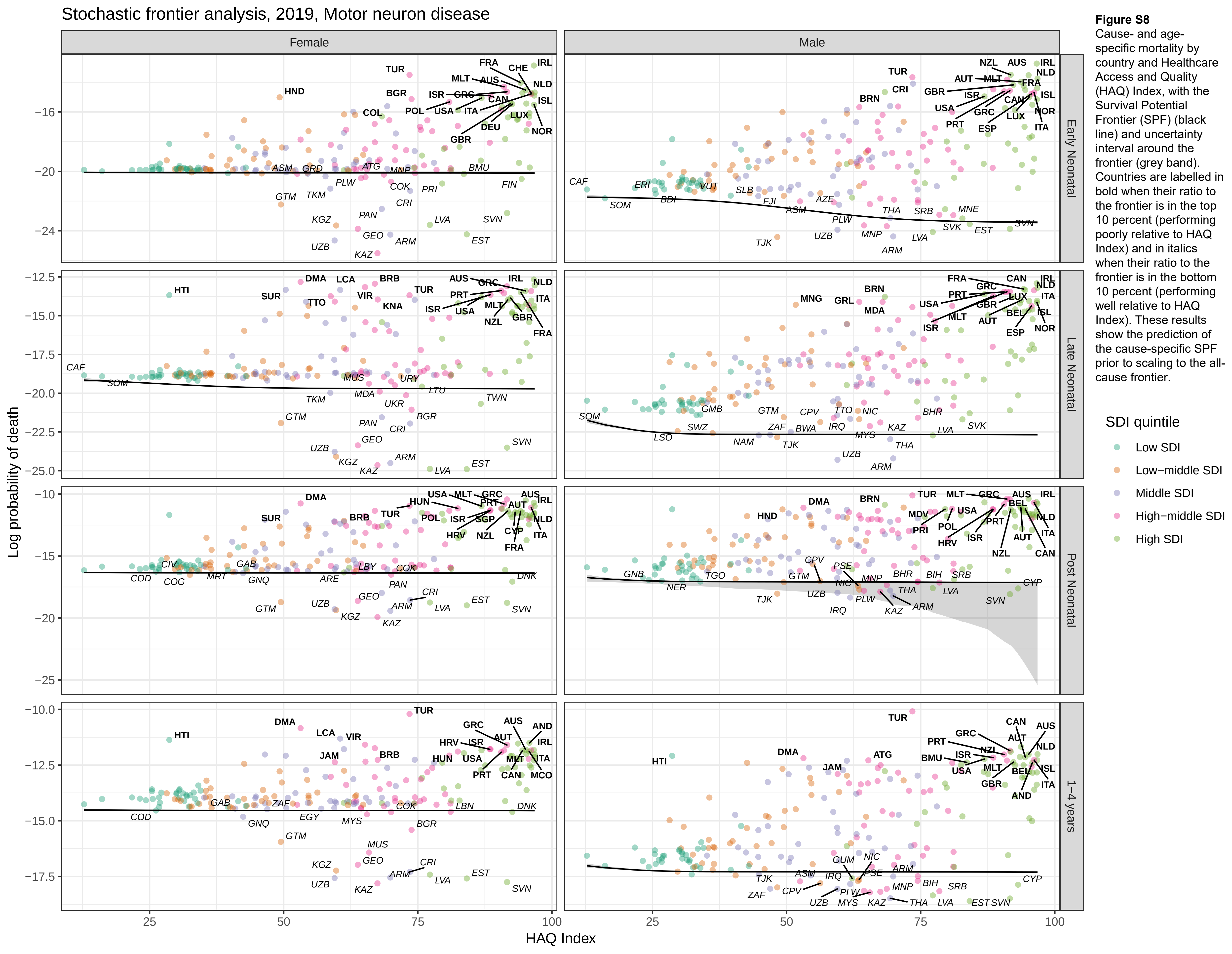

Figure S8
Cause- and age-specific mortality by country and Healthcare Access and Quality (HAQ) Index, with the Survival Potential Frontier (SPF) (black line) and uncertainty interval around the frontier (grey band). Countries are labelled in bold when their ratio to the frontier is in the top 10 percent (performing poorly relative to HAQ Index) and in italics when their ratio to the frontier is in the bottom 10 percent (performing well relative to HAQ Index). These results show the prediction of the cause-specific SPF prior to scaling to the all-cause frontier.

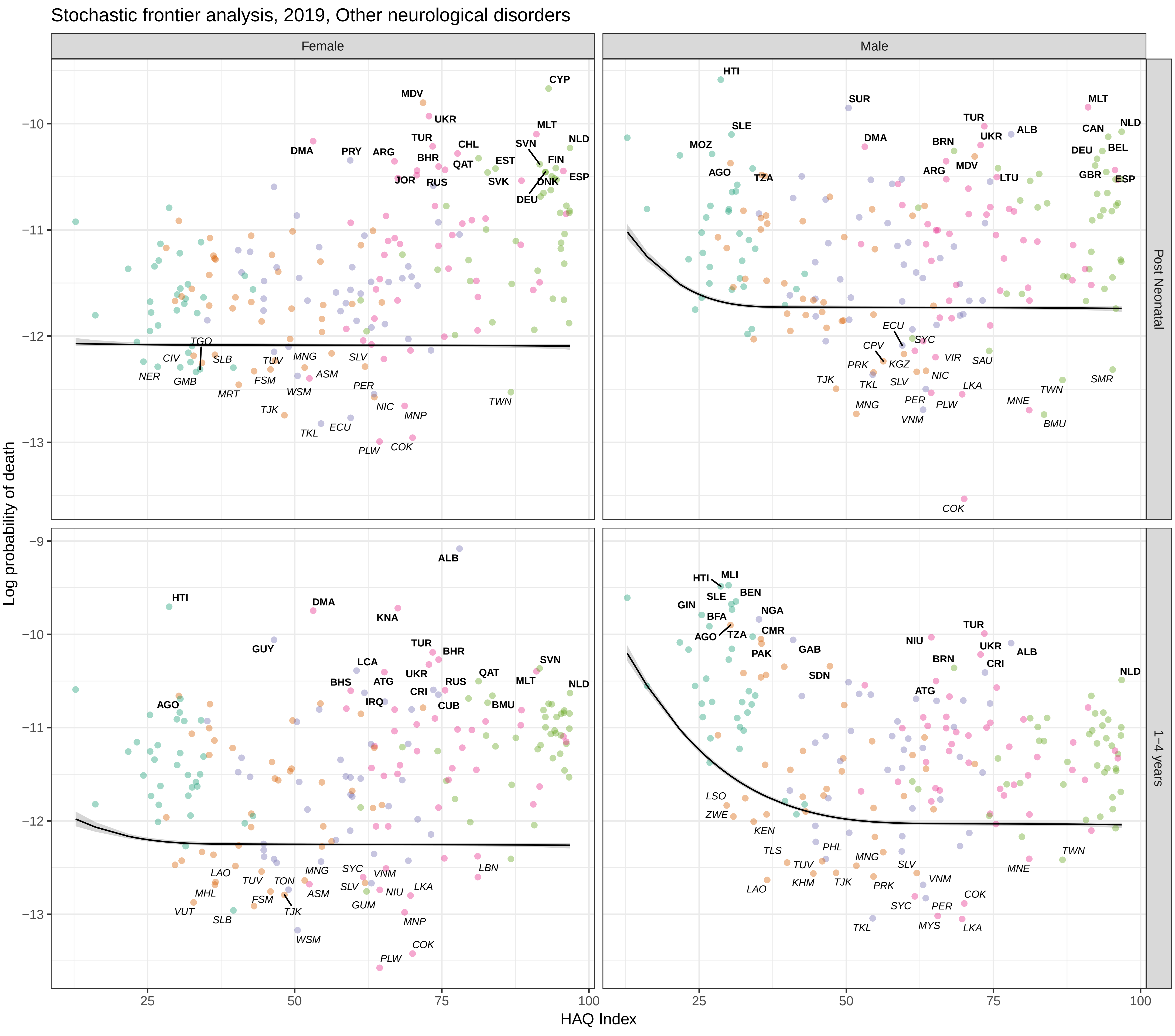

Figure S8
Cause- and age-specific mortality by country and Healthcare Access and Quality (HAQ) Index, with the Survival Potential Frontier (SPF) (black line) and uncertainty interval around the frontier (grey band). Countries are labelled in bold when their ratio to the frontier is in the top 10 percent (performing poorly relative to HAQ Index) and in italics when their ratio to the frontier is in the bottom 10 percent (performing well relative to HAQ Index). These results show the prediction of the cause-specific SPF prior to scaling to the all-cause frontier.

SDI quintile
- Low SDI
- Low−middle SDI
- Middle SDI
- High−middle SDI
- High SDI

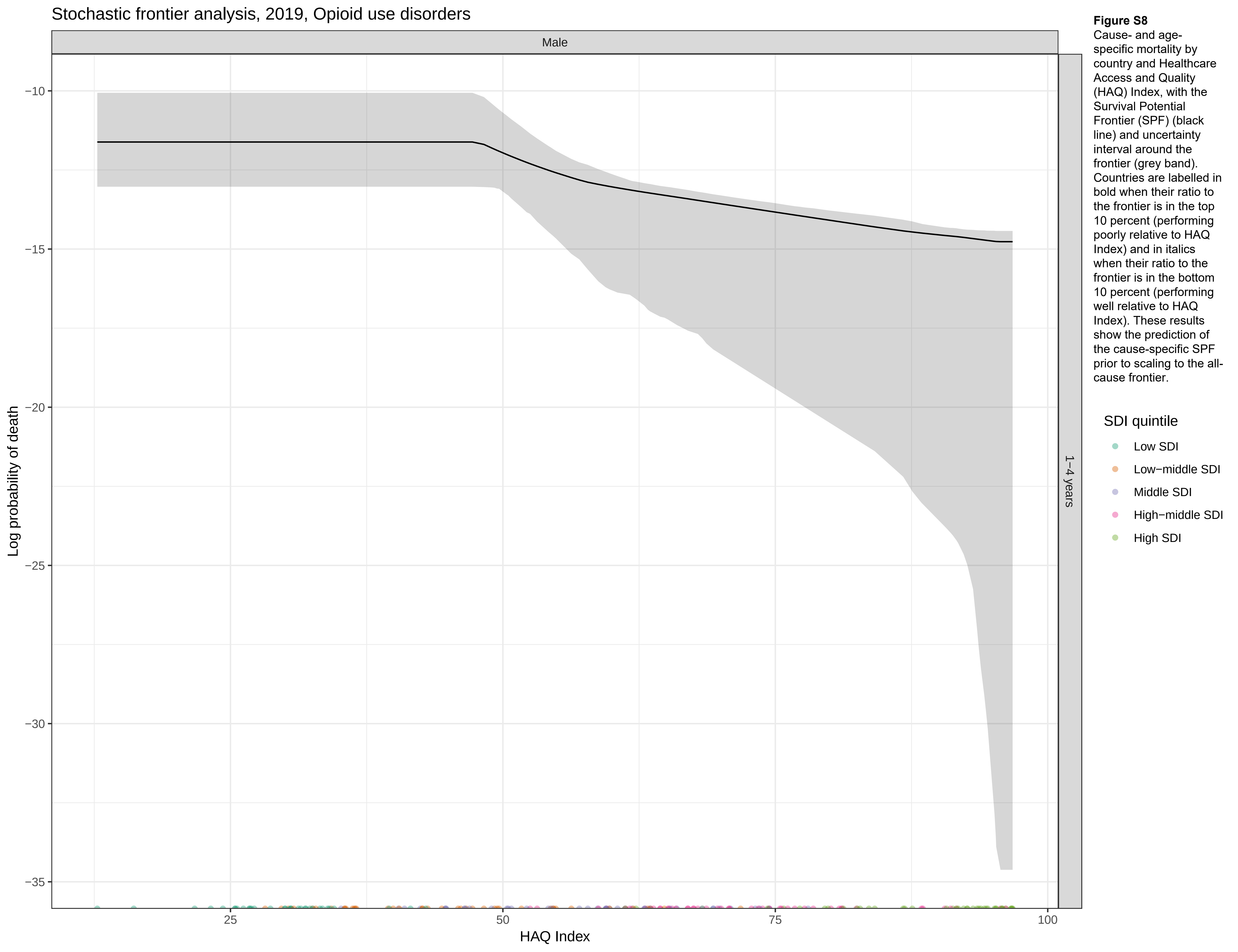

Figure S8
Cause- and age-specific mortality by country and Healthcare Access and Quality (HAQ) Index, with the Survival Potential Frontier (SPF) (black line) and uncertainty interval around the frontier (grey band). Countries are labelled in bold when their ratio to the frontier is in the top 10 percent (performing poorly relative to HAQ Index) and in italics when their ratio to the frontier is in the bottom 10 percent (performing well relative to HAQ Index). These results show the prediction of the cause-specific SPF prior to scaling to the all-cause frontier.

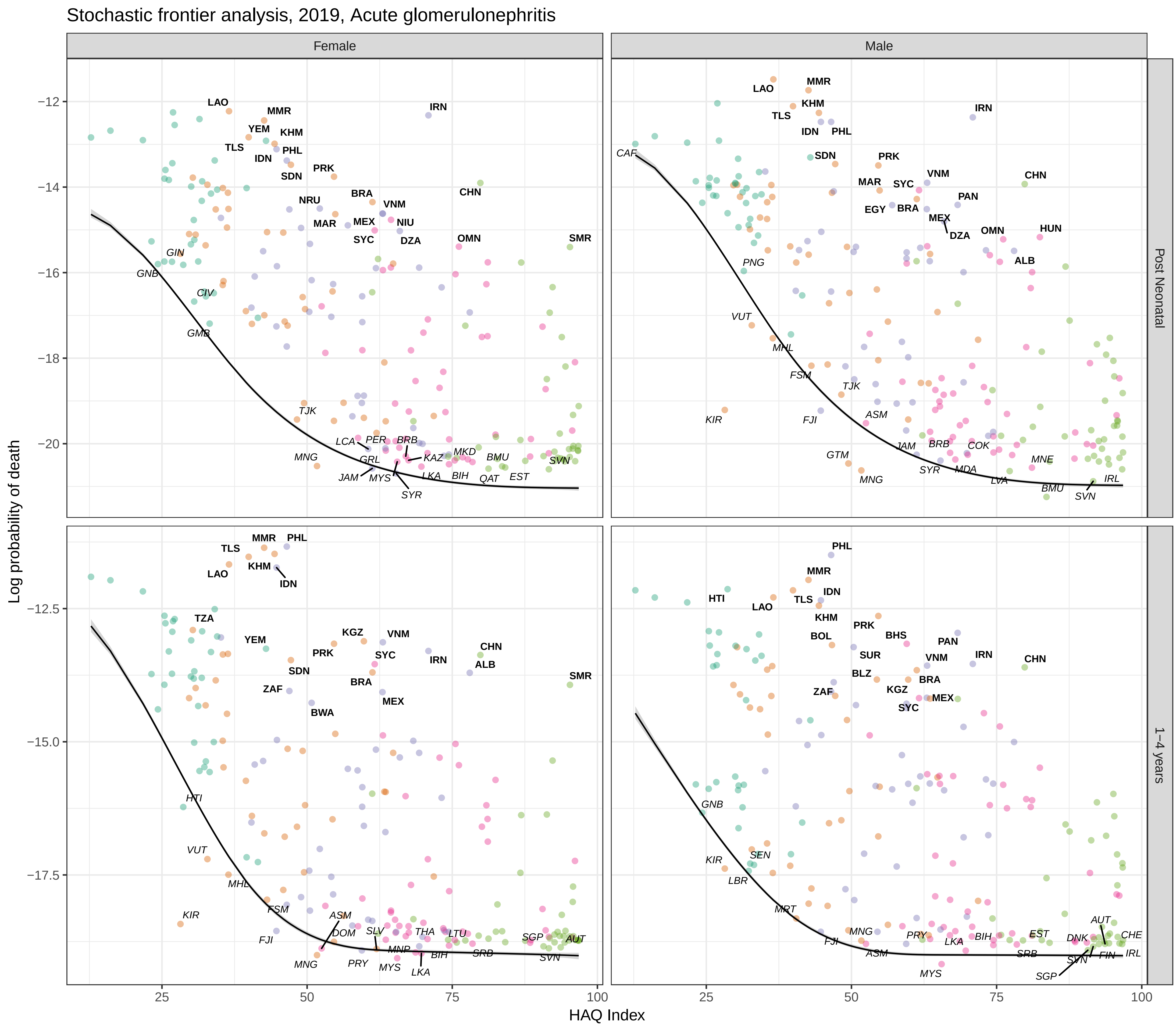

Figure S8
Cause- and age-specific mortality by country and Healthcare Access and Quality (HAQ) Index, with the Survival Potential Frontier (SPF) (black line) and uncertainty interval around the frontier (grey band). Countries are labelled in bold when their ratio to the frontier is in the top 10 percent (performing poorly relative to HAQ Index) and in italics when their ratio to the frontier is in the bottom 10 percent (performing well relative to HAQ Index). These results show the prediction of the cause-specific SPF prior to scaling to the all-cause frontier.

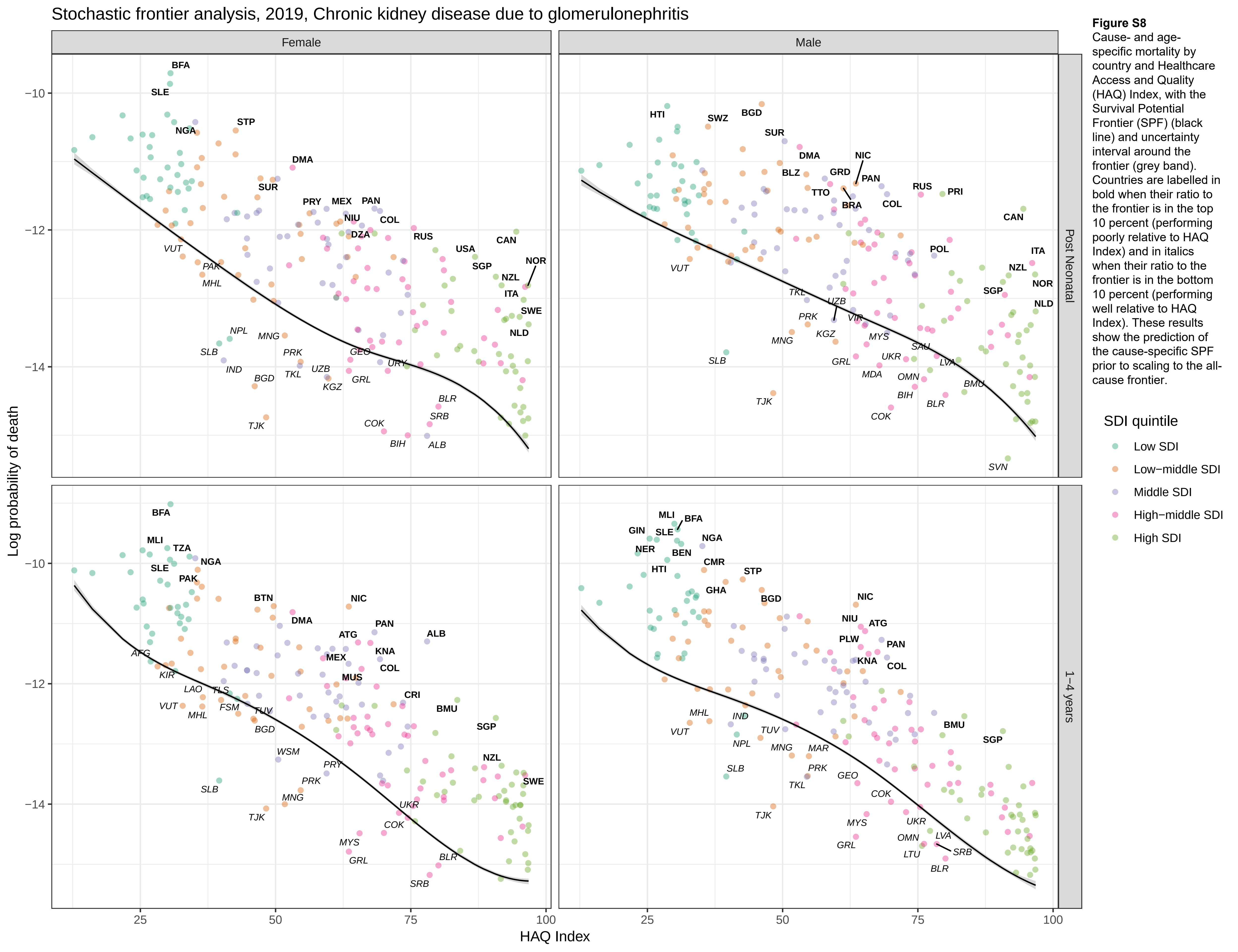

Figure S8
Cause- and age-specific mortality by country and Healthcare Access and Quality (HAQ) Index, with the Survival Potential Frontier (SPF) (black line) and uncertainty interval around the frontier (grey band). Countries are labelled in bold when their ratio to the frontier is in the top 10 percent (performing poorly relative to HAQ Index) and in italics when their ratio to the frontier is in the bottom 10 percent (performing well relative to HAQ Index). These results show the prediction of the cause-specific SPF prior to scaling to the all-cause frontier.

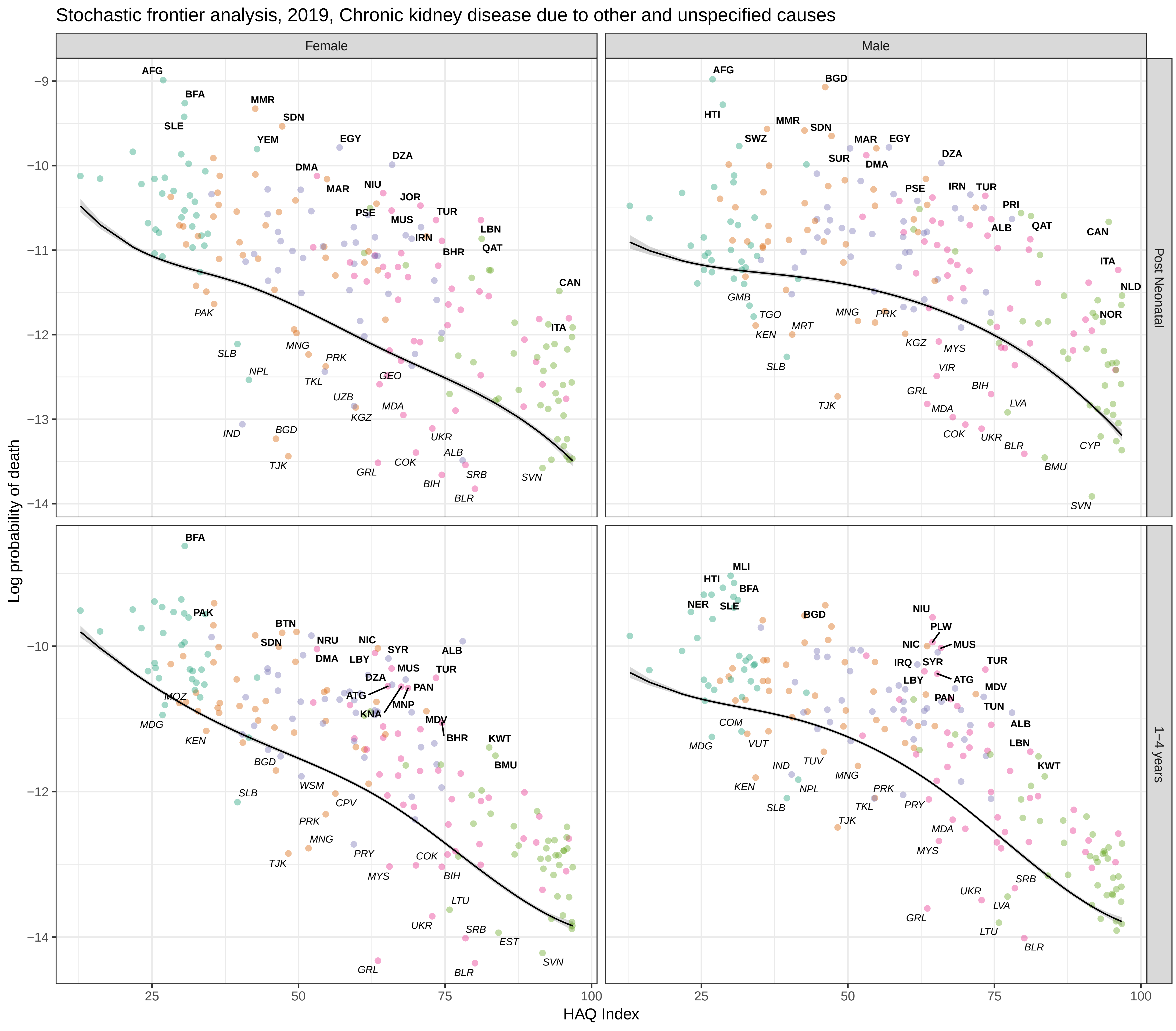

Figure S8
Cause- and age-specific mortality by country and Healthcare Access and Quality (HAQ) Index, with the Survival Potential Frontier (SPF) (black line) and uncertainty interval around the frontier (grey band). Countries are labelled in bold when their ratio to the frontier is in the top 10 percent (performing poorly relative to HAQ Index) and in italics when their ratio to the frontier is in the bottom 10 percent (performing well relative to HAQ Index). These results show the prediction of the cause-specific SPF prior to scaling to the all-cause frontier.

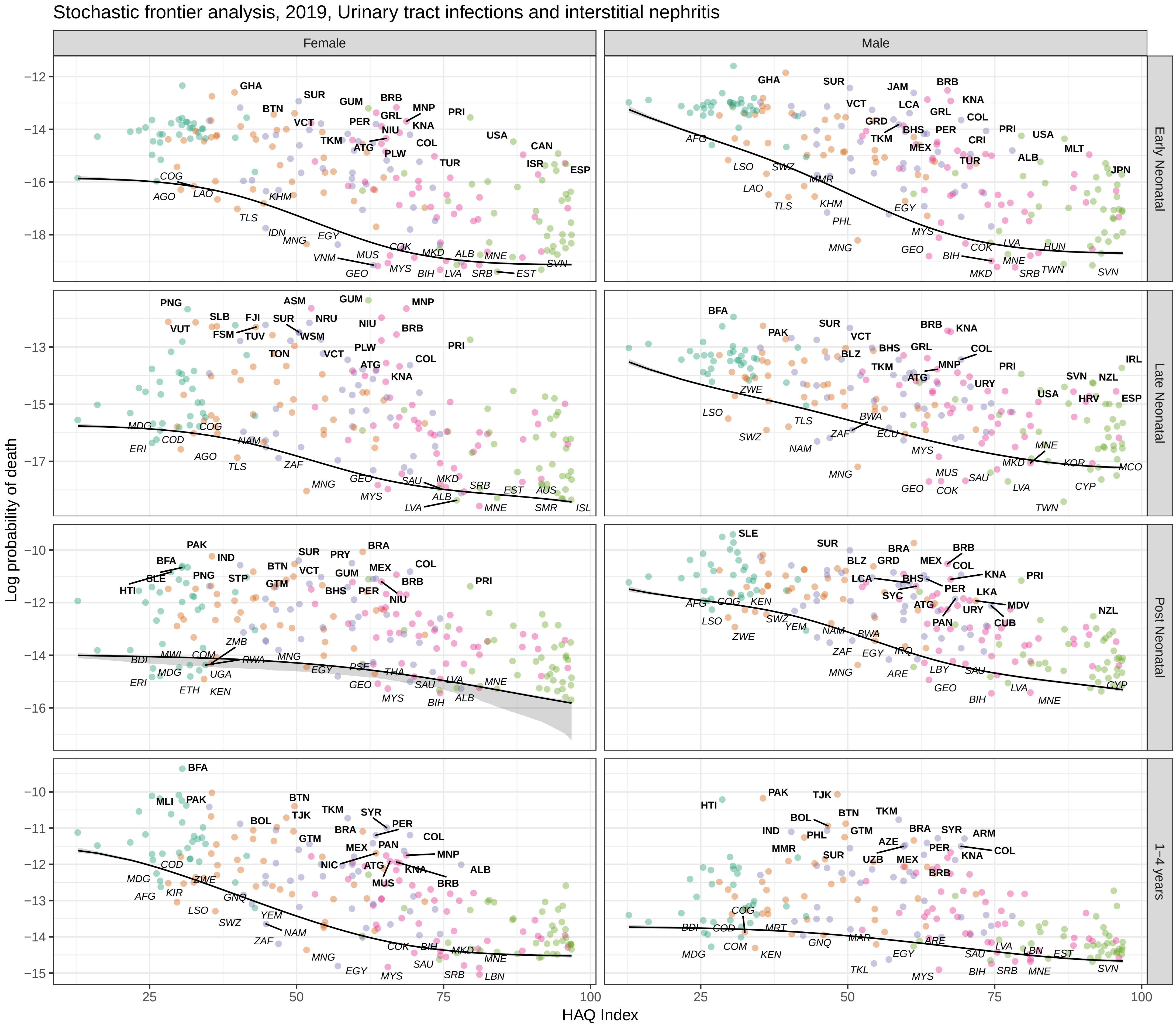

Figure S8
Cause- and age-specific mortality by country and Healthcare Access and Quality (HAQ) Index, with the Survival Potential Frontier (SPF) (black line) and uncertainty interval around the frontier (grey band). Countries are labelled in bold when their ratio to the frontier is in the top 10 percent (performing poorly relative to HAQ Index) and in italics when their ratio to the frontier is in the bottom 10 percent (performing well relative to HAQ Index). These results show the prediction of the cause-specific SPF prior to scaling to the all-cause frontier.

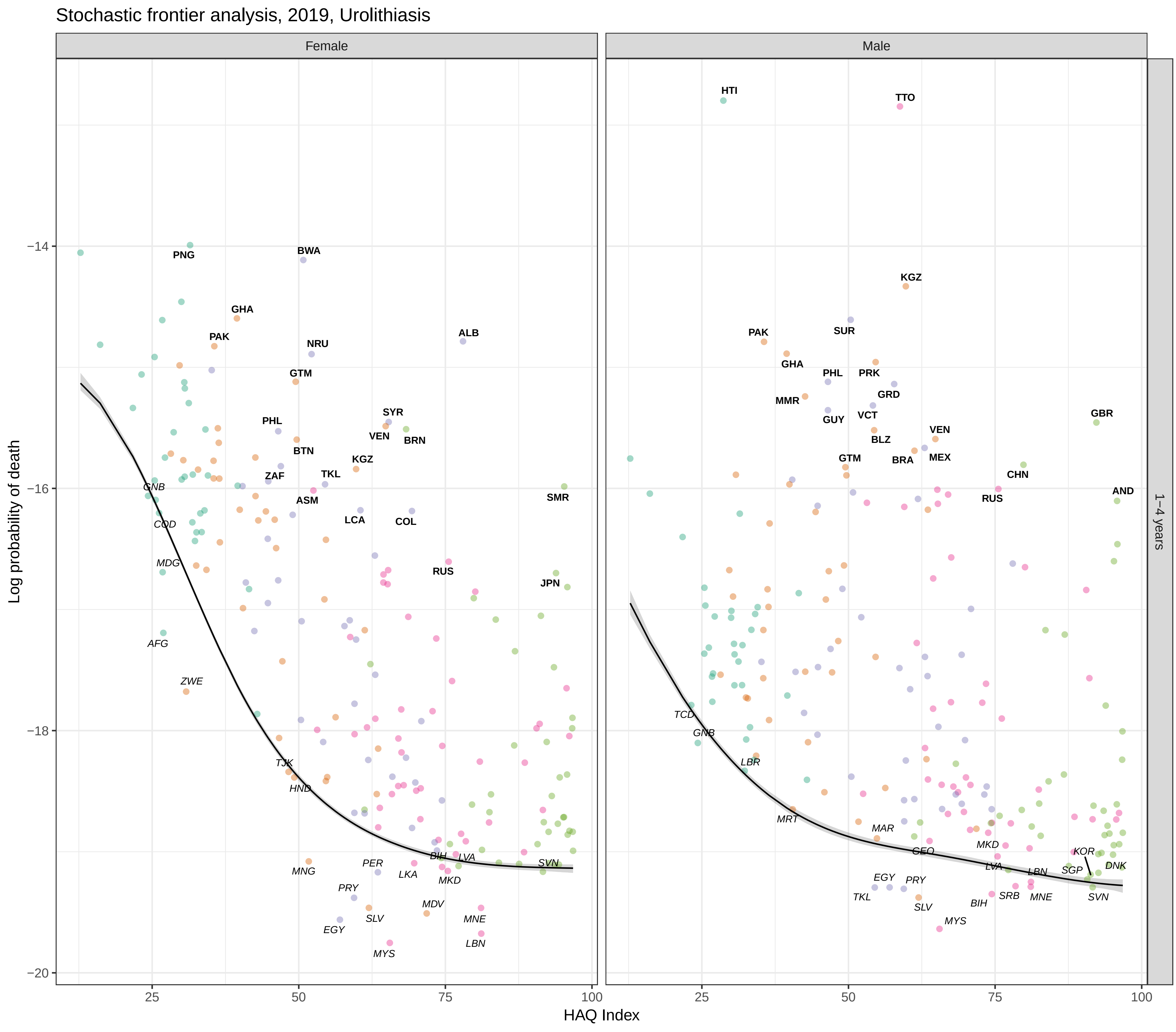

Figure S8
Cause- and age-specific mortality by country and Healthcare Access and Quality (HAQ) Index, with the Survival Potential Frontier (SPF) (black line) and uncertainty interval around the frontier (grey band). Countries are labelled in bold when their ratio to the frontier is in the top 10 percent (performing poorly relative to HAQ Index) and in italics when their ratio to the frontier is in the bottom 10 percent (performing well relative to HAQ Index). These results show the prediction of the cause-specific SPF prior to scaling to the all-cause frontier.

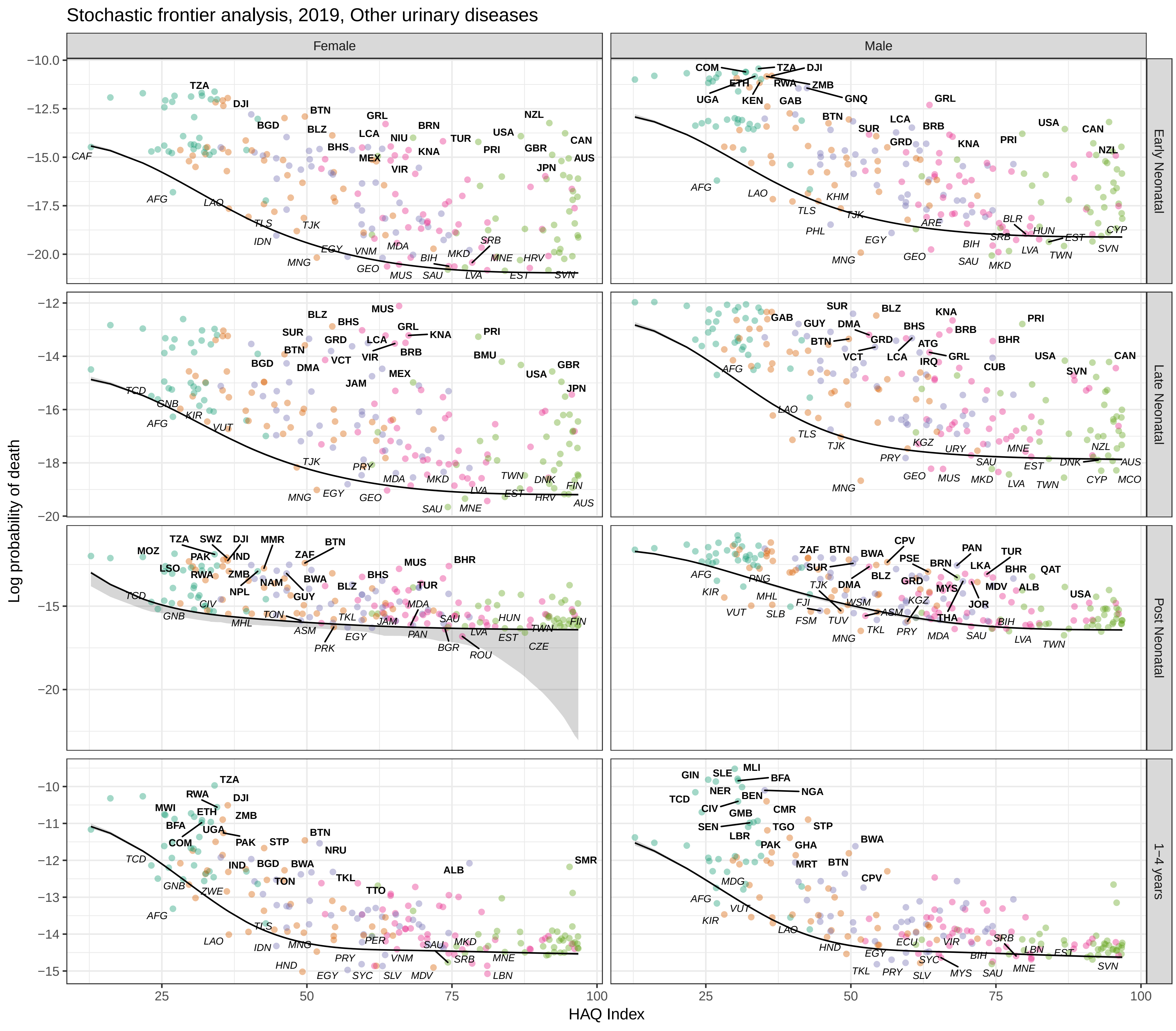

Figure S8
Cause- and age-specific mortality by country and Healthcare Access and Quality (HAQ) Index, with the Survival Potential Frontier (SPF) (black line) and uncertainty interval around the frontier (grey band). Countries are labelled in bold when their ratio to the frontier is in the top 10 percent (performing poorly relative to HAQ Index) and in italics when their ratio to the frontier is in the bottom 10 percent (performing well relative to HAQ Index). These results show the prediction of the cause-specific SPF prior to scaling to the all-cause frontier.

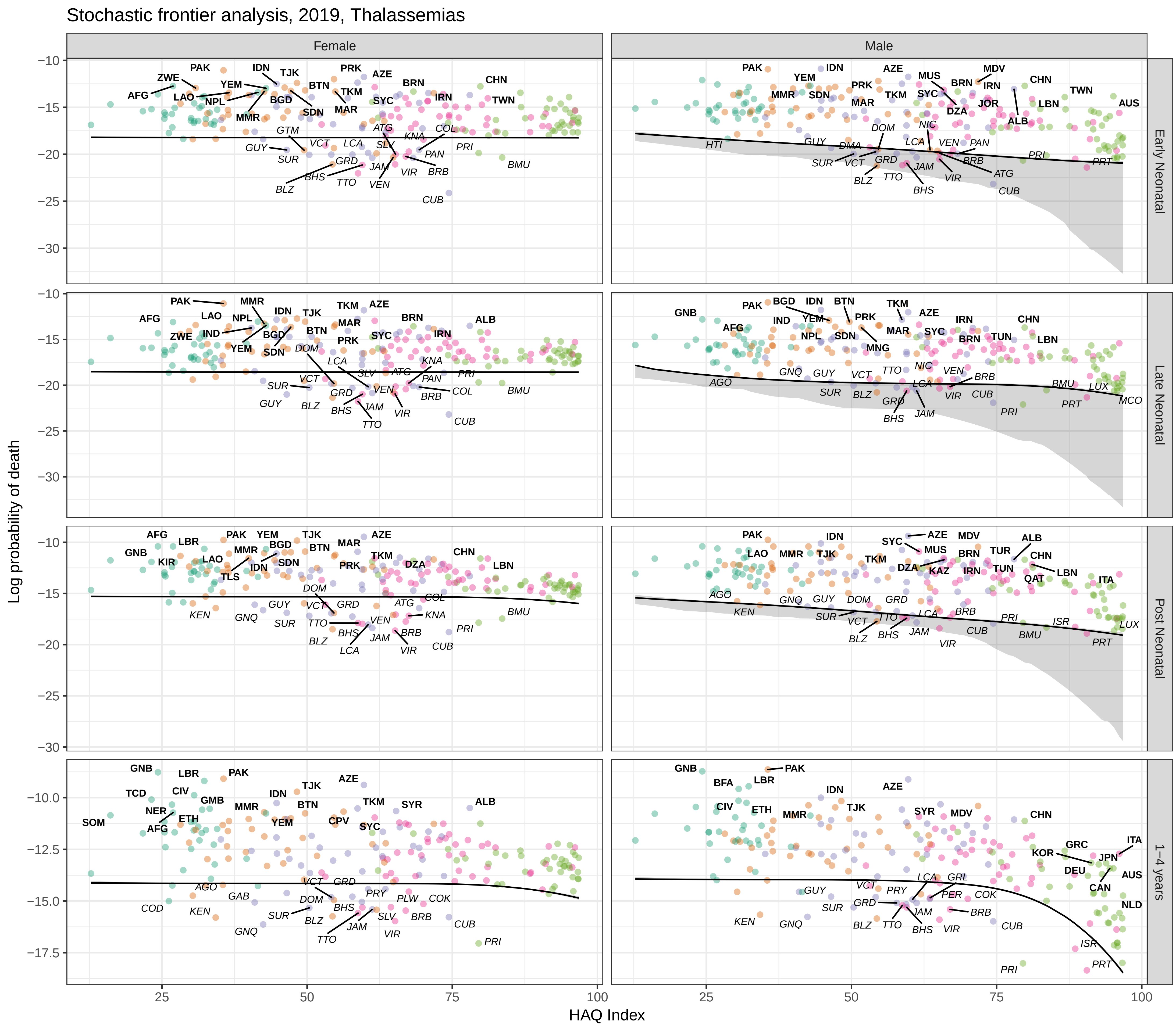

Figure S8
Cause- and age-specific mortality by country and Healthcare Access and Quality (HAQ) Index, with the Survival Potential Frontier (SPF) (black line) and uncertainty interval around the frontier (grey band). Countries are labelled in bold when their ratio to the frontier is in the top 10 percent (performing poorly relative to HAQ Index) and in italics when their ratio to the frontier is in the bottom 10 percent (performing well relative to HAQ Index). These results show the prediction of the cause-specific SPF prior to scaling to the all-cause frontier.

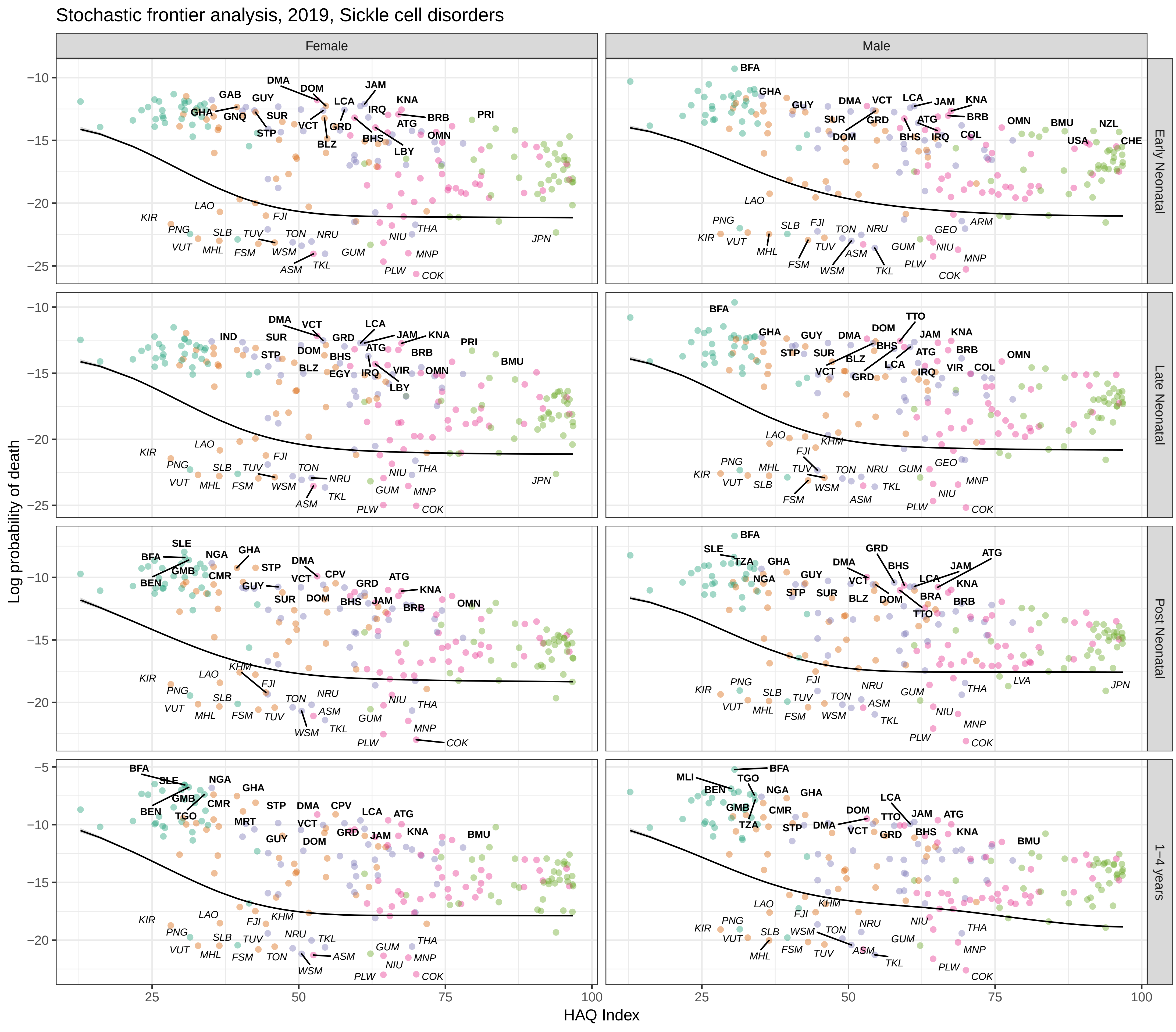

Figure S8
Cause- and age-specific mortality by country and Healthcare Access and Quality (HAQ) Index, with the Survival Potential Frontier (SPF) (black line) and uncertainty interval around the frontier (grey band). Countries are labelled in bold when their ratio to the frontier is in the top 10 percent (performing poorly relative to HAQ Index) and in italics when their ratio to the frontier is in the bottom 10 percent (performing well relative to HAQ Index). These results show the prediction of the cause-specific SPF prior to scaling to the all-cause frontier.

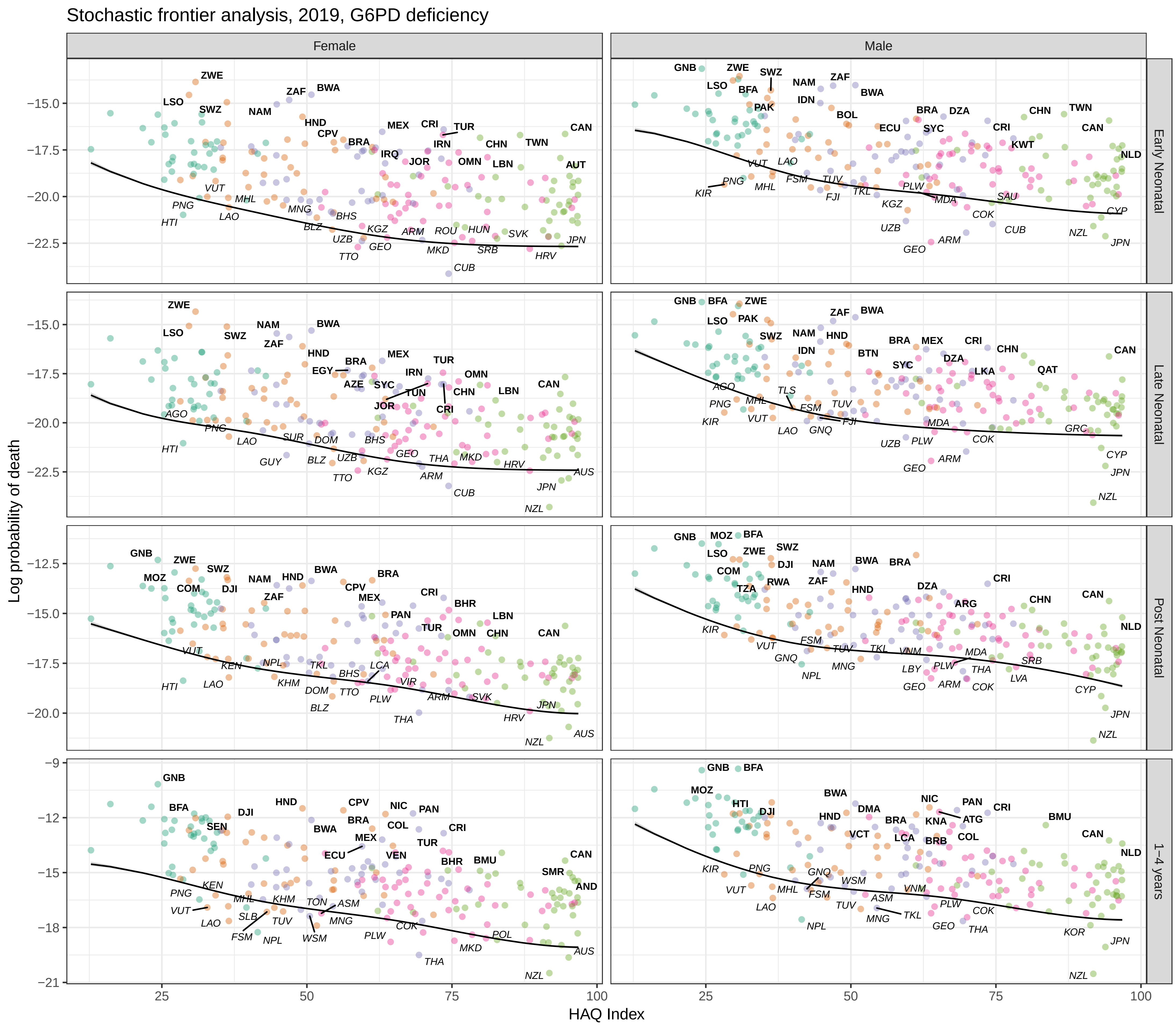

Figure S8
Cause- and age-specific mortality by country and Healthcare Access and Quality (HAQ) Index, with the Survival Potential Frontier (SPF) (black line) and uncertainty interval around the frontier (grey band). Countries are labelled in bold when their ratio to the frontier is in the top 10 percent (performing poorly relative to HAQ Index) and in italics when their ratio to the frontier is in the bottom 10 percent (performing well relative to HAQ Index). These results show the prediction of the cause-specific SPF prior to scaling to the all-cause frontier.

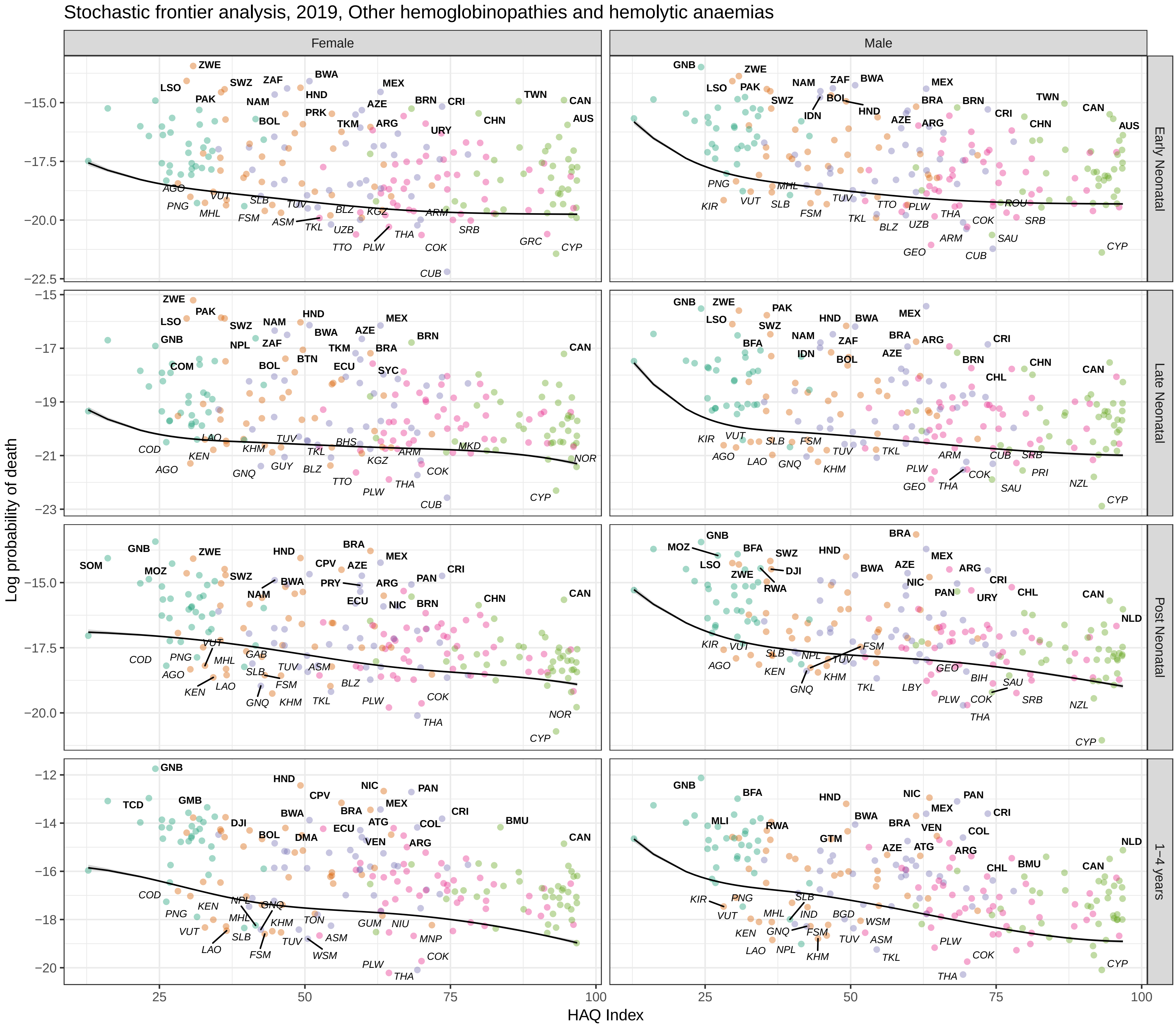

Figure S8
Cause- and age-specific mortality by country and Healthcare Access and Quality (HAQ) Index, with the Survival Potential Frontier (SPF) (black line) and uncertainty interval around the frontier (grey band). Countries are labelled in bold when their ratio to the frontier is in the top 10 percent (performing poorly relative to HAQ Index) and in italics when their ratio to the frontier is in the bottom 10 percent (performing well relative to HAQ Index). These results show the prediction of the cause-specific SPF prior to scaling to the all-cause frontier.

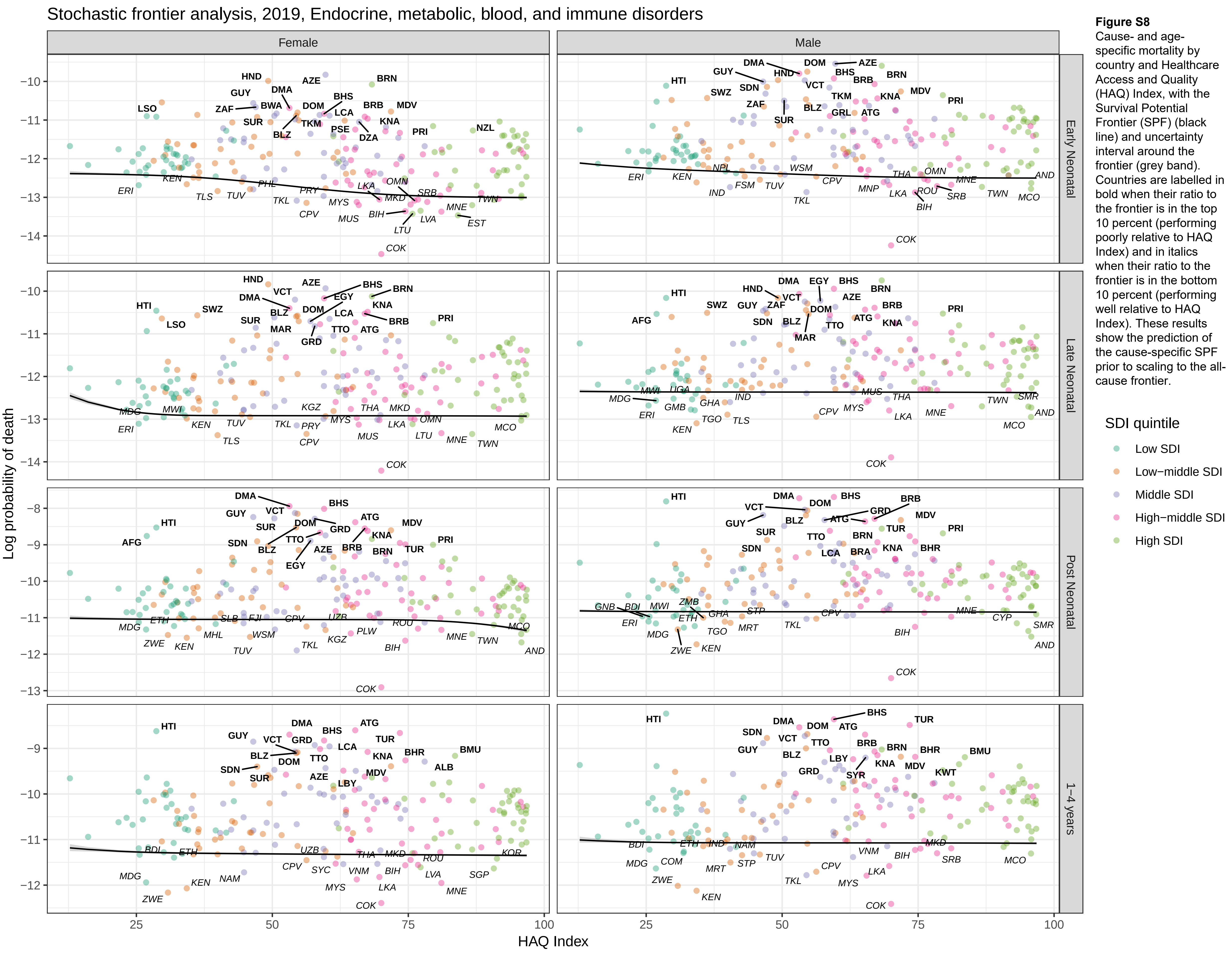

Figure S8
Cause- and age-specific mortality by country and Healthcare Access and Quality (HAQ) Index, with the Survival Potential Frontier (SPF) (black line) and uncertainty interval around the frontier (grey band). Countries are labelled in bold when their ratio to the frontier is in the top 10 percent (performing poorly relative to HAQ Index) and in italics when their ratio to the frontier is in the bottom 10 percent (performing well relative to HAQ Index). These results show the prediction of the cause-specific SPF prior to scaling to the all-cause frontier.

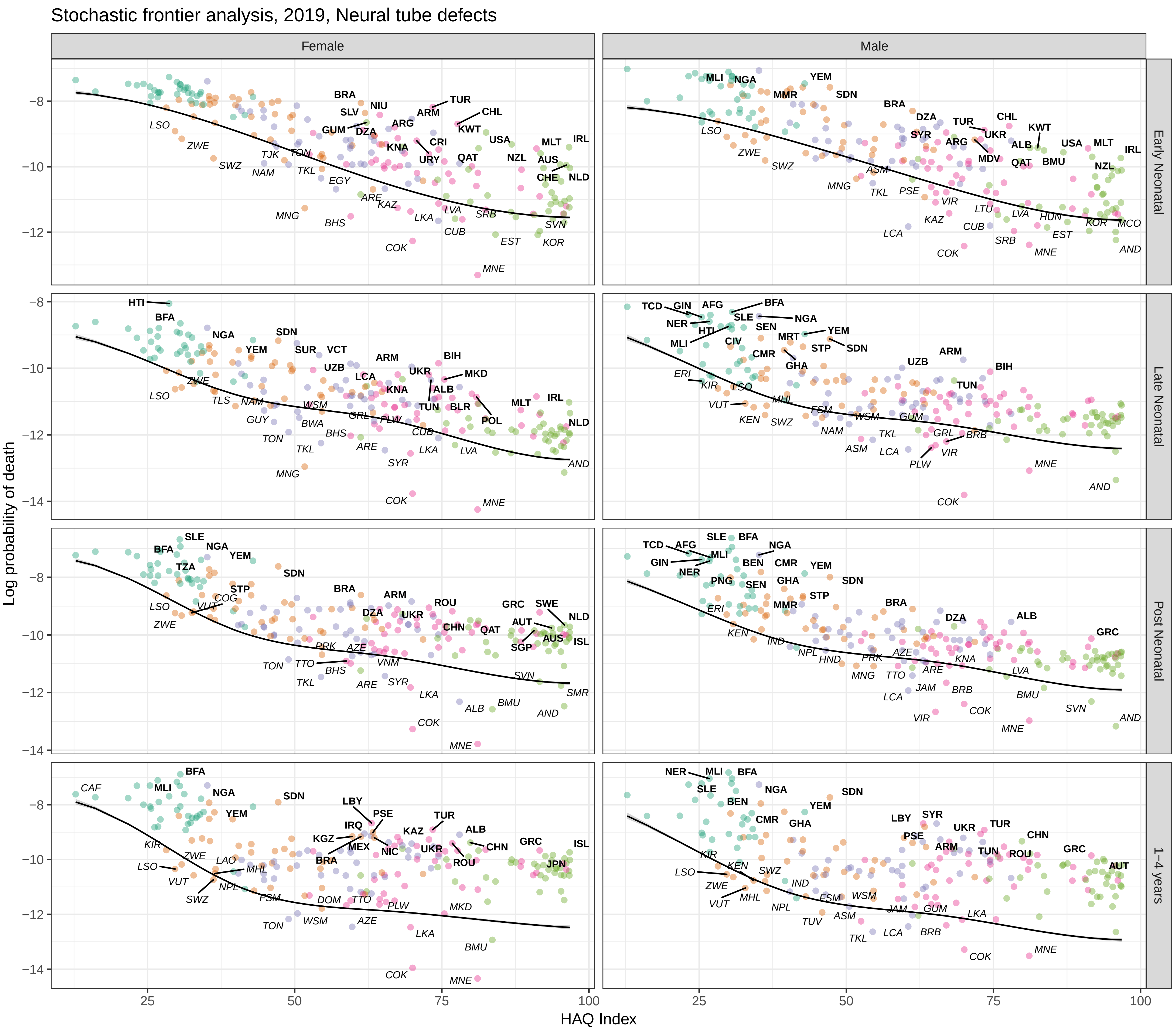

Figure S8
Cause- and age-specific mortality by country and Healthcare Access and Quality (HAQ) Index, with the Survival Potential Frontier (SPF) (black line) and uncertainty interval around the frontier (grey band). Countries are labelled in bold when their ratio to the frontier is in the top 10 percent (performing poorly relative to HAQ Index) and in italics when their ratio to the frontier is in the bottom 10 percent (performing well relative to HAQ Index). These results show the prediction of the cause-specific SPF prior to scaling to the all-cause frontier.

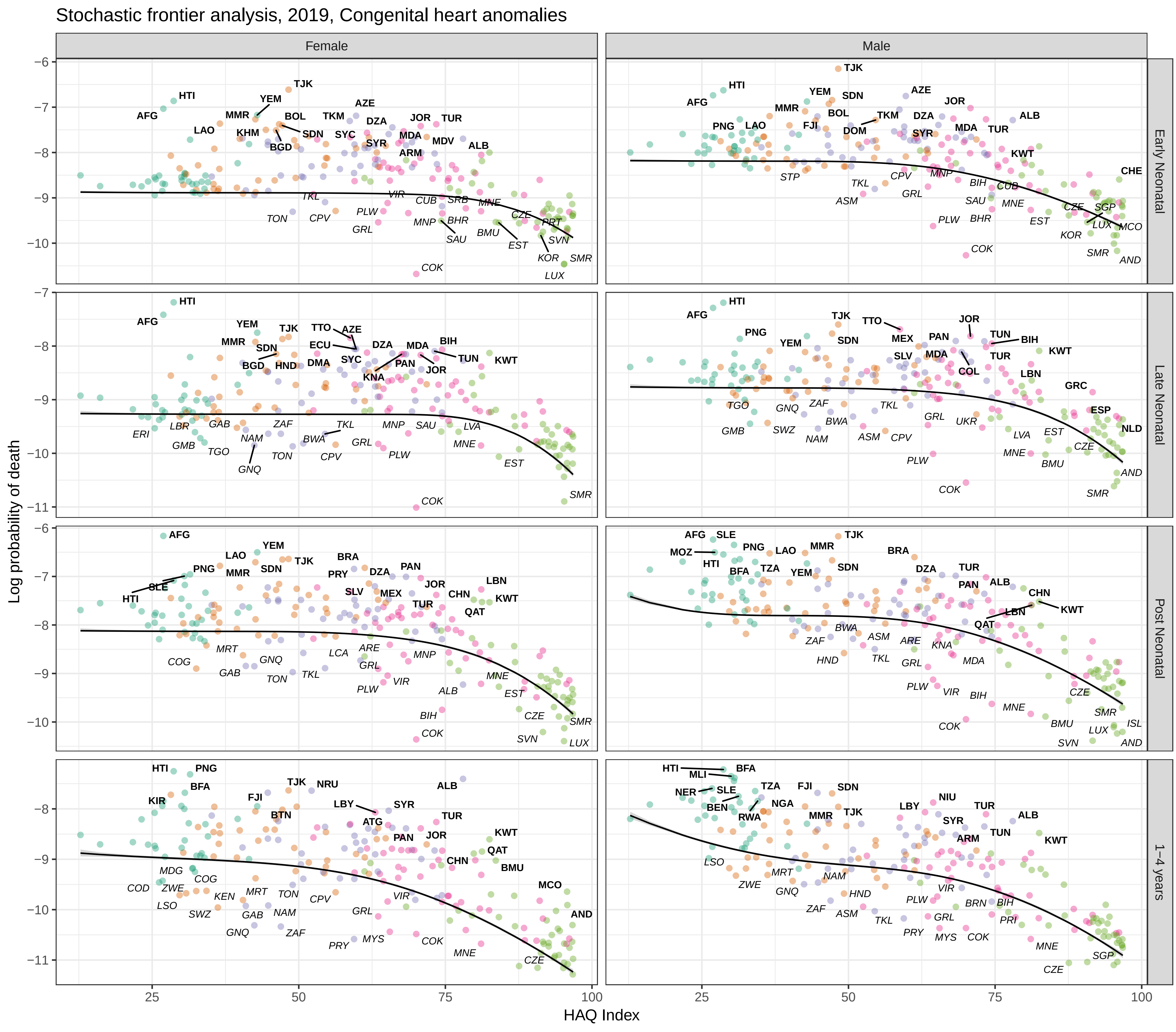

Figure S8
Cause- and age-specific mortality by country and Healthcare Access and Quality (HAQ) Index, with the Survival Potential Frontier (SPF) (black line) and uncertainty interval around the frontier (grey band). Countries are labelled in bold when their ratio to the frontier is in the top 10 percent (performing poorly relative to HAQ Index) and in italics when their ratio to the frontier is in the bottom 10 percent (performing well relative to HAQ Index). These results show the prediction of the cause-specific SPF prior to scaling to the all-cause frontier.

Stochastic frontier analysis, 2019, Orofacial clefts

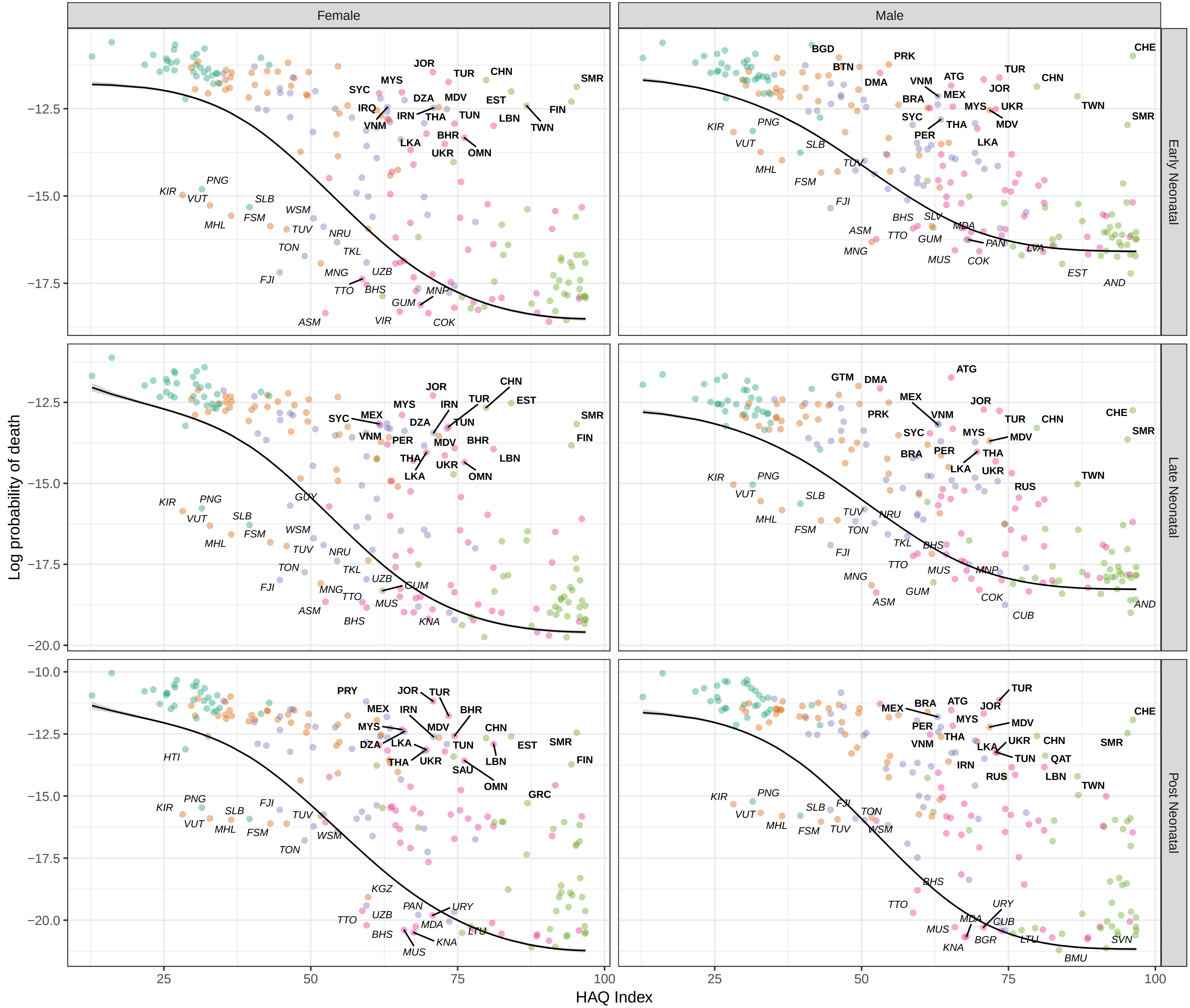

Figure S8
Cause- and age-specific mortality by country and Healthcare Access and Quality (HAQ) Index, with the Survival Potential Frontier (SPF) (black line) and uncertainty interval around the frontier (grey band). Countries are labelled in bold when their ratio to the frontier is in the top 10 percent (performing poorly relative to HAQ Index) and in italics when their ratio to the frontier is in the bottom 10 percent (performing well relative to HAQ Index). These results show the prediction of the cause-specific SPF prior to scaling to the all-cause frontier.

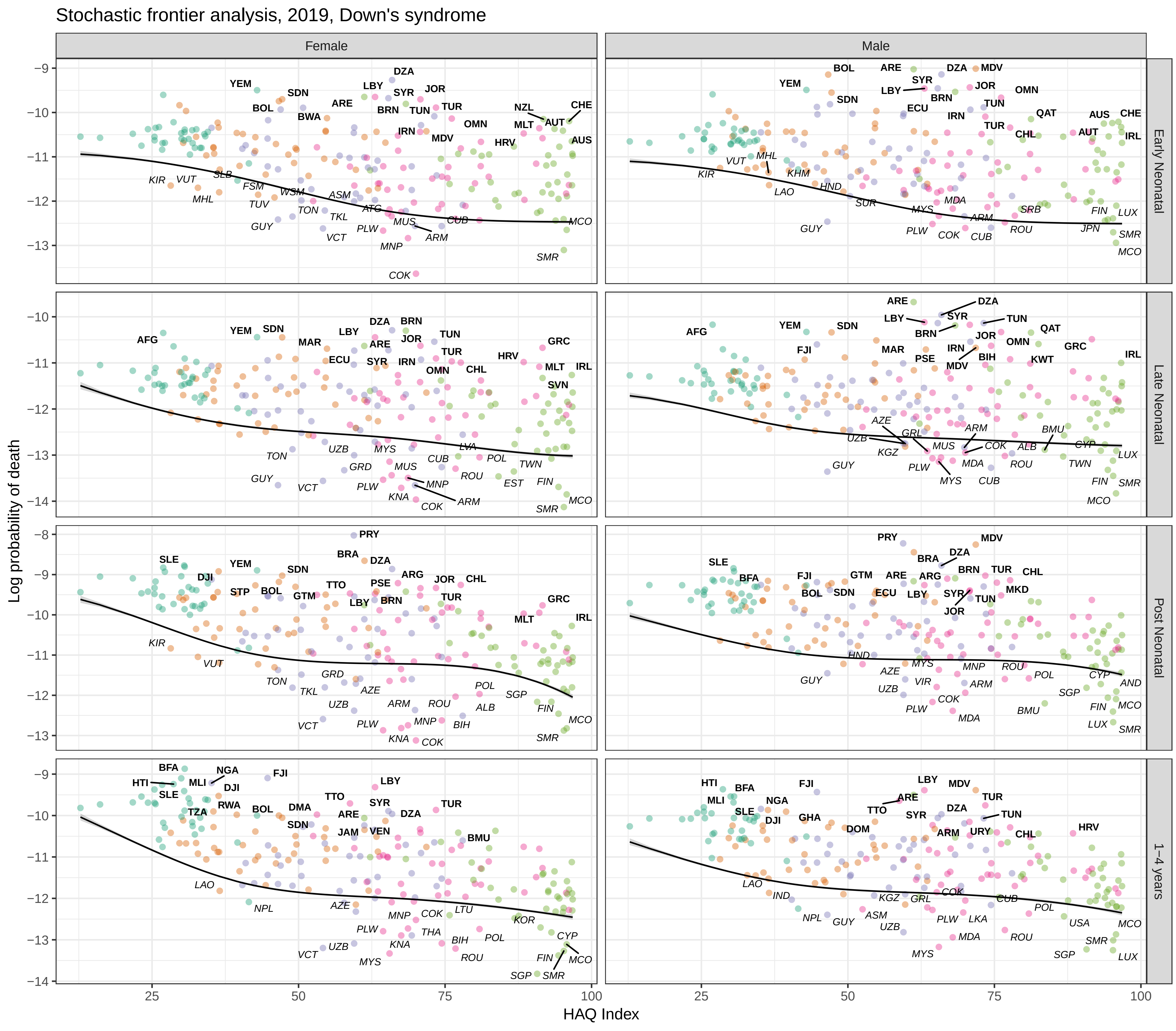

Figure S8
Cause- and age-specific mortality by country and Healthcare Access and Quality (HAQ) Index, with the Survival Potential Frontier (SPF) (black line) and uncertainty interval around the frontier (grey band). Countries are labelled in bold when their ratio to the frontier is in the top 10 percent (performing poorly relative to HAQ Index) and in italics when their ratio to the frontier is in the bottom 10 percent (performing well relative to HAQ Index). These results show the prediction of the cause-specific SPF prior to scaling to the all-cause frontier.

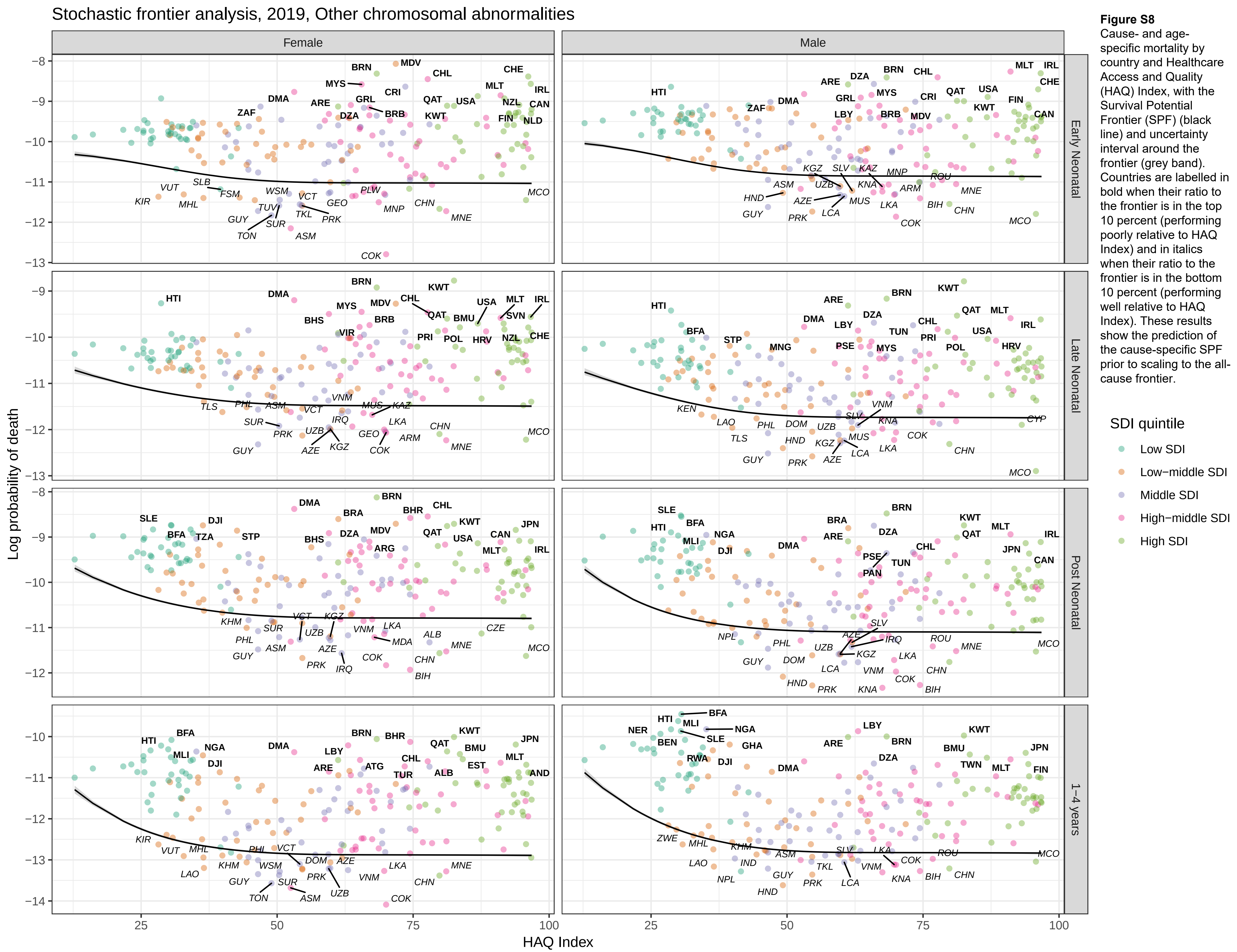

Figure S8
Cause- and age-specific mortality by country and Healthcare Access and Quality (HAQ) Index, with the Survival Potential Frontier (SPF) (black line) and uncertainty interval around the frontier (grey band). Countries are labelled in bold when their ratio to the frontier is in the top 10 percent (performing poorly relative to HAQ Index) and in italics when their ratio to the frontier is in the bottom 10 percent (performing well relative to HAQ Index). These results show the prediction of the cause-specific SPF prior to scaling to the all-cause frontier.

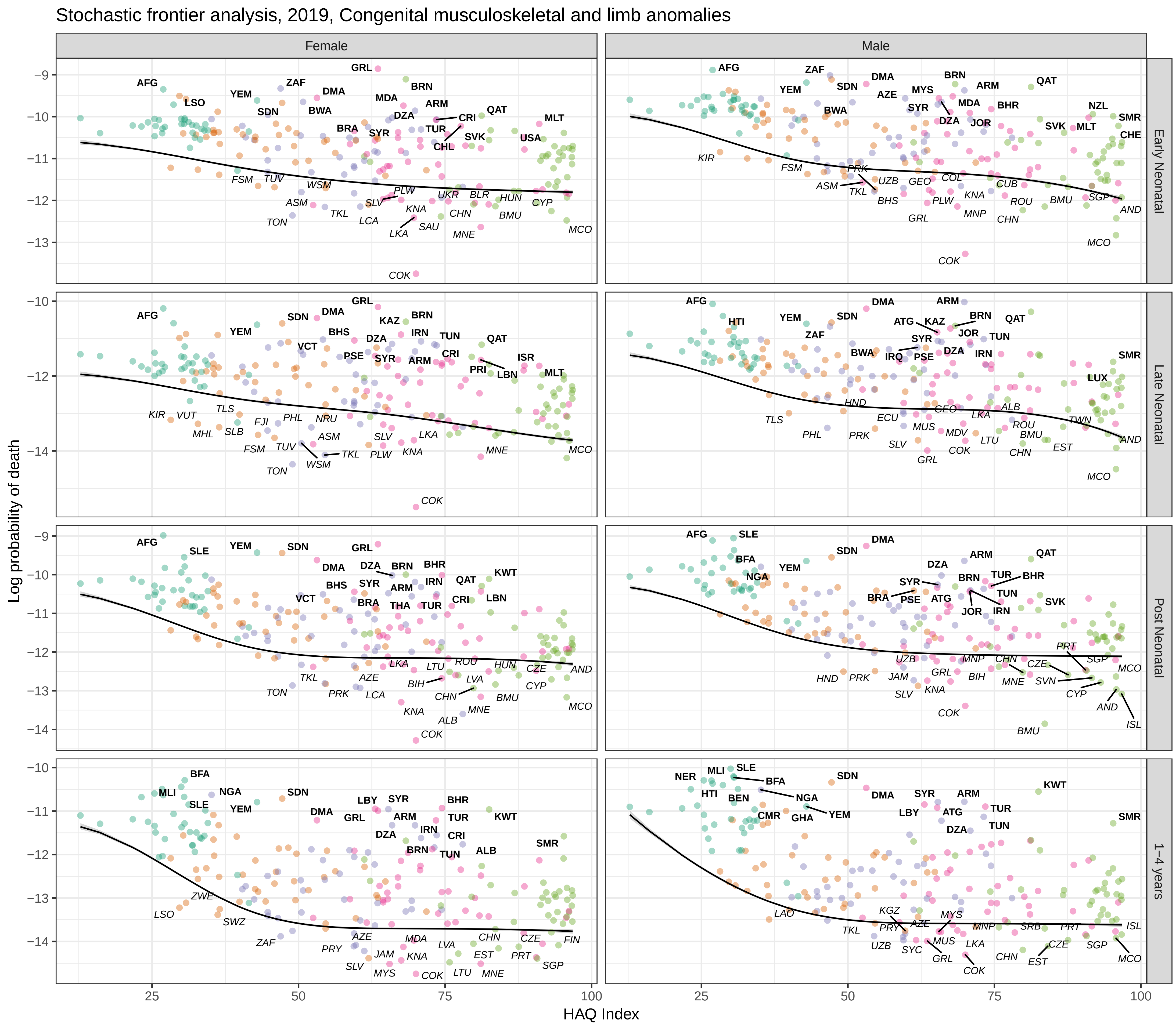

Figure S8
Cause- and age-specific mortality by country and Healthcare Access and Quality (HAQ) Index, with the Survival Potential Frontier (SPF) (black line) and uncertainty interval around the frontier (grey band). Countries are labelled in bold when their ratio to the frontier is in the top 10 percent (performing poorly relative to HAQ Index) and in italics when their ratio to the frontier is in the bottom 10 percent (performing well relative to HAQ Index). These results show the prediction of the cause-specific SPF prior to scaling to the all-cause frontier.

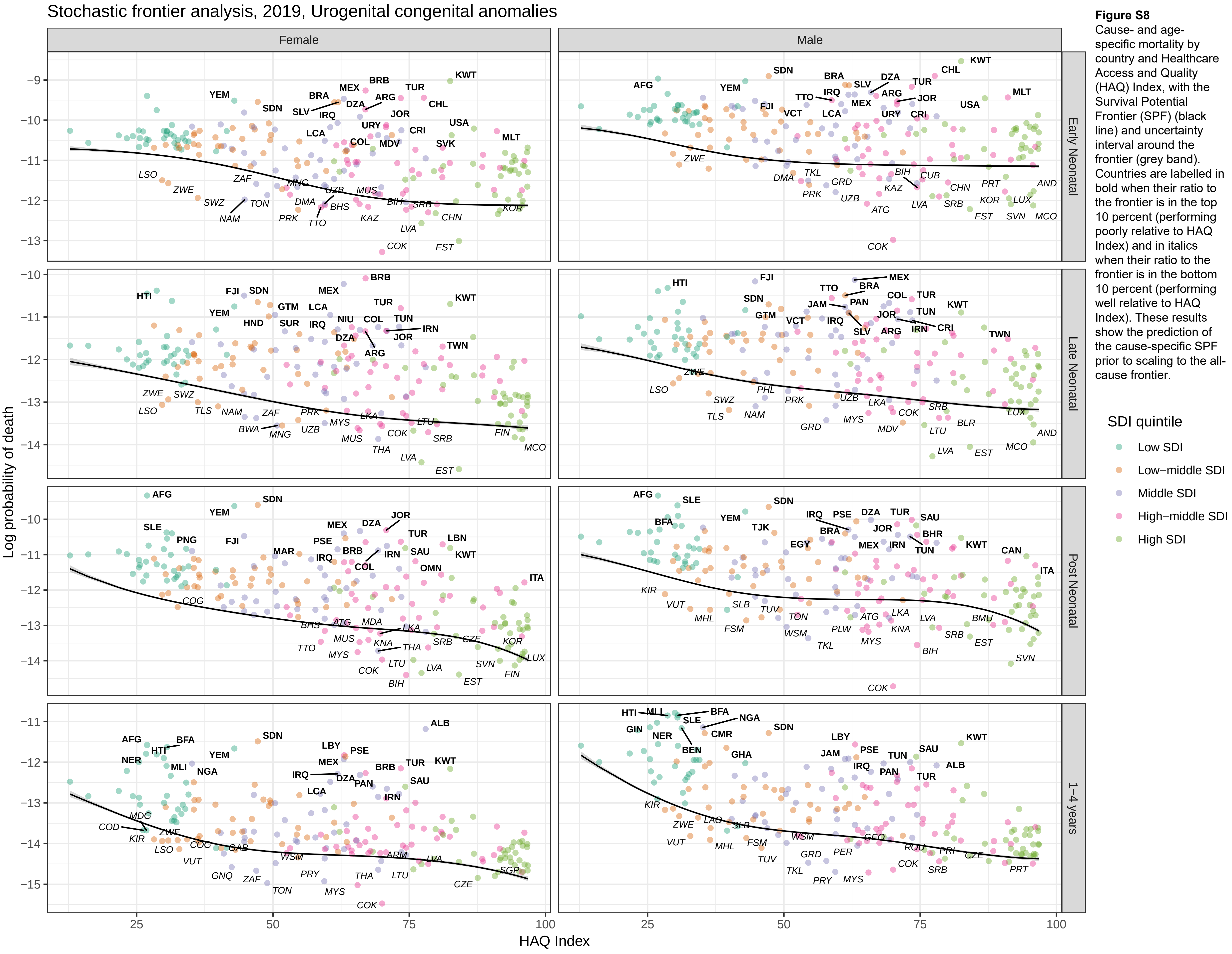

Figure S8
Cause- and age-specific mortality by country and Healthcare Access and Quality (HAQ) Index, with the Survival Potential Frontier (SPF) (black line) and uncertainty interval around the frontier (grey band). Countries are labelled in bold when their ratio to the frontier is in the top 10 percent (performing poorly relative to HAQ Index) and in italics when their ratio to the frontier is in the bottom 10 percent (performing well relative to HAQ Index). These results show the prediction of the cause-specific SPF prior to scaling to the all-cause frontier.

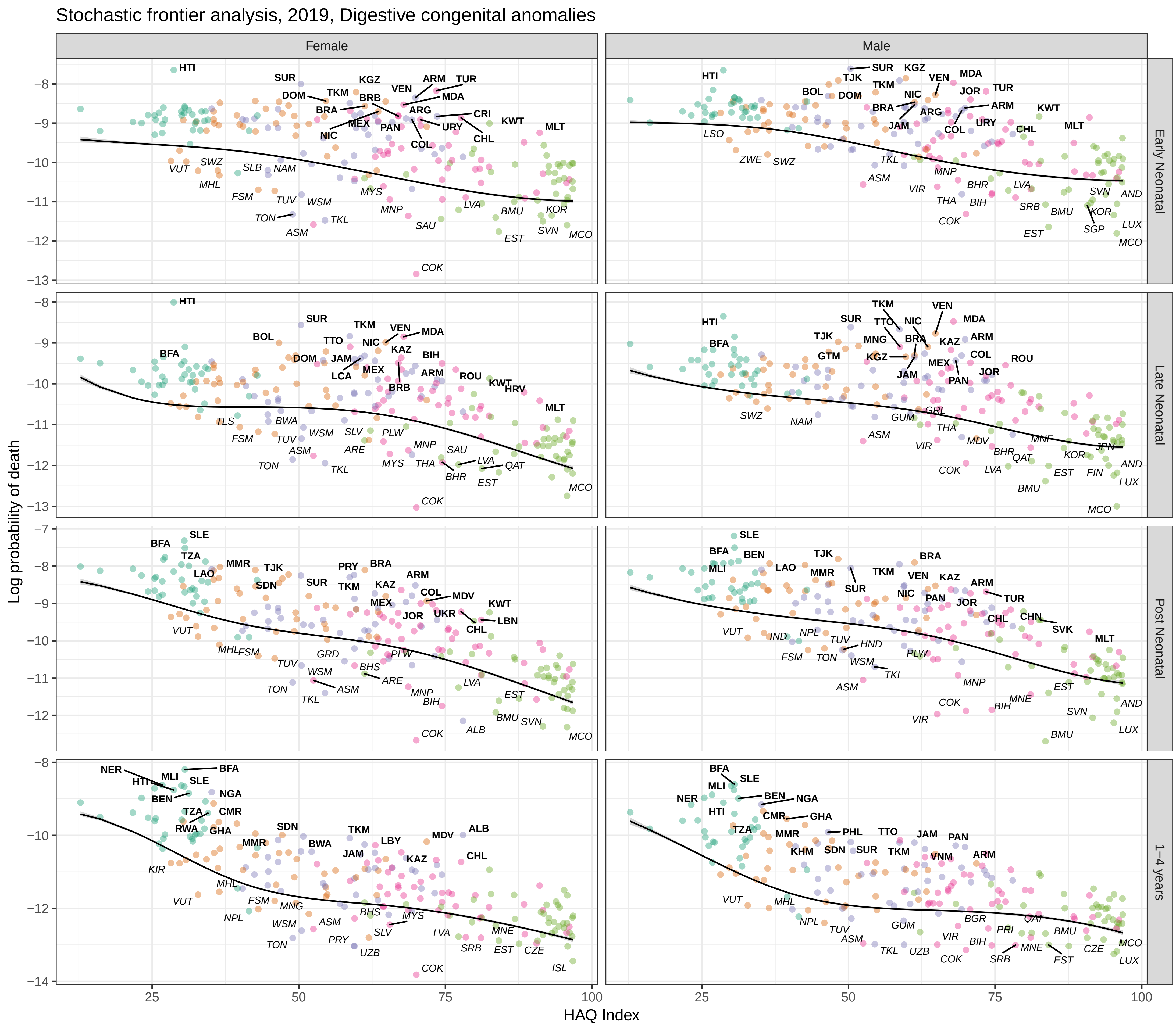

Figure S8
Cause- and age-specific mortality by country and Healthcare Access and Quality (HAQ) Index, with the Survival Potential Frontier (SPF) (black line) and uncertainty interval around the frontier (grey band). Countries are labelled in bold when their ratio to the frontier is in the top 10 percent (performing poorly relative to HAQ Index) and in italics when their ratio to the frontier is in the bottom 10 percent (performing well relative to HAQ Index). These results show the prediction of the cause-specific SPF prior to scaling to the all-cause frontier.

SDI quintile
- Low SDI
- Low−middle SDI
- Middle SDI
- High−middle SDI
- High SDI

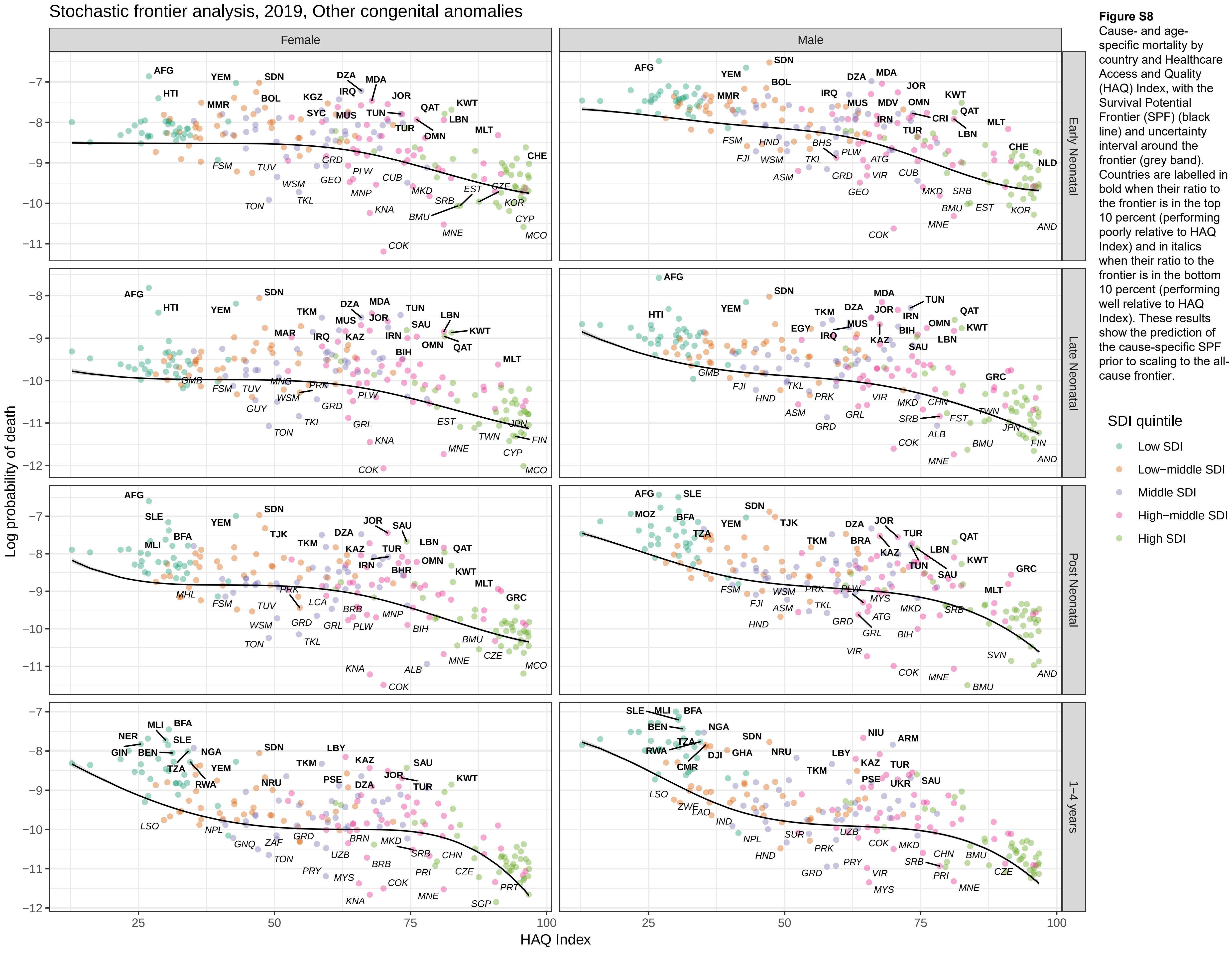

Figure S8
Cause- and age-specific mortality by country and Healthcare Access and Quality (HAQ) Index, with the Survival Potential Frontier (SPF) (black line) and uncertainty interval around the frontier (grey band). Countries are labelled in bold when their ratio to the frontier is in the top 10 percent (performing poorly relative to HAQ Index) and in italics when their ratio to the frontier is in the bottom 10 percent (performing well relative to HAQ Index). These results show the prediction of the cause-specific SPF prior to scaling to the all-cause frontier.

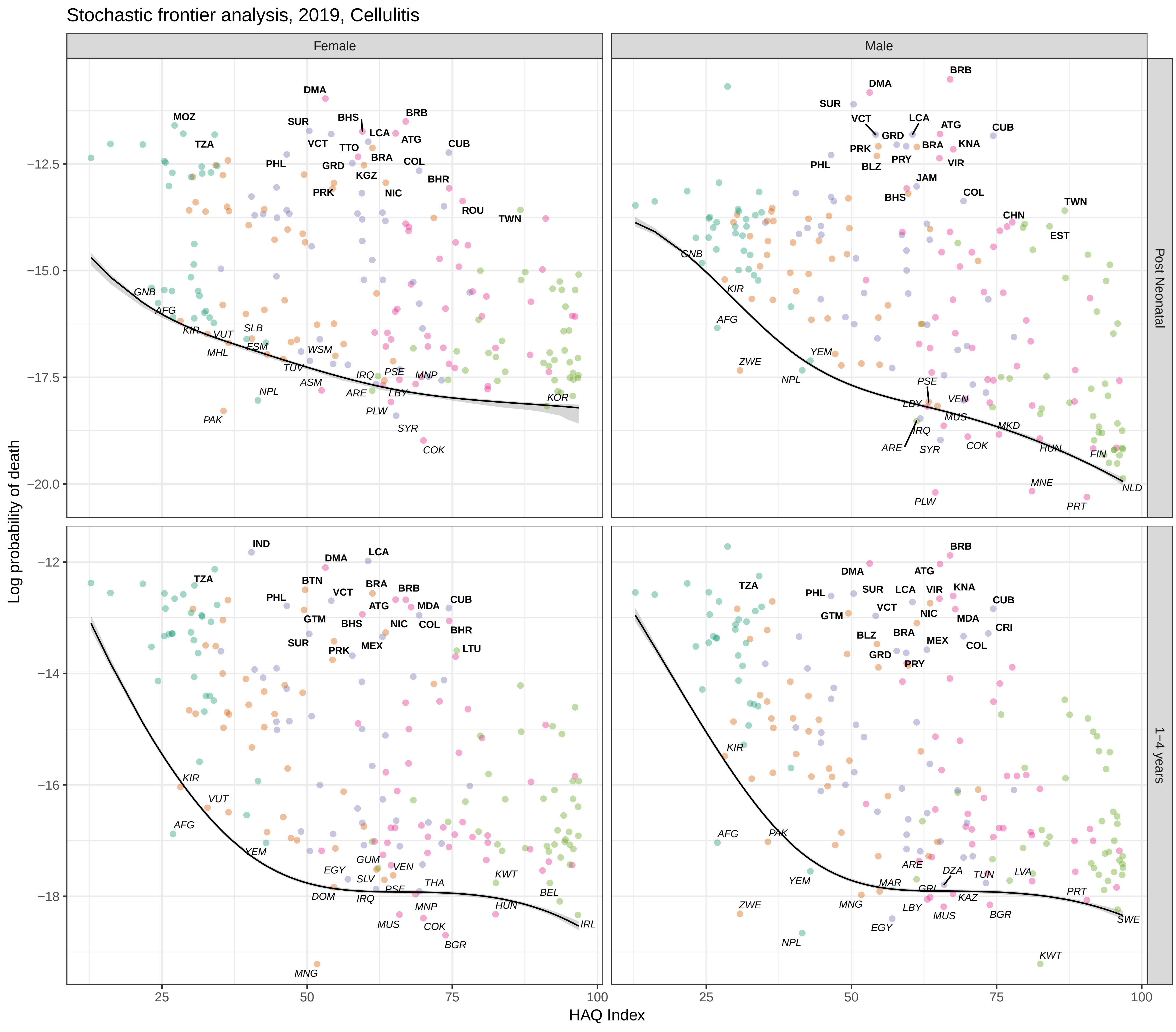

Figure S8
Cause- and age-specific mortality by country and Healthcare Access and Quality (HAQ) Index, with the Survival Potential Frontier (SPF) (black line) and uncertainty interval around the frontier (grey band). Countries are labelled in bold when their ratio to the frontier is in the top 10 percent (performing poorly relative to HAQ Index) and in italics when their ratio to the frontier is in the bottom 10 percent (performing well relative to HAQ Index). These results show the prediction of the cause-specific SPF prior to scaling to the all-cause frontier.

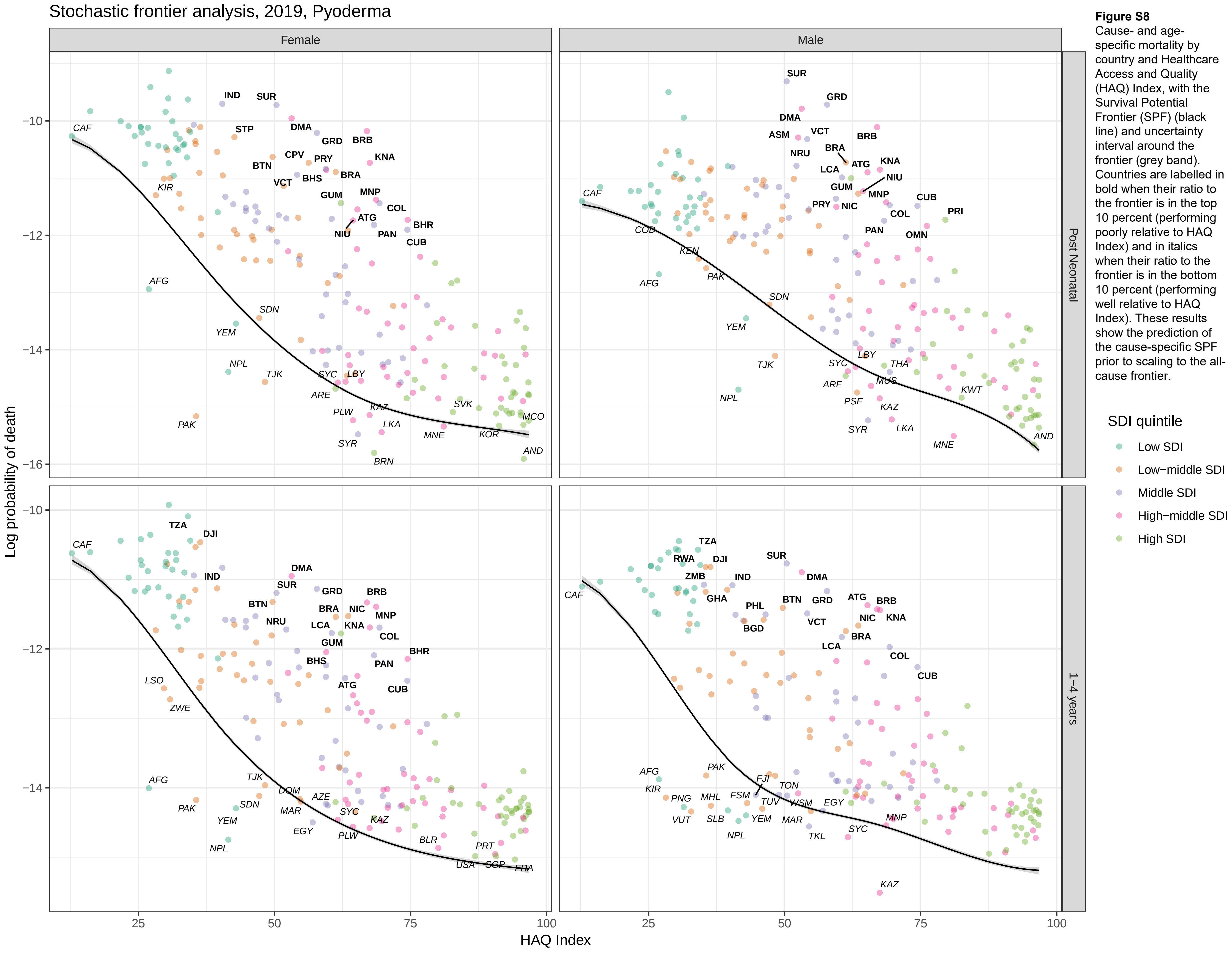

Figure S8
Cause- and age-specific mortality by country and Healthcare Access and Quality (HAQ) Index, with the Survival Potential Frontier (SPF) (black line) and uncertainty interval around the frontier (grey band). Countries are labelled in bold when their ratio to the frontier is in the top 10 percent (performing poorly relative to HAQ Index) and in italics when their ratio to the frontier is in the bottom 10 percent (performing well relative to HAQ Index). These results show the prediction of the cause-specific SPF prior to scaling to the all-cause frontier.

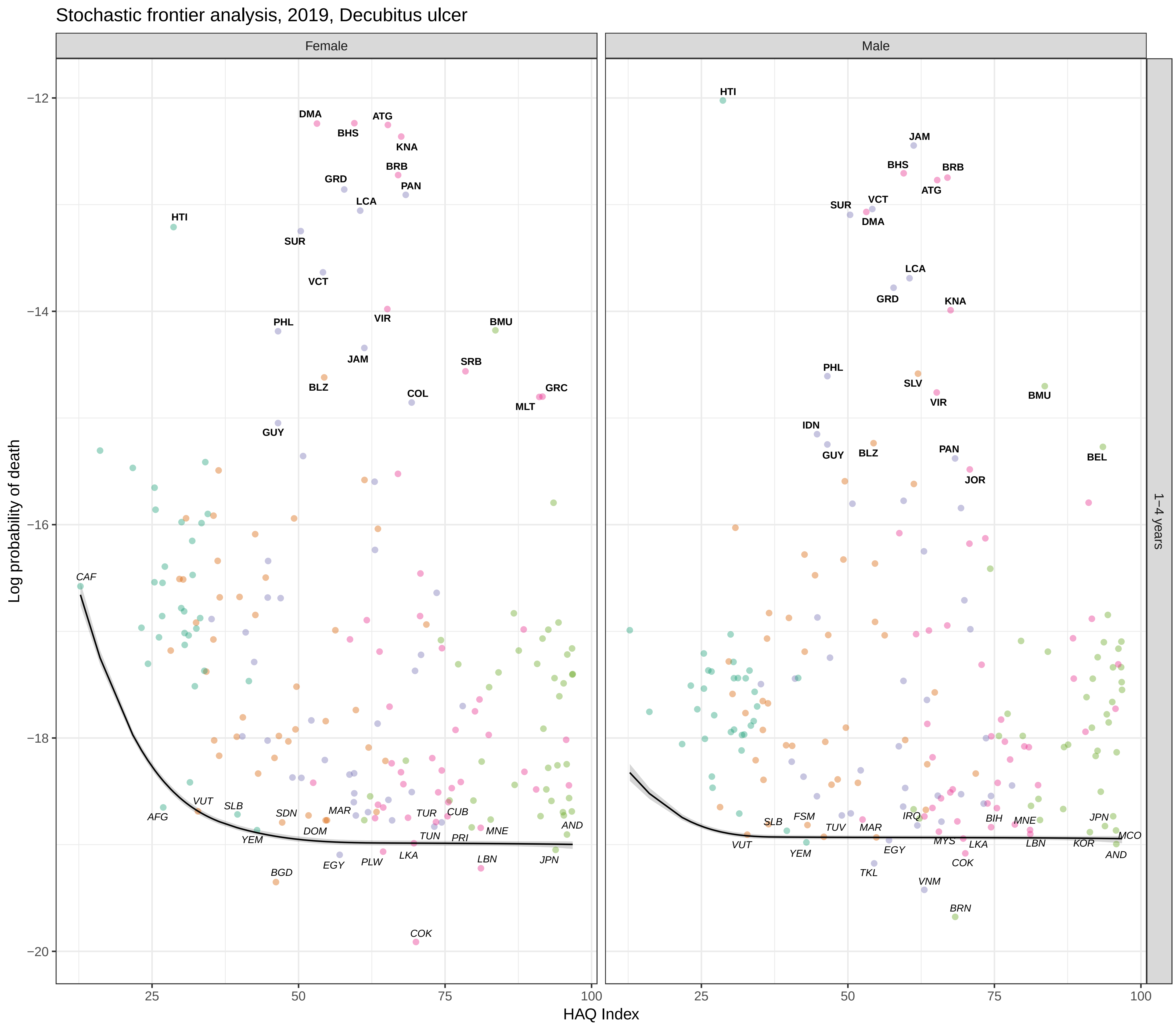

Figure S8
Cause- and age-specific mortality by country and Healthcare Access and Quality (HAQ) Index, with the Survival Potential Frontier (SPF) (black line) and uncertainty interval around the frontier (grey band). Countries are labelled in bold when their ratio to the frontier is in the top 10 percent (performing poorly relative to HAQ Index) and in italics when their ratio to the frontier is in the bottom 10 percent (performing well relative to HAQ Index). These results show the prediction of the cause-specific SPF prior to scaling to the all-cause frontier.

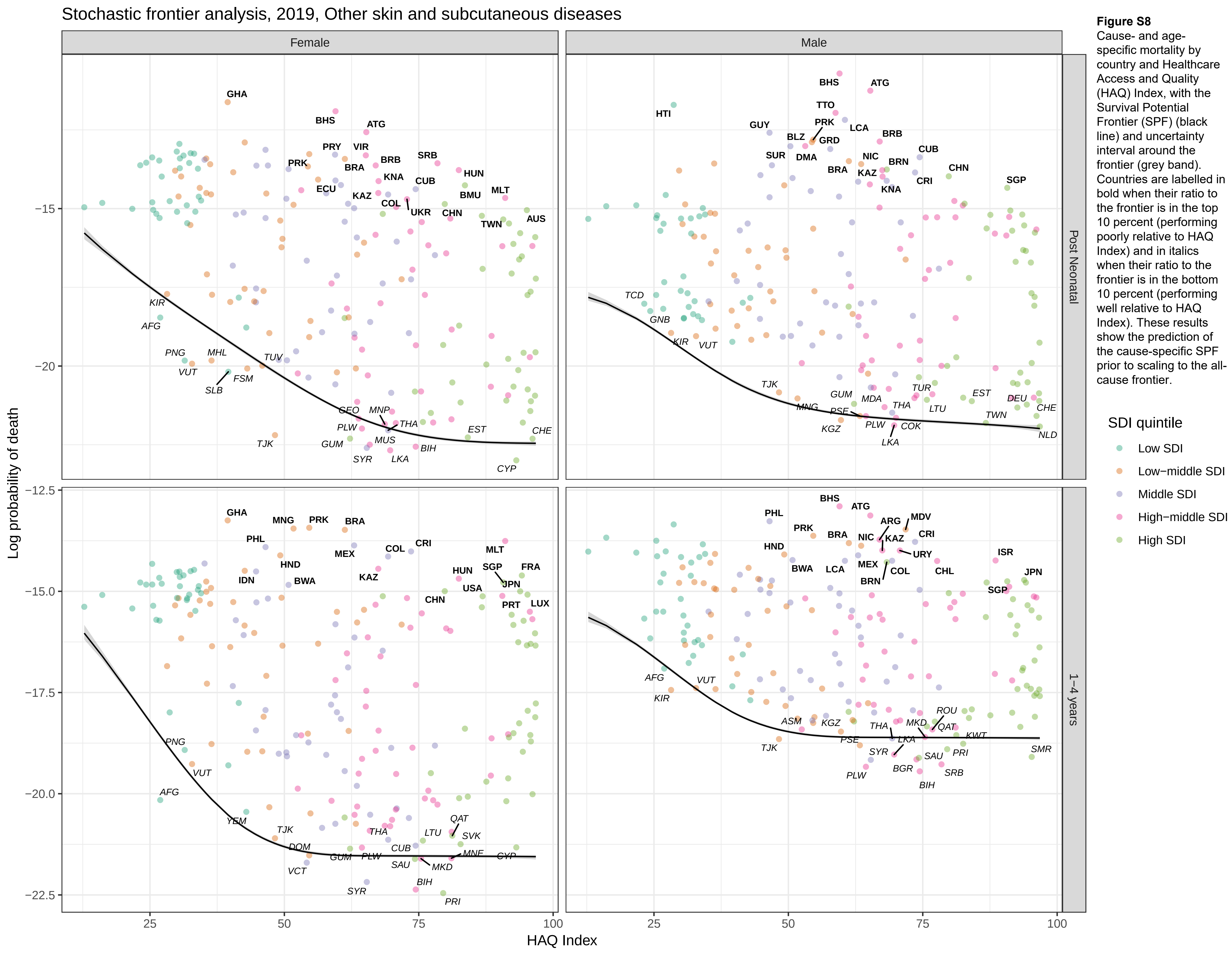

Figure S8
Cause- and age-specific mortality by country and Healthcare Access and Quality (HAQ) Index, with the Survival Potential Frontier (SPF) (black line) and uncertainty interval around the frontier (grey band). Countries are labelled in bold when their ratio to the frontier is in the top 10 percent (performing poorly relative to HAQ Index) and in italics when their ratio to the frontier is in the bottom 10 percent (performing well relative to HAQ Index). These results show the prediction of the cause-specific SPF prior to scaling to the all-cause frontier.

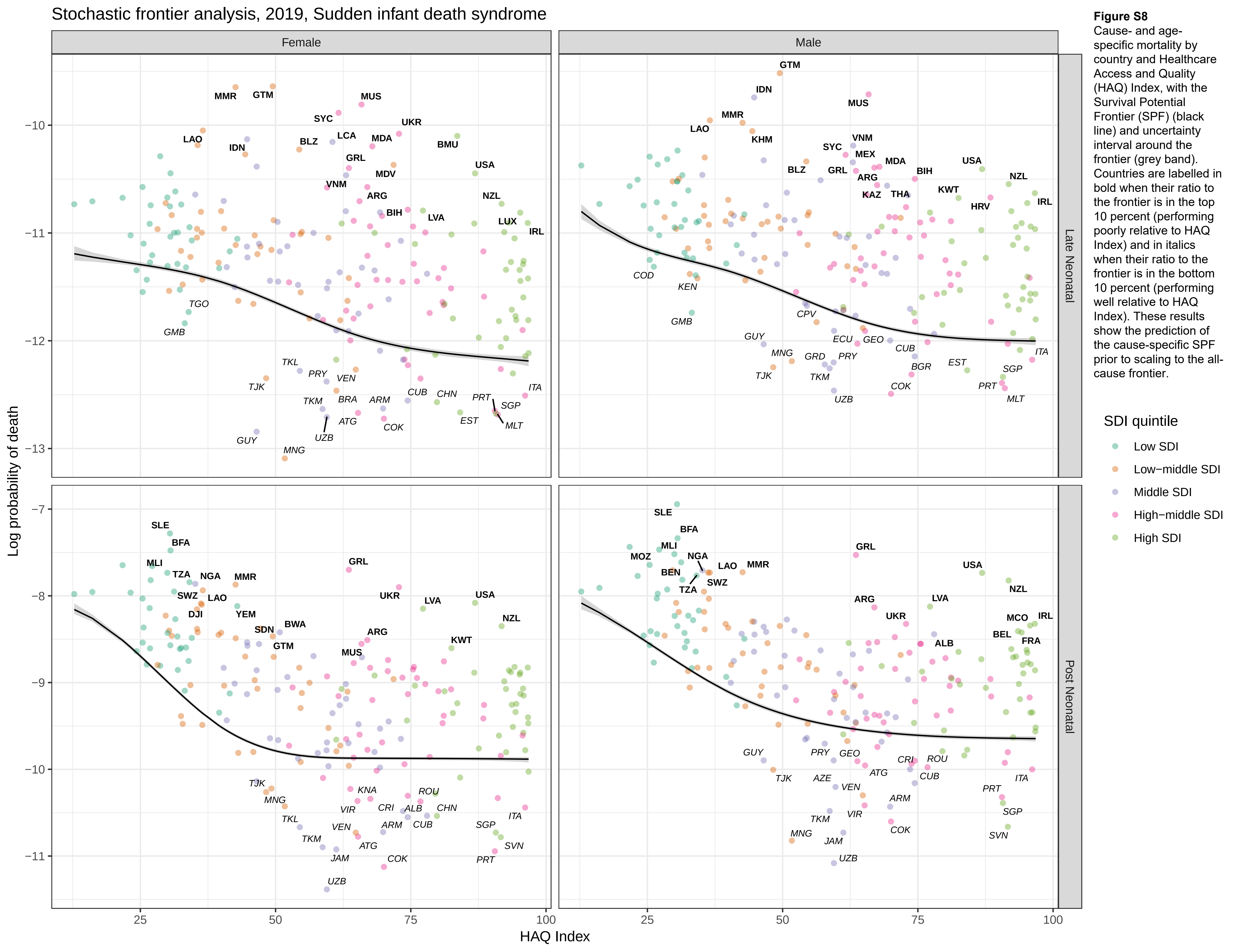

Figure S8
Cause- and age-specific mortality by country and Healthcare Access and Quality (HAQ) Index, with the Survival Potential Frontier (SPF) (black line) and uncertainty interval around the frontier (grey band). Countries are labelled in bold when their ratio to the frontier is in the top 10 percent (performing poorly relative to HAQ Index) and in italics when their ratio to the frontier is in the bottom 10 percent (performing well relative to HAQ Index). These results show the prediction of the cause-specific SPF prior to scaling to the all-cause frontier.

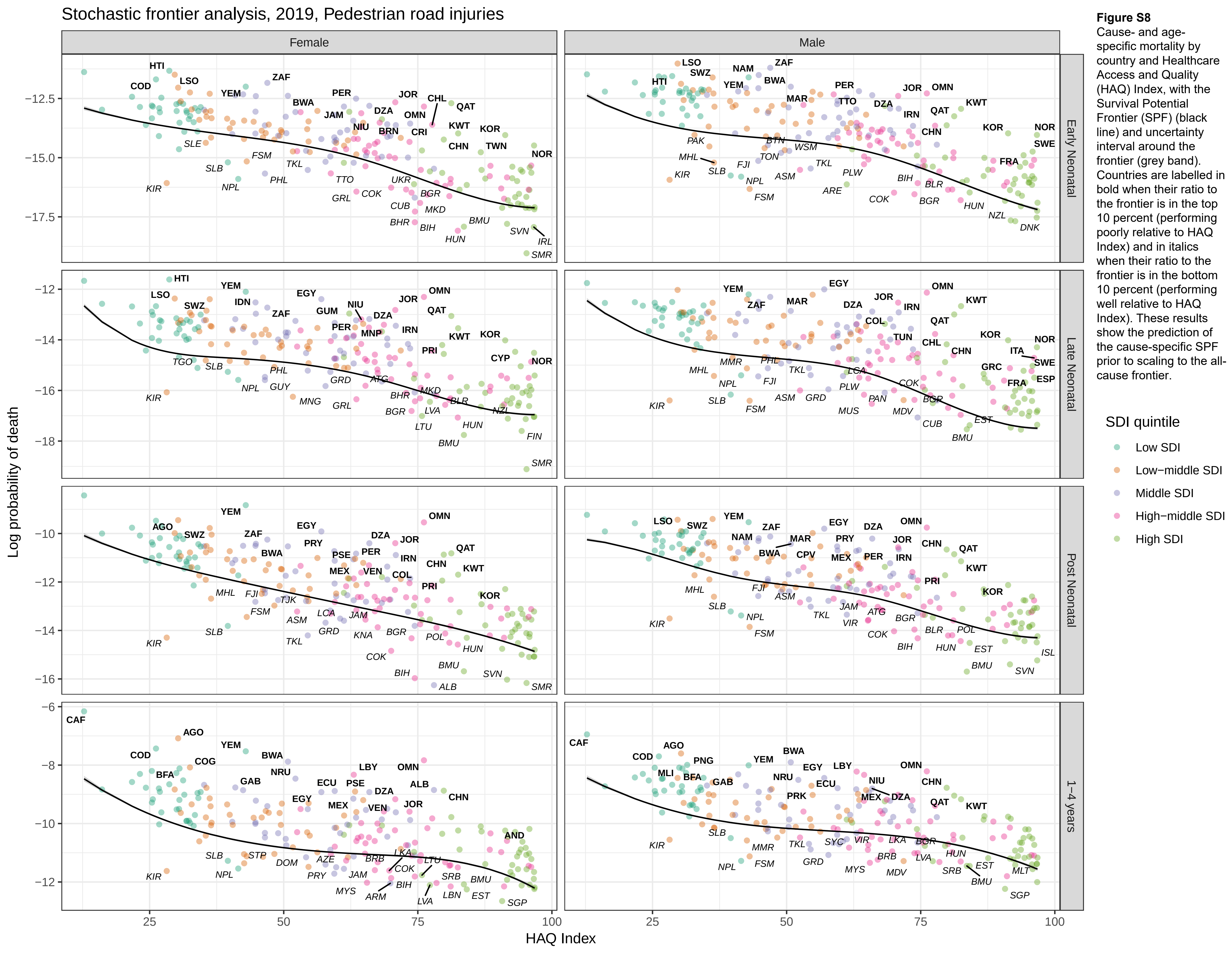

Figure S8
Cause- and age-specific mortality by country and Healthcare Access and Quality (HAQ) Index, with the Survival Potential Frontier (SPF) (black line) and uncertainty interval around the frontier (grey band). Countries are labelled in bold when their ratio to the frontier is in the top 10 percent (performing poorly relative to HAQ Index) and in italics when their ratio to the frontier is in the bottom 10 percent (performing well relative to HAQ Index). These results show the prediction of the cause-specific SPF prior to scaling to the all-cause frontier.

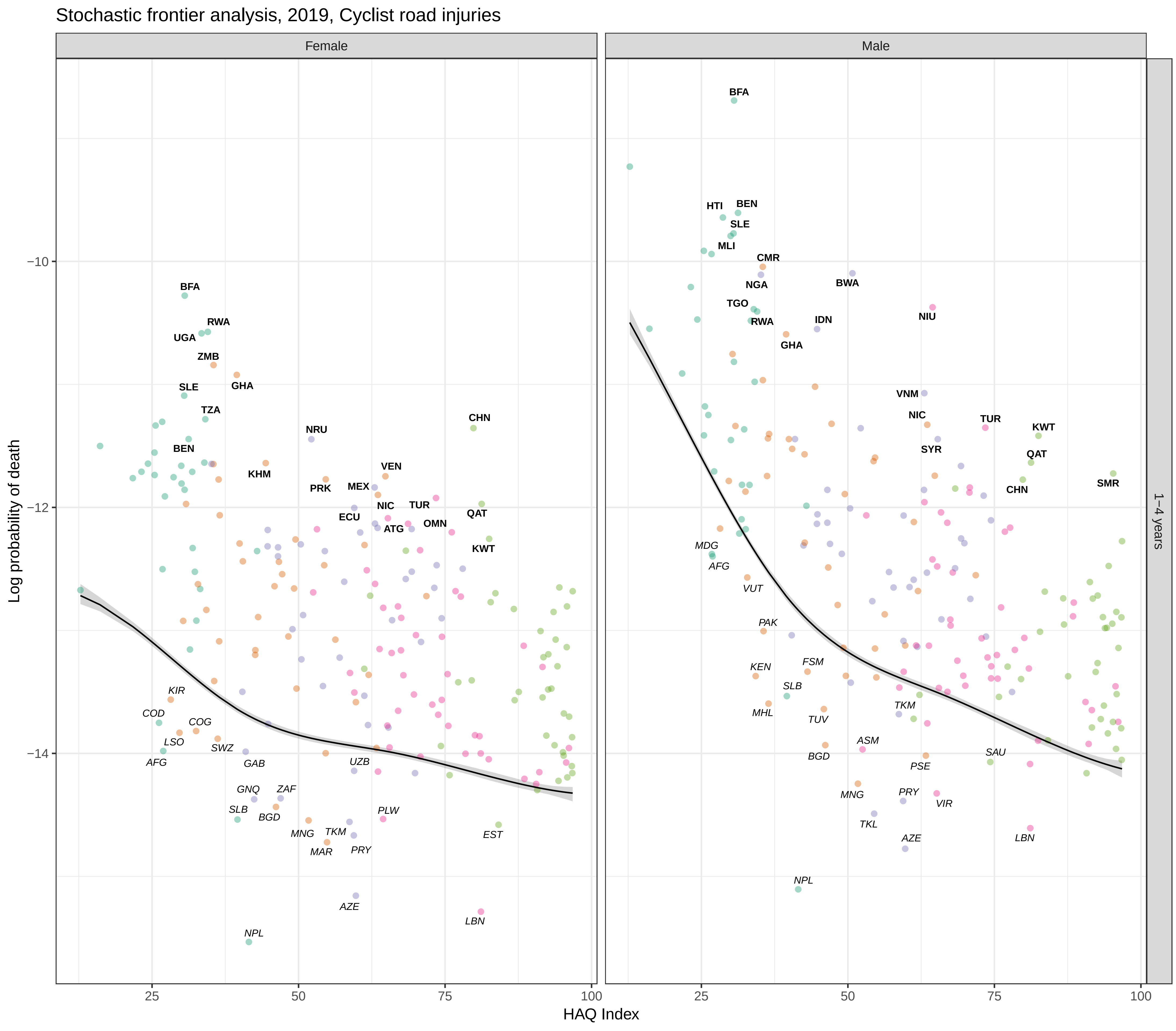

Figure S8
Cause- and age-specific mortality by country and Healthcare Access and Quality (HAQ) Index, with the Survival Potential Frontier (SPF) (black line) and uncertainty interval around the frontier (grey band). Countries are labelled in bold when their ratio to the frontier is in the top 10 percent (performing poorly relative to HAQ Index) and in italics when their ratio to the frontier is in the bottom 10 percent (performing well relative to HAQ Index). These results show the prediction of the cause-specific SPF prior to scaling to the all-cause frontier.

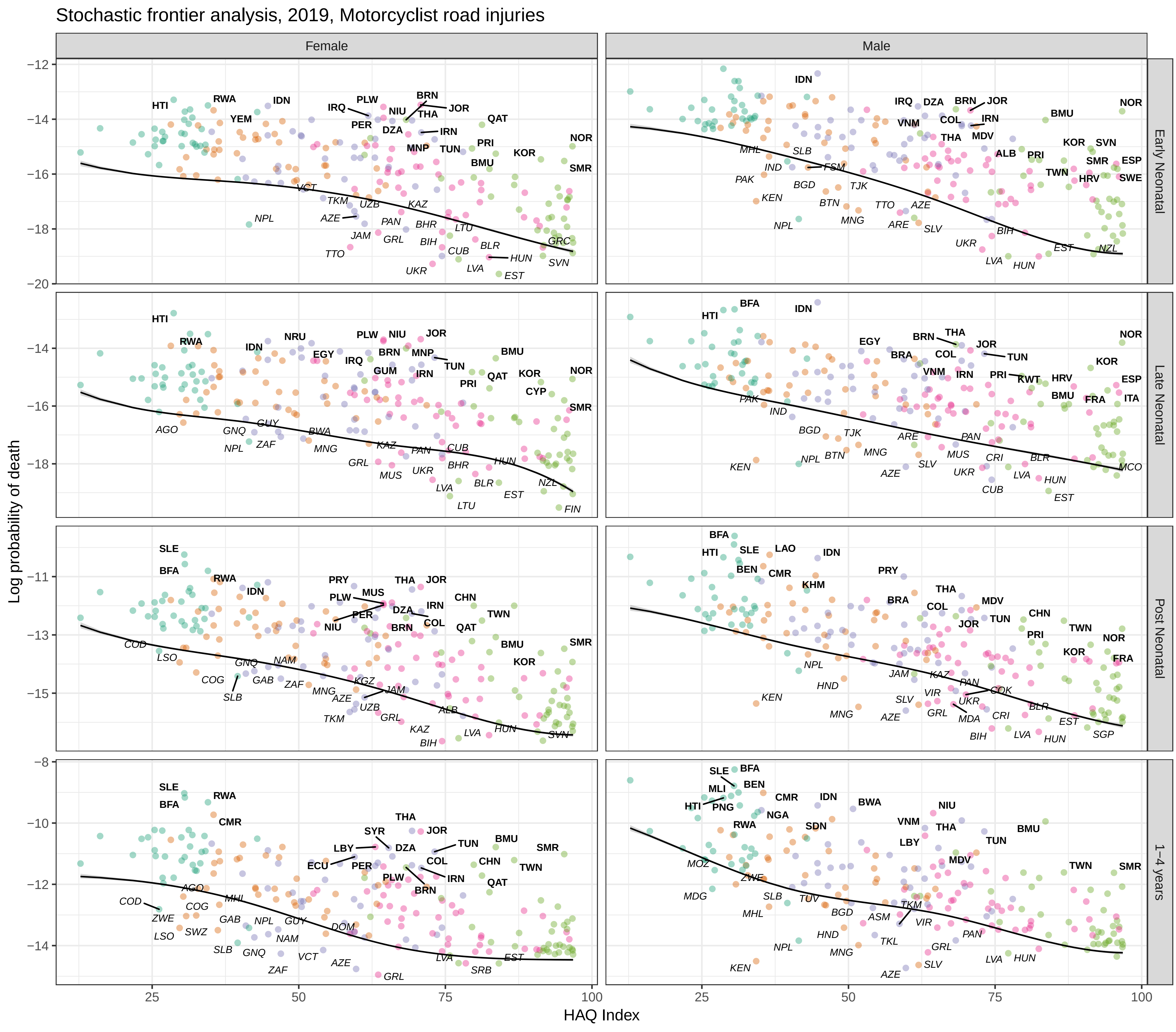

Figure S8
Cause- and age-specific mortality by country and Healthcare Access and Quality (HAQ) Index, with the Survival Potential Frontier (SPF) (black line) and uncertainty interval around the frontier (grey band). Countries are labelled in bold when their ratio to the frontier is in the top 10 percent (performing poorly relative to HAQ Index) and in italics when their ratio to the frontier is in the bottom 10 percent (performing well relative to HAQ Index). These results show the prediction of the cause-specific SPF prior to scaling to the all-cause frontier.

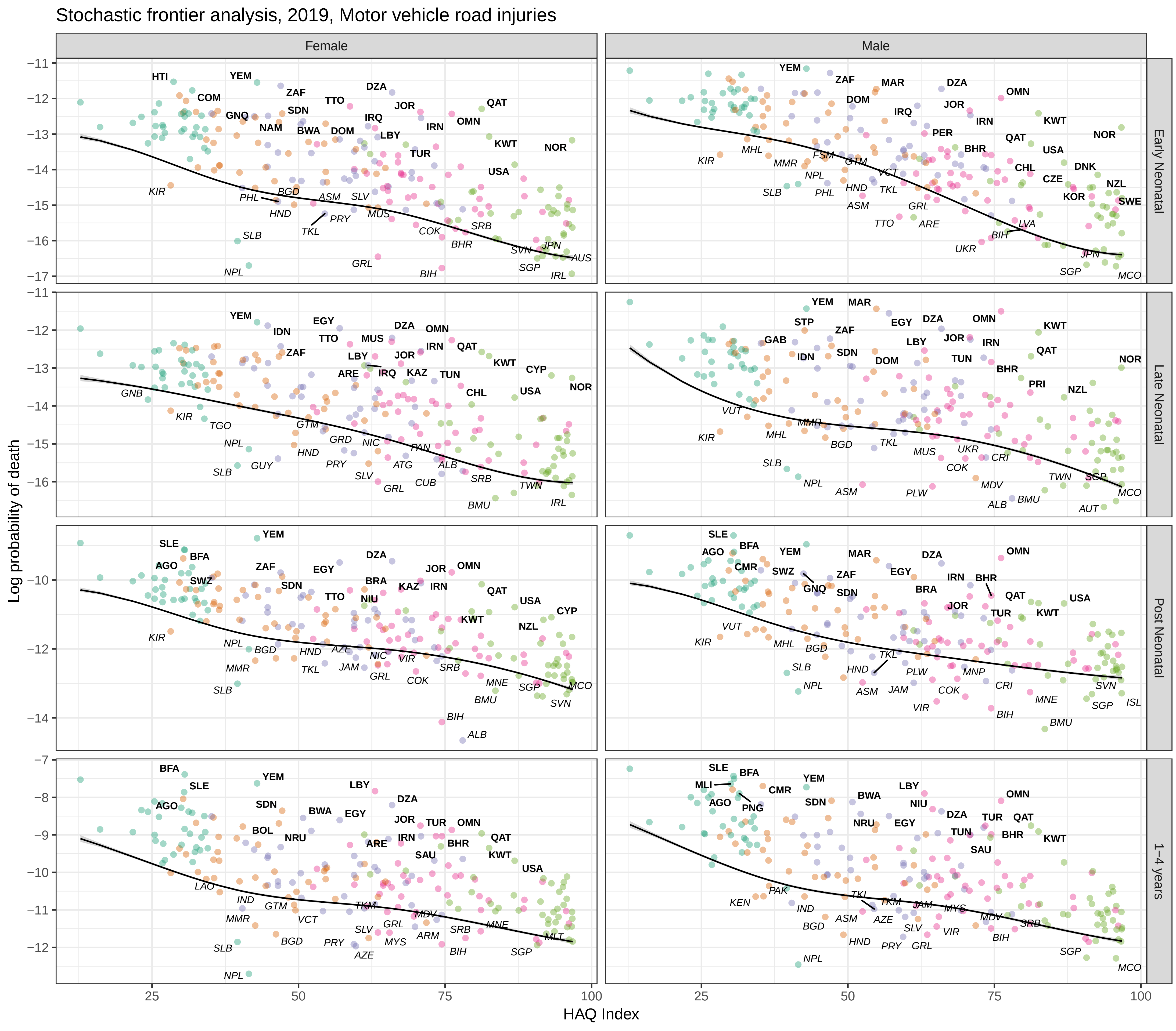

Figure S8
Cause- and age-specific mortality by country and Healthcare Access and Quality (HAQ) Index, with the Survival Potential Frontier (SPF) (black line) and uncertainty interval around the frontier (grey band). Countries are labelled in bold when their ratio to the frontier is in the top 10 percent (performing poorly relative to HAQ Index) and in italics when their ratio to the frontier is in the bottom 10 percent (performing well relative to HAQ Index). These results show the prediction of the cause-specific SPF prior to scaling to the all-cause frontier.

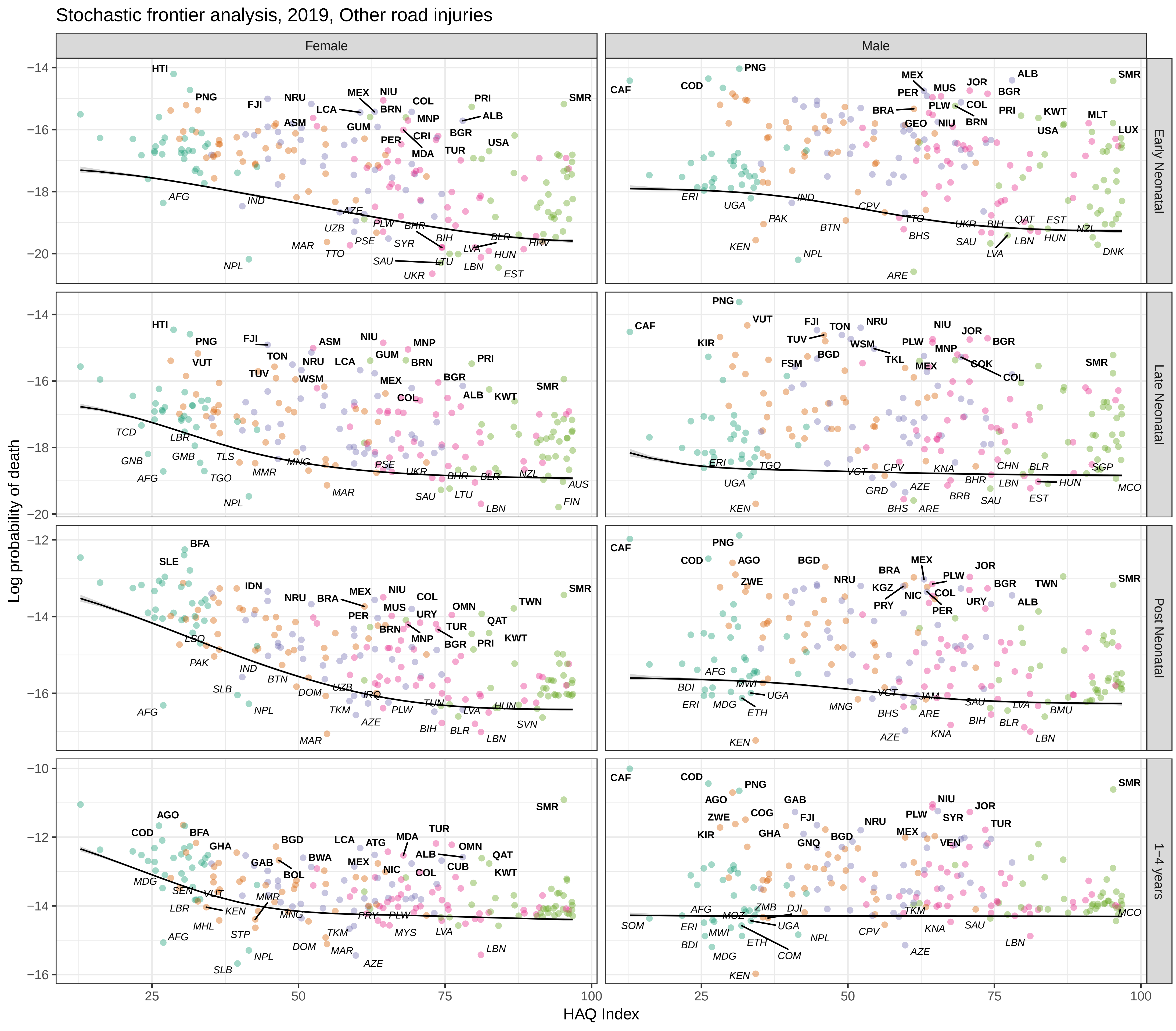

Figure S8
Cause- and age-specific mortality by country and Healthcare Access and Quality (HAQ) Index, with the Survival Potential Frontier (SPF) (black line) and uncertainty interval around the frontier (grey band). Countries are labelled in bold when their ratio to the frontier is in the top 10 percent (performing poorly relative to HAQ Index) and in italics when their ratio to the frontier is in the bottom 10 percent (performing well relative to HAQ Index). These results show the prediction of the cause-specific SPF prior to scaling to the all-cause frontier.

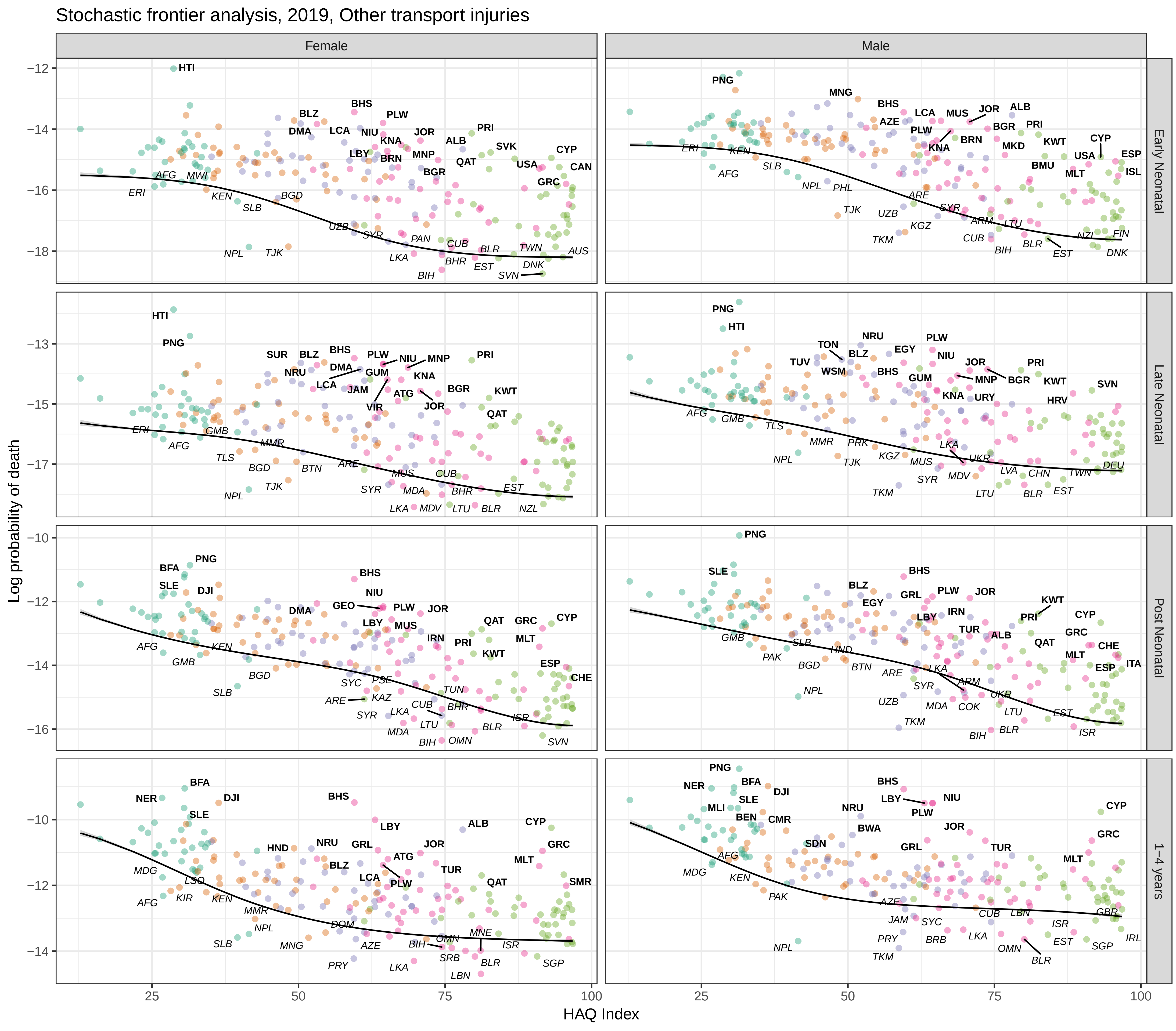

Figure S8
Cause- and age-specific mortality by country and Healthcare Access and Quality (HAQ) Index, with the Survival Potential Frontier (SPF) (black line) and uncertainty interval around the frontier (grey band). Countries are labelled in bold when their ratio to the frontier is in the top 10 percent (performing poorly relative to HAQ Index) and in italics when their ratio to the frontier is in the bottom 10 percent (performing well relative to HAQ Index). These results show the prediction of the cause-specific SPF prior to scaling to the all-cause frontier.

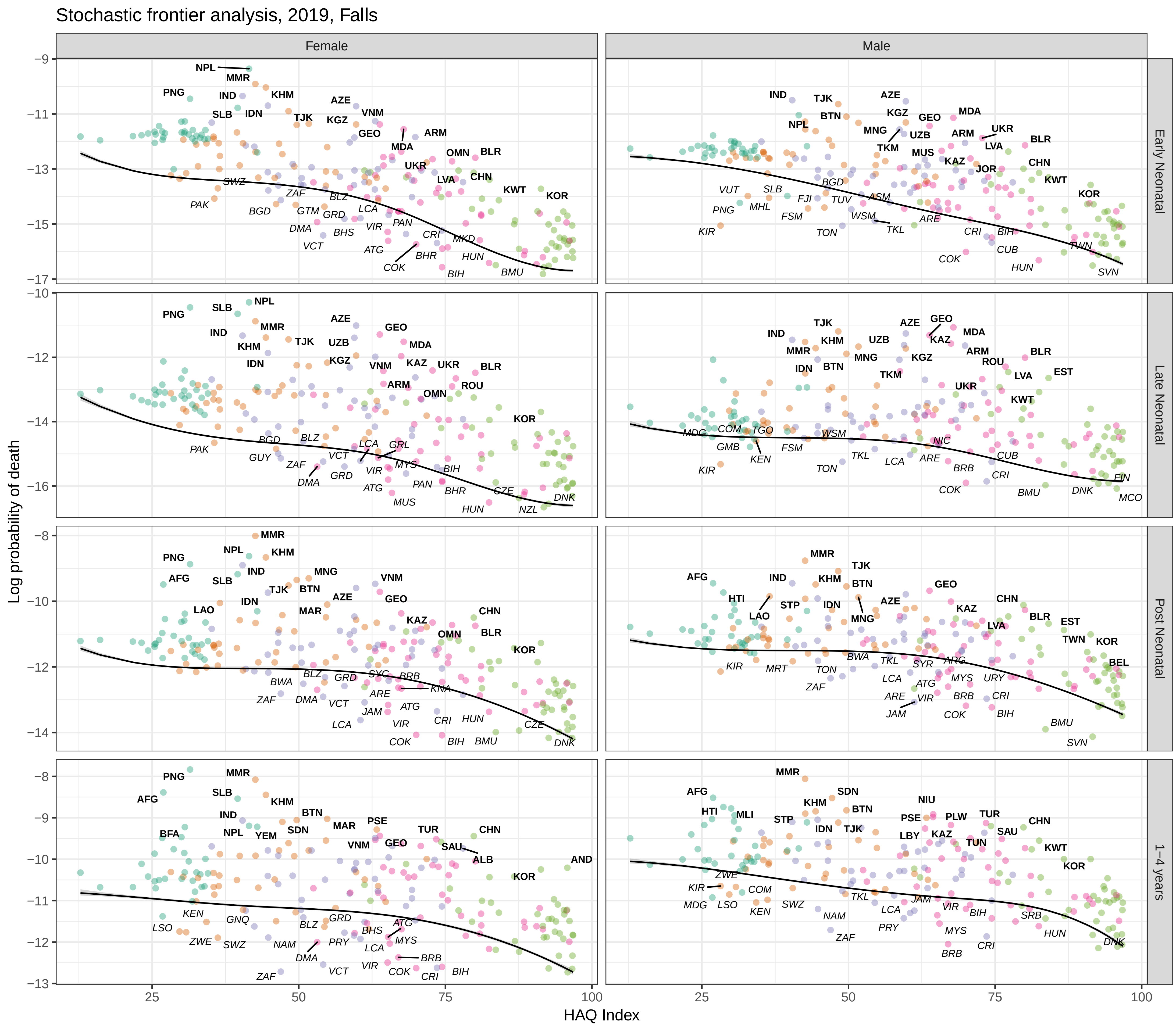

Figure S8
Cause- and age-specific mortality by country and Healthcare Access and Quality (HAQ) Index, with the Survival Potential Frontier (SPF) (black line) and uncertainty interval around the frontier (grey band). Countries are labelled in bold when their ratio to the frontier is in the top 10 percent (performing poorly relative to HAQ Index) and in italics when their ratio to the frontier is in the bottom 10 percent (performing well relative to HAQ Index). These results show the prediction of the cause-specific SPF prior to scaling to the all-cause frontier.

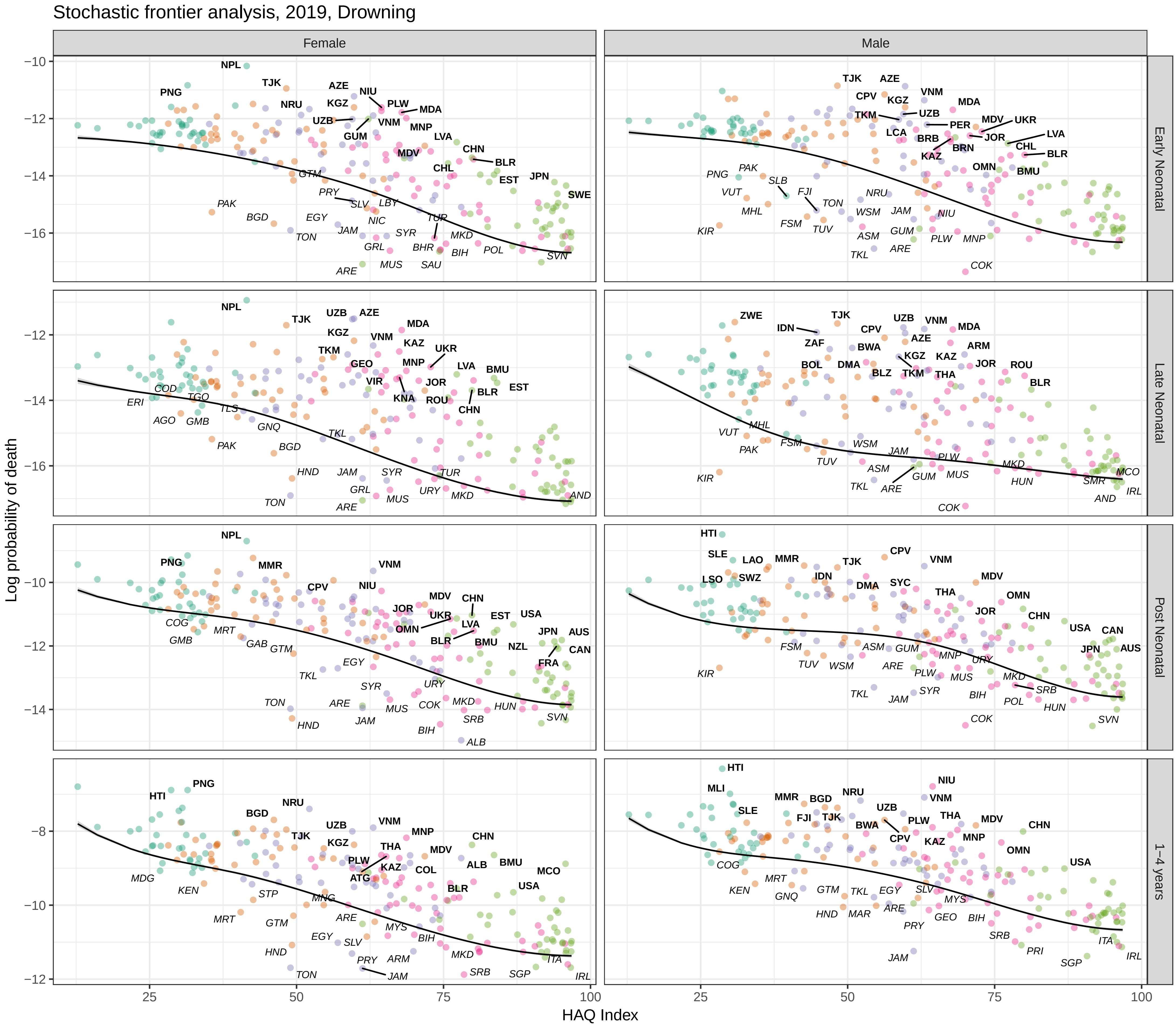

Figure S8
Cause- and age-specific mortality by country and Healthcare Access and Quality (HAQ) Index, with the Survival Potential Frontier (SPF) (black line) and uncertainty interval around the frontier (grey band). Countries are labelled in bold when their ratio to the frontier is in the top 10 percent (performing poorly relative to HAQ Index) and in italics when their ratio to the frontier is in the bottom 10 percent (performing well relative to HAQ Index). These results show the prediction of the cause-specific SPF prior to scaling to the all-cause frontier.

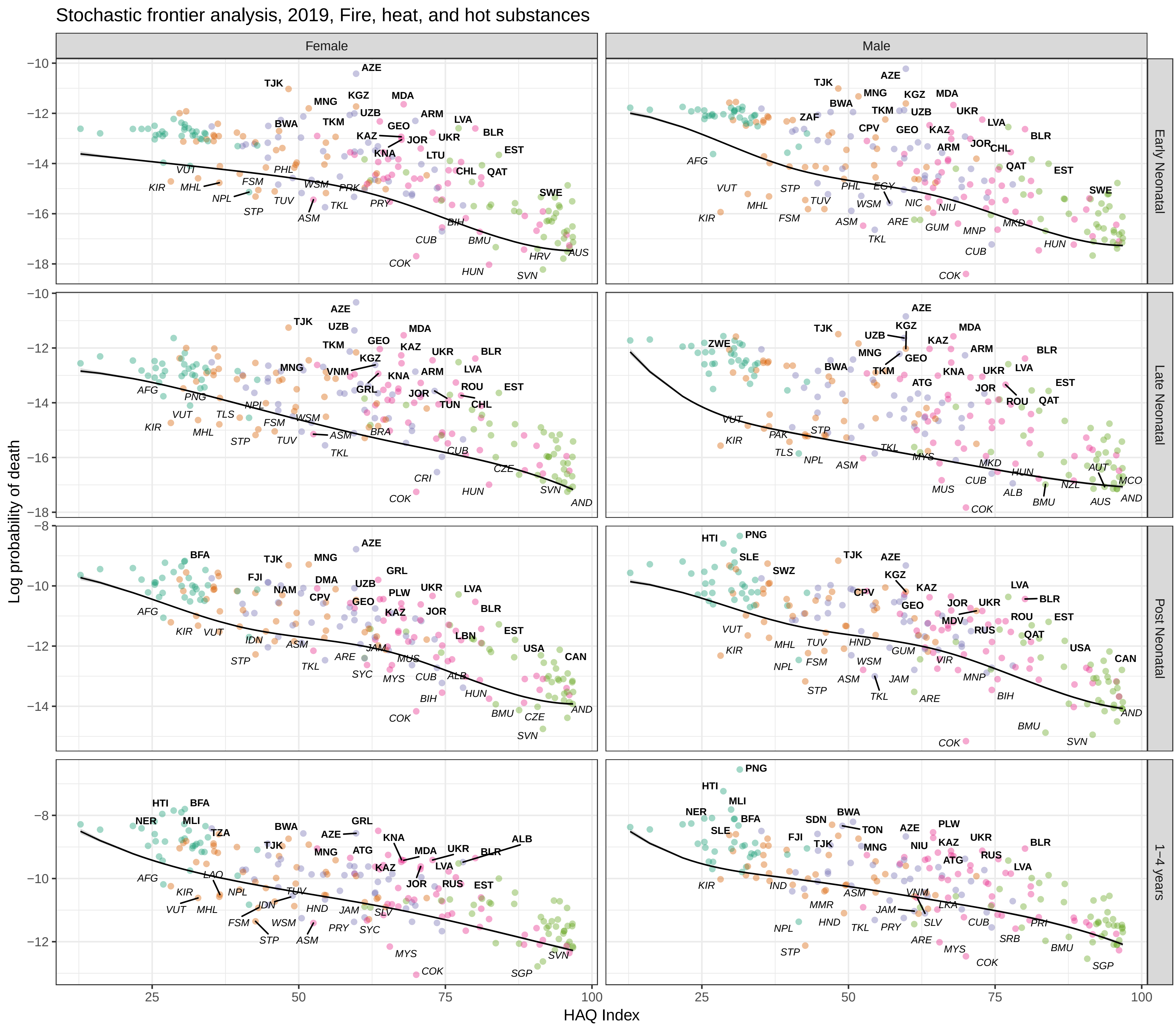

Figure S8
Cause- and age-specific mortality by country and Healthcare Access and Quality (HAQ) Index, with the Survival Potential Frontier (SPF) (black line) and uncertainty interval around the frontier (grey band). Countries are labelled in bold when their ratio to the frontier is in the top 10 percent (performing poorly relative to HAQ Index) and in italics when their ratio to the frontier is in the bottom 10 percent (performing well relative to HAQ Index). These results show the prediction of the cause-specific SPF prior to scaling to the all-cause frontier.

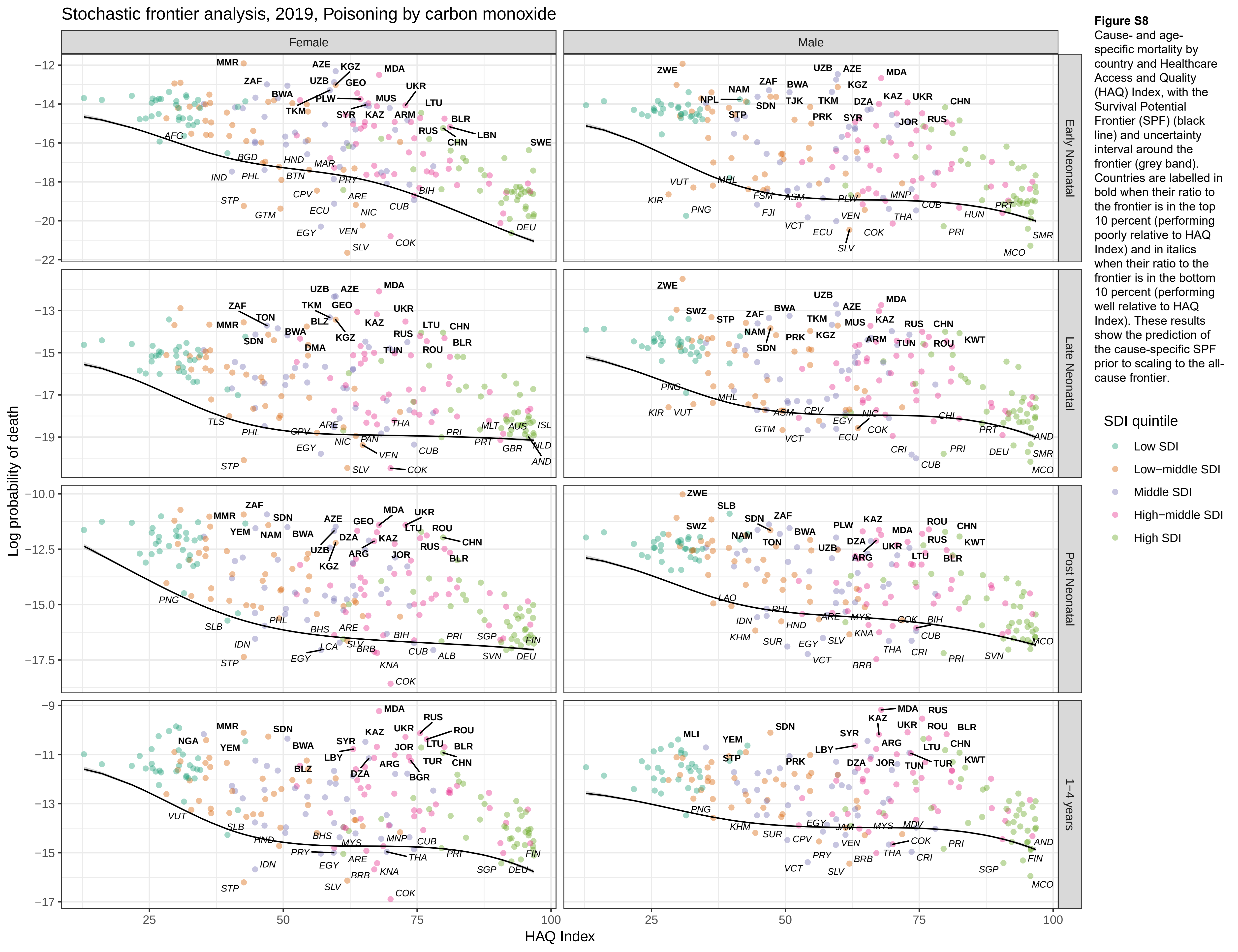

Figure S8
Cause- and age-specific mortality by country and Healthcare Access and Quality (HAQ) Index, with the Survival Potential Frontier (SPF) (black line) and uncertainty interval around the frontier (grey band). Countries are labelled in bold when their ratio to the frontier is in the top 10 percent (performing poorly relative to HAQ Index) and in italics when their ratio to the frontier is in the bottom 10 percent (performing well relative to HAQ Index). These results show the prediction of the cause-specific SPF prior to scaling to the all-cause frontier.

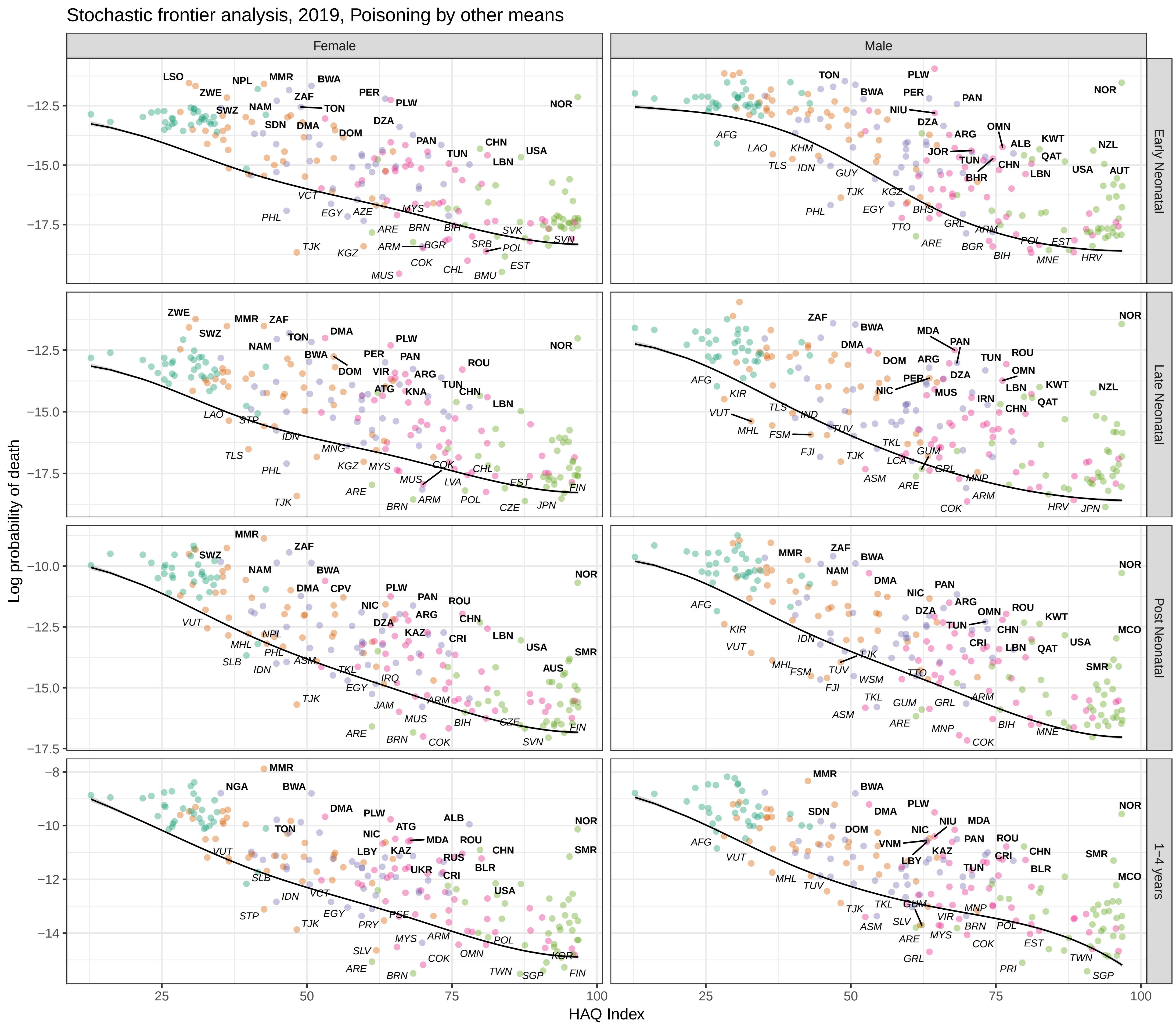

Figure S8
Cause- and age-specific mortality by country and Healthcare Access and Quality (HAQ) Index, with the Survival Potential Frontier (SPF) (black line) and uncertainty interval around the frontier (grey band). Countries are labelled in bold when their ratio to the frontier is in the top 10 percent (performing poorly relative to HAQ Index) and in italics when their ratio to the frontier is in the bottom 10 percent (performing well relative to HAQ Index). These results show the prediction of the cause-specific SPF prior to scaling to the all-cause frontier.

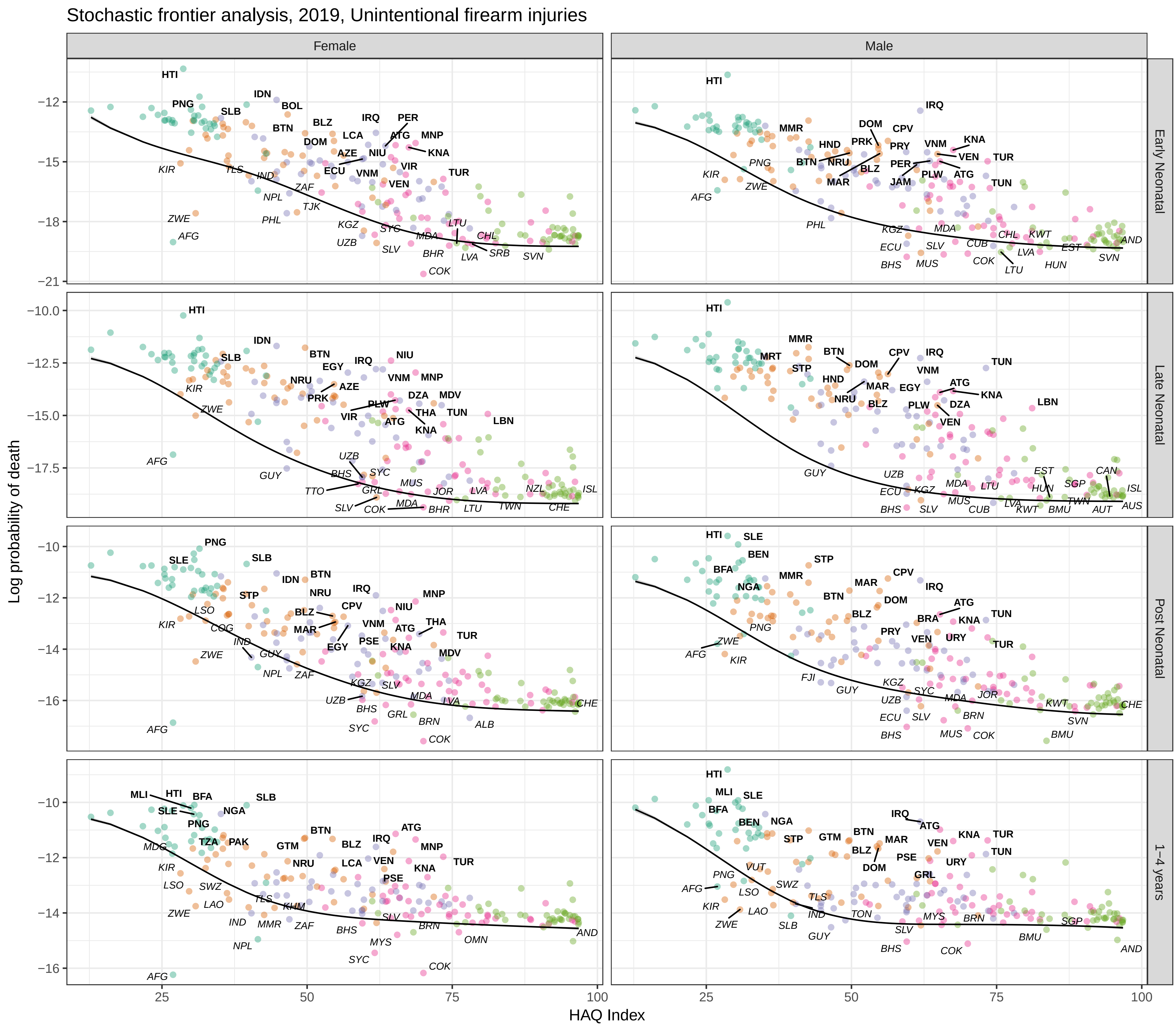

Figure S8
Cause- and age-specific mortality by country and Healthcare Access and Quality (HAQ) Index, with the Survival Potential Frontier (SPF) (black line) and uncertainty interval around the frontier (grey band). Countries are labelled in bold when their ratio to the frontier is in the top 10 percent (performing poorly relative to HAQ Index) and in italics when their ratio to the frontier is in the bottom 10 percent (performing well relative to HAQ Index). These results show the prediction of the cause-specific SPF prior to scaling to the all-cause frontier.

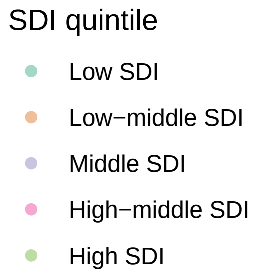

Stochastic frontier analysis, 2019, Other exposure to mechanical forces

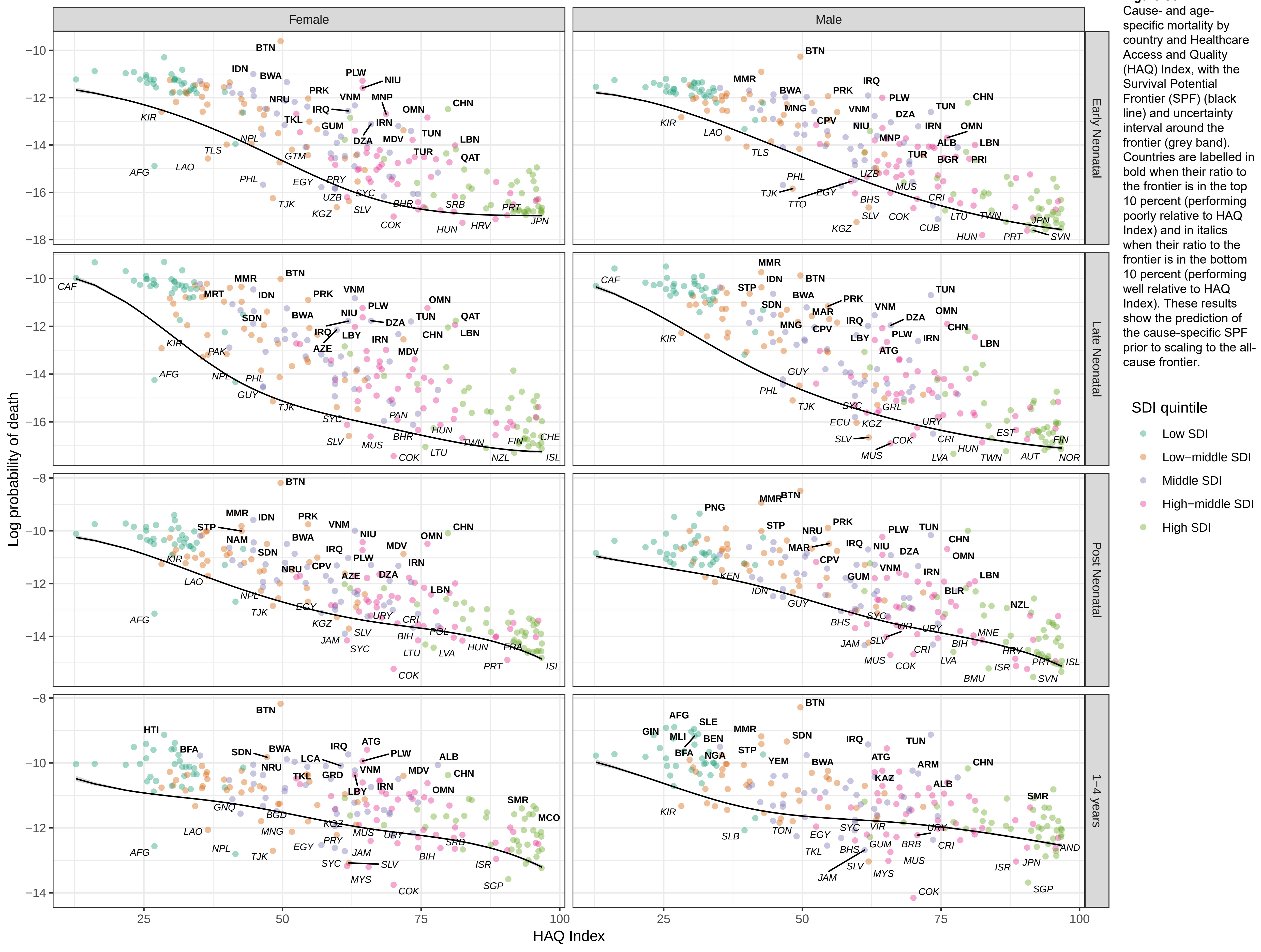

Figure S8
Cause- and age-specific mortality by country and Healthcare Access and Quality (HAQ) Index, with the Survival Potential Frontier (SPF) (black line) and uncertainty interval around the frontier (grey band). Countries are labelled in bold when their ratio to the frontier is in the top 10 percent (performing poorly relative to HAQ Index) and in italics when their ratio to the frontier is in the bottom 10 percent (performing well relative to HAQ Index). These results show the prediction of the cause-specific SPF prior to scaling to the all-cause frontier.

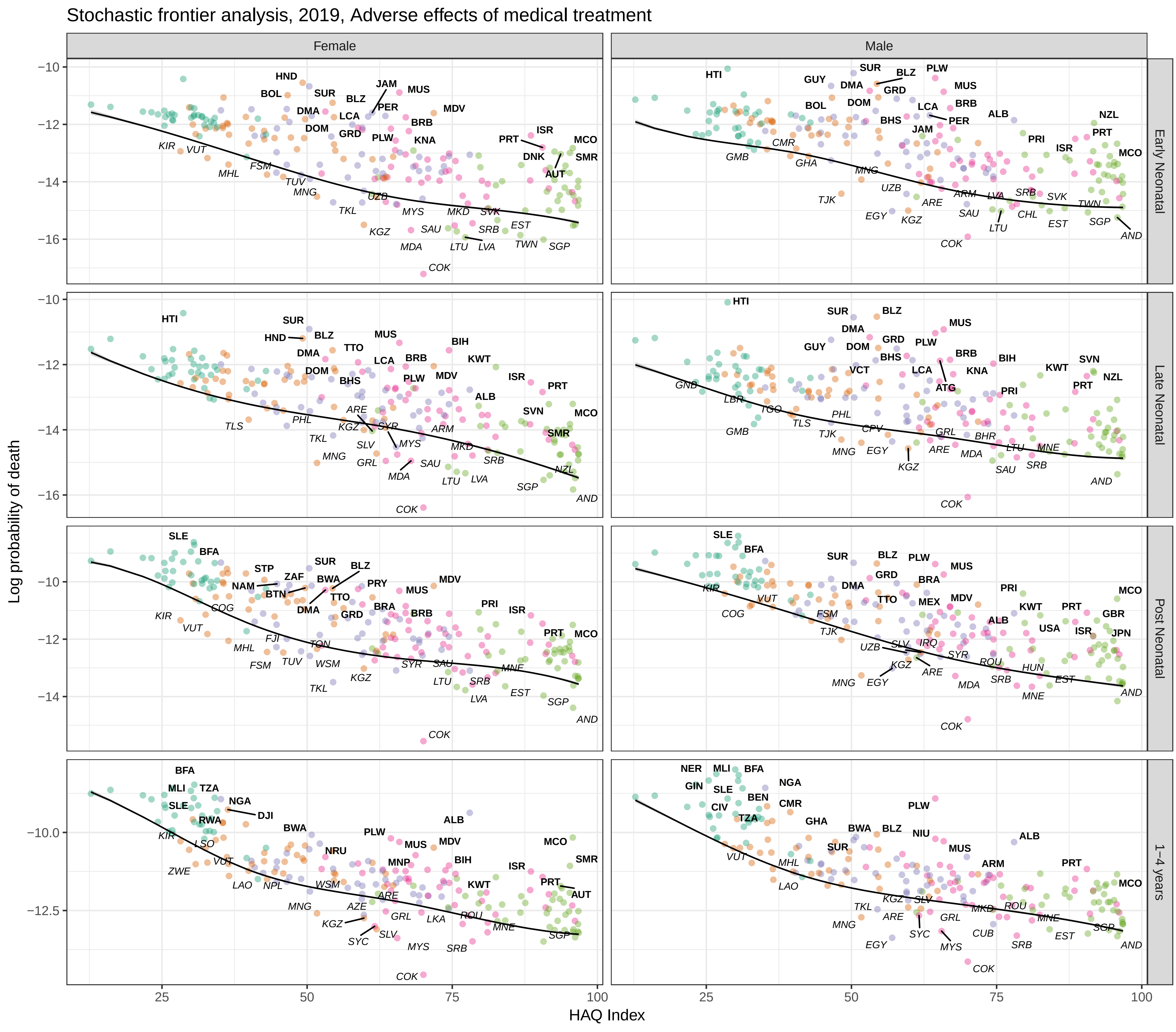

Figure S8
Cause- and age-specific mortality by country and Healthcare Access and Quality (HAQ) Index, with the Survival Potential Frontier (SPF) (black line) and uncertainty interval around the frontier (grey band). Countries are labelled in bold when their ratio to the frontier is in the top 10 percent (performing poorly relative to HAQ Index) and in italics when their ratio to the frontier is in the bottom 10 percent (performing well relative to HAQ Index). These results show the prediction of the cause-specific SPF prior to scaling to the all-cause frontier.

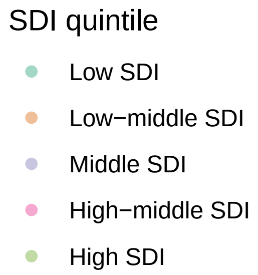

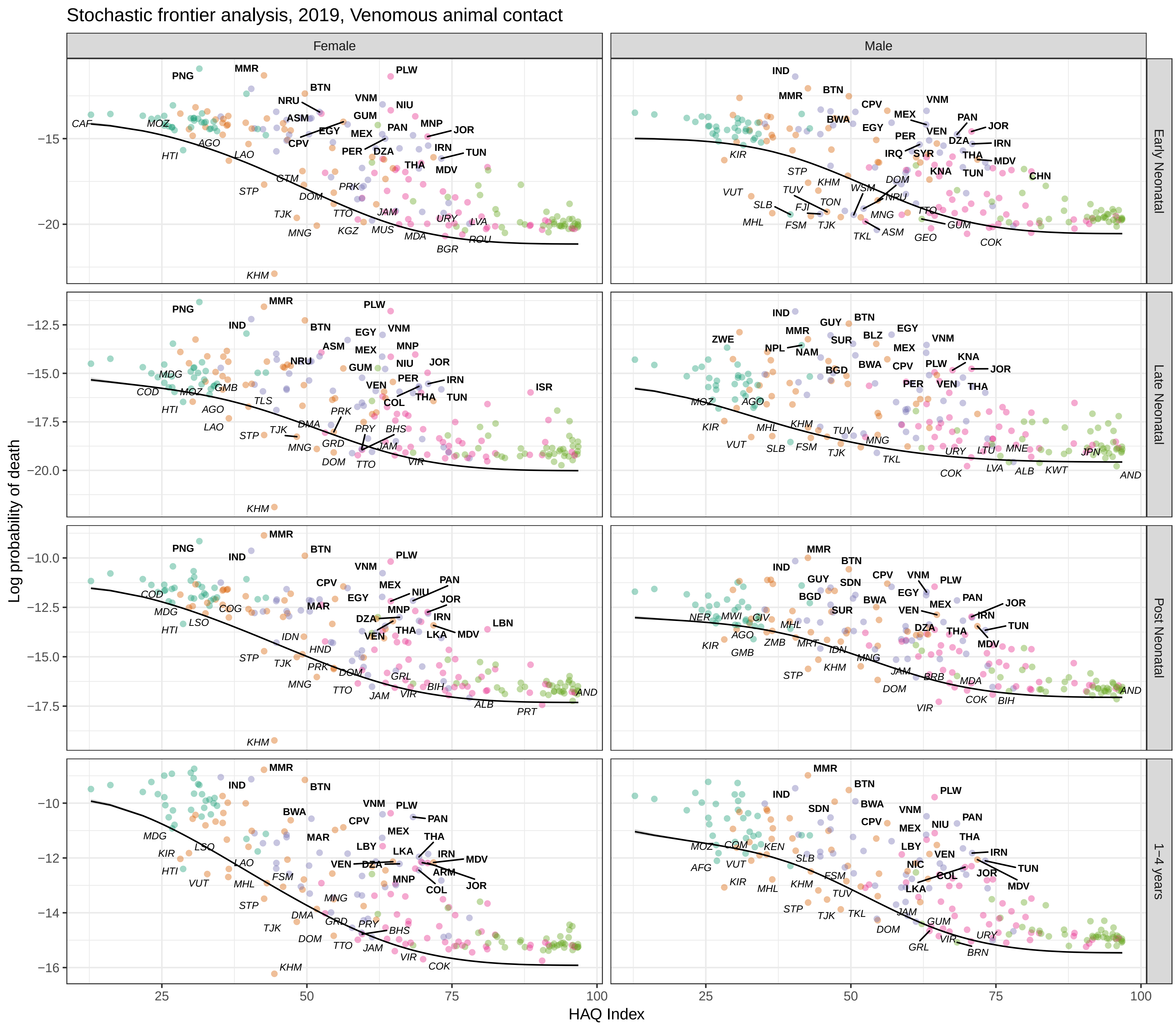

Figure S8
Cause- and age-specific mortality by country and Healthcare Access and Quality (HAQ) Index, with the Survival Potential Frontier (SPF) (black line) and uncertainty interval around the frontier (grey band). Countries are labelled in bold when their ratio to the frontier is in the top 10 percent (performing poorly relative to HAQ Index) and in italics when their ratio to the frontier is in the bottom 10 percent (performing well relative to HAQ Index). These results show the prediction of the cause-specific SPF prior to scaling to the all-cause frontier.

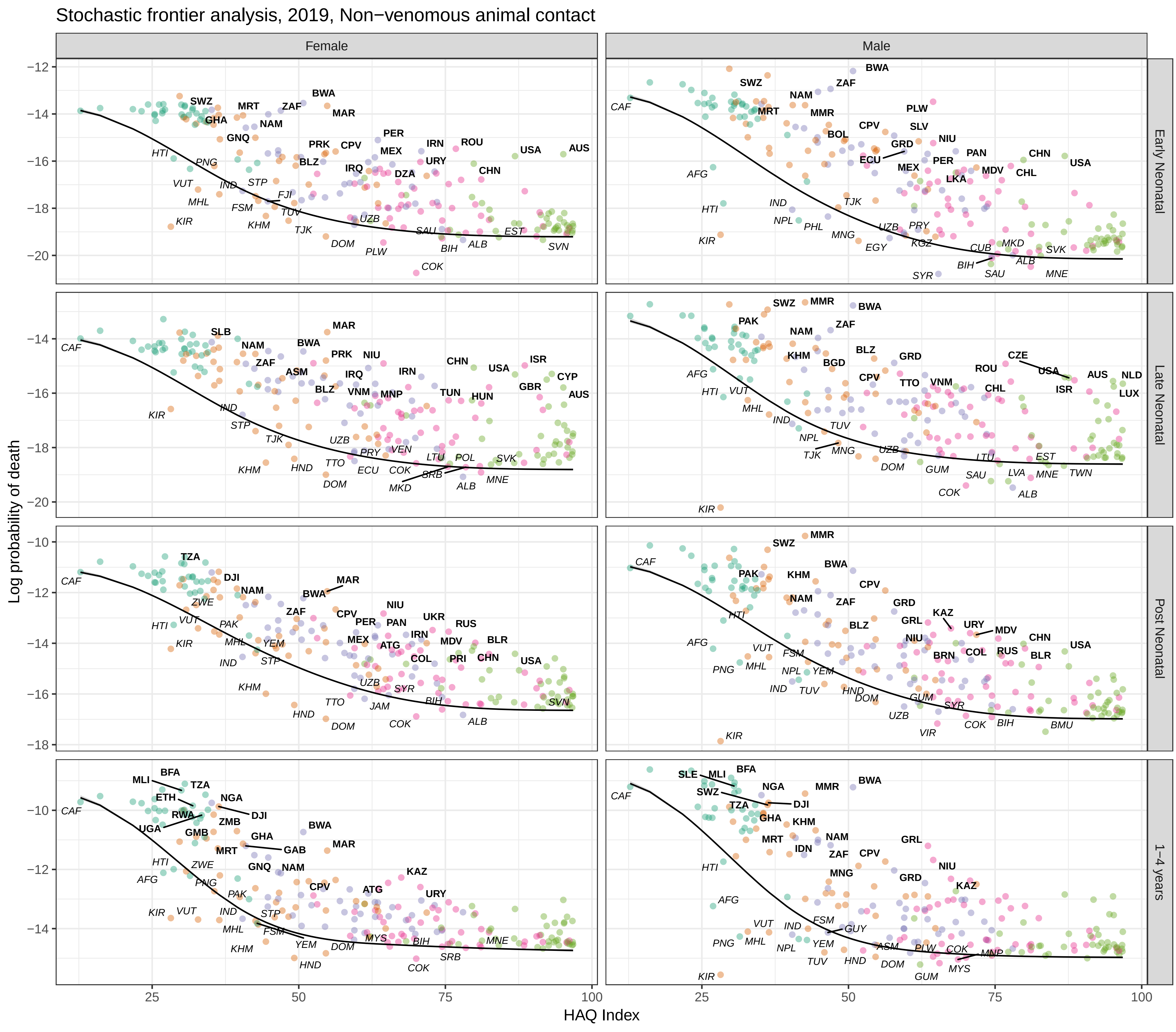

Figure S8
Cause- and age-specific mortality by country and Healthcare Access and Quality (HAQ) Index, with the Survival Potential Frontier (SPF) (black line) and uncertainty interval around the frontier (grey band). Countries are labelled in bold when their ratio to the frontier is in the top 10 percent (performing poorly relative to HAQ Index) and in italics when their ratio to the frontier is in the bottom 10 percent (performing well relative to HAQ Index). These results show the prediction of the cause-specific SPF prior to scaling to the all-cause frontier.

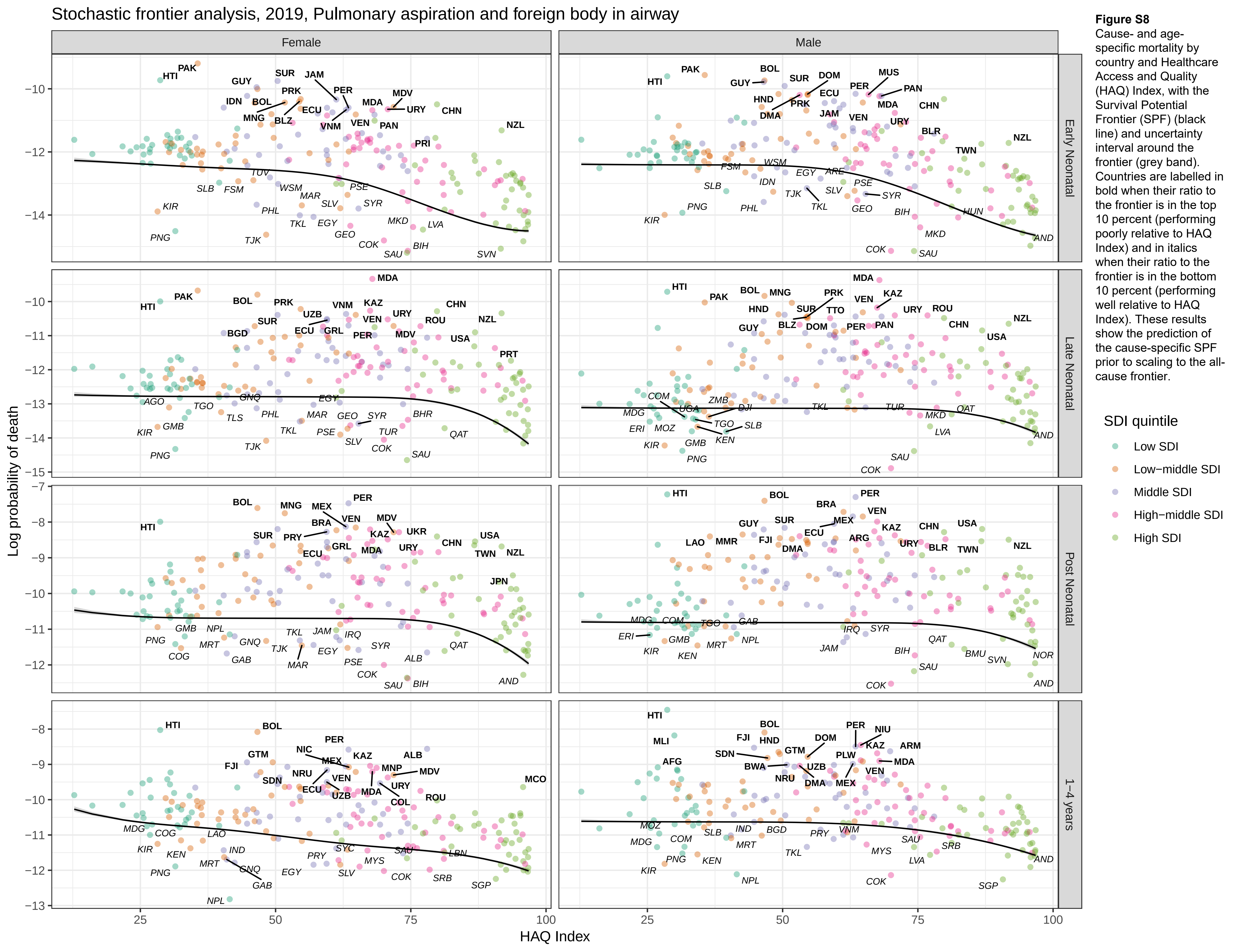

Figure S8
Cause- and age-specific mortality by country and Healthcare Access and Quality (HAQ) Index, with the Survival Potential Frontier (SPF) (black line) and uncertainty interval around the frontier (grey band). Countries are labelled in bold when their ratio to the frontier is in the top 10 percent (performing poorly relative to HAQ Index) and in italics when their ratio to the frontier is in the bottom 10 percent (performing well relative to HAQ Index). These results show the prediction of the cause-specific SPF prior to scaling to the all-cause frontier.

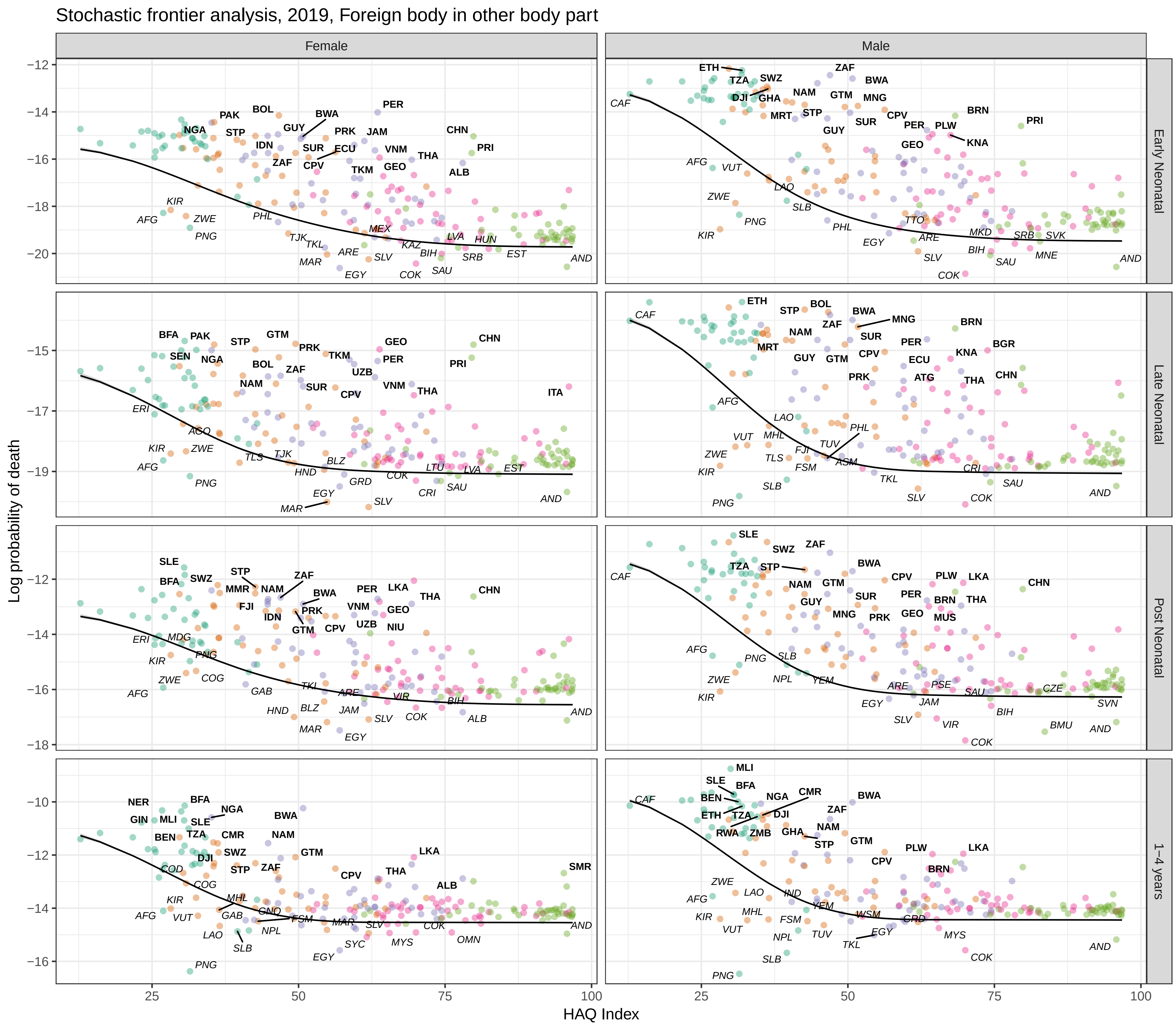

Figure S8
Cause- and age-specific mortality by country and Healthcare Access and Quality (HAQ) Index, with the Survival Potential Frontier (SPF) (black line) and uncertainty interval around the frontier (grey band). Countries are labelled in bold when their ratio to the frontier is in the top 10 percent (performing poorly relative to HAQ Index) and in italics when their ratio to the frontier is in the bottom 10 percent (performing well relative to HAQ Index). These results show the prediction of the cause-specific SPF prior to scaling to the all-cause frontier.

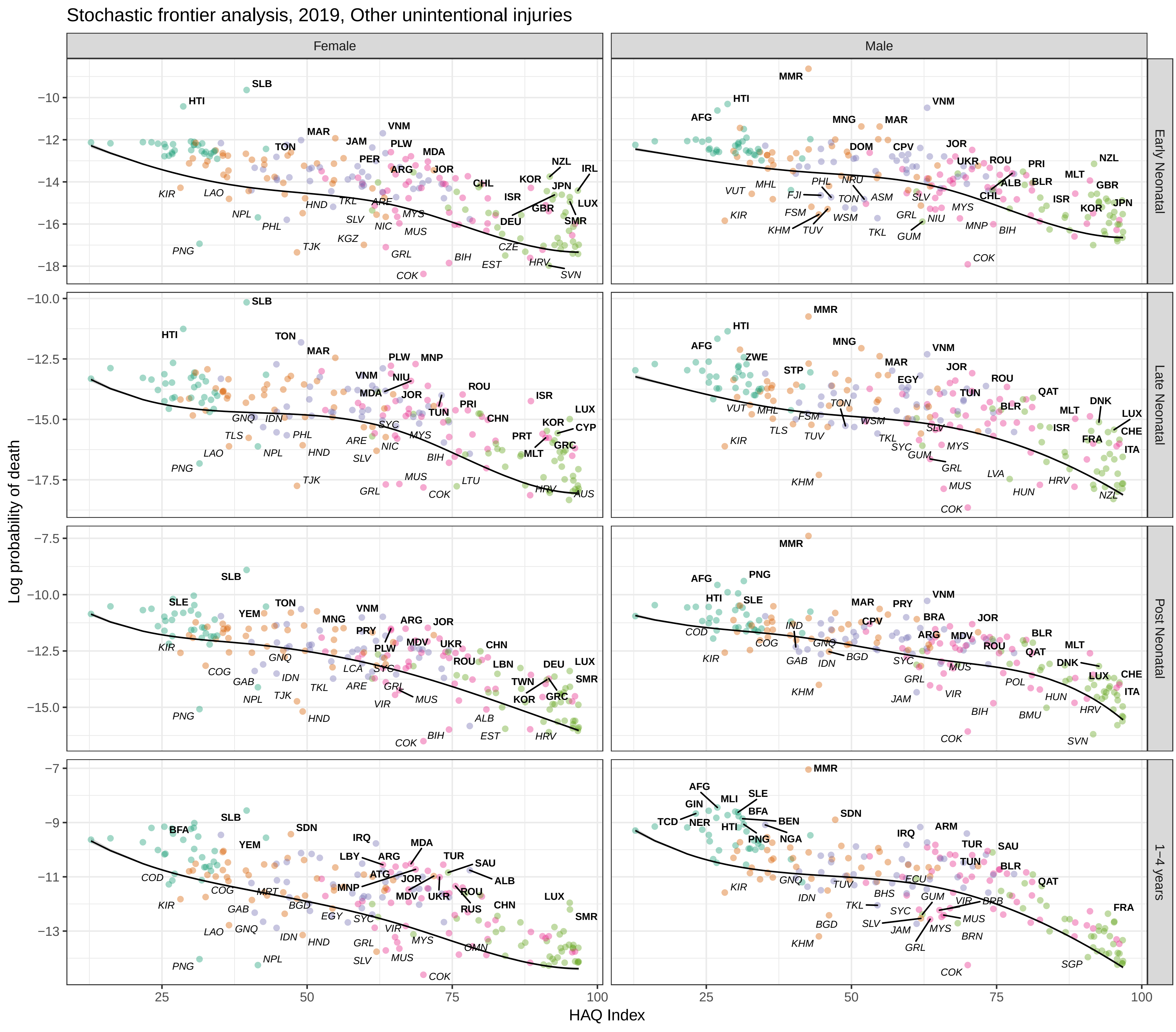

Figure S8
Cause- and age-specific mortality by country and Healthcare Access and Quality (HAQ) Index, with the Survival Potential Frontier (SPF) (black line) and uncertainty interval around the frontier (grey band). Countries are labelled in bold when their ratio to the frontier is in the top 10 percent (performing poorly relative to HAQ Index) and in italics when their ratio to the frontier is in the bottom 10 percent (performing well relative to HAQ Index). These results show the prediction of the cause-specific SPF prior to scaling to the all-cause frontier.

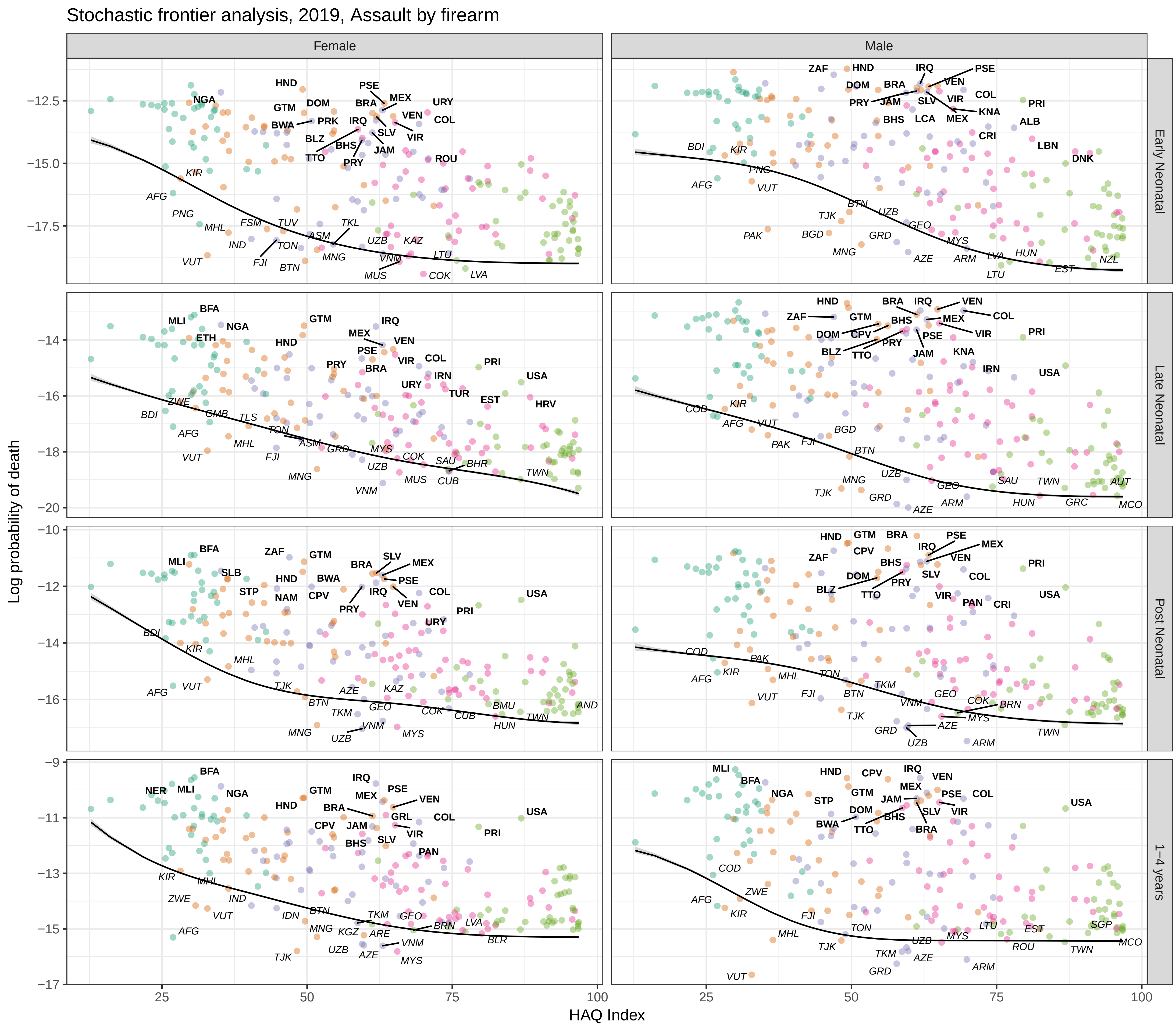

Figure S8
Cause- and age-specific mortality by country and Healthcare Access and Quality (HAQ) Index, with the Survival Potential Frontier (SPF) (black line) and uncertainty interval around the frontier (grey band). Countries are labelled in bold when their ratio to the frontier is in the top 10 percent (performing poorly relative to HAQ Index) and in italics when their ratio to the frontier is in the bottom 10 percent (performing well relative to HAQ Index). These results show the prediction of the cause-specific SPF prior to scaling to the all-cause frontier.

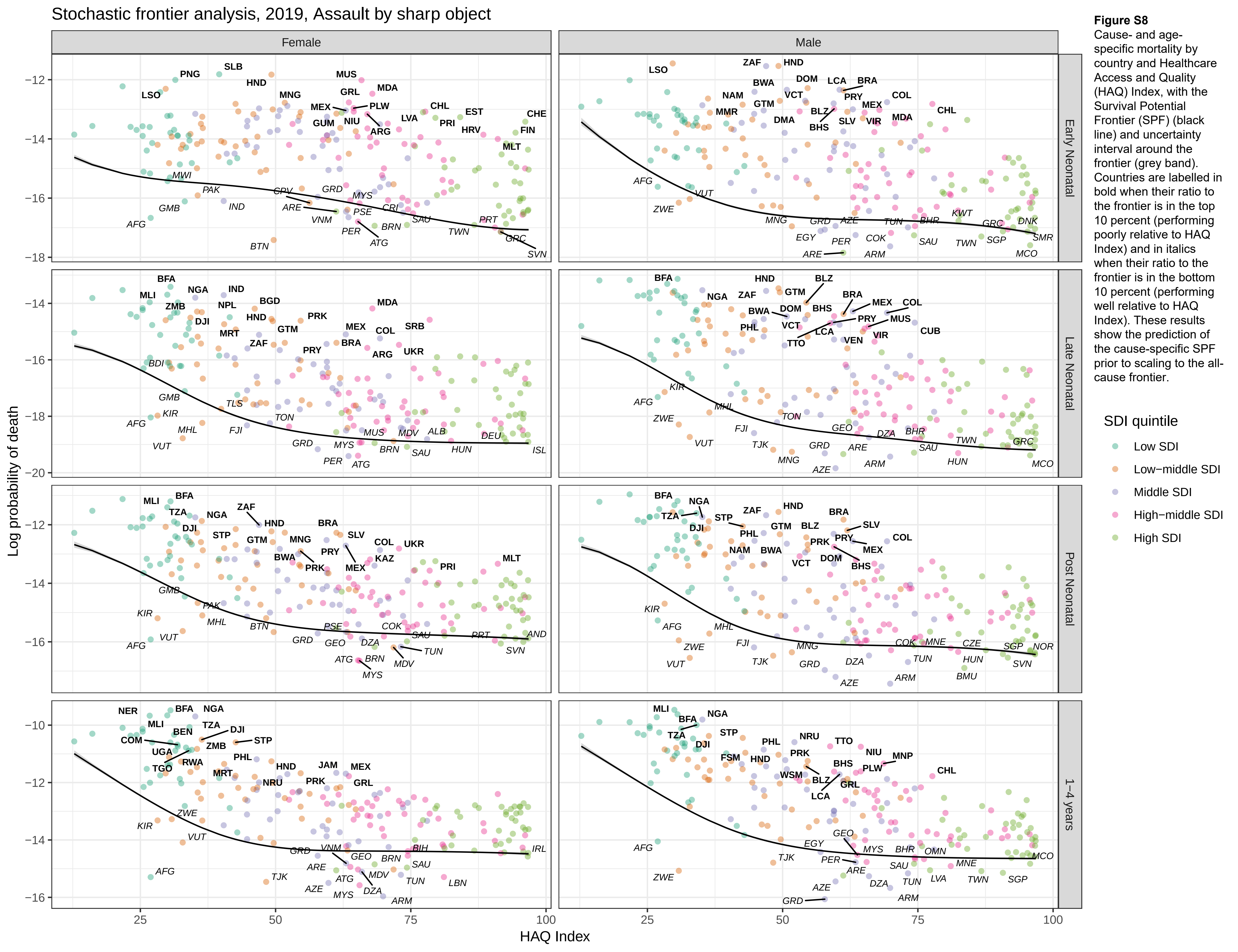

Figure S8
Cause- and age-specific mortality by country and Healthcare Access and Quality (HAQ) Index, with the Survival Potential Frontier (SPF) (black line) and uncertainty interval around the frontier (grey band). Countries are labelled in bold when their ratio to the frontier is in the top 10 percent (performing poorly relative to HAQ Index) and in italics when their ratio to the frontier is in the bottom 10 percent (performing well relative to HAQ Index). These results show the prediction of the cause-specific SPF prior to scaling to the all-cause frontier.

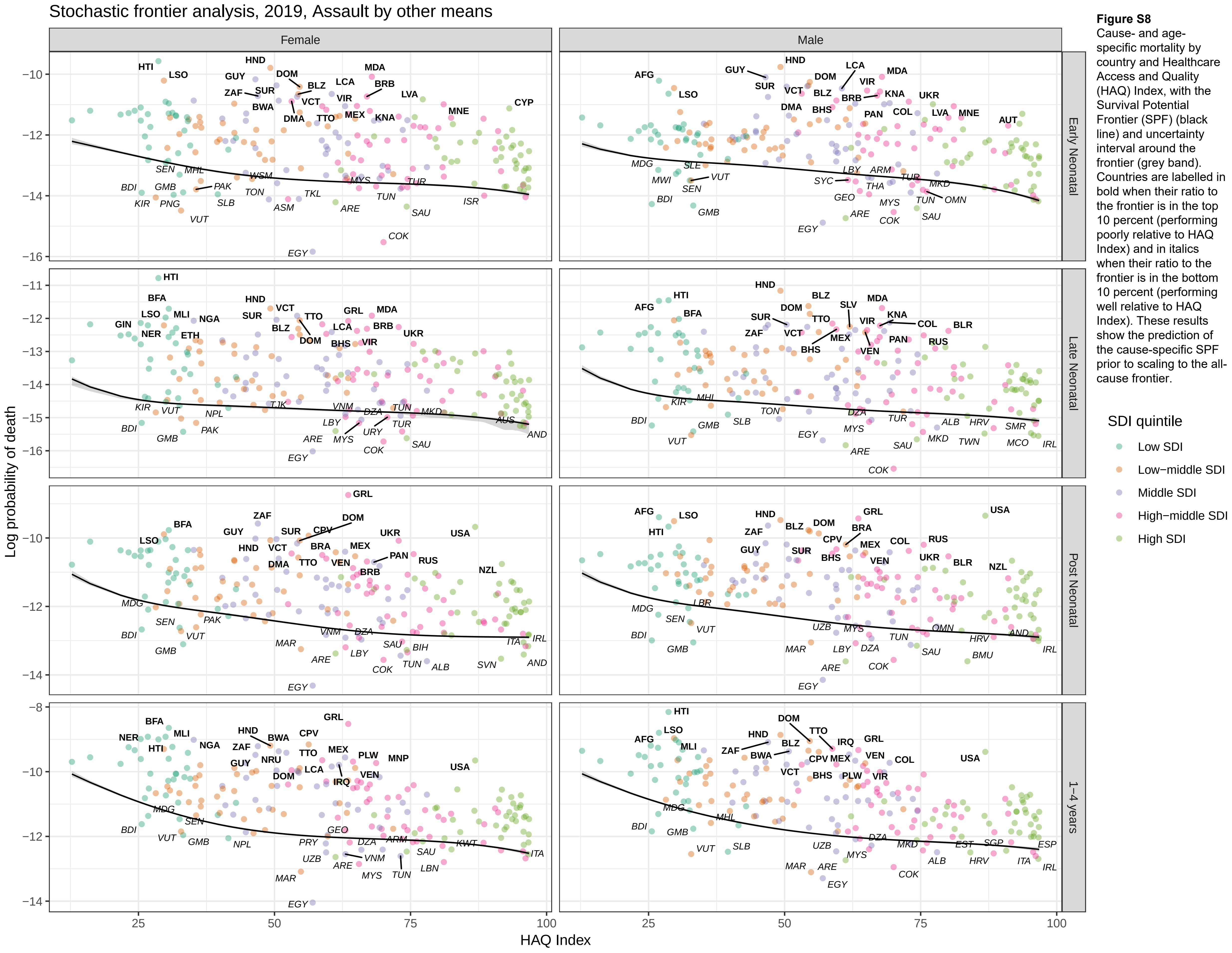

Figure S8
Cause- and age-specific mortality by country and Healthcare Access and Quality (HAQ) Index, with the Survival Potential Frontier (SPF) (black line) and uncertainty interval around the frontier (grey band). Countries are labelled in bold when their ratio to the frontier is in the top 10 percent (performing poorly relative to HAQ Index) and in italics when their ratio to the frontier is in the bottom 10 percent (performing well relative to HAQ Index). These results show the prediction of the cause-specific SPF prior to scaling to the all-cause frontier.

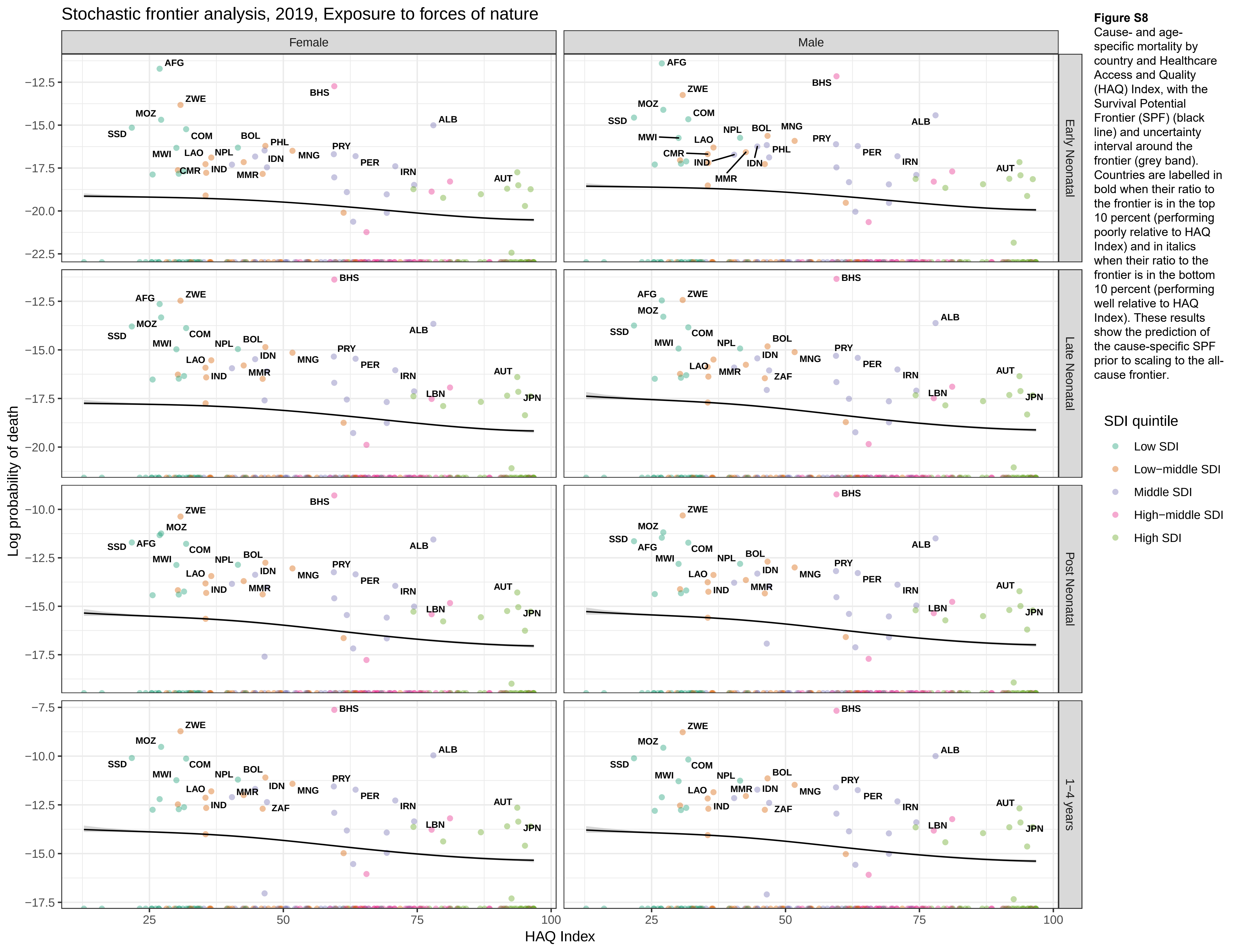

Figure S8
Cause- and age-specific mortality by country and Healthcare Access and Quality (HAQ) Index, with the Survival Potential Frontier (SPF) (black line) and uncertainty interval around the frontier (grey band). Countries are labelled in bold when their ratio to the frontier is in the top 10 percent (performing poorly relative to HAQ Index) and in italics when their ratio to the frontier is in the bottom 10 percent (performing well relative to HAQ Index). These results show the prediction of the cause-specific SPF prior to scaling to the all-cause frontier.

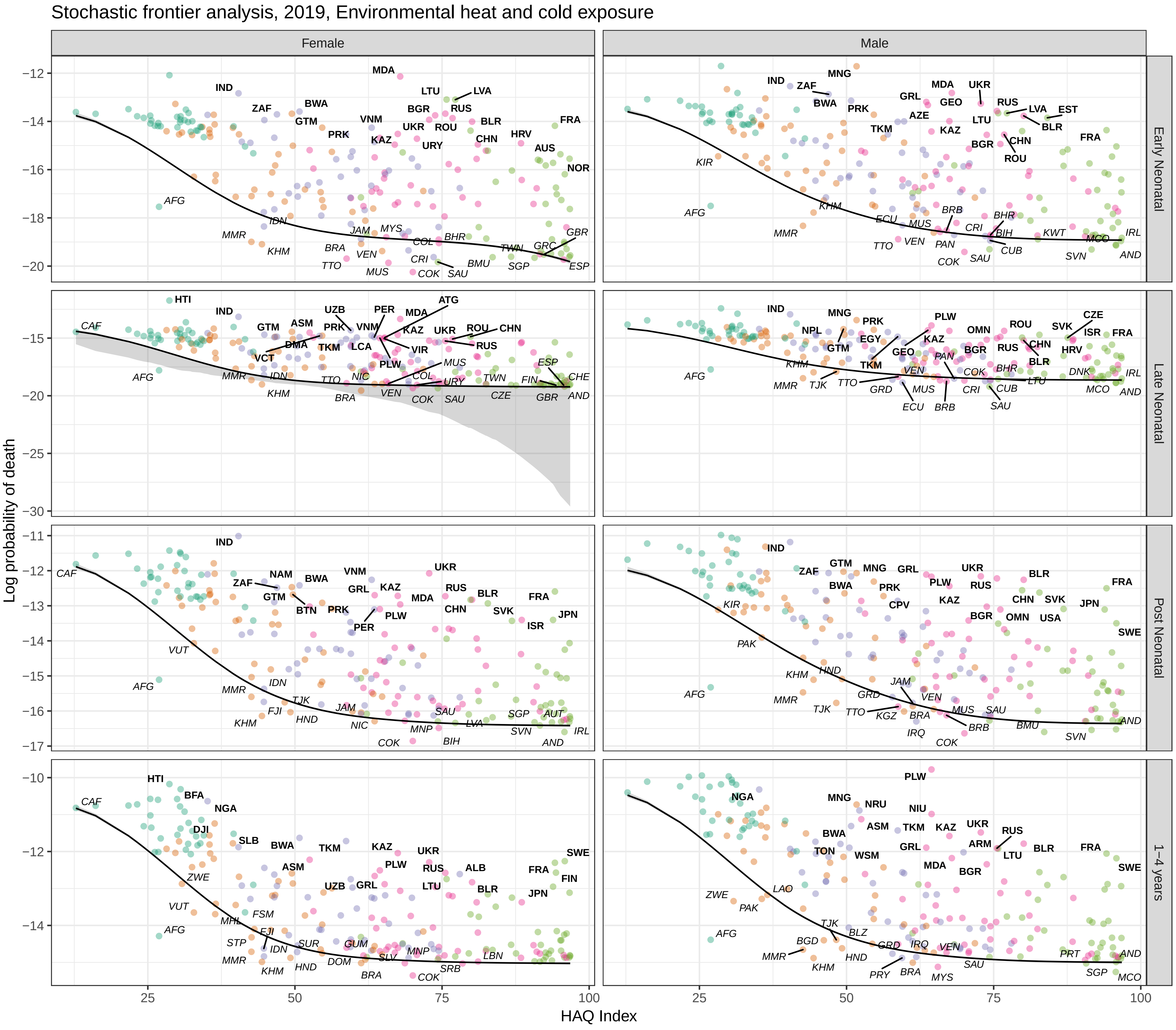

Figure S8
Cause- and age-specific mortality by country and Healthcare Access and Quality (HAQ) Index, with the Survival Potential Frontier (SPF) (black line) and uncertainty interval around the frontier (grey band). Countries are labelled in bold when their ratio to the frontier is in the top 10 percent (performing poorly relative to HAQ Index) and in italics when their ratio to the frontier is in the bottom 10 percent (performing well relative to HAQ Index). These results show the prediction of the cause-specific SPF prior to scaling to the all-cause frontier.

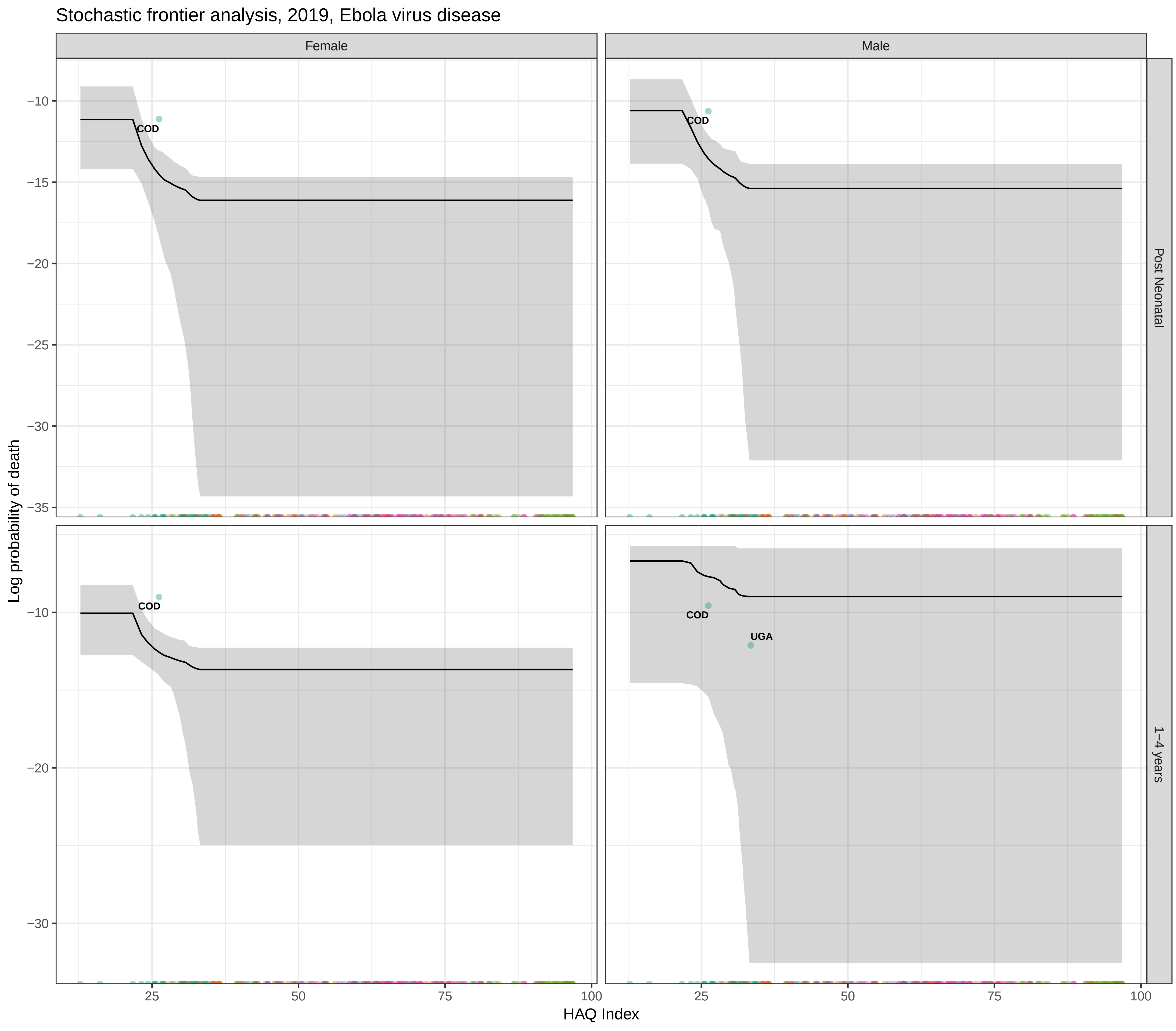

Figure S8
Cause- and age-specific mortality by country and Healthcare Access and Quality (HAQ) Index, with the Survival Potential Frontier (SPF) (black line) and uncertainty interval around the frontier (grey band). Countries are labelled in bold when their ratio to the frontier is in the top 10 percent (performing poorly relative to HAQ Index) and in italics when their ratio to the frontier is in the bottom 10 percent (performing well relative to HAQ Index). These results show the prediction of the cause-specific SPF prior to scaling to the all-cause frontier.

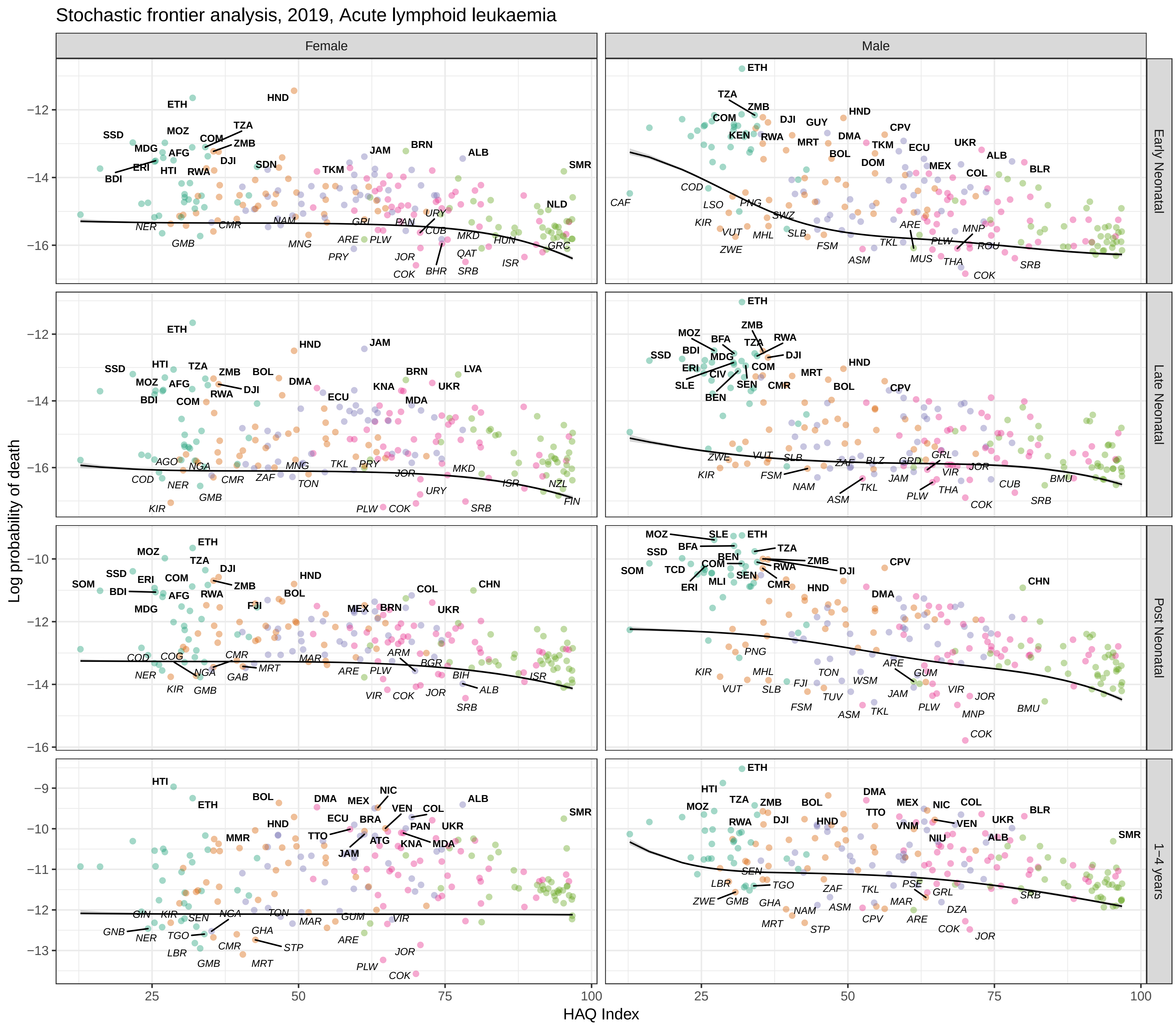

Figure S8
Cause- and age-specific mortality by country and Healthcare Access and Quality (HAQ) Index, with the Survival Potential Frontier (SPF) (black line) and uncertainty interval around the frontier (grey band). Countries are labelled in bold when their ratio to the frontier is in the top 10 percent (performing poorly relative to HAQ Index) and in italics when their ratio to the frontier is in the bottom 10 percent (performing well relative to HAQ Index). These results show the prediction of the cause-specific SPF prior to scaling to the all-cause frontier.

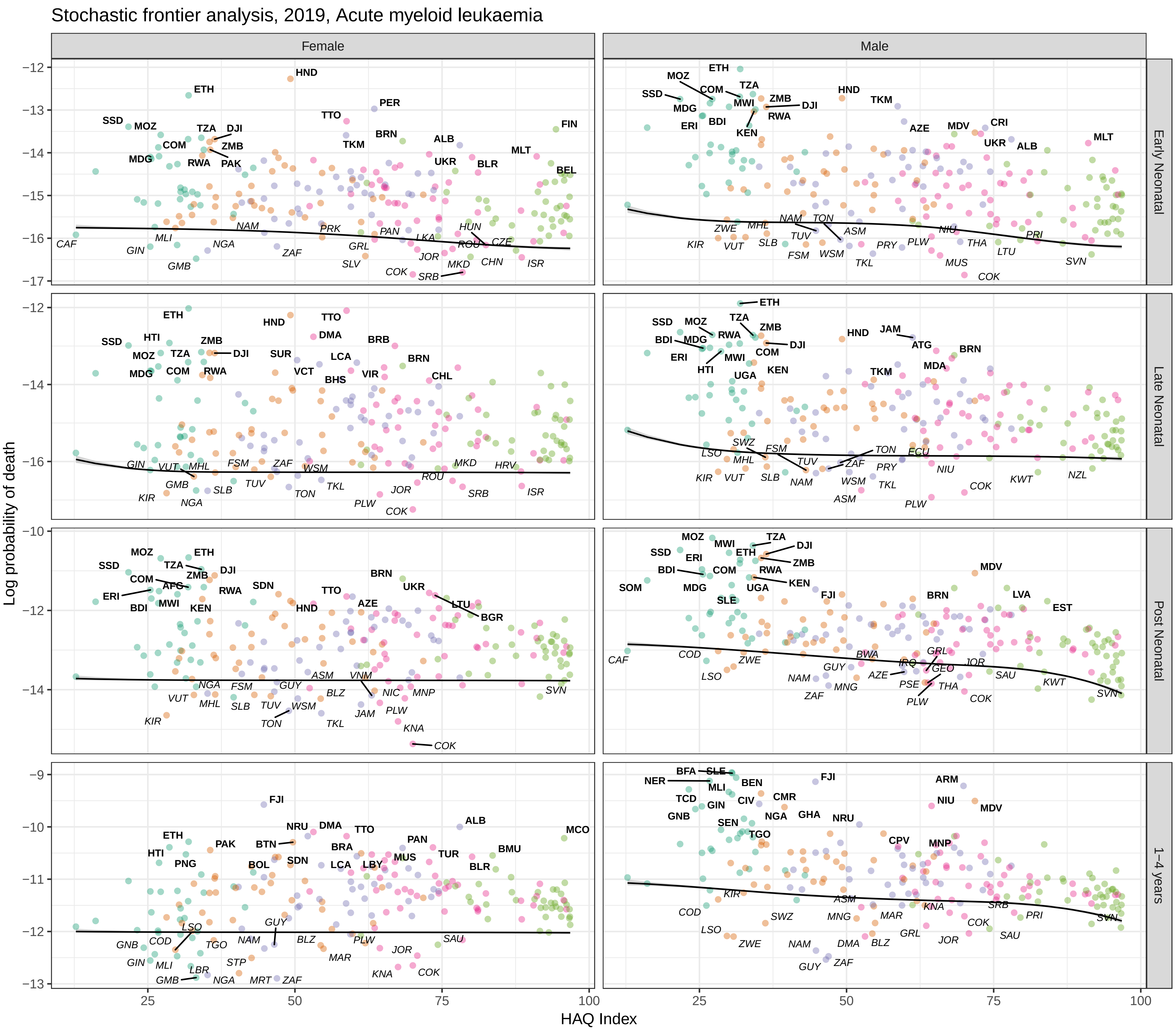

Figure S8
Cause- and age-specific mortality by country and Healthcare Access and Quality (HAQ) Index, with the Survival Potential Frontier (SPF) (black line) and uncertainty interval around the frontier (grey band). Countries are labelled in bold when their ratio to the frontier is in the top 10 percent (performing poorly relative to HAQ Index) and in italics when their ratio to the frontier is in the bottom 10 percent (performing well relative to HAQ Index). These results show the prediction of the cause-specific SPF prior to scaling to the all-cause frontier.

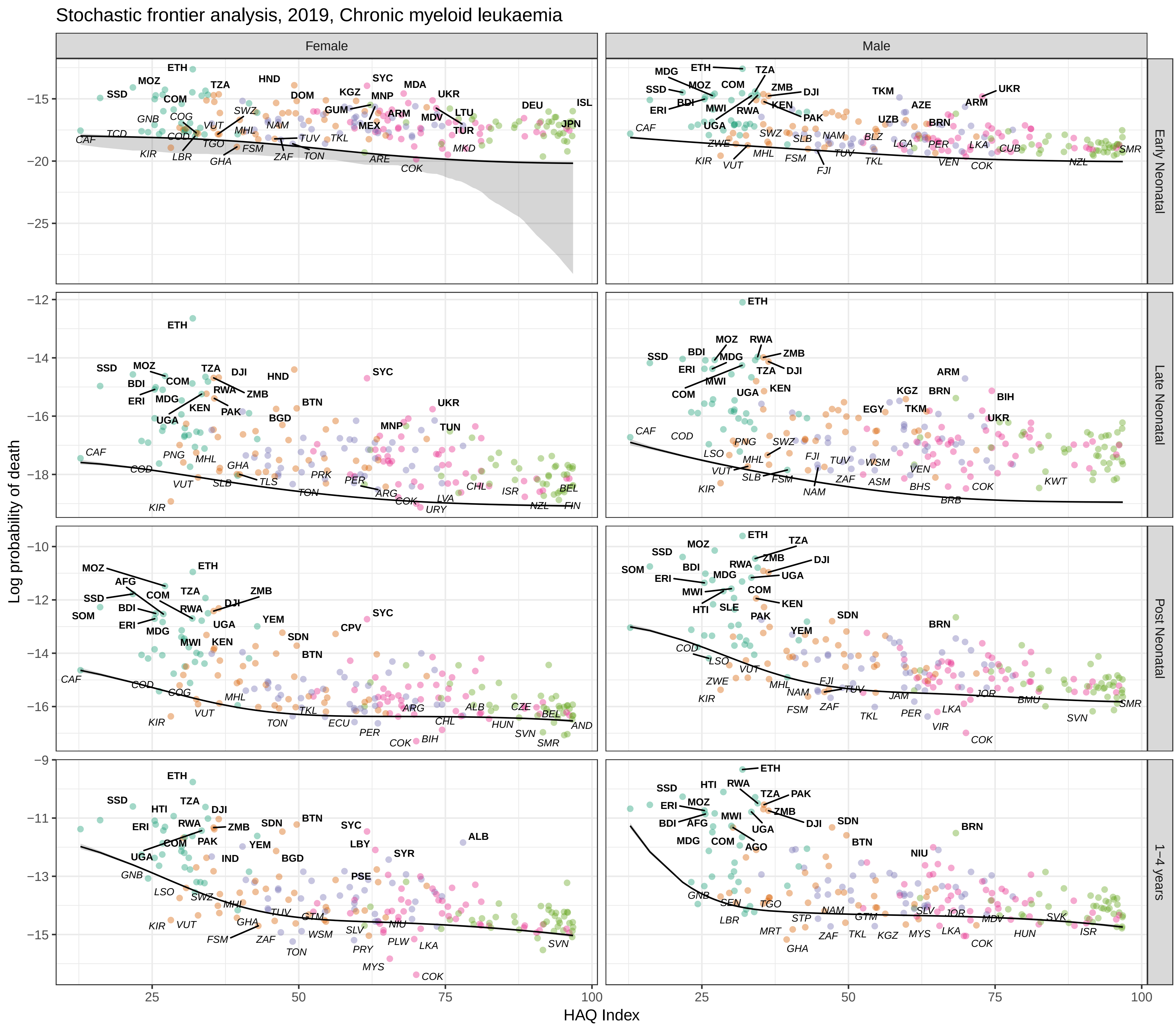

Figure S8
Cause- and age-specific mortality by country and Healthcare Access and Quality (HAQ) Index, with the Survival Potential Frontier (SPF) (black line) and uncertainty interval around the frontier (grey band). Countries are labelled in bold when their ratio to the frontier is in the top 10 percent (performing poorly relative to HAQ Index) and in italics when their ratio to the frontier is in the bottom 10 percent (performing well relative to HAQ Index). These results show the prediction of the cause-specific SPF prior to scaling to the all-cause frontier.

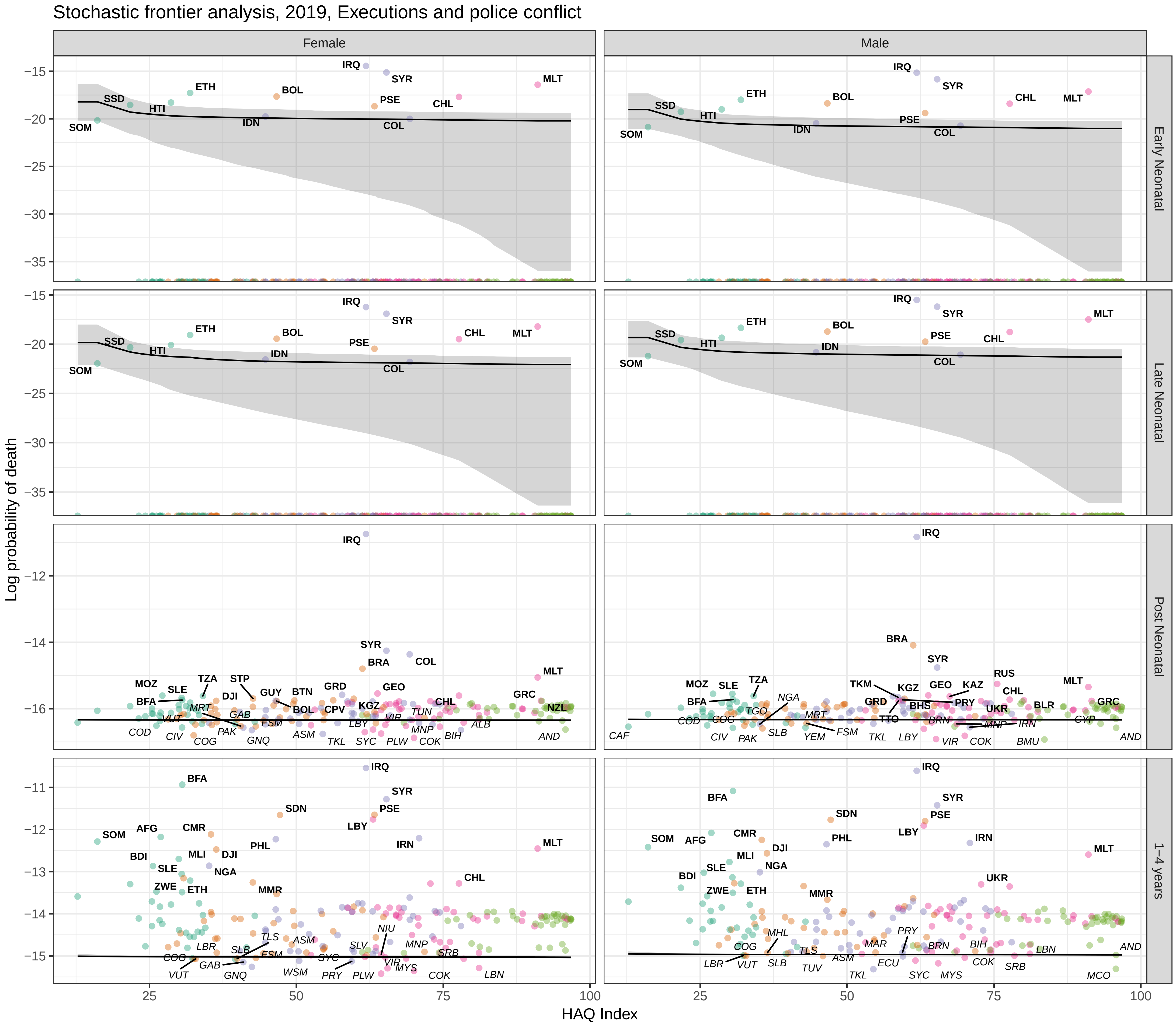

Figure S8
Cause- and age-specific mortality by country and Healthcare Access and Quality (HAQ) Index, with the Survival Potential Frontier (SPF) (black line) and uncertainty interval around the frontier (grey band). Countries are labelled in bold when their ratio to the frontier is in the top 10 percent (performing poorly relative to HAQ Index) and in italics when their ratio to the frontier is in the bottom 10 percent (performing well relative to HAQ Index). These results show the prediction of the cause-specific SPF prior to scaling to the all-cause frontier.

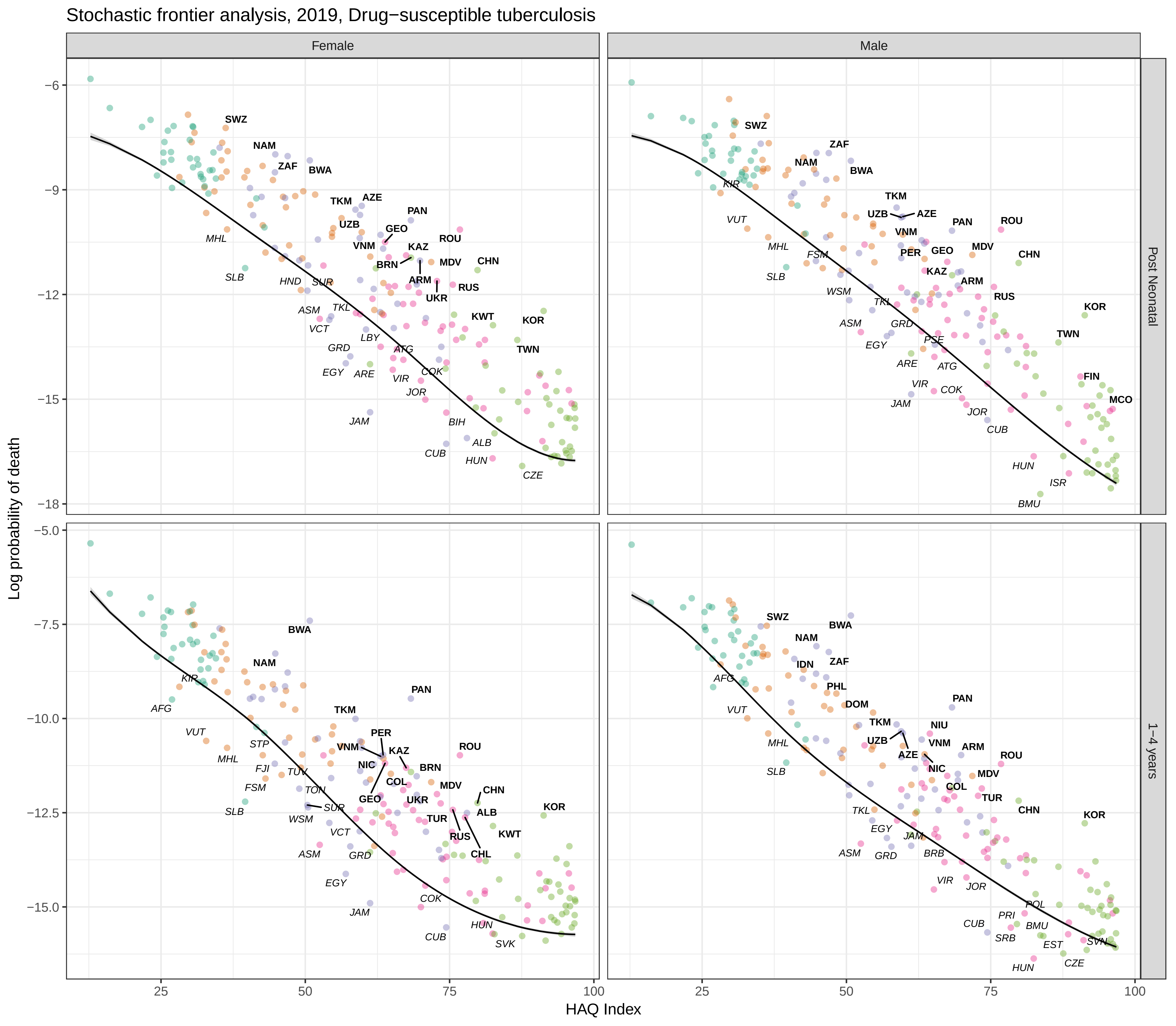

Figure S8
Cause- and age-specific mortality by country and Healthcare Access and Quality (HAQ) Index, with the Survival Potential Frontier (SPF) (black line) and uncertainty interval around the frontier (grey band). Countries are labelled in bold when their ratio to the frontier is in the top 10 percent (performing poorly relative to HAQ Index) and in italics when their ratio to the frontier is in the bottom 10 percent (performing well relative to HAQ Index). These results show the prediction of the cause-specific SPF prior to scaling to the all-cause frontier.

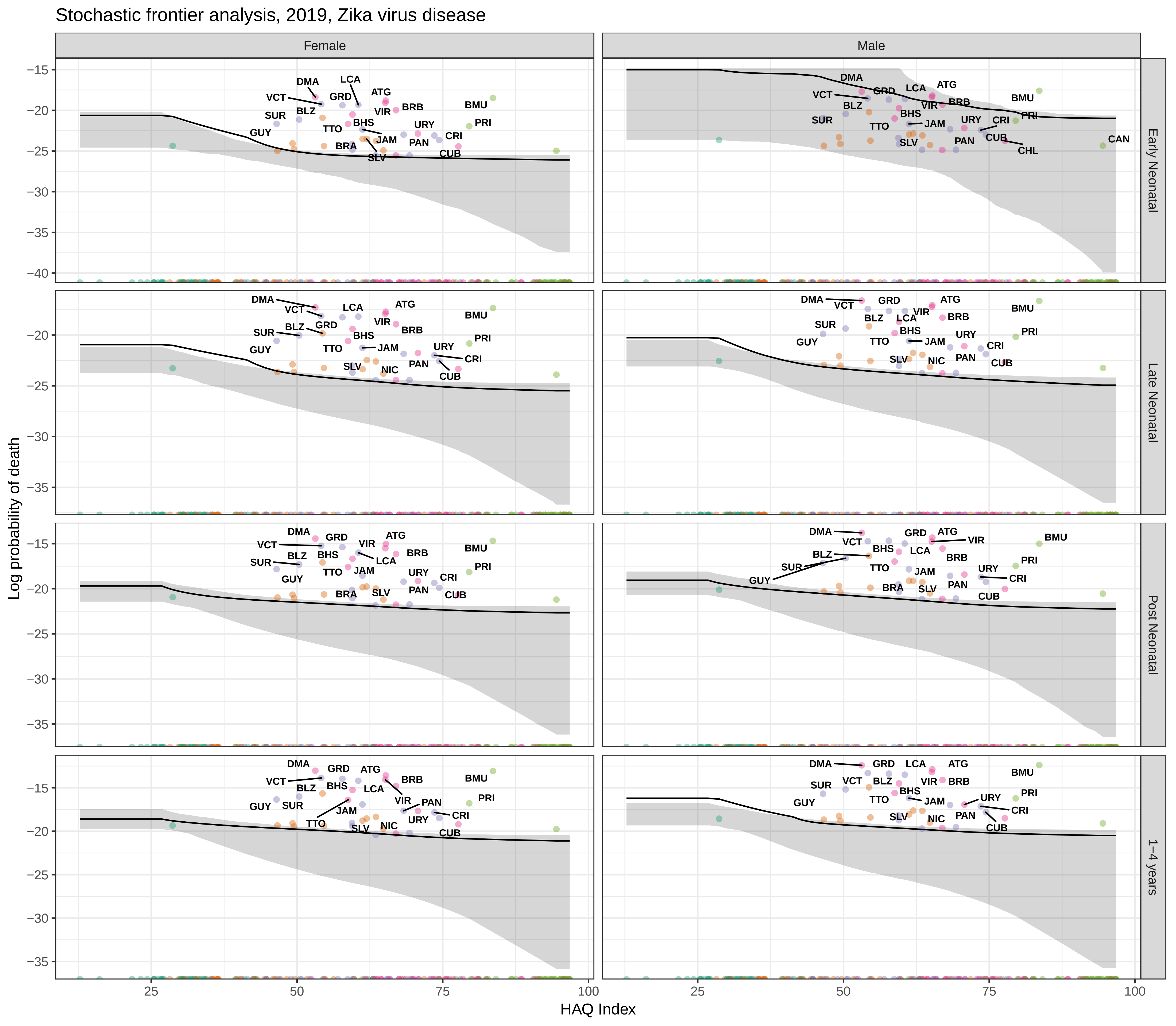

Figure S8
Cause- and age-specific mortality by country and Healthcare Access and Quality (HAQ) Index, with the Survival Potential Frontier (SPF) (black line) and uncertainty interval around the frontier (grey band). Countries are labelled in bold when their ratio to the frontier is in the top 10 percent (performing poorly relative to HAQ Index) and in italics when their ratio to the frontier is in the bottom 10 percent (performing well relative to HAQ Index). These results show the prediction of the cause-specific SPF prior to scaling to the all-cause frontier.

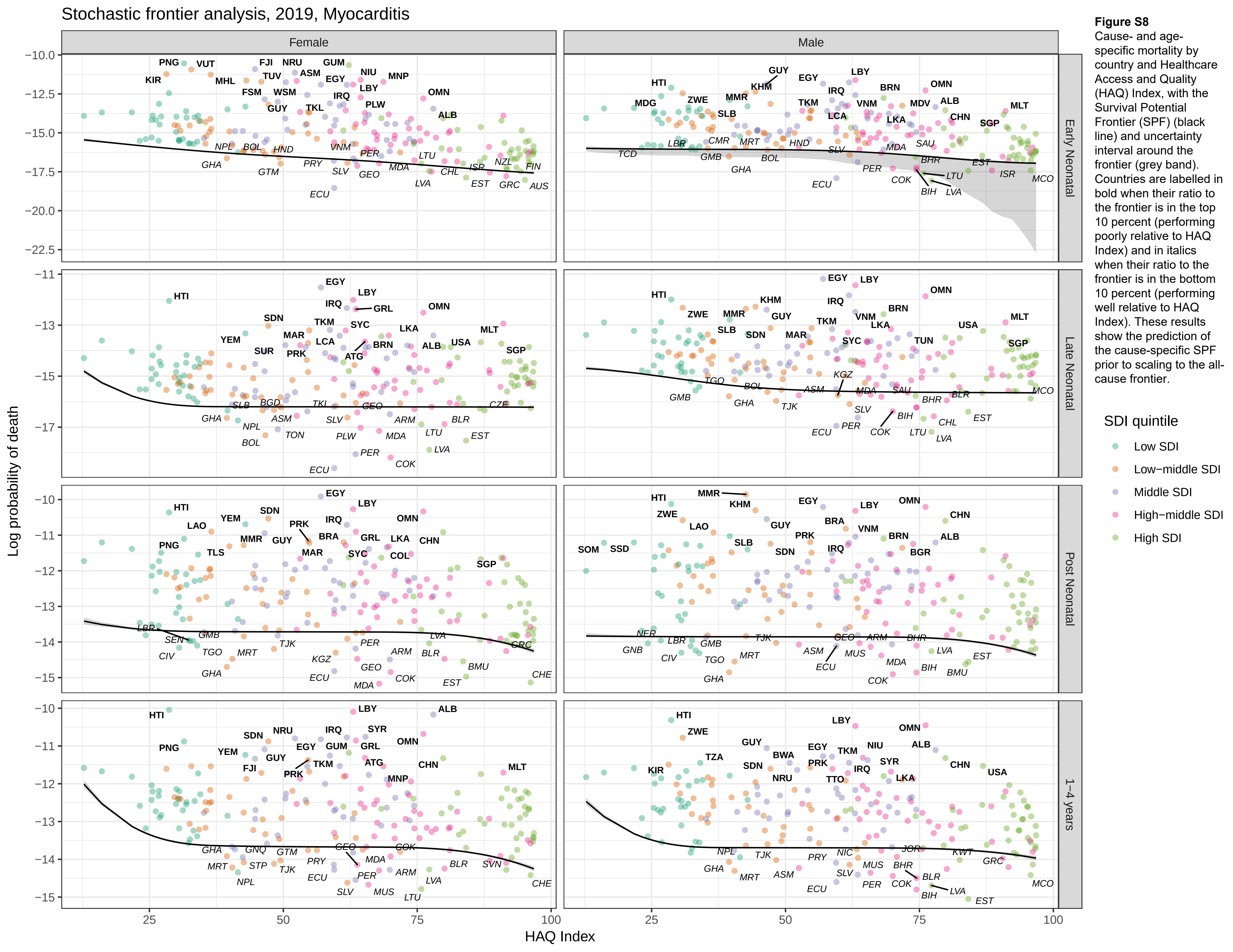

Figure S8
Cause- and age-specific mortality by country and Healthcare Access and Quality (HAQ) Index, with the Survival Potential Frontier (SPF) (black line) and uncertainty interval around the frontier (grey band). Countries are labelled in bold when their ratio to the frontier is in the top 10 percent (performing poorly relative to HAQ Index) and in italics when their ratio to the frontier is in the bottom 10 percent (performing well relative to HAQ Index). These results show the prediction of the cause-specific SPF prior to scaling to the all-cause frontier.

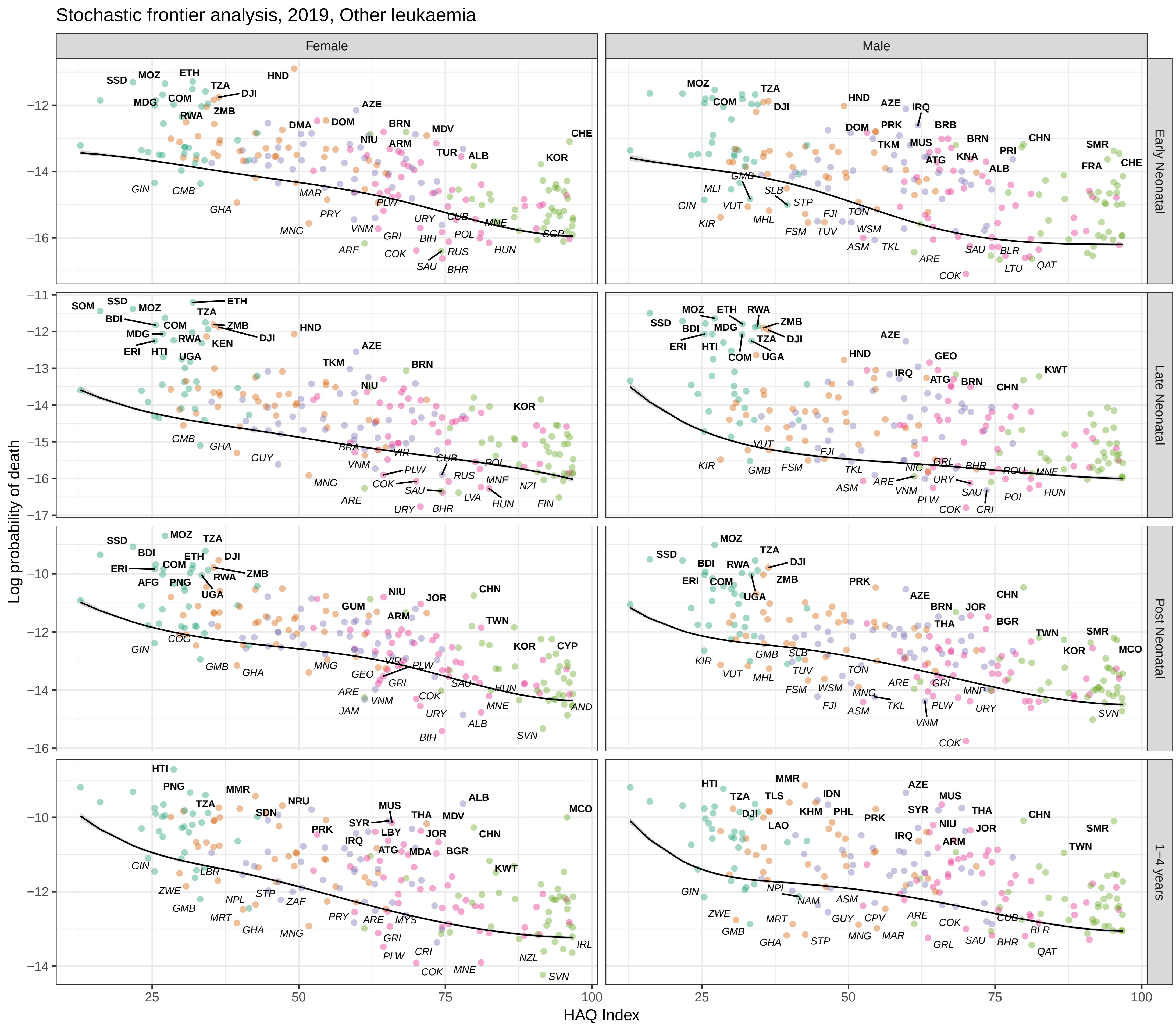

Figure S8
Cause- and age-specific mortality by country and Healthcare Access and Quality (HAQ) Index, with the Survival Potential Frontier (SPF) (black line) and uncertainty interval around the frontier (grey band). Countries are labelled in bold when their ratio to the frontier is in the top 10 percent (performing poorly relative to HAQ Index) and in italics when their ratio to the frontier is in the bottom 10 percent (performing well relative to HAQ Index). These results show the prediction of the cause-specific SPF prior to scaling to the all-cause frontier.

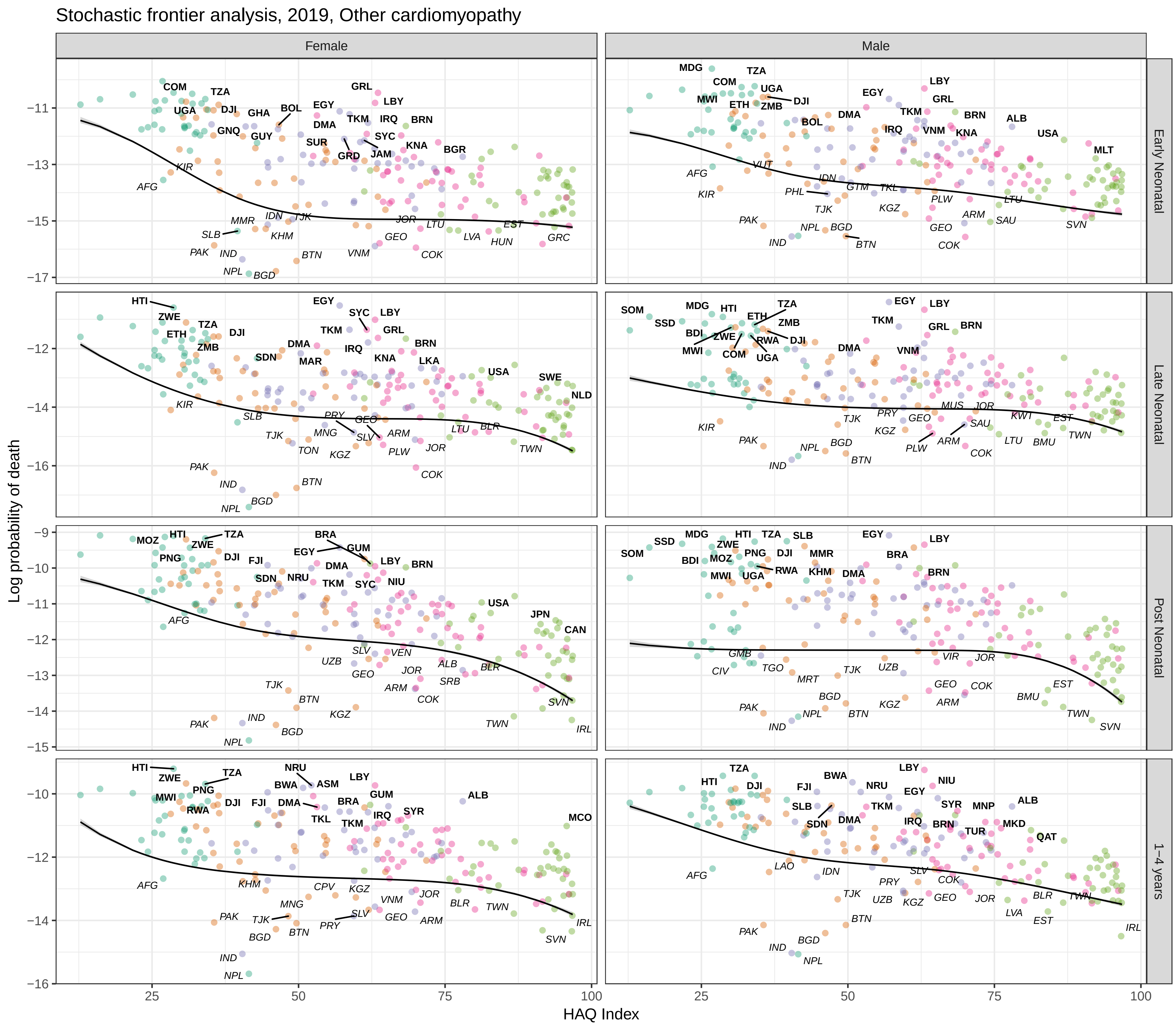

Figure S8
Cause- and age-specific mortality by country and Healthcare Access and Quality (HAQ) Index, with the Survival Potential Frontier (SPF) (black line) and uncertainty interval around the frontier (grey band). Countries are labelled in bold when their ratio to the frontier is in the top 10 percent (performing poorly relative to HAQ Index) and in italics when their ratio to the frontier is in the bottom 10 percent (performing well relative to HAQ Index). These results show the prediction of the cause-specific SPF prior to scaling to the all-cause frontier.

SDI quintile
- Low SDI
- Low−middle SDI
- Middle SDI
- High−middle SDI
- High SDI

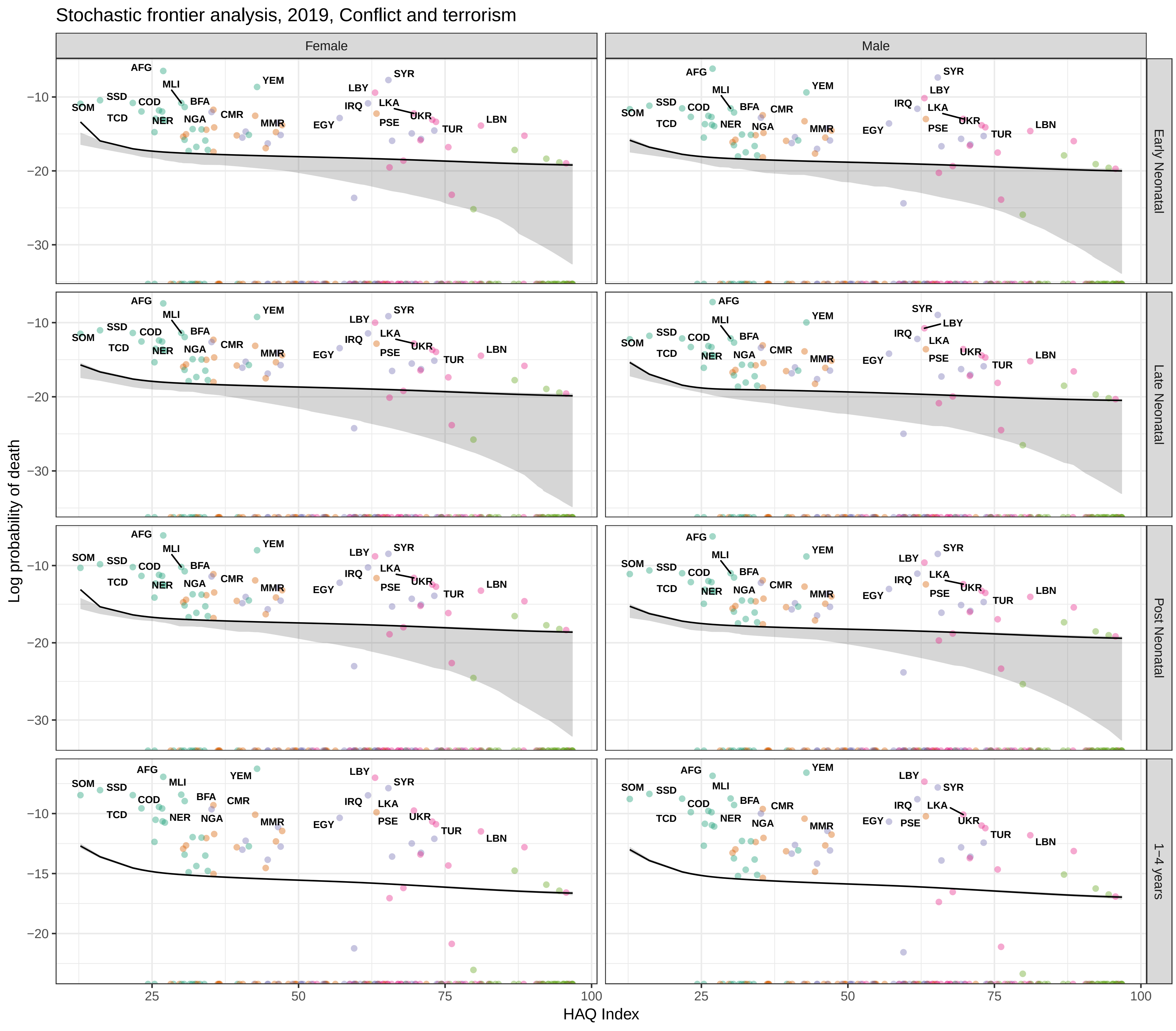

Figure S8
Cause- and age-specific mortality by country and Healthcare Access and Quality (HAQ) Index, with the Survival Potential Frontier (SPF) (black line) and uncertainty interval around the frontier (grey band). Countries are labelled in bold when their ratio to the frontier is in the top 10 percent (performing poorly relative to HAQ Index) and in italics when their ratio to the frontier is in the bottom 10 percent (performing well relative to HAQ Index). These results show the prediction of the cause-specific SPF prior to scaling to the all-cause frontier.

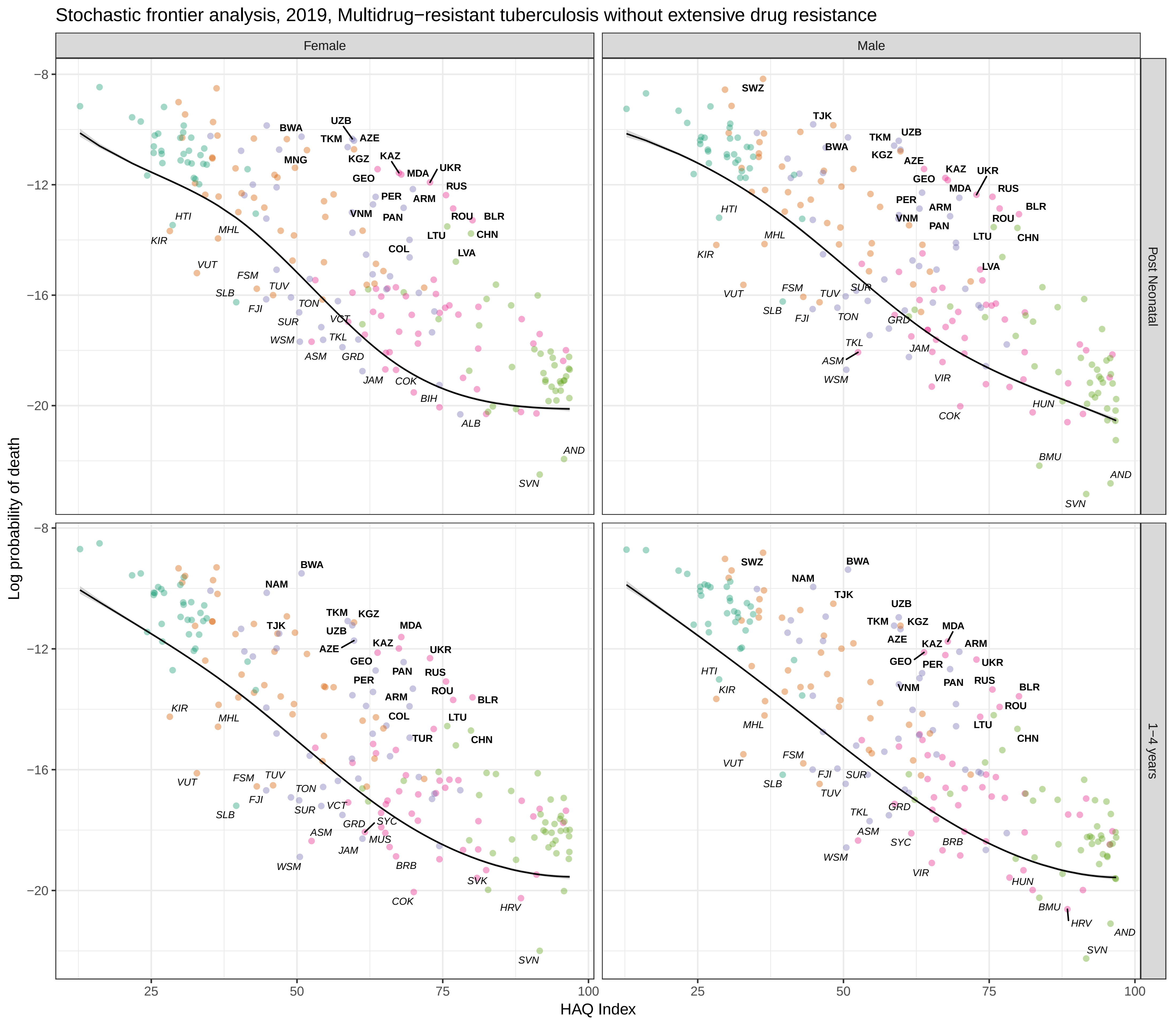

Figure S8
Cause- and age-specific mortality by country and Healthcare Access and Quality (HAQ) Index, with the Survival Potential Frontier (SPF) (black line) and uncertainty interval around the frontier (grey band). Countries are labelled in bold when their ratio to the frontier is in the top 10 percent (performing poorly relative to HAQ Index) and in italics when their ratio to the frontier is in the bottom 10 percent (performing well relative to HAQ Index). These results show the prediction of the cause-specific SPF prior to scaling to the all-cause frontier.

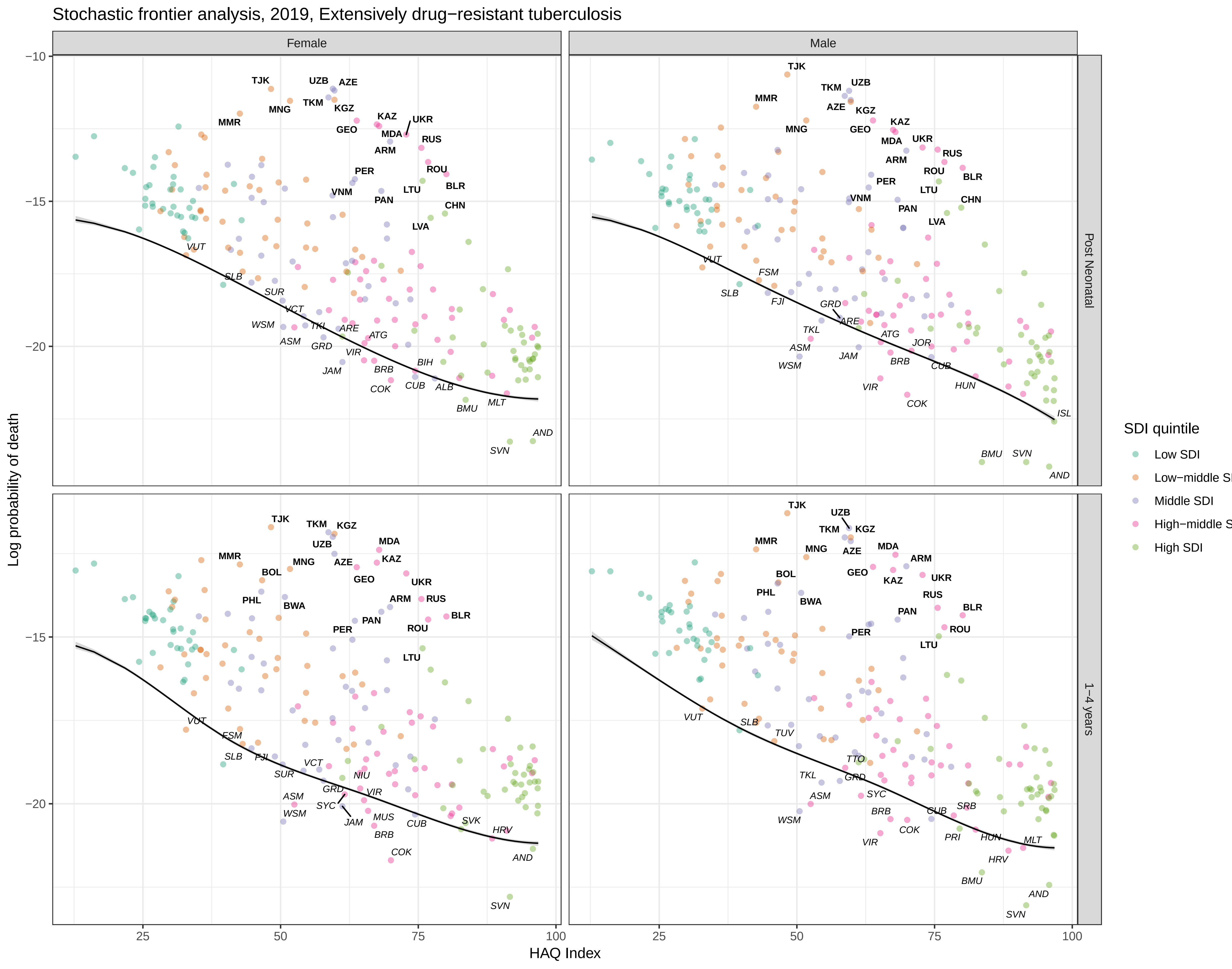

Figur
Caus
speci
coun
Acce
(HAC
Survi
Front
line) a
interv
fronti
Coun
bold
the fr
10 pe
poorl
Index
when
fronti
10 pe
well r
Index
show
the ca
prior
caus

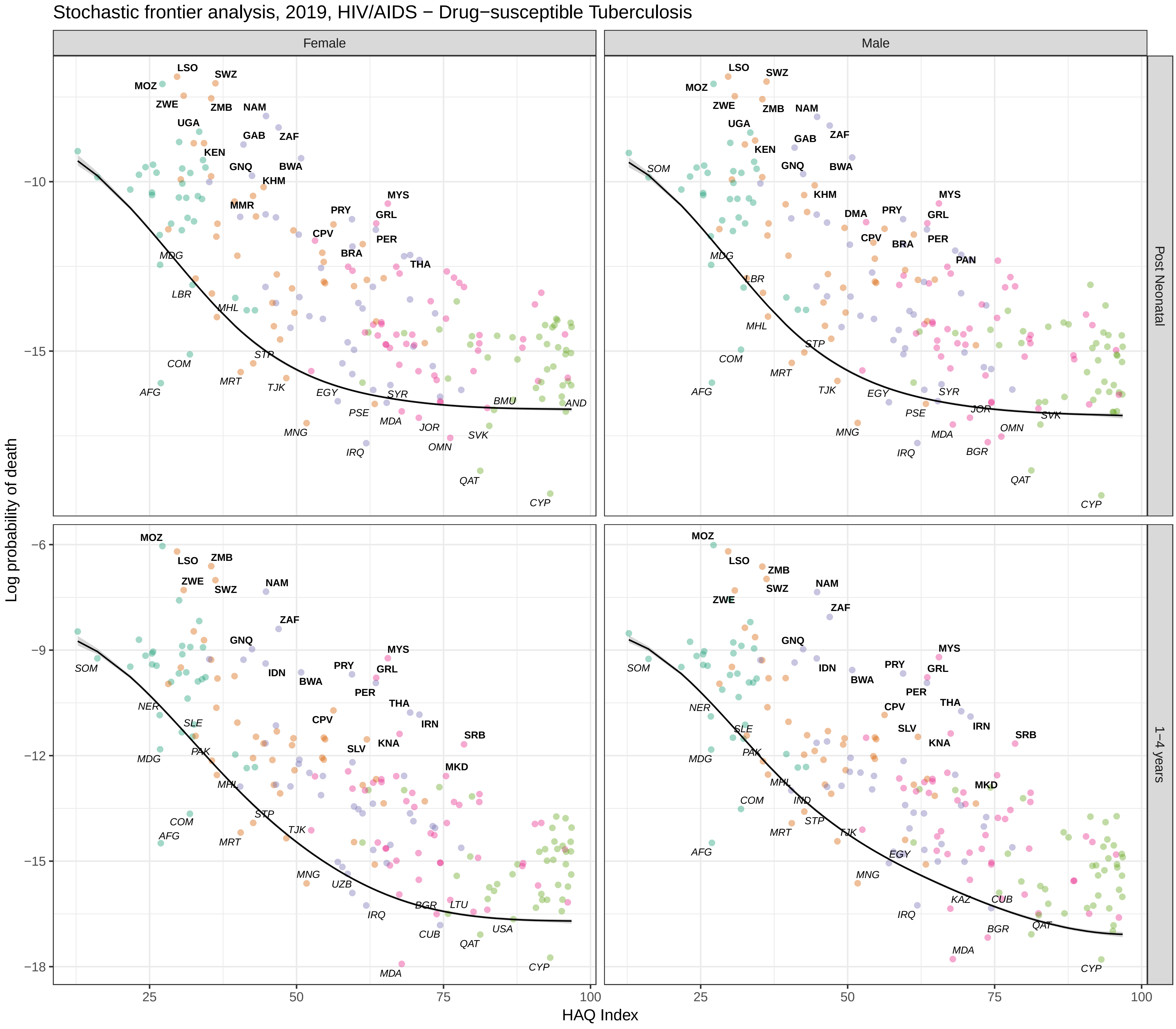

Figure S8
Cause- and age-specific mortality by country and Healthcare Access and Quality (HAQ) Index, with the Survival Potential Frontier (SPF) (black line) and uncertainty interval around the frontier (grey band). Countries are labelled in bold when their ratio to the frontier is in the top 10 percent (performing poorly relative to HAQ Index) and in italics when their ratio to the frontier is in the bottom 10 percent (performing well relative to HAQ Index). These results show the prediction of the cause-specific SPF prior to scaling to the all-cause frontier.

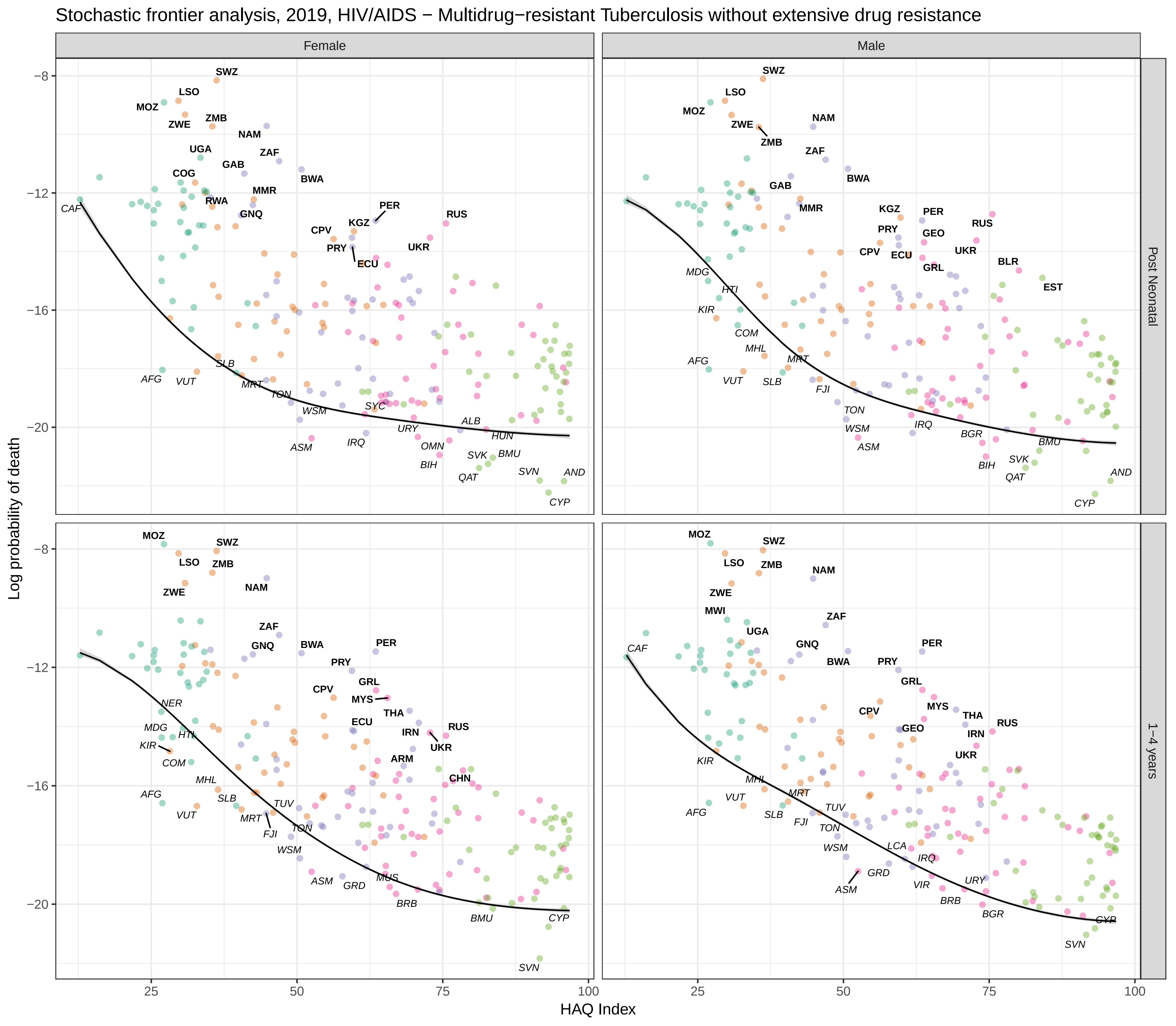

Figure S8
Cause- and age-specific mortality by country and Healthcare Access and Quality (HAQ) Index, with the Survival Potential Frontier (SPF) (black line) and uncertainty interval around the frontier (grey band). Countries are labelled in bold when their ratio to the frontier is in the top 10 percent (performing poorly relative to HAQ Index) and in italics when their ratio to the frontier is in the bottom 10 percent (performing well relative to HAQ Index). These results show the prediction of the cause-specific SPF prior to scaling to the all-cause frontier.

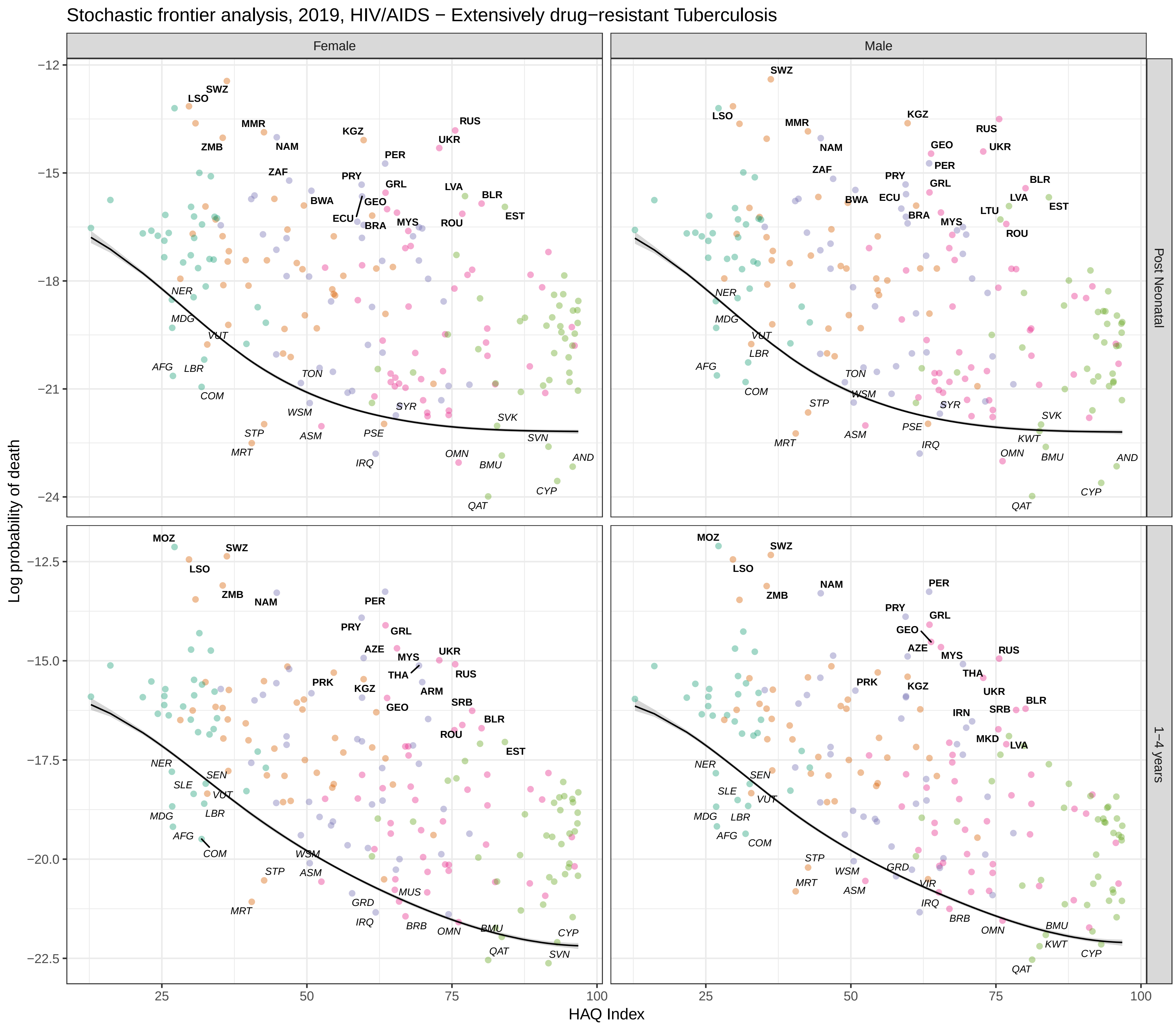

Figure S8
Cause- and age-specific mortality by country and Healthcare Access and Quality (HAQ) Index, with the Survival Potential Frontier (SPF) (black line) and uncertainty interval around the frontier (grey band). Countries are labelled in bold when their ratio to the frontier is in the top 10 percent (performing poorly relative to HAQ Index) and in italics when their ratio to the frontier is in the bottom 10 percent (performing well relative to HAQ Index). These results show the prediction of the cause-specific SPF prior to scaling to the all-cause frontier.

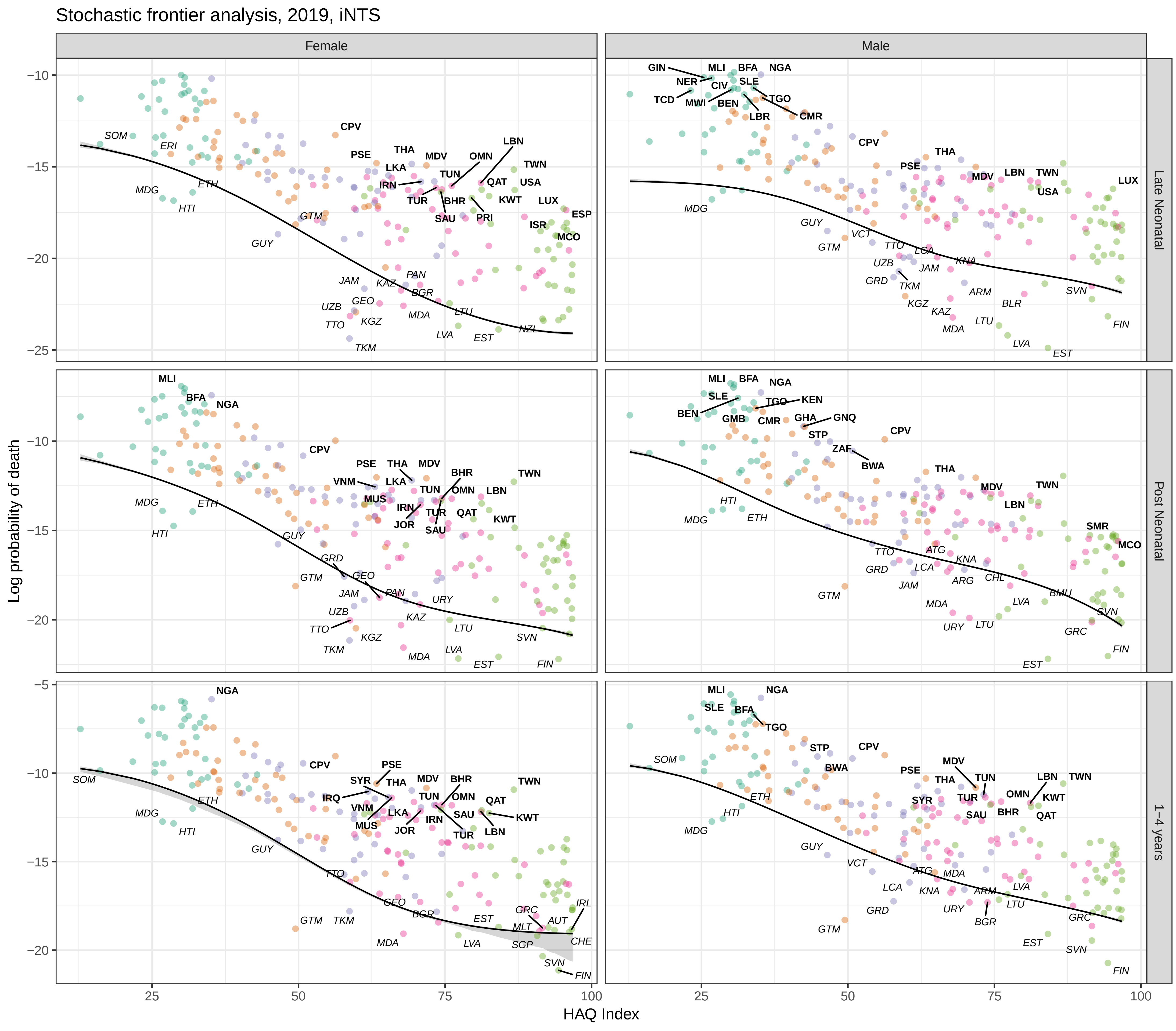

Figure S8
Cause- and age-specific mortality by country and Healthcare Access and Quality (HAQ) Index, with the Survival Potential Frontier (SPF) (black line) and uncertainty interval around the frontier (grey band). Countries are labelled in bold when their ratio to the frontier is in the top 10 percent (performing poorly relative to HAQ Index) and in italics when their ratio to the frontier is in the bottom 10 percent (performing well relative to HAQ Index). These results show the prediction of the cause-specific SPF prior to scaling to the all-cause frontier.

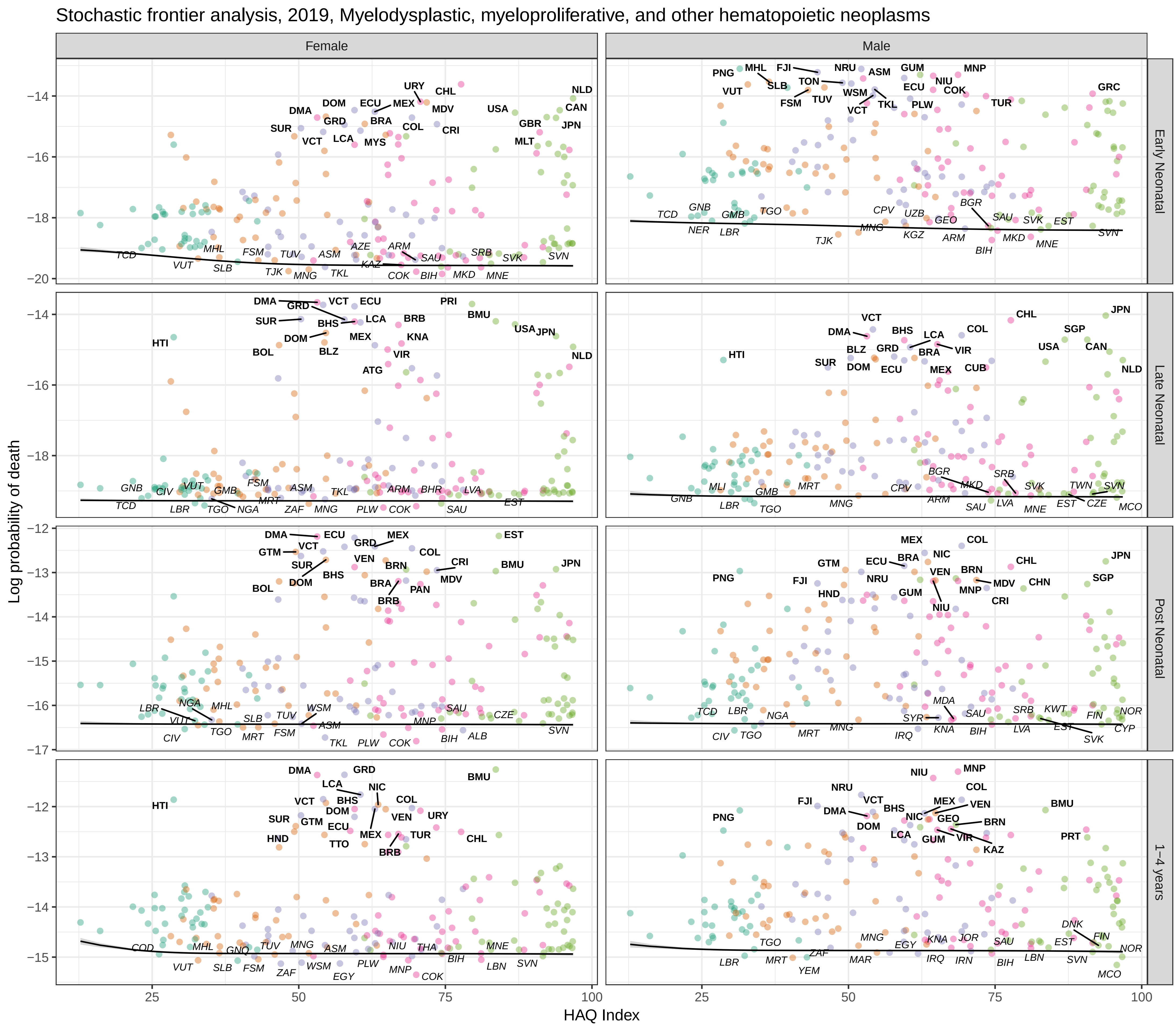

Figure S8
Cause- and age-specific mortality by country and Healthcare Access and Quality (HAQ) Index, with the Survival Potential Frontier (SPF) (black line) and uncertainty interval around the frontier (grey band). Countries are labelled in bold when their ratio to the frontier is in the top 10 percent (performing poorly relative to HAQ Index) and in italics when their ratio to the frontier is in the bottom 10 percent (performing well relative to HAQ Index). These results show the prediction of the cause-specific SPF prior to scaling to the all-cause frontier.

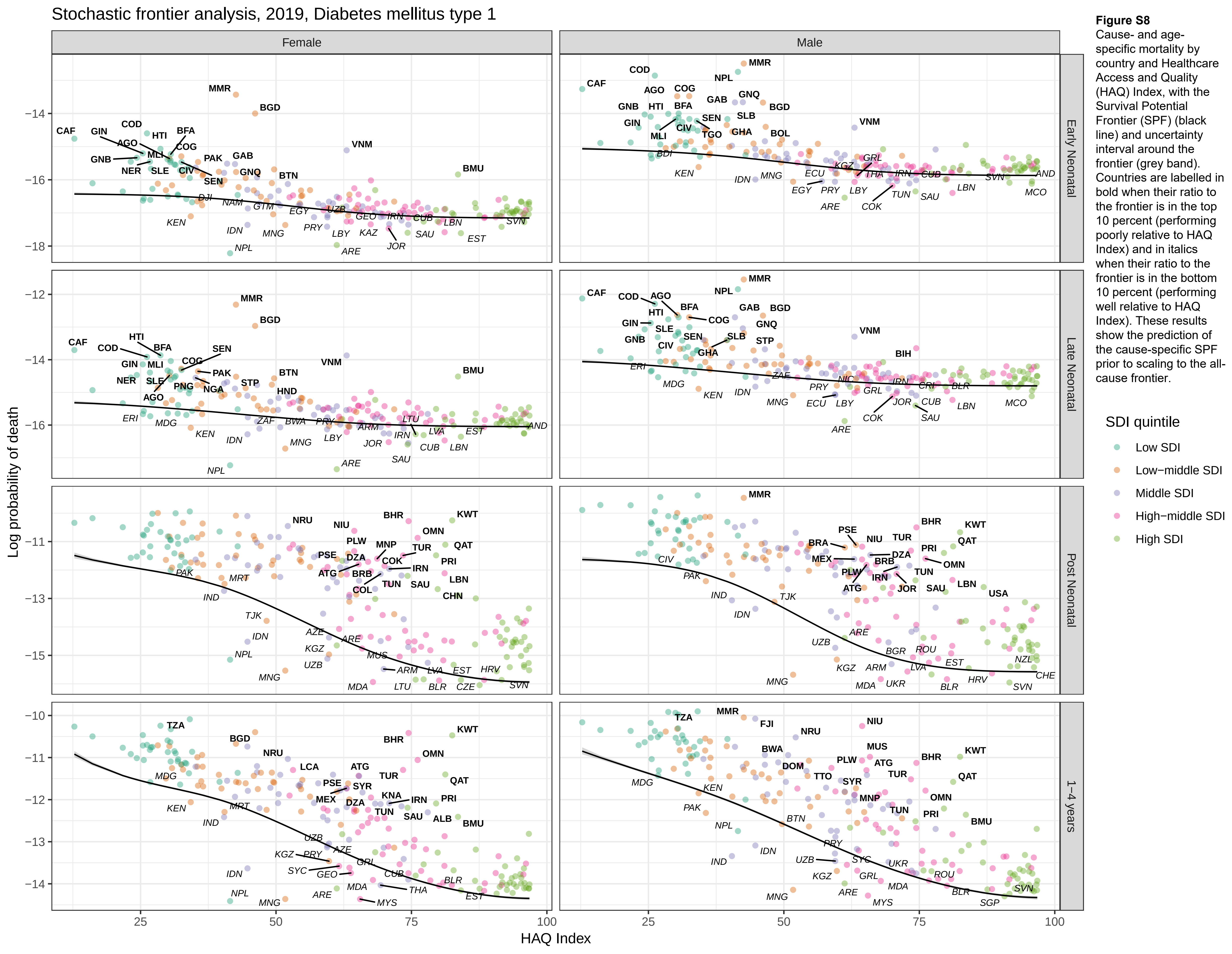

Figure S8
Cause- and age-specific mortality by country and Healthcare Access and Quality (HAQ) Index, with the Survival Potential Frontier (SPF) (black line) and uncertainty interval around the frontier (grey band). Countries are labelled in bold when their ratio to the frontier is in the top 10 percent (performing poorly relative to HAQ Index) and in italics when their ratio to the frontier is in the bottom 10 percent (performing well relative to HAQ Index). These results show the prediction of the cause-specific SPF prior to scaling to the all-cause frontier.

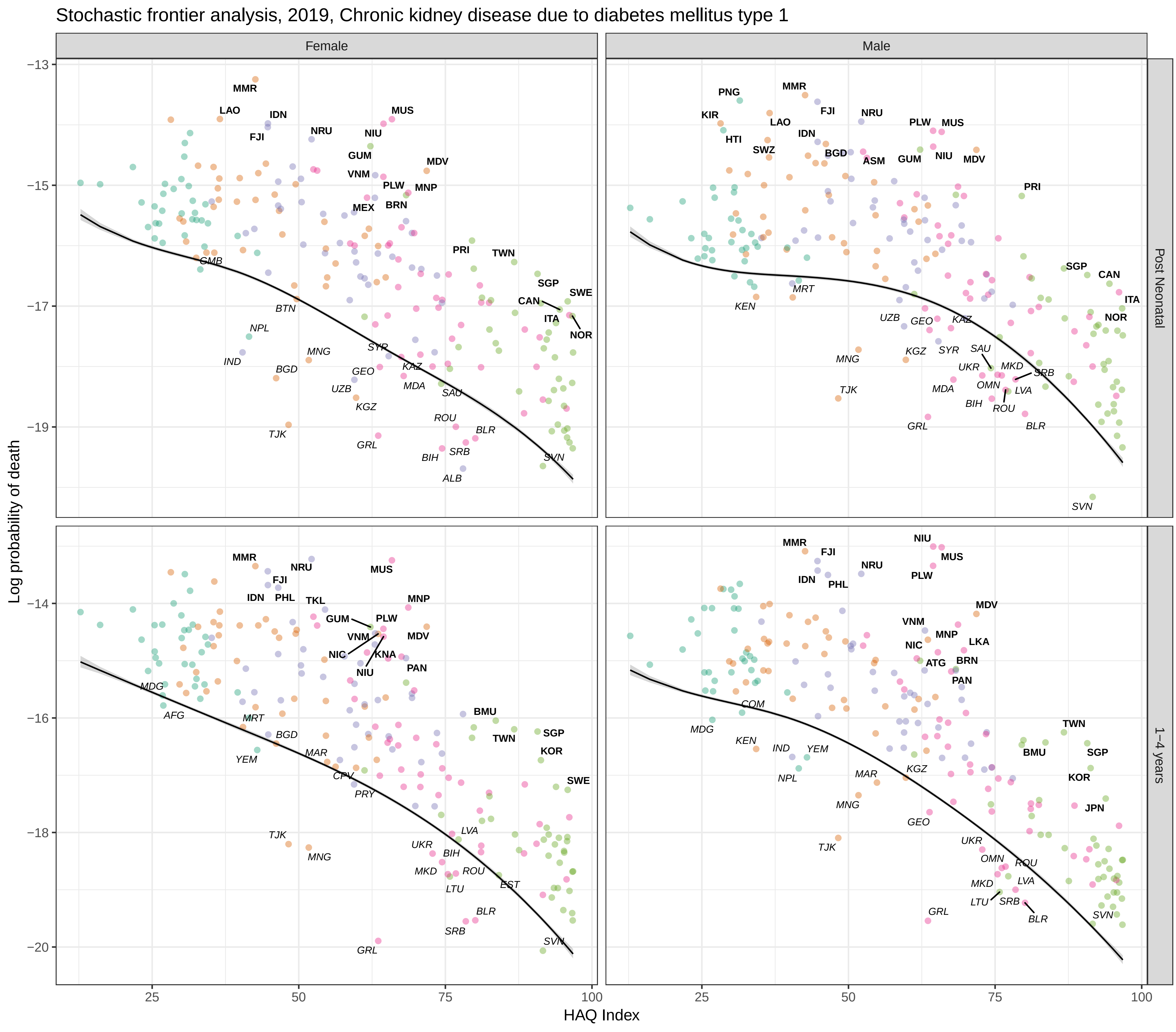

Figure S8
Cause- and age-specific mortality by country and Healthcare Access and Quality (HAQ) Index, with the Survival Potential Frontier (SPF) (black line) and uncertainty interval around the frontier (grey band). Countries are labelled in bold when their ratio to the frontier is in the top 10 percent (performing poorly relative to HAQ Index) and in italics when their ratio to the frontier is in the bottom 10 percent (performing well relative to HAQ Index). These results show the prediction of the cause-specific SPF prior to scaling to the all-cause frontier.

Cambodia

Figure S9. Results by location

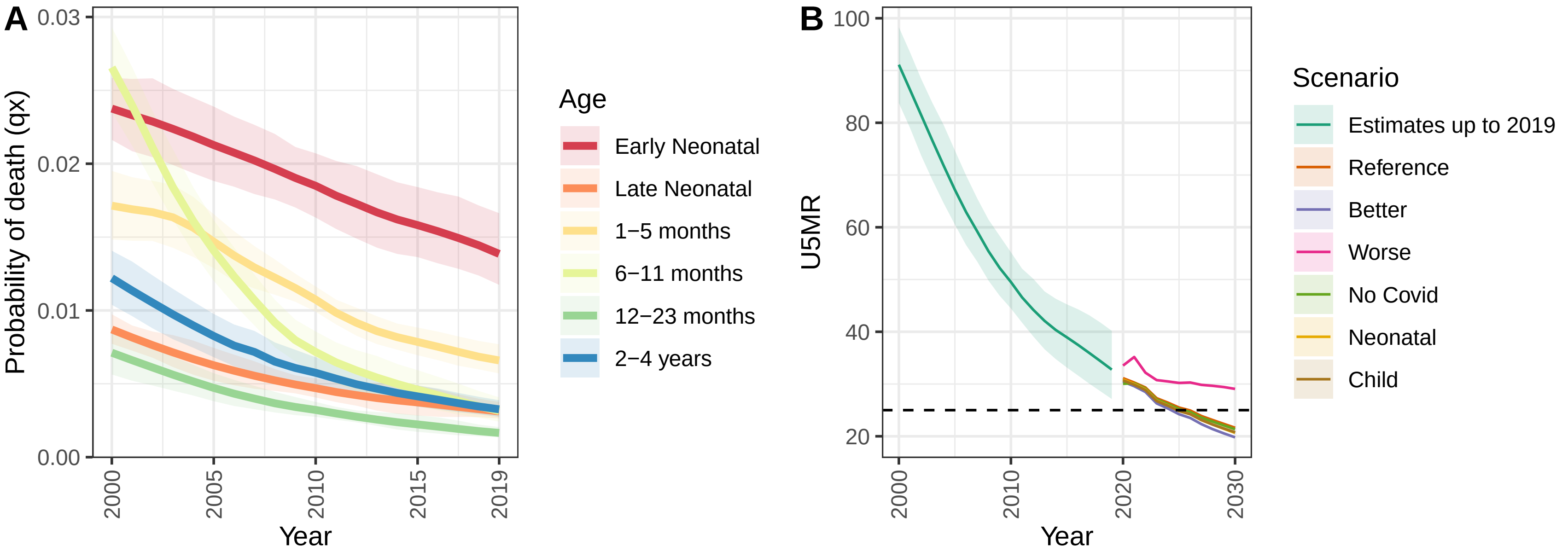

C

Cause of Death	Neonatal Female 2000	Neonatal Female 2019	Neonatal Male 2000	Neonatal Male 2019	Under 5 Female 2000	Under 5 Female 2019	Under 5 Male 2000	Under 5 Male 2019
Neonatal disorders	1	1	1	1	2	1	2	1
Lower respiratory infections	2	2	2	2	1	2	1	2
Congenital anomalies	3	3	3	3	3	3	3	3
Drowning	12	12	13	13	5	4	5	4
Meningitis	4	6	5	7	4	5	4	5
Sudden infant death syndrome	6	5	6	5	6	6	6	6
Road injuries	8	9	9	10	7	7	7	7
Other unspecified infectious diseases	5	4	4	4	9	9	9	8
Foreign body	7	7	8	8	8	8	8	9
Cardiomyopathy and myocarditis	14	14	7	6	14	14	10	10
Other malignant neoplasms	13	13	14	14	12	10	13	12
Endocrine, metabolic, blood, and immune disorders	11	10	10	9	11	11	11	11
Adverse effects of medical treatment	9	11	11	12	10	12	12	13
Interpersonal violence	10	8	12	11	13	13	14	14

Year

D Cambodia: NN ratio to SPF: 1.34 // U5 ratio to SPF: 1.35

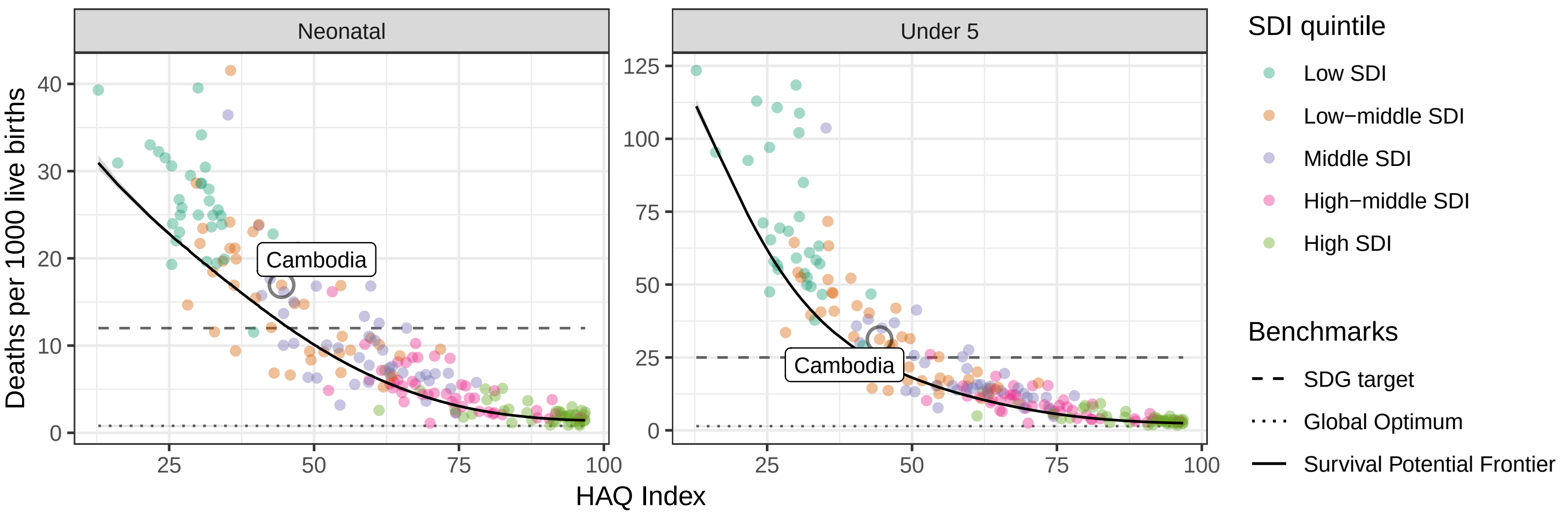

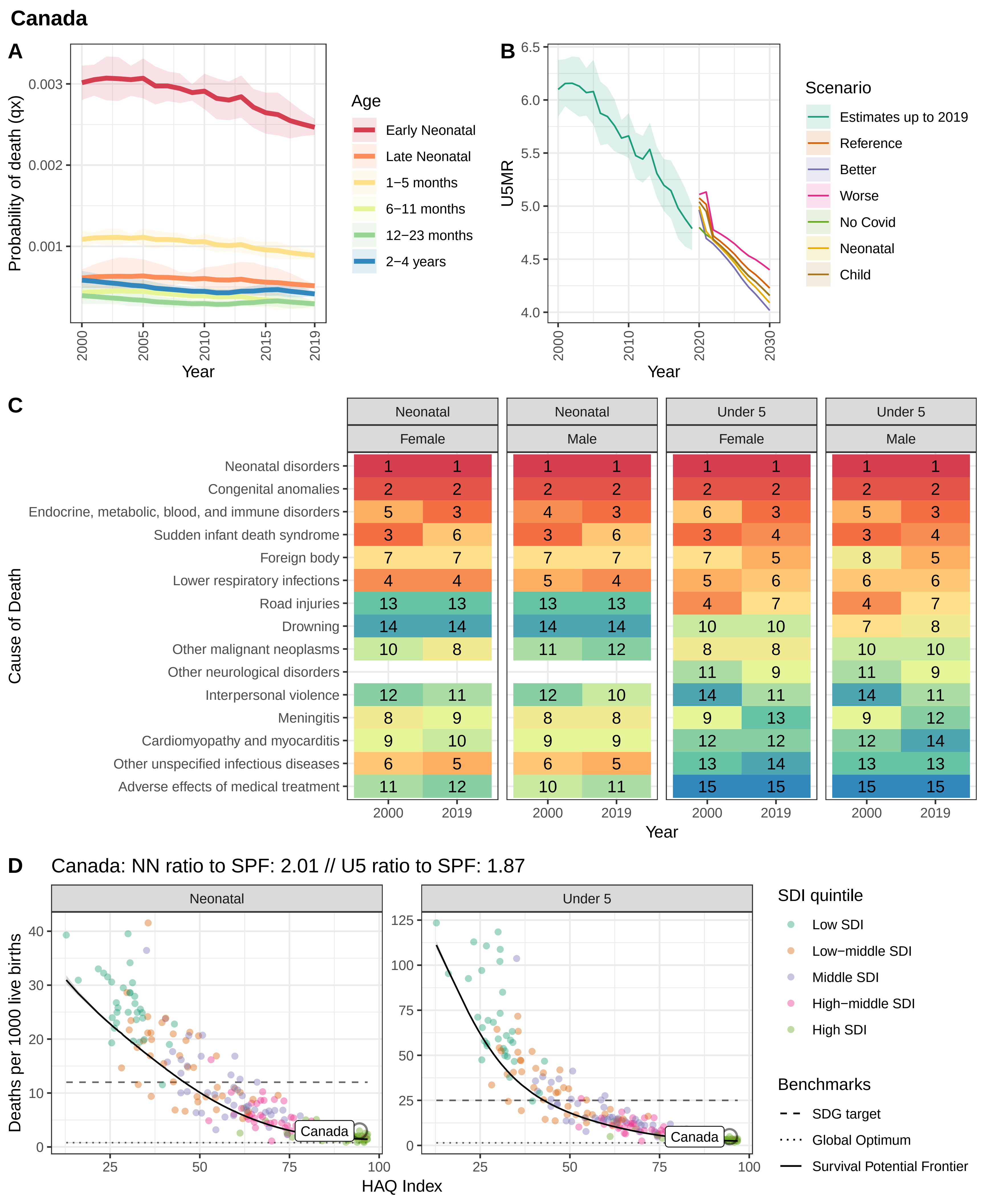
Canada
A
Probability of death (qx)
0.003
0.002
0.001
2000
2005
2010
2015
2019
Year
Age
Early Neonatal
Late Neonatal
1−5 months
6−11 months
12−23 months
2−4 years
B
U5MR
6.5
6.0
5.5
5.0
4.5
4.0
2000
2010
2020
2030
Year
Scenario
Estimates up to 2019
Reference
Better
Worse
No Covid
Neonatal
Child
C
Cause of Death
Neonatal / Female
Neonatal / Male
Under 5 / Female
Under 5 / Male
Neonatal disorders 1 1 1 1 1 1 1 1
Congenital anomalies 2 2 2 2 2 2 2 2
Endocrine, metabolic, blood, and immune disorders 5 3 4 3 6 3 5 3
Sudden infant death syndrome 3 6 3 6 3 4 3 4
Foreign body 7 7 7 7 7 5 8 5
Lower respiratory infections 4 4 5 4 5 6 6 6
Road injuries 13 13 13 13 4 7 4 7
Drowning 14 14 14 14 10 10 7 8
Other malignant neoplasms 10 8 11 12 8 8 10 10
Other neurological disorders 11 9 11 9
Interpersonal violence 12 11 12 10 14 11 14 11
Meningitis 8 9 8 8 9 13 9 12
Cardiomyopathy and myocarditis 9 10 9 9 12 12 12 14
Other unspecified infectious diseases 6 5 6 5 13 14 13 13
Adverse effects of medical treatment 11 12 10 11 15 15 15 15
2000
2019
Year
D
Canada: NN ratio to SPF: 2.01 // U5 ratio to SPF: 1.87
Neonatal
Under 5
Deaths per 1000 live births
40
30
20
10
0
125
100
75
50
25
0
25
50
75
100
HAQ Index
Canada
SDI quintile
Low SDI
Low−middle SDI
Middle SDI
High−middle SDI
High SDI
Benchmarks
SDG target
Global Optimum
Survival Potential Frontier

United States of America

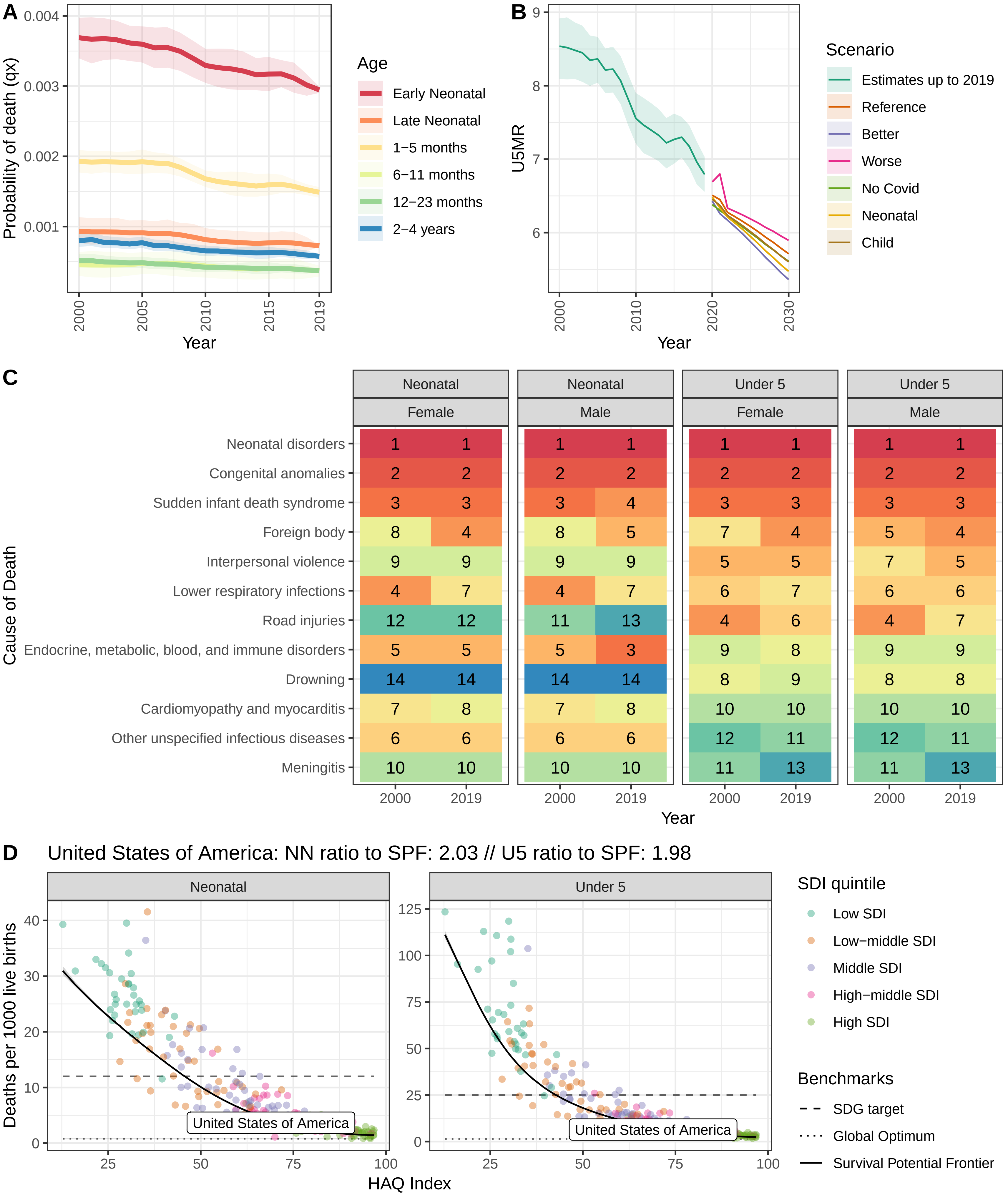

Antigua and Barbuda

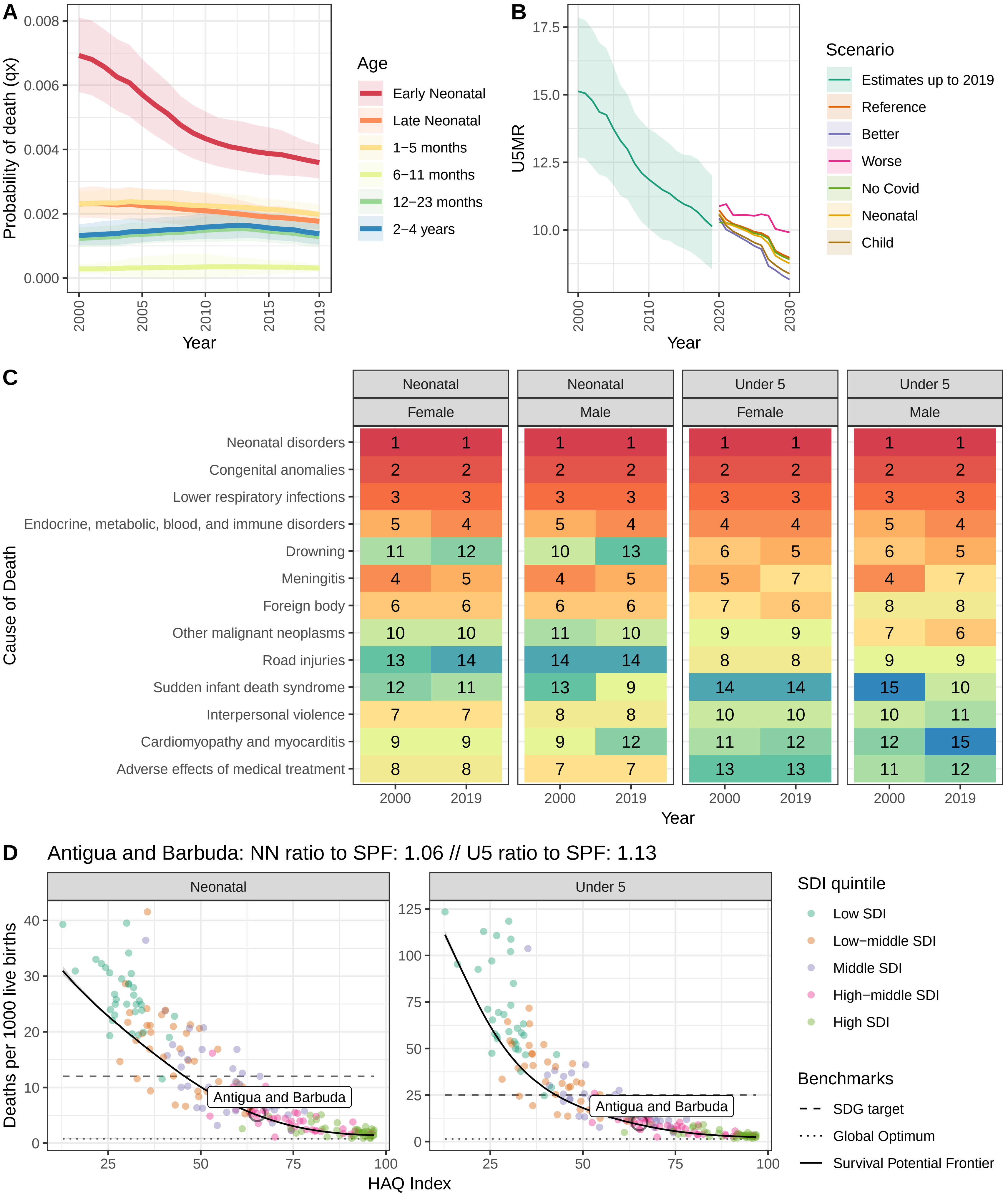

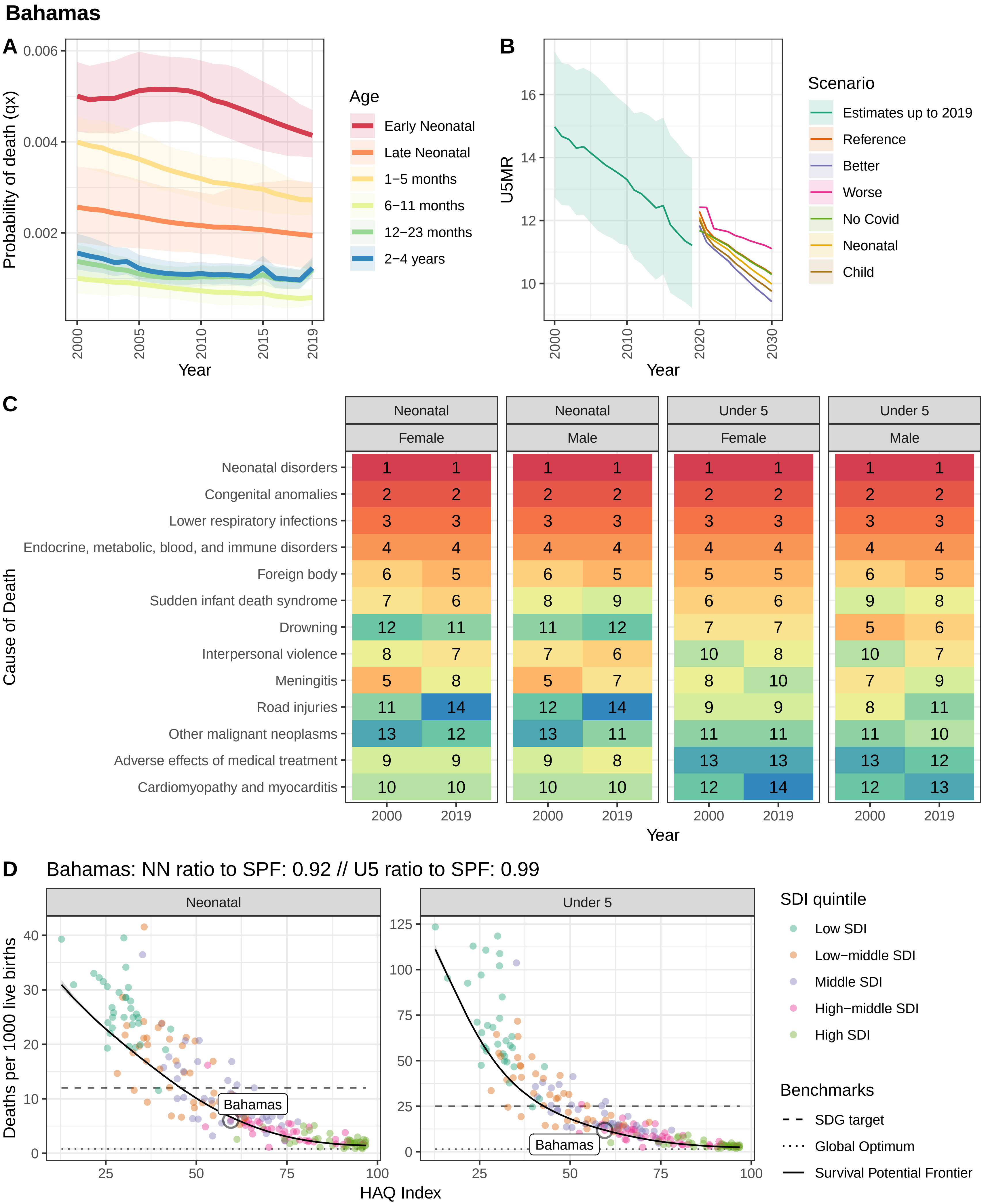
Bahamas
A
Probability of death (qx)
Year
Age
Early Neonatal
Late Neonatal
1−5 months
6−11 months
12−23 months
2−4 years
B
U5MR
Year
Scenario
Estimates up to 2019
Reference
Better
Worse
No Covid
Neonatal
Child
C
Neonatal
Female
Neonatal
Male
Under 5
Female
Under 5
Male
Cause of Death
Neonatal disorders
Congenital anomalies
Lower respiratory infections
Endocrine, metabolic, blood, and immune disorders
Foreign body
Sudden infant death syndrome
Drowning
Interpersonal violence
Meningitis
Road injuries
Other malignant neoplasms
Adverse effects of medical treatment
Cardiomyopathy and myocarditis
Year
D
Bahamas: NN ratio to SPF: 0.92 // U5 ratio to SPF: 0.99
Neonatal
Under 5
Deaths per 1000 live births
HAQ Index
Bahamas
SDI quintile
Low SDI
Low−middle SDI
Middle SDI
High−middle SDI
High SDI
Benchmarks
SDG target
Global Optimum
Survival Potential Frontier

Barbados

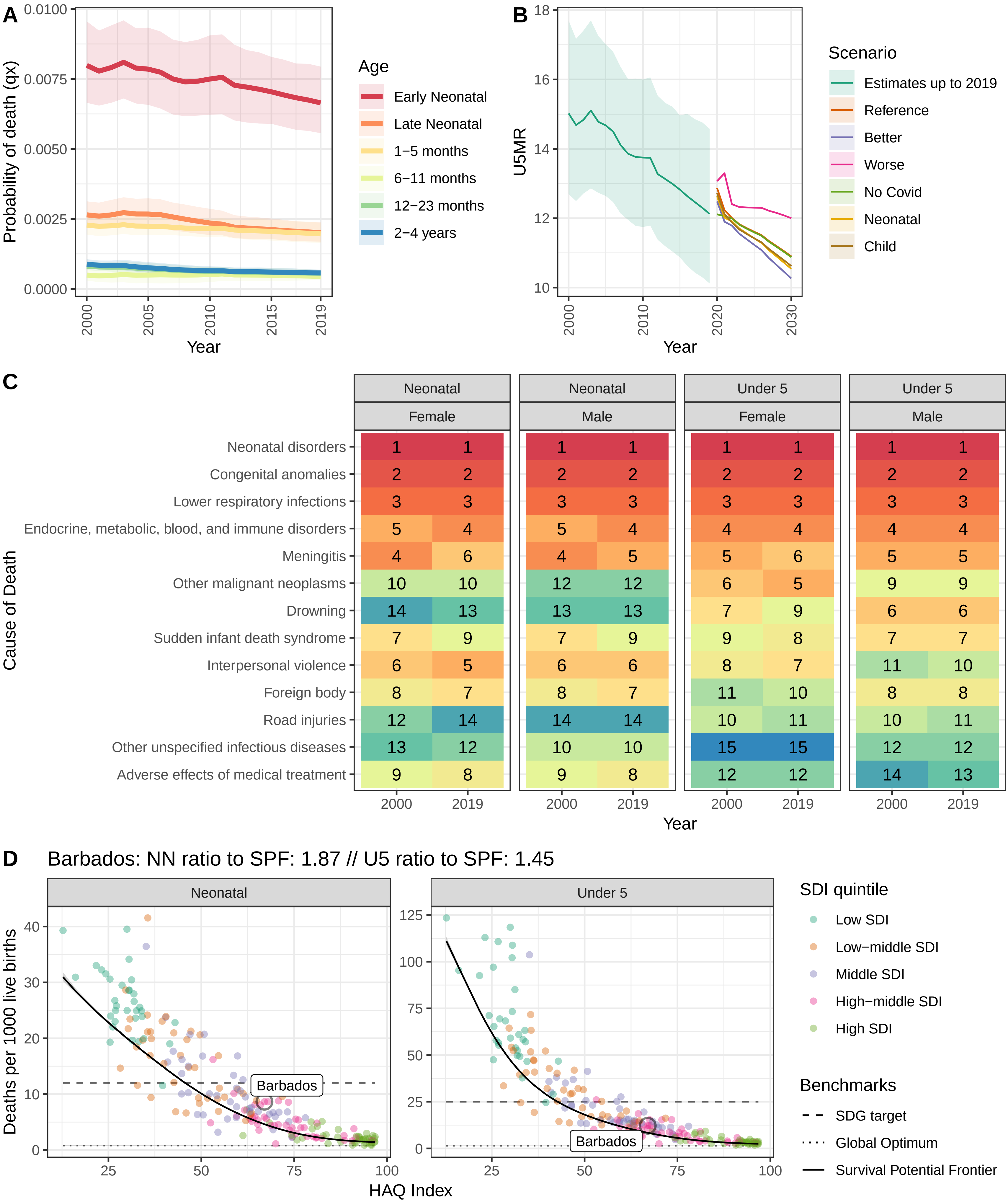
A
Probability of death (qx)
Year
Age
Early Neonatal
Late Neonatal
1−5 months
6−11 months
12−23 months
2−4 years
B
U5MR
Year
Scenario
Estimates up to 2019
Reference
Better
Worse
No Covid
Neonatal
Child
C
Cause of Death
Neonatal Female
Neonatal Male
Under 5 Female
Under 5 Male
Neonatal disorders
Congenital anomalies
Lower respiratory infections
Endocrine, metabolic, blood, and immune disorders
Meningitis
Other malignant neoplasms
Drowning
Sudden infant death syndrome
Interpersonal violence
Foreign body
Road injuries
Other unspecified infectious diseases
Adverse effects of medical treatment
Year
D
Barbados: NN ratio to SPF: 1.87 // U5 ratio to SPF: 1.45
Neonatal
Under 5
Deaths per 1000 live births
HAQ Index
Barbados
SDI quintile
Low SDI
Low−middle SDI
Middle SDI
High−middle SDI
High SDI
Benchmarks
SDG target
Global Optimum
Survival Potential Frontier

Belize

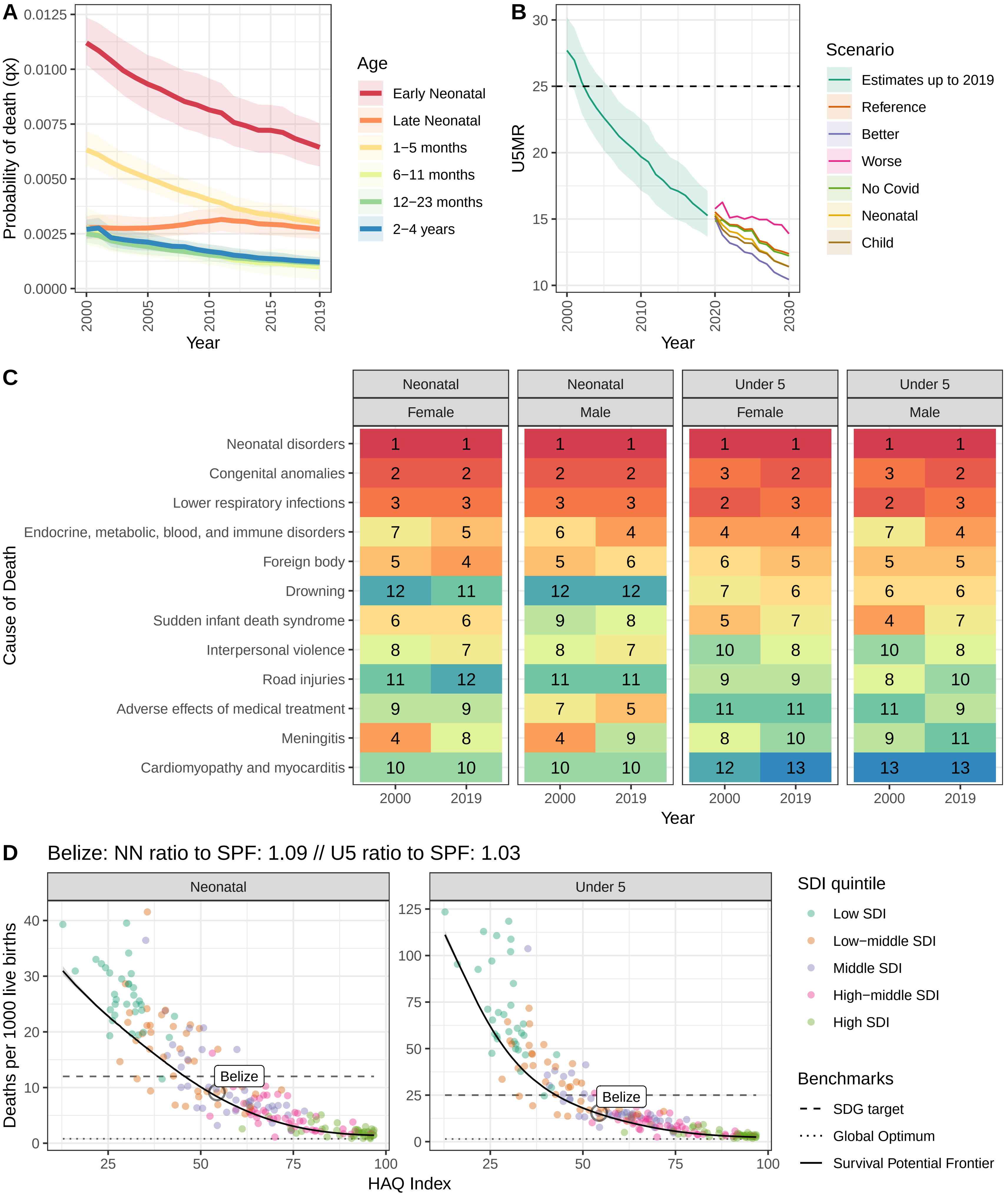

A
Probability of death (qx)
0.0125
0.0100
0.0075
0.0050
0.0025
0.0000
2000
2005
2010
2015
2019
Year
Age
Early Neonatal
Late Neonatal
1−5 months
6−11 months
12−23 months
2−4 years
B
U5MR
30
25
20
15
10
2000
2010
2020
2030
Year
Scenario
Estimates up to 2019
Reference
Better
Worse
No Covid
Neonatal
Child
C
Neonatal
Female
Neonatal
Male
Under 5
Female
Under 5
Male
Cause of Death
Neonatal disorders 1 1 1 1 1 1 1 1
Congenital anomalies 2 2 2 2 3 2 3 2
Lower respiratory infections 3 3 3 3 2 3 2 3
Endocrine, metabolic, blood, and immune disorders 7 5 6 4 4 4 7 4
Foreign body 5 4 5 6 6 5 5 5
Drowning 12 11 12 12 7 6 6 6
Sudden infant death syndrome 6 6 9 8 5 7 4 7
Interpersonal violence 8 7 8 7 10 8 10 8
Road injuries 11 12 11 11 9 9 8 10
Adverse effects of medical treatment 9 9 7 5 11 11 11 9
Meningitis 4 8 4 9 8 10 9 11
Cardiomyopathy and myocarditis 10 10 10 10 12 13 13 13
2000
2019
Year
D
Belize: NN ratio to SPF: 1.09 // U5 ratio to SPF: 1.03
Neonatal
Under 5
Deaths per 1000 live births
40
30
20
10
0
125
100
75
50
25
0
Belize
25
50
75
100
HAQ Index
SDI quintile
Low SDI
Low−middle SDI
Middle SDI
High−middle SDI
High SDI
Benchmarks
SDG target
Global Optimum
Survival Potential Frontier

Cuba

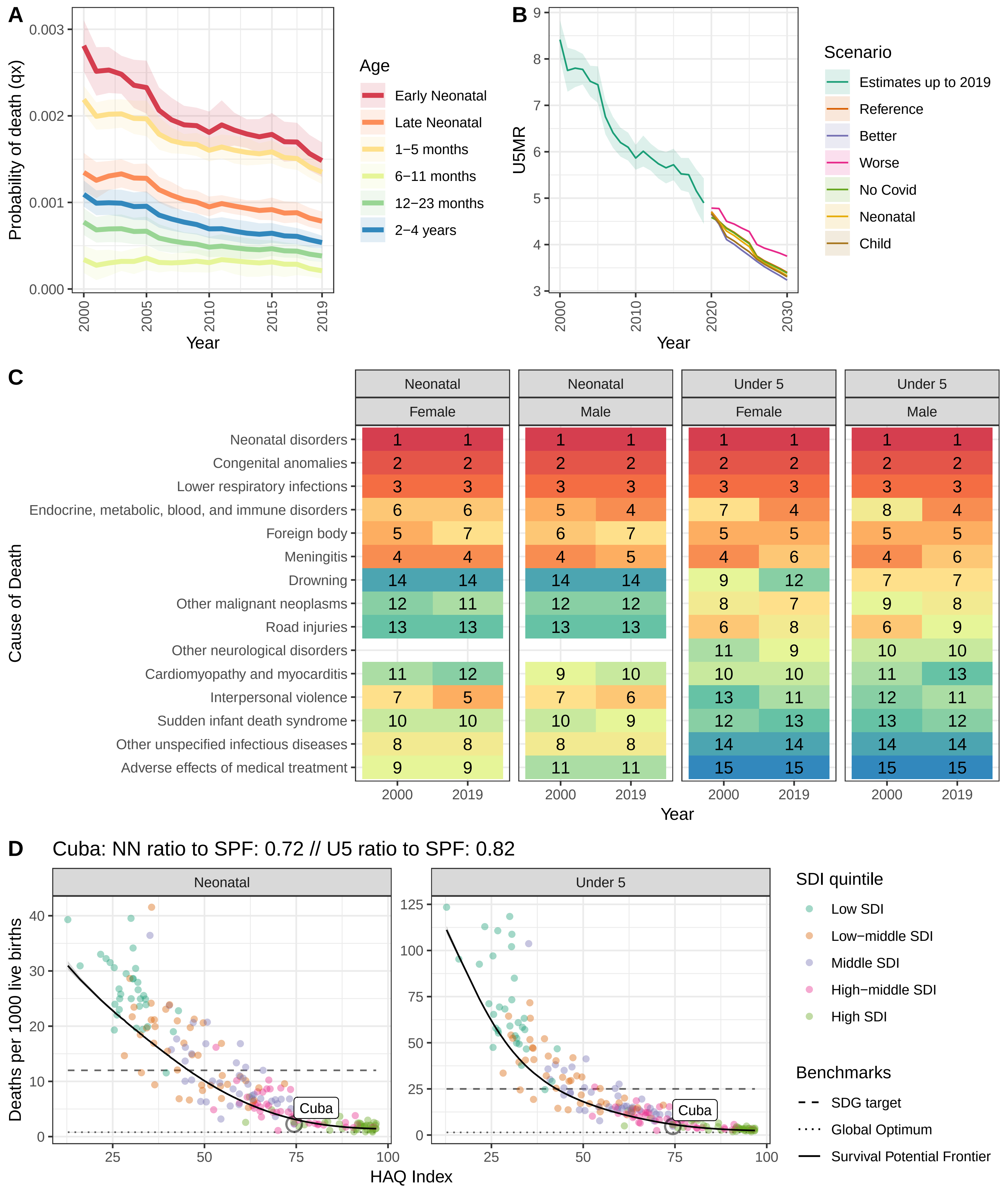
A
Probability of death (qx)
0.003
0.002
0.001
0.000
2000
2005
2010
2015
2019
Year
Age
Early Neonatal
Late Neonatal
1−5 months
6−11 months
12−23 months
2−4 years
B
U5MR
9
8
7
6
5
4
3
2000
2010
2020
2030
Year
Scenario
Estimates up to 2019
Reference
Better
Worse
No Covid
Neonatal
Child
C
Cause of Death
Neonatal | Female; Neonatal | Male; Under 5 | Female; Under 5 | Male
Neonatal disorders 1 1 | 1 1 | 1 1 | 1 1
Congenital anomalies 2 2 | 2 2 | 2 2 | 2 2
Lower respiratory infections 3 3 | 3 3 | 3 3 | 3 3
Endocrine, metabolic, blood, and immune disorders 6 6 | 5 4 | 7 4 | 8 4
Foreign body 5 7 | 6 7 | 5 5 | 5 5
Meningitis 4 4 | 4 5 | 4 6 | 4 6
Drowning 14 14 | 14 14 | 9 12 | 7 7
Other malignant neoplasms 12 11 | 12 12 | 8 7 | 9 8
Road injuries 13 13 | 13 13 | 6 8 | 6 9
Other neurological disorders | | 11 9 | 10 10
Cardiomyopathy and myocarditis 11 12 | 9 10 | 10 10 | 11 13
Interpersonal violence 7 5 | 7 6 | 13 11 | 12 11
Sudden infant death syndrome 10 10 | 10 9 | 12 13 | 13 12
Other unspecified infectious diseases 8 8 | 8 8 | 14 14 | 14 14
Adverse effects of medical treatment 9 9 | 11 11 | 15 15 | 15 15
2000 2019
Year
D
Cuba: NN ratio to SPF: 0.72 // U5 ratio to SPF: 0.82
Neonatal
Under 5
Deaths per 1000 live births
40
30
20
10
0
125
100
75
50
25
0
25
50
75
100
Cuba
HAQ Index
SDI quintile
Low SDI
Low−middle SDI
Middle SDI
High−middle SDI
High SDI
Benchmarks
SDG target
Global Optimum
Survival Potential Frontier

Indonesia

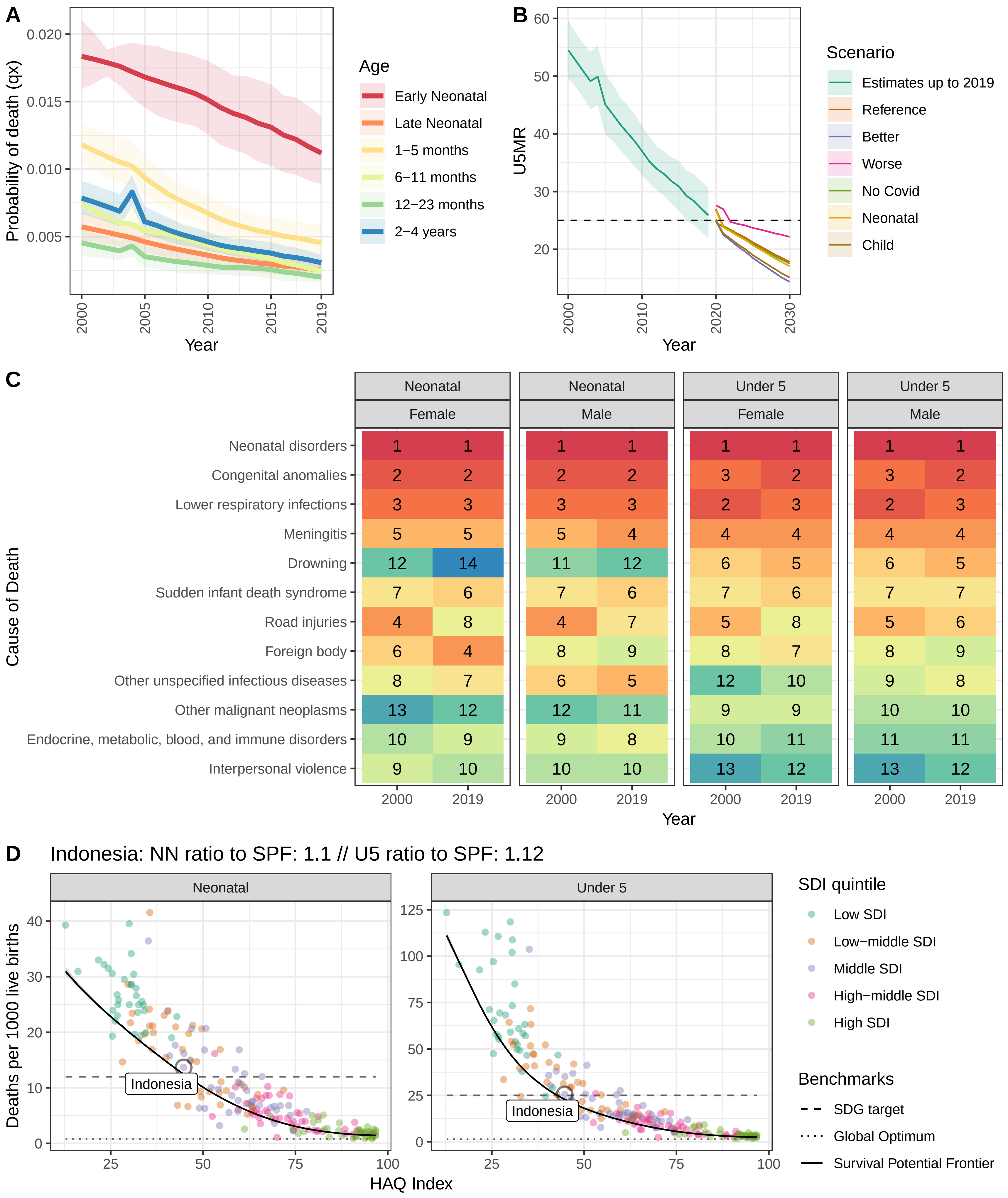
A
Probability of death (qx)
Year
Age
Early Neonatal
Late Neonatal
1−5 months
6−11 months
12−23 months
2−4 years
B
U5MR
Year
Scenario
Estimates up to 2019
Reference
Better
Worse
No Covid
Neonatal
Child
C
Cause of Death
Neonatal
Under 5
Female
Male
Neonatal disorders
Congenital anomalies
Lower respiratory infections
Meningitis
Drowning
Sudden infant death syndrome
Road injuries
Foreign body
Other unspecified infectious diseases
Other malignant neoplasms
Endocrine, metabolic, blood, and immune disorders
Interpersonal violence
Year
D
Indonesia: NN ratio to SPF: 1.1 // U5 ratio to SPF: 1.12
Deaths per 1000 live births
HAQ Index
Indonesia
SDI quintile
Low SDI
Low−middle SDI
Middle SDI
High−middle SDI
High SDI
Benchmarks
SDG target
Global Optimum
Survival Potential Frontier

Dominica

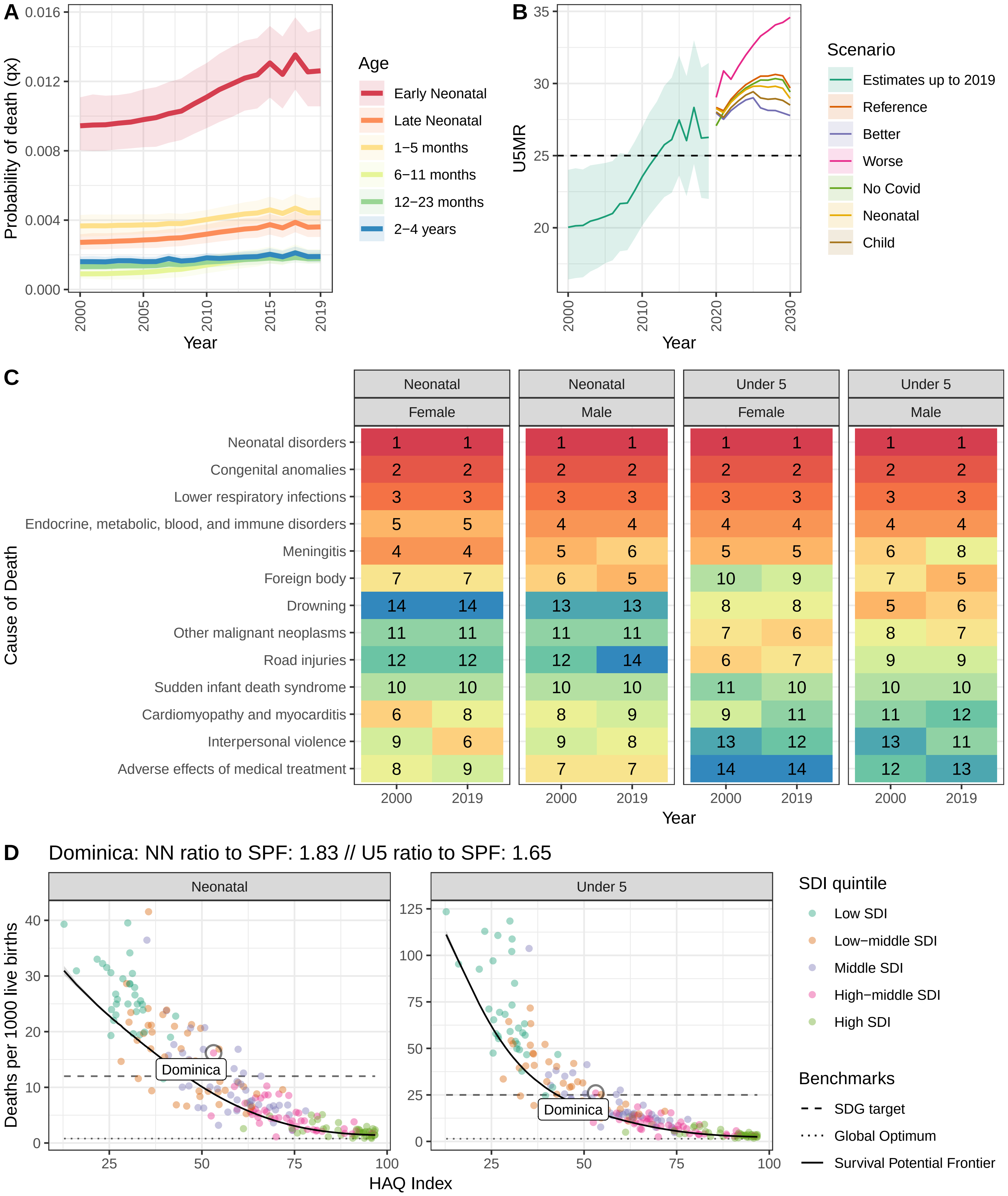
A
Probability of death (qx)
Year
Age
Early Neonatal
Late Neonatal
1−5 months
6−11 months
12−23 months
2−4 years
B
U5MR
Year
Scenario
Estimates up to 2019
Reference
Better
Worse
No Covid
Neonatal
Child
C
Cause of Death
Neonatal
Under 5
Female
Male
Neonatal disorders
Congenital anomalies
Lower respiratory infections
Endocrine, metabolic, blood, and immune disorders
Meningitis
Foreign body
Drowning
Other malignant neoplasms
Road injuries
Sudden infant death syndrome
Cardiomyopathy and myocarditis
Interpersonal violence
Adverse effects of medical treatment
Year
D
Dominica: NN ratio to SPF: 1.83 // U5 ratio to SPF: 1.65
Neonatal
Under 5
Deaths per 1000 live births
HAQ Index
Dominica
SDI quintile
Low SDI
Low−middle SDI
Middle SDI
High−middle SDI
High SDI
Benchmarks
SDG target
Global Optimum
Survival Potential Frontier

Dominican Republic

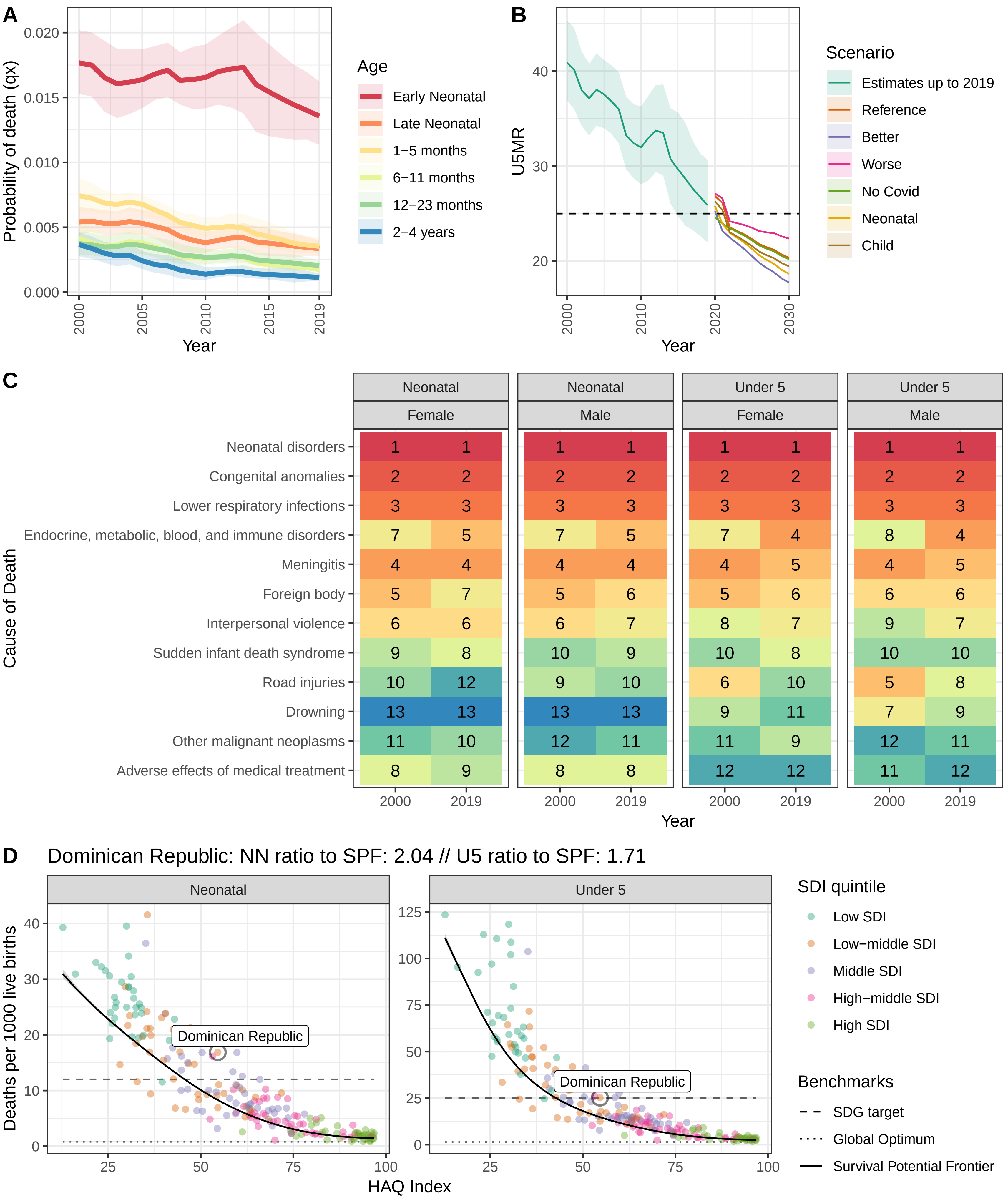

Grenada

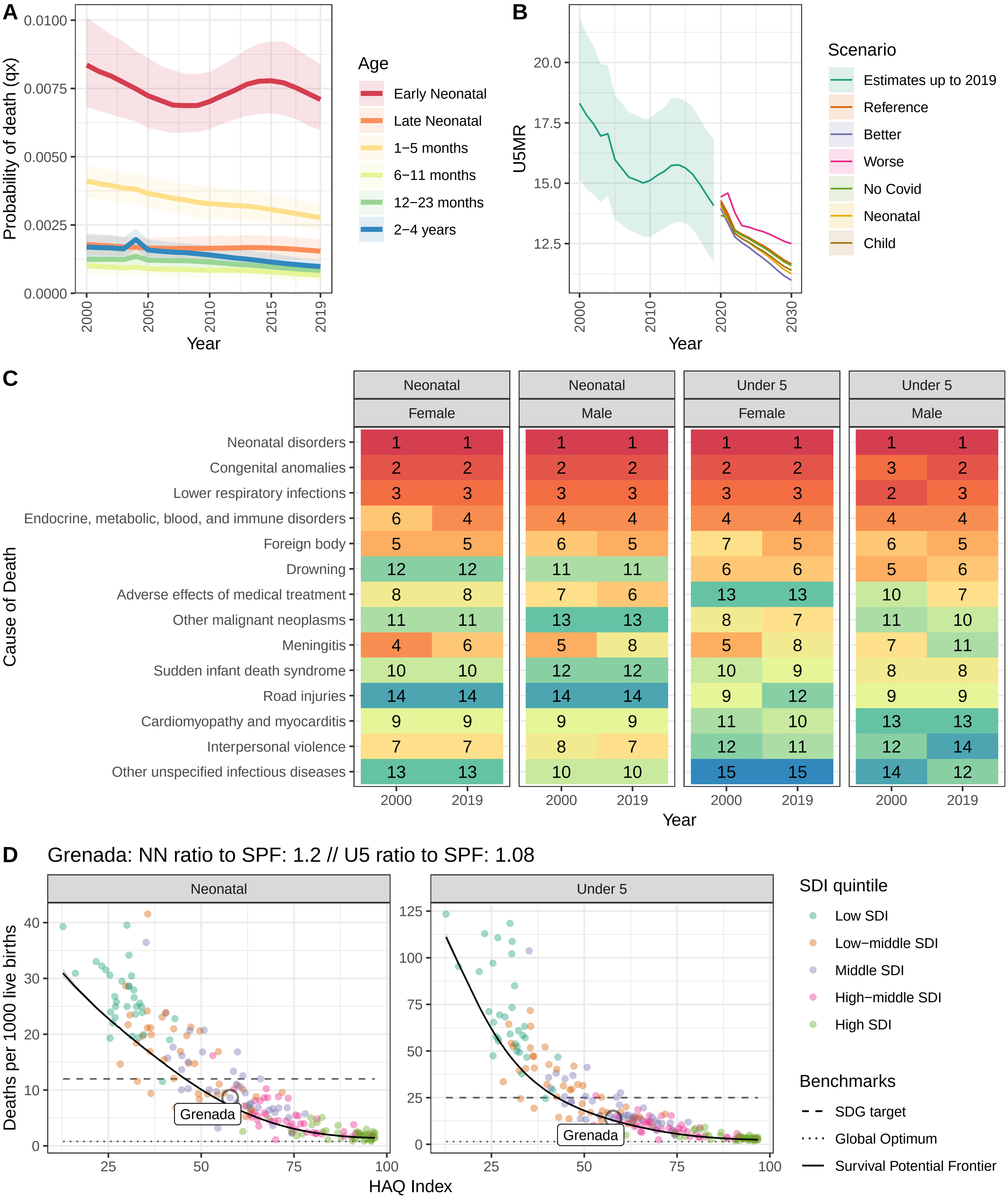

A
Probability of death (qx)
Year
Age
Early Neonatal
Late Neonatal
1−5 months
6−11 months
12−23 months
2−4 years
B
U5MR
Year
Scenario
Estimates up to 2019
Reference
Better
Worse
No Covid
Neonatal
Child
C
Cause of Death
Neonatal
Under 5
Female
Male
Neonatal disorders
Congenital anomalies
Lower respiratory infections
Endocrine, metabolic, blood, and immune disorders
Foreign body
Drowning
Adverse effects of medical treatment
Other malignant neoplasms
Meningitis
Sudden infant death syndrome
Road injuries
Cardiomyopathy and myocarditis
Interpersonal violence
Other unspecified infectious diseases
Year
D
Grenada: NN ratio to SPF: 1.2 // U5 ratio to SPF: 1.08
Neonatal
Under 5
Deaths per 1000 live births
HAQ Index
Grenada
SDI quintile
Low SDI
Low−middle SDI
Middle SDI
High−middle SDI
High SDI
Benchmarks
SDG target
Global Optimum
Survival Potential Frontier

Guyana

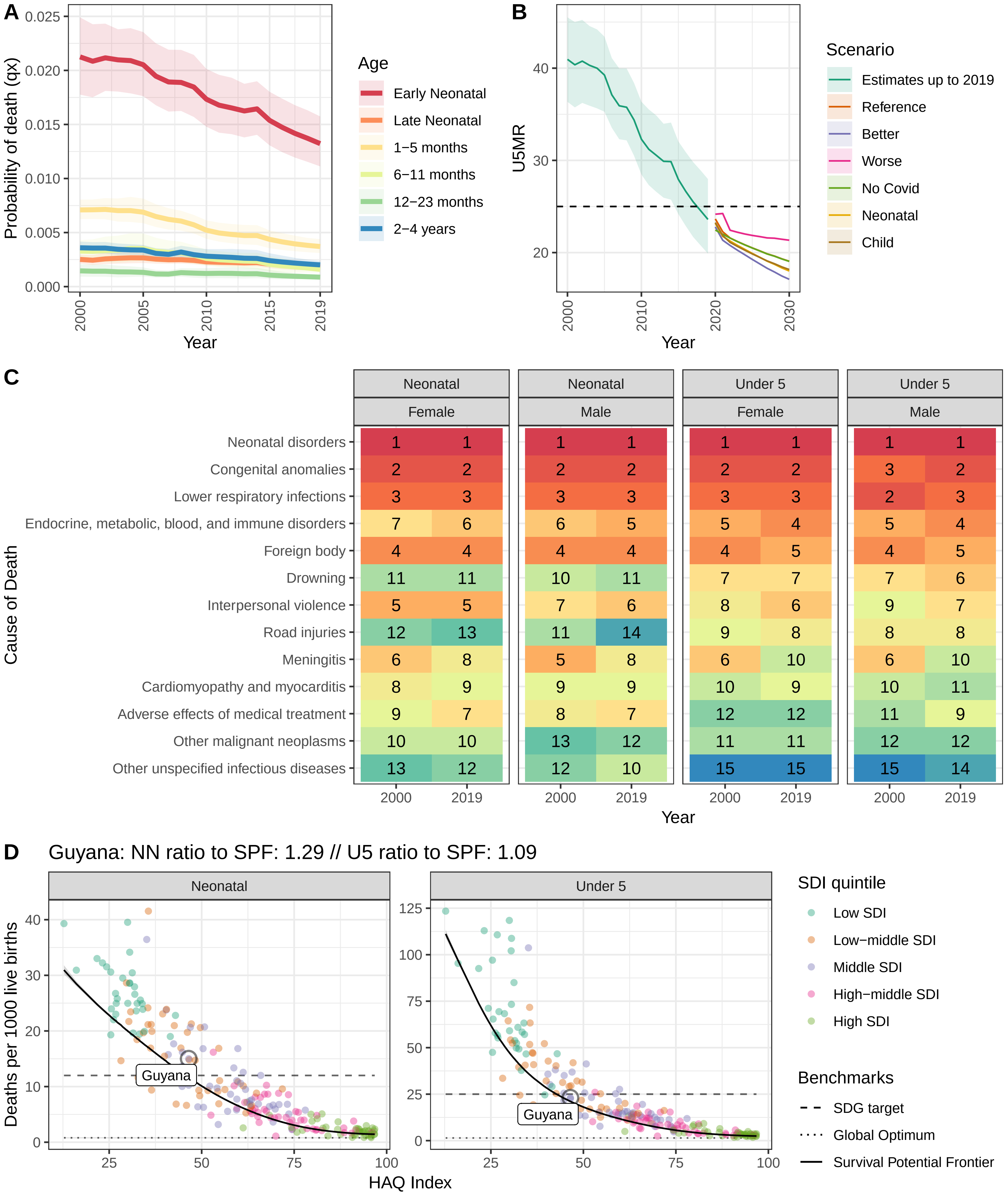

A
Probability of death (qx)
0.025
0.020
0.015
0.010
0.005
0.000
2000
2005
2010
2015
2019
Year
Age
Early Neonatal
Late Neonatal
1–5 months
6–11 months
12–23 months
2–4 years
B
U5MR
40
30
20
2000
2010
2020
2030
Year
Scenario
Estimates up to 2019
Reference
Better
Worse
No Covid
Neonatal
Child
C
Cause of Death
Neonatal Female
Neonatal Male
Under 5 Female
Under 5 Male
Neonatal disorders 1 1 1 1 1 1 1 1
Congenital anomalies 2 2 2 2 2 2 3 2
Lower respiratory infections 3 3 3 3 3 3 2 3
Endocrine, metabolic, blood, and immune disorders 7 6 6 5 5 4 5 4
Foreign body 4 4 4 4 4 5 4 5
Drowning 11 11 10 11 7 7 7 6
Interpersonal violence 5 5 7 6 8 6 9 7
Road injuries 12 13 11 14 9 8 8 8
Meningitis 6 8 5 8 6 10 6 10
Cardiomyopathy and myocarditis 8 9 9 9 10 9 10 11
Adverse effects of medical treatment 9 7 8 7 12 12 11 9
Other malignant neoplasms 10 10 13 12 11 11 12 12
Other unspecified infectious diseases 13 12 12 10 15 15 15 14
2000
2019
Year
D
Guyana: NN ratio to SPF: 1.29 // U5 ratio to SPF: 1.09
Neonatal
Under 5
Deaths per 1000 live births
40
30
20
10
0
125
100
75
50
25
0
Guyana
HAQ Index
25
50
75
100
SDI quintile
Low SDI
Low−middle SDI
Middle SDI
High−middle SDI
High SDI
Benchmarks
SDG target
Global Optimum
Survival Potential Frontier

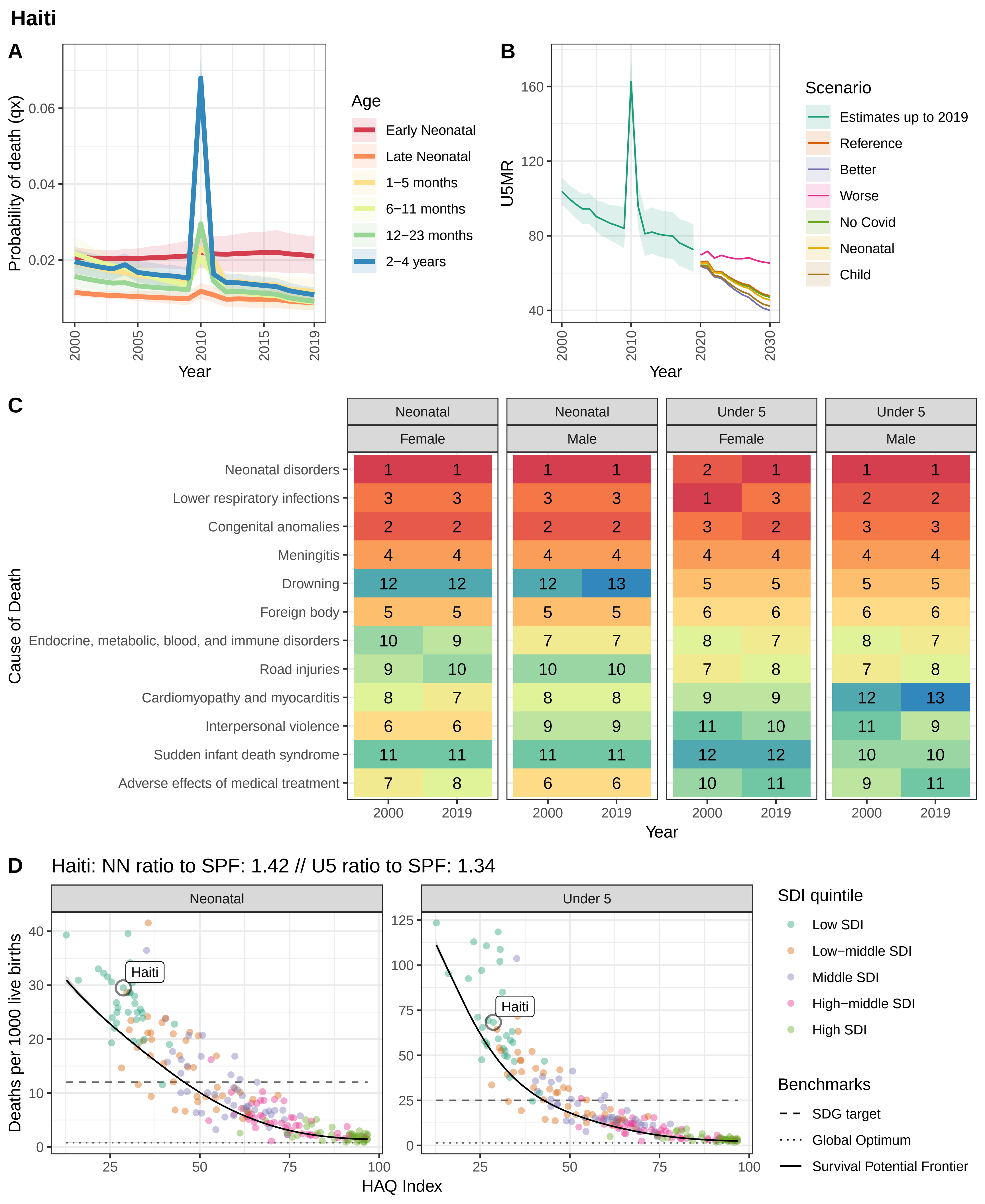
Haiti
A
Probability of death (qx)
0.06
0.04
0.02
2000
2005
2010
2015
2019
Year
Age
Early Neonatal
Late Neonatal
1−5 months
6−11 months
12−23 months
2−4 years
B
U5MR
160
120
80
40
2000
2010
2020
2030
Year
Scenario
Estimates up to 2019
Reference
Better
Worse
No Covid
Neonatal
Child
C
Neonatal
Female
Neonatal
Male
Under 5
Female
Under 5
Male
Cause of Death
Neonatal disorders 1 1 1 1 2 1 1 1
Lower respiratory infections 3 3 3 3 1 3 2 2
Congenital anomalies 2 2 2 2 3 2 3 3
Meningitis 4 4 4 4 4 4 4 4
Drowning 12 12 12 13 5 5 5 5
Foreign body 5 5 5 5 6 6 6 6
Endocrine, metabolic, blood, and immune disorders 10 9 7 7 8 7 8 7
Road injuries 9 10 10 10 7 8 7 8
Cardiomyopathy and myocarditis 8 7 8 8 9 9 12 13
Interpersonal violence 6 6 9 9 11 10 11 9
Sudden infant death syndrome 11 11 11 11 12 12 10 10
Adverse effects of medical treatment 7 8 6 6 10 11 9 11
2000 2019 2000 2019 2000 2019 2000 2019
Year
D
Haiti: NN ratio to SPF: 1.42 // U5 ratio to SPF: 1.34
Neonatal
Under 5
Deaths per 1000 live births
40
30
20
10
0
125
100
75
50
25
0
Haiti
Haiti
25
50
75
100
HAQ Index
SDI quintile
Low SDI
Low−middle SDI
Middle SDI
High−middle SDI
High SDI
Benchmarks
SDG target
Global Optimum
Survival Potential Frontier

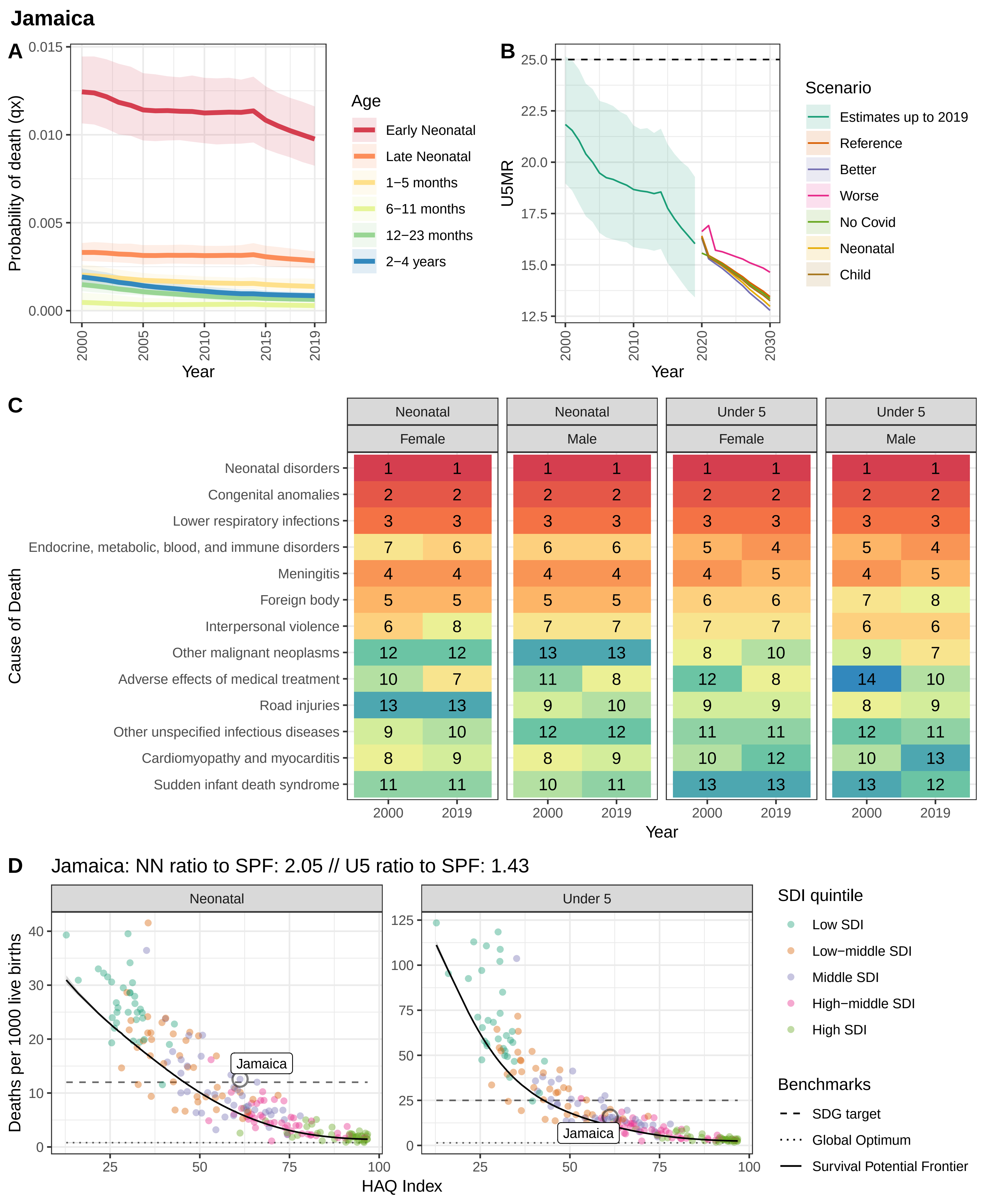
Jamaica
A
Probability of death (qx)
0.015
0.010
0.005
0.000
2000
2005
2010
2015
2019
Year
Age
Early Neonatal
Late Neonatal
1−5 months
6−11 months
12−23 months
2−4 years
B
U5MR
25.0
22.5
20.0
17.5
15.0
12.5
2000
2010
2020
2030
Year
Scenario
Estimates up to 2019
Reference
Better
Worse
No Covid
Neonatal
Child
C
Cause of Death
Neonatal Female
Neonatal Male
Under 5 Female
Under 5 Male
Neonatal disorders 1 1 1 1 1 1 1 1
Congenital anomalies 2 2 2 2 2 2 2 2
Lower respiratory infections 3 3 3 3 3 3 3 3
Endocrine, metabolic, blood, and immune disorders 7 6 6 6 5 4 5 4
Meningitis 4 4 4 4 4 5 4 5
Foreign body 5 5 5 5 6 6 7 8
Interpersonal violence 6 8 7 7 7 7 6 6
Other malignant neoplasms 12 12 13 13 8 10 9 7
Adverse effects of medical treatment 10 7 11 8 12 8 14 10
Road injuries 13 13 9 10 9 9 8 9
Other unspecified infectious diseases 9 10 12 12 11 11 12 11
Cardiomyopathy and myocarditis 8 9 8 9 10 12 10 13
Sudden infant death syndrome 11 11 10 11 13 13 13 12
2000 2019 2000 2019 2000 2019 2000 2019
Year
D
Jamaica: NN ratio to SPF: 2.05 // U5 ratio to SPF: 1.43
Neonatal
Under 5
Deaths per 1000 live births
40
30
20
10
0
125
100
75
50
25
0
Jamaica
25
50
75
100
HAQ Index
SDI quintile
Low SDI
Low−middle SDI
Middle SDI
High−middle SDI
High SDI
Benchmarks
SDG target
Global Optimum
Survival Potential Frontier

Saint Lucia

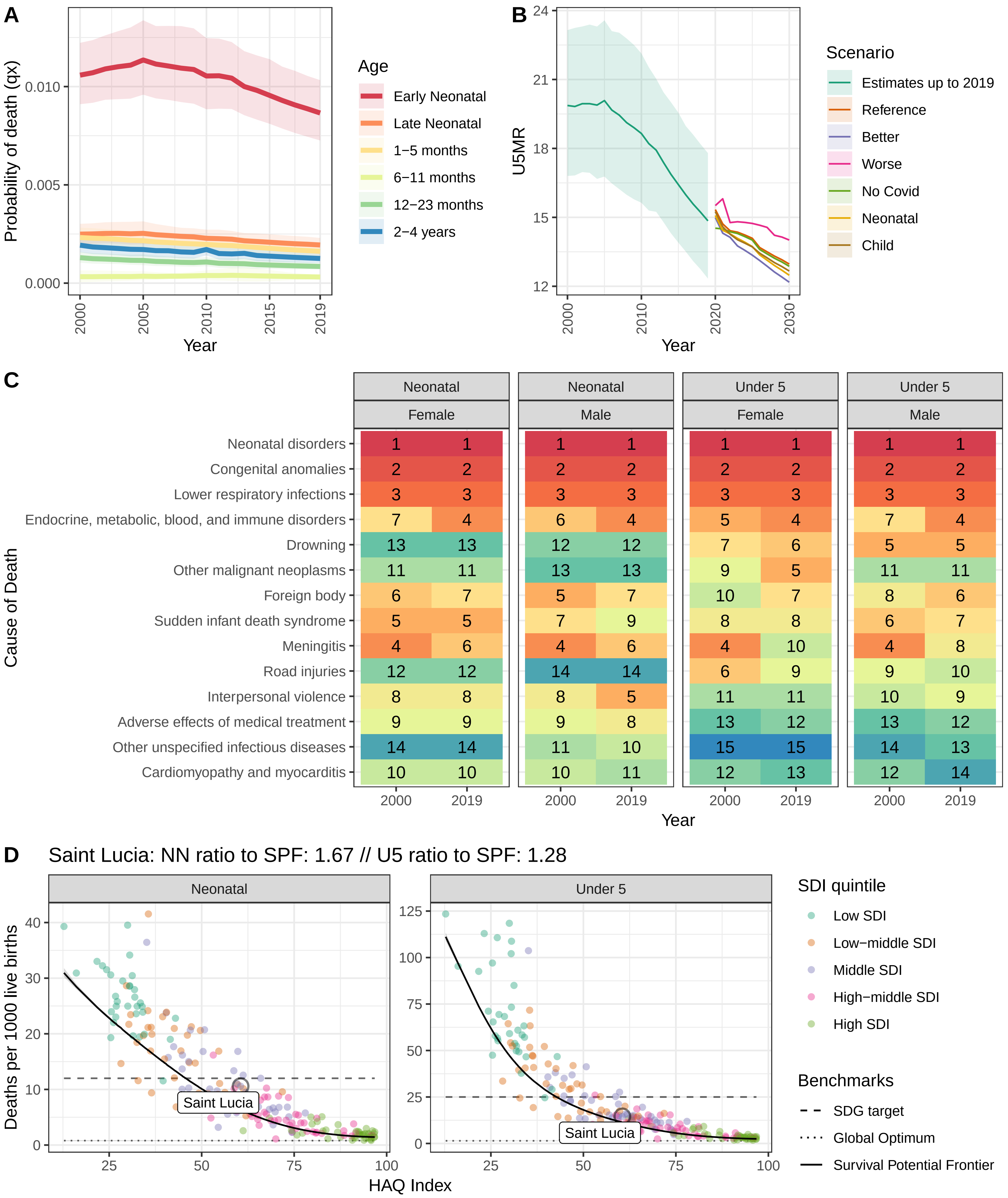

A
Probability of death (qx)
Year
Age
Early Neonatal
Late Neonatal
1−5 months
6−11 months
12−23 months
2−4 years
B
U5MR
Year
Scenario
Estimates up to 2019
Reference
Better
Worse
No Covid
Neonatal
Child
C
Neonatal Female
Neonatal Male
Under 5 Female
Under 5 Male
Cause of Death
Neonatal disorders
Congenital anomalies
Lower respiratory infections
Endocrine, metabolic, blood, and immune disorders
Drowning
Other malignant neoplasms
Foreign body
Sudden infant death syndrome
Meningitis
Road injuries
Interpersonal violence
Adverse effects of medical treatment
Other unspecified infectious diseases
Cardiomyopathy and myocarditis
Year
D
Saint Lucia: NN ratio to SPF: 1.67 // U5 ratio to SPF: 1.28
Neonatal
Under 5
Deaths per 1000 live births
HAQ Index
Saint Lucia
SDI quintile
Low SDI
Low−middle SDI
Middle SDI
High−middle SDI
High SDI
Benchmarks
SDG target
Global Optimum
Survival Potential Frontier

Saint Vincent and the Grenadines

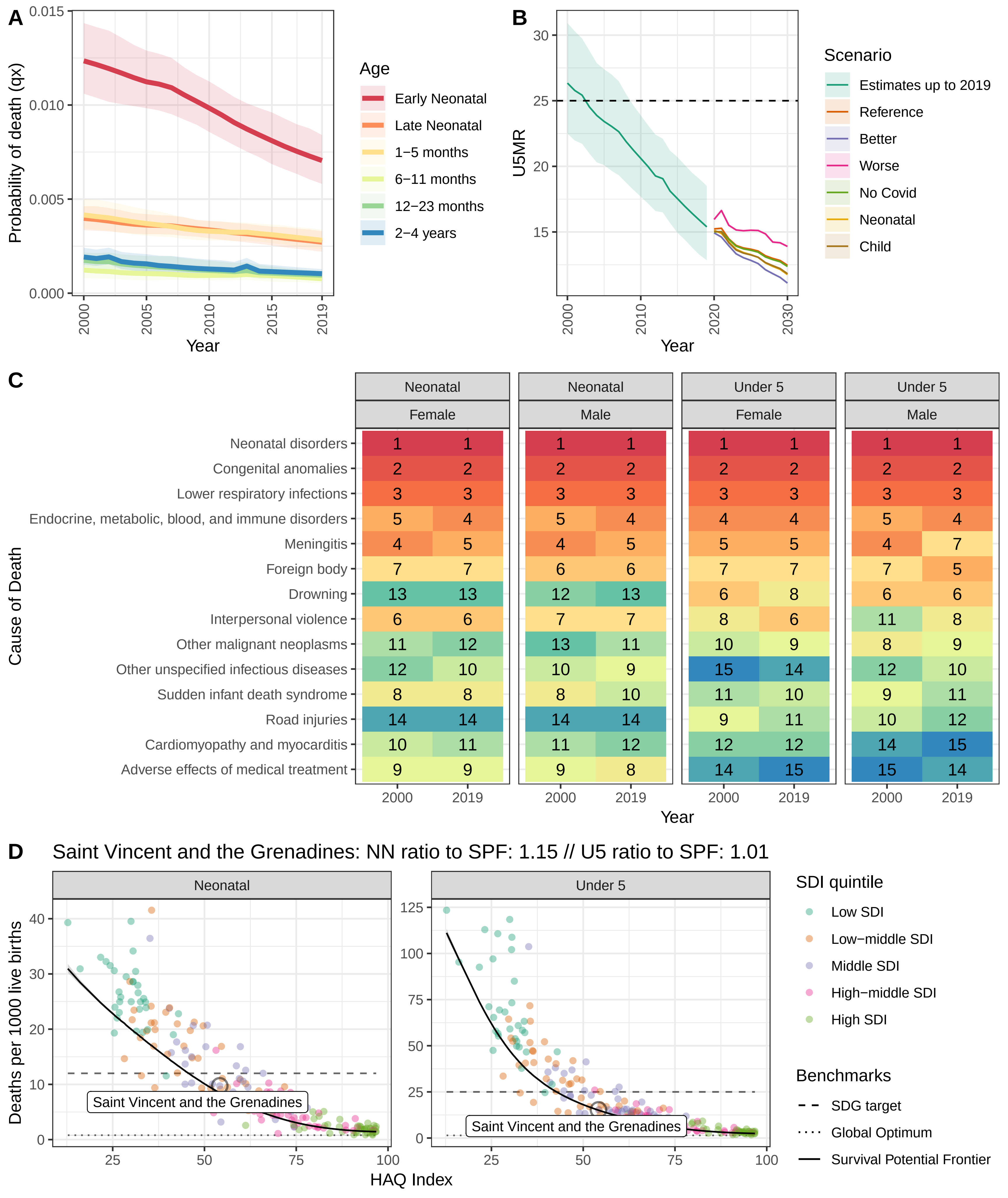

Suriname

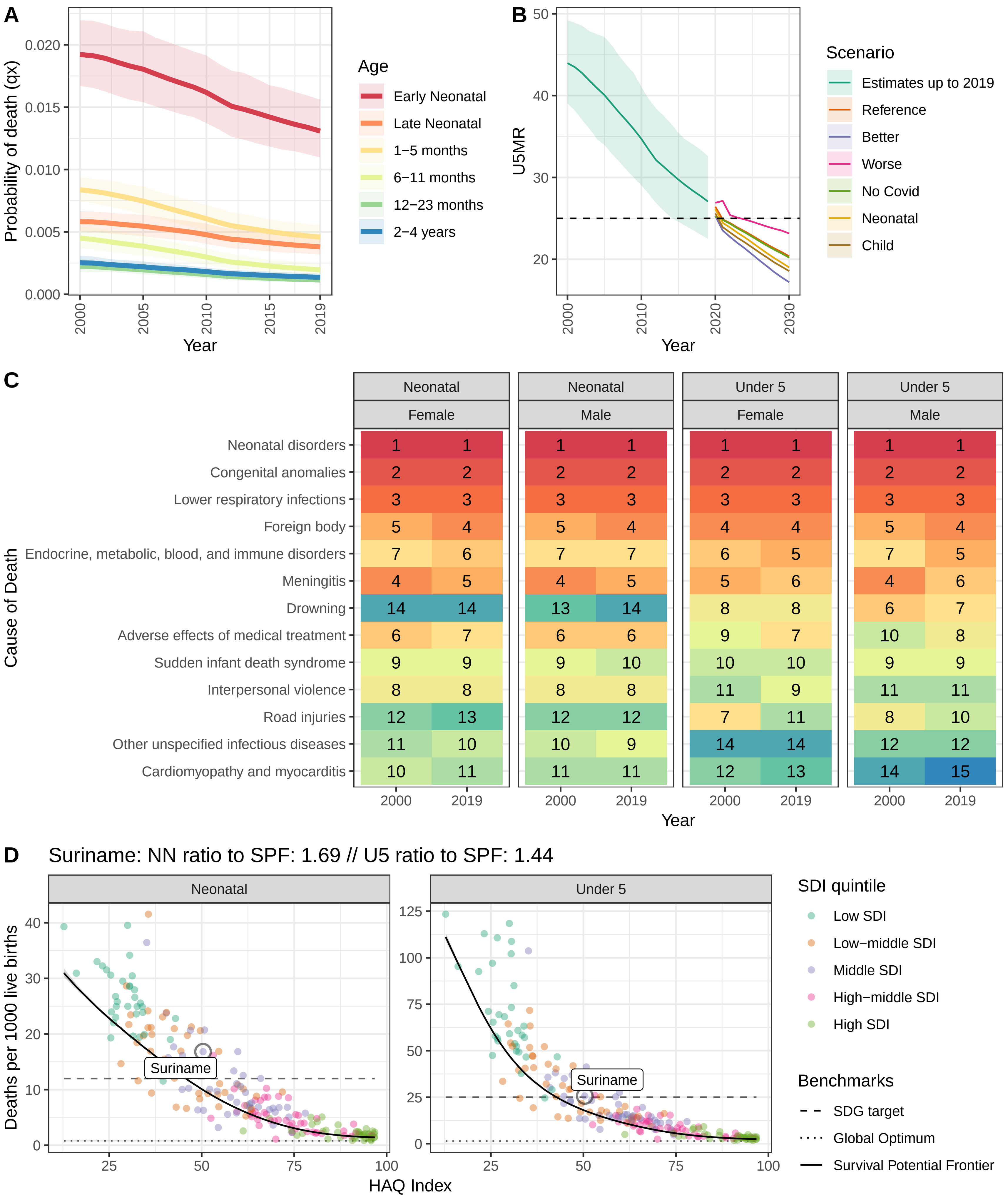
A
Probability of death (qx)
0.020
0.015
0.010
0.005
0.000
2000
2005
2010
2015
2019
Year
Age
Early Neonatal
Late Neonatal
1−5 months
6−11 months
12−23 months
2−4 years
B
U5MR
50
40
30
20
2000
2010
2020
2030
Year
Scenario
Estimates up to 2019
Reference
Better
Worse
No Covid
Neonatal
Child
C
Neonatal
Female
Neonatal
Male
Under 5
Female
Under 5
Male
Cause of Death
Neonatal disorders 1 1 1 1 1 1 1 1
Congenital anomalies 2 2 2 2 2 2 2 2
Lower respiratory infections 3 3 3 3 3 3 3 3
Foreign body 5 4 5 4 4 4 5 4
Endocrine, metabolic, blood, and immune disorders 7 6 7 7 6 5 7 5
Meningitis 4 5 4 5 5 6 4 6
Drowning 14 14 13 14 8 8 6 7
Adverse effects of medical treatment 6 7 6 6 9 7 10 8
Sudden infant death syndrome 9 9 9 10 10 10 9 9
Interpersonal violence 8 8 8 8 11 9 11 11
Road injuries 12 13 12 12 7 11 8 10
Other unspecified infectious diseases 11 10 10 9 14 14 12 12
Cardiomyopathy and myocarditis 10 11 11 11 12 13 14 15
2000
2019
Year
D
Suriname: NN ratio to SPF: 1.69 // U5 ratio to SPF: 1.44
Neonatal
Under 5
Deaths per 1000 live births
40
30
20
10
0
125
100
75
50
25
0
Suriname
25
50
75
100
HAQ Index
SDI quintile
Low SDI
Low−middle SDI
Middle SDI
High−middle SDI
High SDI
Benchmarks
SDG target
Global Optimum
Survival Potential Frontier

Trinidad and Tobago

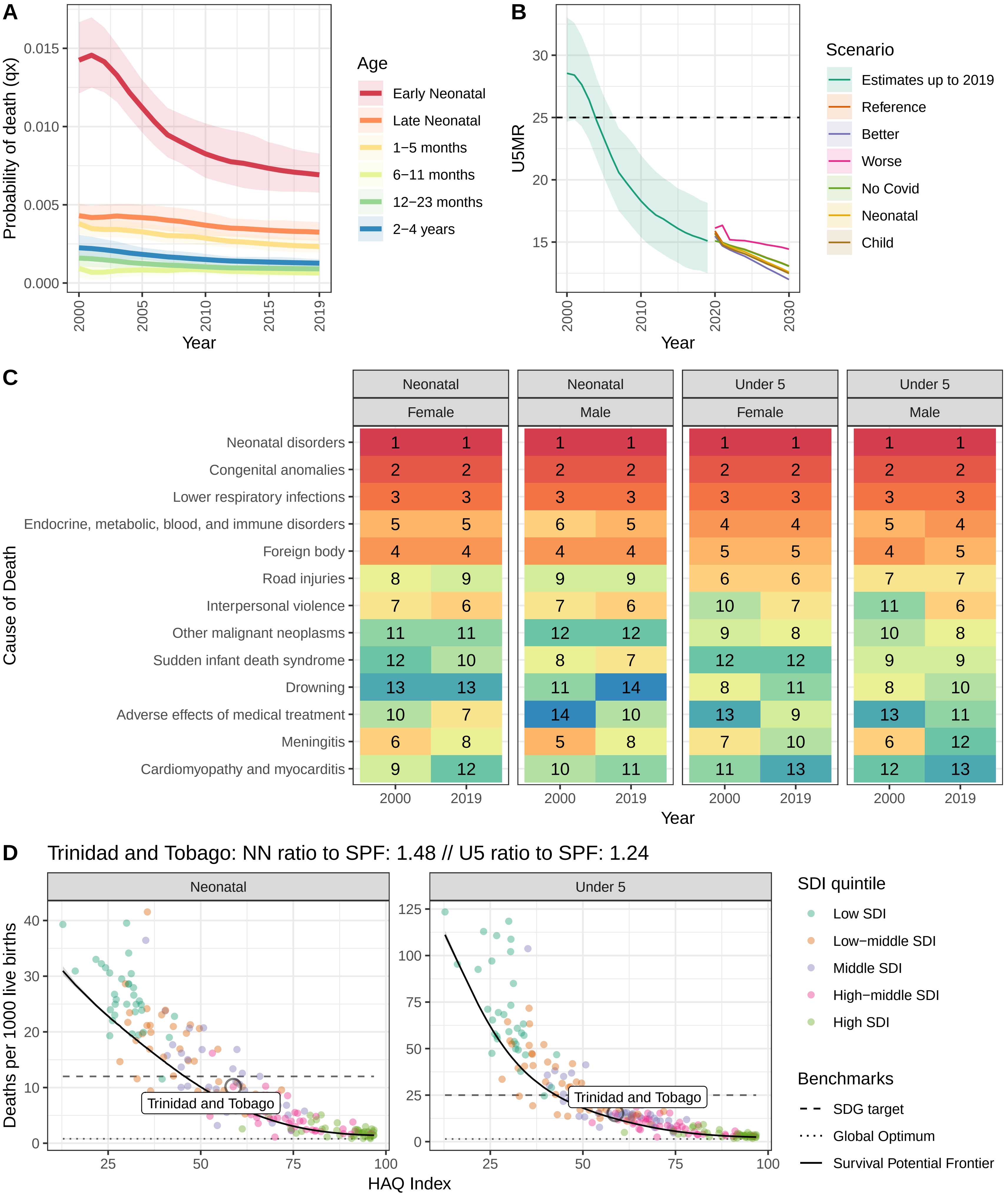

Lao People's Democratic Republic

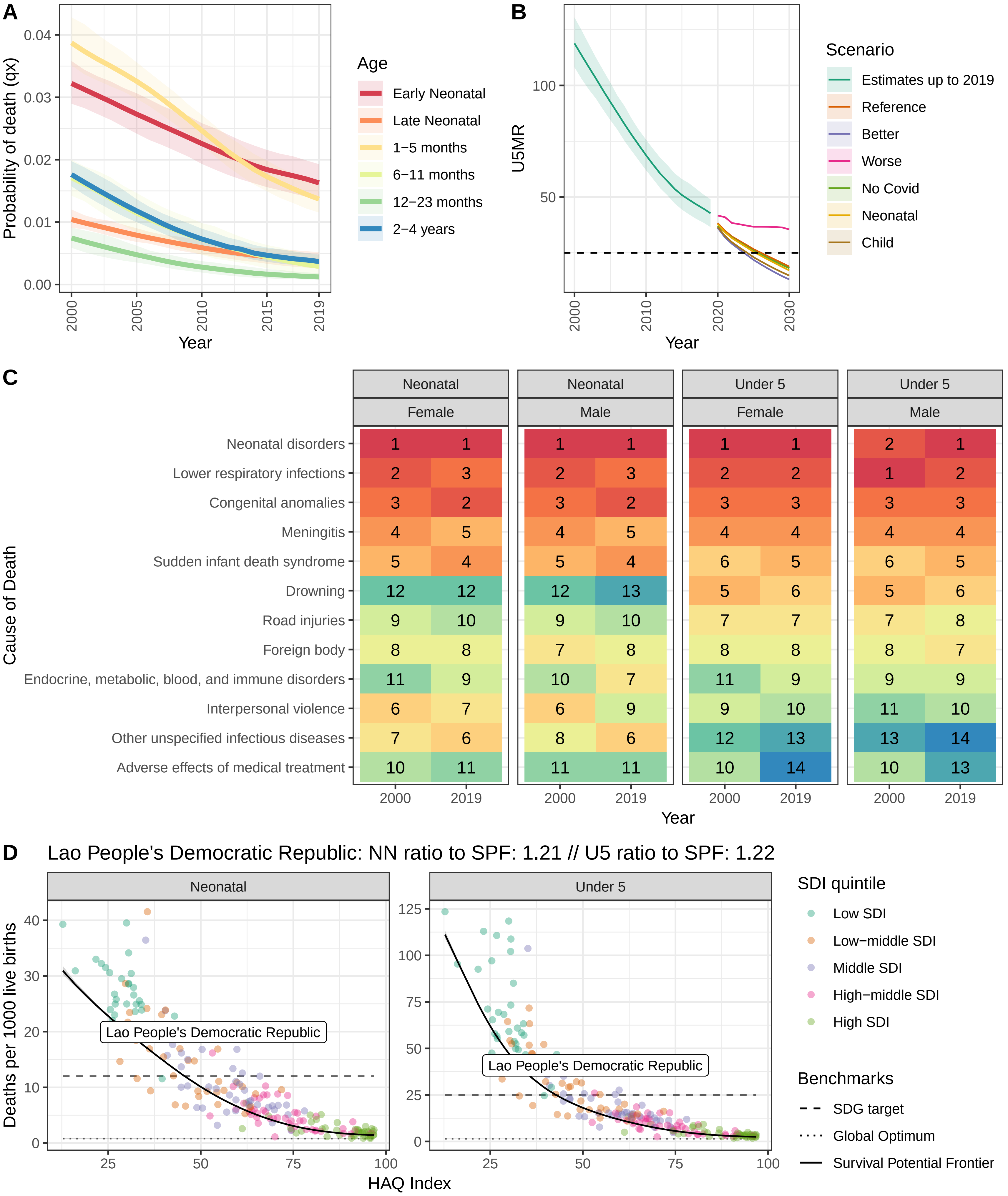

Bolivia (Plurinational State of)

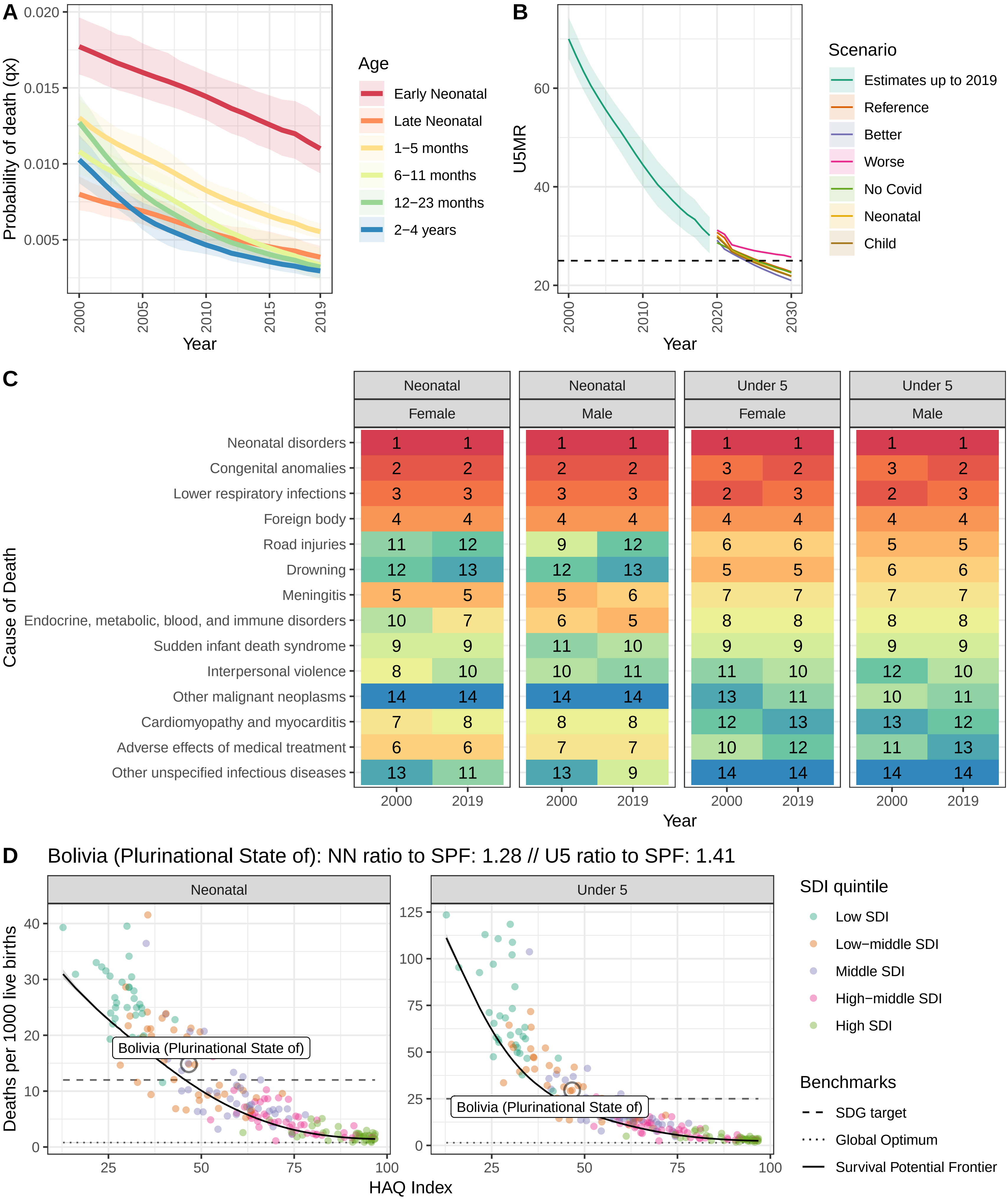

Ecuador

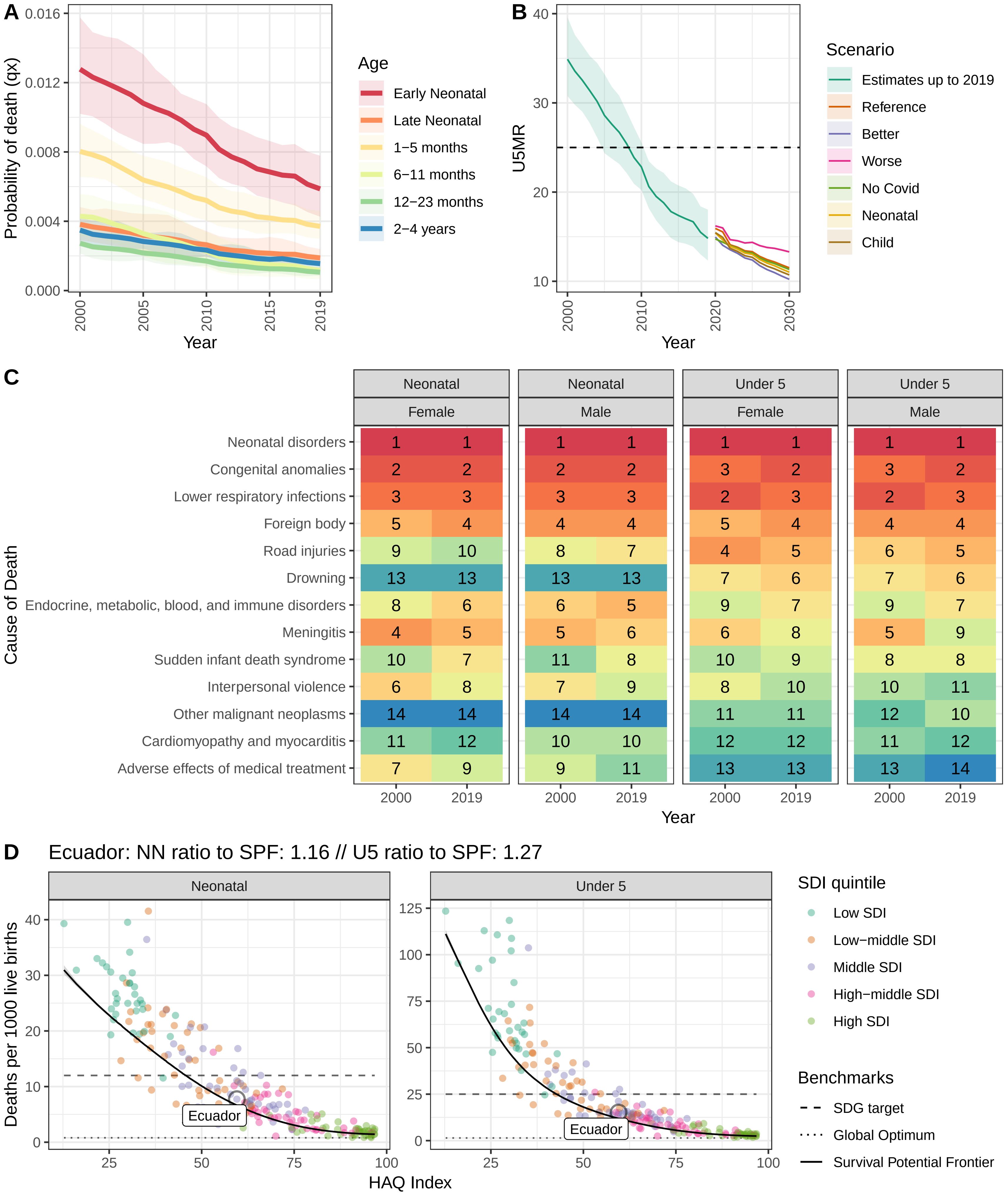
A
Probability of death (qx)
0.016
0.012
0.008
0.004
0.000
2000
2005
2010
2015
2019
Year
Age
Early Neonatal
Late Neonatal
1−5 months
6−11 months
12−23 months
2−4 years
B
U5MR
40
30
20
10
2000
2010
2020
2030
Year
Scenario
Estimates up to 2019
Reference
Better
Worse
No Covid
Neonatal
Child
C
Cause of Death
Neonatal Female
Neonatal Male
Under 5 Female
Under 5 Male
Neonatal disorders 1 1 | 1 1 | 1 1 | 1 1
Congenital anomalies 2 2 | 2 2 | 3 2 | 3 2
Lower respiratory infections 3 3 | 3 3 | 2 3 | 2 3
Foreign body 5 4 | 4 4 | 5 4 | 4 4
Road injuries 9 10 | 8 7 | 4 5 | 6 5
Drowning 13 13 | 13 13 | 7 6 | 7 6
Endocrine, metabolic, blood, and immune disorders 8 6 | 6 5 | 9 7 | 9 7
Meningitis 4 5 | 5 6 | 6 8 | 5 9
Sudden infant death syndrome 10 7 | 11 8 | 10 9 | 8 8
Interpersonal violence 6 8 | 7 9 | 8 10 | 10 11
Other malignant neoplasms 14 14 | 14 14 | 11 11 | 12 10
Cardiomyopathy and myocarditis 11 12 | 10 10 | 12 12 | 11 12
Adverse effects of medical treatment 7 9 | 9 11 | 13 13 | 13 14
2000 2019
Year
D
Ecuador: NN ratio to SPF: 1.16 // U5 ratio to SPF: 1.27
Neonatal
Under 5
Deaths per 1000 live births
40
30
20
10
0
125
100
75
50
25
0
Ecuador
25
50
75
100
HAQ Index
SDI quintile
Low SDI
Low−middle SDI
Middle SDI
High−middle SDI
High SDI
Benchmarks
SDG target
Global Optimum
Survival Potential Frontier

Peru

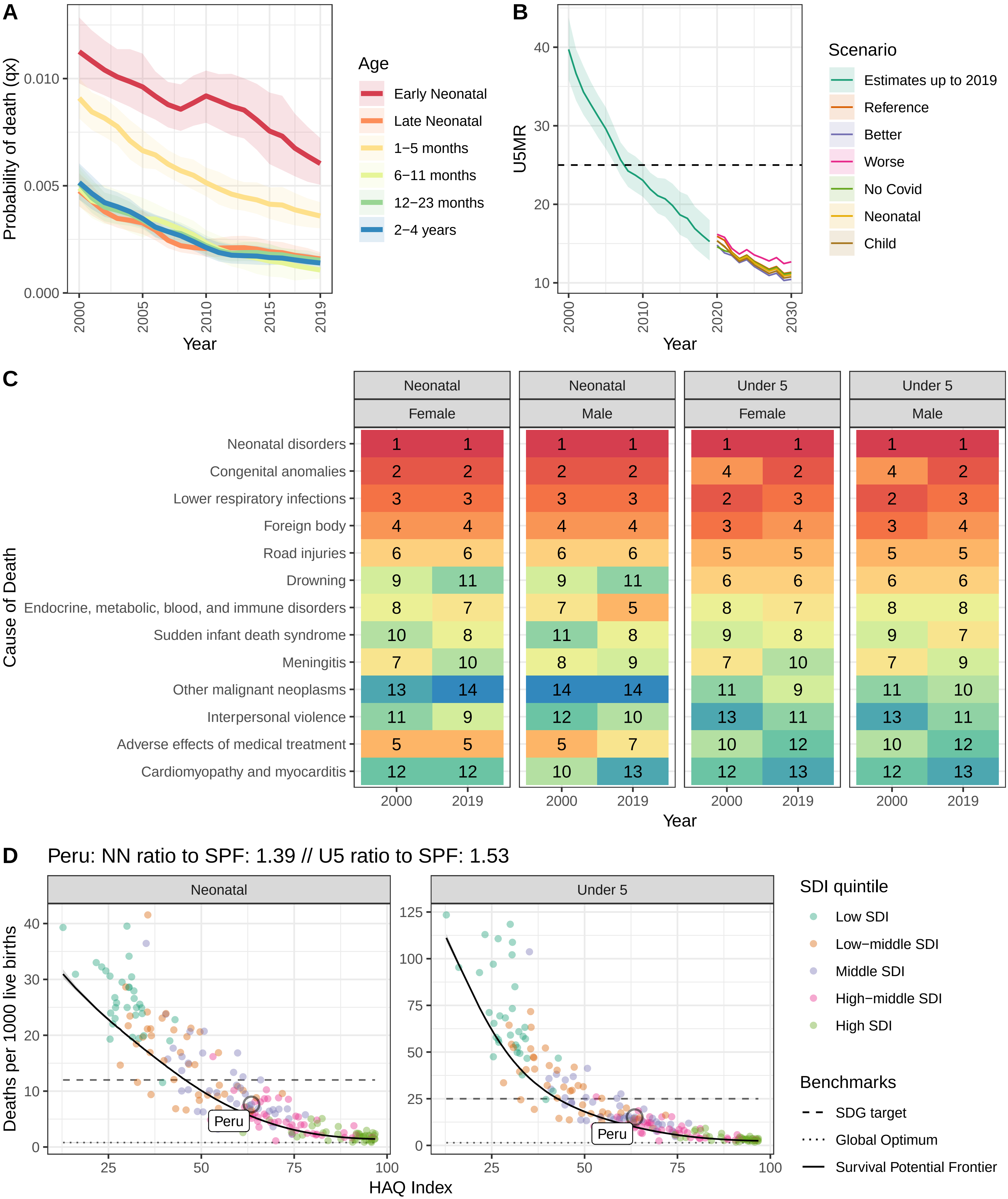
A
Probability of death (qx)
0.010
0.005
0.000
2000
2005
2010
2015
2019
Year
Age
Early Neonatal
Late Neonatal
1−5 months
6−11 months
12−23 months
2−4 years
B
U5MR
40
30
20
10
2000
2010
2020
2030
Year
Scenario
Estimates up to 2019
Reference
Better
Worse
No Covid
Neonatal
Child
C
Neonatal
Female
Neonatal
Male
Under 5
Female
Under 5
Male
Cause of Death
Neonatal disorders 1 1 1 1 1 1 1 1
Congenital anomalies 2 2 2 2 4 2 4 2
Lower respiratory infections 3 3 3 3 2 3 2 3
Foreign body 4 4 4 4 3 4 3 4
Road injuries 6 6 6 6 5 5 5 5
Drowning 9 11 9 11 6 6 6 6
Endocrine, metabolic, blood, and immune disorders 8 7 7 5 8 7 8 8
Sudden infant death syndrome 10 8 11 8 9 8 9 7
Meningitis 7 10 8 9 7 10 7 9
Other malignant neoplasms 13 14 14 14 11 9 11 10
Interpersonal violence 11 9 12 10 13 11 13 11
Adverse effects of medical treatment 5 5 5 7 10 12 10 12
Cardiomyopathy and myocarditis 12 12 10 13 12 13 12 13
2000 2019 2000 2019 2000 2019 2000 2019
Year
D
Peru: NN ratio to SPF: 1.39 // U5 ratio to SPF: 1.53
Neonatal
Under 5
Deaths per 1000 live births
40
30
20
10
0
125
100
75
50
25
0
25
50
75
100
Peru
Peru
HAQ Index
SDI quintile
Low SDI
Low−middle SDI
Middle SDI
High−middle SDI
High SDI
Benchmarks
SDG target
Global Optimum
Survival Potential Frontier

Colombia

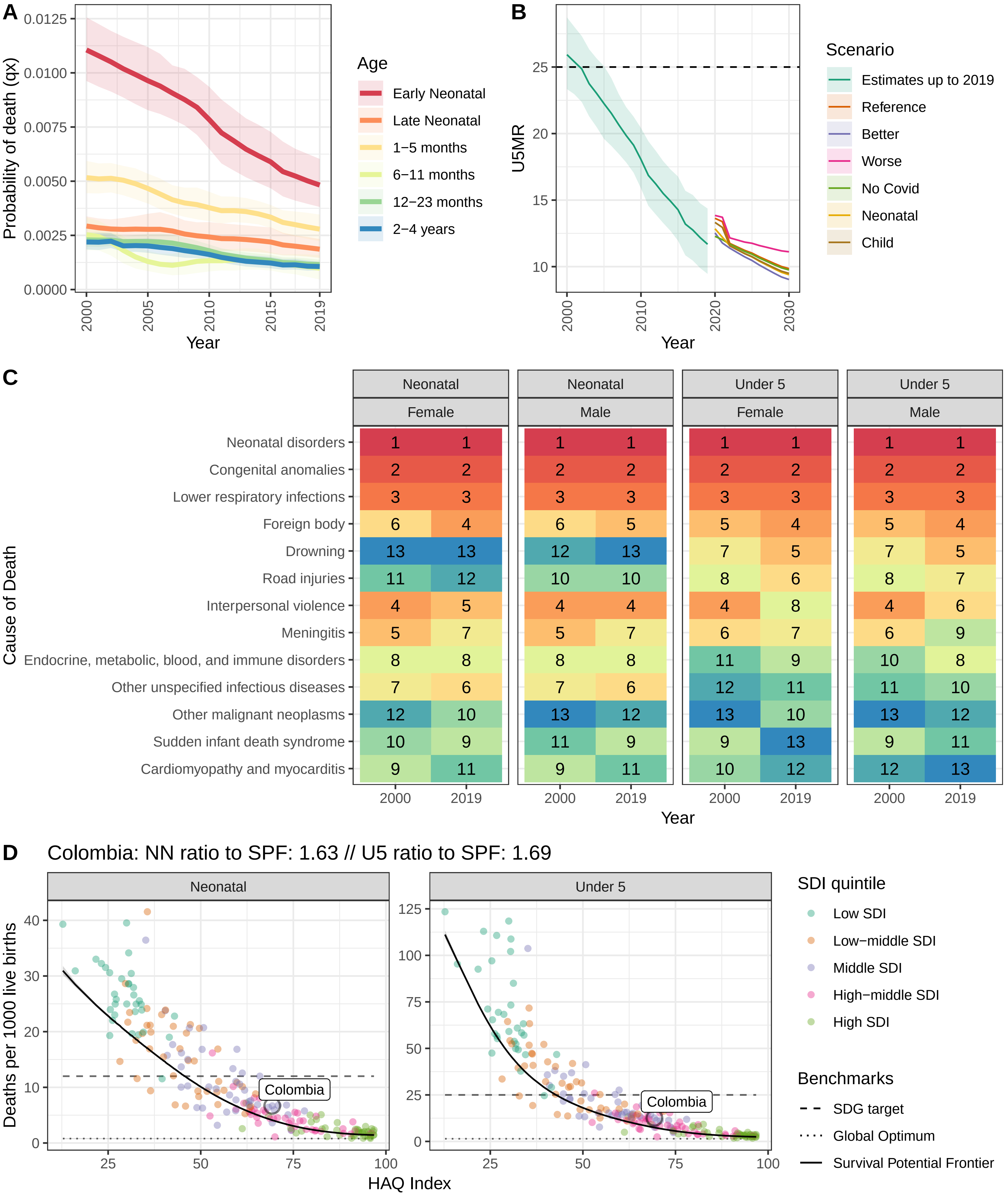

A
Probability of death (qx)
0.0125
0.0100
0.0075
0.0050
0.0025
0.0000
2000
2005
2010
2015
2019
Year
Age
Early Neonatal
Late Neonatal
1−5 months
6−11 months
12−23 months
2−4 years
B
U5MR
25
20
15
10
2000
2010
2020
2030
Year
Scenario
Estimates up to 2019
Reference
Better
Worse
No Covid
Neonatal
Child
C
Cause of Death
Neonatal Female
Neonatal Male
Under 5 Female
Under 5 Male
Neonatal disorders: 1 1 | 1 1 | 1 1 | 1 1
Congenital anomalies: 2 2 | 2 2 | 2 2 | 2 2
Lower respiratory infections: 3 3 | 3 3 | 3 3 | 3 3
Foreign body: 6 4 | 6 5 | 5 4 | 5 4
Drowning: 13 13 | 12 13 | 7 5 | 7 5
Road injuries: 11 12 | 10 10 | 8 6 | 8 7
Interpersonal violence: 4 5 | 4 4 | 4 8 | 4 6
Meningitis: 5 7 | 5 7 | 6 7 | 6 9
Endocrine, metabolic, blood, and immune disorders: 8 8 | 8 8 | 11 9 | 10 8
Other unspecified infectious diseases: 7 6 | 7 6 | 12 11 | 11 10
Other malignant neoplasms: 12 10 | 13 12 | 13 10 | 13 12
Sudden infant death syndrome: 10 9 | 11 9 | 9 13 | 9 11
Cardiomyopathy and myocarditis: 9 11 | 9 11 | 10 12 | 12 13
2000
2019
Year
D
Colombia: NN ratio to SPF: 1.63 // U5 ratio to SPF: 1.69
Neonatal
Under 5
Deaths per 1000 live births
40
30
20
10
0
125
100
75
50
25
0
Colombia
25
50
75
100
HAQ Index
SDI quintile
Low SDI
Low−middle SDI
Middle SDI
High−middle SDI
High SDI
Benchmarks
SDG target
Global Optimum
Survival Potential Frontier

Costa Rica

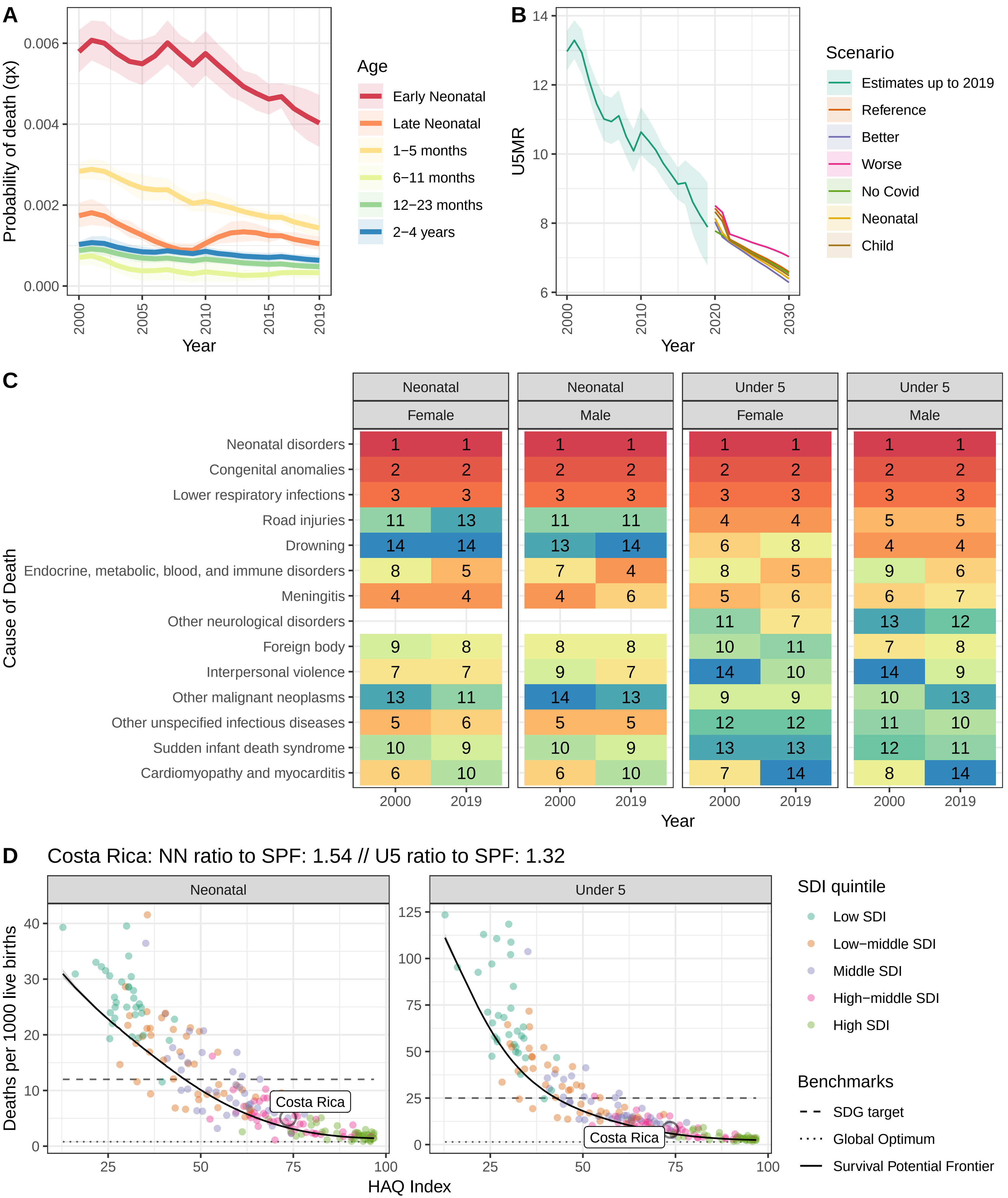
A
Probability of death (qx)
Year
Age
Early Neonatal
Late Neonatal
1−5 months
6−11 months
12−23 months
2−4 years
B
U5MR
Year
Scenario
Estimates up to 2019
Reference
Better
Worse
No Covid
Neonatal
Child
C
Cause of Death
Neonatal Female
Neonatal Male
Under 5 Female
Under 5 Male
Neonatal disorders 1 1 1 1 1 1 1 1
Congenital anomalies 2 2 2 2 2 2 2 2
Lower respiratory infections 3 3 3 3 3 3 3 3
Road injuries 11 13 11 11 4 4 5 5
Drowning 14 14 13 14 6 8 4 4
Endocrine, metabolic, blood, and immune disorders 8 5 7 4 8 5 9 6
Meningitis 4 4 4 6 5 6 6 7
Other neurological disorders 11 7 13 12
Foreign body 9 8 8 8 10 11 7 8
Interpersonal violence 7 7 9 7 14 10 14 9
Other malignant neoplasms 13 11 14 13 9 9 10 13
Other unspecified infectious diseases 5 6 5 5 12 12 11 10
Sudden infant death syndrome 10 9 10 9 13 13 12 11
Cardiomyopathy and myocarditis 6 10 6 10 7 14 8 14
2000 2019
Year
D
Costa Rica: NN ratio to SPF: 1.54 // U5 ratio to SPF: 1.32
Neonatal
Under 5
Deaths per 1000 live births
HAQ Index
Costa Rica
SDI quintile
Low SDI
Low−middle SDI
Middle SDI
High−middle SDI
High SDI
Benchmarks
SDG target
Global Optimum
Survival Potential Frontier

El Salvador

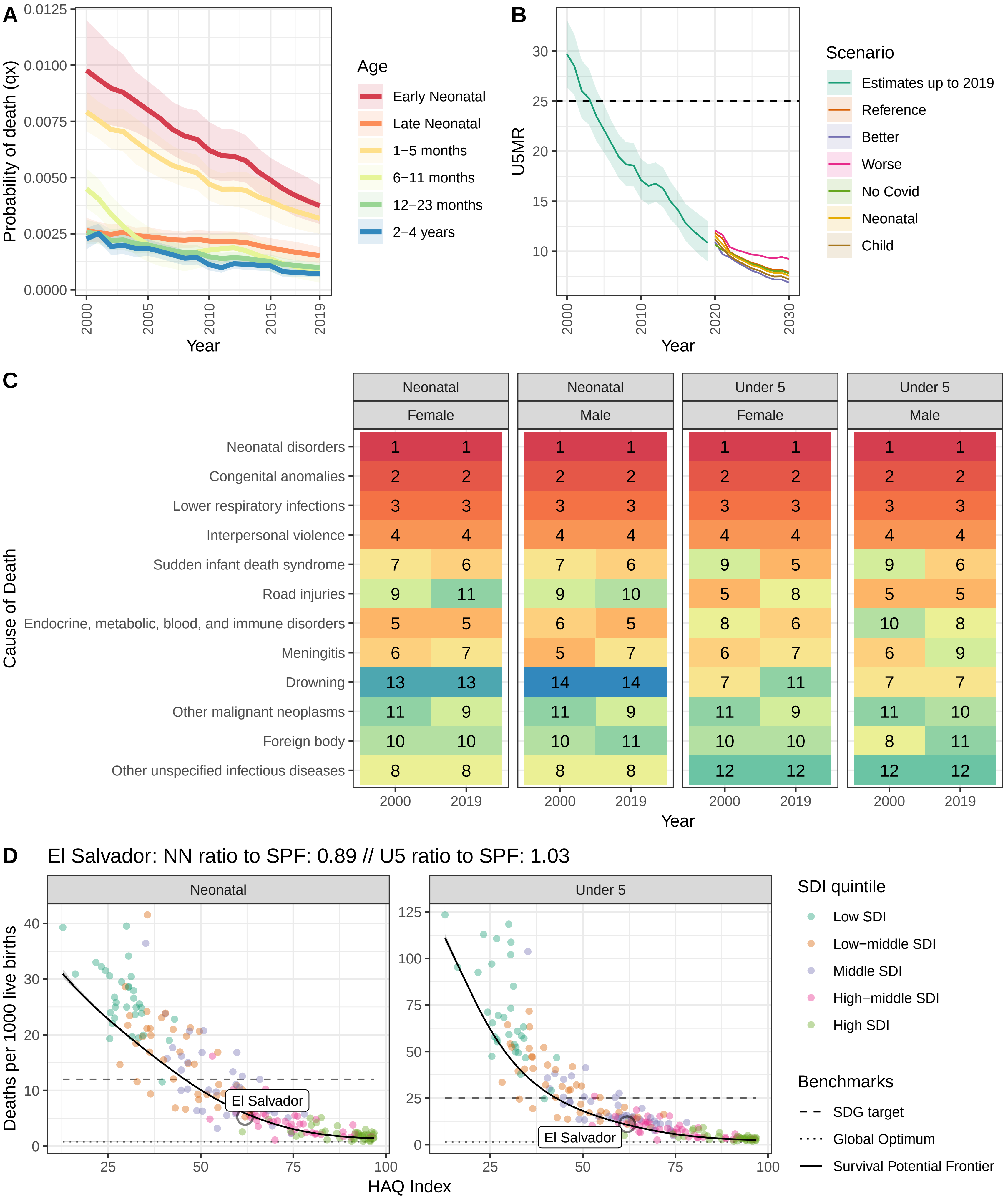
A
Probability of death (qx)
0.0125
0.0100
0.0075
0.0050
0.0025
0.0000
2000
2005
2010
2015
2019
Year
Age
Early Neonatal
Late Neonatal
1−5 months
6−11 months
12−23 months
2−4 years
B
U5MR
30
25
20
15
10
2000
2010
2020
2030
Year
Scenario
Estimates up to 2019
Reference
Better
Worse
No Covid
Neonatal
Child
C
Neonatal
Female
Neonatal
Male
Under 5
Female
Under 5
Male
Cause of Death
Neonatal disorders 1 1 1 1 1 1 1 1
Congenital anomalies 2 2 2 2 2 2 2 2
Lower respiratory infections 3 3 3 3 3 3 3 3
Interpersonal violence 4 4 4 4 4 4 4 4
Sudden infant death syndrome 7 6 7 6 9 5 9 6
Road injuries 9 11 9 10 5 8 5 5
Endocrine, metabolic, blood, and immune disorders 5 5 6 5 8 6 10 8
Meningitis 6 7 5 7 6 7 6 9
Drowning 13 13 14 14 7 11 7 7
Other malignant neoplasms 11 9 11 9 11 9 11 10
Foreign body 10 10 10 11 10 10 8 11
Other unspecified infectious diseases 8 8 8 8 12 12 12 12
2000
2019
Year
D
El Salvador: NN ratio to SPF: 0.89 // U5 ratio to SPF: 1.03
Neonatal
Under 5
Deaths per 1000 live births
40
30
20
10
0
125
100
75
50
25
0
25
50
75
100
El Salvador
HAQ Index
SDI quintile
Low SDI
Low−middle SDI
Middle SDI
High−middle SDI
High SDI
Benchmarks
SDG target
Global Optimum
Survival Potential Frontier

Guatemala

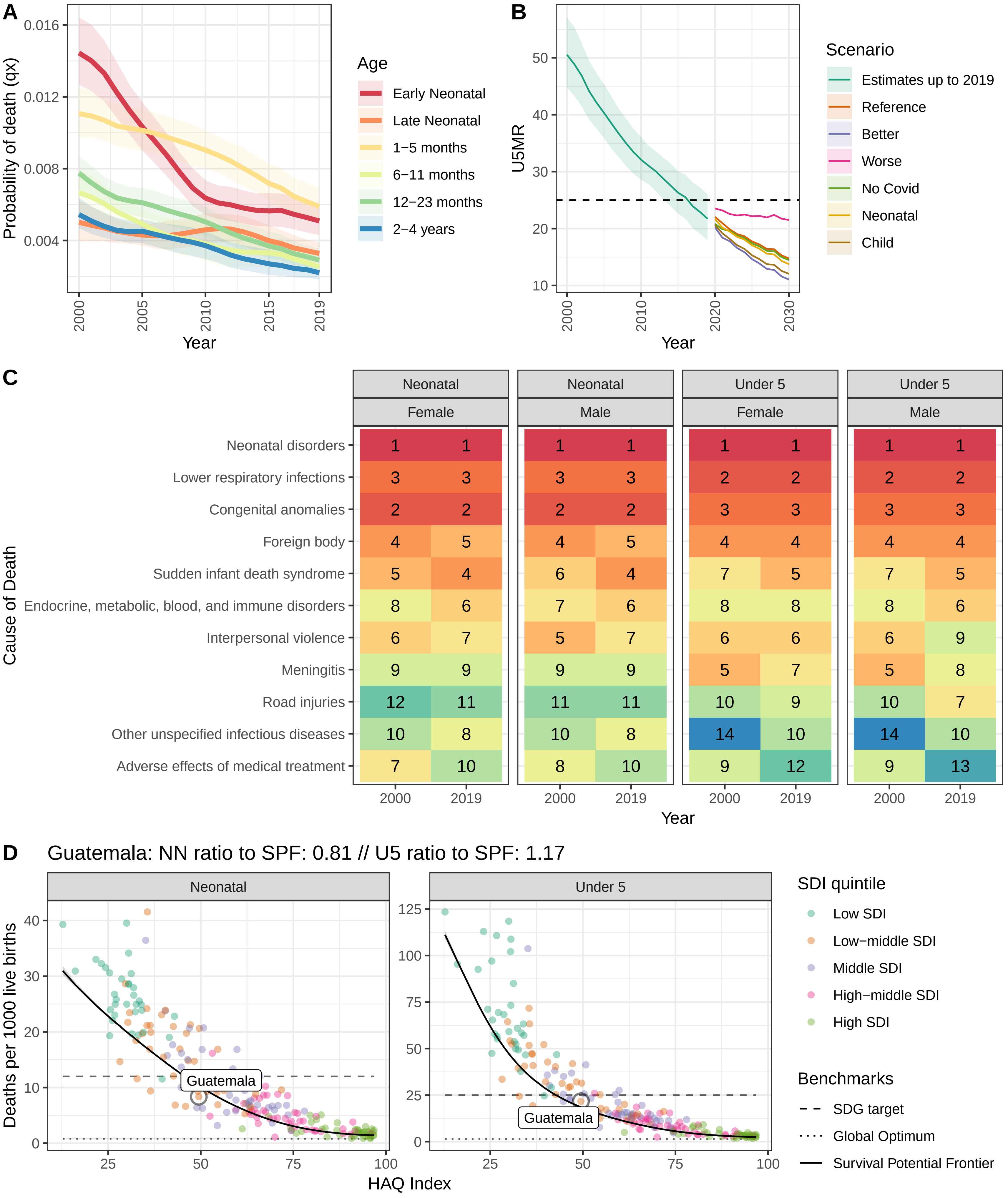
A
Probability of death (qx)
Year
Age
Early Neonatal
Late Neonatal
1−5 months
6−11 months
12−23 months
2−4 years
B
U5MR
Year
Scenario
Estimates up to 2019
Reference
Better
Worse
No Covid
Neonatal
Child
C
Neonatal
Female
Neonatal
Male
Under 5
Female
Under 5
Male
Cause of Death
Neonatal disorders
Lower respiratory infections
Congenital anomalies
Foreign body
Sudden infant death syndrome
Endocrine, metabolic, blood, and immune disorders
Interpersonal violence
Meningitis
Road injuries
Other unspecified infectious diseases
Adverse effects of medical treatment
Year
D
Guatemala: NN ratio to SPF: 0.81 // U5 ratio to SPF: 1.17
Neonatal
Under 5
Deaths per 1000 live births
HAQ Index
Guatemala
SDI quintile
Low SDI
Low−middle SDI
Middle SDI
High−middle SDI
High SDI
Benchmarks
SDG target
Global Optimum
Survival Potential Frontier

Honduras

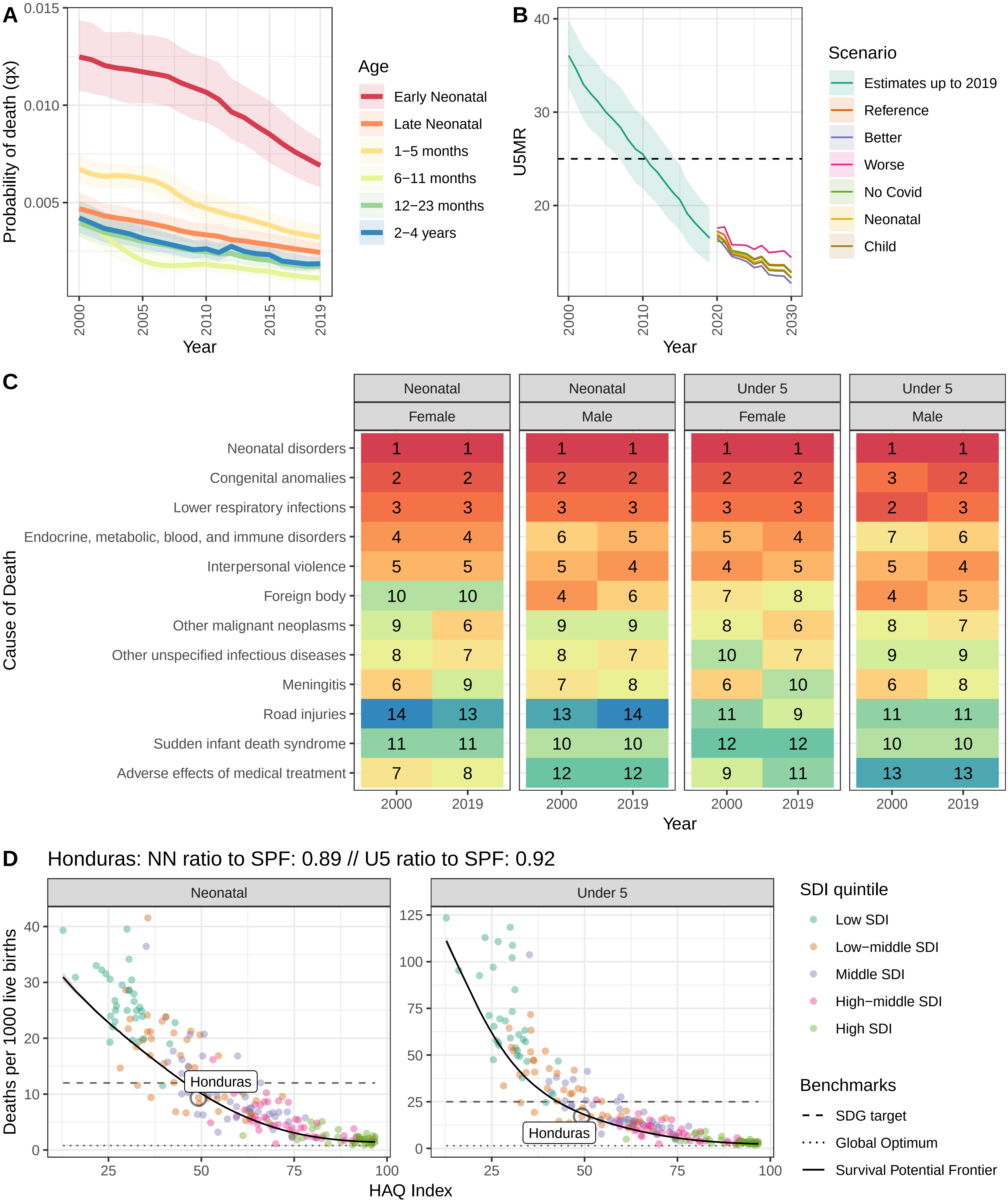
A
Probability of death (qx)
0.015
0.010
0.005
2000
2005
2010
2015
2019
Year
Age
Early Neonatal
Late Neonatal
1−5 months
6−11 months
12−23 months
2−4 years
B
U5MR
40
30
20
2000
2010
2020
2030
Year
Scenario
Estimates up to 2019
Reference
Better
Worse
No Covid
Neonatal
Child
C
Cause of Death
Neonatal
Female
Neonatal
Male
Under 5
Female
Under 5
Male
Neonatal disorders 1 1 1 1 1 1 1 1
Congenital anomalies 2 2 2 2 2 2 3 2
Lower respiratory infections 3 3 3 3 3 3 2 3
Endocrine, metabolic, blood, and immune disorders 4 4 6 5 5 4 7 6
Interpersonal violence 5 5 5 4 4 5 5 4
Foreign body 10 10 4 6 7 8 4 5
Other malignant neoplasms 9 6 9 9 8 6 8 7
Other unspecified infectious diseases 8 7 8 7 10 7 9 9
Meningitis 6 9 7 8 6 10 6 8
Road injuries 14 13 13 14 11 9 11 11
Sudden infant death syndrome 11 11 10 10 12 12 10 10
Adverse effects of medical treatment 7 8 12 12 9 11 13 13
2000
2019
2000
2019
2000
2019
2000
2019
Year
D
Honduras: NN ratio to SPF: 0.89 // U5 ratio to SPF: 0.92
Neonatal
Under 5
Deaths per 1000 live births
40
30
20
10
0
125
100
75
50
25
0
Honduras
Honduras
25
50
75
100
25
50
75
100
HAQ Index
SDI quintile
Low SDI
Low−middle SDI
Middle SDI
High−middle SDI
High SDI
Benchmarks
SDG target
Global Optimum
Survival Potential Frontier

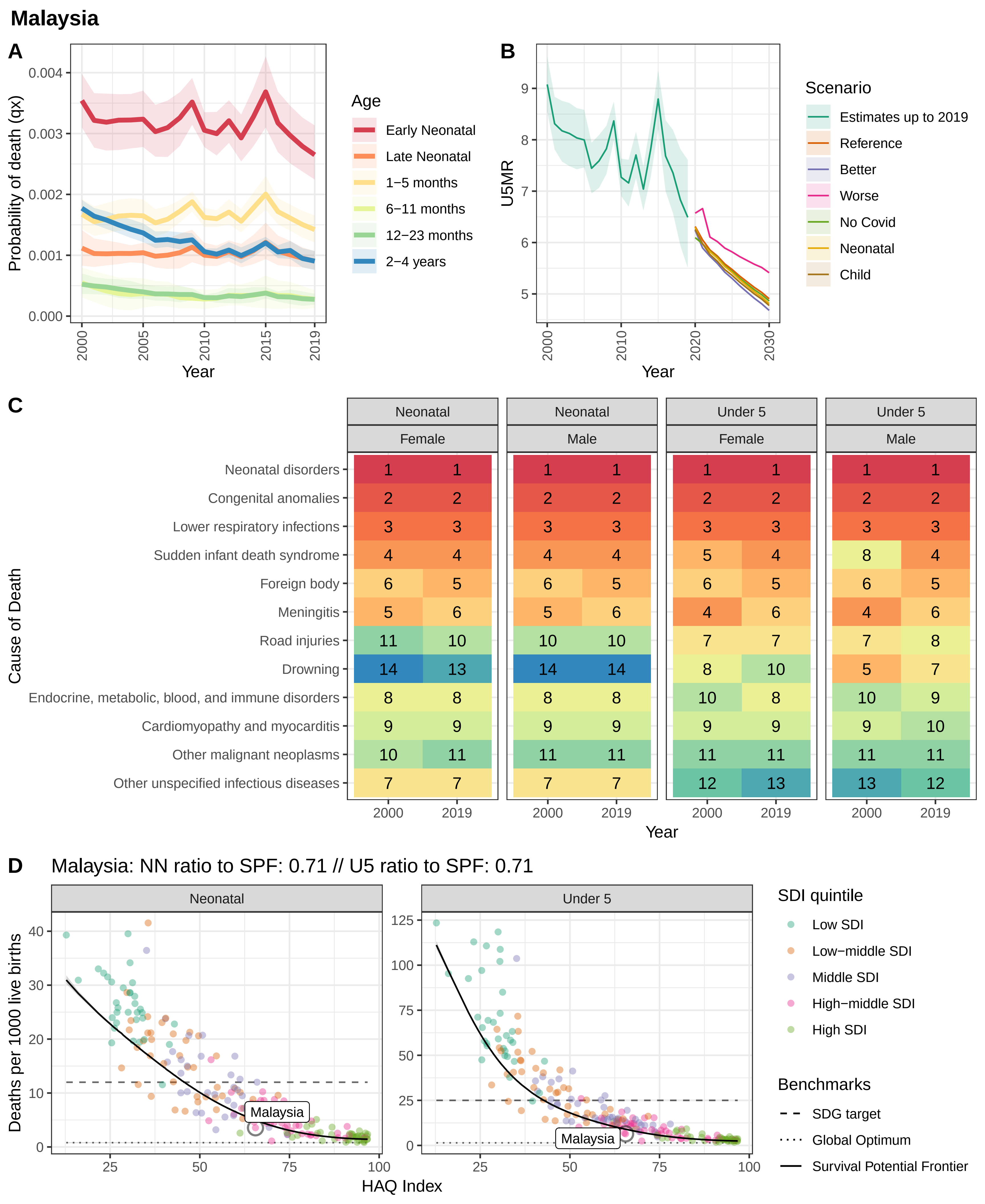

Malaysia
A
Probability of death (qx)
Year
Age
Early Neonatal
Late Neonatal
1–5 months
6–11 months
12–23 months
2–4 years
B
U5MR
Year
Scenario
Estimates up to 2019
Reference
Better
Worse
No Covid
Neonatal
Child
C
Neonatal
Female
Neonatal
Male
Under 5
Female
Under 5
Male
Cause of Death
Neonatal disorders
Congenital anomalies
Lower respiratory infections
Sudden infant death syndrome
Foreign body
Meningitis
Road injuries
Drowning
Endocrine, metabolic, blood, and immune disorders
Cardiomyopathy and myocarditis
Other malignant neoplasms
Other unspecified infectious diseases
Year
D
Malaysia: NN ratio to SPF: 0.71 // U5 ratio to SPF: 0.71
Neonatal
Under 5
Deaths per 1000 live births
HAQ Index
Malaysia
SDI quintile
Low SDI
Low−middle SDI
Middle SDI
High−middle SDI
High SDI
Benchmarks
SDG target
Global Optimum
Survival Potential Frontier

Mexico

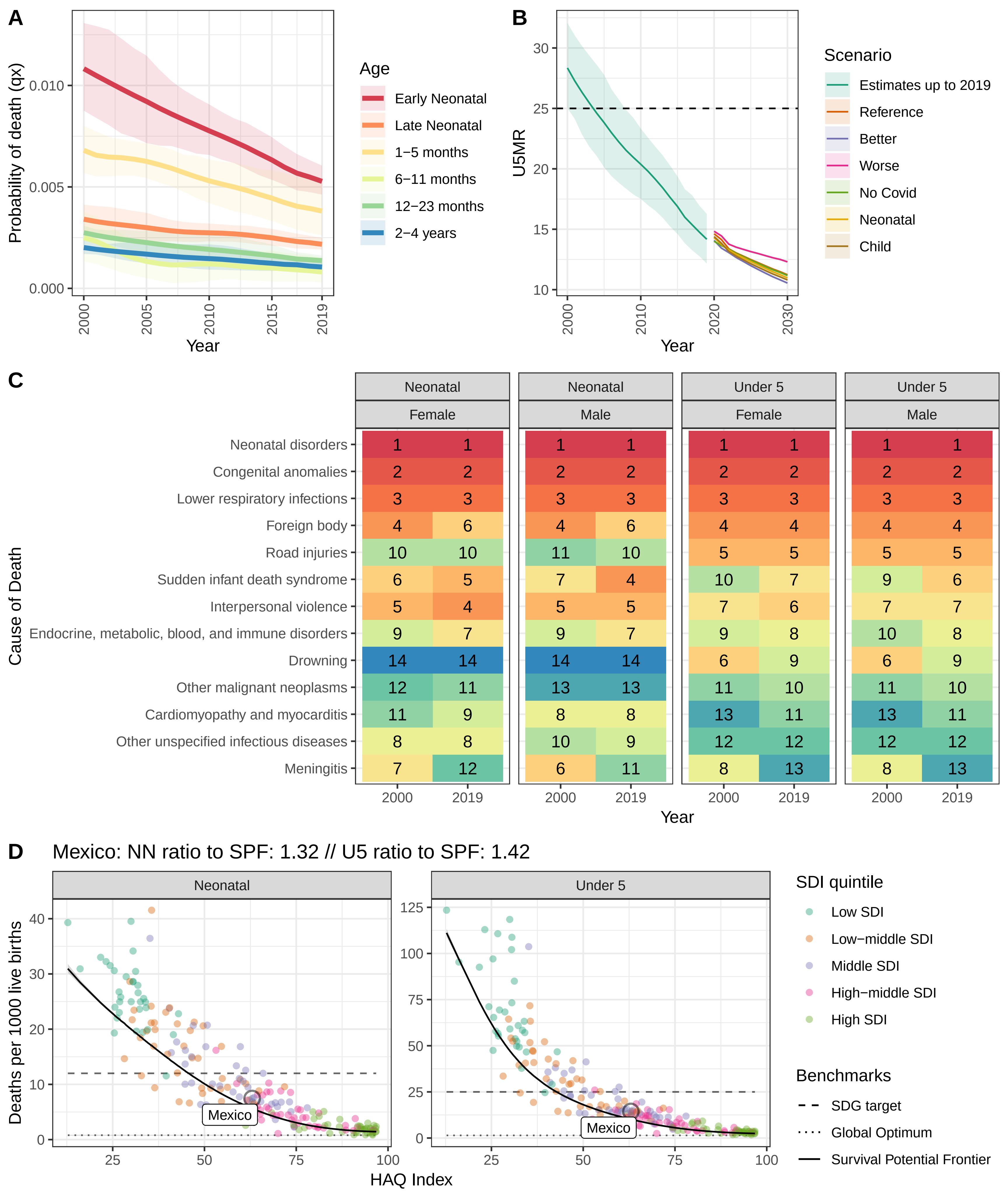

A
Probability of death (qx)
0.010
0.005
0.000
2000
2005
2010
2015
2019
Year
Age
Early Neonatal
Late Neonatal
1−5 months
6−11 months
12−23 months
2−4 years
B
U5MR
30
25
20
15
10
2000
2010
2020
2030
Year
Scenario
Estimates up to 2019
Reference
Better
Worse
No Covid
Neonatal
Child
C
Neonatal Female
Neonatal Male
Under 5 Female
Under 5 Male
Cause of Death
Neonatal disorders 1 1 1 1 1 1 1 1
Congenital anomalies 2 2 2 2 2 2 2 2
Lower respiratory infections 3 3 3 3 3 3 3 3
Foreign body 4 6 4 6 4 4 4 4
Road injuries 10 10 11 10 5 5 5 5
Sudden infant death syndrome 6 5 7 4 10 7 9 6
Interpersonal violence 5 4 5 5 7 6 7 7
Endocrine, metabolic, blood, and immune disorders 9 7 9 7 9 8 10 8
Drowning 14 14 14 14 6 9 6 9
Other malignant neoplasms 12 11 13 13 11 10 11 10
Cardiomyopathy and myocarditis 11 9 8 8 13 11 13 11
Other unspecified infectious diseases 8 8 10 9 12 12 12 12
Meningitis 7 12 6 11 8 13 8 13
2000 2019
Year
D
Mexico: NN ratio to SPF: 1.32 // U5 ratio to SPF: 1.42
Neonatal
Under 5
Deaths per 1000 live births
40
30
20
10
0
125
100
75
50
25
0
Mexico
25
50
75
100
HAQ Index
SDI quintile
Low SDI
Low−middle SDI
Middle SDI
High−middle SDI
High SDI
Benchmarks
SDG target
Global Optimum
Survival Potential Frontier

Nicaragua

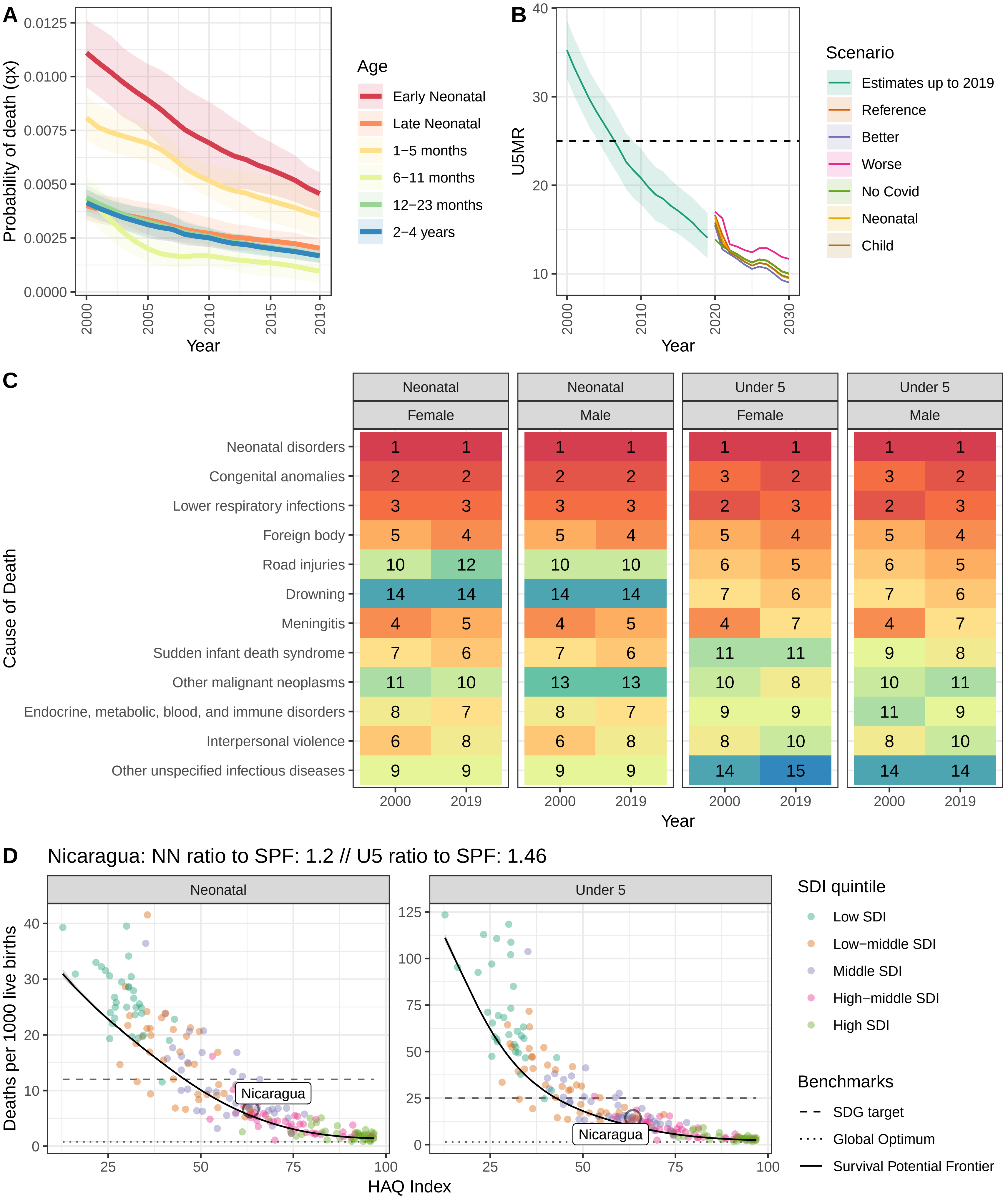

A
Probability of death (qx)
0.0125
0.0100
0.0075
0.0050
0.0025
0.0000
2000
2005
2010
2015
2019
Year
Age
Early Neonatal
Late Neonatal
1−5 months
6−11 months
12−23 months
2−4 years
B
U5MR
40
30
20
10
2000
2010
2020
2030
Year
Scenario
Estimates up to 2019
Reference
Better
Worse
No Covid
Neonatal
Child
C
Cause of Death
Neonatal Female
Neonatal Male
Under 5 Female
Under 5 Male
Neonatal disorders 1 1 | 1 1 | 1 1 | 1 1
Congenital anomalies 2 2 | 2 2 | 3 2 | 3 2
Lower respiratory infections 3 3 | 3 3 | 2 3 | 2 3
Foreign body 5 4 | 5 4 | 5 4 | 5 4
Road injuries 10 12 | 10 10 | 6 5 | 6 5
Drowning 14 14 | 14 14 | 7 6 | 7 6
Meningitis 4 5 | 4 5 | 4 7 | 4 7
Sudden infant death syndrome 7 6 | 7 6 | 11 11 | 9 8
Other malignant neoplasms 11 10 | 13 13 | 10 8 | 10 11
Endocrine, metabolic, blood, and immune disorders 8 7 | 8 7 | 9 9 | 11 9
Interpersonal violence 6 8 | 6 8 | 8 10 | 8 10
Other unspecified infectious diseases 9 9 | 9 9 | 14 15 | 14 14
2000
2019
Year
D
Nicaragua: NN ratio to SPF: 1.2 // U5 ratio to SPF: 1.46
Neonatal
Under 5
Deaths per 1000 live births
Nicaragua
HAQ Index
25
50
75
100
SDI quintile
Low SDI
Low−middle SDI
Middle SDI
High−middle SDI
High SDI
Benchmarks
SDG target
Global Optimum
Survival Potential Frontier

Panama

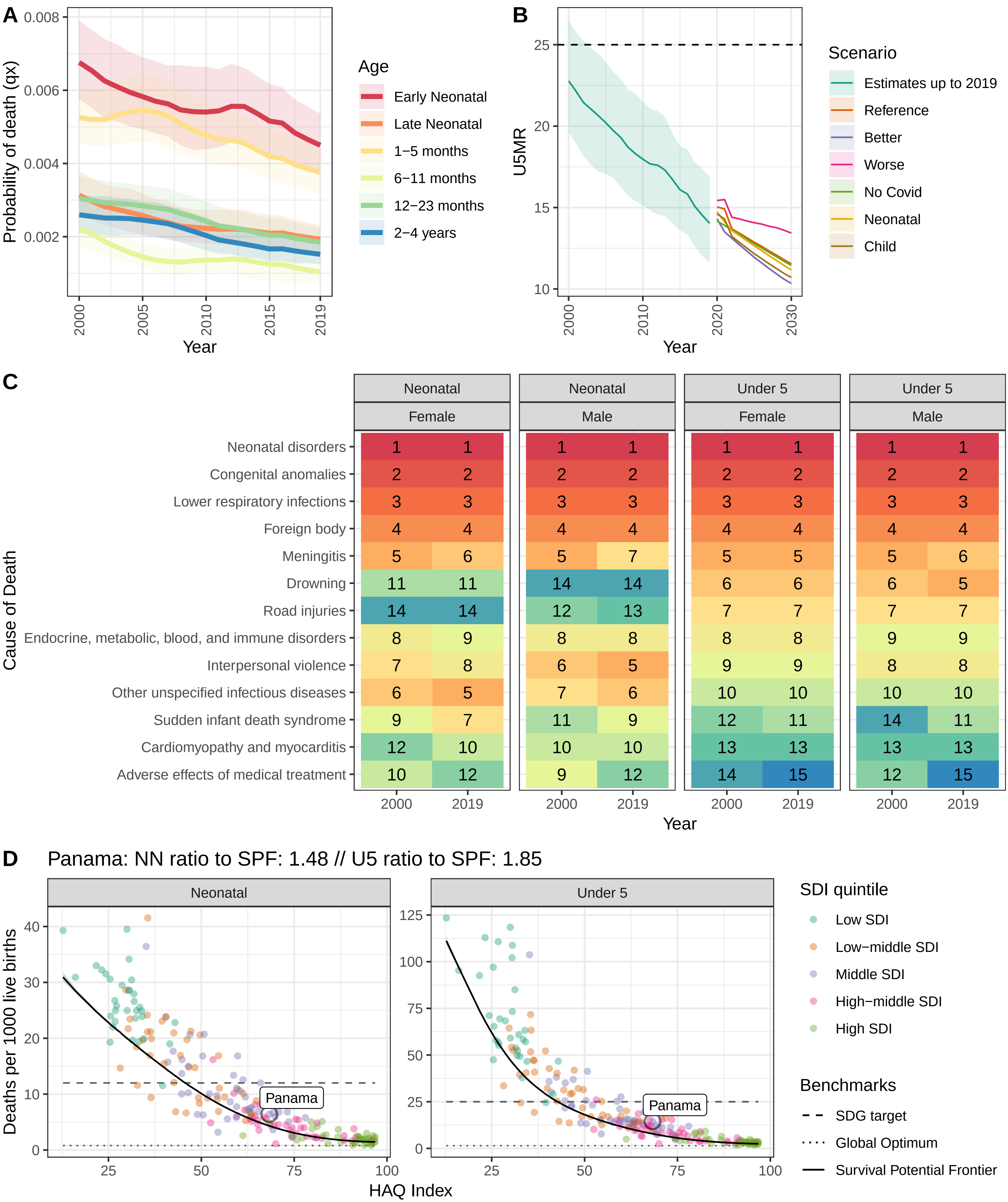
A
Probability of death (qx)
0.008
0.006
0.004
0.002
2000
2005
2010
2015
2019
Year
Age
Early Neonatal
Late Neonatal
1−5 months
6−11 months
12−23 months
2−4 years
B
U5MR
25
20
15
10
2000
2010
2020
2030
Year
Scenario
Estimates up to 2019
Reference
Better
Worse
No Covid
Neonatal
Child
C
Cause of Death
Neonatal Female | Neonatal Male | Under 5 Female | Under 5 Male
Neonatal disorders 1 1 | 1 1 | 1 1 | 1 1
Congenital anomalies 2 2 | 2 2 | 2 2 | 2 2
Lower respiratory infections 3 3 | 3 3 | 3 3 | 3 3
Foreign body 4 4 | 4 4 | 4 4 | 4 4
Meningitis 5 6 | 5 7 | 5 5 | 5 6
Drowning 11 11 | 14 14 | 6 6 | 6 5
Road injuries 14 14 | 12 13 | 7 7 | 7 7
Endocrine, metabolic, blood, and immune disorders 8 9 | 8 8 | 8 8 | 9 9
Interpersonal violence 7 8 | 6 5 | 9 9 | 8 8
Other unspecified infectious diseases 6 5 | 7 6 | 10 10 | 10 10
Sudden infant death syndrome 9 7 | 11 9 | 12 11 | 14 11
Cardiomyopathy and myocarditis 12 10 | 10 10 | 13 13 | 13 13
Adverse effects of medical treatment 10 12 | 9 12 | 14 15 | 12 15
2000 2019
Year
D
Panama: NN ratio to SPF: 1.48 // U5 ratio to SPF: 1.85
Neonatal
Under 5
Deaths per 1000 live births
40
30
20
10
0
125
100
75
50
25
0
Panama
25
50
75
100
HAQ Index
SDI quintile
Low SDI
Low−middle SDI
Middle SDI
High−middle SDI
High SDI
Benchmarks
SDG target
Global Optimum
Survival Potential Frontier

Venezuela (Bolivarian Republic of)

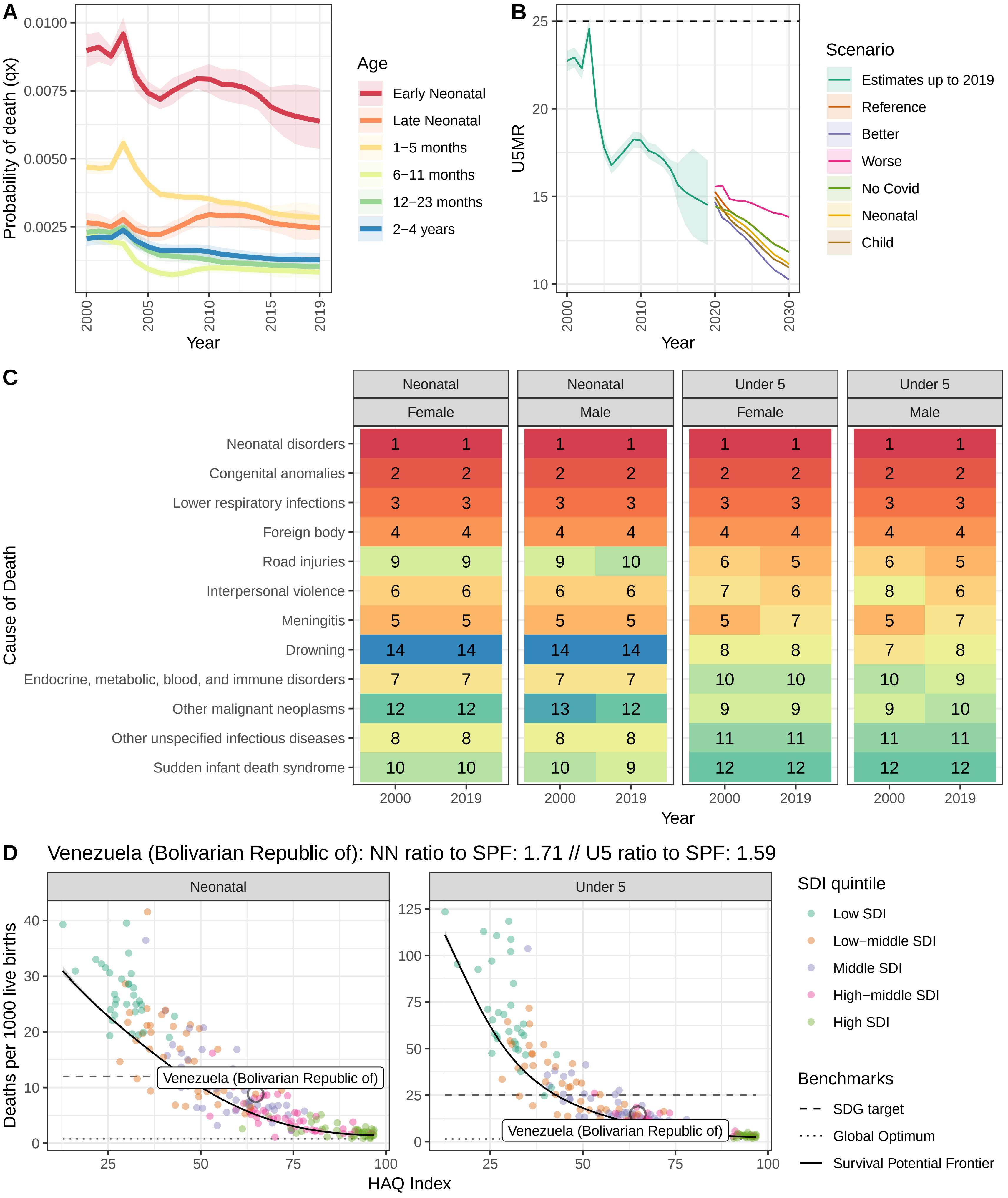

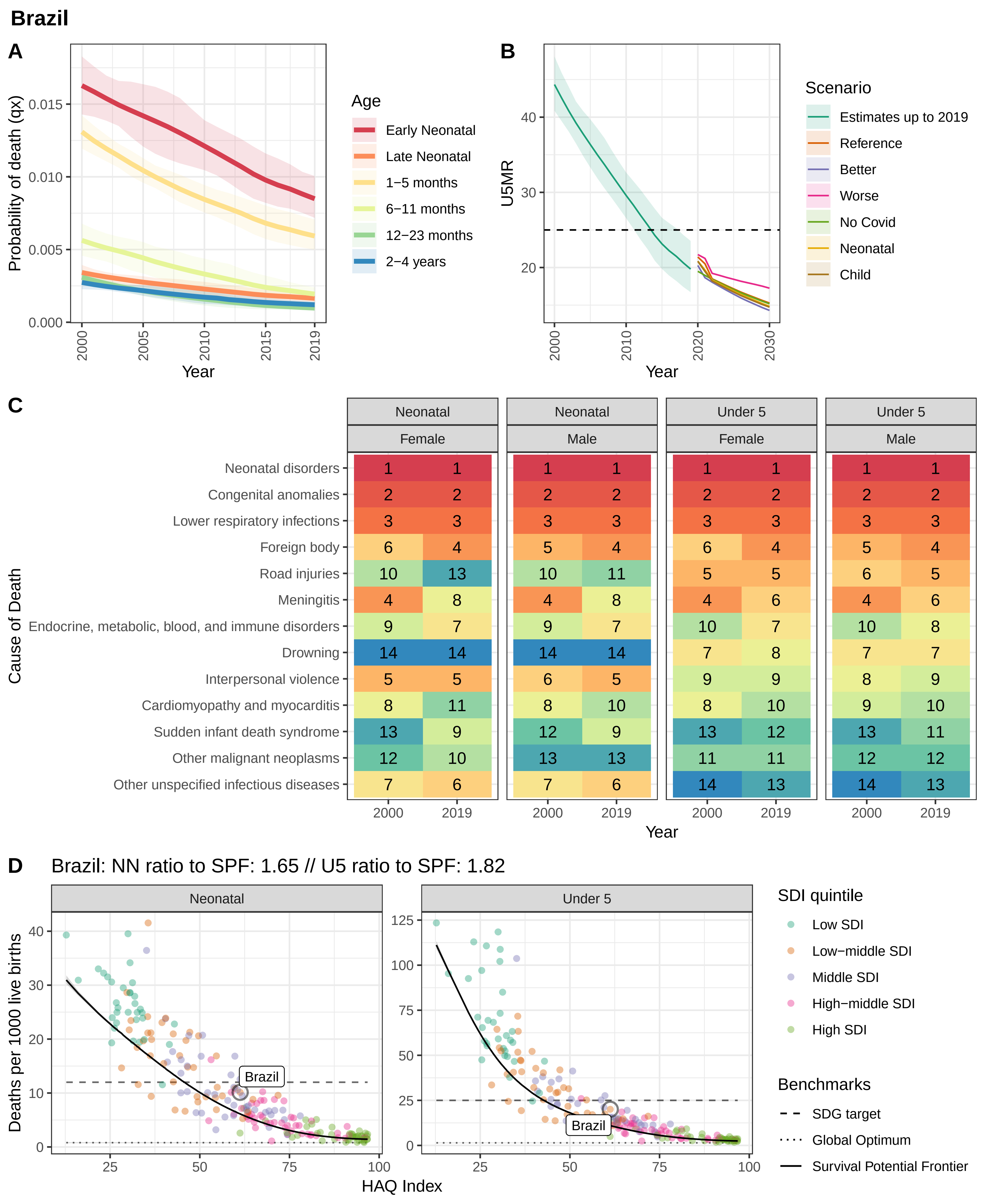
Brazil
A
Probability of death (qx)
0.015
0.010
0.005
0.000
2000
2005
2010
2015
2019
Year
Age
Early Neonatal
Late Neonatal
1−5 months
6−11 months
12−23 months
2−4 years
B
U5MR
40
30
20
2000
2010
2020
2030
Year
Scenario
Estimates up to 2019
Reference
Better
Worse
No Covid
Neonatal
Child
C
Neonatal
Female
Neonatal
Male
Under 5
Female
Under 5
Male
Cause of Death
Neonatal disorders 1 1 1 1 1 1 1 1
Congenital anomalies 2 2 2 2 2 2 2 2
Lower respiratory infections 3 3 3 3 3 3 3 3
Foreign body 6 4 5 4 6 4 5 4
Road injuries 10 13 10 11 5 5 6 5
Meningitis 4 8 4 8 4 6 4 6
Endocrine, metabolic, blood, and immune disorders 9 7 9 7 10 7 10 8
Drowning 14 14 14 14 7 8 7 7
Interpersonal violence 5 5 6 5 9 9 8 9
Cardiomyopathy and myocarditis 8 11 8 10 8 10 9 10
Sudden infant death syndrome 13 9 12 9 13 12 13 11
Other malignant neoplasms 12 10 13 13 11 11 12 12
Other unspecified infectious diseases 7 6 7 6 14 13 14 13
2000
2019
Year
D
Brazil: NN ratio to SPF: 1.65 // U5 ratio to SPF: 1.82
Neonatal
Under 5
Deaths per 1000 live births
40
30
20
10
0
125
100
75
50
25
0
Brazil
25
50
75
100
HAQ Index
SDI quintile
Low SDI
Low−middle SDI
Middle SDI
High−middle SDI
High SDI
Benchmarks
SDG target
Global Optimum
Survival Potential Frontier

Paraguay

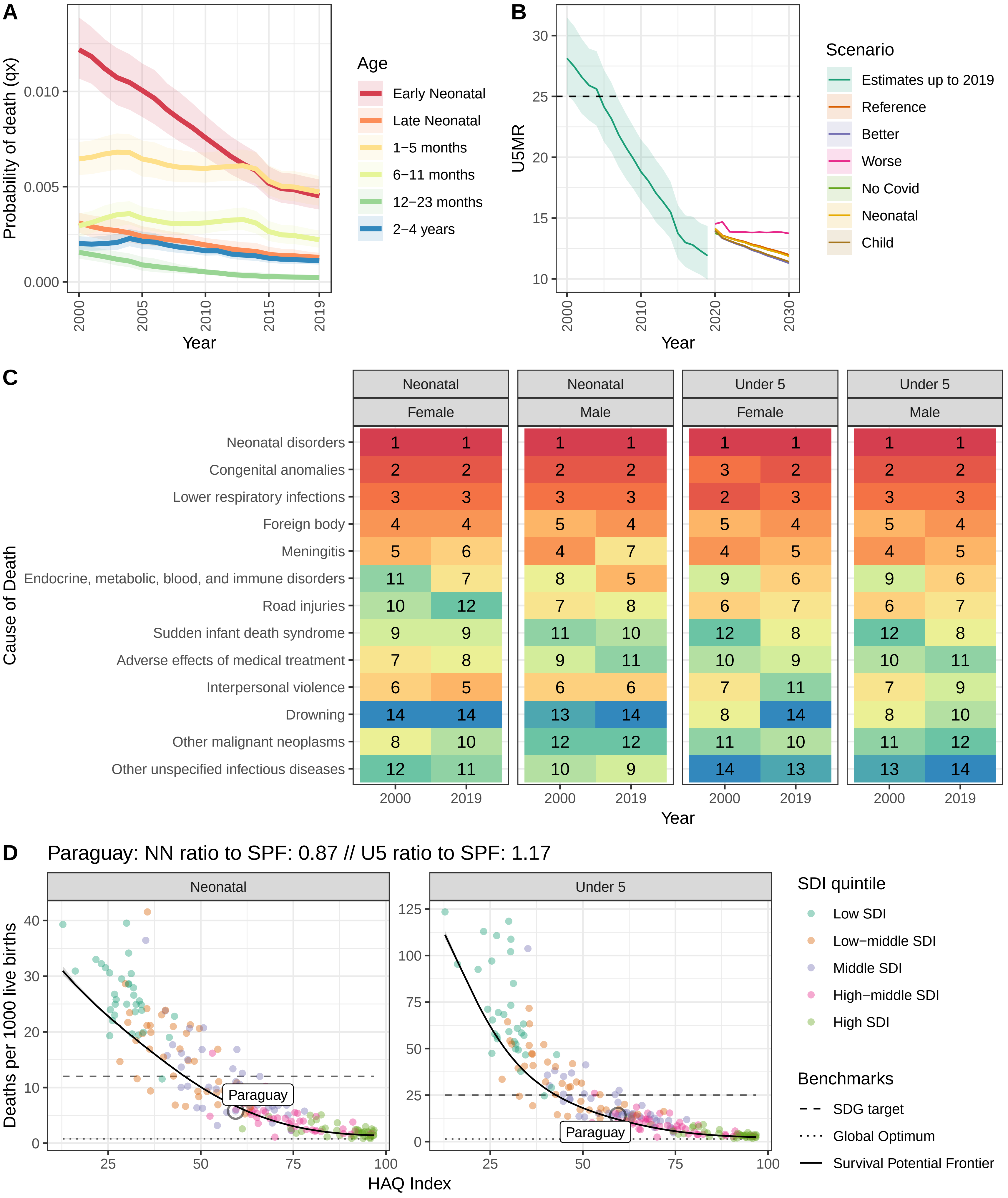
A
Age
Early Neonatal
Late Neonatal
1−5 months
6−11 months
12−23 months
2−4 years
Probability of death (qx)
0.000
0.005
0.010
2000
2005
2010
2015
2019
Year
B
Scenario
Estimates up to 2019
Reference
Better
Worse
No Covid
Neonatal
Child
U5MR
10
15
20
25
30
2000
2010
2020
2030
Year
C
Neonatal Female
Neonatal Male
Under 5 Female
Under 5 Male
Cause of Death
Neonatal disorders 1 1 1 1 1 1 1 1
Congenital anomalies 2 2 2 2 3 2 2 2
Lower respiratory infections 3 3 3 3 2 3 3 3
Foreign body 4 4 5 4 5 4 5 4
Meningitis 5 6 4 7 4 5 4 5
Endocrine, metabolic, blood, and immune disorders 11 7 8 5 9 6 9 6
Road injuries 10 12 7 8 6 7 6 7
Sudden infant death syndrome 9 9 11 10 12 8 12 8
Adverse effects of medical treatment 7 8 9 11 10 9 10 11
Interpersonal violence 6 5 6 6 7 11 7 9
Drowning 14 14 13 14 8 14 8 10
Other malignant neoplasms 8 10 12 12 11 10 11 12
Other unspecified infectious diseases 12 11 10 9 14 13 13 14
2000
2019
Year
D
Paraguay: NN ratio to SPF: 0.87 // U5 ratio to SPF: 1.17
Neonatal
Under 5
Paraguay
Deaths per 1000 live births
0
10
20
30
40
25
50
75
100
125
HAQ Index
SDI quintile
Low SDI
Low−middle SDI
Middle SDI
High−middle SDI
High SDI
Benchmarks
SDG target
Global Optimum
Survival Potential Frontier

Algeria

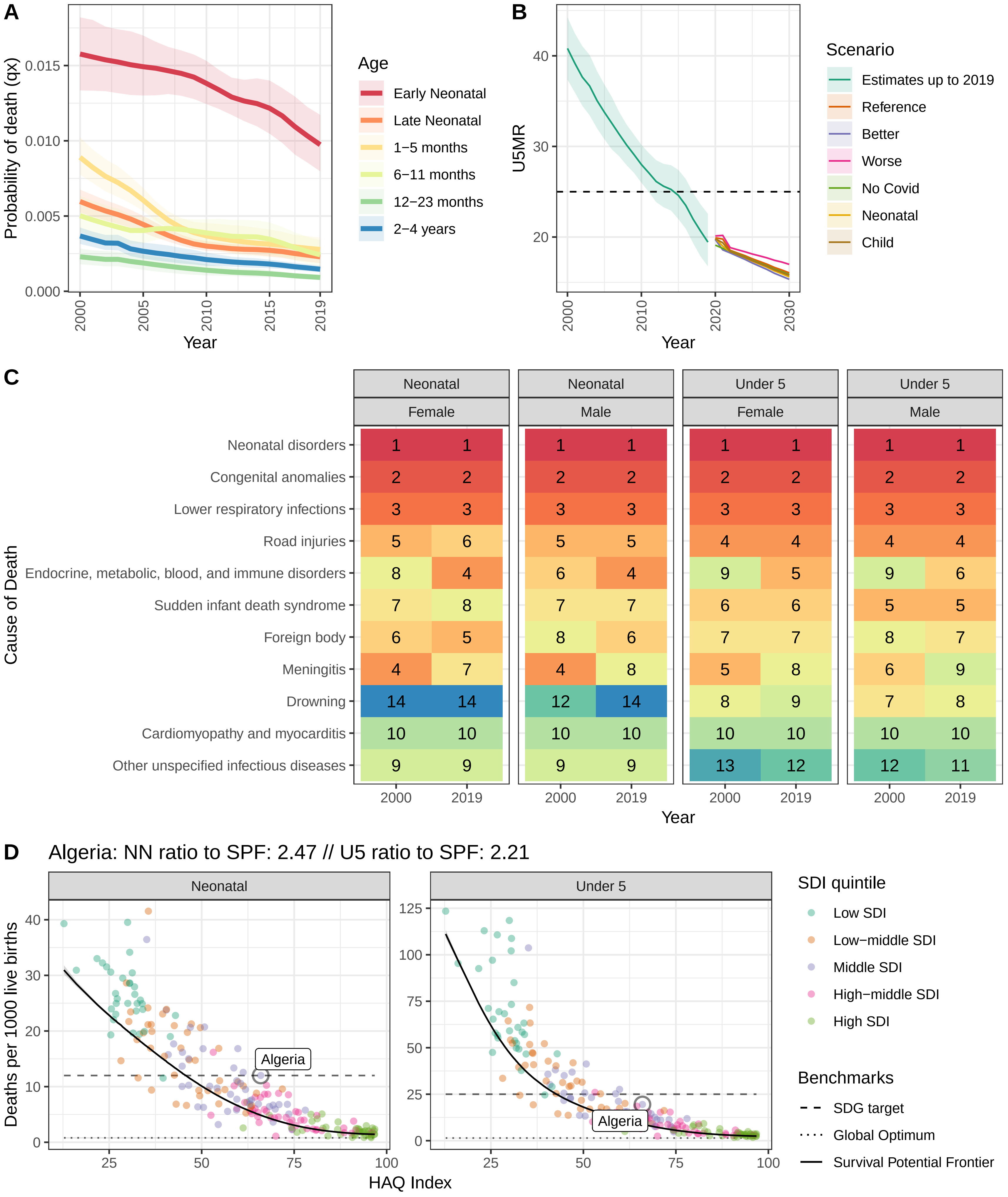
A
Probability of death (qx)
0.015
0.010
0.005
0.000
2000
2005
2010
2015
2019
Year
Age
Early Neonatal
Late Neonatal
1−5 months
6−11 months
12−23 months
2−4 years
B
U5MR
40
30
20
2000
2010
2020
2030
Year
Scenario
Estimates up to 2019
Reference
Better
Worse
No Covid
Neonatal
Child
C
Cause of Death
Neonatal
Female
Neonatal
Male
Under 5
Female
Under 5
Male
Neonatal disorders 1 1 1 1 1 1 1 1
Congenital anomalies 2 2 2 2 2 2 2 2
Lower respiratory infections 3 3 3 3 3 3 3 3
Road injuries 5 6 5 5 4 4 4 4
Endocrine, metabolic, blood, and immune disorders 8 4 6 4 9 5 9 6
Sudden infant death syndrome 7 8 7 7 6 6 5 5
Foreign body 6 5 8 6 7 7 8 7
Meningitis 4 7 4 8 5 8 6 9
Drowning 14 14 12 14 8 9 7 8
Cardiomyopathy and myocarditis 10 10 10 10 10 10 10 10
Other unspecified infectious diseases 9 9 9 9 13 12 12 11
2000
2019
Year
D
Algeria: NN ratio to SPF: 2.47 // U5 ratio to SPF: 2.21
Neonatal
Under 5
Deaths per 1000 live births
40
30
20
10
0
125
100
75
50
25
0
Algeria
Algeria
25
50
75
100
HAQ Index
SDI quintile
Low SDI
Low−middle SDI
Middle SDI
High−middle SDI
High SDI
Benchmarks
SDG target
Global Optimum
Survival Potential Frontier

Maldives

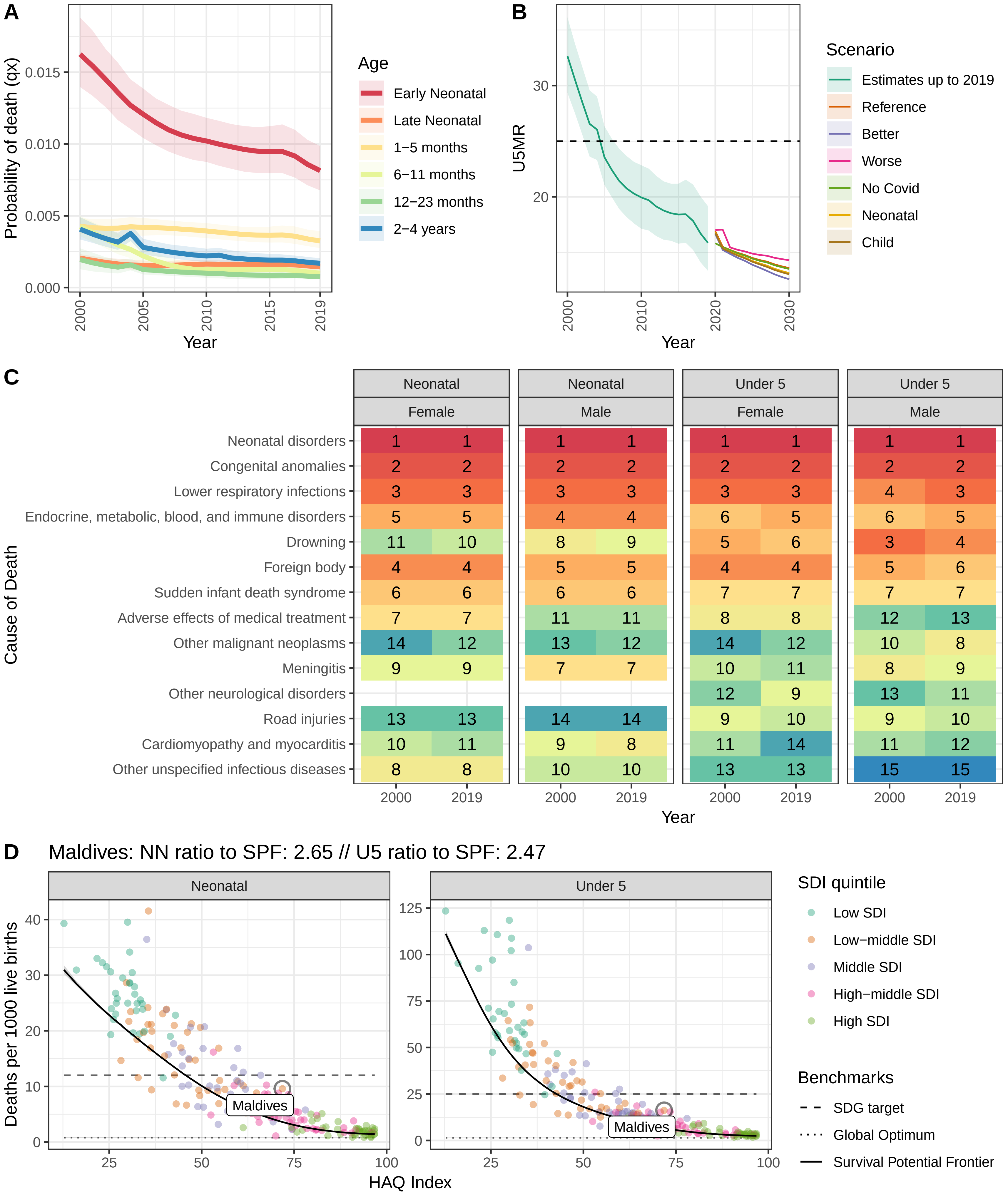
A
Probability of death (qx)
0.015
0.010
0.005
0.000
2000
2005
2010
2015
2019
Year
Age
Early Neonatal
Late Neonatal
1−5 months
6−11 months
12−23 months
2−4 years
B
U5MR
30
20
2000
2010
2020
2030
Year
Scenario
Estimates up to 2019
Reference
Better
Worse
No Covid
Neonatal
Child
C
Cause of Death
Neonatal
Female
Neonatal
Male
Under 5
Female
Under 5
Male
Neonatal disorders 1 1 1 1 1 1 1 1
Congenital anomalies 2 2 2 2 2 2 2 2
Lower respiratory infections 3 3 3 3 3 3 4 3
Endocrine, metabolic, blood, and immune disorders 5 5 4 4 6 5 6 5
Drowning 11 10 8 9 5 6 3 4
Foreign body 4 4 5 5 4 4 5 6
Sudden infant death syndrome 6 6 6 6 7 7 7 7
Adverse effects of medical treatment 7 7 11 11 8 8 12 13
Other malignant neoplasms 14 12 13 12 14 12 10 8
Meningitis 9 9 7 7 10 11 8 9
Other neurological disorders 12 9 13 11
Road injuries 13 13 14 14 9 10 9 10
Cardiomyopathy and myocarditis 10 11 9 8 11 14 11 12
Other unspecified infectious diseases 8 8 10 10 13 13 15 15
2000
2019
Year
D
Maldives: NN ratio to SPF: 2.65 // U5 ratio to SPF: 2.47
Neonatal
Under 5
Deaths per 1000 live births
40
30
20
10
0
125
100
75
50
25
0
25
50
75
100
HAQ Index
Maldives
SDI quintile
Low SDI
Low−middle SDI
Middle SDI
High−middle SDI
High SDI
Benchmarks
SDG target
Global Optimum
Survival Potential Frontier

Bahrain

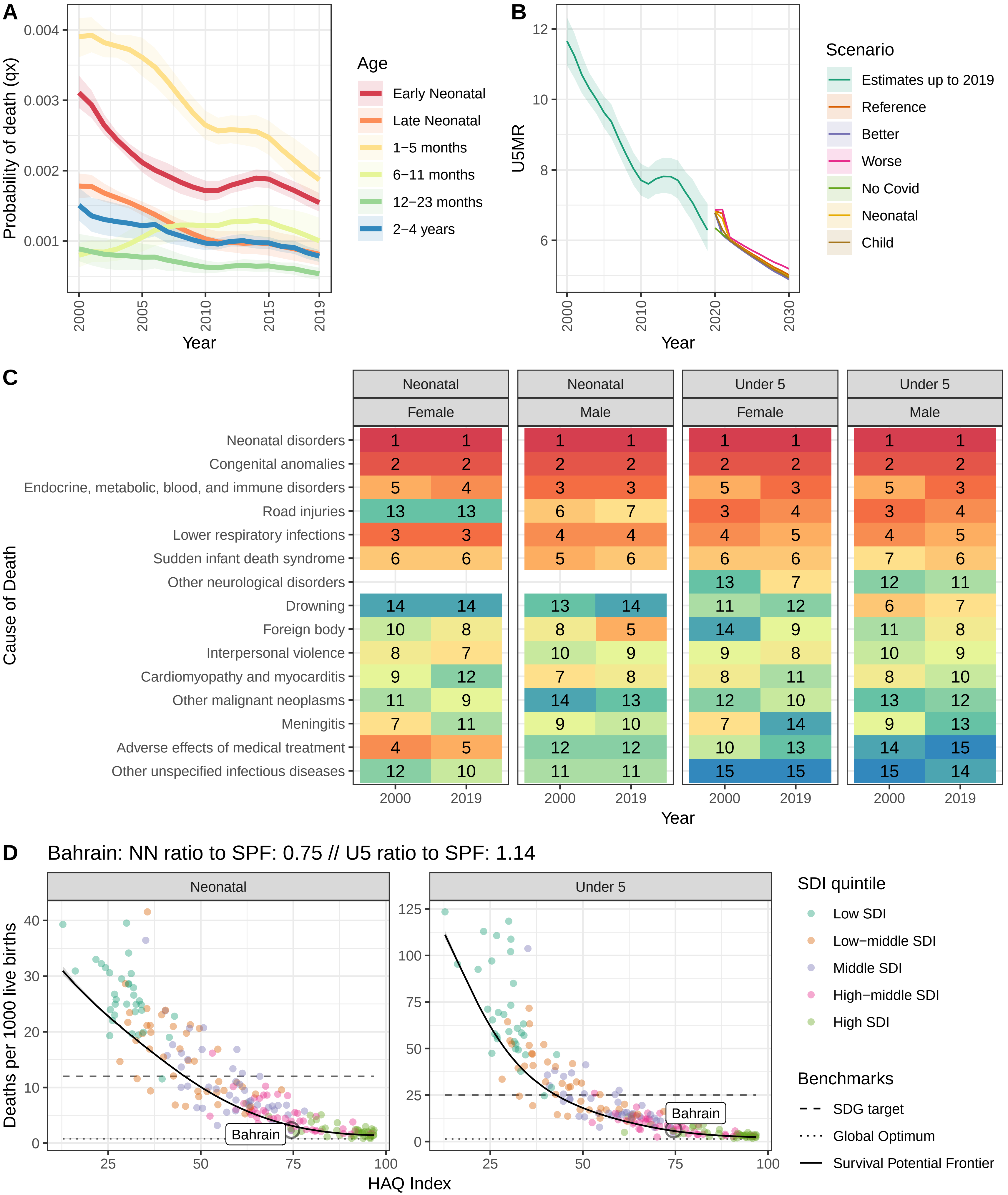

A
Probability of death (qx)
0.004
0.003
0.002
0.001
2000
2005
2010
2015
2019
Year
Age
Early Neonatal
Late Neonatal
1−5 months
6−11 months
12−23 months
2−4 years
B
U5MR
12
10
8
6
2000
2010
2020
2030
Year
Scenario
Estimates up to 2019
Reference
Better
Worse
No Covid
Neonatal
Child
C
Cause of Death
Neonatal Female
Neonatal Male
Under 5 Female
Under 5 Male
Neonatal disorders 1 1 1 1 1 1 1 1
Congenital anomalies 2 2 2 2 2 2 2 2
Endocrine, metabolic, blood, and immune disorders 5 4 3 3 5 3 5 3
Road injuries 13 13 6 7 3 4 3 4
Lower respiratory infections 3 3 4 4 4 5 4 5
Sudden infant death syndrome 6 6 5 6 6 6 7 6
Other neurological disorders 13 7 12 11
Drowning 14 14 13 14 11 12 6 7
Foreign body 10 8 8 5 14 9 11 8
Interpersonal violence 8 7 10 9 9 8 10 9
Cardiomyopathy and myocarditis 9 12 7 8 8 11 8 10
Other malignant neoplasms 11 9 14 13 12 10 13 12
Meningitis 7 11 9 10 7 14 9 13
Adverse effects of medical treatment 4 5 12 12 10 13 14 15
Other unspecified infectious diseases 12 10 11 11 15 15 15 14
2000 2019 2000 2019 2000 2019 2000 2019
Year
D
Bahrain: NN ratio to SPF: 0.75 // U5 ratio to SPF: 1.14
Neonatal
Under 5
Deaths per 1000 live births
40
30
20
10
0
125
100
75
50
25
0
25
50
75
100
HAQ Index
Bahrain
SDI quintile
Low SDI
Low−middle SDI
Middle SDI
High−middle SDI
High SDI
Benchmarks
SDG target
Global Optimum
Survival Potential Frontier

Egypt

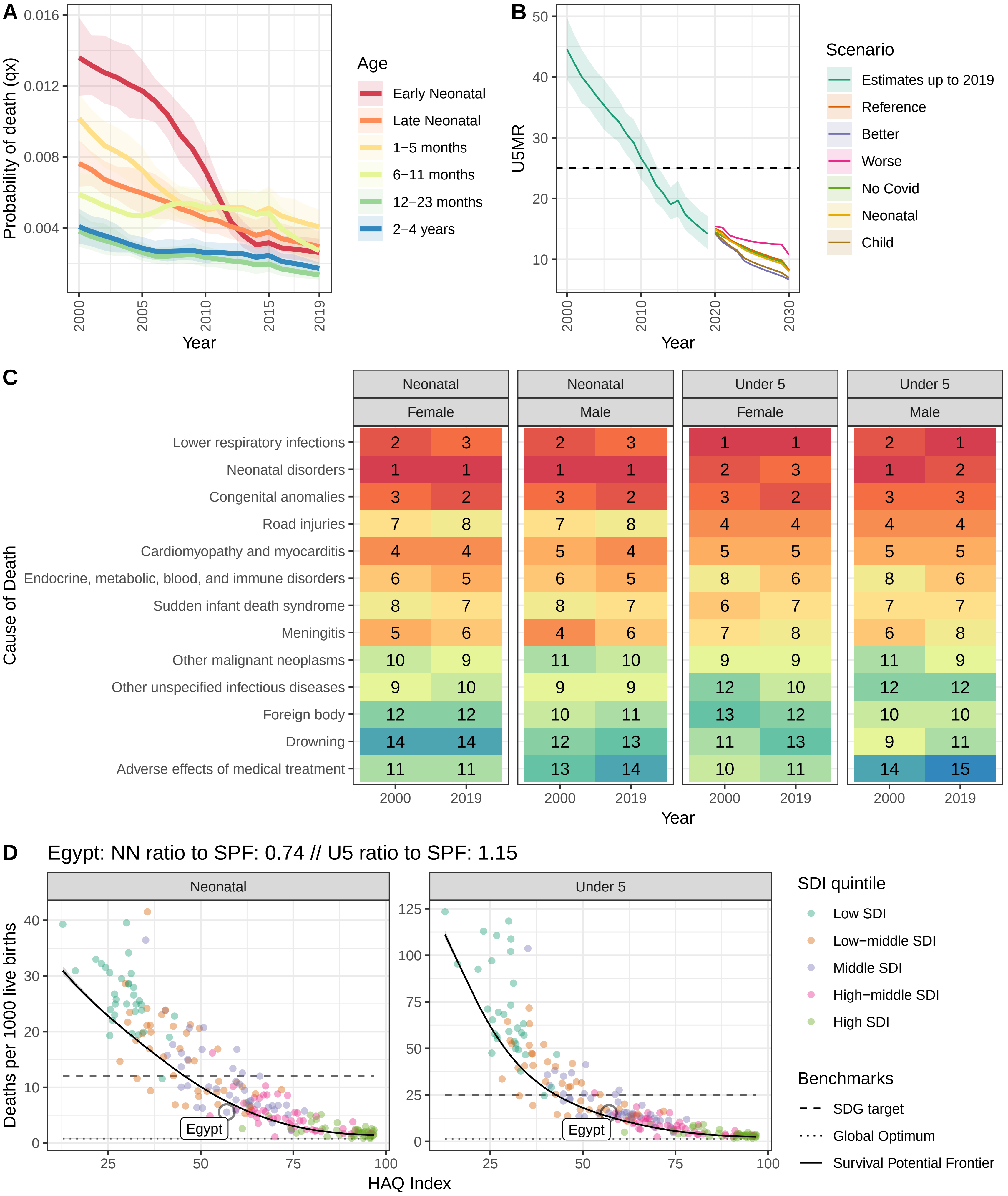

A
Probability of death (qx)
Year
Age
Early Neonatal
Late Neonatal
1−5 months
6−11 months
12−23 months
2−4 years
B
U5MR
Year
Scenario
Estimates up to 2019
Reference
Better
Worse
No Covid
Neonatal
Child
C
Cause of Death
Neonatal
Female
Male
Under 5
Lower respiratory infections
Neonatal disorders
Congenital anomalies
Road injuries
Cardiomyopathy and myocarditis
Endocrine, metabolic, blood, and immune disorders
Sudden infant death syndrome
Meningitis
Other malignant neoplasms
Other unspecified infectious diseases
Foreign body
Drowning
Adverse effects of medical treatment
Year
D
Egypt: NN ratio to SPF: 0.74 // U5 ratio to SPF: 1.15
Neonatal
Under 5
Deaths per 1000 live births
HAQ Index
Egypt
SDI quintile
Low SDI
Low−middle SDI
Middle SDI
High−middle SDI
High SDI
Benchmarks
SDG target
Global Optimum
Survival Potential Frontier

Iran (Islamic Republic of)

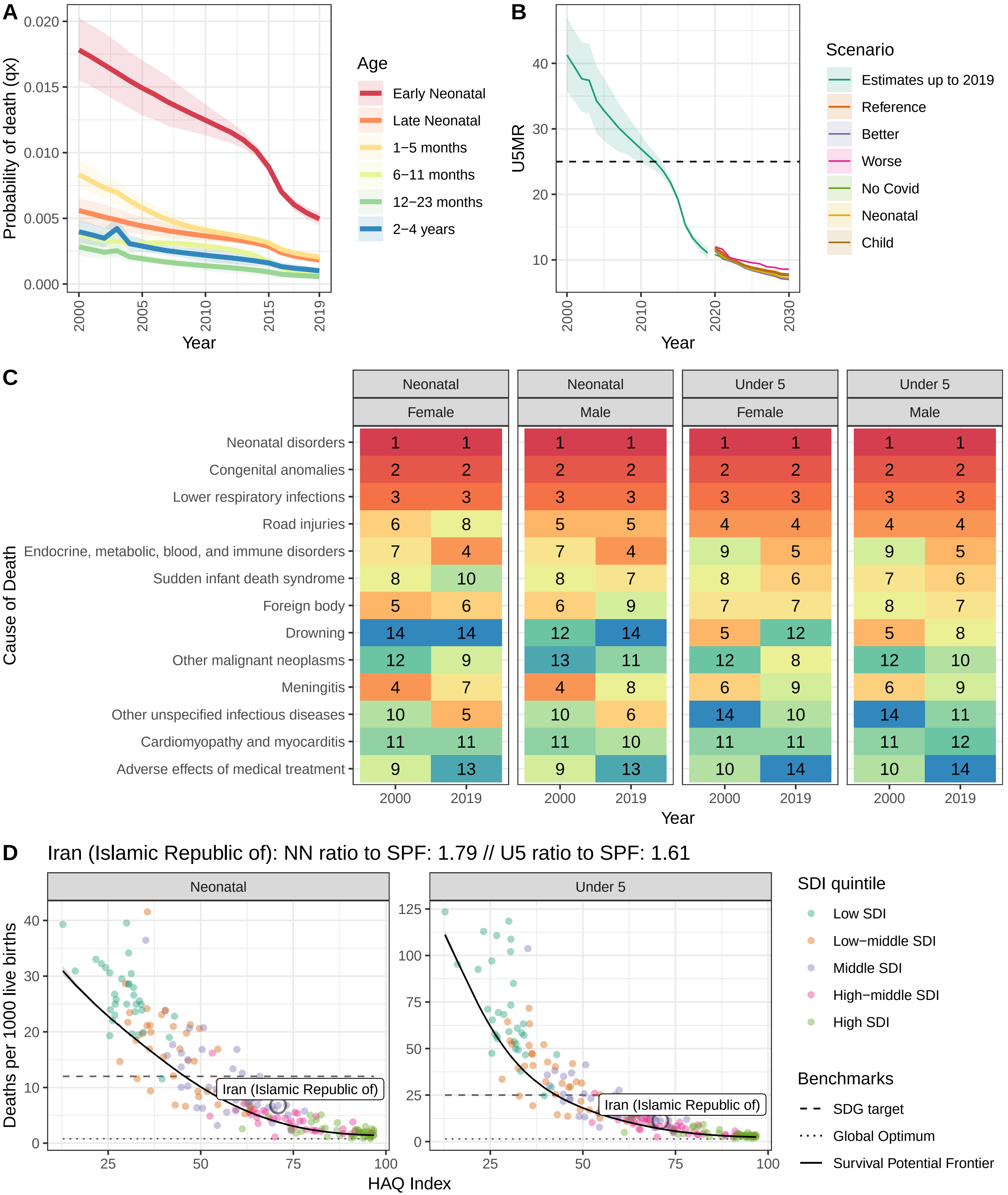

Iraq

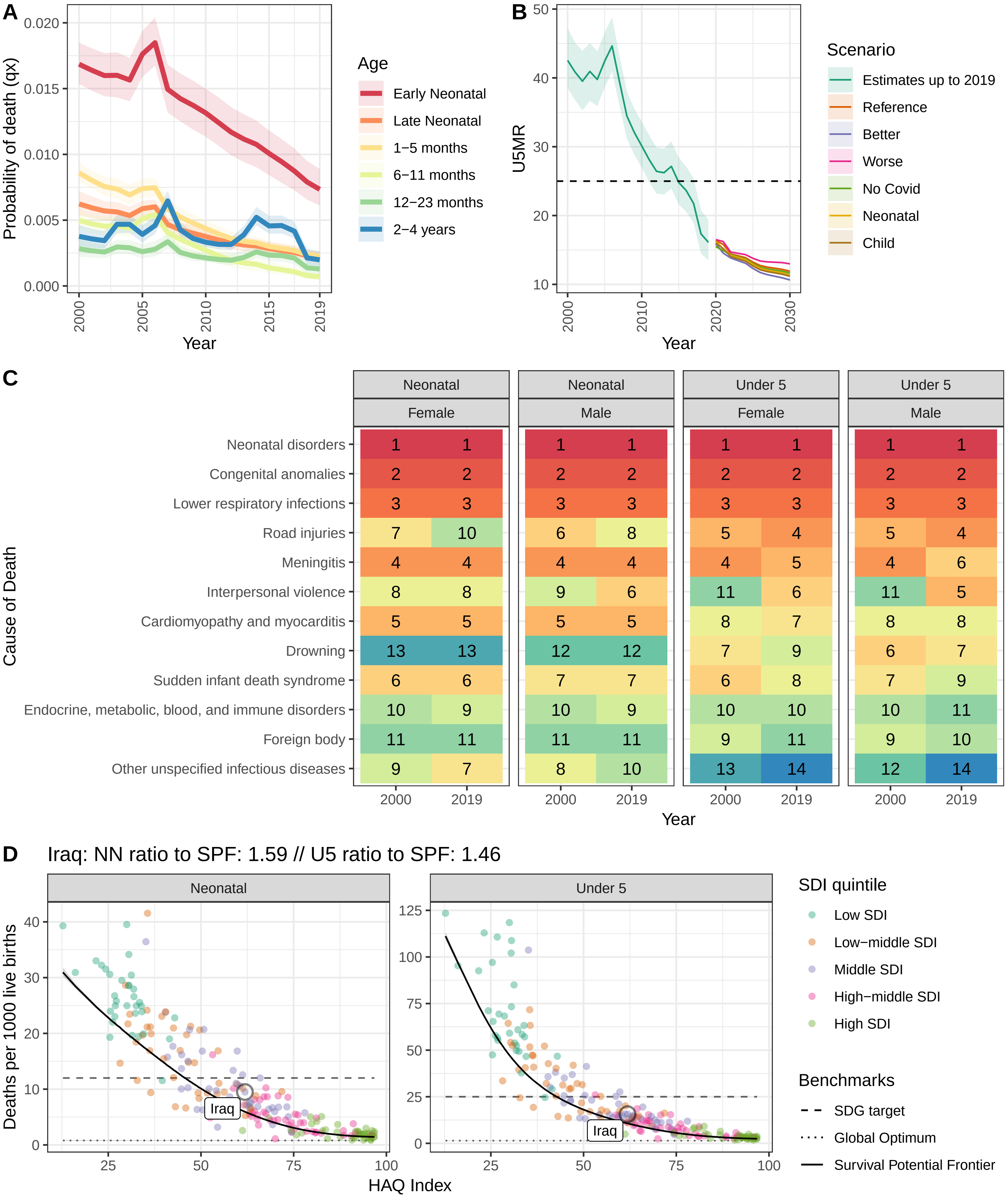
A
Probability of death (qx)
Year
Age
Early Neonatal
Late Neonatal
1−5 months
6−11 months
12−23 months
2−4 years
B
U5MR
Year
Scenario
Estimates up to 2019
Reference
Better
Worse
No Covid
Neonatal
Child
C
Cause of Death
Neonatal
Female
Male
Under 5
Neonatal disorders
Congenital anomalies
Lower respiratory infections
Road injuries
Meningitis
Interpersonal violence
Cardiomyopathy and myocarditis
Drowning
Sudden infant death syndrome
Endocrine, metabolic, blood, and immune disorders
Foreign body
Other unspecified infectious diseases
Year
D
Iraq: NN ratio to SPF: 1.59 // U5 ratio to SPF: 1.46
Neonatal
Under 5
Deaths per 1000 live births
HAQ Index
Iraq
SDI quintile
Low SDI
Low−middle SDI
Middle SDI
High−middle SDI
High SDI
Benchmarks
SDG target
Global Optimum
Survival Potential Frontier

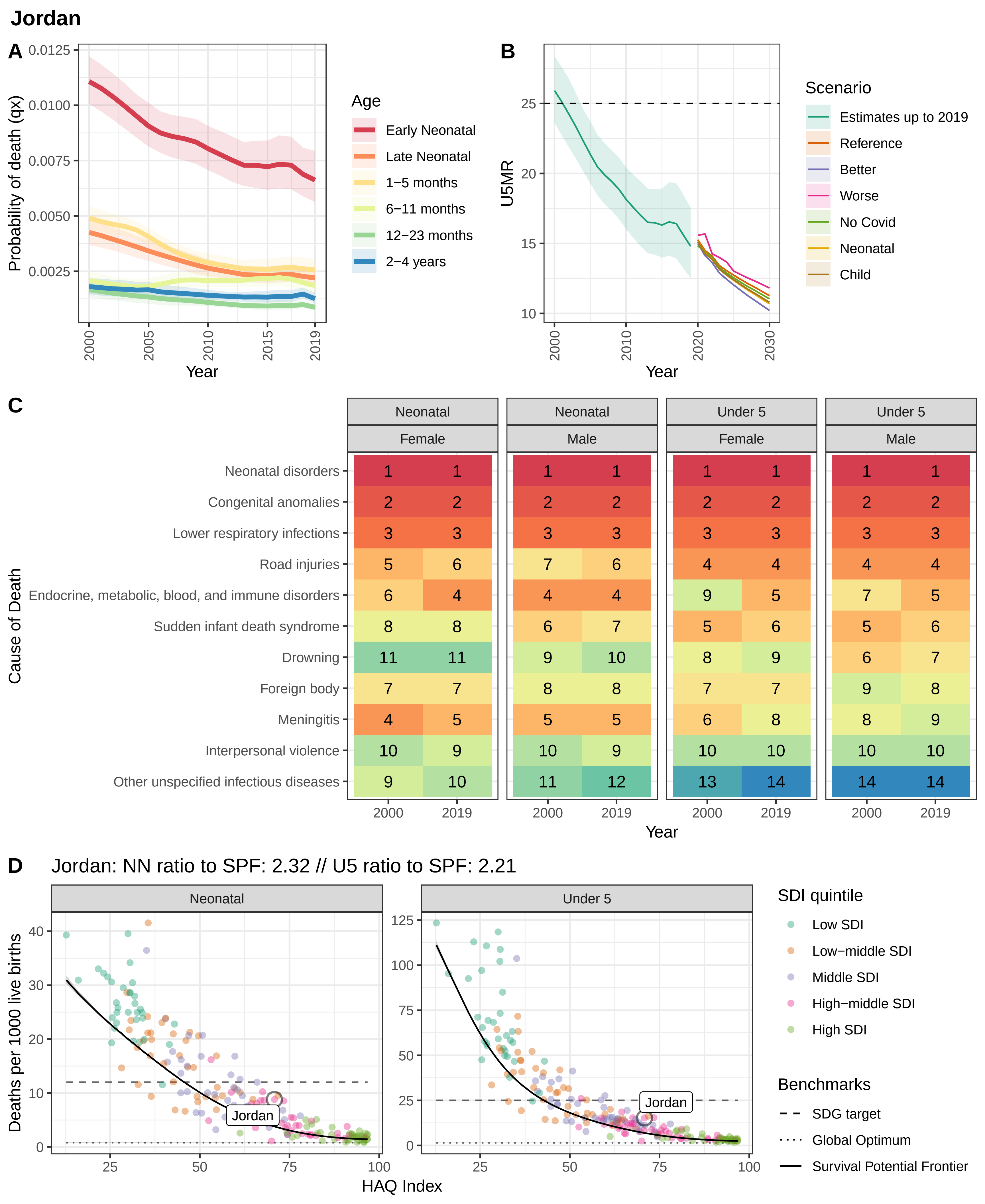

Jordan
A
Probability of death (qx)
0.0125
0.0100
0.0075
0.0050
0.0025
2000
2005
2010
2015
2019
Year
Age
Early Neonatal
Late Neonatal
1−5 months
6−11 months
12−23 months
2−4 years
B
U5MR
25
20
15
10
2000
2010
2020
2030
Year
Scenario
Estimates up to 2019
Reference
Better
Worse
No Covid
Neonatal
Child
C
Cause of Death
Neonatal Female
Neonatal Male
Under 5 Female
Under 5 Male
Neonatal disorders 1 1 1 1 1 1 1 1
Congenital anomalies 2 2 2 2 2 2 2 2
Lower respiratory infections 3 3 3 3 3 3 3 3
Road injuries 5 6 7 6 4 4 4 4
Endocrine, metabolic, blood, and immune disorders 6 4 4 4 9 5 7 5
Sudden infant death syndrome 8 8 6 7 5 6 5 6
Drowning 11 11 9 10 8 9 6 7
Foreign body 7 7 8 8 7 7 9 8
Meningitis 4 5 5 5 6 8 8 9
Interpersonal violence 10 9 10 9 10 10 10 10
Other unspecified infectious diseases 9 10 11 12 13 14 14 14
2000
2019
Year
D
Jordan: NN ratio to SPF: 2.32 // U5 ratio to SPF: 2.21
Neonatal
Under 5
Deaths per 1000 live births
40
30
20
10
0
125
100
75
50
25
0
25
50
75
100
HAQ Index
Jordan
Jordan
SDI quintile
Low SDI
Low−middle SDI
Middle SDI
High−middle SDI
High SDI
Benchmarks
SDG target
Global Optimum
Survival Potential Frontier

Kuwait

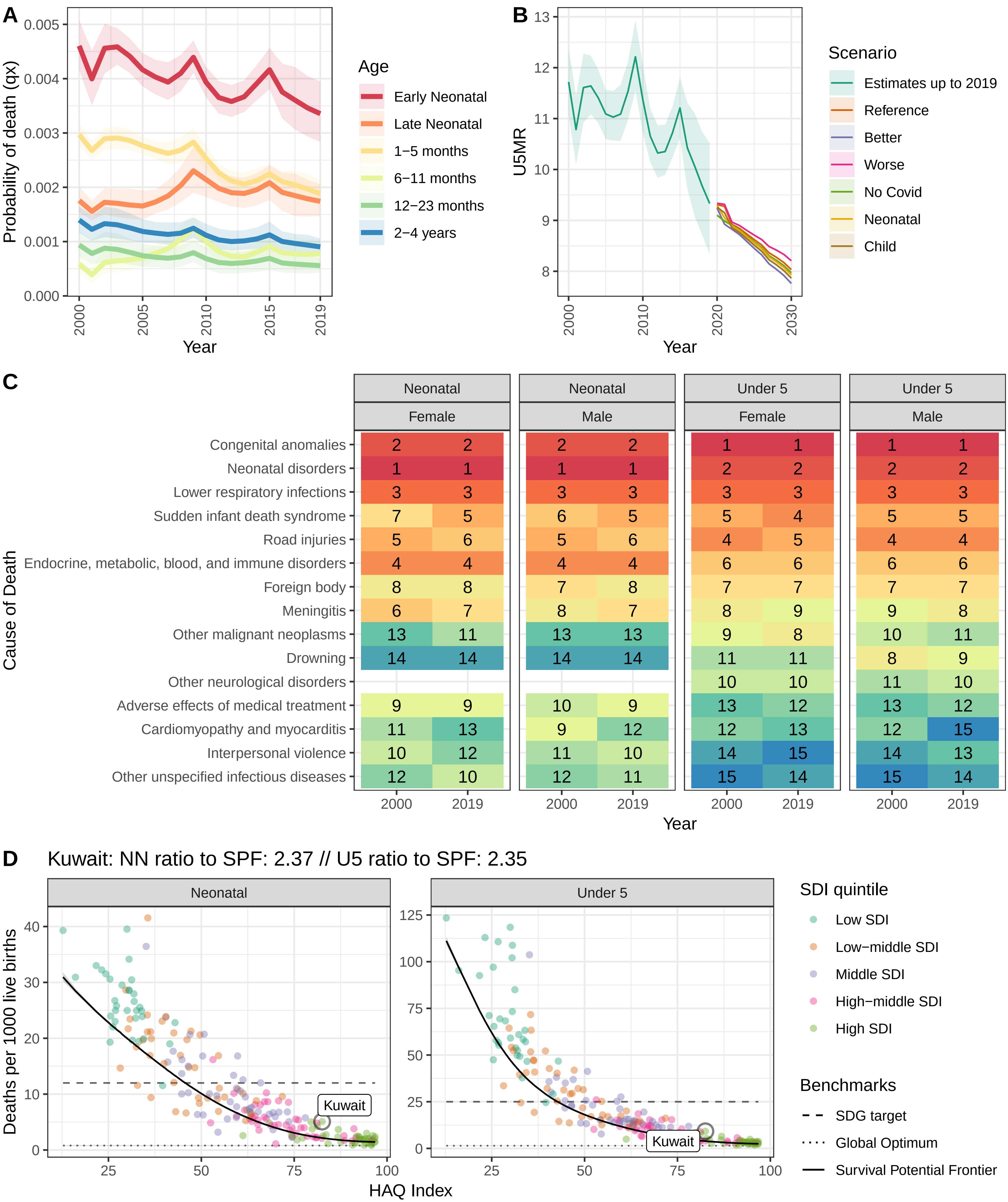

A
Probability of death (qx)
Year
Age
Early Neonatal
Late Neonatal
1−5 months
6−11 months
12−23 months
2−4 years
B
U5MR
Year
Scenario
Estimates up to 2019
Reference
Better
Worse
No Covid
Neonatal
Child
C
Cause of Death
Neonatal Female
Neonatal Male
Under 5 Female
Under 5 Male
Congenital anomalies
Neonatal disorders
Lower respiratory infections
Sudden infant death syndrome
Road injuries
Endocrine, metabolic, blood, and immune disorders
Foreign body
Meningitis
Other malignant neoplasms
Drowning
Other neurological disorders
Adverse effects of medical treatment
Cardiomyopathy and myocarditis
Interpersonal violence
Other unspecified infectious diseases
Year
D
Kuwait: NN ratio to SPF: 2.37 // U5 ratio to SPF: 2.35
Neonatal
Under 5
Deaths per 1000 live births
HAQ Index
Kuwait
SDI quintile
Low SDI
Low−middle SDI
Middle SDI
High−middle SDI
High SDI
Benchmarks
SDG target
Global Optimum
Survival Potential Frontier

Lebanon

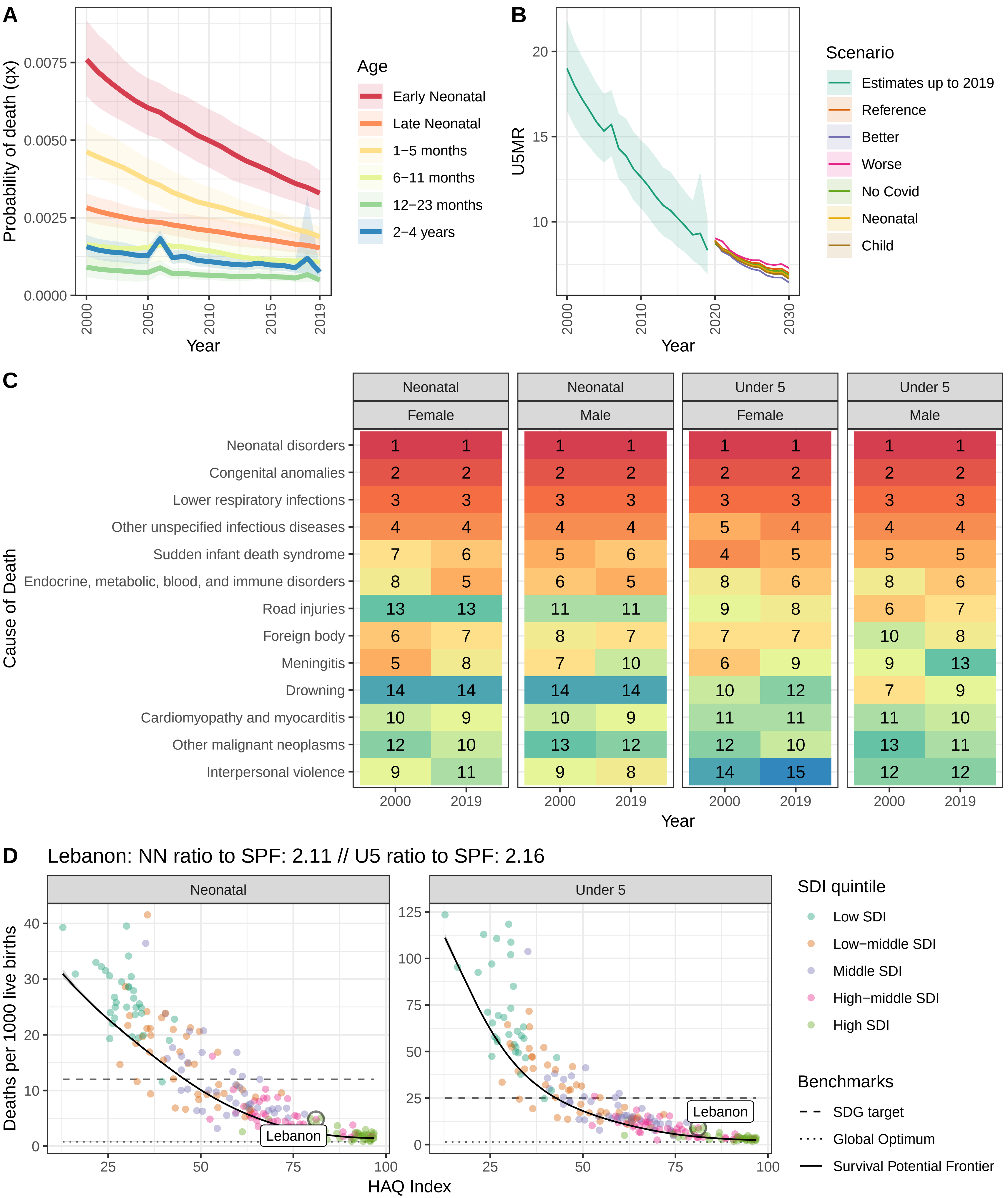
A
Probability of death (qx)
0.0075
0.0050
0.0025
0.0000
2000
2005
2010
2015
2019
Year
Age
Early Neonatal
Late Neonatal
1−5 months
6−11 months
12−23 months
2−4 years
B
U5MR
20
15
10
2000
2010
2020
2030
Year
Scenario
Estimates up to 2019
Reference
Better
Worse
No Covid
Neonatal
Child
C
Neonatal
Female
Neonatal
Male
Under 5
Female
Under 5
Male
Cause of Death
Neonatal disorders 1 1 1 1 1 1 1 1
Congenital anomalies 2 2 2 2 2 2 2 2
Lower respiratory infections 3 3 3 3 3 3 3 3
Other unspecified infectious diseases 4 4 4 4 5 4 4 4
Sudden infant death syndrome 7 6 5 6 4 5 5 5
Endocrine, metabolic, blood, and immune disorders 8 5 6 5 8 6 8 6
Road injuries 13 13 11 11 9 8 6 7
Foreign body 6 7 8 7 7 7 10 8
Meningitis 5 8 7 10 6 9 9 13
Drowning 14 14 14 14 10 12 7 9
Cardiomyopathy and myocarditis 10 9 10 9 11 11 11 10
Other malignant neoplasms 12 10 13 12 12 10 13 11
Interpersonal violence 9 11 9 8 14 15 12 12
2000
2019
Year
D
Lebanon: NN ratio to SPF: 2.11 // U5 ratio to SPF: 2.16
Neonatal
Under 5
Deaths per 1000 live births
40
30
20
10
0
125
100
75
50
25
0
25
50
75
100
HAQ Index
Lebanon
SDI quintile
Low SDI
Low−middle SDI
Middle SDI
High−middle SDI
High SDI
Benchmarks
SDG target
Global Optimum
Survival Potential Frontier

Libya

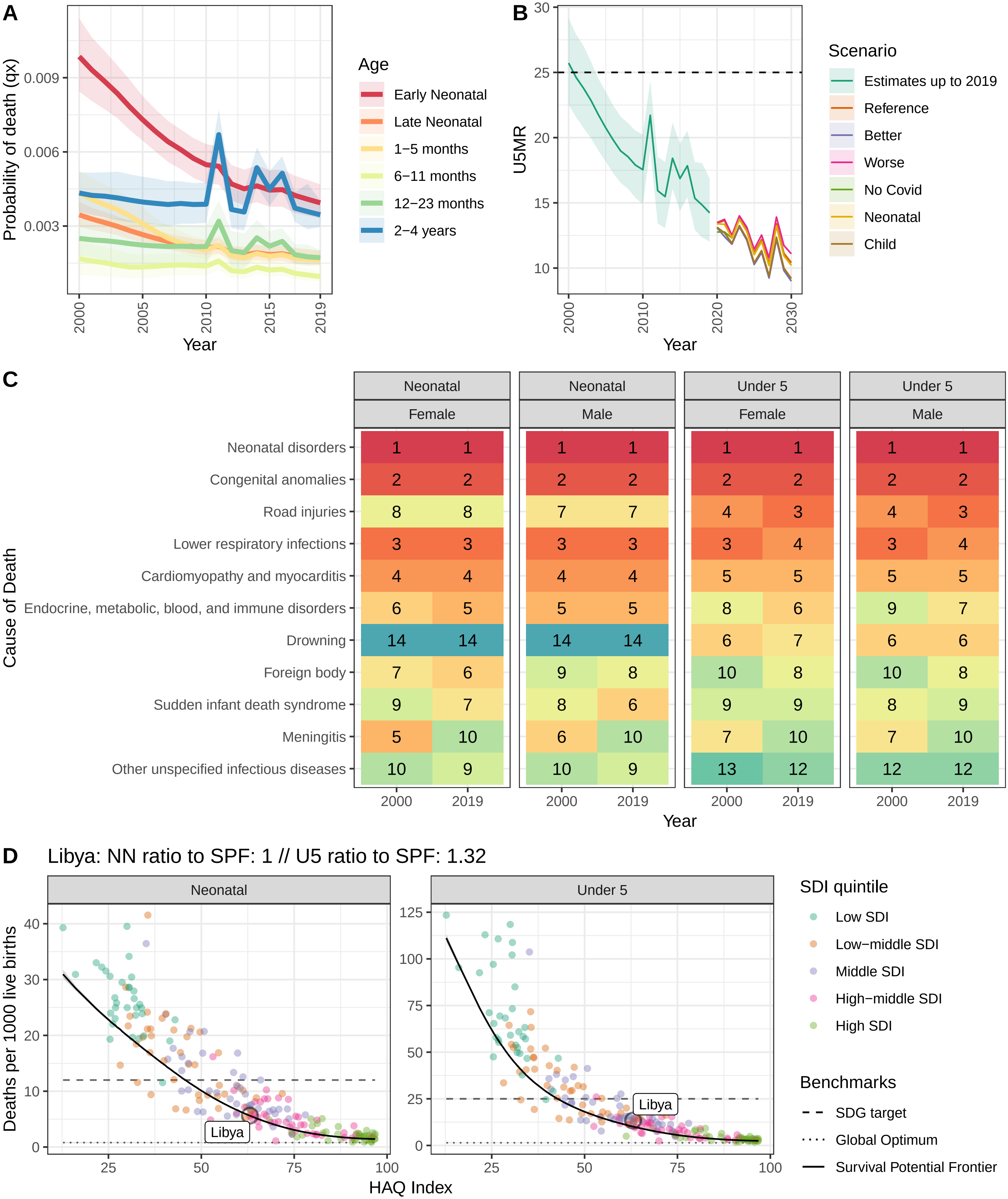

A
Probability of death (qx)
0.009
0.006
0.003
2000
2005
2010
2015
2019
Year
Age
Early Neonatal
Late Neonatal
1−5 months
6−11 months
12−23 months
2−4 years
B
U5MR
30
25
20
15
10
2000
2010
2020
2030
Year
Scenario
Estimates up to 2019
Reference
Better
Worse
No Covid
Neonatal
Child
C
Neonatal Female
Neonatal Male
Under 5 Female
Under 5 Male
Cause of Death
Neonatal disorders 1 1 1 1 1 1 1 1
Congenital anomalies 2 2 2 2 2 2 2 2
Road injuries 8 8 7 7 4 3 4 3
Lower respiratory infections 3 3 3 3 3 4 3 4
Cardiomyopathy and myocarditis 4 4 4 4 5 5 5 5
Endocrine, metabolic, blood, and immune disorders 6 5 5 5 8 6 9 7
Drowning 14 14 14 14 6 7 6 6
Foreign body 7 6 9 8 10 8 10 8
Sudden infant death syndrome 9 7 8 6 9 9 8 9
Meningitis 5 10 6 10 7 10 7 10
Other unspecified infectious diseases 10 9 10 9 13 12 12 12
2000
2019
Year
D
Libya: NN ratio to SPF: 1 // U5 ratio to SPF: 1.32
Neonatal
Under 5
Deaths per 1000 live births
40
30
20
10
0
125
100
75
50
25
Libya
HAQ Index
SDI quintile
Low SDI
Low−middle SDI
Middle SDI
High−middle SDI
High SDI
Benchmarks
SDG target
Global Optimum
Survival Potential Frontier

Morocco

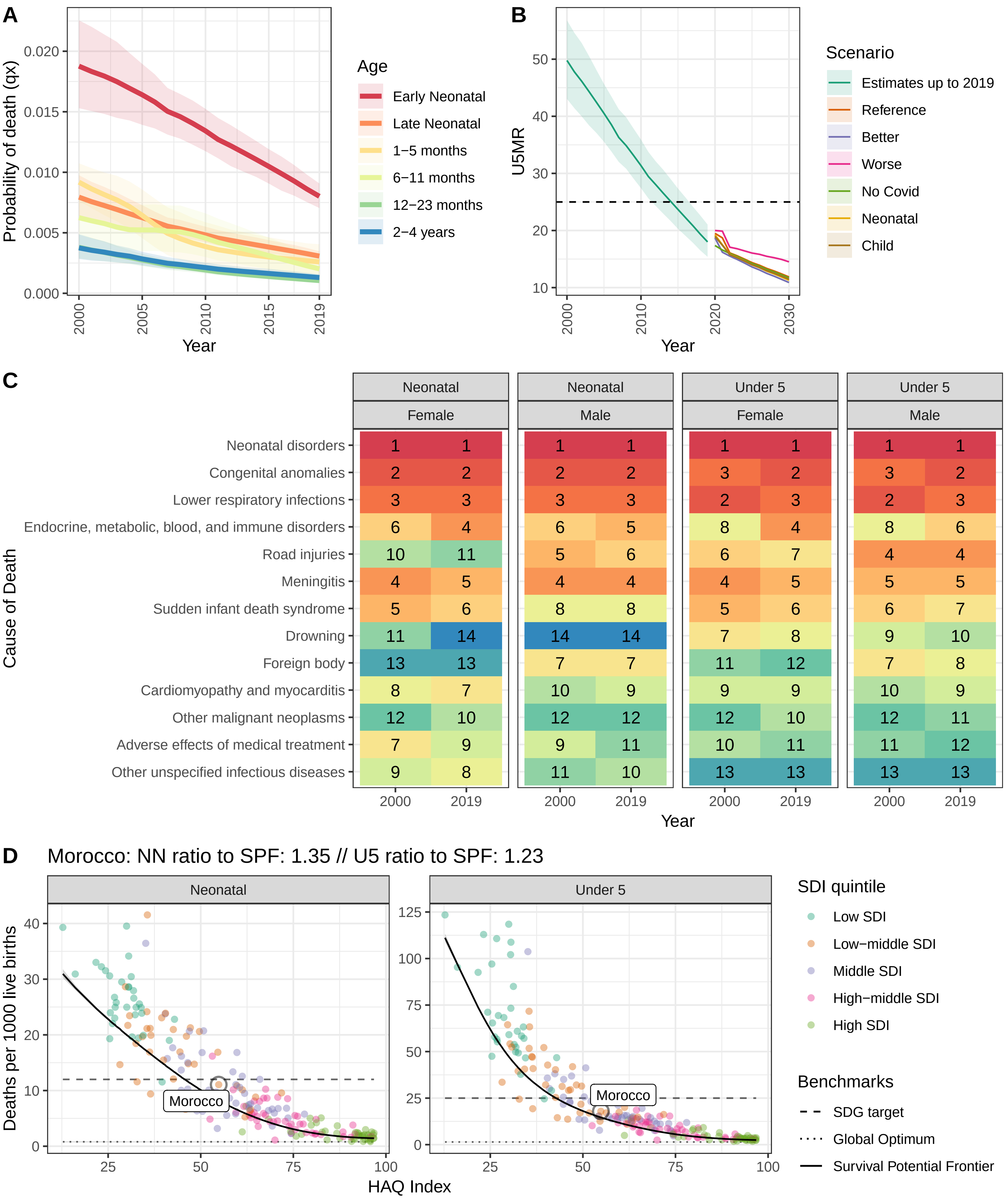
A
Probability of death (qx)
0.020
0.015
0.010
0.005
0.000
2000
2005
2010
2015
2019
Year
Age
Early Neonatal
Late Neonatal
1−5 months
6−11 months
12−23 months
2−4 years
B
U5MR
50
40
30
20
10
2000
2010
2020
2030
Year
Scenario
Estimates up to 2019
Reference
Better
Worse
No Covid
Neonatal
Child
C
Cause of Death
Neonatal
Female
Neonatal
Male
Under 5
Female
Under 5
Male
Neonatal disorders 1 1 1 1 1 1 1 1
Congenital anomalies 2 2 2 2 3 2 3 2
Lower respiratory infections 3 3 3 3 2 3 2 3
Endocrine, metabolic, blood, and immune disorders 6 4 6 5 8 4 8 6
Road injuries 10 11 5 6 6 7 4 4
Meningitis 4 5 4 4 4 5 5 5
Sudden infant death syndrome 5 6 8 8 5 6 6 7
Drowning 11 14 14 14 7 8 9 10
Foreign body 13 13 7 7 11 12 7 8
Cardiomyopathy and myocarditis 8 7 10 9 9 9 10 9
Other malignant neoplasms 12 10 12 12 12 10 12 11
Adverse effects of medical treatment 7 9 9 11 10 11 11 12
Other unspecified infectious diseases 9 8 11 10 13 13 13 13
2000 2019 2000 2019 2000 2019 2000 2019
Year
D
Morocco: NN ratio to SPF: 1.35 // U5 ratio to SPF: 1.23
Neonatal
Under 5
Deaths per 1000 live births
40
30
20
10
0
125
100
75
50
25
0
Morocco
Morocco
25
50
75
100
HAQ Index
SDI quintile
Low SDI
Low−middle SDI
Middle SDI
High−middle SDI
High SDI
Benchmarks
SDG target
Global Optimum
Survival Potential Frontier

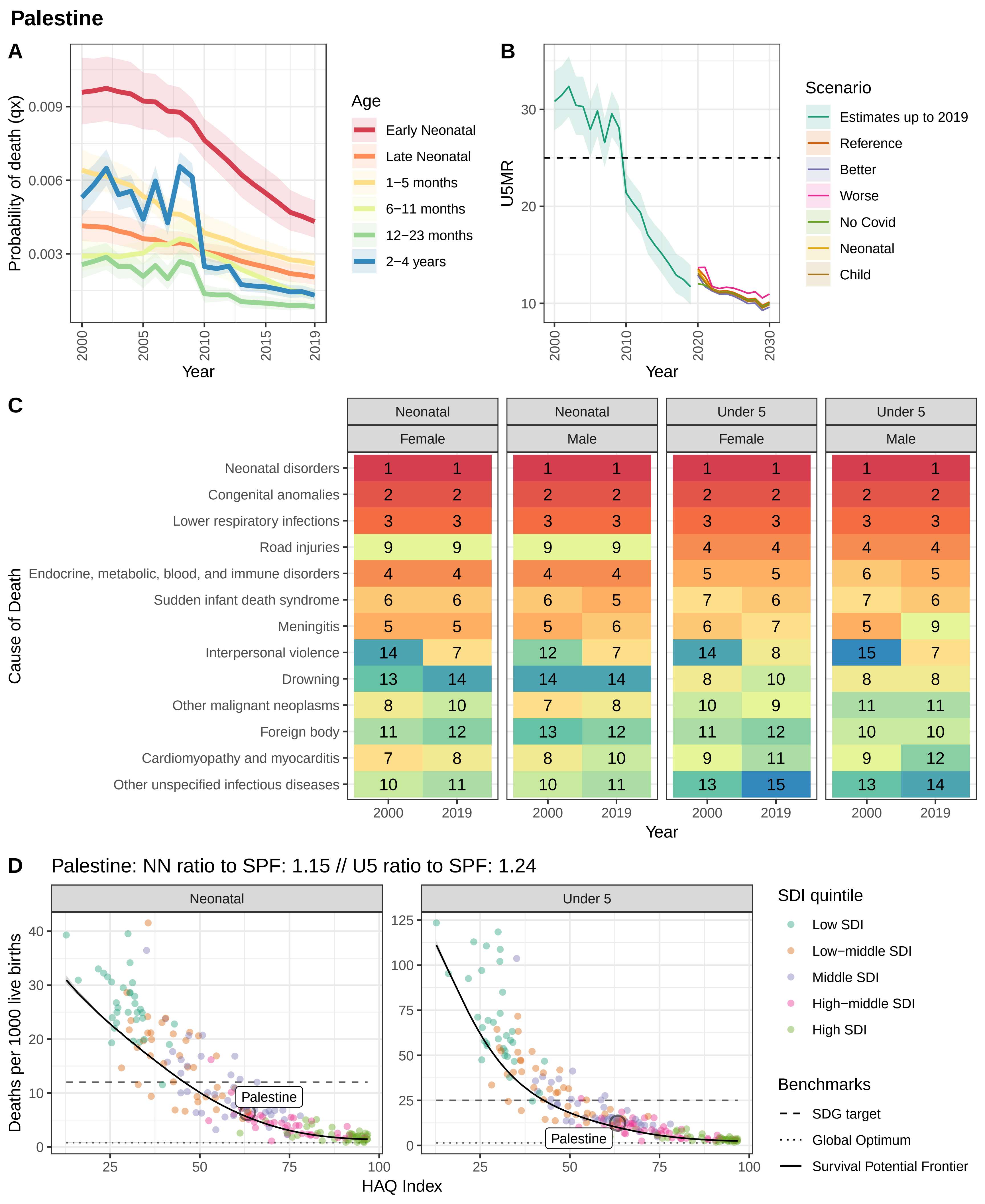
Palestine
A
Probability of death (qx)
0.009
0.006
0.003
2000
2005
2010
2015
2019
Year
Age
Early Neonatal
Late Neonatal
1−5 months
6−11 months
12−23 months
2−4 years
B
U5MR
30
20
10
2000
2010
2020
2030
Year
Scenario
Estimates up to 2019
Reference
Better
Worse
No Covid
Neonatal
Child
C
Neonatal Female
Neonatal Male
Under 5 Female
Under 5 Male
Cause of Death
Neonatal disorders 1 1 1 1 1 1 1 1
Congenital anomalies 2 2 2 2 2 2 2 2
Lower respiratory infections 3 3 3 3 3 3 3 3
Road injuries 9 9 9 9 4 4 4 4
Endocrine, metabolic, blood, and immune disorders 4 4 4 4 5 5 6 5
Sudden infant death syndrome 6 6 6 5 7 6 7 6
Meningitis 5 5 5 6 6 7 5 9
Interpersonal violence 14 7 12 7 14 8 15 7
Drowning 13 14 14 14 8 10 8 8
Other malignant neoplasms 8 10 7 8 10 9 11 11
Foreign body 11 12 13 12 11 12 10 10
Cardiomyopathy and myocarditis 7 8 8 10 9 11 9 12
Other unspecified infectious diseases 10 11 10 11 13 15 13 14
2000 2019
Year
D
Palestine: NN ratio to SPF: 1.15 // U5 ratio to SPF: 1.24
Neonatal
Under 5
Deaths per 1000 live births
Palestine
HAQ Index
SDI quintile
Low SDI
Low−middle SDI
Middle SDI
High−middle SDI
High SDI
Benchmarks
SDG target
Global Optimum
Survival Potential Frontier

Myanmar

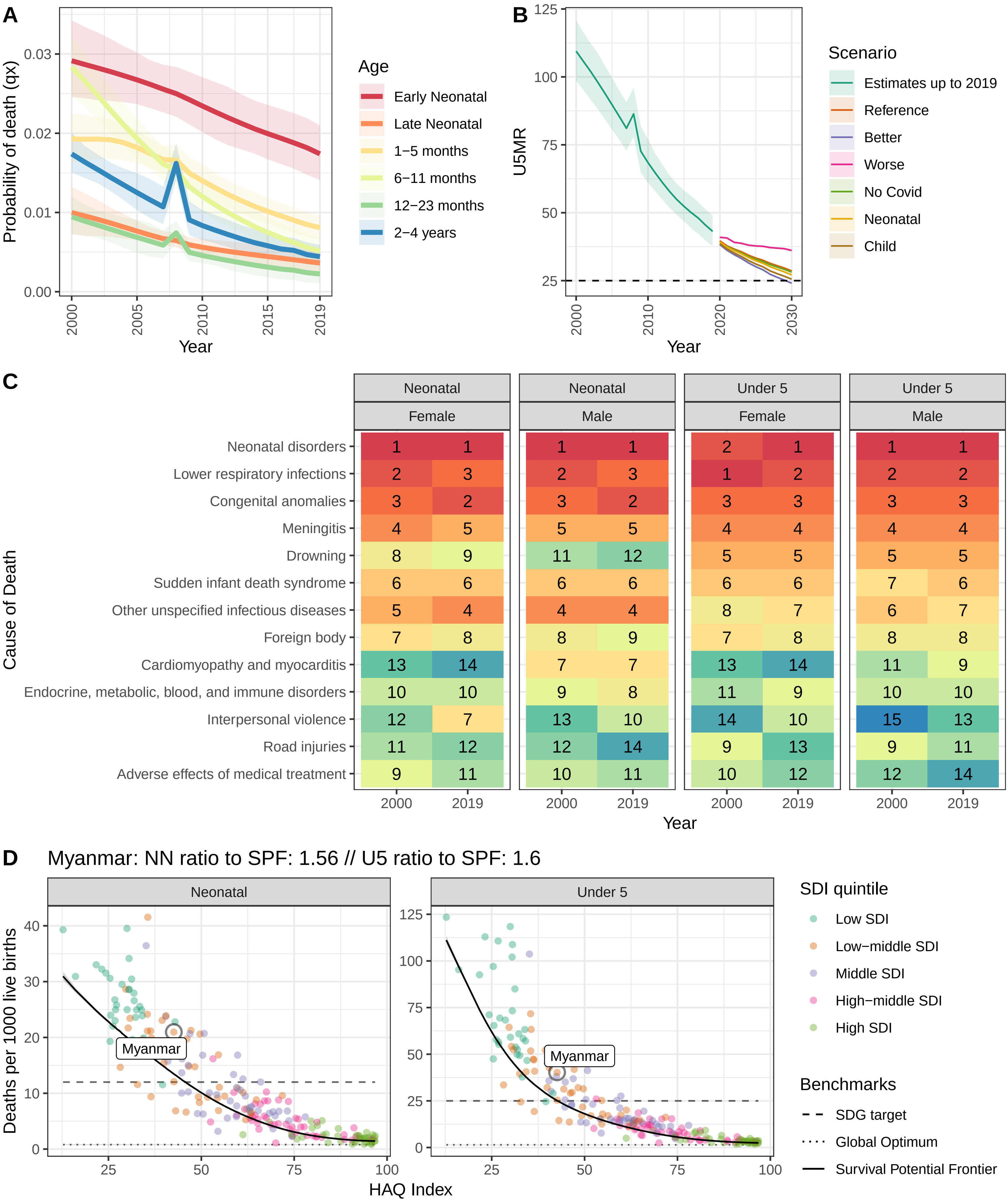
A
Probability of death (qx)
0.03
0.02
0.01
0.00
2000
2005
2010
2015
2019
Year
Age
Early Neonatal
Late Neonatal
1−5 months
6−11 months
12−23 months
2−4 years
B
U5MR
125
100
75
50
25
2000
2010
2020
2030
Year
Scenario
Estimates up to 2019
Reference
Better
Worse
No Covid
Neonatal
Child
C
Neonatal Female
Neonatal Male
Under 5 Female
Under 5 Male
Cause of Death
Neonatal disorders 1 1 1 1 2 1 1 1
Lower respiratory infections 2 3 2 3 1 2 2 2
Congenital anomalies 3 2 3 2 3 3 3 3
Meningitis 4 5 5 5 4 4 4 4
Drowning 8 9 11 12 5 5 5 5
Sudden infant death syndrome 6 6 6 6 6 6 7 6
Other unspecified infectious diseases 5 4 4 4 8 7 6 7
Foreign body 7 8 8 9 7 8 8 8
Cardiomyopathy and myocarditis 13 14 7 7 13 14 11 9
Endocrine, metabolic, blood, and immune disorders 10 10 9 8 11 9 10 10
Interpersonal violence 12 7 13 10 14 10 15 13
Road injuries 11 12 12 14 9 13 9 11
Adverse effects of medical treatment 9 11 10 11 10 12 12 14
2000
2019
Year
D
Myanmar: NN ratio to SPF: 1.56 // U5 ratio to SPF: 1.6
Neonatal
Under 5
Deaths per 1000 live births
Myanmar
HAQ Index
SDI quintile
Low SDI
Low−middle SDI
Middle SDI
High−middle SDI
High SDI
Benchmarks
SDG target
Global Optimum
Survival Potential Frontier

Oman

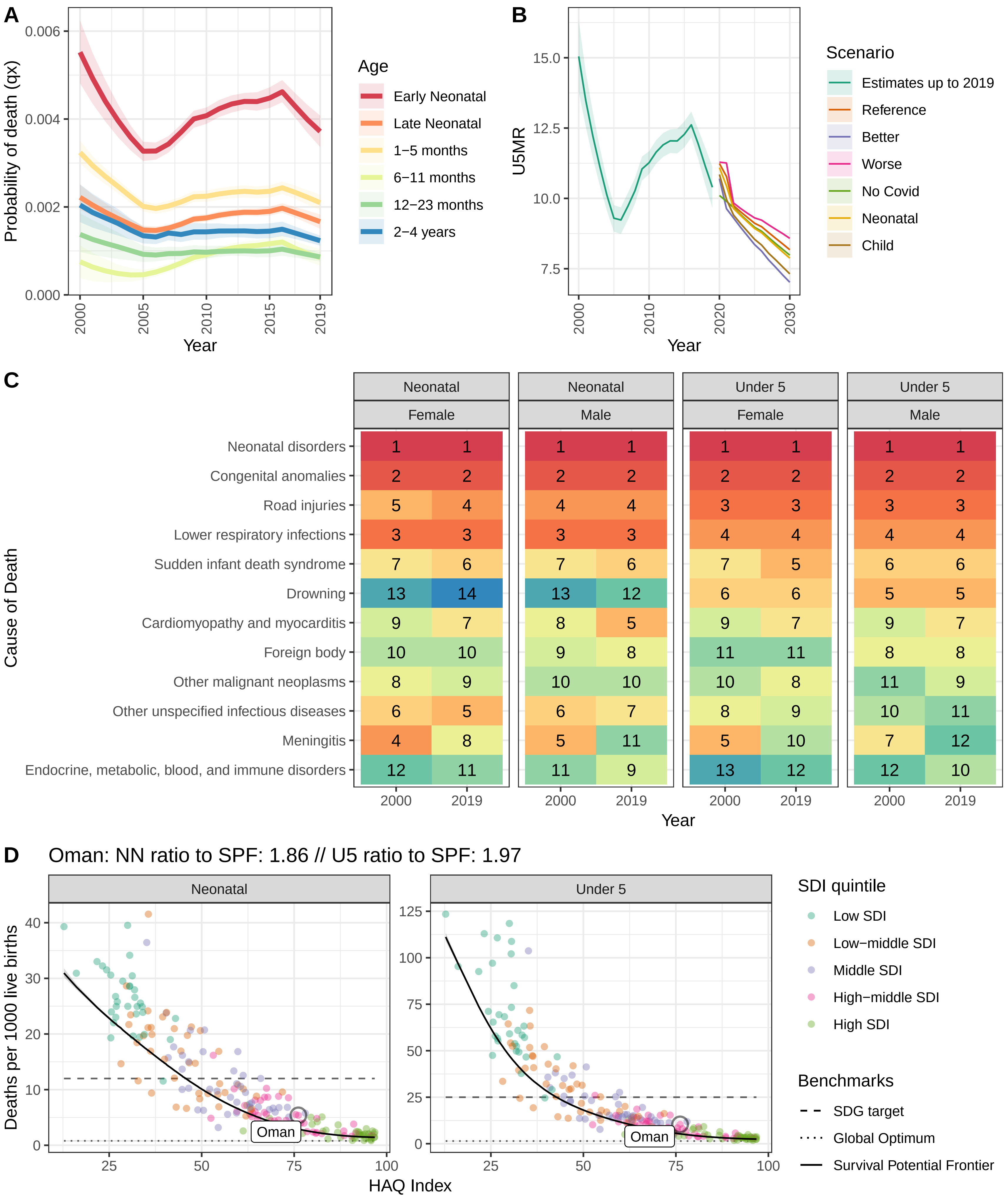
A
Probability of death (qx)
Year
Age
Early Neonatal
Late Neonatal
1−5 months
6−11 months
12−23 months
2−4 years
B
U5MR
Year
Scenario
Estimates up to 2019
Reference
Better
Worse
No Covid
Neonatal
Child
C
Neonatal Female
Neonatal Male
Under 5 Female
Under 5 Male
Cause of Death
Neonatal disorders
Congenital anomalies
Road injuries
Lower respiratory infections
Sudden infant death syndrome
Drowning
Cardiomyopathy and myocarditis
Foreign body
Other malignant neoplasms
Other unspecified infectious diseases
Meningitis
Endocrine, metabolic, blood, and immune disorders
Year
D
Oman: NN ratio to SPF: 1.86 // U5 ratio to SPF: 1.97
Neonatal
Under 5
Deaths per 1000 live births
HAQ Index
Oman
SDI quintile
Low SDI
Low−middle SDI
Middle SDI
High−middle SDI
High SDI
Benchmarks
SDG target
Global Optimum
Survival Potential Frontier

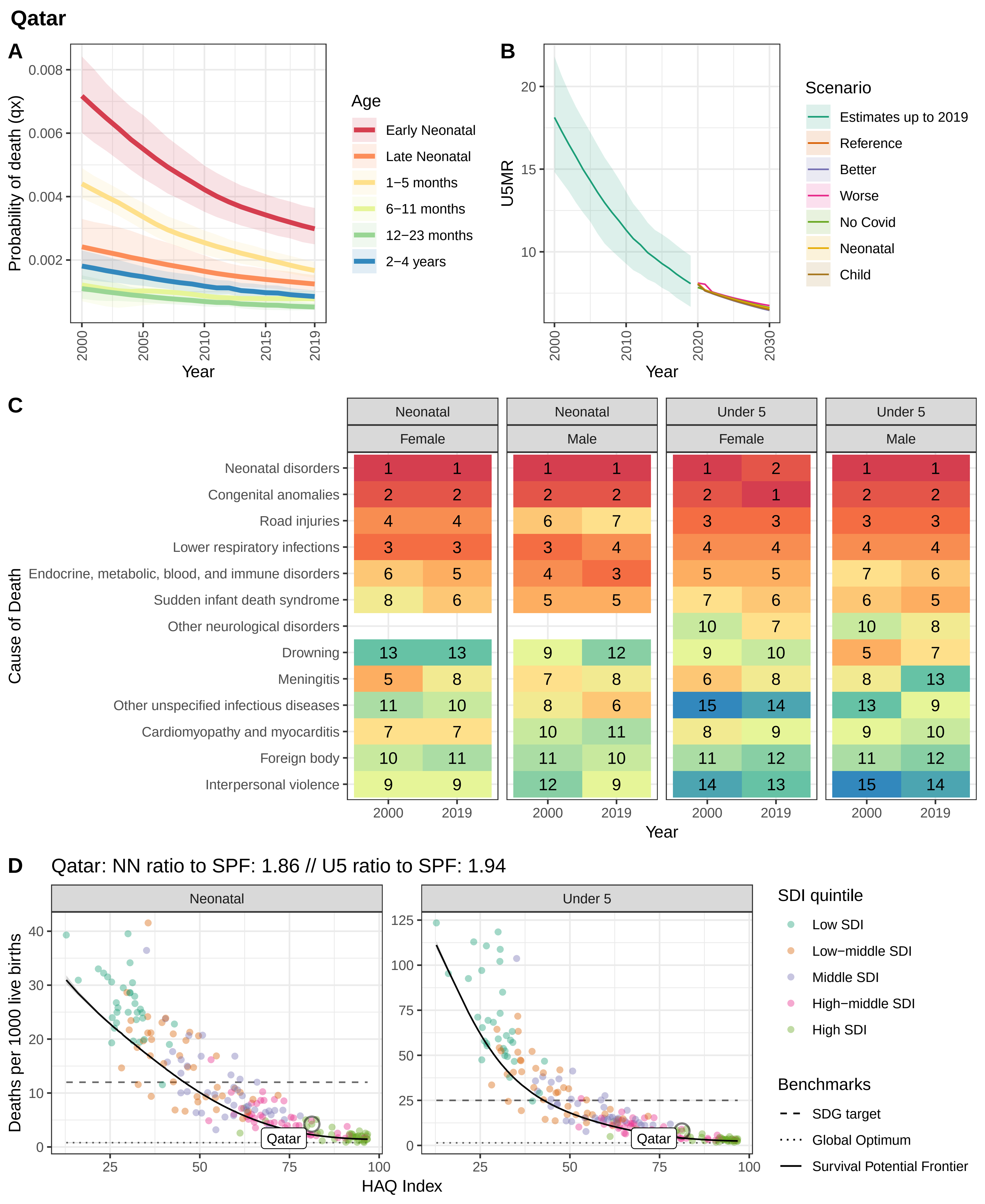
Qatar
A
Probability of death (qx)
0.008
0.006
0.004
0.002
2000
2005
2010
2015
2019
Year
Age
Early Neonatal
Late Neonatal
1−5 months
6−11 months
12−23 months
2−4 years
B
U5MR
20
15
10
2000
2010
2020
2030
Year
Scenario
Estimates up to 2019
Reference
Better
Worse
No Covid
Neonatal
Child
C
Cause of Death
Neonatal Female
Neonatal Male
Under 5 Female
Under 5 Male
Neonatal disorders 1 1 1 1 1 2 1 1
Congenital anomalies 2 2 2 2 2 1 2 2
Road injuries 4 4 6 7 3 3 3 3
Lower respiratory infections 3 3 3 4 4 4 4 4
Endocrine, metabolic, blood, and immune disorders 6 5 4 3 5 5 7 6
Sudden infant death syndrome 8 6 5 5 7 6 6 5
Other neurological disorders 10 7 10 8
Drowning 13 13 9 12 9 10 5 7
Meningitis 5 8 7 8 6 8 8 13
Other unspecified infectious diseases 11 10 8 6 15 14 13 9
Cardiomyopathy and myocarditis 7 7 10 11 8 9 9 10
Foreign body 10 11 11 10 11 12 11 12
Interpersonal violence 9 9 12 9 14 13 15 14
2000
2019
Year
D
Qatar: NN ratio to SPF: 1.86 // U5 ratio to SPF: 1.94
Neonatal
Under 5
Deaths per 1000 live births
40
30
20
10
0
125
100
75
50
25
0
Qatar
25
50
75
100
HAQ Index
SDI quintile
Low SDI
Low−middle SDI
Middle SDI
High−middle SDI
High SDI
Benchmarks
SDG target
Global Optimum
Survival Potential Frontier

Saudi Arabia

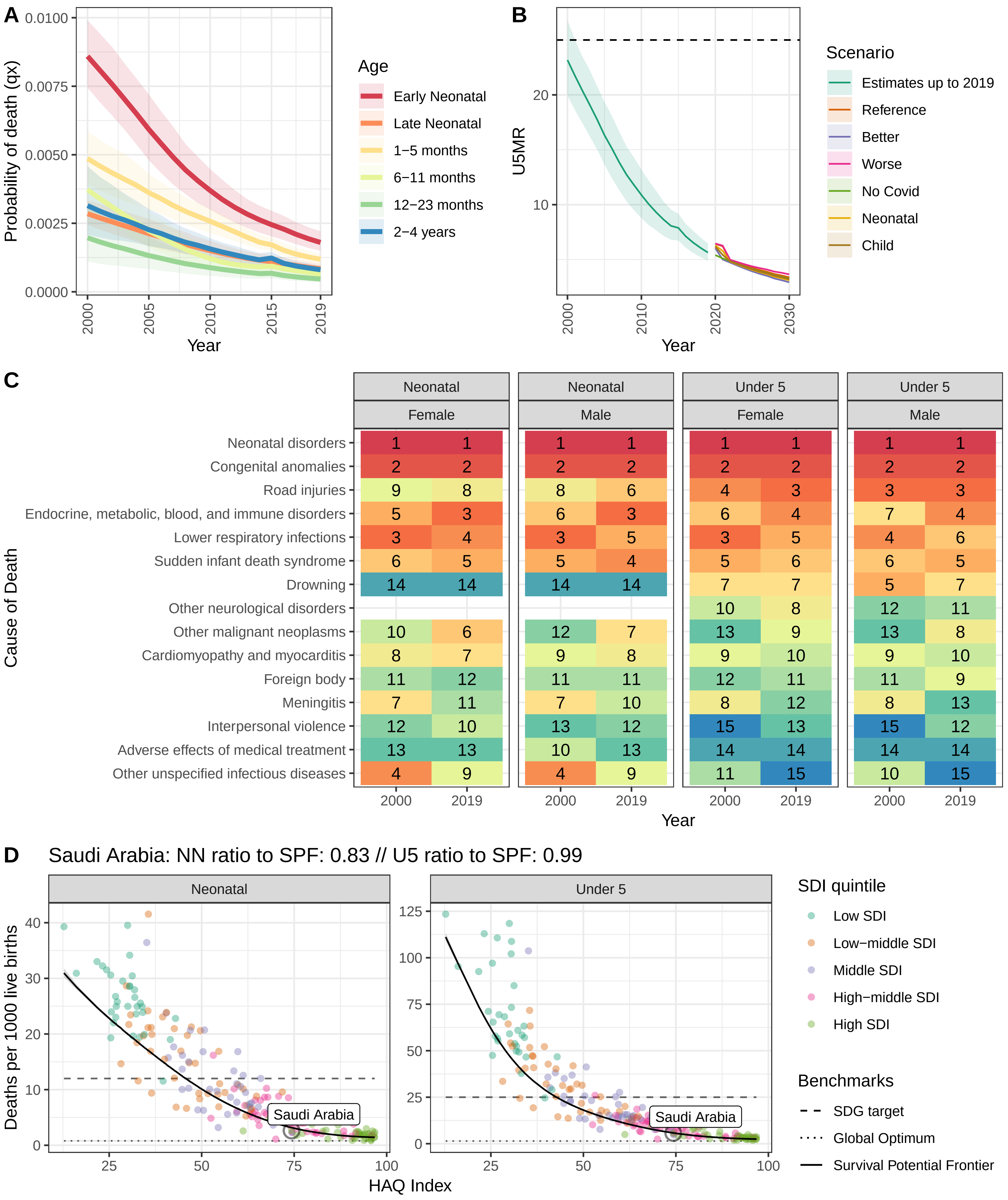

Syrian Arab Republic

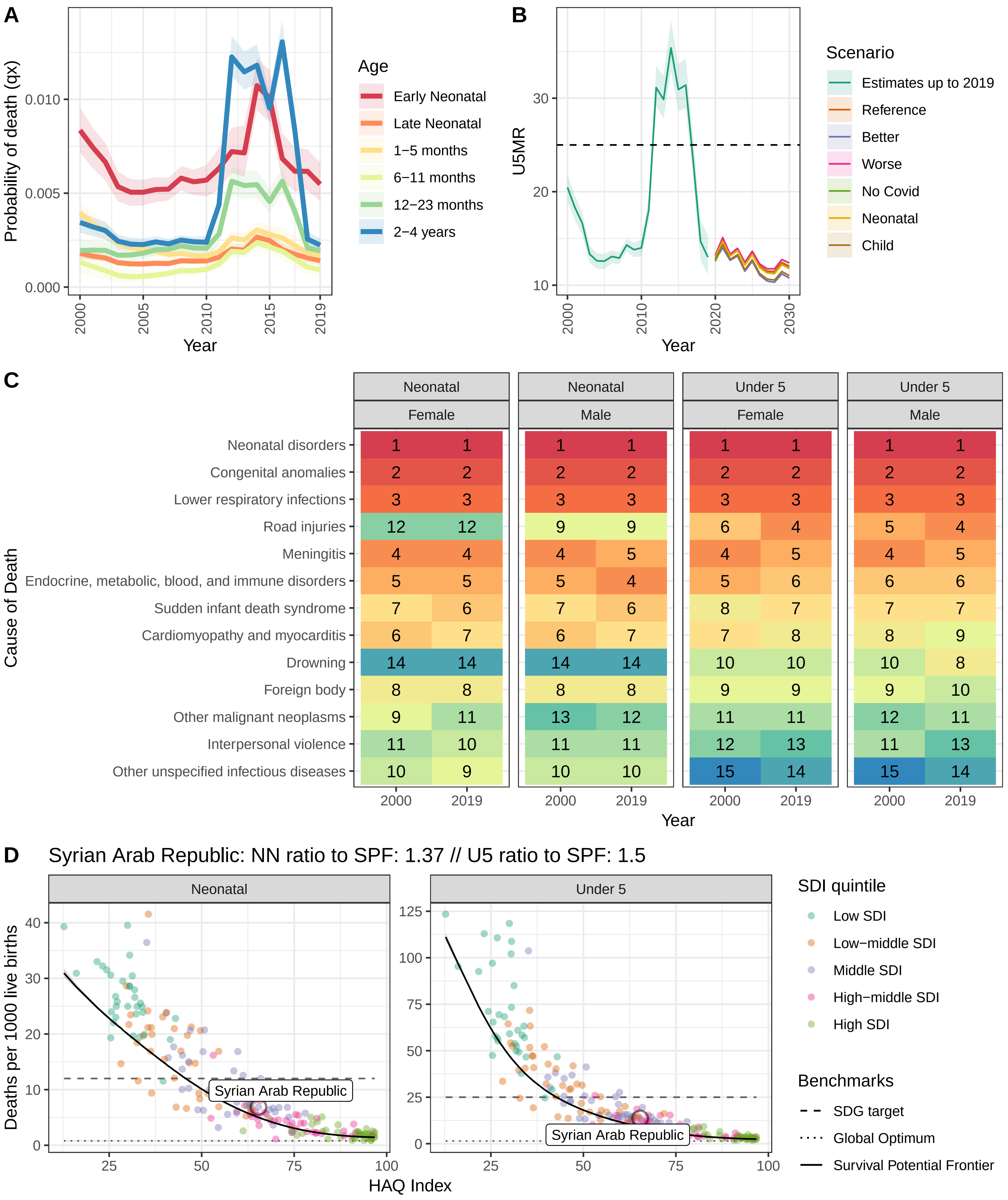

Tunisia

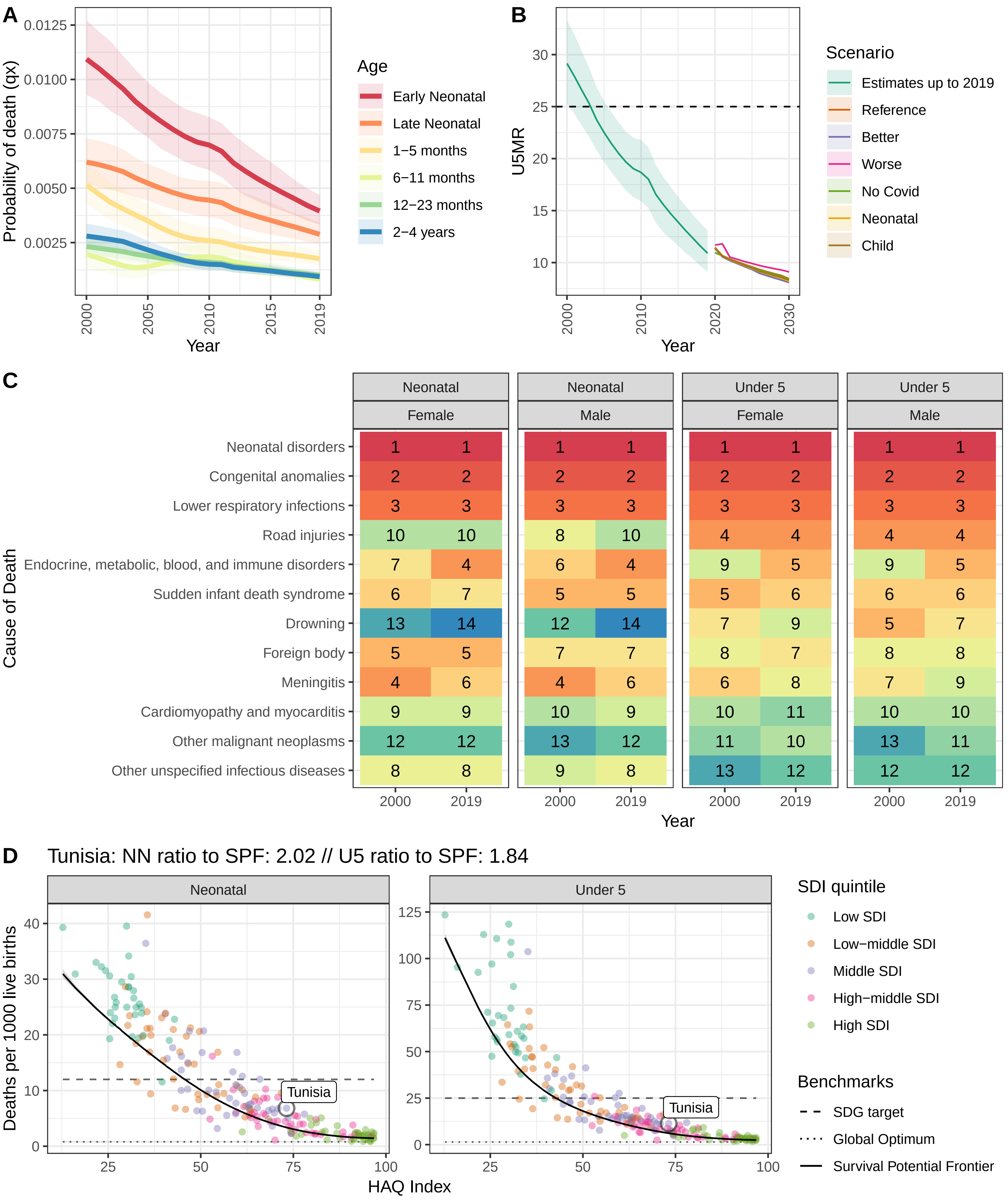
A
Probability of death (qx)
0.0125
0.0100
0.0075
0.0050
0.0025
2000
2005
2010
2015
2019
Year
Age
Early Neonatal
Late Neonatal
1−5 months
6−11 months
12−23 months
2−4 years
B
U5MR
30
25
20
15
10
2000
2010
2020
2030
Year
Scenario
Estimates up to 2019
Reference
Better
Worse
No Covid
Neonatal
Child
C
Neonatal
Female
Neonatal
Male
Under 5
Female
Under 5
Male
Cause of Death
Neonatal disorders 1 1 1 1 1 1 1 1
Congenital anomalies 2 2 2 2 2 2 2 2
Lower respiratory infections 3 3 3 3 3 3 3 3
Road injuries 10 10 8 10 4 4 4 4
Endocrine, metabolic, blood, and immune disorders 7 4 6 4 9 5 9 5
Sudden infant death syndrome 6 7 5 5 5 6 6 6
Drowning 13 14 12 14 7 9 5 7
Foreign body 5 5 7 7 8 7 8 8
Meningitis 4 6 4 6 6 8 7 9
Cardiomyopathy and myocarditis 9 9 10 9 10 11 10 10
Other malignant neoplasms 12 12 13 12 11 10 13 11
Other unspecified infectious diseases 8 8 9 8 13 12 12 12
2000
2019
Year
D
Tunisia: NN ratio to SPF: 2.02 // U5 ratio to SPF: 1.84
Neonatal
Under 5
Deaths per 1000 live births
40
30
20
10
0
125
100
75
50
25
0
Tunisia
Tunisia
25
50
75
100
HAQ Index
SDI quintile
Low SDI
Low−middle SDI
Middle SDI
High−middle SDI
High SDI
Benchmarks
SDG target
Global Optimum
Survival Potential Frontier

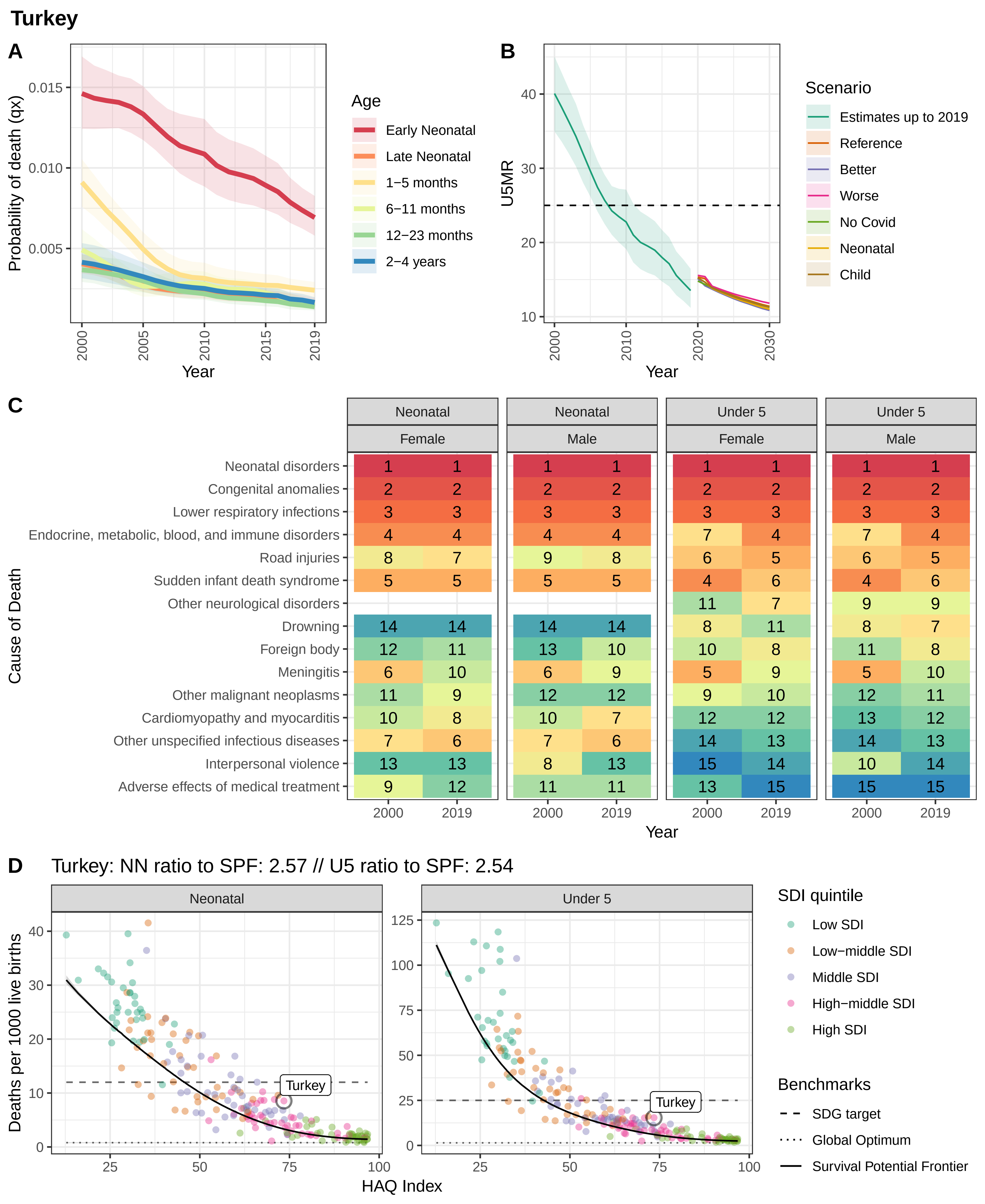
Turkey
A
Probability of death (qx)
0.015
0.010
0.005
2000
2005
2010
2015
2019
Year
Age
Early Neonatal
Late Neonatal
1−5 months
6−11 months
12−23 months
2−4 years
B
U5MR
40
30
20
10
2000
2010
2020
2030
Year
Scenario
Estimates up to 2019
Reference
Better
Worse
No Covid
Neonatal
Child
C
Neonatal Female
Neonatal Male
Under 5 Female
Under 5 Male
Cause of Death
Neonatal disorders 1 1 1 1 1 1 1 1
Congenital anomalies 2 2 2 2 2 2 2 2
Lower respiratory infections 3 3 3 3 3 3 3 3
Endocrine, metabolic, blood, and immune disorders 4 4 4 4 7 4 7 4
Road injuries 8 7 9 8 6 5 6 5
Sudden infant death syndrome 5 5 5 5 4 6 4 6
Other neurological disorders 11 7 9 9
Drowning 14 14 14 14 8 11 8 7
Foreign body 12 11 13 10 10 8 11 8
Meningitis 6 10 6 9 5 9 5 10
Other malignant neoplasms 11 9 12 12 9 10 12 11
Cardiomyopathy and myocarditis 10 8 10 7 12 12 13 12
Other unspecified infectious diseases 7 6 7 6 14 13 14 13
Interpersonal violence 13 13 8 13 15 14 10 14
Adverse effects of medical treatment 9 12 11 11 13 15 15 15
2000 2019 2000 2019 2000 2019 2000 2019
Year
D
Turkey: NN ratio to SPF: 2.57 // U5 ratio to SPF: 2.54
Neonatal
Under 5
Deaths per 1000 live births
40
30
20
10
0
125
100
75
50
25
0
Turkey
25
50
75
100
HAQ Index
SDI quintile
Low SDI
Low−middle SDI
Middle SDI
High−middle SDI
High SDI
Benchmarks
SDG target
Global Optimum
Survival Potential Frontier

United Arab Emirates

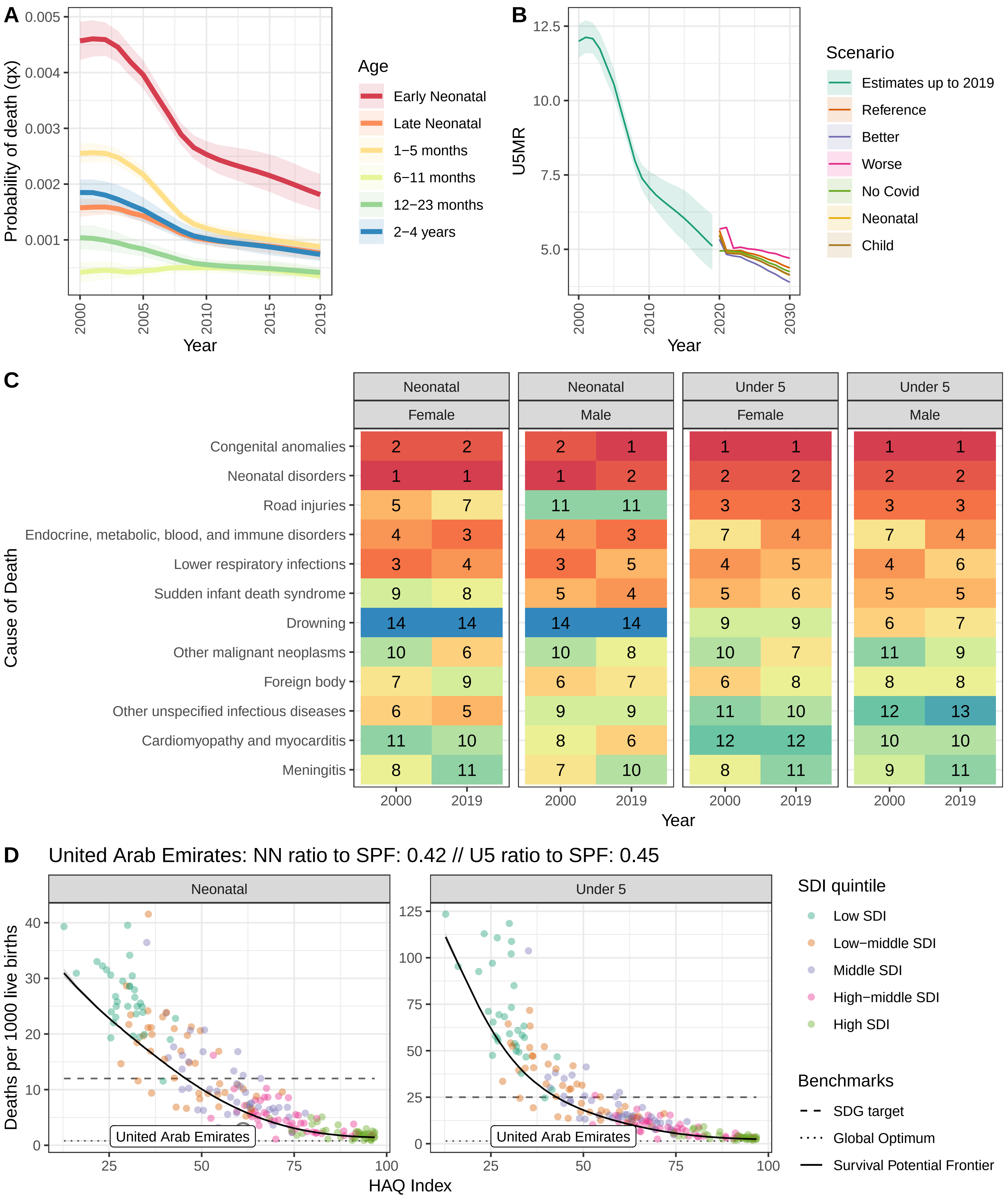

Yemen

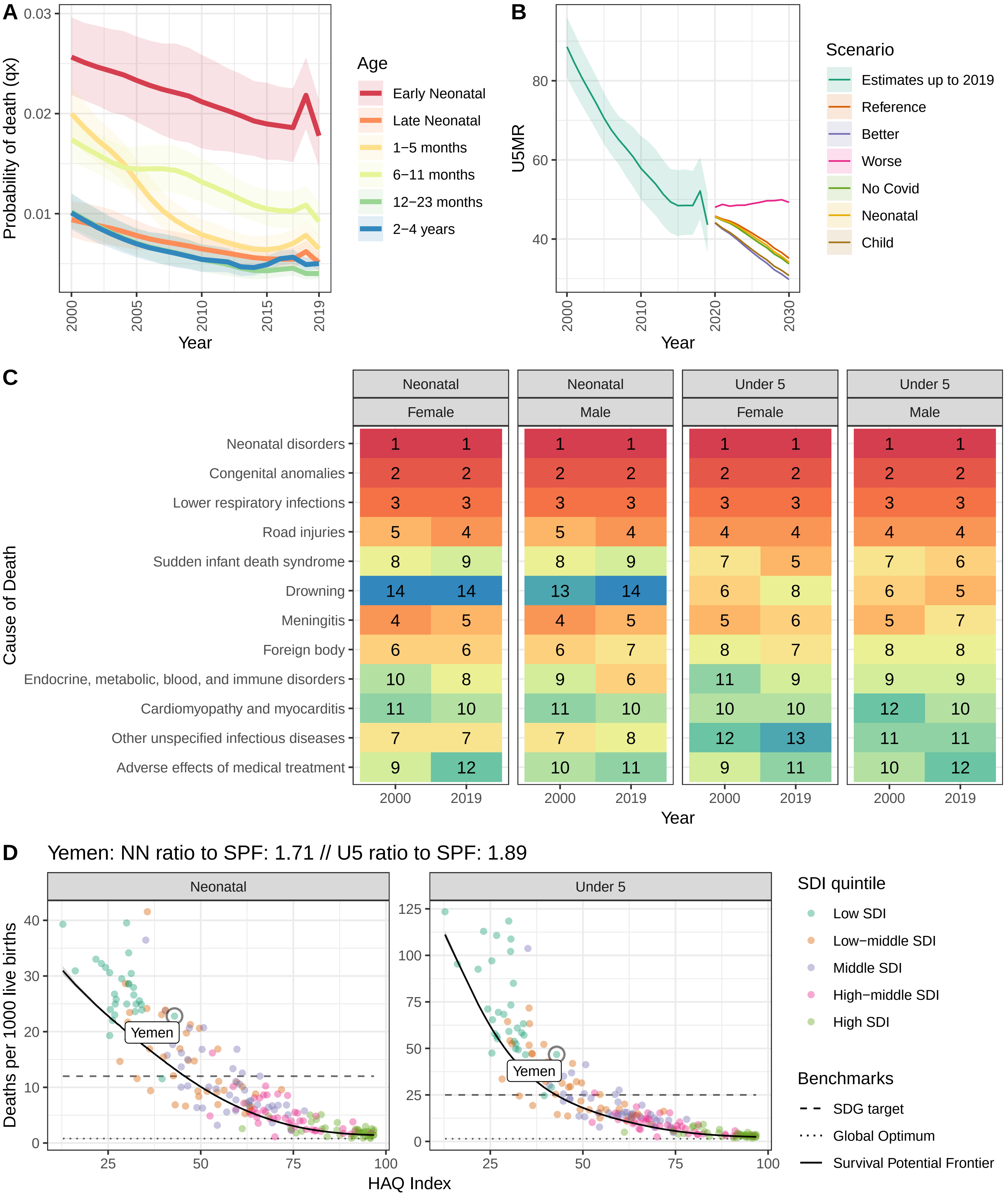

A
Probability of death (qx)
Year
Age
Early Neonatal
Late Neonatal
1−5 months
6−11 months
12−23 months
2−4 years
B
U5MR
Year
Scenario
Estimates up to 2019
Reference
Better
Worse
No Covid
Neonatal
Child
C
Cause of Death
Neonatal Female
Neonatal Male
Under 5 Female
Under 5 Male
Neonatal disorders 1 1 1 1 1 1 1 1
Congenital anomalies 2 2 2 2 2 2 2 2
Lower respiratory infections 3 3 3 3 3 3 3 3
Road injuries 5 4 5 4 4 4 4 4
Sudden infant death syndrome 8 9 8 9 7 5 7 6
Drowning 14 14 13 14 6 8 6 5
Meningitis 4 5 4 5 5 6 5 7
Foreign body 6 6 6 7 8 7 8 8
Endocrine, metabolic, blood, and immune disorders 10 8 9 6 11 9 9 9
Cardiomyopathy and myocarditis 11 10 11 10 10 10 12 10
Other unspecified infectious diseases 7 7 7 8 12 13 11 11
Adverse effects of medical treatment 9 12 10 11 9 11 10 12
2000 2019
Year
D
Yemen: NN ratio to SPF: 1.71 // U5 ratio to SPF: 1.89
Neonatal
Under 5
Deaths per 1000 live births
HAQ Index
Yemen
SDI quintile
Low SDI
Low−middle SDI
Middle SDI
High−middle SDI
High SDI
Benchmarks
SDG target
Global Optimum
Survival Potential Frontier

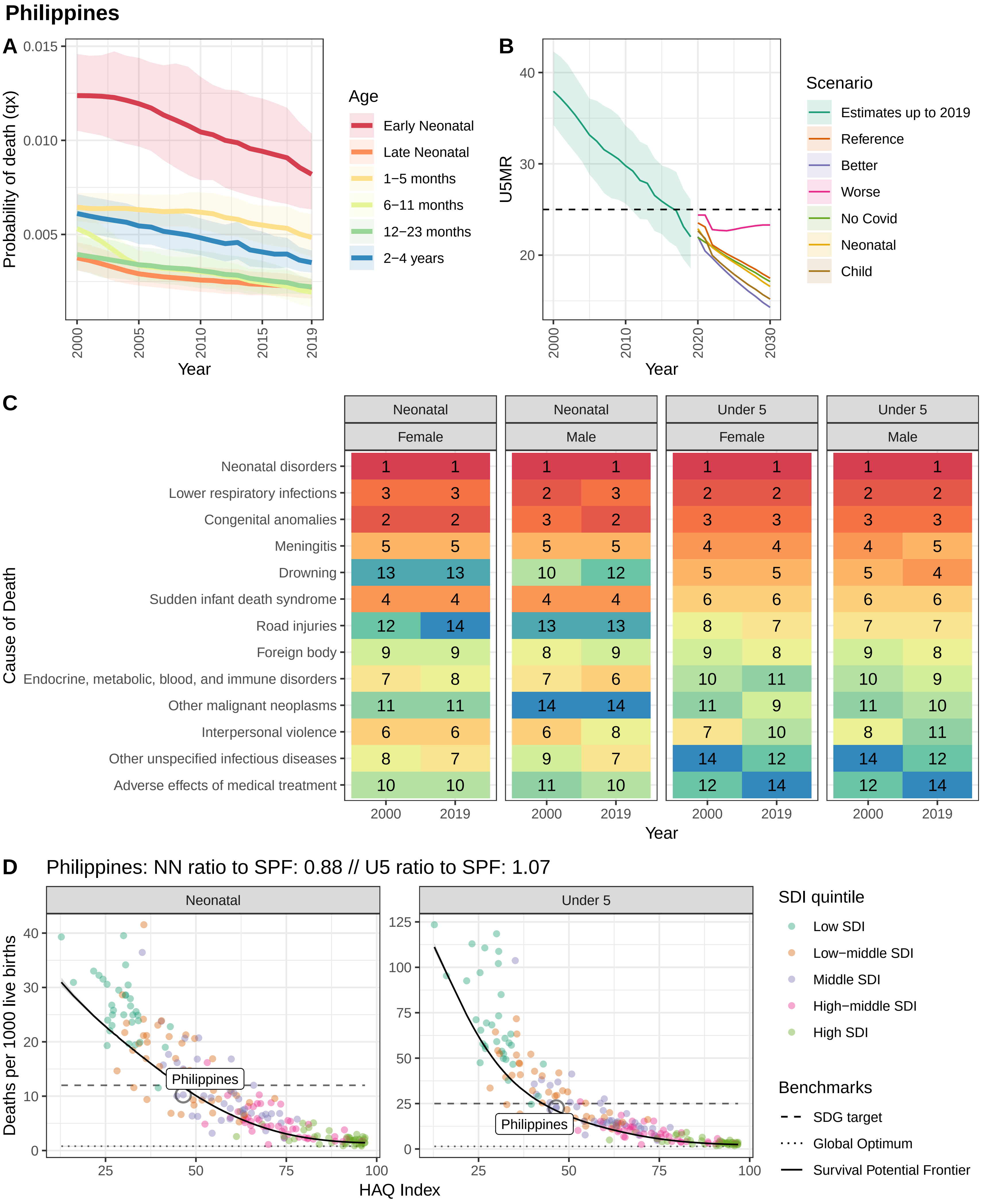
Philippines
A
Probability of death (qx)
Year
Age
Early Neonatal
Late Neonatal
1−5 months
6−11 months
12−23 months
2−4 years
B
U5MR
Year
Scenario
Estimates up to 2019
Reference
Better
Worse
No Covid
Neonatal
Child
C
Cause of Death
Neonatal
Under 5
Female
Male
Neonatal disorders
Lower respiratory infections
Congenital anomalies
Meningitis
Drowning
Sudden infant death syndrome
Road injuries
Foreign body
Endocrine, metabolic, blood, and immune disorders
Other malignant neoplasms
Interpersonal violence
Other unspecified infectious diseases
Adverse effects of medical treatment
Year
D
Philippines: NN ratio to SPF: 0.88 // U5 ratio to SPF: 1.07
Neonatal
Under 5
Deaths per 1000 live births
HAQ Index
Philippines
SDI quintile
Low SDI
Low−middle SDI
Middle SDI
High−middle SDI
High SDI
Benchmarks
SDG target
Global Optimum
Survival Potential Frontier

Afghanistan

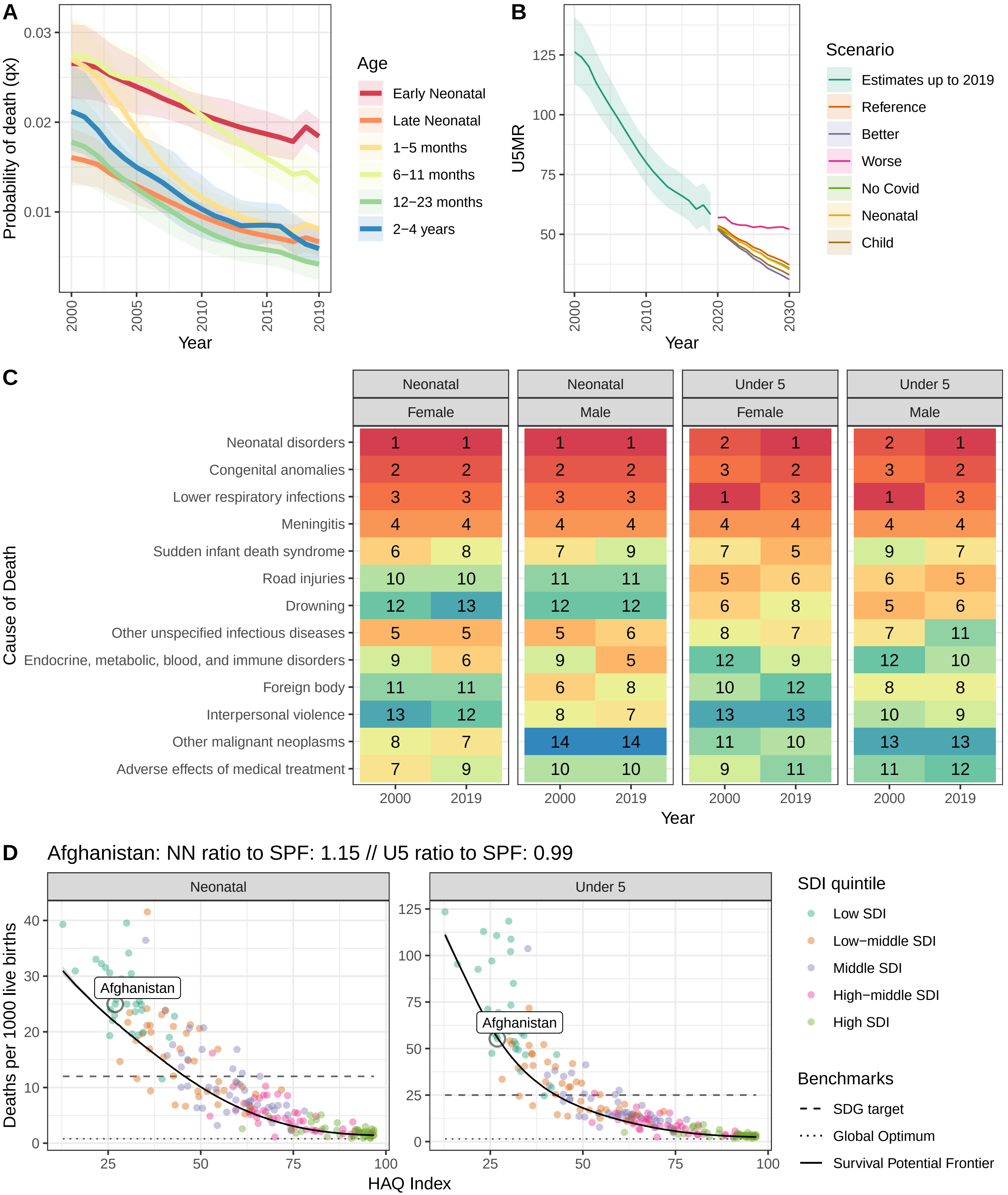

Bangladesh

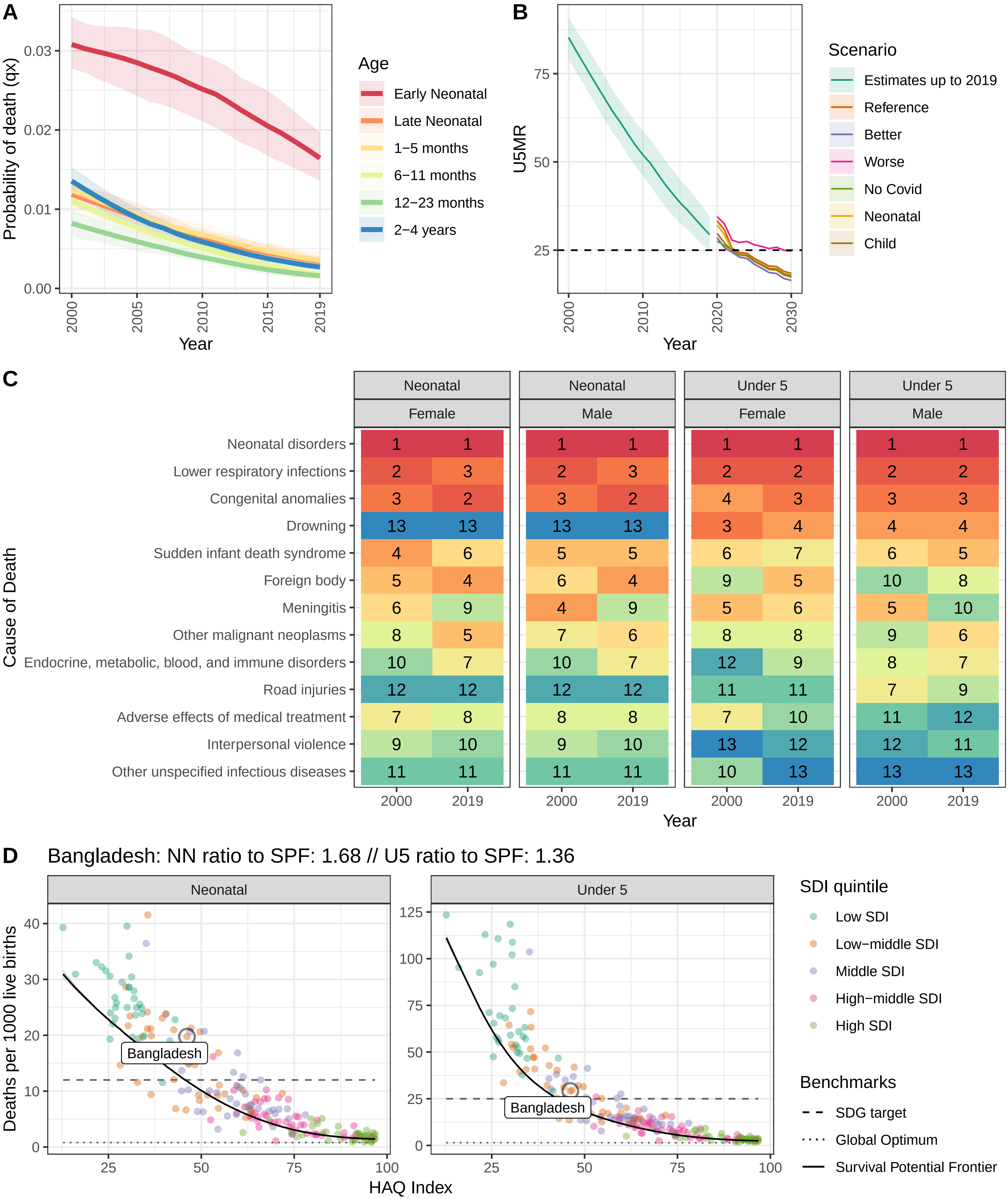

A
Probability of death (qx)
0.03
0.02
0.01
0.00
2000
2005
2010
2015
2019
Year
Age
Early Neonatal
Late Neonatal
1−5 months
6−11 months
12−23 months
2−4 years
B
U5MR
75
50
25
2000
2010
2020
2030
Year
Scenario
Estimates up to 2019
Reference
Better
Worse
No Covid
Neonatal
Child
C
Neonatal
Female
Neonatal
Male
Under 5
Female
Under 5
Male
Cause of Death
Neonatal disorders 1 1 1 1 1 1 1 1
Lower respiratory infections 2 3 2 3 2 2 2 2
Congenital anomalies 3 2 3 2 4 3 3 3
Drowning 13 13 13 13 3 4 4 4
Sudden infant death syndrome 4 6 5 5 6 7 6 5
Foreign body 5 4 6 4 9 5 10 8
Meningitis 6 9 4 9 5 6 5 10
Other malignant neoplasms 8 5 7 6 8 8 9 6
Endocrine, metabolic, blood, and immune disorders 10 7 10 7 12 9 8 7
Road injuries 12 12 12 12 11 11 7 9
Adverse effects of medical treatment 7 8 8 8 7 10 11 12
Interpersonal violence 9 10 9 10 13 12 12 11
Other unspecified infectious diseases 11 11 11 11 10 13 13 13
2000
2019
Year
D
Bangladesh: NN ratio to SPF: 1.68 // U5 ratio to SPF: 1.36
Neonatal
Under 5
Deaths per 1000 live births
40
30
20
10
0
125
100
75
50
25
0
Bangladesh
25
50
75
100
HAQ Index
SDI quintile
Low SDI
Low−middle SDI
Middle SDI
High−middle SDI
High SDI
Benchmarks
SDG target
Global Optimum
Survival Potential Frontier

Bhutan

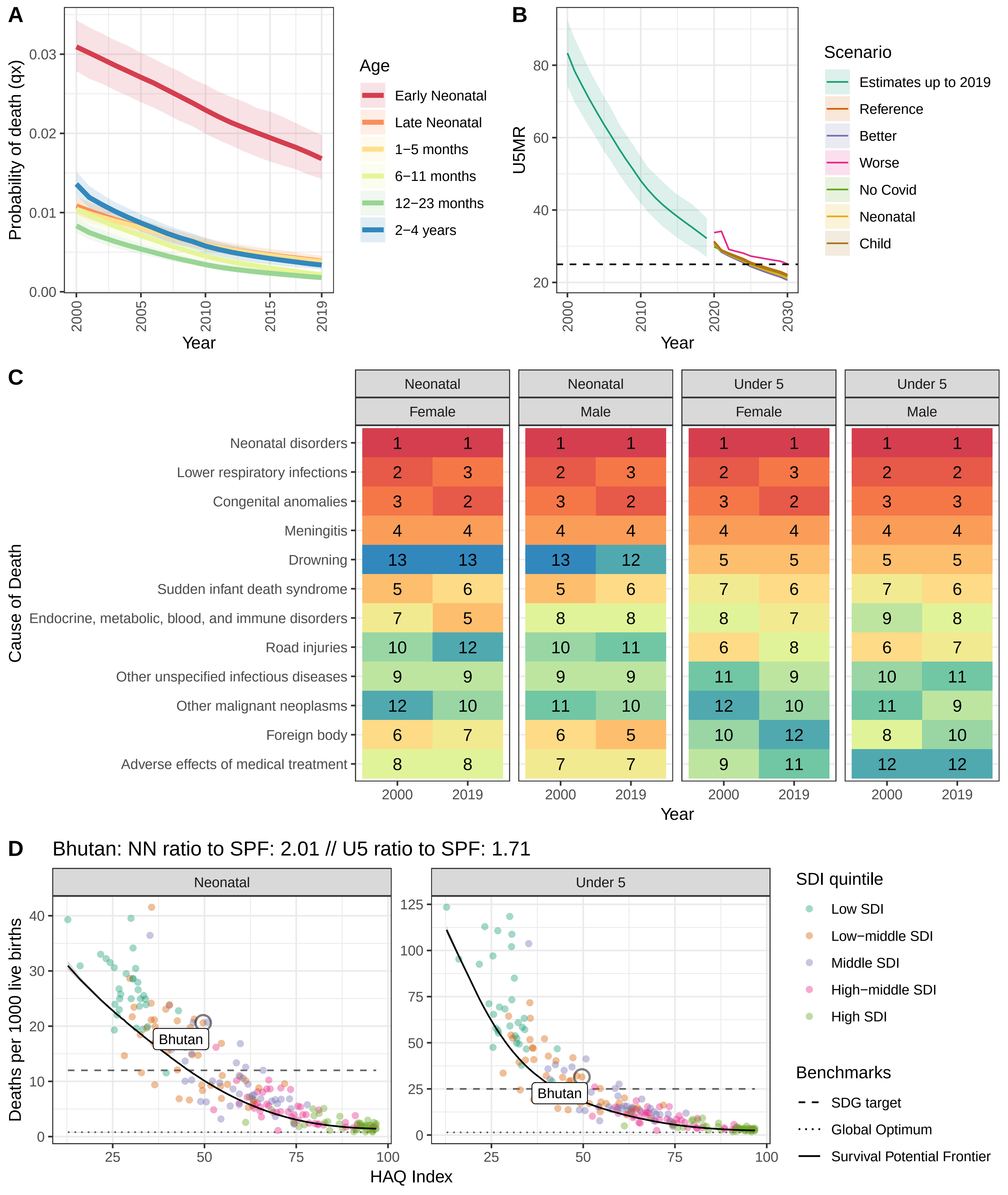
A
Probability of death (qx)
0.03
0.02
0.01
0.00
2000
2005
2010
2015
2019
Year
Age
Early Neonatal
Late Neonatal
1−5 months
6−11 months
12−23 months
2−4 years
B
U5MR
80
60
40
20
2000
2010
2020
2030
Year
Scenario
Estimates up to 2019
Reference
Better
Worse
No Covid
Neonatal
Child
C
Neonatal Female
Neonatal Male
Under 5 Female
Under 5 Male
Cause of Death
Neonatal disorders 1 1 1 1 1 1 1 1
Lower respiratory infections 2 3 2 3 2 3 2 2
Congenital anomalies 3 2 3 2 3 2 3 3
Meningitis 4 4 4 4 4 4 4 4
Drowning 13 13 13 12 5 5 5 5
Sudden infant death syndrome 5 6 5 6 7 6 7 6
Endocrine, metabolic, blood, and immune disorders 7 5 8 8 8 7 9 8
Road injuries 10 12 10 11 6 8 6 7
Other unspecified infectious diseases 9 9 9 9 11 9 10 11
Other malignant neoplasms 12 10 11 10 12 10 11 9
Foreign body 6 7 6 5 10 12 8 10
Adverse effects of medical treatment 8 8 7 7 9 11 12 12
2000 2019 2000 2019 2000 2019 2000 2019
Year
D
Bhutan: NN ratio to SPF: 2.01 // U5 ratio to SPF: 1.71
Neonatal
Under 5
Deaths per 1000 live births
40
30
20
10
0
125
100
75
50
25
0
Bhutan
25
50
75
100
HAQ Index
SDI quintile
Low SDI
Low−middle SDI
Middle SDI
High−middle SDI
High SDI
Benchmarks
SDG target
Global Optimum
Survival Potential Frontier

India

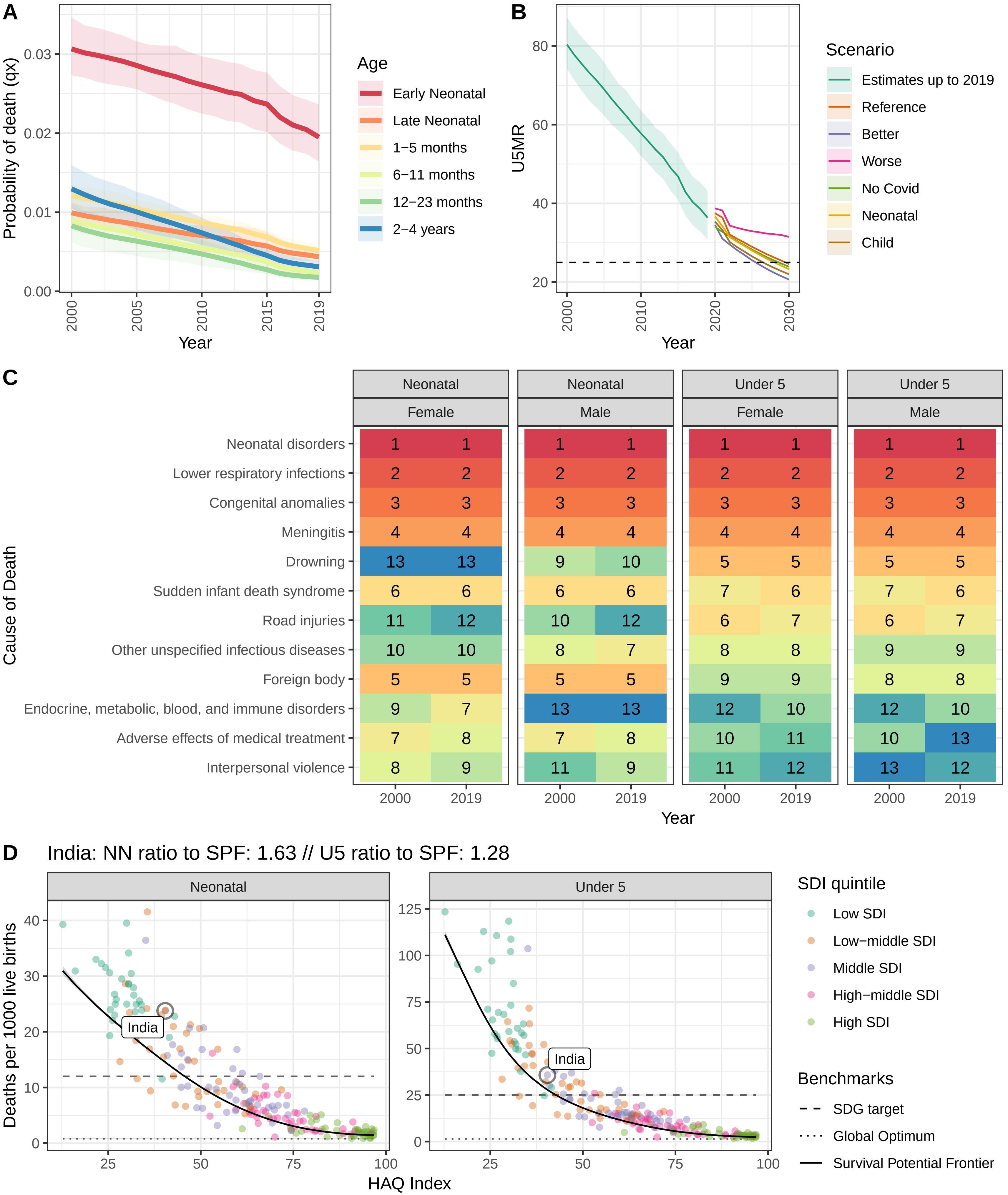
A
Probability of death (qx)
0.03
0.02
0.01
0.00
2000
2005
2010
2015
2019
Year
Age
Early Neonatal
Late Neonatal
1−5 months
6−11 months
12−23 months
2−4 years
B
U5MR
80
60
40
20
2000
2010
2020
2030
Year
Scenario
Estimates up to 2019
Reference
Better
Worse
No Covid
Neonatal
Child
C
Neonatal
Female
Neonatal
Male
Under 5
Female
Under 5
Male
Cause of Death
Neonatal disorders
Lower respiratory infections
Congenital anomalies
Meningitis
Drowning
Sudden infant death syndrome
Road injuries
Other unspecified infectious diseases
Foreign body
Endocrine, metabolic, blood, and immune disorders
Adverse effects of medical treatment
Interpersonal violence
Neonatal disorders: 1 1 | 1 1 | 1 1 | 1 1
Lower respiratory infections: 2 2 | 2 2 | 2 2 | 2 2
Congenital anomalies: 3 3 | 3 3 | 3 3 | 3 3
Meningitis: 4 4 | 4 4 | 4 4 | 4 4
Drowning: 13 13 | 9 10 | 5 5 | 5 5
Sudden infant death syndrome: 6 6 | 6 6 | 7 6 | 7 6
Road injuries: 11 12 | 10 12 | 6 7 | 6 7
Other unspecified infectious diseases: 10 10 | 8 7 | 8 8 | 9 9
Foreign body: 5 5 | 5 5 | 9 9 | 8 8
Endocrine, metabolic, blood, and immune disorders: 9 7 | 13 13 | 12 10 | 12 10
Adverse effects of medical treatment: 7 8 | 7 8 | 10 11 | 10 13
Interpersonal violence: 8 9 | 11 9 | 11 12 | 13 12
2000
2019
Year
D
India: NN ratio to SPF: 1.63 // U5 ratio to SPF: 1.28
Neonatal
Under 5
Deaths per 1000 live births
40
30
20
10
0
125
100
75
50
25
0
25
50
75
100
India
HAQ Index
SDI quintile
Low SDI
Low−middle SDI
Middle SDI
High−middle SDI
High SDI
Benchmarks
SDG target
Global Optimum
Survival Potential Frontier

Nepal

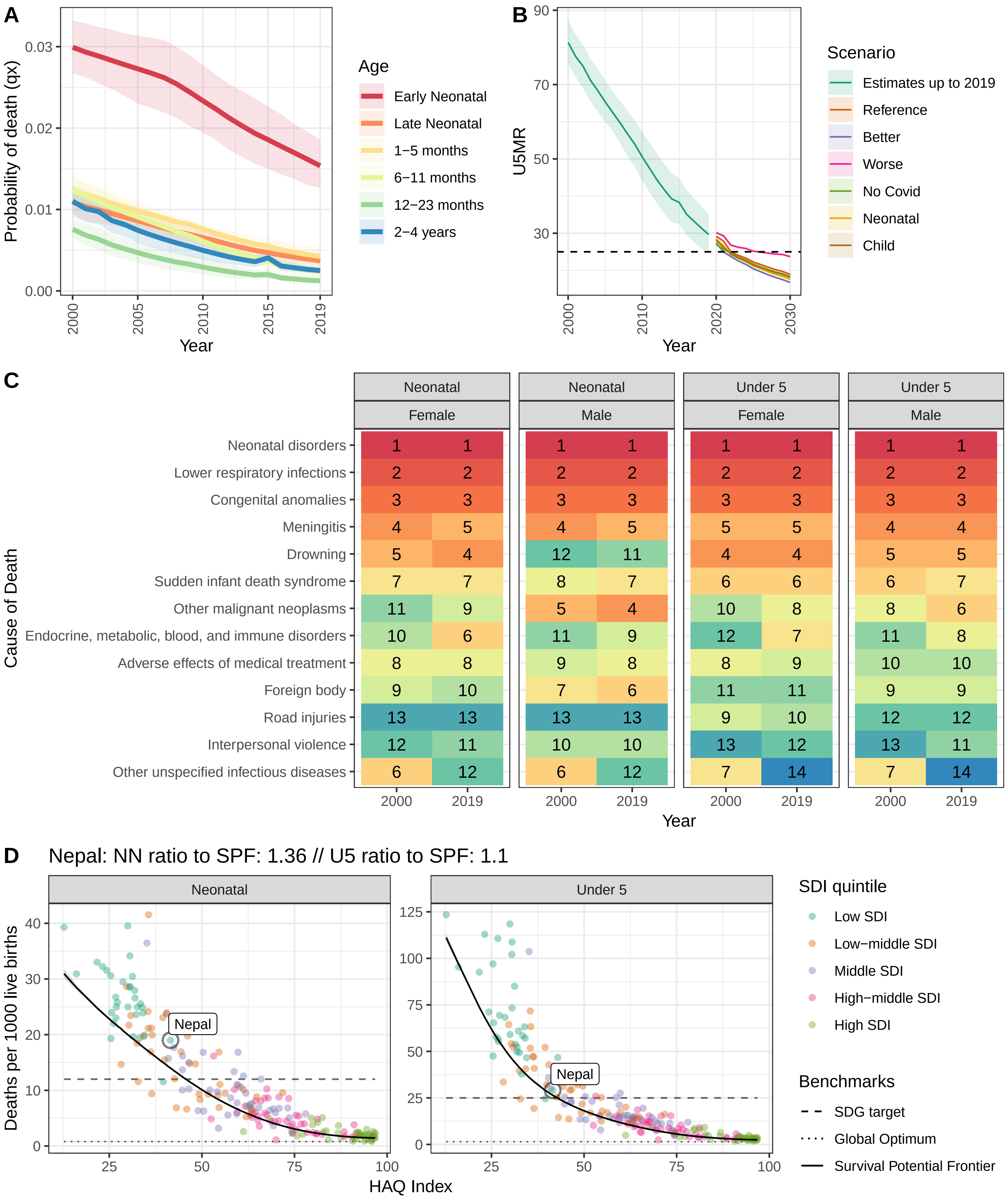
A
Probability of death (qx)
0.03
0.02
0.01
0.00
2000
2005
2010
2015
2019
Year
Age
Early Neonatal
Late Neonatal
1−5 months
6−11 months
12−23 months
2−4 years
B
U5MR
90
70
50
30
2000
2010
2020
2030
Year
Scenario
Estimates up to 2019
Reference
Better
Worse
No Covid
Neonatal
Child
C
Cause of Death
Neonatal Female
Neonatal Male
Under 5 Female
Under 5 Male
Neonatal disorders 1 1 1 1 1 1 1 1
Lower respiratory infections 2 2 2 2 2 2 2 2
Congenital anomalies 3 3 3 3 3 3 3 3
Meningitis 4 5 4 5 5 5 4 4
Drowning 5 4 12 11 4 4 5 5
Sudden infant death syndrome 7 7 8 7 6 6 6 7
Other malignant neoplasms 11 9 5 4 10 8 8 6
Endocrine, metabolic, blood, and immune disorders 10 6 11 9 12 7 11 8
Adverse effects of medical treatment 8 8 9 8 8 9 10 10
Foreign body 9 10 7 6 11 11 9 9
Road injuries 13 13 13 13 9 10 12 12
Interpersonal violence 12 11 10 10 13 12 13 11
Other unspecified infectious diseases 6 12 6 12 7 14 7 14
2000
2019
Year
D
Nepal: NN ratio to SPF: 1.36 // U5 ratio to SPF: 1.1
Neonatal
Under 5
Deaths per 1000 live births
40
30
20
10
0
125
100
75
50
25
0
Nepal
25
50
75
100
HAQ Index
SDI quintile
Low SDI
Low−middle SDI
Middle SDI
High−middle SDI
High SDI
Benchmarks
SDG target
Global Optimum
Survival Potential Frontier

Pakistan

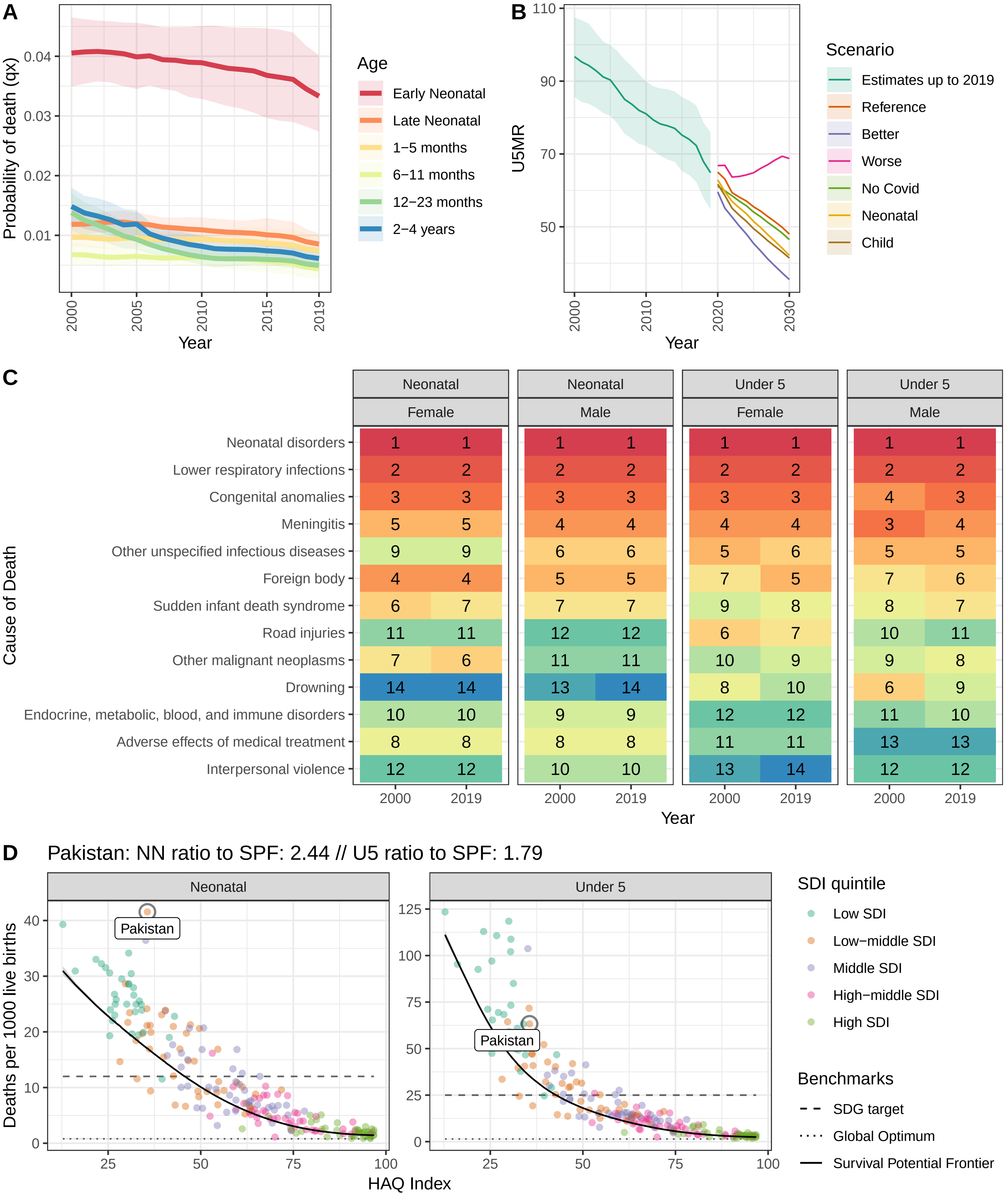

A
Probability of death (qx)
Year
Age
Early Neonatal
Late Neonatal
1−5 months
6−11 months
12−23 months
2−4 years
B
U5MR
Year
Scenario
Estimates up to 2019
Reference
Better
Worse
No Covid
Neonatal
Child
C
Cause of Death
Neonatal Female
Neonatal Male
Under 5 Female
Under 5 Male
Neonatal disorders 1 1 | 1 1 | 1 1 | 1 1
Lower respiratory infections 2 2 | 2 2 | 2 2 | 2 2
Congenital anomalies 3 3 | 3 3 | 3 3 | 4 3
Meningitis 5 5 | 4 4 | 4 4 | 3 4
Other unspecified infectious diseases 9 9 | 6 6 | 5 6 | 5 5
Foreign body 4 4 | 5 5 | 7 5 | 7 6
Sudden infant death syndrome 6 7 | 7 7 | 9 8 | 8 7
Road injuries 11 11 | 12 12 | 6 7 | 10 11
Other malignant neoplasms 7 6 | 11 11 | 10 9 | 9 8
Drowning 14 14 | 13 14 | 8 10 | 6 9
Endocrine, metabolic, blood, and immune disorders 10 10 | 9 9 | 12 12 | 11 10
Adverse effects of medical treatment 8 8 | 8 8 | 11 11 | 13 13
Interpersonal violence 12 12 | 10 10 | 13 14 | 12 12
Year
D
Pakistan: NN ratio to SPF: 2.44 // U5 ratio to SPF: 1.79
Neonatal
Under 5
Pakistan
Deaths per 1000 live births
HAQ Index
SDI quintile
Low SDI
Low−middle SDI
Middle SDI
High−middle SDI
High SDI
Benchmarks
SDG target
Global Optimum
Survival Potential Frontier

Angola

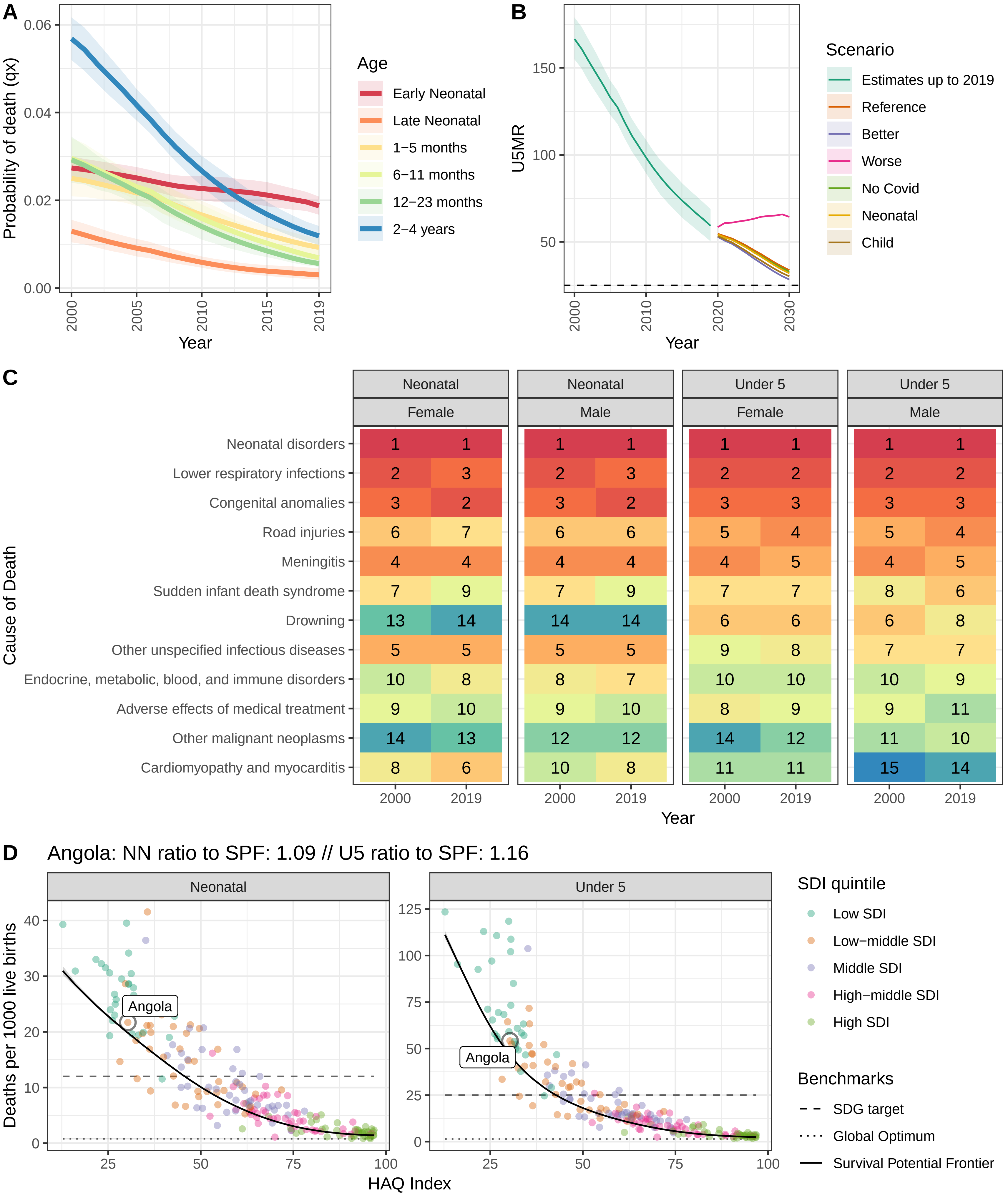

A
Probability of death (qx)
0.06
0.04
0.02
0.00
2000
2005
2010
2015
2019
Year
Age
Early Neonatal
Late Neonatal
1−5 months
6−11 months
12−23 months
2−4 years
B
U5MR
150
100
50
2000
2010
2020
2030
Year
Scenario
Estimates up to 2019
Reference
Better
Worse
No Covid
Neonatal
Child
C
Cause of Death
Neonatal
Female
Neonatal
Male
Under 5
Female
Under 5
Male
Neonatal disorders 1 1 1 1 1 1 1 1
Lower respiratory infections 2 3 2 3 2 2 2 2
Congenital anomalies 3 2 3 2 3 3 3 3
Road injuries 6 7 6 6 5 4 5 4
Meningitis 4 4 4 4 4 5 4 5
Sudden infant death syndrome 7 9 7 9 7 7 8 6
Drowning 13 14 14 14 6 6 6 8
Other unspecified infectious diseases 5 5 5 5 9 8 7 7
Endocrine, metabolic, blood, and immune disorders 10 8 8 7 10 10 10 9
Adverse effects of medical treatment 9 10 9 10 8 9 9 11
Other malignant neoplasms 14 13 12 12 14 12 11 10
Cardiomyopathy and myocarditis 8 6 10 8 11 11 15 14
2000
2019
Year
D
Angola: NN ratio to SPF: 1.09 // U5 ratio to SPF: 1.16
Neonatal
Under 5
Deaths per 1000 live births
40
30
20
10
0
125
100
75
50
25
0
Angola
Angola
25
50
75
100
HAQ Index
SDI quintile
Low SDI
Low−middle SDI
Middle SDI
High−middle SDI
High SDI
Benchmarks
SDG target
Global Optimum
Survival Potential Frontier

Central African Republic

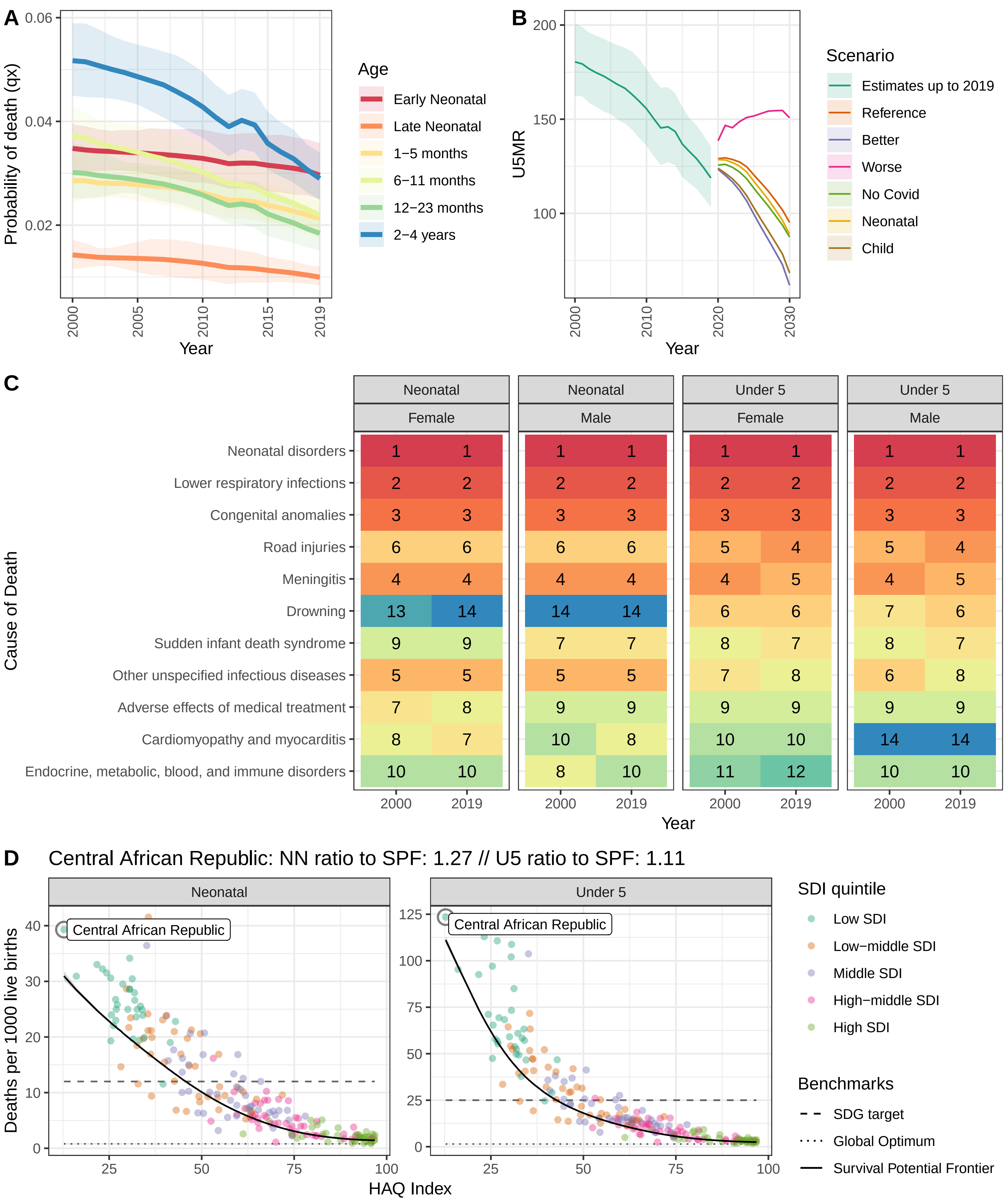

Sri Lanka

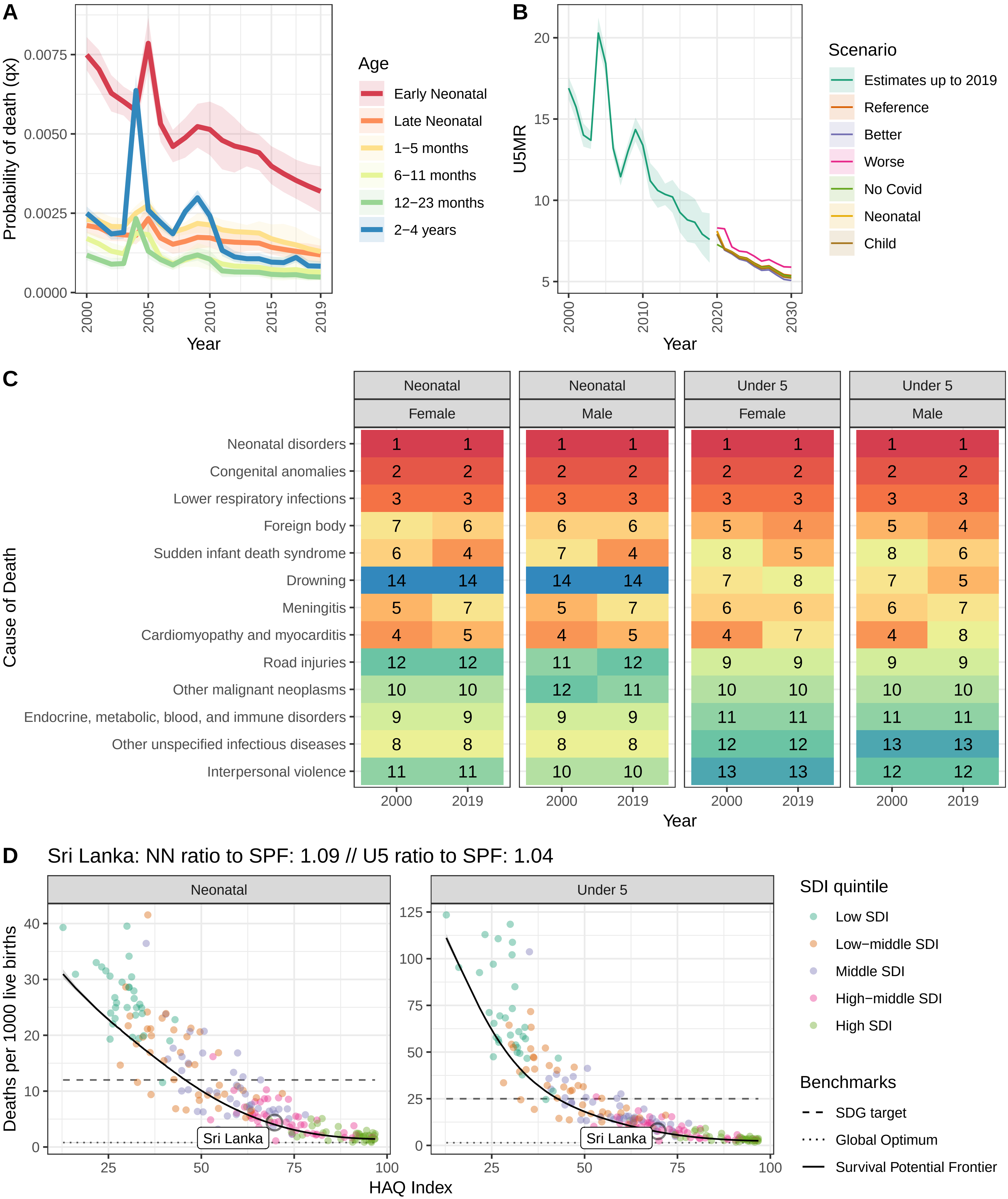
A
Probability of death (qx)
0.0075
0.0050
0.0025
0.0000
2000
2005
2010
2015
2019
Year
Age
Early Neonatal
Late Neonatal
1−5 months
6−11 months
12−23 months
2−4 years
B
U5MR
20
15
10
5
2000
2010
2020
2030
Year
Scenario
Estimates up to 2019
Reference
Better
Worse
No Covid
Neonatal
Child
C
Neonatal
Female
Neonatal
Male
Under 5
Female
Under 5
Male
Cause of Death
Neonatal disorders
Congenital anomalies
Lower respiratory infections
Foreign body
Sudden infant death syndrome
Drowning
Meningitis
Cardiomyopathy and myocarditis
Road injuries
Other malignant neoplasms
Endocrine, metabolic, blood, and immune disorders
Other unspecified infectious diseases
Interpersonal violence
Neonatal disorders: 1 1 | 1 1 | 1 1 | 1 1
Congenital anomalies: 2 2 | 2 2 | 2 2 | 2 2
Lower respiratory infections: 3 3 | 3 3 | 3 3 | 3 3
Foreign body: 7 6 | 6 6 | 5 4 | 5 4
Sudden infant death syndrome: 6 4 | 7 4 | 8 5 | 8 6
Drowning: 14 14 | 14 14 | 7 8 | 7 5
Meningitis: 5 7 | 5 7 | 6 6 | 6 7
Cardiomyopathy and myocarditis: 4 5 | 4 5 | 4 7 | 4 8
Road injuries: 12 12 | 11 12 | 9 9 | 9 9
Other malignant neoplasms: 10 10 | 12 11 | 10 10 | 10 10
Endocrine, metabolic, blood, and immune disorders: 9 9 | 9 9 | 11 11 | 11 11
Other unspecified infectious diseases: 8 8 | 8 8 | 12 12 | 13 13
Interpersonal violence: 11 11 | 10 10 | 13 13 | 12 12
2000
2019
Year
D
Sri Lanka: NN ratio to SPF: 1.09 // U5 ratio to SPF: 1.04
Neonatal
Under 5
Deaths per 1000 live births
40
30
20
10
0
125
100
75
50
25
0
Sri Lanka
Sri Lanka
25
50
75
100
HAQ Index
SDI quintile
Low SDI
Low−middle SDI
Middle SDI
High−middle SDI
High SDI
Benchmarks
SDG target
Global Optimum
Survival Potential Frontier

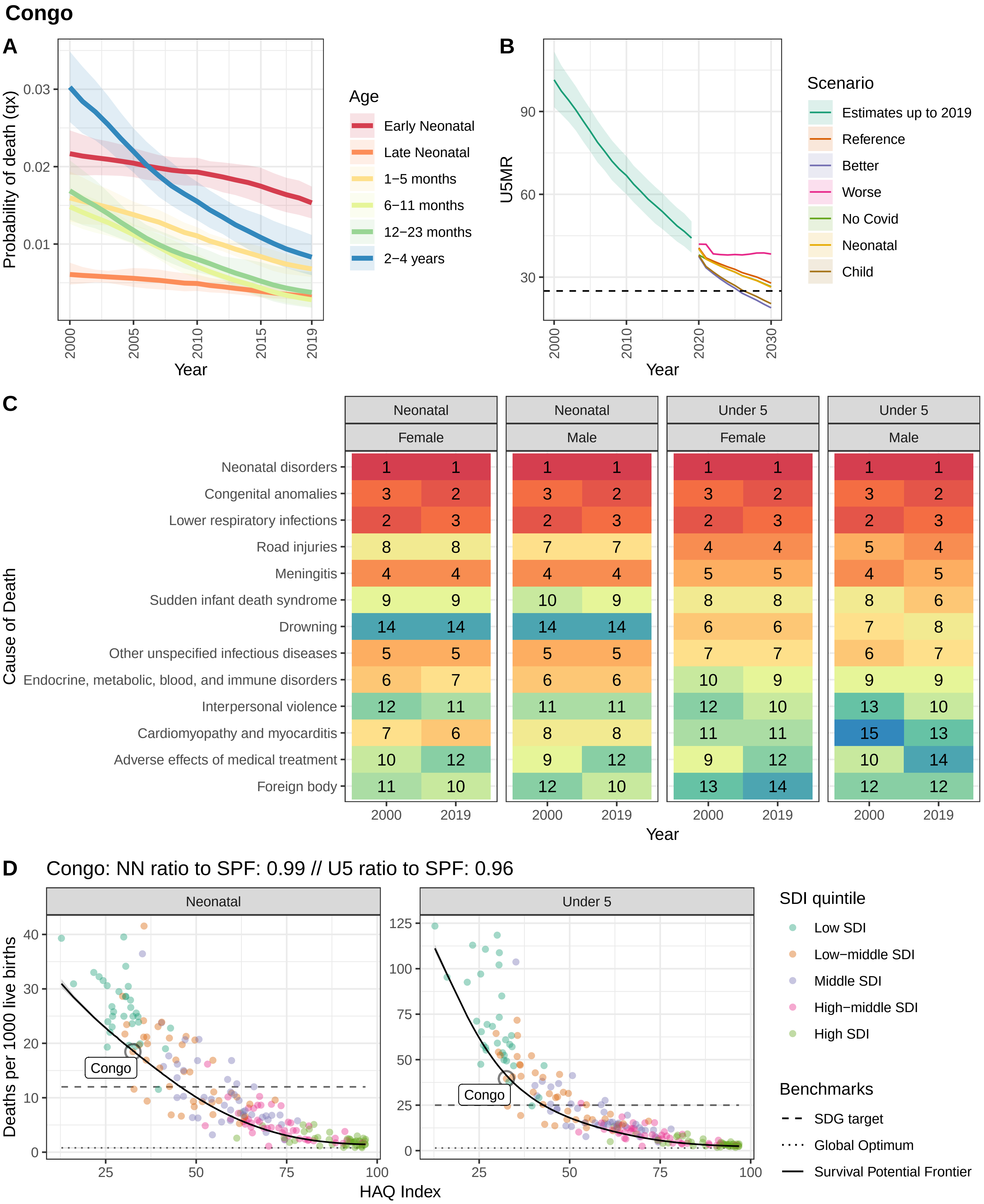
Congo
A
Probability of death (qx)
0.03
0.02
0.01
2000
2005
2010
2015
2019
Year
Age
Early Neonatal
Late Neonatal
1−5 months
6−11 months
12−23 months
2−4 years
B
U5MR
90
60
30
2000
2010
2020
2030
Year
Scenario
Estimates up to 2019
Reference
Better
Worse
No Covid
Neonatal
Child
C
Neonatal
Female
Neonatal
Male
Under 5
Female
Under 5
Male
Cause of Death
Neonatal disorders 1 1 1 1 1 1 1 1
Congenital anomalies 3 2 3 2 3 2 3 2
Lower respiratory infections 2 3 2 3 2 3 2 3
Road injuries 8 8 7 7 4 4 5 4
Meningitis 4 4 4 4 5 5 4 5
Sudden infant death syndrome 9 9 10 9 8 8 8 6
Drowning 14 14 14 14 6 6 7 8
Other unspecified infectious diseases 5 5 5 5 7 7 6 7
Endocrine, metabolic, blood, and immune disorders 6 7 6 6 10 9 9 9
Interpersonal violence 12 11 11 11 12 10 13 10
Cardiomyopathy and myocarditis 7 6 8 8 11 11 15 13
Adverse effects of medical treatment 10 12 9 12 9 12 10 14
Foreign body 11 10 12 10 13 14 12 12
2000
2019
Year
D
Congo: NN ratio to SPF: 0.99 // U5 ratio to SPF: 0.96
Neonatal
Under 5
Deaths per 1000 live births
40
30
20
10
0
125
100
75
50
25
0
Congo
Congo
25
50
75
100
HAQ Index
SDI quintile
Low SDI
Low−middle SDI
Middle SDI
High−middle SDI
High SDI
Benchmarks
SDG target
Global Optimum
Survival Potential Frontier

Democratic Republic of the Congo

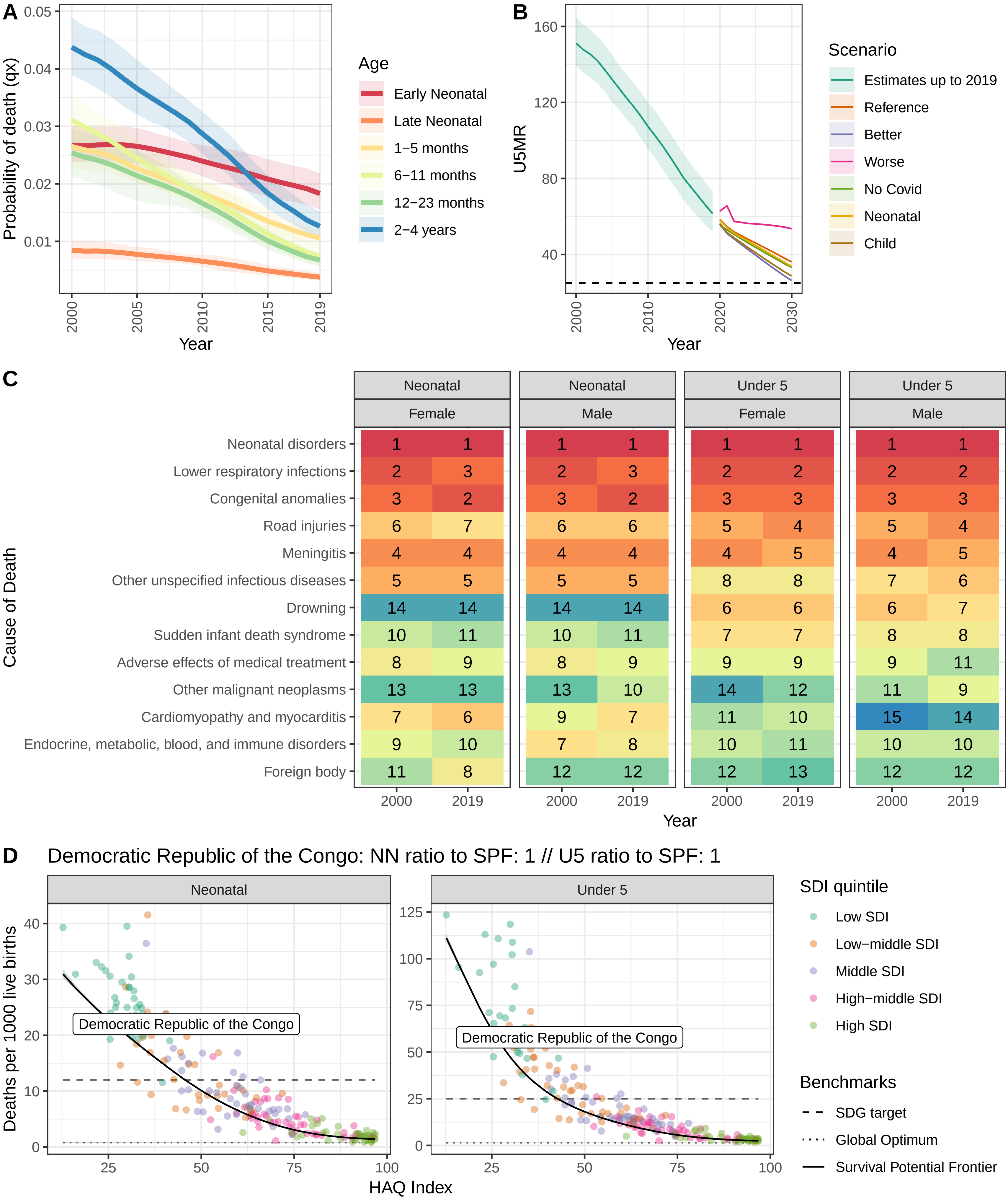

Equatorial Guinea

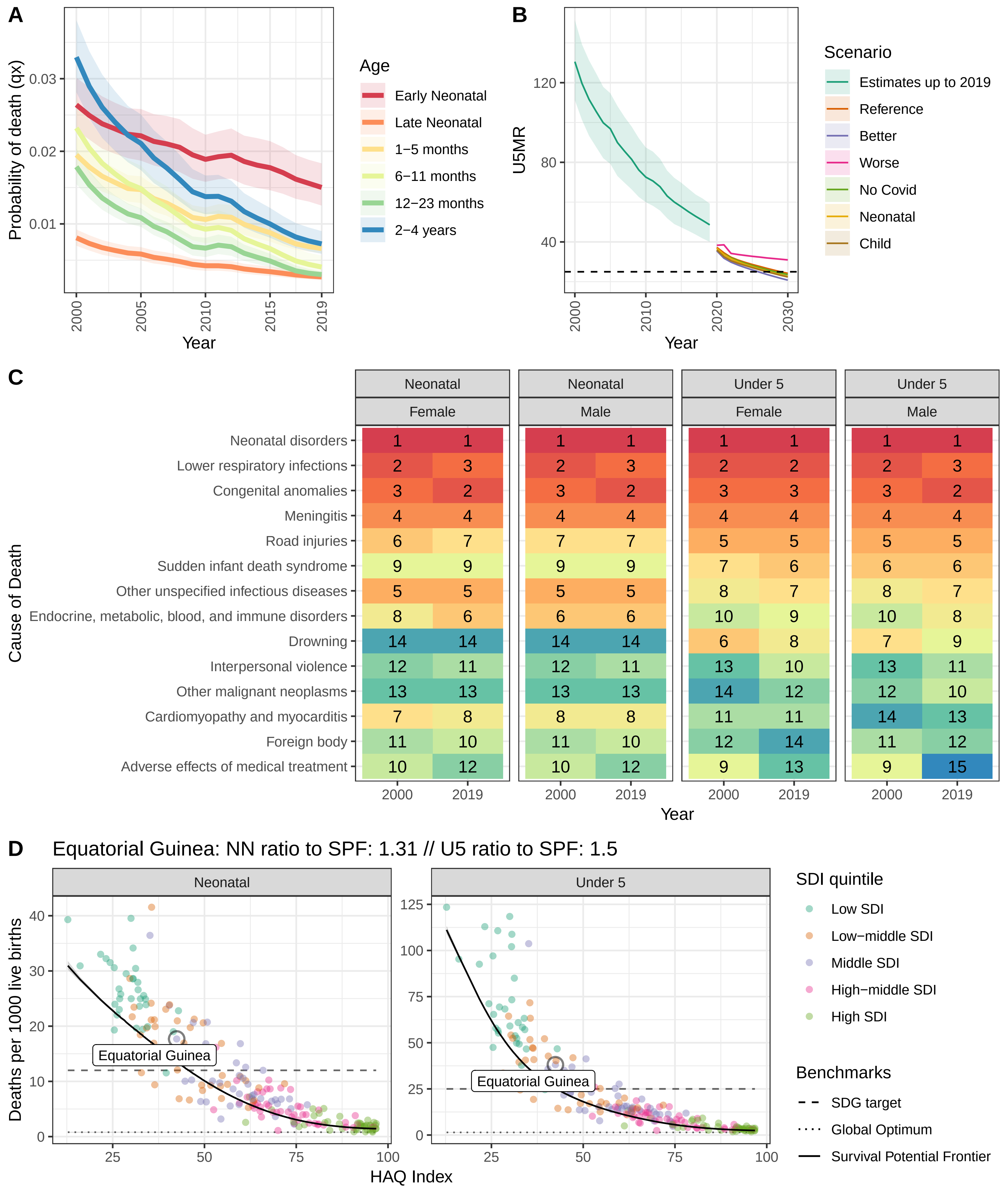

Gabon

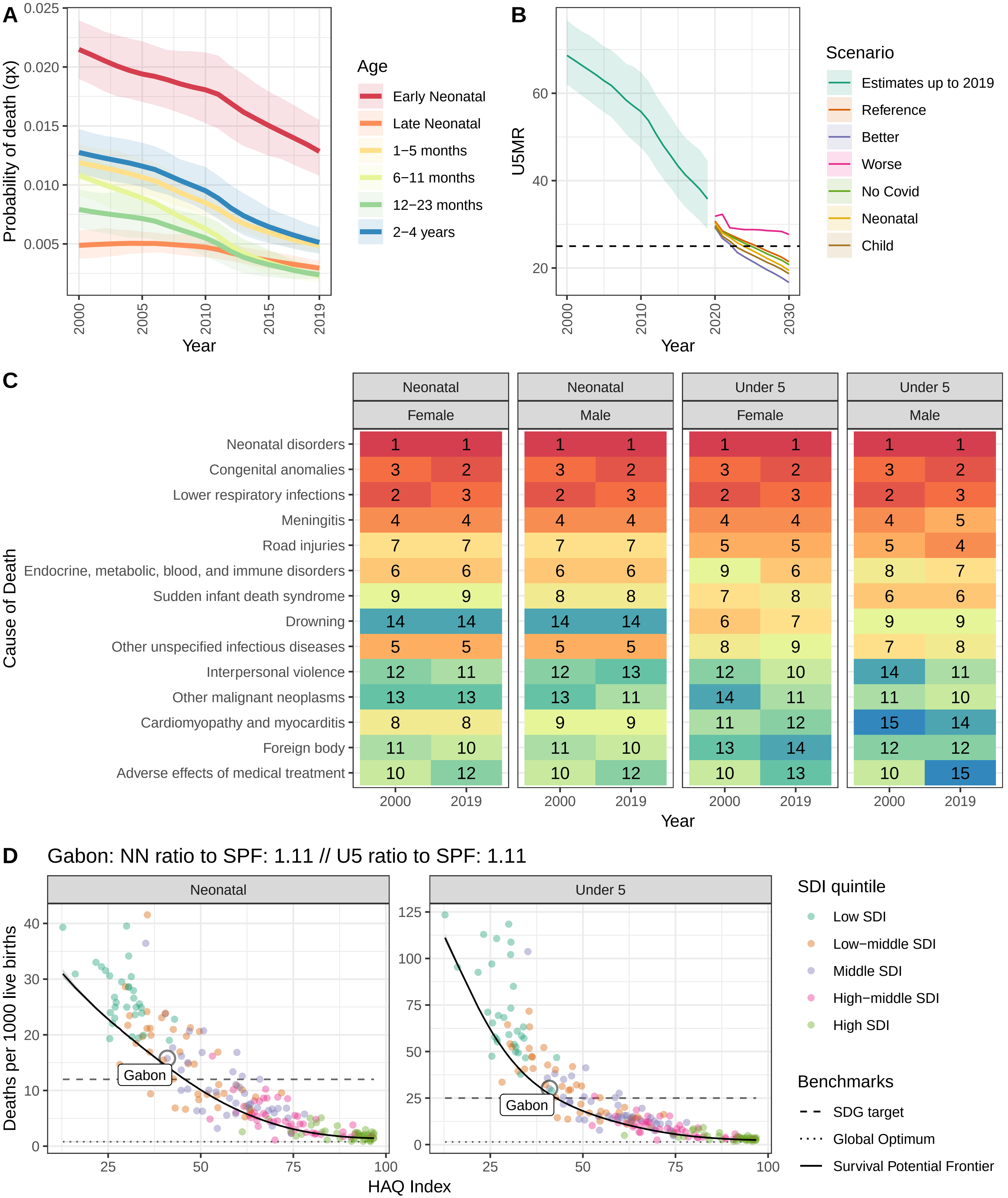
A
Probability of death (qx)
0.025
0.020
0.015
0.010
0.005
2000
2005
2010
2015
2019
Year
Age
Early Neonatal
Late Neonatal
1−5 months
6−11 months
12−23 months
2−4 years
B
U5MR
60
40
20
2000
2010
2020
2030
Year
Scenario
Estimates up to 2019
Reference
Better
Worse
No Covid
Neonatal
Child
C
Neonatal
Female
Neonatal
Male
Under 5
Female
Under 5
Male
Cause of Death
Neonatal disorders 1 1 1 1 1 1 1 1
Congenital anomalies 3 2 3 2 3 2 3 2
Lower respiratory infections 2 3 2 3 2 3 2 3
Meningitis 4 4 4 4 4 4 4 5
Road injuries 7 7 7 7 5 5 5 4
Endocrine, metabolic, blood, and immune disorders 6 6 6 6 9 6 8 7
Sudden infant death syndrome 9 9 8 8 7 8 6 6
Drowning 14 14 14 14 6 7 9 9
Other unspecified infectious diseases 5 5 5 5 8 9 7 8
Interpersonal violence 12 11 12 13 12 10 14 11
Other malignant neoplasms 13 13 13 11 14 11 11 10
Cardiomyopathy and myocarditis 8 8 9 9 11 12 15 14
Foreign body 11 10 11 10 13 14 12 12
Adverse effects of medical treatment 10 12 10 12 10 13 10 15
2000
2019
Year
D
Gabon: NN ratio to SPF: 1.11 // U5 ratio to SPF: 1.11
Neonatal
Under 5
Deaths per 1000 live births
40
30
20
10
0
125
100
75
50
25
0
Gabon
Gabon
25
50
75
100
HAQ Index
SDI quintile
Low SDI
Low−middle SDI
Middle SDI
High−middle SDI
High SDI
Benchmarks
SDG target
Global Optimum
Survival Potential Frontier

Burundi

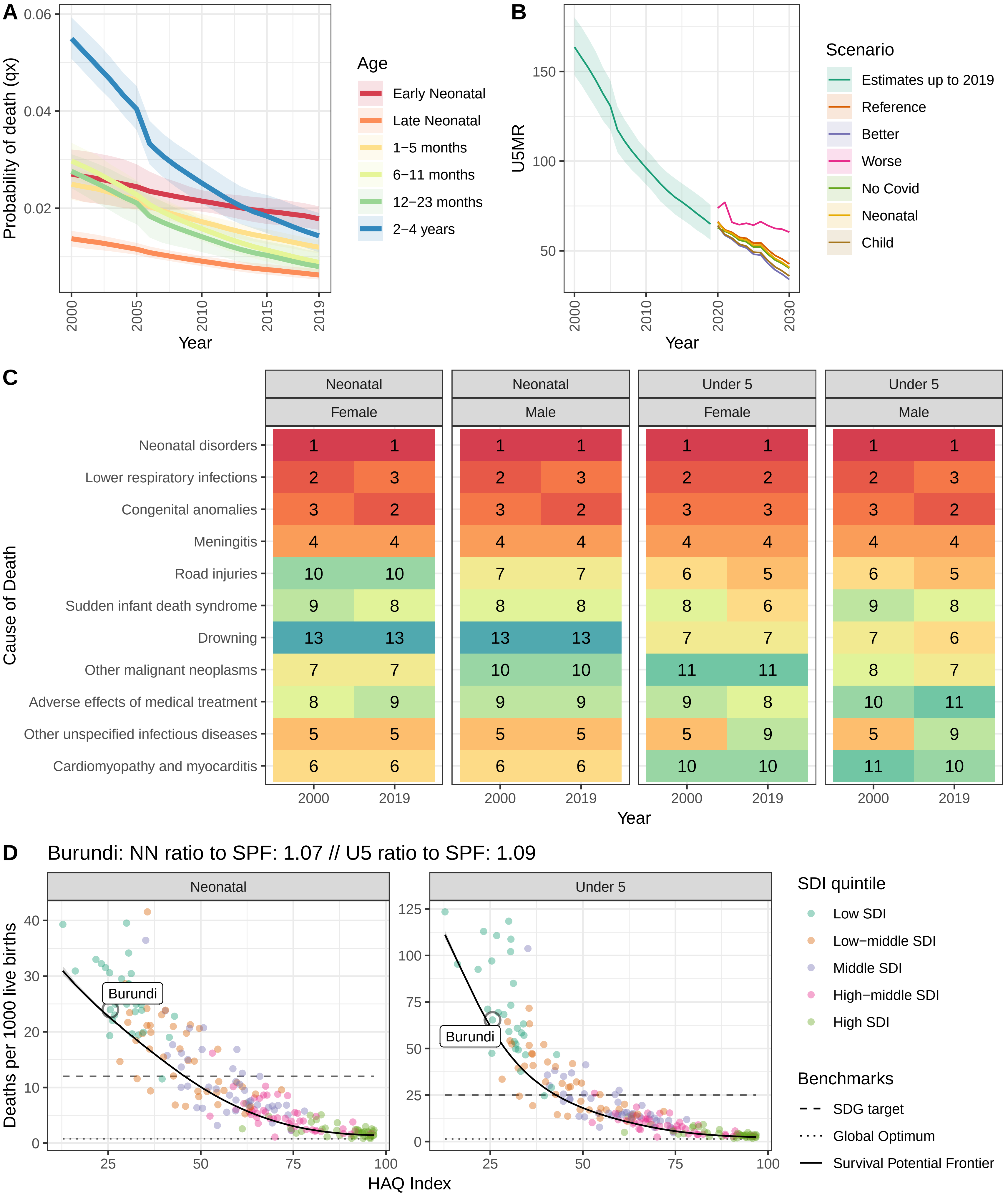

A
Probability of death (qx)
Year
Age
Early Neonatal
Late Neonatal
1−5 months
6−11 months
12−23 months
2−4 years
B
U5MR
Year
Scenario
Estimates up to 2019
Reference
Better
Worse
No Covid
Neonatal
Child
C
Cause of Death
Neonatal Female
Neonatal Male
Under 5 Female
Under 5 Male
Year
2000
2019
Neonatal disorders 1 1 1 1 1 1 1 1
Lower respiratory infections 2 3 2 3 2 2 2 3
Congenital anomalies 3 2 3 2 3 3 3 2
Meningitis 4 4 4 4 4 4 4 4
Road injuries 10 10 7 7 6 5 6 5
Sudden infant death syndrome 9 8 8 8 8 6 9 8
Drowning 13 13 13 13 7 7 7 6
Other malignant neoplasms 7 7 10 10 11 11 8 7
Adverse effects of medical treatment 8 9 9 9 9 8 10 11
Other unspecified infectious diseases 5 5 5 5 5 9 5 9
Cardiomyopathy and myocarditis 6 6 6 6 10 10 11 10
D
Burundi: NN ratio to SPF: 1.07 // U5 ratio to SPF: 1.09
Neonatal
Under 5
Burundi
Deaths per 1000 live births
HAQ Index
SDI quintile
Low SDI
Low−middle SDI
Middle SDI
High−middle SDI
High SDI
Benchmarks
SDG target
Global Optimum
Survival Potential Frontier

Comoros

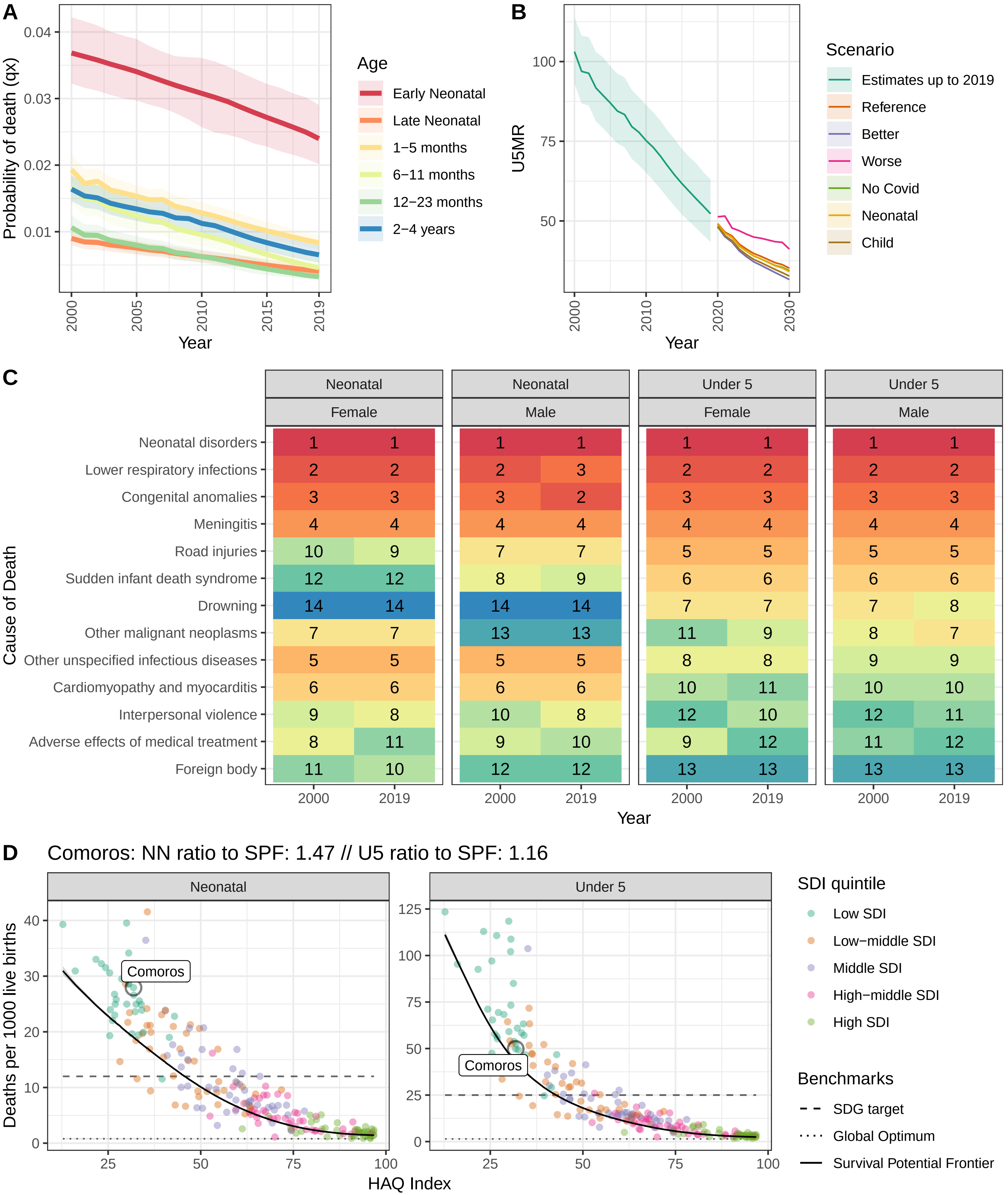

A
Probability of death (qx)
0.04
0.03
0.02
0.01
2000
2005
2010
2015
2019
Year
Age
Early Neonatal
Late Neonatal
1−5 months
6−11 months
12−23 months
2−4 years
B
U5MR
100
75
50
2000
2010
2020
2030
Year
Scenario
Estimates up to 2019
Reference
Better
Worse
No Covid
Neonatal
Child
C
Neonatal Female
Neonatal Male
Under 5 Female
Under 5 Male
Cause of Death
Neonatal disorders 1 1 1 1 1 1 1 1
Lower respiratory infections 2 2 2 3 2 2 2 2
Congenital anomalies 3 3 3 2 3 3 3 3
Meningitis 4 4 4 4 4 4 4 4
Road injuries 10 9 7 7 5 5 5 5
Sudden infant death syndrome 12 12 8 9 6 6 6 6
Drowning 14 14 14 14 7 7 7 8
Other malignant neoplasms 7 7 13 13 11 9 8 7
Other unspecified infectious diseases 5 5 5 5 8 8 9 9
Cardiomyopathy and myocarditis 6 6 6 6 10 11 10 10
Interpersonal violence 9 8 10 8 12 10 12 11
Adverse effects of medical treatment 8 11 9 10 9 12 11 12
Foreign body 11 10 12 12 13 13 13 13
2000 2019
Year
D
Comoros: NN ratio to SPF: 1.47 // U5 ratio to SPF: 1.16
Neonatal
Under 5
Deaths per 1000 live births
40
30
20
10
0
125
100
75
50
25
0
Comoros
25
50
75
100
HAQ Index
SDI quintile
Low SDI
Low−middle SDI
Middle SDI
High−middle SDI
High SDI
Benchmarks
SDG target
Global Optimum
Survival Potential Frontier

Djibouti

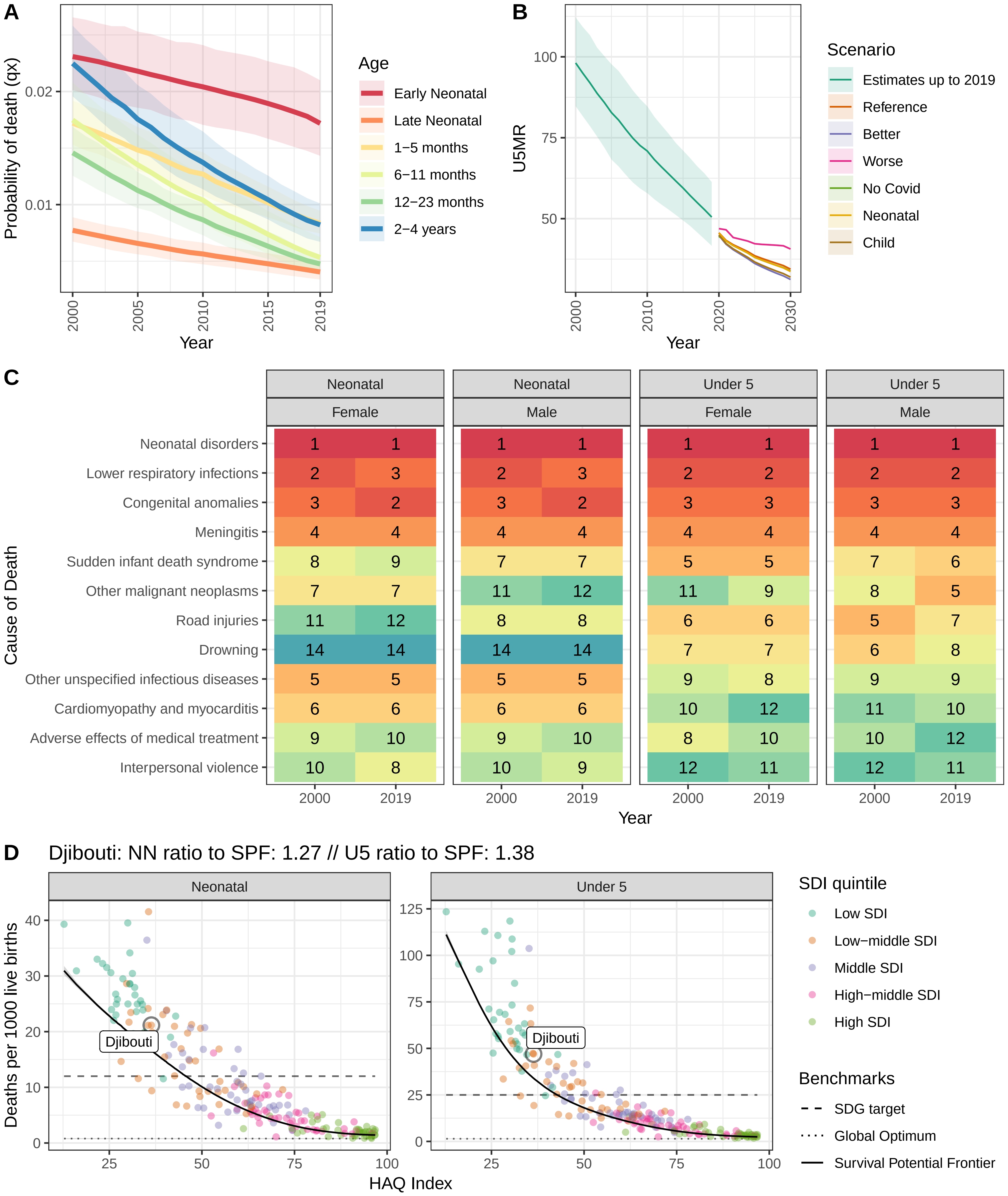
A
Probability of death (qx)
0.02
0.01
2000
2005
2010
2015
2019
Year
Age
Early Neonatal
Late Neonatal
1−5 months
6−11 months
12−23 months
2−4 years
B
U5MR
100
75
50
2000
2010
2020
2030
Year
Scenario
Estimates up to 2019
Reference
Better
Worse
No Covid
Neonatal
Child
C
Cause of Death
Neonatal
Female
Neonatal
Male
Under 5
Female
Under 5
Male
Neonatal disorders: 1 1 | 1 1 | 1 1 | 1 1
Lower respiratory infections: 2 3 | 2 3 | 2 2 | 2 2
Congenital anomalies: 3 2 | 3 2 | 3 3 | 3 3
Meningitis: 4 4 | 4 4 | 4 4 | 4 4
Sudden infant death syndrome: 8 9 | 7 7 | 5 5 | 7 6
Other malignant neoplasms: 7 7 | 11 12 | 11 9 | 8 5
Road injuries: 11 12 | 8 8 | 6 6 | 5 7
Drowning: 14 14 | 14 14 | 7 7 | 6 8
Other unspecified infectious diseases: 5 5 | 5 5 | 9 8 | 9 9
Cardiomyopathy and myocarditis: 6 6 | 6 6 | 10 12 | 11 10
Adverse effects of medical treatment: 9 10 | 9 10 | 8 10 | 10 12
Interpersonal violence: 10 8 | 10 9 | 12 11 | 12 11
2000
2019
Year
D
Djibouti: NN ratio to SPF: 1.27 // U5 ratio to SPF: 1.38
Neonatal
Under 5
Deaths per 1000 live births
40
30
20
10
0
125
100
75
50
25
0
Djibouti
25
50
75
100
HAQ Index
SDI quintile
Low SDI
Low−middle SDI
Middle SDI
High−middle SDI
High SDI
Benchmarks
SDG target
Global Optimum
Survival Potential Frontier

Eritrea

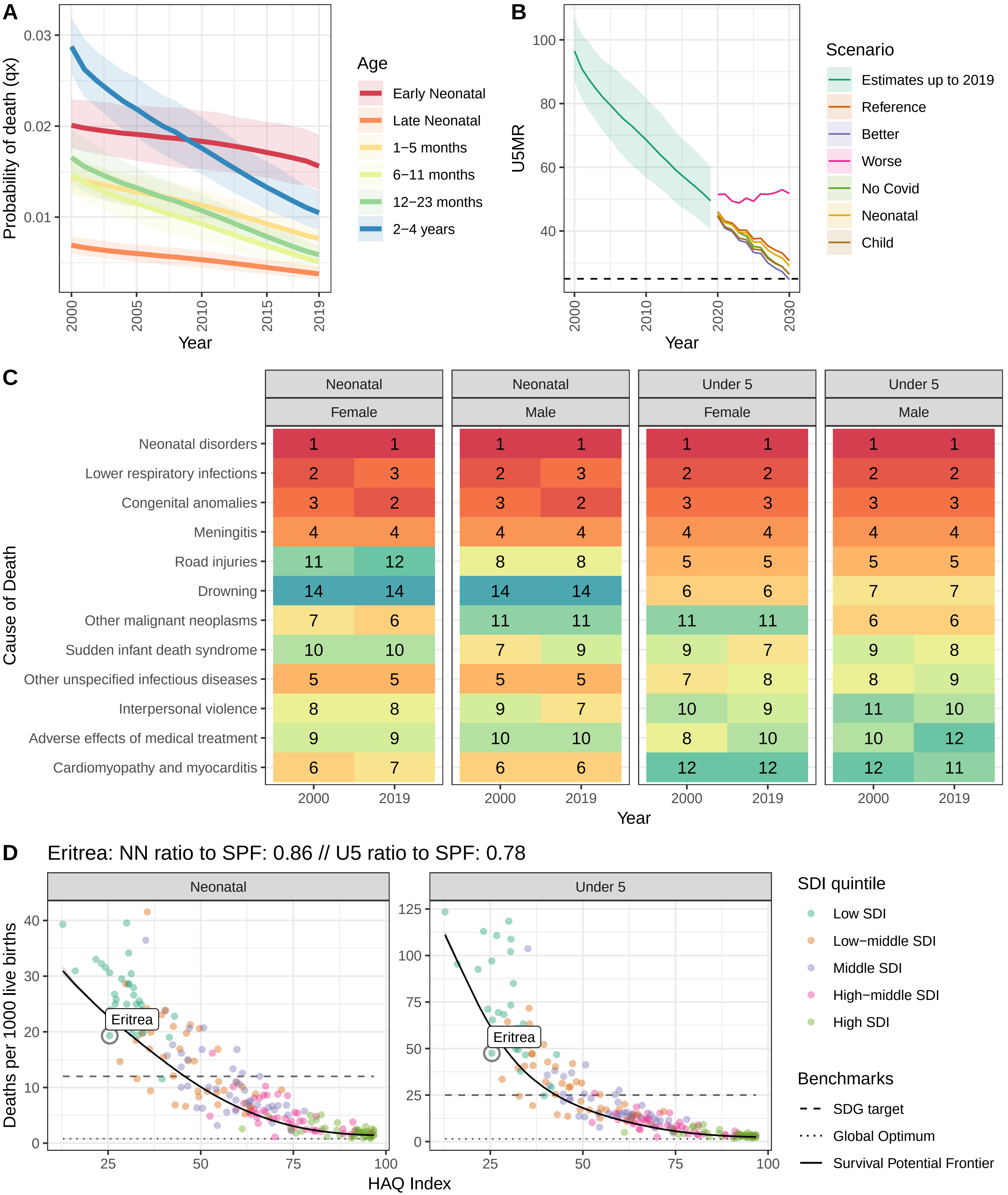

Cause of Death	Neonatal Female 2000	Neonatal Female 2019	Neonatal Male 2000	Neonatal Male 2019	Under 5 Female 2000	Under 5 Female 2019	Under 5 Male 2000	Under 5 Male 2019
Neonatal disorders	1	1	1	1	1	1	1	1
Lower respiratory infections	2	3	2	3	2	2	2	2
Congenital anomalies	3	2	3	2	3	3	3	3
Meningitis	4	4	4	4	4	4	4	4
Road injuries	11	12	8	8	5	5	5	5
Drowning	14	14	14	14	6	6	7	7
Other malignant neoplasms	7	6	11	11	11	11	6	6
Sudden infant death syndrome	10	10	7	9	9	7	9	8
Other unspecified infectious diseases	5	5	5	5	7	8	8	9
Interpersonal violence	8	8	9	7	10	9	11	10
Adverse effects of medical treatment	9	9	10	10	8	10	10	12
Cardiomyopathy and myocarditis	6	7	6	6	12	12	12	11

Ethiopia

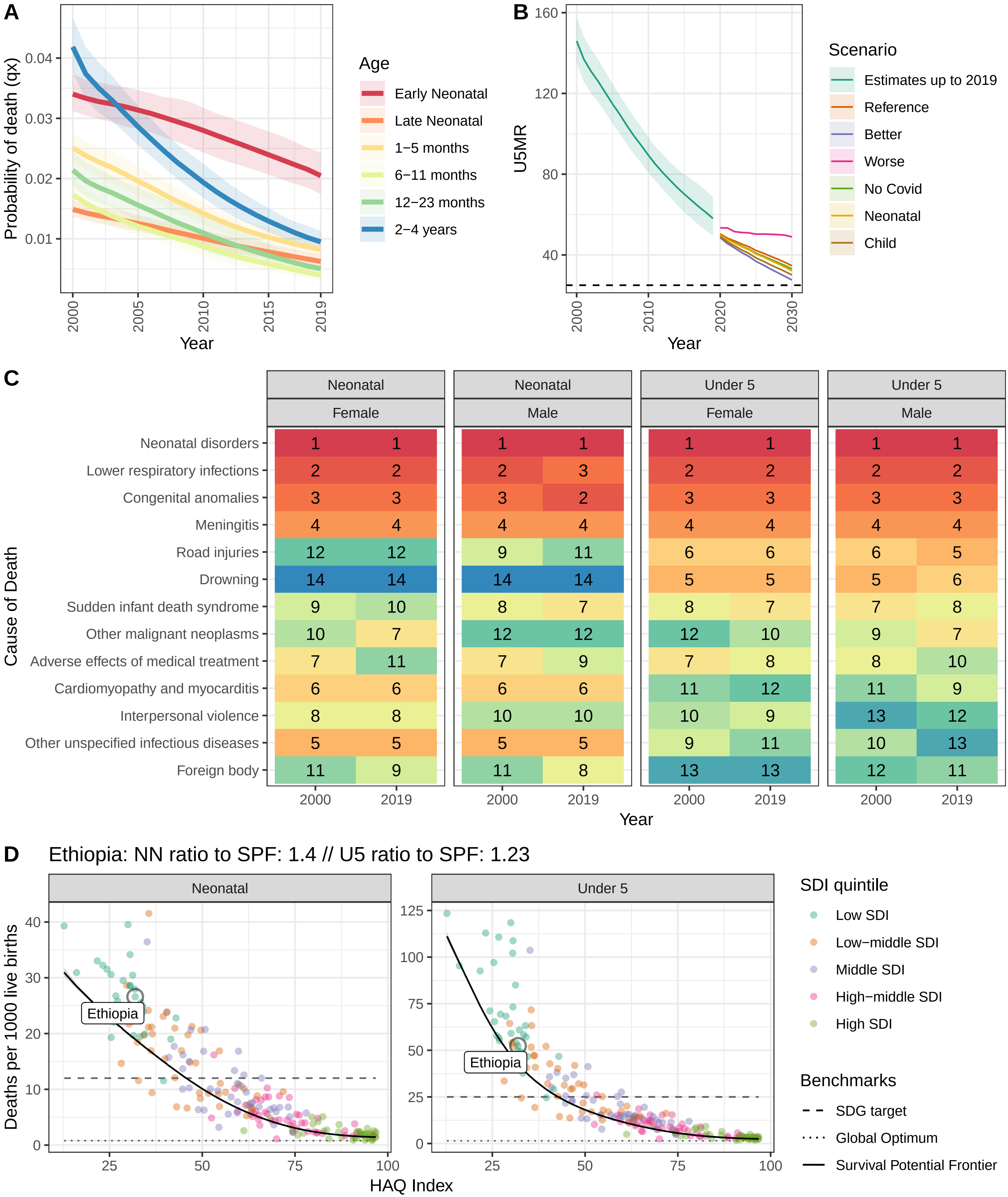
A
Probability of death (qx)
0.04
0.03
0.02
0.01
2000
2005
2010
2015
2019
Year
Age
Early Neonatal
Late Neonatal
1−5 months
6−11 months
12−23 months
2−4 years
B
U5MR
160
120
80
40
2000
2010
2020
2030
Year
Scenario
Estimates up to 2019
Reference
Better
Worse
No Covid
Neonatal
Child
C
Cause of Death
Neonatal Female
Neonatal Male
Under 5 Female
Under 5 Male
Neonatal disorders 1 1 1 1 1 1 1 1
Lower respiratory infections 2 2 2 3 2 2 2 2
Congenital anomalies 3 3 3 2 3 3 3 3
Meningitis 4 4 4 4 4 4 4 4
Road injuries 12 12 9 11 6 6 6 5
Drowning 14 14 14 14 5 5 5 6
Sudden infant death syndrome 9 10 8 7 8 7 7 8
Other malignant neoplasms 10 7 12 12 12 10 9 7
Adverse effects of medical treatment 7 11 7 9 7 8 8 10
Cardiomyopathy and myocarditis 6 6 6 6 11 12 11 9
Interpersonal violence 8 8 10 10 10 9 13 12
Other unspecified infectious diseases 5 5 5 5 9 11 10 13
Foreign body 11 9 11 8 13 13 12 11
2000
2019
Year
D
Ethiopia: NN ratio to SPF: 1.4 // U5 ratio to SPF: 1.23
Neonatal
Under 5
Deaths per 1000 live births
40
30
20
10
0
125
100
75
50
25
0
Ethiopia
Ethiopia
25
50
75
100
HAQ Index
SDI quintile
Low SDI
Low−middle SDI
Middle SDI
High−middle SDI
High SDI
Benchmarks
SDG target
Global Optimum
Survival Potential Frontier

Thailand

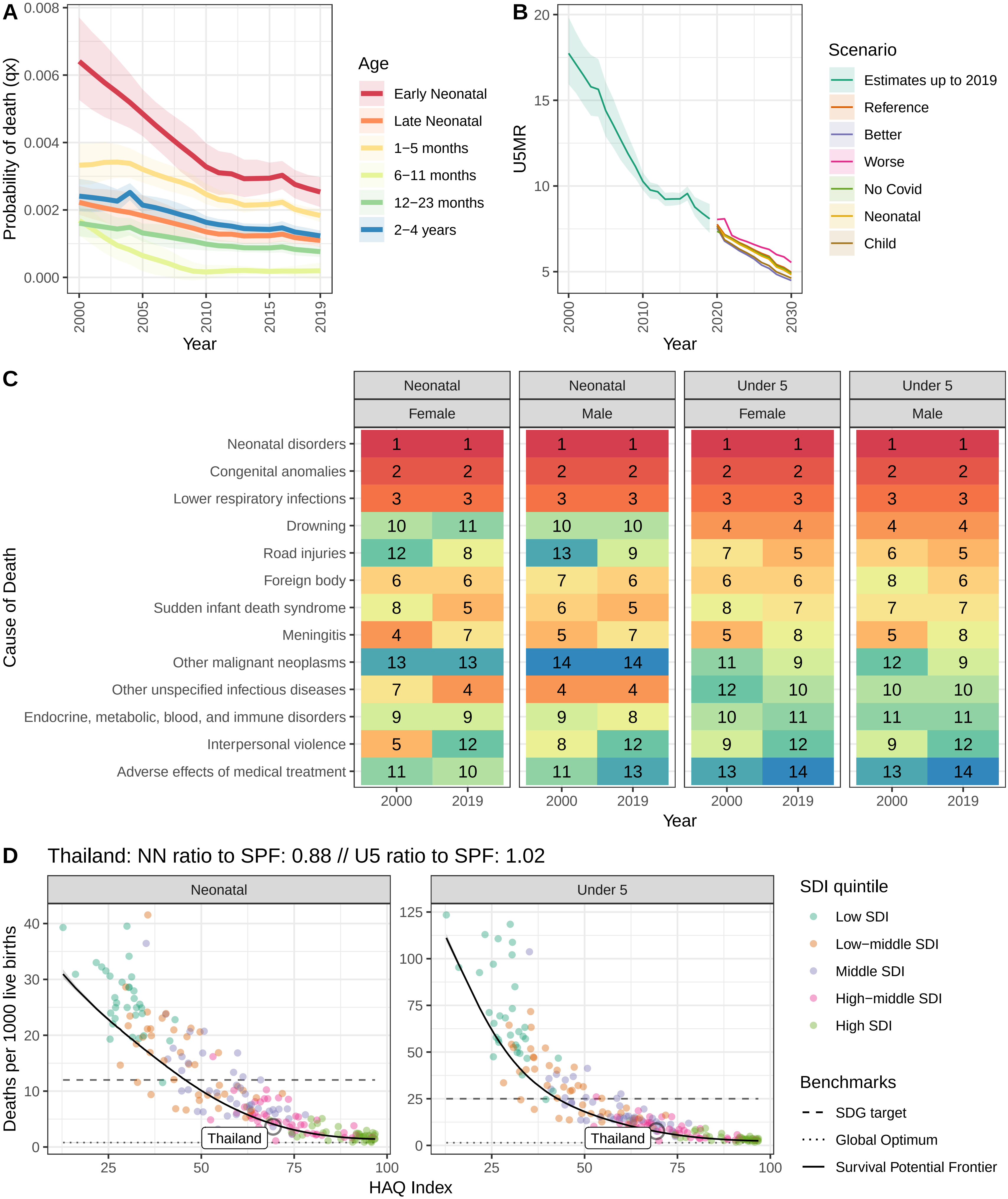
A
Probability of death (qx)
Year
Age
Early Neonatal
Late Neonatal
1−5 months
6−11 months
12−23 months
2−4 years
B
U5MR
Year
Scenario
Estimates up to 2019
Reference
Better
Worse
No Covid
Neonatal
Child
C
Cause of Death
Neonatal Female
Neonatal Male
Under 5 Female
Under 5 Male
Neonatal disorders 1 1 1 1 1 1 1 1
Congenital anomalies 2 2 2 2 2 2 2 2
Lower respiratory infections 3 3 3 3 3 3 3 3
Drowning 10 11 10 10 4 4 4 4
Road injuries 12 8 13 9 7 5 6 5
Foreign body 6 6 7 6 6 6 8 6
Sudden infant death syndrome 8 5 6 5 8 7 7 7
Meningitis 4 7 5 7 5 8 5 8
Other malignant neoplasms 13 13 14 14 11 9 12 9
Other unspecified infectious diseases 7 4 4 4 12 10 10 10
Endocrine, metabolic, blood, and immune disorders 9 9 9 8 10 11 11 11
Interpersonal violence 5 12 8 12 9 12 9 12
Adverse effects of medical treatment 11 10 11 13 13 14 13 14
2000 2019
Year
D
Thailand: NN ratio to SPF: 0.88 // U5 ratio to SPF: 1.02
Neonatal
Under 5
Thailand
Deaths per 1000 live births
HAQ Index
SDI quintile
Low SDI
Low−middle SDI
Middle SDI
High−middle SDI
High SDI
Benchmarks
SDG target
Global Optimum
Survival Potential Frontier

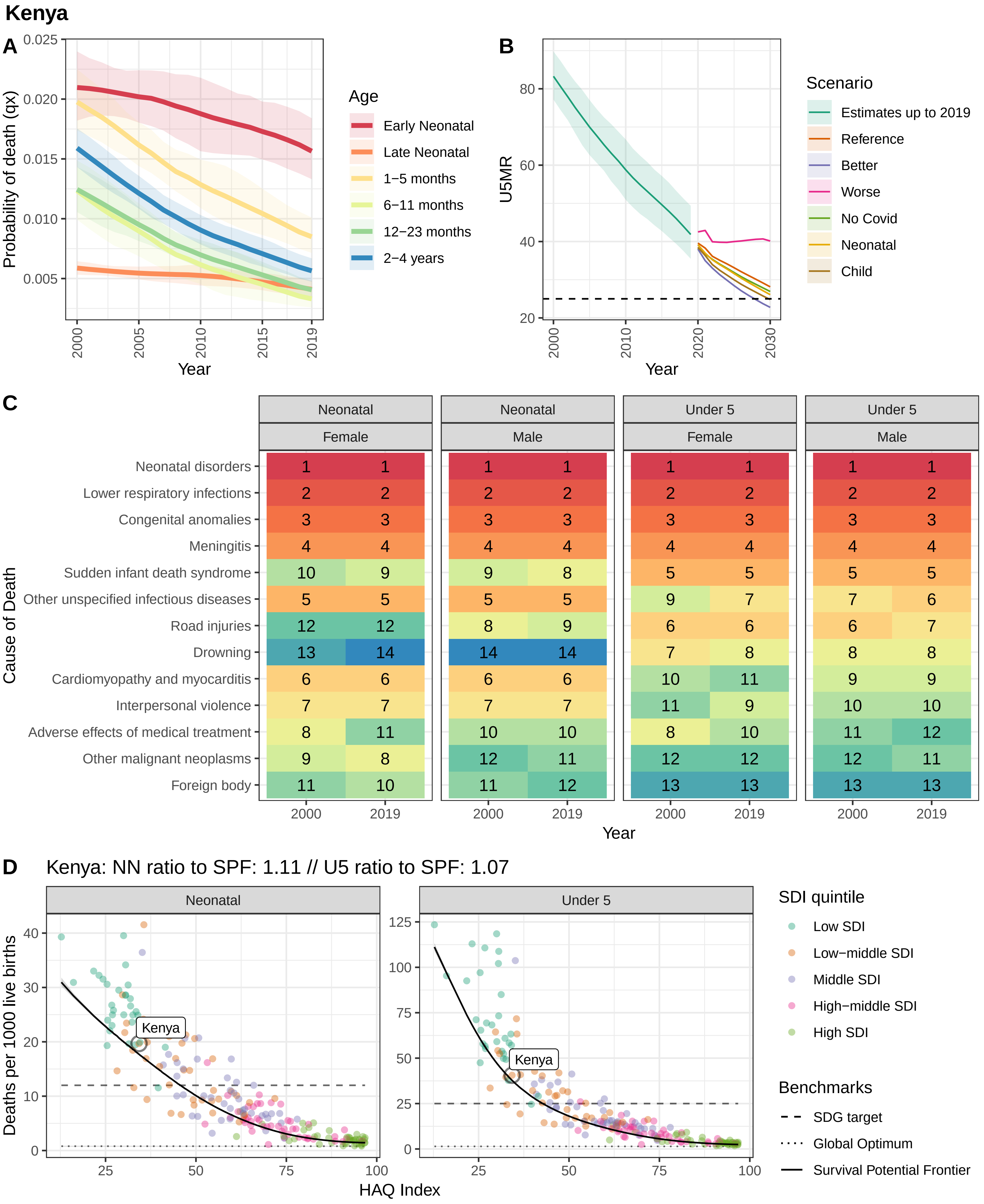
Kenya
A
Probability of death (qx)
Year
Age
Early Neonatal
Late Neonatal
1−5 months
6−11 months
12−23 months
2−4 years
B
U5MR
Year
Scenario
Estimates up to 2019
Reference
Better
Worse
No Covid
Neonatal
Child
C
Cause of Death
Neonatal disorders
Lower respiratory infections
Congenital anomalies
Meningitis
Sudden infant death syndrome
Other unspecified infectious diseases
Road injuries
Drowning
Cardiomyopathy and myocarditis
Interpersonal violence
Adverse effects of medical treatment
Other malignant neoplasms
Foreign body
Neonatal Female
Neonatal Male
Under 5 Female
Under 5 Male
Year
D
Kenya: NN ratio to SPF: 1.11 // U5 ratio to SPF: 1.07
Neonatal
Under 5
Kenya
Deaths per 1000 live births
HAQ Index
SDI quintile
Low SDI
Low−middle SDI
Middle SDI
High−middle SDI
High SDI
Benchmarks
SDG target
Global Optimum
Survival Potential Frontier

Madagascar

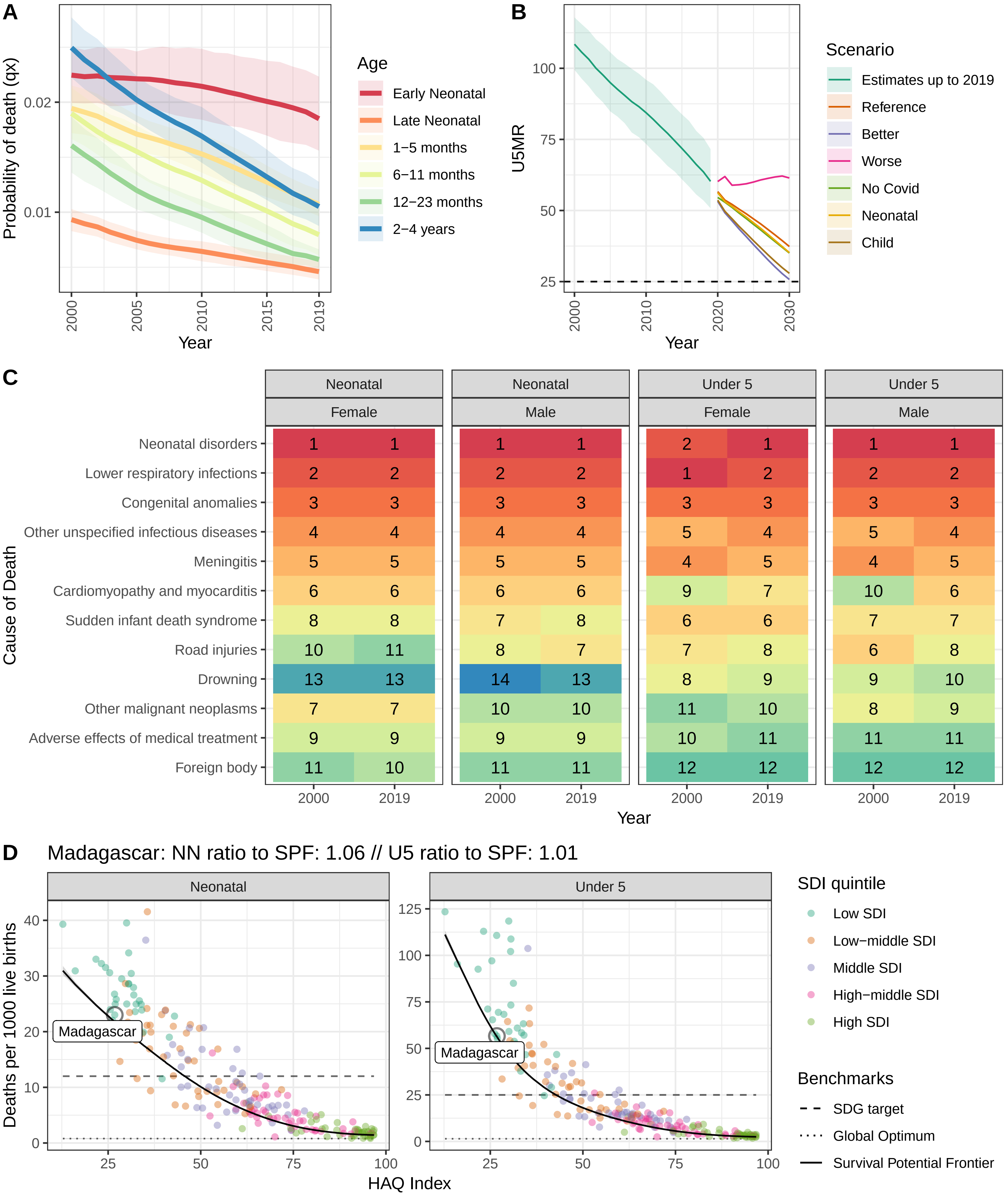
A
Probability of death (qx)
Year
Age
Early Neonatal
Late Neonatal
1−5 months
6−11 months
12−23 months
2−4 years
B
U5MR
Year
Scenario
Estimates up to 2019
Reference
Better
Worse
No Covid
Neonatal
Child
C
Neonatal
Female
Neonatal
Male
Under 5
Female
Under 5
Male
Cause of Death
Neonatal disorders
Lower respiratory infections
Congenital anomalies
Other unspecified infectious diseases
Meningitis
Cardiomyopathy and myocarditis
Sudden infant death syndrome
Road injuries
Drowning
Other malignant neoplasms
Adverse effects of medical treatment
Foreign body
Year
D
Madagascar: NN ratio to SPF: 1.06 // U5 ratio to SPF: 1.01
Neonatal
Under 5
Madagascar
Deaths per 1000 live births
HAQ Index
SDI quintile
Low SDI
Low−middle SDI
Middle SDI
High−middle SDI
High SDI
Benchmarks
SDG target
Global Optimum
Survival Potential Frontier

Malawi

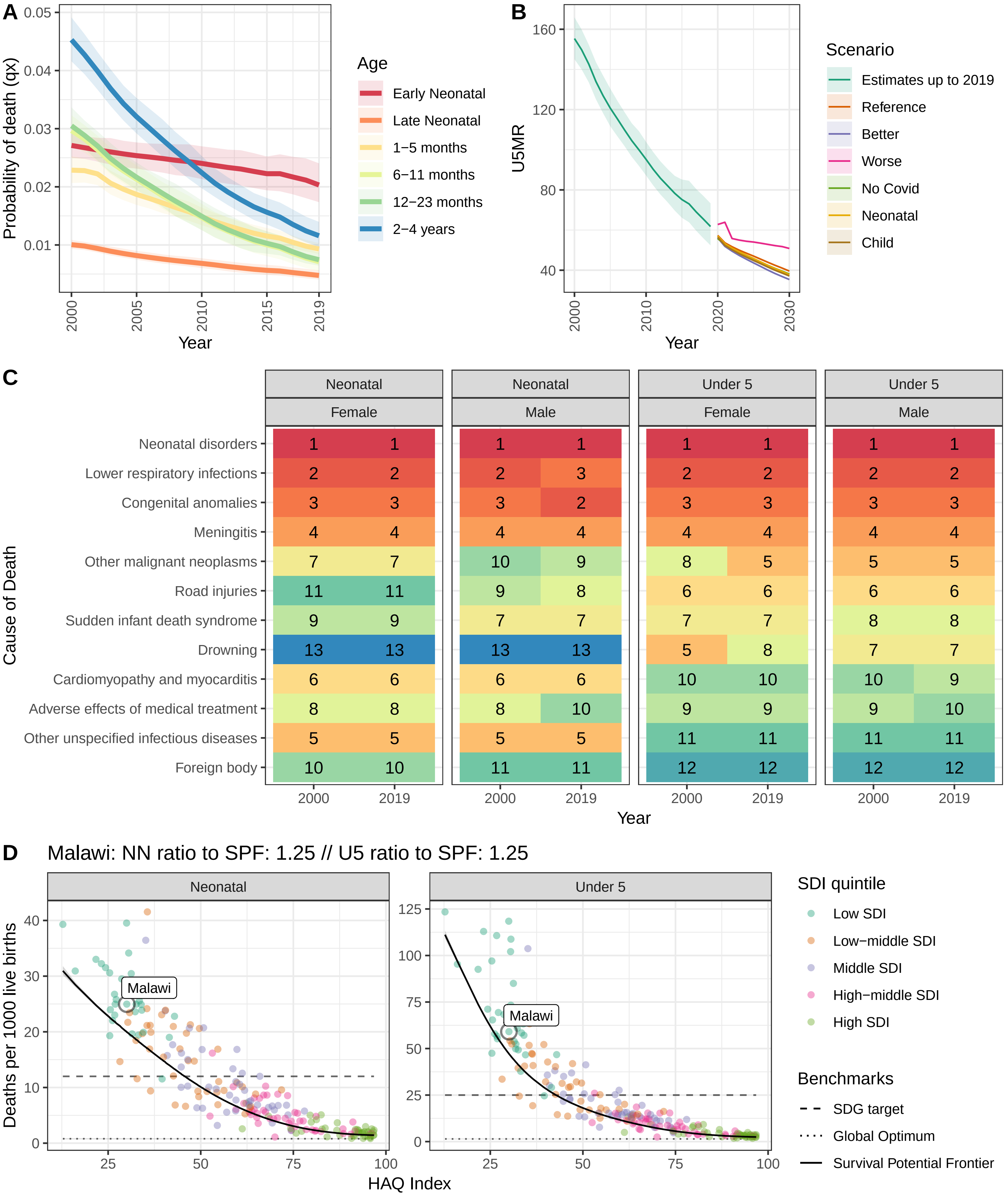
A
Probability of death (qx)
Year
Age
Early Neonatal
Late Neonatal
1−5 months
6−11 months
12−23 months
2−4 years
B
U5MR
Year
Scenario
Estimates up to 2019
Reference
Better
Worse
No Covid
Neonatal
Child
C
Neonatal
Female
Neonatal
Male
Under 5
Female
Under 5
Male
Cause of Death
Neonatal disorders 1 1 1 1 1 1 1 1
Lower respiratory infections 2 2 2 3 2 2 2 2
Congenital anomalies 3 3 3 2 3 3 3 3
Meningitis 4 4 4 4 4 4 4 4
Other malignant neoplasms 7 7 10 9 8 5 5 5
Road injuries 11 11 9 8 6 6 6 6
Sudden infant death syndrome 9 9 7 7 7 7 8 8
Drowning 13 13 13 13 5 8 7 7
Cardiomyopathy and myocarditis 6 6 6 6 10 10 10 9
Adverse effects of medical treatment 8 8 8 10 9 9 9 10
Other unspecified infectious diseases 5 5 5 5 11 11 11 11
Foreign body 10 10 11 11 12 12 12 12
2000 2019
Year
D
Malawi: NN ratio to SPF: 1.25 // U5 ratio to SPF: 1.25
Neonatal
Under 5
Malawi
Deaths per 1000 live births
HAQ Index
SDI quintile
Low SDI
Low−middle SDI
Middle SDI
High−middle SDI
High SDI
Benchmarks
SDG target
Global Optimum
Survival Potential Frontier

Mauritius

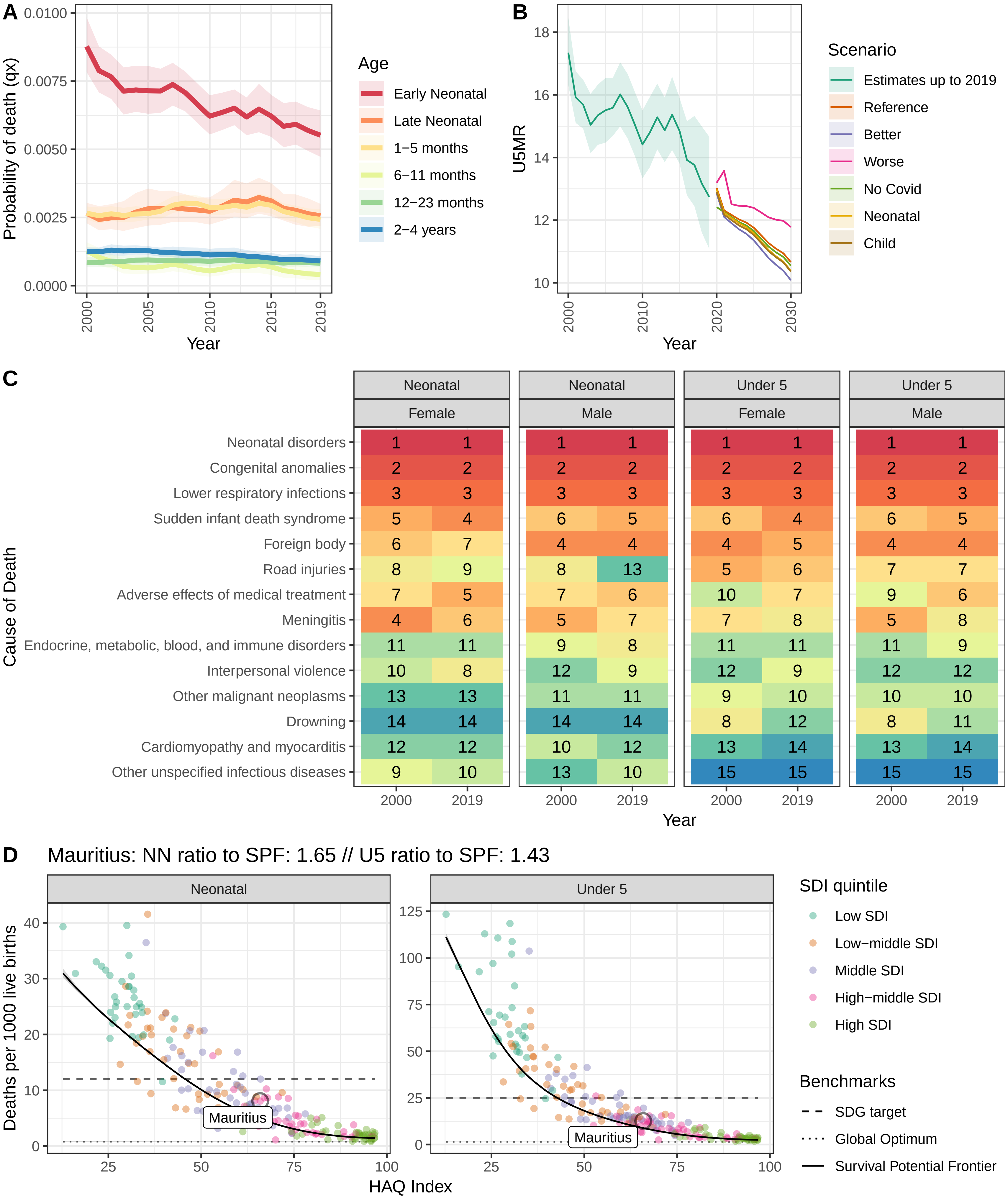

A
Probability of death (qx)
0.0100
0.0075
0.0050
0.0025
0.0000
2000
2005
2010
2015
2019
Year
Age
Early Neonatal
Late Neonatal
1−5 months
6−11 months
12−23 months
2−4 years
B
U5MR
18
16
14
12
10
2000
2010
2020
2030
Year
Scenario
Estimates up to 2019
Reference
Better
Worse
No Covid
Neonatal
Child
C
Cause of Death
Neonatal
Female
Neonatal
Male
Under 5
Female
Under 5
Male
Neonatal disorders 1 1 1 1 1 1 1 1
Congenital anomalies 2 2 2 2 2 2 2 2
Lower respiratory infections 3 3 3 3 3 3 3 3
Sudden infant death syndrome 5 4 6 5 6 4 6 5
Foreign body 6 7 4 4 4 5 4 4
Road injuries 8 9 8 13 5 6 7 7
Adverse effects of medical treatment 7 5 7 6 10 7 9 6
Meningitis 4 6 5 7 7 8 5 8
Endocrine, metabolic, blood, and immune disorders 11 11 9 8 11 11 11 9
Interpersonal violence 10 8 12 9 12 9 12 12
Other malignant neoplasms 13 13 11 11 9 10 10 10
Drowning 14 14 14 14 8 12 8 11
Cardiomyopathy and myocarditis 12 12 10 12 13 14 13 14
Other unspecified infectious diseases 9 10 13 10 15 15 15 15
2000
2019
Year
D
Mauritius: NN ratio to SPF: 1.65 // U5 ratio to SPF: 1.43
Neonatal
Under 5
Deaths per 1000 live births
40
30
20
10
0
125
100
75
50
25
0
25
50
75
100
HAQ Index
Mauritius
SDI quintile
Low SDI
Low−middle SDI
Middle SDI
High−middle SDI
High SDI
Benchmarks
SDG target
Global Optimum
Survival Potential Frontier

Mozambique

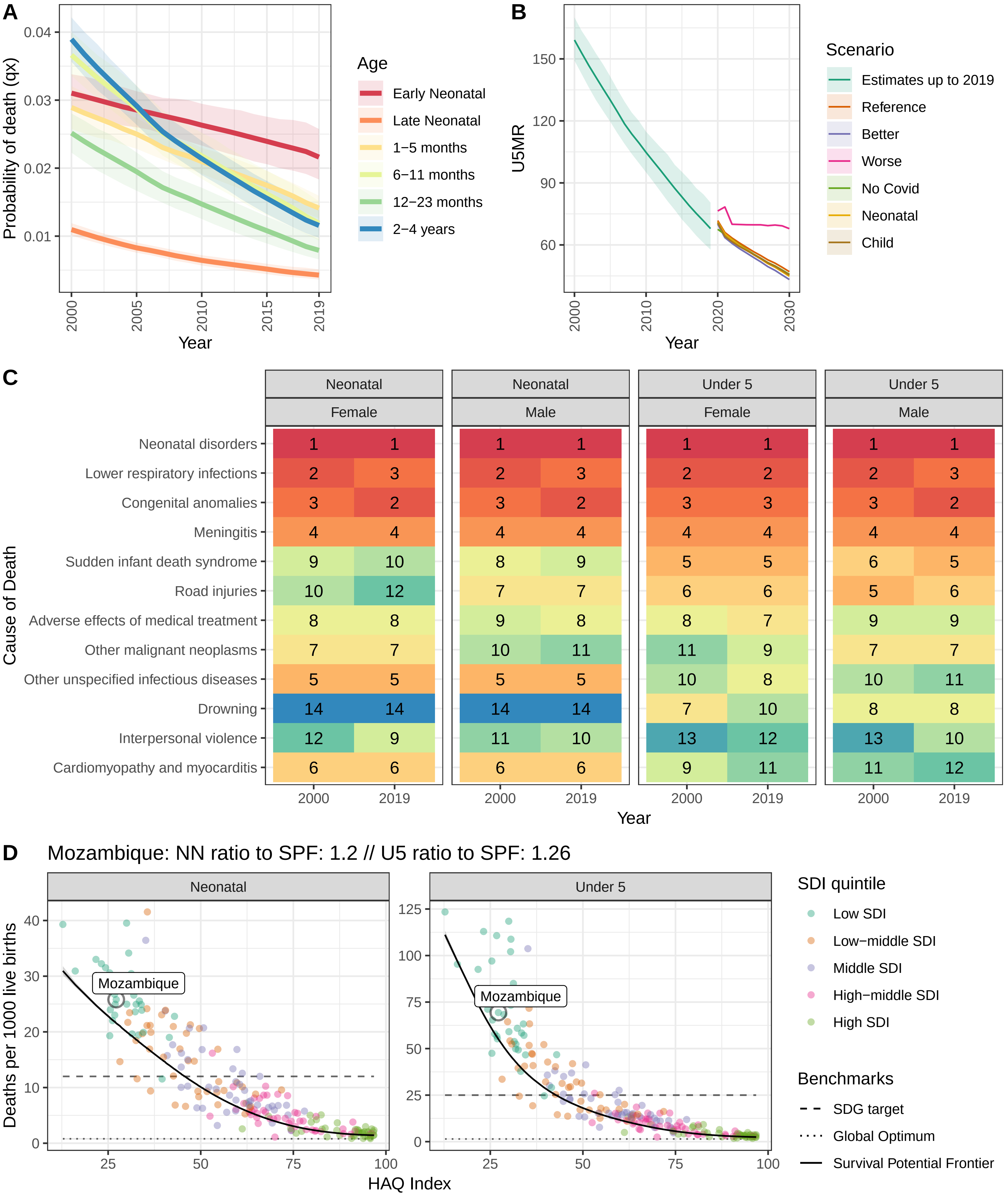
A
Probability of death (qx)
Year
Age
Early Neonatal
Late Neonatal
1−5 months
6−11 months
12−23 months
2−4 years
B
U5MR
Year
Scenario
Estimates up to 2019
Reference
Better
Worse
No Covid
Neonatal
Child
C
Cause of Death
Neonatal Female
Neonatal Male
Under 5 Female
Under 5 Male
Neonatal disorders 1 1 | 1 1 | 1 1 | 1 1
Lower respiratory infections 2 3 | 2 3 | 2 2 | 2 3
Congenital anomalies 3 2 | 3 2 | 3 3 | 3 2
Meningitis 4 4 | 4 4 | 4 4 | 4 4
Sudden infant death syndrome 9 10 | 8 9 | 5 5 | 6 5
Road injuries 10 12 | 7 7 | 6 6 | 5 6
Adverse effects of medical treatment 8 8 | 9 8 | 8 7 | 9 9
Other malignant neoplasms 7 7 | 10 11 | 11 9 | 7 7
Other unspecified infectious diseases 5 5 | 5 5 | 10 8 | 10 11
Drowning 14 14 | 14 14 | 7 10 | 8 8
Interpersonal violence 12 9 | 11 10 | 13 12 | 13 10
Cardiomyopathy and myocarditis 6 6 | 6 6 | 9 11 | 11 12
2000 2019
Year
D
Mozambique: NN ratio to SPF: 1.2 // U5 ratio to SPF: 1.26
Neonatal
Under 5
Mozambique
Deaths per 1000 live births
HAQ Index
SDI quintile
Low SDI
Low−middle SDI
Middle SDI
High−middle SDI
High SDI
Benchmarks
SDG target
Global Optimum
Survival Potential Frontier

Rwanda

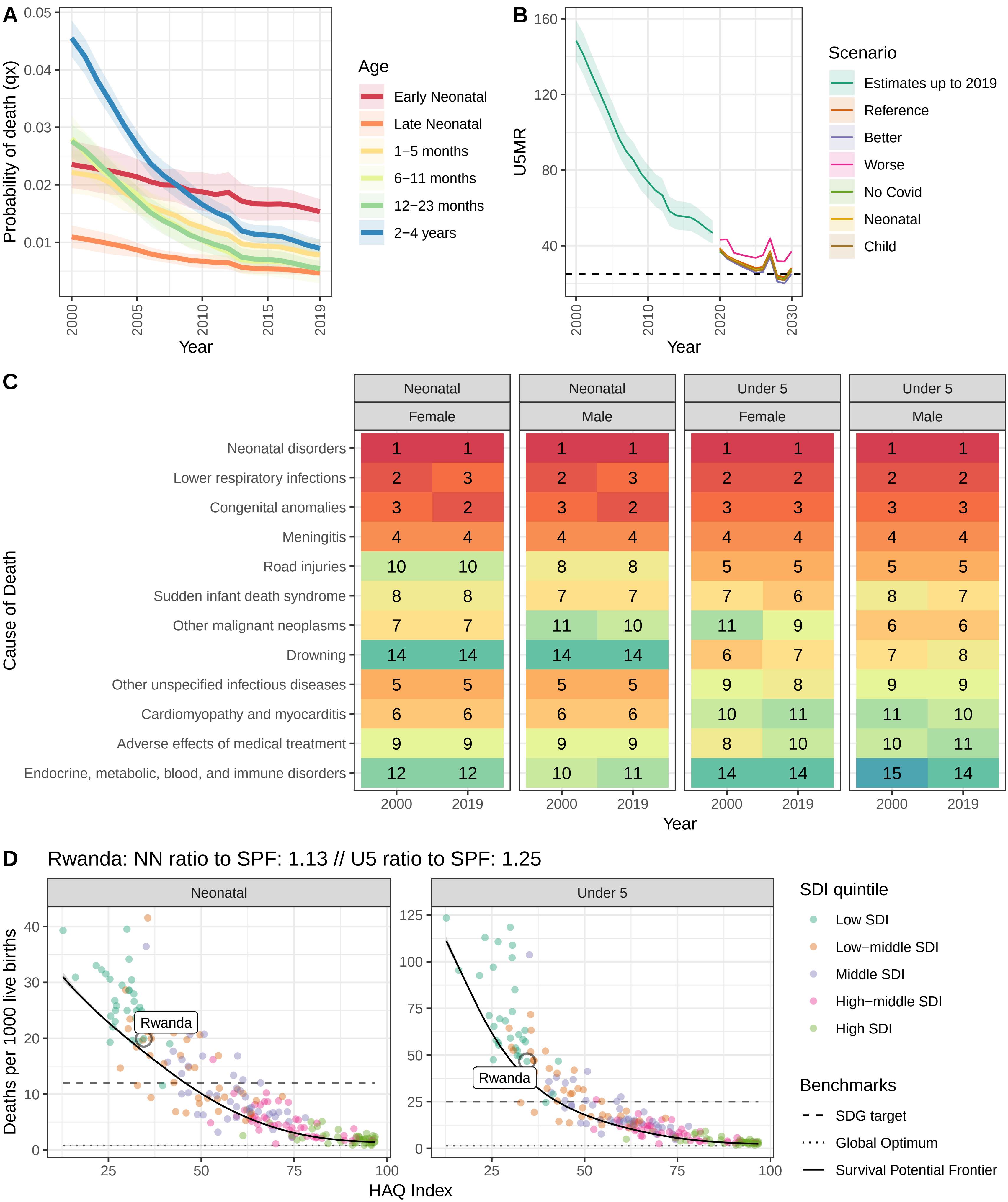

A
Probability of death (qx)
0.05
0.04
0.03
0.02
0.01
2000
2005
2010
2015
2019
Year
Age
Early Neonatal
Late Neonatal
1−5 months
6−11 months
12−23 months
2−4 years
B
U5MR
160
120
80
40
2000
2010
2020
2030
Year
Scenario
Estimates up to 2019
Reference
Better
Worse
No Covid
Neonatal
Child
C
Neonatal Female
Neonatal Male
Under 5 Female
Under 5 Male
Cause of Death
Neonatal disorders 1 1 1 1 1 1 1 1
Lower respiratory infections 2 3 2 3 2 2 2 2
Congenital anomalies 3 2 3 2 3 3 3 3
Meningitis 4 4 4 4 4 4 4 4
Road injuries 10 10 8 8 5 5 5 5
Sudden infant death syndrome 8 8 7 7 7 6 8 7
Other malignant neoplasms 7 7 11 10 11 9 6 6
Drowning 14 14 14 14 6 7 7 8
Other unspecified infectious diseases 5 5 5 5 9 8 9 9
Cardiomyopathy and myocarditis 6 6 6 6 10 11 11 10
Adverse effects of medical treatment 9 9 9 9 8 10 10 11
Endocrine, metabolic, blood, and immune disorders 12 12 10 11 14 14 15 14
2000 2019
Year
D
Rwanda: NN ratio to SPF: 1.13 // U5 ratio to SPF: 1.25
Neonatal
Under 5
Deaths per 1000 live births
Rwanda
40
30
20
10
0
125
100
75
50
25
0
25
50
75
100
HAQ Index
SDI quintile
Low SDI
Low−middle SDI
Middle SDI
High−middle SDI
High SDI
Benchmarks
SDG target
Global Optimum
Survival Potential Frontier

Seychelles

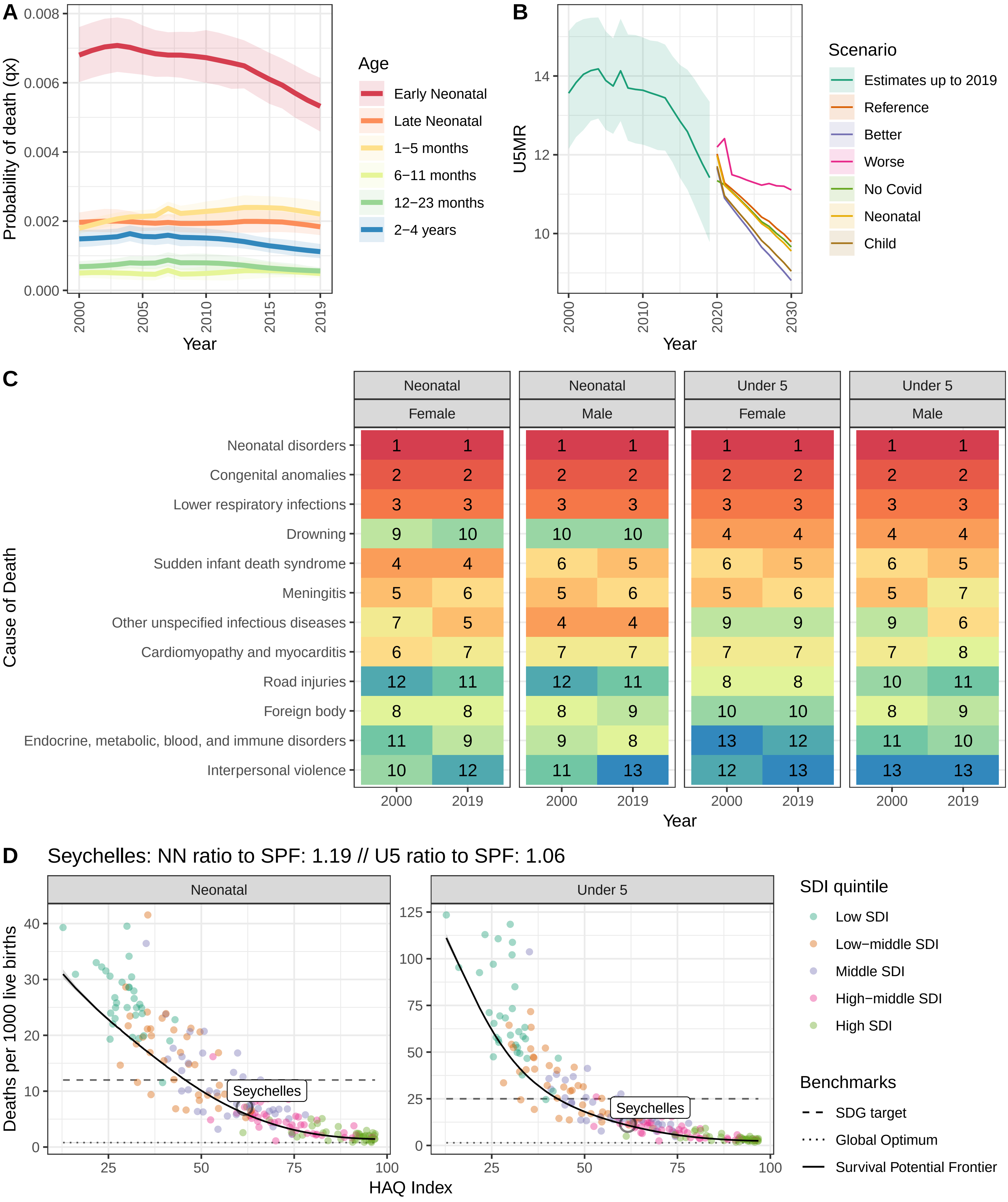
A
Probability of death (qx)
Year
Age
Early Neonatal
Late Neonatal
1−5 months
6−11 months
12−23 months
2−4 years
B
U5MR
Year
Scenario
Estimates up to 2019
Reference
Better
Worse
No Covid
Neonatal
Child
C
Neonatal
Female
Neonatal
Male
Under 5
Female
Under 5
Male
Cause of Death
Neonatal disorders
Congenital anomalies
Lower respiratory infections
Drowning
Sudden infant death syndrome
Meningitis
Other unspecified infectious diseases
Cardiomyopathy and myocarditis
Road injuries
Foreign body
Endocrine, metabolic, blood, and immune disorders
Interpersonal violence
Year
D
Seychelles: NN ratio to SPF: 1.19 // U5 ratio to SPF: 1.06
Neonatal
Under 5
Deaths per 1000 live births
HAQ Index
Seychelles
SDI quintile
Low SDI
Low−middle SDI
Middle SDI
High−middle SDI
High SDI
Benchmarks
SDG target
Global Optimum
Survival Potential Frontier

Somalia

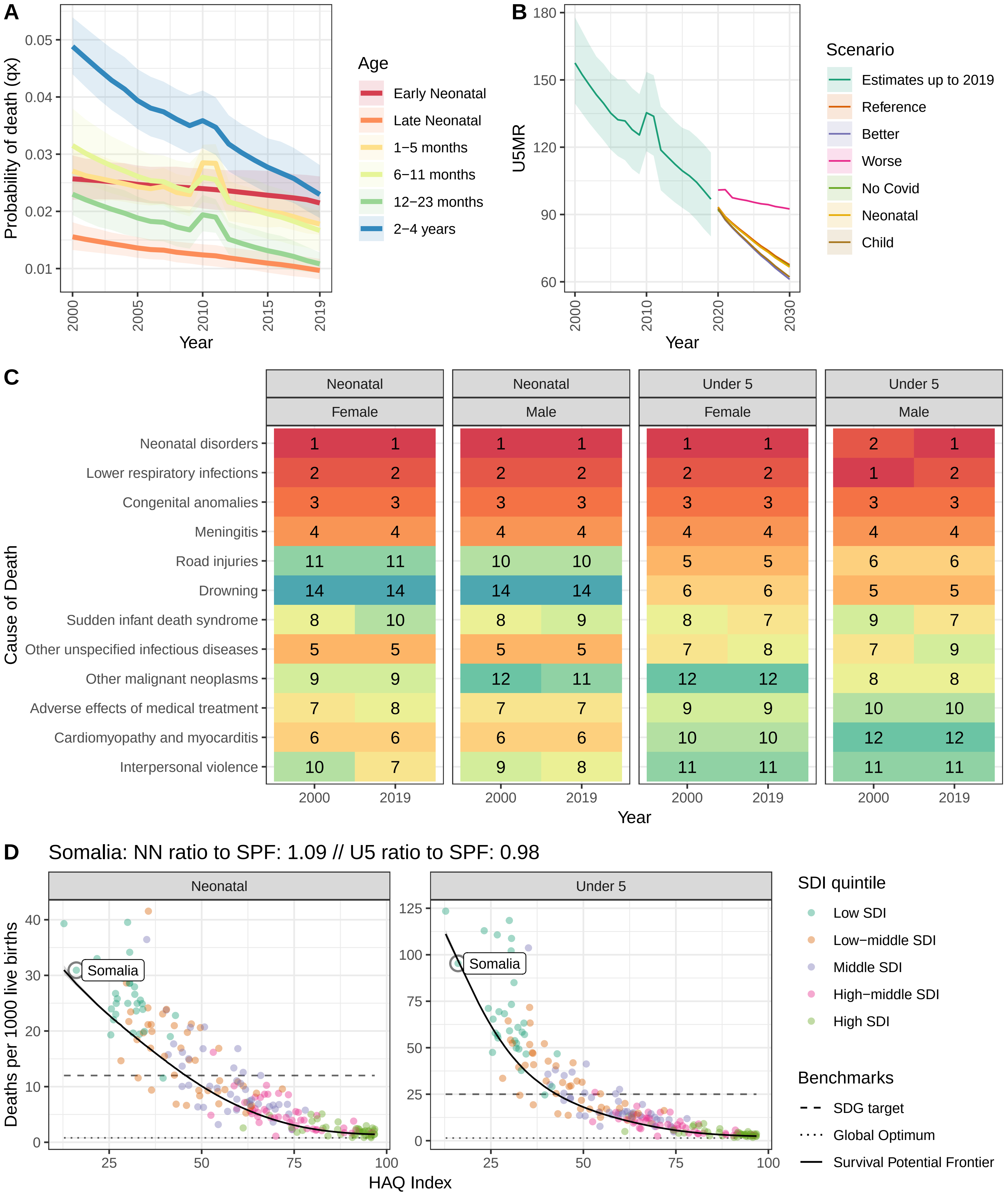

A
Probability of death (qx)
Year
Age
Early Neonatal
Late Neonatal
1−5 months
6−11 months
12−23 months
2−4 years
B
U5MR
Year
Scenario
Estimates up to 2019
Reference
Better
Worse
No Covid
Neonatal
Child
C
Neonatal
Female
Neonatal
Male
Under 5
Female
Under 5
Male
Cause of Death
Neonatal disorders
Lower respiratory infections
Congenital anomalies
Meningitis
Road injuries
Drowning
Sudden infant death syndrome
Other unspecified infectious diseases
Other malignant neoplasms
Adverse effects of medical treatment
Cardiomyopathy and myocarditis
Interpersonal violence
Year
D
Somalia: NN ratio to SPF: 1.09 // U5 ratio to SPF: 0.98
Neonatal
Under 5
Somalia
Deaths per 1000 live births
HAQ Index
SDI quintile
Low SDI
Low−middle SDI
Middle SDI
High−middle SDI
High SDI
Benchmarks
SDG target
Global Optimum
Survival Potential Frontier

United Republic of Tanzania

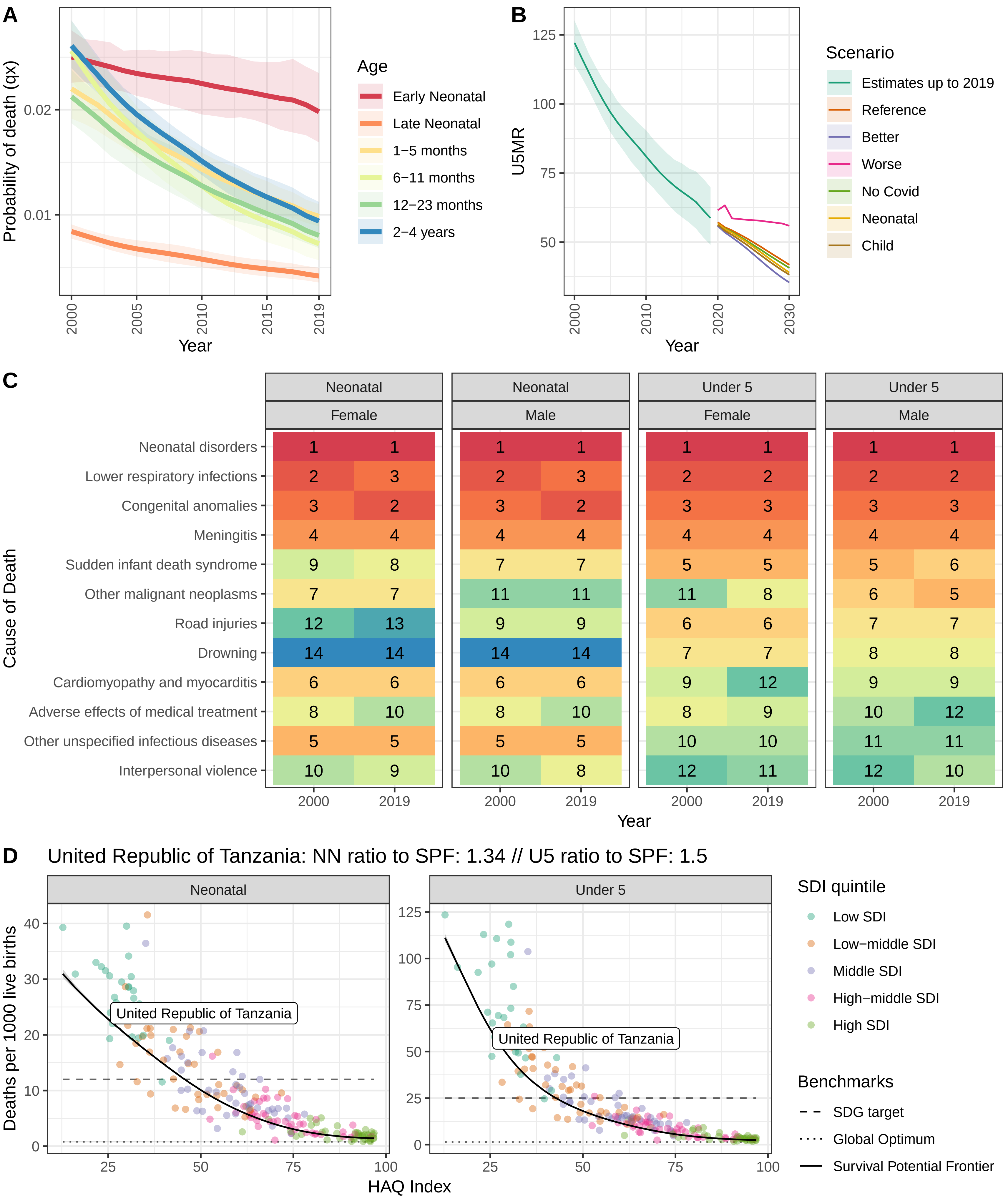

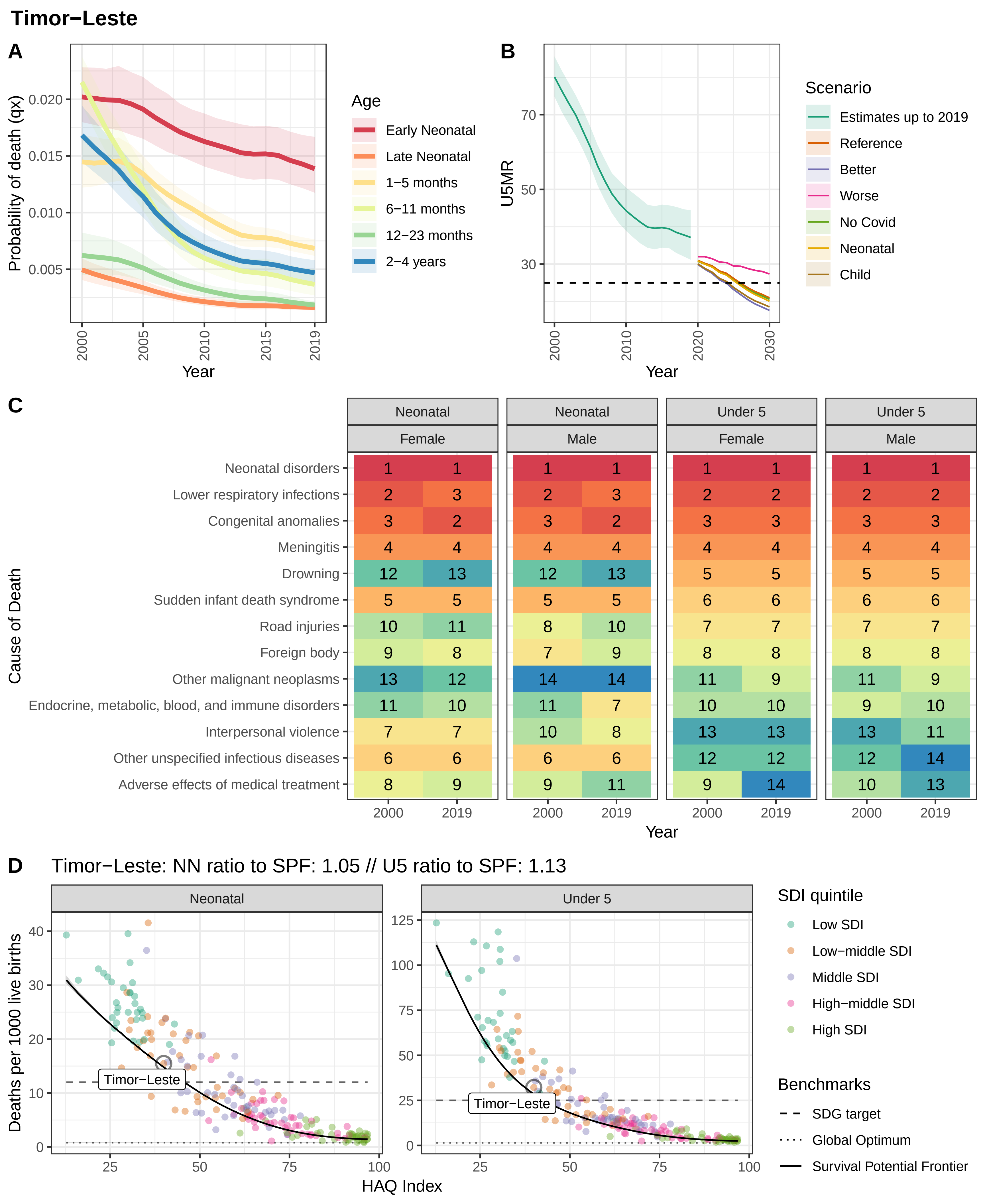
Timor−Leste
A
Probability of death (qx)
0.020
0.015
0.010
0.005
2000
2005
2010
2015
2019
Year
Age
Early Neonatal
Late Neonatal
1−5 months
6−11 months
12−23 months
2−4 years
B
U5MR
70
50
30
2000
2010
2020
2030
Year
Scenario
Estimates up to 2019
Reference
Better
Worse
No Covid
Neonatal
Child
C
Cause of Death
Neonatal Female
Neonatal Male
Under 5 Female
Under 5 Male
Neonatal disorders 1 1 1 1 1 1 1 1
Lower respiratory infections 2 3 2 3 2 2 2 2
Congenital anomalies 3 2 3 2 3 3 3 3
Meningitis 4 4 4 4 4 4 4 4
Drowning 12 13 12 13 5 5 5 5
Sudden infant death syndrome 5 5 5 5 6 6 6 6
Road injuries 10 11 8 10 7 7 7 7
Foreign body 9 8 7 9 8 8 8 8
Other malignant neoplasms 13 12 14 14 11 9 11 9
Endocrine, metabolic, blood, and immune disorders 11 10 11 7 10 10 9 10
Interpersonal violence 7 7 10 8 13 13 13 11
Other unspecified infectious diseases 6 6 6 6 12 12 12 14
Adverse effects of medical treatment 8 9 9 11 9 14 10 13
2000 2019 2000 2019 2000 2019 2000 2019
Year
D
Timor−Leste: NN ratio to SPF: 1.05 // U5 ratio to SPF: 1.13
Neonatal
Under 5
Deaths per 1000 live births
40
30
20
10
0
125
100
75
50
25
0
Timor−Leste
Timor−Leste
25
50
75
100
HAQ Index
SDI quintile
Low SDI
Low−middle SDI
Middle SDI
High−middle SDI
High SDI
Benchmarks
SDG target
Global Optimum
Survival Potential Frontier

Uganda

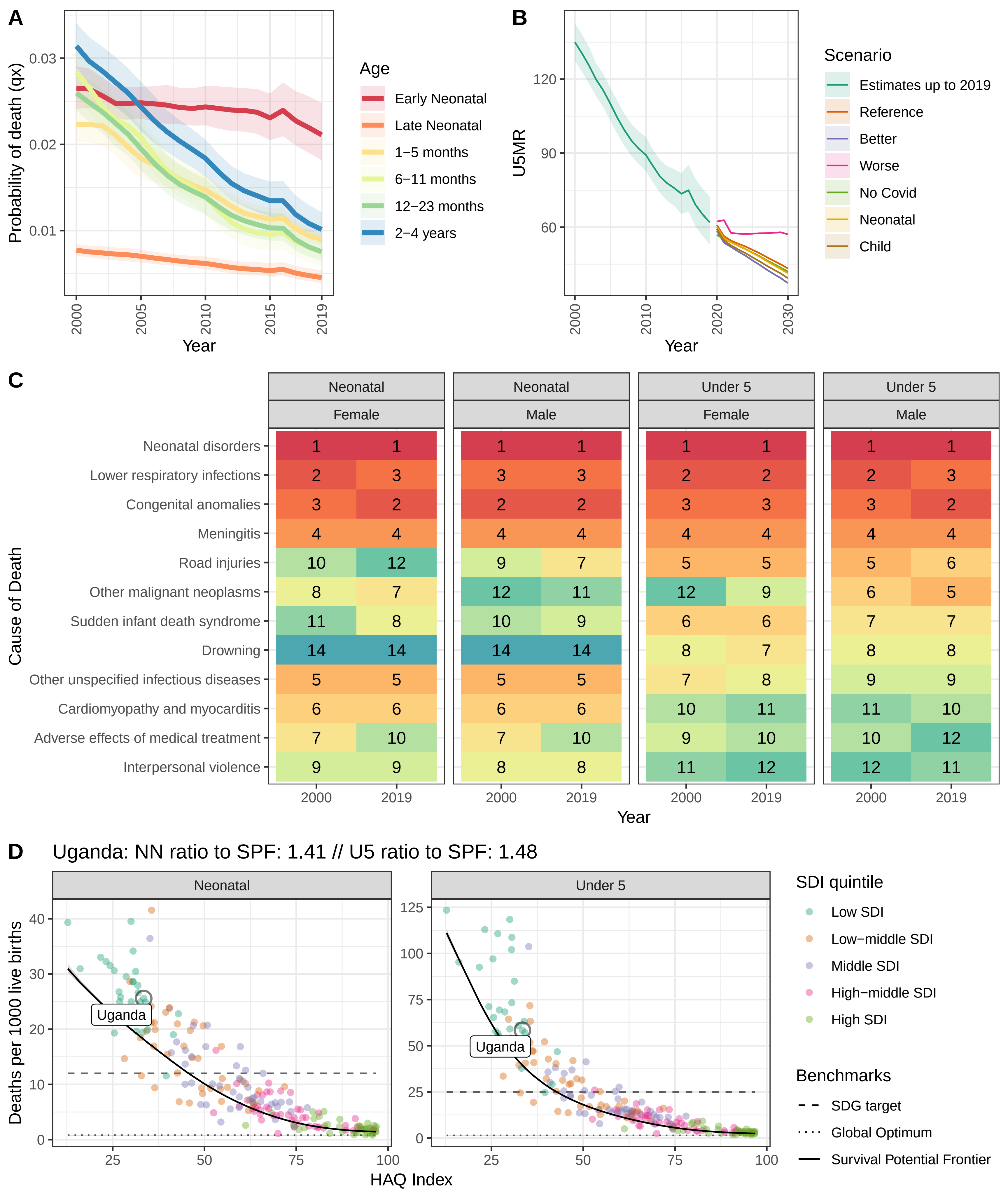
A
Probability of death (qx)
Year
Age
Early Neonatal
Late Neonatal
1−5 months
6−11 months
12−23 months
2−4 years
B
U5MR
Year
Scenario
Estimates up to 2019
Reference
Better
Worse
No Covid
Neonatal
Child
C
Cause of Death
Neonatal Female
Neonatal Male
Under 5 Female
Under 5 Male
Neonatal disorders
Lower respiratory infections
Congenital anomalies
Meningitis
Road injuries
Other malignant neoplasms
Sudden infant death syndrome
Drowning
Other unspecified infectious diseases
Cardiomyopathy and myocarditis
Adverse effects of medical treatment
Interpersonal violence
Year
D
Uganda: NN ratio to SPF: 1.41 // U5 ratio to SPF: 1.48
Neonatal
Under 5
Uganda
Deaths per 1000 live births
HAQ Index
SDI quintile
Low SDI
Low−middle SDI
Middle SDI
High−middle SDI
High SDI
Benchmarks
SDG target
Global Optimum
Survival Potential Frontier

Zambia

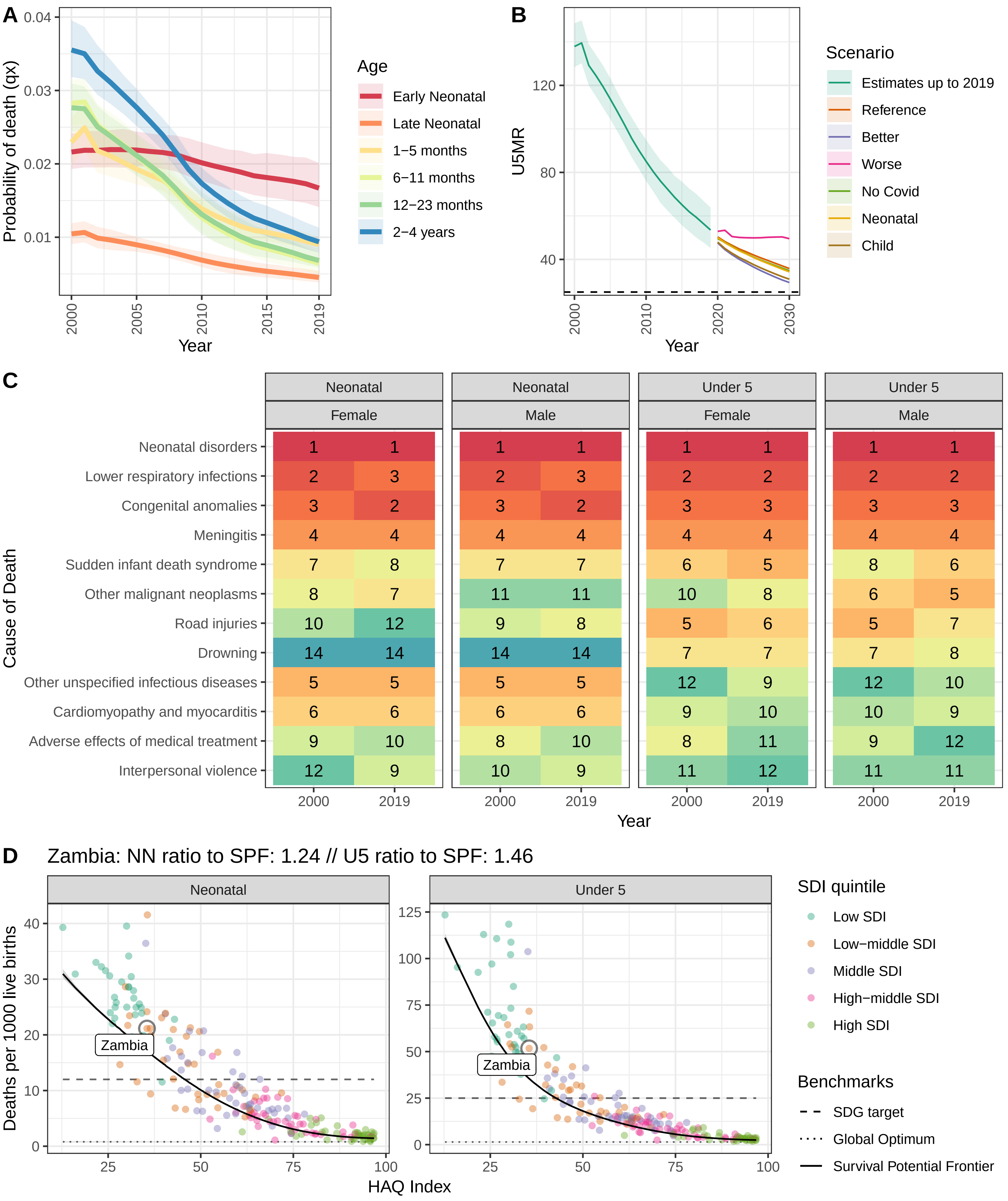
A
Probability of death (qx)
0.04
0.03
0.02
0.01
2000
2005
2010
2015
2019
Year
Age
Early Neonatal
Late Neonatal
1−5 months
6−11 months
12−23 months
2−4 years
B
U5MR
120
80
40
2000
2010
2020
2030
Year
Scenario
Estimates up to 2019
Reference
Better
Worse
No Covid
Neonatal
Child
C
Cause of Death
Neonatal
Female
Neonatal
Male
Under 5
Female
Under 5
Male
Neonatal disorders 1 1 1 1 1 1 1 1
Lower respiratory infections 2 3 2 3 2 2 2 2
Congenital anomalies 3 2 3 2 3 3 3 3
Meningitis 4 4 4 4 4 4 4 4
Sudden infant death syndrome 7 8 7 7 6 5 8 6
Other malignant neoplasms 8 7 11 11 10 8 6 5
Road injuries 10 12 9 8 5 6 5 7
Drowning 14 14 14 14 7 7 7 8
Other unspecified infectious diseases 5 5 5 5 12 9 12 10
Cardiomyopathy and myocarditis 6 6 6 6 9 10 10 9
Adverse effects of medical treatment 9 10 8 10 8 11 9 12
Interpersonal violence 12 9 10 9 11 12 11 11
2000
2019
Year
D
Zambia: NN ratio to SPF: 1.24 // U5 ratio to SPF: 1.46
Neonatal
Under 5
Deaths per 1000 live births
40
30
20
10
0
125
100
75
50
25
Zambia
Zambia
HAQ Index
SDI quintile
Low SDI
Low−middle SDI
Middle SDI
High−middle SDI
High SDI
Benchmarks
SDG target
Global Optimum
Survival Potential Frontier

Botswana

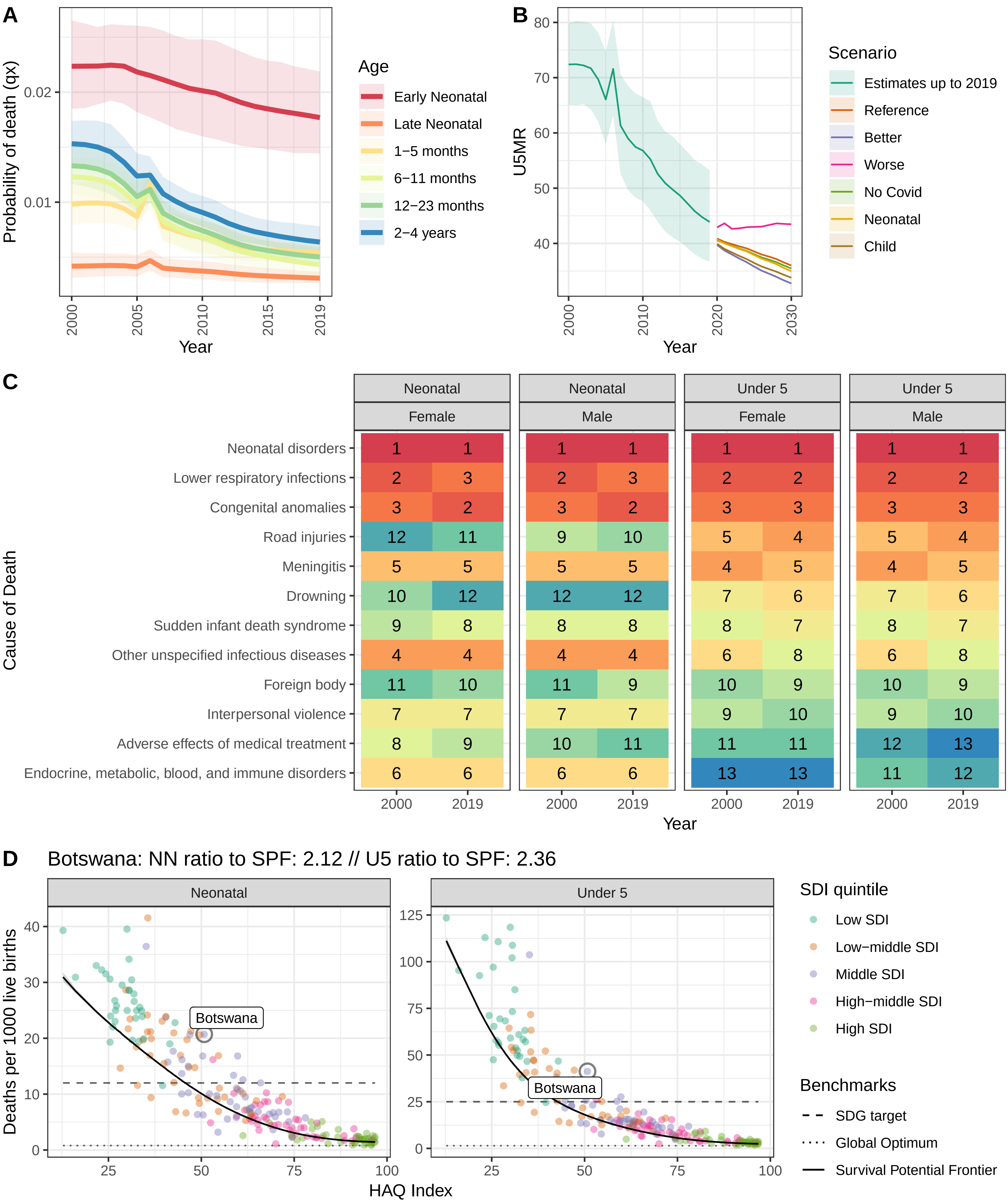
A
Probability of death (qx)
0.02
0.01
2000
2005
2010
2015
2019
Year
Age
Early Neonatal
Late Neonatal
1−5 months
6−11 months
12−23 months
2−4 years
B
U5MR
80
70
60
50
40
2000
2010
2020
2030
Year
Scenario
Estimates up to 2019
Reference
Better
Worse
No Covid
Neonatal
Child
C
Neonatal Female
Neonatal Male
Under 5 Female
Under 5 Male
Cause of Death
Neonatal disorders 1 1 1 1 1 1 1 1
Lower respiratory infections 2 3 2 3 2 2 2 2
Congenital anomalies 3 2 3 2 3 3 3 3
Road injuries 12 11 9 10 5 4 5 4
Meningitis 5 5 5 5 4 5 4 5
Drowning 10 12 12 12 7 6 7 6
Sudden infant death syndrome 9 8 8 8 8 7 8 7
Other unspecified infectious diseases 4 4 4 4 6 8 6 8
Foreign body 11 10 11 9 10 9 10 9
Interpersonal violence 7 7 7 7 9 10 9 10
Adverse effects of medical treatment 8 9 10 11 11 11 12 13
Endocrine, metabolic, blood, and immune disorders 6 6 6 6 13 13 11 12
2000
2019
Year
D
Botswana: NN ratio to SPF: 2.12 // U5 ratio to SPF: 2.36
Neonatal
Under 5
Deaths per 1000 live births
40
30
20
10
0
125
100
75
50
25
0
Botswana
Botswana
25
50
75
100
HAQ Index
SDI quintile
Low SDI
Low−middle SDI
Middle SDI
High−middle SDI
High SDI
Benchmarks
SDG target
Global Optimum
Survival Potential Frontier

Lesotho

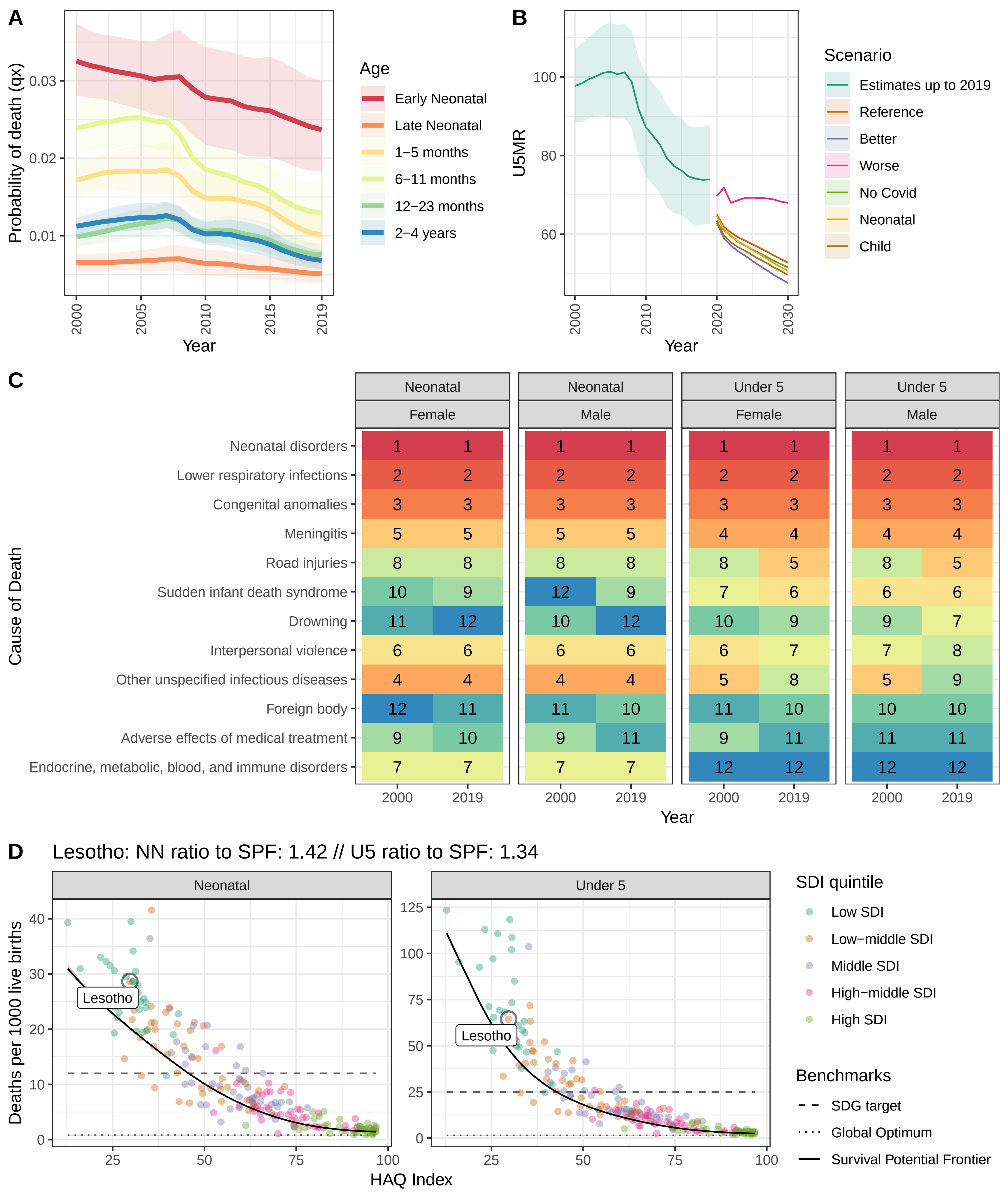
A
Probability of death (qx)
0.03
0.02
0.01
2000
2005
2010
2015
2019
Year
Age
Early Neonatal
Late Neonatal
1−5 months
6−11 months
12−23 months
2−4 years
B
U5MR
100
80
60
2000
2010
2020
2030
Year
Scenario
Estimates up to 2019
Reference
Better
Worse
No Covid
Neonatal
Child
C
Neonatal
Female
Neonatal
Male
Under 5
Female
Under 5
Male
Cause of Death
Neonatal disorders 1 1 1 1 1 1 1 1
Lower respiratory infections 2 2 2 2 2 2 2 2
Congenital anomalies 3 3 3 3 3 3 3 3
Meningitis 5 5 5 5 4 4 4 4
Road injuries 8 8 8 8 8 5 8 5
Sudden infant death syndrome 10 9 12 9 7 6 6 6
Drowning 11 12 10 12 10 9 9 7
Interpersonal violence 6 6 6 6 6 7 7 8
Other unspecified infectious diseases 4 4 4 4 5 8 5 9
Foreign body 12 11 11 10 11 10 10 10
Adverse effects of medical treatment 9 10 9 11 9 11 11 11
Endocrine, metabolic, blood, and immune disorders 7 7 7 7 12 12 12 12
2000
2019
Year
D
Lesotho: NN ratio to SPF: 1.42 // U5 ratio to SPF: 1.34
Neonatal
Under 5
Deaths per 1000 live births
40
30
20
10
0
125
100
75
50
25
0
Lesotho
Lesotho
25
50
75
100
HAQ Index
SDI quintile
Low SDI
Low−middle SDI
Middle SDI
High−middle SDI
High SDI
Benchmarks
SDG target
Global Optimum
Survival Potential Frontier

Namibia

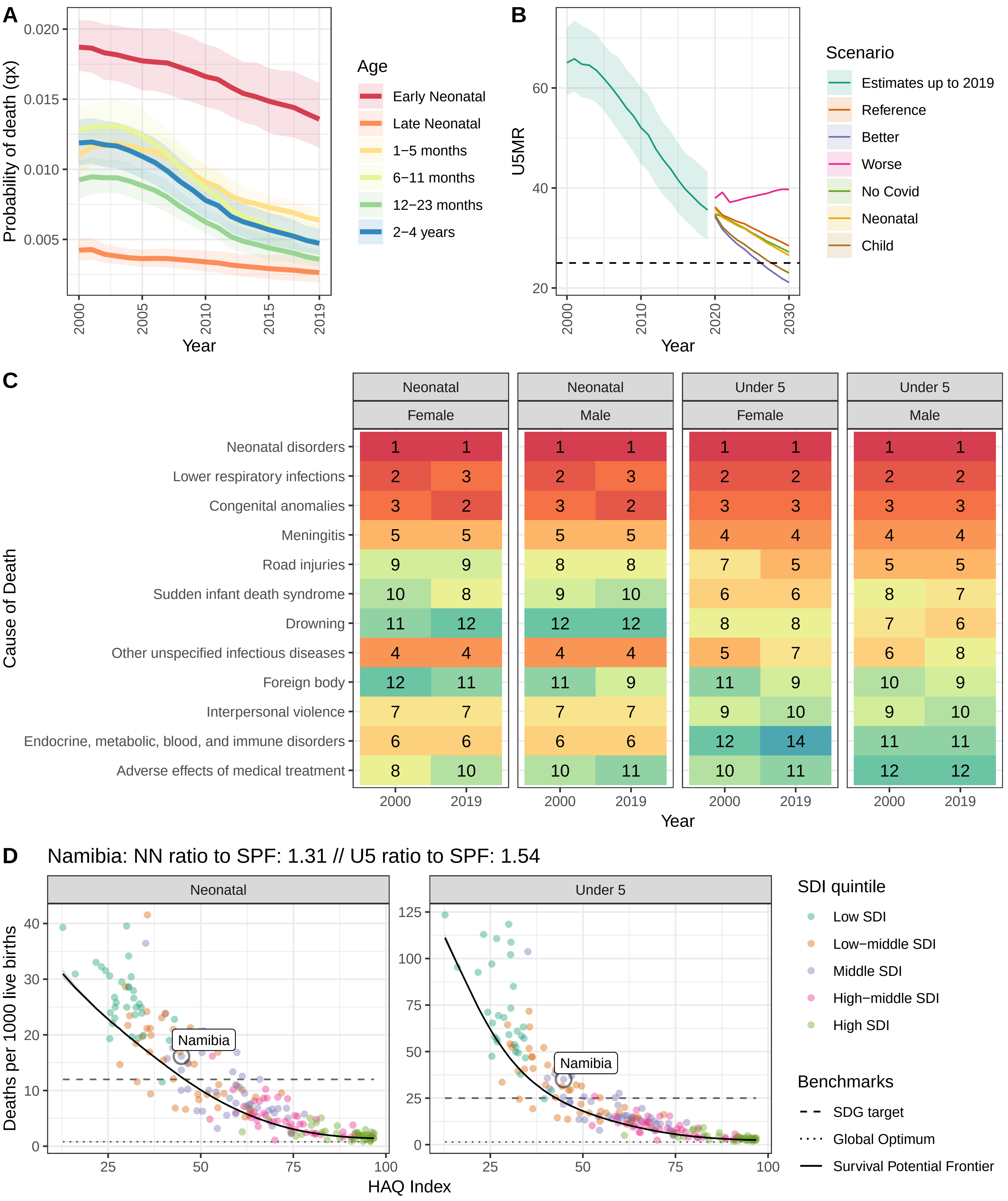
A
Probability of death (qx)
0.020
0.015
0.010
0.005
2000
2005
2010
2015
2019
Year
Age
Early Neonatal
Late Neonatal
1−5 months
6−11 months
12−23 months
2−4 years
B
U5MR
60
40
20
2000
2010
2020
2030
Year
Scenario
Estimates up to 2019
Reference
Better
Worse
No Covid
Neonatal
Child
C
Neonatal Female
Neonatal Male
Under 5 Female
Under 5 Male
Cause of Death
Neonatal disorders 1 1 1 1 1 1 1 1
Lower respiratory infections 2 3 2 3 2 2 2 2
Congenital anomalies 3 2 3 2 3 3 3 3
Meningitis 5 5 5 5 4 4 4 4
Road injuries 9 9 8 8 7 5 5 5
Sudden infant death syndrome 10 8 9 10 6 6 8 7
Drowning 11 12 12 12 8 8 7 6
Other unspecified infectious diseases 4 4 4 4 5 7 6 8
Foreign body 12 11 11 9 11 9 10 9
Interpersonal violence 7 7 7 7 9 10 9 10
Endocrine, metabolic, blood, and immune disorders 6 6 6 6 12 14 11 11
Adverse effects of medical treatment 8 10 10 11 10 11 12 12
2000
2019
Year
D
Namibia: NN ratio to SPF: 1.31 // U5 ratio to SPF: 1.54
Neonatal
Under 5
Deaths per 1000 live births
40
30
20
10
0
125
100
75
50
25
Namibia
HAQ Index
SDI quintile
Low SDI
Low−middle SDI
Middle SDI
High−middle SDI
High SDI
Benchmarks
SDG target
Global Optimum
Survival Potential Frontier

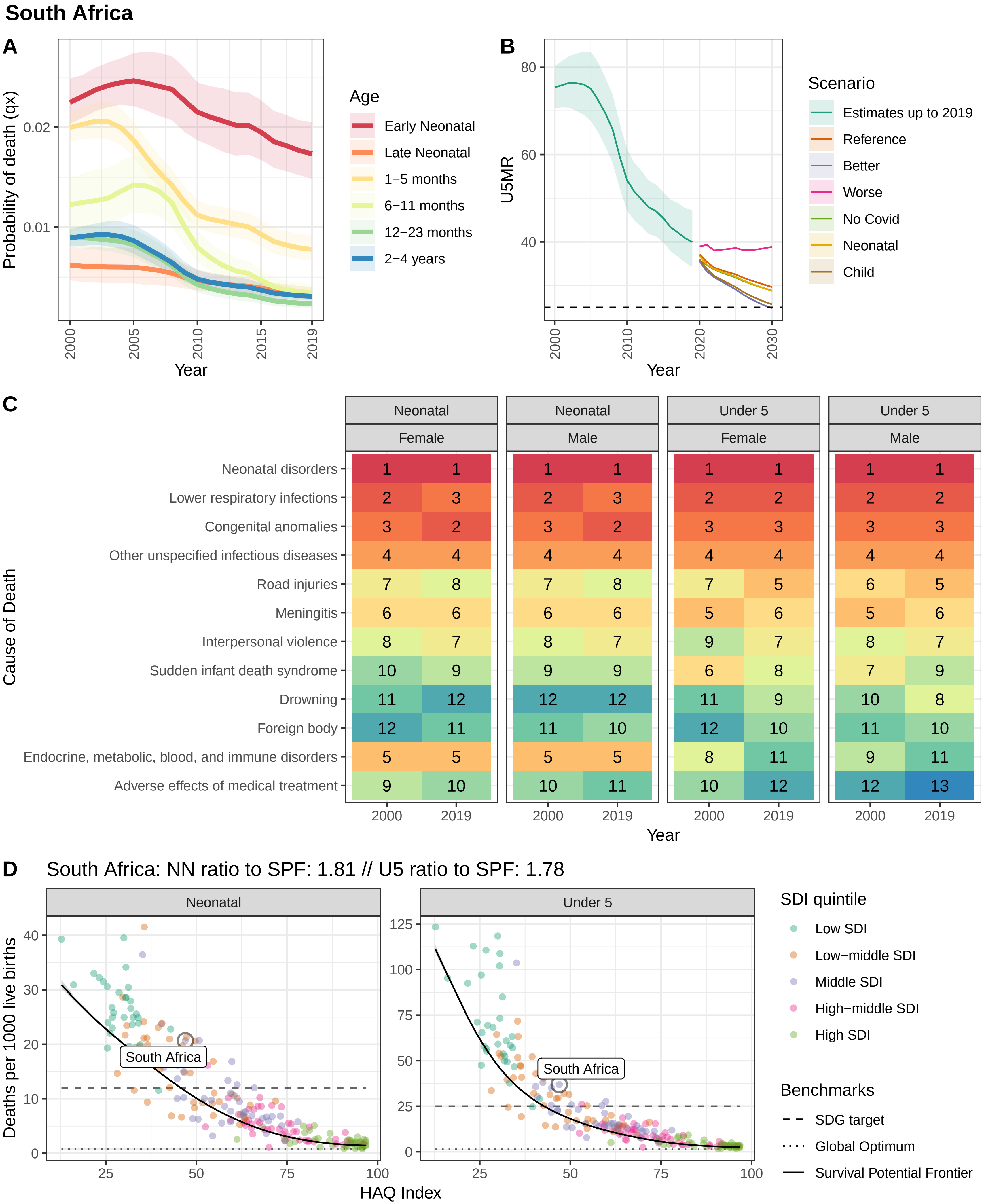
South Africa
A
Probability of death (qx)
0.02
0.01
2000
2005
2010
2015
2019
Year
Age
Early Neonatal
Late Neonatal
1−5 months
6−11 months
12−23 months
2−4 years
B
U5MR
80
60
40
2000
2010
2020
2030
Year
Scenario
Estimates up to 2019
Reference
Better
Worse
No Covid
Neonatal
Child
C
Neonatal Female
Neonatal Male
Under 5 Female
Under 5 Male
Cause of Death
Neonatal disorders 1 1 1 1 1 1 1 1
Lower respiratory infections 2 3 2 3 2 2 2 2
Congenital anomalies 3 2 3 2 3 3 3 3
Other unspecified infectious diseases 4 4 4 4 4 4 4 4
Road injuries 7 8 7 8 7 5 6 5
Meningitis 6 6 6 6 5 6 5 6
Interpersonal violence 8 7 8 7 9 7 8 7
Sudden infant death syndrome 10 9 9 9 6 8 7 9
Drowning 11 12 12 12 11 9 10 8
Foreign body 12 11 11 10 12 10 11 10
Endocrine, metabolic, blood, and immune disorders 5 5 5 5 8 11 9 11
Adverse effects of medical treatment 9 10 10 11 10 12 12 13
2000 2019 2000 2019 2000 2019 2000 2019
Year
D
South Africa: NN ratio to SPF: 1.81 // U5 ratio to SPF: 1.78
Neonatal
Under 5
Deaths per 1000 live births
40
30
20
10
0
125
100
75
50
25
0
South Africa
South Africa
25
50
75
100
HAQ Index
SDI quintile
Low SDI
Low−middle SDI
Middle SDI
High−middle SDI
High SDI
Benchmarks
SDG target
Global Optimum
Survival Potential Frontier

Eswatini

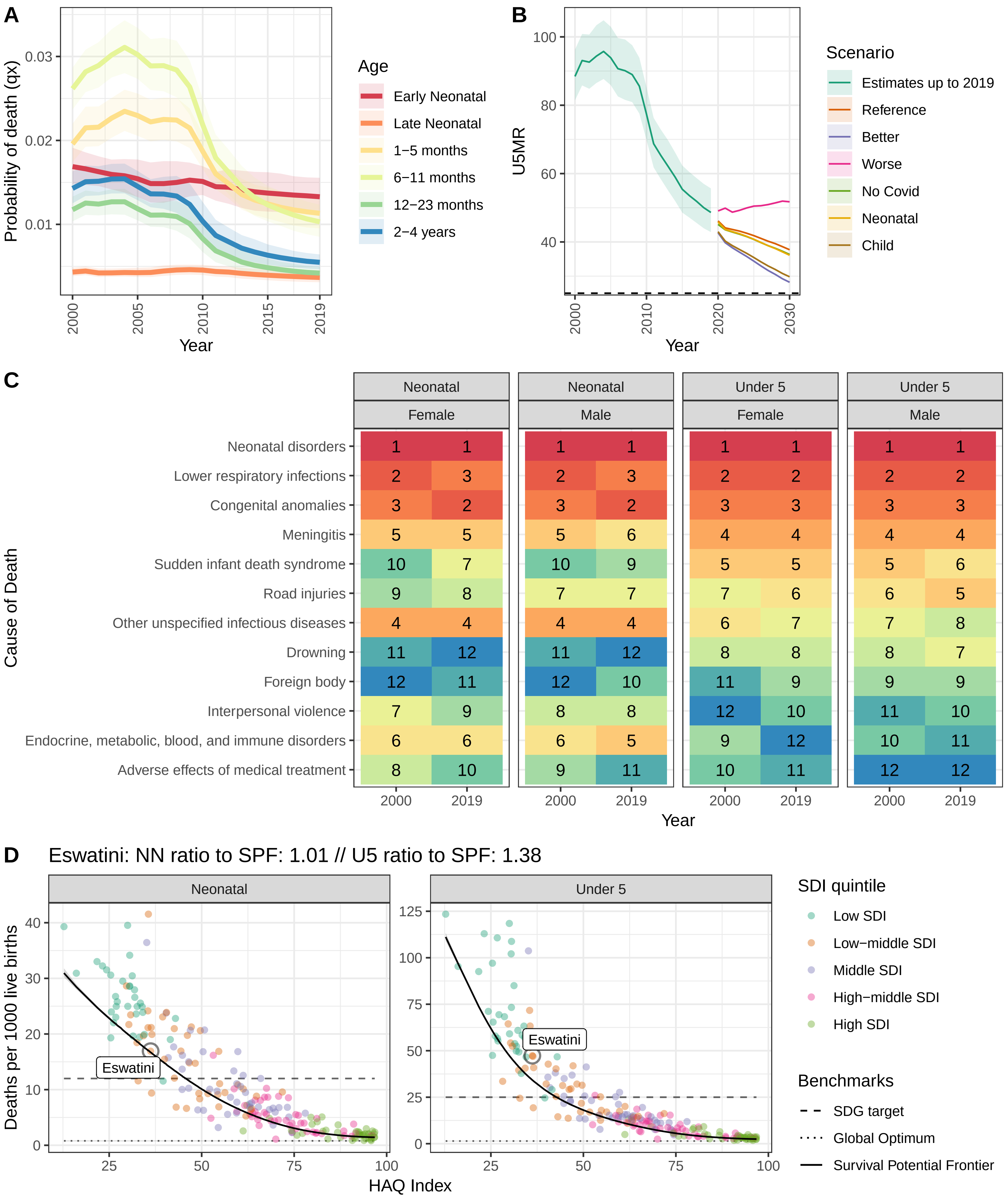
A
Probability of death (qx)
Year
Age
Early Neonatal
Late Neonatal
1−5 months
6−11 months
12−23 months
2−4 years
B
U5MR
Year
Scenario
Estimates up to 2019
Reference
Better
Worse
No Covid
Neonatal
Child
C
Neonatal Female
Neonatal Male
Under 5 Female
Under 5 Male
Cause of Death
Neonatal disorders
Lower respiratory infections
Congenital anomalies
Meningitis
Sudden infant death syndrome
Road injuries
Other unspecified infectious diseases
Drowning
Foreign body
Interpersonal violence
Endocrine, metabolic, blood, and immune disorders
Adverse effects of medical treatment
Year
D
Eswatini: NN ratio to SPF: 1.01 // U5 ratio to SPF: 1.38
Neonatal
Under 5
Deaths per 1000 live births
HAQ Index
Eswatini
SDI quintile
Low SDI
Low−middle SDI
Middle SDI
High−middle SDI
High SDI
Benchmarks
SDG target
Global Optimum
Survival Potential Frontier

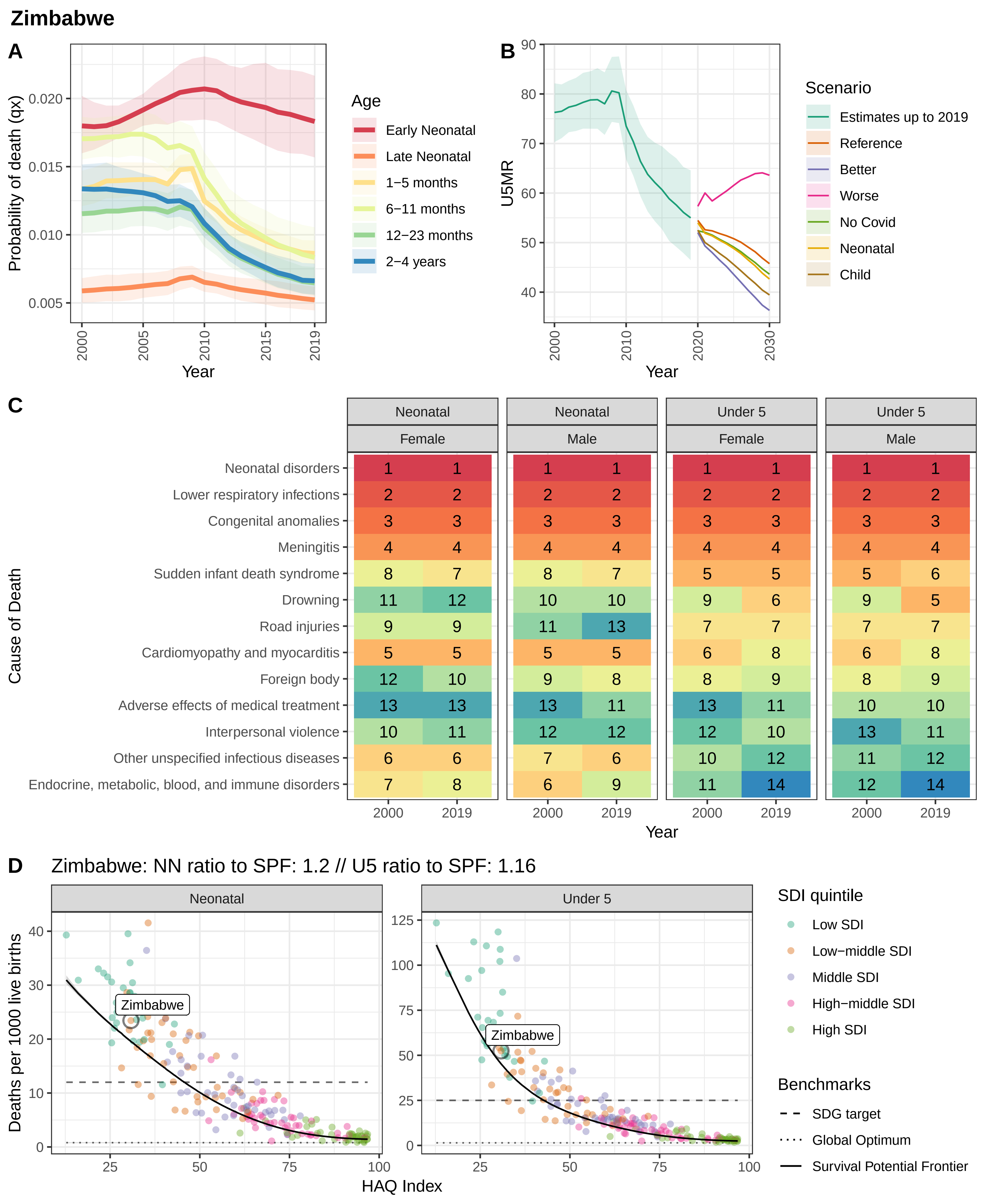
Zimbabwe
A
Probability of death (qx)
Year
Age
Early Neonatal
Late Neonatal
1−5 months
6−11 months
12−23 months
2−4 years
B
U5MR
Year
Scenario
Estimates up to 2019
Reference
Better
Worse
No Covid
Neonatal
Child
C
Neonatal Female
Neonatal Male
Under 5 Female
Under 5 Male
Cause of Death
Neonatal disorders
Lower respiratory infections
Congenital anomalies
Meningitis
Sudden infant death syndrome
Drowning
Road injuries
Cardiomyopathy and myocarditis
Foreign body
Adverse effects of medical treatment
Interpersonal violence
Other unspecified infectious diseases
Endocrine, metabolic, blood, and immune disorders
Year
D
Zimbabwe: NN ratio to SPF: 1.2 // U5 ratio to SPF: 1.16
Neonatal
Under 5
Zimbabwe
Deaths per 1000 live births
HAQ Index
SDI quintile
Low SDI
Low−middle SDI
Middle SDI
High−middle SDI
High SDI
Benchmarks
SDG target
Global Optimum
Survival Potential Frontier

Viet Nam

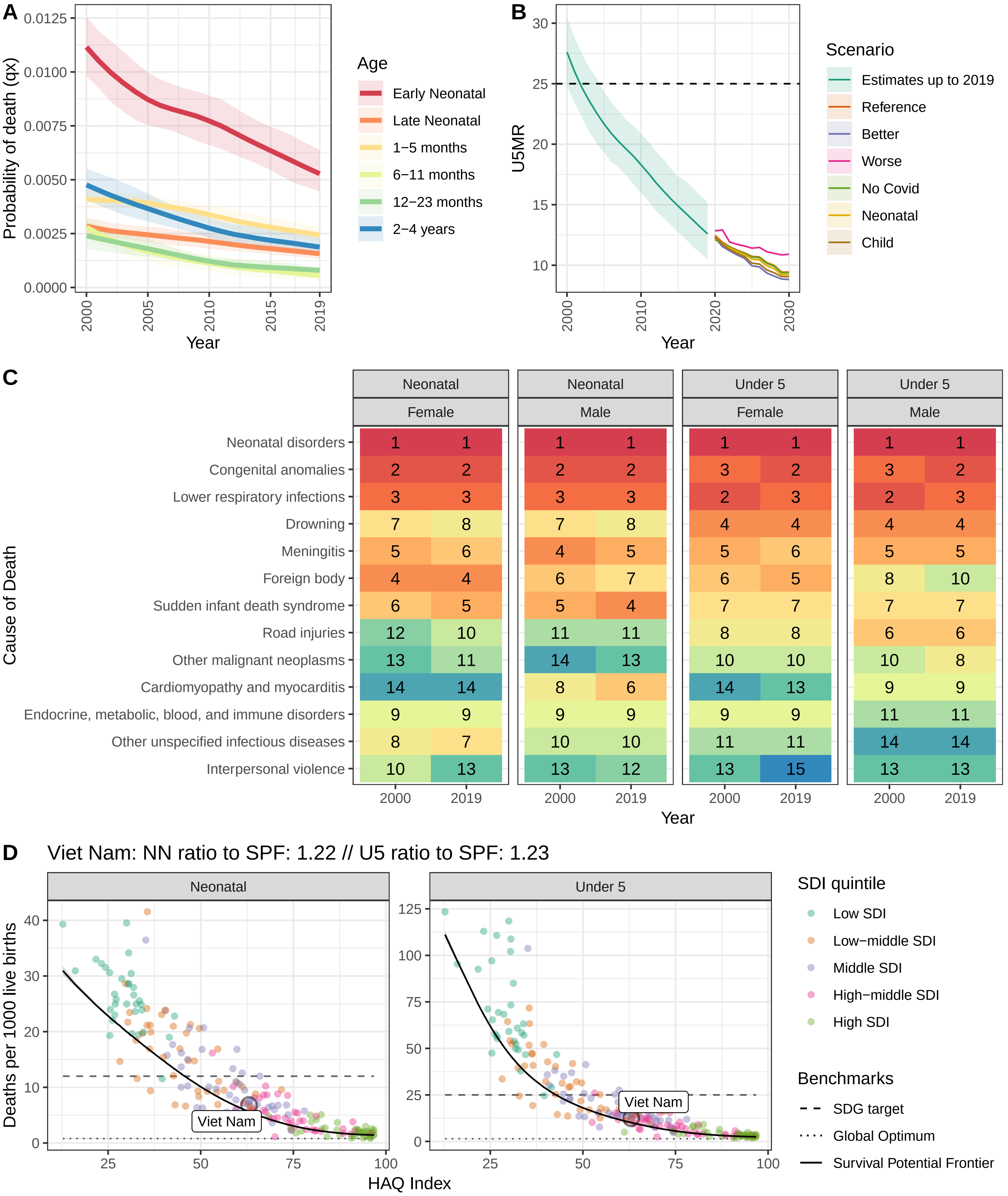
A
Probability of death (qx)
0.0125
0.0100
0.0075
0.0050
0.0025
0.0000
2000
2005
2010
2015
2019
Year
Age
Early Neonatal
Late Neonatal
1−5 months
6−11 months
12−23 months
2−4 years
B
U5MR
30
25
20
15
10
2000
2010
2020
2030
Year
Scenario
Estimates up to 2019
Reference
Better
Worse
No Covid
Neonatal
Child
C
Neonatal
Female
Neonatal
Male
Under 5
Female
Under 5
Male
Cause of Death
Neonatal disorders 1 1 1 1 1 1 1 1
Congenital anomalies 2 2 2 2 3 2 3 2
Lower respiratory infections 3 3 3 3 2 3 2 3
Drowning 7 8 7 8 4 4 4 4
Meningitis 5 6 4 5 5 6 5 5
Foreign body 4 4 6 7 6 5 8 10
Sudden infant death syndrome 6 5 5 4 7 7 7 7
Road injuries 12 10 11 11 8 8 6 6
Other malignant neoplasms 13 11 14 13 10 10 10 8
Cardiomyopathy and myocarditis 14 14 8 6 14 13 9 9
Endocrine, metabolic, blood, and immune disorders 9 9 9 9 9 9 11 11
Other unspecified infectious diseases 8 7 10 10 11 11 14 14
Interpersonal violence 10 13 13 12 13 15 13 13
2000
2019
Year
D
Viet Nam: NN ratio to SPF: 1.22 // U5 ratio to SPF: 1.23
Neonatal
Under 5
Deaths per 1000 live births
40
30
20
10
0
125
100
75
50
25
0
25
50
75
100
Viet Nam
HAQ Index
SDI quintile
Low SDI
Low−middle SDI
Middle SDI
High−middle SDI
High SDI
Benchmarks
SDG target
Global Optimum
Survival Potential Frontier

Benin

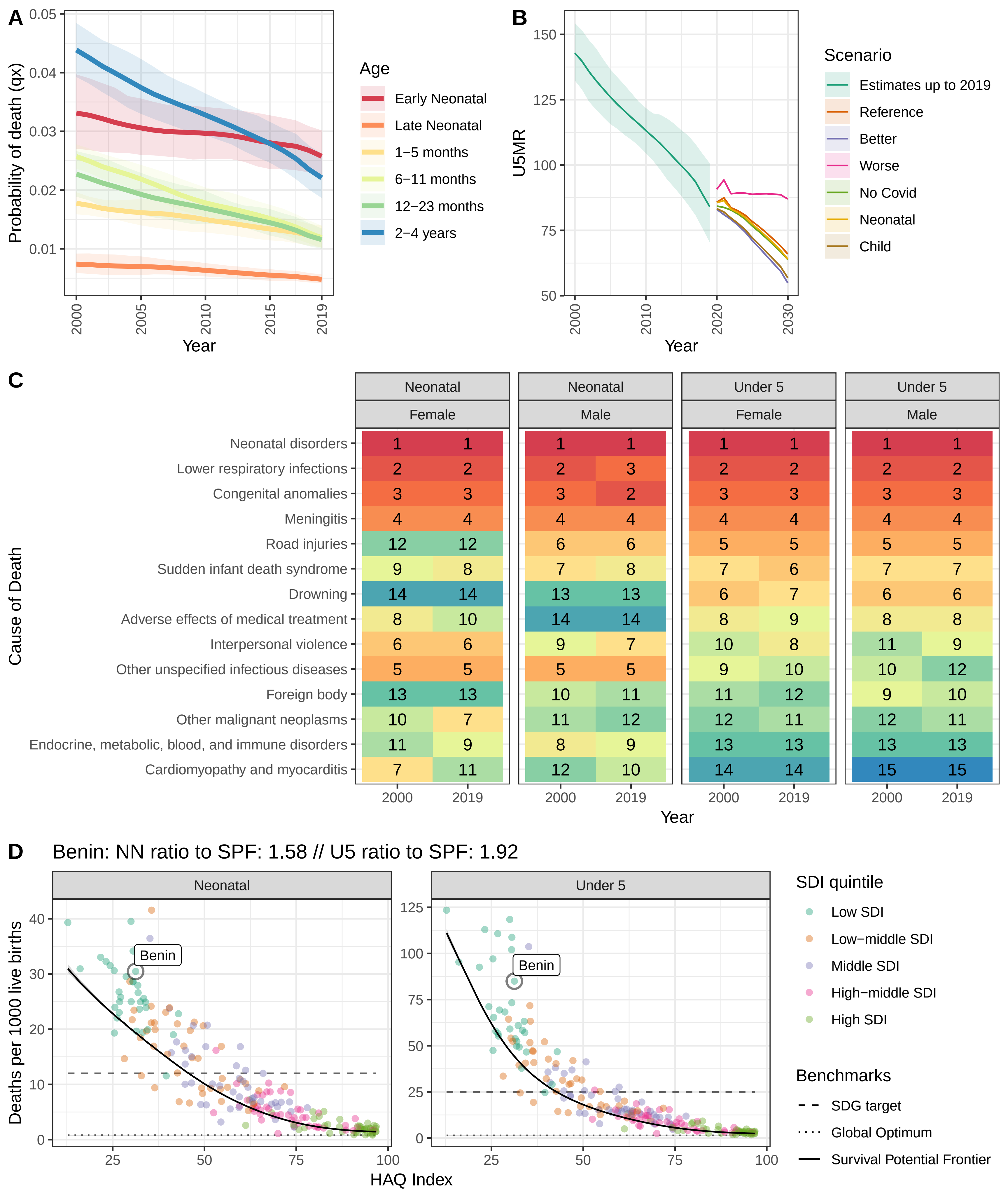
A
Probability of death (qx)
Year
Age
Early Neonatal
Late Neonatal
1−5 months
6−11 months
12−23 months
2−4 years
B
U5MR
Year
Scenario
Estimates up to 2019
Reference
Better
Worse
No Covid
Neonatal
Child
C
Neonatal
Female
Neonatal
Male
Under 5
Female
Under 5
Male
Cause of Death
Neonatal disorders
Lower respiratory infections
Congenital anomalies
Meningitis
Road injuries
Sudden infant death syndrome
Drowning
Adverse effects of medical treatment
Interpersonal violence
Other unspecified infectious diseases
Foreign body
Other malignant neoplasms
Endocrine, metabolic, blood, and immune disorders
Cardiomyopathy and myocarditis
Year
D
Benin: NN ratio to SPF: 1.58 // U5 ratio to SPF: 1.92
Neonatal
Under 5
Benin
Deaths per 1000 live births
HAQ Index
SDI quintile
Low SDI
Low−middle SDI
Middle SDI
High−middle SDI
High SDI
Benchmarks
SDG target
Global Optimum
Survival Potential Frontier

Burkina Faso

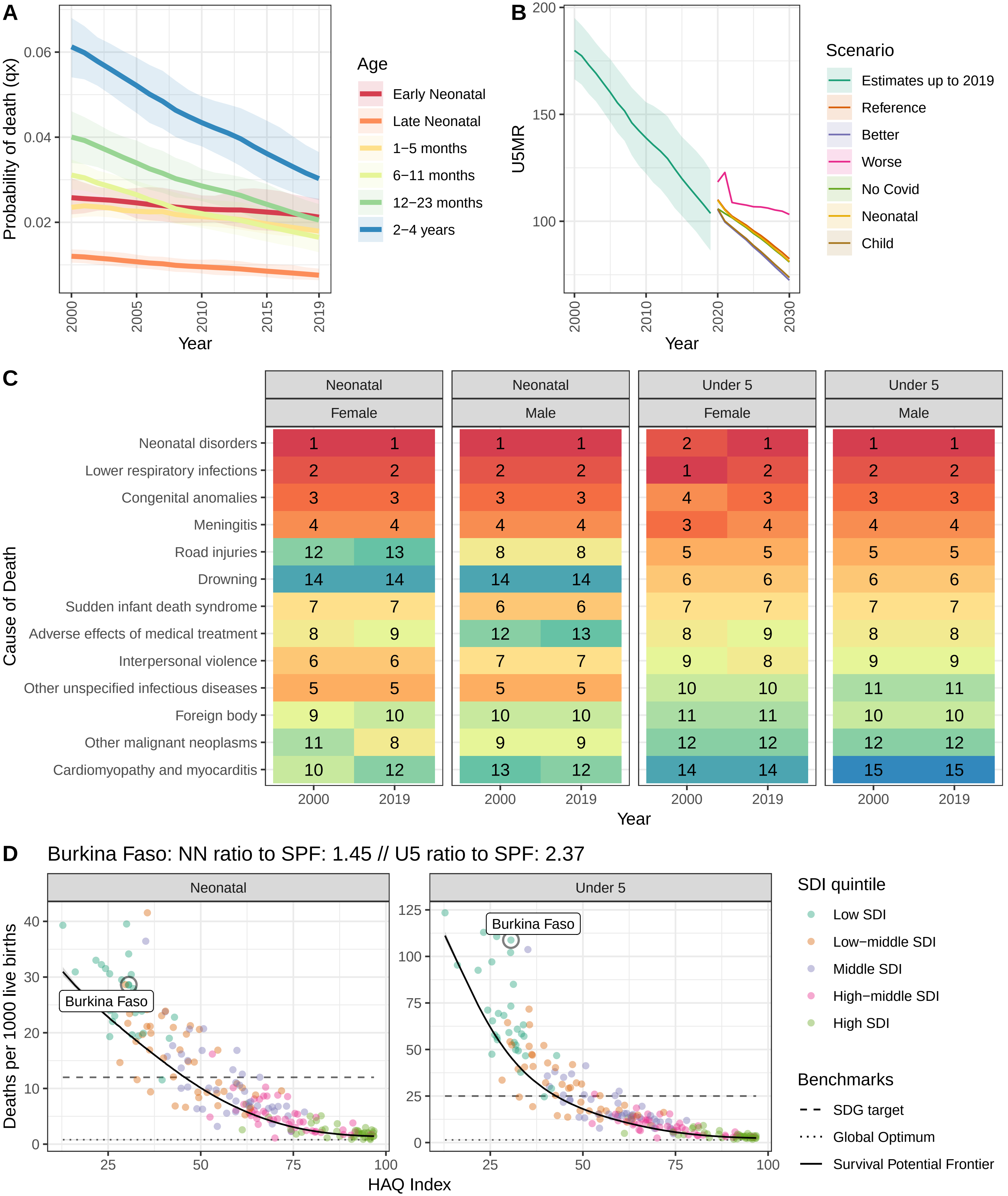

Cameroon

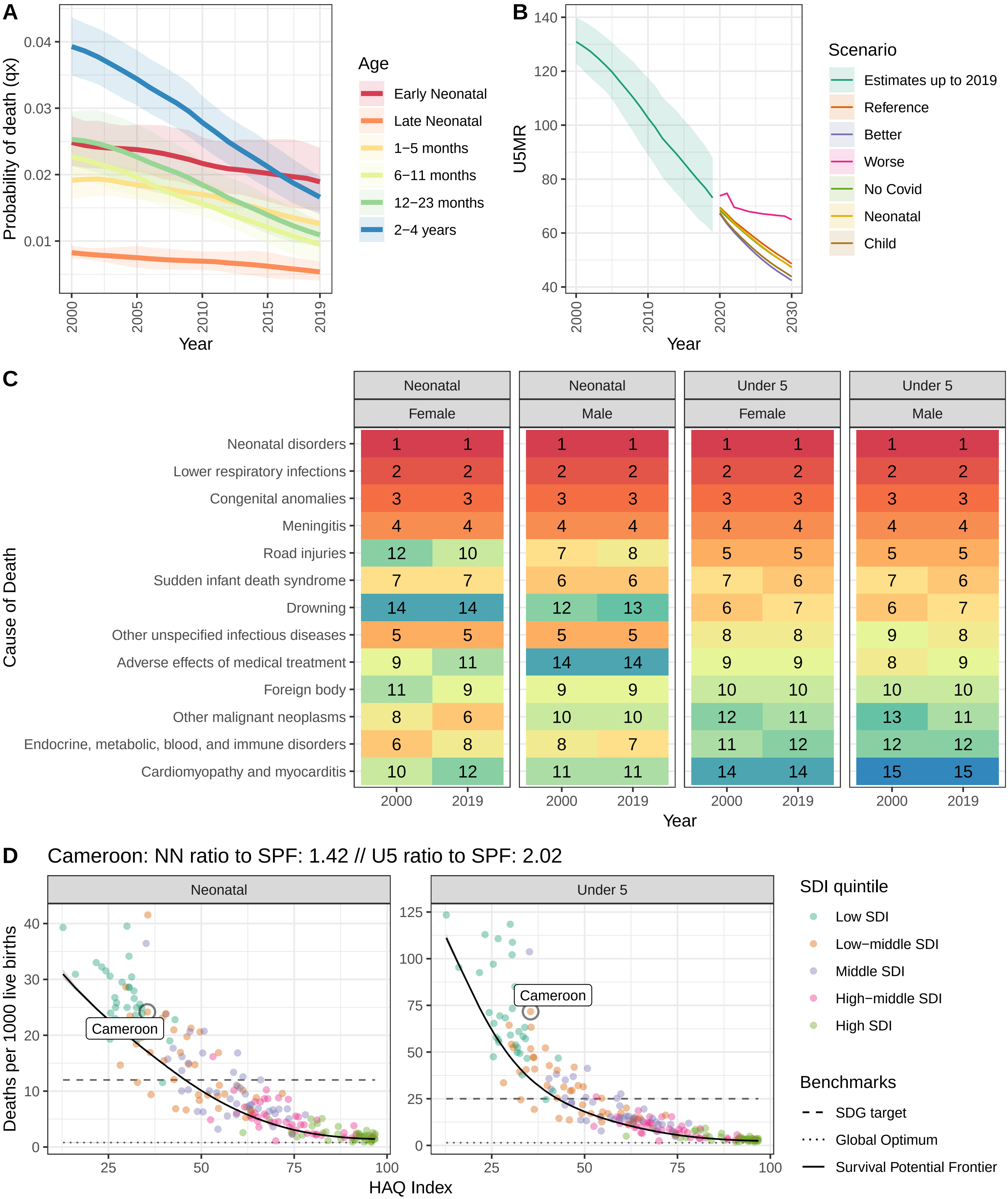

A
Probability of death (qx)
Year
Age
Early Neonatal
Late Neonatal
1−5 months
6−11 months
12−23 months
2−4 years
B
U5MR
Year
Scenario
Estimates up to 2019
Reference
Better
Worse
No Covid
Neonatal
Child
C
Neonatal Female
Neonatal Male
Under 5 Female
Under 5 Male
Cause of Death
Neonatal disorders 1 1 1 1 1 1 1 1
Lower respiratory infections 2 2 2 2 2 2 2 2
Congenital anomalies 3 3 3 3 3 3 3 3
Meningitis 4 4 4 4 4 4 4 4
Road injuries 12 10 7 8 5 5 5 5
Sudden infant death syndrome 7 7 6 6 7 6 7 6
Drowning 14 14 12 13 6 7 6 7
Other unspecified infectious diseases 5 5 5 5 8 8 9 8
Adverse effects of medical treatment 9 11 14 14 9 9 8 9
Foreign body 11 9 9 9 10 10 10 10
Other malignant neoplasms 8 6 10 10 12 11 13 11
Endocrine, metabolic, blood, and immune disorders 6 8 8 7 11 12 12 12
Cardiomyopathy and myocarditis 10 12 11 11 14 14 15 15
2000 2019
Year
D
Cameroon: NN ratio to SPF: 1.42 // U5 ratio to SPF: 2.02
Neonatal
Under 5
Cameroon
Deaths per 1000 live births
HAQ Index
SDI quintile
Low SDI
Low−middle SDI
Middle SDI
High−middle SDI
High SDI
Benchmarks
SDG target
Global Optimum
Survival Potential Frontier

Cabo Verde

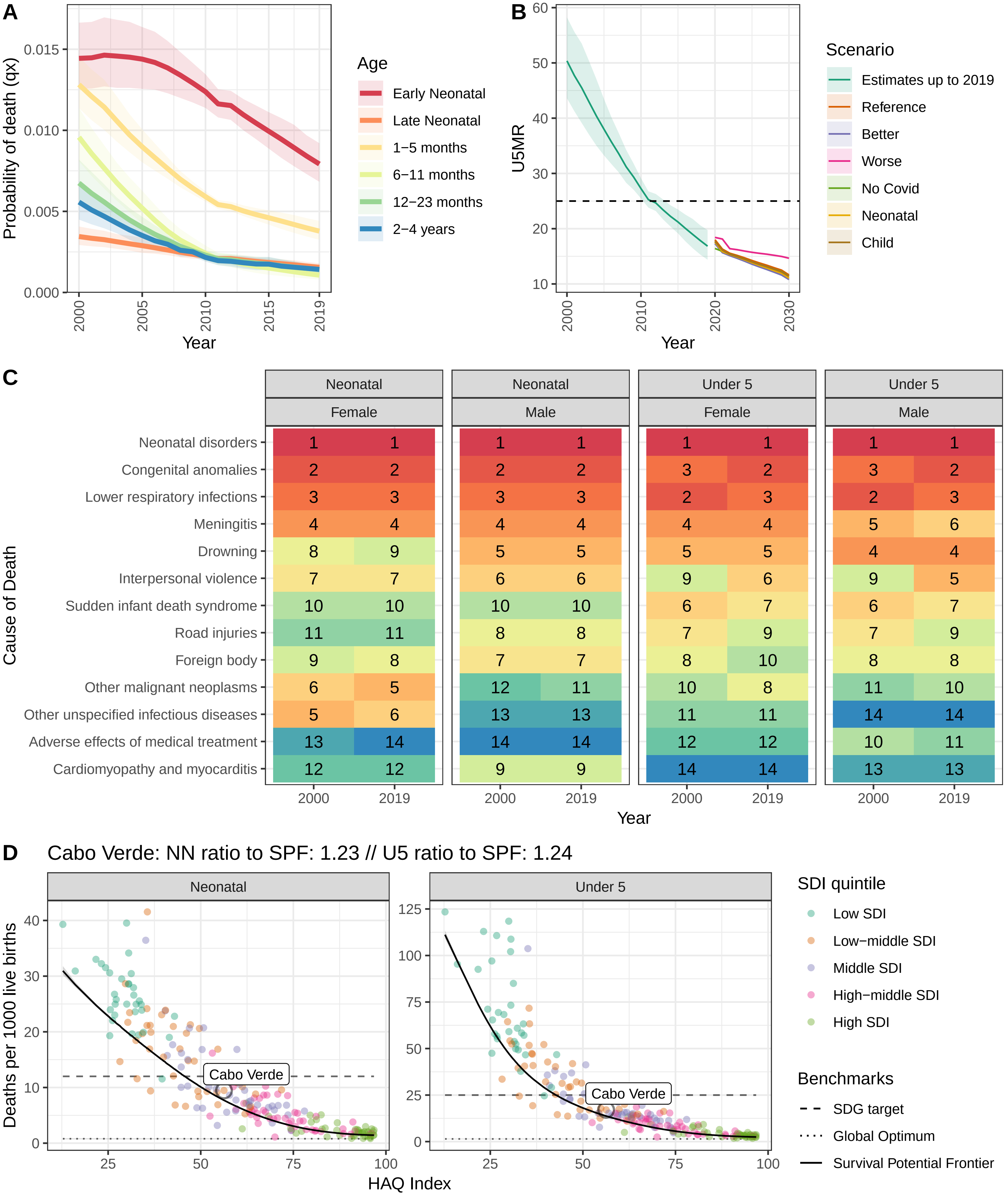

A
Probability of death (qx)
Year
Age
Early Neonatal
Late Neonatal
1−5 months
6−11 months
12−23 months
2−4 years
B
U5MR
Year
Scenario
Estimates up to 2019
Reference
Better
Worse
No Covid
Neonatal
Child
C
Neonatal
Female
Neonatal
Male
Under 5
Female
Under 5
Male
Cause of Death
Neonatal disorders
Congenital anomalies
Lower respiratory infections
Meningitis
Drowning
Interpersonal violence
Sudden infant death syndrome
Road injuries
Foreign body
Other malignant neoplasms
Other unspecified infectious diseases
Adverse effects of medical treatment
Cardiomyopathy and myocarditis
Year
D
Cabo Verde: NN ratio to SPF: 1.23 // U5 ratio to SPF: 1.24
Neonatal
Under 5
Deaths per 1000 live births
Cabo Verde
HAQ Index
SDI quintile
Low SDI
Low−middle SDI
Middle SDI
High−middle SDI
High SDI
Benchmarks
SDG target
Global Optimum
Survival Potential Frontier

Chad

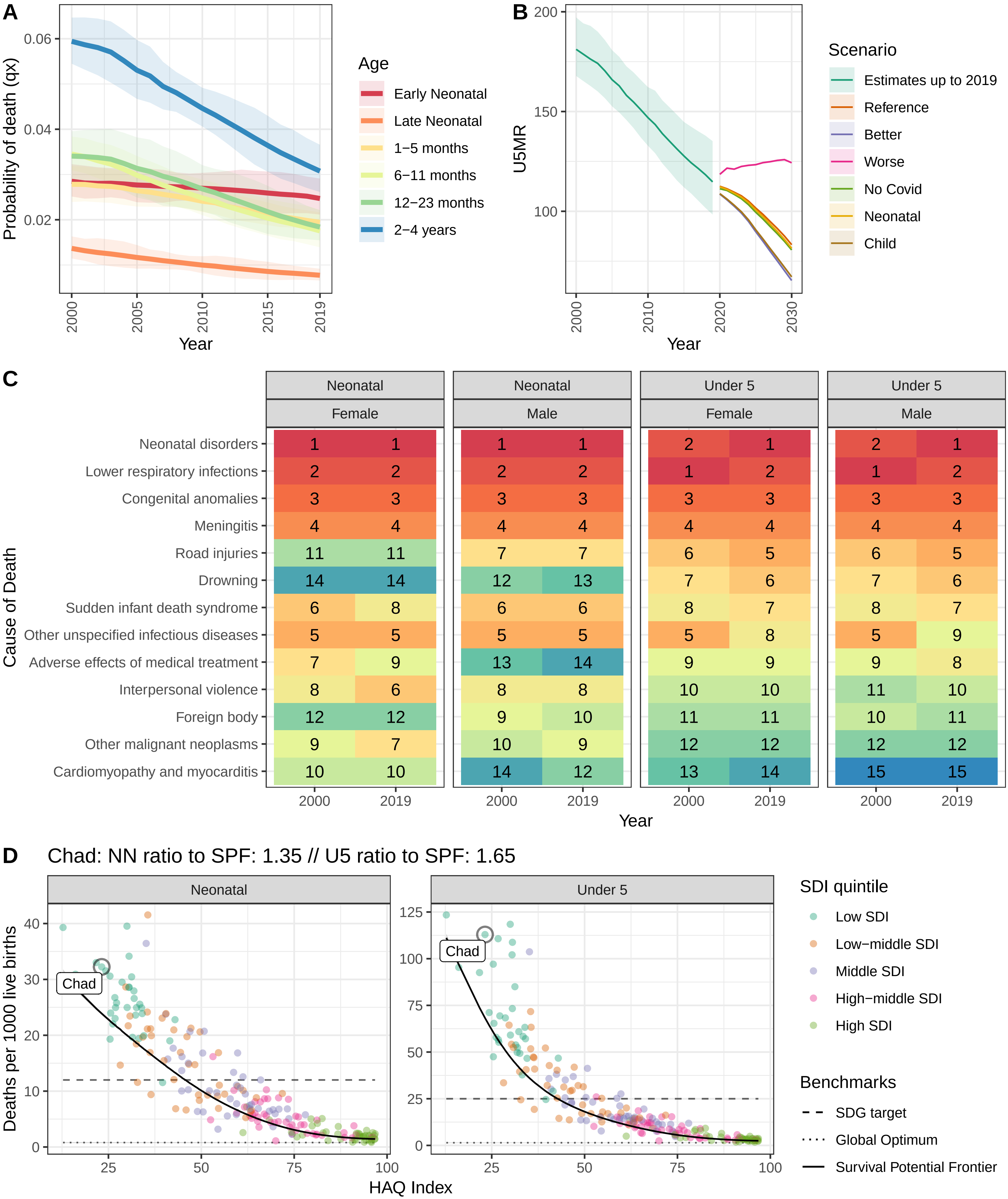
A
Probability of death (qx)
0.06
0.04
0.02
2000
2005
2010
2015
2019
Year
Age
Early Neonatal
Late Neonatal
1−5 months
6−11 months
12−23 months
2−4 years
B
U5MR
200
150
100
2000
2010
2020
2030
Year
Scenario
Estimates up to 2019
Reference
Better
Worse
No Covid
Neonatal
Child
C
Neonatal Female
Neonatal Male
Under 5 Female
Under 5 Male
Cause of Death
Neonatal disorders 1 1 1 1 2 1 2 1
Lower respiratory infections 2 2 2 2 1 2 1 2
Congenital anomalies 3 3 3 3 3 3 3 3
Meningitis 4 4 4 4 4 4 4 4
Road injuries 11 11 7 7 6 5 6 5
Drowning 14 14 12 13 7 6 7 6
Sudden infant death syndrome 6 8 6 6 8 7 8 7
Other unspecified infectious diseases 5 5 5 5 5 8 5 9
Adverse effects of medical treatment 7 9 13 14 9 9 9 8
Interpersonal violence 8 6 8 8 10 10 11 10
Foreign body 12 12 9 10 11 11 10 11
Other malignant neoplasms 9 7 10 9 12 12 12 12
Cardiomyopathy and myocarditis 10 10 14 12 13 14 15 15
2000
2019
Year
D
Chad: NN ratio to SPF: 1.35 // U5 ratio to SPF: 1.65
Neonatal
Under 5
Deaths per 1000 live births
40
30
20
10
0
125
100
75
50
25
0
Chad
25
50
75
100
HAQ Index
SDI quintile
Low SDI
Low−middle SDI
Middle SDI
High−middle SDI
High SDI
Benchmarks
SDG target
Global Optimum
Survival Potential Frontier

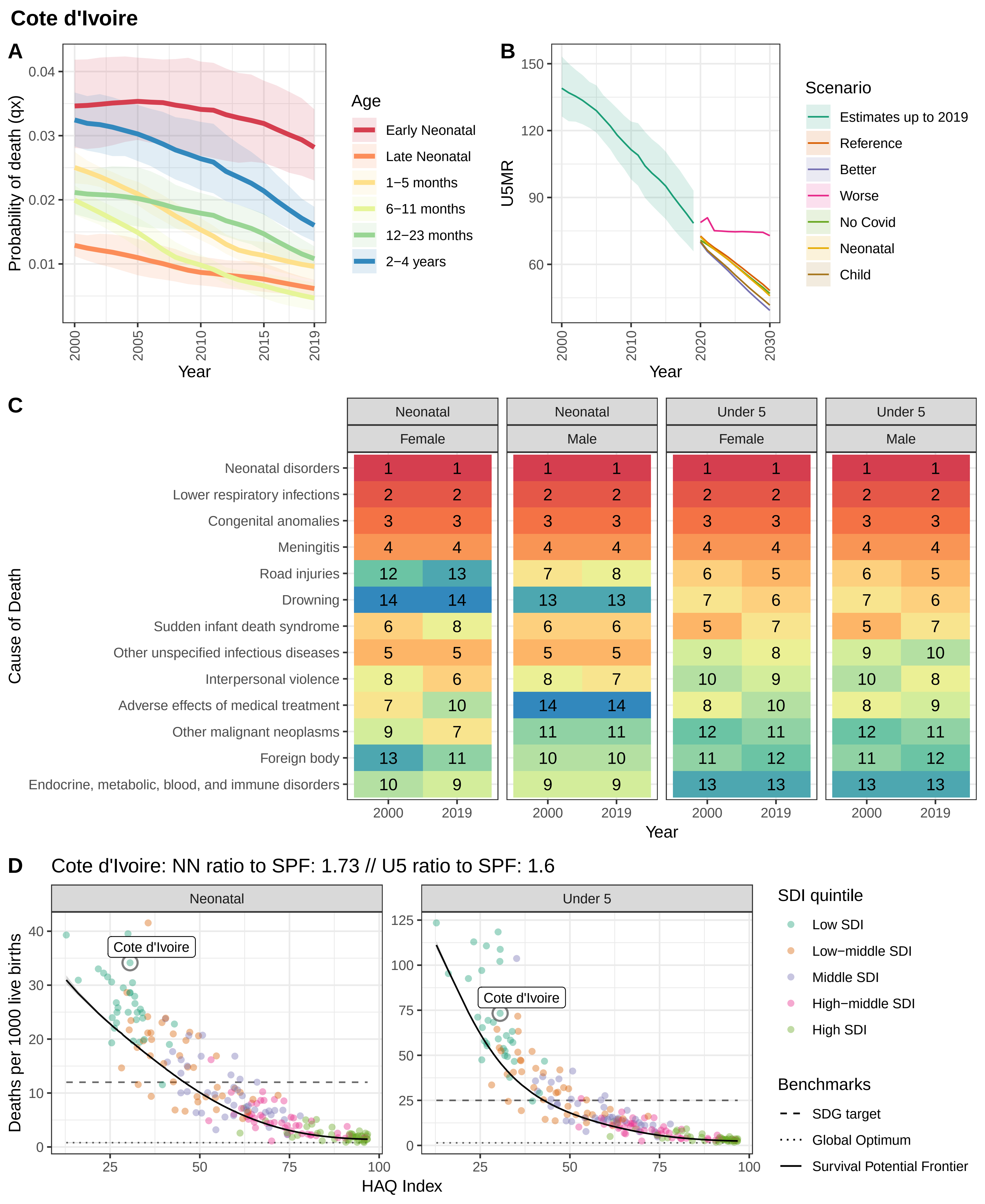
Cote d'Ivoire
A
Probability of death (qx)
0.04
0.03
0.02
0.01
2000
2005
2010
2015
2019
Year
Age
Early Neonatal
Late Neonatal
1−5 months
6−11 months
12−23 months
2−4 years
B
U5MR
150
120
90
60
2000
2010
2020
2030
Year
Scenario
Estimates up to 2019
Reference
Better
Worse
No Covid
Neonatal
Child
C
Cause of Death
Neonatal Female
Neonatal Male
Under 5 Female
Under 5 Male
Neonatal disorders 1 1 1 1 1 1 1 1
Lower respiratory infections 2 2 2 2 2 2 2 2
Congenital anomalies 3 3 3 3 3 3 3 3
Meningitis 4 4 4 4 4 4 4 4
Road injuries 12 13 7 8 6 5 6 5
Drowning 14 14 13 13 7 6 7 6
Sudden infant death syndrome 6 8 6 6 5 7 5 7
Other unspecified infectious diseases 5 5 5 5 9 8 9 10
Interpersonal violence 8 6 8 7 10 9 10 8
Adverse effects of medical treatment 7 10 14 14 8 10 8 9
Other malignant neoplasms 9 7 11 11 12 11 12 11
Foreign body 13 11 10 10 11 12 11 12
Endocrine, metabolic, blood, and immune disorders 10 9 9 9 13 13 13 13
2000 2019 2000 2019 2000 2019 2000 2019
Year
D
Cote d'Ivoire: NN ratio to SPF: 1.73 // U5 ratio to SPF: 1.6
Neonatal
Under 5
Deaths per 1000 live births
40
30
20
10
0
125
100
75
50
25
0
Cote d'Ivoire
Cote d'Ivoire
25
50
75
100
HAQ Index
SDI quintile
Low SDI
Low−middle SDI
Middle SDI
High−middle SDI
High SDI
Benchmarks
SDG target
Global Optimum
Survival Potential Frontier

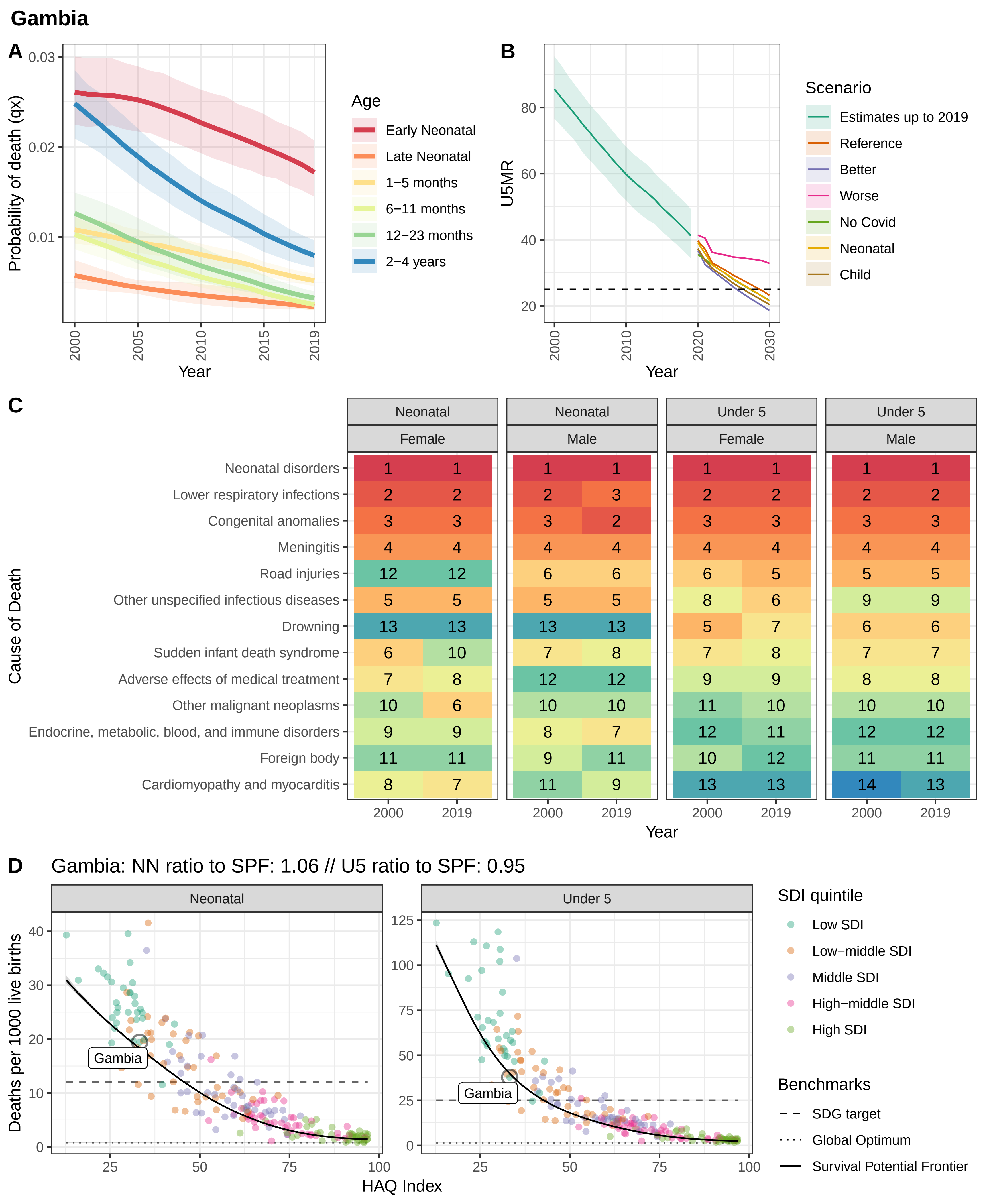
Gambia
A
Probability of death (qx)
0.03
0.02
0.01
2000
2005
2010
2015
2019
Year
Age
Early Neonatal
Late Neonatal
1−5 months
6−11 months
12−23 months
2−4 years
B
U5MR
80
60
40
20
2000
2010
2020
2030
Year
Scenario
Estimates up to 2019
Reference
Better
Worse
No Covid
Neonatal
Child
C
Cause of Death
Neonatal Female
Neonatal Male
Under 5 Female
Under 5 Male
Neonatal disorders 1 1 1 1 1 1 1 1
Lower respiratory infections 2 2 2 3 2 2 2 2
Congenital anomalies 3 3 3 2 3 3 3 3
Meningitis 4 4 4 4 4 4 4 4
Road injuries 12 12 6 6 6 5 5 5
Other unspecified infectious diseases 5 5 5 5 8 6 9 9
Drowning 13 13 13 13 5 7 6 6
Sudden infant death syndrome 6 10 7 8 7 8 7 7
Adverse effects of medical treatment 7 8 12 12 9 9 8 8
Other malignant neoplasms 10 6 10 10 11 10 10 10
Endocrine, metabolic, blood, and immune disorders 9 9 8 7 12 11 12 12
Foreign body 11 11 9 11 10 12 11 11
Cardiomyopathy and myocarditis 8 7 11 9 13 13 14 13
2000
2019
Year
D
Gambia: NN ratio to SPF: 1.06 // U5 ratio to SPF: 0.95
Neonatal
Under 5
Deaths per 1000 live births
40
30
20
10
0
125
100
75
50
25
0
Gambia
Gambia
25
50
75
100
HAQ Index
SDI quintile
Low SDI
Low−middle SDI
Middle SDI
High−middle SDI
High SDI
Benchmarks
SDG target
Global Optimum
Survival Potential Frontier

Ghana

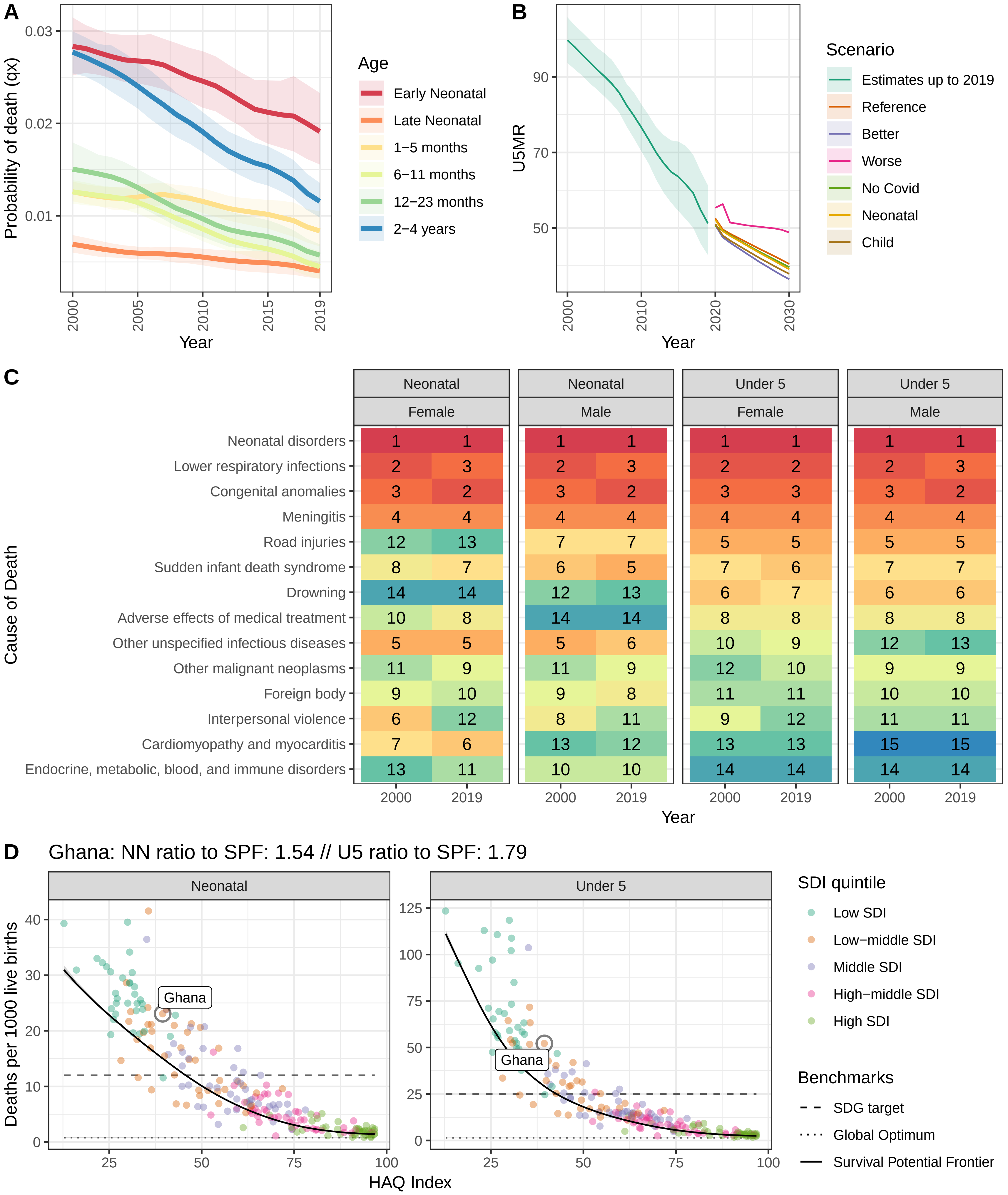
A
Probability of death (qx)
0.03
0.02
0.01
2000
2005
2010
2015
2019
Year
Age
Early Neonatal
Late Neonatal
1−5 months
6−11 months
12−23 months
2−4 years
B
U5MR
90
70
50
2000
2010
2020
2030
Year
Scenario
Estimates up to 2019
Reference
Better
Worse
No Covid
Neonatal
Child
C
Neonatal
Female
Neonatal
Male
Under 5
Female
Under 5
Male
Cause of Death
Neonatal disorders 1 1 1 1 1 1 1 1
Lower respiratory infections 2 3 2 3 2 2 2 3
Congenital anomalies 3 2 3 2 3 3 3 2
Meningitis 4 4 4 4 4 4 4 4
Road injuries 12 13 7 7 5 5 5 5
Sudden infant death syndrome 8 7 6 5 7 6 7 7
Drowning 14 14 12 13 6 7 6 6
Adverse effects of medical treatment 10 8 14 14 8 8 8 8
Other unspecified infectious diseases 5 5 5 6 10 9 12 13
Other malignant neoplasms 11 9 11 9 12 10 9 9
Foreign body 9 10 9 8 11 11 10 10
Interpersonal violence 6 12 8 11 9 12 11 11
Cardiomyopathy and myocarditis 7 6 13 12 13 13 15 15
Endocrine, metabolic, blood, and immune disorders 13 11 10 10 14 14 14 14
2000
2019
Year
D
Ghana: NN ratio to SPF: 1.54 // U5 ratio to SPF: 1.79
Neonatal
Under 5
Deaths per 1000 live births
Ghana
Ghana
40
30
20
10
0
125
100
75
50
25
0
25
50
75
100
HAQ Index
SDI quintile
Low SDI
Low−middle SDI
Middle SDI
High−middle SDI
High SDI
Benchmarks
SDG target
Global Optimum
Survival Potential Frontier

Guinea

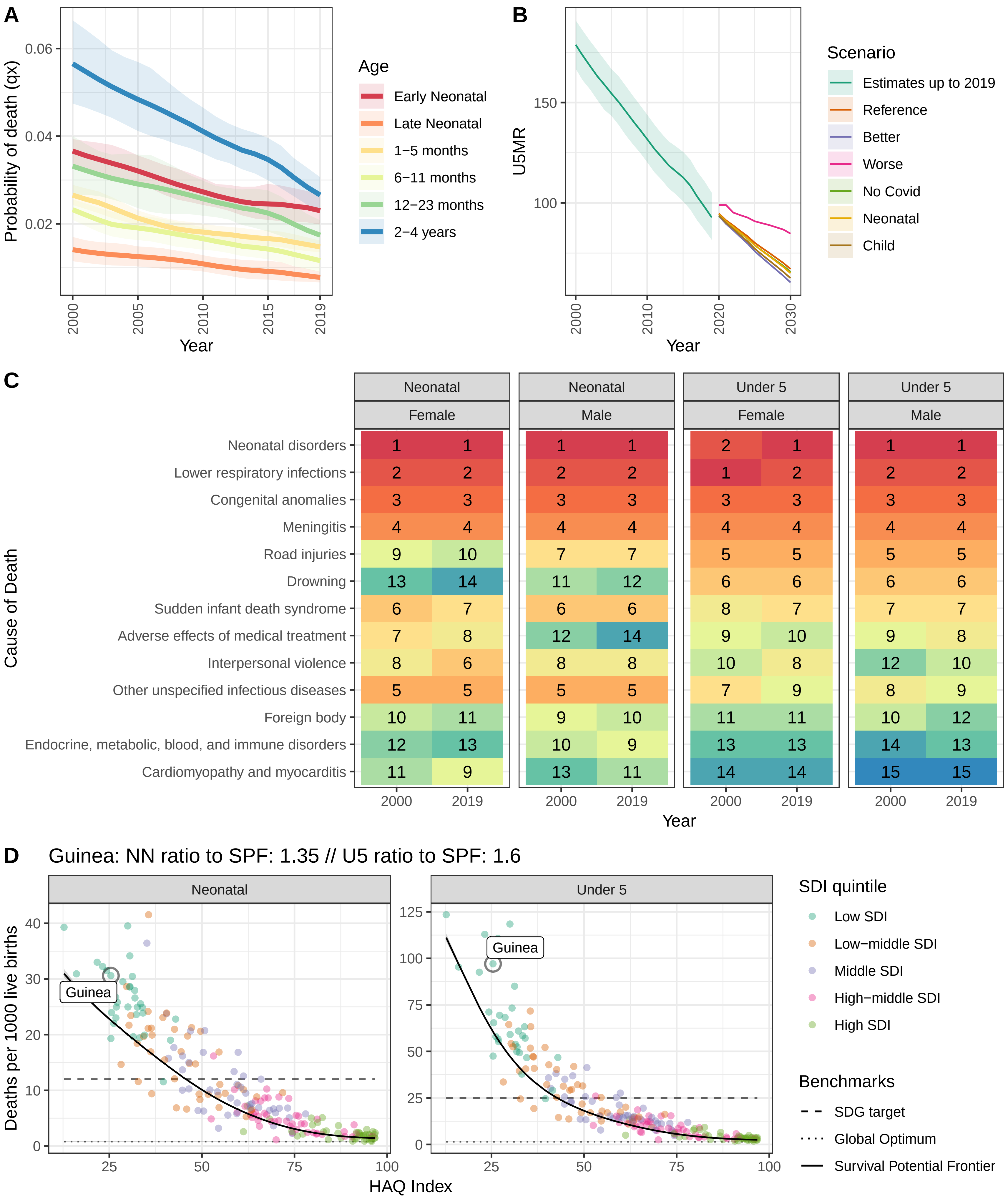
A
Probability of death (qx)
0.06
0.04
0.02
2000
2005
2010
2015
2019
Year
Age
Early Neonatal
Late Neonatal
1−5 months
6−11 months
12−23 months
2−4 years
B
U5MR
150
100
2000
2010
2020
2030
Year
Scenario
Estimates up to 2019
Reference
Better
Worse
No Covid
Neonatal
Child
C
Neonatal
Female
Neonatal
Male
Under 5
Female
Under 5
Male
Cause of Death
Neonatal disorders 1 1 1 1 2 1 1 1
Lower respiratory infections 2 2 2 2 1 2 2 2
Congenital anomalies 3 3 3 3 3 3 3 3
Meningitis 4 4 4 4 4 4 4 4
Road injuries 9 10 7 7 5 5 5 5
Drowning 13 14 11 12 6 6 6 6
Sudden infant death syndrome 6 7 6 6 8 7 7 7
Adverse effects of medical treatment 7 8 12 14 9 10 9 8
Interpersonal violence 8 6 8 8 10 8 12 10
Other unspecified infectious diseases 5 5 5 5 7 9 8 9
Foreign body 10 11 9 10 11 11 10 12
Endocrine, metabolic, blood, and immune disorders 12 13 10 9 13 13 14 13
Cardiomyopathy and myocarditis 11 9 13 11 14 14 15 15
2000
2019
Year
D
Guinea: NN ratio to SPF: 1.35 // U5 ratio to SPF: 1.6
Neonatal
Under 5
Deaths per 1000 live births
40
30
20
10
0
125
100
75
50
25
0
Guinea
25
50
75
100
HAQ Index
SDI quintile
Low SDI
Low−middle SDI
Middle SDI
High−middle SDI
High SDI
Benchmarks
SDG target
Global Optimum
Survival Potential Frontier

Guinea−Bissau

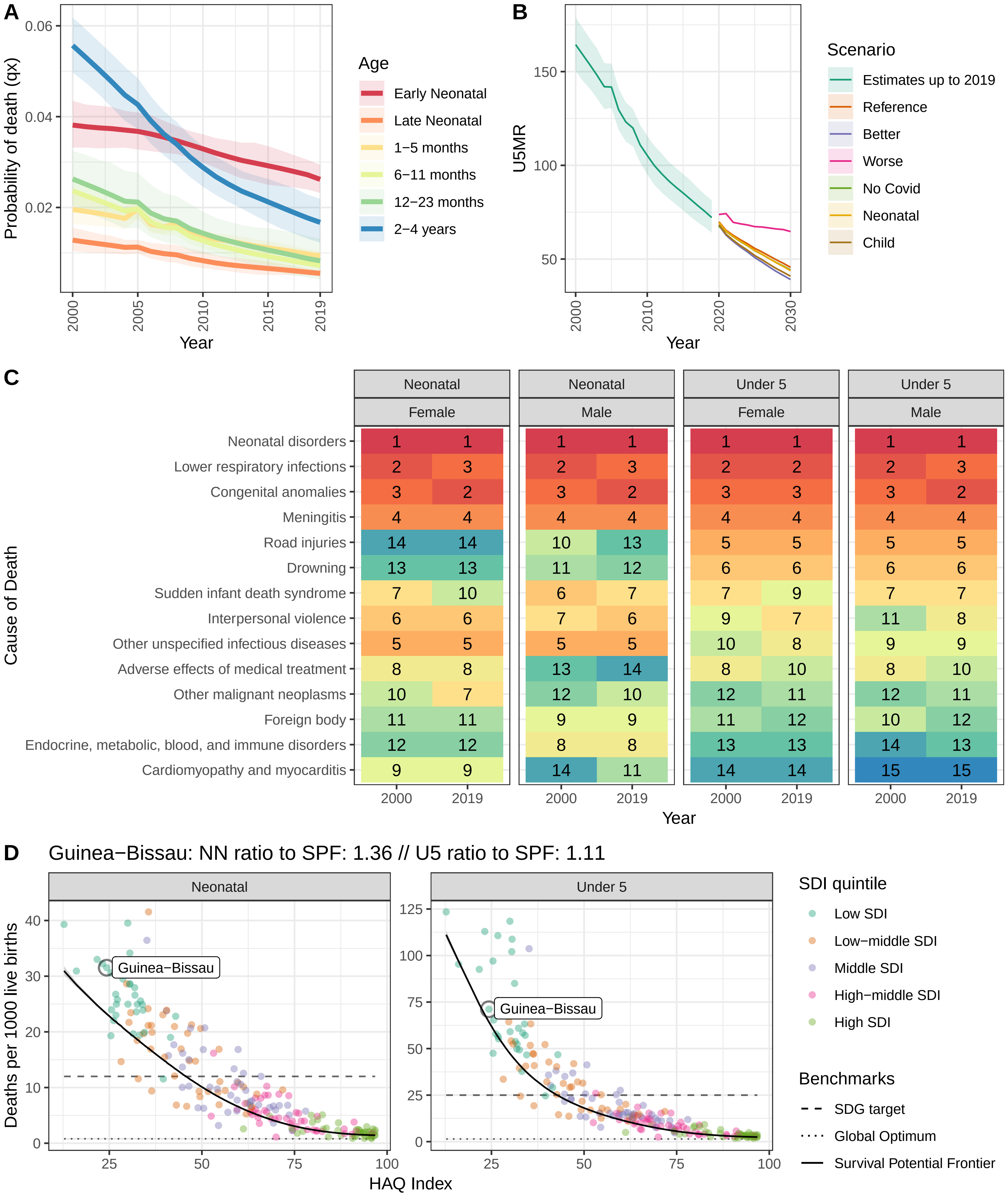

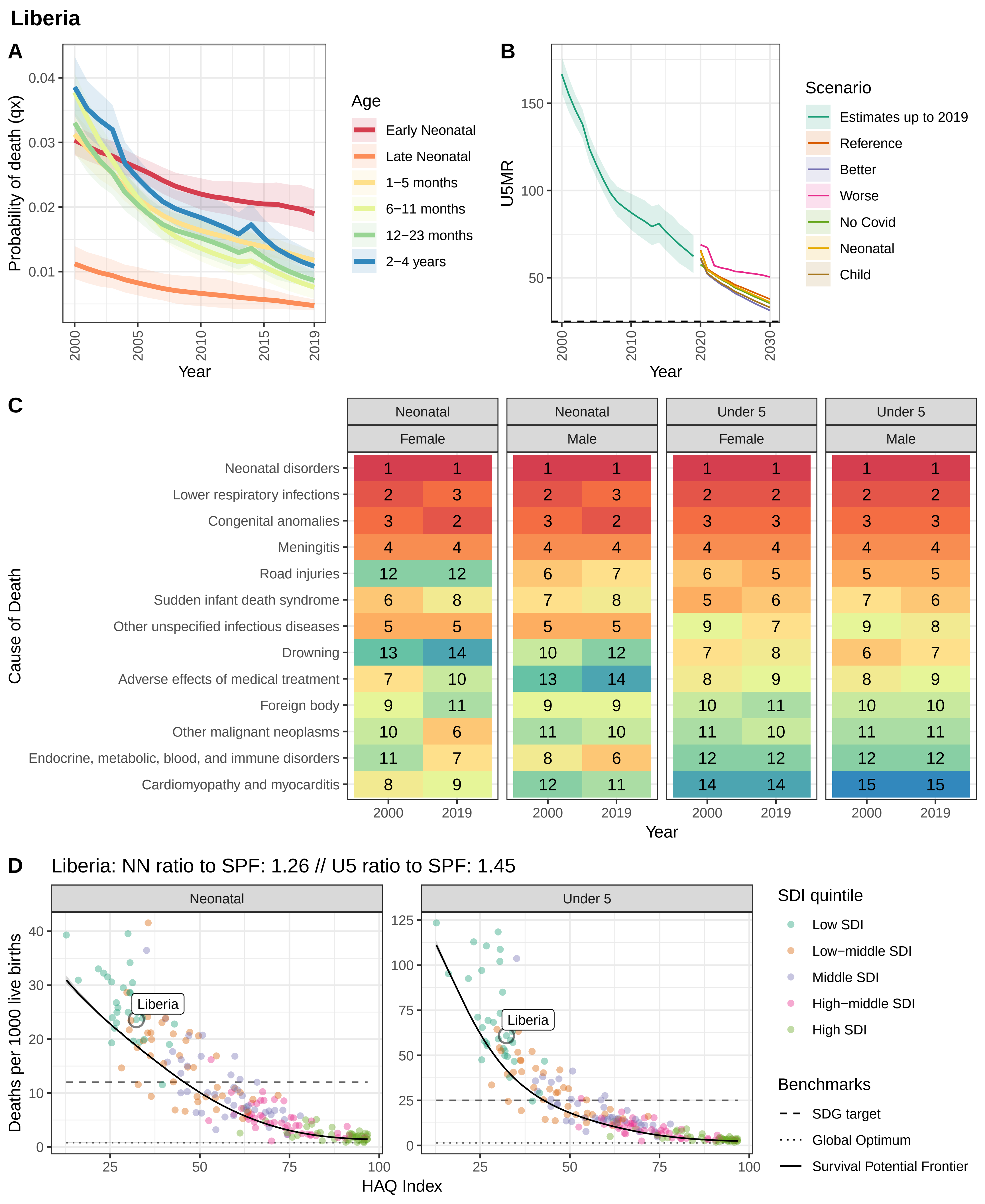
Liberia
A
Probability of death (qx)
Year
Age
Early Neonatal
Late Neonatal
1−5 months
6−11 months
12−23 months
2−4 years
B
U5MR
Year
Scenario
Estimates up to 2019
Reference
Better
Worse
No Covid
Neonatal
Child
C
Cause of Death
Neonatal Female
Neonatal Male
Under 5 Female
Under 5 Male
Neonatal disorders 1 1 1 1 1 1 1 1
Lower respiratory infections 2 3 2 3 2 2 2 2
Congenital anomalies 3 2 3 2 3 3 3 3
Meningitis 4 4 4 4 4 4 4 4
Road injuries 12 12 6 7 6 5 5 5
Sudden infant death syndrome 6 8 7 8 5 6 7 6
Other unspecified infectious diseases 5 5 5 5 9 7 9 8
Drowning 13 14 10 12 7 8 6 7
Adverse effects of medical treatment 7 10 13 14 8 9 8 9
Foreign body 9 11 9 9 10 11 10 10
Other malignant neoplasms 10 6 11 10 11 10 11 11
Endocrine, metabolic, blood, and immune disorders 11 7 8 6 12 12 12 12
Cardiomyopathy and myocarditis 8 9 12 11 14 14 15 15
2000 2019
Year
D
Liberia: NN ratio to SPF: 1.26 // U5 ratio to SPF: 1.45
Neonatal
Under 5
Deaths per 1000 live births
HAQ Index
Liberia
SDI quintile
Low SDI
Low−middle SDI
Middle SDI
High−middle SDI
High SDI
Benchmarks
SDG target
Global Optimum
Survival Potential Frontier

Mali

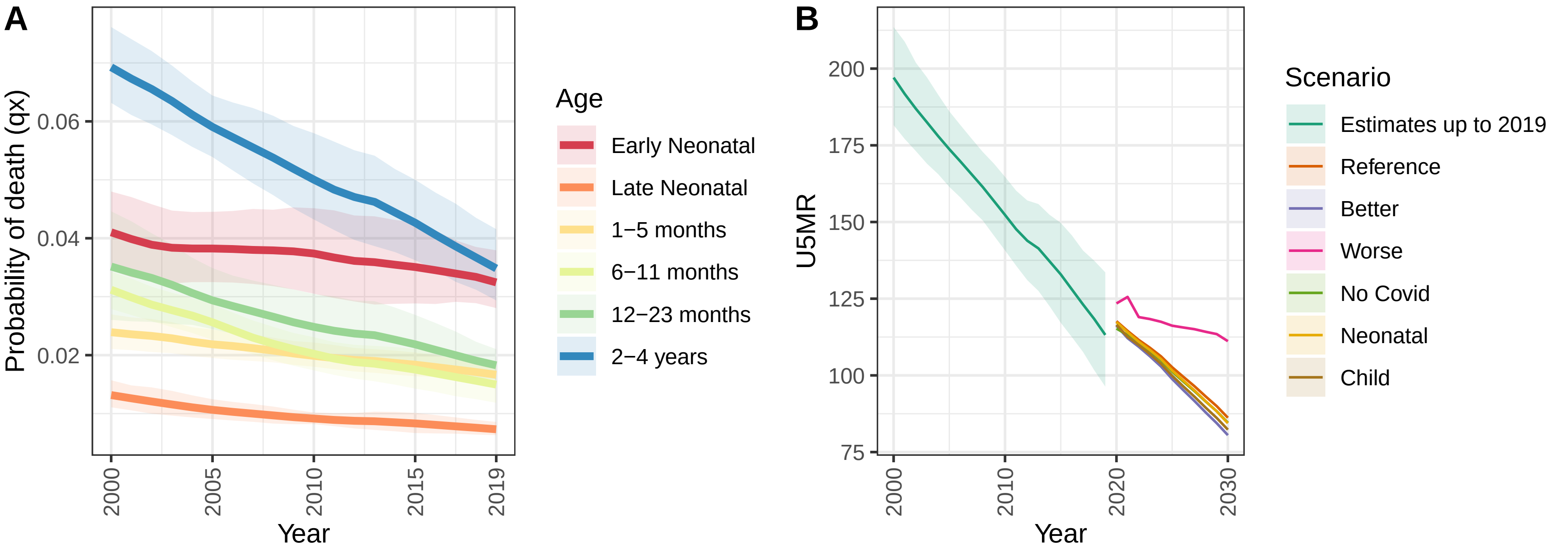

C

Cause of Death	Neonatal Female 2000	Neonatal Female 2019	Neonatal Male 2000	Neonatal Male 2019	Under 5 Female 2000	Under 5 Female 2019	Under 5 Male 2000	Under 5 Male 2019
Neonatal disorders	1	1	1	1	1	1	1	1
Lower respiratory infections	2	2	2	3	2	2	2	2
Congenital anomalies	3	3	3	2	4	3	4	3
Meningitis	4	4	4	4	3	4	3	4
Road injuries	11	11	7	8	6	5	5	5
Drowning	14	14	13	14	5	6	6	6
Sudden infant death syndrome	8	7	6	7	7	7	8	7
Adverse effects of medical treatment	9	9	12	13	8	9	7	8
Interpersonal violence	6	6	8	6	10	8	11	11
Foreign body	10	10	9	11	11	11	10	9
Other unspecified infectious diseases	5	5	5	5	9	10	9	10
Endocrine, metabolic, blood, and immune disorders	13	13	10	10	14	13	14	13
Cardiomyopathy and myocarditis	7	8	11	9	12	14	15	15

Year

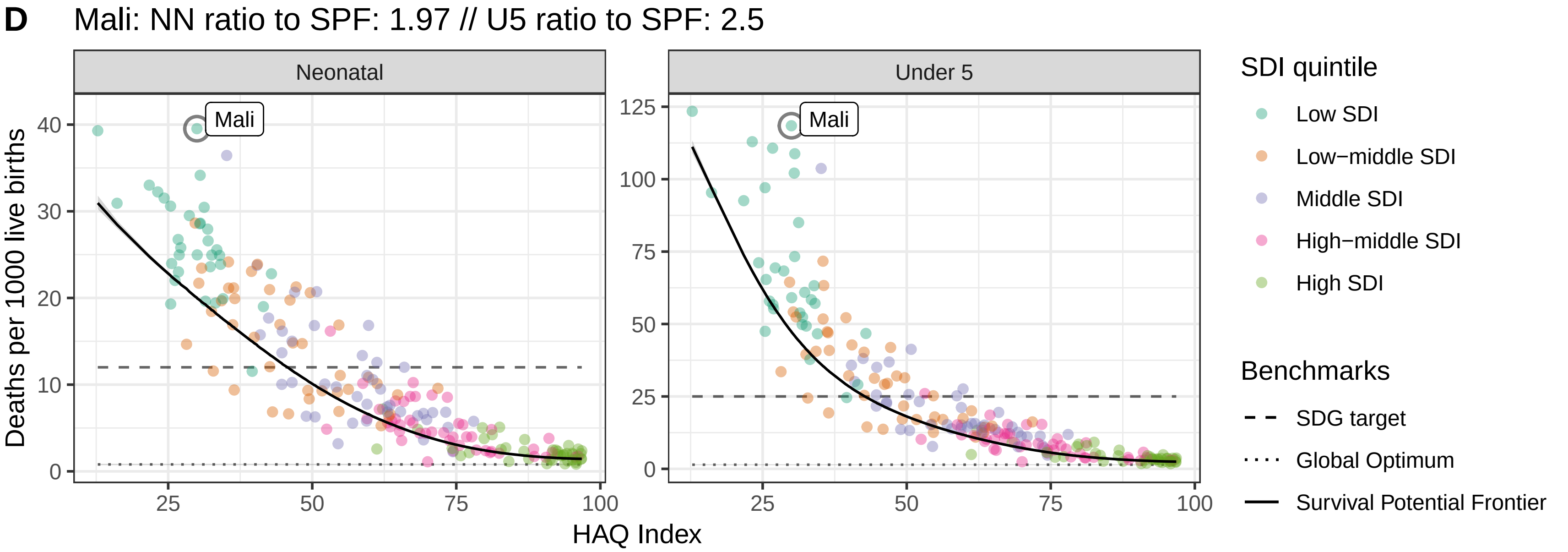

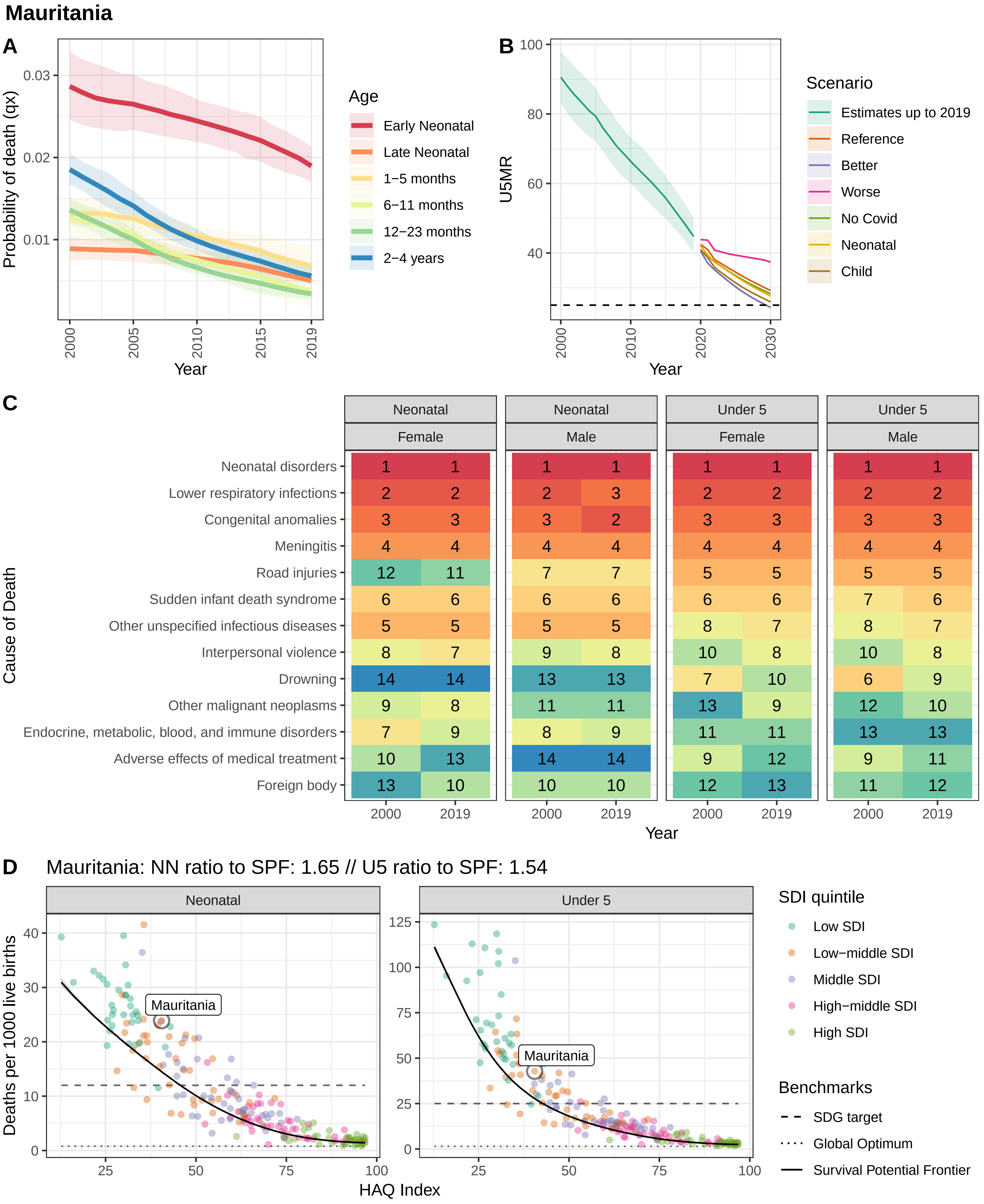
Mauritania
A
Probability of death (qx)
Year
Age
Early Neonatal
Late Neonatal
1−5 months
6−11 months
12−23 months
2−4 years
B
U5MR
Year
Scenario
Estimates up to 2019
Reference
Better
Worse
No Covid
Neonatal
Child
C
Cause of Death
Neonatal Female
Neonatal Male
Under 5 Female
Under 5 Male
Neonatal disorders
Lower respiratory infections
Congenital anomalies
Meningitis
Road injuries
Sudden infant death syndrome
Other unspecified infectious diseases
Interpersonal violence
Drowning
Other malignant neoplasms
Endocrine, metabolic, blood, and immune disorders
Adverse effects of medical treatment
Foreign body
Year
D
Mauritania: NN ratio to SPF: 1.65 // U5 ratio to SPF: 1.54
Neonatal
Under 5
Deaths per 1000 live births
HAQ Index
Mauritania
SDI quintile
Low SDI
Low−middle SDI
Middle SDI
High−middle SDI
High SDI
Benchmarks
SDG target
Global Optimum
Survival Potential Frontier

Niger

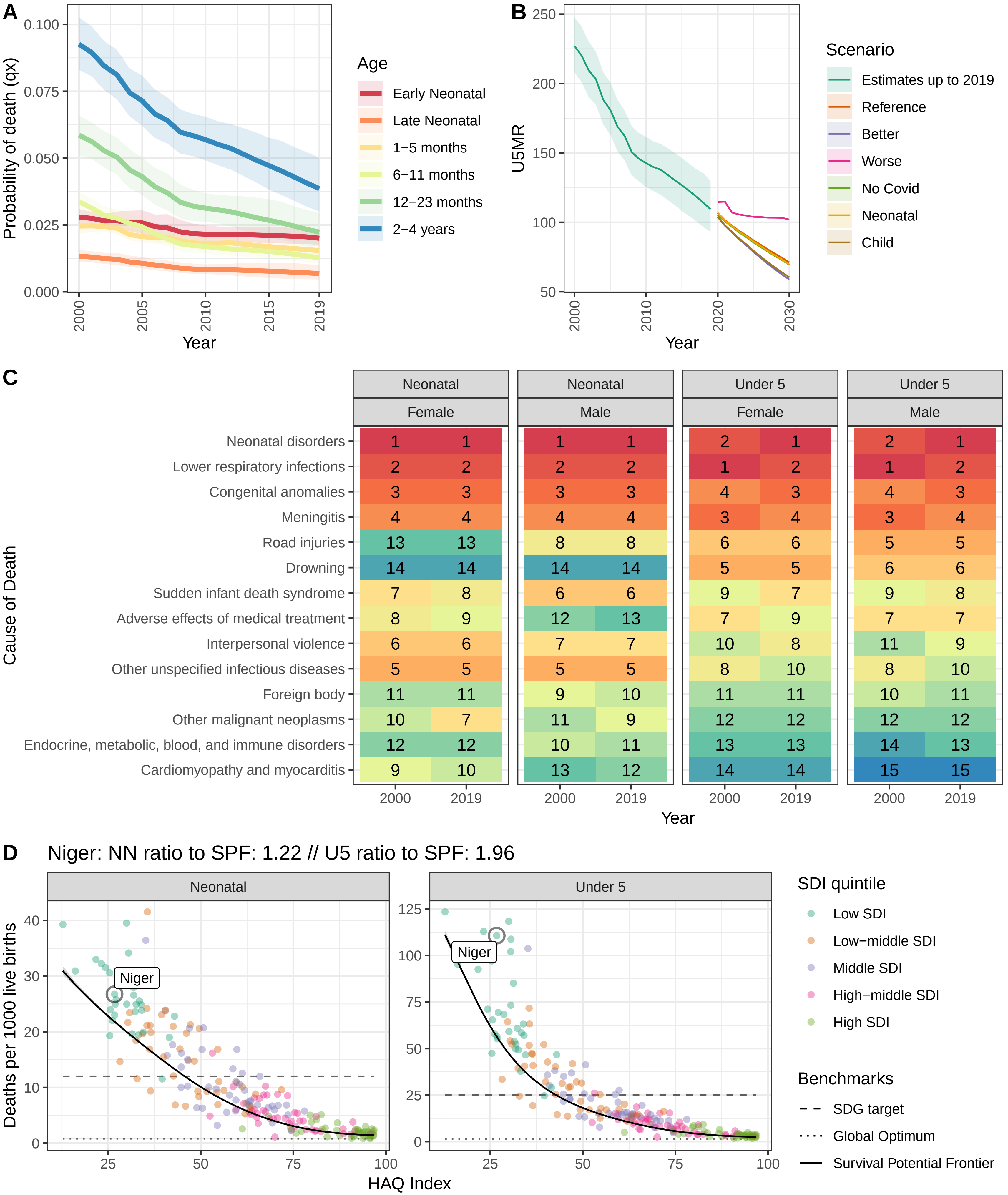

A
Probability of death (qx)
0.100
0.075
0.050
0.025
0.000
2000
2005
2010
2015
2019
Year
Age
Early Neonatal
Late Neonatal
1−5 months
6−11 months
12−23 months
2−4 years
B
U5MR
250
200
150
100
50
2000
2010
2020
2030
Year
Scenario
Estimates up to 2019
Reference
Better
Worse
No Covid
Neonatal
Child
C
Cause of Death
Neonatal Female
Neonatal Male
Under 5 Female
Under 5 Male
Neonatal disorders 1 1 | 1 1 | 2 1 | 2 1
Lower respiratory infections 2 2 | 2 2 | 1 2 | 1 2
Congenital anomalies 3 3 | 3 3 | 4 3 | 4 3
Meningitis 4 4 | 4 4 | 3 4 | 3 4
Road injuries 13 13 | 8 8 | 6 6 | 5 5
Drowning 14 14 | 14 14 | 5 5 | 6 6
Sudden infant death syndrome 7 8 | 6 6 | 9 7 | 9 8
Adverse effects of medical treatment 8 9 | 12 13 | 7 9 | 7 7
Interpersonal violence 6 6 | 7 7 | 10 8 | 11 9
Other unspecified infectious diseases 5 5 | 5 5 | 8 10 | 8 10
Foreign body 11 11 | 9 10 | 11 11 | 10 11
Other malignant neoplasms 10 7 | 11 9 | 12 12 | 12 12
Endocrine, metabolic, blood, and immune disorders 12 12 | 10 11 | 13 13 | 14 13
Cardiomyopathy and myocarditis 9 10 | 13 12 | 14 14 | 15 15
2000 2019
Year
D
Niger: NN ratio to SPF: 1.22 // U5 ratio to SPF: 1.96
Neonatal
Under 5
Deaths per 1000 live births
40
30
20
10
0
125
100
75
50
25
0
Niger
25
50
75
100
HAQ Index
SDI quintile
Low SDI
Low−middle SDI
Middle SDI
High−middle SDI
High SDI
Benchmarks
SDG target
Global Optimum
Survival Potential Frontier

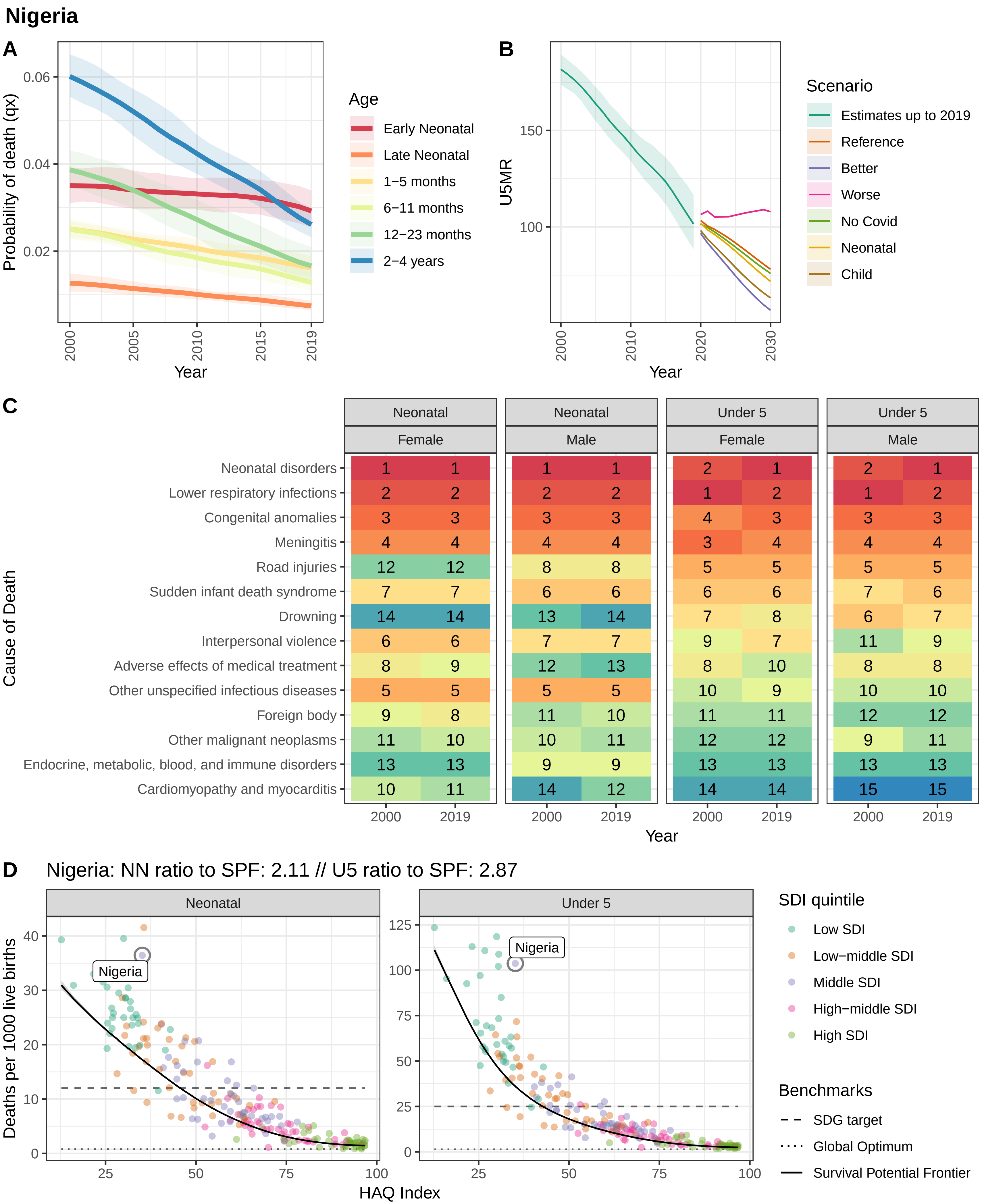

Nigeria
A
Probability of death (qx)
Year
Age
Early Neonatal
Late Neonatal
1−5 months
6−11 months
12−23 months
2−4 years
B
U5MR
Year
Scenario
Estimates up to 2019
Reference
Better
Worse
No Covid
Neonatal
Child
C
Cause of Death
Neonatal Female
Neonatal Male
Under 5 Female
Under 5 Male
Neonatal disorders
Lower respiratory infections
Congenital anomalies
Meningitis
Road injuries
Sudden infant death syndrome
Drowning
Interpersonal violence
Adverse effects of medical treatment
Other unspecified infectious diseases
Foreign body
Other malignant neoplasms
Endocrine, metabolic, blood, and immune disorders
Cardiomyopathy and myocarditis
Year
D
Nigeria: NN ratio to SPF: 2.11 // U5 ratio to SPF: 2.87
Neonatal
Under 5
Deaths per 1000 live births
HAQ Index
Nigeria
SDI quintile
Low SDI
Low−middle SDI
Middle SDI
High−middle SDI
High SDI
Benchmarks
SDG target
Global Optimum
Survival Potential Frontier

Sao Tome and Principe

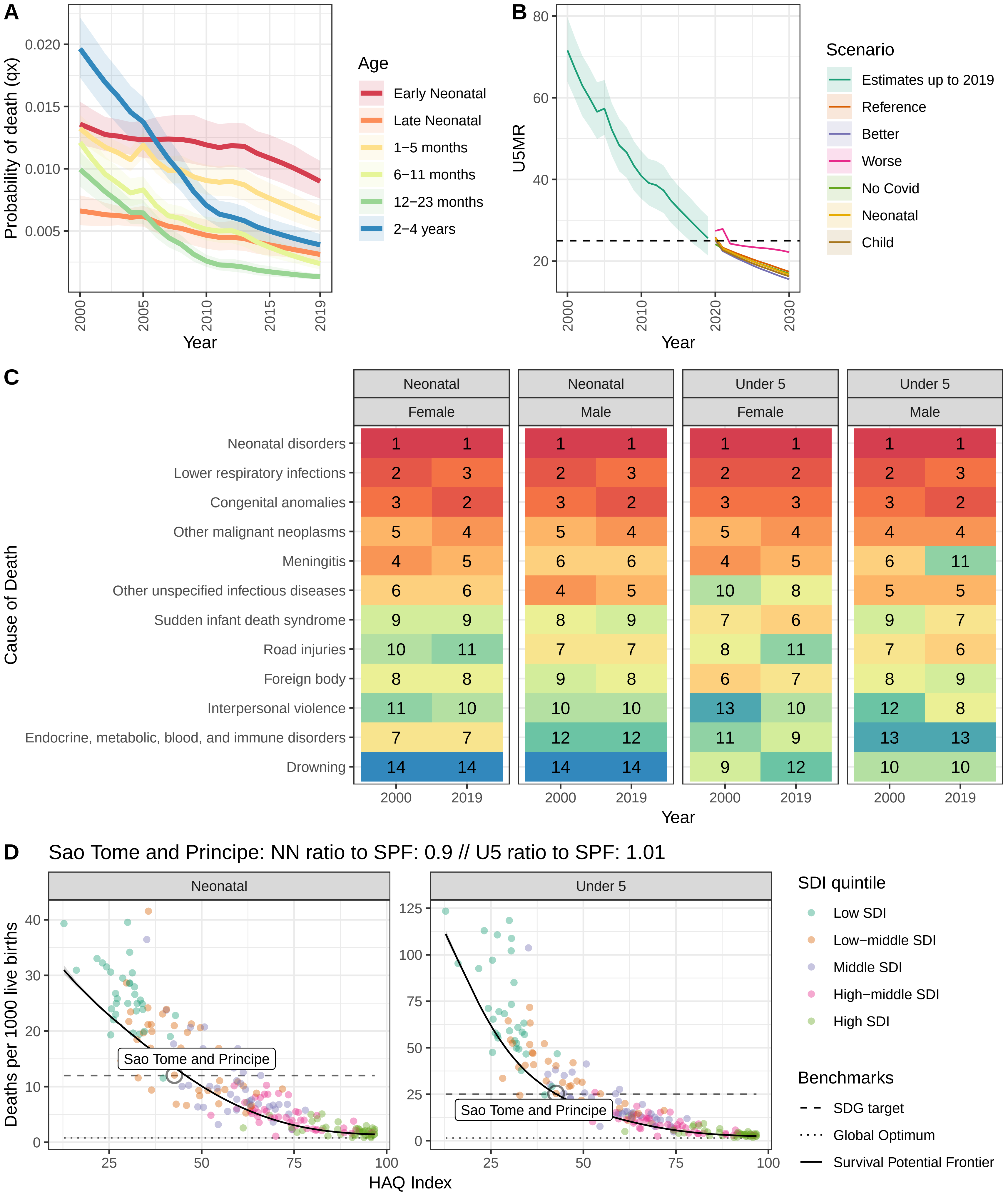

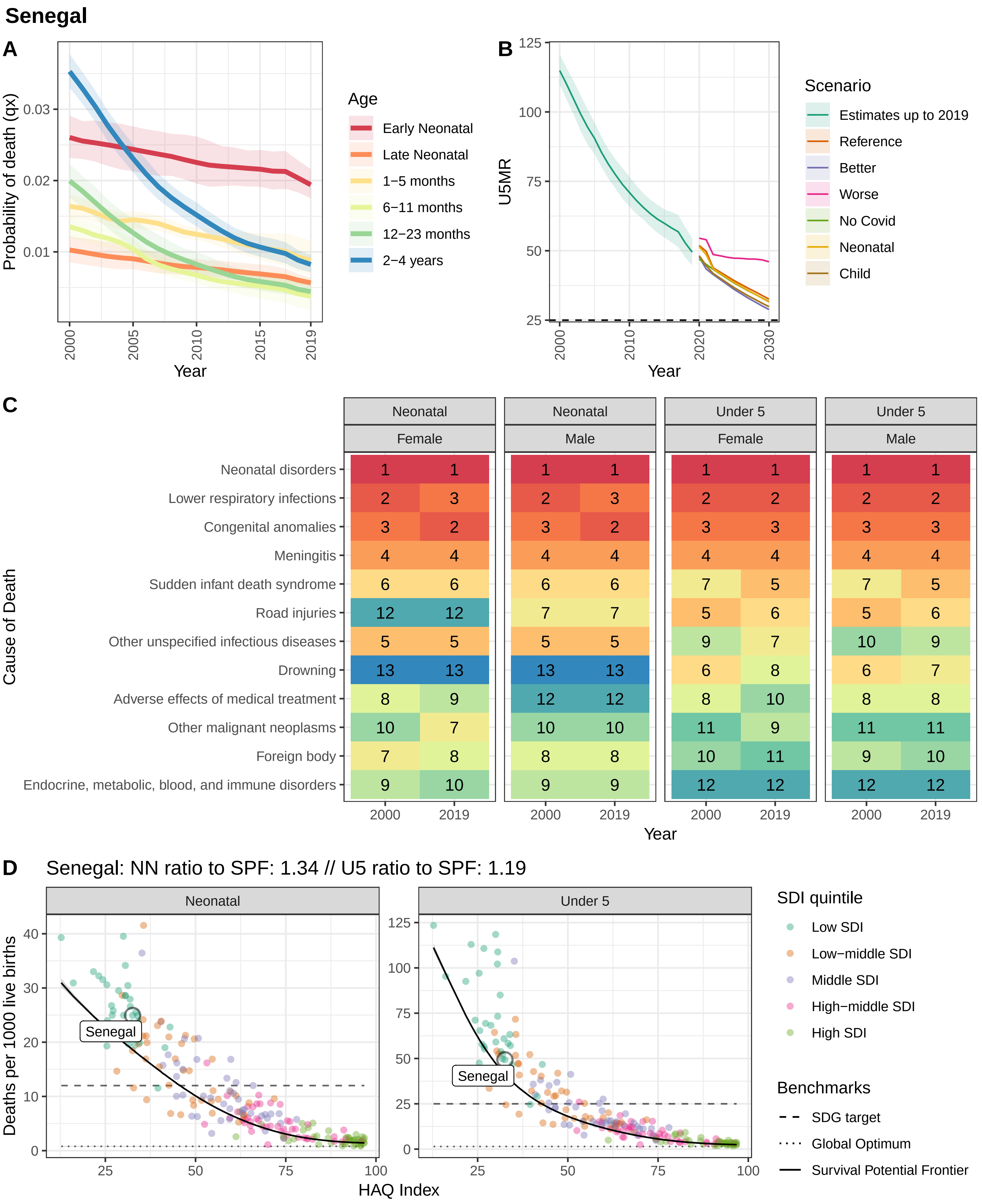
Senegal
A
Probability of death (qx)
0.03
0.02
0.01
2000
2005
2010
2015
2019
Year
Age
Early Neonatal
Late Neonatal
1−5 months
6−11 months
12−23 months
2−4 years
B
U5MR
125
100
75
50
25
2000
2010
2020
2030
Year
Scenario
Estimates up to 2019
Reference
Better
Worse
No Covid
Neonatal
Child
C
Neonatal Female
Neonatal Male
Under 5 Female
Under 5 Male
Cause of Death
Neonatal disorders 1 1 1 1 1 1 1 1
Lower respiratory infections 2 3 2 3 2 2 2 2
Congenital anomalies 3 2 3 2 3 3 3 3
Meningitis 4 4 4 4 4 4 4 4
Sudden infant death syndrome 6 6 6 6 7 5 7 5
Road injuries 12 12 7 7 5 6 5 6
Other unspecified infectious diseases 5 5 5 5 9 7 10 9
Drowning 13 13 13 13 6 8 6 7
Adverse effects of medical treatment 8 9 12 12 8 10 8 8
Other malignant neoplasms 10 7 10 10 11 9 11 11
Foreign body 7 8 8 8 10 11 9 10
Endocrine, metabolic, blood, and immune disorders 9 10 9 9 12 12 12 12
2000
2019
Year
D
Senegal: NN ratio to SPF: 1.34 // U5 ratio to SPF: 1.19
Neonatal
Under 5
Deaths per 1000 live births
40
30
20
10
0
125
100
75
50
25
0
Senegal
Senegal
25
50
75
100
HAQ Index
SDI quintile
Low SDI
Low−middle SDI
Middle SDI
High−middle SDI
High SDI
Benchmarks
SDG target
Global Optimum
Survival Potential Frontier

Sierra Leone

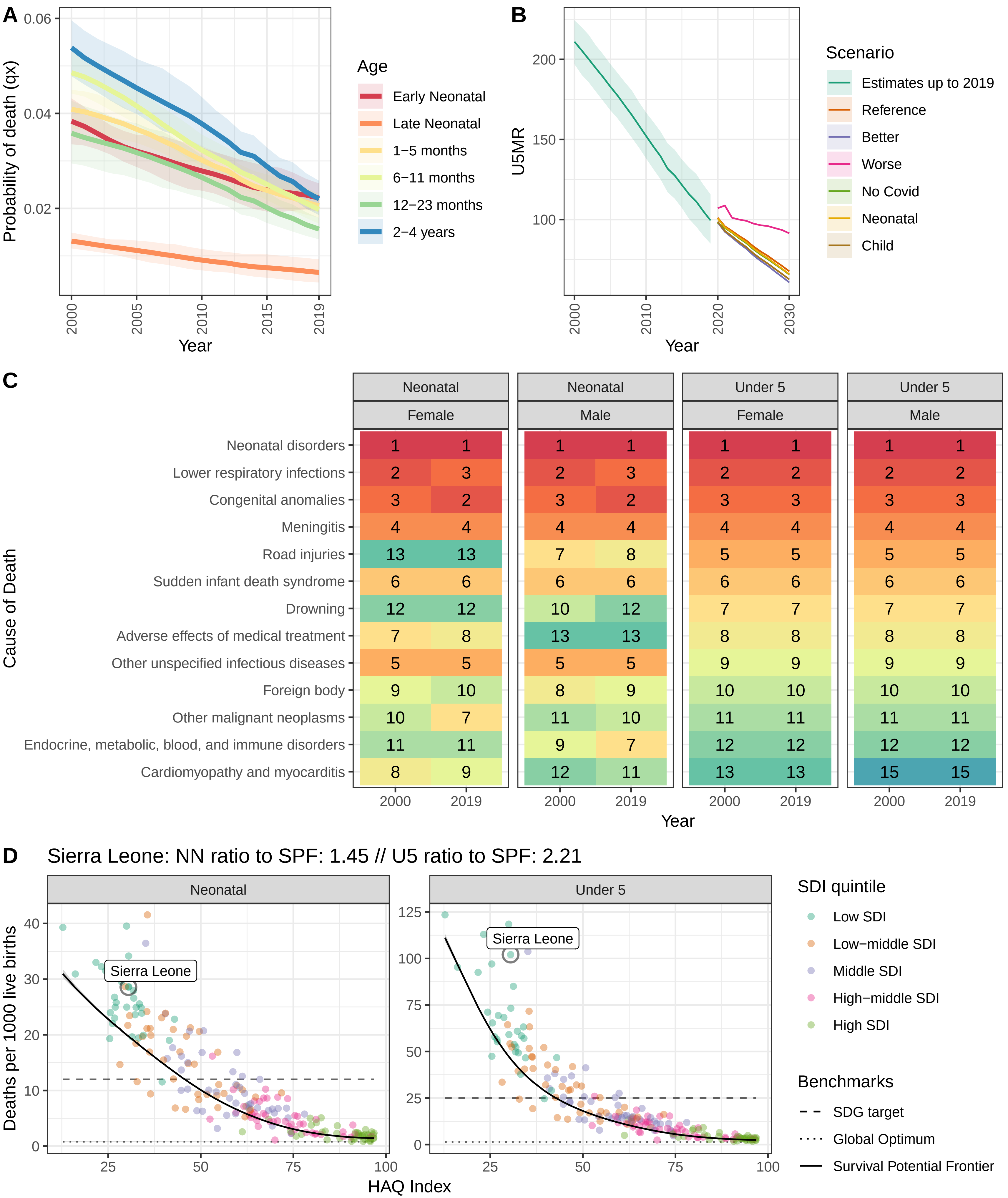

Togo

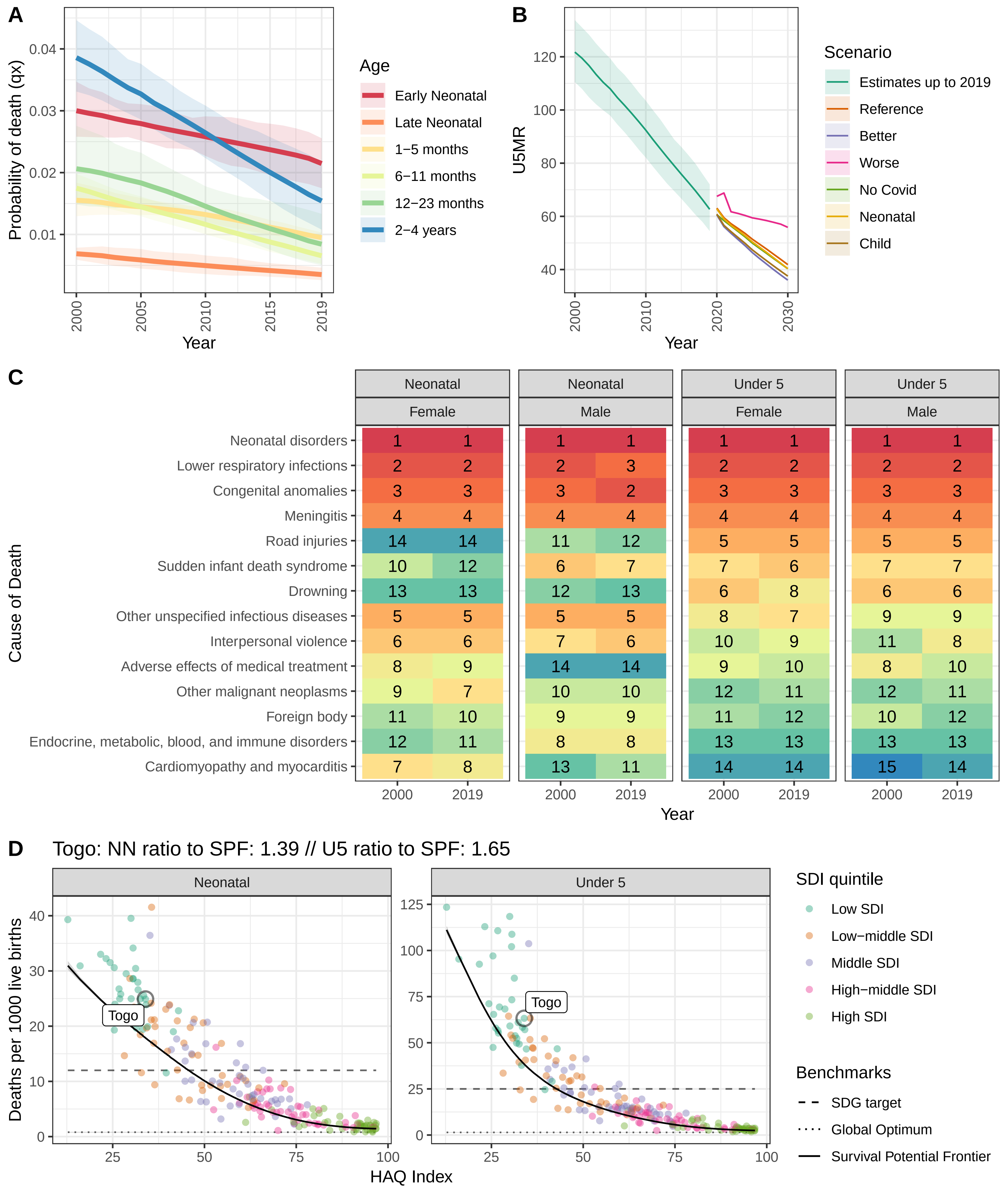
A
Probability of death (qx)
Year
Age
Early Neonatal
Late Neonatal
1−5 months
6−11 months
12−23 months
2−4 years
B
U5MR
Year
Scenario
Estimates up to 2019
Reference
Better
Worse
No Covid
Neonatal
Child
C
Cause of Death
Neonatal Female
Neonatal Male
Under 5 Female
Under 5 Male
Neonatal disorders 1 1 1 1 1 1 1 1
Lower respiratory infections 2 2 2 3 2 2 2 2
Congenital anomalies 3 3 3 2 3 3 3 3
Meningitis 4 4 4 4 4 4 4 4
Road injuries 14 14 11 12 5 5 5 5
Sudden infant death syndrome 10 12 6 7 7 6 7 7
Drowning 13 13 12 13 6 8 6 6
Other unspecified infectious diseases 5 5 5 5 8 7 9 9
Interpersonal violence 6 6 7 6 10 9 11 8
Adverse effects of medical treatment 8 9 14 14 9 10 8 10
Other malignant neoplasms 9 7 10 10 12 11 12 11
Foreign body 11 10 9 9 11 12 10 12
Endocrine, metabolic, blood, and immune disorders 12 11 8 8 13 13 13 13
Cardiomyopathy and myocarditis 7 8 13 11 14 14 15 14
2000 2019
Year
D
Togo: NN ratio to SPF: 1.39 // U5 ratio to SPF: 1.65
Neonatal
Under 5
Togo
Deaths per 1000 live births
HAQ Index
SDI quintile
Low SDI
Low−middle SDI
Middle SDI
High−middle SDI
High SDI
Benchmarks
SDG target
Global Optimum
Survival Potential Frontier

Fiji

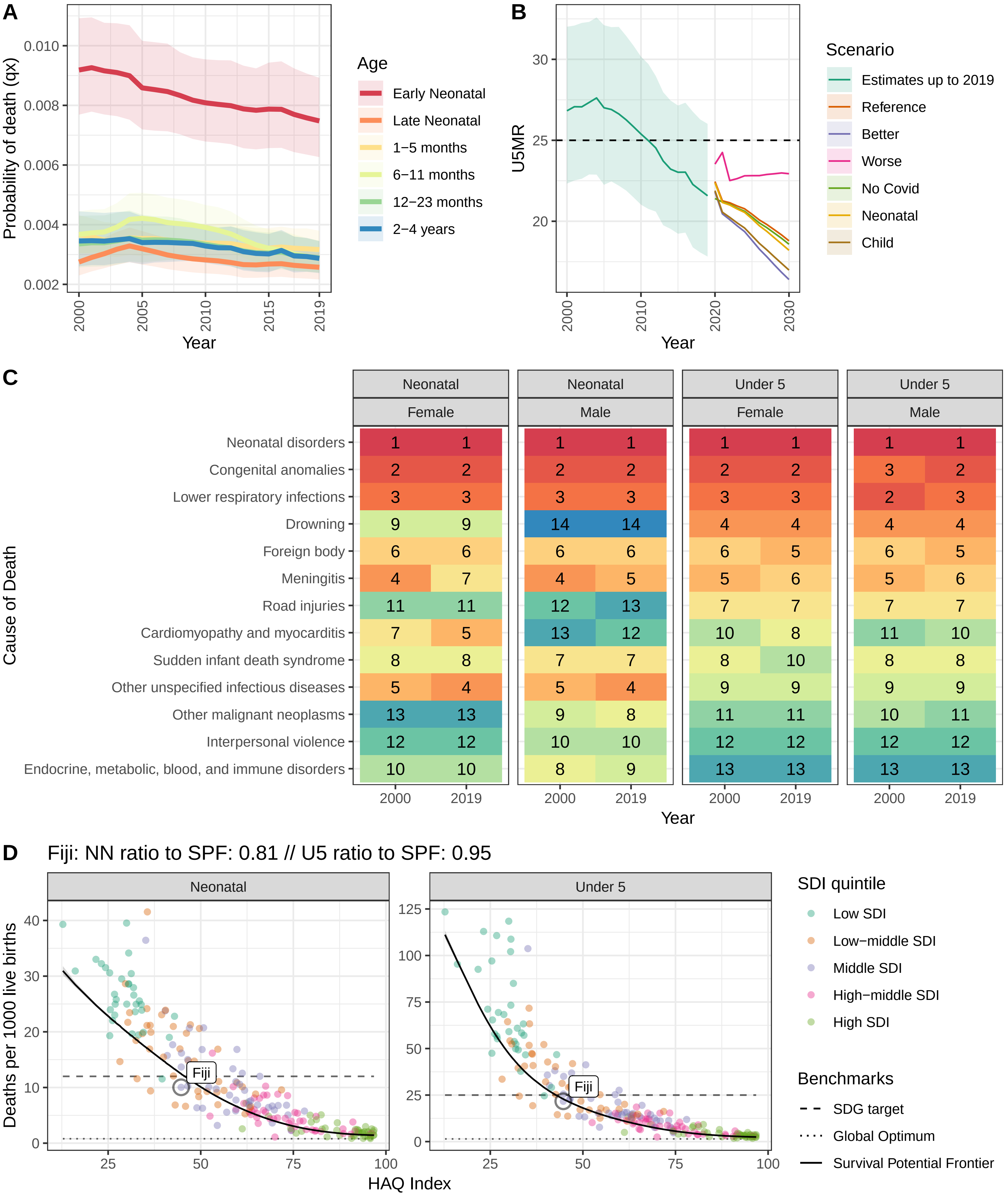
A
Probability of death (qx)
0.010
0.008
0.006
0.004
0.002
2000
2005
2010
2015
2019
Year
Age
Early Neonatal
Late Neonatal
1−5 months
6−11 months
12−23 months
2−4 years
B
U5MR
30
25
20
2000
2010
2020
2030
Year
Scenario
Estimates up to 2019
Reference
Better
Worse
No Covid
Neonatal
Child
C
Neonatal Female
Neonatal Male
Under 5 Female
Under 5 Male
Cause of Death
Neonatal disorders 1 1 1 1 1 1 1 1
Congenital anomalies 2 2 2 2 2 2 3 2
Lower respiratory infections 3 3 3 3 3 3 2 3
Drowning 9 9 14 14 4 4 4 4
Foreign body 6 6 6 6 6 5 6 5
Meningitis 4 7 4 5 5 6 5 6
Road injuries 11 11 12 13 7 7 7 7
Cardiomyopathy and myocarditis 7 5 13 12 10 8 11 10
Sudden infant death syndrome 8 8 7 7 8 10 8 8
Other unspecified infectious diseases 5 4 5 4 9 9 9 9
Other malignant neoplasms 13 13 9 8 11 11 10 11
Interpersonal violence 12 12 10 10 12 12 12 12
Endocrine, metabolic, blood, and immune disorders 10 10 8 9 13 13 13 13
2000
2019
Year
D
Fiji: NN ratio to SPF: 0.81 // U5 ratio to SPF: 0.95
Neonatal
Under 5
Deaths per 1000 live births
40
30
20
10
0
125
100
75
50
25
0
Fiji
25
50
75
100
HAQ Index
SDI quintile
Low SDI
Low−middle SDI
Middle SDI
High−middle SDI
High SDI
Benchmarks
SDG target
Global Optimum
Survival Potential Frontier

Kiribati

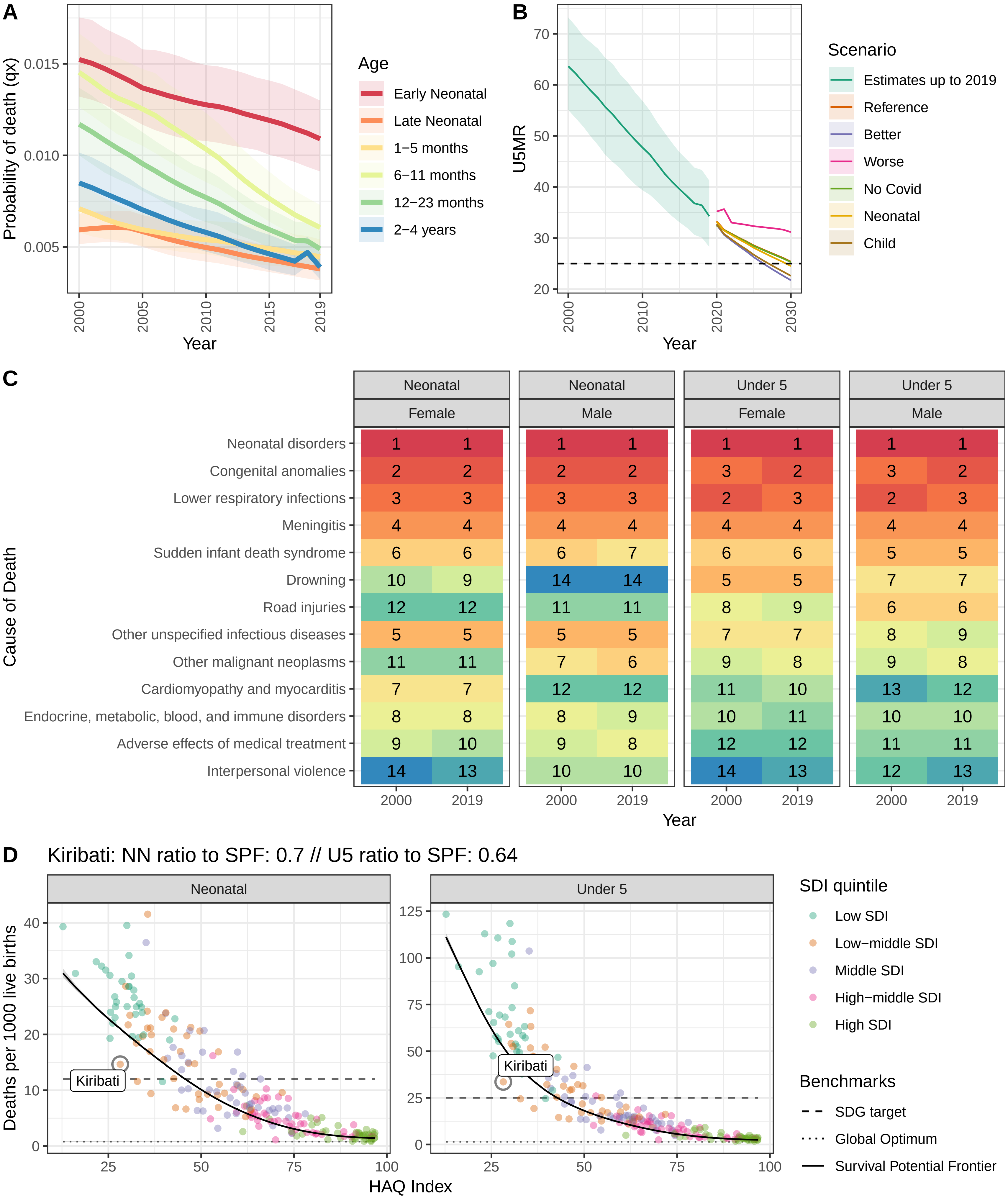

A
Probability of death (qx)
Year
Age
Early Neonatal
Late Neonatal
1−5 months
6−11 months
12−23 months
2−4 years
B
U5MR
Year
Scenario
Estimates up to 2019
Reference
Better
Worse
No Covid
Neonatal
Child
C
Cause of Death
Neonatal Female
Neonatal Male
Under 5 Female
Under 5 Male
Neonatal disorders 1 1 1 1 1 1 1 1
Congenital anomalies 2 2 2 2 3 2 3 2
Lower respiratory infections 3 3 3 3 2 3 2 3
Meningitis 4 4 4 4 4 4 4 4
Sudden infant death syndrome 6 6 6 7 6 6 5 5
Drowning 10 9 14 14 5 5 7 7
Road injuries 12 12 11 11 8 9 6 6
Other unspecified infectious diseases 5 5 5 5 7 7 8 9
Other malignant neoplasms 11 11 7 6 9 8 9 8
Cardiomyopathy and myocarditis 7 7 12 12 11 10 13 12
Endocrine, metabolic, blood, and immune disorders 8 8 8 9 10 11 10 10
Adverse effects of medical treatment 9 10 9 8 12 12 11 11
Interpersonal violence 14 13 10 10 14 13 12 13
2000 2019
Year
D
Kiribati: NN ratio to SPF: 0.7 // U5 ratio to SPF: 0.64
Neonatal
Under 5
Kiribati
Deaths per 1000 live births
HAQ Index
SDI quintile
Low SDI
Low−middle SDI
Middle SDI
High−middle SDI
High SDI
Benchmarks
SDG target
Global Optimum
Survival Potential Frontier

Marshall Islands

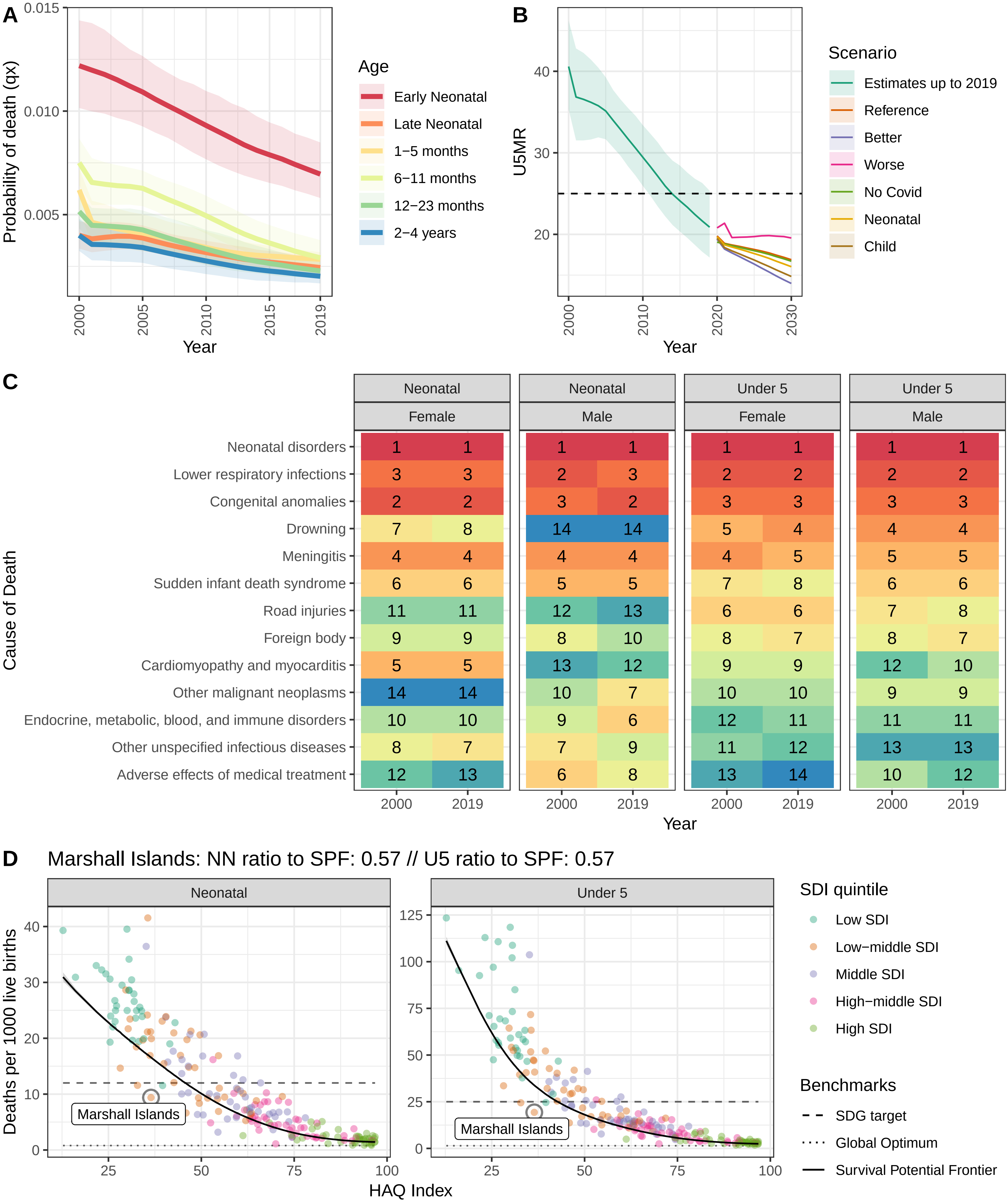

Micronesia (Federated States of)

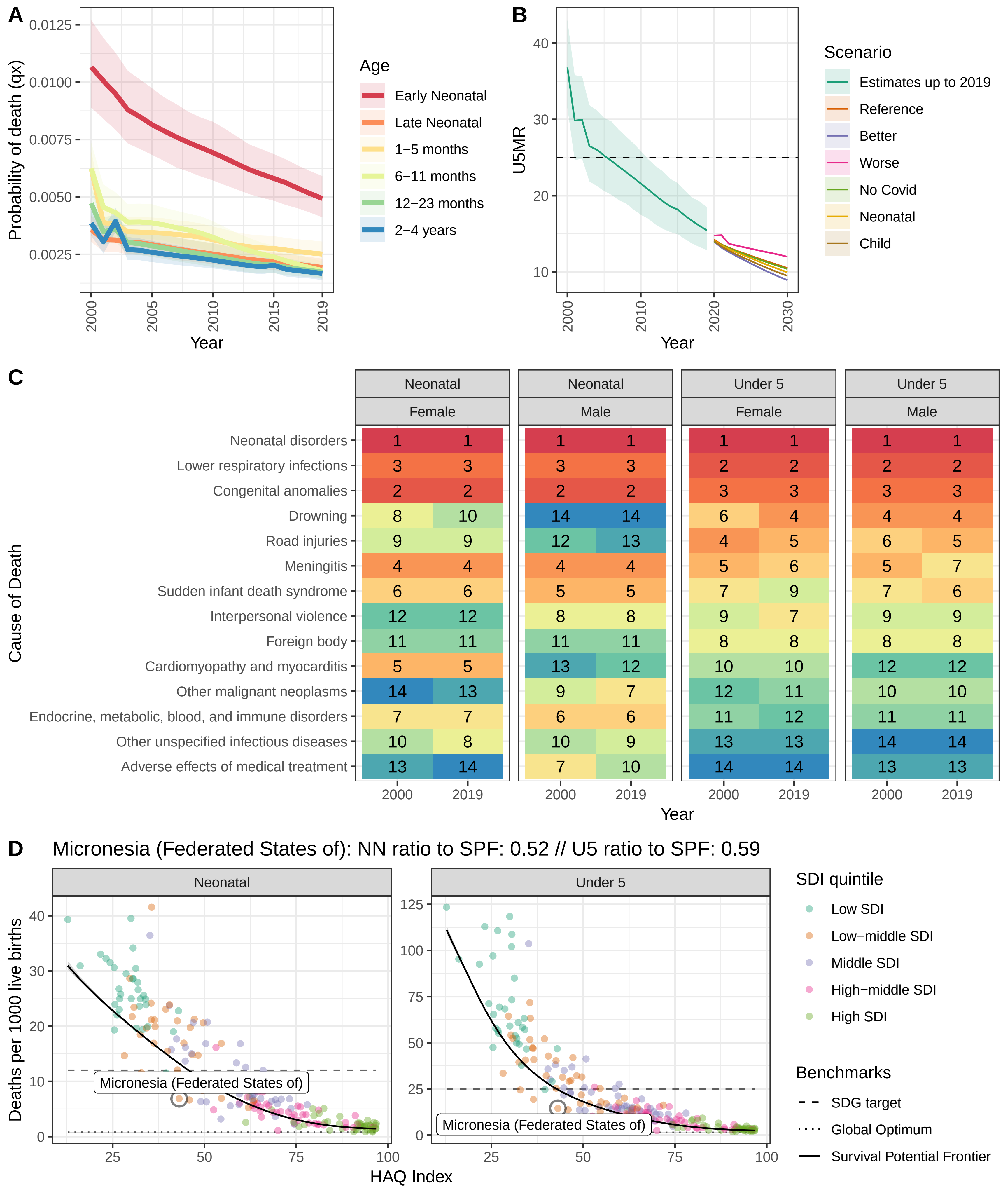

Papua New Guinea

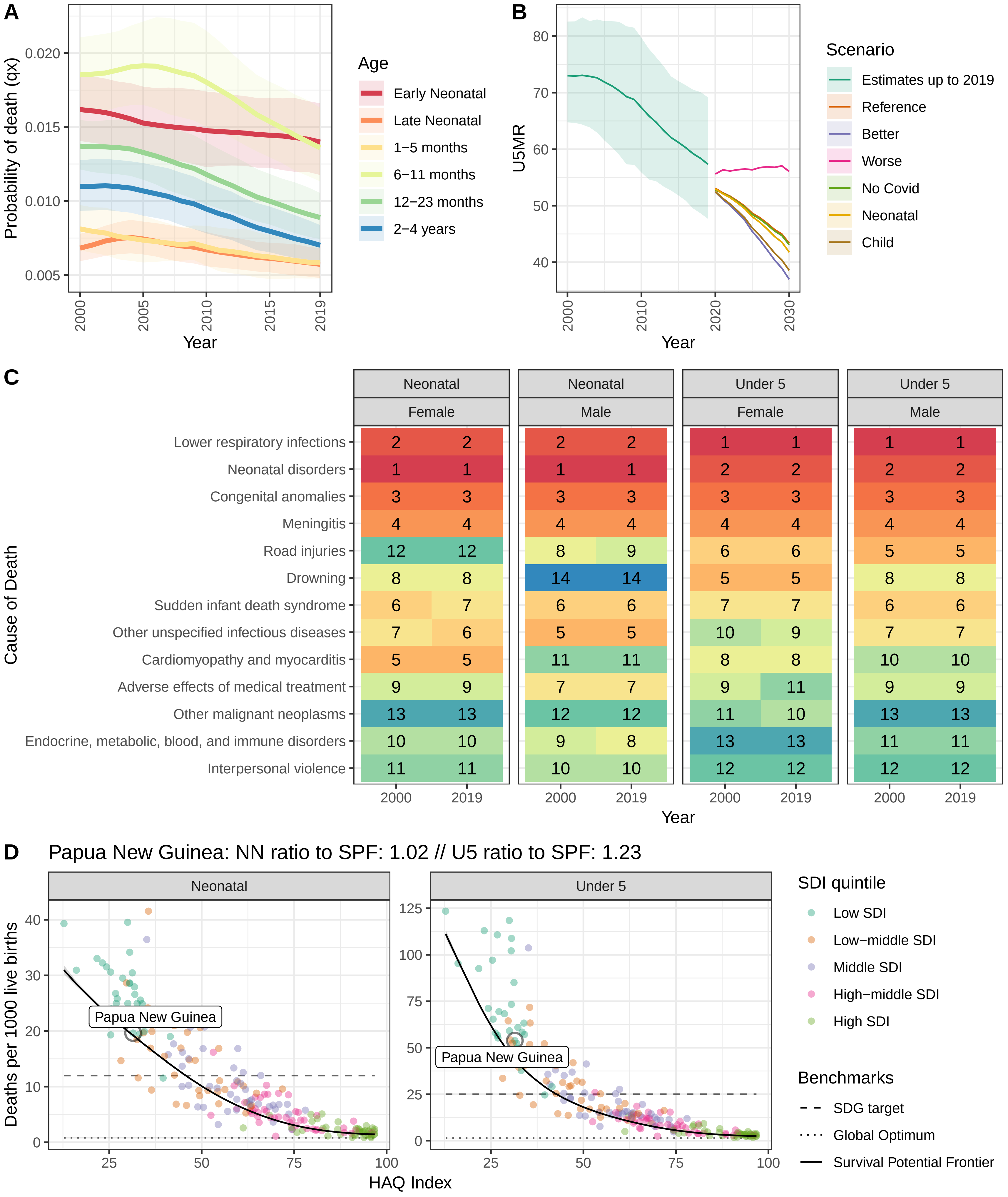

Samoa

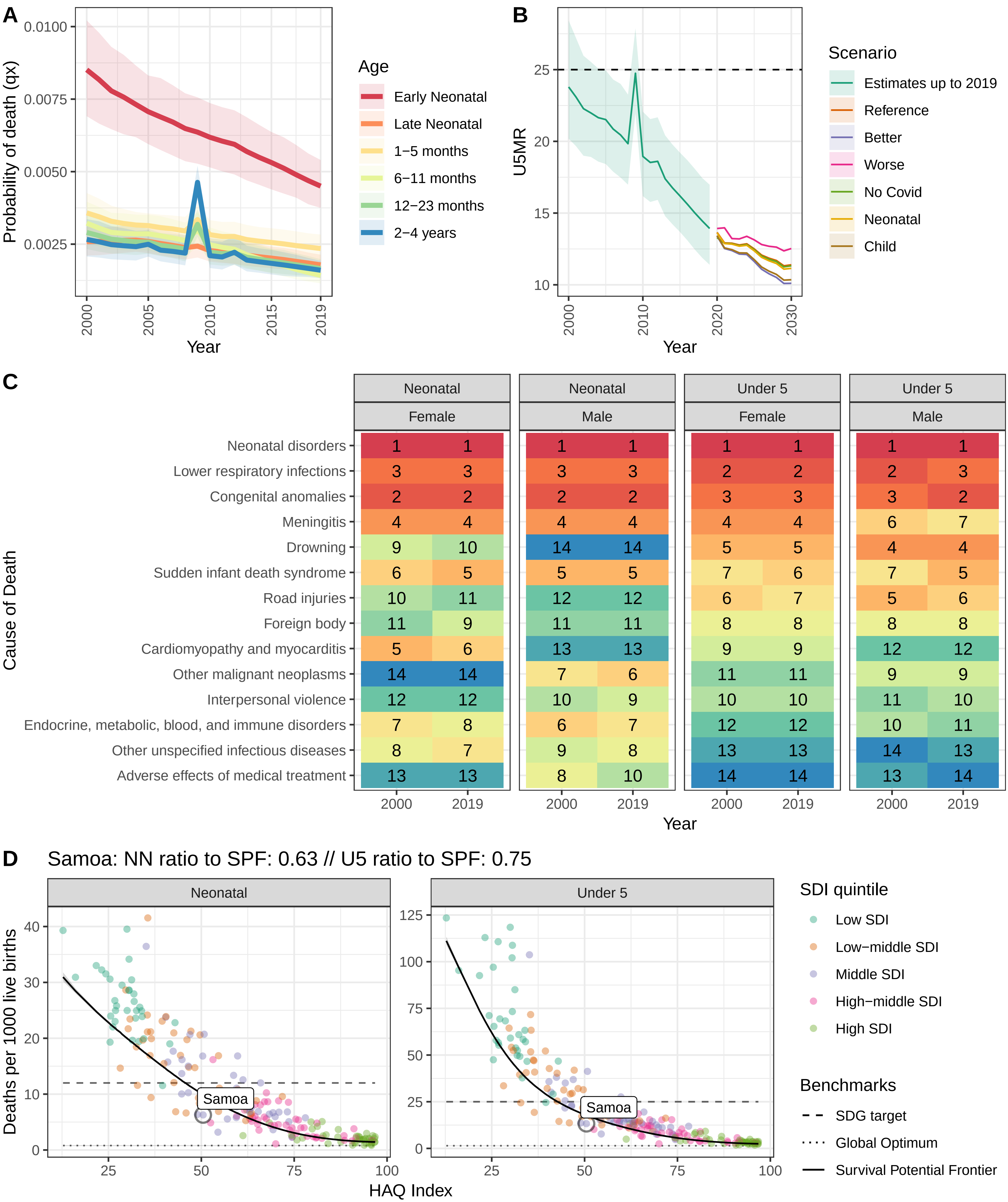
A
Probability of death (qx)
0.0100
0.0075
0.0050
0.0025
2000
2005
2010
2015
2019
Year
Age
Early Neonatal
Late Neonatal
1−5 months
6−11 months
12−23 months
2−4 years
B
U5MR
25
20
15
10
2000
2010
2020
2030
Year
Scenario
Estimates up to 2019
Reference
Better
Worse
No Covid
Neonatal
Child
C
Cause of Death
Neonatal Female
Neonatal Male
Under 5 Female
Under 5 Male
Neonatal disorders 1 1 1 1 1 1 1 1
Lower respiratory infections 3 3 3 3 2 2 2 3
Congenital anomalies 2 2 2 2 3 3 3 2
Meningitis 4 4 4 4 4 4 6 7
Drowning 9 10 14 14 5 5 4 4
Sudden infant death syndrome 6 5 5 5 7 6 7 5
Road injuries 10 11 12 12 6 7 5 6
Foreign body 11 9 11 11 8 8 8 8
Cardiomyopathy and myocarditis 5 6 13 13 9 9 12 12
Other malignant neoplasms 14 14 7 6 11 11 9 9
Interpersonal violence 12 12 10 9 10 10 11 10
Endocrine, metabolic, blood, and immune disorders 7 8 6 7 12 12 10 11
Other unspecified infectious diseases 8 7 9 8 13 13 14 13
Adverse effects of medical treatment 13 13 8 10 14 14 13 14
2000
2019
Year
D
Samoa: NN ratio to SPF: 0.63 // U5 ratio to SPF: 0.75
Neonatal
Under 5
Deaths per 1000 live births
40
30
20
10
0
125
100
75
50
25
0
Samoa
HAQ Index
SDI quintile
Low SDI
Low−middle SDI
Middle SDI
High−middle SDI
High SDI
Benchmarks
SDG target
Global Optimum
Survival Potential Frontier

Solomon Islands

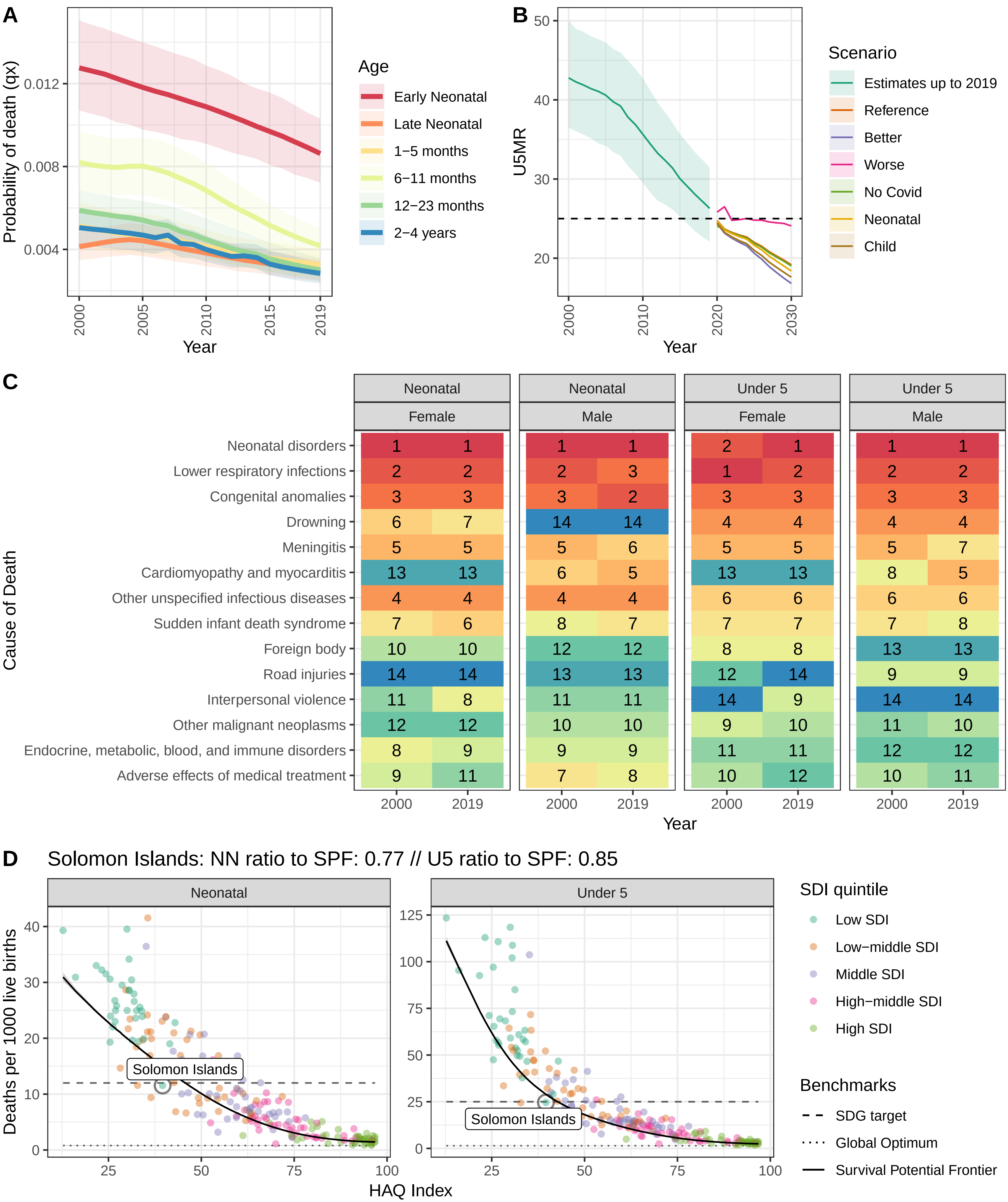

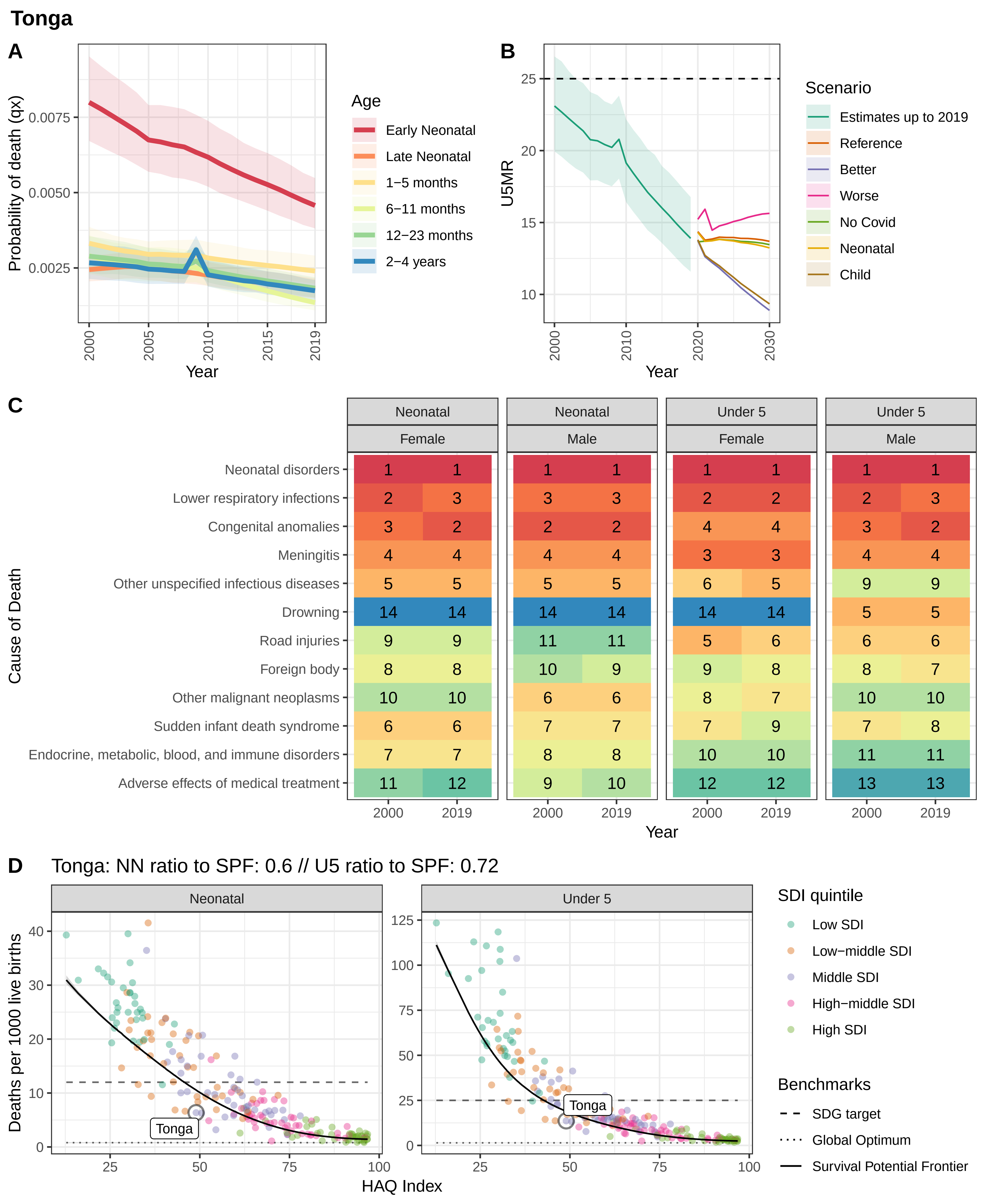
Tonga
A
Probability of death (qx)
0.0075
0.0050
0.0025
2000
2005
2010
2015
2019
Year
Age
Early Neonatal
Late Neonatal
1−5 months
6−11 months
12−23 months
2−4 years
B
U5MR
25
20
15
10
2000
2010
2020
2030
Year
Scenario
Estimates up to 2019
Reference
Better
Worse
No Covid
Neonatal
Child
C
Neonatal
Female
Neonatal
Male
Under 5
Female
Under 5
Male
Cause of Death
Neonatal disorders
Lower respiratory infections
Congenital anomalies
Meningitis
Other unspecified infectious diseases
Drowning
Road injuries
Foreign body
Other malignant neoplasms
Sudden infant death syndrome
Endocrine, metabolic, blood, and immune disorders
Adverse effects of medical treatment
1 1 1 1 1 1 1 1
2 3 3 3 2 2 2 3
3 2 2 2 4 4 3 2
4 4 4 4 3 3 4 4
5 5 5 5 6 5 9 9
14 14 14 14 14 14 5 5
9 9 11 11 5 6 6 6
8 8 10 9 9 8 8 7
10 10 6 6 8 7 10 10
6 6 7 7 7 9 7 8
7 7 8 8 10 10 11 11
11 12 9 10 12 12 13 13
2000
2019
Year
D
Tonga: NN ratio to SPF: 0.6 // U5 ratio to SPF: 0.72
Neonatal
Under 5
Deaths per 1000 live births
40
30
20
10
0
125
100
75
50
25
0
Tonga
Tonga
25
50
75
100
HAQ Index
SDI quintile
Low SDI
Low−middle SDI
Middle SDI
High−middle SDI
High SDI
Benchmarks
SDG target
Global Optimum
Survival Potential Frontier

American Samoa

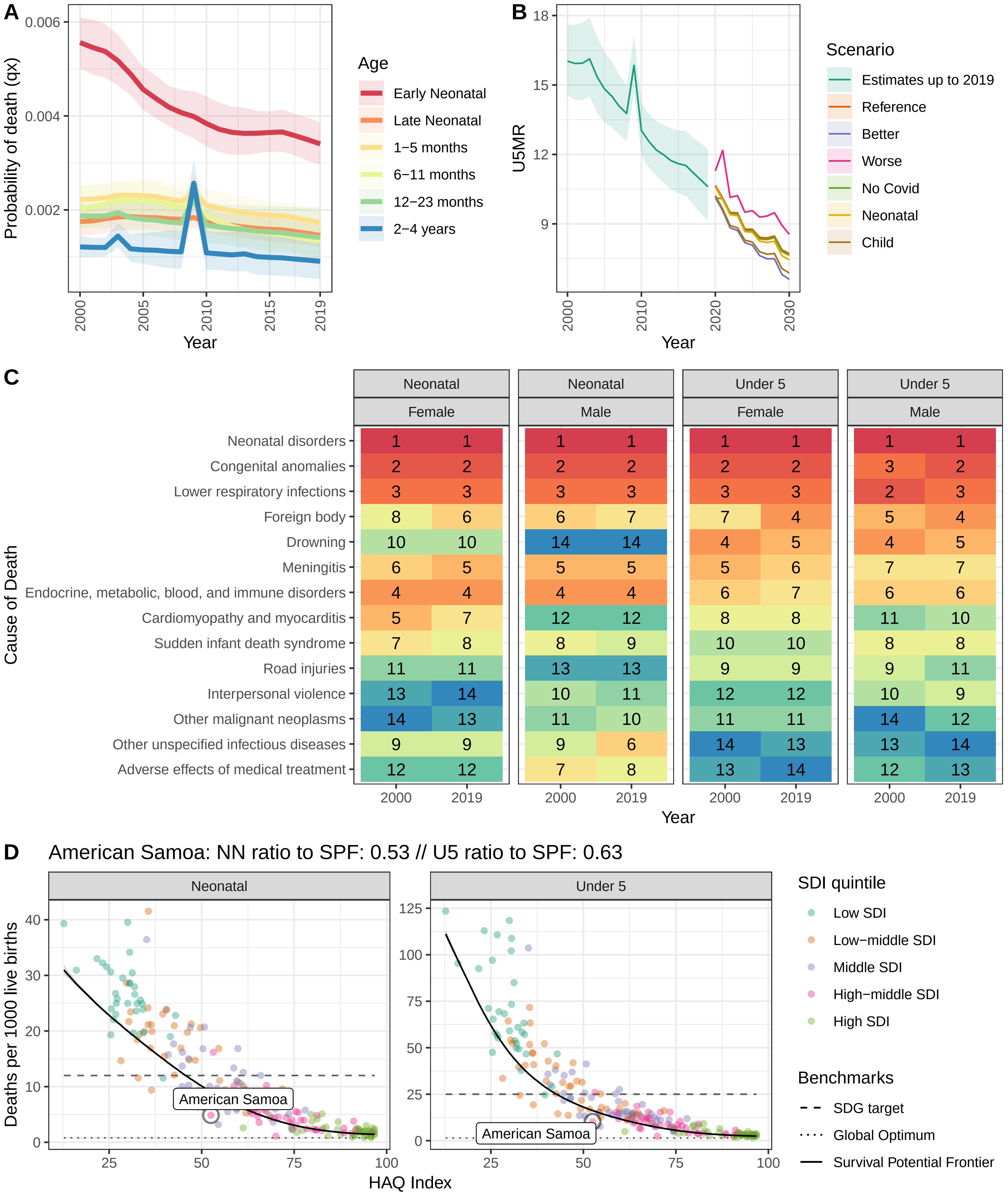

Vanuatu

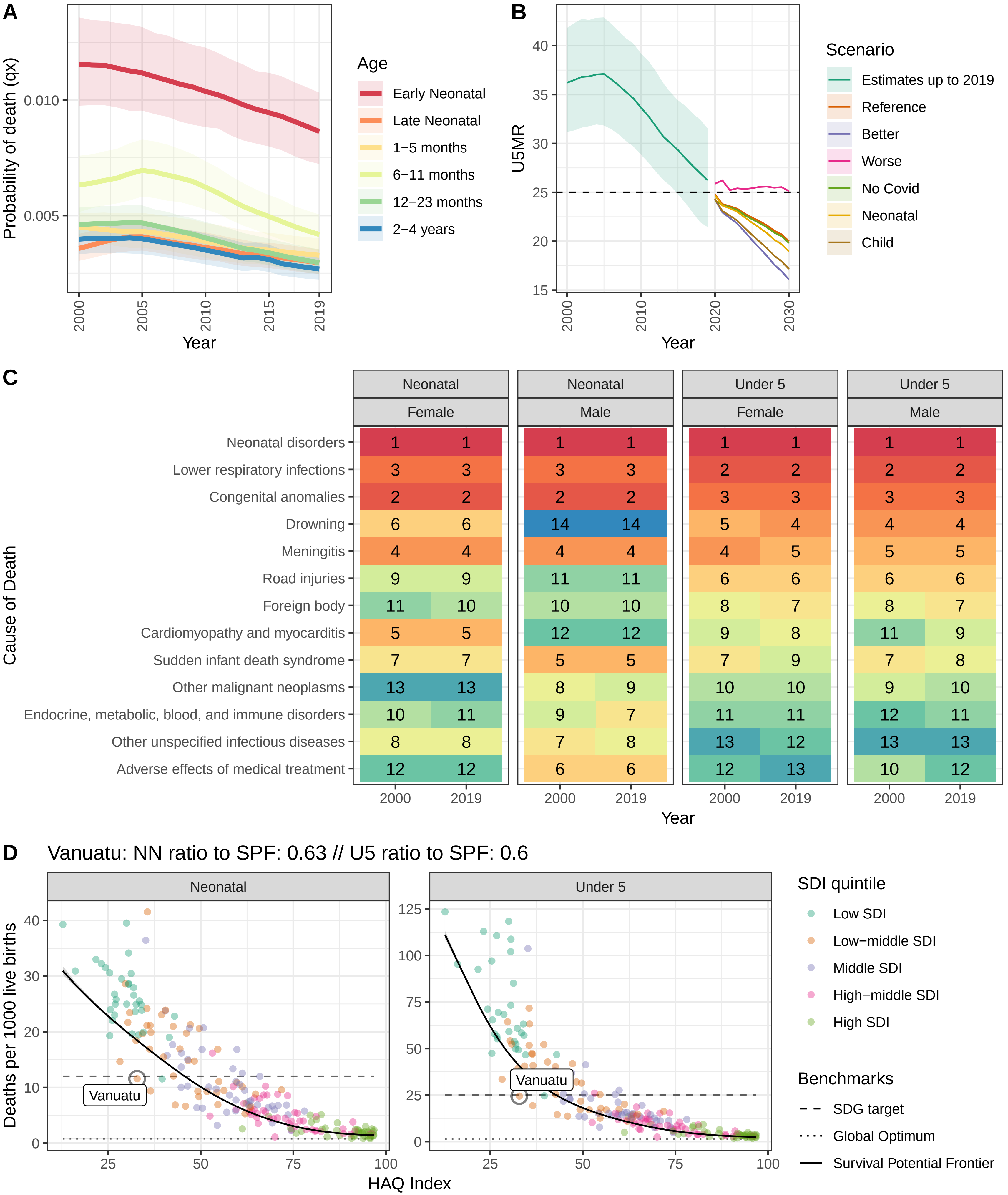
A
Probability of death (qx)
0.010
0.005
2000
2005
2010
2015
2019
Year
Age
Early Neonatal
Late Neonatal
1−5 months
6−11 months
12−23 months
2−4 years
B
U5MR
40
35
30
25
20
15
2000
2010
2020
2030
Year
Scenario
Estimates up to 2019
Reference
Better
Worse
No Covid
Neonatal
Child
C
Cause of Death
Neonatal
Female
Neonatal
Male
Under 5
Female
Under 5
Male
Neonatal disorders 1 1 1 1 1 1 1 1
Lower respiratory infections 3 3 3 3 2 2 2 2
Congenital anomalies 2 2 2 2 3 3 3 3
Drowning 6 6 14 14 5 4 4 4
Meningitis 4 4 4 4 4 5 5 5
Road injuries 9 9 11 11 6 6 6 6
Foreign body 11 10 10 10 8 7 8 7
Cardiomyopathy and myocarditis 5 5 12 12 9 8 11 9
Sudden infant death syndrome 7 7 5 5 7 9 7 8
Other malignant neoplasms 13 13 8 9 10 10 9 10
Endocrine, metabolic, blood, and immune disorders 10 11 9 7 11 11 12 11
Other unspecified infectious diseases 8 8 7 8 13 12 13 13
Adverse effects of medical treatment 12 12 6 6 12 13 10 12
2000
2019
Year
D
Vanuatu: NN ratio to SPF: 0.63 // U5 ratio to SPF: 0.6
Neonatal
Under 5
Deaths per 1000 live births
40
30
20
10
0
125
100
75
50
25
0
Vanuatu
Vanuatu
25
50
75
100
HAQ Index
SDI quintile
Low SDI
Low−middle SDI
Middle SDI
High−middle SDI
High SDI
Benchmarks
SDG target
Global Optimum
Survival Potential Frontier

Bermuda

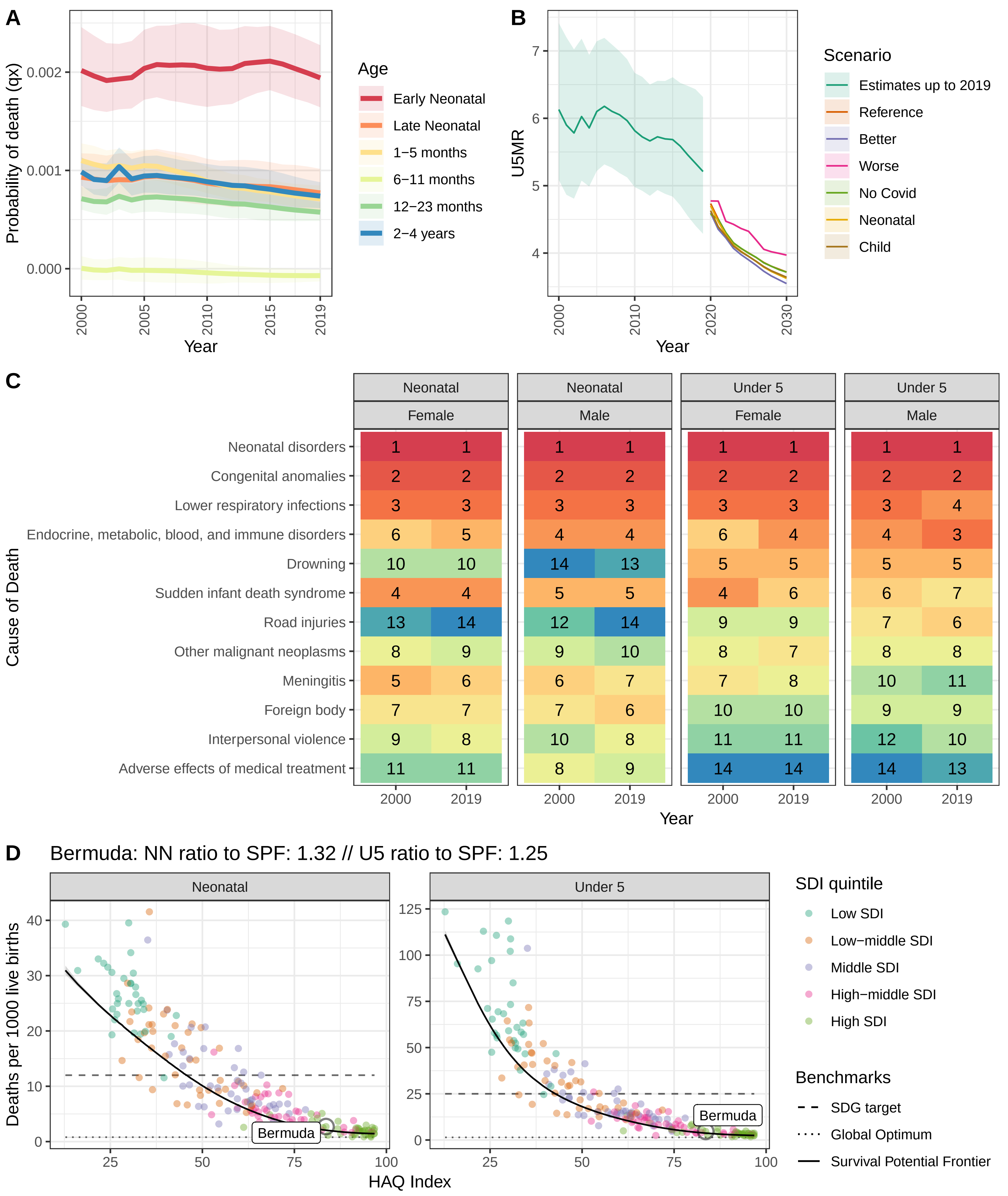
A
Probability of death (qx)
0.002
0.001
0.000
2000
2005
2010
2015
2019
Year
Age
Early Neonatal
Late Neonatal
1−5 months
6−11 months
12−23 months
2−4 years
B
U5MR
7
6
5
4
2000
2010
2020
2030
Year
Scenario
Estimates up to 2019
Reference
Better
Worse
No Covid
Neonatal
Child
C
Neonatal Female
Neonatal Male
Under 5 Female
Under 5 Male
Cause of Death
Neonatal disorders 1 1 1 1 1 1 1 1
Congenital anomalies 2 2 2 2 2 2 2 2
Lower respiratory infections 3 3 3 3 3 3 3 4
Endocrine, metabolic, blood, and immune disorders 6 5 4 4 6 4 4 3
Drowning 10 10 14 13 5 5 5 5
Sudden infant death syndrome 4 4 5 5 4 6 6 7
Road injuries 13 14 12 14 9 9 7 6
Other malignant neoplasms 8 9 9 10 8 7 8 8
Meningitis 5 6 6 7 7 8 10 11
Foreign body 7 7 7 6 10 10 9 9
Interpersonal violence 9 8 10 8 11 11 12 10
Adverse effects of medical treatment 11 11 8 9 14 14 14 13
2000 2019
Year
D
Bermuda: NN ratio to SPF: 1.32 // U5 ratio to SPF: 1.25
Neonatal
Under 5
Deaths per 1000 live births
40
30
20
10
0
125
100
75
50
25
0
25
50
75
100
HAQ Index
Bermuda
SDI quintile
Low SDI
Low−middle SDI
Middle SDI
High−middle SDI
High SDI
Benchmarks
SDG target
Global Optimum
Survival Potential Frontier

Cook Islands

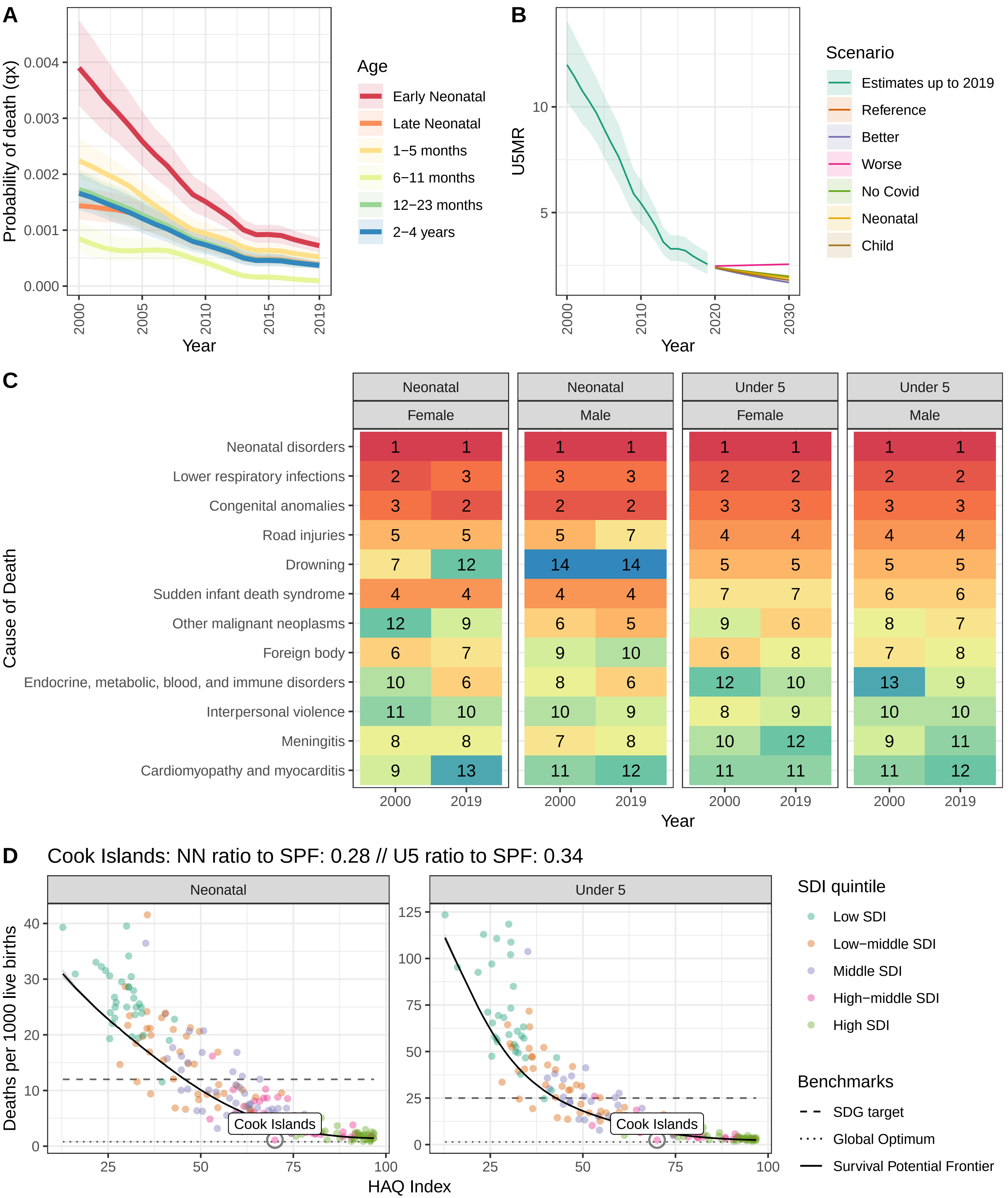

Armenia

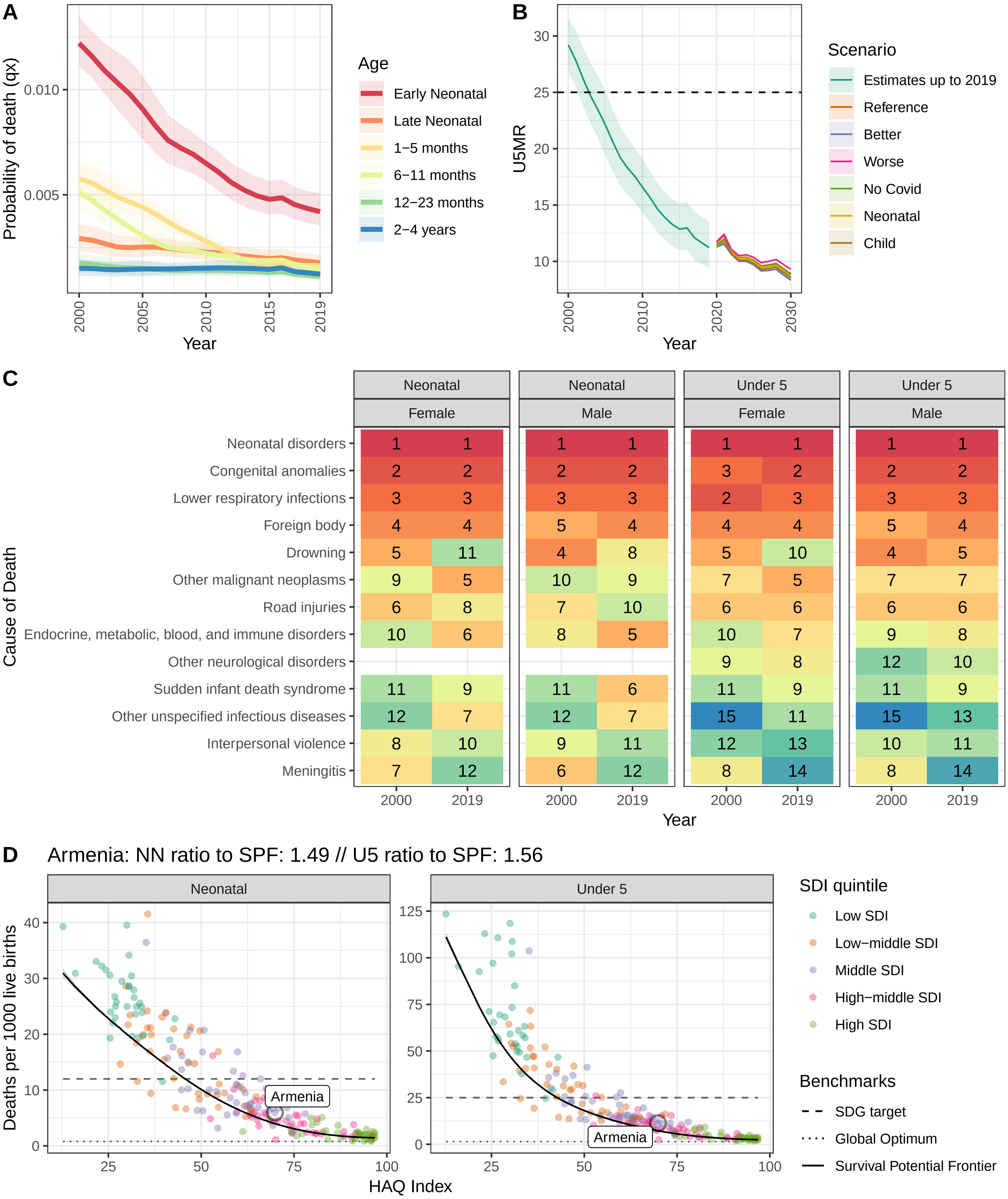
A
Probability of death (qx)
Year
Age
Early Neonatal
Late Neonatal
1−5 months
6−11 months
12−23 months
2−4 years
B
U5MR
Scenario
Estimates up to 2019
Reference
Better
Worse
No Covid
Neonatal
Child
C
Neonatal
Under 5
Female
Male
Cause of Death
Neonatal disorders
Congenital anomalies
Lower respiratory infections
Foreign body
Drowning
Other malignant neoplasms
Road injuries
Endocrine, metabolic, blood, and immune disorders
Other neurological disorders
Sudden infant death syndrome
Other unspecified infectious diseases
Interpersonal violence
Meningitis
D
Armenia: NN ratio to SPF: 1.49 // U5 ratio to SPF: 1.56
Deaths per 1000 live births
HAQ Index
Armenia
SDI quintile
Low SDI
Low−middle SDI
Middle SDI
High−middle SDI
High SDI
Benchmarks
SDG target
Global Optimum
Survival Potential Frontier

Azerbaijan

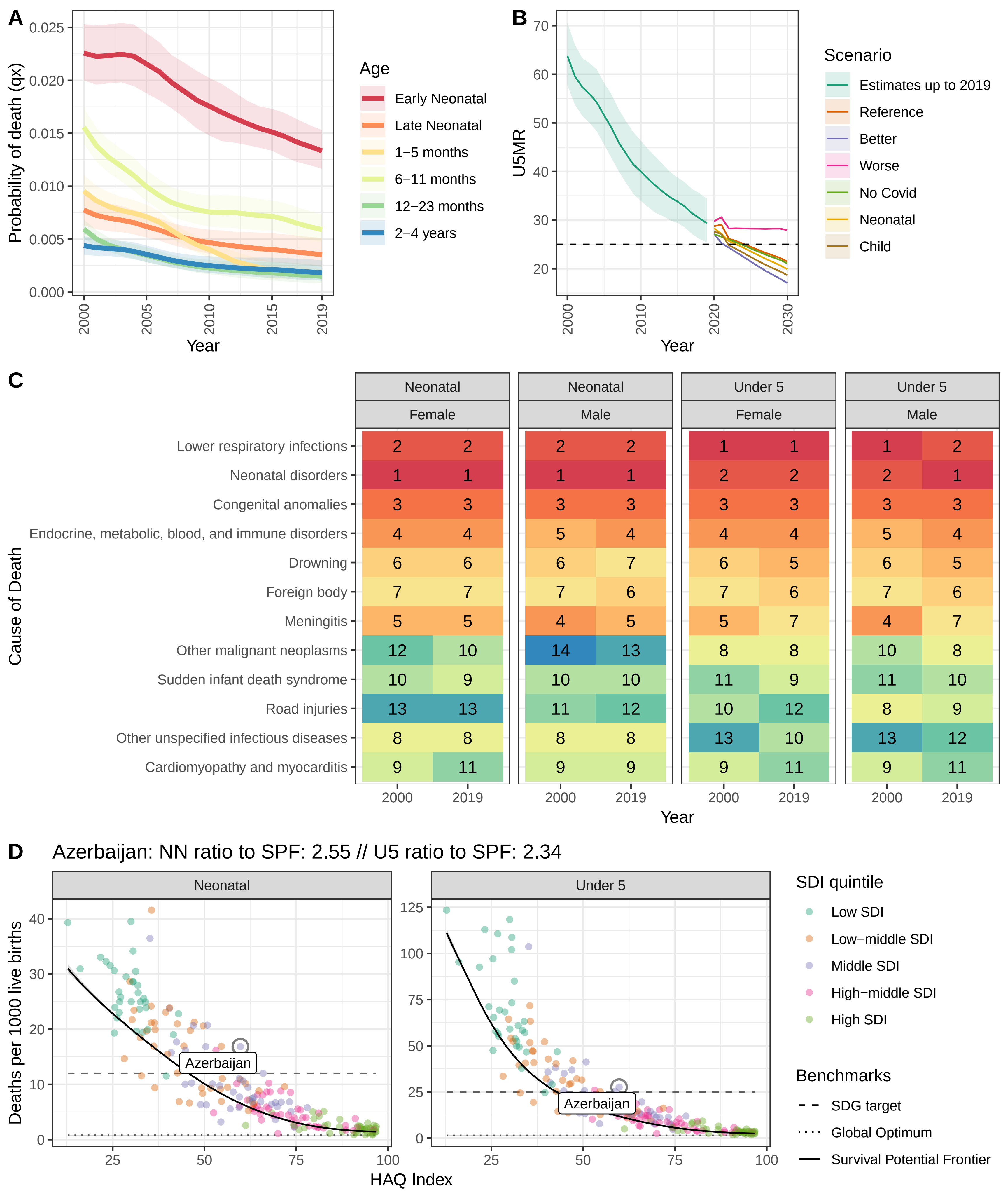
A
Probability of death (qx)
Year
Age
Early Neonatal
Late Neonatal
1−5 months
6−11 months
12−23 months
2−4 years
B
U5MR
Year
Scenario
Estimates up to 2019
Reference
Better
Worse
No Covid
Neonatal
Child
C
Neonatal
Female
Neonatal
Male
Under 5
Female
Under 5
Male
Cause of Death
Lower respiratory infections
Neonatal disorders
Congenital anomalies
Endocrine, metabolic, blood, and immune disorders
Drowning
Foreign body
Meningitis
Other malignant neoplasms
Sudden infant death syndrome
Road injuries
Other unspecified infectious diseases
Cardiomyopathy and myocarditis
Year
D
Azerbaijan: NN ratio to SPF: 2.55 // U5 ratio to SPF: 2.34
Neonatal
Under 5
Deaths per 1000 live births
HAQ Index
Azerbaijan
SDI quintile
Low SDI
Low−middle SDI
Middle SDI
High−middle SDI
High SDI
Benchmarks
SDG target
Global Optimum
Survival Potential Frontier

Greenland

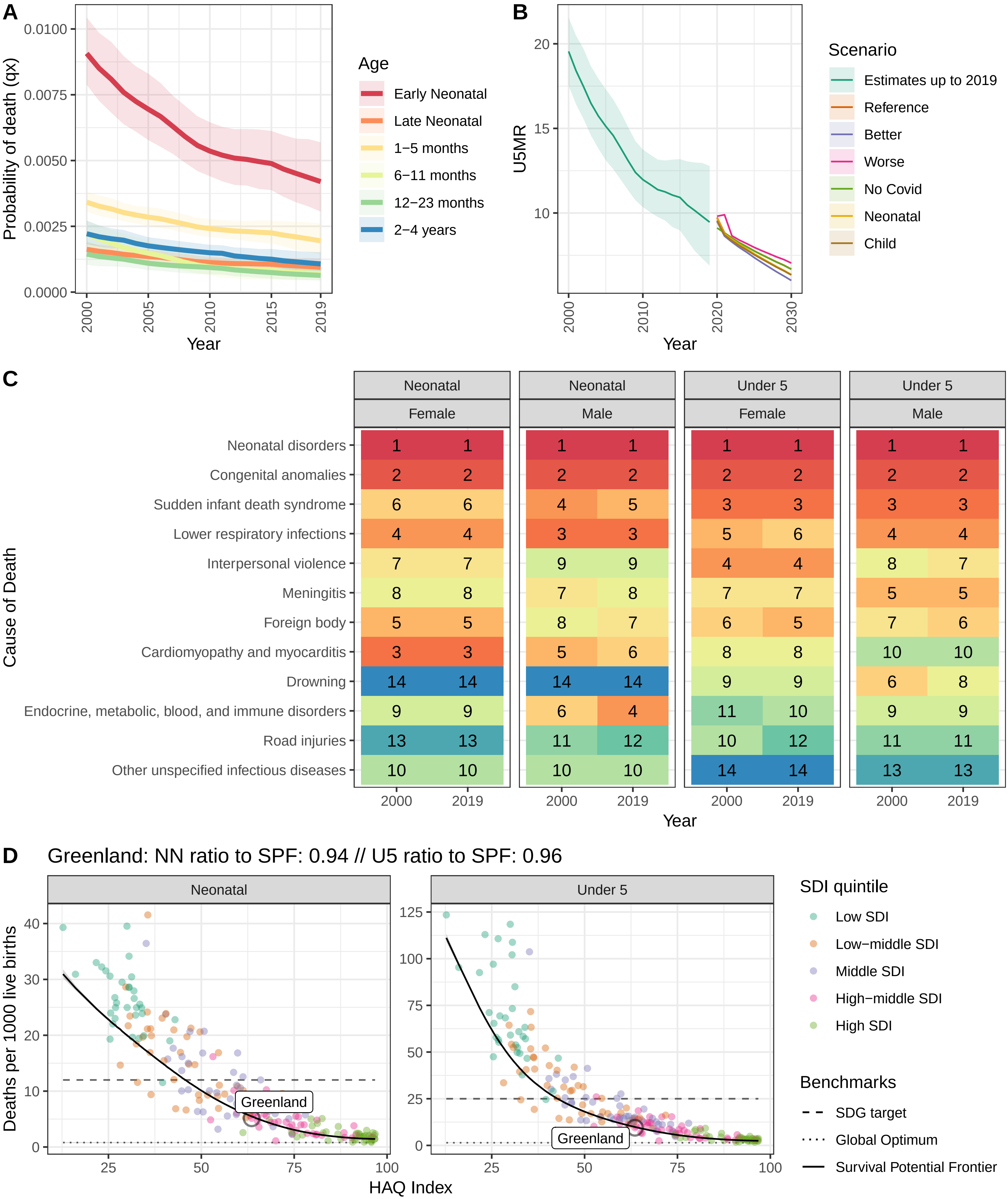
A
Probability of death (qx)
Year
Age
Early Neonatal
Late Neonatal
1−5 months
6−11 months
12−23 months
2−4 years
B
U5MR
Year
Scenario
Estimates up to 2019
Reference
Better
Worse
No Covid
Neonatal
Child
C
Cause of Death
Neonatal
Female
Male
Under 5
Neonatal disorders
Congenital anomalies
Sudden infant death syndrome
Lower respiratory infections
Interpersonal violence
Meningitis
Foreign body
Cardiomyopathy and myocarditis
Drowning
Endocrine, metabolic, blood, and immune disorders
Road injuries
Other unspecified infectious diseases
Year
D
Greenland: NN ratio to SPF: 0.94 // U5 ratio to SPF: 0.96
Neonatal
Under 5
Deaths per 1000 live births
HAQ Index
Greenland
SDI quintile
Low SDI
Low−middle SDI
Middle SDI
High−middle SDI
High SDI
Benchmarks
SDG target
Global Optimum
Survival Potential Frontier

Georgia

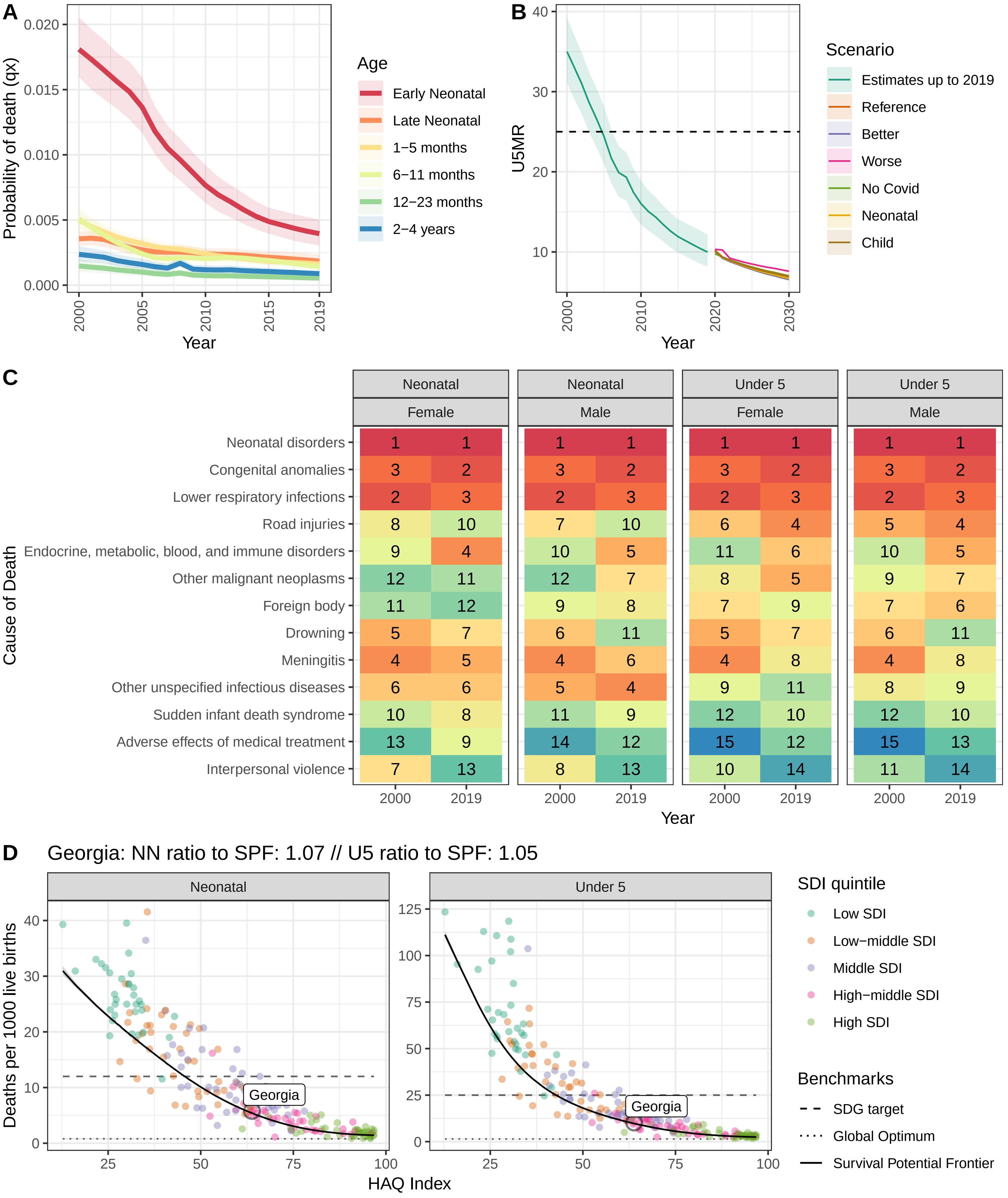

A
Probability of death (qx)
0.020
0.015
0.010
0.005
0.000
2000
2005
2010
2015
2019
Year
Age
Early Neonatal
Late Neonatal
1−5 months
6−11 months
12−23 months
2−4 years
B
U5MR
40
30
20
10
2000
2010
2020
2030
Year
Scenario
Estimates up to 2019
Reference
Better
Worse
No Covid
Neonatal
Child
C
Neonatal
Female
Neonatal
Male
Under 5
Female
Under 5
Male
Cause of Death
Neonatal disorders 1 1 1 1 1 1 1 1
Congenital anomalies 3 2 3 2 3 2 3 2
Lower respiratory infections 2 3 2 3 2 3 2 3
Road injuries 8 10 7 10 6 4 5 4
Endocrine, metabolic, blood, and immune disorders 9 4 10 5 11 6 10 5
Other malignant neoplasms 12 11 12 7 8 5 9 7
Foreign body 11 12 9 8 7 9 7 6
Drowning 5 7 6 11 5 7 6 11
Meningitis 4 5 4 6 4 8 4 8
Other unspecified infectious diseases 6 6 5 4 9 11 8 9
Sudden infant death syndrome 10 8 11 9 12 10 12 10
Adverse effects of medical treatment 13 9 14 12 15 12 15 13
Interpersonal violence 7 13 8 13 10 14 11 14
2000
2019
Year
D
Georgia: NN ratio to SPF: 1.07 // U5 ratio to SPF: 1.05
Neonatal
Under 5
Deaths per 1000 live births
40
30
20
10
0
125
100
75
50
25
0
Georgia
HAQ Index
SDI quintile
Low SDI
Low−middle SDI
Middle SDI
High−middle SDI
High SDI
Benchmarks
SDG target
Global Optimum
Survival Potential Frontier

Guam

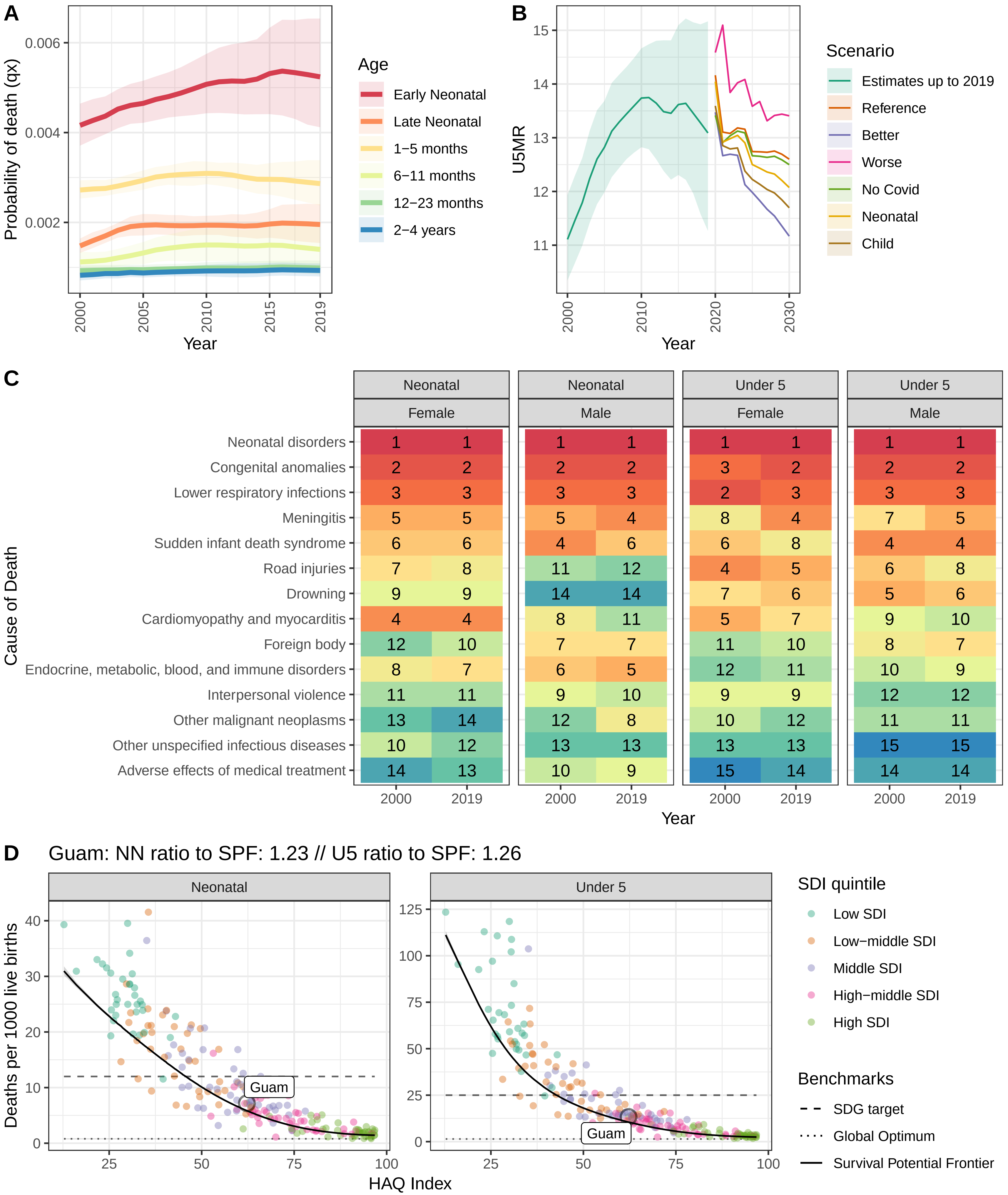
A
Probability of death (qx)
0.006
0.004
0.002
2000
2005
2010
2015
2019
Year
Age
Early Neonatal
Late Neonatal
1−5 months
6−11 months
12−23 months
2−4 years
B
U5MR
15
14
13
12
11
2000
2010
2020
2030
Year
Scenario
Estimates up to 2019
Reference
Better
Worse
No Covid
Neonatal
Child
C
Neonatal
Female
Neonatal
Male
Under 5
Female
Under 5
Male
Cause of Death
Neonatal disorders 1 1 1 1 1 1 1 1
Congenital anomalies 2 2 2 2 3 2 2 2
Lower respiratory infections 3 3 3 3 2 3 3 3
Meningitis 5 5 5 4 8 4 7 5
Sudden infant death syndrome 6 6 4 6 6 8 4 4
Road injuries 7 8 11 12 4 5 6 8
Drowning 9 9 14 14 7 6 5 6
Cardiomyopathy and myocarditis 4 4 8 11 5 7 9 10
Foreign body 12 10 7 7 11 10 8 7
Endocrine, metabolic, blood, and immune disorders 8 7 6 5 12 11 10 9
Interpersonal violence 11 11 9 10 9 9 12 12
Other malignant neoplasms 13 14 12 8 10 12 11 11
Other unspecified infectious diseases 10 12 13 13 13 13 15 15
Adverse effects of medical treatment 14 13 10 9 15 14 14 14
2000
2019
Year
D
Guam: NN ratio to SPF: 1.23 // U5 ratio to SPF: 1.26
Neonatal
Under 5
Deaths per 1000 live births
40
30
20
10
0
125
100
75
50
25
0
Guam
25
50
75
100
HAQ Index
SDI quintile
Low SDI
Low−middle SDI
Middle SDI
High−middle SDI
High SDI
Benchmarks
SDG target
Global Optimum
Survival Potential Frontier

Kazakhstan

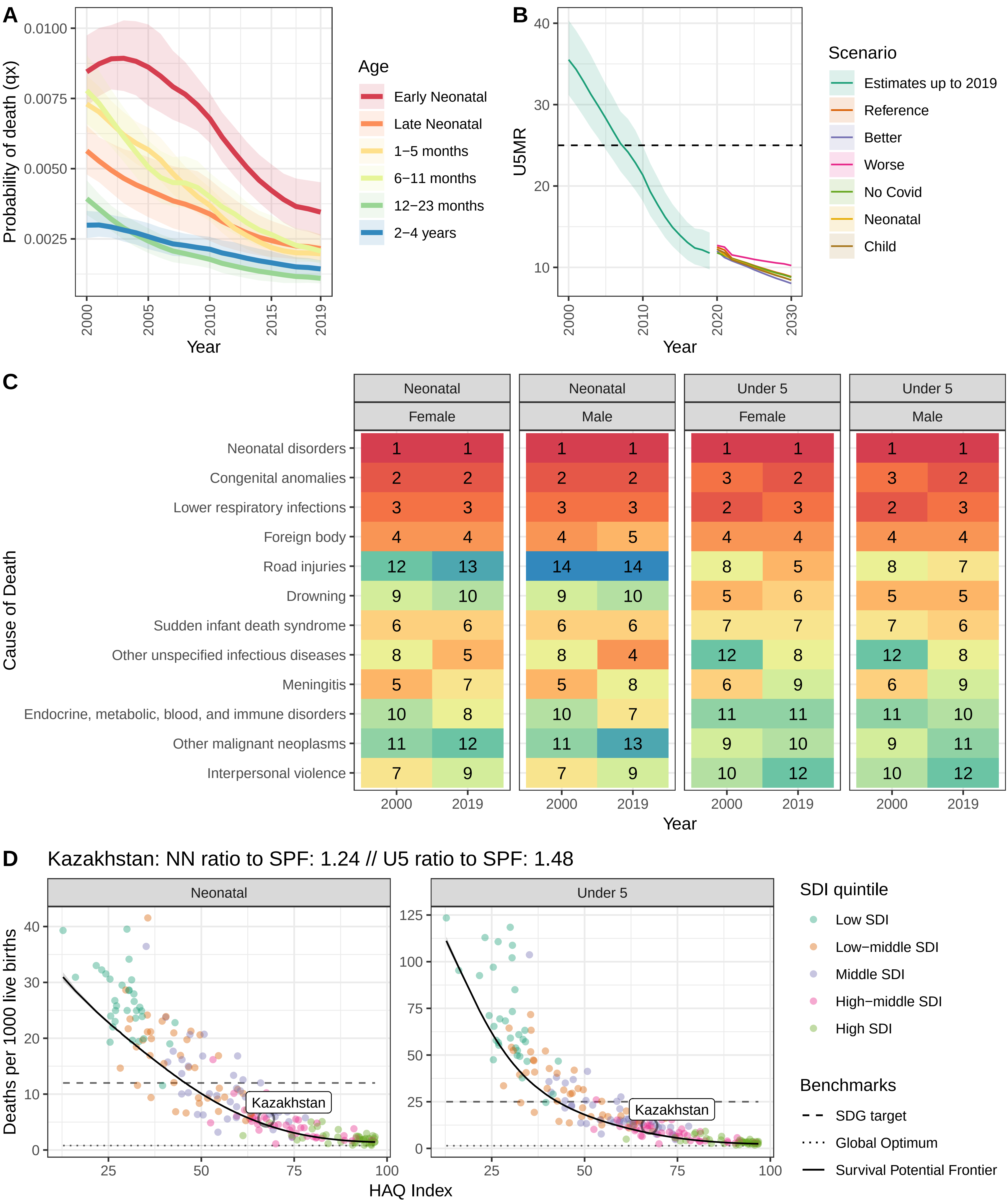
A
Probability of death (qx)
0.0100
0.0075
0.0050
0.0025
Year
2000
2005
2010
2015
2019
Age
Early Neonatal
Late Neonatal
1−5 months
6−11 months
12−23 months
2−4 years
B
U5MR
40
30
20
10
Year
2000
2010
2020
2030
Scenario
Estimates up to 2019
Reference
Better
Worse
No Covid
Neonatal
Child
C
Neonatal Female
Neonatal Male
Under 5 Female
Under 5 Male
Cause of Death
Neonatal disorders 1 1 1 1 1 1 1 1
Congenital anomalies 2 2 2 2 3 2 3 2
Lower respiratory infections 3 3 3 3 2 3 2 3
Foreign body 4 4 4 5 4 4 4 4
Road injuries 12 13 14 14 8 5 8 7
Drowning 9 10 9 10 5 6 5 5
Sudden infant death syndrome 6 6 6 6 7 7 7 6
Other unspecified infectious diseases 8 5 8 4 12 8 12 8
Meningitis 5 7 5 8 6 9 6 9
Endocrine, metabolic, blood, and immune disorders 10 8 10 7 11 11 11 10
Other malignant neoplasms 11 12 11 13 9 10 9 11
Interpersonal violence 7 9 7 9 10 12 10 12
Year
2000
2019
D
Kazakhstan: NN ratio to SPF: 1.24 // U5 ratio to SPF: 1.48
Neonatal
Under 5
Deaths per 1000 live births
Kazakhstan
HAQ Index
SDI quintile
Low SDI
Low−middle SDI
Middle SDI
High−middle SDI
High SDI
Benchmarks
SDG target
Global Optimum
Survival Potential Frontier

Monaco

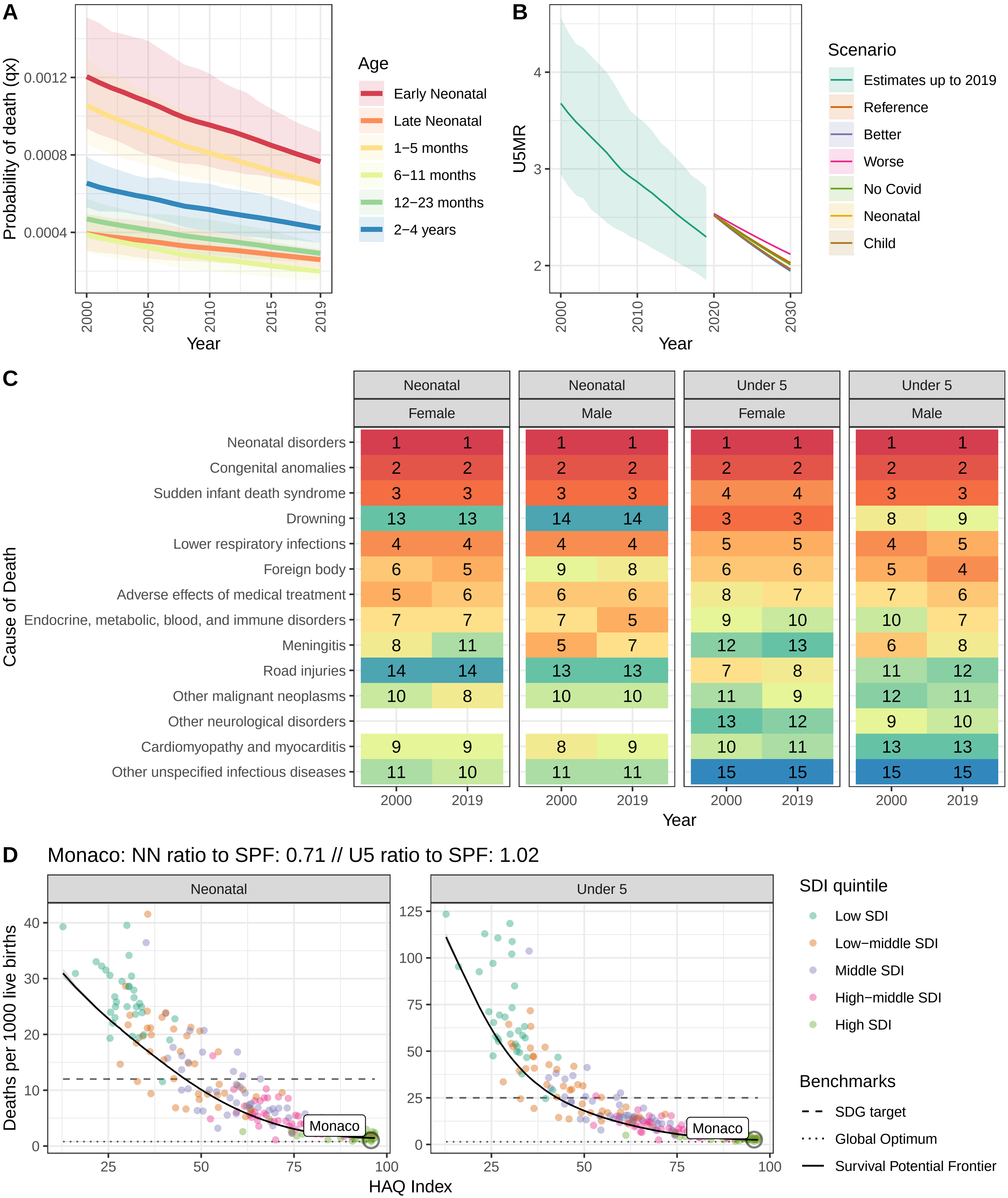
A
Probability of death (qx)
0.0012
0.0008
0.0004
Year
2000
2005
2010
2015
2019
Age
Early Neonatal
Late Neonatal
1−5 months
6−11 months
12−23 months
2−4 years
B
U5MR
4
3
2
Year
2000
2010
2020
2030
Scenario
Estimates up to 2019
Reference
Better
Worse
No Covid
Neonatal
Child
C
Neonatal Female
Neonatal Male
Under 5 Female
Under 5 Male
Cause of Death
Neonatal disorders 1 1 1 1 1 1 1 1
Congenital anomalies 2 2 2 2 2 2 2 2
Sudden infant death syndrome 3 3 3 3 4 4 3 3
Drowning 13 13 14 14 3 3 8 9
Lower respiratory infections 4 4 4 4 5 5 4 5
Foreign body 6 5 9 8 6 6 5 4
Adverse effects of medical treatment 5 6 6 6 8 7 7 6
Endocrine, metabolic, blood, and immune disorders 7 7 7 5 9 10 10 7
Meningitis 8 11 5 7 12 13 6 8
Road injuries 14 14 13 13 7 8 11 12
Other malignant neoplasms 10 8 10 10 11 9 12 11
Other neurological disorders 13 12 9 10
Cardiomyopathy and myocarditis 9 9 8 9 10 11 13 13
Other unspecified infectious diseases 11 10 11 11 15 15 15 15
2000 2019
Year
D
Monaco: NN ratio to SPF: 0.71 // U5 ratio to SPF: 1.02
Neonatal
Under 5
Deaths per 1000 live births
40
30
20
10
0
125
100
75
50
25
0
Monaco
HAQ Index
25
50
75
100
SDI quintile
Low SDI
Low−middle SDI
Middle SDI
High−middle SDI
High SDI
Benchmarks
SDG target
Global Optimum
Survival Potential Frontier

Nauru

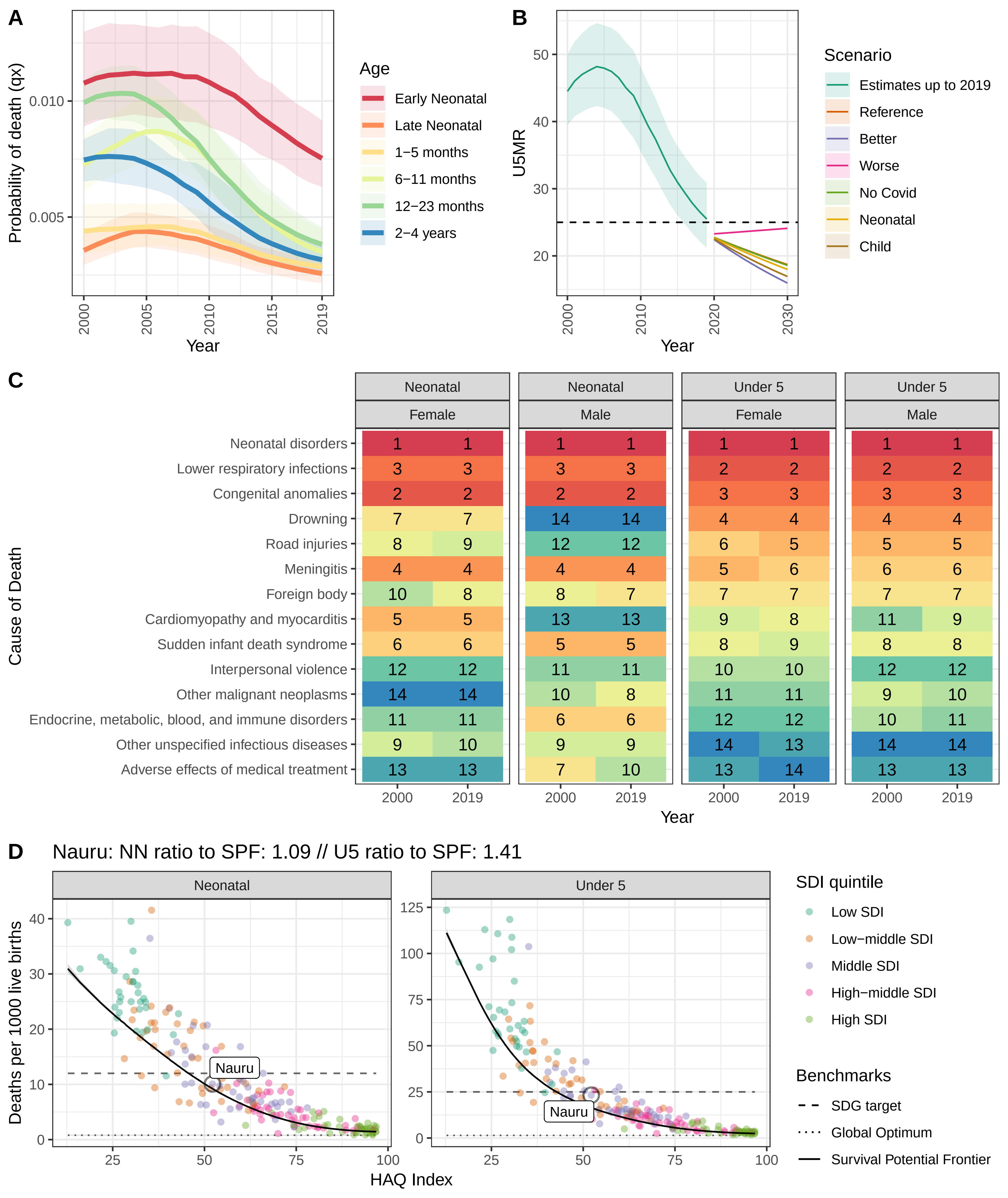
A
Probability of death (qx)
Year
Age
Early Neonatal
Late Neonatal
1−5 months
6−11 months
12−23 months
2−4 years
B
U5MR
Year
Scenario
Estimates up to 2019
Reference
Better
Worse
No Covid
Neonatal
Child
C
Neonatal Female
Neonatal Male
Under 5 Female
Under 5 Male
Cause of Death
Neonatal disorders
Lower respiratory infections
Congenital anomalies
Drowning
Road injuries
Meningitis
Foreign body
Cardiomyopathy and myocarditis
Sudden infant death syndrome
Interpersonal violence
Other malignant neoplasms
Endocrine, metabolic, blood, and immune disorders
Other unspecified infectious diseases
Adverse effects of medical treatment
Year
D
Nauru: NN ratio to SPF: 1.09 // U5 ratio to SPF: 1.41
Neonatal
Under 5
Deaths per 1000 live births
HAQ Index
Nauru
SDI quintile
Low SDI
Low−middle SDI
Middle SDI
High−middle SDI
High SDI
Benchmarks
SDG target
Global Optimum
Survival Potential Frontier

Kyrgyzstan

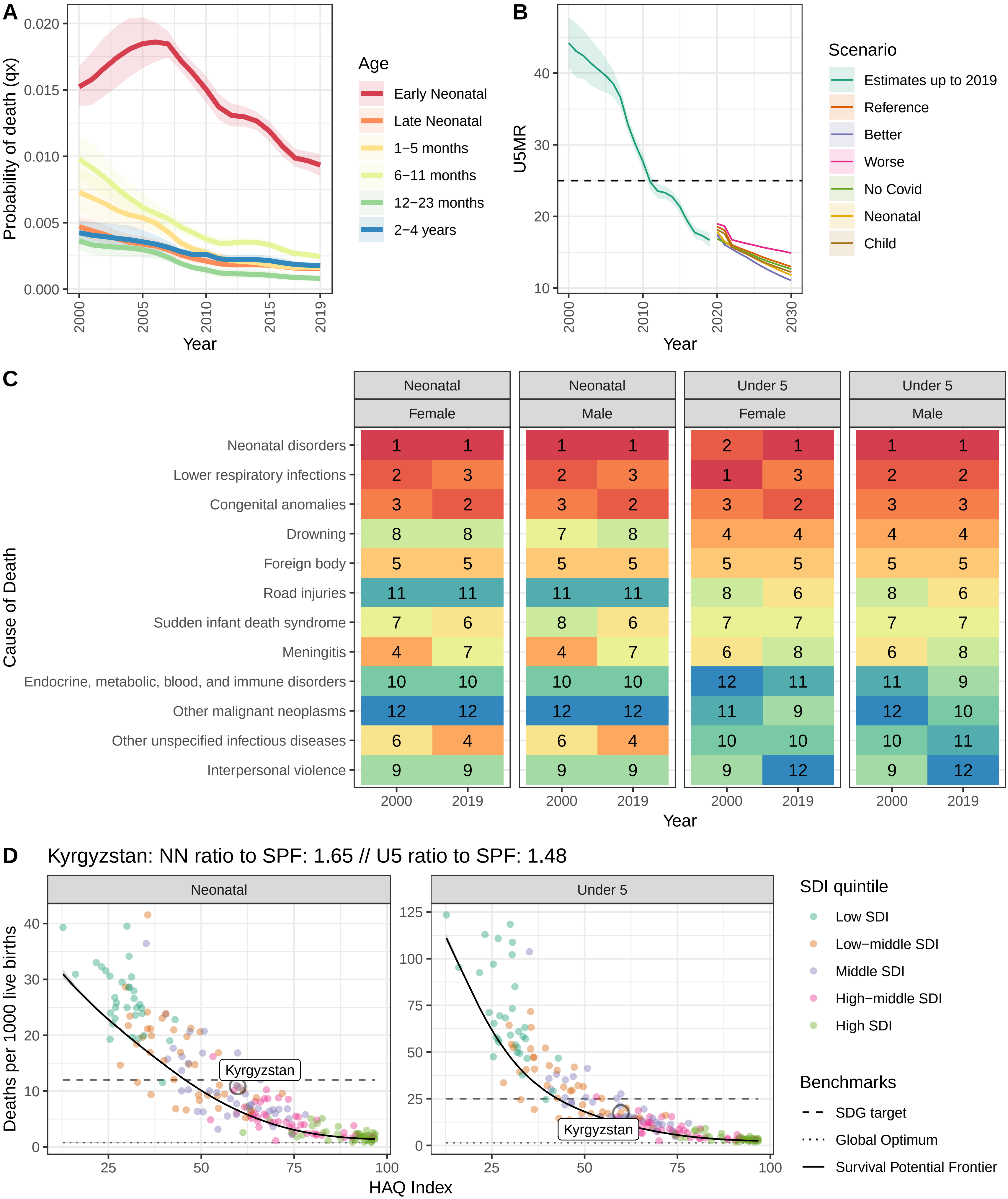
A
Probability of death (qx)
0.020
0.015
0.010
0.005
0.000
2000
2005
2010
2015
2019
Year
Age
Early Neonatal
Late Neonatal
1−5 months
6−11 months
12−23 months
2−4 years
B
U5MR
40
30
20
10
2000
2010
2020
2030
Year
Scenario
Estimates up to 2019
Reference
Better
Worse
No Covid
Neonatal
Child
C
Neonatal
Female
Neonatal
Male
Under 5
Female
Under 5
Male
Cause of Death
Neonatal disorders 1 1 1 1 2 1 1 1
Lower respiratory infections 2 3 2 3 1 3 2 2
Congenital anomalies 3 2 3 2 3 2 3 3
Drowning 8 8 7 8 4 4 4 4
Foreign body 5 5 5 5 5 5 5 5
Road injuries 11 11 11 11 8 6 8 6
Sudden infant death syndrome 7 6 8 6 7 7 7 7
Meningitis 4 7 4 7 6 8 6 8
Endocrine, metabolic, blood, and immune disorders 10 10 10 10 12 11 11 9
Other malignant neoplasms 12 12 12 12 11 9 12 10
Other unspecified infectious diseases 6 4 6 4 10 10 10 11
Interpersonal violence 9 9 9 9 9 12 9 12
2000
2019
Year
D
Kyrgyzstan: NN ratio to SPF: 1.65 // U5 ratio to SPF: 1.48
Neonatal
Under 5
Deaths per 1000 live births
40
30
20
10
0
125
100
75
50
25
0
Kyrgyzstan
25
50
75
100
HAQ Index
SDI quintile
Low SDI
Low−middle SDI
Middle SDI
High−middle SDI
High SDI
Benchmarks
SDG target
Global Optimum
Survival Potential Frontier

Niue

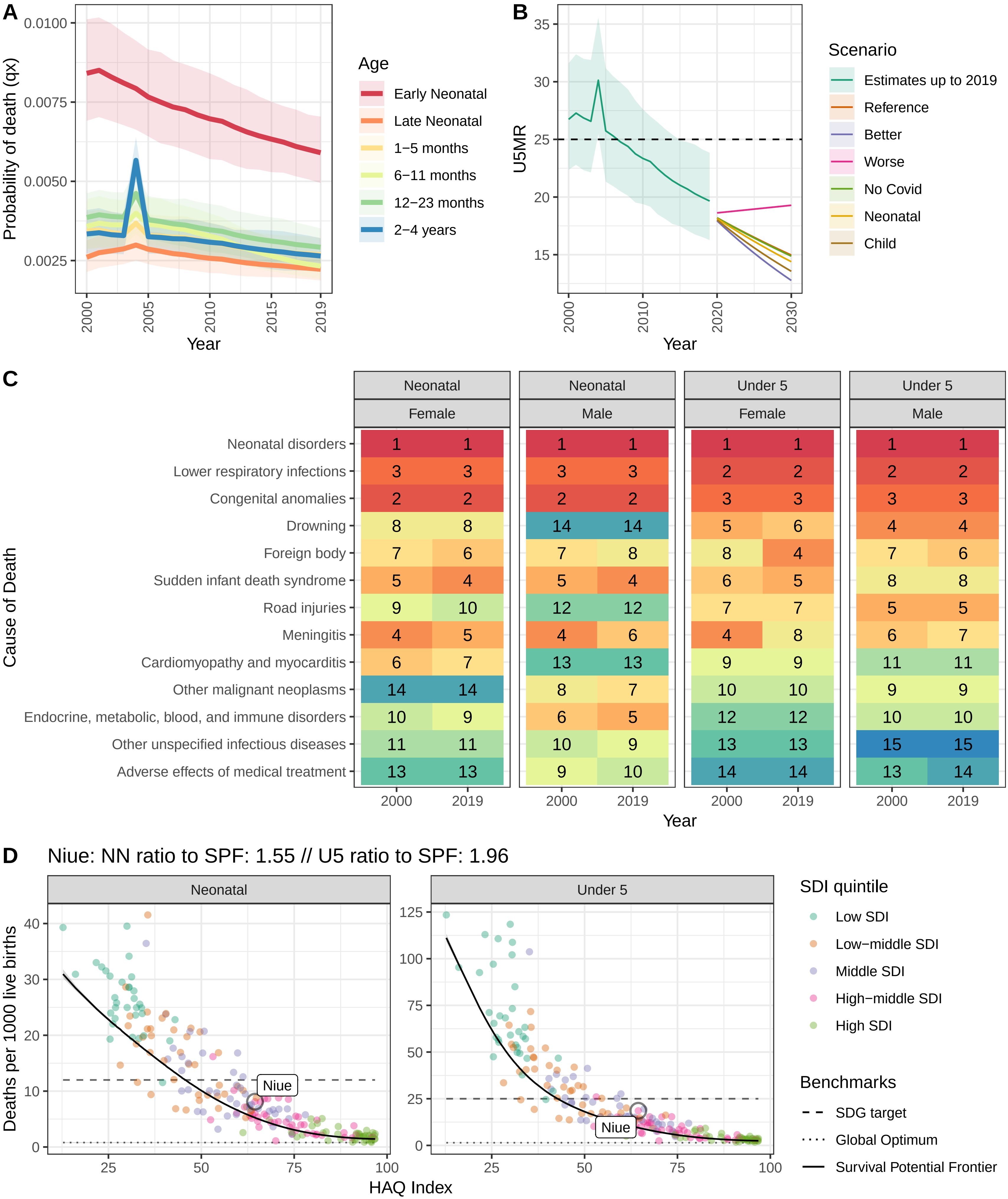
A
Probability of death (qx)
0.0100
0.0075
0.0050
0.0025
2000
2005
2010
2015
2019
Year
Age
Early Neonatal
Late Neonatal
1−5 months
6−11 months
12−23 months
2−4 years
B
U5MR
35
30
25
20
15
2000
2010
2020
2030
Year
Scenario
Estimates up to 2019
Reference
Better
Worse
No Covid
Neonatal
Child
C
Cause of Death
Neonatal Female | Neonatal Male | Under 5 Female | Under 5 Male
Neonatal disorders 1 1 | 1 1 | 1 1 | 1 1
Lower respiratory infections 3 3 | 3 3 | 2 2 | 2 2
Congenital anomalies 2 2 | 2 2 | 3 3 | 3 3
Drowning 8 8 | 14 14 | 5 6 | 4 4
Foreign body 7 6 | 7 8 | 8 4 | 7 6
Sudden infant death syndrome 5 4 | 5 4 | 6 5 | 8 8
Road injuries 9 10 | 12 12 | 7 7 | 5 5
Meningitis 4 5 | 4 6 | 4 8 | 6 7
Cardiomyopathy and myocarditis 6 7 | 13 13 | 9 9 | 11 11
Other malignant neoplasms 14 14 | 8 7 | 10 10 | 9 9
Endocrine, metabolic, blood, and immune disorders 10 9 | 6 5 | 12 12 | 10 10
Other unspecified infectious diseases 11 11 | 10 9 | 13 13 | 15 15
Adverse effects of medical treatment 13 13 | 9 10 | 14 14 | 13 14
2000 2019
Year
D
Niue: NN ratio to SPF: 1.55 // U5 ratio to SPF: 1.96
Neonatal
Under 5
Deaths per 1000 live births
HAQ Index
Niue
SDI quintile
Low SDI
Low−middle SDI
Middle SDI
High−middle SDI
High SDI
Benchmarks
SDG target
Global Optimum
Survival Potential Frontier

Northern Mariana Islands

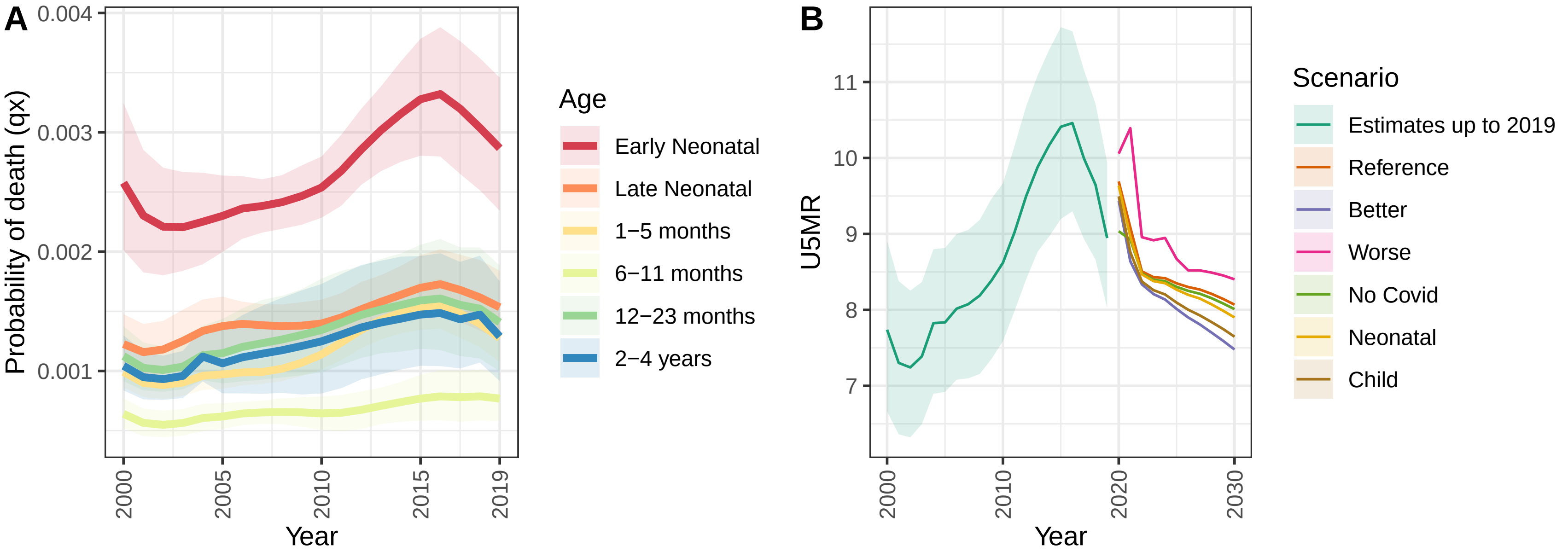

C

Cause of Death (ranks by Year)

Cause of Death	Neonatal Female 2000	Neonatal Female 2019	Neonatal Male 2000	Neonatal Male 2019	Under 5 Female 2000	Under 5 Female 2019	Under 5 Male 2000	Under 5 Male 2019
Neonatal disorders	1	1	1	1	1	1	1	1
Congenital anomalies	2	2	2	2	2	2	2	2
Lower respiratory infections	3	3	3	3	3	3	3	3
Drowning	10	10	14	14	5	4	4	4
Road injuries	6	11	11	13	4	6	5	5
Foreign body	11	4	7	7	8	5	7	6
Meningitis	5	5	5	5	9	7	9	8
Sudden infant death syndrome	4	6	4	4	7	11	6	7
Interpersonal violence	13	13	9	10	6	8	8	10
Cardiomyopathy and myocarditis	12	7	12	11	10	9	12	12
Other malignant neoplasms	14	14	8	8	13	14	10	9
Endocrine, metabolic, blood, and immune disorders	7	9	6	6	11	10	11	11
Other unspecified infectious diseases	8	8	10	9	14	12	14	13
Adverse effects of medical treatment	9	12	13	12	12	13	15	15

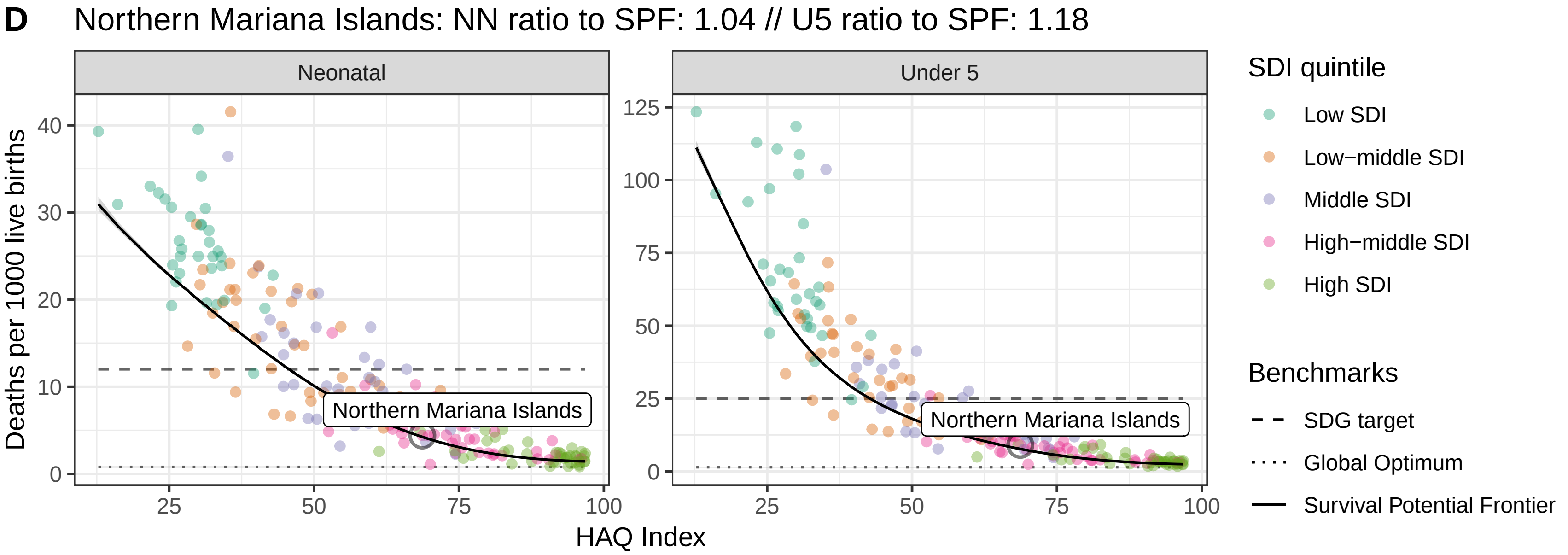

Mongolia

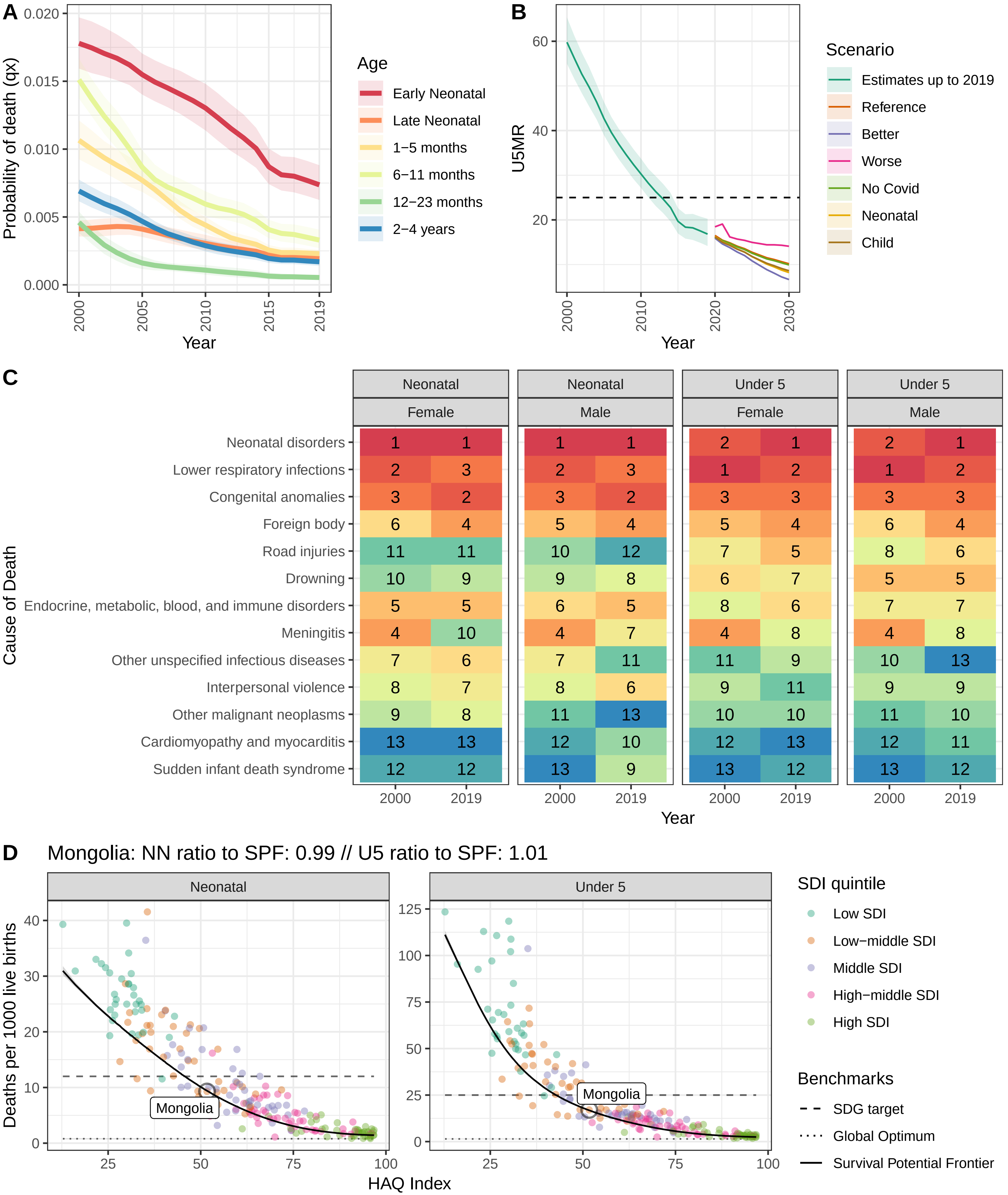

A
Probability of death (qx)
0.020
0.015
0.010
0.005
0.000
2000
2005
2010
2015
2019
Year
Age
Early Neonatal
Late Neonatal
1−5 months
6−11 months
12−23 months
2−4 years
B
U5MR
60
40
20
2000
2010
2020
2030
Year
Scenario
Estimates up to 2019
Reference
Better
Worse
No Covid
Neonatal
Child
C
Neonatal Female
Neonatal Male
Under 5 Female
Under 5 Male
Cause of Death
Neonatal disorders 1 1 1 1 2 1 2 1
Lower respiratory infections 2 3 2 3 1 2 1 2
Congenital anomalies 3 2 3 2 3 3 3 3
Foreign body 6 4 5 4 5 4 6 4
Road injuries 11 11 10 12 7 5 8 6
Drowning 10 9 9 8 6 7 5 5
Endocrine, metabolic, blood, and immune disorders 5 5 6 5 8 6 7 7
Meningitis 4 10 4 7 4 8 4 8
Other unspecified infectious diseases 7 6 7 11 11 9 10 13
Interpersonal violence 8 7 8 6 9 11 9 9
Other malignant neoplasms 9 8 11 13 10 10 11 10
Cardiomyopathy and myocarditis 13 13 12 10 12 13 12 11
Sudden infant death syndrome 12 12 13 9 13 12 13 12
2000
2019
Year
D
Mongolia: NN ratio to SPF: 0.99 // U5 ratio to SPF: 1.01
Neonatal
Under 5
Deaths per 1000 live births
40
30
20
10
0
125
100
75
50
25
0
25
50
75
100
HAQ Index
Mongolia
SDI quintile
Low SDI
Low−middle SDI
Middle SDI
High−middle SDI
High SDI
Benchmarks
SDG target
Global Optimum
Survival Potential Frontier

Palau

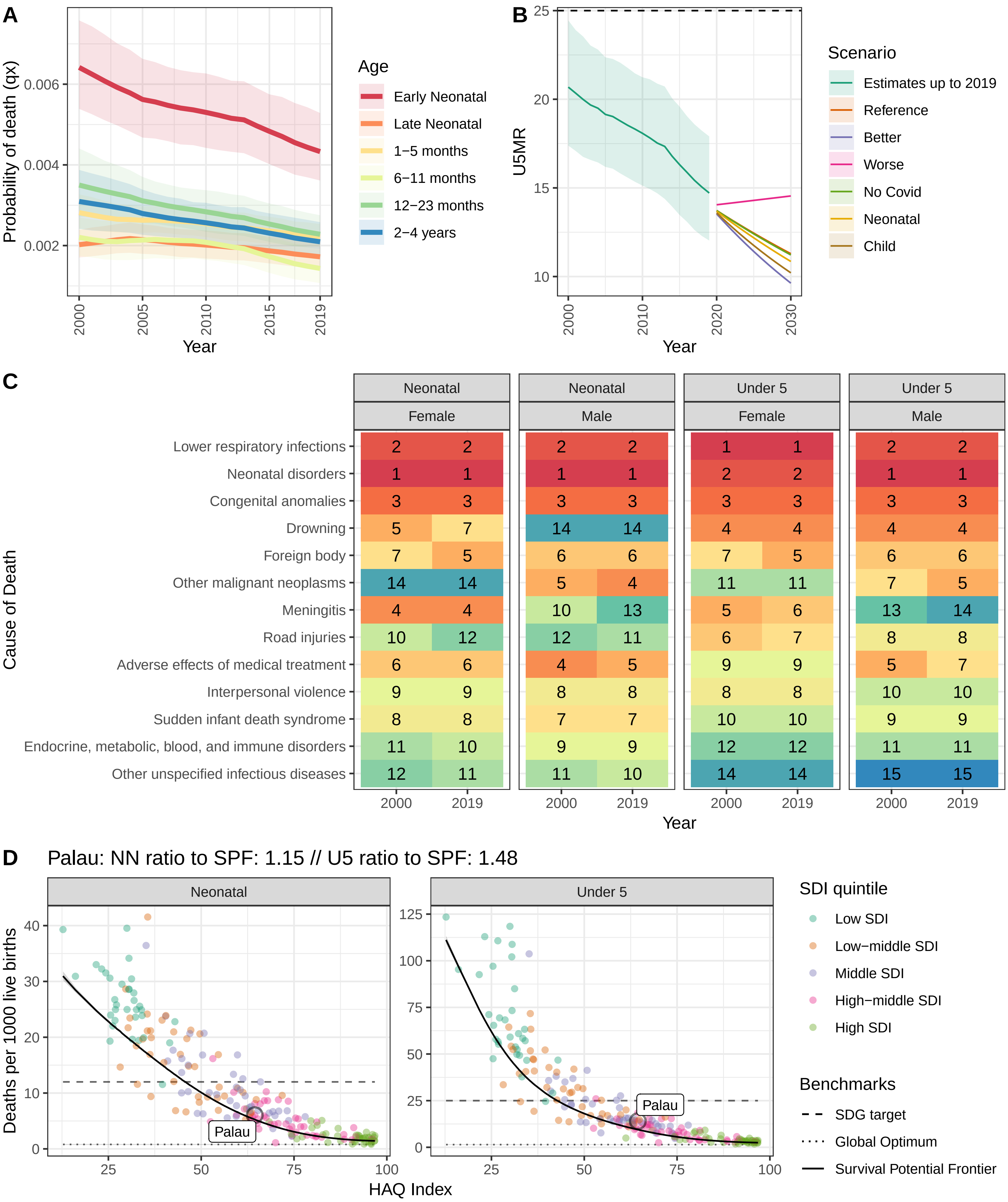
A
Probability of death (qx)
0.006
0.004
0.002
2000
2005
2010
2015
2019
Year
Age
Early Neonatal
Late Neonatal
1−5 months
6−11 months
12−23 months
2−4 years
B
U5MR
25
20
15
10
2000
2010
2020
2030
Year
Scenario
Estimates up to 2019
Reference
Better
Worse
No Covid
Neonatal
Child
C
Cause of Death
Neonatal Female
Neonatal Male
Under 5 Female
Under 5 Male
Lower respiratory infections 2 2 2 2 1 1 2 2
Neonatal disorders 1 1 1 1 2 2 1 1
Congenital anomalies 3 3 3 3 3 3 3 3
Drowning 5 7 14 14 4 4 4 4
Foreign body 7 5 6 6 7 5 6 6
Other malignant neoplasms 14 14 5 4 11 11 7 5
Meningitis 4 4 10 13 5 6 13 14
Road injuries 10 12 12 11 6 7 8 8
Adverse effects of medical treatment 6 6 4 5 9 9 5 7
Interpersonal violence 9 9 8 8 8 8 10 10
Sudden infant death syndrome 8 8 7 7 10 10 9 9
Endocrine, metabolic, blood, and immune disorders 11 10 9 9 12 12 11 11
Other unspecified infectious diseases 12 11 11 10 14 14 15 15
2000 2019 2000 2019 2000 2019 2000 2019
Year
D
Palau: NN ratio to SPF: 1.15 // U5 ratio to SPF: 1.48
Neonatal
Under 5
Deaths per 1000 live births
40
30
20
10
0
125
100
75
50
25
0
Palau
Palau
25
50
75
100
HAQ Index
SDI quintile
Low SDI
Low−middle SDI
Middle SDI
High−middle SDI
High SDI
Benchmarks
SDG target
Global Optimum
Survival Potential Frontier

Puerto Rico

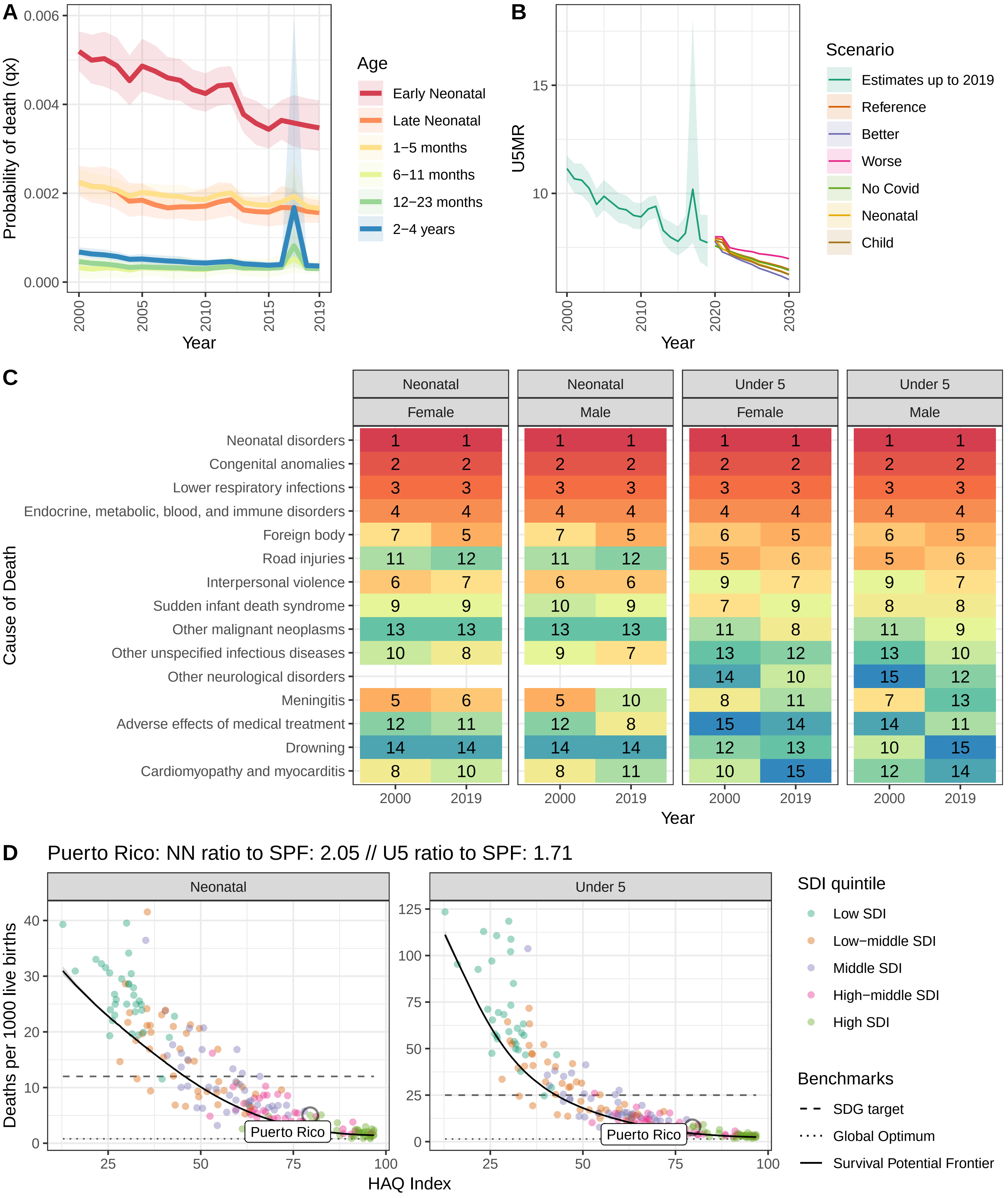

A
Probability of death (qx)
Year
Age
Early Neonatal
Late Neonatal
1−5 months
6−11 months
12−23 months
2−4 years
B
U5MR
Year
Scenario
Estimates up to 2019
Reference
Better
Worse
No Covid
Neonatal
Child
C
Neonatal Female
Neonatal Male
Under 5 Female
Under 5 Male
Cause of Death
Neonatal disorders 1 1 1 1 1 1 1 1
Congenital anomalies 2 2 2 2 2 2 2 2
Lower respiratory infections 3 3 3 3 3 3 3 3
Endocrine, metabolic, blood, and immune disorders 4 4 4 4 4 4 4 4
Foreign body 7 5 7 5 6 5 6 5
Road injuries 11 12 11 12 5 6 5 6
Interpersonal violence 6 7 6 6 9 7 9 7
Sudden infant death syndrome 9 9 10 9 7 9 8 8
Other malignant neoplasms 13 13 13 13 11 8 11 9
Other unspecified infectious diseases 10 8 9 7 13 12 13 10
Other neurological disorders 14 10 15 12
Meningitis 5 6 5 10 8 11 7 13
Adverse effects of medical treatment 12 11 12 8 15 14 14 11
Drowning 14 14 14 14 12 13 10 15
Cardiomyopathy and myocarditis 8 10 8 11 10 15 12 14
Year
D
Puerto Rico: NN ratio to SPF: 2.05 // U5 ratio to SPF: 1.71
Neonatal
Under 5
Deaths per 1000 live births
HAQ Index
Puerto Rico
Puerto Rico
SDI quintile
Low SDI
Low−middle SDI
Middle SDI
High−middle SDI
High SDI
Benchmarks
SDG target
Global Optimum
Survival Potential Frontier

Tajikistan

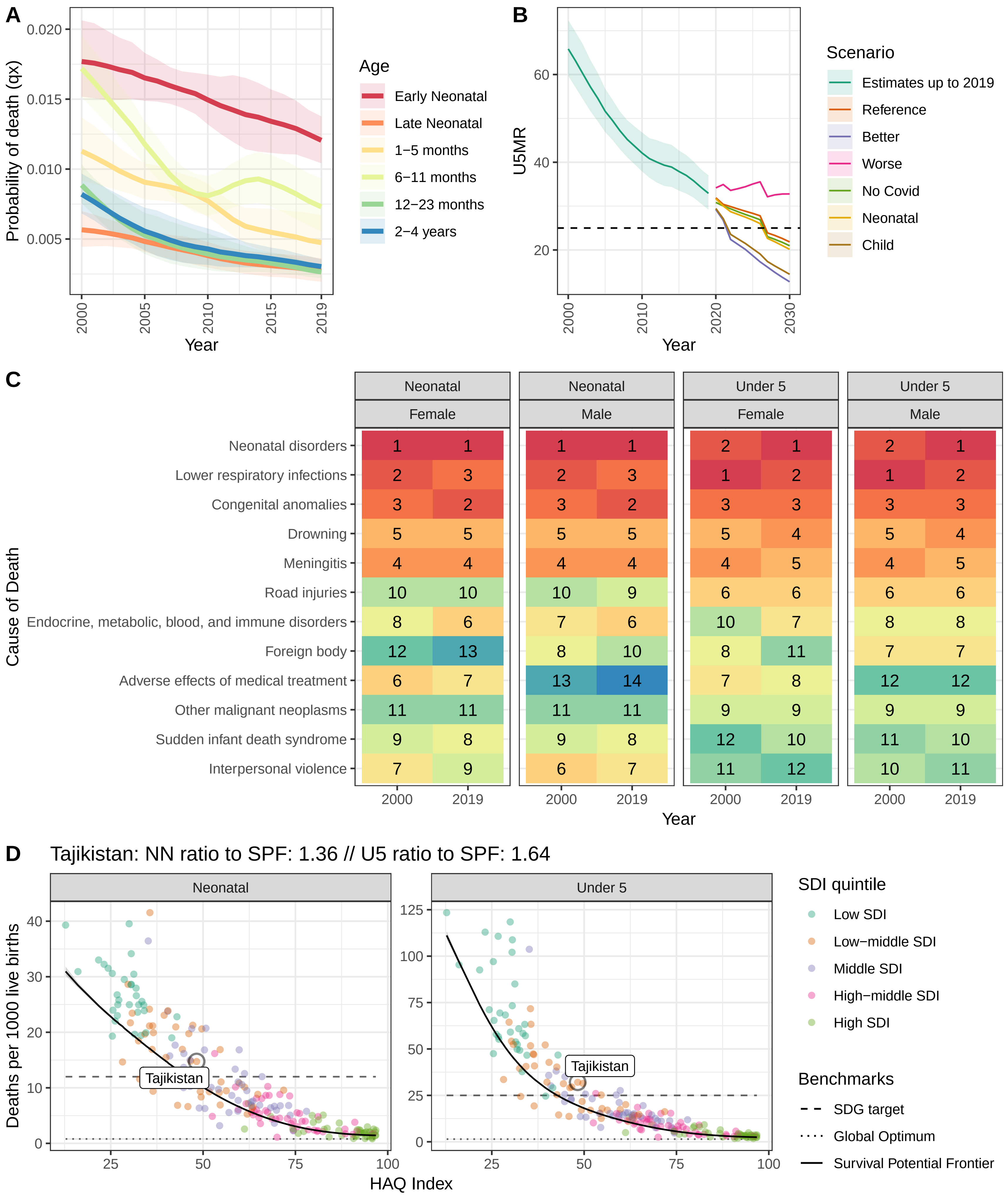
A
Probability of death (qx)
Year
Age
Early Neonatal
Late Neonatal
1−5 months
6−11 months
12−23 months
2−4 years
B
U5MR
Year
Scenario
Estimates up to 2019
Reference
Better
Worse
No Covid
Neonatal
Child
C
Cause of Death
Neonatal Female
Neonatal Male
Under 5 Female
Under 5 Male
Neonatal disorders 1 1 1 1 2 1 2 1
Lower respiratory infections 2 3 2 3 1 2 1 2
Congenital anomalies 3 2 3 2 3 3 3 3
Drowning 5 5 5 5 5 4 5 4
Meningitis 4 4 4 4 4 5 4 5
Road injuries 10 10 10 9 6 6 6 6
Endocrine, metabolic, blood, and immune disorders 8 6 7 6 10 7 8 8
Foreign body 12 13 8 10 8 11 7 7
Adverse effects of medical treatment 6 7 13 14 7 8 12 12
Other malignant neoplasms 11 11 11 11 9 9 9 9
Sudden infant death syndrome 9 8 9 8 12 10 11 10
Interpersonal violence 7 9 6 7 11 12 10 11
2000 2019
Year
D
Tajikistan: NN ratio to SPF: 1.36 // U5 ratio to SPF: 1.64
Neonatal
Under 5
Deaths per 1000 live births
HAQ Index
Tajikistan
SDI quintile
Low SDI
Low−middle SDI
Middle SDI
High−middle SDI
High SDI
Benchmarks
SDG target
Global Optimum
Survival Potential Frontier

Saint Kitts and Nevis

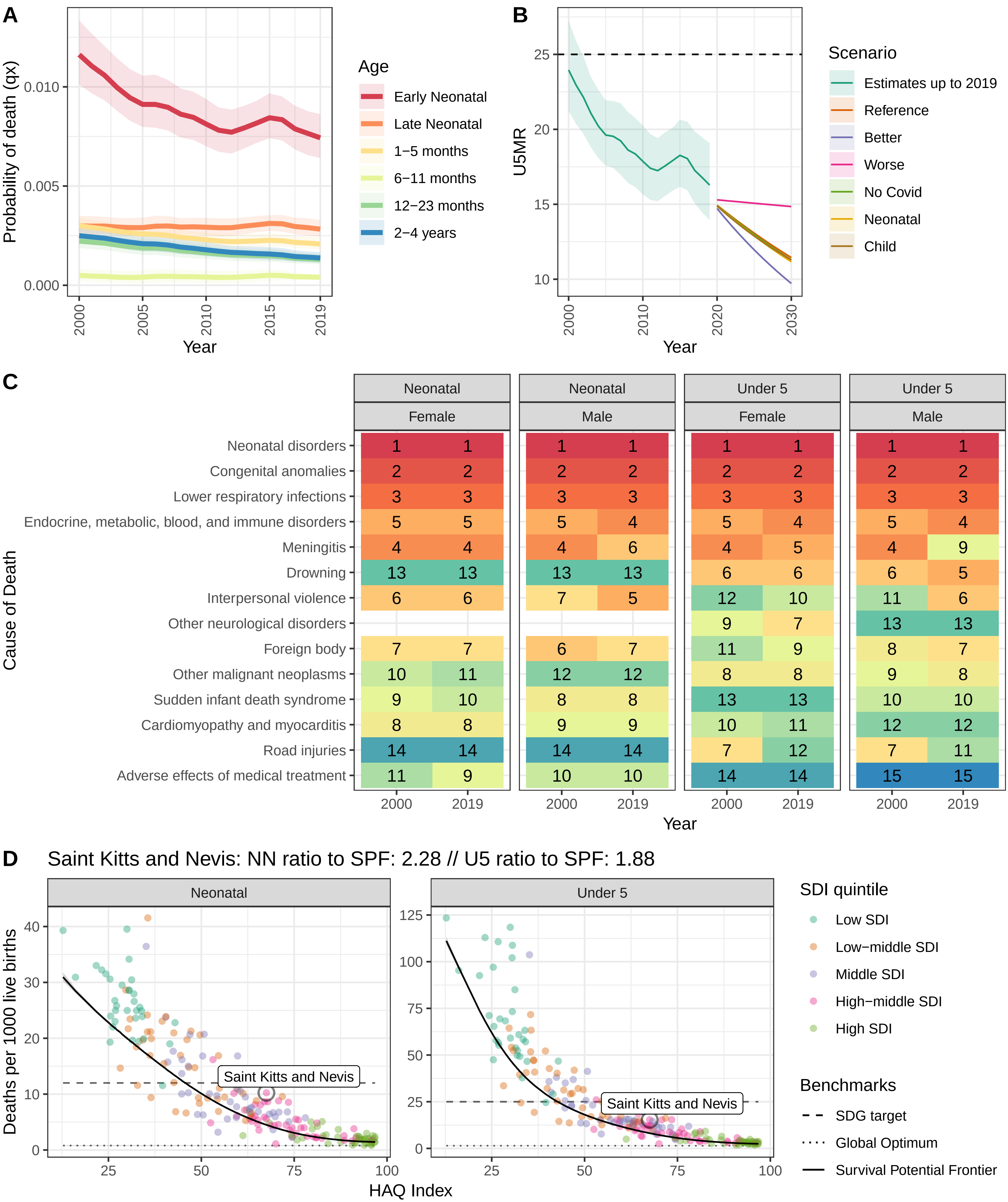

San Marino

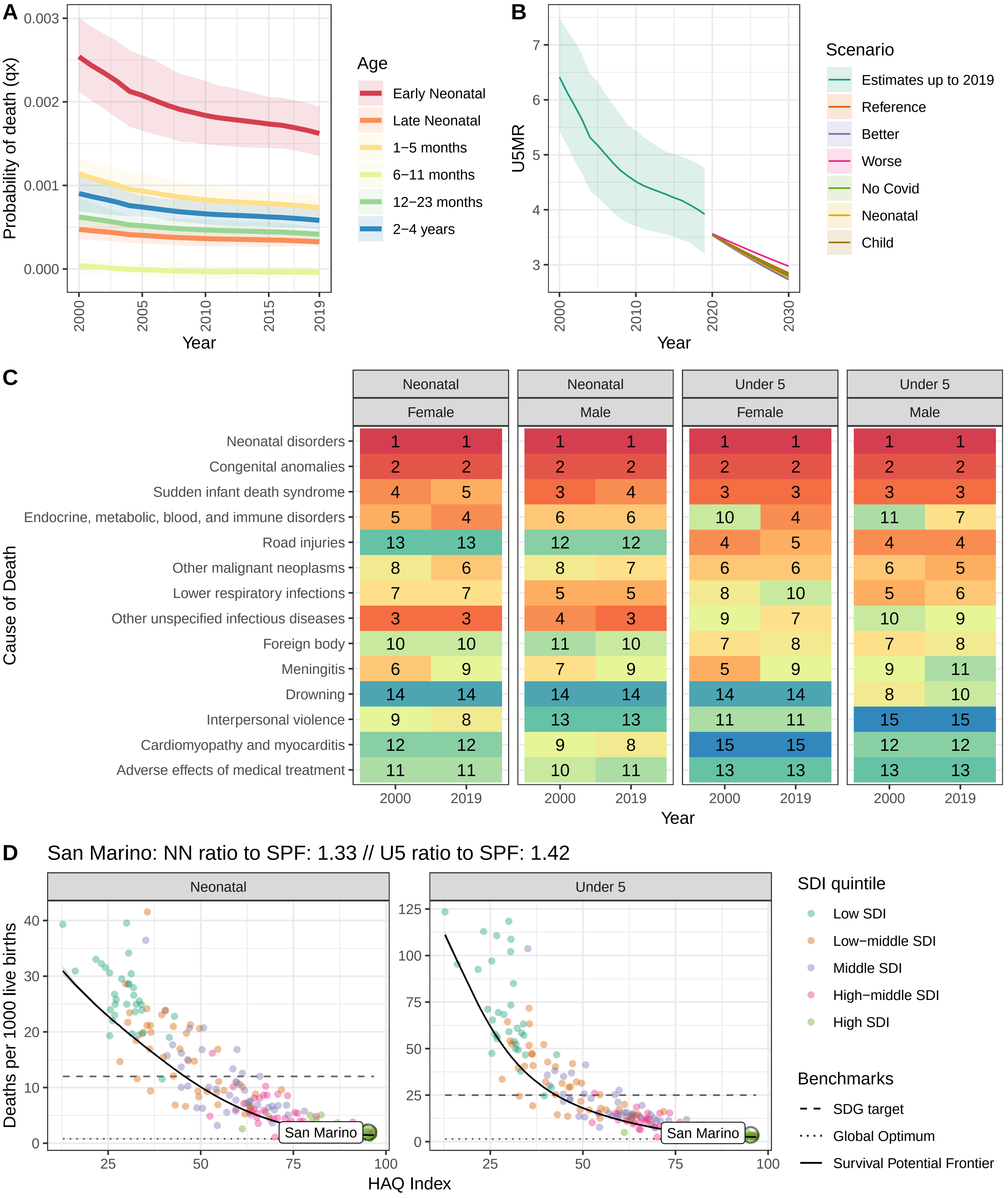
A
Probability of death (qx)
0.003
0.002
0.001
0.000
2000
2005
2010
2015
2019
Year
Age
Early Neonatal
Late Neonatal
1−5 months
6−11 months
12−23 months
2−4 years
B
U5MR
7
6
5
4
3
2000
2010
2020
2030
Year
Scenario
Estimates up to 2019
Reference
Better
Worse
No Covid
Neonatal
Child
C
Cause of Death
Neonatal Female
Neonatal Male
Under 5 Female
Under 5 Male
Neonatal disorders 1 1 1 1 1 1 1 1
Congenital anomalies 2 2 2 2 2 2 2 2
Sudden infant death syndrome 4 5 3 4 3 3 3 3
Endocrine, metabolic, blood, and immune disorders 5 4 6 6 10 4 11 7
Road injuries 13 13 12 12 4 5 4 4
Other malignant neoplasms 8 6 8 7 6 6 6 5
Lower respiratory infections 7 7 5 5 8 10 5 6
Other unspecified infectious diseases 3 3 4 3 9 7 10 9
Foreign body 10 10 11 10 7 8 7 8
Meningitis 6 9 7 9 5 9 9 11
Drowning 14 14 14 14 14 14 8 10
Interpersonal violence 9 8 13 13 11 11 15 15
Cardiomyopathy and myocarditis 12 12 9 8 15 15 12 12
Adverse effects of medical treatment 11 11 10 11 13 13 13 13
2000 2019
Year
D
San Marino: NN ratio to SPF: 1.33 // U5 ratio to SPF: 1.42
Neonatal
Under 5
Deaths per 1000 live births
HAQ Index
San Marino
SDI quintile
Low SDI
Low−middle SDI
Middle SDI
High−middle SDI
High SDI
Benchmarks
SDG target
Global Optimum
Survival Potential Frontier

Turkmenistan

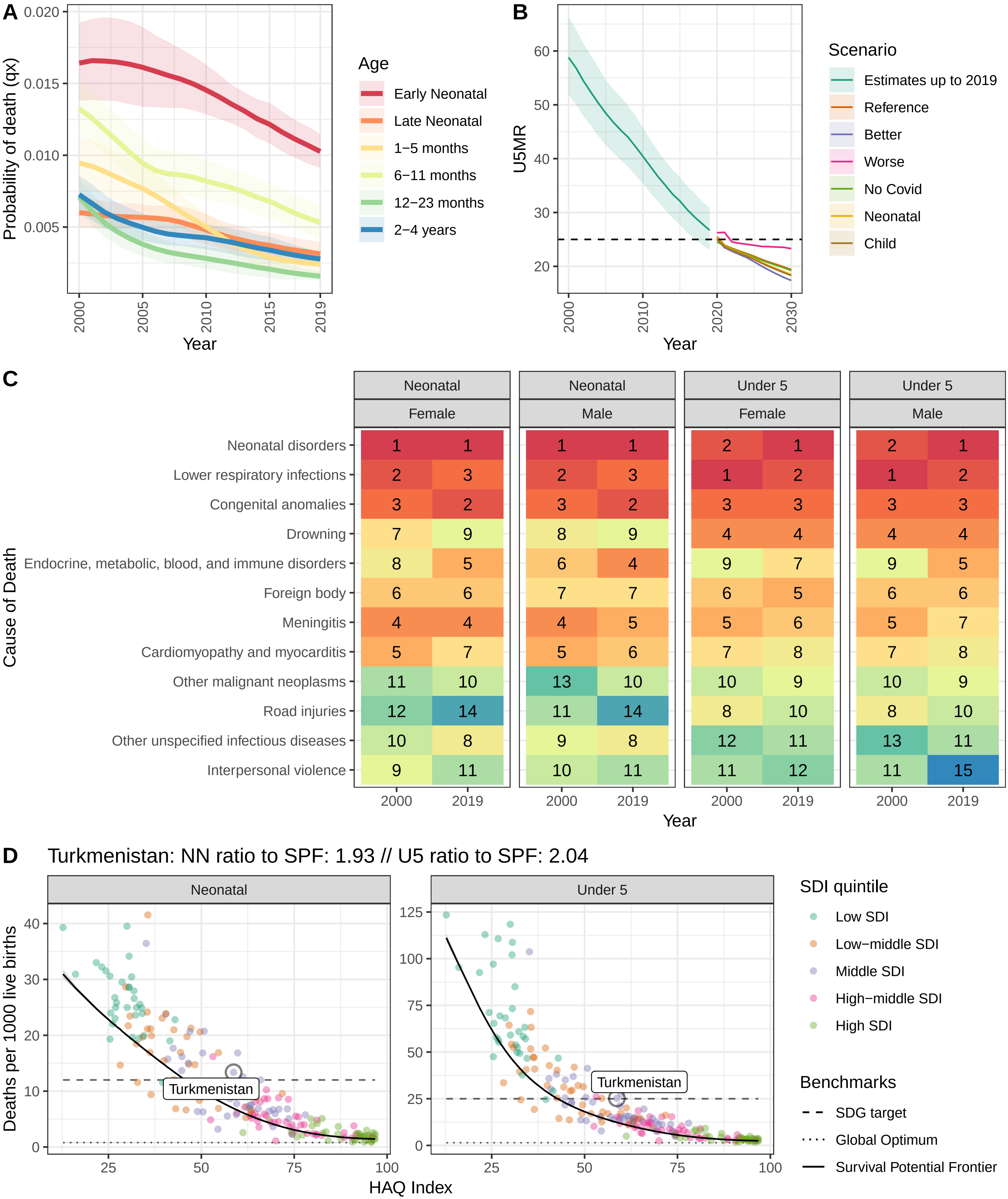

Uzbekistan

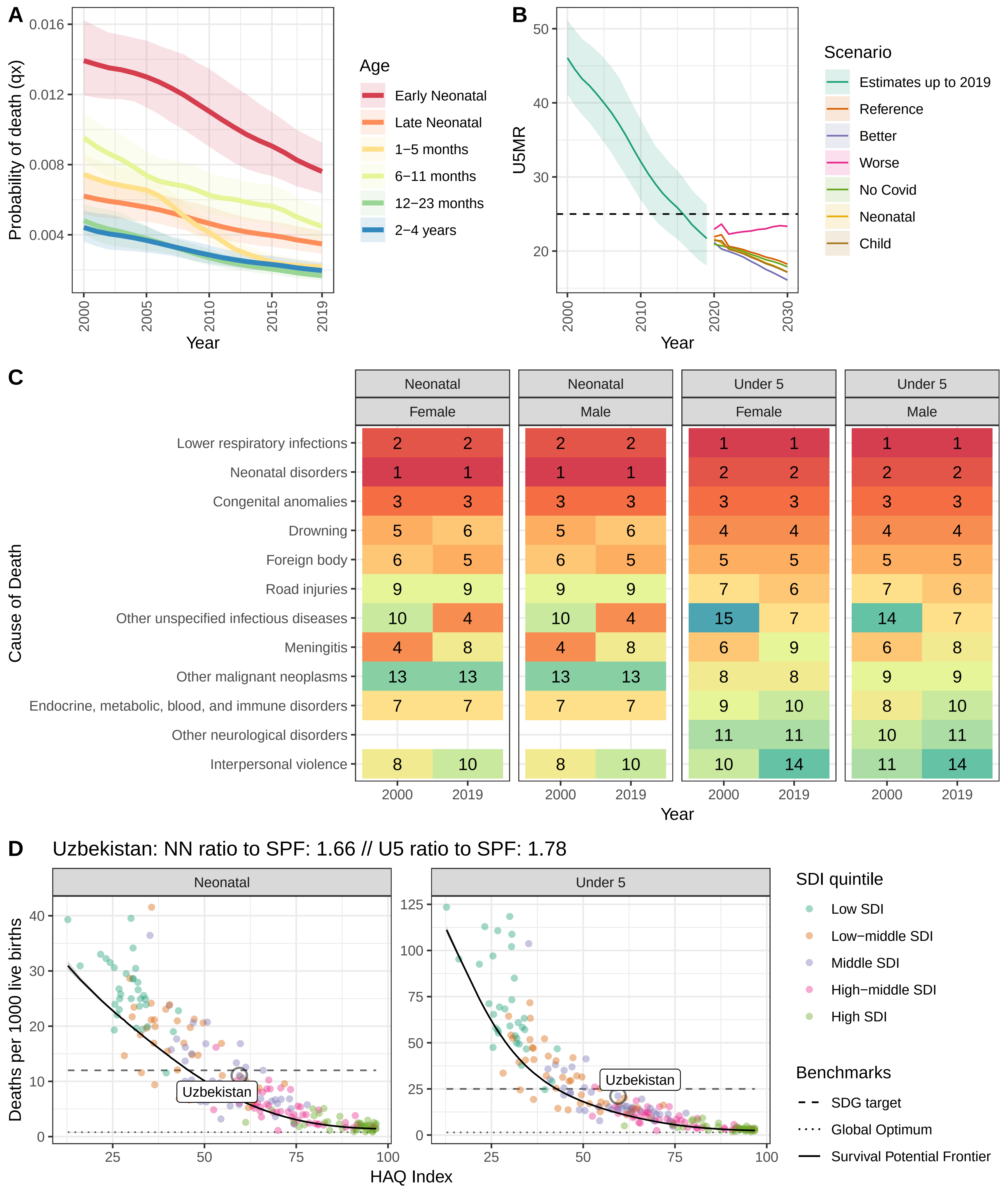
A
Probability of death (qx)
0.016
0.012
0.008
0.004
2000
2005
2010
2015
2019
Year
Age
Early Neonatal
Late Neonatal
1−5 months
6−11 months
12−23 months
2−4 years
B
U5MR
50
40
30
20
2000
2010
2020
2030
Year
Scenario
Estimates up to 2019
Reference
Better
Worse
No Covid
Neonatal
Child
C
Neonatal Female
Neonatal Male
Under 5 Female
Under 5 Male
Cause of Death
Lower respiratory infections 2 2 2 2 1 1 1 1
Neonatal disorders 1 1 1 1 2 2 2 2
Congenital anomalies 3 3 3 3 3 3 3 3
Drowning 5 6 5 6 4 4 4 4
Foreign body 6 5 6 5 5 5 5 5
Road injuries 9 9 9 9 7 6 7 6
Other unspecified infectious diseases 10 4 10 4 15 7 14 7
Meningitis 4 8 4 8 6 9 6 8
Other malignant neoplasms 13 13 13 13 8 8 9 9
Endocrine, metabolic, blood, and immune disorders 7 7 7 7 9 10 8 10
Other neurological disorders 11 11 10 11
Interpersonal violence 8 10 8 10 10 14 11 14
2000
2019
Year
D
Uzbekistan: NN ratio to SPF: 1.66 // U5 ratio to SPF: 1.78
Neonatal
Under 5
Deaths per 1000 live births
40
30
20
10
0
125
100
75
50
25
0
25
50
75
100
HAQ Index
Uzbekistan
Uzbekistan
SDI quintile
Low SDI
Low−middle SDI
Middle SDI
High−middle SDI
High SDI
Benchmarks
SDG target
Global Optimum
Survival Potential Frontier

Tokelau

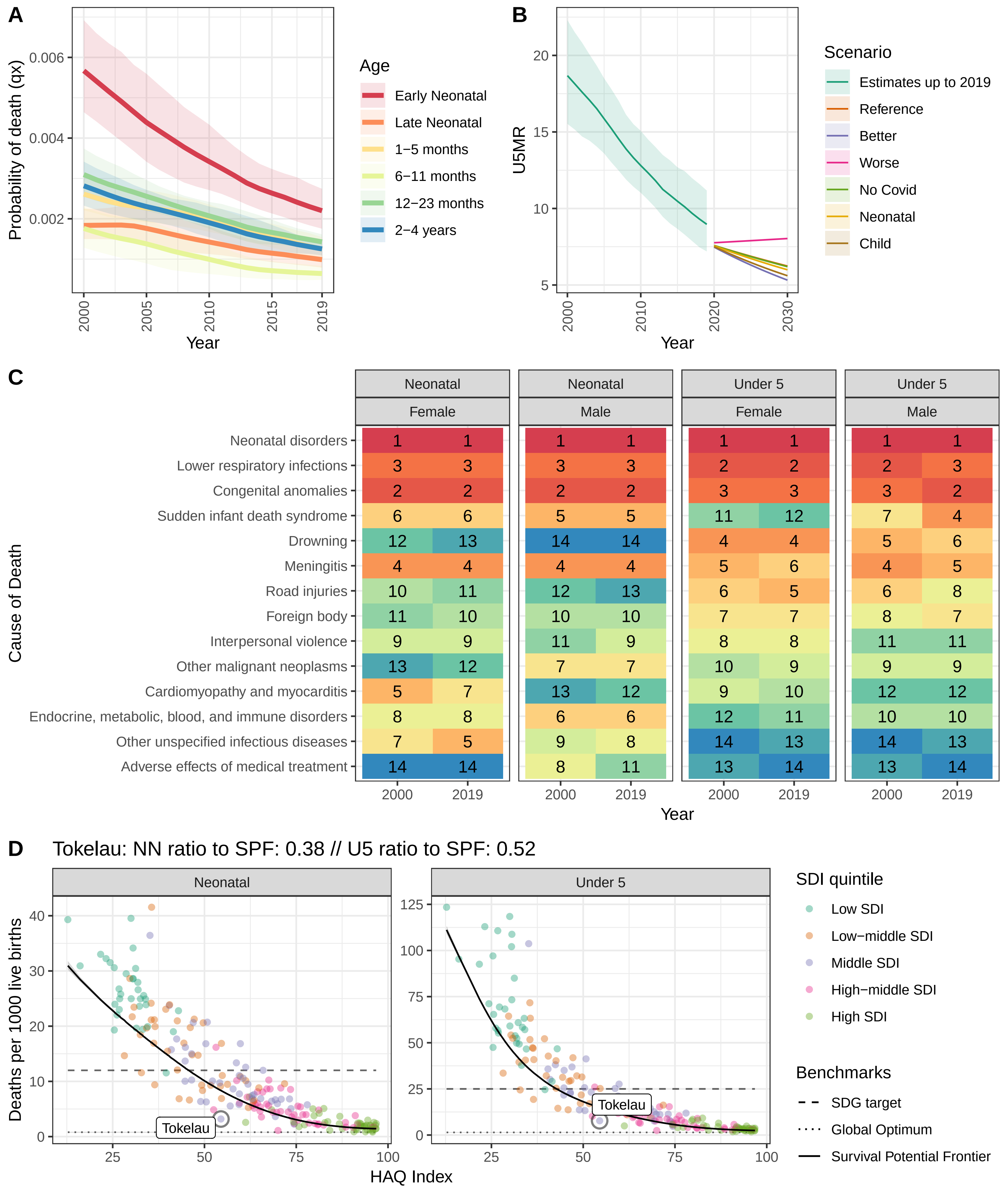
A
Probability of death (qx)
0.006
0.004
0.002
2000
2005
2010
2015
2019
Year
Age
Early Neonatal
Late Neonatal
1−5 months
6−11 months
12−23 months
2−4 years
B
U5MR
20
15
10
5
2000
2010
2020
2030
Year
Scenario
Estimates up to 2019
Reference
Better
Worse
No Covid
Neonatal
Child
C
Neonatal
Female
Neonatal
Male
Under 5
Female
Under 5
Male
Cause of Death
Neonatal disorders 1 1 1 1 1 1 1 1
Lower respiratory infections 3 3 3 3 2 2 2 3
Congenital anomalies 2 2 2 2 3 3 3 2
Sudden infant death syndrome 6 6 5 5 11 12 7 4
Drowning 12 13 14 14 4 4 5 6
Meningitis 4 4 4 4 5 6 4 5
Road injuries 10 11 12 13 6 5 6 8
Foreign body 11 10 10 10 7 7 8 7
Interpersonal violence 9 9 11 9 8 8 11 11
Other malignant neoplasms 13 12 7 7 10 9 9 9
Cardiomyopathy and myocarditis 5 7 13 12 9 10 12 12
Endocrine, metabolic, blood, and immune disorders 8 8 6 6 12 11 10 10
Other unspecified infectious diseases 7 5 9 8 14 13 14 13
Adverse effects of medical treatment 14 14 8 11 13 14 13 14
2000
2019
Year
D
Tokelau: NN ratio to SPF: 0.38 // U5 ratio to SPF: 0.52
Neonatal
Under 5
Deaths per 1000 live births
40
30
20
10
0
125
100
75
50
25
0
Tokelau
Tokelau
25
50
75
100
HAQ Index
SDI quintile
Low SDI
Low−middle SDI
Middle SDI
High−middle SDI
High SDI
Benchmarks
SDG target
Global Optimum
Survival Potential Frontier

Tuvalu

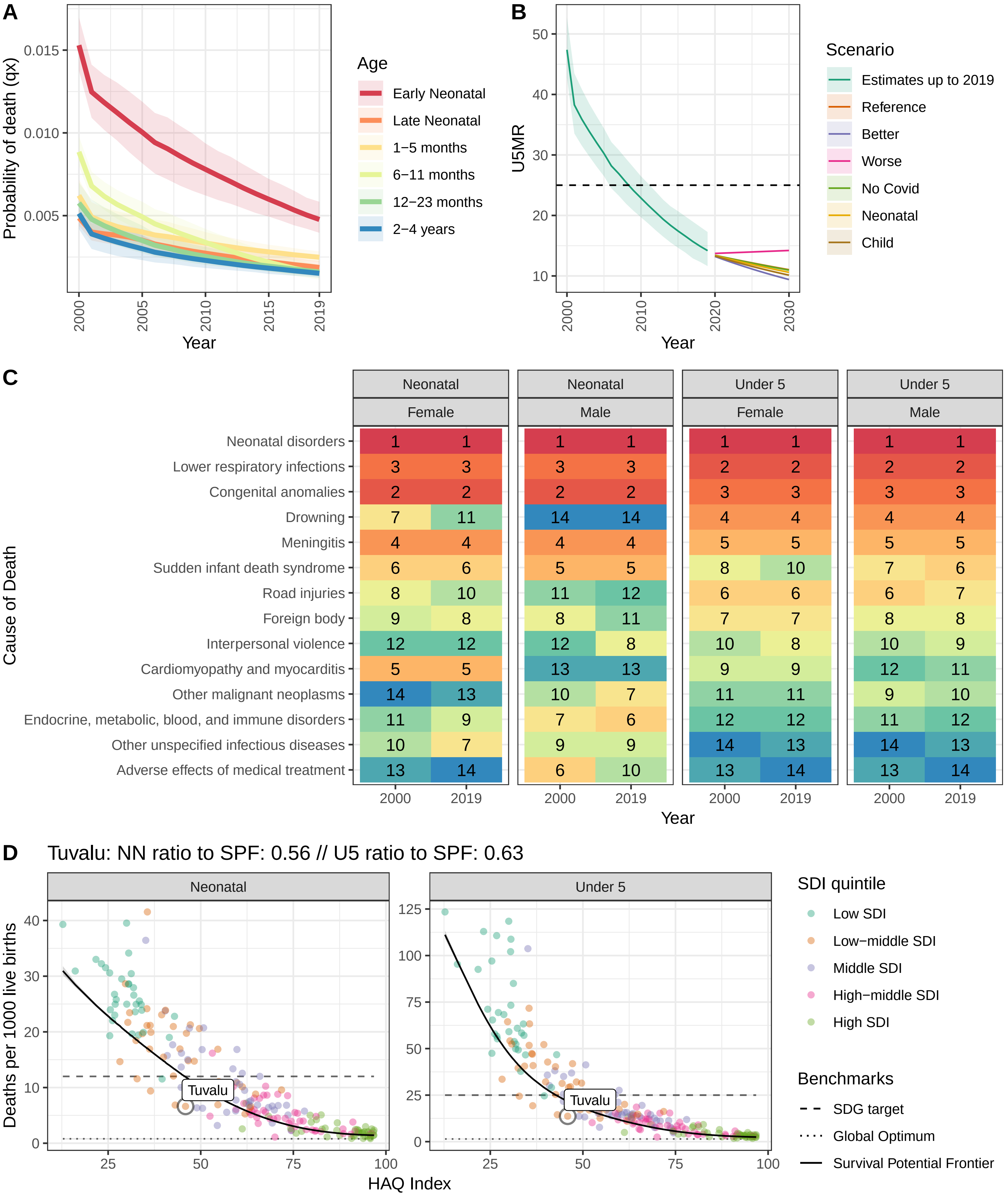

A
Probability of death (qx)
0.015
0.010
0.005
2000
2005
2010
2015
2019
Year
Age
Early Neonatal
Late Neonatal
1−5 months
6−11 months
12−23 months
2−4 years
B
U5MR
50
40
30
20
10
2000
2010
2020
2030
Year
Scenario
Estimates up to 2019
Reference
Better
Worse
No Covid
Neonatal
Child
C
Neonatal Female
Neonatal Male
Under 5 Female
Under 5 Male
Cause of Death
Neonatal disorders 1 1 1 1 1 1 1 1
Lower respiratory infections 3 3 3 3 2 2 2 2
Congenital anomalies 2 2 2 2 3 3 3 3
Drowning 7 11 14 14 4 4 4 4
Meningitis 4 4 4 4 5 5 5 5
Sudden infant death syndrome 6 6 5 5 8 10 7 6
Road injuries 8 10 11 12 6 6 6 7
Foreign body 9 8 8 11 7 7 8 8
Interpersonal violence 12 12 12 8 10 8 10 9
Cardiomyopathy and myocarditis 5 5 13 13 9 9 12 11
Other malignant neoplasms 14 13 10 7 11 11 9 10
Endocrine, metabolic, blood, and immune disorders 11 9 7 6 12 12 11 12
Other unspecified infectious diseases 10 7 9 9 14 13 14 13
Adverse effects of medical treatment 13 14 6 10 13 14 13 14
2000
2019
Year
D
Tuvalu: NN ratio to SPF: 0.56 // U5 ratio to SPF: 0.63
Neonatal
Under 5
Deaths per 1000 live births
40
30
20
10
0
125
100
75
50
25
0
Tuvalu
25
50
75
100
HAQ Index
SDI quintile
Low SDI
Low−middle SDI
Middle SDI
High−middle SDI
High SDI
Benchmarks
SDG target
Global Optimum
Survival Potential Frontier

United States Virgin Islands

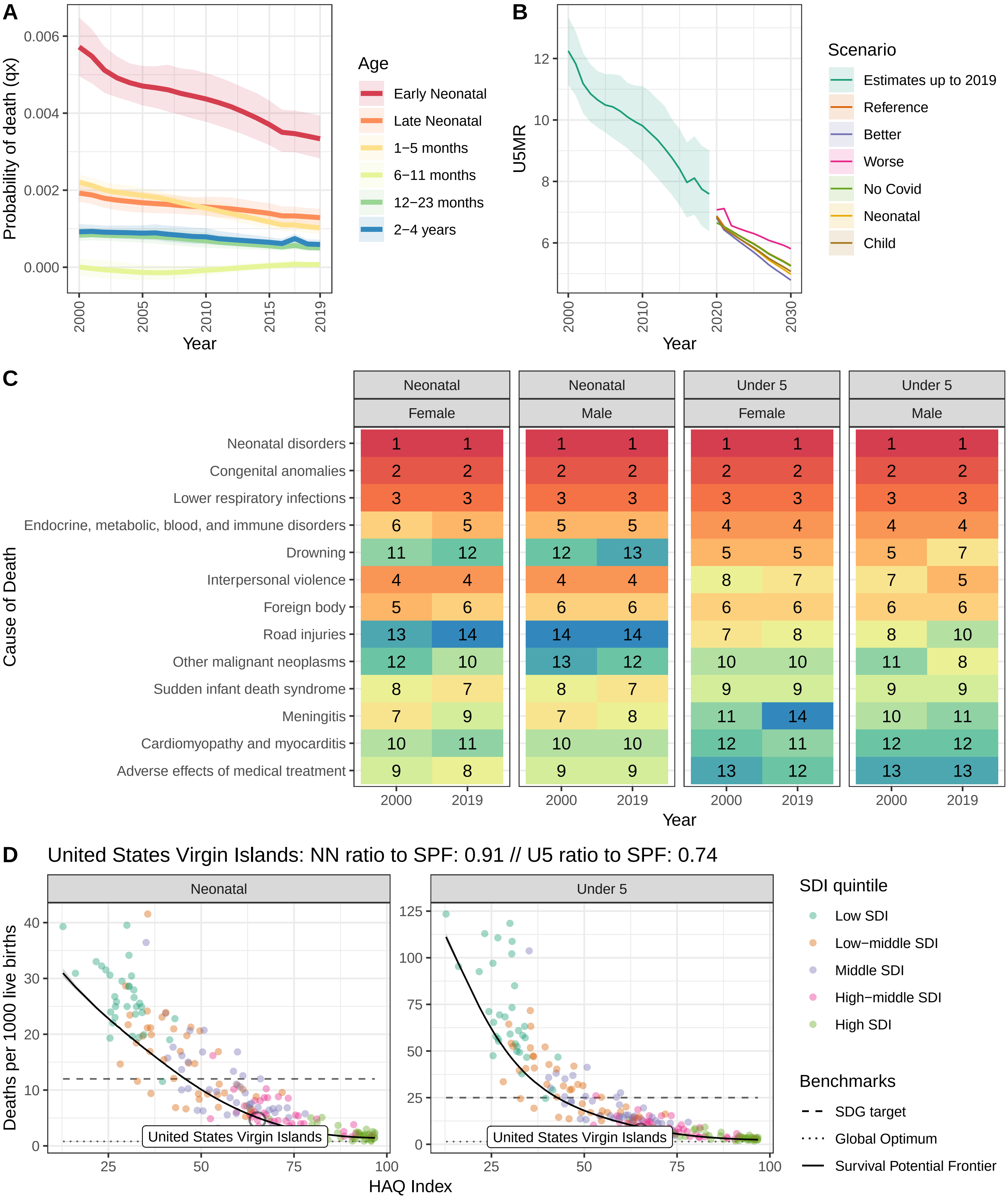

Albania

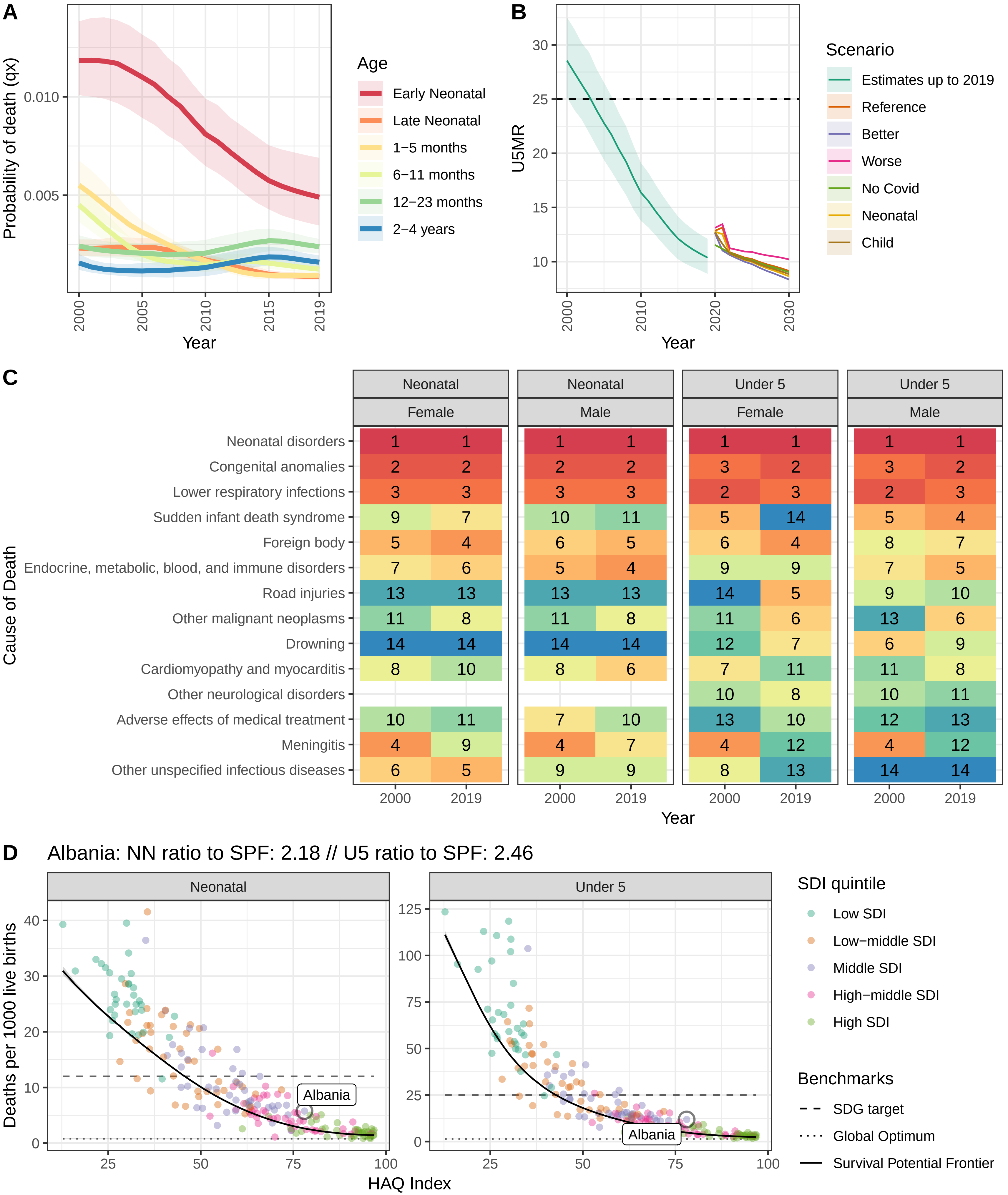
A
Probability of death (qx)
Year
Age
Early Neonatal
Late Neonatal
1−5 months
6−11 months
12−23 months
2−4 years
B
U5MR
Year
Scenario
Estimates up to 2019
Reference
Better
Worse
No Covid
Neonatal
Child
C
Neonatal
Female
Neonatal
Male
Under 5
Female
Under 5
Male
Cause of Death
Neonatal disorders
Congenital anomalies
Lower respiratory infections
Sudden infant death syndrome
Foreign body
Endocrine, metabolic, blood, and immune disorders
Road injuries
Other malignant neoplasms
Drowning
Cardiomyopathy and myocarditis
Other neurological disorders
Adverse effects of medical treatment
Meningitis
Other unspecified infectious diseases
Year
D
Albania: NN ratio to SPF: 2.18 // U5 ratio to SPF: 2.46
Neonatal
Under 5
Deaths per 1000 live births
HAQ Index
Albania
SDI quintile
Low SDI
Low−middle SDI
Middle SDI
High−middle SDI
High SDI
Benchmarks
SDG target
Global Optimum
Survival Potential Frontier

South Sudan

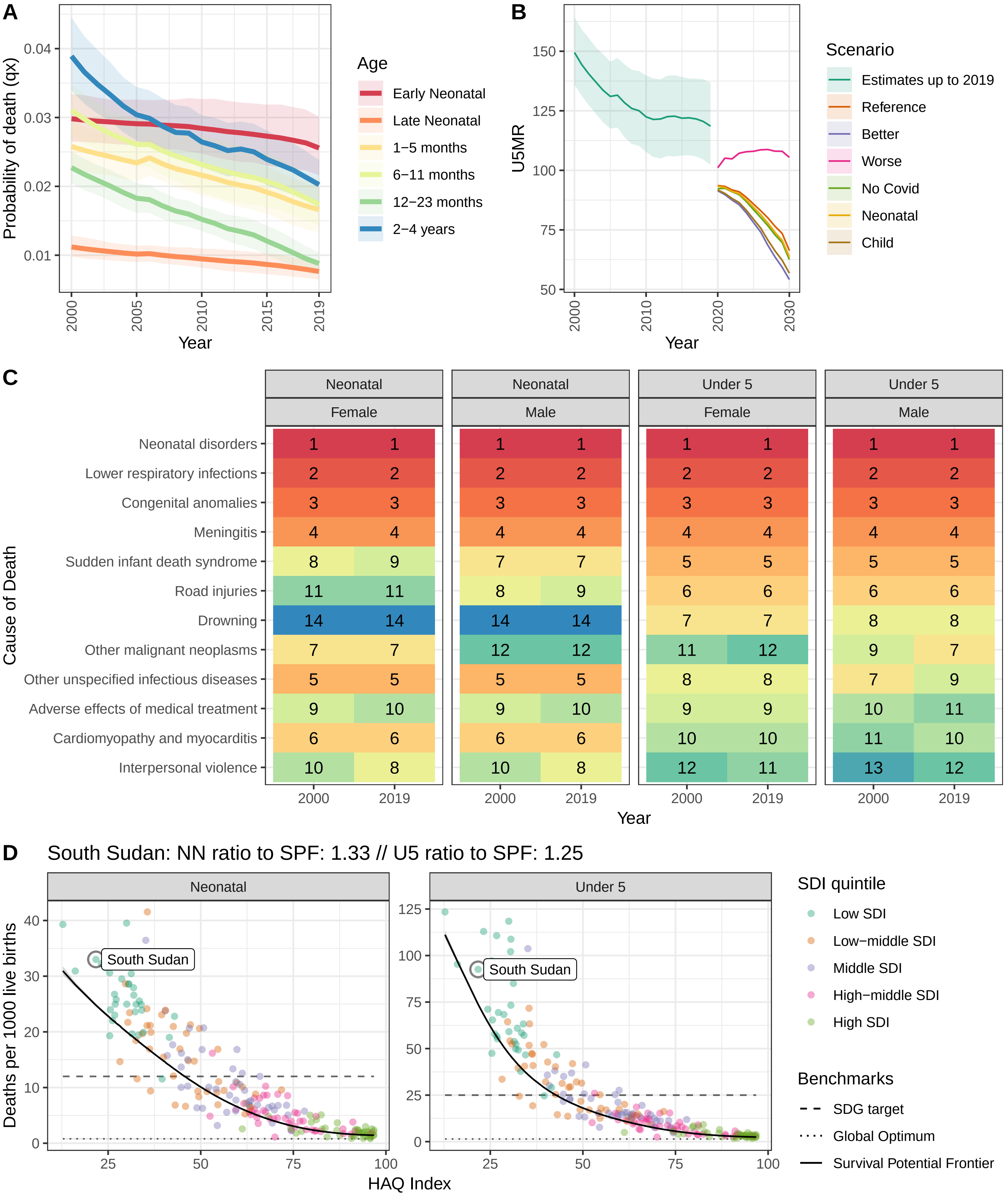
A
Probability of death (qx)
0.04
0.03
0.02
0.01
2000
2005
2010
2015
2019
Year
Age
Early Neonatal
Late Neonatal
1−5 months
6−11 months
12−23 months
2−4 years
B
U5MR
150
125
100
75
50
2000
2010
2020
2030
Year
Scenario
Estimates up to 2019
Reference
Better
Worse
No Covid
Neonatal
Child
C
Neonatal
Female
Neonatal
Male
Under 5
Female
Under 5
Male
Cause of Death
Neonatal disorders 1 1 1 1 1 1 1 1
Lower respiratory infections 2 2 2 2 2 2 2 2
Congenital anomalies 3 3 3 3 3 3 3 3
Meningitis 4 4 4 4 4 4 4 4
Sudden infant death syndrome 8 9 7 7 5 5 5 5
Road injuries 11 11 8 9 6 6 6 6
Drowning 14 14 14 14 7 7 8 8
Other malignant neoplasms 7 7 12 12 11 12 9 7
Other unspecified infectious diseases 5 5 5 5 8 8 7 9
Adverse effects of medical treatment 9 10 9 10 9 9 10 11
Cardiomyopathy and myocarditis 6 6 6 6 10 10 11 10
Interpersonal violence 10 8 10 8 12 11 13 12
2000
2019
Year
D
South Sudan: NN ratio to SPF: 1.33 // U5 ratio to SPF: 1.25
Neonatal
Under 5
Deaths per 1000 live births
South Sudan
South Sudan
HAQ Index
25
50
75
100
SDI quintile
Low SDI
Low−middle SDI
Middle SDI
High−middle SDI
High SDI
Benchmarks
SDG target
Global Optimum
Survival Potential Frontier

Bosnia and Herzegovina

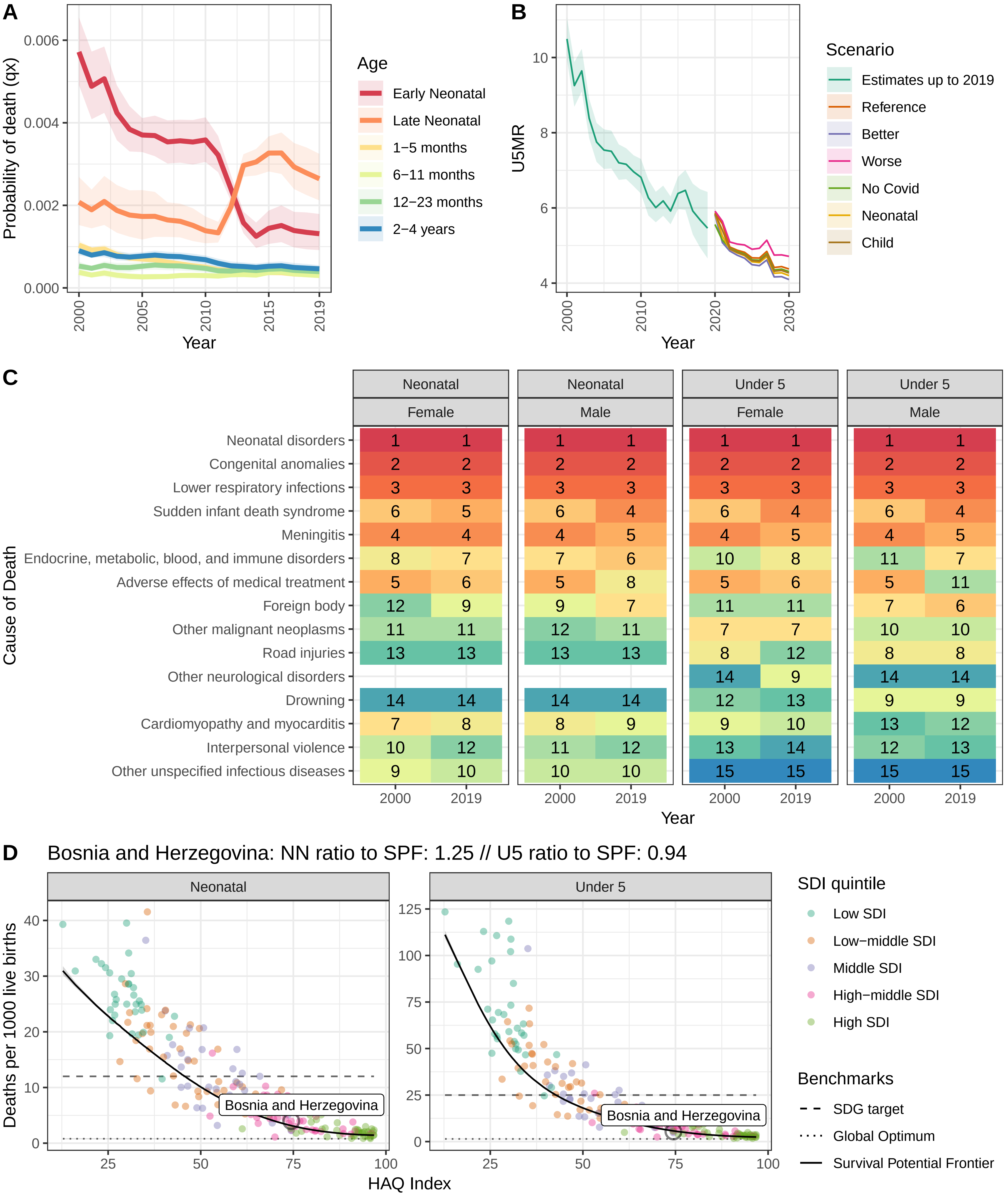

Bulgaria

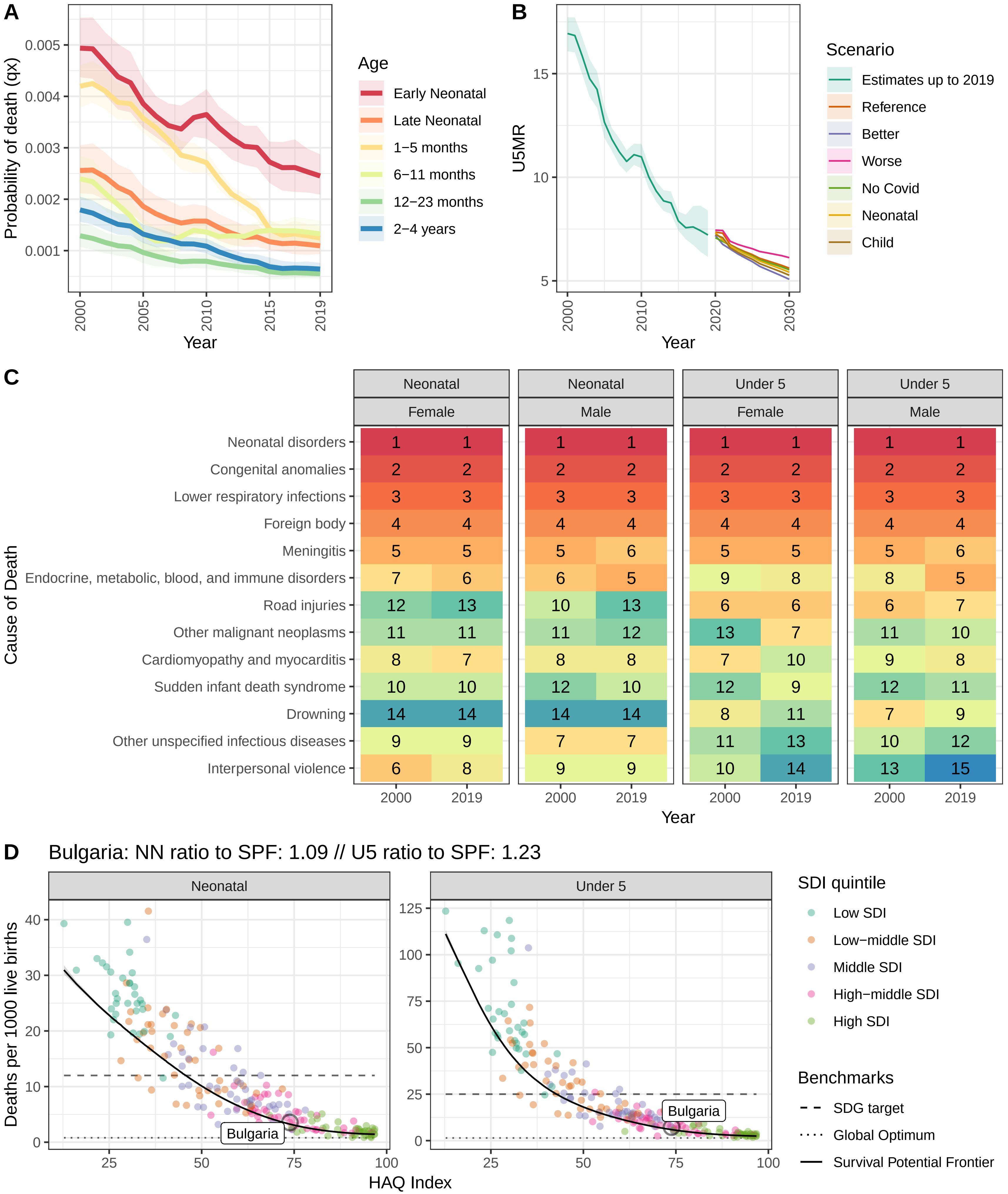
A
Probability of death (qx)
0.005
0.004
0.003
0.002
0.001
2000
2005
2010
2015
2019
Year
Age
Early Neonatal
Late Neonatal
1−5 months
6−11 months
12−23 months
2−4 years
B
U5MR
15
10
5
2000
2010
2020
2030
Year
Scenario
Estimates up to 2019
Reference
Better
Worse
No Covid
Neonatal
Child
C
Neonatal
Female
Neonatal
Male
Under 5
Female
Under 5
Male
Cause of Death
Neonatal disorders 1 1 1 1 1 1 1 1
Congenital anomalies 2 2 2 2 2 2 2 2
Lower respiratory infections 3 3 3 3 3 3 3 3
Foreign body 4 4 4 4 4 4 4 4
Meningitis 5 5 5 6 5 5 5 6
Endocrine, metabolic, blood, and immune disorders 7 6 6 5 9 8 8 5
Road injuries 12 13 10 13 6 6 6 7
Other malignant neoplasms 11 11 11 12 13 7 11 10
Cardiomyopathy and myocarditis 8 7 8 8 7 10 9 8
Sudden infant death syndrome 10 10 12 10 12 9 12 11
Drowning 14 14 14 14 8 11 7 9
Other unspecified infectious diseases 9 9 7 7 11 13 10 12
Interpersonal violence 6 8 9 9 10 14 13 15
2000
2019
Year
D
Bulgaria: NN ratio to SPF: 1.09 // U5 ratio to SPF: 1.23
Neonatal
Under 5
Deaths per 1000 live births
40
30
20
10
0
125
100
75
50
25
Bulgaria
HAQ Index
SDI quintile
Low SDI
Low−middle SDI
Middle SDI
High−middle SDI
High SDI
Benchmarks
SDG target
Global Optimum
Survival Potential Frontier

Croatia

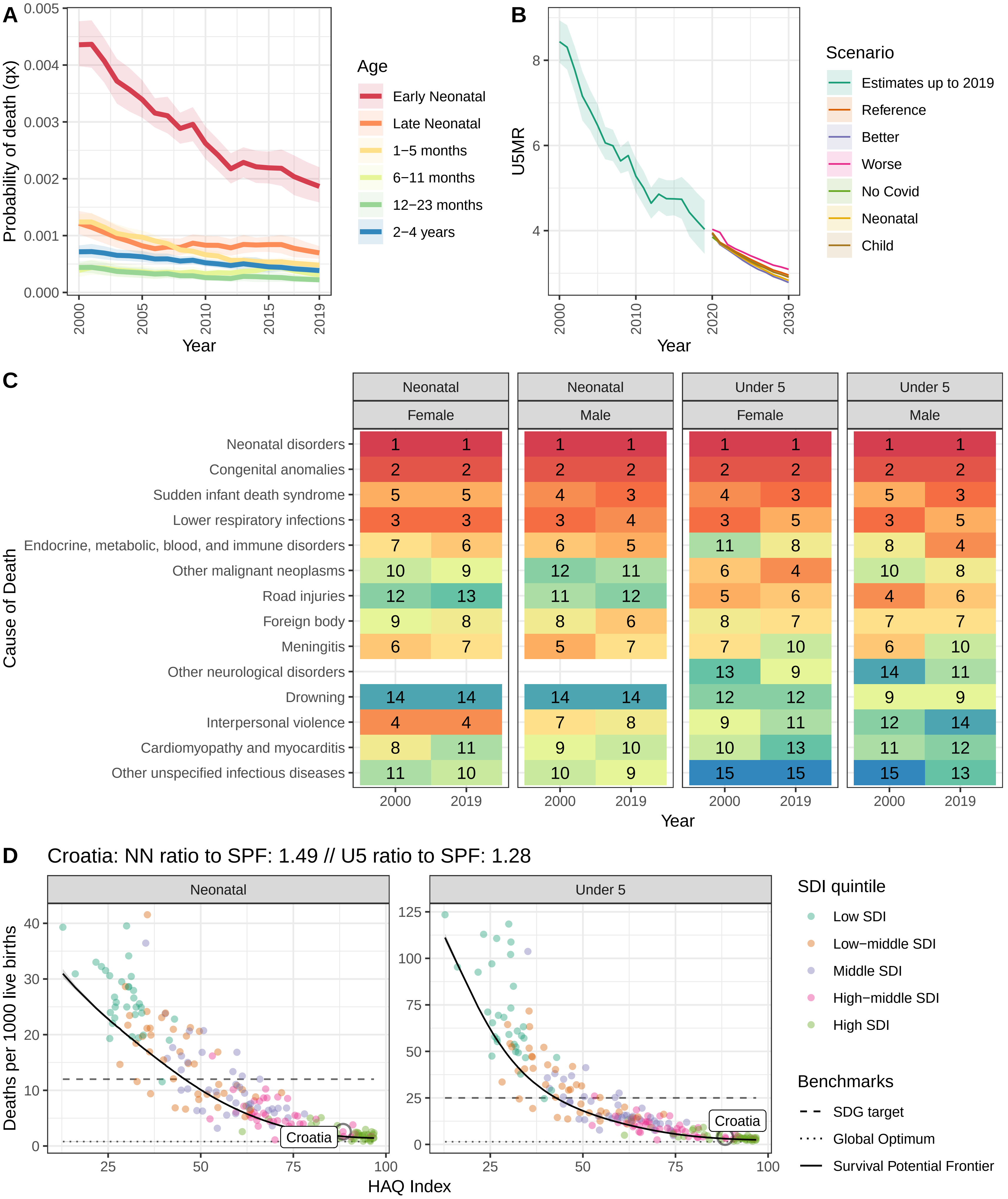
A
Probability of death (qx)
0.005
0.004
0.003
0.002
0.001
0.000
2000
2005
2010
2015
2019
Year
Age
Early Neonatal
Late Neonatal
1−5 months
6−11 months
12−23 months
2−4 years
B
U5MR
8
6
4
2000
2010
2020
2030
Year
Scenario
Estimates up to 2019
Reference
Better
Worse
No Covid
Neonatal
Child
C
Cause of Death
Neonatal
Female
Neonatal
Male
Under 5
Female
Under 5
Male
Neonatal disorders 1 1 1 1 1 1 1 1
Congenital anomalies 2 2 2 2 2 2 2 2
Sudden infant death syndrome 5 5 4 3 4 3 5 3
Lower respiratory infections 3 3 3 4 3 5 3 5
Endocrine, metabolic, blood, and immune disorders 7 6 6 5 11 8 8 4
Other malignant neoplasms 10 9 12 11 6 4 10 8
Road injuries 12 13 11 12 5 6 4 6
Foreign body 9 8 8 6 8 7 7 7
Meningitis 6 7 5 7 7 10 6 10
Other neurological disorders 13 9 14 11
Drowning 14 14 14 14 12 12 9 9
Interpersonal violence 4 4 7 8 9 11 12 14
Cardiomyopathy and myocarditis 8 11 9 10 10 13 11 12
Other unspecified infectious diseases 11 10 10 9 15 15 15 13
2000
2019
Year
D
Croatia: NN ratio to SPF: 1.49 // U5 ratio to SPF: 1.28
Neonatal
Under 5
Deaths per 1000 live births
40
30
20
10
0
125
100
75
50
25
0
25
50
75
100
Croatia
HAQ Index
SDI quintile
Low SDI
Low−middle SDI
Middle SDI
High−middle SDI
High SDI
Benchmarks
SDG target
Global Optimum
Survival Potential Frontier

Czechia

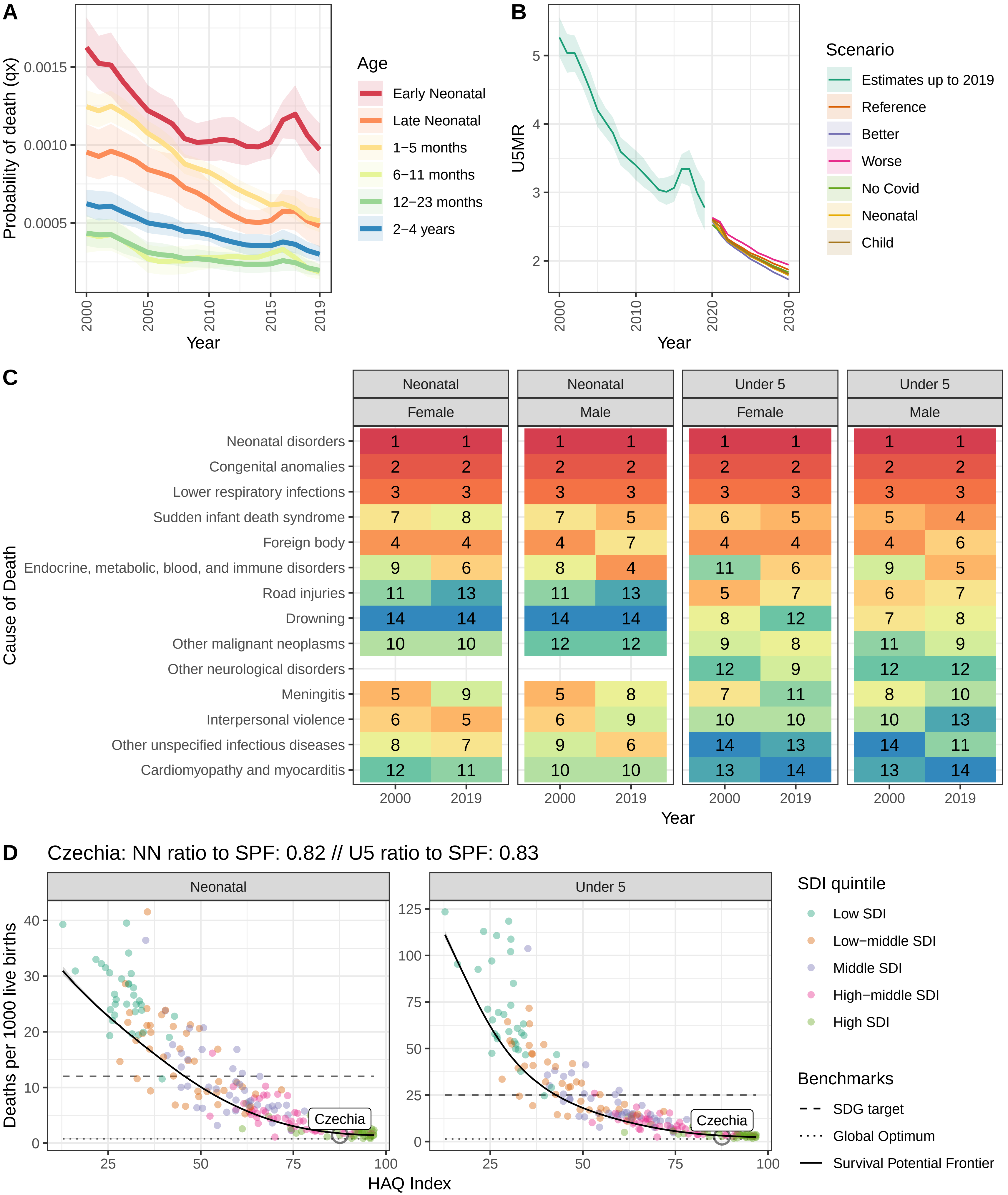

Cause of Death	Neonatal Female 2000	Neonatal Female 2019	Neonatal Male 2000	Neonatal Male 2019	Under 5 Female 2000	Under 5 Female 2019	Under 5 Male 2000	Under 5 Male 2019
Neonatal disorders	1	1	1	1	1	1	1	1
Congenital anomalies	2	2	2	2	2	2	2	2
Lower respiratory infections	3	3	3	3	3	3	3	3
Sudden infant death syndrome	7	8	7	5	6	5	5	4
Foreign body	4	4	4	7	4	4	4	6
Endocrine, metabolic, blood, and immune disorders	9	6	8	4	11	6	9	5
Road injuries	11	13	11	13	5	7	6	7
Drowning	14	14	14	14	8	12	7	8
Other malignant neoplasms	10	10	12	12	9	8	11	9
Other neurological disorders					12	9	12	12
Meningitis	5	9	5	8	7	11	8	10
Interpersonal violence	6	5	6	9	10	10	10	13
Other unspecified infectious diseases	8	7	9	6	14	13	14	11
Cardiomyopathy and myocarditis	12	11	10	10	13	14	13	14

Hungary

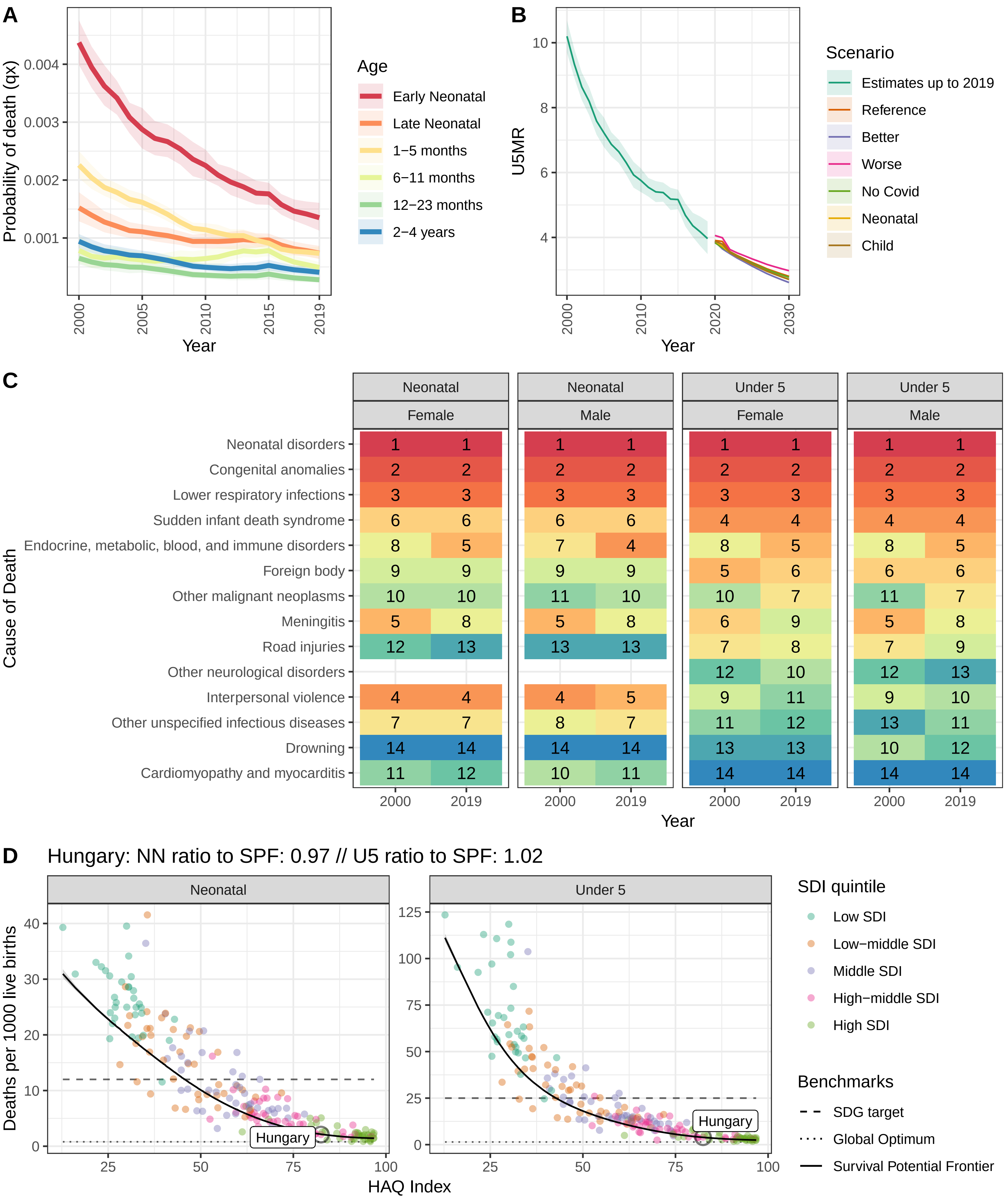
A
Probability of death (qx)
0.004
0.003
0.002
0.001
2000
2005
2010
2015
2019
Year
Age
Early Neonatal
Late Neonatal
1−5 months
6−11 months
12−23 months
2−4 years
B
U5MR
10
8
6
4
2000
2010
2020
2030
Year
Scenario
Estimates up to 2019
Reference
Better
Worse
No Covid
Neonatal
Child
C
Neonatal Female
Neonatal Male
Under 5 Female
Under 5 Male
Cause of Death
Neonatal disorders 1 1 1 1 1 1 1 1
Congenital anomalies 2 2 2 2 2 2 2 2
Lower respiratory infections 3 3 3 3 3 3 3 3
Sudden infant death syndrome 6 6 6 6 4 4 4 4
Endocrine, metabolic, blood, and immune disorders 8 5 7 4 8 5 8 5
Foreign body 9 9 9 9 5 6 6 6
Other malignant neoplasms 10 10 11 10 10 7 11 7
Meningitis 5 8 5 8 6 9 5 8
Road injuries 12 13 13 13 7 8 7 9
Other neurological disorders 12 10 12 13
Interpersonal violence 4 4 4 5 9 11 9 10
Other unspecified infectious diseases 7 7 8 7 11 12 13 11
Drowning 14 14 14 14 13 13 10 12
Cardiomyopathy and myocarditis 11 12 10 11 14 14 14 14
2000
2019
Year
D
Hungary: NN ratio to SPF: 0.97 // U5 ratio to SPF: 1.02
Neonatal
Under 5
Deaths per 1000 live births
40
30
20
10
0
125
100
75
50
25
0
Hungary
Hungary
25
50
75
100
HAQ Index
SDI quintile
Low SDI
Low−middle SDI
Middle SDI
High−middle SDI
High SDI
Benchmarks
SDG target
Global Optimum
Survival Potential Frontier

North Macedonia

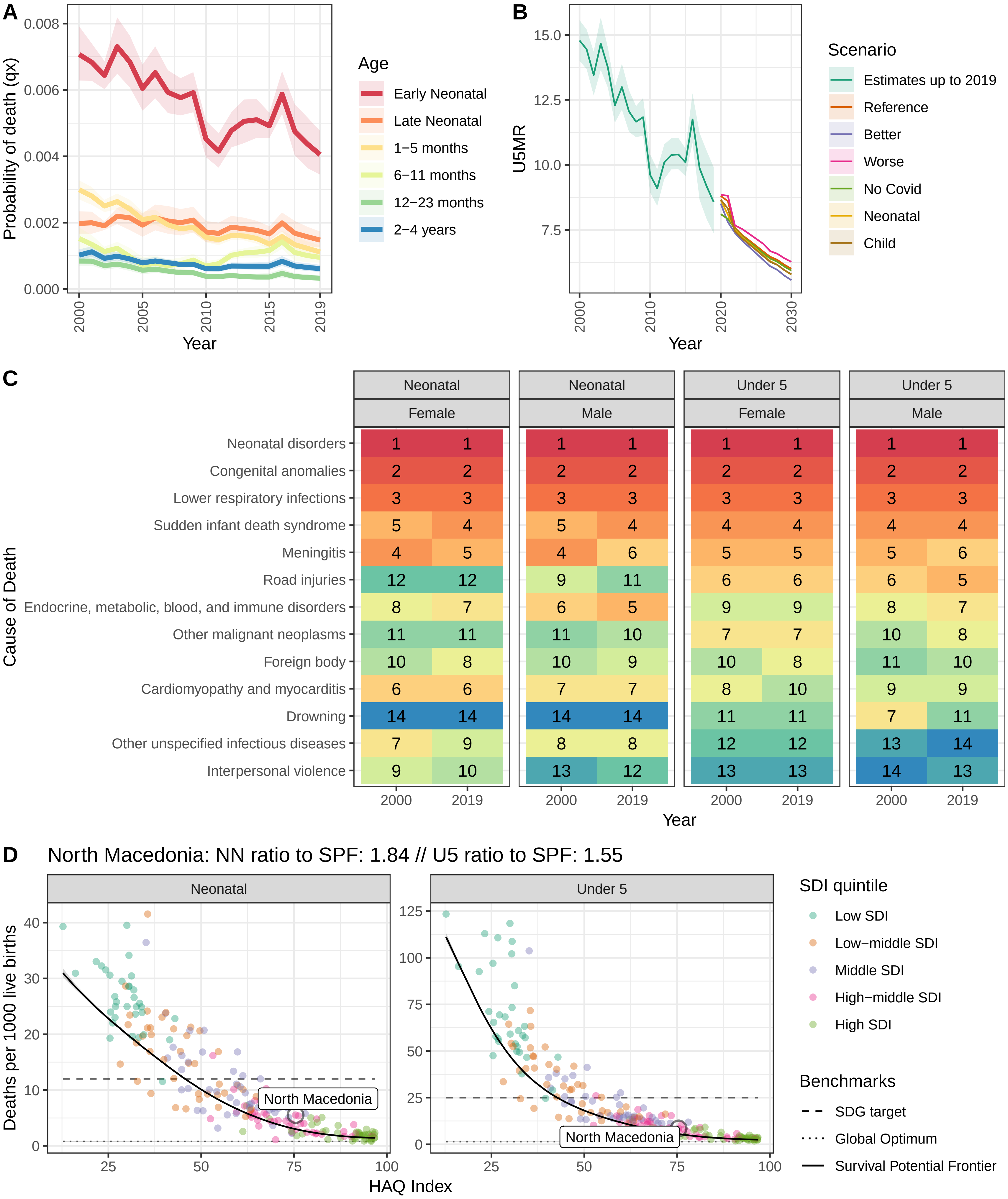

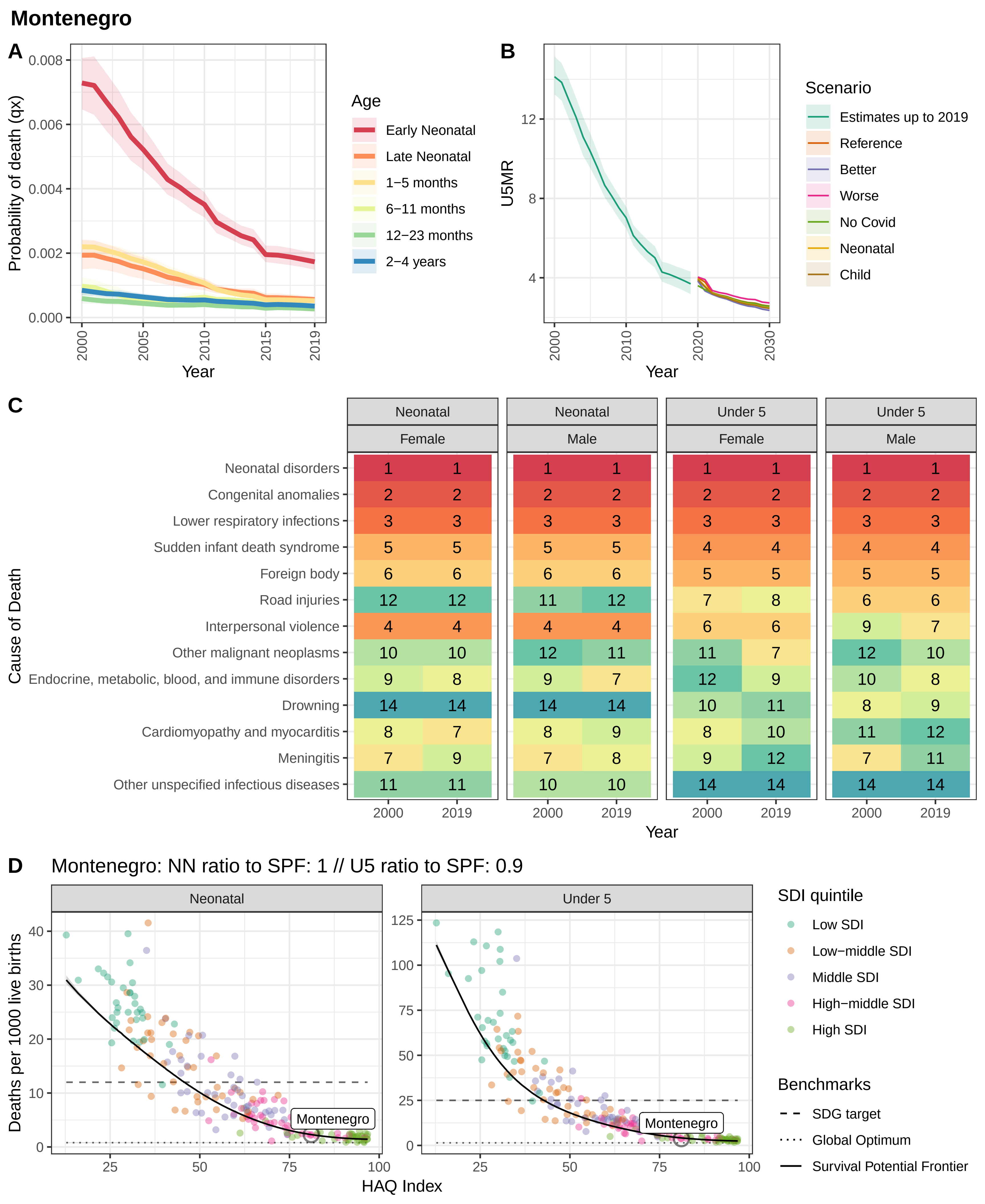

Montenegro
A
Probability of death (qx)
0.008
0.006
0.004
0.002
0.000
2000
2005
2010
2015
2019
Year
Age
Early Neonatal
Late Neonatal
1−5 months
6−11 months
12−23 months
2−4 years
B
U5MR
12
8
4
2000
2010
2020
2030
Year
Scenario
Estimates up to 2019
Reference
Better
Worse
No Covid
Neonatal
Child
C
Neonatal
Female
Neonatal
Male
Under 5
Female
Under 5
Male
Cause of Death
Neonatal disorders 1 1 1 1 1 1 1 1
Congenital anomalies 2 2 2 2 2 2 2 2
Lower respiratory infections 3 3 3 3 3 3 3 3
Sudden infant death syndrome 5 5 5 5 4 4 4 4
Foreign body 6 6 6 6 5 5 5 5
Road injuries 12 12 11 12 7 8 6 6
Interpersonal violence 4 4 4 4 6 6 9 7
Other malignant neoplasms 10 10 12 11 11 7 12 10
Endocrine, metabolic, blood, and immune disorders 9 8 9 7 12 9 10 8
Drowning 14 14 14 14 10 11 8 9
Cardiomyopathy and myocarditis 8 7 8 9 8 10 11 12
Meningitis 7 9 7 8 9 12 7 11
Other unspecified infectious diseases 11 11 10 10 14 14 14 14
2000
2019
Year
D
Montenegro: NN ratio to SPF: 1 // U5 ratio to SPF: 0.9
Neonatal
Under 5
Deaths per 1000 live births
40
30
20
10
0
125
100
75
50
25
0
Montenegro
Montenegro
25
50
75
100
HAQ Index
SDI quintile
Low SDI
Low−middle SDI
Middle SDI
High−middle SDI
High SDI
Benchmarks
SDG target
Global Optimum
Survival Potential Frontier

Poland

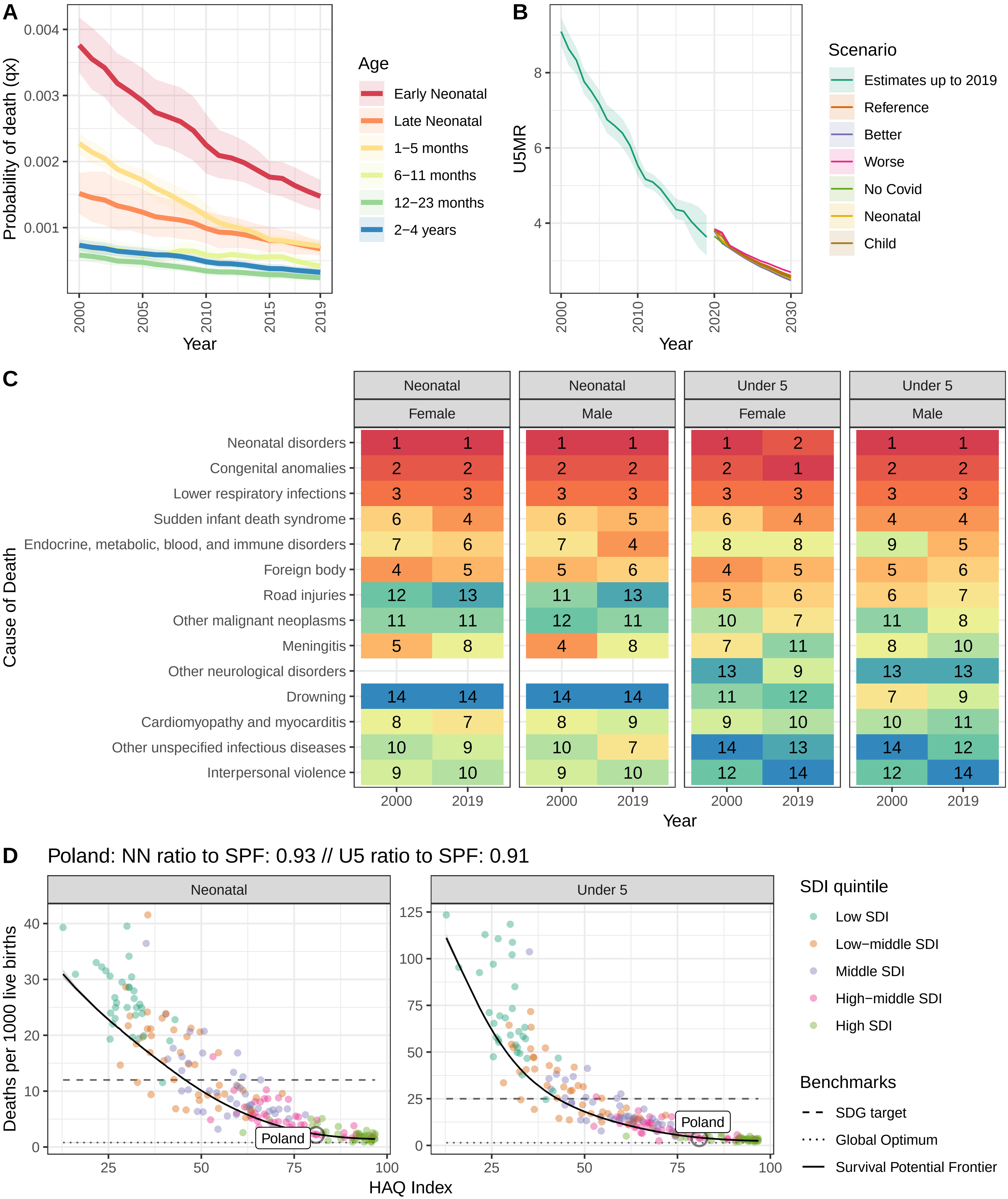
A
Probability of death (qx)
0.004
0.003
0.002
0.001
2000
2005
2010
2015
2019
Year
Age
Early Neonatal
Late Neonatal
1−5 months
6−11 months
12−23 months
2−4 years
B
U5MR
8
6
4
2000
2010
2020
2030
Year
Scenario
Estimates up to 2019
Reference
Better
Worse
No Covid
Neonatal
Child
C
Neonatal
Female
Neonatal
Male
Under 5
Female
Under 5
Male
Cause of Death
Neonatal disorders 1 1 1 1 1 2 1 1
Congenital anomalies 2 2 2 2 2 1 2 2
Lower respiratory infections 3 3 3 3 3 3 3 3
Sudden infant death syndrome 6 4 6 5 6 4 4 4
Endocrine, metabolic, blood, and immune disorders 7 6 7 4 8 8 9 5
Foreign body 4 5 5 6 4 5 5 6
Road injuries 12 13 11 13 5 6 6 7
Other malignant neoplasms 11 11 12 11 10 7 11 8
Meningitis 5 8 4 8 7 11 8 10
Other neurological disorders 13 9 13 13
Drowning 14 14 14 14 11 12 7 9
Cardiomyopathy and myocarditis 8 7 8 9 9 10 10 11
Other unspecified infectious diseases 10 9 10 7 14 13 14 12
Interpersonal violence 9 10 9 10 12 14 12 14
2000
2019
2000
2019
2000
2019
2000
2019
Year
D
Poland: NN ratio to SPF: 0.93 // U5 ratio to SPF: 0.91
Neonatal
Under 5
Deaths per 1000 live births
40
30
20
10
0
125
100
75
50
25
0
Poland
Poland
25
50
75
100
25
50
75
100
HAQ Index
SDI quintile
Low SDI
Low−middle SDI
Middle SDI
High−middle SDI
High SDI
Benchmarks
SDG target
Global Optimum
Survival Potential Frontier

Romania

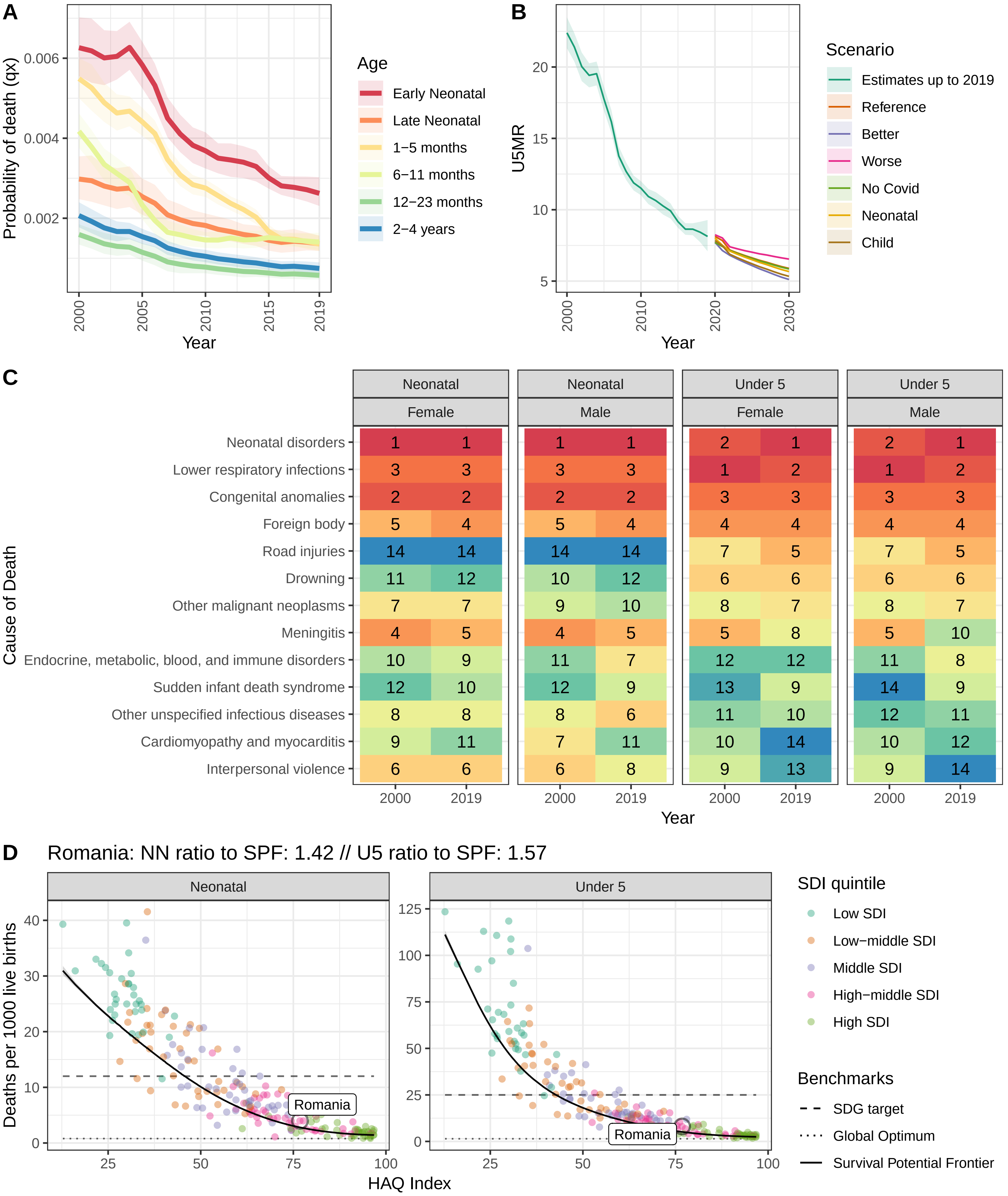
A
Probability of death (qx)
0.006
0.004
0.002
2000
2005
2010
2015
2019
Year
Age
Early Neonatal
Late Neonatal
1−5 months
6−11 months
12−23 months
2−4 years
B
U5MR
20
15
10
5
2000
2010
2020
2030
Year
Scenario
Estimates up to 2019
Reference
Better
Worse
No Covid
Neonatal
Child
C
Neonatal Female
Neonatal Male
Under 5 Female
Under 5 Male
Cause of Death
Neonatal disorders 1 1 1 1 2 1 2 1
Lower respiratory infections 3 3 3 3 1 2 1 2
Congenital anomalies 2 2 2 2 3 3 3 3
Foreign body 5 4 5 4 4 4 4 4
Road injuries 14 14 14 14 7 5 7 5
Drowning 11 12 10 12 6 6 6 6
Other malignant neoplasms 7 7 9 10 8 7 8 7
Meningitis 4 5 4 5 5 8 5 10
Endocrine, metabolic, blood, and immune disorders 10 9 11 7 12 12 11 8
Sudden infant death syndrome 12 10 12 9 13 9 14 9
Other unspecified infectious diseases 8 8 8 6 11 10 12 11
Cardiomyopathy and myocarditis 9 11 7 11 10 14 10 12
Interpersonal violence 6 6 6 8 9 13 9 14
2000 2019 2000 2019 2000 2019 2000 2019
Year
D
Romania: NN ratio to SPF: 1.42 // U5 ratio to SPF: 1.57
Neonatal
Under 5
Deaths per 1000 live births
40
30
20
10
0
125
100
75
50
25
0
Romania
Romania
25
50
75
100
HAQ Index
SDI quintile
Low SDI
Low−middle SDI
Middle SDI
High−middle SDI
High SDI
Benchmarks
SDG target
Global Optimum
Survival Potential Frontier

Sudan

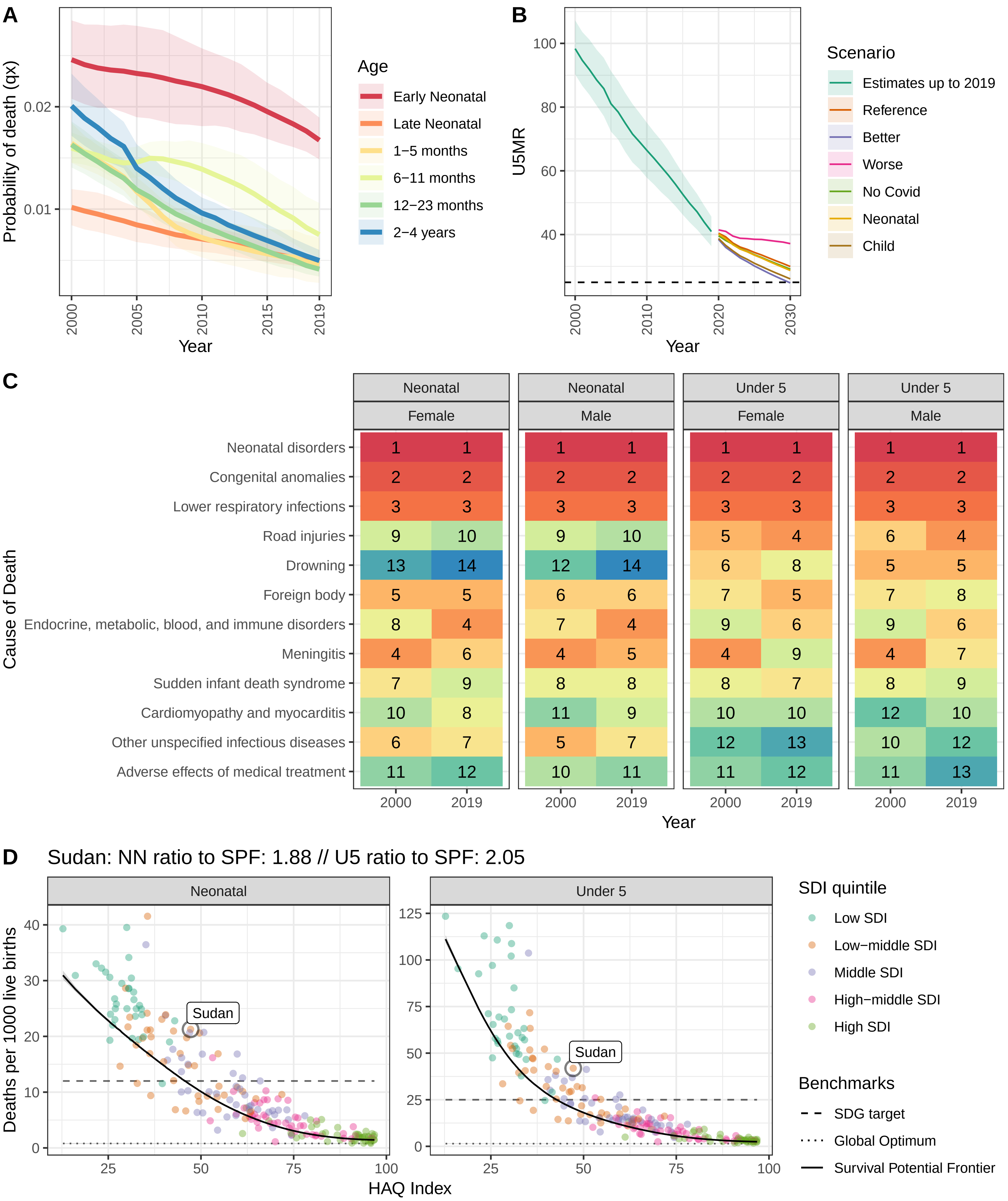

A
Probability of death (qx)
0.02
0.01
2000
2005
2010
2015
2019
Year
Age
Early Neonatal
Late Neonatal
1−5 months
6−11 months
12−23 months
2−4 years
B
U5MR
100
80
60
40
2000
2010
2020
2030
Year
Scenario
Estimates up to 2019
Reference
Better
Worse
No Covid
Neonatal
Child
C
Neonatal
Female
Neonatal
Male
Under 5
Female
Under 5
Male
Cause of Death
Neonatal disorders 1 1 1 1 1 1 1 1
Congenital anomalies 2 2 2 2 2 2 2 2
Lower respiratory infections 3 3 3 3 3 3 3 3
Road injuries 9 10 9 10 5 4 6 4
Drowning 13 14 12 14 6 8 5 5
Foreign body 5 5 6 6 7 5 7 8
Endocrine, metabolic, blood, and immune disorders 8 4 7 4 9 6 9 6
Meningitis 4 6 4 5 4 9 4 7
Sudden infant death syndrome 7 9 8 8 8 7 8 9
Cardiomyopathy and myocarditis 10 8 11 9 10 10 12 10
Other unspecified infectious diseases 6 7 5 7 12 13 10 12
Adverse effects of medical treatment 11 12 10 11 11 12 11 13
2000 2019 2000 2019 2000 2019 2000 2019
Year
D
Sudan: NN ratio to SPF: 1.88 // U5 ratio to SPF: 2.05
Neonatal
Under 5
Deaths per 1000 live births
40
30
20
10
0
125
100
75
50
25
0
Sudan
Sudan
25
50
75
100
HAQ Index
SDI quintile
Low SDI
Low−middle SDI
Middle SDI
High−middle SDI
High SDI
Benchmarks
SDG target
Global Optimum
Survival Potential Frontier

Serbia

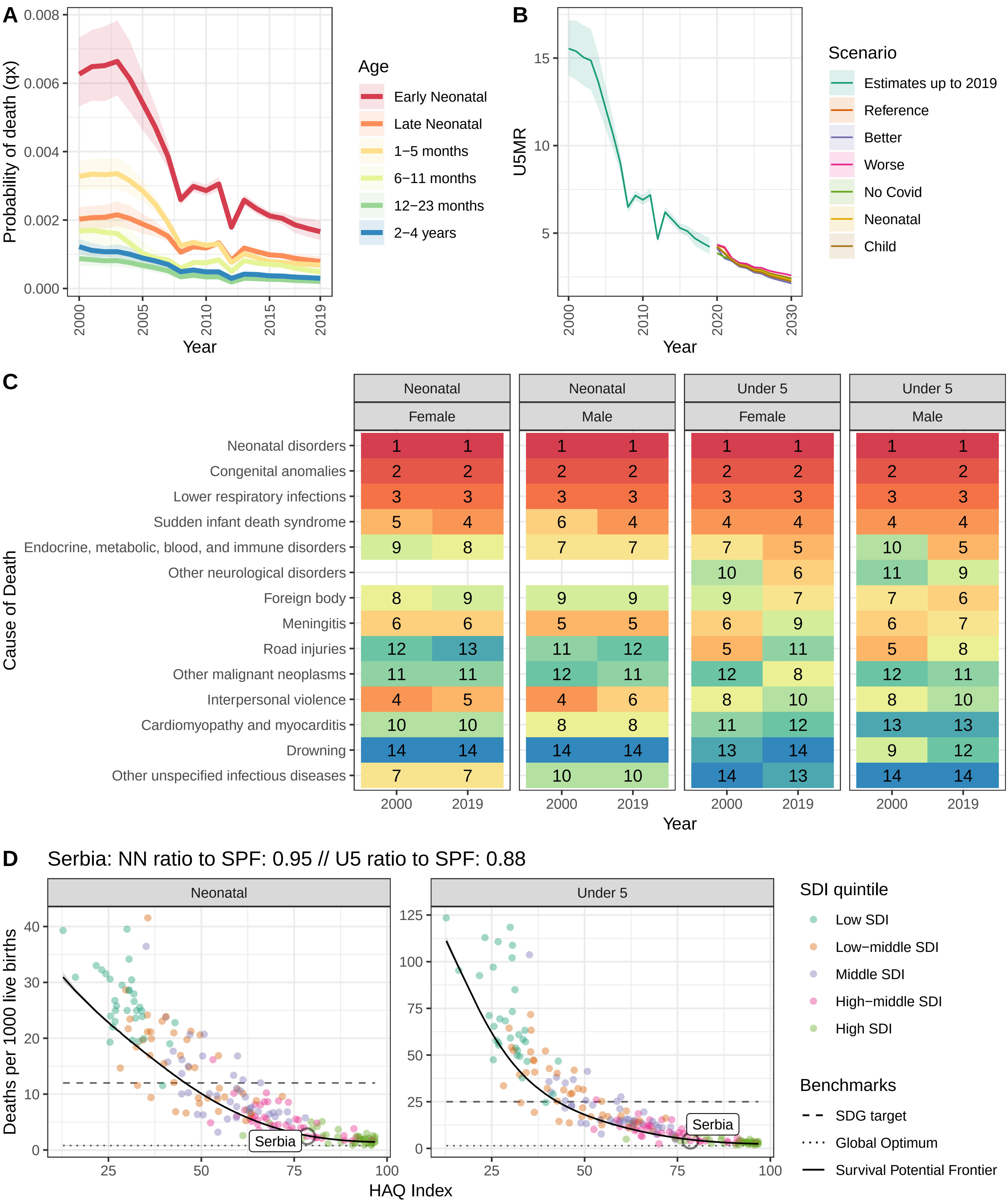

A
Probability of death (qx)
Year
Age
Early Neonatal
Late Neonatal
1−5 months
6−11 months
12−23 months
2−4 years
B
U5MR
Year
Scenario
Estimates up to 2019
Reference
Better
Worse
No Covid
Neonatal
Child
C
Cause of Death
Neonatal
Female
Male
Under 5
Neonatal disorders
Congenital anomalies
Lower respiratory infections
Sudden infant death syndrome
Endocrine, metabolic, blood, and immune disorders
Other neurological disorders
Foreign body
Meningitis
Road injuries
Other malignant neoplasms
Interpersonal violence
Cardiomyopathy and myocarditis
Drowning
Other unspecified infectious diseases
Year
D
Serbia: NN ratio to SPF: 0.95 // U5 ratio to SPF: 0.88
Deaths per 1000 live births
HAQ Index
Serbia
SDI quintile
Low SDI
Low−middle SDI
Middle SDI
High−middle SDI
High SDI
Benchmarks
SDG target
Global Optimum
Survival Potential Frontier

Slovakia

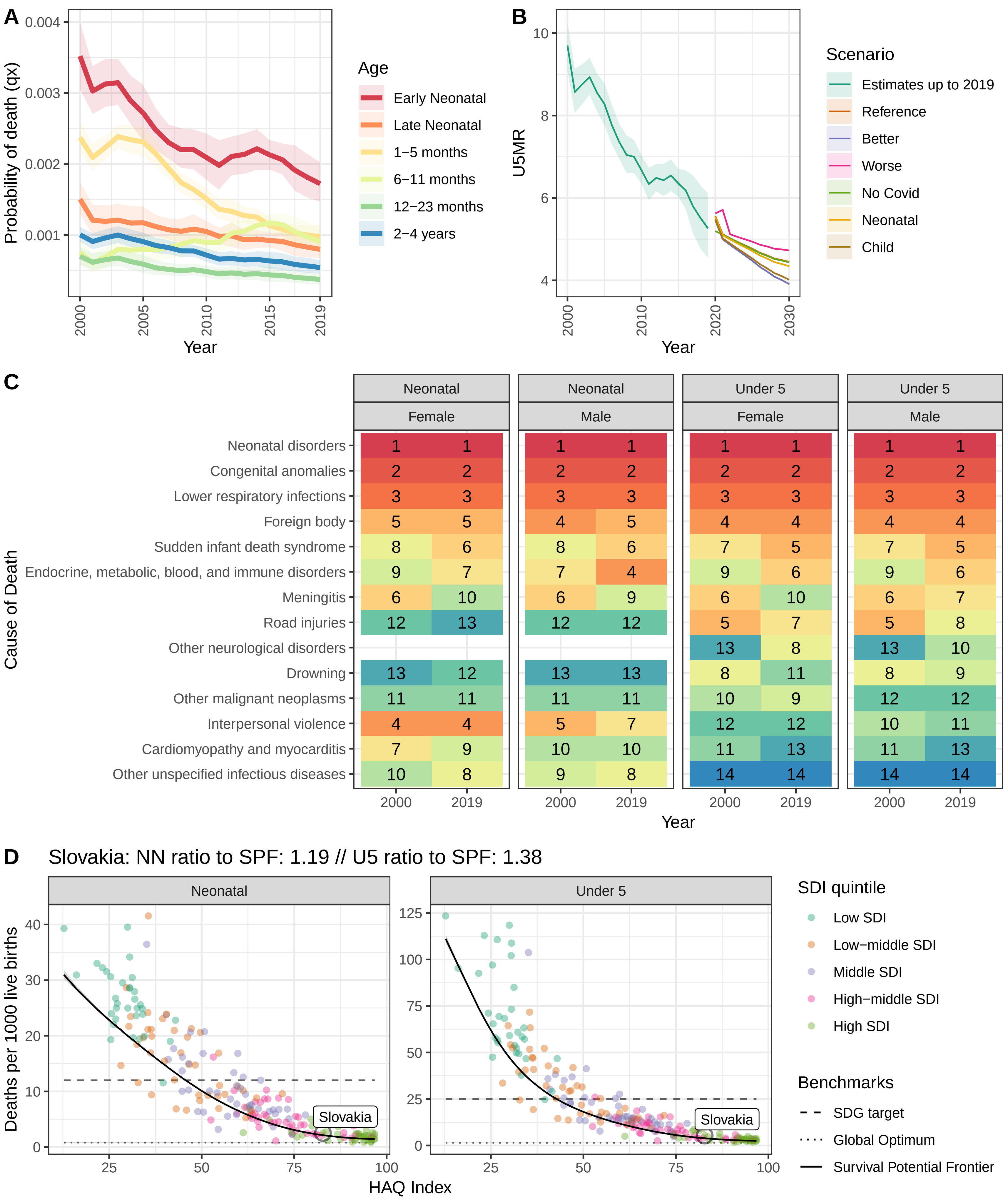
A
Probability of death (qx)
Year
Age
Early Neonatal
Late Neonatal
1−5 months
6−11 months
12−23 months
2−4 years
B
U5MR
Scenario
Estimates up to 2019
Reference
Better
Worse
No Covid
Neonatal
Child
C
Cause of Death
Neonatal Female
Neonatal Male
Under 5 Female
Under 5 Male
Neonatal disorders 1 1 1 1 1 1 1 1
Congenital anomalies 2 2 2 2 2 2 2 2
Lower respiratory infections 3 3 3 3 3 3 3 3
Foreign body 5 5 4 5 4 4 4 4
Sudden infant death syndrome 8 6 8 6 7 5 7 5
Endocrine, metabolic, blood, and immune disorders 9 7 7 4 9 6 9 6
Meningitis 6 10 6 9 6 10 6 7
Road injuries 12 13 12 12 5 7 5 8
Other neurological disorders 13 8 13 10
Drowning 13 12 13 13 8 11 8 9
Other malignant neoplasms 11 11 11 11 10 9 12 12
Interpersonal violence 4 4 5 7 12 12 10 11
Cardiomyopathy and myocarditis 7 9 10 10 11 13 11 13
Other unspecified infectious diseases 10 8 9 8 14 14 14 14
2000 2019
Year
D
Slovakia: NN ratio to SPF: 1.19 // U5 ratio to SPF: 1.38
Neonatal
Under 5
Deaths per 1000 live births
HAQ Index
Slovakia
SDI quintile
Low SDI
Low−middle SDI
Middle SDI
High−middle SDI
High SDI
Benchmarks
SDG target
Global Optimum
Survival Potential Frontier

Slovenia

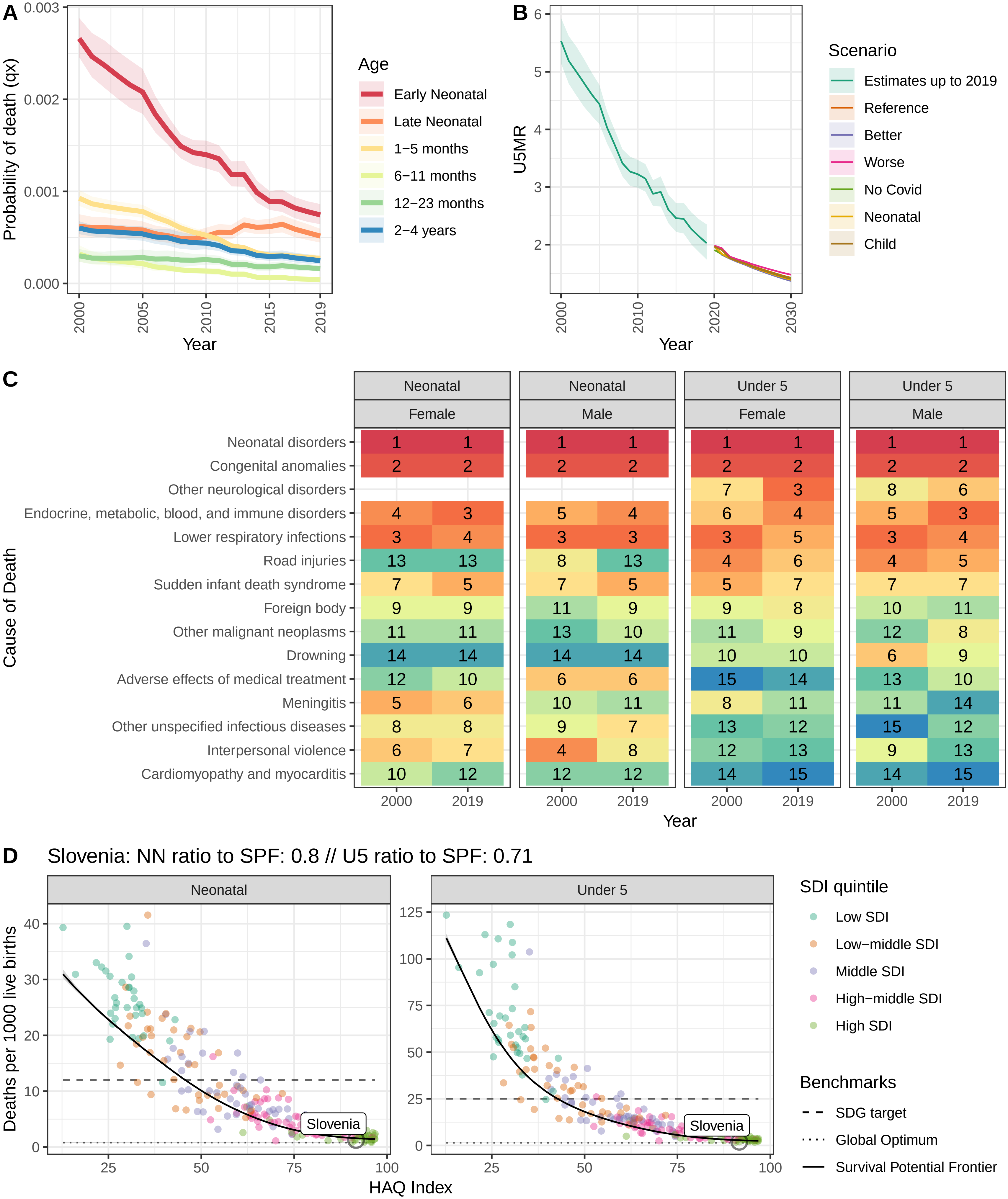
A
Probability of death (qx)
Year
Age
Early Neonatal
Late Neonatal
1−5 months
6−11 months
12−23 months
2−4 years
B
U5MR
Year
Scenario
Estimates up to 2019
Reference
Better
Worse
No Covid
Neonatal
Child
C
Neonatal
Female
Neonatal
Male
Under 5
Female
Under 5
Male
Cause of Death
Neonatal disorders 1 1 1 1 1 1 1 1
Congenital anomalies 2 2 2 2 2 2 2 2
Other neurological disorders 7 3 8 6
Endocrine, metabolic, blood, and immune disorders 4 3 5 4 6 4 5 3
Lower respiratory infections 3 4 3 3 3 5 3 4
Road injuries 13 13 8 13 4 6 4 5
Sudden infant death syndrome 7 5 7 5 5 7 7 7
Foreign body 9 9 11 9 9 8 10 11
Other malignant neoplasms 11 11 13 10 11 9 12 8
Drowning 14 14 14 14 10 10 6 9
Adverse effects of medical treatment 12 10 6 6 15 14 13 10
Meningitis 5 6 10 11 8 11 11 14
Other unspecified infectious diseases 8 8 9 7 13 12 15 12
Interpersonal violence 6 7 4 8 12 13 9 13
Cardiomyopathy and myocarditis 10 12 12 12 14 15 14 15
2000 2019 2000 2019 2000 2019 2000 2019
Year
D
Slovenia: NN ratio to SPF: 0.8 // U5 ratio to SPF: 0.71
Neonatal
Under 5
Deaths per 1000 live births
HAQ Index
Slovenia
Slovenia
SDI quintile
Low SDI
Low−middle SDI
Middle SDI
High−middle SDI
High SDI
Benchmarks
SDG target
Global Optimum
Survival Potential Frontier

Belarus

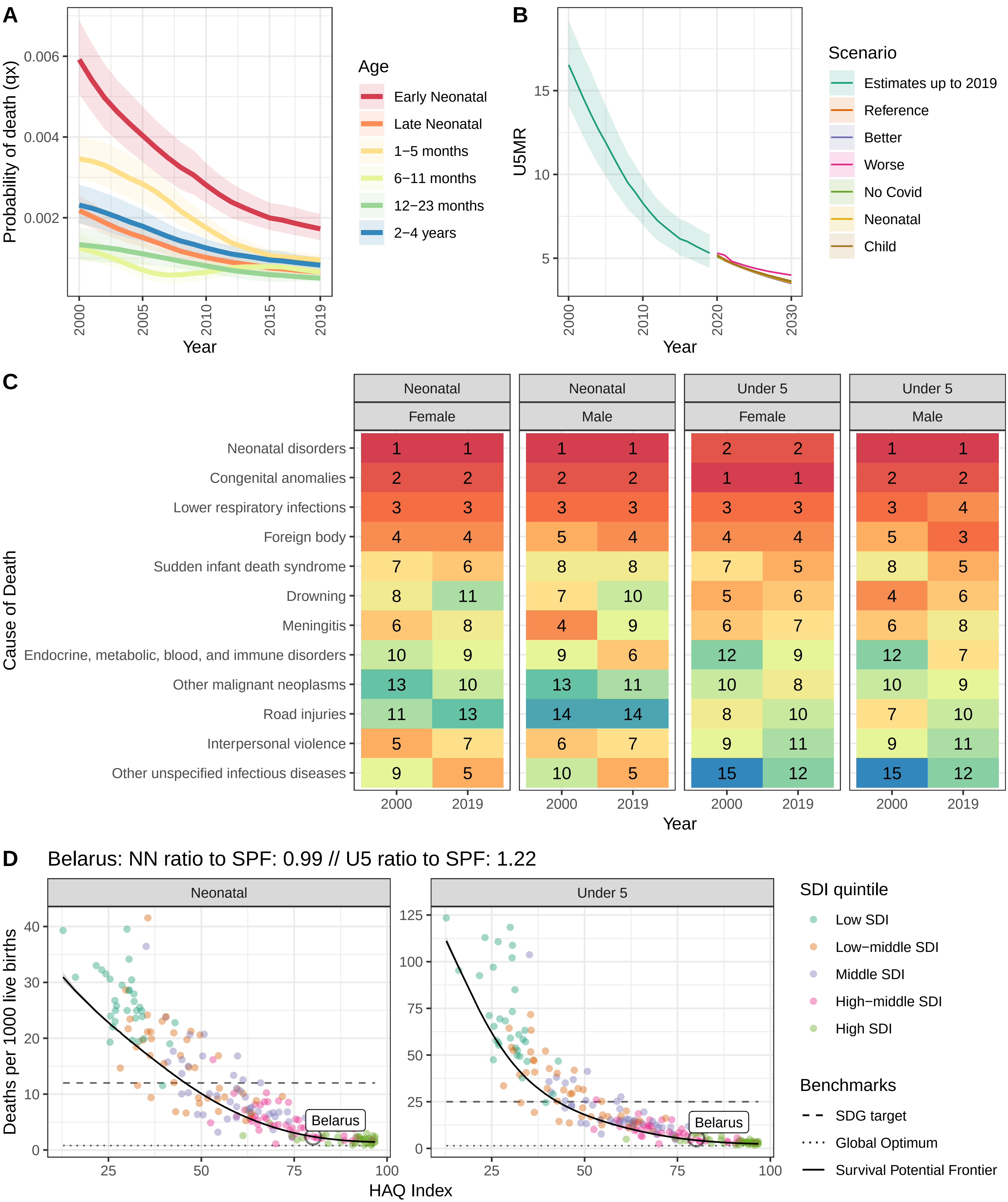
A
Probability of death (qx)
Year
Age
Early Neonatal
Late Neonatal
1−5 months
6−11 months
12−23 months
2−4 years
B
U5MR
Year
Scenario
Estimates up to 2019
Reference
Better
Worse
No Covid
Neonatal
Child
C
Cause of Death
Neonatal disorders
Congenital anomalies
Lower respiratory infections
Foreign body
Sudden infant death syndrome
Drowning
Meningitis
Endocrine, metabolic, blood, and immune disorders
Other malignant neoplasms
Road injuries
Interpersonal violence
Other unspecified infectious diseases
Neonatal Female
Neonatal Male
Under 5 Female
Under 5 Male
Year
D
Belarus: NN ratio to SPF: 0.99 // U5 ratio to SPF: 1.22
Neonatal
Under 5
Deaths per 1000 live births
HAQ Index
Belarus
SDI quintile
Low SDI
Low−middle SDI
Middle SDI
High−middle SDI
High SDI
Benchmarks
SDG target
Global Optimum
Survival Potential Frontier

Estonia

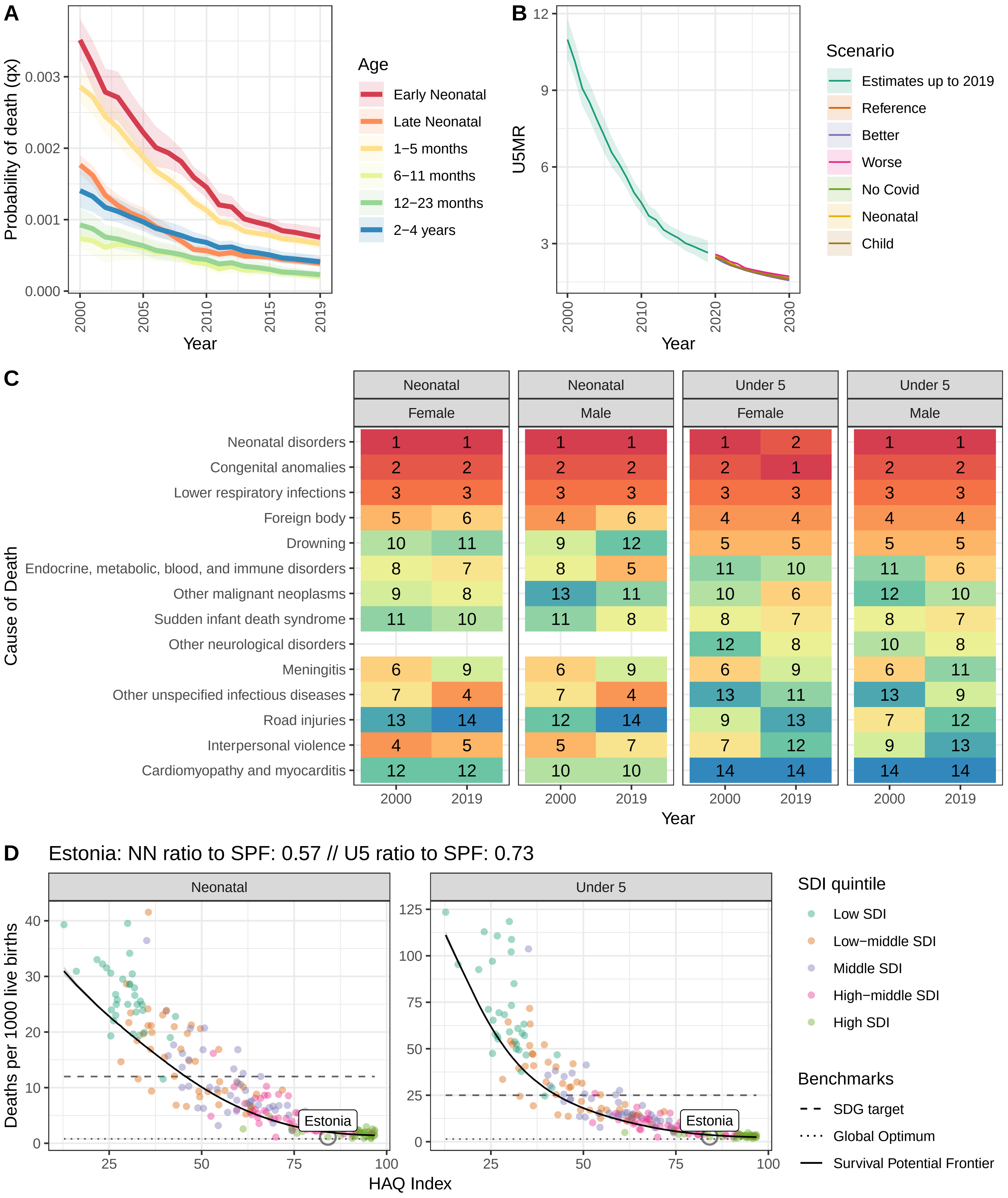

A
Probability of death (qx)
0.003
0.002
0.001
0.000
2000
2005
2010
2015
2019
Year
Age
Early Neonatal
Late Neonatal
1−5 months
6−11 months
12−23 months
2−4 years
B
U5MR
12
9
6
3
2000
2010
2020
2030
Year
Scenario
Estimates up to 2019
Reference
Better
Worse
No Covid
Neonatal
Child
C
Neonatal Female
Neonatal Male
Under 5 Female
Under 5 Male
Cause of Death
Neonatal disorders 1 1 1 1 1 2 1 1
Congenital anomalies 2 2 2 2 2 1 2 2
Lower respiratory infections 3 3 3 3 3 3 3 3
Foreign body 5 6 4 6 4 4 4 4
Drowning 10 11 9 12 5 5 5 5
Endocrine, metabolic, blood, and immune disorders 8 7 8 5 11 10 11 6
Other malignant neoplasms 9 8 13 11 10 6 12 10
Sudden infant death syndrome 11 10 11 8 8 7 8 7
Other neurological disorders 12 8 10 8
Meningitis 6 9 6 9 6 9 6 11
Other unspecified infectious diseases 7 4 7 4 13 11 13 9
Road injuries 13 14 12 14 9 13 7 12
Interpersonal violence 4 5 5 7 7 12 9 13
Cardiomyopathy and myocarditis 12 12 10 10 14 14 14 14
2000
2019
Year
D
Estonia: NN ratio to SPF: 0.57 // U5 ratio to SPF: 0.73
Neonatal
Under 5
Deaths per 1000 live births
40
30
20
10
0
125
100
75
50
25
0
Estonia
HAQ Index
SDI quintile
Low SDI
Low−middle SDI
Middle SDI
High−middle SDI
High SDI
Benchmarks
SDG target
Global Optimum
Survival Potential Frontier

Latvia

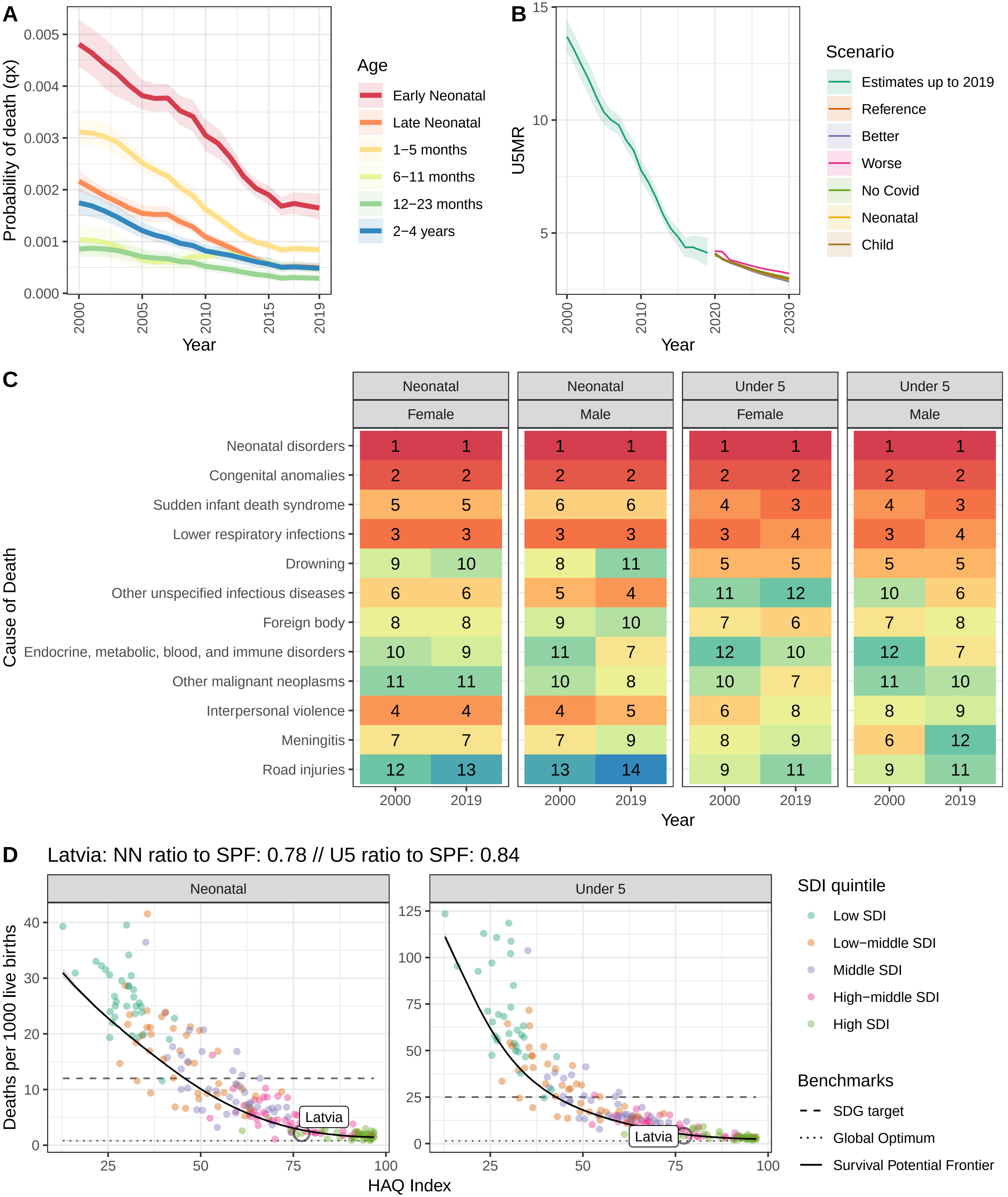
A
Probability of death (qx)
Year
Age
Early Neonatal
Late Neonatal
1−5 months
6−11 months
12−23 months
2−4 years
B
U5MR
Year
Scenario
Estimates up to 2019
Reference
Better
Worse
No Covid
Neonatal
Child
C
Cause of Death
Neonatal
Female
Male
Under 5
Neonatal disorders 1 1 1 1 1 1 1 1
Congenital anomalies 2 2 2 2 2 2 2 2
Sudden infant death syndrome 5 5 6 6 4 3 4 3
Lower respiratory infections 3 3 3 3 3 4 3 4
Drowning 9 10 8 11 5 5 5 5
Other unspecified infectious diseases 6 6 5 4 11 12 10 6
Foreign body 8 8 9 10 7 6 7 8
Endocrine, metabolic, blood, and immune disorders 10 9 11 7 12 10 12 7
Other malignant neoplasms 11 11 10 8 10 7 11 10
Interpersonal violence 4 4 4 5 6 8 8 9
Meningitis 7 7 7 9 8 9 6 12
Road injuries 12 13 13 14 9 11 9 11
2000
2019
Year
D
Latvia: NN ratio to SPF: 0.78 // U5 ratio to SPF: 0.84
Neonatal
Under 5
Deaths per 1000 live births
HAQ Index
Latvia
SDI quintile
Low SDI
Low−middle SDI
Middle SDI
High−middle SDI
High SDI
Benchmarks
SDG target
Global Optimum
Survival Potential Frontier

China

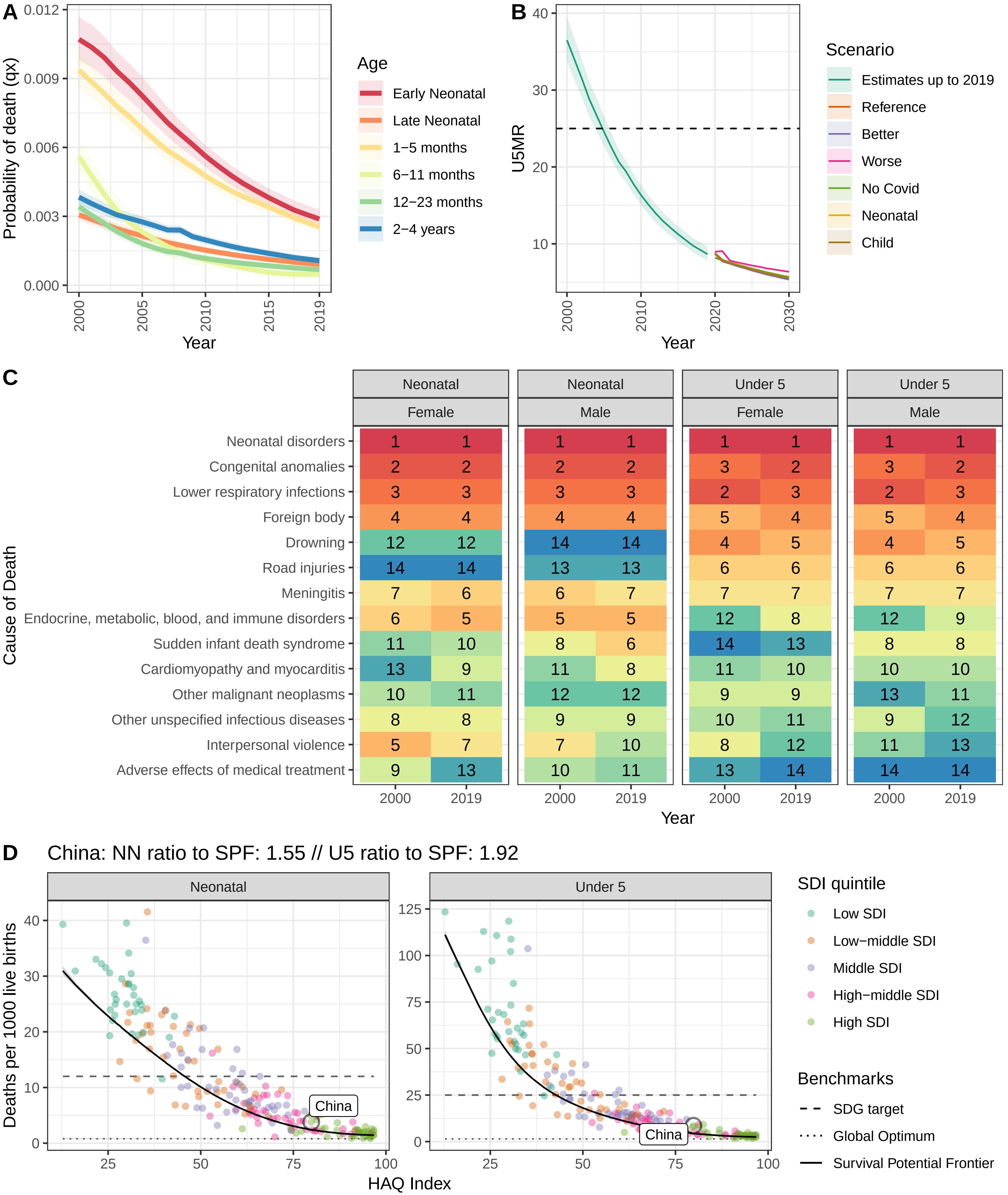

A
Probability of death (qx)
Year
Age
Early Neonatal
Late Neonatal
1−5 months
6−11 months
12−23 months
2−4 years
B
U5MR
Year
Scenario
Estimates up to 2019
Reference
Better
Worse
No Covid
Neonatal
Child
C
Cause of Death
Neonatal Female
Neonatal Male
Under 5 Female
Under 5 Male
Neonatal disorders 1 1 1 1 1 1 1 1
Congenital anomalies 2 2 2 2 3 2 3 2
Lower respiratory infections 3 3 3 3 2 3 2 3
Foreign body 4 4 4 4 5 4 5 4
Drowning 12 12 14 14 4 5 4 5
Road injuries 14 14 13 13 6 6 6 6
Meningitis 7 6 6 7 7 7 7 7
Endocrine, metabolic, blood, and immune disorders 6 5 5 5 12 8 12 9
Sudden infant death syndrome 11 10 8 6 14 13 8 8
Cardiomyopathy and myocarditis 13 9 11 8 11 10 10 10
Other malignant neoplasms 10 11 12 12 9 9 13 11
Other unspecified infectious diseases 8 8 9 9 10 11 9 12
Interpersonal violence 5 7 7 10 8 12 11 13
Adverse effects of medical treatment 9 13 10 11 13 14 14 14
2000 2019
Year
D
China: NN ratio to SPF: 1.55 // U5 ratio to SPF: 1.92
Neonatal
Under 5
China
Deaths per 1000 live births
HAQ Index
SDI quintile
Low SDI
Low−middle SDI
Middle SDI
High−middle SDI
High SDI
Benchmarks
SDG target
Global Optimum
Survival Potential Frontier

Lithuania

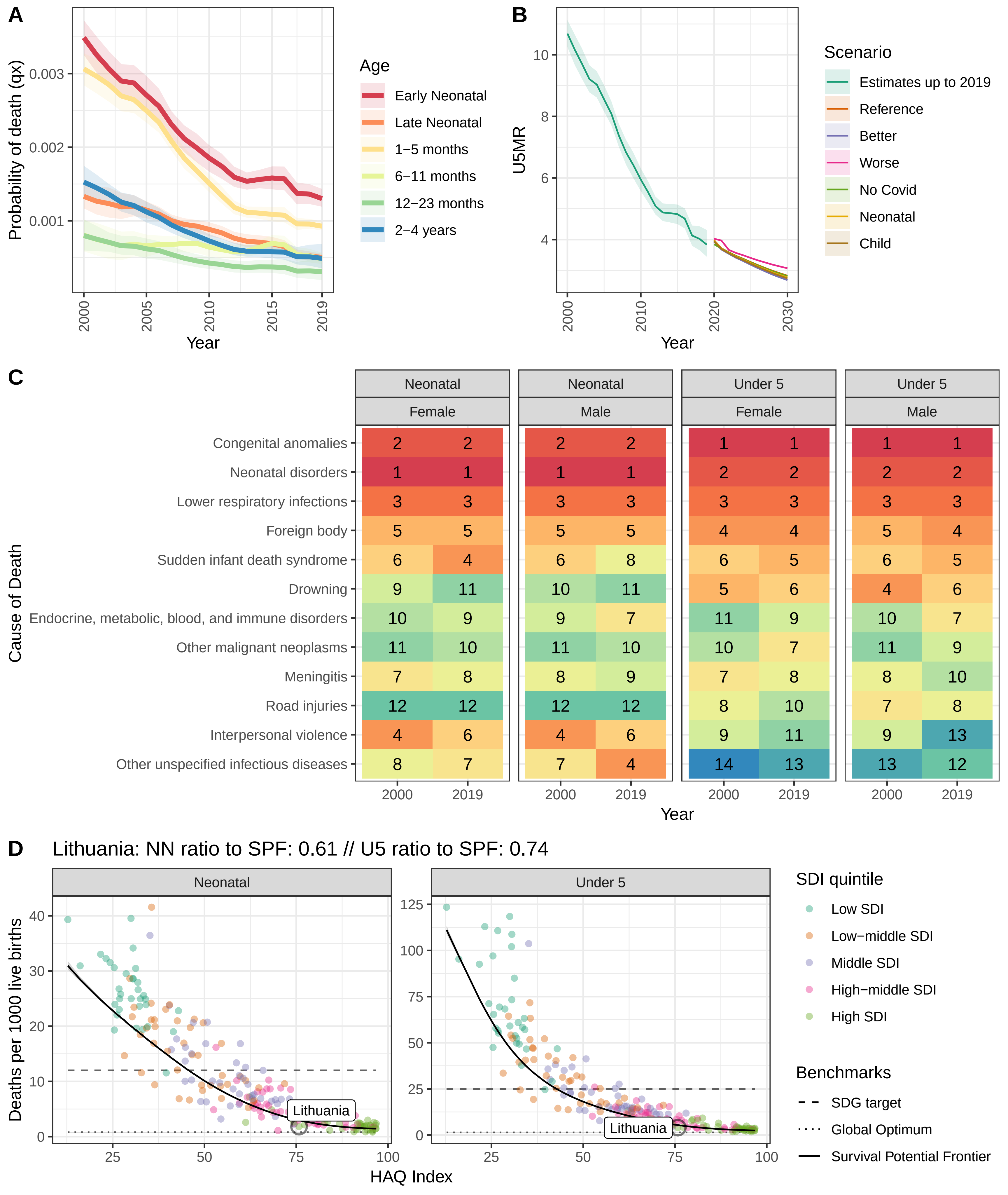

A
Probability of death (qx)
0.003
0.002
0.001
2000
2005
2010
2015
2019
Year
Age
Early Neonatal
Late Neonatal
1−5 months
6−11 months
12−23 months
2−4 years
B
U5MR
10
8
6
4
2000
2010
2020
2030
Year
Scenario
Estimates up to 2019
Reference
Better
Worse
No Covid
Neonatal
Child
C
Cause of Death
Neonatal
Female
Neonatal
Male
Under 5
Female
Under 5
Male
Congenital anomalies 2 2 2 2 1 1 1 1
Neonatal disorders 1 1 1 1 2 2 2 2
Lower respiratory infections 3 3 3 3 3 3 3 3
Foreign body 5 5 5 5 4 4 5 4
Sudden infant death syndrome 6 4 6 8 6 5 6 5
Drowning 9 11 10 11 5 6 4 6
Endocrine, metabolic, blood, and immune disorders 10 9 9 7 11 9 10 7
Other malignant neoplasms 11 10 11 10 10 7 11 9
Meningitis 7 8 8 9 7 8 8 10
Road injuries 12 12 12 12 8 10 7 8
Interpersonal violence 4 6 4 6 9 11 9 13
Other unspecified infectious diseases 8 7 7 4 14 13 13 12
2000
2019
Year
D
Lithuania: NN ratio to SPF: 0.61 // U5 ratio to SPF: 0.74
Neonatal
Under 5
Deaths per 1000 live births
40
30
20
10
0
125
100
75
50
25
0
25
50
75
100
Lithuania
HAQ Index
SDI quintile
Low SDI
Low−middle SDI
Middle SDI
High−middle SDI
High SDI
Benchmarks
SDG target
Global Optimum
Survival Potential Frontier

Republic of Moldova

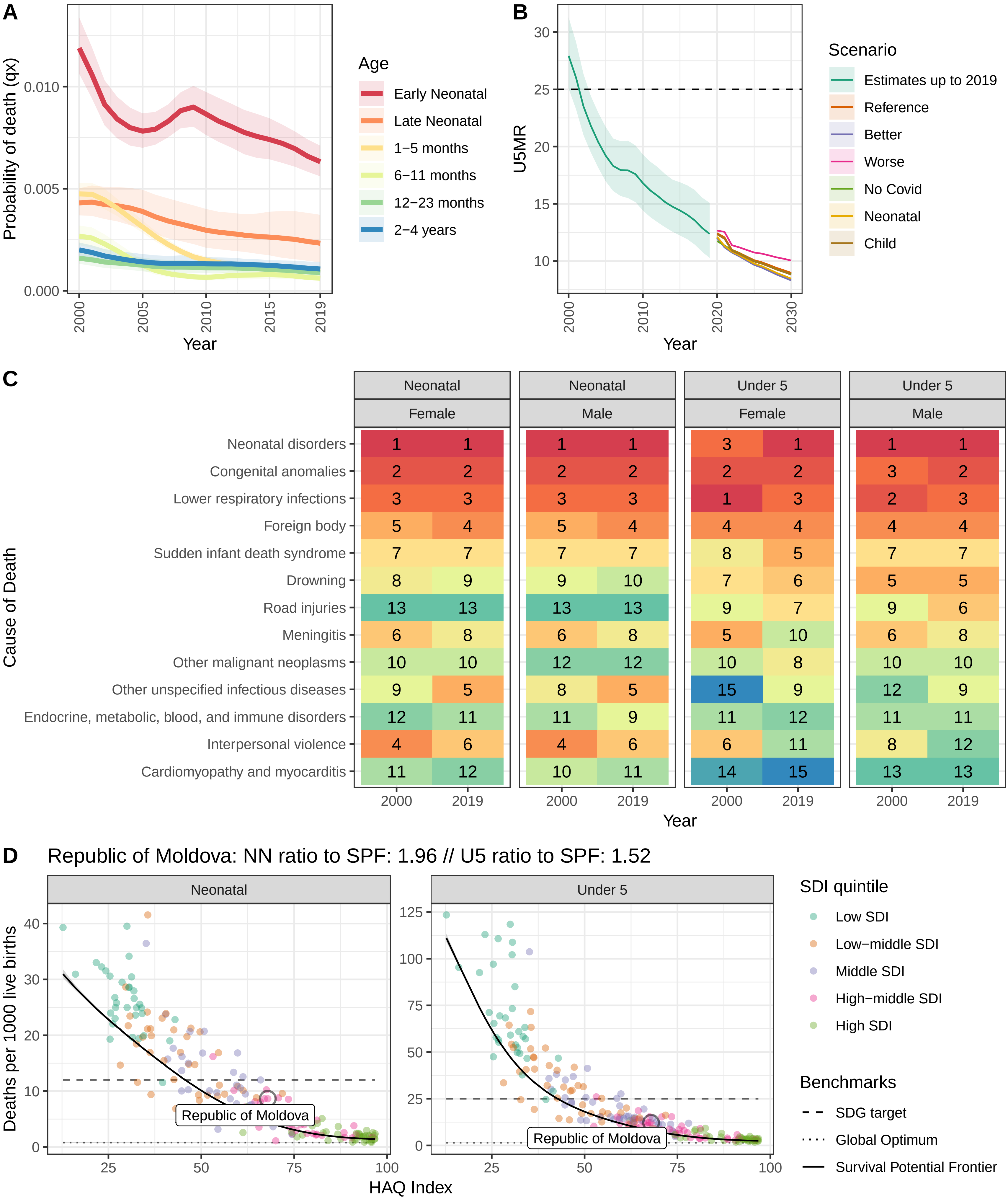

Russian Federation

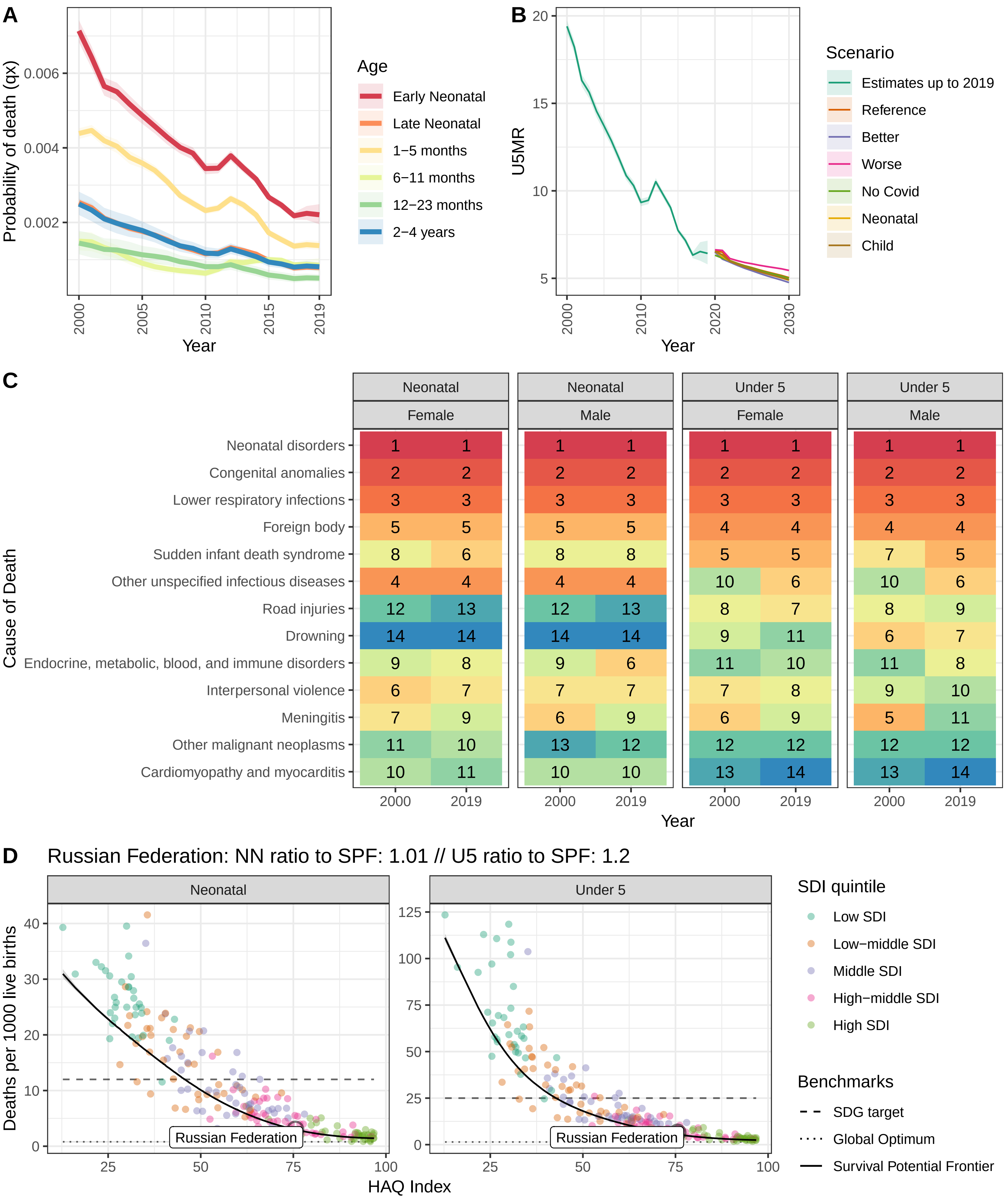

Ukraine

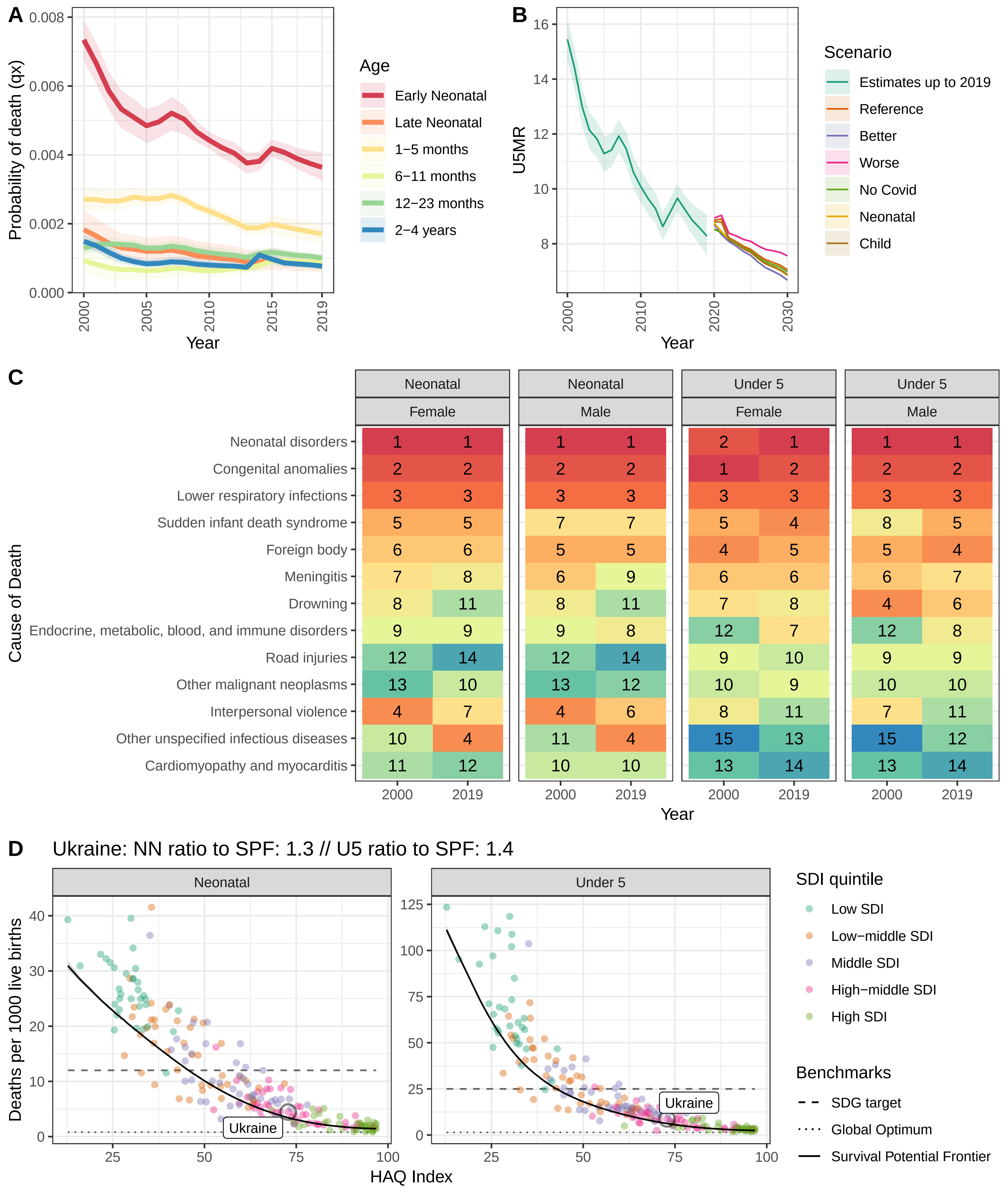

A
Probability of death (qx)
0.008
0.006
0.004
0.002
0.000
2000
2005
2010
2015
2019
Year
Age
Early Neonatal
Late Neonatal
1−5 months
6−11 months
12−23 months
2−4 years
B
U5MR
16
14
12
10
8
2000
2010
2020
2030
Year
Scenario
Estimates up to 2019
Reference
Better
Worse
No Covid
Neonatal
Child
C
Neonatal Female
Neonatal Male
Under 5 Female
Under 5 Male
Cause of Death
Neonatal disorders 1 1 1 1 2 1 1 1
Congenital anomalies 2 2 2 2 1 2 2 2
Lower respiratory infections 3 3 3 3 3 3 3 3
Sudden infant death syndrome 5 5 7 7 5 4 8 5
Foreign body 6 6 5 5 4 5 5 4
Meningitis 7 8 6 9 6 6 6 7
Drowning 8 11 8 11 7 8 4 6
Endocrine, metabolic, blood, and immune disorders 9 9 9 8 12 7 12 8
Road injuries 12 14 12 14 9 10 9 9
Other malignant neoplasms 13 10 13 12 10 9 10 10
Interpersonal violence 4 7 4 6 8 11 7 11
Other unspecified infectious diseases 10 4 11 4 15 13 15 12
Cardiomyopathy and myocarditis 11 12 10 10 13 14 13 14
2000 2019 2000 2019 2000 2019 2000 2019
Year
D
Ukraine: NN ratio to SPF: 1.3 // U5 ratio to SPF: 1.4
Neonatal
Under 5
Deaths per 1000 live births
40
30
20
10
0
125
100
75
50
25
0
Ukraine
Ukraine
25
50
75
100
HAQ Index
SDI quintile
Low SDI
Low−middle SDI
Middle SDI
High−middle SDI
High SDI
Benchmarks
SDG target
Global Optimum
Survival Potential Frontier

Brunei Darussalam

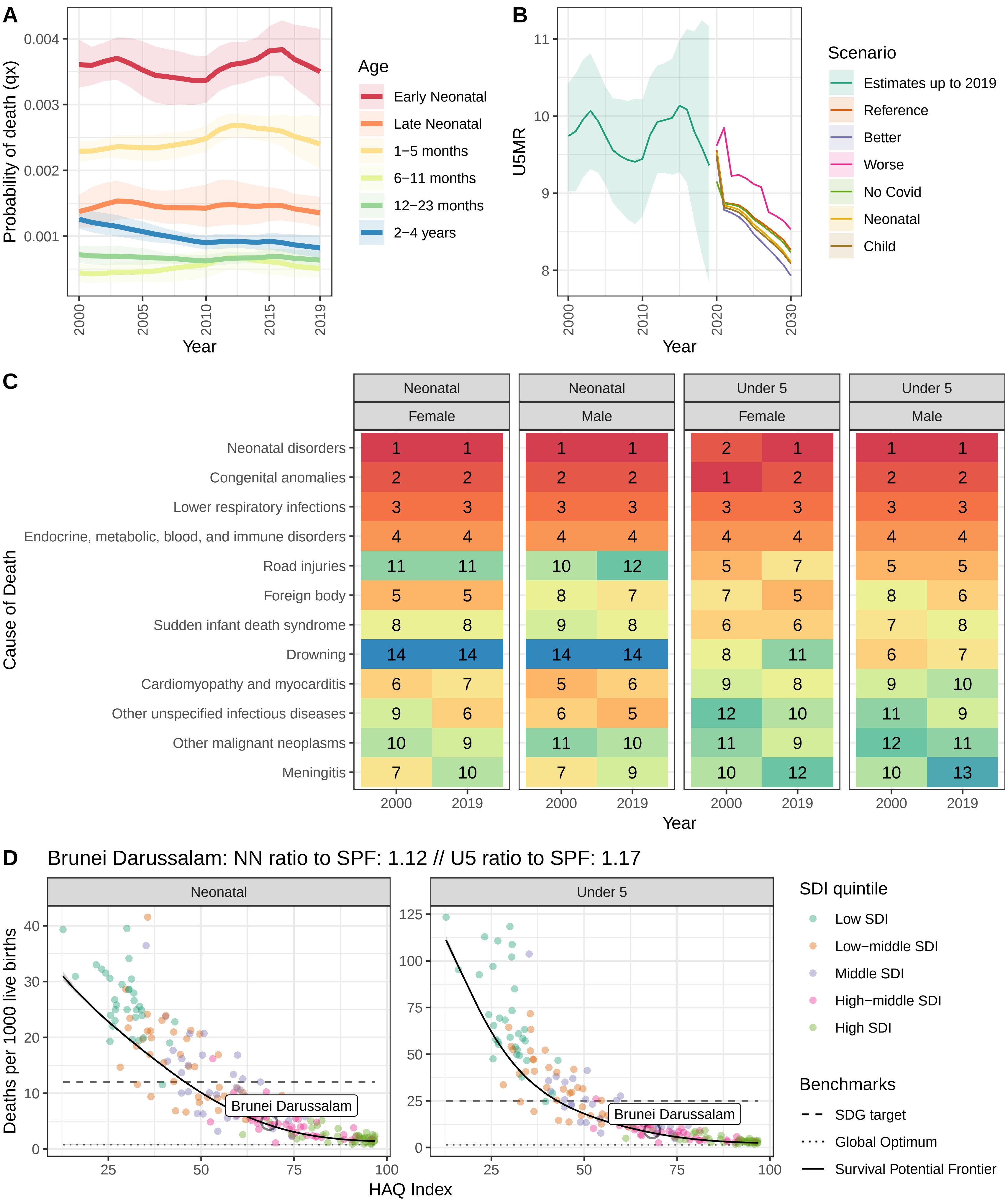

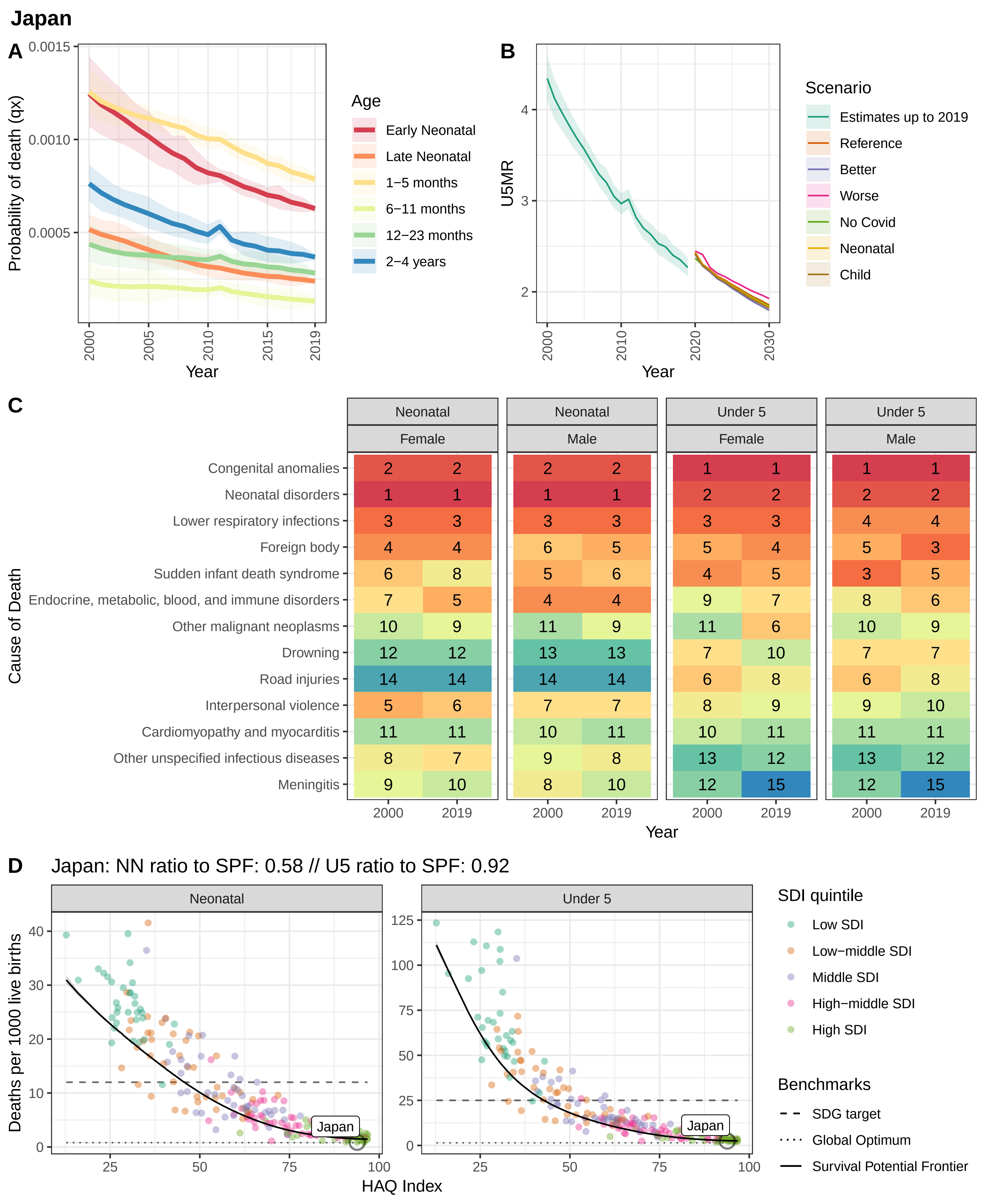

Japan
A
Probability of death (qx)
0.0015
0.0010
0.0005
2000
2005
2010
2015
2019
Year
Age
Early Neonatal
Late Neonatal
1−5 months
6−11 months
12−23 months
2−4 years
B
U5MR
4
3
2
2000
2010
2020
2030
Year
Scenario
Estimates up to 2019
Reference
Better
Worse
No Covid
Neonatal
Child
C
Cause of Death
Neonatal
Female
Neonatal
Male
Under 5
Female
Under 5
Male
Congenital anomalies 2 2 2 2 1 1 1 1
Neonatal disorders 1 1 1 1 2 2 2 2
Lower respiratory infections 3 3 3 3 3 3 4 4
Foreign body 4 4 6 5 5 4 5 3
Sudden infant death syndrome 6 8 5 6 4 5 3 5
Endocrine, metabolic, blood, and immune disorders 7 5 4 4 9 7 8 6
Other malignant neoplasms 10 9 11 9 11 6 10 9
Drowning 12 12 13 13 7 10 7 7
Road injuries 14 14 14 14 6 8 6 8
Interpersonal violence 5 6 7 7 8 9 9 10
Cardiomyopathy and myocarditis 11 11 10 11 10 11 11 11
Other unspecified infectious diseases 8 7 9 8 13 12 13 12
Meningitis 9 10 8 10 12 15 12 15
2000
2019
Year
D
Japan: NN ratio to SPF: 0.58 // U5 ratio to SPF: 0.92
Neonatal
Under 5
Deaths per 1000 live births
40
30
20
10
0
125
100
75
50
25
0
Japan
Japan
25
50
75
100
HAQ Index
SDI quintile
Low SDI
Low−middle SDI
Middle SDI
High−middle SDI
High SDI
Benchmarks
SDG target
Global Optimum
Survival Potential Frontier

Republic of Korea

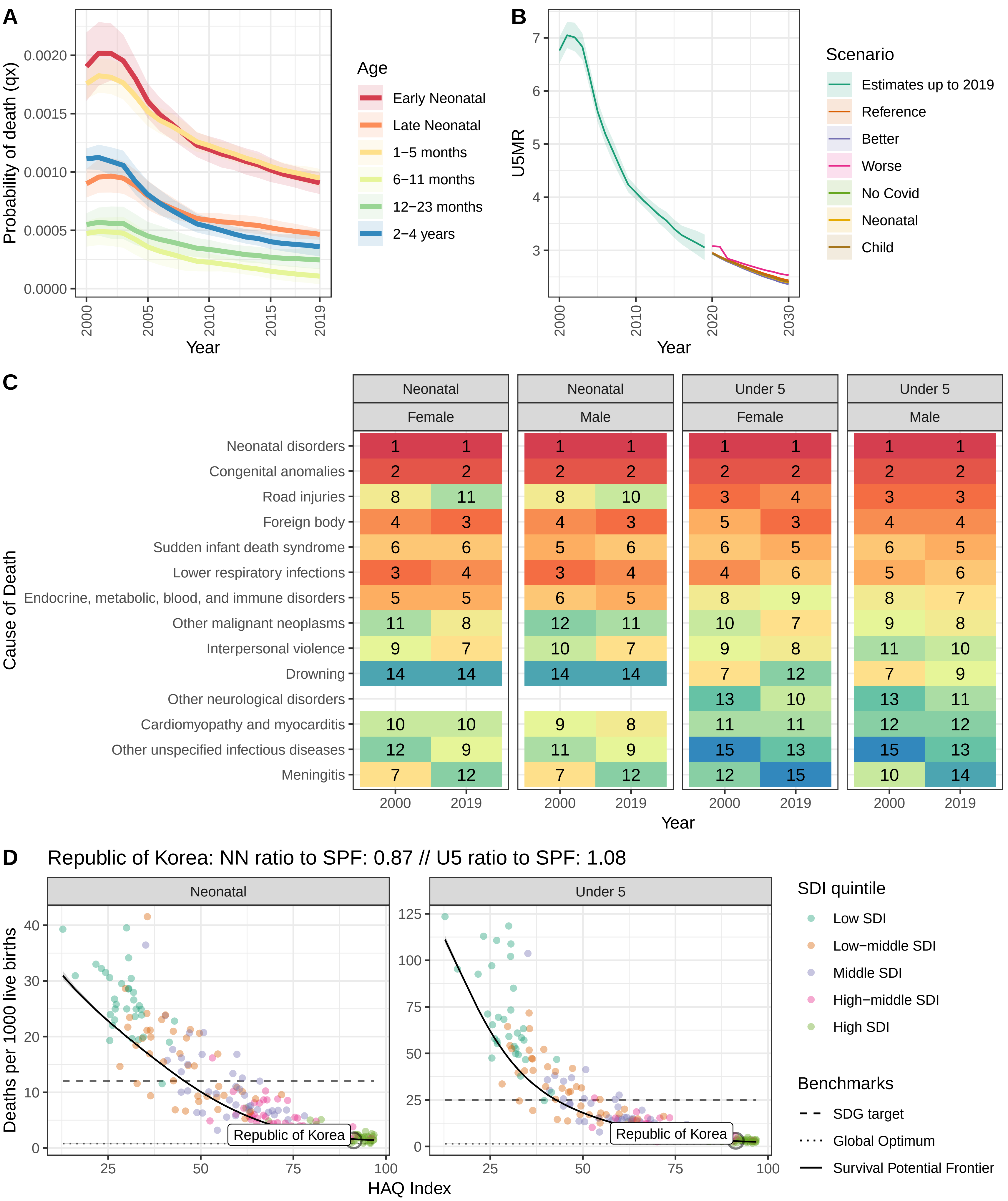

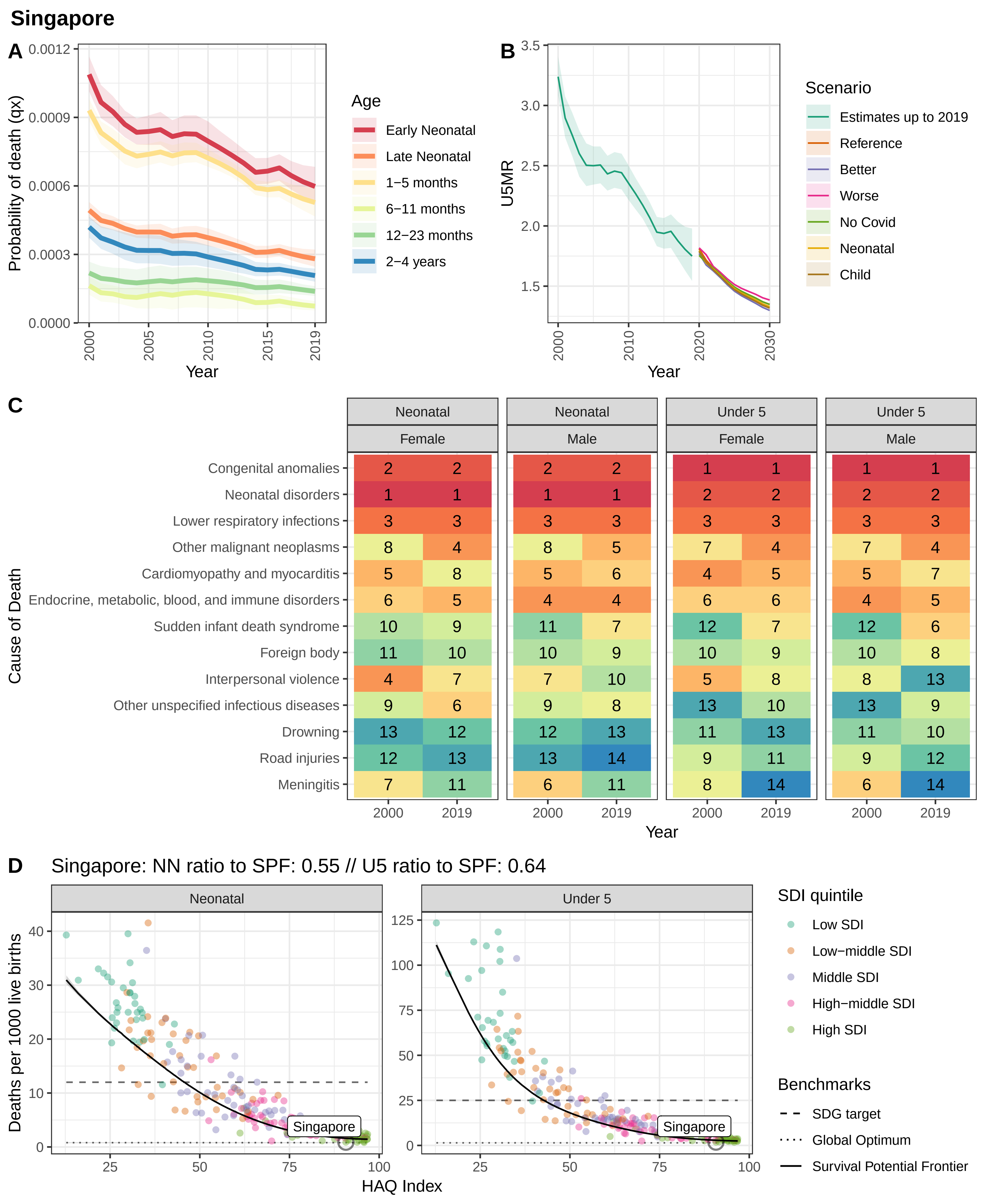

Singapore
A
Probability of death (qx)
0.0012
0.0009
0.0006
0.0003
0.0000
2000
2005
2010
2015
2019
Year
Age
Early Neonatal
Late Neonatal
1−5 months
6−11 months
12−23 months
2−4 years
B
U5MR
3.5
3.0
2.5
2.0
1.5
2000
2010
2020
2030
Year
Scenario
Estimates up to 2019
Reference
Better
Worse
No Covid
Neonatal
Child
C
Cause of Death
Neonatal Female
Neonatal Male
Under 5 Female
Under 5 Male
Congenital anomalies 2 2 2 2 1 1 1 1
Neonatal disorders 1 1 1 1 2 2 2 2
Lower respiratory infections 3 3 3 3 3 3 3 3
Other malignant neoplasms 8 4 8 5 7 4 7 4
Cardiomyopathy and myocarditis 5 8 5 6 4 5 5 7
Endocrine, metabolic, blood, and immune disorders 6 5 4 4 6 6 4 5
Sudden infant death syndrome 10 9 11 7 12 7 12 6
Foreign body 11 10 10 9 10 9 10 8
Interpersonal violence 4 7 7 10 5 8 8 13
Other unspecified infectious diseases 9 6 9 8 13 10 13 9
Drowning 13 12 12 13 11 13 11 10
Road injuries 12 13 13 14 9 11 9 12
Meningitis 7 11 6 11 8 14 6 14
2000
2019
Year
D
Singapore: NN ratio to SPF: 0.55 // U5 ratio to SPF: 0.64
Neonatal
Under 5
Deaths per 1000 live births
40
30
20
10
0
125
100
75
50
25
0
25
50
75
100
HAQ Index
Singapore
SDI quintile
Low SDI
Low−middle SDI
Middle SDI
High−middle SDI
High SDI
Benchmarks
SDG target
Global Optimum
Survival Potential Frontier

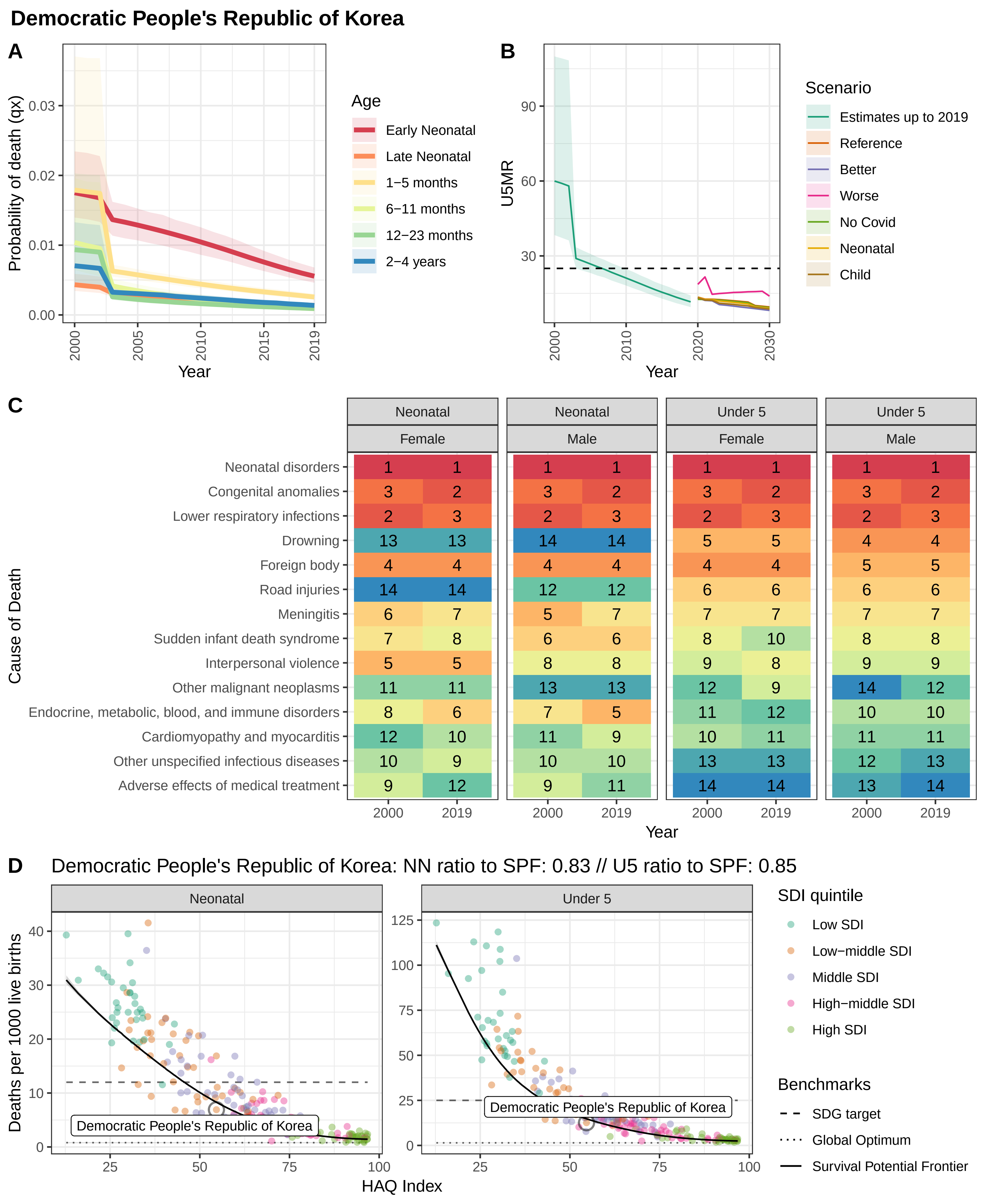

Democratic People's Republic of Korea
A
Probability of death (qx)
0.00
0.01
0.02
0.03
2000
2005
2010
2015
2019
Year
Age
Early Neonatal
Late Neonatal
1−5 months
6−11 months
12−23 months
2−4 years
B
U5MR
30
60
90
2000
2010
2020
2030
Year
Scenario
Estimates up to 2019
Reference
Better
Worse
No Covid
Neonatal
Child
C
Cause of Death
Neonatal Female | Neonatal Male | Under 5 Female | Under 5 Male
2000 2019 | 2000 2019 | 2000 2019 | 2000 2019
Neonatal disorders 1 1 | 1 1 | 1 1 | 1 1
Congenital anomalies 3 2 | 3 2 | 3 2 | 3 2
Lower respiratory infections 2 3 | 2 3 | 2 3 | 2 3
Drowning 13 13 | 14 14 | 5 5 | 4 4
Foreign body 4 4 | 4 4 | 4 4 | 5 5
Road injuries 14 14 | 12 12 | 6 6 | 6 6
Meningitis 6 7 | 5 7 | 7 7 | 7 7
Sudden infant death syndrome 7 8 | 6 6 | 8 10 | 8 8
Interpersonal violence 5 5 | 8 8 | 9 8 | 9 9
Other malignant neoplasms 11 11 | 13 13 | 12 9 | 14 12
Endocrine, metabolic, blood, and immune disorders 8 6 | 7 5 | 11 12 | 10 10
Cardiomyopathy and myocarditis 12 10 | 11 9 | 10 11 | 11 11
Other unspecified infectious diseases 10 9 | 10 10 | 13 13 | 12 13
Adverse effects of medical treatment 9 12 | 9 11 | 14 14 | 13 14
Year
D
Democratic People's Republic of Korea: NN ratio to SPF: 0.83 // U5 ratio to SPF: 0.85
Neonatal
Under 5
Deaths per 1000 live births
0
10
20
30
40
0
25
50
75
100
125
25
50
75
100
HAQ Index
Democratic People's Republic of Korea
Democratic People's Republic of Korea
SDI quintile
Low SDI
Low−middle SDI
Middle SDI
High−middle SDI
High SDI
Benchmarks
SDG target
Global Optimum
Survival Potential Frontier

Australia

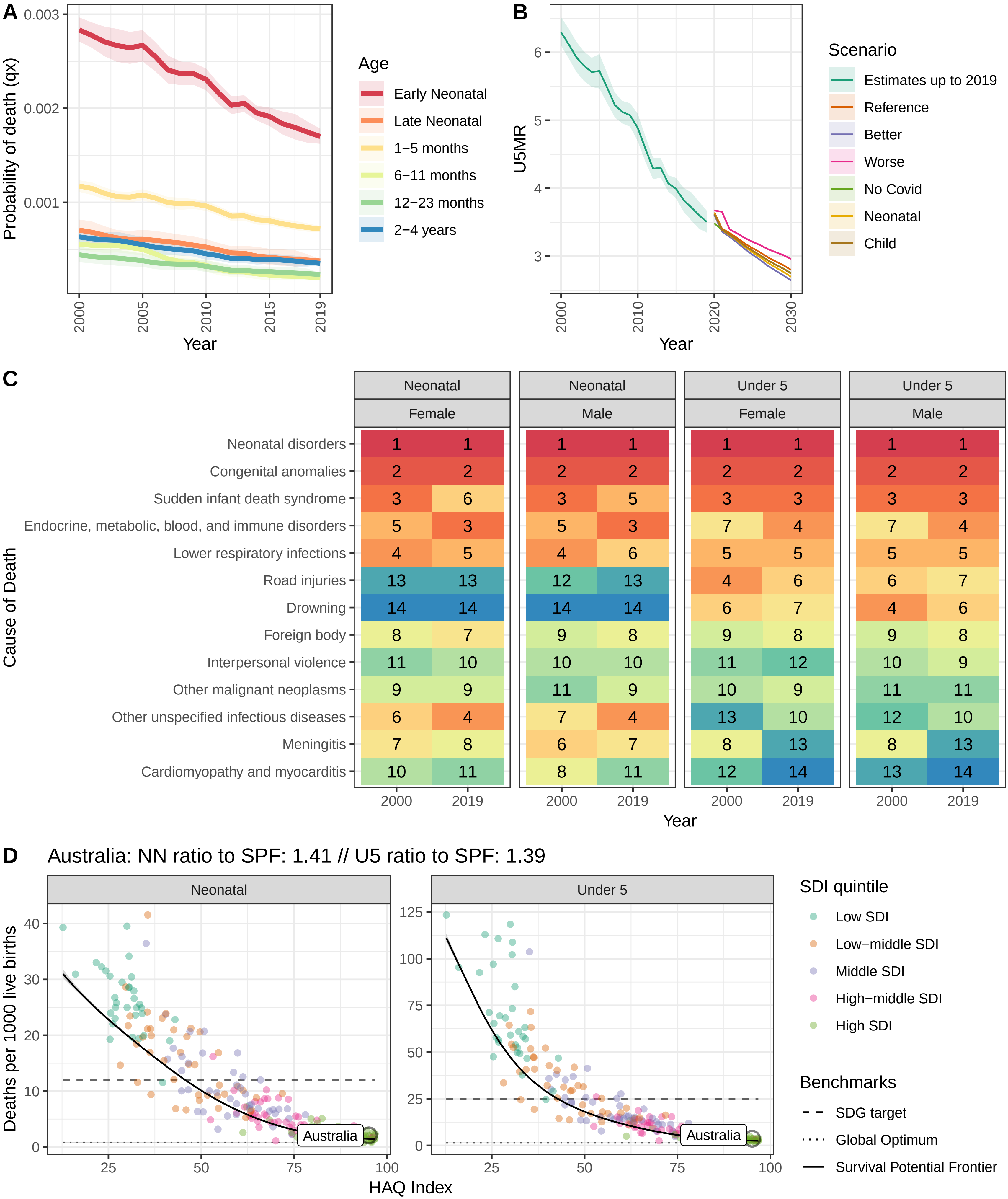
A
Probability of death (qx)
0.003
0.002
0.001
2000
2005
2010
2015
2019
Year
Age
Early Neonatal
Late Neonatal
1−5 months
6−11 months
12−23 months
2−4 years
B
U5MR
6
5
4
3
2000
2010
2020
2030
Year
Scenario
Estimates up to 2019
Reference
Better
Worse
No Covid
Neonatal
Child
C
Cause of Death
Neonatal Female | Neonatal Male | Under 5 Female | Under 5 Male
Neonatal disorders 1 1 | 1 1 | 1 1 | 1 1
Congenital anomalies 2 2 | 2 2 | 2 2 | 2 2
Sudden infant death syndrome 3 6 | 3 5 | 3 3 | 3 3
Endocrine, metabolic, blood, and immune disorders 5 3 | 5 3 | 7 4 | 7 4
Lower respiratory infections 4 5 | 4 6 | 5 5 | 5 5
Road injuries 13 13 | 12 13 | 4 6 | 6 7
Drowning 14 14 | 14 14 | 6 7 | 4 6
Foreign body 8 7 | 9 8 | 9 8 | 9 8
Interpersonal violence 11 10 | 10 10 | 11 12 | 10 9
Other malignant neoplasms 9 9 | 11 9 | 10 9 | 11 11
Other unspecified infectious diseases 6 4 | 7 4 | 13 10 | 12 10
Meningitis 7 8 | 6 7 | 8 13 | 8 13
Cardiomyopathy and myocarditis 10 11 | 8 11 | 12 14 | 13 14
2000 2019
Year
D
Australia: NN ratio to SPF: 1.41 // U5 ratio to SPF: 1.39
Neonatal
Under 5
Deaths per 1000 live births
40
30
20
10
0
125
100
75
50
25
0
25
50
75
100
Australia
HAQ Index
SDI quintile
Low SDI
Low−middle SDI
Middle SDI
High−middle SDI
High SDI
Benchmarks
SDG target
Global Optimum
Survival Potential Frontier

New Zealand

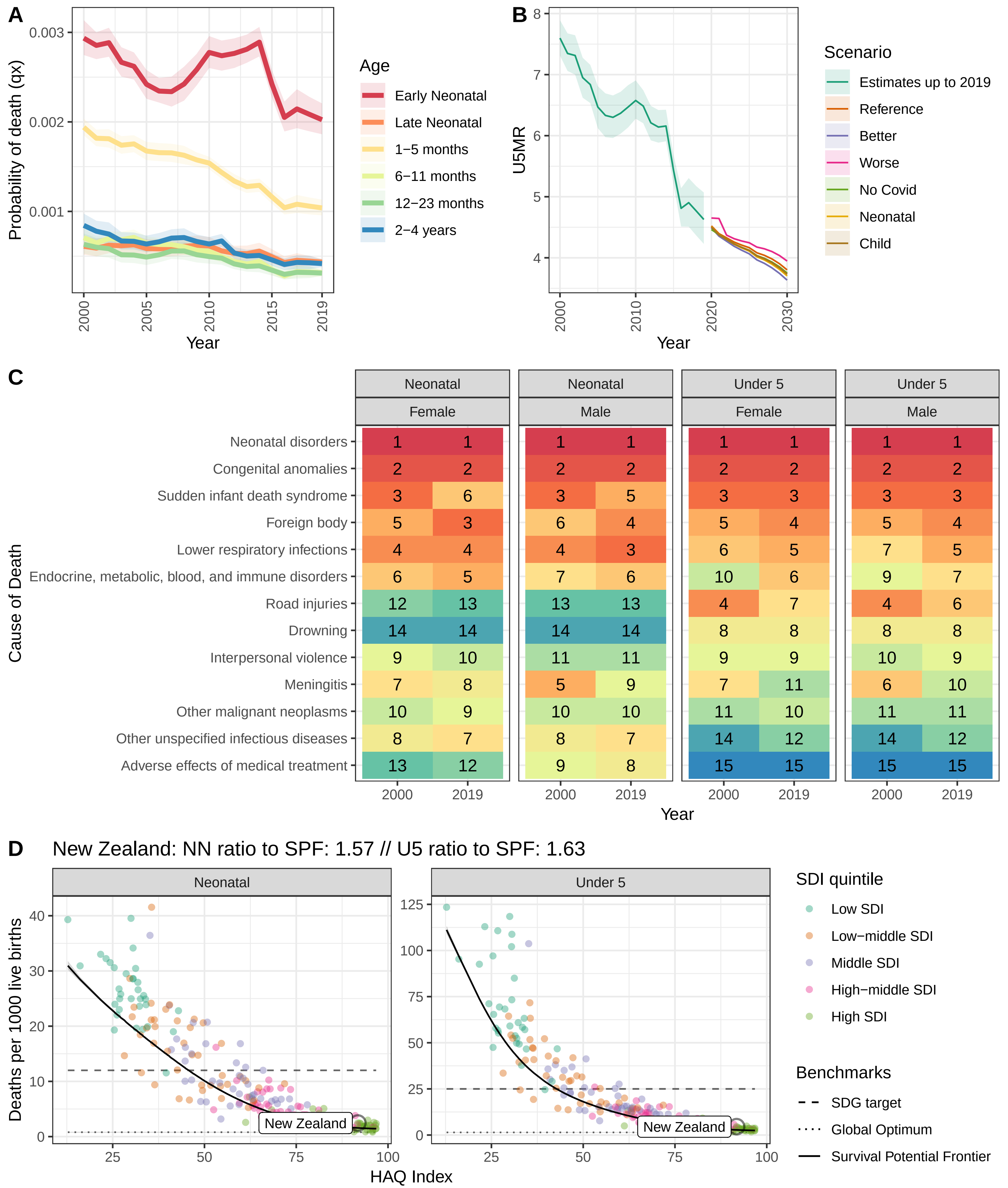
A
Probability of death (qx)
0.003
0.002
0.001
2000
2005
2010
2015
2019
Year
Age
Early Neonatal
Late Neonatal
1−5 months
6−11 months
12−23 months
2−4 years
B
U5MR
8
7
6
5
4
2000
2010
2020
2030
Year
Scenario
Estimates up to 2019
Reference
Better
Worse
No Covid
Neonatal
Child
C
Neonatal
Female
Neonatal
Male
Under 5
Female
Under 5
Male
Cause of Death
Neonatal disorders 1 1 1 1 1 1 1 1
Congenital anomalies 2 2 2 2 2 2 2 2
Sudden infant death syndrome 3 6 3 5 3 3 3 3
Foreign body 5 3 6 4 5 4 5 4
Lower respiratory infections 4 4 4 3 6 5 7 5
Endocrine, metabolic, blood, and immune disorders 6 5 7 6 10 6 9 7
Road injuries 12 13 13 13 4 7 4 6
Drowning 14 14 14 14 8 8 8 8
Interpersonal violence 9 10 11 11 9 9 10 9
Meningitis 7 8 5 9 7 11 6 10
Other malignant neoplasms 10 9 10 10 11 10 11 11
Other unspecified infectious diseases 8 7 8 7 14 12 14 12
Adverse effects of medical treatment 13 12 9 8 15 15 15 15
2000
2019
Year
D
New Zealand: NN ratio to SPF: 1.57 // U5 ratio to SPF: 1.63
Neonatal
Under 5
Deaths per 1000 live births
40
30
20
10
0
125
100
75
50
25
New Zealand
New Zealand
HAQ Index
SDI quintile
Low SDI
Low−middle SDI
Middle SDI
High−middle SDI
High SDI
Benchmarks
SDG target
Global Optimum
Survival Potential Frontier

Andorra

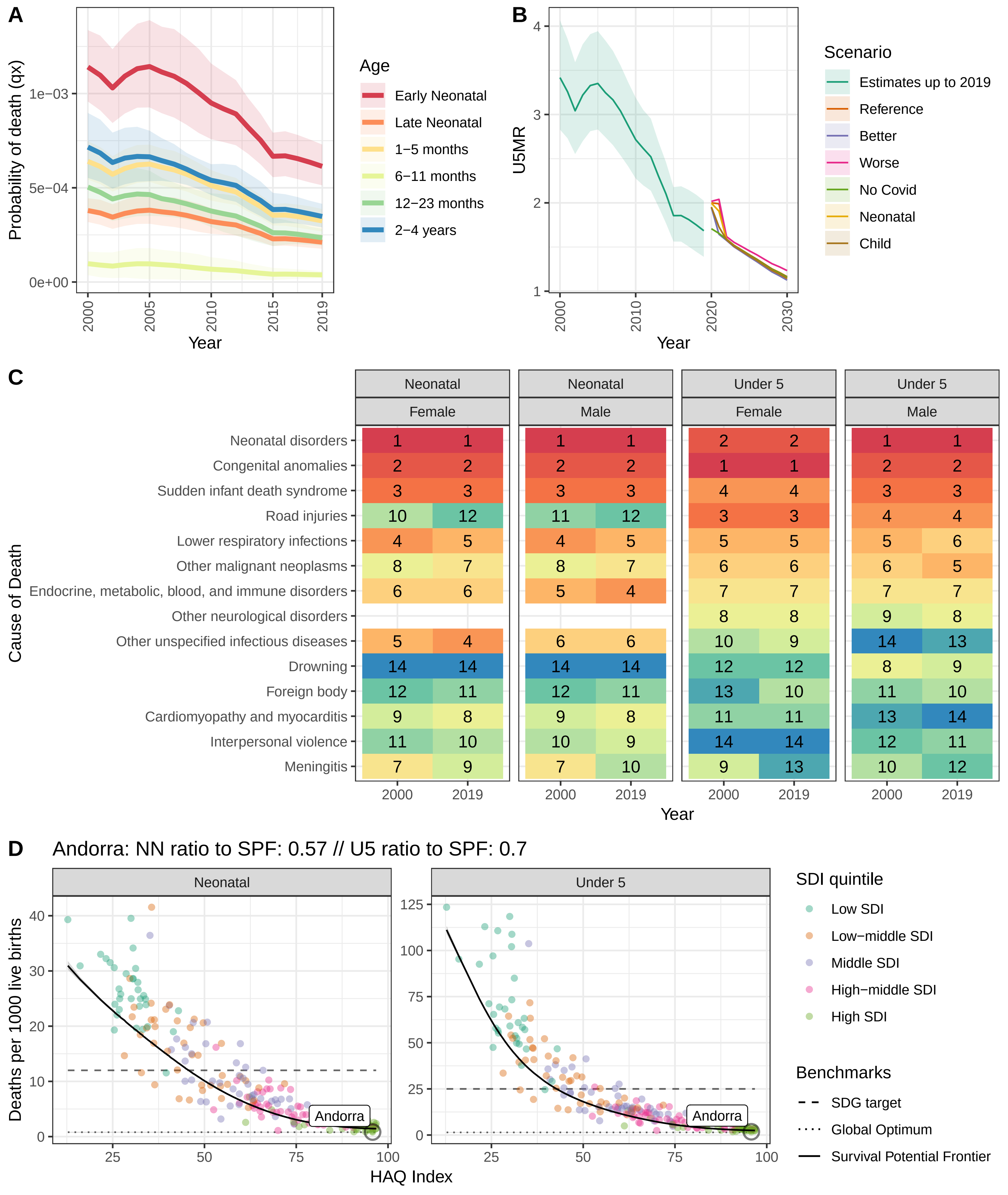

A
Probability of death (qx)
1e−03
5e−04
0e+00
2000
2005
2010
2015
2019
Year
Age
Early Neonatal
Late Neonatal
1−5 months
6−11 months
12−23 months
2−4 years
B
U5MR
4
3
2
1
2000
2010
2020
2030
Year
Scenario
Estimates up to 2019
Reference
Better
Worse
No Covid
Neonatal
Child
C
Cause of Death
Neonatal
Female
Neonatal
Male
Under 5
Female
Under 5
Male
Neonatal disorders 1 1 1 1 2 2 1 1
Congenital anomalies 2 2 2 2 1 1 2 2
Sudden infant death syndrome 3 3 3 3 4 4 3 3
Road injuries 10 12 11 12 3 3 4 4
Lower respiratory infections 4 5 4 5 5 5 5 6
Other malignant neoplasms 8 7 8 7 6 6 6 5
Endocrine, metabolic, blood, and immune disorders 6 6 5 4 7 7 7 7
Other neurological disorders 8 8 9 8
Other unspecified infectious diseases 5 4 6 6 10 9 14 13
Drowning 14 14 14 14 12 12 8 9
Foreign body 12 11 12 11 13 10 11 10
Cardiomyopathy and myocarditis 9 8 9 8 11 11 13 14
Interpersonal violence 11 10 10 9 14 14 12 11
Meningitis 7 9 7 10 9 13 10 12
2000
2019
Year
D
Andorra: NN ratio to SPF: 0.57 // U5 ratio to SPF: 0.7
Neonatal
Under 5
Deaths per 1000 live births
40
30
20
10
0
125
100
75
50
25
0
25
50
75
100
HAQ Index
Andorra
SDI quintile
Low SDI
Low−middle SDI
Middle SDI
High−middle SDI
High SDI
Benchmarks
SDG target
Global Optimum
Survival Potential Frontier

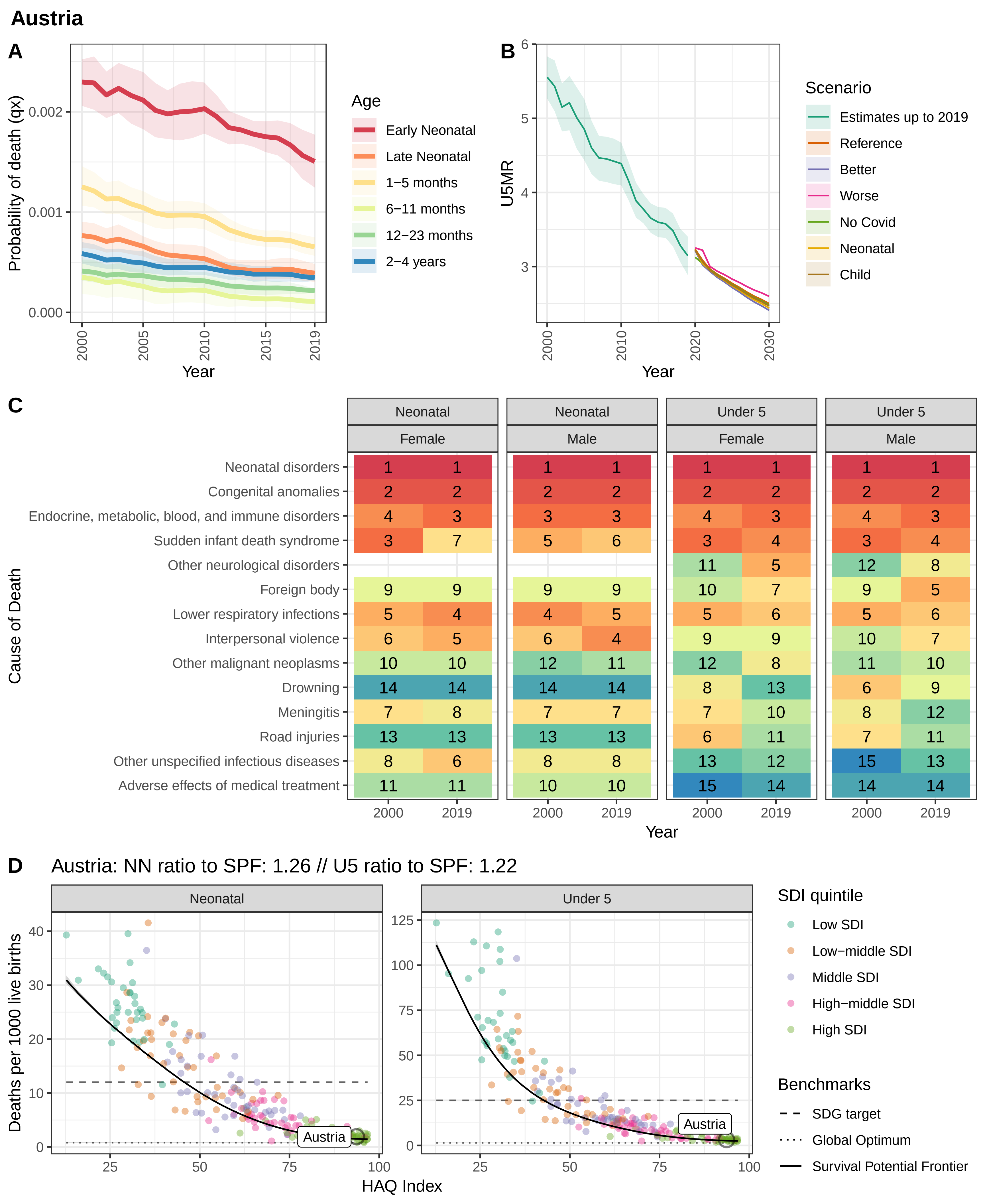
Austria
A
Probability of death (qx)
0.000
0.001
0.002
2000
2005
2010
2015
2019
Year
Age
Early Neonatal
Late Neonatal
1−5 months
6−11 months
12−23 months
2−4 years
B
U5MR
3
4
5
6
2000
2010
2020
2030
Year
Scenario
Estimates up to 2019
Reference
Better
Worse
No Covid
Neonatal
Child
C
Neonatal Female
Neonatal Male
Under 5 Female
Under 5 Male
Cause of Death
Neonatal disorders 1 1 1 1 1 1 1 1
Congenital anomalies 2 2 2 2 2 2 2 2
Endocrine, metabolic, blood, and immune disorders 4 3 3 3 4 3 4 3
Sudden infant death syndrome 3 7 5 6 3 4 3 4
Other neurological disorders 11 5 12 8
Foreign body 9 9 9 9 10 7 9 5
Lower respiratory infections 5 4 4 5 5 6 5 6
Interpersonal violence 6 5 6 4 9 9 10 7
Other malignant neoplasms 10 10 12 11 12 8 11 10
Drowning 14 14 14 14 8 13 6 9
Meningitis 7 8 7 7 7 10 8 12
Road injuries 13 13 13 13 6 11 7 11
Other unspecified infectious diseases 8 6 8 8 13 12 15 13
Adverse effects of medical treatment 11 11 10 10 15 14 14 14
2000
2019
Year
D
Austria: NN ratio to SPF: 1.26 // U5 ratio to SPF: 1.22
Neonatal
Under 5
Deaths per 1000 live births
0
10
20
30
40
0
25
50
75
100
125
25
50
75
100
HAQ Index
Austria
SDI quintile
Low SDI
Low−middle SDI
Middle SDI
High−middle SDI
High SDI
Benchmarks
SDG target
Global Optimum
Survival Potential Frontier

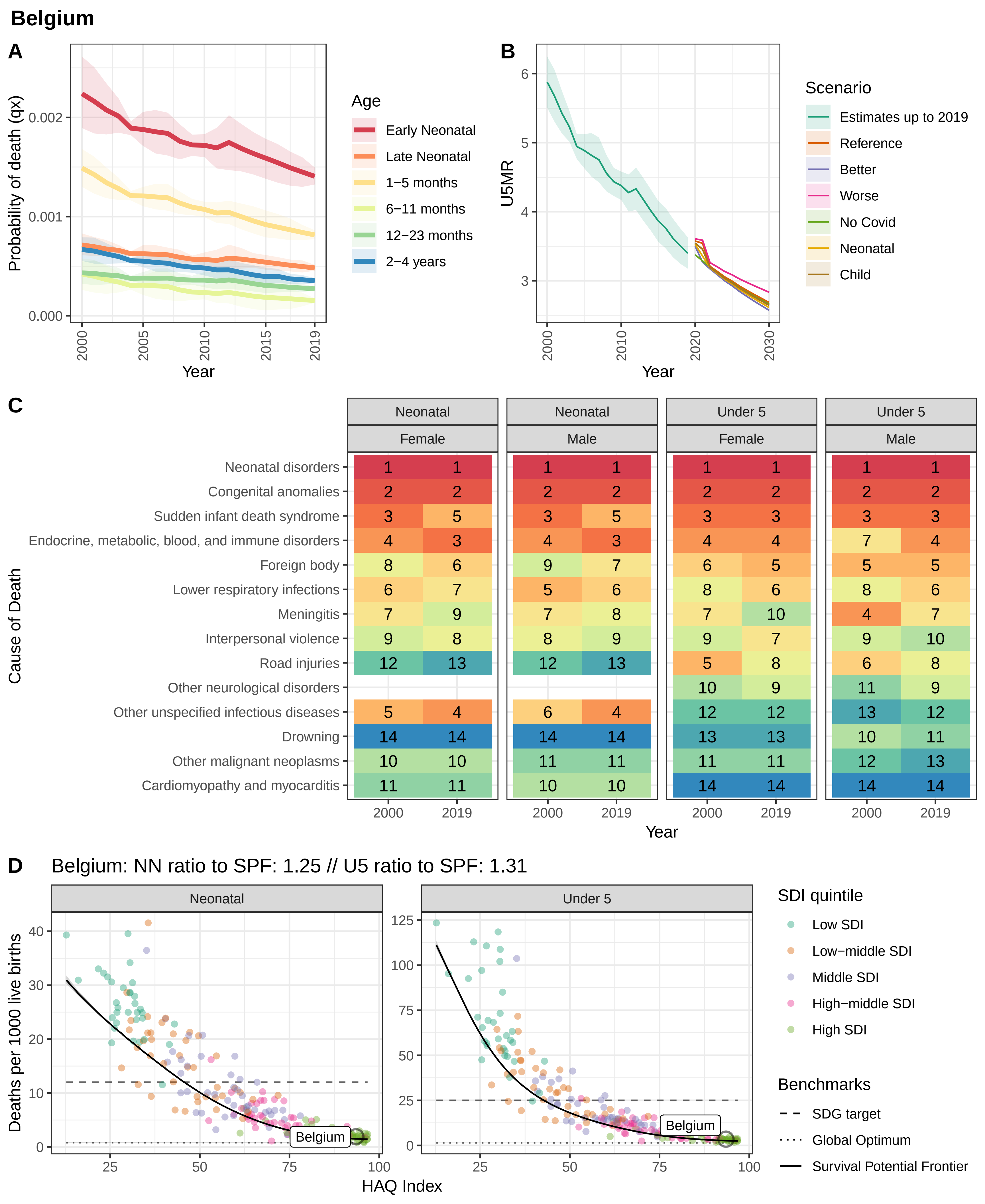
Belgium
A
Probability of death (qx)
0.000
0.001
0.002
2000
2005
2010
2015
2019
Year
Age
Early Neonatal
Late Neonatal
1−5 months
6−11 months
12−23 months
2−4 years
B
U5MR
3
4
5
6
2000
2010
2020
2030
Year
Scenario
Estimates up to 2019
Reference
Better
Worse
No Covid
Neonatal
Child
C
Neonatal
Female
Neonatal
Male
Under 5
Female
Under 5
Male
Cause of Death
Neonatal disorders 1 1 1 1 1 1 1 1
Congenital anomalies 2 2 2 2 2 2 2 2
Sudden infant death syndrome 3 5 3 5 3 3 3 3
Endocrine, metabolic, blood, and immune disorders 4 3 4 3 4 4 7 4
Foreign body 8 6 9 7 6 5 5 5
Lower respiratory infections 6 7 5 6 8 6 8 6
Meningitis 7 9 7 8 7 10 4 7
Interpersonal violence 9 8 8 9 9 7 9 10
Road injuries 12 13 12 13 5 8 6 8
Other neurological disorders 10 9 11 9
Other unspecified infectious diseases 5 4 6 4 12 12 13 12
Drowning 14 14 14 14 13 13 10 11
Other malignant neoplasms 10 10 11 11 11 11 12 13
Cardiomyopathy and myocarditis 11 11 10 10 14 14 14 14
2000
2019
Year
D
Belgium: NN ratio to SPF: 1.25 // U5 ratio to SPF: 1.31
Neonatal
Under 5
Deaths per 1000 live births
0
10
20
30
40
25
50
75
100
125
Belgium
HAQ Index
SDI quintile
Low SDI
Low−middle SDI
Middle SDI
High−middle SDI
High SDI
Benchmarks
SDG target
Global Optimum
Survival Potential Frontier

Cyprus

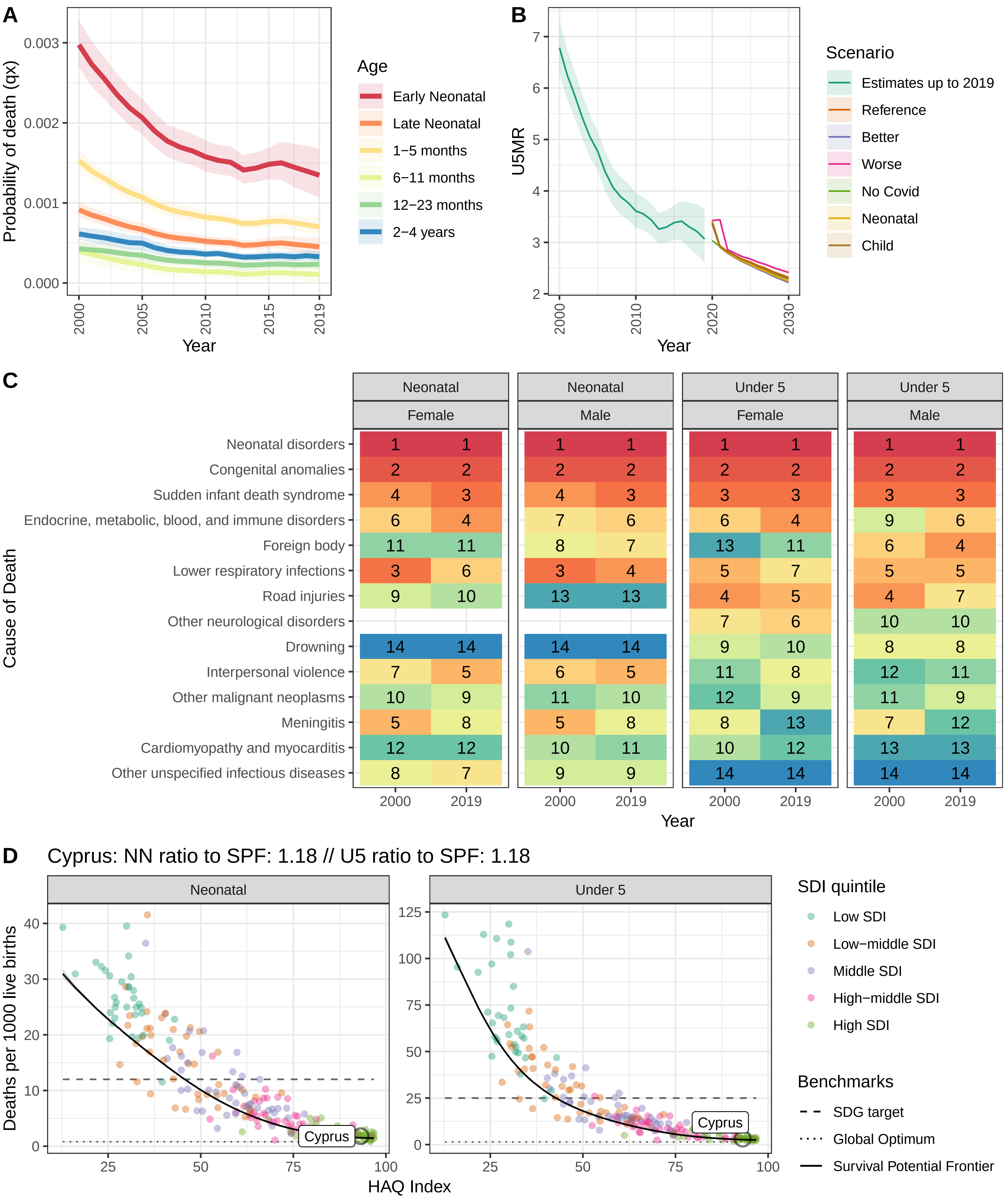

A
Probability of death (qx)
0.003
0.002
0.001
0.000
2000
2005
2010
2015
2019
Year
Age
Early Neonatal
Late Neonatal
1−5 months
6−11 months
12−23 months
2−4 years
B
U5MR
7
6
5
4
3
2
2000
2010
2020
2030
Year
Scenario
Estimates up to 2019
Reference
Better
Worse
No Covid
Neonatal
Child
C
Cause of Death
Neonatal Female
Neonatal Male
Under 5 Female
Under 5 Male
Neonatal disorders 1 1 1 1 1 1 1 1
Congenital anomalies 2 2 2 2 2 2 2 2
Sudden infant death syndrome 4 3 4 3 3 3 3 3
Endocrine, metabolic, blood, and immune disorders 6 4 7 6 6 4 9 6
Foreign body 11 11 8 7 13 11 6 4
Lower respiratory infections 3 6 3 4 5 7 5 5
Road injuries 9 10 13 13 4 5 4 7
Other neurological disorders 7 6 10 10
Drowning 14 14 14 14 9 10 8 8
Interpersonal violence 7 5 6 5 11 8 12 11
Other malignant neoplasms 10 9 11 10 12 9 11 9
Meningitis 5 8 5 8 8 13 7 12
Cardiomyopathy and myocarditis 12 12 10 11 10 12 13 13
Other unspecified infectious diseases 8 7 9 9 14 14 14 14
2000
2019
Year
D
Cyprus: NN ratio to SPF: 1.18 // U5 ratio to SPF: 1.18
Neonatal
Under 5
Deaths per 1000 live births
40
30
20
10
0
125
100
75
50
25
0
25
50
75
100
Cyprus
HAQ Index
SDI quintile
Low SDI
Low−middle SDI
Middle SDI
High−middle SDI
High SDI
Benchmarks
SDG target
Global Optimum
Survival Potential Frontier

Denmark

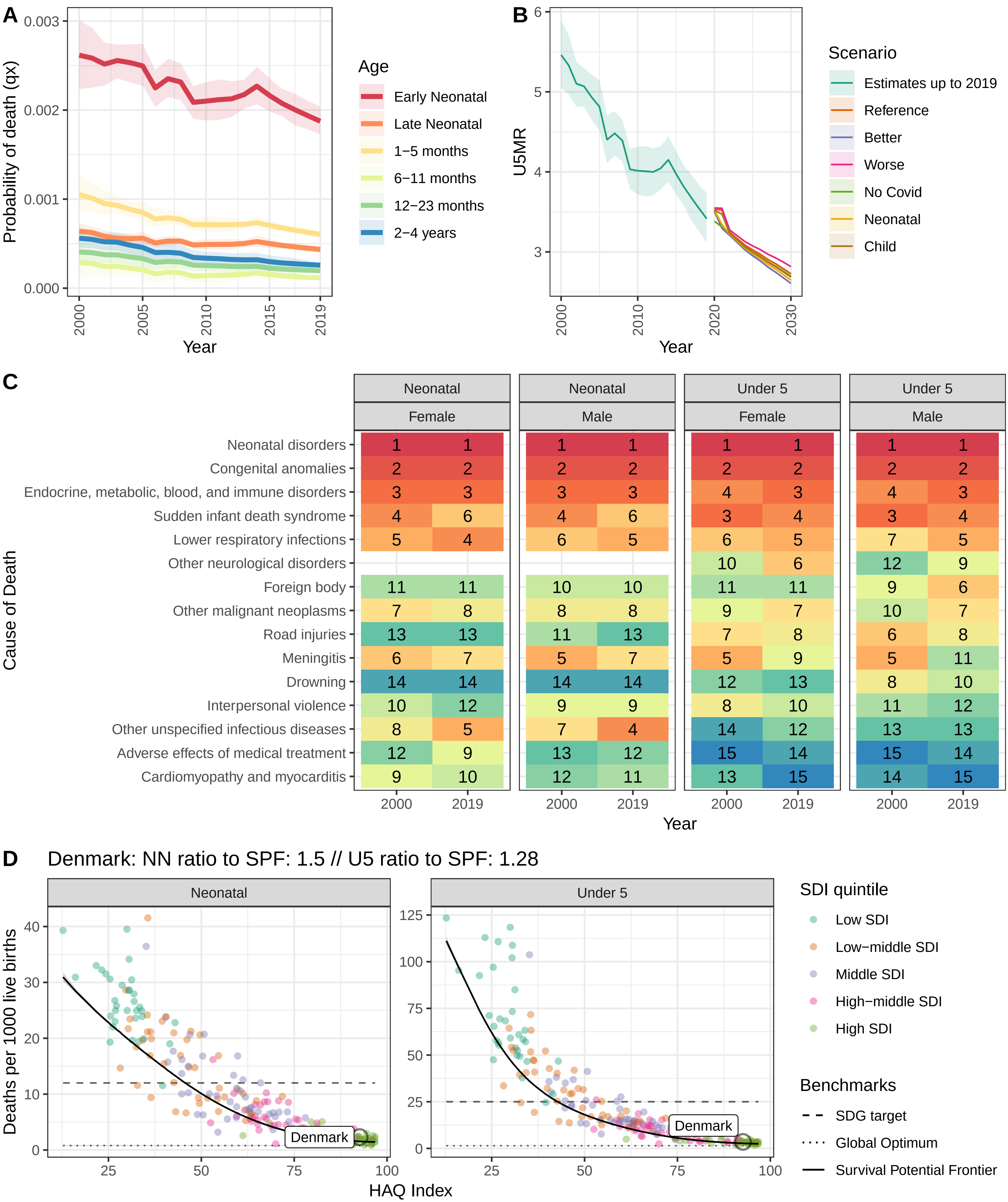

A
Probability of death (qx)
Year
Age
Early Neonatal
Late Neonatal
1−5 months
6−11 months
12−23 months
2−4 years
B
U5MR
Year
Scenario
Estimates up to 2019
Reference
Better
Worse
No Covid
Neonatal
Child
C
Neonatal
Under 5
Female
Male
Cause of Death
Neonatal disorders
Congenital anomalies
Endocrine, metabolic, blood, and immune disorders
Sudden infant death syndrome
Lower respiratory infections
Other neurological disorders
Foreign body
Other malignant neoplasms
Road injuries
Meningitis
Drowning
Interpersonal violence
Other unspecified infectious diseases
Adverse effects of medical treatment
Cardiomyopathy and myocarditis
Year
D
Denmark: NN ratio to SPF: 1.5 // U5 ratio to SPF: 1.28
Neonatal
Under 5
Deaths per 1000 live births
HAQ Index
Denmark
SDI quintile
Low SDI
Low−middle SDI
Middle SDI
High−middle SDI
High SDI
Benchmarks
SDG target
Global Optimum
Survival Potential Frontier

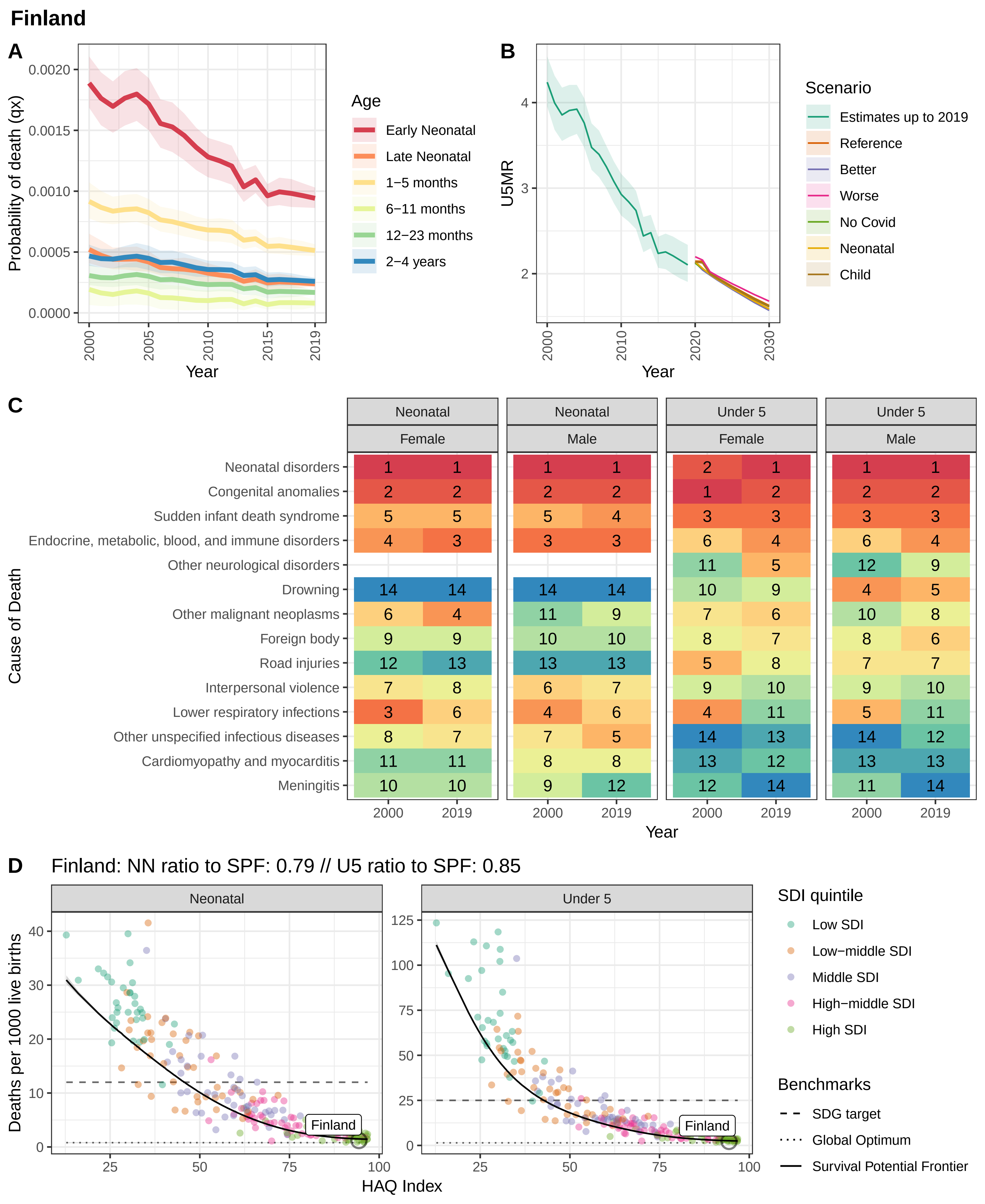

Finland
A
Probability of death (qx)
0.0020
0.0015
0.0010
0.0005
0.0000
2000
2005
2010
2015
2019
Year
Age
Early Neonatal
Late Neonatal
1−5 months
6−11 months
12−23 months
2−4 years
B
U5MR
4
3
2
2000
2010
2020
2030
Year
Scenario
Estimates up to 2019
Reference
Better
Worse
No Covid
Neonatal
Child
C
Neonatal
Female
Neonatal
Male
Under 5
Female
Under 5
Male
Cause of Death
Neonatal disorders
Congenital anomalies
Sudden infant death syndrome
Endocrine, metabolic, blood, and immune disorders
Other neurological disorders
Drowning
Other malignant neoplasms
Foreign body
Road injuries
Interpersonal violence
Lower respiratory infections
Other unspecified infectious diseases
Cardiomyopathy and myocarditis
Meningitis
1 1 1 1 2 1 1 1
2 2 2 2 1 2 2 2
5 5 5 4 3 3 3 3
4 3 3 3 6 4 6 4
11 5 12 9
14 14 14 14 10 9 4 5
6 4 11 9 7 6 10 8
9 9 10 10 8 7 8 6
12 13 13 13 5 8 7 7
7 8 6 7 9 10 9 10
3 6 4 6 4 11 5 11
8 7 7 5 14 13 14 12
11 11 8 8 13 12 13 13
10 10 9 12 12 14 11 14
2000
2019
Year
D
Finland: NN ratio to SPF: 0.79 // U5 ratio to SPF: 0.85
Neonatal
Under 5
Deaths per 1000 live births
40
30
20
10
0
125
100
75
50
25
0
25
50
75
100
HAQ Index
Finland
SDI quintile
Low SDI
Low−middle SDI
Middle SDI
High−middle SDI
High SDI
Benchmarks
SDG target
Global Optimum
Survival Potential Frontier

Taiwan (Province of China)

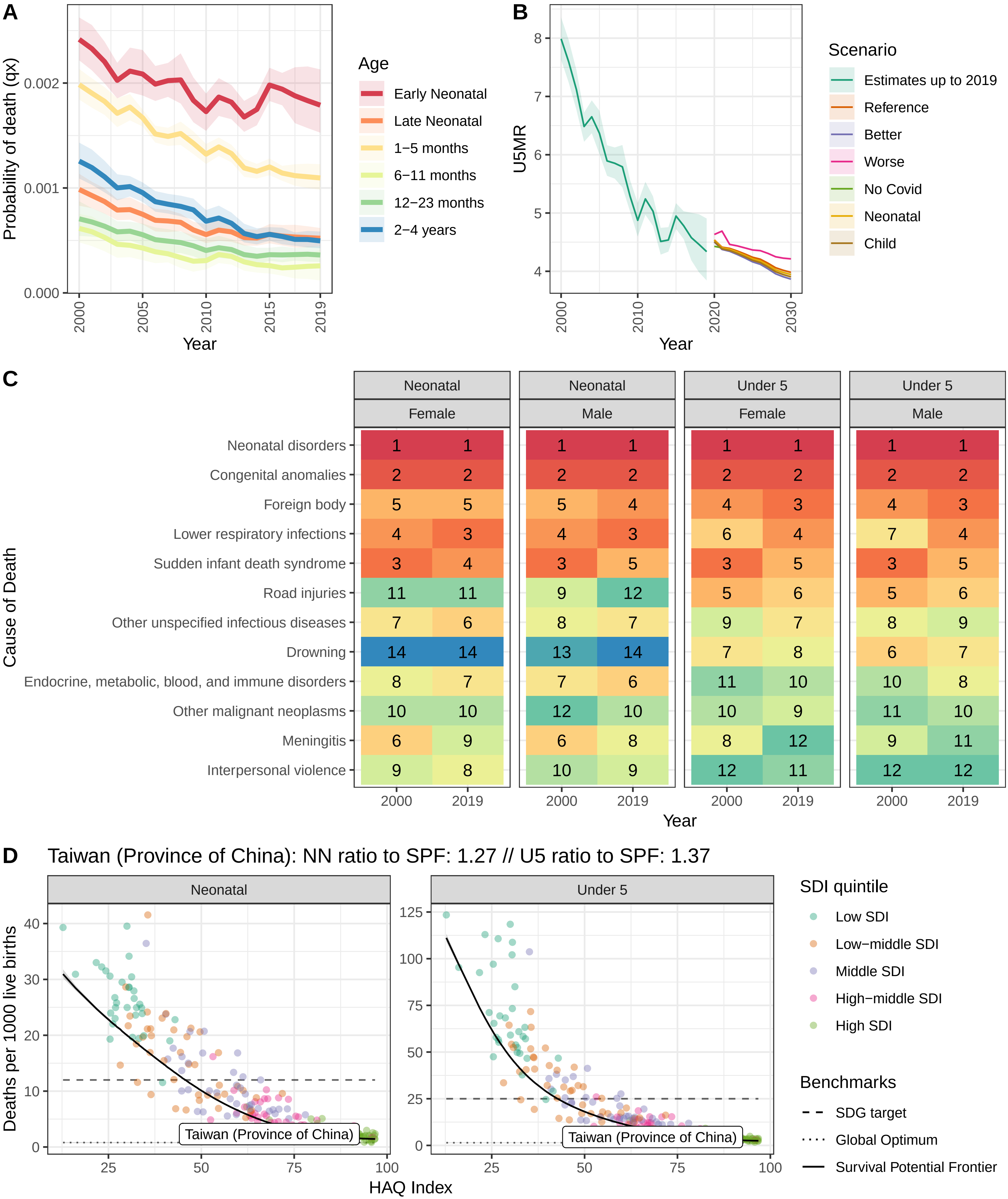

France

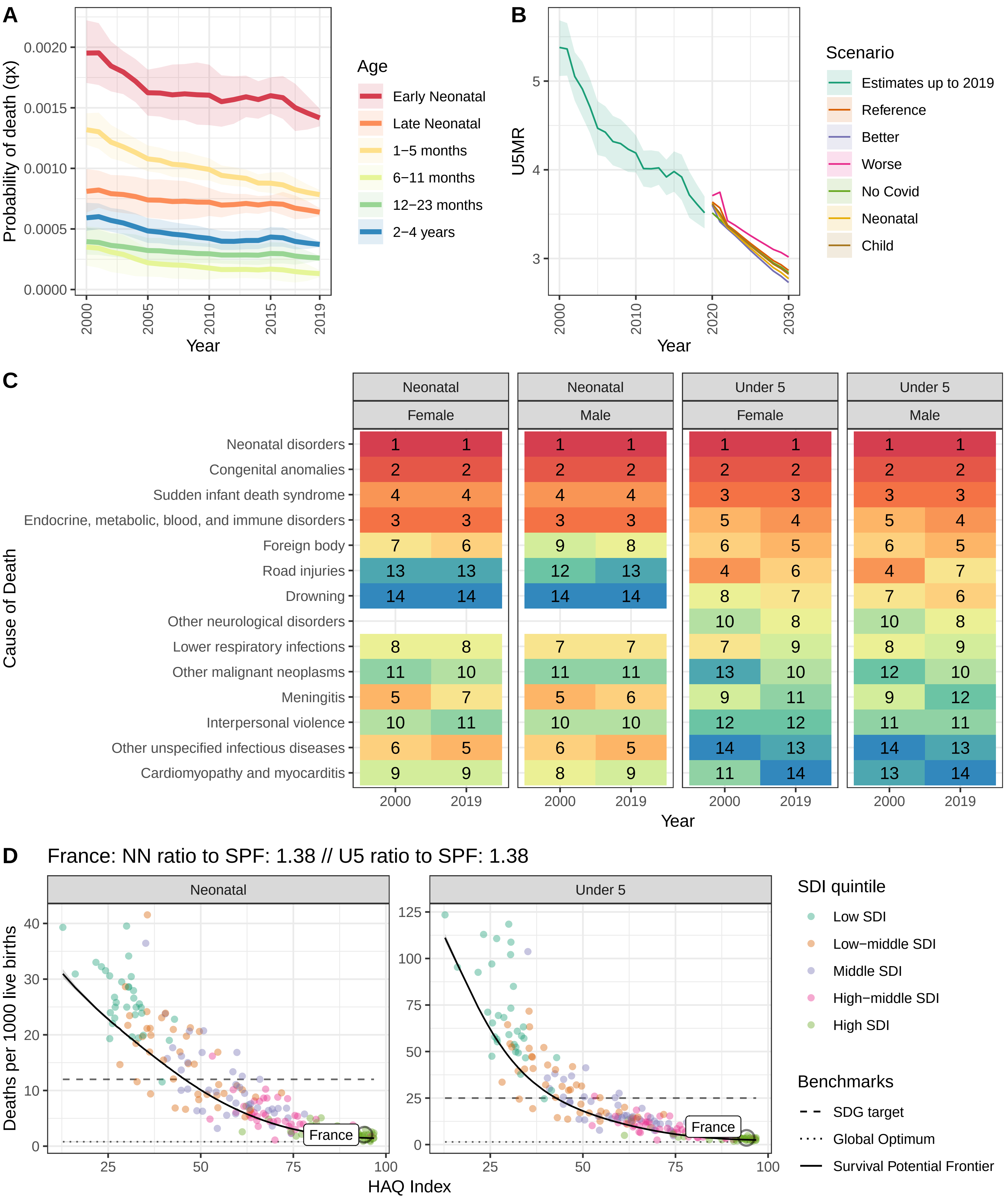
A
Probability of death (qx)
Year
Age
Early Neonatal
Late Neonatal
1−5 months
6−11 months
12−23 months
2−4 years
B
U5MR
Year
Scenario
Estimates up to 2019
Reference
Better
Worse
No Covid
Neonatal
Child
C
Neonatal
Female
Neonatal
Male
Under 5
Female
Under 5
Male
Cause of Death
Neonatal disorders
Congenital anomalies
Sudden infant death syndrome
Endocrine, metabolic, blood, and immune disorders
Foreign body
Road injuries
Drowning
Other neurological disorders
Lower respiratory infections
Other malignant neoplasms
Meningitis
Interpersonal violence
Other unspecified infectious diseases
Cardiomyopathy and myocarditis
Year
D
France: NN ratio to SPF: 1.38 // U5 ratio to SPF: 1.38
Neonatal
Under 5
Deaths per 1000 live births
HAQ Index
France
SDI quintile
Low SDI
Low−middle SDI
Middle SDI
High−middle SDI
High SDI
Benchmarks
SDG target
Global Optimum
Survival Potential Frontier

Germany

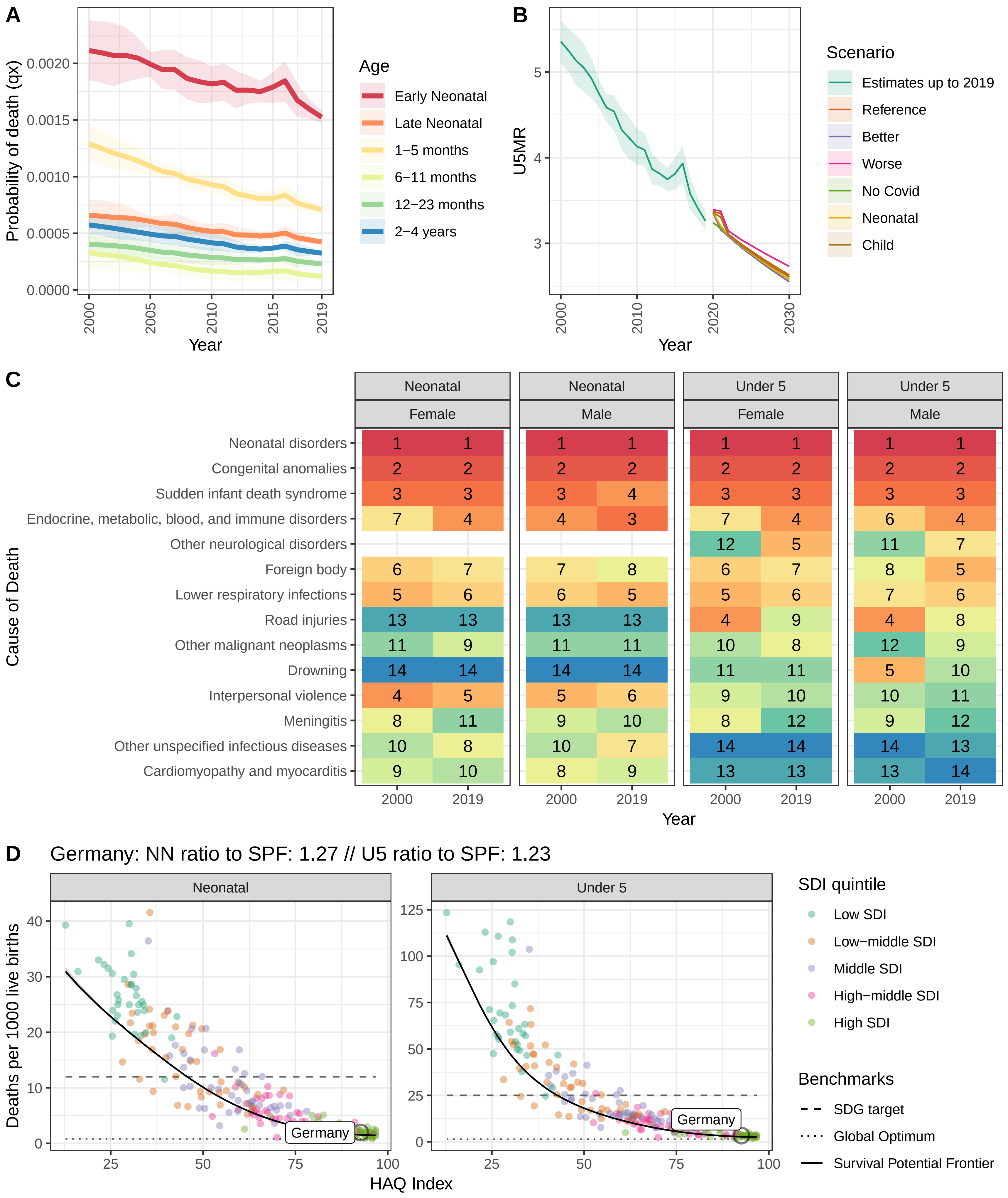
A
Probability of death (qx)
Year
Age
Early Neonatal
Late Neonatal
1−5 months
6−11 months
12−23 months
2−4 years
B
U5MR
Year
Scenario
Estimates up to 2019
Reference
Better
Worse
No Covid
Neonatal
Child
C
Neonatal
Female
Neonatal
Male
Under 5
Female
Under 5
Male
Cause of Death
Neonatal disorders
Congenital anomalies
Sudden infant death syndrome
Endocrine, metabolic, blood, and immune disorders
Other neurological disorders
Foreign body
Lower respiratory infections
Road injuries
Other malignant neoplasms
Drowning
Interpersonal violence
Meningitis
Other unspecified infectious diseases
Cardiomyopathy and myocarditis
Year
D
Germany: NN ratio to SPF: 1.27 // U5 ratio to SPF: 1.23
Neonatal
Under 5
Deaths per 1000 live births
HAQ Index
Germany
SDI quintile
Low SDI
Low−middle SDI
Middle SDI
High−middle SDI
High SDI
Benchmarks
SDG target
Global Optimum
Survival Potential Frontier

Greece

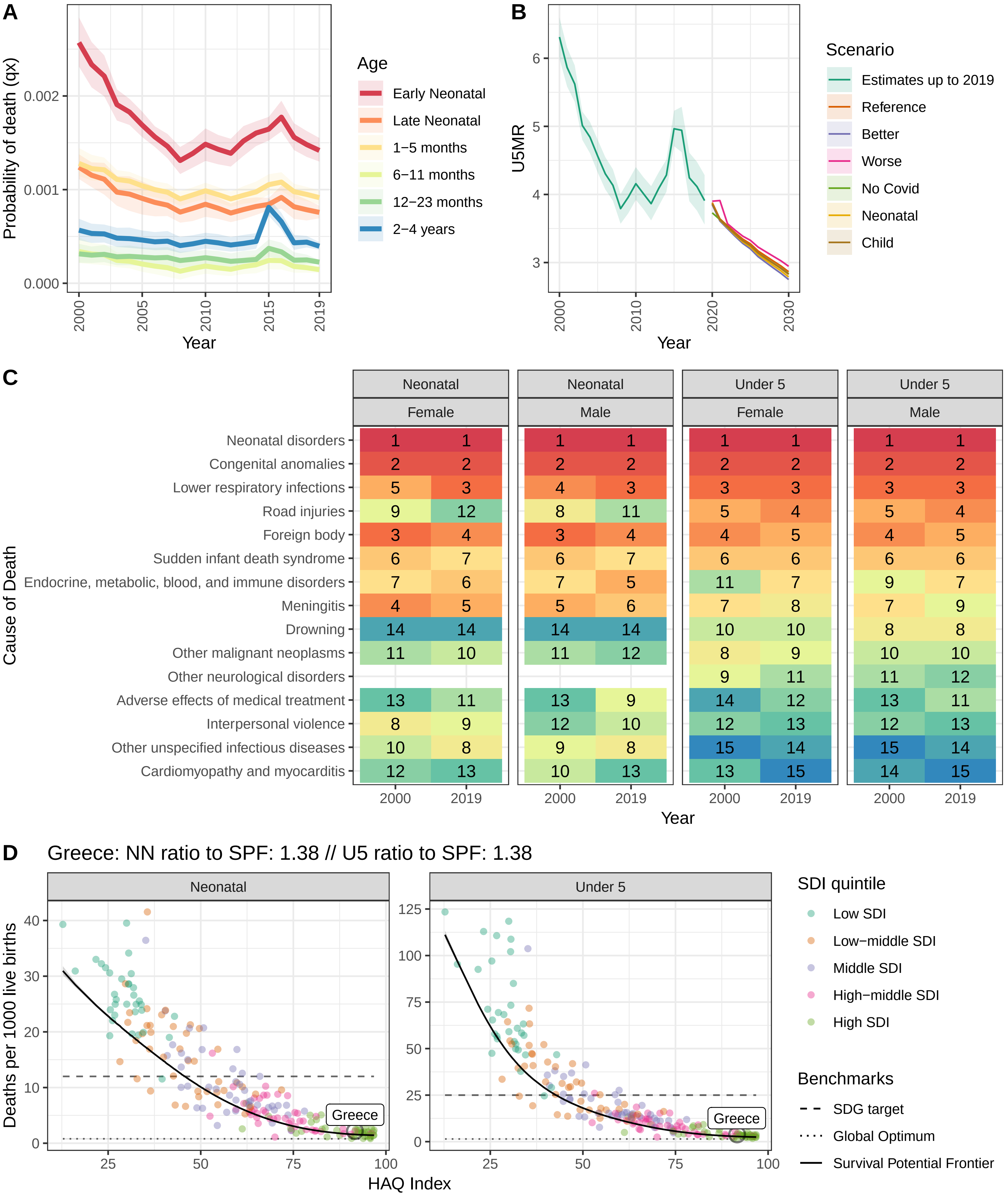

A
Probability of death (qx)
0.000
0.001
0.002
2000
2005
2010
2015
2019
Year
Age
Early Neonatal
Late Neonatal
1−5 months
6−11 months
12−23 months
2−4 years
B
U5MR
3
4
5
6
2000
2010
2020
2030
Year
Scenario
Estimates up to 2019
Reference
Better
Worse
No Covid
Neonatal
Child
C
Cause of Death
Neonatal Female
Neonatal Male
Under 5 Female
Under 5 Male
Neonatal disorders 1 1 1 1 1 1 1 1
Congenital anomalies 2 2 2 2 2 2 2 2
Lower respiratory infections 5 3 4 3 3 3 3 3
Road injuries 9 12 8 11 5 4 5 4
Foreign body 3 4 3 4 4 5 4 5
Sudden infant death syndrome 6 7 6 7 6 6 6 6
Endocrine, metabolic, blood, and immune disorders 7 6 7 5 11 7 9 7
Meningitis 4 5 5 6 7 8 7 9
Drowning 14 14 14 14 10 10 8 8
Other malignant neoplasms 11 10 11 12 8 9 10 10
Other neurological disorders 9 11 11 12
Adverse effects of medical treatment 13 11 13 9 14 12 13 11
Interpersonal violence 8 9 12 10 12 13 12 13
Other unspecified infectious diseases 10 8 9 8 15 14 15 14
Cardiomyopathy and myocarditis 12 13 10 13 13 15 14 15
2000
2019
Year
D
Greece: NN ratio to SPF: 1.38 // U5 ratio to SPF: 1.38
Neonatal
Under 5
Deaths per 1000 live births
0
10
20
30
40
0
25
50
75
100
125
25
50
75
100
HAQ Index
Greece
SDI quintile
Low SDI
Low−middle SDI
Middle SDI
High−middle SDI
High SDI
Benchmarks
SDG target
Global Optimum
Survival Potential Frontier

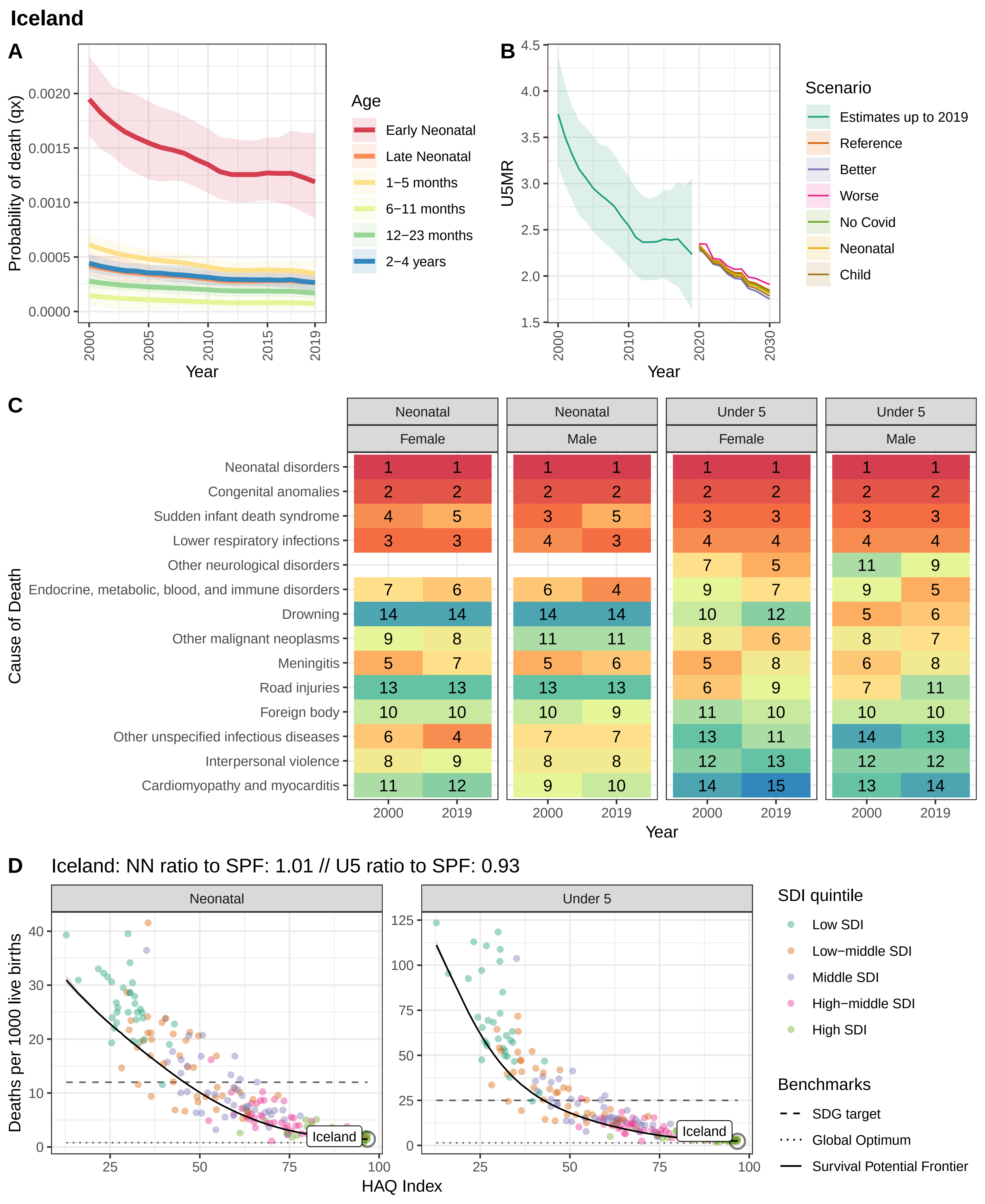
Iceland
A
Probability of death (qx)
0.0020
0.0015
0.0010
0.0005
0.0000
2000
2005
2010
2015
2019
Year
Age
Early Neonatal
Late Neonatal
1–5 months
6–11 months
12–23 months
2–4 years
B
U5MR
4.5
4.0
3.5
3.0
2.5
2.0
1.5
2000
2010
2020
2030
Year
Scenario
Estimates up to 2019
Reference
Better
Worse
No Covid
Neonatal
Child
C
Cause of Death
Neonatal Female
Neonatal Male
Under 5 Female
Under 5 Male
Neonatal disorders 1 1 1 1 1 1 1 1
Congenital anomalies 2 2 2 2 2 2 2 2
Sudden infant death syndrome 4 5 3 5 3 3 3 3
Lower respiratory infections 3 3 4 3 4 4 4 4
Other neurological disorders 7 5 11 9
Endocrine, metabolic, blood, and immune disorders 7 6 6 4 9 7 9 5
Drowning 14 14 14 14 10 12 5 6
Other malignant neoplasms 9 8 11 11 8 6 8 7
Meningitis 5 7 5 6 5 8 6 8
Road injuries 13 13 13 13 6 9 7 11
Foreign body 10 10 10 9 11 10 10 10
Other unspecified infectious diseases 6 4 7 7 13 11 14 13
Interpersonal violence 8 9 8 8 12 13 12 12
Cardiomyopathy and myocarditis 11 12 9 10 14 15 13 14
2000
2019
Year
D
Iceland: NN ratio to SPF: 1.01 // U5 ratio to SPF: 0.93
Neonatal
Under 5
Deaths per 1000 live births
40
30
20
10
0
125
100
75
50
25
0
25
50
75
100
Iceland
HAQ Index
SDI quintile
Low SDI
Low−middle SDI
Middle SDI
High−middle SDI
High SDI
Benchmarks
SDG target
Global Optimum
Survival Potential Frontier

Ireland

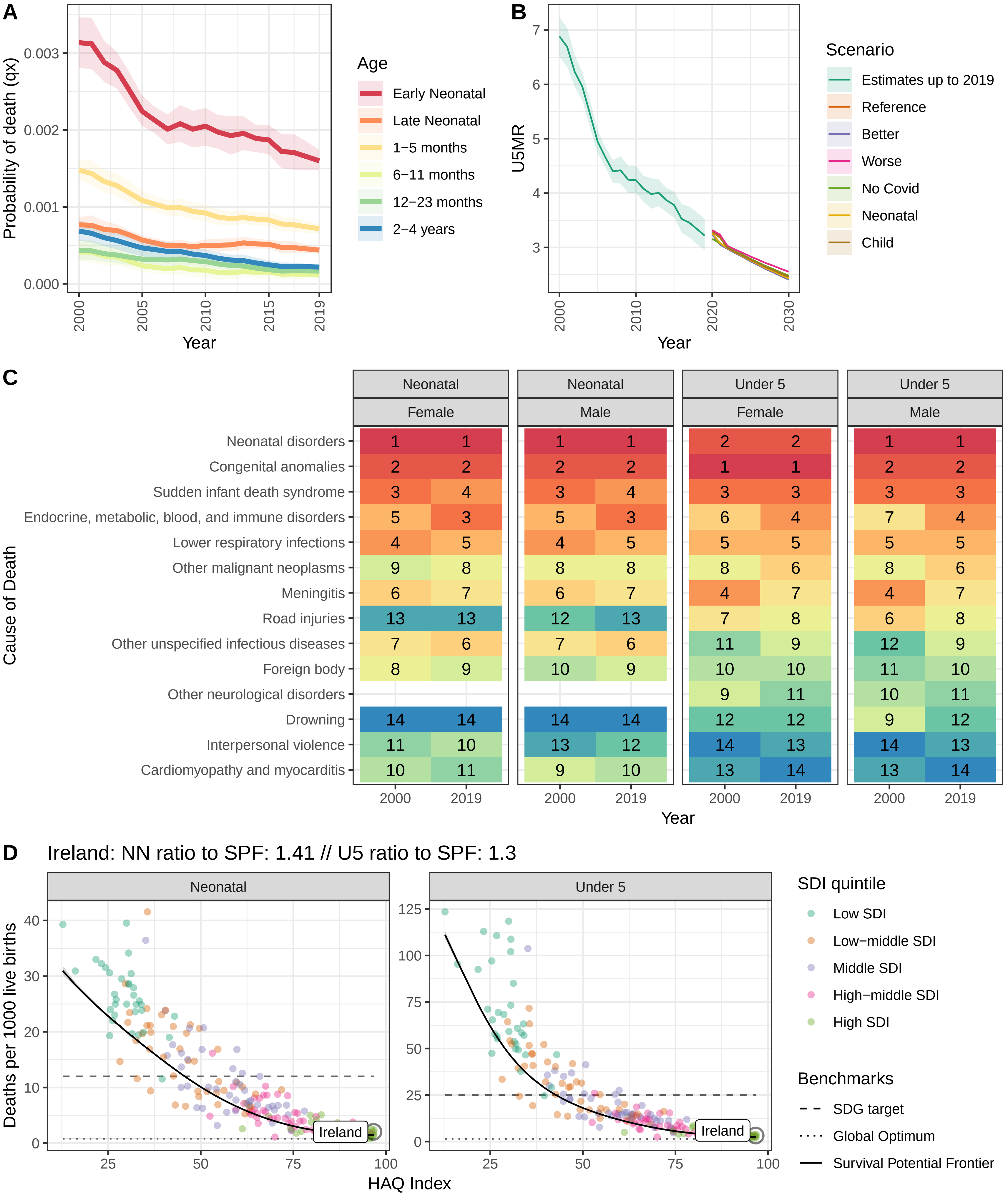
A
Probability of death (qx)
0.003
0.002
0.001
0.000
2000
2005
2010
2015
2019
Year
Age
Early Neonatal
Late Neonatal
1−5 months
6−11 months
12−23 months
2−4 years
B
U5MR
7
6
5
4
3
2000
2010
2020
2030
Year
Scenario
Estimates up to 2019
Reference
Better
Worse
No Covid
Neonatal
Child
C
Cause of Death
Neonatal Female
Neonatal Male
Under 5 Female
Under 5 Male
Neonatal disorders 1 1 1 1 2 2 1 1
Congenital anomalies 2 2 2 2 1 1 2 2
Sudden infant death syndrome 3 4 3 4 3 3 3 3
Endocrine, metabolic, blood, and immune disorders 5 3 5 3 6 4 7 4
Lower respiratory infections 4 5 4 5 5 5 5 5
Other malignant neoplasms 9 8 8 8 8 6 8 6
Meningitis 6 7 6 7 4 7 4 7
Road injuries 13 13 12 13 7 8 6 8
Other unspecified infectious diseases 7 6 7 6 11 9 12 9
Foreign body 8 9 10 9 10 10 11 10
Other neurological disorders 9 11 10 11
Drowning 14 14 14 14 12 12 9 12
Interpersonal violence 11 10 13 12 14 13 14 13
Cardiomyopathy and myocarditis 10 11 9 10 13 14 13 14
2000 2019
Year
D
Ireland: NN ratio to SPF: 1.41 // U5 ratio to SPF: 1.3
Neonatal
Under 5
Deaths per 1000 live births
40
30
20
10
0
125
100
75
50
25
0
Ireland
25
50
75
100
HAQ Index
SDI quintile
Low SDI
Low−middle SDI
Middle SDI
High−middle SDI
High SDI
Benchmarks
SDG target
Global Optimum
Survival Potential Frontier

Israel

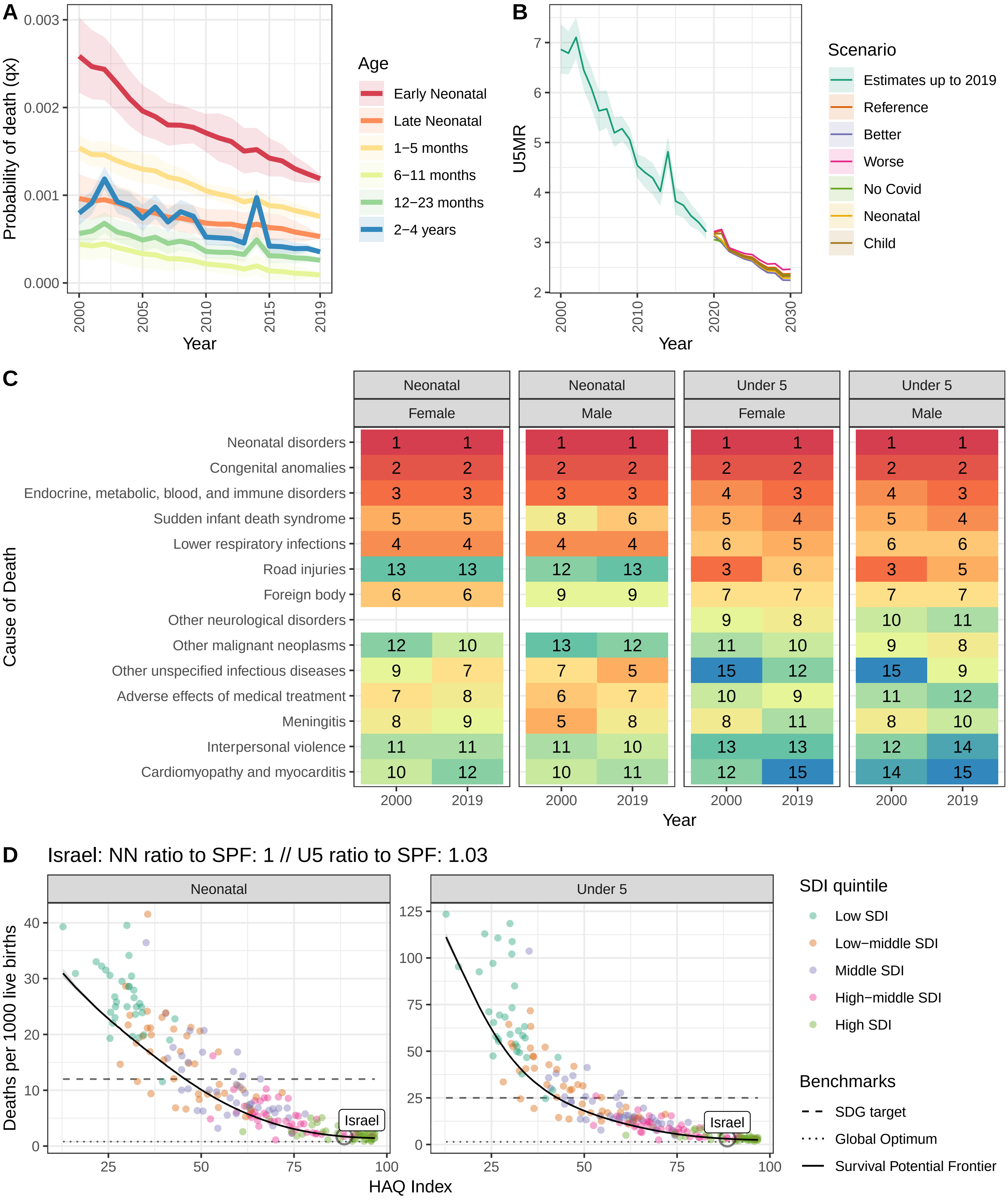

Cause of Death	Neonatal Female 2000	Neonatal Female 2019	Neonatal Male 2000	Neonatal Male 2019	Under 5 Female 2000	Under 5 Female 2019	Under 5 Male 2000	Under 5 Male 2019
Neonatal disorders	1	1	1	1	1	1	1	1
Congenital anomalies	2	2	2	2	2	2	2	2
Endocrine, metabolic, blood, and immune disorders	3	3	3	3	4	3	4	3
Sudden infant death syndrome	5	5	8	6	5	4	5	4
Lower respiratory infections	4	4	4	4	6	5	6	6
Road injuries	13	13	12	13	3	6	3	5
Foreign body	6	6	9	9	7	7	7	7
Other neurological disorders					9	8	10	11
Other malignant neoplasms	12	10	13	12	11	10	9	8
Other unspecified infectious diseases	9	7	7	5	15	12	15	9
Adverse effects of medical treatment	7	8	6	7	10	9	11	12
Meningitis	8	9	5	8	8	11	8	10
Interpersonal violence	11	11	11	10	13	13	12	14
Cardiomyopathy and myocarditis	10	12	10	11	12	15	14	15

Italy

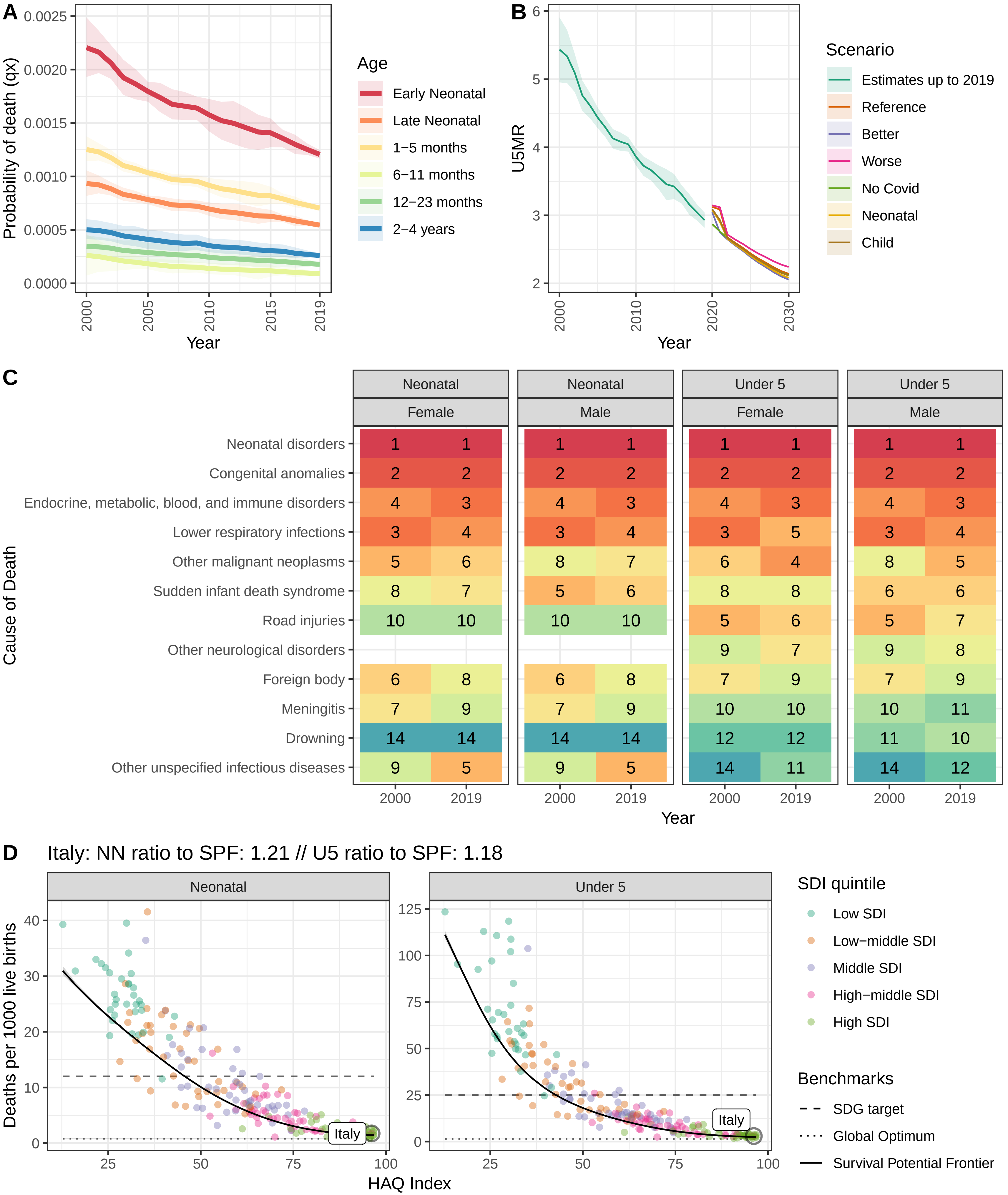
A
Probability of death (qx)
0.0025
0.0020
0.0015
0.0010
0.0005
0.0000
2000
2005
2010
2015
2019
Year
Age
Early Neonatal
Late Neonatal
1−5 months
6−11 months
12−23 months
2−4 years
B
U5MR
6
5
4
3
2
2000
2010
2020
2030
Year
Scenario
Estimates up to 2019
Reference
Better
Worse
No Covid
Neonatal
Child
C
Neonatal
Female
Neonatal
Male
Under 5
Female
Under 5
Male
Cause of Death
Neonatal disorders 1 1 1 1 1 1 1 1
Congenital anomalies 2 2 2 2 2 2 2 2
Endocrine, metabolic, blood, and immune disorders 4 3 4 3 4 3 4 3
Lower respiratory infections 3 4 3 4 3 5 3 4
Other malignant neoplasms 5 6 8 7 6 4 8 5
Sudden infant death syndrome 8 7 5 6 8 8 6 6
Road injuries 10 10 10 10 5 6 5 7
Other neurological disorders 9 7 9 8
Foreign body 6 8 6 8 7 9 7 9
Meningitis 7 9 7 9 10 10 10 11
Drowning 14 14 14 14 12 12 11 10
Other unspecified infectious diseases 9 5 9 5 14 11 14 12
2000
2019
Year
D
Italy: NN ratio to SPF: 1.21 // U5 ratio to SPF: 1.18
Neonatal
Under 5
Deaths per 1000 live births
40
30
20
10
0
125
100
75
50
25
Italy
HAQ Index
SDI quintile
Low SDI
Low−middle SDI
Middle SDI
High−middle SDI
High SDI
Benchmarks
SDG target
Global Optimum
Survival Potential Frontier

Luxembourg

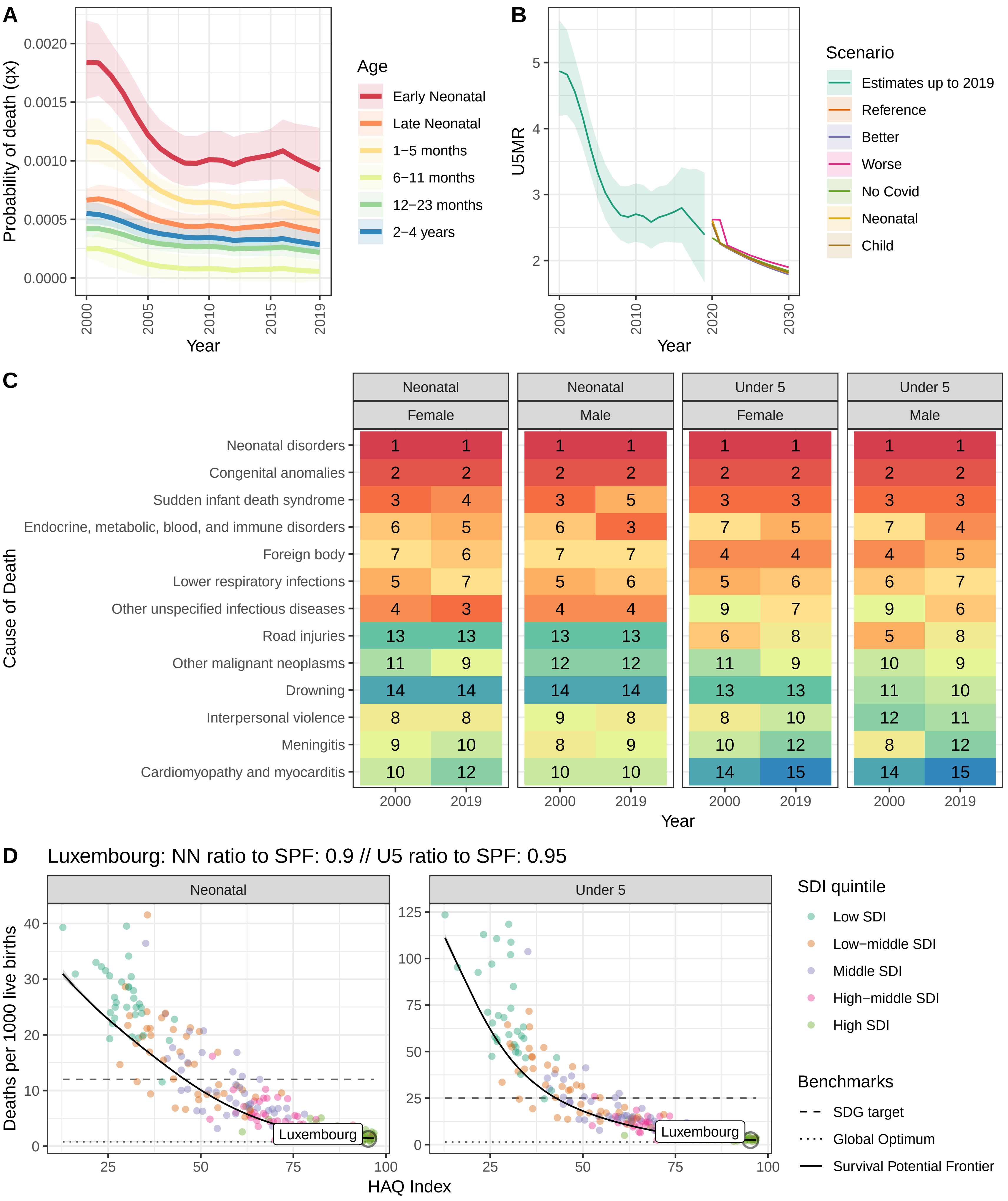

A
Probability of death (qx)
Year
Age
Early Neonatal
Late Neonatal
1−5 months
6−11 months
12−23 months
2−4 years
B
U5MR
Year
Scenario
Estimates up to 2019
Reference
Better
Worse
No Covid
Neonatal
Child
C
Cause of Death
Neonatal Female
Neonatal Male
Under 5 Female
Under 5 Male
Neonatal disorders
Congenital anomalies
Sudden infant death syndrome
Endocrine, metabolic, blood, and immune disorders
Foreign body
Lower respiratory infections
Other unspecified infectious diseases
Road injuries
Other malignant neoplasms
Drowning
Interpersonal violence
Meningitis
Cardiomyopathy and myocarditis
Year
D
Luxembourg: NN ratio to SPF: 0.9 // U5 ratio to SPF: 0.95
Neonatal
Under 5
Deaths per 1000 live births
HAQ Index
Luxembourg
SDI quintile
Low SDI
Low−middle SDI
Middle SDI
High−middle SDI
High SDI
Benchmarks
SDG target
Global Optimum
Survival Potential Frontier

Malta

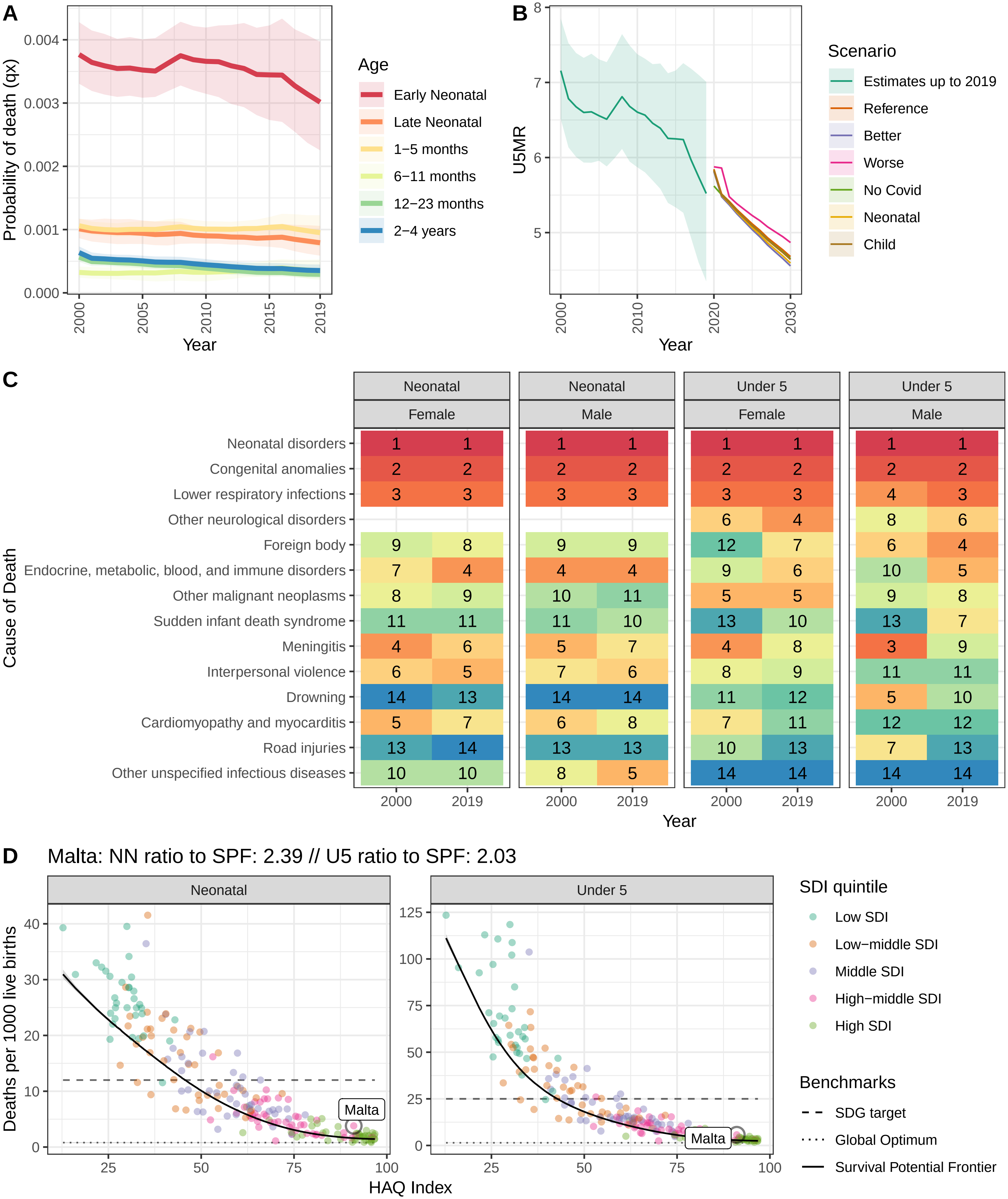
A
Probability of death (qx)
Year
Age
Early Neonatal
Late Neonatal
1−5 months
6−11 months
12−23 months
2−4 years
B
U5MR
Year
Scenario
Estimates up to 2019
Reference
Better
Worse
No Covid
Neonatal
Child
C
Neonatal Female
Neonatal Male
Under 5 Female
Under 5 Male
Cause of Death
Year
Neonatal disorders 1 1 1 1 1 1 1 1
Congenital anomalies 2 2 2 2 2 2 2 2
Lower respiratory infections 3 3 3 3 3 3 4 3
Other neurological disorders 6 4 8 6
Foreign body 9 8 9 9 12 7 6 4
Endocrine, metabolic, blood, and immune disorders 7 4 4 4 9 6 10 5
Other malignant neoplasms 8 9 10 11 5 5 9 8
Sudden infant death syndrome 11 11 11 10 13 10 13 7
Meningitis 4 6 5 7 4 8 3 9
Interpersonal violence 6 5 7 6 8 9 11 11
Drowning 14 13 14 14 11 12 5 10
Cardiomyopathy and myocarditis 5 7 6 8 7 11 12 12
Road injuries 13 14 13 13 10 13 7 13
Other unspecified infectious diseases 10 10 8 5 14 14 14 14
D
Malta: NN ratio to SPF: 2.39 // U5 ratio to SPF: 2.03
Neonatal
Under 5
Malta
Deaths per 1000 live births
HAQ Index
SDI quintile
Low SDI
Low−middle SDI
Middle SDI
High−middle SDI
High SDI
Benchmarks
SDG target
Global Optimum
Survival Potential Frontier

Netherlands

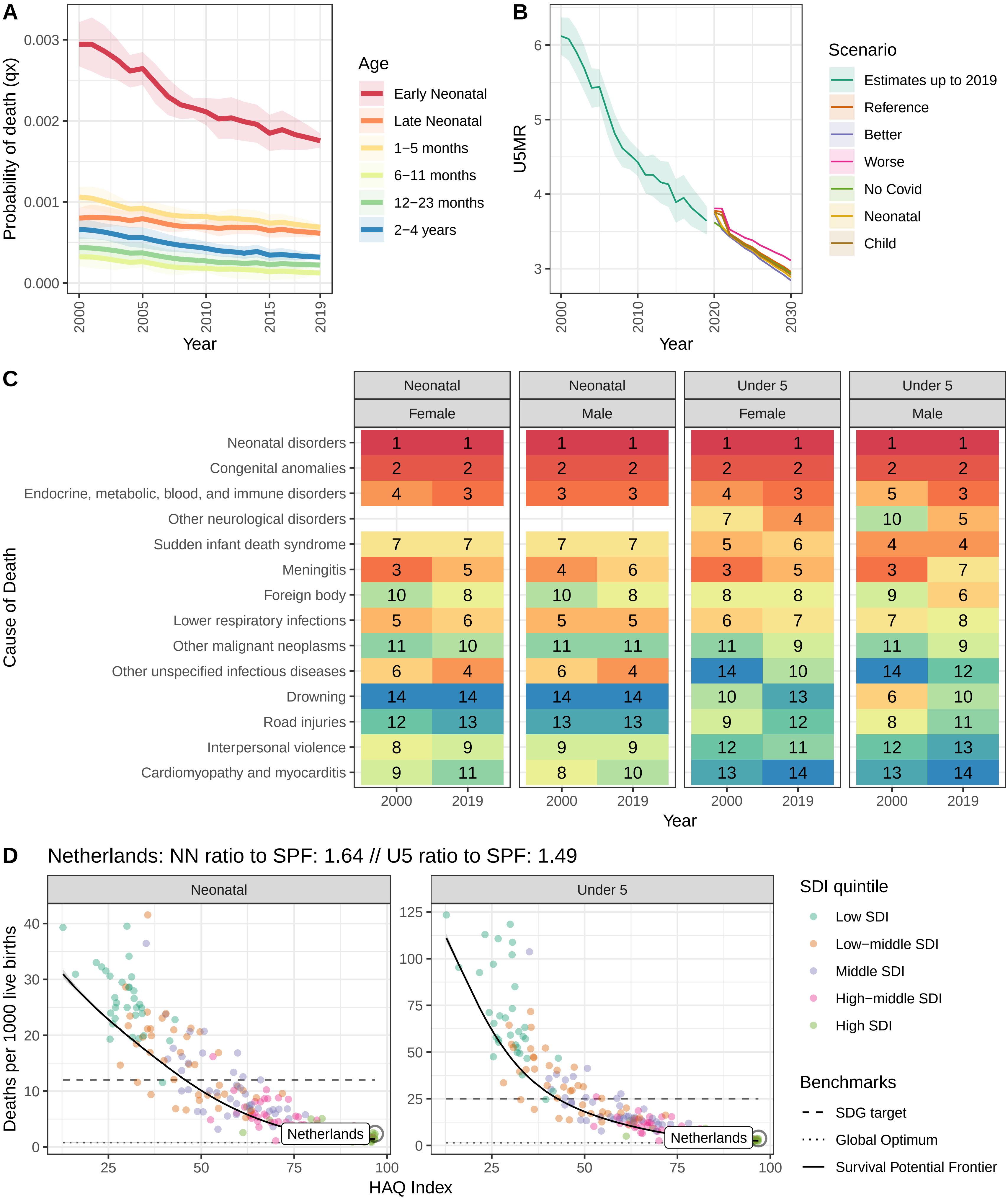

Norway

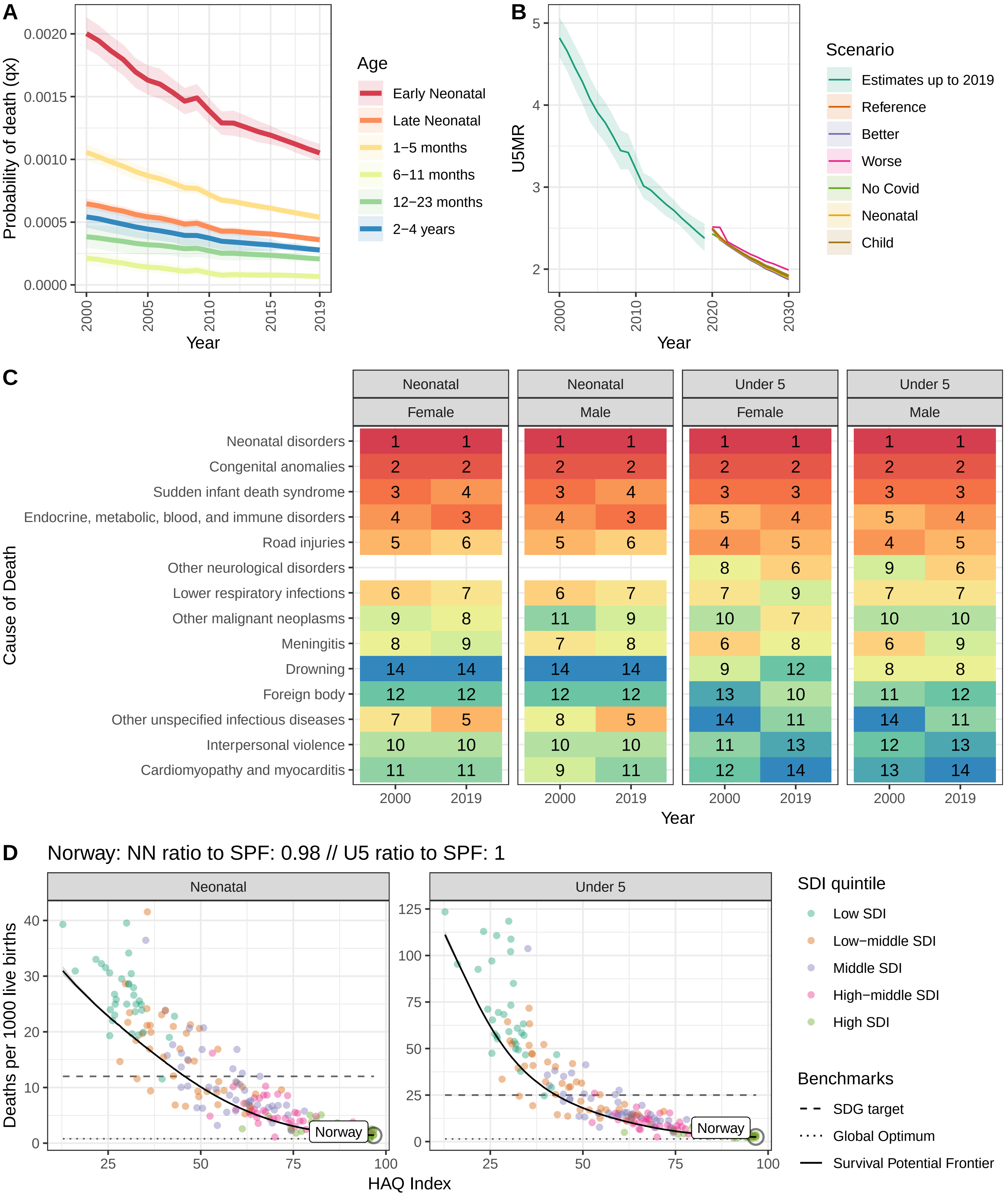
A
Probability of death (qx)
0.0020
0.0015
0.0010
0.0005
0.0000
2000
2005
2010
2015
2019
Year
Age
Early Neonatal
Late Neonatal
1−5 months
6−11 months
12−23 months
2−4 years
B
U5MR
5
4
3
2
2000
2010
2020
2030
Year
Scenario
Estimates up to 2019
Reference
Better
Worse
No Covid
Neonatal
Child
C
Cause of Death
Neonatal Female
Neonatal Male
Under 5 Female
Under 5 Male
Neonatal disorders 1 1 | 1 1 | 1 1 | 1 1
Congenital anomalies 2 2 | 2 2 | 2 2 | 2 2
Sudden infant death syndrome 3 4 | 3 4 | 3 3 | 3 3
Endocrine, metabolic, blood, and immune disorders 4 3 | 4 3 | 5 4 | 5 4
Road injuries 5 6 | 5 6 | 4 5 | 4 5
Other neurological disorders | | 8 6 | 9 6
Lower respiratory infections 6 7 | 6 7 | 7 9 | 7 7
Other malignant neoplasms 9 8 | 11 9 | 10 7 | 10 10
Meningitis 8 9 | 7 8 | 6 8 | 6 9
Drowning 14 14 | 14 14 | 9 12 | 8 8
Foreign body 12 12 | 12 12 | 13 10 | 11 12
Other unspecified infectious diseases 7 5 | 8 5 | 14 11 | 14 11
Interpersonal violence 10 10 | 10 10 | 11 13 | 12 13
Cardiomyopathy and myocarditis 11 11 | 9 11 | 12 14 | 13 14
2000 2019
Year
D
Norway: NN ratio to SPF: 0.98 // U5 ratio to SPF: 1
Neonatal
Under 5
Deaths per 1000 live births
40
30
20
10
0
125
100
75
50
25
HAQ Index
Norway
SDI quintile
Low SDI
Low−middle SDI
Middle SDI
High−middle SDI
High SDI
Benchmarks
SDG target
Global Optimum
Survival Potential Frontier

Portugal

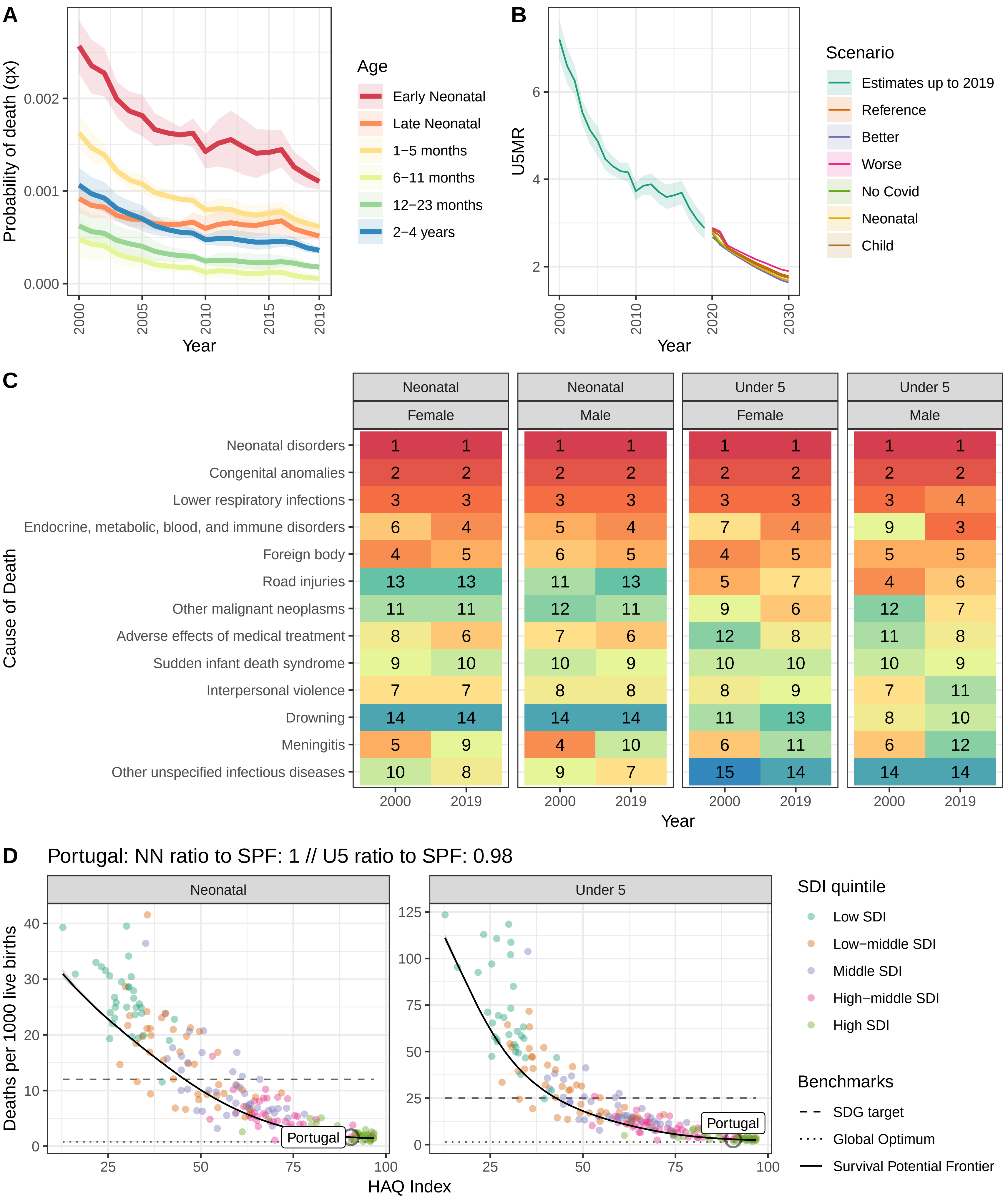
A
Probability of death (qx)
0.000
0.001
0.002
2000
2005
2010
2015
2019
Year
Age
Early Neonatal
Late Neonatal
1−5 months
6−11 months
12−23 months
2−4 years
B
U5MR
2
4
6
2000
2010
2020
2030
Year
Scenario
Estimates up to 2019
Reference
Better
Worse
No Covid
Neonatal
Child
C
Cause of Death
Neonatal Female
Neonatal Male
Under 5 Female
Under 5 Male
Neonatal disorders 1 1 1 1 1 1 1 1
Congenital anomalies 2 2 2 2 2 2 2 2
Lower respiratory infections 3 3 3 3 3 3 3 4
Endocrine, metabolic, blood, and immune disorders 6 4 5 4 7 4 9 3
Foreign body 4 5 6 5 4 5 5 5
Road injuries 13 13 11 13 5 7 4 6
Other malignant neoplasms 11 11 12 11 9 6 12 7
Adverse effects of medical treatment 8 6 7 6 12 8 11 8
Sudden infant death syndrome 9 10 10 9 10 10 10 9
Interpersonal violence 7 7 8 8 8 9 7 11
Drowning 14 14 14 14 11 13 8 10
Meningitis 5 9 4 10 6 11 6 12
Other unspecified infectious diseases 10 8 9 7 15 14 14 14
2000
2019
Year
D
Portugal: NN ratio to SPF: 1 // U5 ratio to SPF: 0.98
Neonatal
Under 5
Deaths per 1000 live births
0
10
20
30
40
0
25
50
75
100
125
25
50
75
100
HAQ Index
Portugal
SDI quintile
Low SDI
Low−middle SDI
Middle SDI
High−middle SDI
High SDI
Benchmarks
SDG target
Global Optimum
Survival Potential Frontier

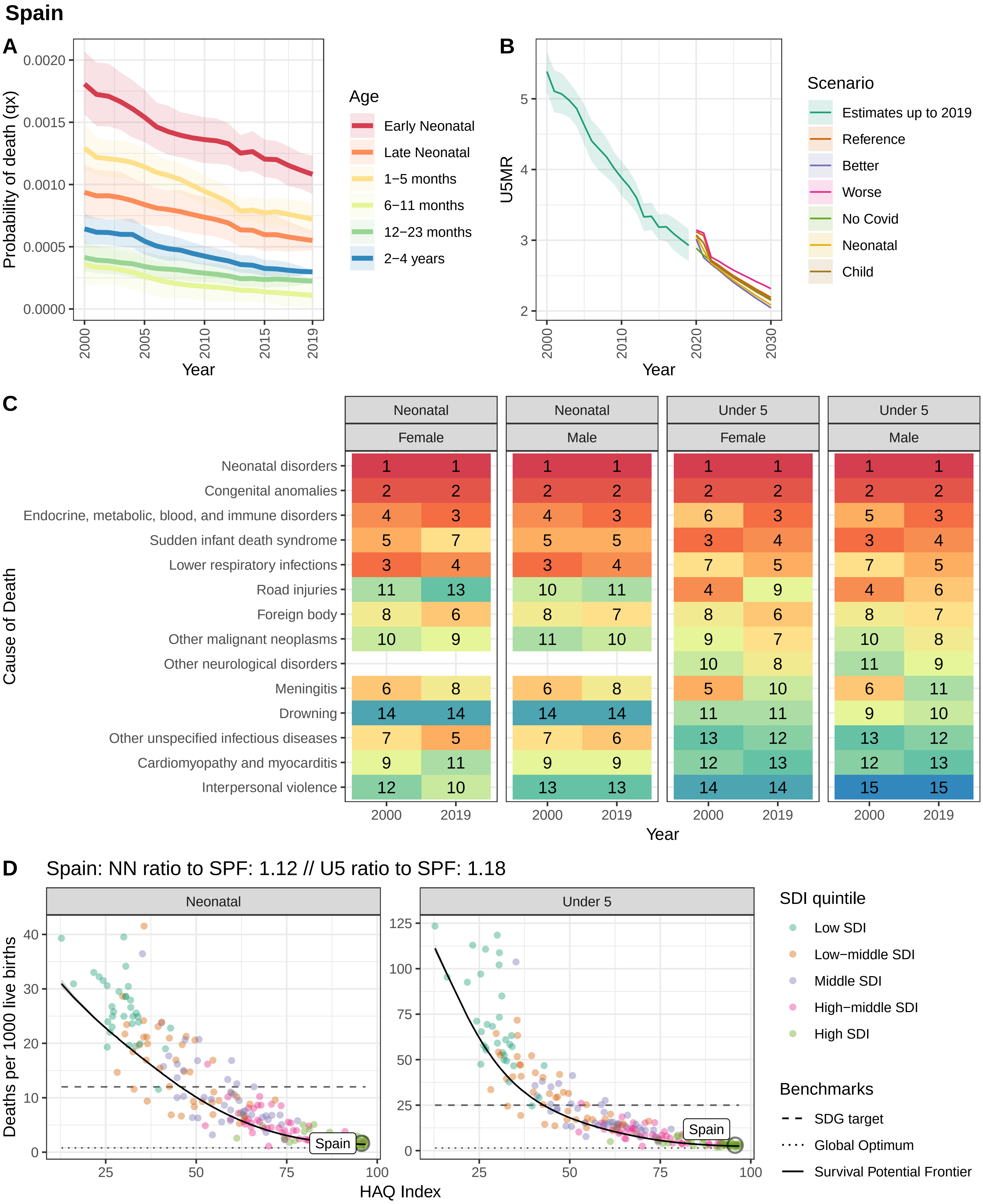
Spain
A
Probability of death (qx)
0.0020
0.0015
0.0010
0.0005
0.0000
2000
2005
2010
2015
2019
Year
Age
Early Neonatal
Late Neonatal
1−5 months
6−11 months
12−23 months
2−4 years
B
U5MR
5
4
3
2
2000
2010
2020
2030
Year
Scenario
Estimates up to 2019
Reference
Better
Worse
No Covid
Neonatal
Child
C
Cause of Death
Neonatal
Female
Neonatal
Male
Under 5
Female
Under 5
Male
Neonatal disorders
Congenital anomalies
Endocrine, metabolic, blood, and immune disorders
Sudden infant death syndrome
Lower respiratory infections
Road injuries
Foreign body
Other malignant neoplasms
Other neurological disorders
Meningitis
Drowning
Other unspecified infectious diseases
Cardiomyopathy and myocarditis
Interpersonal violence
1 1 1 1 1 1 1 1
2 2 2 2 2 2 2 2
4 3 4 3 6 3 5 3
5 7 5 5 3 4 3 4
3 4 3 4 7 5 7 5
11 13 10 11 4 9 4 6
8 6 8 7 8 6 8 7
10 9 11 10 9 7 10 8
10 8 11 9
6 8 6 8 5 10 6 11
14 14 14 14 11 11 9 10
7 5 7 6 13 12 13 12
9 11 9 9 12 13 12 13
12 10 13 13 14 14 15 15
2000
2019
2000
2019
2000
2019
2000
2019
Year
D
Spain: NN ratio to SPF: 1.12 // U5 ratio to SPF: 1.18
Neonatal
Under 5
Deaths per 1000 live births
40
30
20
10
0
125
100
75
50
25
0
Spain
Spain
25
50
75
100
25
50
75
100
HAQ Index
SDI quintile
Low SDI
Low−middle SDI
Middle SDI
High−middle SDI
High SDI
Benchmarks
SDG target
Global Optimum
Survival Potential Frontier

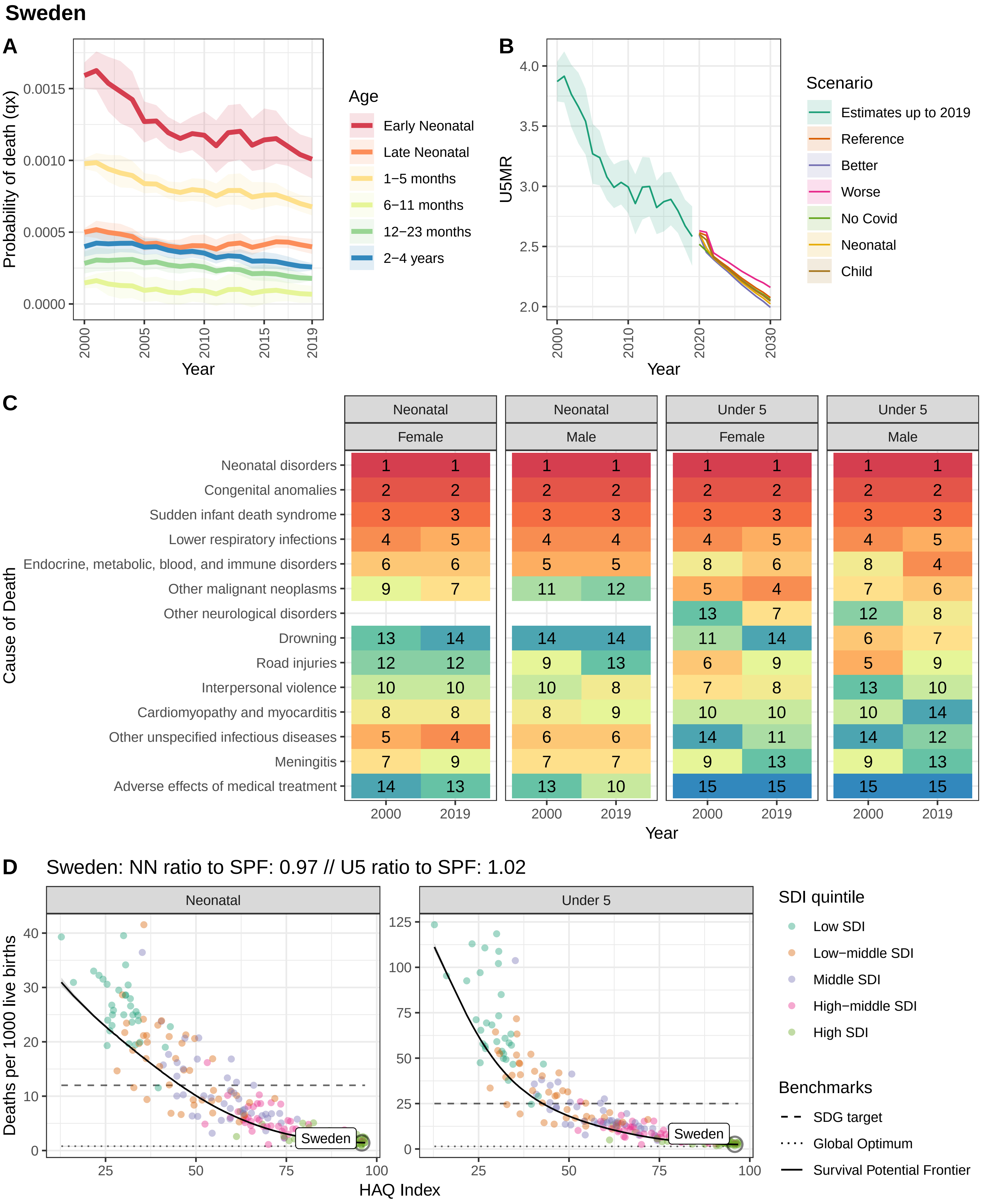
Sweden
A
Probability of death (qx)
0.0015
0.0010
0.0005
0.0000
2000
2005
2010
2015
2019
Year
Age
Early Neonatal
Late Neonatal
1−5 months
6−11 months
12−23 months
2−4 years
B
U5MR
4.0
3.5
3.0
2.5
2.0
2000
2010
2020
2030
Year
Scenario
Estimates up to 2019
Reference
Better
Worse
No Covid
Neonatal
Child
C
Cause of Death
Neonatal Female
Neonatal Male
Under 5 Female
Under 5 Male
Neonatal disorders 1 1 | 1 1 | 1 1 | 1 1
Congenital anomalies 2 2 | 2 2 | 2 2 | 2 2
Sudden infant death syndrome 3 3 | 3 3 | 3 3 | 3 3
Lower respiratory infections 4 5 | 4 4 | 4 5 | 4 5
Endocrine, metabolic, blood, and immune disorders 6 6 | 5 5 | 8 6 | 8 4
Other malignant neoplasms 9 7 | 11 12 | 5 4 | 7 6
Other neurological disorders | | 13 7 | 12 8
Drowning 13 14 | 14 14 | 11 14 | 6 7
Road injuries 12 12 | 9 13 | 6 9 | 5 9
Interpersonal violence 10 10 | 10 8 | 7 8 | 13 10
Cardiomyopathy and myocarditis 8 8 | 8 9 | 10 10 | 10 14
Other unspecified infectious diseases 5 4 | 6 6 | 14 11 | 14 12
Meningitis 7 9 | 7 7 | 9 13 | 9 13
Adverse effects of medical treatment 14 13 | 13 10 | 15 15 | 15 15
2000
2019
Year
D
Sweden: NN ratio to SPF: 0.97 // U5 ratio to SPF: 1.02
Neonatal
Under 5
Deaths per 1000 live births
40
30
20
10
0
125
100
75
50
25
0
25
50
75
100
HAQ Index
Sweden
SDI quintile
Low SDI
Low−middle SDI
Middle SDI
High−middle SDI
High SDI
Benchmarks
SDG target
Global Optimum
Survival Potential Frontier

Switzerland

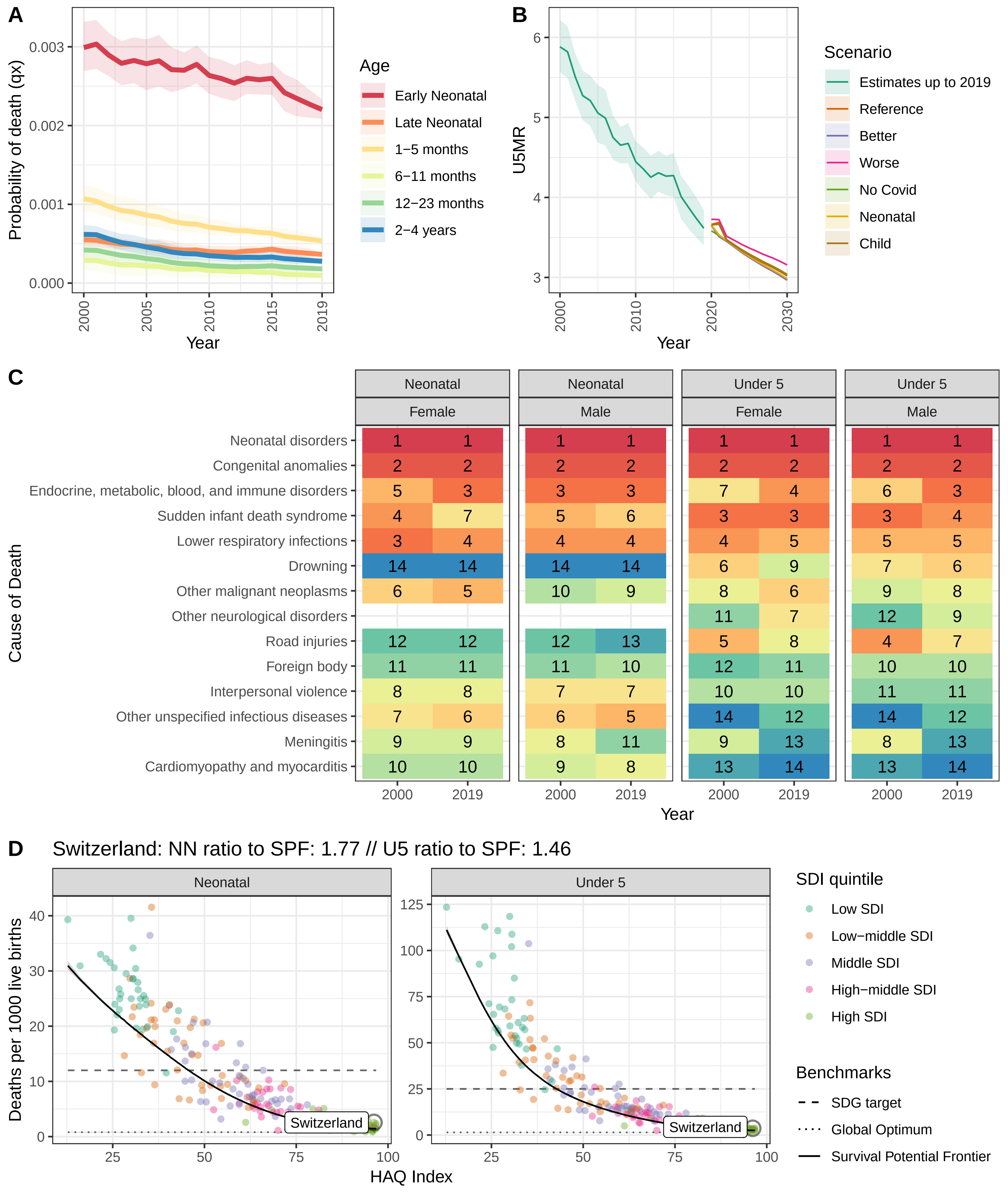

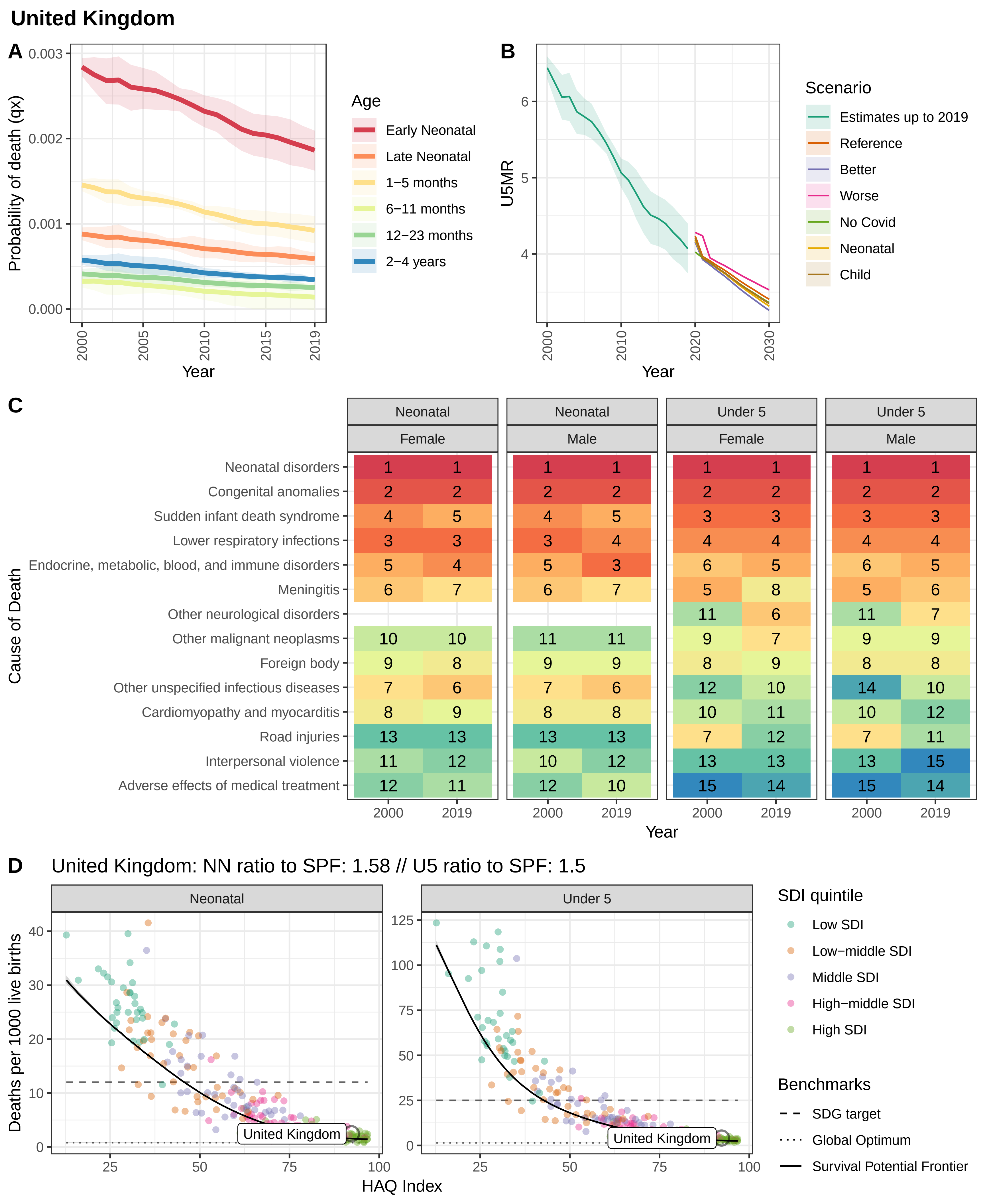
United Kingdom
A
Probability of death (qx)
0.003
0.002
0.001
0.000
2000
2005
2010
2015
2019
Year
Age
Early Neonatal
Late Neonatal
1−5 months
6−11 months
12−23 months
2−4 years
B
U5MR
6
5
4
2000
2010
2020
2030
Year
Scenario
Estimates up to 2019
Reference
Better
Worse
No Covid
Neonatal
Child
C
Cause of Death
Neonatal Female | Neonatal Male | Under 5 Female | Under 5 Male
Neonatal disorders 1 1 | 1 1 | 1 1 | 1 1
Congenital anomalies 2 2 | 2 2 | 2 2 | 2 2
Sudden infant death syndrome 4 5 | 4 5 | 3 3 | 3 3
Lower respiratory infections 3 3 | 3 4 | 4 4 | 4 4
Endocrine, metabolic, blood, and immune disorders 5 4 | 5 3 | 6 5 | 6 5
Meningitis 6 7 | 6 7 | 5 8 | 5 6
Other neurological disorders | | 11 6 | 11 7
Other malignant neoplasms 10 10 | 11 11 | 9 7 | 9 9
Foreign body 9 8 | 9 9 | 8 9 | 8 8
Other unspecified infectious diseases 7 6 | 7 6 | 12 10 | 14 10
Cardiomyopathy and myocarditis 8 9 | 8 8 | 10 11 | 10 12
Road injuries 13 13 | 13 13 | 7 12 | 7 11
Interpersonal violence 11 12 | 10 12 | 13 13 | 13 15
Adverse effects of medical treatment 12 11 | 12 10 | 15 14 | 15 14
2000 2019
Year
D
United Kingdom: NN ratio to SPF: 1.58 // U5 ratio to SPF: 1.5
Neonatal
Under 5
Deaths per 1000 live births
40
30
20
10
0
125
100
75
50
25
0
25
50
75
100
HAQ Index
United Kingdom
SDI quintile
Low SDI
Low−middle SDI
Middle SDI
High−middle SDI
High SDI
Benchmarks
SDG target
Global Optimum
Survival Potential Frontier

Argentina

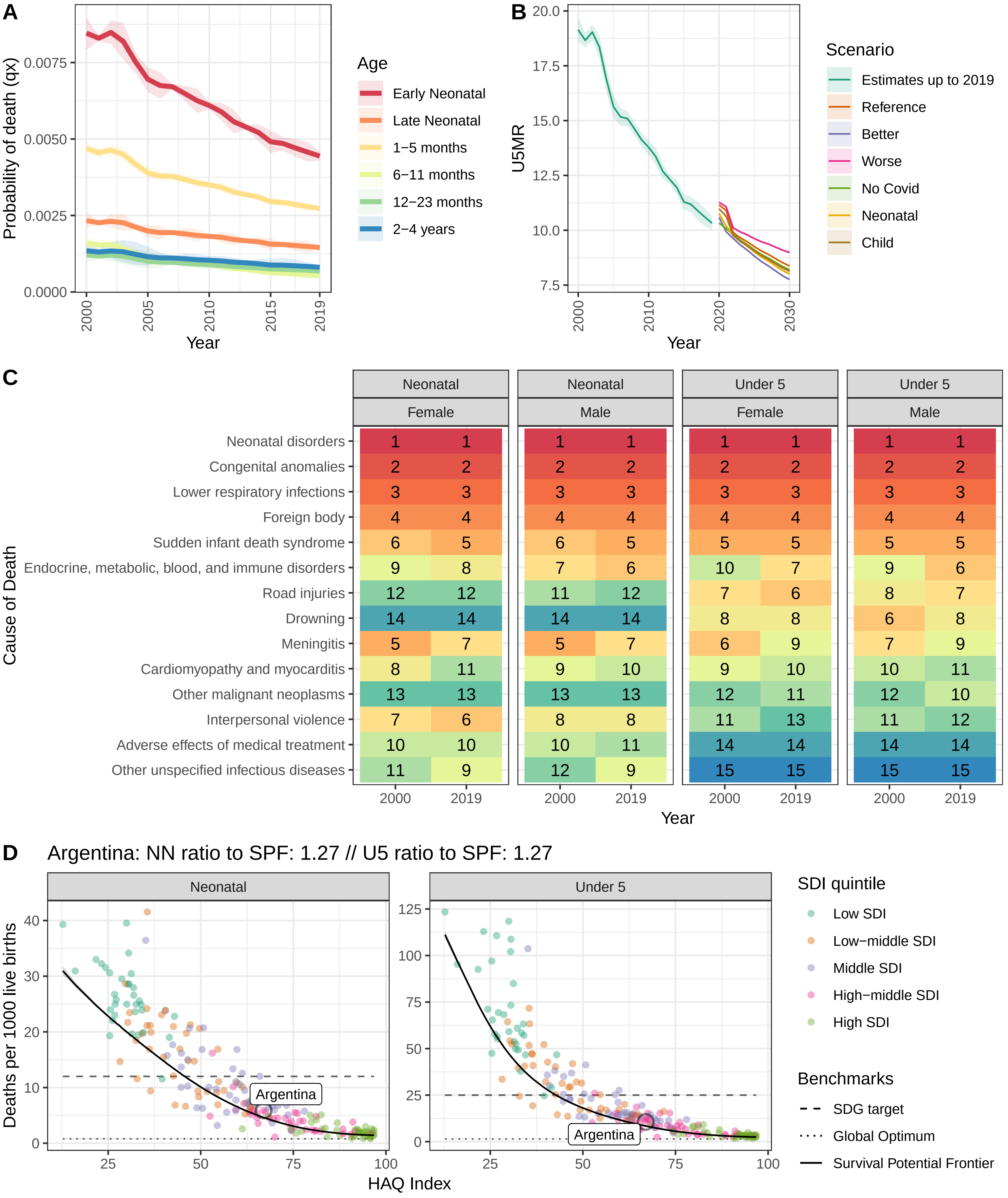

A
Probability of death (qx)
0.0075
0.0050
0.0025
0.0000
2000
2005
2010
2015
2019
Year
Age
Early Neonatal
Late Neonatal
1−5 months
6−11 months
12−23 months
2−4 years
B
U5MR
20.0
17.5
15.0
12.5
10.0
7.5
2000
2010
2020
2030
Year
Scenario
Estimates up to 2019
Reference
Better
Worse
No Covid
Neonatal
Child
C
Neonatal
Female
Neonatal
Male
Under 5
Female
Under 5
Male
Cause of Death
Neonatal disorders 1 1 1 1 1 1 1 1
Congenital anomalies 2 2 2 2 2 2 2 2
Lower respiratory infections 3 3 3 3 3 3 3 3
Foreign body 4 4 4 4 4 4 4 4
Sudden infant death syndrome 6 5 6 5 5 5 5 5
Endocrine, metabolic, blood, and immune disorders 9 8 7 6 10 7 9 6
Road injuries 12 12 11 12 7 6 8 7
Drowning 14 14 14 14 8 8 6 8
Meningitis 5 7 5 7 6 9 7 9
Cardiomyopathy and myocarditis 8 11 9 10 9 10 10 11
Other malignant neoplasms 13 13 13 13 12 11 12 10
Interpersonal violence 7 6 8 8 11 13 11 12
Adverse effects of medical treatment 10 10 10 11 14 14 14 14
Other unspecified infectious diseases 11 9 12 9 15 15 15 15
2000
2019
Year
D
Argentina: NN ratio to SPF: 1.27 // U5 ratio to SPF: 1.27
Neonatal
Under 5
Deaths per 1000 live births
40
30
20
10
0
125
100
75
50
25
0
Argentina
Argentina
25
50
75
100
HAQ Index
SDI quintile
Low SDI
Low−middle SDI
Middle SDI
High−middle SDI
High SDI
Benchmarks
SDG target
Global Optimum
Survival Potential Frontier

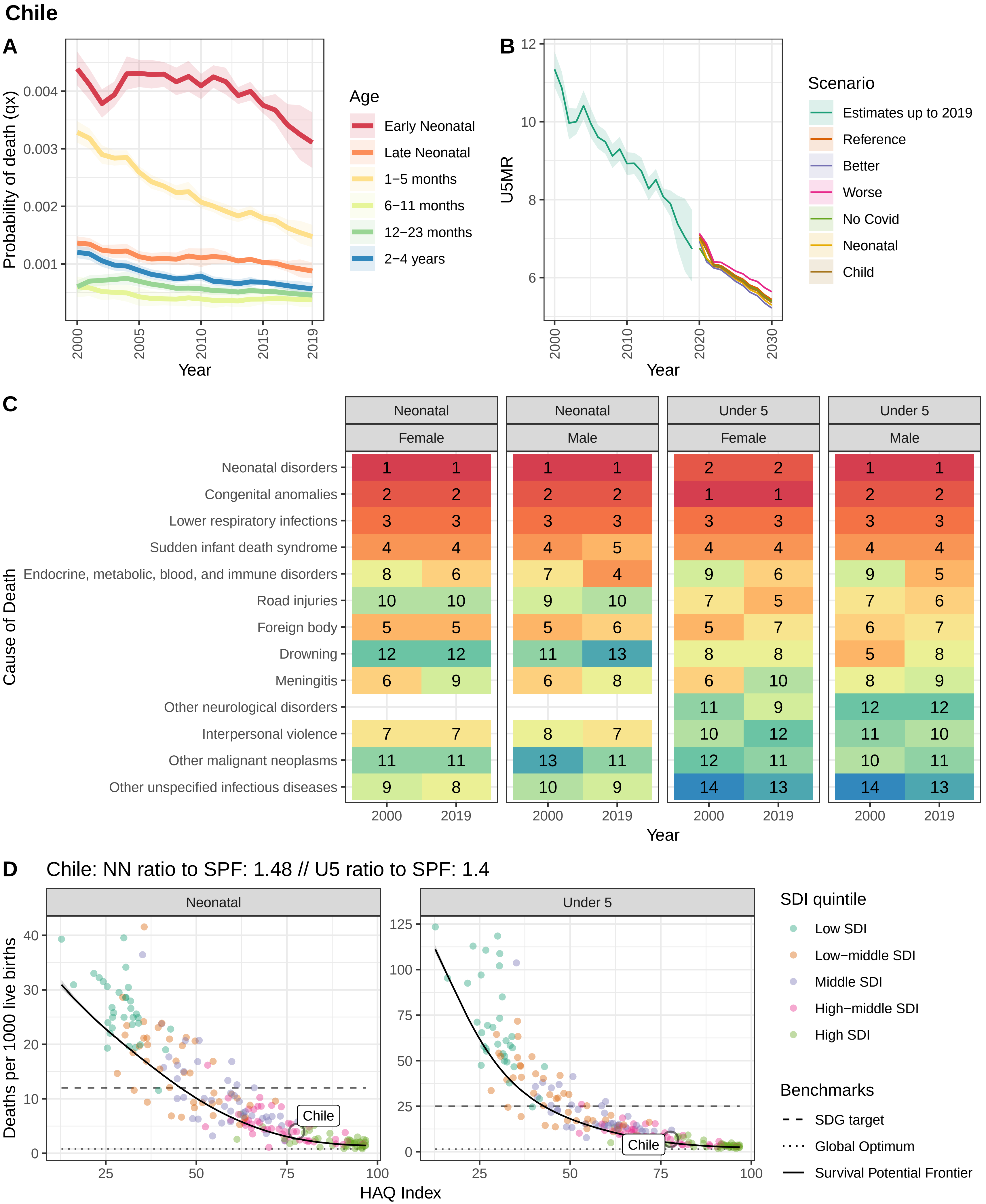
Chile
A
Probability of death (qx)
0.004
0.003
0.002
0.001
2000
2005
2010
2015
2019
Year
Age
Early Neonatal
Late Neonatal
1−5 months
6−11 months
12−23 months
2−4 years
B
U5MR
12
10
8
6
2000
2010
2020
2030
Year
Scenario
Estimates up to 2019
Reference
Better
Worse
No Covid
Neonatal
Child
C
Neonatal
Female
Neonatal
Male
Under 5
Female
Under 5
Male
Cause of Death
Neonatal disorders 1 1 1 1 2 2 1 1
Congenital anomalies 2 2 2 2 1 1 2 2
Lower respiratory infections 3 3 3 3 3 3 3 3
Sudden infant death syndrome 4 4 4 5 4 4 4 4
Endocrine, metabolic, blood, and immune disorders 8 6 7 4 9 6 9 5
Road injuries 10 10 9 10 7 5 7 6
Foreign body 5 5 5 6 5 7 6 7
Drowning 12 12 11 13 8 8 5 8
Meningitis 6 9 6 8 6 10 8 9
Other neurological disorders 11 9 12 12
Interpersonal violence 7 7 8 7 10 12 11 10
Other malignant neoplasms 11 11 13 11 12 11 10 11
Other unspecified infectious diseases 9 8 10 9 14 13 14 13
2000
2019
Year
D
Chile: NN ratio to SPF: 1.48 // U5 ratio to SPF: 1.4
Neonatal
Under 5
Deaths per 1000 live births
40
30
20
10
0
125
100
75
50
25
Chile
HAQ Index
SDI quintile
Low SDI
Low−middle SDI
Middle SDI
High−middle SDI
High SDI
Benchmarks
SDG target
Global Optimum
Survival Potential Frontier

Uruguay

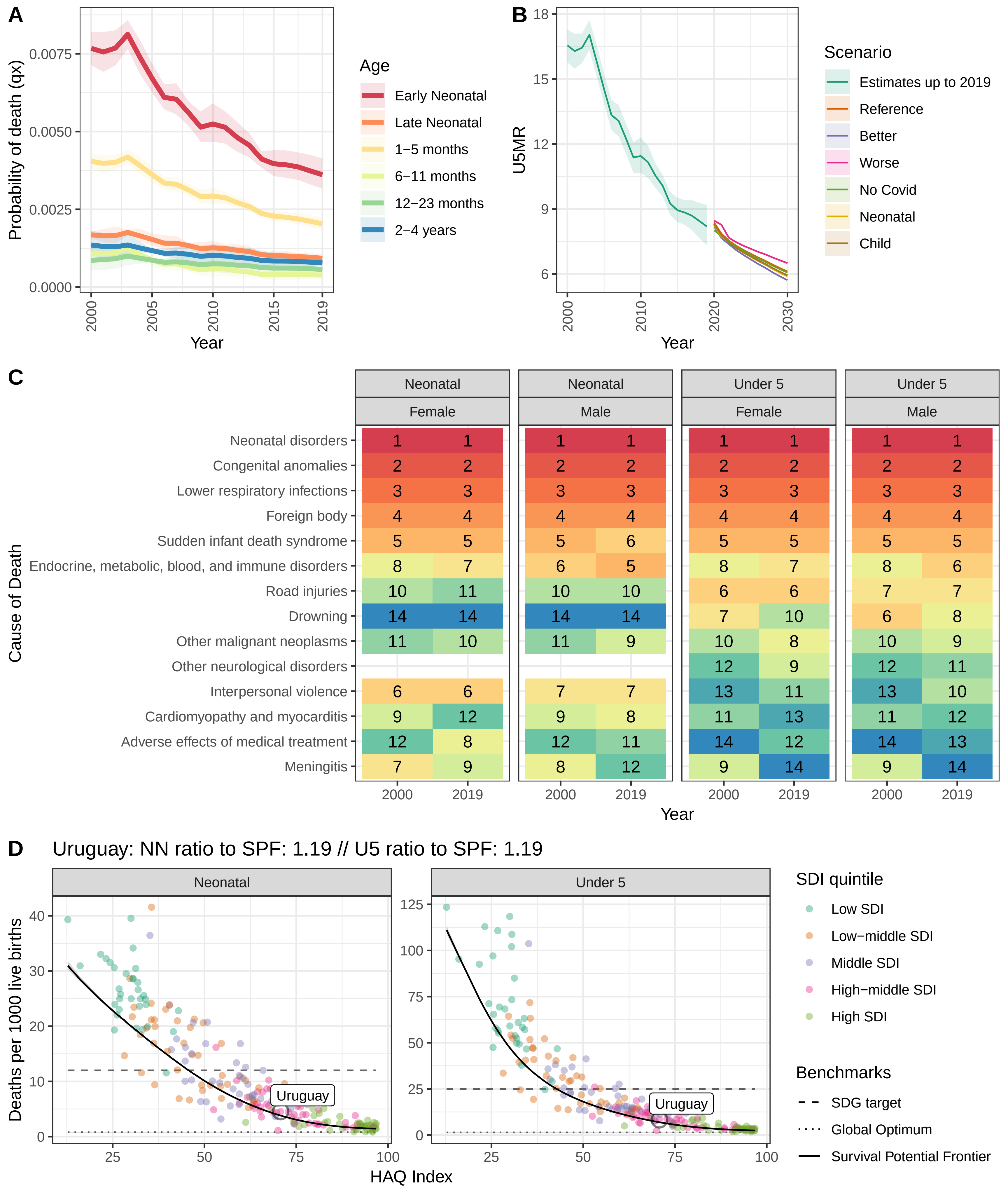

A
Probability of death (qx)
Year
Age
Early Neonatal
Late Neonatal
1−5 months
6−11 months
12−23 months
2−4 years
B
U5MR
Year
Scenario
Estimates up to 2019
Reference
Better
Worse
No Covid
Neonatal
Child
C
Neonatal
Female
Neonatal
Male
Under 5
Female
Under 5
Male
Cause of Death
Neonatal disorders
Congenital anomalies
Lower respiratory infections
Foreign body
Sudden infant death syndrome
Endocrine, metabolic, blood, and immune disorders
Road injuries
Drowning
Other malignant neoplasms
Other neurological disorders
Interpersonal violence
Cardiomyopathy and myocarditis
Adverse effects of medical treatment
Meningitis
Year
D
Uruguay: NN ratio to SPF: 1.19 // U5 ratio to SPF: 1.19
Neonatal
Under 5
Deaths per 1000 live births
HAQ Index
Uruguay
SDI quintile
Low SDI
Low−middle SDI
Middle SDI
High−middle SDI
High SDI
Benchmarks
SDG target
Global Optimum
Survival Potential Frontier
